# Sulfonyl-PYBOX/ErCl_3_ complex enable highly enantioselective synthesis of α,α-dialkyl and α-alkyl-α-aryl hydrazinonitriles

**DOI:** 10.1038/s41467-026-74600-0

**Published:** 2026-06-18

**Authors:** Yi Gong, Zheng Zhang, Qiang Cao, Cai Wang, Min-Chuan Jiang, Zhi-Heng Qiu, Xiao-Jing Le, Yu-Jie Ye, Tao Wang, Yun-Hao Tang, Ying Zhang, Haoran Zhao, Li Cui, Feng Zhou, Bo-Shuai Mu, Xin Wang, Jian Zhou

**Affiliations:** 1 State Key Laboratory of Petroleum Molecular & Process Engineering, Shanghai Engineering Research Center of Molecular Therapeutics and New Drug Development, Shanghai Key Laboratory of Green Chemistry and Chemical Processes, School of Chemistry and Molecular Engineering, East China Normal University, Shanghai, China; 2 State Key Laboratory for Biology of Plant Diseases and Insect Pests, Institute of Plant Protection, Chinese Academy of Agricultural Sciences, Beijing, China; 3 College of Chemistry, Sichuan University, Chengdu, China; 4 State Key Laboratory of Organometallic Chemistry, Shanghai Institute of Organic Chemistry, Shanghai, China

**Keywords:** Asymmetric catalysis, Synthetic chemistry methodology

## Abstract

We report the highly enantioselective nucleophilic addition reaction of simple ketone-derived hydrazones. Accordingly, a ErCl₃-catalyzed cyanation of both aliphatic and aryl ketone hydrazones is achieved for the facile access of C^α^-tetrasubstituted α-hydrazino nitriles in up to 96% ee, by using the sterically confined pyridinebisoxazoline (PYBOX) ligand featuring a sulfonyl group at the pyridine C4 position. This method enables the shortest catalytic enantioselective total synthesis of *L-carbidopa*, a drug that could treat the symptoms of Parkinson’s disease, with 82% overall yield in four steps. These adducts are valuable synthons to various α-tertiary hydrazines and related azacycles that are interesting targets for medicinal studies and pesticide research. From our biological analysis, a chiral α-hydrazino nitrile with good insecticidal activity against *Aphis gossypii* (LC_50_ = 15.11 mg∙L^–1^) was identified, rivaling commercial insecticides.

## Abstract (web summary)

Authors present an ErCl₃-catalyzed cyanation of both aliphatic and aryl ketone hydrazones for the facile access of C(alpha)-tetrasubstituted alpha-hydrazino nitriles in up to 96% ee, by using a sterically confined pyridinebisoxazoline (PYBOX) ligand featuring a sulfonyl group at the pyridine C4 position.

## Introduction

**Fig. 1:**
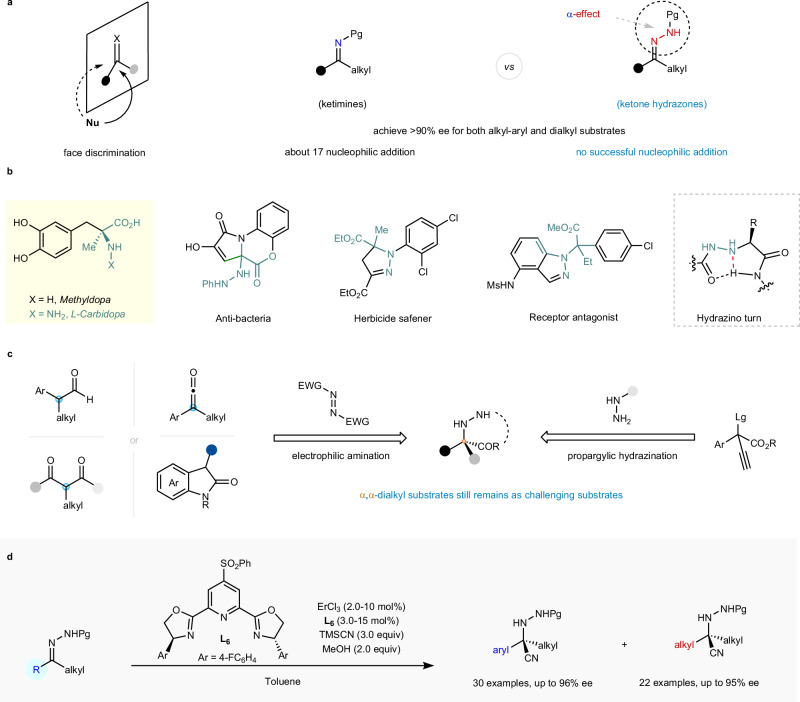
Working hypothesis. **a** Catalytic asymmetric nucleophilic addition (ketimines vs. ketone hydrazones). **b** Value of C^α^-tetrasubstituted α-hydrazine acids. **c** Catalytic asymmetric electrophilic amination and asymmetric propargylic substitution (APS). **d** This work: Strecker reaction.

Catalytic enantioselective synthesis of structurally diverse α-tertiary chiral amine derivatives is of current interest^1–8^, since aza-quaternary stereocenters represent privileged scaffolds for natural products and drugs^9–13^. A straightforward strategy to this end involves differentiating between the prochiral faces of ketone-derived C=N double bonds (Fig. 1a)^14–16^, but achieving excellent enantiofacial control across a broad substrate scope remains highly challenging due to the lesser steric dissimilarity of the two substituents on the prochiral carbon^17–19^. Despite intensive studies in the past decades, successful catalytic nucleophilic additions to C=N double bonds have largely been restricted to alkyl-aryl ketimines, less than 20 reported protocols can deliver >90% enantiomeric excess (ee) for both alkyl-aryl and dialkyl variants^20–33^. The situation is even more challenging for ketone hydrazones^34^, as no highly enantioselective nucleophilic addition has been reported to date^35^, although one 1,3-dipolar cycloaddition^36^ and one umpolung gem-difluoroallylation^37^ could convert both aryl-alkyl and dialkyl ketone hydrazones to the corresponding products in >90% ee.

On the other hand, hydrazine derivatives represent an important subclass of the amine family, serving as both versatile synthetic intermediates for azacycle construction and pharmacophores^38,39^. In particular, C^α^-tetrasubstituted α-hydrazino acid derivatives have attracted increasing attention for drug and agrochemical development due to their complex three-dimensionality and enhanced conformational constraints^40^, as exemplified by some drugs and bioactive compounds shown in Fig. 1b^41,42^. Notably, *L*-*carbidopa*^43^, a drug to treat Parkinson’s disease, was developed by replacing the NH_2_ group of methyldopa (a drug to treat high blood pressure) with a hydrazine group, showing the distinctly different pharmacology of two amines, in terms of functionalities. Moreover, the N–N bond makes α-hydrazino acids aza-analogs of β-amino acids, which is very attractive for the development of backbone-extended peptidomimetics with a unique “hydrazino turn” structure^44,45^. Therefore, catalytic enantioselective synthesis of C^α^-tetrasubstituted α-hydrazino acid derivatives with sufficient structural diversity is highly desirable.

However, because the catalytic enantioselective synthesis of α-tertiary hydrazines is still underdeveloped^46–51^, there are limiting strategies to chiral C^α^-tetrasubstituted α-hydrazino acid derivatives. Currently, besides limited examples via enzymatic resolution^52^, the only established approach is enantioselective electrophilic α-amination of α-branched aldehydes, ketenes or active methines (e.g., α-substituted β-ketoesters) using azadicarboxylates, as shown in Fig. 1c^53,54^. Nevertheless, despite the progress, α,α-dialkyl carbonyls are still considered problematic substrates^55–60^. Accordingly, the use of enantioselective amination as the key step for the total synthesis of *L*-*carbidopa* had to start from α-methyl-β-ketoesters, with a route of at least 7 steps^61,62^. Recently, we reported asymmetric Cu-catalyzed propargylic hydrazination of α-ketoester-derived α-ethynyl carbonates as an approach to access chiral α-tertiary α-hydrazino acid esters; however, the substrates scope and enantioselectivity have ample room to improve—only α-aryl carbonates could deliver the desired product in up to 90% ee^63^.

The catalytic asymmetric cyanation^64–67^ of ketone hydrazones is a straightforward route to optically active C^α^-tetrasubstituted α-hydrazino acid derivatives, yet it remains a challenging task. In the pioneering study on the asymmetric cyanation of hydrazones, Jacobsen et al. disclosed that a pyridinebisoxazoline (PYBOX)-derived ErCl_3_ complex could achieve 90% ee in the cyanation of benzaldehyde-based hydrazone^35^, whereas only 40% ee was observed for the analogous acetophenone-based substrate. This gap in the ee value underscores the inherent difficulty in achieving catalytic enantioselective nucleophilic additions to ketone hydrazones—even the distinction between a phenyl and a methyl group at the prochiral center proves challenging to differentiate highly enantioselectively. This difficulty is further evidenced by the scarcity of successful reactions of ketone hydrazones that give α-tertiary hydrazine derivatives with >90% ee. Apart from Leighton’s^68,69^ stoichiometric chiral allylsilane-based allylation and Mannich reactions of ketone-derived hydrazones, only two catalytic enantioselective protocols exist: Jørgensen et al. achieved an elegant chiral H-bond donor catalyzed 1,3-dipolar cycloaddition with nitroolefins^36^, and Hou et al. realized a Pd-catalyzed asymmetric umpolung *gem*-difluoroallylation^37^. To our knowledge, highly enantioselective catalytic nucleophilic addition of ketone hydrazones is unprecedented to date.

We speculated the difficulty in putting ketone hydrazones to catalytic enantioselective synthesis of α-tertiary hydrazines arises from two factors: 1) the greater stability of hydrazones than imines^70^, due to the repulsion of the lone pairs of both nitrogen atoms (α-effect), and 2) the coordination behavior of hydrazones is often distinct from that of imines^34,71^, owing to the N–N bond and its associated protecting groups (e.g., benzoyl). These differences prevent the direct application of the known ketimine reaction conditions to ketone hydrazones.

Meanwhile, encouraged by Sibi’s concept of relaying and amplifying chirality from the chiral catalysts by decorating the substrates with a bulky achiral template^72^, as well as Tang’s sidearm approach of improving the chiral pocket of bisoxazoline by a sidearm^73^, we are engaging in developing sterically confined PYBOX by installing a “bulky” relay group on the pyridine C4 position. This modification is helpful to tune the electronic and steric properties of the ligand to enhance the enantiofacial control. First, it is flexible to modify the Lewis basicity of the ligand by using various heteroatom-based relay groups, which not only adjust the catalytic activity of the metal center, but also vary the pyridine nitrogen–metal bond length that influences the chiral pocket of the catalyst. Second, for a given reaction, a suitable bulky relay group is helpful to produce a sterically constrained microenvironment to relay the stereochemical information from the ligand chirality via indirect interactions, to reinforce enantiofacial control. The effectiveness of such sterically confined PYBOX has been showcased in some Cu-catalyzed highly enantioselective reactions to construct tetrasubstituted carbon stereocenters, which are unattainable by using the corresponding unmodified ligand. For example, PYBOX with a bulky benzyloxy, phosphonate or sulfonyl group at the C4 position of pyridine enabled some highly enantioselective Cu-catalyzed azide–alkyne cycloaddition (CuAAC) reactions, no matter via desymmetrization or kinetic resolution^74–77^. Notably, PYBOX featuring a bulky benzyloxy group at the C4 position of pyridine and two *cis*-phenyl groups at the C5 position of both oxazoline rings as the relaying groups, exhibited excellent remote enantiofacial control in Cu-catalyzed asymmetric propargylic substitution reactions (APS). This effect allowed both dialkyl and alkylaryl ketone-derived propargylic carbonates to give α-tertiary ethynylamines in unprecedentedly excellent ee values^78^. In addition, most recently, an analogy PYBOX with a benzylthio relay group enabled the asymmetric propargylic hydrazination for highly enantioselective synthesis of α-alkyl–α-aryl and α-dialkylated α-tertiary α-ethynylhydrazine^63^.

**Fig. 2:**
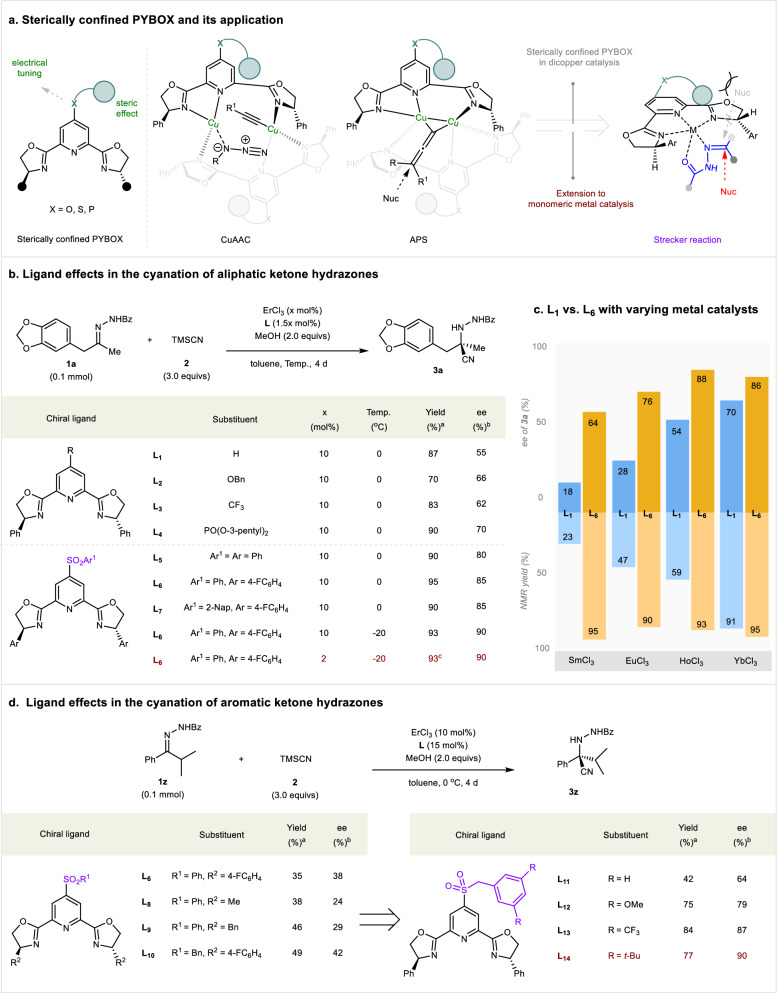
Reaction development. **a** Sterically confined PYBOX and its application. **b** Ligand effect in the cyanation of aliphatic ketone hydrazones. **c** Comparison of ligand performances with varying metal catalysts. **d** Ligand effects in the cyanation of aromatic ketone hydrazones. Reaction conditions: **1** (0.10 mmol), TMSCN (0.30 mmol), ErCl_3_ or other lanthanide metals (10 mol%), PYBOX ligand (15 mol%), MeOH (2.0 equiv), toluene (1.0 mL), 0 °C, 96 h. ^a 1^H NMR yield with 1,3,5-trimethoxybenzene as internal standard. ^b^ The ee was determined by performing chiral HPLC analysis. ^c^ Isolated yield. 2-Nap = 2-Naphthyl.

Our working hypothesis, that a suitable C4 relay group can help the formation of a sterically constrained chiral pocket, is supported by mechanistic studies of both CuAAC and APS reactions. These studies argue that a dicopper catalysis mechanism is involved, enabling the effective distinguishing of the subtle distinction between the substituents on the prochiral carbons, or the stereocenters in the kinetic resolution process. These results prompted us to examine whether these sterically confined PYBOX are workable in other metal-catalyzed asymmetric reactions, and in particular, those involving a monomeric metal center (Fig. 2a). Inspired by Jacobsen’s seminar work, together with the importance of α-chiral nitriles as chiral synthons^79^, as well as prominent structural motifs in natural products, pharmaceuticals, and bioactive compounds^80–82^, we speculate that while the open space resulting from the planar pyridine of traditional PYBOX makes it difficult to discriminate the prochiral plane of ketone hydrazones, a suitable C4 shielding group should be helpful to form a sterically constrained microenvironment to enhance the enantiofacial discrimination for the cyanation reaction. Notably, while the highly enantioselective Strecker reaction of ketimines has been established by Jacobsen^83,84^, Shibasaki^20^, Feng^21,24^ and Hiyashi^85^ and others^64–67^, that of ketone hydrazones still remains a challenge. In this work, the effectiveness of this hypothesis is reported—sulfonyl-PYBOX ligands enable the highly enantioselective Strecker reaction of a broad range of ketone hydrazones (Fig. 1d).

## Results

### Reaction development

Initially, we conducted the Strecker reaction of aliphatic ketone hydrazone **1a**, bearing piperonyl—a common structural moiety in bioactive compounds—to investigate the potency of sterically confined PYBOX ligands with different C4 shielding groups, and typical results are shown in Fig. 2b. Following Jacobsen’s conditions^35^, using a 10 mol% PYBOX **L**_**1**_/ErCl_3_ complex as the catalyst and MeOH as an additive, the reaction of **1a** with 3.0 equivalents of TMSCN in toluene at 0 °C yielded the desired product **3a** in 87% yield but with only 55% ee after 4 days (for initial attempts, see Part 4 of the Supplementary Information, SI). Introducing an electron-donating OBn group in ligand **L**_**2**_ improved the ee of **3a** to 66%, albeit with a decreased yield of 70%. In contrast, PYBOX **L**_**3**_, bearing a bulky electron-withdrawing CF_3_ group, enhanced both enantioselectivity and reactivity, affording **3a** in 83% yield and 62% ee, and phosphonate-PYBOX **L**_**4**_ further improved the yield and enantioselectivity of **3a** to 90% and 70% ee, respectively. Furthermore, sulfonyl-PYBOX **L**_**5**_, featuring an electron-withdrawing C4 phenylsulfonyl group, could give **3a** in 90% yield and 80% ee. Encouraged by these promising results, we modified the oxazoline moiety by replacing the phenyl group with a 4-fluorophenyl group, and the corresponding ligand **L**_**6**_ gave **3a** in improved 85% ee. Further changing phenylsulfonyl to 2-naphthylsulfonyl in **L**_**7**_ did not improve the outcome. Finally, lowering the reaction temperature to –20 °C enabled the synthesis of adduct **3a** in 93% yield and 90% ee. Notably, further reducing the catalyst loading to 2 mol% still furnished **3a** in uncompromised 93% yield and 90% ee.

Subsequent investigations revealed that the sulfonyl-PYBOX ligand **L**_**6**_ outperformed **L**_**1**_ in not only the Er-catalyzed process, but also other lanthanides-mediated reactions (e.g., Sm, Eu, Ho, Yb). In the model Strecker reaction of hydrazone **1a,** **L**_**6**_ consistently delivered higher yields and superior enantioselectivity compared with **L**_**1**_ (Fig. 2c). These results strongly support our hypothesis that introducing a bulky C4 shielding group to PYBOX ligands could make the chiral pocket more sterically confined, enabling better enantiofacial face discrimination of monomeric metal-catalyzed functionalization of unsaturated double bonds.

To further demonstrate the effectiveness of the C4 shielding group of sulfonyl-PYBOX in creating sterically confined chiral pocket, the Strecker reaction of another challenging substrate, isopropyl phenyl ketone derived hydrazone **1z** was undertaken (Fig. 2d). To our knowledge, it is very challenging to distinguish between prochiral face of this ketone derived C=N double bond, and only one successful catalytic enantioselective protocol is available^32^, as well as a chiral reagent realized allylation reaction^68^. Not unexpectedly, the above-established condition using sulfonyl-PYBOX **L**_**6**_ afforded the product **3z** in only 35% yield and 38% ee. Varying the substituent on the chiral center of the oxazoline ring led to poorer outcomes, as witnessed by the decreased 24% and 29% ee obtained by **L**_**8**_ and **L**_**9**_, respectively. However, increasing the flexibility and bulk of the sulfonyl group at the pyridine C4 position could greatly increase the enantiofacial control. With the phenylsulfonyl substituted by a benzylsulfonyl group, the ligand **L**_**10**_ afforded **3z** in a slightly improved 42% ee. It turned out that a phenyl group on the oxazoline could cooperate with the C4 benzylsulfonyl group better, as **L**_**11**_ delivered a better 64% ee for **3aa**. This result promoted us to increase the steric hindrance of the benzyl moiety of the sulfonyl group on the pyridine C4 position to reinforce face discrimination. Gratifyingly, with the phenyl group being enlarged to a bulkier 3,5-dimethoxyphenyl, 3,5-ditrifluoromethylphenyl, or 3,5-di-*tert*-butylphenyl group, the corresponding ligand **L**_**12-14**_ gradually enhanced the ee value of **3aa** to 79%, 87% and 90%, respectively. These results confirmed that introducing a bulky C4 shielding group is a flexible solution to form a sterically restricted chiral pocket to achieve challenging asymmetric reactions unattainable by traditional PYBOX ligands.

### Substrate scope

**Fig. 3:**
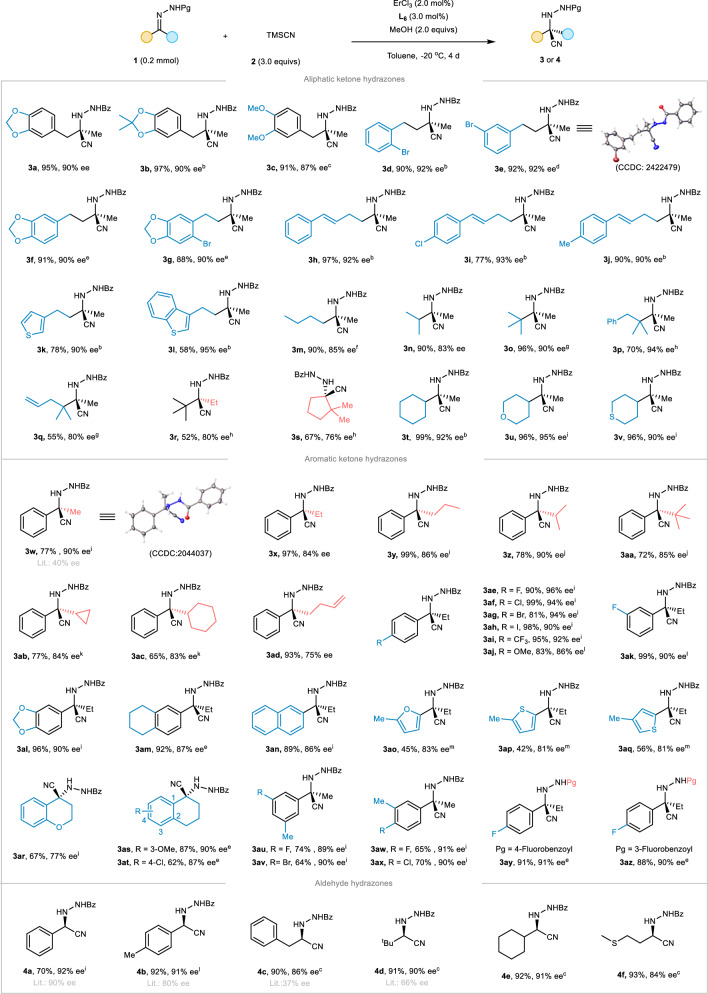
Scope of reaction with hydrazones^a^. ^a^ Isolated yield. The ee values were determined by performing chiral HPLC analysis. ^b^ Using ErCl_3_ (10 mol%) and **L**_**6**_ (15 mol%), ^*n*^Bu_2_O/toluene (1/1, v/v) as solvent, HFIP as additive. ^c^ Using ErCl_3_ (5.0 mol%) and **L**_**6**_ (7.5 mol%). ^d^ at –25 °C. ^e^ Using ErCl_3_ (10.0 mol%) and **L**_**6**_ (15 mol%). ^f^ Using ErCl_3_ (10.0 mol%) and **L**_**6**_ (15.0 mol%) ^*n*^Bu_2_O as solvent, HFIP as additive. ^g^ Using ErCl_3_ (10.0 mol%) and **L**_**8**_ (15.0 mol%) at 0 °C. ^h^ Using ErCl_3_ (15.0 mol%) and **L**_**8**_ (22.5 mol%), 6 days. ^i^ Using ErCl_3_ (10.0 mol%) and **L**_**6**_ (15.0 mol%) at 0 °C. ^j^ Using ErCl_3_ (10.0 mol%) and **L**_**14**_ (15.0 mol%) at 0 °C. ^k^ Using ErCl_3_ (15.0 mol%) and **L**_**14**_ (22.6 mol%), 6 days. ^l^ Using ErCl_3_ (5.0 mol%) and **L**_**6**_ (7.5 mol%) at 0 °C. ^m^ Using ErCl_3_ (15.0 mol%) and **L**_**4**_ (22.5 mol%) at 0 °C. The absolute configuration of (*R*)-**3e** and (*R*)-**3w** was determined by X-ray; for details, see Section 11 of SI.

With the optimal conditions established, the substrate scope of the sulfonyl-PYBOX-enabled enantioselective cyanation of ketone hydrazones was evaluated. Initially, aliphatic ketone hydrazones with diverse α-substituents were examined. As shown in Fig 3, α-benzyl hydrazones performed well, affording **3a**–**c** with 87–90% ee. Various benzylacetone-derived hydrazones underwent the reaction smoothly, furnishing the desired chiral hydrazines **3d**–**g** in 88–92% yield and 90–92% ee, regardless of the nature or position of the substituents on the phenyl group. Linear aliphatic ketone hydrazones containing an alkene moiety also reacted efficiently, producing chiral hydrazines **3h**–**j** in 77–97% yield and 90–93% ee. Notably, 3-thienyl- and 3-benzothienyl-substituted hydrazones were effective substrates, delivering the corresponding hydrazines **3k** and **3l** in 90% and 95% ee, respectively. The 2-heptanone-derived hydrazone provided the corresponding product **3m** in 85% ee. α-Branched ketone hydrazones were also viable substrates, furnishing the desired chiral hydrazines **3n**–**q** in 55–96% yield and 80–94% ee. Additionally, the α-ethyl hydrazone afforded the desired **3r** in 52% yield and 80% ee. The performance of cyclic ketone hydrazone **1s** was also investigated, and the desired hydrazine was obtained in 76% ee. Finally, hydrazones with α-cyclohexyl, α-tetrahydropyranyl, and α-tetrahydrothiopyranyl groups also proceeded smoothly, affording **3t**–**v** with 90–95% ee.

Subsequently, the enantioselective cyanation of aromatic ketone hydrazones was conducted. The acetophenone-derived hydrazone afforded **3w** in 77% yield with 90% ee. Phenyl ethyl and phenyl propyl ketone-derived hydrazones were also effective substrates, providing **3x** and **3y** in 97% and 99% yield, respectively. Hydrazones bearing an α-branched alkyl moiety reacted efficiently as well, producing chiral hydrazines **3z**–**ac** in 65–78% yield and 83–90% ee. The hydrazone **1ad** containing a terminal alkene moiety proved to be a viable substrate as well, giving the desired **3ad** in 93% yield. Next, the influence of the aromatic moiety on the reaction was investigated. Aryl ethyl ketone-derived hydrazones, regardless of the substituent’s nature or position on the phenyl ring, reacted smoothly to afford the corresponding adducts **3ae**–**am** in 81–99% yield and 86–96% ee. Additionally, the enantioselective cyanation of hydrazones bearing α-naphthyl, α-furyl, and α-thienyl groups was also investigated; all substrates proceeded smoothly to give **3an**–**aq** in 81–86% ee.

Notably, cyclic ketone hydrazones **1ar**–**1at** also reacted efficiently, furnishing the desired adducts **3ar**–**at** in 62–87% yield and 77–90% ee. Ketone hydrazones with disubstituted phenyl rings were suitable substrates as well, yielding the corresponding products **3au**–**ax** in 64–74% yield and 89–91% ee. Furthermore, the cyanation of ketone hydrazones bearing *N*−4-fluorobenzoyl or 3-fluorobenzoyl groups was also attempted, affording chiral **3ay** and **3az** in 91% and 90% ee, respectively.

To further showcase the power of the sulfonyl-PYBOX ligand **L**_**6**_ in this cyanation reaction, some aldehyde-hydrazone substrates were also undertaken, particularly those that worked less enantioselectively when using **L**_**1**_ as the ligand. Under standard conditions, generally higher ee values were obtained in the cyanation of some α-aromatic or α-aliphatic aldehyde hydrazones, giving the corresponding chiral products **4a**–**d** with 86–92% ee, which surpassed the results reported in the literature^35^. Notably, the use of **L**_**6**_ significantly improved the enantioselectivity of the cyanation of 2-phenylacetaldehyde or α-tert-butyl aldehyde-derived hydrazones from 37% and 66% ee to 86% and 90% ee, respectively. Moreover, α-cyclohexyl and methylthioethyl-substituted aldehyde hydrazones also worked well to give the desired adducts **4e** and **4f** in 91% and 84% ee, respectively.

### Mechanistic investigation

**Fig. 4:**
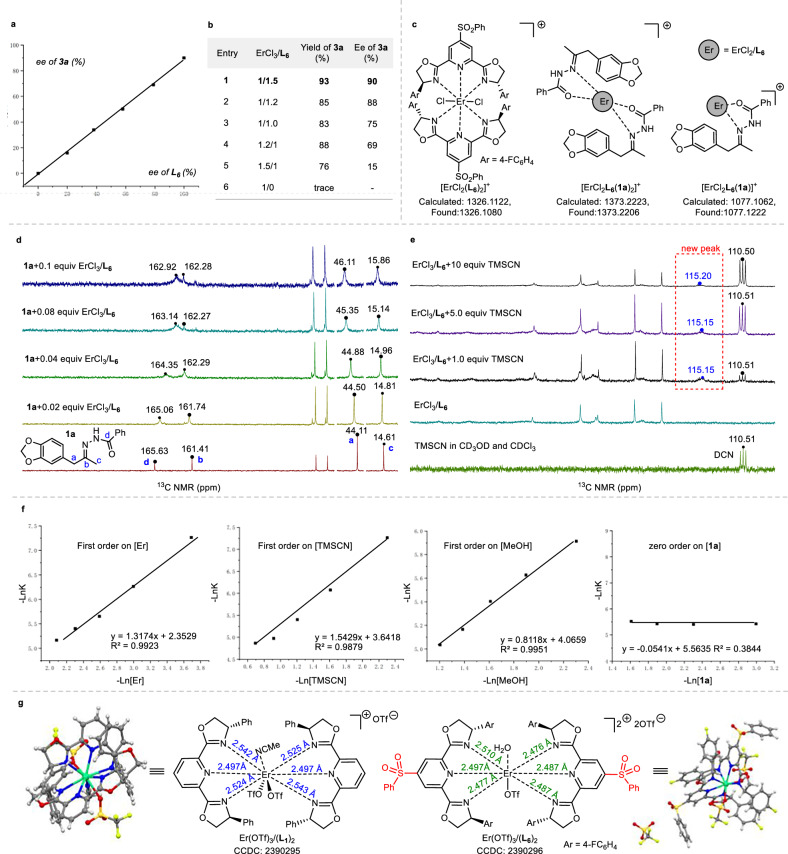
Mechanistic studies. **a** Nonlinear effects study. **b** Variations of ErCl_3_/**L**_**6**_ ratios. **c** HRMS analysis. **d** ^13^C NMR study of ErCl_3_/**L**_**6**_ with **1a**. **e** ^13^C NMR study of ErCl_3_/**L**_**6**_ with TMSCN. **f** Kinetic experiments. **g** X-ray structure of Er(OTf)_3_/(**L**_**1**_)_2_ and Er(OTf)_3_/(**L**_**6**_)_2_.

The excellent enantioselectivity achieved by the sulfonyl-PYBOX **L**_**6**_ in the Er-catalyzed highly enantioselective cyanation of hydrazones is very impressive, intriguing us to conduct the mechanistic studies to shed light on the underlying mechanism, and in particular the role of the C4 sulfonyl group on the pyridine of PYBOX in reinforcing face discrimination (Fig. 4). First, a nonlinear effect study was conducted to investigate the possible reactive chiral Er(III) species involved in the reaction. A linear relationship between the ee value of PYBOX **L**_**6**_ and that of **3a** was observed (Fig. 4a), suggesting the involvement of a monomeric Er-complex in the enantio-discrimination step. The influence of the Er/**L**_**6**_ ratio on the reaction outcome was also examined, indicating that the ideal ratio was approximately 1:1.5 (Fig. 4b). This effect indicated that one chiral ligand may be sufficient for generating the monomeric Er-complex. A HRMS analysis was conducted to detect the possible Er-complexes during the reaction course (Fig. 4c). Upon mixing ErCl_3_ and **L**_**6**_, the formation of the ErCl_3_/(**L**_**6**_)_2_ complex was detected, which is consistent with the X-ray analysis shown in Fig. 4g. After adding hydrazone **1a**, the formation of ErCl_3_/**L**_**6**_/(**1a**)_2_ and ErCl_3_/**L**_**6**_/**1a** was observed, indicating the binding of **1a** to the monomeric Er-complex.

Subsequently, the interaction between hydrazone **1a** and the chiral Er-complex was studied by performing ¹³C NMR analysis (Fig. 4d). When ErCl_3_/**L**_**6**_ was added to a solution of **1a** in CD_3_OD/CDCl_3_ (v/v, 1/1), the characteristic peaks for C^a^, C^c^, and C^b^, attributed to the α-carbon and imine-carbon of the hydrazone moiety, exhibited significant downfield shifts from 44.11, 14.61, and 161.41 ppm to 46.11, 15.86, and 162.28 ppm, respectively. Meanwhile, the carbonyl carbon (C^d^) of the benzoyl group displayed an upfield shift from 165.63 to 162.92 ppm. These changes implied that hydrazone **1a** might coordinate to the ErCl_3_/**L**_**6**_ complex in a bidentate fashion, with both the nitrogen of the C=N bond and the benzoyl group binding to Er(III)^71^. Additionally, the interaction between TMSCN and the ErCl_3_/**L**_**6**_ complex was also studied by ^13^C NMR (Fig. 4e). The conversion of TMSCN to DCN in the presence of CD_3_OD was confirmed by observing a characteristic peak at 110.51 ppm. Furthermore, adding this solution to a solution of the ErCl_3_/**L**_**6**_ complex in CD_3_OD/CDCl_3_ (v/v, 1/1) resulted in the appearance of a new peak at 115.2 ppm. This result suggests the possible formation of isocyanide species resulting from the binding of cyanide to Er(III), which was also supported by the IR spectroscopy analysis (for details, see Part 8 in the SI)^86,87^. Finally, kinetic experiments revealed first-order dependencies on the chiral ErCl_3_/**L**_**6**_ complex, TMSCN, and MeOH, and a zero-order dependency on hydrazone **1a** (Fig. 4f). These results suggest that the possible reactive species of the reaction was a monomeric Er(III) complex consisting of equal amounts of the chiral ligand **L**_**6**_, ErCl_3_, hydrazone **1a** and at least one molecule of cyanide^88^.

Furthermore, single crystals of complexes formed from Er(OTf)_3_ with unmodified ligand **L**_**1**_ and sulfonyl-PYBOX **L**_**6**_ were successfully obtained. As shown in Fig. 4g, the unmodified PYBOX **L**_**1**_ forms a nine-coordinate Er(OTf)_3_/(**L**_**1**_)_2_ complex bound with two triflates and one acetonitrile; however, an eight-coordinate complex of Er(OTf)_3_/(**L**_**6**_)_2_ with one triflate and one water bound to the metal was observed in the case of sulfonyl-PYBOX **L**_**6**_. These data implied an eight-coordinated Er(III) in the reactive catalyst–substrate complex when **L**_**6**_ was used. Based on the above results, the DFT calculations on the possible transition-state (TS) models were tried, but failed to obtain properly converged TS models despite intensive attempts (for details, see Part 8 in the SI). It is worth mentioning that the DFT theoretical study into Er(III)-catalyzed enantioselective synthesis is not so trivial as it first appears to be, and has not been reported to date, to our knowledge^89,90^. While the structure of the catalyst–substrate complex was still unclear, the accumulated data suggested the monomeric tridentate and bidentate coordination of **L**_**6**_ and **1a** to Er(III), respectively, as well as the coordination of one cyanide and the other two unclarified ligands, such as cyanide, chloride, water, or a hydrazone. Importantly, the presence of a C4 sulfonyl group on the pyridine of PYBOX ligand could adjust the steric effect to change the number of ligands that an Er(III) can accept, but enhance the face discrimination of the C=N double bonds for much better enantioselectivity. These results clearly support our hypothesis that compared with traditional PYBOX, our sterically confined ligands bearing C4 bulky shielding group-derived monomeric metal complex could form a more sterically confined chiral pocket for better enantiofacial control (Fig. 2a). This outcome indicates the potential application of our ligands in other challenging asymmetric reactions unattainable by traditional PYBOX.

### Application explorations

**Fig. 5:**
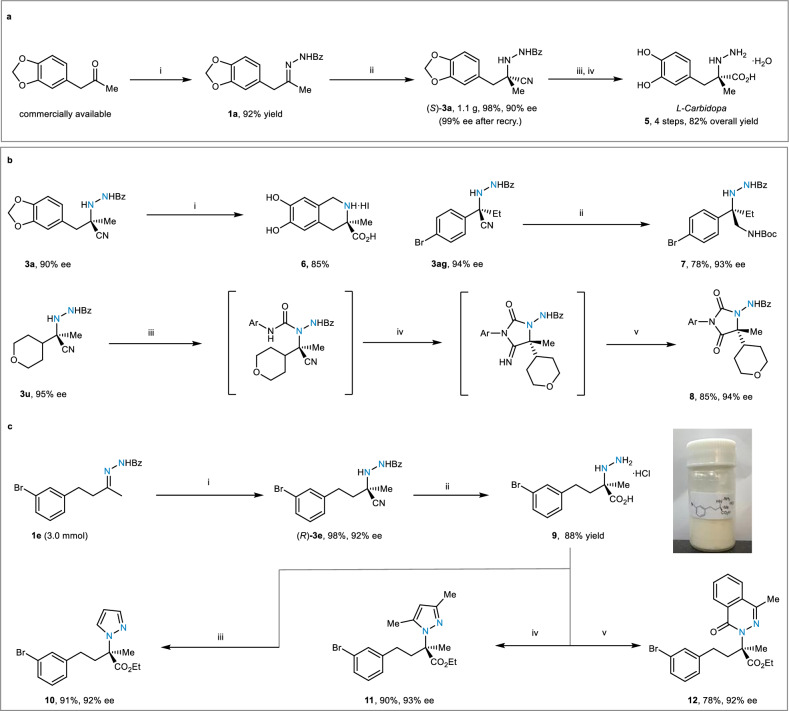
Synthetic applications. **a** Total Synthesis of *L-Carbidopa*: i) benzoyl hydrazine (1.1 equiv), AcOH (10 mol%), EtOH, rt, 8 h; ii) standard conditions with (*R*,*R*)-**L**_**6**_ as ligand; iii) concentrated HCl/EtSH (v/v, 1:2), reflux, overnight; iv) ^*i*^PrOH, dimethylamine (2 M in THF). **b** Transformation of hydrazino nitriles: i) HI (conc.), reflux, 8 h; ii) NiCl_2_ (1.5 equiv), Boc_2_O (3.0 equiv), NaBH_4_ (12.0 equiv), MeOH, rt, 12 h; iii) 4-fluorophenyl isocyanate (1.2 equiv), CH_2_Cl_2_, rt, 24 h; iv) NaOEt (20 mol%), EtOH, rt, 2 h; v) HCl (3 M), 50 °C, 10 h. **c** Synthesis of azacycles bearing quaternary stereocenters: i) standard conditions; ii) HCl (conc.), reflux, 24 h. iii) 1,1,3,3-tetraethoxypropane (1.5 equiv), PTSA (4.0 equiv), EtOH, 140 °C, 24 h; iv) acetylacetone (1.1 equiv), PTSA (0.5 equiv), EtOH, 140 °C, 24 h; v) 2-acetylbenzoic acid (1.1 equiv), PTSA (0.5 equiv), EtOH, 140 °C, 24 h. Isolated yield. The ee values were determined by performing chiral HPLC analysis. PTSA *p*-toluenesulfonic acid.

Having established the highly enantioselective cyanation of ketone hydrazones, we first applied it for the catalytic enantioselective total synthesis of *L-carbidopa* (Fig. 5a). Starting from commercially available 1-(benzo[*d*][1,3]dioxol-5-yl)propan-2-one, ketone hydrazone **1a** was obtained in 92% yield. A gram-scale cyanation of **1a**, mediated by 2.0 mol% (*R*,*R*)-**L**_**6**_/ErCl_3_ complex, gave 1.1 g (*S*)-**3a** in 98% yield with 90% ee (99% ee after recrystallization). The subsequent hydrolysis, deprotection, and pH adjustment furnished the desired *L-carbidopa*. This synthetic route enabled the facile access of *L-carbidopa* in 82% overall yield by only four steps, constituting the shortest enantioselective catalytic total synthesis route, whereas previous methods based on the electrophilic amination of α-methyl-β-ketoesters required at least seven steps with a 50% overall yield^62^.

The versatility of the resulting C^α^-tetrasubstituted α-hydrazino nitriles was further shown by various transformations (Fig. 5b). For instance, the cleavage of the N–N bond in **3a** using hydroiodic acid afforded cyclic quaternary α-amino acid salt **6** in 85% yield. The reduction of the cyano group in **3ag** with NiCl_2_/NaBH_4_ delivered chiral α-amino hydrazine **7** in 78% yield. Additionally, the nucleophilic addition of **3 u** to isocyanates, followed by cyclization and hydrolysis, provided chiral imidazolidine-2,4-dione **8** in 85% yield. Furthermore, leveraging the cyclization of the hydrazine moiety, the synthesized chiral α-hydrazino nitriles could be transformed into various azacycles bearing quaternary stereocenters, such as chiral pyrazole and diazine derivatives, which are highly desirable for drug development but challenging to access via known methods (Fig. 5c). For example, in the presence of *p*-toluenesulfonic acid, hydrazino acids **9** underwent condensation with 1,1,3,3-tetraethoxypropane, acetylacetone, and 2-acetylbenzoic acid to afford chiral pyrazoles **10,** **11**, and benzodiazine **12** in 78–91% yields, with retained enantioselectivity.

**Fig. 6:**
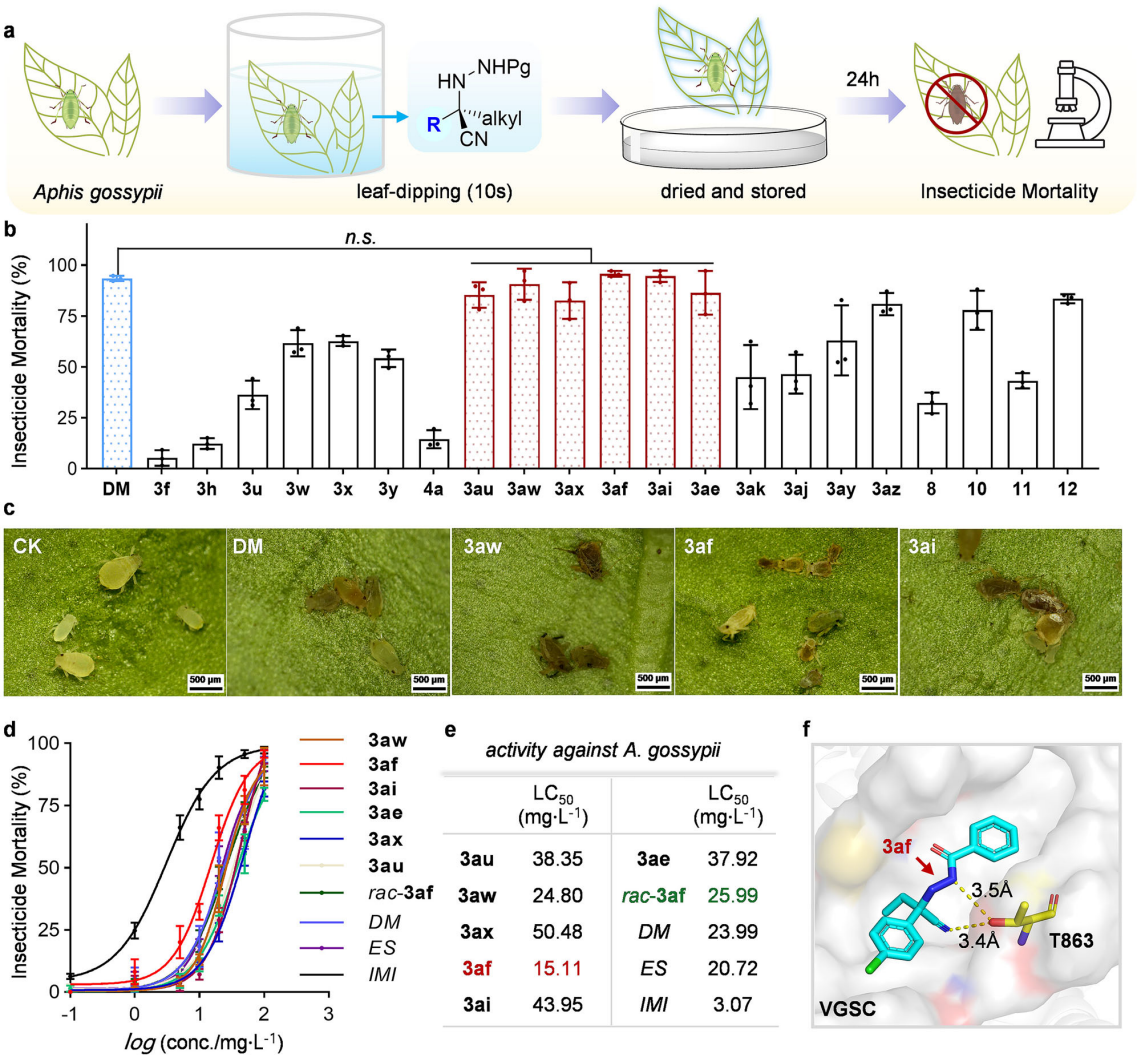
Insecticidal activity against *Aphid gossypii*. **a** The leaf-dipping method was used to evaluate the toxicity of hydrazino nitriles against *A. gossypii* under laboratory conditions. **b** Evaluation of the insecticidal activity of representative compounds at 100 mg∙L^−1^ with *Deltamethrin* (*DM*) as the positive control. Error bars represent mean ± SD (*n* = 3). The significant difference analysis was calculated by using *t* tests of GraphPad Prism (Table S3). **c** 3DSuperDepth-of-FieldMicroscope images of the insecticidal effect of **CK, DM,** **3af,** **3ai,** **3aw**. CK: Control Check. Scale bar = 500 μm. **d** Curve of dose-dependent insecticidal activity (gradient concentration: 0, 0.1, 1, 5, 10, 20, 50, and 100 mg∙L^−1^). Error bars represent mean ± SD (*n* = 3). **e** The LC_50_ values of hydrazino nitriles against *A. gossypii*. The 95% confidence intervals (**3au**: 26.4–65.57; **3aw**: 19.69–31.24; **3ax**: 34.88–73.00; **3af**: 11.70–19.53; **3ai**: 33.25–58.09; **3ae**: 22.11–65.05; *rac*-**3af**: 17.81–37.93; *DM*: 13.51–42.60; *ES*: 15.81–27.16; *IMI*: 2.54–3.67). **f** Binding modes of **3af** and *ES* in the VGSC binding pocket. In the **3af**-bound model, the cyano group of **3af** engages in hydrogen-bonding interactions with T863, indicated by yellow dashed lines.

These optically active α-hydrazino nitriles are not only valuable chiral synthons to C^α^-tetrasubstituted α-hydrazino acid derivatives, including azacycles, but are interesting targets for developing agrochemicals. Considering the importance of chiral cyano-substituted insecticides^91^ (e.g., *Deltamethrin* (*DM*), *Esfenvalerate* (*ES*), and *Cypermethrin*) in the prevention and control of plant pests, we therefore examined the potential applications of such chiral hydrazino nitriles in insecticidal activity. *Aphis gossypii* (*A. gossypii*), a pest insect that severely impairs cotton seedling growth, was chosen as the test organism. The leaf-dipping method was used to evaluate the toxicity of hydrazino nitriles against *A. gossypii* (nonresistant population), and *Deltamethrin* (*DM*) was used as the positive control (Fig. 6a). Based on the modifiable sites of the core structure of α-hydrazino nitriles, the compounds (**3f,** **3h,** **3u,** **3w,** **3x,** **3y, 3ae,** **3af,** **3ai-ak,** **3au,** **3aw,** **3ax,** **3ay,** **3az,** **4a,** **8,** **10,** **11**, and **12**) were selected for the initial investigation of activity against *A. gossypii* (Fig. S11). It was found that aryl-substituted hydrazino nitriles **3au,** **3aw,** **3ax,** **3af,** **3ai** and **3ae** exhibited insecticidal activity comparable to that of the positive control *DM* at a concentration of 100 mg∙L^−1^(Fig. 6b). In particular, electron-deficient aryl-substituted compounds **3af,** **3ai**, and **3aw** achieved insect mortalities of over 90%. The insecticidal effect images of **3af,** **3ai**, and **3aw** against *A. gossypii* are shown in Fig. 6c. Then, the LC_50_ values of candidate compounds **3au,** **3aw,** **3ax,** **3af,** **3ai** and **3ae** were determined, with *DM*, *ES*, and *Imidacloprid* (*IMI*) serving as positive controls. The dose–response relationship between the logarithm of compound concentration and insect mortality is shown in Fig. 6d. Hydrazino nitrile **3af** exhibited the lowest LC_50_ value (15.11 mg∙L^−^^1^) against *A. gossypii*, outperforming **3au** (38.35 mg∙L^−1^), **3aw** (24.80 mg∙L^−1^), **3ax** (50.48 mg∙L^−1^), **3ai** (43.95 mg∙L^−1^), and **3ae** (37.92 mg∙L^−1^). The LC_50_ value of **3af** was lower than those of the positive controls *DM* (23.99 mg∙L^−1^) and *ES* (20.72 mg∙L^−1^), but higher than that of *IMI* (3.07 mg∙L^−1^). Furthermore, the LC_50_ value of the enantiopure form of **3af** was significantly lower than that of its racemic counterpart, highlighting the critical role of chirality in insecticide design (Fig. 6e)^91^.

To further elucidate the insecticidal mechanism of α-hydrazino nitrile **3af**, we conducted computational analysis. Given that compound **3af** shares a similar scaffold with pyrethroid insecticides, and that *ES* shows the highest structural similarity to **3af** according to Morgan fingerprint analysis, we hypothesized that compound **3af** targets the same voltage-gated sodium channel (VGSC) as *ES*. Based on this hypothesis, molecular docking and molecular dynamics simulations were performed focusing on VGSC. The results indicate that compound **3af** can form a stable binding mode with VGSC, preliminarily supporting VGSC as the target for this class of compounds (Fig. 6f and Fig. S12 in SI). Further investigation of the insecticidal mechanism will be carried out in subsequent studies.

## Discussion

In summary, we have developed the highly enantioselective nucleophilic addition reaction of ketone hydrazones. Accordingly, ErCl_3_ in combination with a sterically confined PYBOX, featuring a C4 sulfonyl group at the pyridine and 2-fluorophenyl group at the oxazoline, was identified as a powerful catalyst for the cyanation of both aryl and aliphatic ketone hydrazones, allowing the diverse synthesis of C^α^-tetrasubstituted α-hydrazino nitriles in up to 96% ee. This result demonstrates that introducing a bulky shielding group at the C4 position of pyridine may improve the chiral pocket of PYBOX-derived monomeric metal complexes. The latter effect is conducive to attaining a better enantiofacial control in some challenging asymmetric reactions that involve monomeric metal center but traditional PYBOX fails to yield excellent enantioselectivity. Thus, the developed method contributes to the shortest enantioselective catalytic total synthesis of *L*-*carbidopa*, an important drug for Parkinson’s disease, in just four steps. The resulting chiral α-hydrazino nitriles are multifunctional synthons to various α-tertiary hydrazine derivatives and azacycles, as well as interesting targets for the development of agrochemicals, as shown by the excellent insecticidal activity of compound **3af** against *A. gossypii* (LC_50_ = 15.11 mg∙L^−1^), rivaling commercial insecticides. We believe that the proposed methodology will be of broad interest to researchers in medicinal, agrochemical, and materials science.

## Methods

General procedure for the synthesis of chiral α-tertiary hydrazines **3**. To a 5 mL vial equipped with a screw-cap were added ErCl_3_ (0.004 mmol, 2.0 mol%) and PYBOX ligand **L**_**6**_ (0.006 mmol, 3.0 mol%), followed by 2.0 mL of anhydrous Toluene. The solution was stirred at 25 *°*C for 2.0 h, and then hydrazones **1** (0.2 mmol, 1.0 equiv) and were added. After cooling the mixture to −20 *°*C for 0.5 h, TMSCN (0.6 mmol, 3.0 equiv) and MeOH (0.4 mmol, 2.0 equiv) were added. The resulting mixture was stirred at −20 *°*C for 4 days till almost full conversion of **1** by TLC analysis, and then quickly passed through a short column to remove the catalyst using EtOAc/CH_2_Cl_2_ as the eluent. The thus-obtained solution was concentrated under reduced pressure, and the residue was directly subjected to column chromatography purification using petroleum ether/EtOAc (10:1 to 3:1, v/v) as the eluent to afford the desired chiral α-tertiary hydrazines **3**.

General procedure for the insecticidal activity evaluation of chiral hydrazino nitriles against *A. gossypii*. The stock solution of chiral hydrazino nitriles and *Deltamethrin* (10 g ∙ L^−1^ in DMSO) was diluted using an aqueous solution of 0.1% (w/v) Tween 80 to six concentrations. Each concentration was replicated thrice. Individual cotton leaves are infested with about 30–60 *A. gossypii* were dipped in hydrazino nitrile solutions for 10 s and dried on blotting paper. Then individual leaves were transferred to 60-mm petri dishes which contain a water-moistened filter paper. After further air-drying, the petri dishes were sealed with parafilm. All petri dishes were stored in an incubator at 25 ± 1 *°*C, 70 ± 10% RH and a 14:10 h light:dark photo period for 24 h until mortality was assessed. Control aphids treated with 0.1% tween 80 aqueous solution containing DMSO (20 mg∙L^−1^) showed mortality <10% in all bioassays. All data on insecticidal activity were calibrated based on the control group.

## Supplementary information

Supplementary information

Transparent Peer Review file

## Data Availability

The data supporting the findings of this study are available within the paper and its Supplementary Information. The X-ray crystallographic data for structures reported in this study have been deposited at the Cambridge Crystallographic Data Centre (CCDC), under deposition number **1b** (CCDC 2221754), **3e** (CCDC 2422479), **3w** (CCDC 2044037), ErCl_3_/**L**_**1**_ (CCDC 2390295) and ErCl_3_/**L**_**6**_ (CCDC 2390296). These data can be obtained free of charge from The Cambridge Crystallographic Data Centre via www.ccdc.cam.ac.uk/data_request/cif. All source data in support of the findings of this study are available within the Article and its Supplementary Information or from the corresponding author upon request.
