# Supplementary material for: Sulfonyl-PYBOX/ErCl3 complex enable highly enantioselective synthesis of α,α-dialkyl and α-alkyl-α-aryl hydrazinonitriles
Source: Nat Commun. 2026 Jun 18;17:7688. doi: 10.1038/s41467-026-74600-0 (PMC13434049; doi:10.1038/s41467-026-74600-0)
Supplement: Supplementary file 1 [file 41467_2026_74600_MOESM1_ESM.pdf]

Sulfonyl-PYBOX/$ErCl_3$ Complex Enable Highly Enantioselective Synthesis of α,α-Dialkyl and α-Alkyl-α-aryl Hydrazinonitriles

Yi Gong[1+], Zheng Zhang[1+], Qiang Cao[1], Cai Wang[1], Min-Chuan Jiang[2], Zhi-Heng Qiu[1], Xiao-Jing Le[1], Yu-Jie Ye[1], Tao Wang[1], Yun-Hao Tang[1], Ying Zhang[1], Haoran Zhao[2], Li Cui[2], Feng Zhou[1★], Bo-Shuai Mu[2★], Xin Wang[3] and Jian Zhou[1,4★]

[1] State Key Laboratory of Petroleum Molecular & Process Engineering, Shanghai Engineering Research Center of Molecular Therapeutics and New Drug Development, Shanghai Key Laboratory of Green Chemistry and Chemical Processes, School of Chemistry and Molecular Engineering, East China Normal University, Shanghai 200062, China.
E-mail: fzhou@chem.ecnu.edu.cn; jzhou@chem.ecnu.edu.cn
[2] State Key Laboratory for Biology of Plant Diseases and Insect Pests, Institute of Plant Protection, Chinese Academy of Agricultural Sciences, Beijing 100193, China.
E-mail: muboshuai@caas.cn;
[3] College of Chemistry, Sichuan University, Chengdu, 610064, China.
[4] State Key Laboratory of Organometallic Chemistry, Shanghai Institute of Organic Chemistry, Shanghai 200032, China.
+ Y. Gong and Z. Zhang contributed equally to this work.

Table of Contents	Page
1. General information	2
2. General procedure for the synthesis of new PYBOX ligands **L5**-**L14**	3-6
3. General procedure for the synthesis of hydrazones **1**	7-21
4. Condition optimization	22-25
5. Enantioselective Er-catalyzed cyanation of hydrazone	26-46
6. Total synthesis of *L*-carbidopa	47-48
7. Transformation of hydrazino nitrile products	49-53
8. Mechanistic studies	54-63
9. Insecticidal activity studies	64-65
10. Computational analysis of insecticidal mechanism	65-67
11. X-ray crystallographic data of (*E*)-**1b**, (*R*)-**3e**, (*R*)-**3w**, $ErCl_3$/**L1** and $ErCl_3$/**L6**	67-112
12. ^{1}H, ^{13}C, and ^{19}F NMR spectra	113-397
13. HPLC spectra	398-461
14. References	462

1. General information

Reactions were monitored by thin layer chromatography using UV light to visualize the course of reaction. Purification of reaction products was carried out by flash chromatography on silica gel. Chemical yields refer to pure isolated substances. Infrared (IR) spectra were obtained using SHIMADZU TRT racer-100. The $[\alpha]_D$ was recorded using Anton Paar MCP 5500. Chiral HPLC analysis was performed on a Shimadzu LC-20AD instrument using Daicel chiral columns at room temperature and a mixture of HPLC-grade hexanes and isopropanol as eluent. ^{1}H, ^{13}C and ^{19}F spectra were obtained using a Bruker DPX-400 and Bruker DPX-500 spectrometer. Chemical shifts were reported in ppm from $CDCl_3$, DMSO-d_6 or D_2O with the solvent resonance as the internal standard. The following abbreviations were used to designate chemical shift multiplicities: s = singlet, d = doublet, t = triplet, q = quartet, m = multiplet, br = broad.

Unless mentioned, all reactions were performed under an atmosphere of N_2. All solvents were rigorously dried and purified prior to use under inert atmosphere. $ErCl_3$ (99.99%) were purchased from Alfa-Aesar and used as received. Other metal salts were purchased from Aldrich or Strem. Chiral PYBOX ligands **L_1-L_4** were prepared following literature methods.[1] Hydrazones **1** was synthesized according literature reports with *E*-configuration.[2] As the eluent, the petroleum ether, EtOAc and CH_2Cl_2 were purchased from Shanghai Titan Scientific Co. Ltd without further purification.

List of abbreviation:

Entry	Chemical name	Abbreviation
1	Petroleum ether	PE
2	Ethyl acetate	EtOAc
3	Tetrahydrofuran	THF
4	N,N-Dimethylformamide	DMF
5	Diethylaminosulfur trifluoride	DAST
6	*p*-Toluenesulfonic acid	PTSA
7	2,2,2-Trifluoroethanol	TFE
8	1,1,1,3,3,3-Hexafluoro-2-propanol	HFIP

2. General procedure for the synthesis of new PYBOX ligands L_5-L_{14}

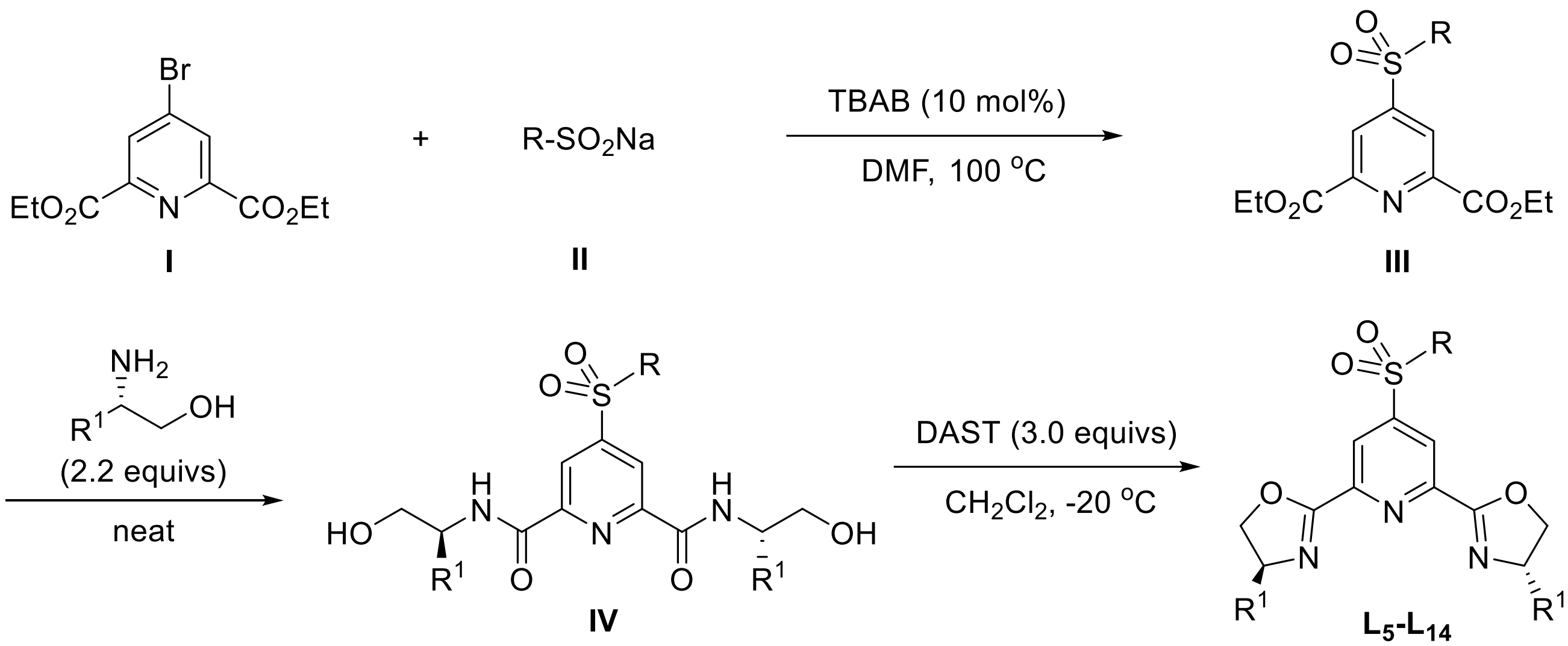

PYBOX ligands **L_5-L_{14}** were prepared following our previous methods.[3] Diethyl 4-bromopyridine-2,6-dicarboxylate **I** (2.0 mmol), sodium sulfinate **II** (2.2 equiv.) and tetrabutylammonium bromide (TBAB, 64.4 mg, 0.2 mmol) was added into a solution of DMF (30 mL). The resulting solution was heated to 100 °C with stirring under N_2 atmosphere for about 1 h. After the mixture was cooled down to room temperature, H_2O (30 mL) was added to the mixture, and brown solid immediately appeared. The intermediate **III** was obtained by filtration and used for next step without purification.

To a 10 mL sealed tube was added **III** and (*S*)-2-amino-2-arylethanol (2.2 equiv.), then the mixture was heated at 100 °C until full consumption of **III**, monitored by TLC analysis. The residue was recrystallized in MeOH/CH_2Cl_2 to afford **IV**.

To a flame-fried 25 mL Schlenk tube was added **IV** (0.5 mmol), followed by the addition of CH_2Cl_2 (20 mL). The resulting solution was cooled to -20 °C, followed by dropwise addition of DAST (253.8 mg, 1.5 mmol) over 5 min. The reaction was allowed to proceed at -20 °C for 8 h, and then quenched by saturated aqueous NH_4OH and diluted with H_2O (20 mL). The aqueous layer was extracted with CH_2Cl_2 (20 mL × 3). The organic phase was combined, dried with sodium sulfate, and concentrated under reduced pressure. The residue was subjected to column chromatography using CH_2Cl_2/EtOAc (20/1, v/v) as the elution to afford **L_5-L_{14}**.

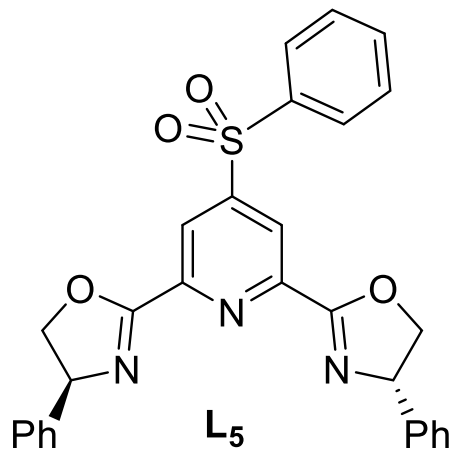

$\mathbf{L_5}$ was obtained in 55% yield as white solid.[3] ^{1}H NMR (400 MHz, $CDCl_3$): δ 8.77 (s, 2H), 8.03-8.01 (m, 2H), 7.67-7.63 (m, 1H), 7.59-7.54 (m, 2H), 7.41-7.37 (m, 4H), 7.34-7.30 (m, 6H), 5.48 (dd, J = 10.4, 8.7 Hz, 2H), 4.94 (dd, J = 10.4, 8.7 Hz, 2H), 4.45 (t, J = 8.7 Hz, 2H); ^{13}C NMR (100 MHz, $CDCl_3$): δ162.3, 151.9, 148.5, 141.1, 139.0, 134.5, 129.8, 128.9, 128.5, 128.0, 126.8, 123.3, 75.8, 70.5.

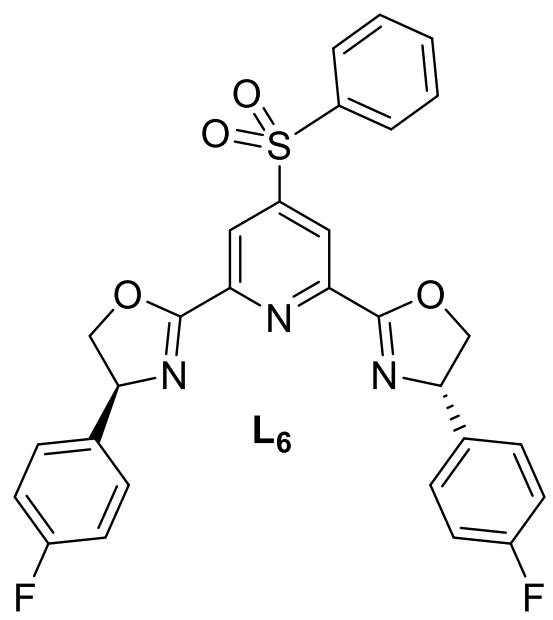

$\mathbf{L_6}$ was obtained in 50% yield as white solid. m.p. 180-182 ºC, ^{1}H NMR (400 MHz, $CDCl_3$): ^{1}H NMR (400 MHz, $CDCl_3$): δ 8.75 (s, 2H), 8.03-8.01 (m, 2H), 7.68-7.64 (m, 1H), 7.59-7.55 (m, 2H), 7.30-7.26 (m, 4H), 7.09-7.04 (m, 4H), 5.47 (dd, J = 10.4, 8.7 Hz, 2H), 4.93 (dd, J = 10.4, 8.8 Hz, 2H), 4.40 (t, J = 8.7 Hz, 2H); ^{13}C NMR (100 MHz, $CDCl_3$): δ 162.37 (d, J = 245.0 Hz), 162.36, 152.0, 148.4, 139.0, 136.9 (d, J = 3.2 Hz), 134.5, 129.8, 128.5, 128.4, 123.2, 115.7 (d, J = 21.6 Hz), 75.7, 69.8; ^{19}F NMR (376 MHz, $CDCl_3$): δ -114.19. IR (neat): 2360, 1640, 1508, 1329, 1157, 835, 603, 578 cm^{-1}; HRMS (ESI): Exact mass calcd for $C_{29}H_{21}F_2N_3NaO_4S$ [M+Na]$^+$: 568.1113, Found: 568.1124.

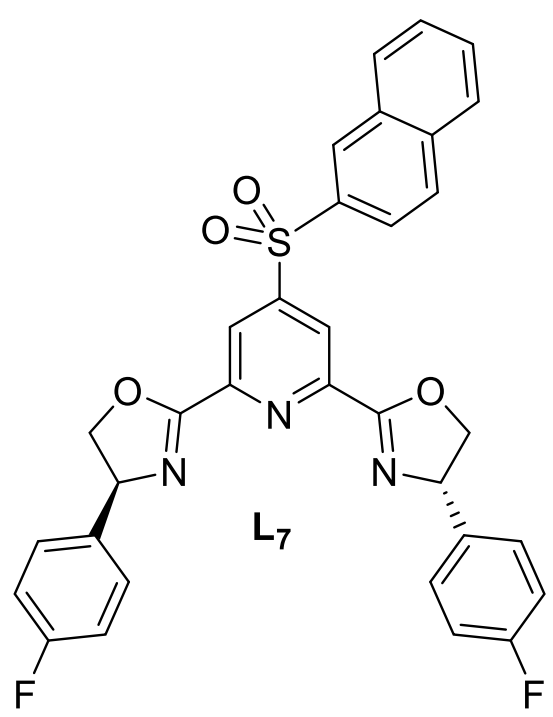

$\mathbf{L_7}$ was obtained in 47% yield as white solid. m.p. 200-202 ºC; ^{1}H NMR (400 MHz, $CDCl_3$): ^{1}H NMR (400 MHz, $CDCl_3$): δ 8.74 (s, 2H), 8.58 (d, J = 1.9 Hz, 1H), 7.97-7.92 (m, 2H), 7.86-7.83 (m, 2H), 7.63-7.58 (m, 2H), 7.23-7.20 (m, 4H), 7.02-6.97 (m, 4H), 5.41 (dd, J = 10.4, 8.7 Hz, 2H), 4.86 (dd, J = 10.4, 8.8 Hz, 2H), 4.33 (t, J = 8.7 Hz, 2H); ^{13}C NMR (100 MHz, $CDCl_3$): δ 162.40 (d, J = 245.0 Hz), 162.39, 152.1, 148.4, 136.9 (d, J = 3.2 Hz), 135.6 (d, J = 17.9 Hz), 132.2, 130.6, 130.3, 129.9, 129.6, 128.5 (d, J = 8.1 Hz), 128.0 (d, J = 2.8 Hz), 123.3, 122.6, 115.8 (d, J = 21.6 Hz), 75.7, 69.8; ^{19}F NMR (376 MHz, $CDCl_3$): δ -114.20. IR (neat): 2360, 1640, 1508, 1329, 1157, 1134, 835, 667, 569 cm^{-1}; HRMS (ESI): Exact mass calcd for $C_{33}H_{23}F_2N_3NaO_4S$ [M+Na]$^+$: 618.1270, Found: 618.1285.

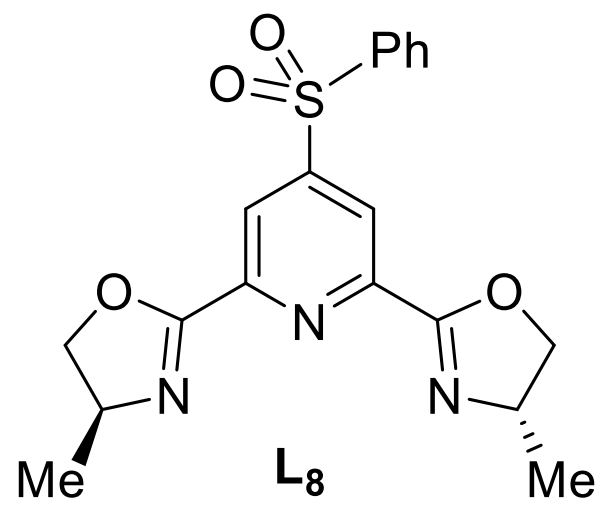

L_8

L8 was obtained in 35% yield as white solid. m.p. 175-177 °C; ^{1}H NMR (400 MHz, $CDCl_3$): δ 8.59 (s, 2H), 8.00 (d, *J* = 7.2 Hz, 2H), 7.64-7.54 (m, 3H), 4.61 (t, *J* = 9.2 Hz, 2H), 4.46 (t, *J* = 7.2 Hz, 2H), 4.07 (t, *J* = 8.0 Hz, 2H), 1.37 (d, *J* = 6.4 Hz, 6H); ^{13}C NMR (100 MHz, $CDCl_3$): δ 161.1, 151.7, 148.6, 139.1, 134.4, 129.7, 128.4, 122.6, 62.5, 21.2. IR (neat): 1638, 1447, 1329, 1157, 974, 719, 605 cm^{-1}; HRMS (ESI): Exact mass calcd for $C_{19}H_{20}N_3O_4S$ $[M+H]^+$: 386.1169, Found: 386.1162.

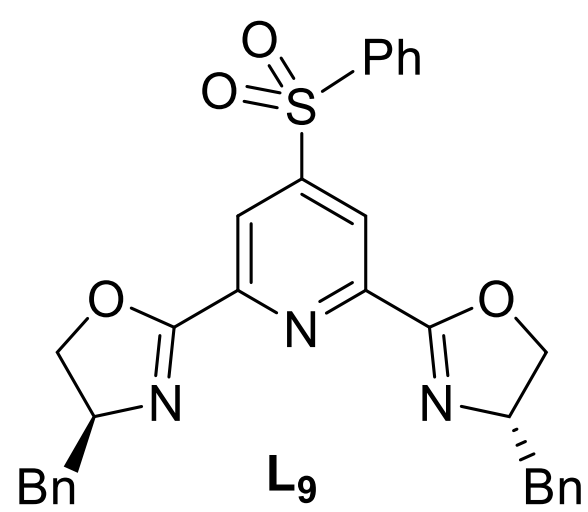

L_9

L9 was obtained in 40% yield as white solid. m.p. 185-186 °C; ^{1}H NMR (400 MHz, $CDCl_3$): δ 8.62 (s, 2H), 8.03 (d, *J* = 7.6 Hz, 2H), 7.66-7.56 (m, 3H), 7.33-7.22 (m, 10H), 4.69-4.66 (m, 2H), 4.47 (t, *J* = 9.2 Hz, 2H), 4.26 (t, *J* = 8.0 Hz, 2H), 3.29-3.25 (m, 2H), 2.78-2.72 (m, 2H); ^{13}C NMR (100 MHz, $CDCl_3$): δ 161.5, 151.7, 148.5, 139.0, 137.3, 134.4, 129.7, 129.1, 128.6, 128.3, 126.6, 122.6, 72.8, 68.1, 41.4. IR (neat): 2360, 1641, 1329, 1159, 974, 719, 606, 579 cm^{-1}; HRMS (ESI): Exact mass calcd for $C_{31}H_{28}N_3O_4S$ $[M+H]^+$: 538.1795, Found: 538.1789.

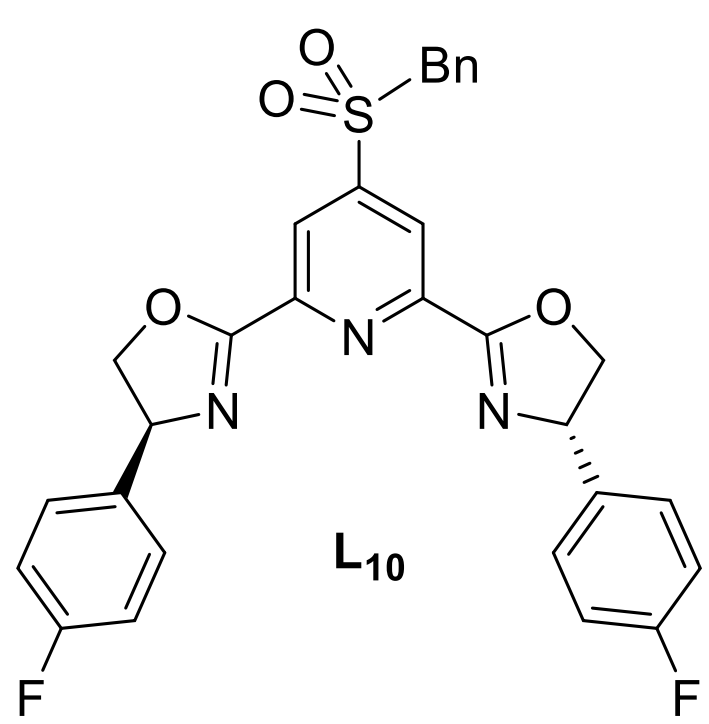

L_{10}

L10 was obtained in 38% yield as white solid. m.p. 196-198 °C; ^{11}H NMR (400 MHz, $CDCl_3$): δ 8.49 (s, 2H), 7.31-7.25 (m, 8H), 7.14 (d, *J* = 6.4 Hz, 2H), 7.08 (t, *J* = 8.4 Hz, 4H), 5.47 (t, *J* = 9.2 Hz, 2H), 4.93 (t, *J* = 8.8 Hz, 2H), 4.45-4.36 (m, 4H); ^{13}C NMR (100 MHz, $CDCl_3$): δ 162.4 (d, *J* = 245 Hz), 162.2, 148.1 (d, *J* = 2.7 Hz), 136.9 (d, *J* = 3.0 Hz), 130.9, 129.3, 129.0, 128.4 (d, *J* = 8.2 Hz), 126.4, 124.2, 115.7 (d, *J* = 21.5 Hz), 75.7, 69.7, 62.3; ^{19}F NMR (376 MHz, $CDCl_3$): δ -114.20. IR (neat): 1639, 1508, 1265, 1153, 976, 733, 624, 519 cm^{-1}; HRMS (ESI): Exact mass calcd for $C_{30}H_{24}N_3O_4F_2S$ $[M+H]^+$: 560.1450, Found: 560.1446.

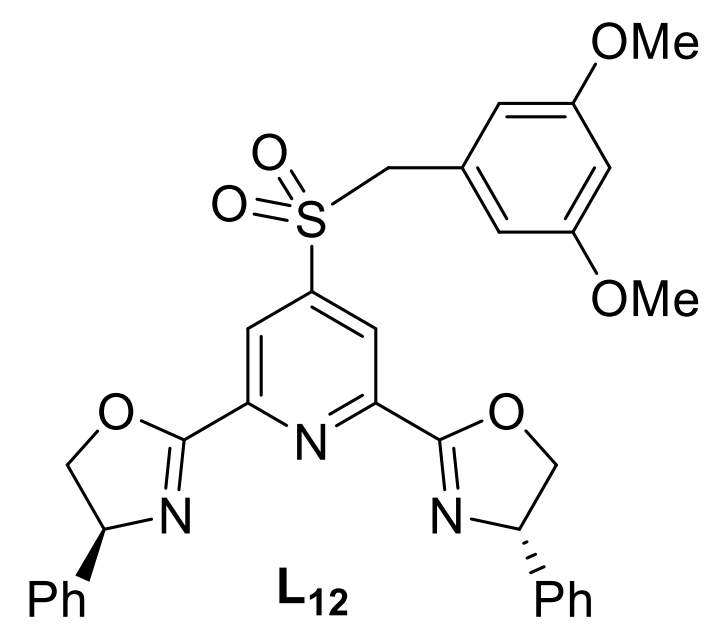

L12 was obtained in 23% yield as white solid. m.p. 203-205 ºC; ^{1}H NMR (400 MHz, $CDCl_3$): δ 8.57 (s, 2H), 7.41-7.29 (m, 10H), 6.39 (s, 1H), 6.30-6.29 (m, 2H), 5.48 (t, *J* = 9.6 Hz, 2H), 4.95 (t, *J* = 8.8 Hz, 2H), 4.44 (t, *J* = 8.8 Hz, 2H), 4.38-4.28 (m, 2H), 3.67 (s, 6H); ^{13}C NMR (100 MHz, $CDCl_3$): δ 162.2, 161.0, 148.2, 141.1, 128.9, 128.2, 128.0, 126.8, 124.2, 108.7, 101.6, 75.7, 70.4, 62.4, 55.4;. IR (neat): 1636, 1429, 1265, 1155, 924, 737, 698 cm^{-1}; HRMS (ESI): Exact mass calcd for $C_{32}H_{30}N_3O_6S$ [M+H]$^{+}$: 584.1850, Found: 584.1842.

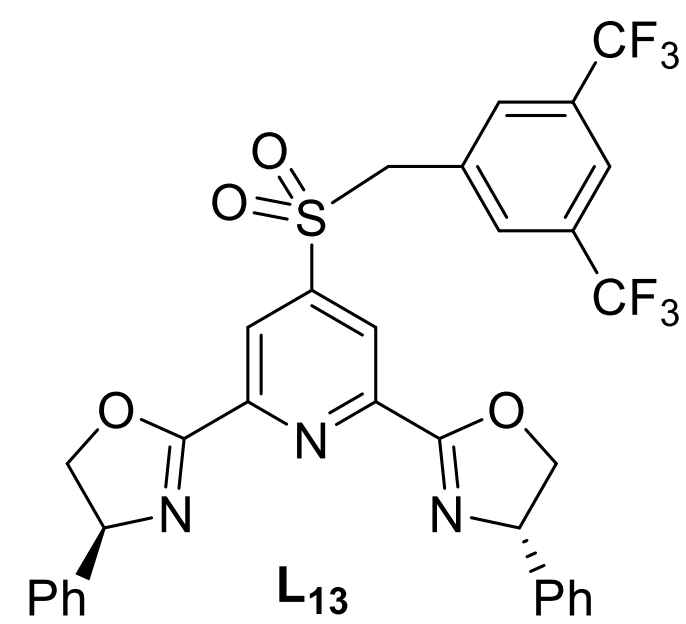

L13 was obtained in 32% yield as white solid. m.p. 199-201 ºC; ^{1}H NMR (400 MHz, $CDCl_3$): δ 8.60 (s, 2H), 7.88 (s, 1H), 7.67 (s, 2H), 7.31-7.41 (m, 10H), 5.50 (t, *J* = 9.6 Hz, 2H), 4.97 (t, *J* = 8.8 Hz, 2H), 4.61-4.46 (m, 4H); ^{13}C NMR (100 MHz, $CDCl_3$): δ 162.0, 148.7, 148.0 141.0, 132.4 (q, *J* = 33.7 Hz), 131.1, 128.9, 128.1, 123.8, 123.4, 122.7 (q, *J* = 270 Hz), 75.8, 70.5, 61.0; ^{19}F NMR (376 MHz, $CDCl_3$): δ -62.93. IR (neat): 1638, 1375, 1279, 1136, 923, 742, 682, 523 cm^{-1}; HRMS (ESI): Exact mass calcd for $C_{32}H_{24}F_6N_3O_4S$ [M+H]$^{+}$: 660.1386, Found: 660.1377.

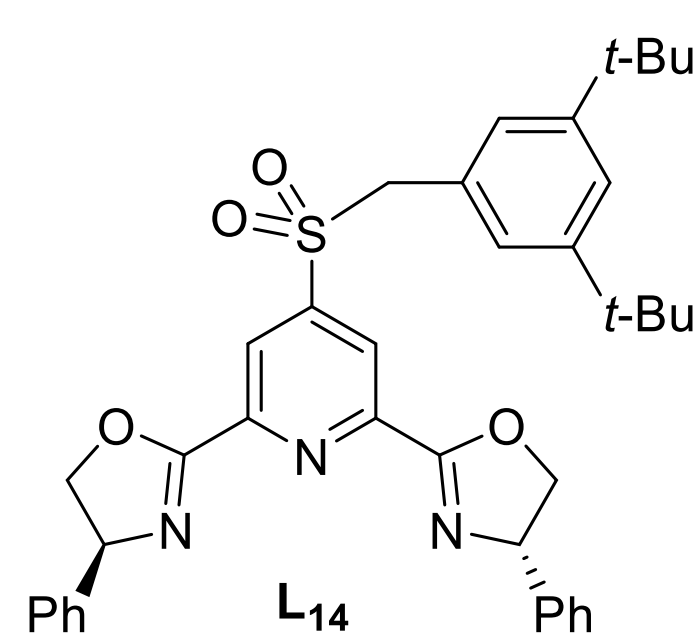

L14 was obtained in 21% yield as white solid. m.p. 209-211 ºC; ^{1}H NMR (400 MHz, $CDCl_3$): δ 8.44 (s, 2H), 7.37-7.28 (m, 11H), 6.94 (d, *J* = 2.0 Hz, 2H), 5.46 (t, *J* = 12.4 Hz, 2H), 4.92 (t, *J* = 12.0 Hz, 2H), 4.45-4.40 (m, 4H), 1.20 (s, 18H); ^{13}C NMR (100 MHz, $CDCl_3$): δ 162.1, 151.6, 148.2, 148.0, 141.1, 128.8, 127.9, 126.7, 125.8, 125.1, 124.3, 123.1, 75.6, 70.4, 62.9, 34.7, 31.2. IR (neat): 1638, 1423, 1267, 1144, 980, 741, 527 cm^{-1}; HRMS (ESI): Exact mass calcd for $C_{38}H_{42}N_3O_4S$ [M+H]$^{+}$: 636.2891, Found: 636.2881.

3. General procedure for the synthesis of hydrazones 1

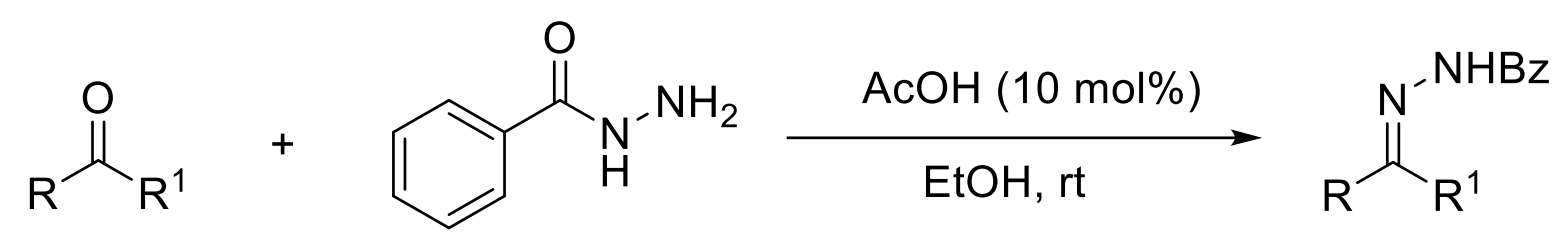

The hydrazones **1** were prepared from the corresponding ketone or aldehyde through the following general procedure[4]: To a solution of **S1** (5.0 mmol) and benzoyl hydrazine (1.1 equiv) in ethanol (5.0 mL) was slowly added AcOH (10 mol%) at room temperature. The resulting mixture was stirred for 2-8 h. The precipitate was flitered and recrystallized from ethanol to obtain the desired products with *E*-configuration.

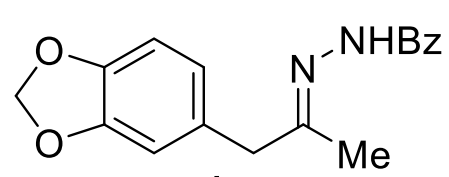

1a

1a was obtained in 72% yield as white solid (Rf value: 0.2, PE/EtOAc = 2:1). m.p. 197-199 °C; ^{1}H NMR (400 MHz, DMSO-d_6): δ 10.48 (s, 1H), 7.83 (d, *J* = 7.5 Hz, 2H), 7.55-7.46 (m, 3H), 6.88-6.73 (m, 3H), 5.99 (s, 2H), 3.53 (s, 2H), 1.86 (s, 3H); ^{13}C NMR (100 MHz, DMSO-d_6): δ 163.4, 161.8, 147.4, 146.0, 134.1, 131.4, 130.9, 130.0, 128.3, 127.7, 122.0, 109.2, 108.3, 100.9, 44.2, 16.3. IR (neat): 3005, 2360, 1717, 1440, 1364, 1225 cm^{-1}; HRMS (ESI): Exact mass calcd for $C_{17}H_{16}N_2NaO_3$ [M+Na]$^+$: 319.1053, Found: 319.1052.

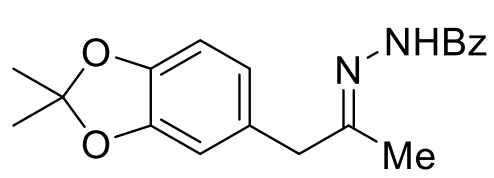

1b

1b was obtained in 86% yield as white solid (Rf value: 0.2, PE/EtOAc = 2:1). m.p. 201-203 °C; ^{1}H NMR (400 MHz, DMSO-d_6): 10.49 (s, 1H), 7.84 (d, *J* = 7.5 Hz, 2H), 7.55-7.44 (m, 3H), 6.78-6.68 (m, 3H), 3.51 (s, 2H), 1.86 (s, 3H), 1.62 (s, 6H); ^{13}C NMR (100 MHz, DMSO-d_6): δ 163.4, 161.9, 147.0, 145.6, 134.1, 131.3, 130.3, 128.3, 127.7, 121.5, 117.8, 109.0, 108.1, 44.3, 25.6, 16.4; IR (neat): 3223, 1668, 1527, 1494, 1255, 980, 713 cm^{-1}; HRMS (ESI): Exact mass calcd for $C_{19}H_{20}N_2NaO_3$ [M+Na]$^+$: 347.1366, Found: 347.1368. The X-ray analysis of (*E*)-**1b**, see section 11.

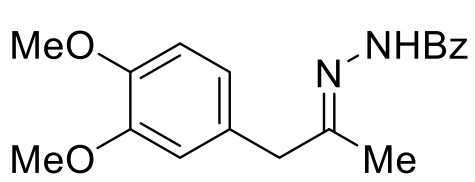

1c

1c[5] was obtained in 77% yield as white solid (Rf value: 0.2, PE/EtOAc = 2:1).^{1}H NMR (400 MHz, DMSO-d_6): δ 10.50 (s, 1H), 7.84 (d, *J* = 7.5 Hz, 2H), 7.55-7.45 (m, 3H), 6.92-6.78 (m, 3H), 3.74 (s, 3H), 3.73 (s, 3H), 3.55 (s, 2H), 1.86 (s, 3H); ^{13}C NMR (100 MHz,

DMSO-d_6): δ 163.4, 162.0, 148.8, 147.6, 134.1, 131.3, 129.5, 128.3, 127.7, 121.0, 112.8, 112.0, 55.5, 55.4, 44.2, 16.3.

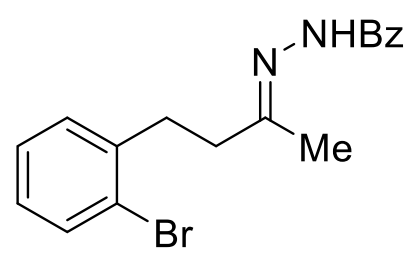

1d

1d was obtained in 82% yield as white solid (Rf value: 0.2, PE/EtOAc = 3:1). m.p. 193-195 °C; ^{1}H NMR (400 MHz, DMSO-d_6): δ 10.48 (s, 1H), 7.83 (d, J = 7.5 Hz, 2H), 7.61-7.44 (m, 5H), 7.33 (t, J = 7.5 Hz, 1H), 7.16 (td, J = 7.7, 1.8 Hz, 1H)),7.40-7.23 (m, 3H), 2.97 (t, J = 8.0 Hz, 2H), 2.58 (t, J = 8.0 Hz, 2H), 2.01 (s, 3H); ^{13}C NMR (100 MHz, DMSO-d_6): δ 163.4, 161.1, 140.4, 134.2, 132.5, 131.3, 130.9, 130.6, 128.2, 127.9, 127.7, 123.7, 32.3, 31.2, 17.1; IR (neat): 3223, 1653, 1602, 1527, 1282, 1026, 750 cm^{-1}; HRMS (ESI): Exact mass calcd for $C_{17}H_{17}{}^{79}BrN_2NaO$ [M+Na]$^+$: 367.0416, Found: 367.0412.

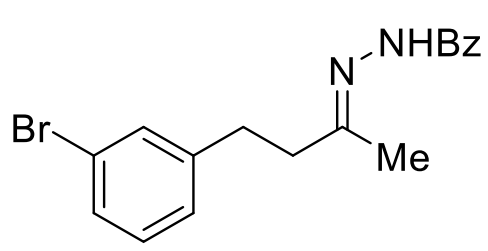

1e

1e was obtained in 79% yield as white solid (Rf value: 0.2, PE/EtOAc = 3:1). m.p. 196-198 °C; ^{1}H NMR (400 MHz, DMSO-d_6): δ 10.45 (s, 1H), 7.83 (d, J = 7.5 Hz, 2H), 7.55-7.46 (m, 4H), 7.40-7.23 (m, 3H), 2.87 (t, J = 8.0 Hz, 2H), 2.60, (t, J = 8.0 Hz, 2H) 1.98 (s, 3H); ^{13}C NMR (100 MHz, DMSO-d_6): δ 163.8, 161.7, 144.8, 134.6, 131.7, 130.9, 129.4, 129.2, 128.7, 128.2, 128.1, 122.1, 31.8, 30.8, 17.5; IR (neat): 3230, 1631, 1529, 1276, 1136, 1072, 750, 696 cm^{-1}; HRMS (ESI): Exact mass calcd for $C_{17}H_{17}{}^{79}BrN_2NaO$ [M+Na]$^+$: 367.0416, Found: 367.0421.

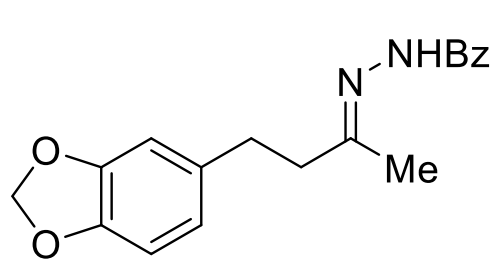

1f

1f was obtained in 89% yield as white solid (Rf value: 0.2, PE/EtOAc = 2:1). m.p. 240-242 °C; ^{1}H NMR (400 MHz, DMSO-d_6): δ 10.43 (s, 1H), 7.82 (d, J = 7.5 Hz, 2H), 7.55-7.46 (m, 3H), 6.88 (s, 1H), 6.82-6.80 (m, 1H), 6.72 (d, J = 7.8 Hz, 1H), 5.96 (s, 2H), 2.78 (t, J = 8.0 Hz, 2H), 2.55 (t, J = 8.0 Hz, 2H), 1.97 (s, 3H); ^{13}C NMR (100 MHz, DMSO-d_6): δ 163.8, 162.2, 147.6, 145.8, 135.7, 134.6, 131.7, 128.7, 128.1, 121.6, 109.4, 108.5, 101.1, 41.1, 32.1, 31.0, 17.4; IR (neat): 3250, 1633, 1530, 1500, 1489, 1240, 1036, 810 cm^{-1}; HRMS (ESI): Exact mass calcd for $C_{18}H_{18}N_2NaO_3$ [M+Na]$^+$: 333.1210, Found: 333.1204.

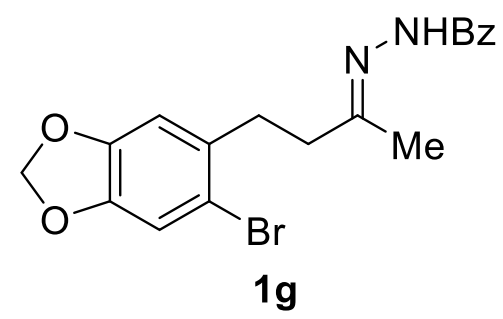

1g was obtained in 56% yield as white solid (Rf value: 0.2, PE/EtOAc = 2:1). m.p. 238-240 ºC; ^{1}H NMR (400 MHz, DMSO-d_6): δ 10.49 (s, 1H), 7.86-7.79 (m, 3H), 7.56-7.49 (m, 3H), 7.17 (s, 1H), 7.08 (s, 1H), 6.04 (s, 2H), 2.91-2.81 (m, 2H), 2.56-2.53 (m, 2H), 2.02 (s, 3H); ^{13}C NMR (100 MHz, DMSO-d_6): δ 163.5, 161.3, 147.3, 146.7, 133.6, 131.4, 128.4, 127.8, 113.6, 112.2, 110.5, 101.8, 32.1, 31.3, 31.1, 17.1; IR (neat): 3203, 1631, 1478, 1275, 1236, 1040, 1225, 1039 cm^{-1}; HRMS (ESI): Exact mass calcd for $C_{18}H_{17}{}^{79}BrN_2NaO_3$ [M+Na]$^+$: 411.0315, Found: 411.0324.

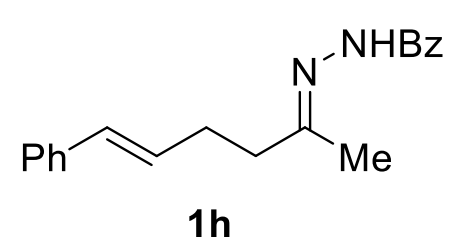

1h was obtained in 40% yield as white solid (Rf value: 0.2, PE/EtOAc = 3:1). m.p. 220-222 ºC; ^{1}H NMR (400 MHz, DMSO-d_6): δ 10.47 (s, 1H), 7.84 (d, J = 7.5 Hz, 2H), 7.56-7.46 (m, 3H), 7.41 -7.36 (m, 2H), 7.34 -7.29 (m, 2H), 7.24-7.19 (m, 1H), 6.52-6.47 (m, 1H), 6.38-6.28 (m, 1H), 2.53-2.49 (m, 4H), 1.99 (s, 3H). ^{13}C NMR (125 MHz, DMSO-d_6): δ 163.8, 162.6, 137.7, 134.6, 131.8, 130.4, 130.2, 129.0, 128.8, 128.2, 127.5, 126.3, 38.7, 29.9, 17.4 IR (neat): 3213, 1632, 1455, 1275, 1237, 1037, 788 cm^{-1}; HRMS (ESI): Exact mass calcd for $C_{19}H_{20}N_2NaO$ [M+Na]$^+$: 315.1468, Found: 315.1477.

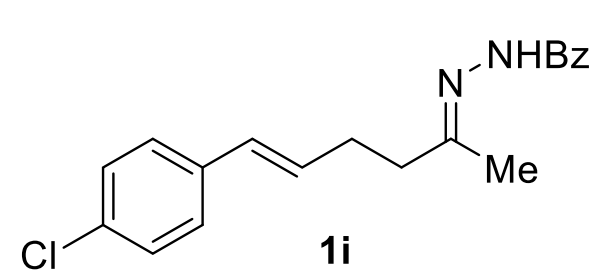

1i was obtained in 60% yield as white solid (Rf value: 0.2, PE/EtOAc = 3:1). m.p. 238-240 ºC; ^{1}H NMR (400 MHz, DMSO-d_6): δ 10.48 (s, 1H), 7.84 (d, *J* = 7.5 Hz, 2H), 7.56-7.35 (m, 7H), 7.52-7.36 (m, 2H), 6.52-6.36 (m, 2H), 2.51-2.48 (m, 4H), 1.98 (s, 3H); ^{13}C NMR (125 MHz, DMSO-d_6): δ 163.8, 162.5, 136.6, 134.6, 131.8, 131.4, 131.0, 129.2, 129.0, 128.8, 128.2, 128.0, 38.5, 29.8, 17.4; IR (neat): 3208, 1628, 1475, 1280, 1230, 1048, 1030 cm^{-1}; HRMS (ESI): Exact mass calcd for $C_{19}H_{19}ClN_2NaO$ [M+Na]$^+$: 349.1075, Found: 349.1078.

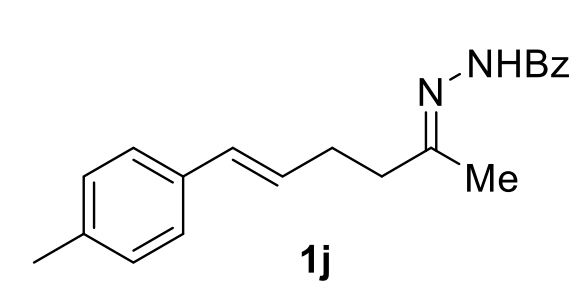

1j was obtained in 56% yield as white solid (Rf value: 0.2, PE/EtOAc = 3:1). m.p. 230-232 ºC; ^{1}H NMR (400 MHz, DMSO-d_6): δ 10.47 (s, 1H), 7.85-7.77 (m, 2H), 7.54-7.46 (m, 3H), 7.29-7.25 (m, 2H), 7.14-7.11 (m, 2H), 7.47-7.40 (m, 1H), 6.30-6.24 (m, 1H), 2.51-2.47 (m, 4H), 2.28 (s, 3H), 1.98 (s, 3H); ^{13}C NMR (25MHz, DMSO-

d$_6$): δ 163.7, 162.6, 136.6, 134.9, 134.6, 131.8, 130.3, 129.6, 129.1, 128.7, 128.1, 126.2, 39.4, 29.9, 21.2, 17.3; IR (neat): 3200, 1635, 1482, 1270, 1240, 1045, 770 cm^{-1}; HRMS (ESI): Exact mass calcd for $C_{20}H_{22}N_2NaO$ [M+Na]$^+$: 329.1624, Found: 329.1628.

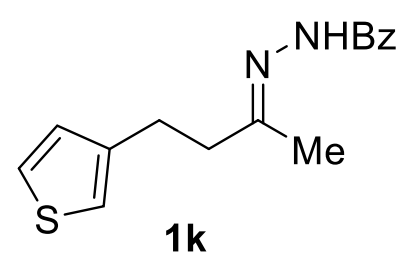

1k

1k was obtained in 50% yield as white solid (Rf value: 0.2, PE/EtOAc = 3:1). m.p. 218-220 ºC; ^{1}H NMR (400 MHz, CD_3CN): δ 9.19 (s, 1H), 7.82 (d, *J* = 7.5 Hz, 2H), 7.58-7.54 (m, 1H), 7.50-7.46 (m, 2H), 7.33 (dd, *J* = 4.9, 2.9 Hz, 1H), 7.08 (d, *J* = 30.1 Hz, 2H), 2.93 (t, *J* = 8.0 Hz, 2H), 2.66 (d, *J* = 8.2 Hz, 2H), 1.97 (s, 3H).; ^{13}C NMR (125 MHz, CD_3CN): δ 165.8, 161.4, 143.9, 136.2, 133.6, 130.5, 129.6, 127.5, 122.7, 41.7, 28.6, 17.5; IR (neat): 3195, 1630, 1480, 1268, 1238, 1038, 1048, 772 cm^{-1}; HRMS (ESI): Exact mass calcd for $C_{15}H_{16}N_2NaOS$ [M+Na]$^+$: 295.0876, Found: 295.0878.

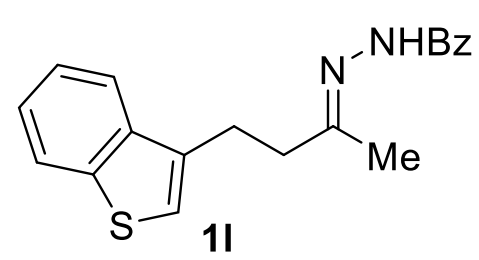

1l

1l was obtained in 44% yield as white solid (Rf value: 0.2, PE/EtOAc = 3:1). m.p. 233-235 ºC; ^{1}H NMR (400 MHz, DMSO-*d*$_6$): δ 10.53 (s, 1H), 7.99 (d, *J* = 7.5 Hz, 2H), 7.56-7.37 (m, 7H), 3.14-3.07 (m, 2H), 2.76-2.73 (m, 2H), 2.04 (s, 3H); ^{13}C NMR (125 MHz, DMSO-*d*$_6$): δ 163.9, 162.3, 140.2, 139.2, 136.1, 134.7, 131.8, 128.8, 128.2, 124.6, 123.5, 122.9, 122.2, 38.5, 25.1, 17.5; IR (neat): 3210, 1625, 1470, 1282, 1242, 1228 cm^{-1}; HRMS (ESI): Exact mass calcd for $C_{19}H_{18}N_2NaOS$ [M+Na]$^+$: 345.1032, Found: 345.1031.

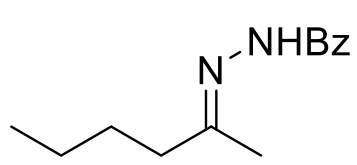

1m

1m was obtained in 63% yield as white solid (Rf value: 0.3, PE/EtOAc = 3:1). m.p. 190-192 ºC; ^{1}H NMR (400 MHz, DMSO-*d*$_6$): δ 10.40 (s, 1H), 7.82 (d, *J* = 7.5 Hz, 2H), 7.55-7.46 (m, 3H), 2.29 (t, *J* = 7.6 Hz, 2H), 1.93 (s, 3H), 1.54-1.47 (m, 2H), 1.37-1.28 (m, 2H), 0.90 (t, *J* = 6.7 Hz, 3H); ^{13}C NMR (100 MHz, DMSO-*d*$_6$): δ 163.18, 163.16, 134.2, 131.2, 128.3, 127.6, 38.1, 28.1, 21.8, 13.81, 13.76; IR (neat): 3005, 2364, 1716, 1437, 1364, 1223, 1094, 532 cm^{-1};HRMS (ESI): Exact mass calcd for $C_{13}H_{18}N_2NaO$ [M+Na]$^+$: 241.1311, Found: 241.1317.

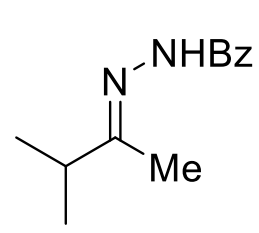

1n

1n[4] was obtained in 48% yield as pale yellow solid (Rf value: 0.3, PE/EtOAc = 3:1).^{1}H NMR (400 MHz, DMSO-*d*$_6$): δ 10.36 (s, 1H), 7.83-7.81 (m, 2H), 7.54-7.44 (m, 3H), 2.59-

2.52 (m, 1H), 1.91 (s, 3H), 1.09 (s, 3H), 1.08 (s, 3H); ^{13}C NMR (100 MHz, DMSO-d_6): δ 166.5, 163.3, 134.3, 131.2, 128.3, 127.6, 36.6, 19.8, 14.2.

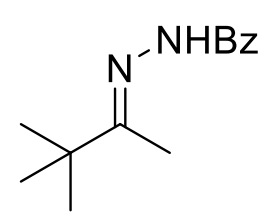

1o

1o was obtained in 78% yield as white solid (Rf value: 0.4, PE/EtOAc = 6:1). m.p. 209-210 °C; ^{1}H NMR (400 MHz, DMSO-d_6): δ 10.31 (s, 1H), 7.83-7.81 (m, 2H), 7.55-7.44 (m, 3H), 1.93 (s, 3H), 1.14 (s, 9H); ^{13}C NMR (100 MHz, DMSO-d_6): δ 165.9, 163.3, 134.4, 133.2, 131.1, 128.5, 128.3, 127.6, 127.5, 127.0, 38.7, 27.6, 12.9. IR (neat): 3143, 1645, 1483, 1267, 1101, 743, 706 cm^{-1}; HRMS (ESI): Exact mass calcd for $C_{13}H_{19}N_2O$ $[M+H]^+$: 219.1492, Found: 218.1488.

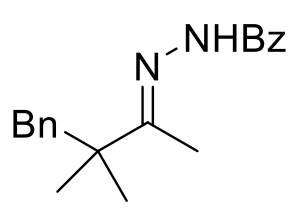

1p

1p was obtained in 76% yield as white solid (Rf value: 0.2, PE/EtOAc = 2:1). m.p. 211-213 °C; ^{1}H NMR (400 MHz, DMSO-d_6): δ 10.35 (s, 1H), 7.84-7.83 (m, 1H), 7.53-7.08 (m, 9H), 2.81-2.78 (m, 2H), 2.04 (s, 3H), 1.09-1.03 (m, 6H); ^{13}C NMR (100 MHz, DMSO-d_6): δ 166.6, 163.3, 138.4, 134.3, 130.3, 128.3, 127.9, 126.0, 45.4, 42.9, 25.4, 23.7. IR (neat): 1647, 1485, 1267, 1001, 741, 704 cm^{-1}; HRMS (ESI): Exact mass calcd for $C_{19}H_{23}N_2O$ $[M+H]^+$: 295.1805, Found: 295.1800.

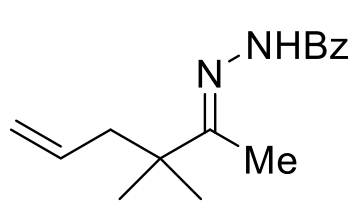

1q

1q was obtained in 31% yield as white solid (Rf value: 0.4, PE/EtOAc = 6:1). m.p. 207-208 °C; ^{1}H NMR (400 MHz, DMSO-d_6): δ 10.35 (s, 1H), 7.83-7.82 (m, 2H), 7.54-7.47 (m, 3H), 5.75-5.73 (m, 1H), 5.06-5.00 (m, 2H), 2.26-2.25 (m, 2H), 1.93 (s, 3H), 1.11 (s, 6H); ^{13}C NMR (100 MHz, DMSO-d_6): δ 167.0, 163.1, 135.3, 134.3, 131.2, 128.2, 127.6, 117.2, 44.3, 41.8, 25.4, 13.0. IR (neat): 1647, 1483, 1423, 1267, 999, 740, 706 cm^{-1}; HRMS (ESI): Exact mass calcd for $C_{15}H_{21}N_2O$ $[M+H]^+$: 245.1648, Found: 245.1642.

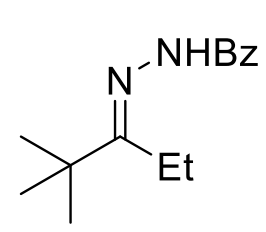

1r

1r was obtained in 37% yield as white solid (Rf value: 0.4, PE/EtOAc = 6:1). m.p. 209-211 °C; ^{1}H NMR (500 MHz, CD_3CN): δ 9.12 (s, 1H), 7.80 (d, *J* = 7.5 Hz, 2H), 7.55-7.47 (m, 3H), 2.46-2.44 (m, 2H), 1.17-1.11 (m, 12H); ^{13}C NMR (125 MHz, CD_3CN): δ 168.9, 164.6, 135.3, 132.5, 129.5, 128.4, 40.0, 28.1, 19.6, 10.9. IR (neat): 3210, 1647, 1579, 1477, 1267, 1001, 740, 706 cm^{-1}; HRMS (ESI): Exact mass calcd for $C_{14}H_{21}N_2O$ $[M+H]^+$: 233.1648, Found: 233.1643.

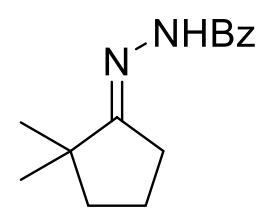

1s

1s was obtained in 49% yield as white solid (Rf value: 0.5, PE/EtOAc = 5:1). m.p. 149-151 ºC; ^{1}H NMR (500 MHz, DMSO-*d6*): δ 10.19 (s, 1H), 7.80-7.78 (m, 2H), 7.54-7.47 (m, 3H), 2.53-2.50 (m, 2H), 1.77-1.74 (m, 2H), 1.62-1.60 (m, 2H), 1.12 (s, 6H); ^{13}C NMR (125 MHz, DMSO-*d6*): δ 174.8, 163.3, 134.3, 131.3, 128.3, 127.7, 42.6, 28.3, 26.5, 20.4. IR (neat): 1645, 1485, 1418, 1265, 897, 738, 706 cm^{-1}; HRMS (ESI): Exact mass calcd for $C_{14}H_{19}N_2O$ $[M+H]^+$: 231.1492, Found: 231.1489.

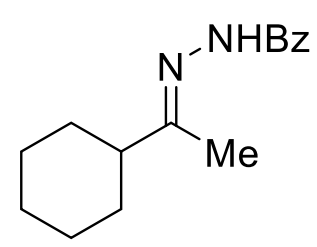

1t

1t[6] was obtained in 71% yield as white solid (Rf value: 0.3, PE/EtOAc = 3:1). m.p. 100-102 ºC; ^{1}H NMR (400 MHz, DMSO-*d6*): δ 10.33 (s, 1H), 7.81 (d, *J* = 7.5 Hz, 2H), 7.54-7.45 (m, 3H), 2.24 (s, 1H), 1.90 (s, 3H), 1.76-1.64 (m, 5H), 1.31-1.19 (m, 5H); ^{13}C NMR (100 MHz, DMSO-*d6*): δ 165.8, 163.2, 134.3, 131.2, 128.2, 127.6, 46.6, 29.7, 25.7, 25.6, 14.9.

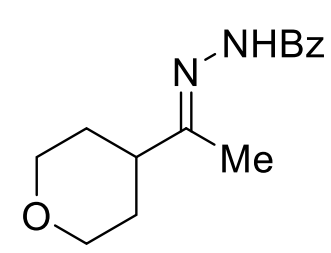

1u

1u was obtained in 50% yield as white solid (Rf value: 0.2, PE/EtOAc = 3:1). m.p. 195-197 ºC; ^{1}H NMR (400 MHz, DMSO-*d6*): δ 10.41 (s, 1H), 7.82 (d, *J* = 7.5 Hz, 2H), 7.55-7.46 (m, 3H), 3.93-3.89 (m, 2H), 3.39-3.33 (m, 3H), 1.93 (s, 3H), 1.70-1.56 (m, 4H); ^{13}C NMR (100 MHz, DMSO-*d6*): δ 164.2, 163.3, 134.2, 131.2, 128.3, 127.6, 66.8, 40.2, 29.6, 15.1; IR (neat): 3005, 2951, 2841, 2361, 1717, 1437, 1364, 1223, 1091, 530 cm^{-1}; HRMS (ESI): Exact mass calcd for $C_{14}H_{18}N_2NaO_2$ $[M+Na]^+$: 269.1260, Found: 269.1267.

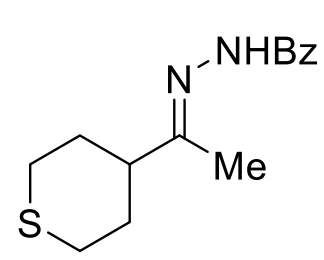

1v

1v was obtained in 80% yield as white solid (Rf value: 0.3, PE/EtOAc = 3:1). m.p. 226-227 ºC; ^{1}H NMR (400 MHz, DMSO-*d6*): δ 10.40 (s, 1H), 7.81 (d, *J* = 7.5 Hz, 2H), 7.55-7.46 (m, 3H), 2.73-2.62 (m, 4H), 2.34 (t, *J* = 13.2 Hz, 1H), 2.05 (t, *J* = 12.3 Hz, 2H), 1.92 (s, 3H), 1.66-1.63 (m, 2H); ^{13}C NMR (100 MHz, DMSO-*d6*): δ 164.6, 163.3, 134.2, 131.2, 128.3, 127.6, 45.8, 30.9, 27.5, 15.1; IR (neat): 3230, 1664, 1649, 1526, 1273, 1136, 1076, 750 cm^{-1}; HRMS (ESI): Exact mass calcd for $C_{14}H_{18}N_2NaOS$ $[M+Na]^+$: 285.1032, Found: 285.1031.

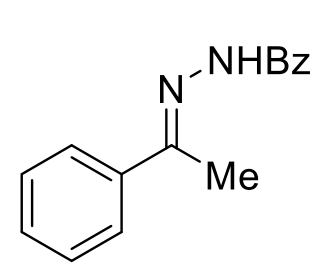

1w

1w[6] was obtained in 74% yield as white solid (Rf value: 0.3, PE/EtOAc = 2:1). ^{1}H NMR (400 MHz, DMSO-*d6*): 10.78 (s, 1H), 7.90-7.86 (m, 4H), 7.58-7.43 (m, 6H), 2.37 (s, 3H);

^{13}C NMR (100 MHz, DMSO-d_6): δ 163.9, 155.5, 138.1, 134.1, 131.5, 129.4, 128.4, 127.9, 126.4, 14.6.

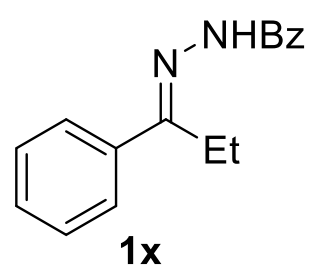

1x

1x[7] was obtained in 79% yield as white solid (Rf value: 0.2, PE/EtOAc = 2:1). ^{1}H NMR (400 MHz, DMSO-d_6): δ 10.84 (s, 1H), 7.83 (s, 4H), 7.58-7.42 (m, 6H), 2.92 (q, J = 7.5 Hz, 2H), 1.08 (t, J = 7.5 Hz, 3H); ^{13}C NMR (100 MHz, DMSO-d_6): δ 164.3, 159.1, 137.0, 134.3, 131.4, 129.3, 128.4, 128.2, 128.0, 126.6, 19.9, 10.9.

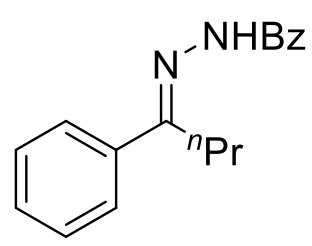

1y

1y[4] was obtained in 85% yield as white solid (Rf value: 0.2, PE/EtOAc = 2:1). ^{1}H NMR (400 MHz, DMSO-d_6): δ 10.85 (s, 1H), 7.82 (s, 4H), 7.58-7.42 (m, 6H), 2.90 (t, J = 7.9 Hz, 2H), 1.50 (q, J = 7.6 Hz, 2H), 0.95 (t, J = 7.3 Hz, 3H); ^{13}C NMR (100 MHz, DMSO-d_6): δ 164.2, 157.9, 137.4, 134.4, 131.4, 129.2, 128.4, 128.3, 128.0, 126.6, 28.3, 19.5, 13.8.

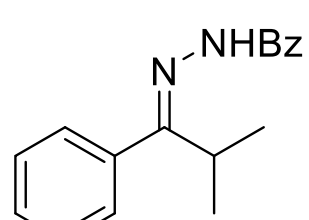

1z

1z was obtained in 80% yield as white solid (Rf value: 0.5, PE/EtOAc = 4:1). m.p. 223-225 ^{o}C; ^{1}H NMR (400 MHz, DMSO-d_6): δ 9.66 (s, 1H), 7.55-7.34 (m, 10H), 2.92 (s, 1H), 1.15-1.09 (m, 6H); ^{13}C NMR (100 MHz, DMSO-d_6): δ 164.9, 162.6, 137.5, 133.9, 133.7, 131.4, 129.2, 129.1, 128.5, 127.9, 127.3, 127.0, 35.9, 20.0, 19.7; IR (neat): 3053, 1678, 1477, 1265, 1117, 895, 739, 706 cm^{-1}; HRMS (ESI): Exact mass calcd for $C_{17}H_{19}N_2O$ [M+H]$^+$: 267.1492, Found: 267.1486.

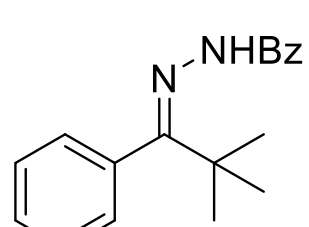

1aa

1aa was obtained in 74% yield as white solid (Rf value: 0.3, PE/EtOAc = 4:1). m.p. 241-243 ^{o}C; ^{1}H NMR (400 MHz, DMSO-d_6): δ 8.92 (s, 1H), 7.57-7.38 (m, 8H), 7.23 (d, J = 6.8 Hz, 2H), 1.17 (s, 9H); ^{13}C NMR (100 MHz, DMSO-d_6): δ 162.2, 133.7, 131.6, 129.0, 128.6, 127.8, 126.7, 38.2, 28.1; IR (neat): 3057, 1684, 1506, 1483, 1265, 1007, 894, 739 cm^{-1}; HRMS (ESI): Exact mass calcd for $C_{18}H_{21}N_2O$ [M+H]$^+$: 281.1648, Found: 281.1642.

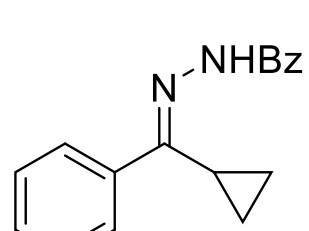

1ab

1ab was obtained in 31% yield as white solid (Rf value: 0.5, PE/EtOAc = 4:1). m.p. 211-213 ^{o}C; ^{1}H NMR (500 MHz, DMSO-d_6): δ 10.80 (s, 1H), 7.91-7.80 (m, 3H), 7.60-7.41 (m, 7H), 2.04 (s, 1H), 1.17-1.15 (m, 2H), 0.57-0.56 (m, 2H); ^{13}C NMR (125 MHz, DMSO-d_6): δ 163.0, 156.3, 136.8, 133.9, 131.7, 129.3, 128.9, 128.7, 128.6, 128.1, 127.9, 127.6, 127.3, 127.1,

17.7, 9.0, 6.7. IR (neat): 3055, 1601, 1481, 1267, 897, 741, 706 cm^{-1}; HRMS (ESI): Exact mass calcd for $C_{17}H_{17}N_2O$ $[M+H]^+$: 265.1335, Found: 265.1332.

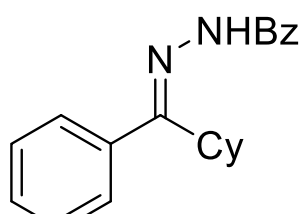
1ac

1ac was obtained in 87% yield as white solid (Rf value: 0.5, PE/EtOAc = 4:1). m.p. 227-229 ºC; ^{1}H NMR (400 MHz, DMSO-*d6*): δ 9.64 (s, 1H), 7.54-7.32 (m, 10H), 2.58 (s, 1H), 1.78-1.59 (m, 5H), 1.41-1.13 (m, 5H); ^{13}C NMR (100 MHz, DMSO-*d6*): δ 164.1, 162.6, 138.2, 133.9, 131.4, 129.1, 129.1, 128.4, 128.0, 127.8, 127.2, 127.0, 45.4, 30.1, 29.3, 26.0, 25.5, 25.4. IR (neat): 1684, 1506, 1481, 1265, 1099, 903, 739, 704 cm^{-1}; HRMS (ESI): Exact mass calcd for $C_{20}H_{23}N_2O$ $[M+H]^+$: 307.1805, Found: 307.1796.

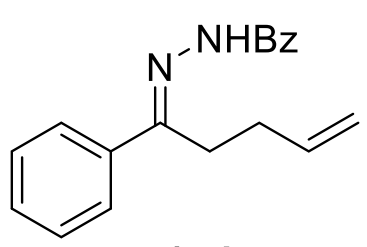
1ad

1ad was obtained in 52% yield as white solid (Rf value: 0.2, PE/EtOAc = 2:1). m.p. 205-206 ºC; ^{1}H NMR (400 MHz, DMSO-*d6*): δ 10.88 (s, 1H), 7.82 (s, 4H), 7.58-7.42 (m, 6H), 5.92-5.82 (m, 1H), 5.08-4.97 (m, 2H), 3.02 (t, J = 7.9 Hz, 2H), 2.24 (q, J = 7.6 Hz, 2H); ^{13}C NMR (100 MHz, DMSO-*d6*): δ 164.3, 157.3, 137.2, 134.3, 131.4, 129.4, 128.4, 128.3, 127.2, 127.0, 126.6, 115.7, 30.1, 25.9. IR (neat): 1776, 1697, 1680, 1620, 1275, 1142, 750 cm^{-1}; HRMS (ESI): Exact mass calcd for $C_{18}H_{18}N_2NaO$ $[M+Na]^+$: 302.1311, Found: 302.1316.

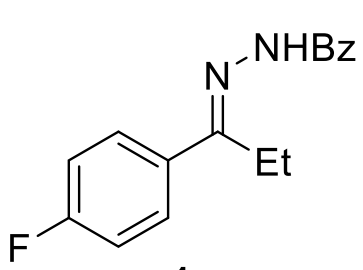
1ae

1ae was obtained in 77% yield as white solid (Rf value: 0.2, PE/EtOAc = 2:1). m.p. 220-222 ºC; ^{1}H NMR (400 MHz, DMSO-*d6*): δ 10.85 (s, 1H), 7.84 (s, 4H), 7.58-7.49 (m, 3H), 7.26 (s, 2H), 2.91 (q, J = 7.7 Hz), 1.06 (t, J = 7.6 Hz, 3H); ^{13}C NMR (100 MHz, DMSO-*d6*): δ 164.4, 162.9 (d, J = 246.0 Hz), 158.1, 134.2, 133.5 (d, J = 3.1 Hz), 131.4, 128.8, 128.3, 128.0, 115.4 (d, J = 21.5 Hz), 19.9, 10.8; ^{19}F NMR (376 MHz, DMSO-*d6*): δ -112.28. IR (neat): 3005, 2364, 1717, 1436, 1363, 1223, 1093, 530 cm^{-1}; HRMS (ESI): Exact mass calcd for $C_{16}H_{15}FN_2NaO$ $[M+Na]^+$: 293.1061, Found: 293.1064.

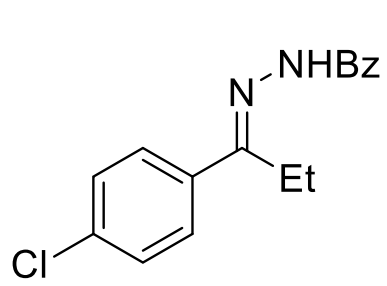
1af

1af[8] was obtained in 90% yield as white solid (Rf value: 0.2, PE/EtOAc = 2:1). ^{1}H NMR (400 MHz, DMSO-*d6*): δ 10.90 (s, 1H), 7.84 (s, 4H), 7.59-7.50 (m, 5H), 2.92 (q, J = 7.6 Hz, 2H), 1.07 (t, J = 7.5 Hz, 3H); ^{13}C NMR (100 MHz, DMSO-*d6*): δ 164.4, 157.4, 135.8, 134.2, 134.0, 131.4, 128.5, 128.3, 128.2, 128.1, 19.7, 10.8.

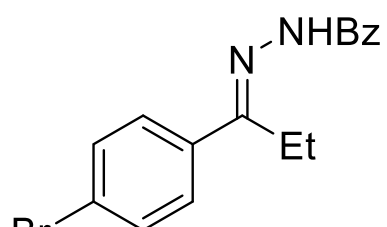

1ag

1ag was obtained in 93% yield as white solid (Rf value: 0.2, PE/EtOAc = 2:1). m.p. 259-260 ºC; ^{1}H NMR (400 MHz, DMSO-d_6): δ 10.89 (s, 1H), 7.83-7.77 (m, 4H), 7.64-7.49 (m, 5H), 2.91 (q, J = 7.6 Hz, 2H), 1.06 (t, J = 7.5 Hz, 3H); ^{13}C NMR (100 MHz, DMSO-d_6): δ 164.4, 157.5, 136.2, 134.2, 131.4, 129.6, 128.6, 128.2, 128.1, 122.8, 19.6, 10.8. IR (neat): 3005, 2362, 1717, 1436, 1363, 1223, 1093, 532 cm^{-1}; HRMS (ESI): Exact mass calcd for $C_{16}H_{15}{}^{79}BrN_2NaO$ [M+Na]$^+$: 353.0260, Found: 353.0257.

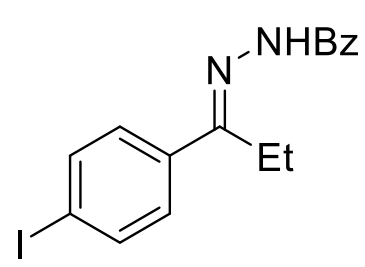

1ah

1ah was obtained in 93% yield as white solid (Rf value: 0.2, PE/EtOAc = 2:1). m.p. 276-278 ºC; ^{1}H NMR (400 MHz, DMSO-d_6): δ 10.88 (s, 1H), 7.85-7.81 (m, 4H), 7.60-7.49 (m, 5H), 2.89 (q, J = 7.5 Hz, 2H), 1.05 (t, J = 7.5 Hz, 3H); ^{13}C NMR (100 MHz, DMSO-d_6): δ 164.4, 157.7, 137.3, 136.5, 134.2, 131.5, 128.6, 128.2, 128.1, 96.2, 19.5, 10.8. IR (neat): 3005, 2361, 1717, 1437, 1364, 1223, 1094, 530 cm^{-1}; HRMS (ESI): Exact mass calcd for $C_{16}H_{15}IN_2NaO$ [M+Na]$^+$: 401.0121, Found: 401.0126.

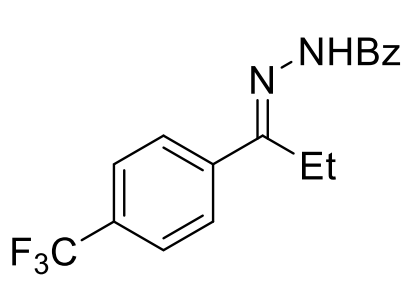

1ai

1ai was obtained in 59% yield as white solid (Rf value: 0.2, PE/EtOAc = 2:1). m.p. 260-262ºC; ^{1}H NMR (400 MHz, DMSO-d_6): δ 11.00 (s, 1H), 8.03 (s, 2H), 7.85-7.81 (m, 4H), 7.59-7.50 (m, 3H), 2.97 (q, J = 7.5 Hz, 2H), 1.08 (t, J = 7.5 Hz, 3H); ^{13}C NMR (100 MHz, DMSO-d_6): δ 164.7, 156.9, 141.0, 134.1, 131.5, 129.2 (q, J = 33.0 Hz), 128.3, 128.2, 127.2, 125.4 (q, J = 3.8 Hz), 124.2 (q, J = 271.0 Hz), 19.7, 10.7; ^{19}F NMR (376 MHz, DMSO-d_6): δ -56.33. IR (neat): 3005, 2360, 1717, 1521, 1436, 1364, 1223, 1067, 844, 532 cm^{-1}; HRMS (ESI): Exact mass calcd for $C_{17}H_{15}F_3N_2NaO$ [M+Na]$^+$: 343.1029, Found: 343.1024.

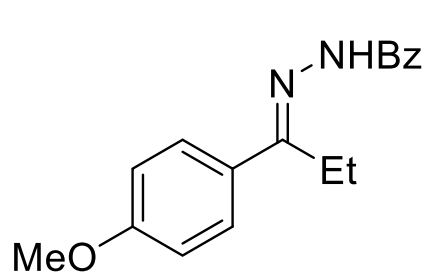

1aj

1aj[9] was obtained in 74% yield as white solid (Rf value: 0.2, PE/EtOAc = 1:1). ^{1}H NMR (400 MHz, DMSO-d_6): δ 10.75 (s, 1H), 7.84-7.81 (m, 4H), 7.58-7.50 (m, 3H), 7.01-6.99 (m, 2H), 3.81 (s, 3H), 2.89 (q, J = 7.5 Hz, 2H), 1.07 (t, J = 7.5 Hz, 3H); ^{13}C NMR (100 MHz, DMSO-d_6): δ 164.1, 160.3, 159.4, 134.4, 131.4, 129.3, 128.3, 128.2, 127.9, 113.8, 55.2, 19.8, 11.0.

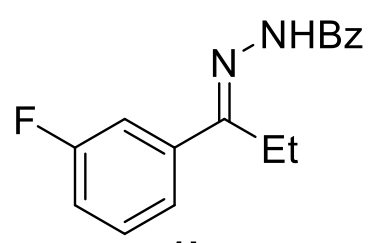

1k

1ak was obtained in 79% yield as white solid (Rf value: 0.2, PE/EtOAc = 2:1). m.p. 226-228 ºC; ^{1}H NMR (400 MHz, DMSO-*d$_6$*): δ 10.92 (s, 1H), 7.83 (s, 2H), 7.59-7.48 (m, 6H), 7.26 (s, 1H), 2.92 (q, *J* = 7.8 Hz, 2H), 1.07 (t, *J* = 7.5 Hz, 3H); ^{13}C NMR (100 MHz, DMSO-*d$_6$*): δ 164.4, 162.3 (d, *J* = 243.0 Hz, 1H), 157.1, 139.6 (d, *J* = 7.5 Hz), 131.2, 131.5, 130.4 (d, *J* = 8.4 Hz), 128.2, 122.7, 116.03 (d, *J* = 20.8 Hz), 112.99 (d, *J* = 23.7 Hz) 19.7, 10.8; ^{19}F NMR (376 MHz, DMSO-*d$_6$*): δ -108.36. IR (neat): 3005, 2363, 1717, 1436, 1363, 1223, 1093, 902, 786, 530 cm^{-1}; HRMS (ESI): Exact mass calcd for $C_{16}H_{15}FN_2NaO$ [M+Na]$^+$: 293.1061, Found: 293.1062.

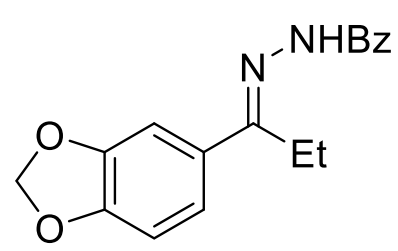

1al

1al was obtained in 75% yield as white solid (Rf value: 0.2, PE/EtOAc = 1:1). m.p. 253-255 ºC; ^{1}H NMR (400 MHz, DMSO-*d$_6$*): δ 10.76 (s, 1H), 7.83 (s, 2H), 7.58-7.49 (m, 3H), 7.43-7.34 (m, 2H), 6.97 (d, *J* = 8.1 Hz, 1H), 6.08 (s, 2H), 3.81 (s, 3H), 2.87 (d, *J* = 8.0 Hz, 2H), 1.06 (t, *J* = 7.5 Hz, 3H); ^{13}C NMR (100 MHz, DMSO-*d$_6$*): δ 164.2, 158.9, 148.4, 147.7, 134.3, 131.4, 131.2, 128.3, 128.0, 121.2, 108.0, 106.4, 101.4, 19.90, 11.1; IR (neat): 3005, 2365, 1717, 1437, 1364, 1223, 530 cm^{-1}; HRMS (ESI): Exact mass calcd for $C_{17}H_{16}N_2NaO_3$ [M+Na]$^+$:319.1053, Found: 319.1047.

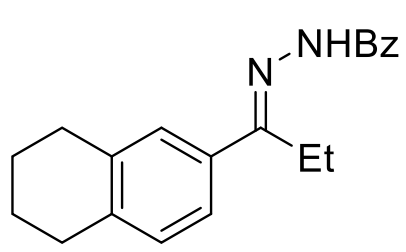

1am

1am was obtained in 64% yield as white solid (Rf value: 0.2, PE/EtOAc = 2:1). m.p. 205-207 ºC; ^{1}H NMR (400 MHz, DMSO-*d$_6$*): δ 10.76 (s, 1H), 7.83 (d, *J* = 7.5 Hz, 1H), 7.56-7.49 (m, 5H), 7.09 (s, 1H), 2.88 (d, *J* = 7.6 Hz, 2H), 2.75-2.66 (m, 4H), 1.74-1.74 (m, 4H), 1.06 (t, *J* = 7.5 Hz, 3H); ^{13}C NMR (100 MHz, DMSO-*d$_6$*): δ 164.1, 159.5, 138.2, 136.6, 134.3, 134.1, 131.3, 129.0, 128.3, 127.9, 127.1, 123.8, 28.9, 28.7, 22.7, 22.6, 11.0; IR (neat): 3215, 1530, 1275, 1136, 913, 750 cm^{-1}; HRMS (ESI): Exact mass calcd for $C_{20}H_{22}N_2NaO$ [M+Na]$^+$: 329.1624, Found: 329.1624.

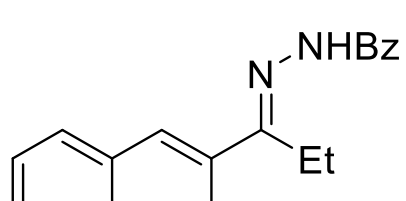

1an

1an was obtained in 64% yield as white solid (Rf value: 0.2, PE/EtOAc = 2:1). m.p. 249-251 ºC; ^{1}H NMR (400 MHz, DMSO-*d$_6$*): δ 10.92 (s, 1H), 8.32 (s, 1H), 8.12-7.86 (m, 6H), 7.62-7.51 (m, 5H), 3.06 (q, *J* = 7.7 Hz, 2H), 1.16 (t, *J* = 7.5 Hz, 3H); ^{13}C NMR (100 MHz, DMSO-*d$_6$*): δ 164.4, 158.5, 134.3, 133.3, 132.8, 131.4, 128.6, 128.2, 128.1, 127.8,

127.4, 126.8, 126.4, 126.3, 124.0, 19.6, 11.1; IR (neat): 3005, 2538, 1713, 1437, 1362, 1221, 1092, 904, 530 cm^{-1}; HRMS (ESI): Exact mass calcd for $C_{20}H_{18}N_2NaO$ $[M+Na]^+$: 325.1311, Found: 325.1308.

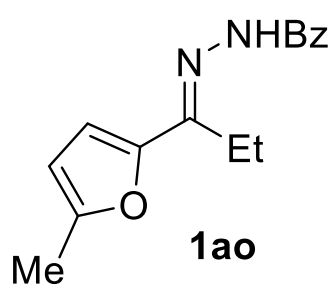

1ao

1ao was obtained in 39% yield as white solid (Rf value: 0.5, PE/EtOAc = 5:1). m.p. 188-190 ºC; ^1H NMR (400 MHz, DMSO-d_6): δ 10.68 (s, 1H), 7.83-7.81 (m, 2H), 7.59-7.48 (m, 3H), 6.86 (s, 1H), 6.23 (s, 1H), 2.78-2.76 (m, 2H), 2.33 (s, 3H), 1.09 (d, J = 7.2 Hz, 3H); ^{13}C NMR (100 MHz, DMSO-d_6): δ 153.9, 149.6, 134.2, 131.4, 128.3, 113.2, 108.3, 19.8, 13.6, 11.2. IR (neat): 3057, 1869, 1647, 1479, 1267, 1028, 741 cm^{-1}; HRMS (ESI): Exact mass calcd for $C_{15}H_{17}N_2O_2$ $[M+H]^+$: 257.1285, Found: 257.1278.

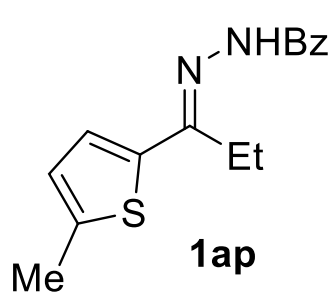

1ap

1ap was obtained in 40% yield as white solid (Rf value: 0.5, PE/EtOAc = 5:1). m.p. 192-194 ºC; ^1H NMR (500 MHz, DMSO-d_6): δ 10.73 (s, 1H), 7.83-7.81 (m, 2H), 7.57-7.44 (m, 3H), 7.31 (s, 1H), 6.79 (s, 1H), 2.86-2.84 (m, 2H), 2.44 (s, 3H), 1.11 (d, J = 7.5 Hz, 3H); ^{13}C NMR (125 MHz, DMSO-d_6): δ 163.8, 156.5, 142.8, 140.0, 134.2, 133.3, 131.4, 131.0, 128.3, 127.9, 126.9, 126.0, 20.5, 15.3, 11.3. IR (neat): 1645, 1476, 1423, 1267, 999, 895, 741 cm^{-1}; HRMS (ESI): Exact mass calcd for $C_{15}H_{17}N_2OS$ $[M+H]^+$: 273.1056, Found: 273.1047.

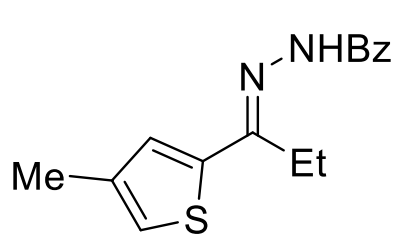

1aq

1aq was obtained in 75% yield as white solid (Rf value: 0.5, PE/EtOAc = 5:1). m.p. 203-205 ºC; ^1H NMR (500 MHz, DMSO-d_6): δ 10.75 (s, 1H), 7.80 (s, 2H), 7.57-7.50 (m, 3H), 7.34 (s, 1H), 7.17 (s, 1H), 2.88-2.86 (m, 2H), 2.21 (s, 3H), 1.12 (t, J = 7.5 Hz, 3H); ^{13}C NMR (125 MHz, DMSO-d_6): δ 164.0. 156.2. 142.1. 137.5. 134.2. 131.4. 130.0. 128.3. 127.9. 124.2. 20.8. 15.5. 11.3. IR (neat): 1869, 1647, 1476, 1421, 1267, 743, 706 cm^{-1}; HRMS (ESI): Exact mass calcd for $C_{15}H_{17}N_2OS$ $[M+H]^+$: 273.1056, Found: 273.1049.

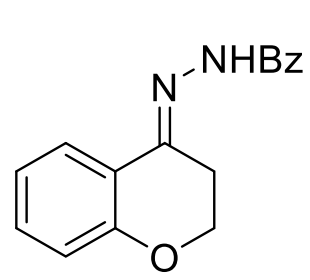

1ar

1ar[10] was obtained in 58% yield as white solid (Rf value: 0.2, PE/EtOAc = 2:1). ^1H NMR (400 MHz, DMSO-d_6): δ 10.87 (s, 1H), 8.02 (s, 1H), 7.88 (d, J = 7.5 Hz, 2H), 7.60-7.57 (m, 1H), 7.53-7.49 (m, 2H), 7.33 (s, 1H), 7.02 (s, 1H), 6.93 (d, J = 8.2 Hz, 1H), 4.27 (t,

J = 6.2 Hz, 2H), 2.96 (t, J = 6.2 Hz, 2H).; ^{13}C NMR (100 MHz, DMSO-d_6): δ 163.8, 157.1, 150.4, 134.0, 131.5, 128.3, 127.9, 124.9, 121.2, 120.3, 117.4, 64.8, 26.0.

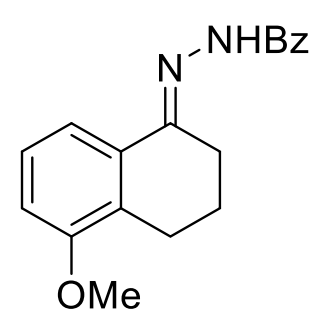

1as

1as was obtained in 66% yield as white solid (Rf value: 0.2, PE/EtOAc = 1:1). m.p. 220-222 ºC; ^{1}H NMR (400 MHz, DMSO-d_6): δ 10.74 (s, 1H), 7.88 (s, 1H), 7.74 (s, 1H), 7.58-7.49 (m, 3H), 7.22 (s, 1H), 7.00-6.98 (m, 3H), 3.80 (s, 3H), 2.75-2.68 (m, 4H), 1.85-1.78 (m, 2H); ^{13}C NMR (100 MHz, DMSO-d_6): δ 163.8, 156.3, 155.5, 134.1, 133.2, 131.4, 128.6, 128.3, 127.9, 126.4, 116.9, 110.9, 55.5, 25.7, 21.9, 20.9; IR (neat): 3230, 1649, 1578, 1452, 1258, 1130, 505 cm^{-1}; HRMS (ESI): Exact mass calcd for $C_{18}H_{18}N_2NaO_2$ $[M+Na]^+$: 317.1260, Found: 317.1255.

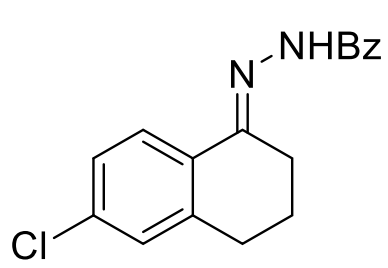

1at

1at was obtained in 58% yield as white solid (Rf value: 0.2, PE/EtOAc = 2:1). m.p. 248-249 ºC; ^{1}H NMR (400 MHz, DMSO-d_6): δ 10.78 (s, 1H), 8.13 (s, 1H), 7.89 (d, *J* = 0.8 Hz, 2H), 7.62-7.51 (m, 3H), 7.33 (s, 2H), 2.81-2.78 (m, 4H), 1.89-1.82 (m, 2H); ^{13}C NMR (100 MHz, DMSO-d_6): δ 163.9, 153.9, 142.3, 134.0, 131.5, 131.3, 128.3, 128.2, 127.9, 126.7, 126.4, 28.6, 25.9, 21.2; IR (neat): 3215, 1651, 1521, 1476, 1275, 1267, 748, 707 cm^{-1}; HRMS (ESI): Exact mass calcd for $C_{17}H_{15}{}^{35}ClN_2NaO$ $[M+Na]^+$: 321.0765, Found: 321.0766.

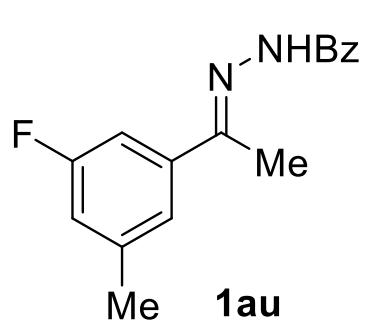

1au

1au was obtained in 82% yield as white solid (Rf value: 0.2, PE/EtOAc = 2:1). m.p. 215-216 ºC; ^{1}H NMR (400 MHz, DMSO-d_6): δ 10.81 (s, 1H), 7.88 (s, 2H), 7.61-7.43 (m, 5H), 7.10 (s, *J* = 9.5 Hz, 1H), 2.37 (s, 3H), 2.36 (s, 3H); ^{13}C NMR (100 MHz, DMSO-d_6): δ 164.0, 162.2 (d, *J* = 242.2 Hz), 153.9, 140.32 (d, *J* = 2.2 Hz), 140.24 (d, *J* = 2.3 Hz), 134.0, 131.4, 128.2, 128.0, 123.1, 116.53 (d, *J* = 21.0 Hz), 110.03 (d, *J* = 22.9 Hz), 20.88 (d, *J* = 1.9 Hz), 14.5; ^{19}F NMR (376 MHz, DMSO-d_6): δ -114.24. IR (neat): 3005, 1717, 1581, 1366, 1223, 1139, 916, 694, 532 cm^{-1}; HRMS (ESI): Exact mass calcd for $C_{16}H_{15}FN_2NaO$ $[M+Na]^+$: 293.1061, Found: 293.1066.

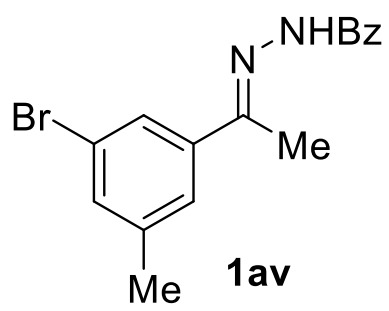

1av

1av was obtained in 70% yield as white solid (Rf value: 0.2, PE/EtOAc = 2:1). m.p. 240-242 ºC; ^{1}H NMR (400 MHz, DMSO-d_6): δ 10.82 (s, 1H), 7.88-7.82 (m, 3H), 7.64-7.46 (m, 5H), 2.36 (s, 3H), 2.35 (s, 3H); ^{13}C NMR (100 MHz, DMSO-d_6): δ 164.1, 153.5, 140.4, 140.2, 134.0, 132.4, 131.6, 128.3, 128.0, 126.2, 126.0, 121.7, 20.7, 14.5. IR (neat): 3005, 2360, 1717, 1436, 1363, 1223, 1093, 530 cm^{-1}; HRMS (ESI): Exact mass calcd for $C_{16}H_{15}{}^{79}BrN_2NaO$ [M+Na]$^+$: 353.0260, Found: 353.0259.

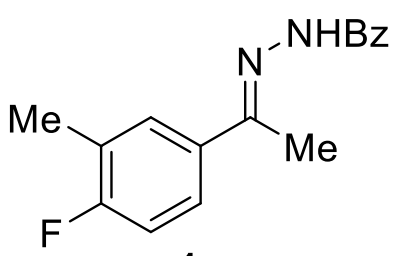

1aw

1aw was obtained in 77% yield as white solid (Rf value: 0.2, PE/EtOAc = 2:1). m.p. 224-226 ºC; ^{1}H NMR (400 MHz, DMSO-d_6): δ 10.75 (s, 1H), 7.88-7.71 (m, 4H), 7.60-7.49 (m, 3H), 7.19 (t, *J* = 9.3 Hz, 1H), 2.35 (s, 3H), 2.29 (s, 3H); ^{13}C NMR (100 MHz, DMSO-d_6): δ 163.9, 161.5 (d, *J* = 246.2 Hz, 1H), 154.8, 134.3 (d, *J* = 3.3 Hz), 134.1, 129.8 (d, *J* = 5.1 Hz), 128.2, 128.9, 126.0, 124.1 (d, *J* = 17.6 Hz), 114.8 (d, *J* = 22.4 Hz), 14.5, 14.2 (d, *J* = 3.2 Hz); ^{19}F NMR (376 MHz, DMSO-d_6): δ -116.43. IR (neat): 3005, 2360, 1717, 1521, 1363, 1224, 1093, 711, 532 cm^{-1}; HRMS (ESI): Exact mass calcd for $C_{16}H_{15}FN_2NaO$ [M+Na]$^+$: 293.1061, Found: 293.1065.

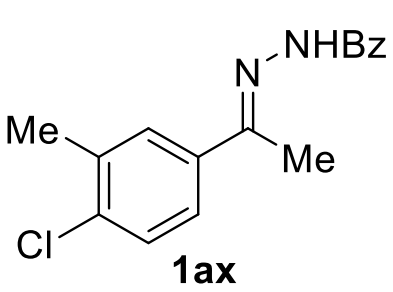

1ax

1ax was obtained in 78% yield as white solid (Rf value: 0.2, PE/EtOAc = 2:1). m.p. 242-243 ºC; ^{1}H NMR (400 MHz, DMSO-d_6): δ 10.79 (s, 1H), 7.88-7.45 (m, 8H), 2.38 (s, 3H), 2.36 (s, 3H); ^{13}C NMR (100 MHz, DMSO-d_6): δ 164.0, 154.2, 137.0, 135.3, 134.4, 134.1, 131.4, 129.0, 128.8, 128.2, 127.9, 125.5, 19.6, 14.4. IR (neat): 3005, 1717, 1544, 1425, 1365, 1223, 1093, 704, 532 cm^{-1}; HRMS (ESI): Exact mass calcd for $C_{16}H_{15}{}^{35}ClN_2NaO$ [M+Na]$^+$: 309.0765, Found: 309.0765.

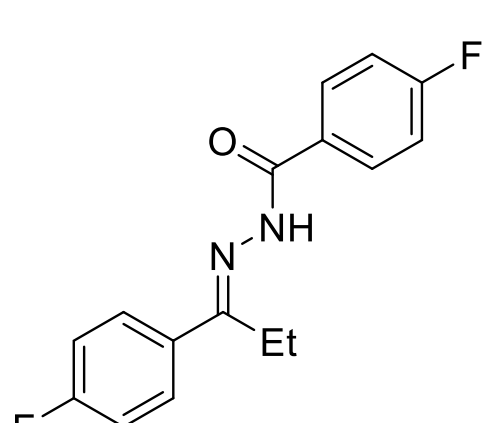

1ay

1ay was obtained in 82% yield as yellow solid (Rf value: 0.2, PE/EtOAc = 2:1). m.p. 221-223 ºC; ^{1}H NMR (400 MHz, DMSO-d_6): δ 10.86 (s, 1H), 7.92-7.87 (m, 4H), 7.36-7.24 (m, 4H), 2.91 (q, *J* = 7.1 Hz, 2H), 1.06 (t, *J* = 7.6 Hz, 3H); ^{13}C NMR (100 MHz, DMSO-d_6): δ 164.5 (d, *J* = 247.2 Hz), 163.8, 163.3 (d, *J* = 245.9 Hz), 158.6, 133.84 (d, *J* = 3.1 Hz), 131.2, 129.3, 115.9, 115.7, 115.5, 20.4, 11.3. ^{19}F NMR (376 MHz, DMSO-d_6): δ -112.11, -112.77. IR (neat): 3173, 1537, 1504, 1275, 1209, 1053,

841, 750 cm^{-1}; HRMS (ESI): Exact mass calcd for $C_{16}H_{14}F_2N_2NaO$ [M+Na]$^+$: 311.0966, Found: 311.0967.

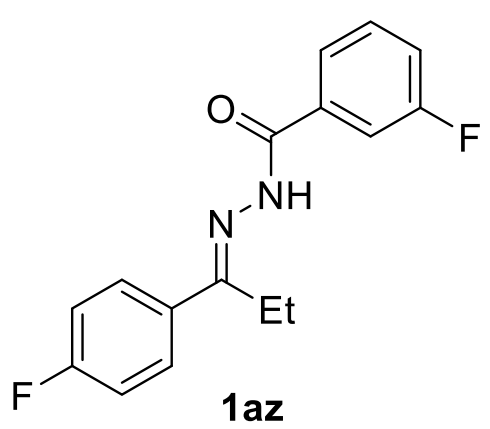
1az

1az was obtained in 76% yield as white solid (Rf value: 0.2, PE/EtOAc = 2:1). m.p. 238-240 °C; ^{1}H NMR (400 MHz, DMSO-d_6): δ 10.94 (s, 1H), 7.91-7.87 (m, 1H), 7.70-7.66 (m, 1H), 7.62-7.48 (m, 2H), 7.37-7.26 (m, 2H), 7.14 (t, *J* = 8.7 Hz, 1H), 2.89-2.82 (m, 2H), 1.08-1.01 (m, 1H); ^{13}C NMR (125 MHz, DMSO-d_6): δ 163.1, 162.9 (d, *J* = 245.1 Hz), 161.8 (d, *J* = 243.9 Hz), 158.8, 136.4, 133.3, 130.5, 129.8 (d, *J* = 8.5 Hz), 128.9, 124.2, 118.32 (d, *J* = 20.3 Hz), 115.4 (d, *J* = 21.6 Hz), 114.8 (d, *J* = 22.6 Hz), 20.0, 10.9. ^{19}F NMR (376 MHz, DMSO-d_6): δ -108.81, -112.24. IR (neat): 3230, 1709, 1670, 1632, 1530, 1277, 1136, 1072, 750 cm^{-1}; HRMS (ESI): Exact mass calcd for $C_{16}H_{14}F_2N_2NaO$ [M+Na]$^+$: 311.0966, Found: 311.0959.

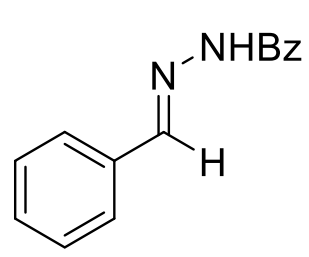
1ba

1ba[6] was obtained in 83% yield as white solid (Rf value: 0.2, PE/EtOAc = 3:1). ^{1}H NMR (400 MHz, DMSO-d_6): δ 11.85 (s, 1H), 7.92 (d, *J* = 7.2 Hz, 1H), 7.75-7.73 (m, 2H), 7.62-7.58 (m, 1H), 7.55-7.51 (m, 2H), 7.48-7.44 (m, 3H),; ^{13}C NMR (100 MHz, DMSO-d_6): δ 163.2, 147.8, 134.4, 133.4, 131.8, 130.1, 128.8, 128.5, 127.6, 127.1.

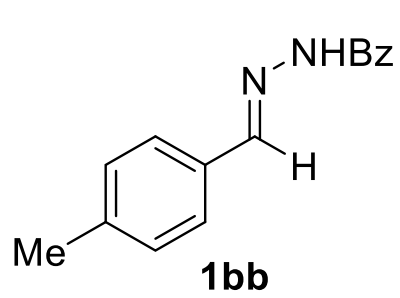
1bb

1bb[6] was obtained in 80% yield as white solid (Rf value: 0.2, PE/EtOAc = 3:1). ^{1}H NMR (400 MHz, DMSO-d_6): δ 11.79 (s, 1H), 8.42 (s ,1H), 7.91 (d, *J* = 7.2 Hz, 1H), 7.64-7.51 (m, 5H), 7.28 (d, *J* = 7.0 Hz, 1H), 2.35 (s ,3H); ^{13}C NMR (100 MHz, DMSO-d_6): δ 163.1, 147.9, 139.9, 133.5, 131.7, 131.6, 129.4, 128.5, 127.6, 127.1, 21.0.

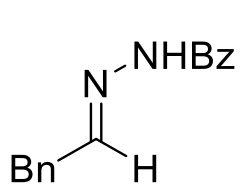
1bc

1bc[6] was obtained in 78% yield as white solid (Rf value: 0.2, PE/EtOAc = 3:1). ^{1}H NMR (400 MHz, DMSO-d_6): δ 11.50 (s, 1H), 7.85-7.81 (m, 3H), 7.56-7.46 (m, 3H), 7.38-7.25 (m, 5H), 3.63 (d, *J* = 5.8 Hz, 2H); ^{13}C NMR (100 MHz, DMSO-d_6): δ 163.0, 150.6, 136.9, 133.5, 131.6, 128.8, 128.7, 128.4, 127.5, 126.6, 38.4.

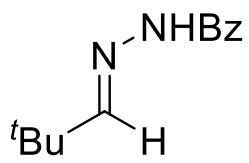

1bd

1bd[6] was obtained in 71% yield as white solid (Rf value: 0.2, PE/EtOAc = 3:1). ^{1}H NMR (400 MHz, DMSO-d_6): δ 11.35 (s, 1H), 7.84-7.82 (m, 2H), 7.70 (s, 1H), 7.75-7.73 (m, 2H), 7.58-7.54 (m, 1H), 7.51-7.47 (m, 2H), 1.10 (s, 9H),; ^{13}C NMR (100 MHz, DMSO-d_6): δ 162.9, 159.2, 133.7, 131.4, 128.4, 127.4, 34.6, 27.2.

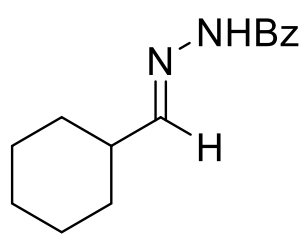

1be

1be[11] was obtained in 63% yield as white solid (Rf value: 0.2, PE/EtOAc = 3:1). ^{1}H NMR (400 MHz, DMSO-d_6): δ 11.36 (s, 1H), 7.83 (d, J = 7.0 Hz, 2H), 7.65 (d, J = 5.5 Hz, 1H), 7.55 (t, J = 7.2 Hz, 1H), 7.48 (t, J = 7.3 Hz, 2H), 2.25 (s, 1H), 1.78-1.62 (m, 5H), 1.29-1.22 (m, 5H); ^{13}C NMR (100 MHz, DMSO-d_6): δ 163.4, 156.3, 134.1, 132.0, 128.9, 128.0, 30.2, 26.0, 25.5.

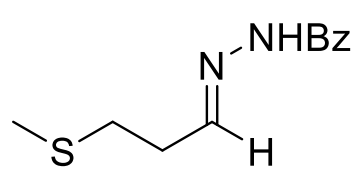

1bf

1bf was obtained in 40% yield as yellow solid (Rf value: 0.2, PE/EtOAc = 3:1). m.p. 202-204 ºC; ^{1}H NMR (400 MHz, CDCl$_3$): δ 10.18 (s, 1H), 7.83 (d, J = 7.3 Hz, 2H), 7.74 (t, J = 5.0 Hz, 1H), 7.48 (t, J = 7.4 Hz, 1H), 7.37 (t, J = 7.6 Hz, 2H), 2.67-2.59 (m, 4H), 2.08 (s, 3H); ^{13}C NMR (100 MHz, CDCl$_3$): δ 164.4, 150.8, 132.9, 131.8, 128.5, 127.4, 31.8, 30.8, 15.3. IR (neat): 3005, 2970, 2341, 1717, 1437, 1364, 1223, 1094, 900, 530 cm^{-1}; HRMS (ESI): Exact mass calcd for $C_{11}H_{14}N_2NaO$ [M+Na]$^+$: 245.0719, Found: 245.0726.

4. Condition optimization

First, attempts were made on PYBOX ligands with different skeletons under the conditions of Jacobsen's work.[12] By using 5 mol% PYBOX **L1**/$ErCl_3$ complex as the catalyst and MeOH as an additive, the reaction of **1a** with 3.0 equivalents of TMSCN in toluene at 0 °C gave the desired product **3a** in 22% yield with only 15% ee after 4 days. The performance of PYBOX ligand **L15**-**L17** bearing different oxazoline moiety was studied, but no better result was obtained (Figure S1).

Figure S1. Screened PYBOX Ligand

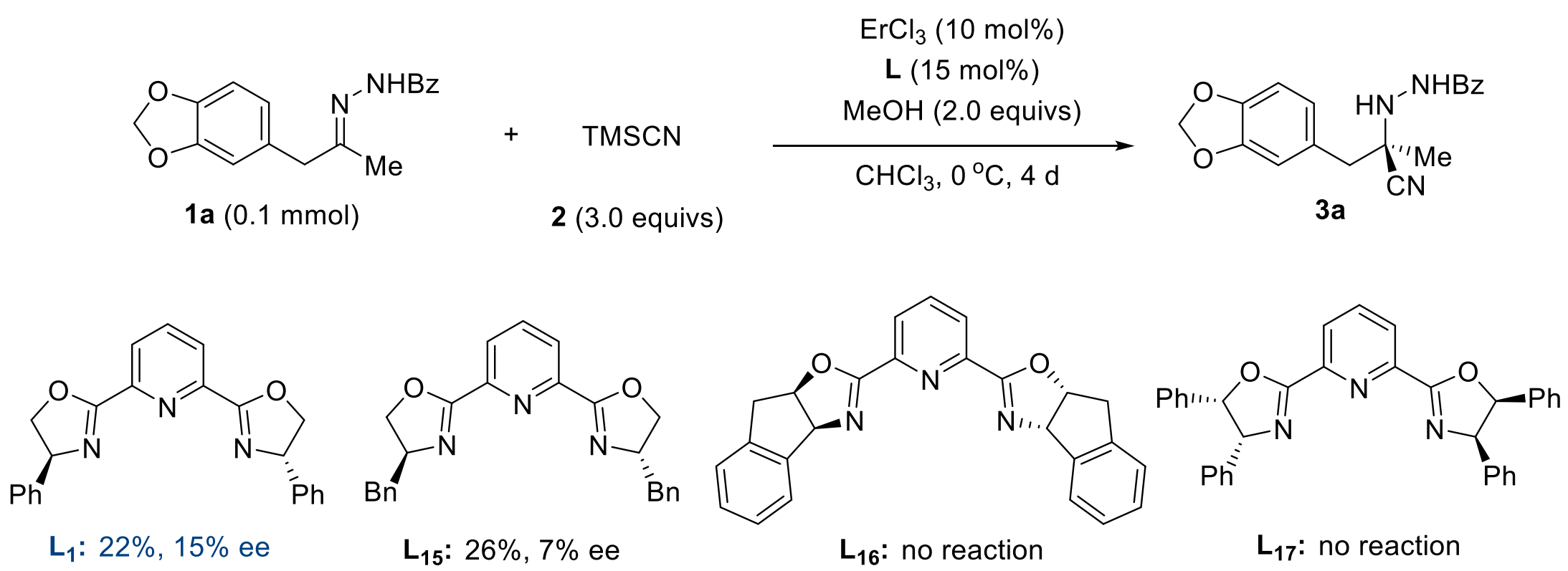

Subsequently, the influence of the solvent was examined by running the reaction at 0 ºC, in the presence of PYBOX ligand **L1** derived $ErCl_3$. As shown in Table S1, by changing $CHCl_3$ to EtOAc the ee decreased from 15% to 10% (entries 1-2). By performing the reaction in THF and Et_2O, 25% and 20% ee were obtained, respectively (entries 3-4). Almost no reaction occurred using CH_3CN as solvent (entry 5). Notably, $ErCl_3$/**L1** demonstrated superior activity in aromatic solvent. Complete conversion of **1a** was achieved in toluene, giving **3a** in 87% yield with 55% ee (entry 6). Then, the reaction in ethylbenzene, butylbenzene, pentylbenzene, *p*-xylene and fluorobenzene was studied, but no better results were obtained (entries 7-11). Then the performance of various Er(III) salts in toluene was systematically evaluated. The $Er(O^iPr)_3$ could give **3a** in 76% yield with only 10% ee, and no reaction occurred by using ErF_3, $Er(SbF_6)_3$, or $Er(BF_4)_3$ as catalyst (entries 12-15). These results highlight the critical role of both the $ErCl_3$ catalyst and solvent for the reaction. Then the performance of PYBOX ligands beating different C4 shielding group was studied, with results show in Figure 2 of the main text.

Table S1. The performance of solvents and Er(III) salts.

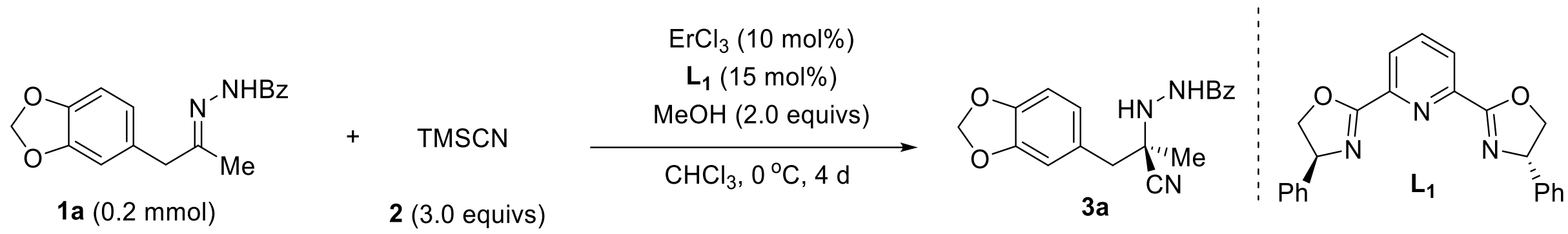

Entry	ErX_3	Solvent	Conv. (%)[a]	Yield (%)[a]	Ee (%)[b]
1	$ErCl_3$	$CHCl_3$	30	22	15
2	$ErCl_3$	EtOAc	22	15	10
3	$ErCl_3$	THF	47	33	25
4	$ErCl_3$	Et_2O	55	40	20
5	$ErCl_3$	CH_3CN	0	NR	-
**6**	**$ErCl_3$**	**Toluene**	**100**	**87**	**55**
7	$ErCl_3$	Ethylbenzene	100	66	42
8	$ErCl_3$	Butylbenzene	100	72	53
9	$ErCl_3$	Pentylbenzene	100	77	51
10	$ErCl_3$	*p*-Xylene	50	40	42
11	$ErCl_3$	Fluorobenzene	63	57	37
12	$Er(O^iPr)_3$	Toluene	100	76	10
13	ErF_3	Toluene	0	NR	-
14	$Er(SbF_6)_3$	Toluene	0	NR	-
15	$Er(BF_4)_3$	Toluene	0	NR	-

[a] The conversion of **1a** and the NMR yield of **3a** was determined by ^1H NMR using 1,3,5-trimethoxybenzene as internal standard. [b] The ee was determined by chiral HPLC.

Notably, for the reaction of 2-hexanone derived hydrazone **1m**, only 70% ee was obtained under the conditions established shown in Figure 2. To further increasing the enantioselectivity for such type of substrates, the influence of solvent and alcohol additives was studied. As shown in Table S2, when $^nBu_2O$ was used as the solvent, the ee of **3m** was significantly improved from 70% to 80% (entry 8).

Furthermore, by changing MeOH to HFIP as an additive, the reaction could give **3m** in increased 85% ee with 82% yield (entry 12).

Table S2. The influence of erbium salts and solvents

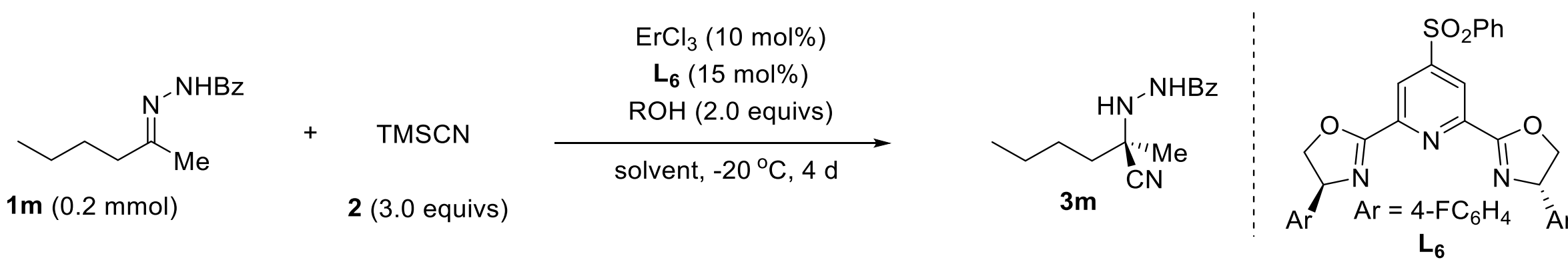

Entry	Solvent	ROH	Conv. (%)[a]	Yield (%)[a]	Ee (%)[b]
1	Toluene	MeOH	100	87	70
2	EtOAc	MeOH	32	30	6
3	$CHCl_3$	MeOH	-	Trace	-
4	CH_3CN	MeOH	0	NR	-
5	THF	MeOH	44	37	5
6	Et_2O	MeOH	50	41	75
7	$^{i}Pr_2O$	MeOH	20	10	52
8	$^{n}Bu_2O$	MeOH	100	73	80
9	MTBE	MeOH	76	62	55
10	Cyclopentyl methyl ether	MeOH	82	77	56
11	$^{n}Bu_2O$	TFE	53	40	70
**12**	**$^{n}Bu_2O$**	**HFIP**	**100**	**82**	**85**

[a] The conversion of **1m** and the NMR yield of **3m** was determined by ^{1}H NMR using 1,3,5-trimethoxybenzene as internal standard. [b] The ee was determined by chiral HPLC.

In addition, we also investigated the effect of PYBOX ligands on the reaction of the α-tert-butyl-α-methyl ketone hydrazone substrate. After evaluation of the ligand, we found that employing PYBOX **L8** afforded the chiral hydrazine **3o** in 96% yield and 90% ee, and other ligands did not achieve ideal results.

Figure S2. Screened PYBOX Ligand for 1o

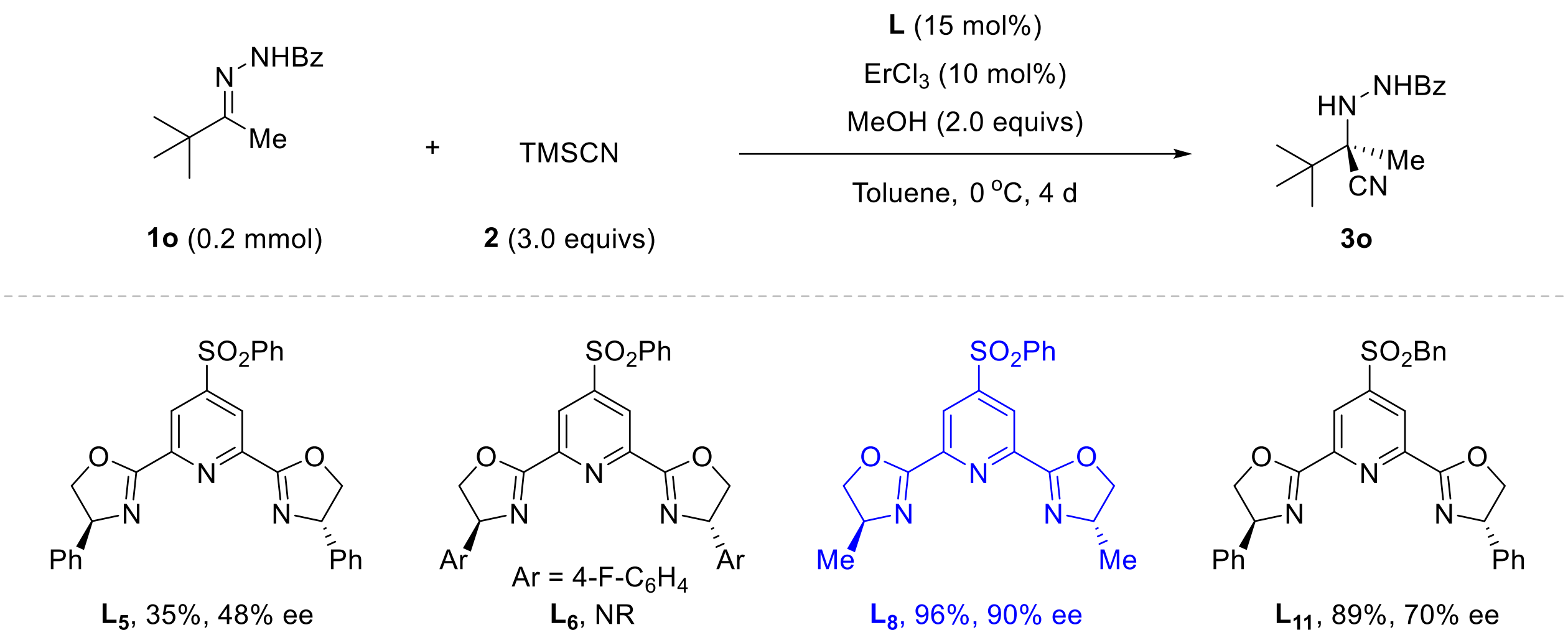

5. Asymmetric catalysis hydrocyanation of hydrazone

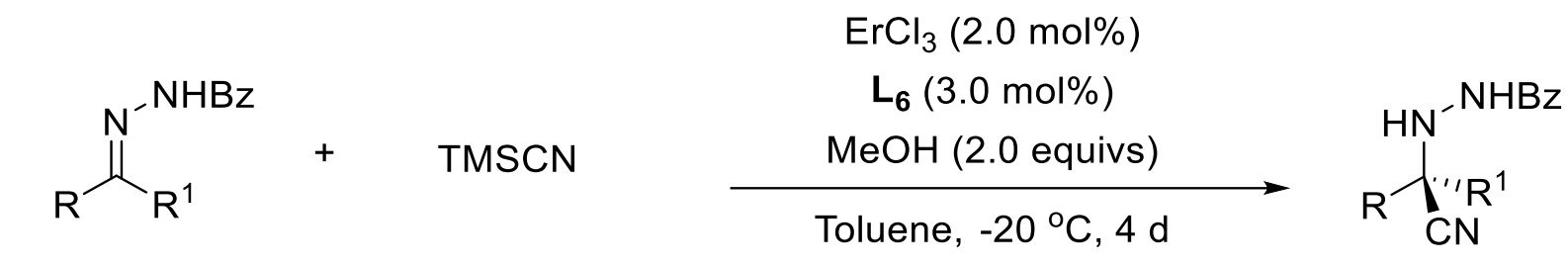

Under an atmosphere of N_2, to a 10 mL oven-dried sealed tube were added $ErCl_3$ (1.1 mg, 0.004 mmol) and **L6** (3.3 mg, 0.006 mmol), followed by 2.0 mL of anhydrous toluene. The solution was stirred at 25 °C for 2 h, and then hydrazone **1** (0.2 mmol) was added. After the mixture was continued to stir at 25 °C for 15 min. Then the mixture was cooled to -20 °C for 0.5 h, TMSCN (59.4 mg, 0.6 mmol) and MeOH (12.8 mg, 0.4 mmol) were added. The resulting mixture was stirred at -20 °C for 4 d till full conversion of **1** by TLC analysis. The mixture was directly subjected to a column chromatography purification using PE/EtOAc (3:1, v/v) as the eluent to afford the desired product **3** or **4**.

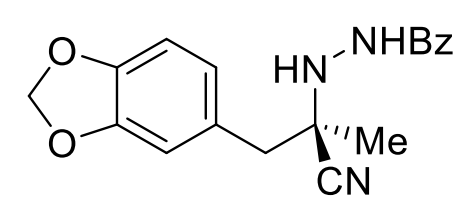

(*R*)-**3a**

(*R*)**-3a** was obtained in 95% yield (61.3 mg) as white solid. HPLC analysis (Chiralcel AD-H, Hexane/^{i}PrOH = 80/20, 1.0 mL/min, 230 nm; t_r (major) = 49.70 min, t_r (minor) = 45.08 min) gave the product: 90% ee, $[\alpha]^{20}_D$ = +54.5 (c = 1.0, $CHCl_3$), m.p. 180-182°C; ^{1}H NMR (400 MHz, $CDCl_3$): δ 7.81 (d, *J* = 6.2 Hz, 1H), 7.74-7.72 (m, 2H), 7.53-7.49 (m, 1H), 7.43-7.39 (m, 2H), 6.85 (s, 1H), 6.79-6.77 (m, 2H), 5.95 (s, 2H), 5.32 (d, *J* = 6.3 Hz, 1H), 3.04 (d, *J* = 13.8 Hz, 1H), 2.91 (d, *J* = 13.8 Hz, 1H), 1.51 (s, 3H); ^{13}C NMR (100 MHz, $CDCl_3$): δ 167.8, 147.8, 147.2, 132.3, 131.8, 128.7, 127.4, 127.1, 123.6, 120.8, 110.6, 108.4, 101.1, 59.7, 44.1, 23.5. IR (neat): 3005, 2970, 2361, 1717, 1437, 1364, 1223, 1094, 530 cm^{-1}; HRMS (ESI): Exact mass calcd for $C_{18}H_{17}N_3NaO_3$ $[M+Na]^+$: 346.1162, Found: 346.1155.

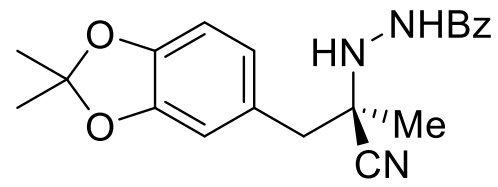

(*R*)-**3b**

(*R*)**-3b** was obtained in 97% yield (67.9 mg) as white solid. HPLC analysis (Chiralcel AD-H, Hexane/^{i}PrOH = 80/20, 1.0 mL/min, 230 nm; t_r (major) = 18.77 min, t_r (minor) = 23.89 min) gave the product: 90% ee, $[\alpha]^{20}_D$ = +98.6 (c = 1.0, $CHCl_3$), m.p. 229-231 °C; ^{1}H NMR (400 MHz, $CDCl_3$): δ 7.73-7.70 (m, 3H), 7.53-7.49 (m, 1H), 7.42-7.38 (m, 2H), 6.78-6.75 (m, 2H), 6.72-6.70 (m, 1H), 5.31 (d, *J* = 6.4 Hz, 1H), 3.02 (d, *J* = 13.8 Hz, 1H),

2.91 (d, J = 13.7 Hz, 1H), 1.67 (s, 3H), 1.66 (s, 3H), 1.52 (s, 3H); ^{13}C NMR (100 MHz, $CDCl_3$): δ 167.6, 147.7, 147.1, 132.2, 131.9, 128.7, 127.1, 126.9, 123.0, 120.8, 118.2, 110.4, 108.3, 59.7, 44.4, 25.83, 25.82, 23.6. IR (neat): 3057, 2970, 2359, 1717, 1497, 1437, 1364, 1225, 908, 530 cm^{-1}; HRMS (ESI): Exact mass calcd for $C_{20}H_{21}N_3NaO_3$ $[M+Na]^+$: 374.1475, Found: 374.1471.

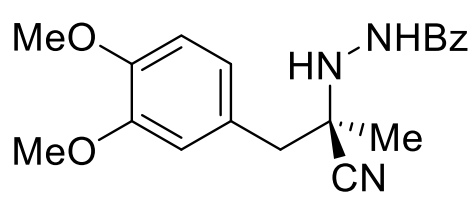
(*R*)-**3c**

(*R*)**-3c** was obtained in 91% yield (61.6 mg) as white solid. HPLC analysis (Chiralcel AD-H, Hexane/^{i}PrOH = 80/20, 1.0 mL/min, 230 nm; t_r (major) = 31.70 min, t_r (minor) = 28.86 min) gave the product: 87% ee, $[\alpha]^{20}_D$ = +16.0 (c = 1.0, $CHCl_3$), m.p. 175-176 ºC; ^{1}H NMR (400 MHz, $CDCl_3$): δ 7.71-7.68 (m, 2H), 7.54-7.50 (m, 2H), 7.42 (t, J = 7.6 Hz, 2H), 6.95-6.85 (m, 3H), 5.29 (s, br, 1H), 3.89 (s, 3H), 3.88 (s, 3H), 3.08 (d, J = 13.8 Hz, 1H), 2.98 (d, J = 13.8 Hz, 1H), 1.55 (s, 3H); ^{13}C NMR (100 MHz, $CDCl_3$): δ 167.6, 149.0, 148.7, 132.3, 131.8, 128.7, 127.1, 126.5, 122.5, 120.9, 113.3, 111.3, 59.8, 55.9, 55.8, 44.4, 23.8. IR (neat): 3265, 1656, 1516, 1452, 1267, 1143, 1026, 770 cm^{-1}; HRMS (ESI): Exact mass calcd for $C_{19}H_{21}N_3NaO$ $[M+Na]^+$: 362.1475, Found: 362.1482.

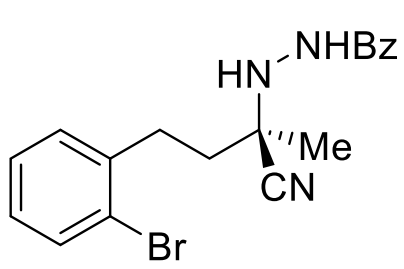
(*R*)-**3d**

(*R*)**-3d** was obtained in 90% yield (66.7 mg) as white solid. HPLC analysis (Chiralpak IE-3, Hexane/^{i}PrOH = 80/20, 1.0 mL/min, 230 nm; t_r (major) = 30.50 min, t_r (minor) = 18.74 min) gave the product: 92% ee, $[\alpha]^{20}_D$ = +28.9 (c = 1.0, $CHCl_3$), m.p. 178-179 ºC; ^{1}H NMR (400 MHz, $CDCl_3$): δ 7.87 (d, J = 6.4 Hz, 1H), 7.81-7.79 (m, 2H), 7.54-7.52 (m, 2H), 7.45-7.42 (m, 2H), 7.27-7.24 (m, 2H), 7.11-7.07 (m, 1H), 5.37 (d, J = 6.5 Hz, 1H), 3.04-2.94 (m, 2H), 2.14-1.99 (m, 2H), 1.62 (s, 3H); ^{13}C NMR (100 MHz, $CDCl_3$): δ 168.2, 139.5, 133.0, 132.4, 131.8, 130.5, 128.7, 128.3, 127.8, 127.2, 124.2, 120.9, 58.8, 38.0, 31.2, 23.1. IR (neat): 3275, 2930, 1657, 1508, 1473, 1313, 775 cm^{-1}; HRMS (ESI): Exact mass calcd for $C_{18}H_{18}{}^{79}BrN_3NaO$ $[M+Na]^+$: 394.0525, Found: 394.0531.

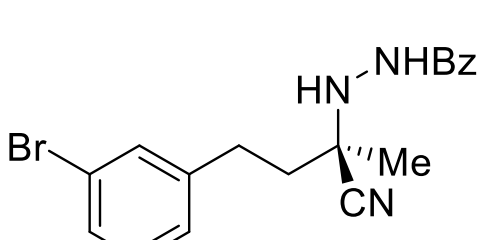
(*R*)-**3e**

(*R*)**-3e** was obtained in 92% yield (68.2 mg) as white solid. HPLC analysis (Chiralpak IE, Hexane/^{i}PrOH = 80/20, 1.0 mL/min, 230 nm; t_r (major) = 20.89 min, t_r (minor) = 15.77 min) gave the product: 92% ee, $[\alpha]^{20}_D$ = +88.5 (c = 1.0, $CHCl_3$),

m.p. 181-183 ºC; ^{1}H NMR (400 MHz, $CDCl_3$): δ 7.81-7.78 (m, 2H), 7.72 (s, br, 1H), 7.57-7.52 (m, 1H), 7.47-7.43 (m, 2H), 7.38-7.34 (m, 2H), 7.19-7.13 (m, 2H), 5.30 (d, *J* = 6.0 Hz, 1H), 2.89-2.84 (m, 2H), 2.16-2.09 (m, 1H), 2.04-1.97 (m, 1H), 1.58 (s, 3H); ^{13}C NMR (100 MHz, $CDCl_3$): δ 168.2, 142.5, 132.5, 131.7, 131.4, 130.2, 129.6, 128.8, 127.2, 127.0, 122.6, 120.8, 59.0, 39.6, 30.3, 23.4. IR (neat): 3018, 1680, 1649, 1473, 1215, 1072, 746 cm^{-1}; HRMS (ESI): Exact mass calcd for $C_{18}H_{18}{}^{79}BrN_3NaO$ $[M+Na]^+$: 394.0525, Found: 394.0529.

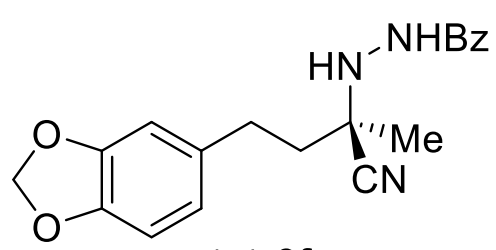

(*R*)-**3f**

(*R*)**-3f** was obtained in 91% yield (61.3 mg) as white solid. HPLC analysis (Chiralpak IF, Hexane/^{i}PrOH = 70/30, 1.0 mL/min, 254 nm; t_r (major) = 35.53 min, t_r (minor) = 19.03 min) gave the product: 90% ee, $[\alpha]^{20}_D$ = +13.1 (c = 1.0, $CHCl_3$), m.p. 212-213 ºC; ^{1}H NMR (400 MHz, $CDCl_3$): δ 7.81-7.78 (m, 2H), 7.63 (d, *J* = 5.2 Hz, 1H), 7.57-7.53 (m, 1H), 7.48-7.43 (m, 2H), 6.74 (d, *J* = 8.0, Hz, 1H), 6.71-6.66 (m, 2H), 5.93 (s, 2H), 5.28 (d, *J* = 5.6 Hz, 1H), 2.84-2.79 (m, 1H), 2.15-2.07 (m, 1H), 2.02-1.95 (m, 1H), 1.58 (s, 3H); ^{13}C NMR (100 MHz, $CDCl_3$): δ 168.2, 147.8, 146.1, 134.0, 132.4, 131.8, 128.8, 127.2, 121.2, 121.0, 108.8, 108.4, 100.9, 59.0, 40.1, 30.5, 23.5. IR (neat): 3275, 1659, 1502, 1489, 1250, 1219, 1040, 771cm^{-1}; HRMS (ESI): Exact mass calcd for $C_{19}H_{19}N_3NaO_3$ $[M+Na]^+$: 360.1319, Found: 360.1317.

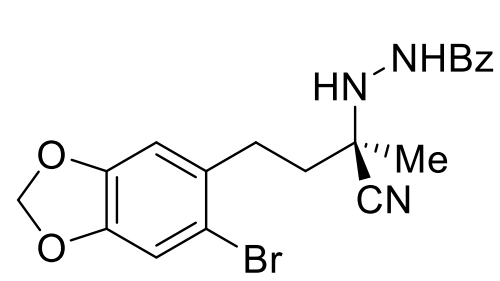

(*R*)-**3g**

(*R*)**-3g** was obtained in 88% yield (73.0 mg) as white solid. HPLC analysis (Chiralpak IE, Hexane/^{i}PrOH = 60/40, 1.0 mL/min, 230 nm; t_r (major) = 16.55 min, t_r (minor) = 11.73 min) gave the product: 90% ee, $[\alpha]^{20}_D$ = +35.0 (c = 1.0, $CHCl_3$), m.p. 229-331 ºC; ^{1}H NMR (400 MHz, $CDCl_3$): δ 7.85 (d, *J* = 6.5 Hz, 1H), 7.81-7.79 (m, 2H), 7.55-7.51 (m, 1H), 7.44 (t, *J* = 7.6 Hz, 2H), 6.98 (s, 1H), 6.74 (s, 1H), 5.94 (s, 2H), 5.34 (d, *J* = 6.4 Hz, 1H), 2.94-2.85 (m, 2H), 2.08-1.94 (m, 2H), 1.60 (s, 3H); ^{13}C NMR (100 MHz, $CDCl_3$): δ 168.2, 147.6, 147.1, 132.4, 132.3, 131.8, 128.8, 127.2, 120.9, 114.2, 112.8, 110.0, 101.7, 58.7, 38.2, 31.1, 23.2. IR (neat): 3291, 1508, 1475, 1456, 1231, 1038, 770 cm^{-1}; HRMS (ESI): Exact mass calcd for $C_{19}H_{18}{}^{79}BrN_3NaO_3$ $[M+Na]^+$: 438.0424, Found: 438.0434.

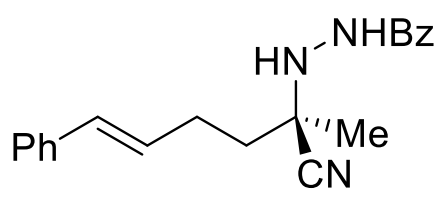

(*R*)-3h

(*R*)**-3h** was obtained in 97% yield (61.8 mg) as white solid. HPLC analysis (Chiralpak IB-3, Hexane/^{i}PrOH = 80/20, 1.0 mL/min, 254 nm; t_r (major) = 30.78 min, t_r (minor) = 24.85 min) gave the product: 92% ee, $[\alpha]^{20}_D$ = +61.1 (c = 1.0, $CHCl_3$), m.p. 230-232 ºC; ^{1}H NMR (500 MHz, $CDCl_3$): δ 7.92 (s, 1H), 7.81 (d, *J* = 7.7 Hz, 2H), 7.53 (t, *J* = 7.4 Hz, 1H), 7.43 (t, *J* = 7.6 Hz, 2H), 7.35-7.26 (m, 4H), 7.22 (t, *J* = 7.2 Hz, 1H), 6.47 (d, *J* = 15.8 Hz, 1H), 6.20 (dt, *J* = 15.7, 6.8 Hz, 1H), 5.34 (s, 1H, br), 2.52-2.47 (m, 2H), 2.06-2.00 (m, 1H), 1.91-1.85 (m, 1H), 1.57 (s, 3H); ^{13}C NMR (125 MHz, $CDCl_3$): δ 168.1, 137.0, 132.4, 131.7, 131.2, 128.7, 128.5, 128.0, 127.24, 127.18, 126.0, 121.1, 58.9, 37.6, 27.9, 23.3. IR (neat): 3271, 1651, 1535, 1458, 1303, 786 cm^{-1}; HRMS (ESI): Exact mass calcd for $C_{20}H_{21}N_3NaO$ $[M+Na]^+$: 342.1577, Found: 342.1573.

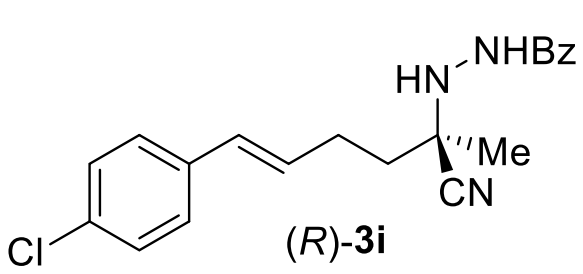

(*R*)**-3i** was obtained in 77% yield (54.0 mg) as white solid. HPLC analysis (Chiralpak IB-3, Hexane/^{i}PrOH = 80/20, 1.0 mL/min, 254 nm; t_r (major) = 16.74 min, t_r (minor) = 15.38 min) gave the product: 93% ee, $[\alpha]^{20}_D$ = +25.3 (c = 1.0, $CHCl_3$), m.p. 230-232 ºC; ^{1}H NMR (400 MHz, $CDCl_3$): δ 7.74-7.71 (m, 3H), 7.49-7.45 (m, 1H), 7.38 (dd, *J* = 8.3, 6.9 Hz, 2H), 7.19 (s, 4H), 6.35 (d, *J* = 15.9 Hz, 1H), 6.10 (dt, *J* = 15.8, 6.8 Hz, 1H), 5.26 (s, 1H, br), 2.46-2.39 (m, 2H), 1.97-1.92 (m, 1H), 1.85-1.77 (m, 1H), 1.50 (s, 3H); ^{13}C NMR (100 MHz, $CDCl_3$): δ 168.2, 135.6, 132.8, 132.4, 131.7, 130.2, 128.8, 128.7, 127.24, 127.17, 121.0, 59.0, 37.5, 27.9, 23.4. IR (neat): 3271, 1656, 1458, 1303, 1095, 709 cm^{-1}; HRMS (ESI): Exact mass calcd for $C_{20}H_{20}ClN_3NaO$ $[M+Na]^+$: 376.1187, Found: 376.1185.

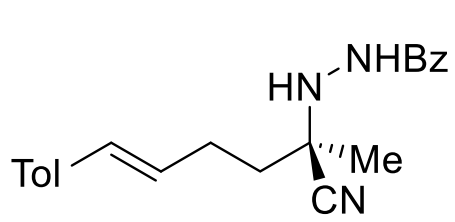

(*R*)-3j

(*R*)**-3j** was obtained in 90% yield (59.4 mg) as white solid. HPLC analysis (Chiralpak IB-3, Hexane/^{i}PrOH = 80/20, 1.0 mL/min, 254 nm; t_r (major) = 15.74 min, t_r (minor) = 14.15 min) gave the product: 90% ee, $[\alpha]^{20}_D$ = +52.5 (c = 1.0, $CHCl_3$), m.p. 222-224 ºC; ^{1}H NMR (400 MHz, $CDCl_3$): δ 7.81-7.79 (m, 3H), 7.53 (t, *J* = 7.4 Hz, 1H), 7.43 (t, *J* = 7.5 Hz, 2H), 7.23 (d, *J* = 7.8 Hz, 2H), 7.10 (d, *J* = 7.7 Hz, 2H), 6.44 (d, *J* = 15.8 Hz, 1H), 6.14 (dt, *J* = 15.8, 6.8 Hz, 1H), 5.31 (s, 1H, br), 2.51-2.45 (m, 2H), 2.32 (s, 3H), 2.06-1.98 (m, 1H), 1.91-1.84 (m, 1H), 1.56 (s, 3H); ^{13}C NMR (100 MHz, $CDCl_3$): δ 168.1, 137.0, 134.3, 132.4, 131.8,

131.1, 129.2, 128.7, 127.2, 127.0, 125.9, 121.0, 59.0, 37.7, 27.9, 23.4, 21.1. IR (neat): 3273, 1651, 1533, 1458, 1311, 848, 763 cm^{-1}; HRMS (ESI): Exact mass calcd for $C_{21}H_{23}N_3NaO$ $[M+Na]^+$: 356.1733, Found: 356.1738.

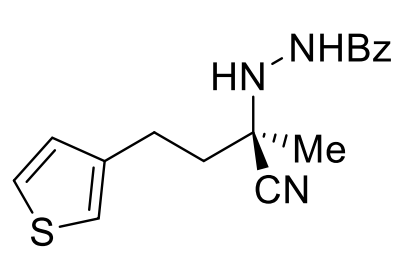

(*R*)-**3k**

(*R*)**-3k** was obtained in 78% yield (46.6 mg) as white solid. HPLC analysis (Chiralpak IF-3, Hexane/^{i}PrOH = 80/20, 1.0 mL/min, 230 nm; t_r (major) = 33.81 min, t_r (minor) = 22.91 min) gave the product: 90% ee, $[\alpha]^{20}_D$ = +40.8 (c = 1.0, $CHCl_3$), m.p. 200-202 ºC; ^1H NMR (500 MHz, $CDCl_3$): δ 7.87-7.81 (m, 3H), 7.56 (t, *J* = 7.5 Hz, 1H), 7.46 (t, *J* = 7.5 Hz, 2H), 7.31-7.28 (m, 1H), 7.03-6.98 (m, 2H), 5.35 (s, 1H), 2.95-2.92 (m, 2H), 2.23-2.16 (m, 1H), 2.09-2.04 (m, 1H), 1.59 (s, 3H); ^{13}C NMR (125 MHz, $CDCl_3$): δ 168.2, 140.3, 132.4, 131.7, 128.7, 127.9, 127.2, 125.9, 121.0, 120.7, 58.9, 38.7, 25.1, 23.3. IR (neat): 3271, 1651, 1535, 1458, 1303, 786, cm^{-1}; HRMS (ESI): Exact mass calcd for $C_{16}H_{17}N_3NaOS$ $[M+Na]^+$: 322.0985, Found: 322.0985.

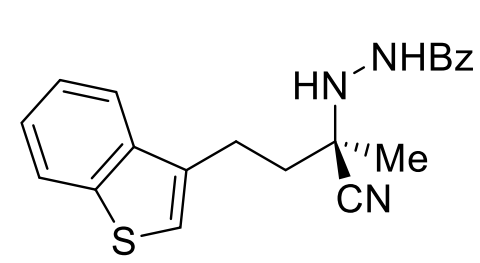

(*R*)-**3l**

(*R*)**-3l** was obtained in 58% yield (40.0 mg) as white solid. HPLC analysis (Chiralpak IA-3, Hexane/^{i}PrOH = 80/20, 1.0 mL/min, 230 nm; t_r (major) = 22.07 min, t_r (minor) = 27.15 min) gave the product: 95% ee, $[\alpha]^{20}_D$ = +23.2 (c = 1.0, $CHCl_3$), m.p. 213-215 ºC; ^1H NMR (500 MHz, $CDCl_3$): δ 7.87 (d, *J* = 7.7 Hz, 1H), 7.79-7.75 (m, 4H), 7.53 (t, *J* = 7.4 Hz, 1H), 7.44-7.35 (m, 4H), 7.16 (s, 1H), 3.16-3.11 (m, 2H), 2.31-2.25 (m, 1H), 2.19-2.13 (m, 1H), 1.63 (s, 3H); ^{13}C NMR (125 MHz, $CDCl_3$): δ 168.2, 140.4, 138.3, 134.4, 132.4, 131.6, 128.7, 127.2, 124.4, 124.1, 123.0, 121.9, 121.4, 121.0, 59.0, 37.5, 23.5, 23.4. IR (neat): 3278, 1651, 1543, 1458, 1311, 848 cm^{-1}; HRMS (ESI): Exact mass calcd for $C_{20}H_{19}N_3NaOS$ $[M+Na]^+$: 372.1141, Found: 372.1143.

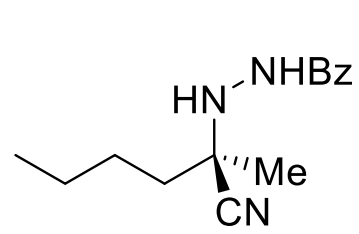

(*R*)-**3m**

(*R*)**-3m** was obtained in 90% (44.1 mg) yield as white solid. HPLC analysis (Chiralpak IF, Hexane/^{i}PrOH = 85/15, 1.0 mL/min, 230 nm; t_r (major) = 19.45 min, t_r (minor) = 14.47 min) gave the product: 85% ee, $[\alpha]^{20}_D$ = +22.6 (c = 1.0, $CHCl_3$), m.p. 164-165 ºC; ^1H NMR (400 MHz, $CDCl_3$): δ 8.08 (s, 1H), 7.80-7.78 (m, 2H), 7.53-7.48 (m, 1H), 7.43-7.39 (m, 2H), 5.31 (s, 1H), 1.82-1.78 (m, 1H), 1.69-1.62 (m, 1H), 1.53-1.51 (m, 2H), 1.49 (s, 3H), 1.41-1.32 (m,

2H), 0.92 (t, J = 7.3 Hz, 3H); ^{13}C NMR (100 MHz, $CDCl_3$): δ 168.1, 132.2, 131.9, 128.6, 127.2, 121.3, 59.2, 37.7, 26.4, 23.3, 22.6, 13.8. IR (neat): 3003, 2963, 2361, 1717, 1437, 1364, 1223, 1094, 530 cm^{-1}; HRMS (ESI): Exact mass calcd for $C_{14}H_{19}N_3NaO$ $[M+Na]^+$: 268.1420, Found: 268.1426.

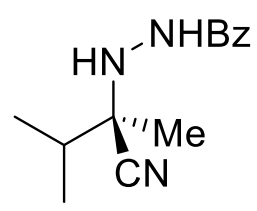

(R)-**3n**

(R)**-3n** was obtained in 90% (41.5 mg) yield as white solid. HPLC analysis (Chiralpak IA-3, Hexane/^{i}PrOH = 95/05, 1.0 mL/min, 230 nm; t_r (major) = 41.69 min, t_r (minor) = 39.33 min) gave the product: 83% ee, $[\alpha]^{20}_D$ = +43.2 (c = 1.0, $CHCl_3$), m.p. 172-174 ºC; ^{1}H NMR (400 MHz, $CDCl_3$): δ 8.00 (s, br, 1H), 7.80-7.77 (m, 2H), 7.52-7.48 (m, 1H), 7.43-7.38 (m, 1H), 5.32 (d, J = 6.4 Hz, 1H), 2.04-1.97 (m, 1H), 1.42 (s, 3H), 1.16 (d, J = 6.8 Hz, 3H), 1.08 (d, J = 6.8 Hz, 3H); ^{13}C NMR (100 MHz, $CDCl_3$): δ 168.0, 132.2, 132.0, 128.6, 127.2, 120.9, 63.3, 34.4, 19.9, 17.8, 16.9. IR (neat): 3277, 1657, 1647, 1458, 1307, 1290, 754, 692 cm^{-1}; HRMS (ESI): Exact mass calcd for $C_{13}H_{17}N_3NaO$ $[M+Na]^+$: 254.1264, Found: 254.1270.

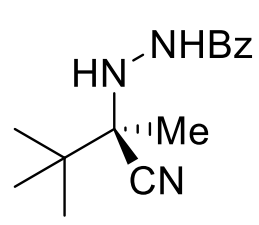

(R)-**3o**

(R)**-3o** was obtained in 96% yield (47.1 mg) as white solid. HPLC analysis (Chiralpak IE, Hexane/^{i}PrOH = 70/30, 1.0 mL/min, 230 nm; t_r (major) = 9.87 min, t_r (minor) = 7.90 min) gave the isomeric composition of the product: 90% ee, $[\alpha]^{20}_D$ = +51.2 (c = 1.0, $CHCl_3$), m.p. 173-175 ºC; ^{1}H NMR (400 MHz, $CDCl_3$): δ 7.81-7.80 (m, 2H), 7.56-7.53 (m, 2H), 7.48-7.45 (m, 2H), 5.41 (d, J = 5.2 Hz, 1H), 1.50 (s, 3H), 1.19 (s, 9H); ^{13}C NMR (100 MHz, $CDCl_3$): δ 168.1, 132.3, 132.0, 128.8, 127.1, 120.9, 66.5, 36.8, 25.4, 19.1. IR (neat): 3270, 1636, 1474, 1267, 999, 896, 745, 706 cm^{-1}; HRMS (ESI): Exact mass calcd for $C_{14}H_{20}N_3O$ $[M+H]^+$: 246.1601, Found: 246.1592.

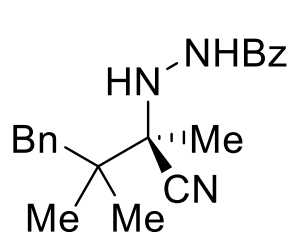

(R)-**3p**

(R)**-3p** was obtained in 70% yield (44.9 mg) as white solid. HPLC analysis (Chiralpak IA, Hexane/^{i}PrOH = 80/20, 1.0 mL/min, 230 nm; t_r (major) = 9.71 min, t_r (minor) = 7.86 min) gave the isomeric composition of the product: 94% ee, $[\alpha]^{20}_D$ = +62.0 (c = 0.6, $CHCl_3$), m.p. 183-185 ºC; ^{1}H NMR (400 MHz, $CDCl_3$): δ 7.83 (d, J = 7.2 Hz, 2H), 7.64 (d, J = 6.4 Hz, 1H), 7.57-7.54 (m, 1H), 7.49-7.45 (m, 2H), 7.32-7.26 (m, 3H), 7.18 (d, J = 6.8 Hz, 2H), 5.50 (d, J = 6.4Hz, 1H), 2.88 (s, 2H), 1.61 (s, 3H), 1.08 (s, 6H); ^{13}C NMR (100 MHz, $CDCl_3$): δ 168.2, 137.1, 132.4, 131.9, 131.1, 128.8, 128.0, 127.1, 126.5, 120.8, 67.4, 42.5, 40.6, 21.9, 21.6, 19.2. IR (neat): 3273, 1636, 1603,

1423, 1267, 999, 743, 706 cm^{-1}; HRMS (ESI): Exact mass calcd for $C_{20}H_{24}N_3O$ $[M+H]^+$: 322.1914, Found: 322.1909.

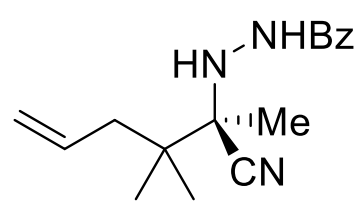
(*R*)-**3q**

(*R*)**-3q** was obtained in 55% yield (29.7 mg) as white solid. HPLC analysis (Chiralpak IE, Hexane/^{i}PrOH = 80/20, 1.0 mL/min, 230 nm; t_r (major) = 18.86 min, t_r (minor) = 11.41 min) gave the isomeric composition of the product: 80% ee, $[\alpha]^{20}_D$ = +22.1 (c = 0.6, $CHCl_3$), m.p. 173-175 °C; ^{1}H NMR (500 MHz, $CDCl_3$): δ 7.82-7.80 (m, 2H), 7.63 (d, *J* = 6.5 Hz, 1H), 7.56-7.53 (m, 1H), 7.47-7.44 (m, 2H), 5.89-5.82 (m, 1H), 5.44 (d, *J* = 7.0 Hz, 1H), 5.17-5.13 (m, 2H), 2.32 (d, *J* = 7.0 Hz, 2H), 1.53 (s, 3H), 1.15 (s, 3H), 1.14 (s, 3H); ^{13}C NMR (125 MHz, $CDCl_3$): δ 168.1, 133.7, 132.3, 131.9, 128.8, 127.1, 120.8, 118.9, 67.0, 41.5, 39.6, 25.3, 22.2, 20.0, 19.1. IR (neat): 3270, 1636, 1489, 1267, 999, 897, 743, 706 cm^{-1}; HRMS (ESI): Exact mass calcd for $C_{16}H_{22}N_3O$ $[M+H]^+$: 272.1757, Found: 272.1750.

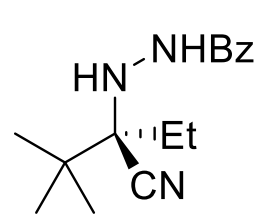
(*R*)-**3r**

(*R*)**-3r** was obtained in 52% yield (27.1 mg) as white solid. HPLC analysis (Chiralpak IE, Hexane/^{i}PrOH = 70/30, 1.0 mL/min, 254 nm; t_r (major) = 11.69 min, t_r (minor) = 14.42 min) gave the isomeric composition of the product: 80% ee, $[\alpha]^{20}_D$ = +25.7 (c = 0.6, $CHCl_3$), m.p. 173-175 °C; ^{1}H NMR (500 MHz, $CDCl_3$): δ 7.77 (d, *J* = 7.5 Hz, 2H), 7.54-7.42 (m, 4H), 5.37 (d, *J* = 7.0 Hz, 1H), 1.92-1.86 (m, 1H), 1.73-1.66 (m, 1H), 1.33 (t, *J* = 7.5 Hz, 2H), 1.17 (s, 9H); ^{13}C NMR (125 MHz, $CDCl_3$): δ 167.9, 132.2, 132.1, 128.7, 127.1, 118.6, 71.3, 38.5, 25.8, 10.6. IR (neat): 3266, 1636, 1474, 1267, 897, 741, 706 cm^{-1}; HRMS (ESI): Exact mass calcd for $C_{15}H_{22}N_3O$ $[M+H]^+$: 260.1757, Found: 260.1754.

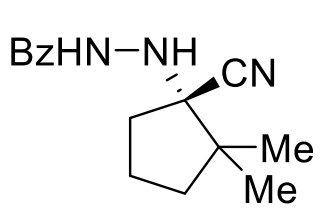
(*R*)-**3s**

(*R*)**-3s** was obtained in 67% yield (34.3 mg) as white solid. HPLC analysis (Chiralpak IE, Hexane/^{i}PrOH = 70/30, 1.0 mL/min, 254 nm; t_r (major) = 17.45 min, t_r (minor) = 14.67 min) gave the isomeric composition of the product: 76% ee, $[\alpha]^{20}_D$ = 20.7 (c = 0.5, $CHCl_3$), m.p. 180-182 °C; ^{1}H NMR (400 MHz, $CDCl_3$): δ 7.80-7.78 (m, 2H), 7.72 (d, *J* = 5.6 Hz, 1H), 7.54-7.51 (m, 1H), 7.45-7.42 (m, 2H), 5.27 (d, *J* = 6.4Hz, 1H), 2.26-2.12 (m, 2H), 1.90-1.70 (m, 4H), 1.24 (s, 3H), 1.19 (s, 3H); ^{13}C NMR (100 MHz, $CDCl_3$): δ 167.9, 132.2, 132.0, 128.7, 127.1, 120.4, 71.0, 46.0, 37.9,

34.5, 26.1, 21.6, 19.5. IR (neat): 3269, 1636, 1603, 1474, 1267, 739, 706 cm^{-1}; HRMS (ESI): Exact mass calcd for $C_{15}H_{20}N_3O$ $[M+H]^+$: 258.1601, Found: 258.1597.

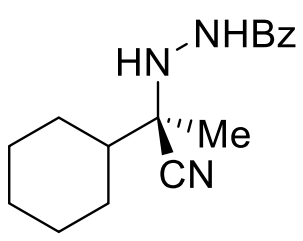
(*R*)-**3t**

(*R*)**-3t** was obtained in 99% yield (53.6 mg) as white solid. HPLC analysis (Chiralpak IA, Hexane/^{i}PrOH = 85/15, 1.0 mL/min, 230 nm; t_r (major) = 23.54 min, t_r (minor) = 17.08 min) gave the product: 92% ee, $[\alpha]^{20}_D$ = +58.4 (c = 1.0, $CHCl_3$), m.p. 196-198 ºC; ^{1}H NMR (400 MHz, $CDCl_3$): δ 8.06 (s, 1H, br), 7.78 (d, *J* = 7.6 Hz, 2H), 7.52-7.47 (m, 1H), 7.42-7.37 (m, 2H), 5.33 (d, *J* = 7.1 Hz, 1H), 2.03-1.93 (m, 2H), 1.84-1.80 (m, 2H), 1.71-1.60 (m, 2H), 1.42 (s, 3H), 1.27-1.13 (m, 5H); ^{13}C NMR (100 MHz, $CDCl_3$): δ 168.0, 132.1, 132.0, 128.6, 127.2, 121.0, 62.9, 44.0, 27.9, 27.0, 26.0, 25.9, 25.8, 20.4. IR (neat): 3005, 2930, 2362, 1717, 1437, 1364, 1223, 1091, 532 cm^{-1}; HRMS (ESI): Exact mass calcd for $C_{16}H_{21}N_3NaO$ $[M+Na]^+$: 294.1577, Found: 294.1581.

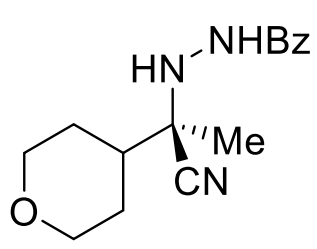
(*R*)-**3u**

(*R*)**-3u** was obtained in 96% yield (52.4 mg) as white solid. HPLC analysis (Chiralpak IA, Hexane/^{i}PrOH = 85/15, 1.0 mL/min, 230 nm; t_r (major) = 28.12 min, t_r (minor) = 26.39 min) gave the product: 95% ee, $[\alpha]^{20}_D$ = +21.3 (c = 1.0, $CHCl_3$), m.p. 177-179 ºC; ^{1}H NMR (400 MHz, $CDCl_3$): δ 7.84-7.79 (m, 3H), 7.56-7.52 (m, 1H), 7.46-7.43 (m, 2H), 5.26 (d, *J* = 6.3 Hz, 1H), 4.08-4.03 (m, 2H), 3.42-3.34 (m, 2H), 1.96-1.89 (m, 2H), 1.86-1.83 (m, 1H), 1.67-1.56 (m, 2H), 1.47 (s, 3H); ^{13}C NMR (100 MHz, $CDCl_3$): δ 168.3, 132.4, 131.8, 128.8, 127.2, 120.3, 67.5, 62.5, 41.6, 28.0, 27.0, 20.2. IR (neat): 3003, 2853, 2359, 1717, 1437, 1364, 1223, 1092, 530 cm^{-1}; HRMS (ESI): Exact mass calcd for $C_{15}H_{19}N_3NaO_2$ $[M+Na]^+$: 296.1369, Found: 296.1377.

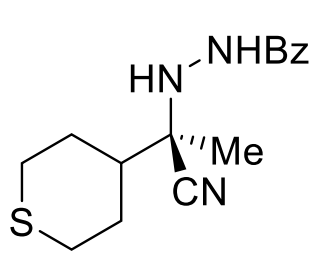
(*R*)-**3v**

(*R*)**-3v** was obtained in 96% yield (55.4 mg) as white solid. HPLC analysis (Chiralpak IA, Hexane/^{i}PrOH = 70/30, 1.0 mL/min, 230 nm; t_r (major) = 38.59 min, t_r (minor) = 12.51 min) gave the product: 90% ee, $[\alpha]^{20}_D$ = +12.7 (c = 1.0, $CHCl_3$), m.p. 183-184 ºC; ^{1}H NMR (400 MHz, $CDCl_3$): δ 7.79 (d, *J* = 7.6 Hz, 2H), 7.68 (d, *J* = 6.3 Hz, 1H), 7.55 (t, *J* = 7.4 Hz, 1H), 7.45 (t, *J* = 7.5 Hz, 2H), 5.20 (d, *J* = 6.2 Hz, 1H), 2.74-2.68 (m, 4H), 2.37 (t, *J* = 12.2 Hz, 2H), 1.74-1.56 (m, 4H), 1.46 (s, 3H); ^{13}C NMR (100 MHz, $CDCl_3$): δ 168.2, 132.4, 131.8, 128.8, 127.2,

120.6, 62.9, 43.5, 29.6, 28.8, 28.7, 28.1, 19.7. IR (neat): 3001, 1717, 1664, 1437, 1364, 1269, 1092, 750 cm^{-1}; HRMS (ESI): Exact mass calcd for $C_{15}H_{19}N_3NaOS$ $[M+Na]^+$: 312.1141, Found: 312.1134.

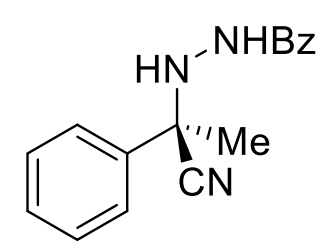

(*R*)-**3w**

(*R*)-**3w**[4] was obtained in 77% yield (40.8 mg) as white solid. HPLC analysis (Chiralcel OD-H, Hexane/^{i}PrOH = 90/10, 1.0 mL/min, 230 nm; t_r (major) = 19.12 min, t_r (minor) = 26.34 min) gave the product: 90% ee, $[\alpha]^{20}_D$ = +66.7 (c = 1.0, $CHCl_3$); ^{1}H NMR (400 MHz, $CDCl_3$): 7.71-7.68 (m, 4H), 7.52-7.49 (m, 2H), 7.47-7.38 (m, 5H), 5.79 (d, *J* = 6.8 Hz, 1H), 1.88 (s, 3H); ^{13}C NMR (100 MHz, $CDCl_3$): δ 167.5, 137.8, 132.3, 131.8, 129.2, 129.1, 128.7, 127.1, 125.9, 120.5, 62.7, 27.3.

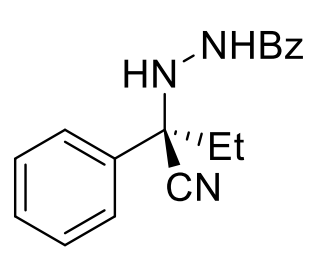

(*R*)-**3x**

(*R*)-**3x** was obtained in 97% yield (40.8 mg) as white solid. HPLC analysis (Chiralpak IE, Hexane/^{i}PrOH = 75/25, 1.0 mL/min, 230 nm; t_r (major) = 14.97 min, t_r (minor) = 13.70 min) gave the product: 84% ee, $[\alpha]^{20}_D$ = +90.9 (c = 1.0, $CHCl_3$), m.p. 182-184°C; ^{1}H NMR (400 MHz, $CDCl_3$): δ 7.72 (s, br, 1H), 7.67-7.62 (m, 4H), 7.49-7.33 (m, 6H), 5.88 (d, *J* = 7.2 Hz, 1H), 2.23-2.16 (m, 1H), 2.07-1.98 (m, 1H), 0.97 (t, *J* = 7.4 Hz, 3H); ^{13}C NMR (100 MHz, $CDCl_3$): δ 167.3, 136.4, 132.1, 131.9, 129.0, 128.9, 128.5, 127.1, 126.5, 119.6, 68.4, 33.2, 8.8. IR (neat): 3005, 2970, 2360, 2341, 1717, 1437, 1364, 1223, 1094, 530 cm^{-1}; HRMS (ESI): Exact mass calcd for $C_{17}H_{17}N_3NaO$ $[M+Na]^+$: 302.1264, Found: 302.1263.

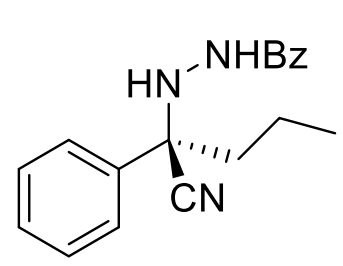

(*R*)-**3y**

(*R*)-**3y** was obtained in 99% yield (58.0 mg) as white solid. HPLC analysis (Chiralpak IA, Hexane/^{i}PrOH = 90/10, 1.0 mL/min, 230 nm; t_r (major) = 30.69 min, t_r (minor) = 23.82 min) gave the product: 86% ee, $[\alpha]^{20}_D$ = +107.4 (c = 1.0, $CHCl_3$), m.p. 178-180 °C; ^{1}H NMR (400 MHz, $CDCl_3$): δ 7.68-7.65 (m, 4H), 7.52-7.37 (m, 7H), 5.91 (d, *J* = 7.4 Hz, 1H), 2.17-2.10 (m, 1H), 2.01-1.94 (m, 1H), 1.63-1.54 (m, 1H), 1.31-1.24 (m, 1H), 0.93 (t, *J* = 7.3 Hz, 3H); ^{13}C NMR (100 MHz, $CDCl_3$): δ 167.3, 136.8, 132.0, 131.9, 129.0, 128.8, 128.5, 127.1, 126.4, 119.7, 67.5, 42.0, 17.8, 13.7. IR (neat): 3005, 2873, 2361, 1717, 1437, 1364, 1223, 1094, 700, 530 cm^{-1}; HRMS (ESI): Exact mass calcd for $C_{18}H_{19}N_3NaO$ $[M+Na]^+$: 316.1420, Found: 316.1411.

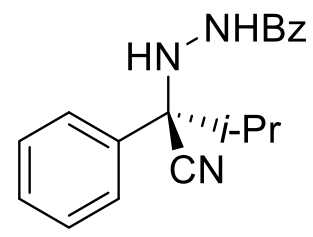
(*R*)-**3z**

(*R*)-**3z** was obtained in 78% yield (45.5 mg) as white solid. HPLC analysis (Chiralpak IE, Hexane/^{i}PrOH = 85/15, 1.0 mL/min, 230 nm; t_r (major) = 26.65 min, t_r (minor) = 22.05 min) gave the isomeric composition of the product: 90% ee, $[\alpha]^{20}_D$ = +110.2 (c = 1.0, $CHCl_3$), m.p. 181-183 ºC; ^1H NMR (400 MHz, $CDCl_3$): δ 7.62 (q, *J* = 6.8 Hz, 4H), 7.50 – 7.33 (m, 6H), 7.24 (s, 1H), 6.17 (d, *J* = 7.6 Hz, 1H), 2.31 (p, *J* = 6.8 Hz, 1H), 1.32 (d, *J* = 6.4 Hz, 3H), 0.87 (d, *J* = 6.8 Hz, 3H); ^{13}C NMR (100 MHz, $CDCl_3$) δ 167.0, 136.2, 132.1, 131.9, 129.0, 128.8, 128.6, 127.0, 126.9, 118.8, 72.4, 37.1, 17.9, 17.8. IR (neat): 3269, 1636, 1429, 1267, 1026, 897, 742 cm^{-1}; HRMS (ESI): Exact mass calcd for $C_{18}H_{20}N_3O$ $[M+H]^+$: 294.1601, Found: 294.1597.

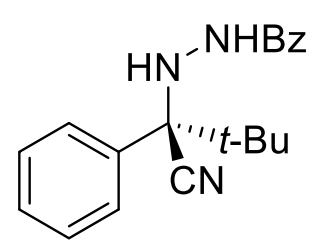
(*R*)-**3aa**

(*R*)-**3aa** was obtained in 72% yield (44.0 mg) as white solid. HPLC analysis (Chiralpak IA, Hexane/^{i}PrOH = 75/25, 1.0 mL/min, 230 nm; t_r (major) = 5.24 min, t_r (minor) = 5.89 min) gave the isomeric composition of the product: 85% ee, $[\alpha]^{20}_D$ = +96.3 (c = 0.8, $CHCl_3$), m.p. 188-190 ºC; ^1H NMR (400 MHz, $CDCl_3$): δ 7.69 (d, *J* = 6.4 Hz, 2H), 7.59 (d, *J* = 6.8 Hz, 2H), 7.51 – 7.32 (m, 6H), 7.23 (d, *J* = 7.6 Hz, 1H), 6.29 (d, *J* = 8.0 Hz, 1H), 1.17 (s, 9H); ^{13}C NMR (100 MHz, $CDCl_3$): δ 166.9, 134.6, 132.1, 131.9, 128.9, 128.6, 128.3, 127.0, 120.0, 74.8, 38.3, 25.7. IR (neat): 3271, 1645, 1489, 1420, 1267, 741, 706 cm^{-1}; HRMS (ESI): Exact mass calcd for $C_{19}H_{22}N_3O$ $[M+H]^+$: 308.1757, Found: 308.1751.

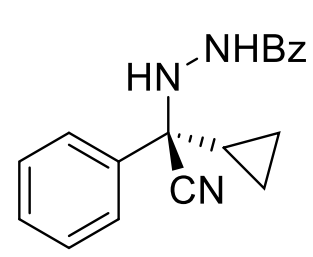
(*R*)-**3ab**

(*R*)-**3ab** was obtained in 77% yield (44.9 mg) as white solid. HPLC analysis (Chiralpak IE, Hexane/^{i}PrOH = 80/20, 1.0 mL/min, 230 nm; t_r (major) = 16.43 min, t_r (minor) = 13.82 min) gave the isomeric composition of the product: 84% ee, $[\alpha]^{20}_D$ = +95.2 (c = 1.0, $CHCl_3$), m.p. 188-190 ºC; ^1H NMR (400 MHz, $CDCl_3$): δ 7.72-7.66 (m, 4H), 7.55-7.37 (m, 7H), 6.01 (d, *J* = 5.6 Hz, 1H), 1.48-1.43 (m, 1H), 0.98-0.93 (m, 1H), 0.84-0.79 (m, 1H), 0.63-0.57 (m, 2H); ^{13}C NMR (100 MHz, $CDCl_3$): δ 167.2, 137.4, 132.2, 132.0, 129.1, 129.0, 128.6, 127.1, 126.3, 117.4, 70.1, 19.4, 3.6, 1.7. IR (neat): 3270, 1636, 1474, 1267, 1001, 897, 743, 706 cm^{-1}; HRMS (ESI): Exact mass calcd for $C_{18}H_{18}N_3O$ $[M+H]^+$: 292.1444, Found: 292.1441.

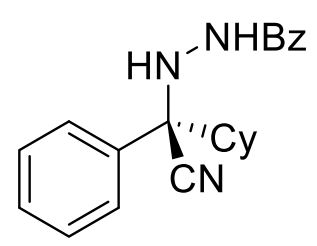

(R)-**3ac**

(*R*)**-3ac** was obtained in 65% yield (43.3 mg) as white solid. HPLC analysis (Chiralpak IE, Hexane/^{i}PrOH = 85/15, 1.0 mL/min, 230 nm; t_r (major) = 18.13 min, t_r (minor) = 15.06 min) gave the isomeric composition of the product: 83% ee, $[\alpha]^{20}_D$ = +88.3 (c = 1.0, $CHCl_3$), m.p. 192-193 °C; ^1H NMR (400 MHz, $CDCl_3$): δ 7.64-7.58 (m, 4H), 7.47-7.16 (m, 7H), 6.19 (d, *J* = 5.2 Hz, 1H), 2.18 (d, *J* = 9.6 Hz, 1H), 1.97-1.88 (m, 2H), 1.70-1.66 (m, 2H), 1.44-1.38 (m, 2H), 1.33-1.26 (m, 1H), 1.16-1.07 (m, 3H); ^{13}C NMR (100 MHz, $CDCl_3$): δ 167.0, 136.3, 132.0, 132.0, 128.9, 128.7, 128.6, 127.0, 119.1, 71.9, 46.4, 28.2, 27.7, 25.9, 25.8, 25.7. IR (neat): 3275, 1636, 1423, 1267, 999, 870, 743, 706 cm^{-1}; HRMS (ESI): Exact mass calcd for $C_{21}H_{24}N_3O$ $[M+H]^+$: 334.1914, Found: 334.1908.

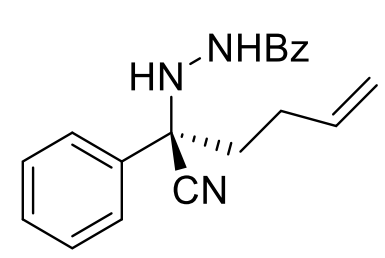

(R)-**3ad**

(*R*)**-3ad** was obtained in 93% yield (56.7 mg) as colorless oil. HPLC analysis (Chiralpak IE, Hexane/^{i}PrOH = 80/20, 1.0 mL/min, 230 nm; t_r (major) = 26.82 min, t_r (minor) = 22.34 min) gave the product: 75% ee, $[\alpha]^{20}_D$ = +61.5 (c = 1.0, $CHCl_3$), m.p. 174-176 °C; ^1H NMR (400 MHz, $CDCl_3$): δ 7.68-7.65 (m, 4H), 7.55 (s, br, 1H), 7.50-7.35 (m, 6H), 5.94 (d, *J* = 7.1 Hz, 1H), 5.77-5.69 (m, 1H), 5.06-4.97 (m, 2H), 2.30-2.22 (m, 2H), 2.14-2.06 (m, 1H), 2.00-1.98 (m, 1H); ^{13}C NMR (100 MHz, $CDCl_3$): δ 167.3, 136.4, 136.0, 132.2, 131.9, 129.2, 129.0, 128.6, 127.1, 126.4, 119.5, 116.0, 67.4, 39.1, 28.6. IR (neat): 3273, 1643, 1518, 1448, 1307, 1290, 918, 698 cm^{-1}; HRMS (ESI): Exact mass calcd for $C_{19}H_{19}N_3NaO$ $[M+Na]^+$: 328.1420, Found: 328.1426.

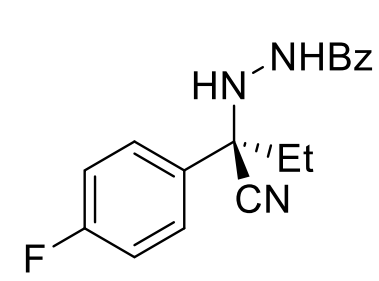

(R)-**3ae**

(*R*)**-3ae** was obtained in 90% (53.4 mg) yield as white solid. HPLC analysis (Chiralpak IA, Hexane/^{i}PrOH = 80/20, 1.0 mL/min, 230 nm; t_r (major) = 13.86 min, t_r (minor) = 12.54 min) gave the isomeric composition of the product: 96% ee, $[\alpha]^{20}_D$ = +97.7 (c = 1.0, $CHCl_3$), m.p. 210-211 °C; ^1H NMR (400 MHz, $CDCl_3$): δ 7.68-7.63 (m, 4H), 7.51-7.49 (m, 1H), 7.43-7.39 (m, 2H), 7.31 (d, *J* = 7.1 Hz, 1H), 7.16-7.12 (m, 2H), 5.86 (d, *J* = 7.2 Hz, 1H), 2.26-2.17 (m, 1H), 2.07-1.98 (m, 1H), 1.01 (t, *J* = 7.4 Hz, 3H); ^{13}C NMR (100 MHz, $CDCl_3$): δ 167.5,162.9 (d, *J* = 248.7 Hz), 132.2 (d, *J* = 15.1 Hz), 132.1, 131.8, 128.5, 128.4 (d, *J* = 8.4 Hz), 127.1, 119.4, 115.8 (d, *J* = 21.7 Hz), 67.8, 33.2, 8.7; ^{19}F NMR (376 MHz, $CDCl_3$): δ -112.36. IR (neat): 3005, 2970, 2362, 1717,

1437, 1364, 1223, 1094, 530 cm^{-1}; HRMS (ESI): Exact mass calcd for $C_{17}H_{16}FN_3NaO$ [M+Na]$^+$: 320.1170, Found: 320.1166.

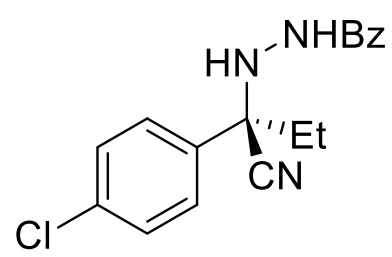
(*R*)-**3af**

(*R*)**-3af** was obtained in 99% yield (61.8 mg) as white solid. HPLC analysis (Chiralpak IA, Hexane/^{i}PrOH = 90/10, 1.0 mL/min, 230 nm; t_r (major) = 36.37 min, t_r (minor) = 33.39 min) gave the product: 94% ee, $[\alpha]^{20}_D$ = +143.9 (c = 1.0, $CHCl_3$), m.p. 217-219 °C; ^1H NMR (400 MHz, $CDCl_3$): δ 7.68-7.66 (m, 2H), 7.63-7.59 (m, 2H), 7.52 (tt, J = 7.6, 1.2 Hz, 1H), 7.45-7.39 (m, 4H), 7.29-7.27 (m, 1H), 5.87 (d, *J* = 7.1 Hz, 1H), 2.27-2.17 (m, 1H), 2.06-1.97 (m, 1H), 1.01 (t, *J* = 7.4 Hz, 3H); ^{13}C NMR (100 MHz, $CDCl_3$): δ 167.5, 135.1, 135.0, 132.2, 131.7, 129.0, 128.6, 128.0, 127.1, 119.2, 67.9, 33.2, 8.7. IR (neat): 3005, 2970, 2360, 2341, 1717, 1437, 1364, 1223, 1094 cm^{-1}; HRMS (ESI): Exact mass calcd for $C_{17}H_{16}{}^{35}ClN_3NaO$ [M+Na]$^+$: 336.0874, Found: 336.0869.

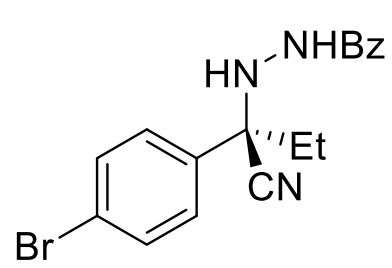
(*R*)-**3ag**

(*R*)**-3ag** was obtained in 81% yield (57.8 mg) as white solid. HPLC analysis (Chiralpak IA, Hexane/^{i}PrOH = 90/10, 1.0 mL/min, 230 nm; t_r (major) = 37.00 min, t_r (minor) = 33.87 min) gave the product: 94% ee, $[\alpha]^{20}_D$ = +133.5 (c = 1.0, $CHCl_3$), m.p. 238-240 °C; ^1H NMR (400 MHz, $CDCl_3$): δ 7.68-7.66 (m, 2H), 7.60-7.50 (m, 5H), 7.43-7.39 (m, 2H), 7.28 (s, 1H), 5.87 (d, *J* = 7.0 Hz, 1H), 2.25-2.16 (m, 1H), 2.06-1.97 (m, 1H), 1.01 (t, *J* = 7.4 Hz, 3H); ^{13}C NMR (100 MHz, $CDCl_3$): δ 167.5,135.7, 132.2, 132.0, 131.7, 128.6, 128.3, 127.1, 123.2, 119.2, 68.0, 33.2, 8.7. IR (neat): 3005, 2970, 2343, 1717, 1437, 1364, 1223, 1094, 530 cm^{-1}; HRMS (ESI): Exact mass calcd for $C_{17}H_{16}{}^{79}BrN_3NaO$ [M+Na]$^+$: 380.0369, Found: 380.0367.

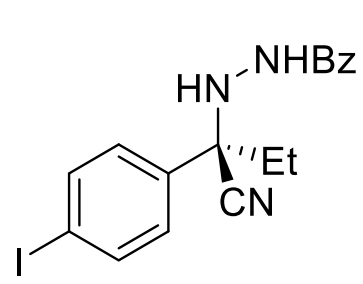
(*R*)-**3ah**

(*R*)-**3ah** was obtained in 98% yield (79.3 mg) as white solid. HPLC analysis (Chiralpak IA, Hexane/^{i}PrOH = 90/10, 1.0 mL/min, 230 nm; t_r (major) = 38.93 min, t_r (minor) = 35.63 min) gave the product: 90% ee, $[\alpha]^{20}_D$ = +153.9 (c = 1.0, $CHCl_3$), m.p. 180-182 °C; ^1H NMR (400 MHz, $CDCl_3$): δ 7.79 (d, *J* = 8.2 Hz, 2H), 7.67 (d, *J* = 7.3 Hz, 2H), 7.52 (t, *J* = 8.4 Hz, 1H), 7.43-7.39 (m, 4H), 7.29 (d, *J* = 7.1 Hz, 1H), 5.87 (d, *J* = 7.2 Hz, 1H), 2.25-2.16 (m, 1H), 2.05-1.96 (m, 1H), 1.01 (t, *J* = 7.4 Hz, 3H); ^{13}C NMR (100 MHz, $CDCl_3$): δ 167.5, 137.9, 136.4, 132.2,

131.6, 128.6, 128.4, 127.1, 119.1, 95.0, 68.0, 33.1, 8.7. IR (neat): 3005, 2970, 2361, 1717, 1437, 1364, 1094 cm^{-1}; HRMS (ESI): Exact mass calcd for $C_{17}H_{16}IN_3NaO$ [M+Na]$^+$: 428.0230, Found: 428.0224.

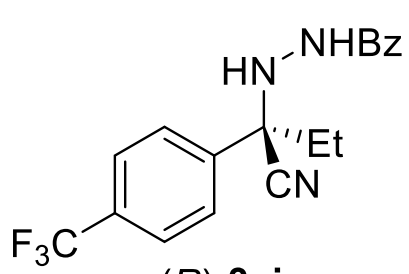

(*R*)-3ai

(*R*)-**3ai** was obtained in 95% yield (65.9 mg) as white solid. HPLC analysis (Chiralpak IA, Hexane/^{i}PrOH = 90/10, 1.0 mL/min, 230 nm; t_r (major) = 28.68 min, t_r (minor) = 26.83 min) gave the product: 92% ee, $[\alpha]^{20}_D$ = +91.3 (c = 1.0, CHCl$_3$), m.p. 187-189 ºC; ^{1}H NMR (400 MHz, CDCl$_3$): δ 7.83 (d, *J* = 7.5 Hz, 1H), 7.77 (d, *J* = 8.0 Hz, 2H), 7.66 (t, *J* = 9.2 Hz, 4H), 7.48 (t, *J* = 7.4 Hz, 1H), 7.36 (t, *J* = 7.6 Hz, 2H), 5.90 (d, *J* = 6.7 Hz, 1H), 2.25-2.16 (m, 1H), 2.05-1.96 (m, 1H), 0.97 (t, *J* = 7.4 Hz, 3H); ^{13}C NMR (100 MHz, CDCl$_3$): δ 167.7, 140.7, 132.3, 131.6, 131.3 (q, *J* = 32.7 Hz), 128.6, 127.14, 127.10, 125.9 (q, *J* = 3.7 Hz), 123.7 (q, *J* = 272.3 Hz), 119.0, 68.2, 33.4, 8.7; ^{19}F NMR (376 MHz, CDCl$_3$): δ -62.70. IR (neat): 3005, 2970, 2342, 1717, 1437, 1364, 1223, 1094, 530 cm^{-1}; HRMS (ESI): Exact mass calcd for $C_{18}H_{16}F_3N_3NaO$ [M+Na]$^+$: 370.1138, Found: 370.1136.

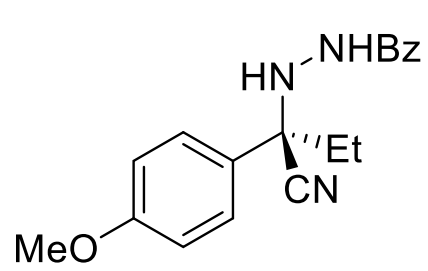

(*R*)-3aj

(*R*)-**3aj** was obtained in 83% yield (51.3 mg) as white solid. HPLC analysis (Chiralpak IA, Hexane/^{i}PrOH = 80/20, 1.0 mL/min, 230 nm; t_r (major) = 17.77 min, t_r (minor) = 15.44 min) gave the product: 86% ee, $[\alpha]^{20}_D$ = +79.4 (c = 1.0, CHCl$_3$), m.p. 185-186ºC; ^{1}H NMR (400 MHz, CDCl$_3$): δ 7.67 (d, *J* = 7.2 Hz, 2H), 7.55 (d, *J* = 8.4 Hz, 3H), 7.48 (t, *J* = 7.4 Hz, 1H), 7.37 (t, *J* = 7.6 Hz, 2H), 6.94 (d, *J* = 8.4 Hz, 2H), 5.83 (d, *J* = 7.3 Hz, 1H), 3.82 (s, 3H), 2.23-2.14 (m, 1H), 2.05-1.96 (m, 1H), 0.97 (t, *J* = 7.4 Hz, 3H); ^{13}C NMR (100 MHz, CDCl$_3$): δ 167.2, 160.0, 132.1, 132.0, 128.6, 128.2, 127.8, 127.1, 119.7, 114.2, 67.9, 55.3, 33.1, 8.8. IR (neat): 3004, 2970, 2360, 2341, 1717, 1437, 1364, 1223, 1094, 529 cm^{-1};HRMS (ESI): Exact mass calcd for $C_{18}H_{19}N_3NaO_2$ [M+Na]$^+$: 332.1369, Found: 332.1370.

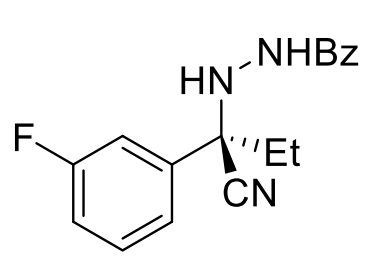

(*R*)-3ak

(*R*)-**3ak** was obtained in 99% (58.8 mg) yield as white solid. HPLC analysis (Chiralpak IE, Hexane/^{i}PrOH = 80/20, 1.0 mL/min, 230 nm; t_r (major) = 16.39 min, t_r (minor) = 14.79 min) gave the product: 90% ee, $[\alpha]^{20}_D$ = +125.0 (c = 1.0, CHCl$_3$), m.p. 165-167 ºC; ^{1}H NMR (400 MHz, CDCl$_3$): δ 7.69-7.66 (m, 2H), 7.51-7.37 (m, 7H), 7.13-7.08 (m, 1H), 5.91 (d, *J*

= 7.2 Hz, 1H), 2.25-2.17 (m, 1H), 2.06-1.97 (m, 1H), 1.02 (t, *J* = 7.4 Hz, 3H); ^{13}C NMR (100 MHz, $CDCl_3$): δ 167.6, 162.9 (d, *J* = 247.3 Hz), 139.2 (d, *J* = 7.2 Hz), 132.2, 131.7, 130.4 (d, *J* = 8.2 Hz), 128.6, 127.1, 122.4 (d, *J* = 2.9 Hz), 119.2, 116.1 (d, *J* = 21.1 Hz), 113.7 (d, *J* = 23.7 Hz), 68.0 (d, *J* = 2.1 Hz), 33.3, 8.7; ^{19}F NMR (376 MHz, $CDCl_3$): δ -111.18. IR (neat): 3005, 2970, 2344, 1717, 1437, 1364, 1223, 1092, 529 cm^{-1}; HRMS (ESI): Exact mass calcd for $C_{17}H_{16}FN_3NaO$ $[M+Na]^+$: 320.1170, Found: 320.1163.

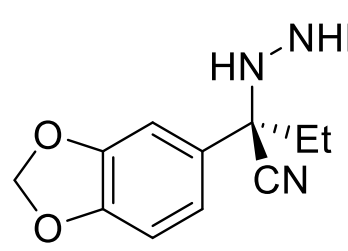

(*R*)-**3al**

(*R*)**-3al** was obtained in 96% yield (62.0 mg) as white solid. HPLC analysis (Chiralpak IA, Hexane/^{i}PrOH = 80/20, 1.0 mL/min, 230 nm; t_r (major) = 21.07 min, t_r (minor) = 18.35 min) gave the product: 90% ee, $[\alpha]^{20}_D$ = +77.3 (c = 1.0, $CHCl_3$), m.p. 190-192 ºC; ^{1}H NMR (400 MHz, $CDCl_3$): δ 7.69-7.67 (m, 2H), 7.50-7.47 (m, 2H), 7.41-7.38 (m, 2H), 7.15 (dd, *J* = 8.1, 1.9 Hz, 1H), 7.11 (d, *J* = 2.0 Hz, 1H), 6.83 (d, *J* = 2.0 Hz, 1H), 6.00 (s, 2H), 5.86 (d, *J* = 7.3 Hz, 1H), 2.20-2.13 (m, 1H), 2.02-1.95 (m, 1H), 0.99 (t, *J* = 7.4 Hz, 3H); ^{13}C NMR (100 MHz, $CDCl_3$): δ 167.2, 148.4, 148.2, 132.2, 131.9, 130.2, 128.7, 127.1, 120.6, 119.6, 108.3, 106.6, 101.6, 68.1, 33.3, 8.8. IR (neat): 3003, 2970, 2344, 1717, 1437, 1364, 1240, 1223, 530 cm^{-1}; HRMS (ESI): Exact mass calcd for $C_{18}H_{17}N_3NaO_3$ $[M+Na]^+$: 346.1162, Found: 346.1152.

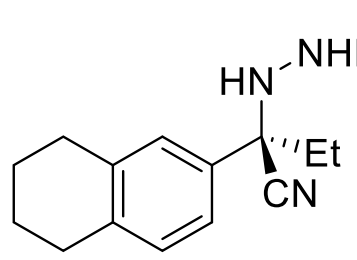

(*R*)-**3am**

(*R*)**-3am** was obtained in 92% yield (61.2 mg) as white solid. HPLC analysis (Chiralpak IE, Hexane/^{i}PrOH = 85/15, 1.0 mL/min, 230 nm; t_r (major) = 27.22 min, t_r (minor) = 31.44 min) gave the product: 87% ee, $[\alpha]^{20}_D$ = +66.7 (c = 1.0, $CHCl_3$), m.p. 181-182 ºC; ^{1}H NMR (400 MHz, $CDCl_3$): δ 7.75-7.68 (m, 3H), 7.47 (t, *J* = 7.4 Hz, 1H), 7.39-7.32 (m, 4H), 7.10 (d, *J* = 8.5 Hz, 1H), 5.85 (d, *J* = 7.2 Hz, 1H), 2.77 (d, *J* = 4.8 Hz, 4H), 2.22-2.13 (m, 1H), 2.05-1.96 (m, 1H), 1.80 (t, *J* = 3.4 Hz, 4H), 0.98 (t, *J* = 7.4 Hz, 3H); ^{13}C NMR (100 MHz, $CDCl_3$): δ 167.1, 138.1, 137.7, 133.2, 132.0, 131.9, 129.6, 128.5, 127.1, 127.0, 123.2, 119.7, 68.1, 33.0, 29.4, 29.0, 22.9, 22.8, 8.8. IR (neat): 3273, 2931, 1641, 1452, 1305, 1138, 754, 692 cm^{-1}; HRMS (ESI): Exact mass calcd for $C_{21}H_{23}N_3NaO$ $[M+Na]^+$: 356.1733, Found: 356.1729.

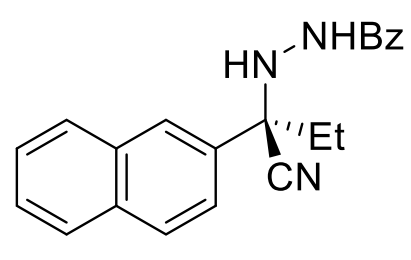

(*R*)-3an

(*R*)**-3an** was obtained in 89% yield (58.5 mg) as white solid. HPLC analysis (Chiralpak IE, Hexane/^{i}PrOH = 80/20, 1.0 mL/min, 230 nm; t_r (major) = 26.72 min, t_r (minor) = 23.93 min) gave the product: 86% ee, $[\alpha]^{20}_D$ = +146.3 (c = 1.0, $CHCl_3$), m.p. 190-192 ºC; ^{1}H NMR (400 MHz, $CDCl_3$): δ 8.17 (d, *J* = 2.0 Hz, 1H), 7.91-7.85 (m, 4H), 7.71-7.69 (m, 1H), 7.66-7.63 (m, 2H), 7.56-7.53 (m, 2H), 7.45-7.41 (m, 1H), 7.33-7.29 (m, 2H), 5.96 (d, *J* = 7.2 Hz, 1H), 2.30-2.21 (m, 1H), 2.15-2.08 (m, 1H), 0.98 (t, *J* = 7.4 Hz, 3H); ^{13}C NMR (100 MHz, $CDCl_3$): δ 167.3, 133.7, 133.3, 132.8, 132.0, 131.8, 129.0, 128.5, 128.3, 127.6, 127.1, 126.8, 126.7, 126.6, 123.0 119.6, 68.5, 32.9, 8.8. IR (neat): 3005, 2970, 2342, 1717, 1437, 1364, 1223, 1094, 530 cm^{-1}; HRMS (ESI): Exact mass calcd for $C_{21}H_{19}N_3NaO$ $[M+Na]^+$: 352.1420, Found: 352.1416.

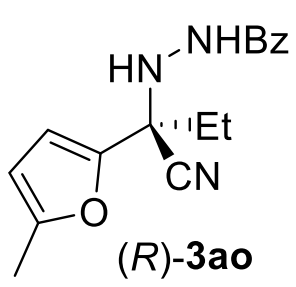

(*R*)-3ao

(*R*)**-3ao** was obtained in 45% yield (27.5 mg) as white solid. HPLC analysis (Chiralpak AD-H, Hexane/^{i}PrOH = 70/30, 1.0 mL/min, 254 nm; t_r (major) = 7.36 min, t_r (minor) = 6.44 min) gave the isomeric composition of the product: 83% ee, $[\alpha]^{20}_D$ = +40.9 (c = 0.5, $CHCl_3$), m.p. 166-168 ºC; ^{1}H NMR (500 MHz, $CDCl_3$): δ 7.73 (d, *J* = 7.5 Hz, 2H), 7.54-7.42 (m, 4H), 6.48 (d, *J* = 3.0 Hz, 1H), 5.96 (d, *J* = 2.0 Hz, 1H), 5.76 (d, *J* = 7.0 Hz, 1H), 2.31 (s, 3H), 2.27-2.17 (m, 2H), 1.09 (t, *J* = 7.0 Hz, 3H); ^{13}C NMR (125 MHz, $CDCl_3$): δ 167.4, 153.9, 146.2, 132.3, 132.0, 128.7, 127.1, 118.3, 111.4, 106.6, 63.6, 29.4, 13.7, 9.0 . IR (neat): 3260, 1636, 1489, 1423, 1267, 897, 743, 706 cm^{-1}; HRMS (ESI): Exact mass calcd for $C_{16}H_{17}N_3O_2Na$ $[M+Na]^+$: 306.1213, Found: 306.1206.

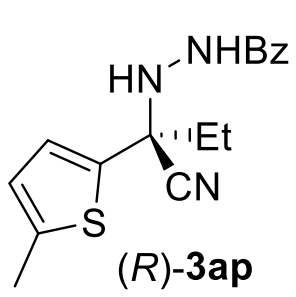

(*R*)-3ap

(*R*)**-3ap** was obtained in 42% yield (25.0 mg) as white solid. HPLC analysis (Chiralpak AD-H, Hexane/^{i}PrOH = 80/20, 1.0 mL/min, 230 nm; t_r (major) = 23.10 min, t_r (minor) = 20.05 min) gave the isomeric composition of the product: 81% ee, $[\alpha]^{20}_D$ = +43.7 (c = 0.5, $CHCl_3$), m.p. 180-181 ºC; ^{1}H NMR (500 MHz, $CDCl_3$): δ 7.72 (d, *J* = 7.0 Hz, 2H), 7.53-7.50 (m, 2H), 7.43-7.40 (m, 2H), 7.10 (d, *J* = 3.5 Hz, 1H), 6.66 (d, *J* = 2.5 Hz, 1H), 5.96 (d, *J* = 7.0 Hz, 1H), 2.49 (s, 3H), 2.26-2.21 (m, 1H), 2.05-2.00 (m, 1H), 1.09 (t, *J* = 7.5 Hz, 3H); ^{13}C NMR (125 MHz, $CDCl_3$): δ 167.2, 141.7, 137.8, 132.3, 131.8, 128.7, 127.7, 127.1, 124.9, 118.7, 65.5, 33.8, 15.5, 9.0. IR (neat): 1636, 1473, 1267, 999, 910, 746, 706 cm^{-1}; HRMS (ESI): Exact mass calcd for $C_{16}H_{17}N_3OS$ $[M]^+$: 299.1092, Found: 299.1085.

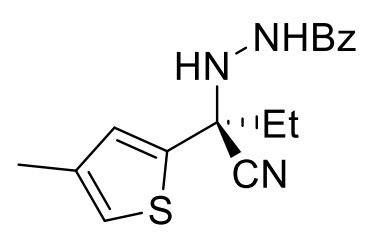
(*R*)-3aq

(*R*)**-3aq** was obtained in 56% yield (33.4 mg) as white solid. HPLC analysis (Chiralpak AD-H, Hexane/^{i}PrOH = 80/20, 1.0 mL/min, 254 nm; t_r (major) = 14.50 min, t_r (minor) = 13.15 min) gave the isomeric composition of the product: 81% ee, $[\alpha]^{20}_D$ = +42.2 (c = 0.5, $CHCl_3$), m.p. 183-185 ºC; ^{1}H NMR (500 MHz, $CDCl_3$): δ 7.73-7.71 (m, 2H), 7.58 (d, *J* = 6.5 Hz, 1H), 7.53-7.50 (m, 1H), 7.43-7.40 (m, 2H), 7.12 (d, *J* = 1.5 Hz, 1H), 6.95 (s, 1H), 5.97 (d, *J* = 7.5 Hz, 1H), 2.28-2.21 (m, 1H), 2.25 (s, 3H), 2.06-2.00 (m, 1H), 1.09 (t, *J* = 7.5 Hz, 3H); ^{13}C NMR (125 MHz, $CDCl_3$): δ 167.3, 140.4, 137.6, 132.3, 131.8, 129.9, 128.7, 127.1, 122.0, 118.8, 65.4, 33.9, 15.8, 9.0. IR (neat): 3270, 1636, 1474, 1267, 910, 740, 708 cm^{-1}; HRMS (ESI): Exact mass calcd for $C_{16}H_{17}N_3OS$ $[M]^+$: 299.1092, Found: 299.1100.

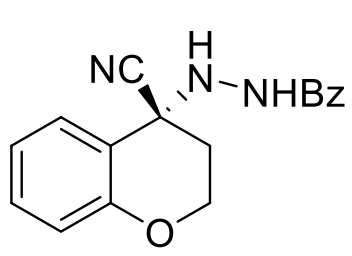
(*R*)-3ar

(*R*)**-3ar** was obtained in 67% yield (39.2 mg) as white solid. HPLC analysis (Chiralpak IA, Hexane/^{i}PrOH = 80/20, 1.0 mL/min, 230 nm; t_r (major) = 12.26 min, t_r (minor) = 10.42 min) gave the product: 77% ee, $[\alpha]^{20}_D$ = +88.8 (c = 1.0, $CHCl_3$), m.p. 216-218 ºC; ^{1}H NMR (400 MHz, $CDCl_3$): δ 7.94 (t, *J* = 4.0 Hz, 1H), 7.83-7.81 (m, 2H), 7.68 (dt, *J* = 7.8, 1.4 Hz, 1H), 7.55-7.53 (m, 1H), 7.45 (td, *J* = 7.7, 1.6 Hz, 2H), 7.33-7.29 (m,1H), 7.01 (td, *J* = 7.5, 1.3 Hz, 1H), 6.90 (dd, *J* = 8.3, 1.2 Hz, 1H), 5.40 (d, *J* = 6.2 Hz, 1H), 4.49-4.43 (m, 1H), 4.31-4.26 (m, 1H), 2.45-2.34 (m, 2H); ^{13}C NMR (100 MHz, $CDCl_3$): δ 168.5, 154.4, 132.6, 131.6, 131.5, 129.4, 128.8, 127.2, 121.3, 120.4, 117.8, 116.7, 61.2, 56.7, 30.8. IR (neat): 3005, 2970, 2361, 1717, 1437, 1364, 1223, 1094, 530 cm^{-1}; HRMS (ESI): Exact mass calcd for $C_{17}H_{15}N_3NaO_2$ $[M+Na]^+$: 316.1056, Found: 316.1052.

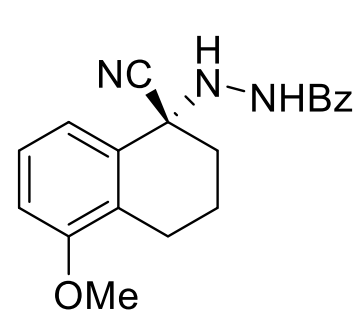
(*R*)-3as

(*R*)**-3as** was obtained in 87% yield (55.8 mg) as white solid. HPLC analysis (Chiralpak IA, Hexane/^{i}PrOH = 80/20, 1.0 mL/min, 230 nm; t_r (major) = 29.33 min, t_r (minor) = 10.22 min) gave the product: 90% ee, $[\alpha]^{20}_D$ = +16.8 (c = 1.0, $CHCl_3$), m.p. 181-183 ºC; ^{1}H NMR (400 MHz, $CDCl_3$): δ 7.83 (d, *J* = 7.7 Hz, 2H), 7.70 (d, *J* = 6.9 Hz, 1H), 7.56 (t, *J* = 7.4 Hz, 1H), 7.47 (t, *J* = 7.7 Hz, 3H), 7.29 (t, *J* = 8.1 Hz, 1H), 6.87 (d, *J* = 8.1 Hz, 1H), 5.43 (d, *J* = 6.8 Hz, 1H), 3.84 (s, 3H), 2.86 (dt, *J* = 17.3, 4.8 Hz, 1H), 2.61-2.53 (m, 1H), 2.39-2.34 (m, 1H), 2.20-2.08 (m, 2H), 1.92-1.88 (m, 1H); ^{13}C NMR (100 MHz, $CDCl_3$): δ 168.1, 157.2, 132.3, 132.0, 131.9, 128.8, 127.19, 127.18, 126.8, 121.5, 120.6, 110.5, 60.5, 55.5, 31.9, 22.5, 17.1. IR (neat): 3310, 1543,

1535, 1508, 1263, 1066, 1028, 771 cm^{-1}; HRMS (ESI): Exact mass calcd for $C_{19}H_{19}N_3NaO_2$ [M+Na]$^+$: 344.1369, Found: 344.1363.

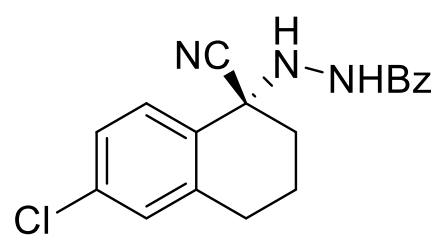
(*R*)-**3at**

(*R*)**-3at** was obtained in 62% yield (41.5 mg) as white solid. HPLC analysis (Chiralpak IA, Hexane/^{i}PrOH = 80/20, 1.0 mL/min, 230 nm; t_r (major) = 11.83 min, t_r (minor) = 9.89 min) gave the product: 87% ee, $[\alpha]^{20}_D$ = +27.4 (c = 1.0, $CHCl_3$), m.p. 189-191 ºC; ^{1}H NMR (400 MHz, $CDCl_3$): δ 7.82-7.79 (m, 2H), 7.75 (d, *J* = 8.4 Hz, 1H), 7.57-7.53 (m, 1H), 7.46 (t, *J* = 7.6 Hz, 2H), 7.27-7.24 (m, 1H), 7.17 (d, *J* = 2.2 Hz, 1H), 5.38 (d, *J* = 6.7 Hz, 1H), 2.86-2.76 (m, 2H), 2.41-2.34 (m, 1H), 2.18-2.13 (m, 2H), 1.89-1.84 (m, 1H); ^{13}C NMR (100 MHz, $CDCl_3$): δ 168.3, 139.4, 135.4, 132.5, 131.7, 130.5, 129.6, 129.5, 128.8, 127.2, 127.1, 121.1, 60.0, 32.0, 28.7, 17.6. IR (neat): 3308, 1543, 1535, 1525, 1489, 1216, 1076, 773 cm^{-1}; HRMS (ESI): Exact mass calcd for $C_{18}H_{16}{}^{35}ClN_3NaO$ [M+Na]$^+$: 348.0874, Found: 348.0866.

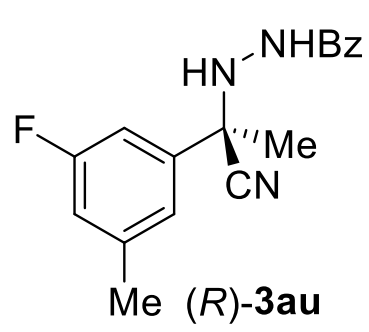
(*R*)-**3au**

(*R*)**-3au** was obtained in 74% yield (43.9 mg) as white solid. HPLC analysis (Chiralpak IE, Hexane/^{i}PrOH = 60/40, 1.0 mL/min, 230 nm; t_r (major) = 14.26 min, t_r (minor) = 7.46 min) gave the product: 89% ee, $[\alpha]^{20}_D$ = +121.2 (c = 1.0, $CHCl_3$), m.p. 184-186 ºC; ^{1}H NMR (400 MHz, $CDCl_3$): δ 7.72-7.69 (m, 3H), 7.50-7.48 (m, 1H), 7.41-7.37 (m, 2H), 7.29 (s, 1H), 7.20 (dt, *J* = 9.2, 2.1 Hz, 1H), 6.90 (dt, *J* = 9.3, 1.8 Hz, 1H), 5.79 (d, *J* = 7.1 Hz, 1H), 2.38 (s, 3H) , 1.83 (s, 3H); ^{13}C NMR (100 MHz, $CDCl_3$): δ 167.6, 162.9 (d, *J* = 246.7 Hz), 141.5 (d, *J* = 8.0 Hz), 140.2 (d, *J* = 7.8 Hz), 132.3, 131.8, 128.6, 127.2, 122.4 (d, *J* = 2.7 Hz), 120.2, 116.8 (d, *J* = 20.9 Hz), 110.1 (d, *J* = 23.9 Hz), 62.3 (d, *J* = 2.3 Hz), 27.4, 21.4 (d, *J* = 1.8 Hz); ^{19}F NMR (376 MHz, $CDCl_3$): δ -112.15. IR (neat): 3003, 2360, 2341, 1717, 1437, 1364, 1223, 1093, 799, 698, 530 cm^{-1}; HRMS (ESI): Exact mass calcd for $C_{17}H_{16}FN_3NaO$ [M+Na]$^+$: 320.1170, Found: 320.1169.

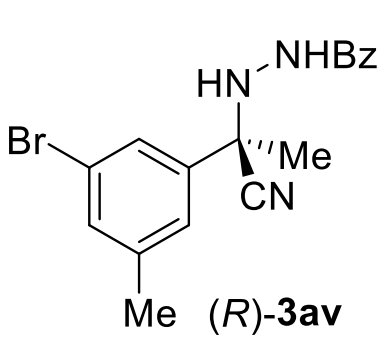
(*R*)-**3av**

(*R*)**-3av** was obtained in 64% yield (45.6 mg) as white solid. HPLC analysis (Chiralpak IE, Hexane/^{i}PrOH = 80/20, 1.0 mL/min, 230 nm; t_r (major) = 18.21 min, t_r (minor) = 14.70 min) gave the product: 90% ee, $[\alpha]^{20}_D$ = +89.7 (c = 1.0, $CHCl_3$), m.p. 202-204 ºC; ^{1}H NMR (400 MHz, $CDCl_3$): 7.76 (d, *J* = 6.3 Hz, 1H), δ 7.72-7.69 (m, 2H), 7.63 (s, 1H), 7.50-7.47

(m, 1H), 7.43-7.34 (m, 4H), 5.78 (d, J = 6.4 Hz, 1H), 2.36 (s, 3H), 1.82 (s, 3H); ^{13}C NMR (100 MHz, $CDCl_3$): δ 167.7, 141.0, 140.0, 132.9, 132.3, 131.7, 128.6, 127.2, 126.0, 125.5, 122.9, 120.0, 62.2, 27.4, 21.2. IR (neat): 3005, 2360, 2144, 1713, 1437, 1362, 1221, 1092, 903, 765, 529 cm^{-1}; HRMS (ESI): Exact mass calcd for $C_{17}H_{16}{}^{79}BrN_3NaO$ [M+Na]$^+$: 380.0369, Found: 380.0368.

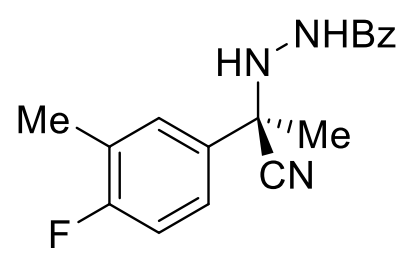

(*R*)-**3aw**

(*R*)**-3aw** was obtained in 65% yield (38.6 mg) as white solid. HPLC analysis (Chiralpak IE, Hexane/^{i}PrOH = 80/20, 1.0 mL/min, 230 nm; t_r (major) = 15.68 min, t_r (minor) = 14.57 min) gave the product: 91% ee, $[\alpha]^{20}_D$ = +169.0 (c = 1.0, $CHCl_3$), m.p. 188-190 ºC; ^{1}H NMR (400 MHz, $CDCl_3$): δ 7.71-7.69 (m, 2H), 7.57 (s, 1H), 7.53-7.46 (m, 3H), 7.43-7.39 (m, 2H), 7.05 (t, J = 8.8 Hz, 1H), 5.73 (d, J = 7.1 Hz, 1H), 2.31 (s, 3H), 1.84 (s, 3H); ^{13}C NMR (100 MHz, $CDCl_3$): δ 167.5, 161.5 (d, J = 247.5 Hz), 133.3 (d, J = 3.6 Hz), 132.3, 131.8, 129.2 (d, J = 5.5 Hz), 128.7, 127.1, 125.8 (d, J = 17.7 Hz), 125.0 (d, J = 8.4 Hz), 120.44, 115.6 (d, J = 22.8 Hz), 62.1, 27.3, 14.6 (d, J = 3.4 Hz); ^{19}F NMR (376 MHz, $CDCl_3$): δ -116.49. IR (neat): 3003, 2964, 2343, 1717, 1437, 1364, 1223, 1093, 800, 698, 530 cm^{-1}; HRMS (ESI): Exact mass calcd for $C_{17}H_{16}FN_3NaO$ [M+Na]$^+$: 320.1170, Found: 320.1165.

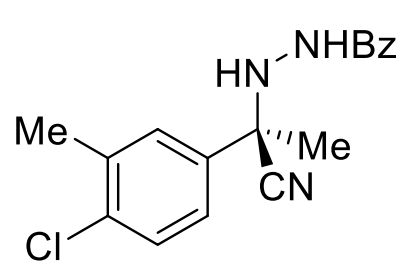

(*R*)-**3ax**

(*R*)**-3ax** was obtained in 70% yield (43.8 mg) as white solid. HPLC analysis (Chiralpak IE, Hexane/^{i}PrOH = 85/15, 1.0 mL/min, 230 nm; t_r (major) = 23.06 min, t_r (minor) = 21.54 min) gave the product: 90% ee, $[\alpha]^{20}_D$ = +59.0 (c = 1.0, $CHCl_3$), m.p. 197-199 ºC; ^{1}H NMR (400 MHz, $CDCl_3$): δ 7.71-7.68 (m, 3H), 7.55 (s, 1H), 7.52-7.47 (m, 1H), 7.46-7.43 (m, 1H), 7.41-7.37 (m, 3H), 5.78 (d, J = 7.0 Hz, 1H), 2.40 (s, 3H) , 1.83 (s, 3H); ^{13}C NMR (100 MHz, $CDCl_3$): δ 167.6, 137.0, 136.4, 135.3, 132.3, 131.7, 129.6, 128.6, 128.5, 127.2, 124.6, 120.3, 62.2, 27.3, 20.2. IR (neat): 3003, 2359, 2341, 1717, 1437, 1364, 1223, 1093, 800, 719, 530 cm^{-1}; HRMS (ESI): Exact mass calcd for $C_{17}H_{16}{}^{35}ClN_3NaO$ [M+Na]$^+$: 336.0874, Found: 336.0873.

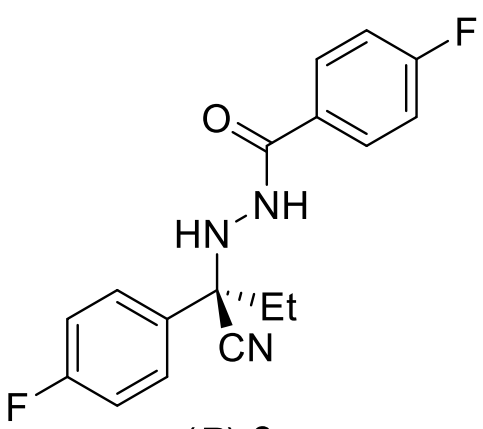

(*R*)-3ay

(*R*)**-3ay** was obtained in 91% yield (57.3 mg) as white solid. HPLC analysis (Chiralpak IA, Hexane/^{i}PrOH = 85/15, 1.0 mL/min, 230 nm; t_r (major) = 14.36 min, t_r (minor) = 10.91 min) gave the isomeric composition of the product: 91% ee, $[\alpha]^{20}_D$ = +88.9 (c = 1.0, $CHCl_3$), m.p. 197-199 ºC; ^{1}H NMR (400 MHz, $CDCl_3$): δ 7.68-7.65 (m, 3H), 7.63-7.59 (m, 2H), 7.11 (t, *J* = 8.6 Hz, 2H), 7.04 (t, *J* = 8.6 Hz, 2H), 5.83 (d, *J* = 6.9 Hz, 1H), 2.23-2.14 (m, 1H), 2.03-1.94 (m, 1H), 0.96 (t, *J* = 7.4 Hz, 3H); ^{13}C NMR (100 MHz, $CDCl_3$): δ 166.5, 165.1 (d, *J* = 251.8 Hz), 162.9 (d, *J* = 247.3 Hz), 132.2 (d, *J* = 3.3 Hz), 129.6 (d, *J* = 9.1 Hz), 128.4 (d, *J* = 8.3 Hz), 127.9 (d, *J* = 3.2 Hz), 119.4, 115.9 (d, *J* = 21.6 Hz), 115.8 (d, *J* = 21.9 Hz), 67.9, 33.3, 8.7; ^{19}F NMR (376 MHz, $CDCl_3$): δ -106.40, -112.27. IR (neat): 3270, 1650, 1508, 1220, 1066, 1051, 772 cm^{-1}; HRMS (ESI): Exact mass calcd for $C_{17}H_{15}F_2N_3NaO$ $[M+Na]^+$: 338.1075, Found: 338.1071.

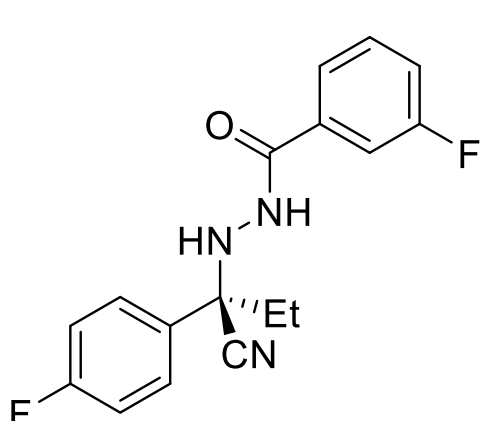

(*R*)-3az

(*R*)**-3az** was obtained in 88% yield (55.4 mg) as white solid. HPLC analysis (Chiralpak IA, Hexane/^{i}PrOH = 85/15, 1.0 mL/min, 230 nm; t_r (major) = 15.89 min, t_r (minor) = 14.50 min) gave the isomeric composition of the product: 90% ee, $[\alpha]^{20}_D$ = +48.0 (c = 1.0, $CHCl_3$), m.p. 198-199 ºC; ^{1}H NMR (400 MHz, $CDCl_3$): δ 7.64-7.61 (m, 3H), 7.42-7.35 (m, 3H), 7.19 (td, *J* = 8.3, 2.5 Hz, 1H), 7.12 (t, *J* = 8.4 Hz, 2H), 5.82 (s, br, 1H), 2.23-2.16 (m, 1H), 2.04-1.97 (m, 1H), 0.97 (t, *J* = 7.4 Hz, 3H); ^{13}C NMR (100 MHz, $CDCl_3$): δ 166.2, 163.0 (d, *J* = 247.4 Hz), 162.6 (d, *J* = 246.9 Hz), 134.0 (d, *J* = 7.2 Hz), 132.1, 130.4 (d, *J* = 7.9 Hz), 128.4 (d, *J* = 8.3 Hz), 122.6 (d, *J* = 3.2 Hz), 119.4 (d, *J* = 5.1 Hz), 119.2, 116.0 (d, *J* = 21.7 Hz), 114.6 (d, *J* = 23.1 Hz), 67.9, 33.3, 8.8; ^{19}F NMR (376 MHz, $CDCl_3$): δ -111.23, -112.19. IR (neat): 3275, 1656, 1649, 1508, 1454, 1226, 752cm^{-1}; HRMS (ESI): Exact mass calcd for $C_{17}H_{15}F_2N_3NaO$ $[M+Na]^+$: 338.1075, Found: 338.1076.

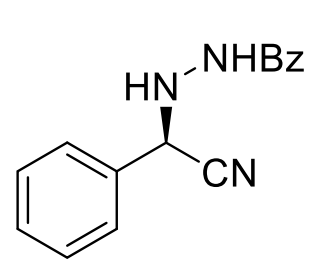

(*R*)-4a

(*R*)**-4a**[4] was obtained in 70% yield (35.1 mg) as white solid. HPLC analysis (Chiralcel OD-3, Hexane/^{i}PrOH = 90/10, 1.0 mL/min, 230 nm; t_r (major) = 17.93 min, t_r (minor) = 22.52 min) gave the product: 92% ee, $[\alpha]^{20}_D$ = +50.6 (c = 1.0, $CHCl_3$); ^{1}H NMR (400 MHz, $CDCl_3$): δ 8.03 (d, *J* = 5.9 Hz,1H), 7.80-7.77 (m, 2H), 7.62-7.54 (m, 3H), 7.48-7.44 (m, 5H), 5.21

(d, J = 3.2 Hz,1H), 5.17-5.15 (m, 1H); ^{13}C NMR (100 MHz, $CDCl_3$): δ 168.2, 132.5, 131.9, 131.7, 130.0, 129.3, 128.9, 128.8, 127.1, 118.3, 56.6.

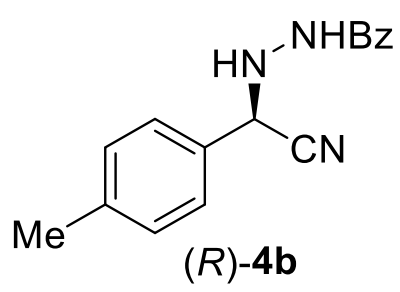

(R)-**4b**

(R)**-4b**[4] was obtained in 92% yield (48.7 mg) as white solid. HPLC analysis (Chiralcel OD-H, Hexane/^{i}PrOH = 80/20, 1.0 mL/min, 230 nm; t_r (major) = 9.49 min, t_r (minor) = 11.98 min) gave the product: 91% ee, $[\alpha]^{20}_D$ = +70.9 (c = 1.0, $CHCl_3$); ^{1}H NMR (400 MHz, $CDCl_3$): δ 7.86 (s, br, 1H), 7.79-7.77 (m, 2H), 7.59-7.55 (m, 1H), 7.51-7.45 (m, 4H), 7.27-7.25 (m, 2H), 5.18 (d, J = 3.1 Hz, 1H), 5.09 (dd, J = 6.0, 3.2 Hz, 1H), 2.39 (s, 3H); ^{13}C NMR (100 MHz, $CDCl_3$): δ 168.2, 140.1, 132.5, 131.7, 129.9, 128.9, 128.8, 128.3, 127.1, 118.5, 56.4, 21.2.

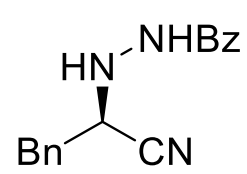

(R)-**4c**

(R)**-4c**[4] was obtained in 90% yield (47.7 mg) as white solid. HPLC analysis (Chiralcel OD-H, Hexane/^{i}PrOH = 90/10, 1.0 mL/min, 230 nm; t_r (major) = 21.15 min, t_r (minor) = 33.21 min) gave the product: 86% ee, $[\alpha]^{20}_D$ = +63.6 (c = 1.0, $CHCl_3$); ^{1}H NMR (400 MHz, $CDCl_3$): δ 7.97 (s, 1H), 7.74 (d, J = 7.3 Hz, 2H), 7.54 (t, J = 7.3 Hz, 1H), 7.43 (t, J = 7.6 Hz, 2H), 7.37-7.32 (m, 5H), 5.09 (s, 1H), 4.34 (t, J = 7.2 Hz, 1H), 3.14 (dd, J = 7.2, 2.7 Hz, 2H); ^{13}C NMR (100 MHz, $CDCl_3$): δ 168.1, 134.3, 132.4, 131.7, 129.2, 128.9, 128.8, 127.8, 127.1, 118.8, 54.1, 37.5.

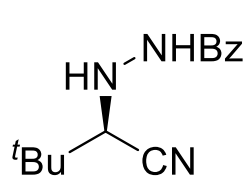

(R)-**4d**

(R)**-4d**[4] was obtained in 91% yield (42.0 mg) as white solid. HPLC analysis (Chiralcel OD-H, Hexane/^{i}PrOH = 90/10, 1.0 mL/min, 230 nm; t_r (major) = 8.20 min, t_r (minor) = 12.17 min) gave the product: 90% ee, $[\alpha]^{20}_D$ = +66.1 (c = 1.0, $CHCl_3$); ^{1}H NMR (400 MHz, $CDCl_3$): δ 7.97 (s, 1H), 7.78 (d, J = 7.6 Hz, 2H), 7.55 (t, J = 7.5 Hz, 1H), 7.45 (t, J = 8.0 Hz, 2H), 5.08 (s, 1H), 3.85 (s, 1H), 1.17 (s, 9H); ^{13}C NMR (100 MHz, $CDCl_3$): δ 168.1, 132.3, 131.8, 128.7, 127.1, 118.6, 63.4, 33.9, 26.1.

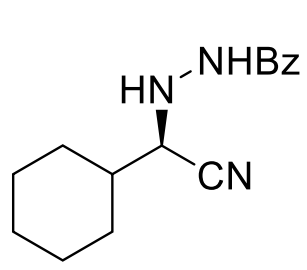

(R)-**4e**

(R)**-4e** was obtained in 92% yield (47.2 mg) as white solid. HPLC analysis (Chiralcel OD-H, Hexane/^{i}PrOH = 90/10, 1.0 mL/min, 230 nm; t_r (major) = 11.17 min, t_r (minor) = 14.94 min) gave the product: 91% ee, $[\alpha]^{20}_D$ = +40.3 (c = 1.0, $CHCl_3$); ^{1}H NMR (400 MHz, $CDCl_3$): δ 8.10 (s, 1H), 7.79-7.77 (m, 2H), 7.54 (t, J = 7.4 Hz, 1H), 7.44 (t, J = 7.6 Hz, 2H), 5.00 (s, br, 1H), 3.92 (d, J = 5.8 Hz, 1H), 2.02-1.99 (m, 1H), 1.87-1.68 (m, 5H), 1.30-1.20 (m, 5H); ^{13}C NMR

(100 MHz, $CDCl_3$): δ 168.1, 132.4, 131.8, 128.7, 127.1, 118.6, 58.9, 38.9, 29.6, 28.6, 25.8, 25.6, 25.4. IR (neat): 3003, 2930, 2358, 1679, 1366, 1223, 1090 cm^{-1}; HRMS (ESI): Exact mass calcd for $C_{15}H_{19}N_3NaO$ $[M+Na]^+$: 280.1420, Found: 280.1427.

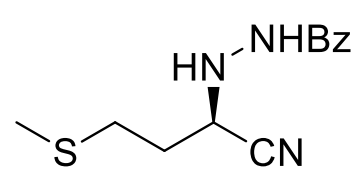

(*R*)-**4f**

(*R*)**-4f** was obtained in 93% yield (46.3 mg) as white solid. HPLC analysis (Chiralcel OD-H, Hexane/^{i}PrOH = 90/10, 1.0 mL/min, 230 nm; t_r (major) = 24.52 min, t_r (minor) = 39.82 min) gave the product: 84% ee, $[\alpha]^{20}_D$ = +52.4 (c = 1.0, $CHCl_3$), m.p. 171-173 ºC; ^{1}H NMR (400 MHz, $CDCl_3$): δ 8.34 (d, *J* = 5.9 Hz, 1H), 7.80-7.77 (m, 2H), 7.55-7.50 (m, 1H), 7.44-7.39 (m, 2H), 5.27 (t, *J* = 5.4 Hz, 1H), 4.27-4.23 (m, 1H), 2.80-2.73 (m, 1H), 2.69-2.62 (m, 1H), 2.13-2.08 (m, 5H); ^{13}C NMR (100 MHz, $CDCl_3$): δ 168.1, 132.4, 131.6, 128.7, 127.1, 118.9, 51.9, 30.3, 29.7, 15.4. IR (neat): 3005, 2970, 2361, 1717, 1437, 1364, 1223, 1094, 530 cm^{-1}; HRMS (ESI): Exact mass calcd for $C_{12}H_{15}N_3NaOS$ $[M+Na]^+$: 272.0828, Found: 272.0834.

6. Total Synthesis of *L*-Carbidopa

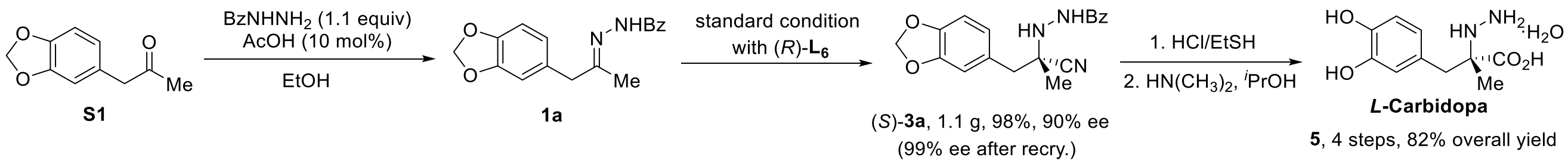

Step I: To a solution of **S1** (5.0 mmol) and benzoyl hydrazine (1.1 equivs) in ethanol (5.0 mL) was slowly added AcOH (10 mol%) at room temperature. The resulting mixture was stirred for 8 h. The precipitate was filtered and recrystallized from ethanol to give **1a** in 92% yield as white solid. m.p. 197-199 °C; ^{1}H NMR (400 MHz, DMSO-d_6): ^{1}H NMR (400 MHz, DMSO-d_6): δ 10.48 (s, 1H), 7.83 (d, J = 7.5 Hz, 2H), 7.55-7.46 (m, 3H), 6.88-6.73 (m, 3H), 5.99 (s, 2H), 3.53 (s, 2H), 1.86 (s, 3H); ^{13}C NMR (100 MHz, DMSO-d_6): δ 163.4, 161.8, 147.4, 146.0, 134.1, 131.4, 130.9, 130.0, 128.3, 127.7, 122.0, 109.2, 108.3, 100.9, 44.2, 16.3. IR (neat): 3005, 2360, 1717, 1440, 1364, 1225, 1094, 532 cm^{-1}; HRMS (ESI): Exact mass calcd for $C_{17}H_{16}N_2NaO_3$ [M+Na]$^+$: 319.1053, Found: 319.1052.

Step II: Under an atmosphere of N_2, to a 100 mL sealed tube were added $ErCl_3$ (47.8 mg, 5.0 mol%) and PYBOX **L_6** (143.1 mg, 7.5 mol%), followed by 50 mL of anhydrous toluene. The solution was stirred at 25 °C for 2 h, and then hydrazone **1a** (1.13 g, 3.5 mmol) was added. After the mixture was continued to stir at 25 °C for 15 min, the mixture was cooled to -40 °C for another 0.5 h. Then, TMSCN (1.04 g, 3.0 equiv) and MeOH (224.0 mg, 2.0 equiv) were added. The resulting mixture was stirred at -40 °C for 4 d till full conversion of **1a** by TLC analysis. Then the reaction mixture was subjected to column chromatography purification using PE/EtOAc (3:1, v/v) as the eluent affording the desired product (*S*)-**3a** in 98% yield (1.1 g) with 90% ee.

Step III: To a 25 mL sealed tube were added (*S*)-**3a** (323 mg, 1.0 mmol, 99% ee), concentrated HCl /EtSH (1/2, v/v, 5 mL). The resulting mixture was stirred at reflux overnight and then the reaction mixture was extracted with Et_2O (10 mL × 3), the aqueous layer was concentrated under vacuum giving the *L*-Carbidopa hydrochloric salt as a brown solid in 95% yield.

Step IV[13]: To obtain the monohydrate derivative, the hydrochloric salt was dissolved in the minimum volume of isopropanol. The pH was adjusted to 6.4 by slowly adding dimethylamine (2 M in THF). During this process, the desired product, *L*-Carbidopa monohydrate, precipitated out. After filtration, it

was obtained as a brown solid in a 95% yield (231.2 mg). $[\alpha]^{20}_{D}$ = -14.1 (c = 0.3, MeOH); m.p. 203-205 ^{o}C. ^{1}H NMR (400 MHz, DMSO-d_6): δ 6.63-6.58 (m, 2H), 6.45 (d, *J* = 8.0 Hz, 1H), 2.80 (dd, *J* = 13.2 Hz, 1H), 2.68 (dd, *J* = 13.2 Hz, 1H), 1.16 (s, 3H); ^{13}C NMR (100 MHz, DMSO-d_6): δ 174.5, 144.8, 144.2, 126.6, 121.3, 118.1, 115.4, 66.1, 40.8, 19.7. IR (neat): 3205, 2970, 2396, 2234, 2108, 1717, 1650, 1456, 1364, 1053, 1024, 758 cm^{-1}; Exact mass calcd for $C_{10}H_{15}N_2O_4$ $[M+H]^+$: 227.1026, Found: 227.1027.

7. Transformation of hydrazino nitrile products

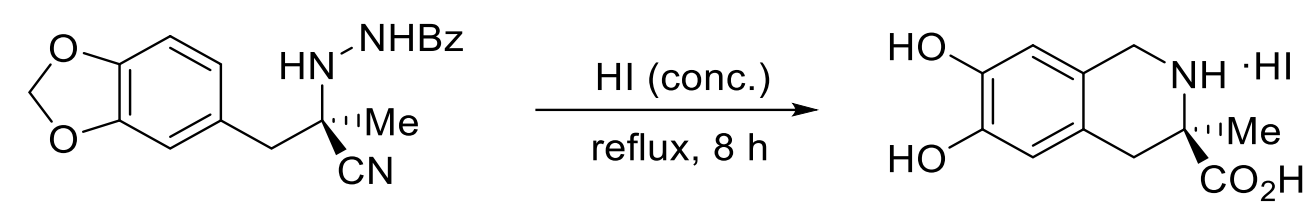

To an oven-dried Schlenk tube were added (*R*)-**3a** (32.3 mg, 0.1 mmol) and HI (conc., 1.0 mL). The resulting mixture was heated at reflux for 8 h. After cooling to room temperature, the reaction mixture was extracted with ethyl ether (5 mL × 3). The aqueous phase was concentrated under vacuum giving the hydriodic acid salt as a brown solid in 85% yield (28.7 mg). $[\alpha]^{20}_{D}$ = -13.23 (c = 0.5, MeOH); m.p. >320 ºC; ^{1}H NMR (400 MHz, D_2O): δ 6.73 (d, *J* = 8.8 Hz, 2H), 4.36 (d, *J* = 16.0 Hz, 1H), 4.25 (d, *J* = 16.0 Hz, 1H)., 3.23 (d, *J* = 17.0 Hz, 1H), 2.99 (d, *J* = 17.1 Hz, 1H), 1.60 (s, 3H). ^{13}C NMR (100 MHz, D_2O): δ 174.0, 144.1, 143.5, 122.0, 118.4, 116.1, 113.4, 59.9, 41.3, 33.7, 20.0; IR (neat): 3587, 3003, 1717, 1522, 1456, 1364, 1225, 501 cm^{-1}; HRMS (ESI): Exact mass calcd for $C_{11}H_{13}NNaO_4$ $[M+Na]^+$: 246.0737, Found: 246.0736.

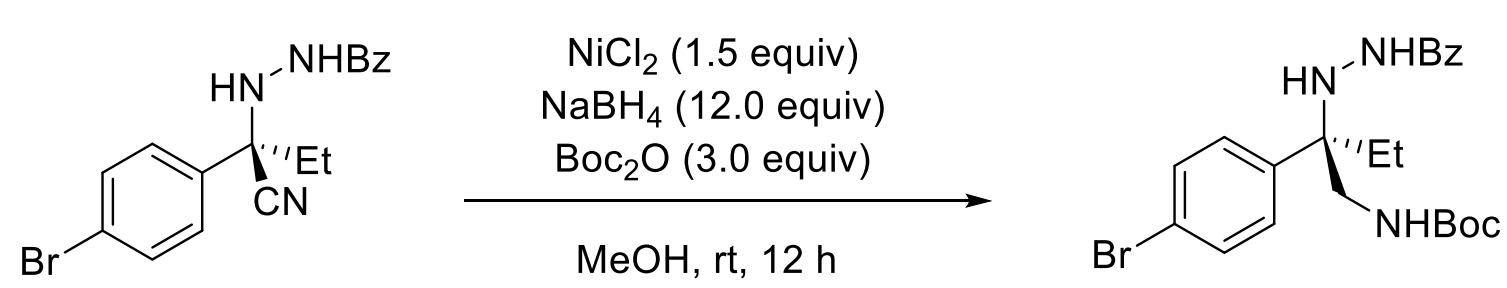

To an oven-dried Schlenk tube were added (*R*)-**3ag** (35.7 mg, 0.1 mmol), $NiCl_2$ (19.5 mg, 0.15 mmol, 1.5 equivs) and Boc_2O (65.4 mg, 3.0 equivs) and MeOH (10 mL). The mixture was cooled to 0 °C and $NaBH_4$ (45.3 mg, 12.0 equivs) was then added slowly. The reaction system was stirred at room temperature for 12 h. After completion of the reaction (TLC), the reaction was quenched with saturated NH_4Cl solution and the mixture was extracted with EtOAc (10 mL × 3). The concentrated organic layer was dried over anhydrous Na_2SO_4. The organic layer was then concentrated under reduced pressure and purified by flash column chromatography on silica gel PE:EtOAc (5:1, v/v) to afford (*R*)**-7** in 78% yield (35.9 mg) as colorless oil. HPLC analysis (Chiralpak IA, Hexane/^{i}PrOH = 85/15, 1.0 mL/min, 230 nm; t_r (major) = 14.70 min, t_r (minor) = 9.99 min) gave the product: 93% ee; $[\alpha]^{20}_{D}$ = +3.0 (c = 0.9, $CHCl_3$); m.p. 156-157 ºC; ^{1}H NMR (400 MHz, $CDCl_3$): δ 8.53 (s, 1H), 7.75-7.72 (m, 3H), 7.39-7.35 (m, 2H),

7.32-7.29 (m, 2H), 5.28 (s, 1H), 5.11 (dd, J = 9.3, 4.6 Hz, 1H), 3.75 (dd, J = 14.7, 9.2 Hz, 1H), 3.05 (dd, J = 14.7, 4.7 Hz, 1H), 1.69-1.57 (m, 2H), 1.37 (s, 9H), 0.70 (t, J = 7.4 Hz 3H); ^{13}C NMR (100 MHz, $CDCl_3$): δ 166.5, 157.4, 140.1, 132.5, 131.72, 131.66, 128.7, 128.5, 126.9, 121.2, 79.9, 65.7, 45.2, 28.4, 27.4, 7.8; IR (neat): 3003, 2970, 1717, 1456, 1364, 1223, 1084, 528 cm^{-1}; HRMS (ESI): Exact mass calcd for $C_{22}H_{28}{}^{79}BrN_3NaO_3$ $[M+Na]^+$: 484.1206, Found: 484.1213.

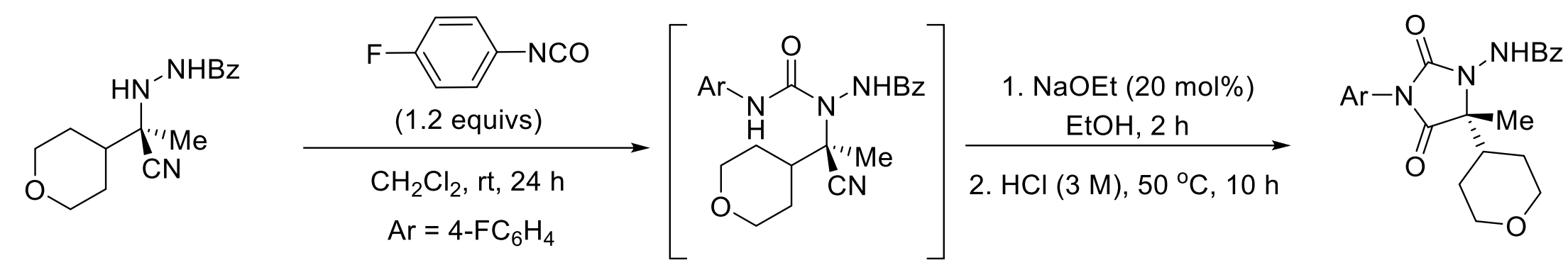

(*R*)-**3u**, 95% ee

(*R*)-**8**, 85%, 94% ee

To an oven-dried Schlenk tube were added (*R*)-**3u** (27.3 mg, 0.1 mmol), 4-fluorophenyl isocyanate (16.5 mg, 1.2 equiv) and anhydrous CH_2Cl_2 (1.0 mL). The resulting mixture was stirred at room temperture for 24 h. Then the precipitated product was filtered by suction and washed with dichloromethane to obtain pure intermediate. To the intermediate in EtOH (1.0 mL) was added NaOEt (1.7 mg, 20 mol%), the reaction system was stirred at room temperature for 2 h. Then HCl (3 M, 0.5 mL) was added to the reaction mixture which was then left at 50 ºC for 10 h. The desired product was precipitated out, after filtration and washed with water give (*R*)-**8** as white solid in 85% yield (34.9 mg). HPLC analysis (Chiralpak IA, Hexane/^{i}PrOH = 85/15, 1.0 mL/min, 230 nm; t_r (major) = 13.97 min, t_r (minor) = 17.33 min) gave the product: 94% ee; $[\alpha]^{20}_D$ = +19.6 (c = 1.0, $CHCl_3$); m.p. 287-289 ºC; ^{1}H NMR (400 MHz, $CDCl_3$): δ 8.92 (s, 1H), 7.75-7.72 (m, 2H), 7.53-7.51 (m, 1H), 7.44-7.36 (m, 4H), 7.17-7.13 (m, 2H), 4.03-3.93 (m, 2H), 3.41-3.28 (m, 2H), 2.07-2.03 (m, 1H), 1.84-1.78 (m, 2H), 1.58-1.46 (m, 5H); ^{13}C NMR (100 MHz, $CDCl_3$): δ 173.1, 167.2, 162.2 (d, J = 248.8 Hz), 155.3, 132.8, 130.5, 128.7, 128.2 (d, J = 8.8 Hz), 127.6, 126.8 (d, J = 3.1 Hz), 116.2 (d, J = 22.8 Hz), 69.0, 67.6 (d, J = 49.4 Hz), 42.0, 26.6 (d, J = 8.8 Hz), 17.2; ^{19}F NMR (376 MHz, $CDCl_3$): δ -112.0. IR (neat): 3005, 2970, 2855, 2326, 1791,1716, 1489, 1363. 1223, 530 cm^{-1}; HRMS (ESI): Exact mass calcd for $C_{22}H_{22}FN_3NaO_4$ $[M+Na]^+$: 434.1487, Found: 434.1495.

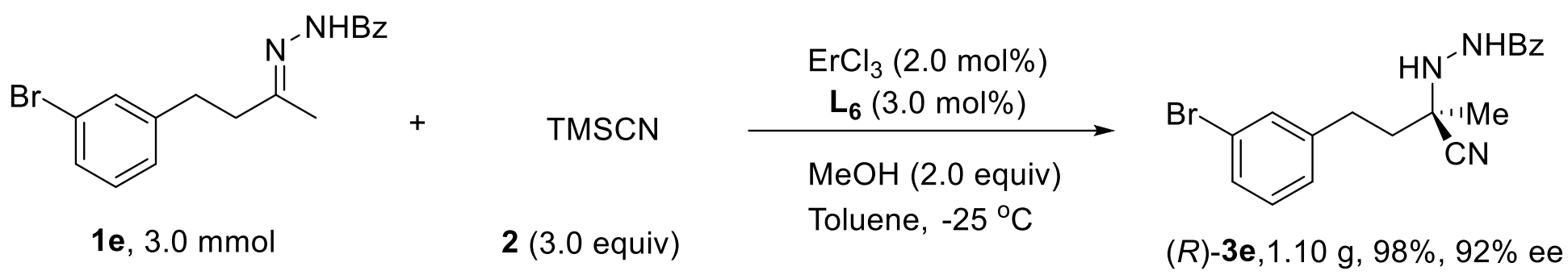

Under an atmosphere of N_2, to a 100 mL sealed tube were added $ErCl_3$ (19.1 mg, 2.0 mol%) and **L_6** (57.3 mg, 3.0 mol%), followed by 30 mL of anhydrous toluene. The solution was stirred at 25 ºC for 2 h, and then hydrazone **1e** (1.03 g, 3.0 mmol) was added. After the mixture was continued to stir at 25 ºC for 15 min. Then the mixture was cooled to -25 ºC for 0.5 h, TMSCN (900 mg, 3.0 equiv) and MeOH (192 mg, 2.0 equiv) were added. The resulting mixture was stirred at -25 ºC for 4 d till full conversion of **1e** by TLC analysis. After concentration, the resulting mixture was subjected to column chromatography purification using PE/EtOAc (3:1, v/v) as the eluent, to afford the desired prduct (*R*)-**3e** in 98% yield (1.09 g) with 92% ee. For the full characterization of (*R*)-**3e**, please see section 5.

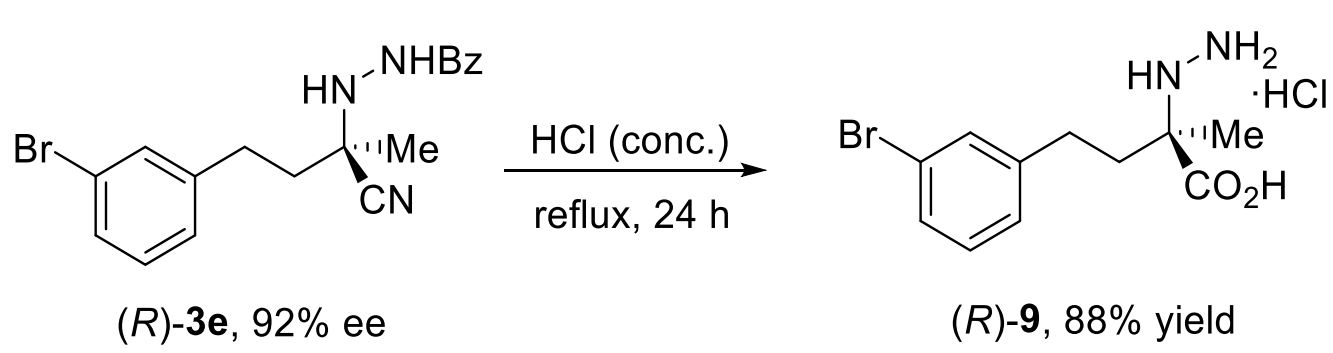

To a 25 mL sealed tube were added (*R*)-**3e** (1.10 g) and HCl (conc., 15 mL). After stirring at reflux for 24 h, the reaction mixture was extracted with Et_2O (30 mL × 3), the aqueous layer was concentrated under vacuum giving the (*R*)-**9** hydrochloric salt as a yellow solid in 88% yield (860.3 mg). $[\alpha]^{20}_D$ = -68.9 (c = 0.5, MeOH); m.p. 240-241 ºC; ^{1}H NMR (400 MHz, D_2O): δ 7.43-7.36 (m, 2H), 7.21-7.16 (m, 2H), 2.63-2.53 (m, 2H), 2.05-2.00 (m, 2H), 1.46-1.44 (m, 3H); ^{13}C NMR (100 MHz, D_2O): 175.5, 143.2, 131.2, 130.4, 129.3, 127.3, 121.9, 66.4, 36.9, 29.0, 18.7; IR (neat): 3030, 2757, 2360, 1741, 1716, 1591, 1184, 831, 786 cm^{-1}; HRMS (ESI): Exact mass calcd for $C_{11}H_{16}{}^{79}BrN_2O_3$ $[M+H]^+$: 287.0390, Found: 287.0381

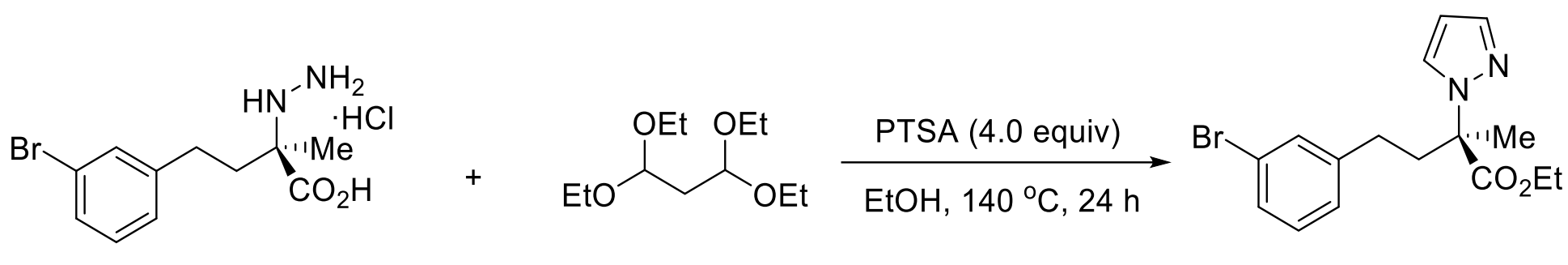

(*R*)-**9**, 92% ee (1.5 equiv) (*R*)-**10**, 91%, 92% ee

Under an atmosphere of N_2, to a 10 mL sealed tube were added **9** (32.2 mg, 0.1 mmol), 1,1,3,3-tetraethoxypropane (33.0 mg, 1.5 equiv), PTSA (68.9 mg, 4.0 equiv) and 1.0 mL of anhydrous EtOH. The reaction mixture was stirred at 140 °C for 24 h. Then the solvent was evaporated under reduced pressure and the resulting mixture was directly subjected to column chromatography purification using PE/EtOAc (15:1, v/v) as the eluent, to afford the desired product (*R*)-**10** in 91% yield (31.8 mg) as colorless oil. HPLC analysis (Chiralpak IC, Hexane/^{i}PrOH = 97/3, 1.0 mL/min, 230 nm; t_r (major) = 12.49 min, t_r (minor) = 19.04 min) gave the product: 92% ee, $[\alpha]^{20}_D$ = -14.2 (c = 0.8, $CHCl_3$); ^{1}H NMR (400 MHz, $CDCl_3$): δ 7.60-7.59 (m, 2H), 7.32-7.30 (m, 2H), 7.15-7.07 (m, 2H), 6.32 (t, *J* = 5.0 Hz, 1H), 4.19 (q, *J* = 7.1 Hz, 2H), 2.64-2.63 (m, 1H), 2.53-2.41 (m, 2H), 2.32-2.25 (m, 1H), 1.89 (s, 3H), 1.22 (t, *J* = 7.1 Hz, 3H); ^{13}C NMR (100 MHz, $CDCl_3$): δ 171.9, 143.4, 139.5, 131.4, 130.0, 129.2, 127.7, 127.1, 122.4, 105.5, 68.8, 61.9, 39.5, 29.6, 22.5, 14.0. IR (neat): 2937, 1743, 1660, 1508, 1219, 1072, 1049, 771 cm^{-1}; HRMS (ESI): Exact mass calcd for $C_{16}H_{19}{}^{79}BrN_2NaO_2$ $[M+Na]^+$: 373.0522, Found: 373.0523.

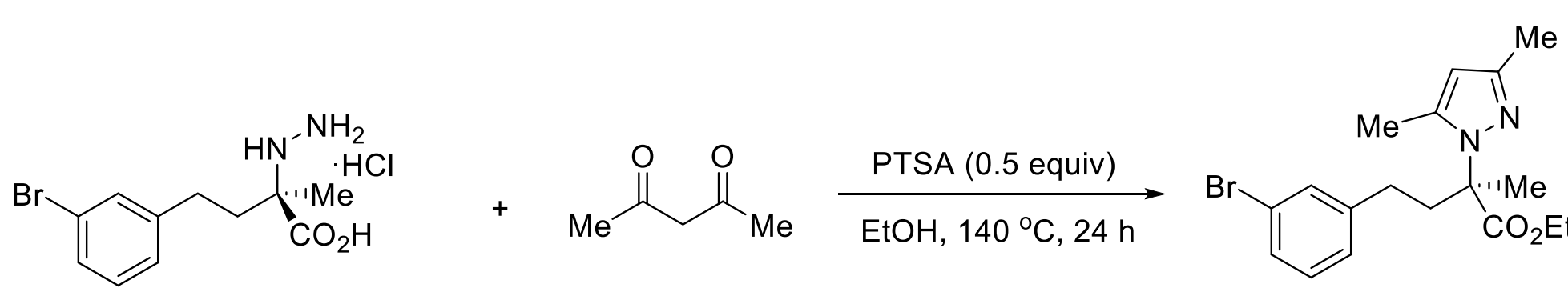

(*R*)-**9**, 92% ee (1.1 equiv) (*R*)-**11**, 90%, 93% ee

Under an atmosphere of N_2, to a 10 mL sealed tube were added **9** (32.2 mg, 0.1 mmol), acetylacetone (11.0 mg, 1.1 equiv), followed by PTSA (8.6 mg, 0.5 equiv) and 1.0 mL of anhydrous EtOH. The reaction mixture was stirred at 140 °C for 24 h. Then the solvent was evaporated under reduced pressure and directly subjected to a column chromatography purification using PE/EtOAc (15:1, v/v) as the eluent, to afford the desired product (*R*)-**11** in 90% yield (34.0 mg) as colorless oil. HPLC analysis (Chiralpak IC, Hexane/^{i}PrOH = 97/3, 1.0 mL/min, 230 nm; t_r (major) = 6.41 min, t_r (minor) = 7.90 min) gave the product: 93% ee, $[\alpha]^{20}_D$ = -10.9 (c = 0.9, $CHCl_3$); ^{1}H NMR (400 MHz, $CDCl_3$): δ

7.31-7.29 (m, 2H), 7.12-7.08 (m, 2H), 5.87 (s, 1H), 4.24 (q, J = 7.1 Hz, 2H), 2.67-2.60 (m, 1H), 2.50-2.44 (m, 2H), 2.26-2.19 (m, 4H), 2.15 (s, 3H), 1.87 (s, 3H), 1.27 (t, J = 7.1 Hz, 3H); ^{13}C NMR (100 MHz, $CDCl_3$): δ 173.2, 146.3, 143.8, 139.0, 131.4, 129.9, 129.0, 127.1, 122.4, 107.7, 66.2, 61.8, 39.6, 29.6, 24.0, 14.1, 13.6, 12.4. IR (neat): 2980, 1743, 1737, 1508, 1219, 1109, 772 cm^{-1}; HRMS (ESI): Exact mass calcd for $C_{18}H_{23}{}^{79}BrN_2NaO_2$ $[M+Na]^+$: 401.0835 Found: 401.0839.

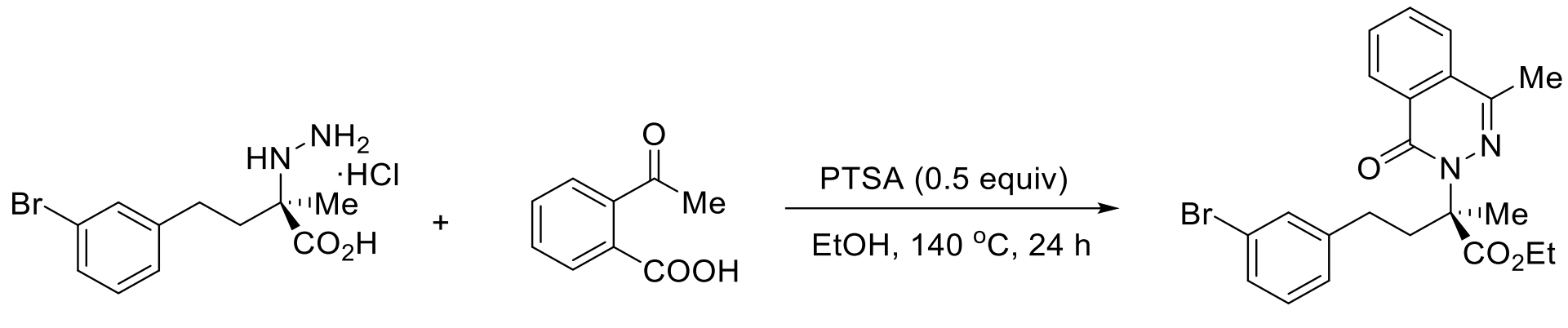

(*R*)-**9** 92% ee (1.1 equiv) (*R*)-**12**, 78%, 92% ee

Under an atmosphere of N_2, to a 10 mL sealed tube were added **9** (32.2 mg, 0.1 mmol), 2-acetylbenzoic acid (18.1 mg, 1.1 equiv), followed by PTSA (8.6 mg, 0.5 equiv) and 1.0 mL of anhydrous EtOH. The reaction mixture was stirred at 140 °C for 24 h. Then the solvent was evaporated under reduced pressure and directly subjected to a column chromatography purification using PE/EtOAc (3:1, v/v) as the eluent, to afford the desired product (*R*)-**12** in 78% yield (34.4 mg) as colorless oil. HPLC analysis (Chiralcel AD-H, Hexane/^{i}PrOH = 95/5, 1.0 mL/min, 230 nm; t_r (major) = 23.23 min, t_r (minor) = 32.25 min) gave the product: 92% ee, $[\alpha]^{20}_D$ = +30.2 (c = 1.0, $CHCl_3$), m.p. 120-122 °C; ^{1}H NMR (400 MHz, $CDCl_3$): δ 8.37-8.35 (m, 1H), 7.82-7.78 (m, 1H), 7.75-7.69 (m, 2H), 7.27-7.26 (m, 1H), 7.15-7.12 (m, 1H), 7.08-7.06 (m, 1H), 7.03-6.99 (m, 1H), 4.25 (q, J = 7.1 Hz, 2H), 2.79-2.69 (m, 2H), 2.62 (s, 3H), 2.49-2.38 (m, 2H), 1.80 (s, 3H), 1.24 (t, J = 7.1 Hz, 3H); ^{13}C NMR (100 MHz, $CDCl_3$): δ 172.5, 159.0, 143.9, 142.6, 133.0, 131.4, 131.0, 129.6, 129.5, 128.7, 127.6, 127.1, 127.0, 124.5, 122.1, 61.2, 36.7, 30.0, 22.2, 19.1, 14.2. IR (neat): 2980, 1736, 1655, 1593, 1473, 1248, 1116, 772 cm^{-1}; HRMS (ESI): Exact mass calcd for $C_{22}H_{23}{}^{79}BrN_2NaO_3$ $[M+Na]^+$: 465.0784, Found: 465.0795

8. Mechanistic studies.

To understand the origin of the high enantioselectivity of C4 sulfonyl-PYBOX enabled cyanation of ketone hydrazones, mechanistic investigations were conducted, including non-linear effects (NLE), the influence of Er/ligand ratio variation, kinetic experiments, and NMR analysis.

8.1 The non-linear effects (NLE) study.

The NLE effects for the cyanation of ketone hydrazone **1a** was performed under the standard reaction condition. Six reactions using a mixture of chiral ligands *(S,R)*-$\mathbf{L_6}$ and *(R,S)*-$\mathbf{L_6}$ with different proportions were conducted. The ee value of $\mathbf{L_6}$ was determined by chiral HPLC analysis. As shown in Figure S3, a linear relationship between the enantiopurities of the products and corresponding chiral ligands was observed, indicating the possible involvement of one catalyst in each enantiodetermining transition state.

Figure S3. NLE study for Strecker reaction of 1a.

Entry	ee of $\mathbf{L_6}$ (%)	ee of **3a** (%)
1	0	0
2	20	16
3	38	34
4	58	50
5	79	69
6	99	90

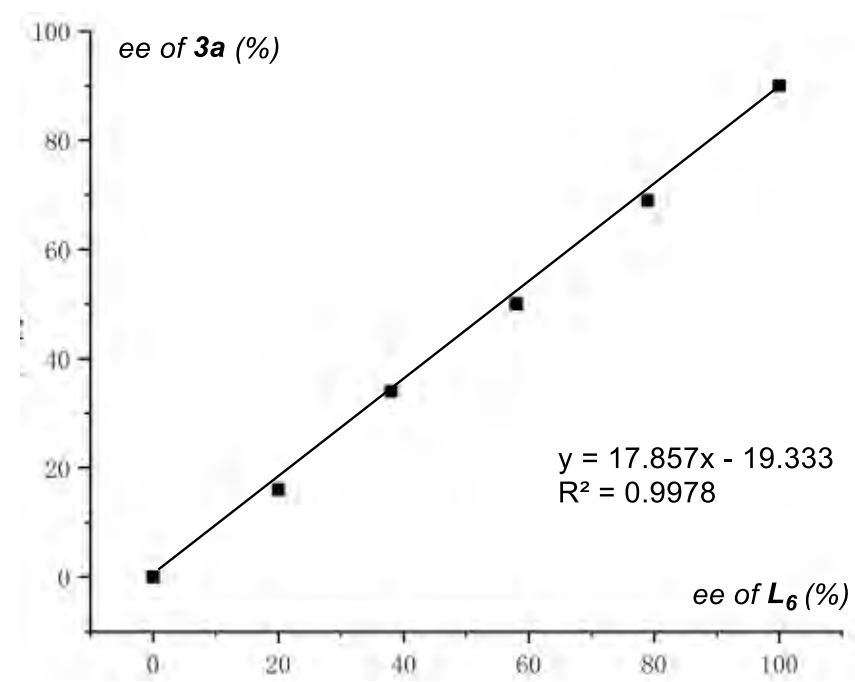

8.2 ESI-MS studies

To get more information of the interaction of **1a** with Er complex, the ESI-MS analysis was conducted, indicating the possible generation of $ErCl_2$($\mathbf{L_6}$)$_2$, $ErCl_2$($\mathbf{L_6}$)(**1a**) and $ErCl_2$($\mathbf{L_6}$)(**1a**)$_2$ complex.

General procedure for measuring a ESI–MS sample: To a test tube was added $ErCl_3$ (0.01 mmol), $\mathbf{L_6}$ (0.015 mmol) and MeCN/CH_2Cl_2 (v:v, 1/1, 1 mL). The mixture was stirred at 25 °C for 2 h. And **1a** (1.0 equiv, 0.03 mmol) was added in one portion. Then the mixture was stirred at 25 °C for 10 min. An aliquot was taken up and diluted in the dilution vial. Lastly, the solution was injected directly into the ESI-MS spectrometer (Waters, UPLC H-CLASS-SQD).

ESI-MS (m/z) calculated for [$ErCl_2(\mathbf{L_6})_2]^+$ ($C_{58}H_{42}N_6O_8F_4S_2Cl_2Er^+$):1326.1122, Found 1326.1080.

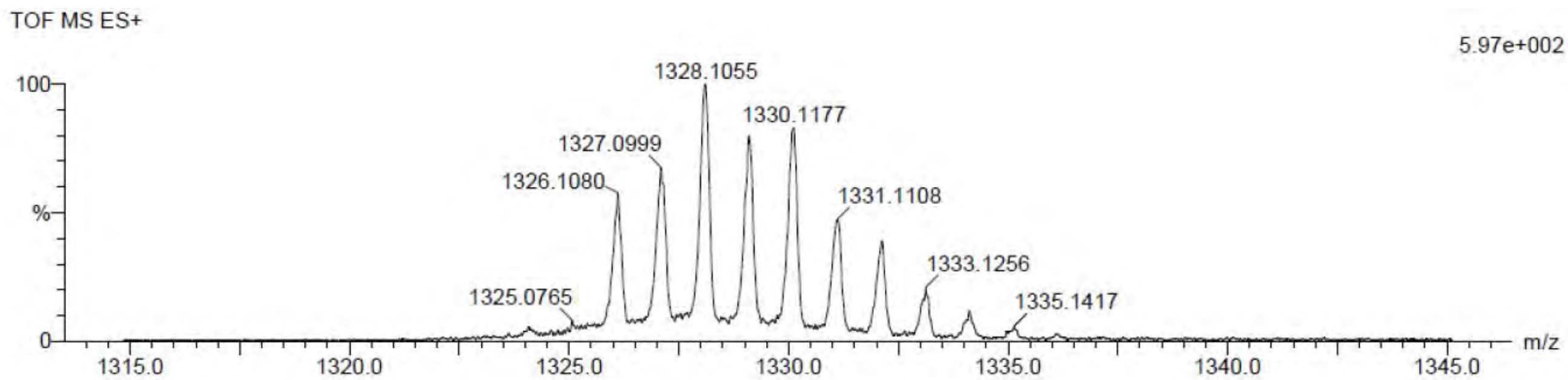

Minimum:				-50.0	
Maximum:		500.0	1000.0	200.0	
Mass	Calc. Mass	mDa	PPM	DBE	Formula
1326.1080	1326.1122	-4.2	-3.2	38.5	C58 H42 N6 O8 F4 S2 Cl2 Er

ESI-MS (m/z) calculated for [$ErCl_2(\mathbf{L_6})(\mathbf{1a})]^+$ ($C_{46}H_{37}N_5O_7F_2SCl_2Er^+$): 1077.1062, Found: 1077.1222.

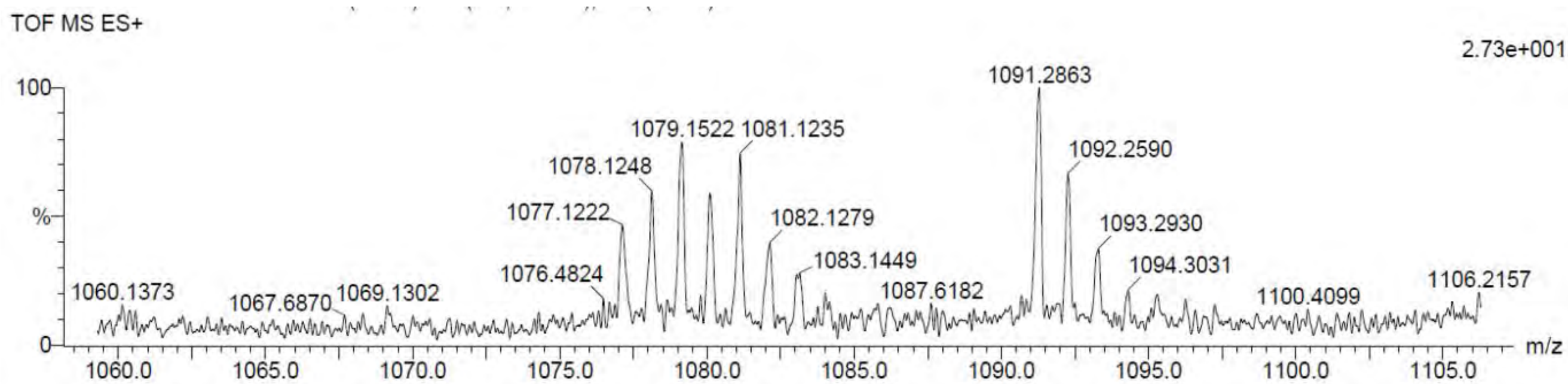

Minimum:				-50.0	
Maximum:		500.0	1000.0	200.0	
Mass	Calc. Mass	mDa	PPM	DBE	Formula
1077.1222	1077.1062	16.0	14.9	29.5	C46 H37 N5 O7 F2 S Cl2 Er

ESI-MS (m/z) calculated for [$ErCl_2(\mathbf{L_6})(\mathbf{1a})_2]^+$ ($C_{63}H_{53}N_7O_{10}F_2SCl_2Er^+$):1373.2223, Found: 1373.2206.

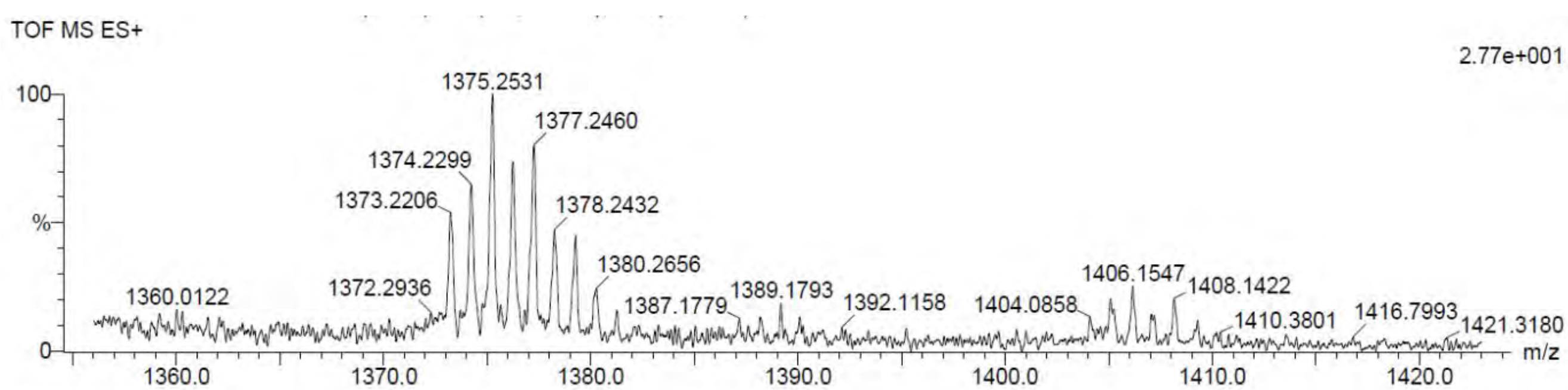

Minimum:				-50.0	
Maximum:		500.0	1000.0	200.0	
Mass	Calc. Mass	mDa	PPM	DBE	Formula
1373.2206	1373.2223	-1.7	-1.2	39.5	C63 H53 N7 O10 F2 S Cl2 Er

8.3 NMR studies.

The interactions between the chiral catalyst $ErCl_3$/**L₆** with **1a** or TMSCN were studied via ^{13}C NMR analysis, as shown in Figure S4. The ^{13}C NMR experiments were carried out at 298 K using a Bruker DPX-500 spectrometer. With the addition of 0.02 to 0.2 equiv of $ErCl_3$/$\mathbf{L_6}$ to the solution of **1a** in $CD_3OD/CDCl_3$ (1/1, v/v), the characteristic peaks for C^a, C^c, and C^b, attributed to the alpha-carbon and imine-carbon of the hydrazone moiety, exhibited significant downfield shifts from 44.11, 14.61 and 161.41 ppm to 46.11, 15.86 and 162.28 ppm, respectively. Meanwhile, the carbonyl carbon (C^d) of the benzoyl group displayed an upfield shift from 165.63 ppm to 162.92 ppm. These results indicated the possible coordination of the imine and carbonyl groups of hydrazone **1a** to the $ErCl_3$/$\mathbf{L_6}$ complex. The imine-carbon (C^b) and carbonyl carbon (C^d) were determined by the 2D NMR.

General procedure for ^{13}C NMR analysis of the interactions between 1a and $ErCl_3$/$\mathbf{L_6}$: The sample was prepared by gradually adding $ErCl_3$/$\mathbf{L_6}$ (0.002 to 0.02 mmol) to a solution of **1a** (29.6 mg, 0.1 mmol) in $CD_3OD/CDCl_3$ (1/1, v:v). After each addition, the mixture was stirred at room temperature for 10 minutes before being subjected to ^{13}C NMR analysis at 298K.

Figure S4. NMR studies

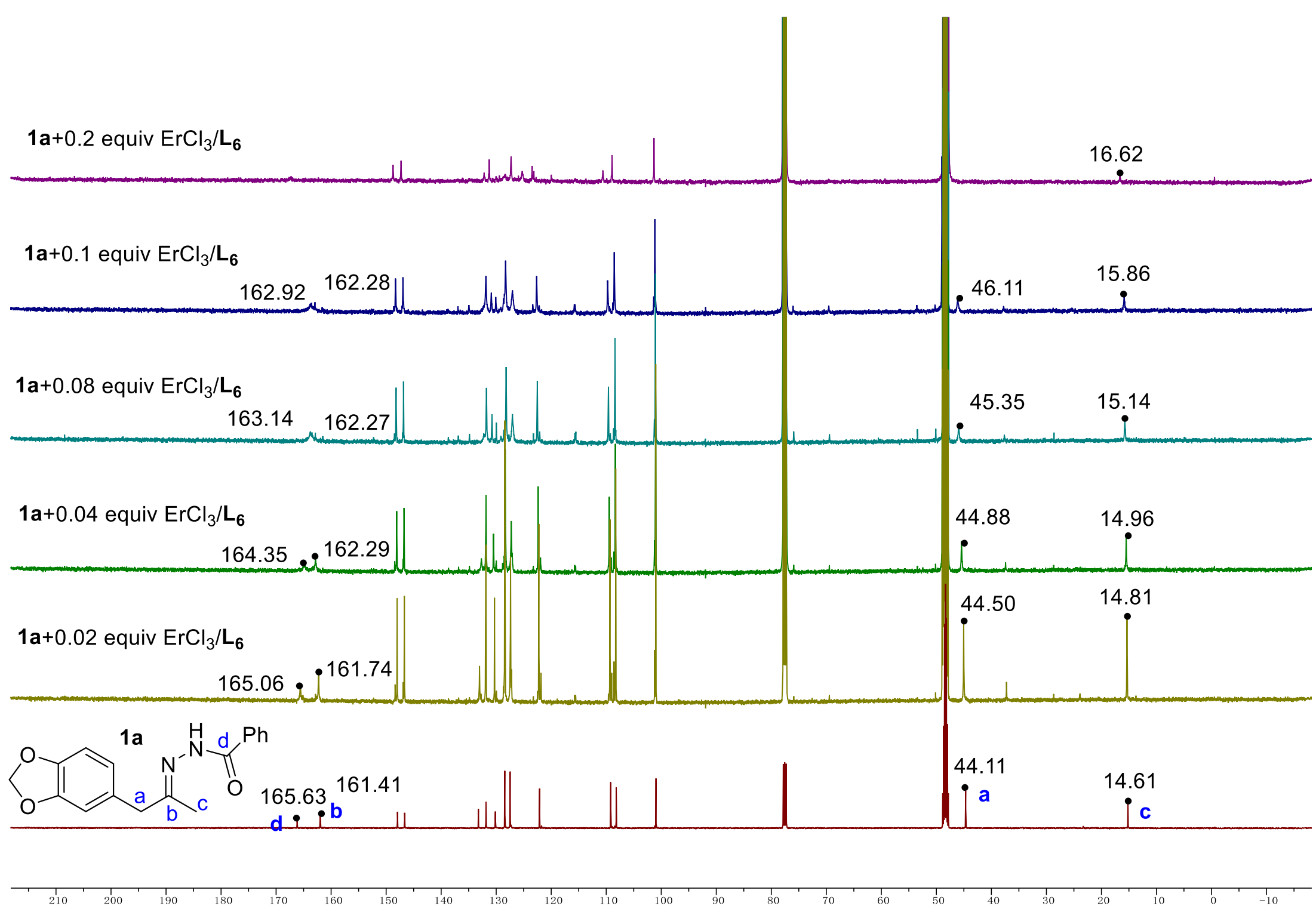

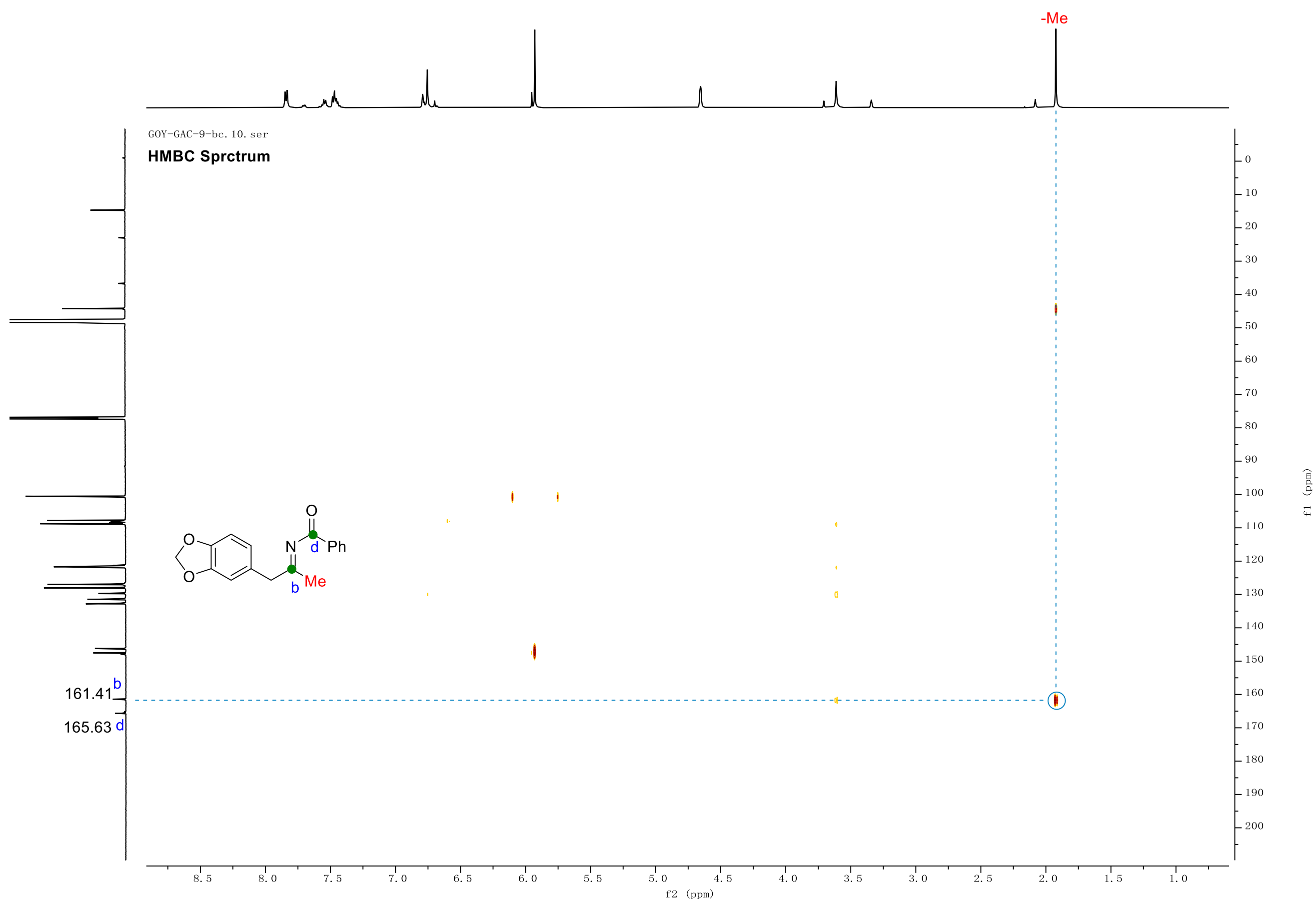

Subsequently, the interaction between TMSCN and $ErCl_3$/**L6** was further investigated. By titrating a solution of TMSCN to a solution of TFE in $CD_3OD/CDCl_3$ (1/1, v/v), with the ratio varying from 1:1 to 10:1, the characteristic peak of DCN at 110.5 ppm was observed, indicating the conversion of TMSCN to DCN in the presence of CD_3OD. Moreover, a new peak at around 115.2 ppm was also observed, suggesting the possible formation of isocyanide species.

General procedure for ^{13}C NMR analysis of the interactions between TMSCN and $ErCl_3$/L6: The TMSCN (0.01 to 0.1 mmol) was gradually added to a solution of $ErCl_3$/**L6** (0.01 mmol) in $CD_3OD/CDCl_3$ (1/1, v:v) with the ratios varying from 1:1 to 110:1. The mixture was stirred at room temperature for 10 minutes before being subjected to ^{13}C NMR analysis at 298K..

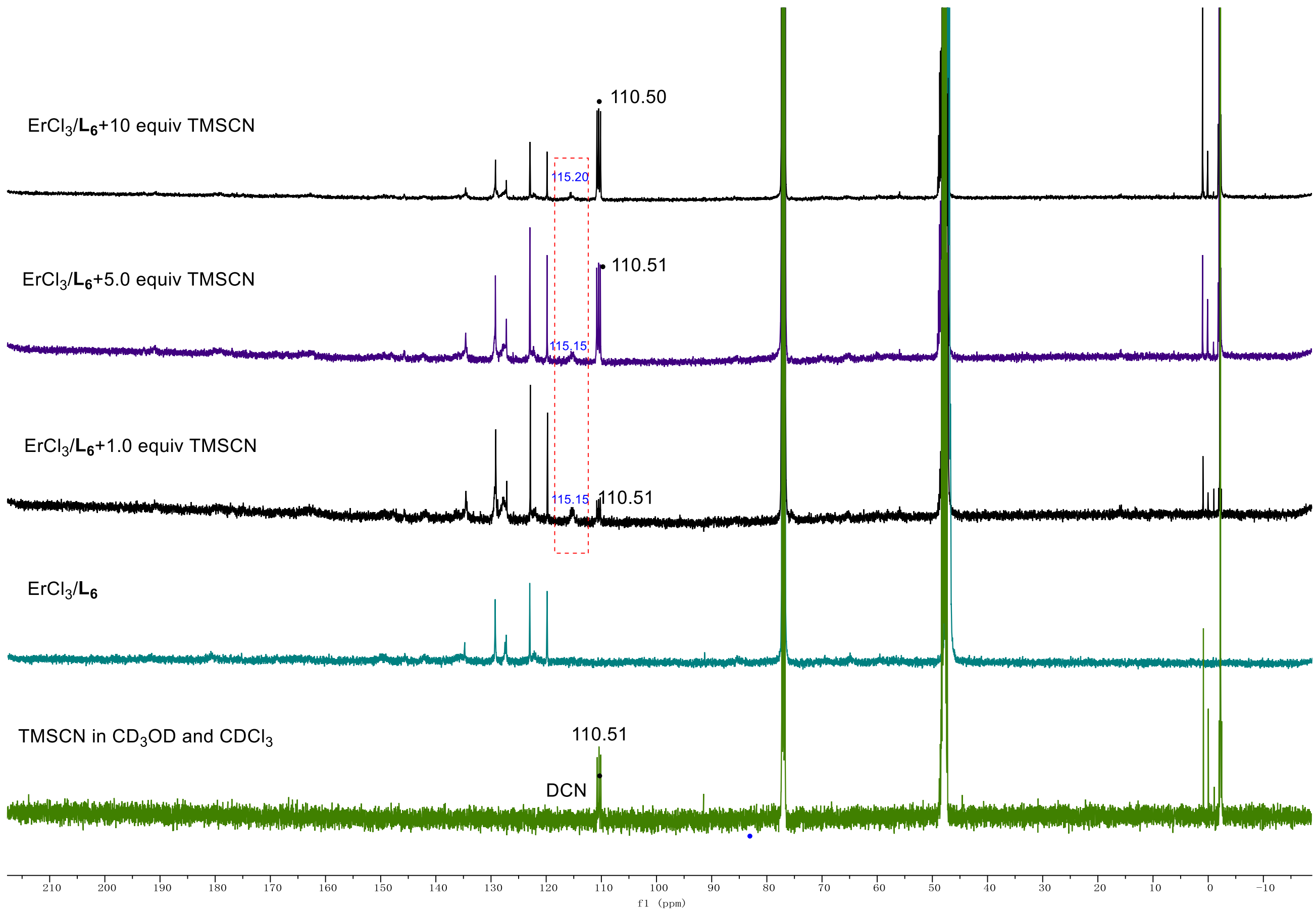

8.4 Kinetic studies.

Due to the turbidity of the reaction mixture at -20 °C, the kinetic experiments were carried out at 20 °C with 10 mol% catalyst.

The determination of the reaction order of 1a.

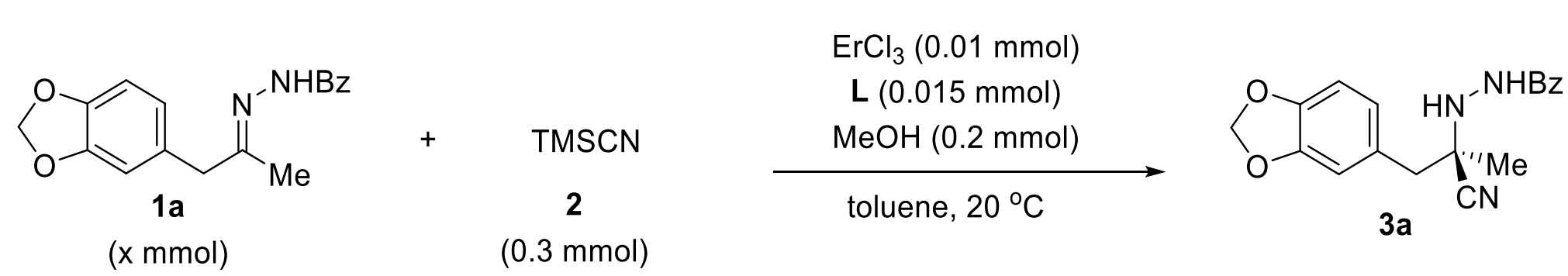

A typical experimental procedure for the determination of reaction order of **1a** is described below. Under an atmosphere of N_2, to a 10 mL oven-dried sealed tube were added $ErCl_3$ (0.01 mmol) and $\mathbf{L_6}$ (0.015 mmol), followed by 1.0 mL of anhydrous toluene. The solution was stirred at 20 °C for 2 h, and then hydrazone **1** (0.05 mmol, 0.1 mmol, 0.15 mmol, 0.2 mmol) was added. After the mixture was continued to stir at 20 °C for 15 min., TMSCN **2** (0.3 mmol) and MeOH (0.2 mmol) were added. Finally,

the yield of **3a** was monitored from 0-30 min by LC analysis of the sample taken from the reaction mixture. Based on these data below, the kinetic order of **1a** was determined to be zero.

Figure S5. Effect of loading of 1a on the reaction rates

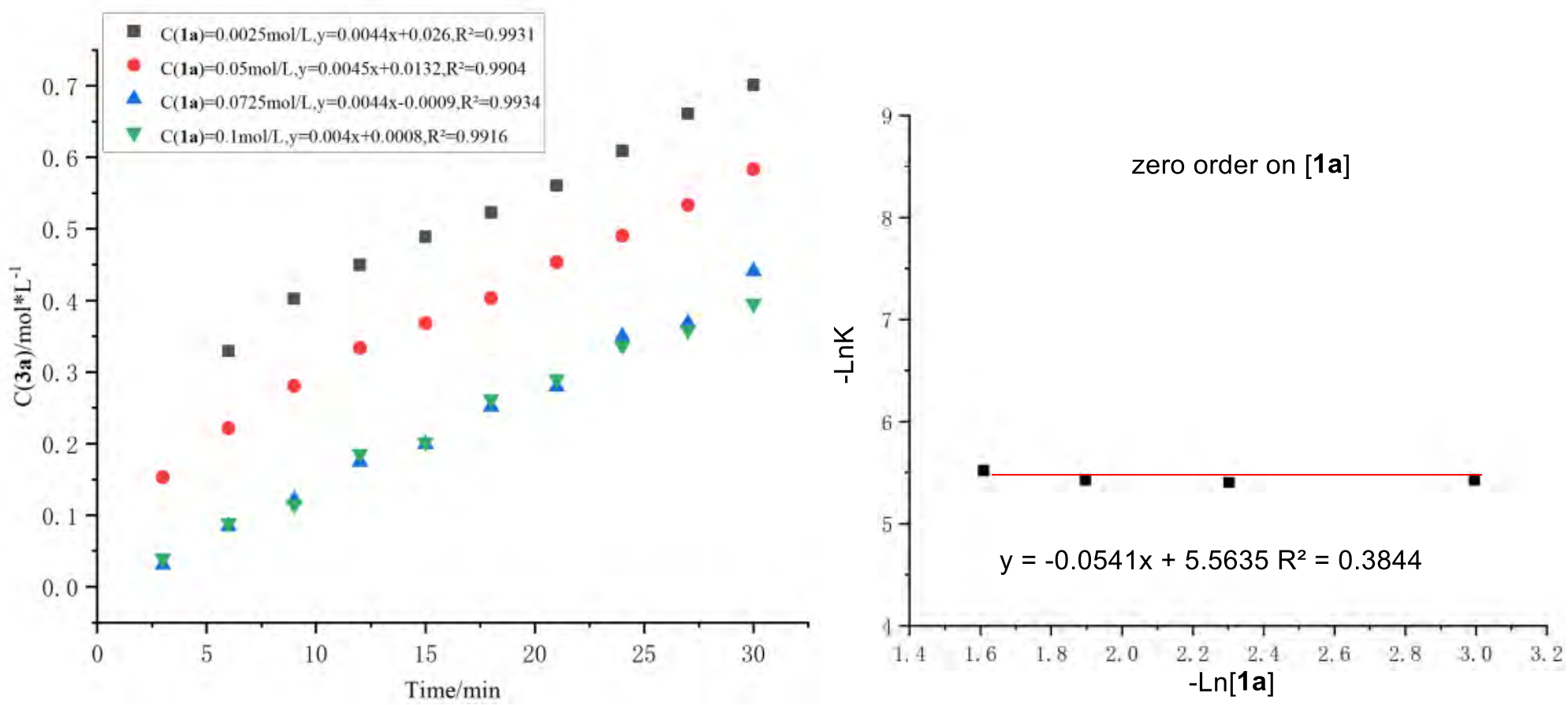

The determination of the reaction order of 2.

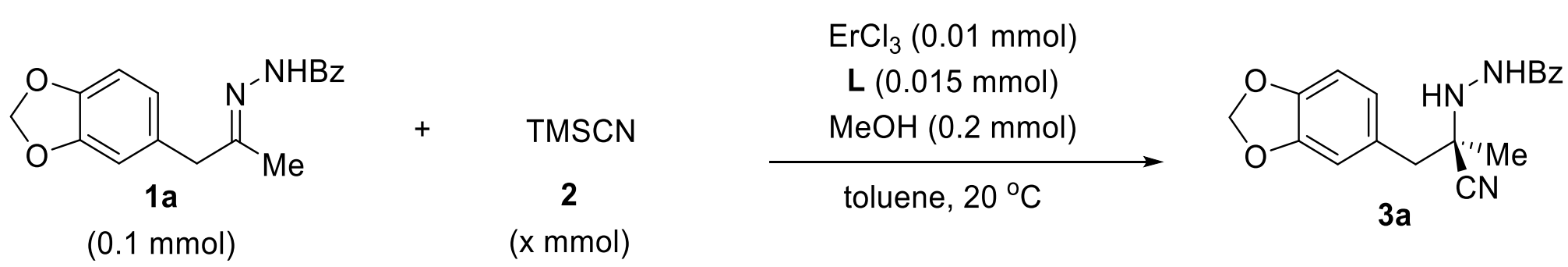

A typical experimental procedure for the determination of reaction order of **2** is described below. Under an atmosphere of N_2, to a 10 mL oven-dried sealed tube were added $ErCl_3$ (0.01 mmol) and **L_6** (0.015 mmol), followed by 1.0 mL of anhydrous toluene. The solution was stirred at 20 °C for 2 h, and then hydrazone **1** (0.01 mmol) was added. After the mixture was continued to stir at 20 °C for 15 min., TMSCN (0.1 mmol, 0.2 mmol, 0.3 mmol, 0.4 mmol, 0.5 mmol) and MeOH (0.2 mmol) were added. Finally, the yield of **3a** was monitored from 0-30 min by LC analysis of the sample taken from the reaction mixture.Based on these data below, the kinetic order of **2** was determined to be 1.54.

Figure S6. Effect of loading of TMSCN on the reaction rates

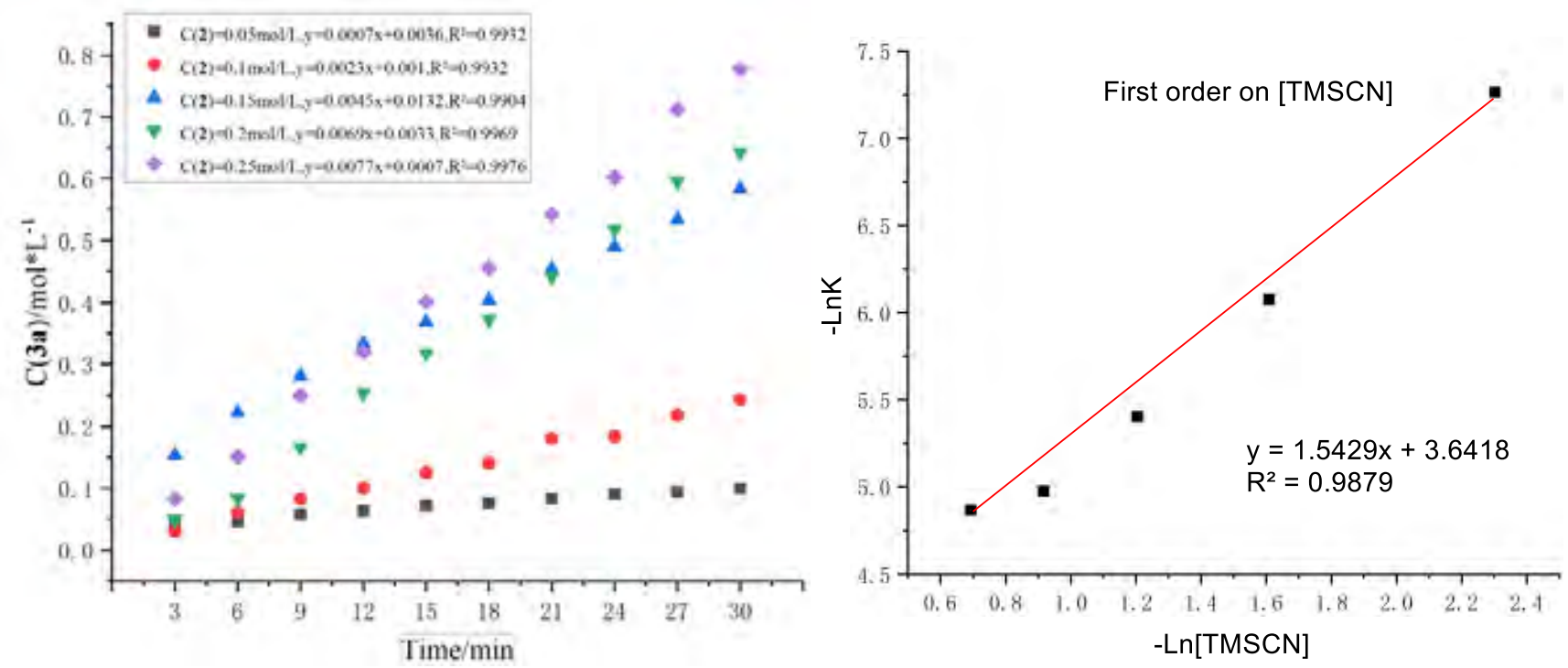

The determination of the reaction order of catalyst.

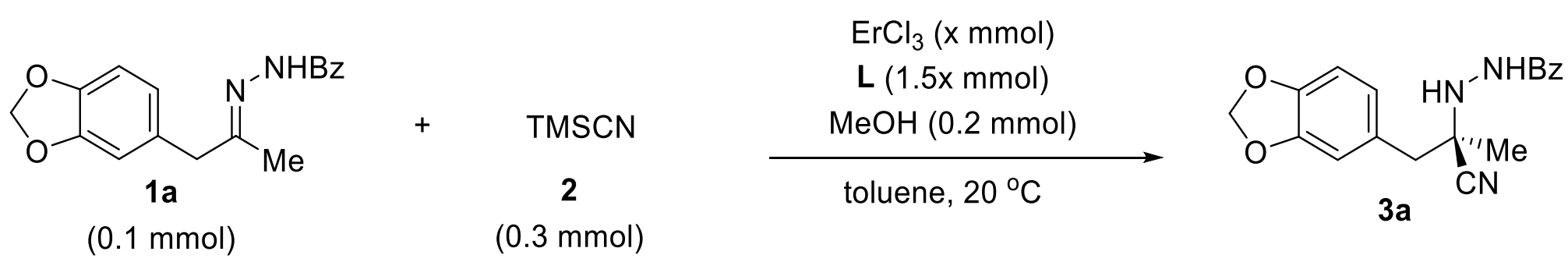

A typical experimental procedure for the determination of reaction order of **1a** is described below. Under an atmosphere of N_2, to a 10 mL oven-dried sealed tube were added $ErCl_3$ (0.0025 mmol, 0.005 mmol, 0.0075 mmol, 0.01 mmol, 0.0125 mmol) and $\mathbf{L_6}$ (0.00375 mmol, 0.0075 mmol, 0.01125 mmol, 0.015 mmol, 0.01875 mmol), followed by 1.0 mL of anhydrous toluene. The solution was stirred at 20 °C for 2 h, and then hydrazone **1** (0.1 mmol) was added. After the mixture was continued to stir at 20 °C for 15 min., TMSCN **2** (0.3 mmol) and MeOH (0.2 mmol) were added. Finally, the yield of **3a** was monitored from 0-30 min by LC analysis of the sample taken from the reaction mixture. Based on these data below, the kinetic order of catalyst was determined to be 1.374.

Figure S7. Effect of loading of catalyst on the reaction rates

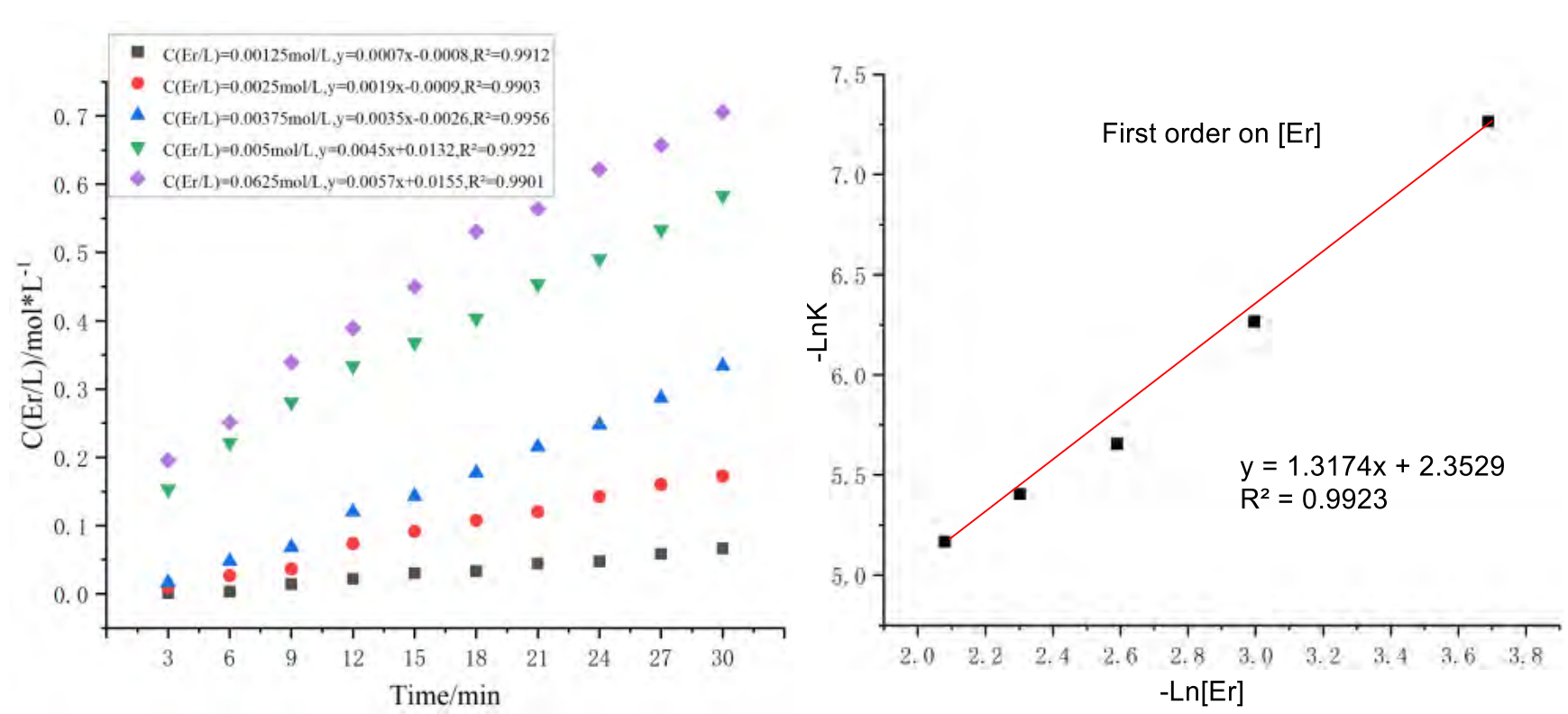

The determination of the reaction order of MeOH.

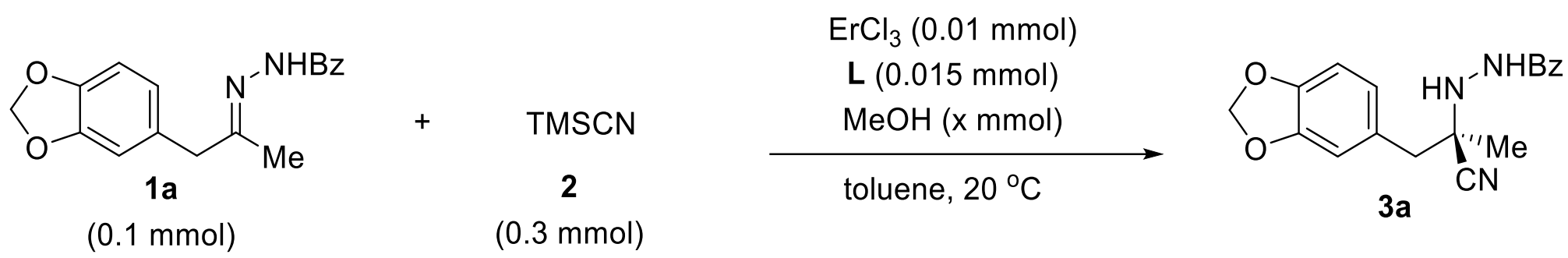

A typical experimental procedure for the determination of reaction order of **1a** is described below. Under an atmosphere of N_2, to a 10 mL oven-dried sealed tube were added $ErCl_3$ (0.01 mmol) and $\mathbf{L_6}$ (0.015 mmol), followed by 1.0 mL of anhydrous toluene. The solution was stirred at 20 °C for 2 h, and then hydrazone **1** (0.1 mmol) was added. After the mixture was continued to stir at 20 °C for 15 min., TMSCN **2** (0.3 mmol) and MeOH (0.1 mmol, 0.15 mmol, 0.2 mmol,0.25 mmol, 0.3 mmol) were added. Finally, the yield of **3a** was monitored from 0-30 min by LC analysis of the sample taken from the reaction mixture. Based on these data below, the kinetic order of **MeOH** was determined to be 0.811.

Figure S8. Effect of loading of MeOH on the reaction rates

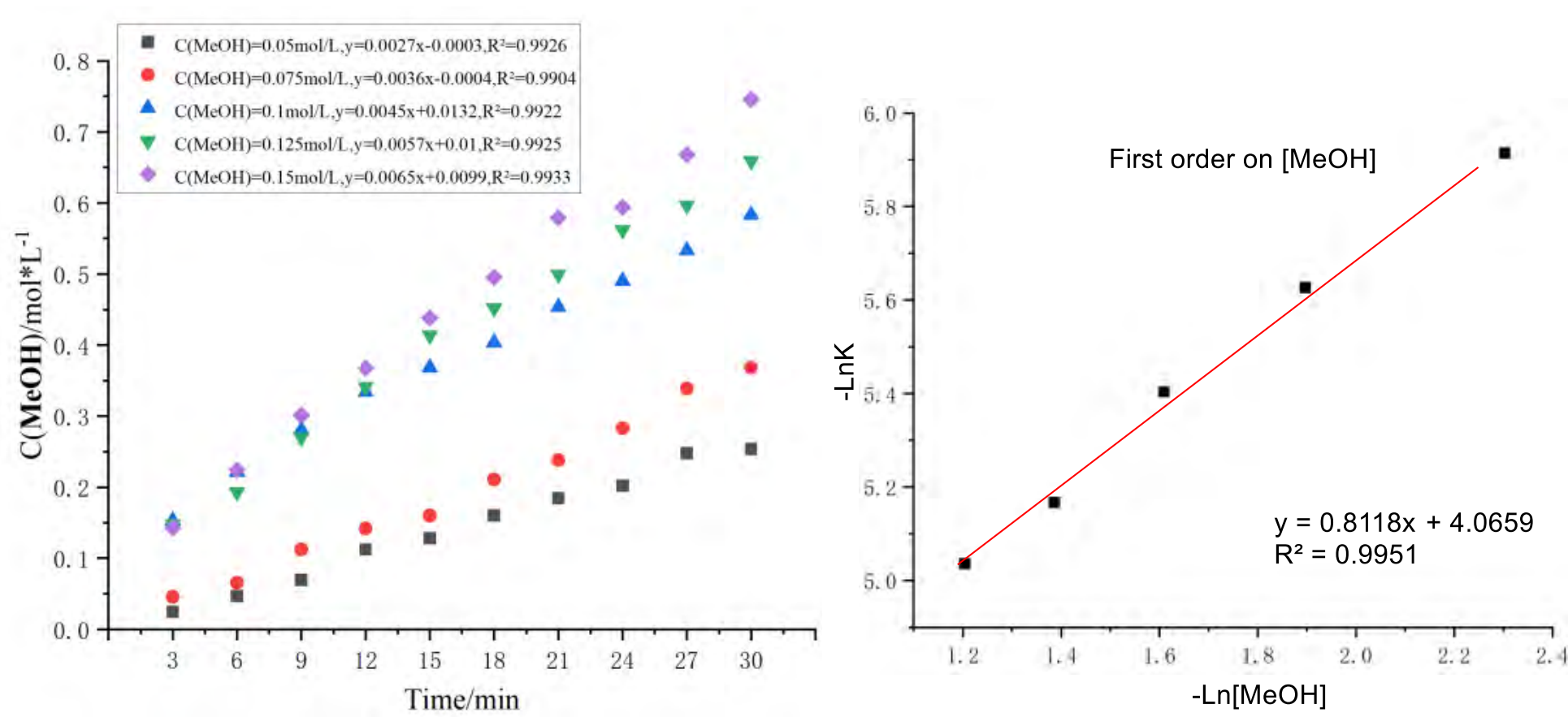

8.5 IR studies

To further validate the possible generation of isocyanide species, IR analysis was conducted. Upon addition of 5.0 equiv of TMSCN to the $ErCl_3$/$\mathbf{L_6}$, a new nitrile (NC) stretching vibration emerged at 2067 cm^{-1}. This observation aligns with Corey's report[14] of a distinct NC stretching band at 2072 cm^{-1} in analogous systems (contrasting with the characteristic TMSCN absorption at 2192 cm^{-1}), as well as Gong's documented values for yttrium-cyano (Y-NC, 2027 cm^{-1}) and lanthanum-cyano (La-NC, 2033

cm⁻¹) stretching frequencies[15]. These results suggest that an isocyanometallic intermediate is likely formed during the reaction process.

Figure S9. IR studies

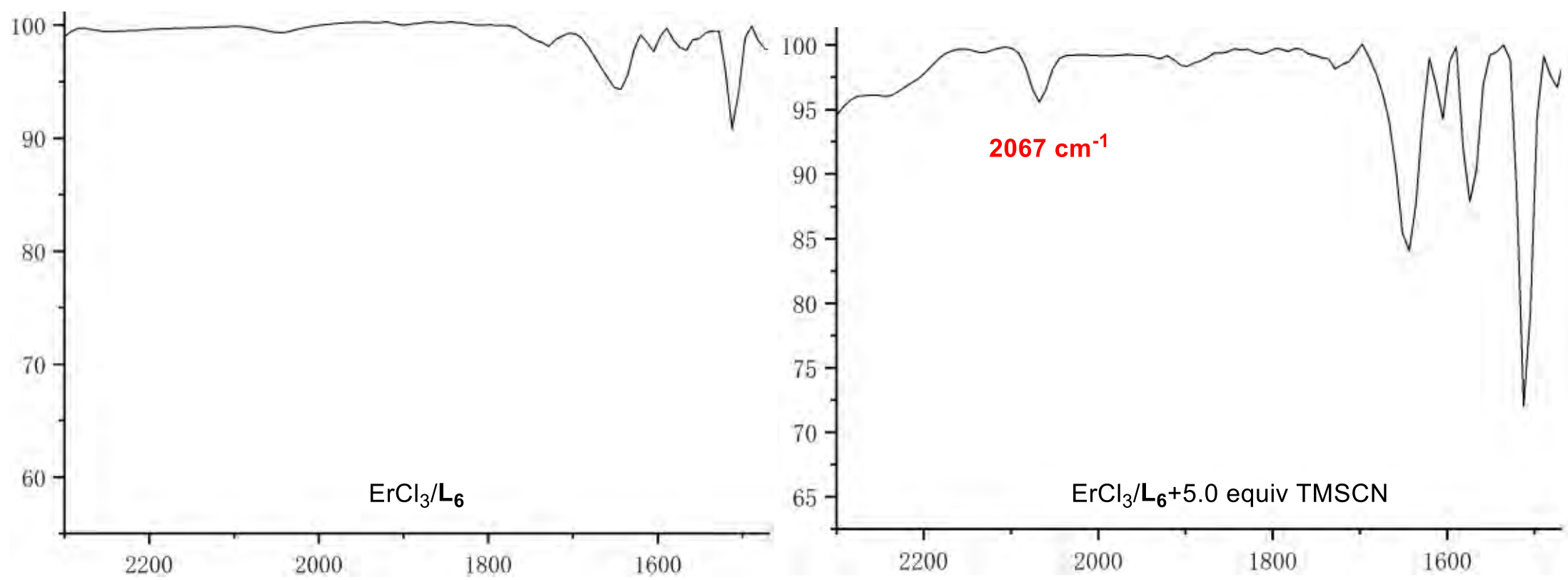

8.6 DFT calculations

In our previous work on sterically confined PYBOX-enabled Cu-catalyzed asymmetric propargylic substitution (*Nat. Chem.* **2024**, *16*, 521; *Nat. Commun.* **2025**, *16*, 4571) and CuAAC reactions (*Angew. Chem. Int. Ed.* **2023**, *62*, e202301470), we studied the role of the pyridine C4 shielding group by control experiments, X-ray analysis and DFT calculations. These studies suggest that the pyridine C4 shielding group may facilitate the formation of a sterically confined microenvironment via the cooperation with the substituents on the chiral center of oxazoline, and modulate the Lewis acidity of the metal catalysts via electronic effects. Based on these studies, we believe the steric hindrance of the C4 sulfonyl group can make the chiral cavity restricted, helpful for differentiating the sterically similar substituents on the prochiral carbon.

To shed light on the role of the C4 sulfonyl group of PYBOX in reinforcing enantiofacial discrimination, we collaborated with the Beijing Computing Center to perform DFT calculations. However, despite attempts, we failed to obtain properly converged transition-state (**TS**) models. As shown below, even the TS structures corresponding to the *R* and *S* configurations were difficult to converge to models with the same coordination number (**TS-L1-*R***: eight-coordinate complex; **TS-L1-*S***: seven-coordinate complex). In addition, the free energy of **TS-L1-*R*** is calculated to be higher than that of **TS-L1-*S*** by 20.7kcal/mol. This is opposite to the experiment result that product (*R*)-**3a** was obtained as the major enantiomer. This is also possibly due to the failure to get accurate structure of the

catalyst-substrate complex. X-ray analysis suggested a coordination number of 8 of Er(III) in the sulfonyl-PYBOX **L6**/Er(III) complex, but the ligand and the substrate account for only 5 coordination sites. It is unclear whether chloride, cyanide, or MeOH occupies the remaining sites, although ESI analysis of the reaction mixture were tried under a variety of different conditions. This made the DFT calculation very difficult. This challenge is well recognized in the theoretical studies of lanthanide compounds, mainly arising from the highly localized and strongly correlated nature of the 4f electrons, which are often not adequately described by conventional DFT methods. In addition, the electronic structures of lanthanide centers frequently involve multiple near-degenerate states, complex spin configurations, and significant spin-orbit coupling effects, all of which can cause severe self-consistent field (SCF) convergence problems and strong sensitivity to the initial guess, the choice of functional, and the treatment of the f electrons (*Phys. Rev. B* **2007**, *75*, 045121). These suggest that enantioselective control in this system is influenced not only by steric effects, but also by the complex electronic structure and coordination behavior of the Er(III) center. It is worth mentioning that DFT calculations of Er(III)-catalyzed reactions are far from trivial. As pointed out by Wilson: "*Lanthanides often require a treatment of static correlation effects via multireference methods* (*J. Chem. Theory Comput.* **2016**, *12*, 1259)." Furthermore, Furche noted that "*The KS-DFT calculations of lanthanide complexes, especially those with unconventional configurations, often suffer from slow convergence and metastable solutions* (*Dalton Trans.* **2024**, *53*, 410)."

Figure S10 Initial results on DFT calculations

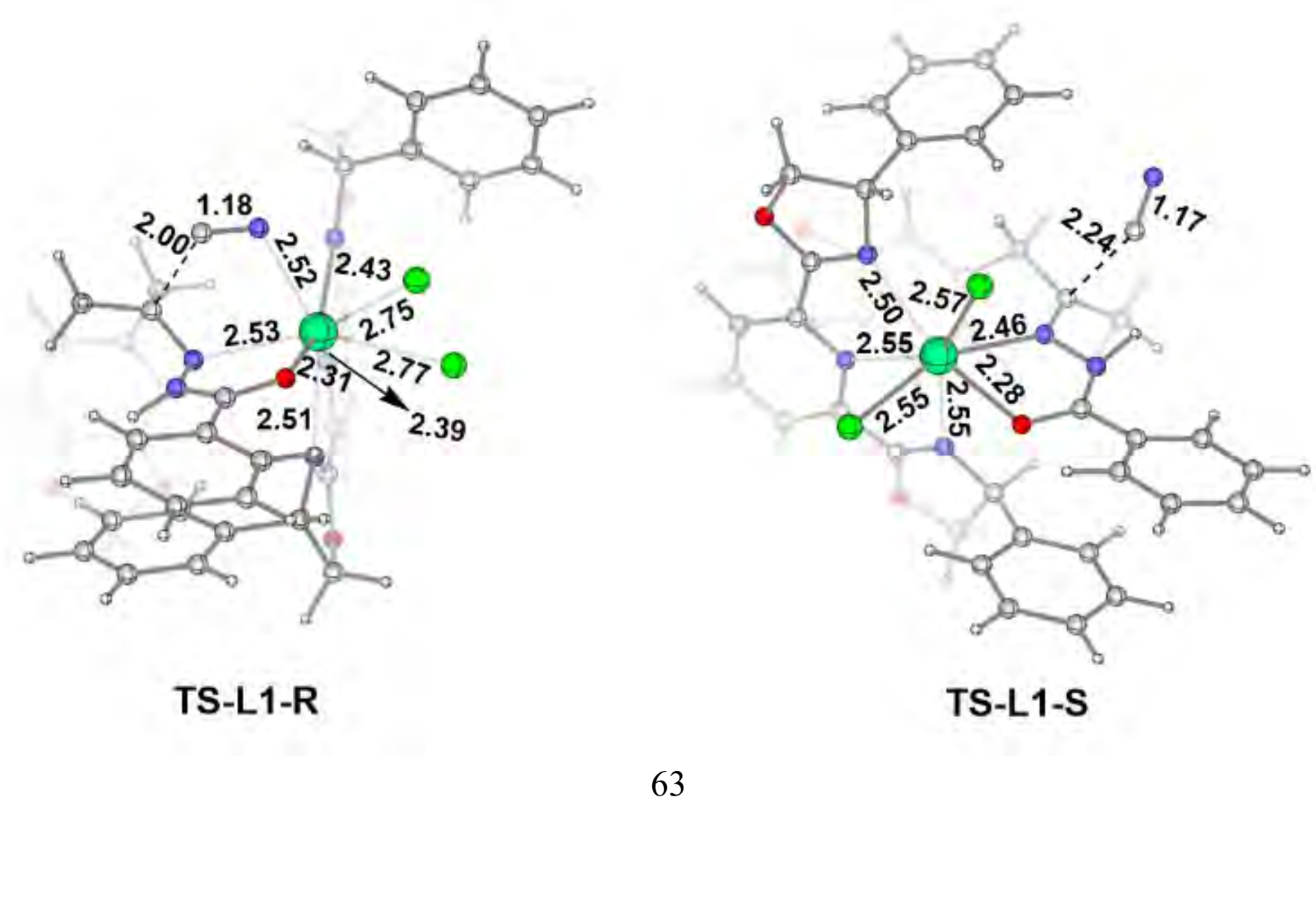

TS-L1-R TS-L1-S

9. Insecticidal activity studies

Based on the different modifiable sites of α-hydrazino nitrile scaffold, we investigate the influence of substituent effects on insecticidal activity (**Figure S11**).

Figure S11. Insecticidal activity against *A. gossypii* of selected α-hydrazino nitriles

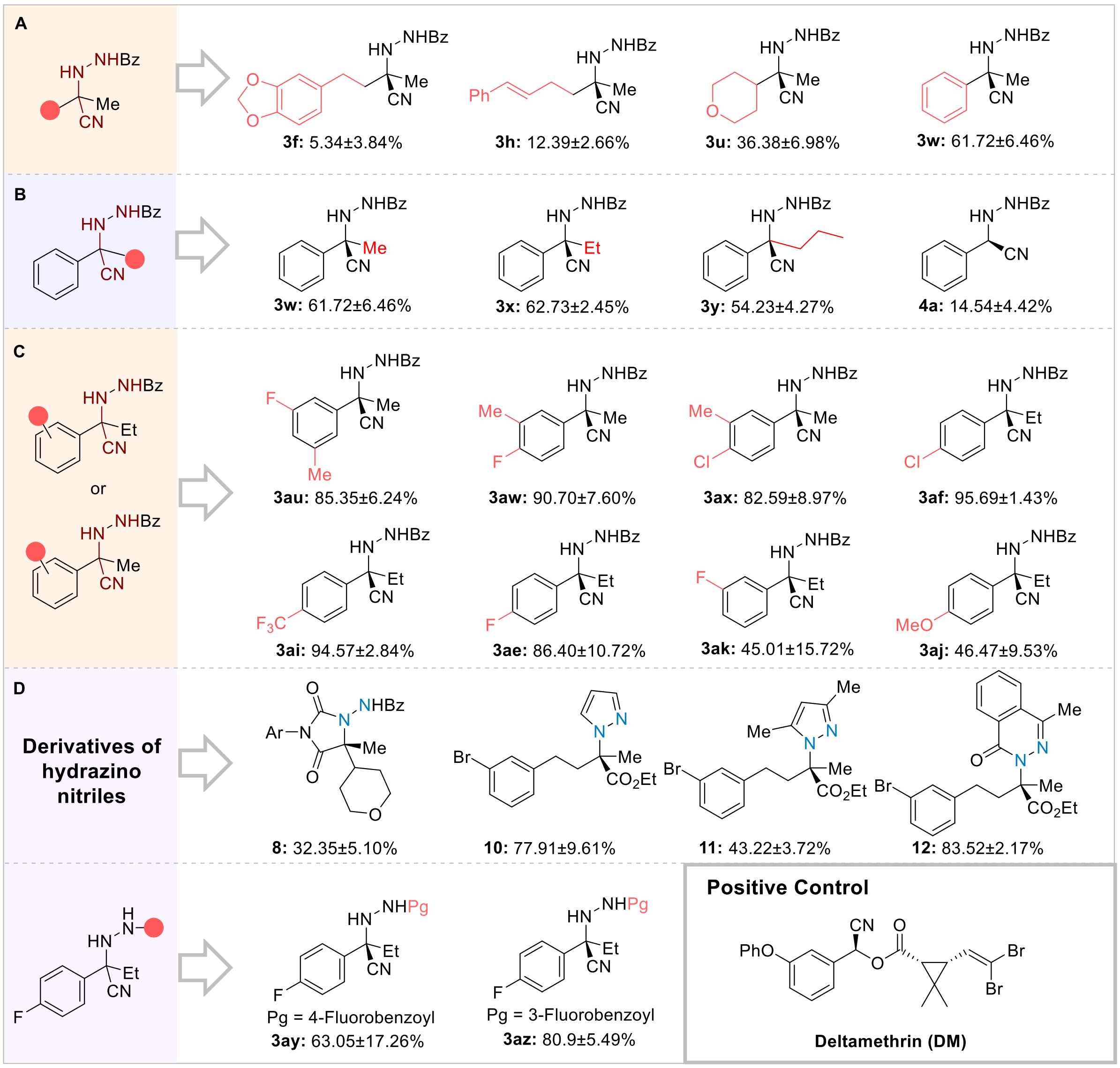

The activities of compounds **3f**, **3h**, **3u**, and **3r** were first evaluated. The results showed that the phenyl-substituted **3w** exhibited significantly higher insecticidal activity (61.72 ± 6.46%) than the alkyl-substituted analogs **3f**, **3h**, and **3u** (**Figure S11A**). Next, the phenyl group was retained, and a series alkyl substituents was investigated. As shown in **Figure S11B**, the methyl-substituted derivative **3w**

and the ethyl-substituted derivative **3x** displayed comparable moderate insecticidal activities, whereas the hydrogen-substituted analog **4a** showed a marked reduction in activity. These findings suggest that the tetra-substituted carbon center contributes positively to enhancing insecticidal activity. The insecticidal activities of various aryl-substituted derivatives (**3au**, **3aw**, **3ax**, **3af**, **3ai**, **3ae**, **3ak**, and **3aj**) were further evaluated. In most cases, the introduction of electron-withdrawing groups (F, Cl, or CF_3) increased the activity to >80% (**Figure S11C**). Finally, insecticidal activity was assessed for the hydrazino nitrile scaffold bearing different protecting groups and its derivatives, and compound **12** exhibited activity >80% (**Figure S11D**). The significant difference analysis of insecticidal activity was calculated by using t tests of GraphPad Prism (**Table S3**).

Table S3. Significant difference analysis of insecticidal activity

Comparisons	Summary	P Value	Comparisons	Summary	P Value
DM vs. 3f	****	<0.0001	DM vs. 3ai	ns	0.5802
DM vs. 3h	****	<0.0001	DM vs. 3ae	ns	0.3189
DM vs. 3u	****	<0.0001	DM vs. 3ak	**	0.0060
DM vs. 3w	**	0.0011	DM vs. 3aj	**	0.0011
DM vs. 3x	****	<0.0001	DM vs. 3ay	*	0.0381
DM vs. 3y	***	0.0001	DM vs. 3az	*	0.0182
DM vs. 4a	****	<0.0001	DM vs. 8	****	<0.0001
DM vs. 3au	ns	0.0914	DM vs. 10	*	0.0497
DM vs. 3aw	ns	0.5648	DM vs. 11	****	<0.0001
DM vs. 3ax	ns	0.1056	DM vs. 12	**	0.0024
DM vs. 3af	ns	0.1168			

10. Computational analysis of insecticidal mechanism

Molecular docking of **3af** and ES into the Aphis gossypii VGSC model was performed using the Glide module in Schrödinger in Standard Precision (SP) mode. The Aphis gossypii VGSC structure was predicted by AlphaFold3. The membrane-embedded orientation of the channel was assigned with reference to the OPM entry for the Periplaneta americana VGSC crystal structure (PDB: 5X0M). Prior to docking, the protein structure was prepared using the Protein Preparation Wizard, in which hydrogen atoms were added, the hydrogen-bonding network was optimized, and restrained energy minimization

was performed. The ligands **3af** and ES were prepared using LigPrep. Representative docking poses were selected for subsequent MD simulations.

MD simulations were carried out using the Desmond module in Schrödinger with the OPLS4 force field. The protein-ligand complexes were embedded in a membrane environment, solvated with the SPC water model, and neutralized with appropriate counterions. NaCl was added to a final concentration of 0.15 M. Following energy minimization, equilibration was performed under NVT and NPT ensembles. Production runs were then conducted for 50 ns at 300 K and 1 atm. The stability of the complexes and the key ligand-protein interactions throughout the simulations were evaluated using the Simulation Interaction Diagram module.

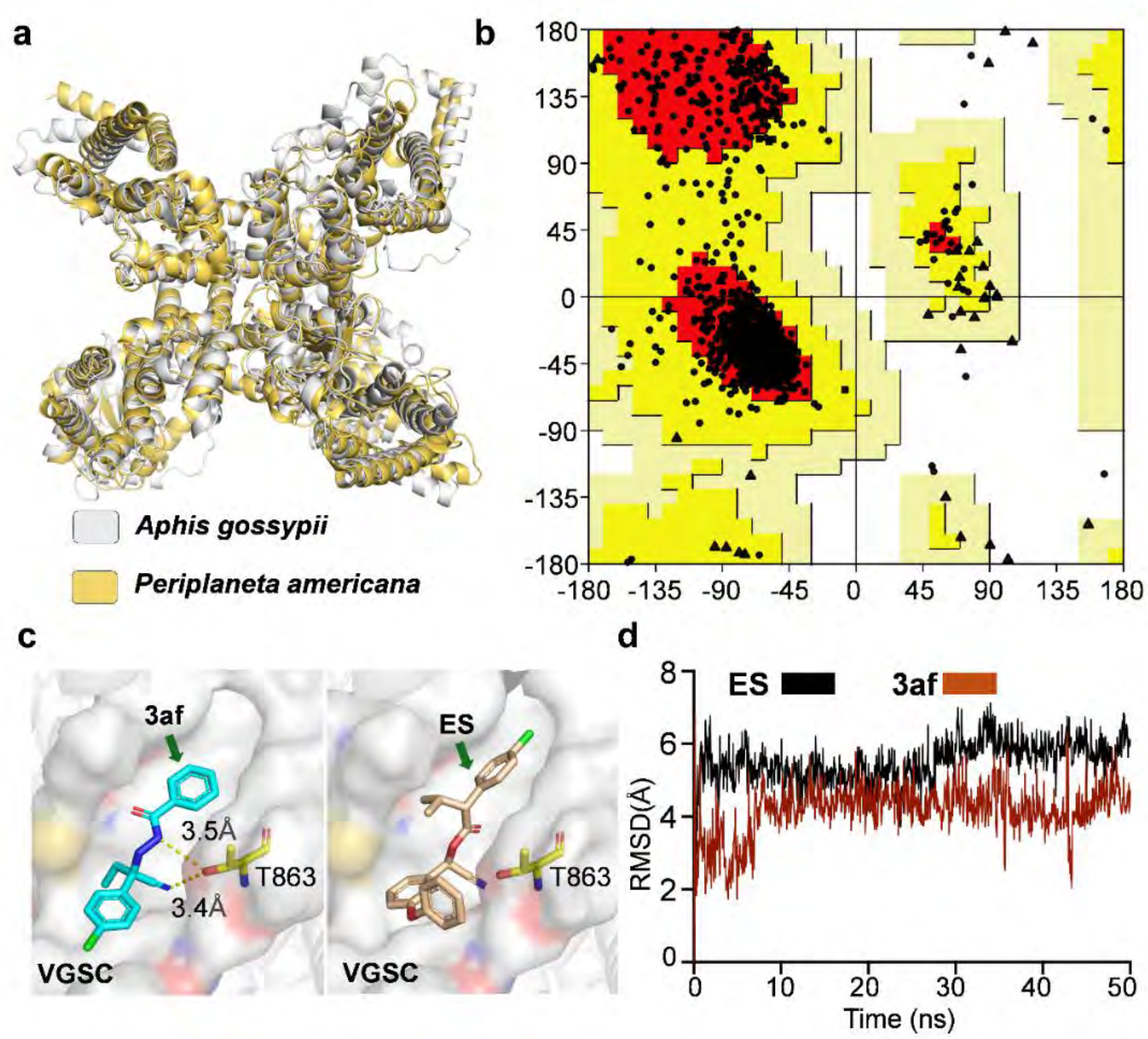

Figure S12. Structural modelling, validation and molecular dynamics analysis of the 3af-VGSC complex. a, The AlphaFold3-predicted Aphis gossypii VGSC model was superposed with the Periplaneta americana VGSC crystal structure (PDB: 5X0M), with an RMSD of 2.03 Å. **b**, Ramachandran plot of the Aphis gossypii VGSC model, showing 90.6% of residues in the most favoured regions, 8.4% in additional allowed regions, 0.5% in generously allowed regions and 0.5% in

disallowed regions. **c**, Binding modes of **3af** and ES in the VGSC binding pocket. In the **3af**-bound model, the cyano group of **3af** engages in hydrogen-bonding interactions with T863, indicated by yellow dashed lines. **d**, RMSD trajectories of **3af** and ES during 50 ns molecular dynamics simulations.

11. X-ray crystallographic data of *E*-1b, (*R*)-3e, (*R*)-3w, Er(OTf)3/L1 and Er(OTf)3/L6

Data intensity of (*E*)-**1b**[16] was collected using a 'Bruker APEX-II CCD' diffractometer at 173(10) K. Data collection and reduction were done by using Olex2 and the structure was solved with the ShelXS structure solution program using direct methods and refined by full-matrix least-squares on F^2 with anisotropic displacement parameters for non-H atoms using SHELX-97. Hydrogen atoms were added at their geometrically idea positions and refined isotropically. Crystal data for (*E*)-**1b**: $C_{19}H_{20}N_2O_3$ (M = 324.37 g/mol): orthorhombic, space group Pbca, a = 11.5357(2) Å, b = 9.13520(10) Å, c = 32.2862(5) Å, β = 90°, V = 3402.35(9) Å^3, Z = 8, T = 173(10) K, μ(CuKα) = 1.54184 mm^{-1}, 78016 reflections measured (9.4 ° ≤ 2Θ ≤ 134.1°), 3034 unique (R_{int} = 0.0602, R_{sigma} = 0.0179) which were used in all calculations. The final R_1 was 0.0343 (I > 2σ(I)) and wR_2 was 0.868 (all data). The crystal was grown in *n*-hexane/CH_2Cl_2 system.

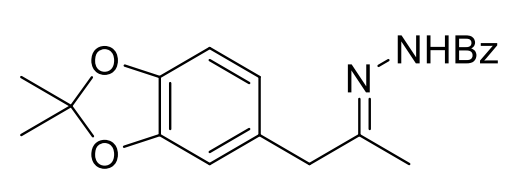

(*E*)-**1b**

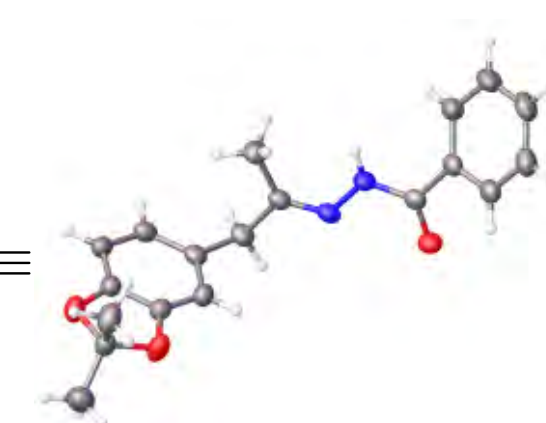

(CCDC: 2221754)

Table S4 Crystal data and structure refinement for (*E*)-**1b.**

Identification code	(*E*)-**1b**
Empirical formula	$C_{19}H_{20}N_2O_3$
Formula weight	324.37
Temperature/K	173.00(10)
Crystal system	orthorhombic
Space group	Pbca
a/Å	11.5357(2)
b/Å	9.13520(10)

c/Å	32.2862(5)
α/°	90
β/°	90
γ/°	90
Volume/Å^3	3402.35(9)
Z	8
ρ_{calc}g/cm^3	1.266
μ/mm^{-1}	0.701
F(000)	1376.0
Crystal size/mm^3	0.32 × 0.26 × 0.18
Radiation	CuKα (λ = 1.54184)
2Θ range for data collection/°	9.422 to 134.15
Index ranges	-13 ≤ h ≤ 13, -10 ≤ k ≤ 10, -38 ≤ l ≤ 38
Reflections collected	78016
Independent reflections	3034 [R_{int} = 0.0602, R_{sigma} = 0.0179]
Data/restraints/parameters	3034/0/221
Goodness-of-fit on F^2	1.057
Final R indexes [I>=2σ (I)]	R_1 = 0.0343, wR_2 = 0.0846
Final R indexes [all data]	R_1 = 0.0378, wR_2 = 0.0868
Largest diff. peak/hole / e Å^{-3}	0.17/-0.19

Table S5 Fractional Atomic Coordinates (×10^4) and Equivalent Isotropic Displacement Parameters (Å^2×10^3) for (*E*)-1b. U_{eq} is defined as 1/3 of of the trace of the orthogonalised U_{IJ} tensor.

Atom	*x*	*y*	*z*	U(eq)
O1	6312.0(8)	2034.8(12)	4095.3(3)	45.2(3)
O2	7671.4(8)	3238.1(12)	3690.4(3)	43.6(3)
O3	6437.9(8)	612.9(10)	6429.1(3)	37.4(2)
N1	7477.4(10)	2550.1(11)	6193.4(3)	32.6(3)

N2	7947.1(9)	1682.7(11)	5880.7(3)	32.5(3)
C1	6444.4(12)	2889.0(16)	3717.8(4)	37.8(3)
C2	8149.7(12)	2866.0(14)	4065.0(4)	33.6(3)
C3	9257.5(12)	3091.5(15)	4198.8(4)	37.0(3)
C4	9539.2(11)	2544.3(15)	4590.6(4)	36.3(3)
C5	8737.6(11)	1814.2(14)	4836.2(4)	32.8(3)
C6	7604.7(12)	1600.0(14)	4688.9(4)	34.8(3)
C7	7339.9(11)	2138.7(14)	4304.6(4)	33.7(3)
C8	5780.3(15)	4285.3(17)	3759.5(5)	51.7(4)
C9	6118.6(17)	1944(2)	3356.9(5)	60.9(5)
C10	9052.9(12)	1328.8(14)	5270.4(4)	35.4(3)
C11	8535.7(11)	2316.7(14)	5597.6(4)	31.1(3)
C12	8787.3(13)	3914.1(14)	5563.6(4)	41.0(3)
C13	6724.4(11)	1906.3(13)	6457.7(4)	30.7(3)
C14	6207.4(12)	2854.3(14)	6787.2(4)	33.0(3)
C15	5062.9(12)	2582.7(15)	6898.5(4)	37.0(3)
C16	4529.2(14)	3418.7(17)	7200.9(4)	45.3(4)
C17	5136.9(16)	4513.4(18)	7397.6(4)	54.2(4)
C18	6276.5(17)	4774.0(18)	7293.3(5)	57.1(4)
C19	6820.4(15)	3952.8(16)	6988.5(4)	44.8(4)

Table S6 Anisotropic Displacement Parameters ($Å^2 \times 10^3$) for (*E*)-1b. The Anisotropic displacement factor exponent takes the form: $-2\pi^2[h^2a^{*2}U_{11}+2hka^*b^*U_{12}+\cdots]$.

Atom	U_{11}	U_{22}	U_{33}	U_{23}	U_{13}	U_{12}
O1	35.2(5)	55.3(6)	45.0(6)	18.5(5)	-5.2(4)	-10.5(4)
O2	40.0(5)	55.3(6)	35.5(5)	10.4(4)	0.7(4)	-8.4(5)
O3	45.2(6)	27.7(5)	39.2(5)	-1.2(4)	-2.5(4)	-1.1(4)
N1	40.2(6)	26.1(5)	31.5(5)	-5.8(4)	1.2(5)	-0.5(4)
N2	36.7(6)	29.8(5)	31.0(5)	-7.1(4)	-2.6(4)	3.1(4)

C1	37.1(7)	41.4(8)	35.1(7)	7.0(6)	-0.4(5)	-6.6(6)
C2	38.4(7)	32.2(7)	30.3(6)	-0.8(5)	3.2(5)	-3.3(5)
C3	36.7(7)	38.5(7)	35.8(7)	-5.1(6)	7.2(6)	-8.1(6)
C4	33.9(7)	37.8(7)	37.2(7)	-9.4(5)	0.8(5)	-2.9(6)
C5	37.5(7)	28.8(6)	32.2(6)	-6.3(5)	1.8(5)	3.3(5)
C6	36.2(7)	33.4(7)	34.9(7)	2.5(5)	5.0(5)	-1.4(5)
C7	32.1(7)	32.6(7)	36.5(7)	0.8(5)	0.4(5)	-2.7(5)
C8	53.8(9)	47.2(9)	54.0(9)	13.1(7)	9.6(7)	2.3(7)
C9	69.5(12)	63.4(11)	49.9(9)	-9.4(8)	-2.2(8)	-15.4(9)
C10	40.0(7)	31.4(7)	34.9(7)	-4.7(5)	0.2(5)	6.5(5)
C11	32.3(6)	30.4(6)	30.5(6)	-3.9(5)	-5.0(5)	3.9(5)
C12	50.1(8)	31.3(7)	41.6(7)	-4.5(6)	7.4(6)	1.7(6)
C13	35.1(7)	28.9(7)	28.1(6)	0.7(5)	-7.2(5)	1.7(5)
C14	45.8(8)	28.9(6)	24.3(6)	3.6(5)	-2.0(5)	1.6(5)
C15	46.4(8)	35.7(7)	29.0(6)	3.5(5)	-0.8(6)	0.8(6)
C16	54.9(9)	46.9(8)	34.1(7)	5.5(6)	8.7(6)	6.2(7)
C17	80.0(12)	47.3(9)	35.2(8)	-4.8(7)	14.9(8)	5.8(8)
C18	85.4(13)	45.8(9)	40.1(8)	-13.8(7)	7.9(8)	-13.6(9)
C19	57.2(9)	42.0(8)	35.2(7)	-4.8(6)	4.0(6)	-10.2(7)

Table S7 Bond Lengths for (*E*)-1b.

**Atom**	**Atom**	**Length/Å**	**Atom**	**Atom**	**Length/Å**
O1	C1	1.4552(15)	C4	C5	1.3888(18)
O1	C7	1.3681(16)	C5	C6	1.4043(19)
O2	C1	1.4536(17)	C5	C10	1.5147(18)
O2	C2	1.3722(15)	C6	C7	1.3696(18)
O3	C13	1.2304(15)	C10	C11	1.5120(17)
N1	N2	1.3933(14)	C11	C12	1.4918(18)
N1	C13	1.3522(16)	C13	C14	1.4956(17)

N2	C11	1.2774(17)	C14	C15	1.3906(19)
C1	C8	1.494(2)	C14	C19	1.3890(19)
C1	C9	1.498(2)	C15	C16	1.3843(19)
C2	C3	1.3646(19)	C16	C17	1.377(2)
C2	C7	1.3829(18)	C17	C18	1.378(3)
C3	C4	1.3984(19)	C18	C19	1.387(2)

Table S8 Bond Angles for (*E*)-1b.

Atom	Atom	Atom	Angle/°	Atom	Atom	Atom	Angle/°
C7	O1	C1	106.59(10)	O1	C7	C2	110.03(11)
C2	O2	C1	106.47(10)	O1	C7	C6	128.02(12)
C13	N1	N2	117.38(10)	C6	C7	C2	121.92(12)
C11	N2	N1	117.86(10)	C11	C10	C5	112.16(10)
O1	C1	C8	109.18(11)	N2	C11	C10	116.04(11)
O1	C1	C9	108.42(12)	N2	C11	C12	126.83(12)
O2	C1	O1	105.71(10)	C12	C11	C10	117.11(11)
O2	C1	C8	108.51(12)	O3	C13	N1	122.87(11)
O2	C1	C9	108.87(12)	O3	C13	C14	120.16(12)
C8	C1	C9	115.69(14)	N1	C13	C14	116.94(11)
O2	C2	C7	109.90(11)	C15	C14	C13	117.33(12)
C3	C2	O2	128.18(12)	C19	C14	C13	123.23(13)
C3	C2	C7	121.88(12)	C19	C14	C15	119.43(13)
C2	C3	C4	116.74(12)	C16	C15	C14	120.41(13)
C5	C4	C3	122.24(12)	C17	C16	C15	119.98(15)
C4	C5	C6	119.55(12)	C16	C17	C18	119.91(14)
C4	C5	C10	120.61(12)	C17	C18	C19	120.76(15)
C6	C5	C10	119.74(12)	C18	C19	C14	119.50(15)
C7	C6	C5	117.66(12)				

Table S9 Hydrogen Atom Coordinates (Å×10^4) and Isotropic Displacement Parameters (Å^2× 10^3) for (*E*)-1b.

Atom	*x*	*y*	*z*	U(eq)
H1	7659.13	3459.13	6217.44	39
H3	9798.72	3584.98	4037.26	44
H4	10288.83	2673.75	4690.04	44
H6	7053.7	1109.59	4846.91	42
H8A	4965.7	4073.76	3770.45	77
H8B	5938.4	4902.76	3525.59	77
H8C	6010.03	4776.69	4009.15	77
H9A	6598.61	1085.48	3354.12	91
H9B	6228.11	2480.62	3104.29	91
H9C	5320.21	1659.77	3381.04	91
H10A	8778.86	336.71	5313.66	43
H10B	9889.96	1324.74	5299.06	43
H12A	8076.15	4455.83	5582.68	62
H12B	9151.31	4111.38	5302.15	62
H12C	9296.18	4203	5784.25	62
H15	4653.56	1834.73	6768.88	44
H16	3760.15	3240.87	7271.26	54
H17	4778.75	5076.33	7600.68	65
H18	6685.92	5508.88	7428.74	68
H19	7589.44	4136.39	6919.43	54

Data intensity of (*R*)-**3e**[17] was collected using a 'Bruker APEX-II CCD' diffractometer at 174(4) K. Data collection and reduction were done by using Olex2 and the structure was solved with the ShelXS structure solution program using direct methods and refined by full-matrix least-squares on F^2 with anisotropic displacement parameters for non-H atoms using SHELX-97. Hydrogen atoms were added at their geometrically idea positions and refined isotropically. The crystal was grown in *n*-hexane/CH_2Cl_2 system.

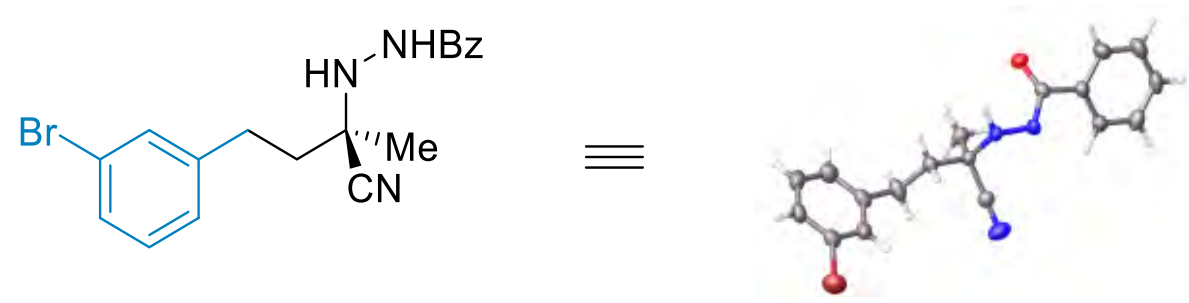

(*R*)-**3e** (CCDC: 2422479)

Table S10 Crystal data and structure refinement for (*R*)-3e.

Identification code	(*R*)-**3e**
Empirical formula	$C_{18}H_{17}BrN_3O$
Formula weight	371.25
Temperature/K	174(4)
Crystal system	orthorhombic
Space group	$P2_12_12_1$
a/Å	6.4606(2)
b/Å	8.0930(3)
c/Å	33.0294(11)
α/°	90
β/°	90
γ/°	90
Volume/Å^3	1726.96(10)
Z	4
ρ_{calc}g/cm^3	1.428
μ/mm^{-1}	3.301
F(000)	756.0
Crystal size/mm^3	0.36 × 0.12 × 0.11

Radiation	Cu Kα (λ = 1.54184)
2Θ range for data collection/°	10.714 to 134.104
Index ranges	$-7 \le h \le 7$, $-9 \le k \le 9$, $-39 \le l \le 39$
Reflections collected	36872
Independent reflections	3095 [R_{int} = 0.1403, R_{sigma} = 0.0489]
Data/restraints/parameters	3095/0/209
Goodness-of-fit on F^2	0.953
Final R indexes [I>=2σ (I)]	R_1 = 0.0407, wR_2 = 0.1080
Final R indexes [all data]	R_1 = 0.0450, wR_2 = 0.1125
Largest diff. peak/hole / e $Å^{-3}$	0.38/-0.59
Flack parameter	-0.02(2)

Table S11 Fractional Atomic Coordinates (×10^4) and Equivalent Isotropic Displacement Parameters ($Å^2$×10^3) for (*R*)-3e. U_{eq} is defined as 1/3 of the trace of the orthogonalised U_{IJ} tensor.

**Atom**	***x***	***y***	***z***	**U(eq)**
Br(1)	4963.0(10)	6837.8(7)	7550.0(2)	55.4(2)
O(1)	1357(5)	5996(4)	4477.9(10)	34.4(8)
N(1)	3592(5)	6634(5)	5178.9(10)	28.2(8)
N(2)	4458(5)	6528(5)	4782.3(9)	29.7(8)
N(3)	7984(7)	4657(6)	5499.9(14)	49.7(11)
C(1)	2593(8)	4409(6)	6548.9(14)	37.7(11)
C(2)	3880(8)	5265(6)	6810.9(13)	37.1(11)
C(3)	3180(8)	5698(6)	7192.9(14)	39.8(11)
C(4)	1183(8)	5324(7)	7317.2(14)	43.5(12)
C(5)	-95(9)	4453(6)	7056.5(14)	46.8(12)
C(6)	609(9)	4004(7)	6677.0(16)	45.9(14)
C(7)	3342(9)	3966(6)	6129.9(13)	42.4(12)
C(8)	3076(7)	5420(6)	5837.9(12)	31.0(9)

Table S11 Fractional Atomic Coordinates (×10^4) and Equivalent Isotropic Displacement Parameters (Å^2 ×10^3) for (*R*)-3e. U_{eq} is defined as 1/3 of the trace of the orthogonalised U_{IJ} tensor.

Atom	*x*	*y*	*z*	U(eq)
$C^{(9)}$	3983(7)	5103(6)	5414.5(13)	27.4(10)
$C^{(10)}$	6255(8)	4843(6)	5458.8(14)	32.8(11)
$C^{(11)}$	3041(7)	3619(6)	5207.5(13)	34.2(10)
$C^{(12)}$	3235(7)	6210(6)	4459.6(13)	29.4(10)
$C^{(13)}$	4397(7)	6078(6)	4067.6(13)	30.1(10)
$C^{(14)}$	3354(8)	6519(6)	3715.2(13)	39.2(11)
$C^{(15)}$	4327(9)	6411(7)	3346.0(15)	46.7(14)
$C^{(16)}$	6333(9)	5831(7)	3321.8(15)	45.3(13)
$C^{(17)}$	7375(8)	5372(7)	3667.5(15)	40.8(12)
$C^{(18)}$	6407(7)	5493(6)	4043.6(13)	32.5(10)

Table S12 Anisotropic Displacement Parameters (Å^2 ×10^3) for (*R*)-3e. The Anisotropic displacement factor exponent takes the form: $-2\pi^2[h^2a^{*2}U_{11}+2hka^*b^*U_{12}+\cdots]$.

Atom	U_{11}	U_{22}	U_{33}	U_{23}	U_{13}	U_{12}
$Br^{(1)}$	58.4(4)	56.4(4)	51.3(3)	-15.2(2)	-11.7(3)	3.1(3)
$O^{(1)}$	25.9(16)	44(2)	33.5(16)	-7.1(14)	-1.2(13)	2.1(14)
$N^{(1)}$	23.8(17)	37(2)	23.5(17)	-1.8(15)	4.6(13)	1.2(17)
$N^{(2)}$	30.8(19)	39(2)	19.5(16)	-4.4(14)	0.6(13)	-0.7(15)
$N^{(3)}$	32(2)	52(3)	65(3)	-8(2)	-3(2)	5(2)
$C^{(1)}$	53(3)	32(3)	28(2)	1.9(19)	-1(2)	7(2)
$C^{(2)}$	39(3)	39(3)	33(2)	6(2)	3(2)	3(2)
$C^{(3)}$	48(3)	40(3)	32(2)	4(2)	-1(2)	3(2)
$C^{(4)}$	49(3)	51(3)	30(2)	7(2)	10(2)	5(3)
$C^{(5)}$	44(3)	50(3)	46(3)	8(2)	11(3)	-1(3)

Table S12 Anisotropic Displacement Parameters (Å^2 × 10^3) for (*R*)-3e. The Anisotropic displacement factor exponent takes the form: -2 π ^{2}[h^{2}a*^{2}U$_{11}$+2hka*b*U$_{12}$+⋯].

Atom	U11	U22	U33	U23	U13	U12
C(6)	52(3)	44(3)	41(3)	0(2)	-3(2)	-5(2)
C(7)	63(3)	35(3)	29(2)	-0.4(19)	6(2)	5(2)
C(8)	35(2)	33(3)	25(2)	-3.5(18)	2.2(18)	2(2)
C(9)	27(2)	31(3)	24(2)	-5.3(18)	-0.2(17)	-0.1(18)
C(10)	33(3)	32(3)	33(2)	-5(2)	0(2)	-1(2)
C(11)	36(2)	31(2)	36(2)	-6.8(19)	2.5(19)	-2(2)
C(12)	34(2)	30(2)	24(2)	-4.1(17)	-0.4(18)	3.6(18)
C(13)	34(2)	29(2)	27(2)	-1.6(18)	2.9(17)	-0.8(17)
C(14)	41(3)	47(3)	30(2)	-3(2)	-5(2)	2(2)
C(15)	61(3)	53(3)	26(2)	2(2)	1(2)	4(3)
C(16)	61(3)	44(3)	31(3)	0(2)	15(3)	-8(3)
C(17)	39(3)	40(3)	43(3)	-8(2)	12(2)	-4(2)
C(18)	36(2)	32(3)	30(2)	-3(2)	1.1(19)	-3(2)

Table S13 Bond Lengths for (*R*)-3e.

Atom	Atom	Length/Å	Atom	Atom	Length/Å
Br(1)	C(3)	1.889(5)	C(5)	C(6)	1.382(7)
O(1)	C(12)	1.227(6)	C(7)	C(8)	1.531(6)
N(1)	N(2)	1.427(4)	C(8)	C(9)	1.538(6)
N(1)	C(9)	1.484(6)	C(9)	C(10)	1.490(6)
N(2)	C(12)	1.352(5)	C(9)	C(11)	1.511(6)
N(3)	C(10)	1.135(6)	C(12)	C(13)	1.500(6)
C(1)	C(2)	1.386(7)	C(13)	C(14)	1.392(6)
C(1)	C(6)	1.389(7)	C(13)	C(18)	1.384(6)
C(1)	C(7)	1.509(6)	C(14)	C(15)	1.375(7)
C(2)	C(3)	1.385(6)	C(15)	C(16)	1.381(8)

Table S13 Bond Lengths for (*R*)-3e.

Atom	Atom	Length/Å	Atom	Atom	Length/Å
$C^{(3)}$	$C^{(4)}$	1.388(7)	$C^{(16)}$	$C^{(17)}$	1.376(7)
$C^{(4)}$	$C^{(5)}$	1.386(7)	$C^{(17)}$	$C^{(18)}$	1.394(6)

Table S14 Bond Angles for(*R*)-3e.

Atom	Atom	Atom	Angle/°	Atom	Atom	Atom	Angle/°
$N^{(2)}$	$N^{(1)}$	$C^{(9)}$	111.4(3)	$N^{(1)}$	$C^{(9)}$	$C^{(11)}$	111.0(3)
$C^{(12)}$	$N^{(2)}$	$N^{(1)}$	120.4(3)	$C^{(10)}$	$C^{(9)}$	$C^{(8)}$	108.0(4)
$C^{(2)}$	$C^{(1)}$	$C^{(6)}$	118.8(5)	$C^{(10)}$	$C^{(9)}$	$C^{(11)}$	109.2(4)
$C^{(2)}$	$C^{(1)}$	$C^{(7)}$	119.9(5)	$C^{(11)}$	$C^{(9)}$	$C^{(8)}$	113.0(4)
$C^{(6)}$	$C^{(1)}$	$C^{(7)}$	121.3(5)	$N^{(3)}$	$C^{(10)}$	$C^{(9)}$	178.7(6)
$C^{(1)}$	$C^{(2)}$	$C^{(3)}$	120.0(5)	$O^{(1)}$	$C^{(12)}$	$N^{(2)}$	124.5(4)
$C^{(2)}$	$C^{(3)}$	$Br^{(1)}$	119.6(4)	$O^{(1)}$	$C^{(12)}$	$C^{(13)}$	121.8(4)
$C^{(2)}$	$C^{(3)}$	$C^{(4)}$	121.2(5)	$N^{(2)}$	$C^{(12)}$	$C^{(13)}$	113.7(4)
$C^{(4)}$	$C^{(3)}$	$Br^{(1)}$	119.3(4)	$C^{(14)}$	$C^{(13)}$	$C^{(12)}$	117.5(4)
$C^{(5)}$	$C^{(4)}$	$C^{(3)}$	118.8(5)	$C^{(18)}$	$C^{(13)}$	$C^{(12)}$	122.9(4)
$C^{(6)}$	$C^{(5)}$	$C^{(4)}$	120.1(5)	$C^{(18)}$	$C^{(13)}$	$C^{(14)}$	119.6(4)
$C^{(5)}$	$C^{(6)}$	$C^{(1)}$	121.2(5)	$C^{(15)}$	$C^{(14)}$	$C^{(13)}$	120.3(5)
$C^{(1)}$	$C^{(7)}$	$C^{(8)}$	111.0(4)	$C^{(14)}$	$C^{(15)}$	$C^{(16)}$	120.1(5)
$C^{(7)}$	$C^{(8)}$	$C^{(9)}$	113.7(4)	$C^{(17)}$	$C^{(16)}$	$C^{(15)}$	120.2(5)
$N^{(1)}$	$C^{(9)}$	$C^{(8)}$	105.8(3)	$C^{(16)}$	$C^{(17)}$	$C^{(18)}$	120.1(5)
$N^{(1)}$	$C^{(9)}$	$C^{(10)}$	109.7(4)	$C^{(13)}$	$C^{(18)}$	$C^{(17)}$	119.7(5)

Table S15 Torsion Angles for (*R*)-3e.

A	B	C	D	Angle/°	A	B	C	D	Angle/°
$Br^{(1)}$	$C^{(3)}$	$C^{(4)}$	$C^{(5)}$	-178.5(4)	$C^{(4)}$	$C^{(5)}$	$C^{(6)}$	$C^{(1)}$	0.0(8)
$O^{(1)}$	$C^{(12)}$	$C^{(13)}$	$C^{(14)}$	31.3(7)	$C^{(6)}$	$C^{(1)}$	$C^{(2)}$	$C^{(3)}$	0.1(7)
$O^{(1)}$	$C^{(12)}$	$C^{(13)}$	$C^{(18)}$	-146.6(5)	$C^{(6)}$	$C^{(1)}$	$C^{(7)}$	$C^{(8)}$	96.0(6)

Table S15 Torsion Angles for (*R*)-3e.

A	B	C	D	Angle/°	A	B	C	D	Angle/°
$N^{(1)}$	$N^{(2)}$	$C^{(12)}$	$O^{(1)}$	0.1(7)	$C^{(7)}$	$C^{(1)}$	$C^{(2)}$	$C^{(3)}$	178.8(4)
$N^{(1)}$	$N^{(2)}$	$C^{(12)}$	$C^{(13)}$	-177.9(4)	$C^{(7)}$	$C^{(1)}$	$C^{(6)}$	$C^{(5)}$	-178.1(5)
$N^{(2)}$	$N^{(1)}$	$C^{(9)}$	$C^{(8)}$	177.1(3)	$C^{(7)}$	$C^{(8)}$	$C^{(9)}$	$N^{(1)}$	-179.7(4)
$N^{(2)}$	$N^{(1)}$	$C^{(9)}$	$C^{(10)}$	60.8(5)	$C^{(7)}$	$C^{(8)}$	$C^{(9)}$	$C^{(10)}$	-62.2(5)
$N^{(2)}$	$N^{(1)}$	$C^{(9)}$	$C^{(11)}$	-60.0(4)	$C^{(7)}$	$C^{(8)}$	$C^{(9)}$	$C^{(11)}$	58.7(5)
$N^{(2)}$	$C^{(12)}$	$C^{(13)}$	$C^{(14)}$	-150.7(4)	$C^{(9)}$	$N^{(1)}$	$N^{(2)}$	$C^{(12)}$	102.2(4)
$N^{(2)}$	$C^{(12)}$	$C^{(13)}$	$C^{(18)}$	31.4(6)	$C^{(12)}$	$C^{(13)}$	$C^{(14)}$	$C^{(15)}$	-179.4(5)
$C^{(1)}$	$C^{(2)}$	$C^{(3)}$	$Br^{(1)}$	179.1(4)	$C^{(12)}$	$C^{(13)}$	$C^{(18)}$	$C^{(17)}$	178.6(4)
$C^{(1)}$	$C^{(2)}$	$C^{(3)}$	$C^{(4)}$	-1.5(7)	$C^{(13)}$	$C^{(14)}$	$C^{(15)}$	$C^{(16)}$	1.4(8)
$C^{(1)}$	$C^{(7)}$	$C^{(8)}$	$C^{(9)}$	175.0(4)	$C^{(14)}$	$C^{(13)}$	$C^{(18)}$	$C^{(17)}$	0.8(7)
$C^{(2)}$	$C^{(1)}$	$C^{(6)}$	$C^{(5)}$	0.7(8)	$C^{(14)}$	$C^{(15)}$	$C^{(16)}$	$C^{(17)}$	-0.6(8)
$C^{(2)}$	$C^{(1)}$	$C^{(7)}$	$C^{(8)}$	-82.7(6)	$C^{(15)}$	$C^{(16)}$	$C^{(17)}$	$C^{(18)}$	-0.1(8)
$C^{(2)}$	$C^{(3)}$	$C^{(4)}$	$C^{(5)}$	2.1(7)	$C^{(16)}$	$C^{(17)}$	$C^{(18)}$	$C^{(13)}$	0.0(7)
$C^{(3)}$	$C^{(4)}$	$C^{(5)}$	$C^{(6)}$	-1.4(8)	$C^{(18)}$	$C^{(13)}$	$C^{(14)}$	$C^{(15)}$	-1.5(8)

Table S16 Hydrogen Atom Coordinates (Å×10^4) and Isotropic Displacement Parameters (Å^2×10^3) for (*R*)-3e.

Atom	*x*	*y*	*z*	U(eq)
$H^{(1)}$	2284.34	6938.94	5179.63	34
$H^{(2)}$	5241.45	5554.33	6728.67	45
$H^{(4)}$	700.49	5658.52	7576.26	52
$H^{(5)}$	-1457.35	4164.72	7138.63	56
$H^{(6)}$	-279.73	3407.15	6500.59	55
$H^{(7A)}$	2551.56	3003.73	6028.18	51
$H^{(7B)}$	4821.53	3650.55	6142.37	51
$H^{(8A)}$	3754.34	6406.27	5955.54	37
$H^{(8B)}$	1581.94	5668.1	5810.64	37

Table S16 Hydrogen Atom Coordinates (Å×10^4) and Isotropic Displacement Parameters (Å^2×10^3) for (*R*)-3e.

Atom		*x*	*y*	*z*	U(eq)
H(11A)	1529.03	3719.24	5208.99	51	
H(11B)	3446.7	2611.68	5351.85	51	
H(11C)	3534.79	3561.93	4927.3	51	
H(14)	1963.33	6895.75	3729.89	47	
H(15)	3617.4	6735.9	3106.95	56	
H(16)	6995.96	5748.7	3065.92	54	
H(17)	8754	4971.79	3649.76	49	
H(18)	7124.32	5175.86	4282.37	39	

Data intensity of (*R*)-**3w**[18] was collected using a 'Bruker APEX-II CCD' diffractometer at 296(2) K. Data collection and reduction were done by using Olex2 and the structure was solved with the ShelXS structure solution program using direct methods and refined by full-matrix least-squares on F^2 with anisotropic displacement parameters for non-H atoms using SHELX-97. Hydrogen atoms were added at their geometrically idea positions and refined isotropically. The crystal was grown in *n*-hexane/CH_2Cl_2 system.

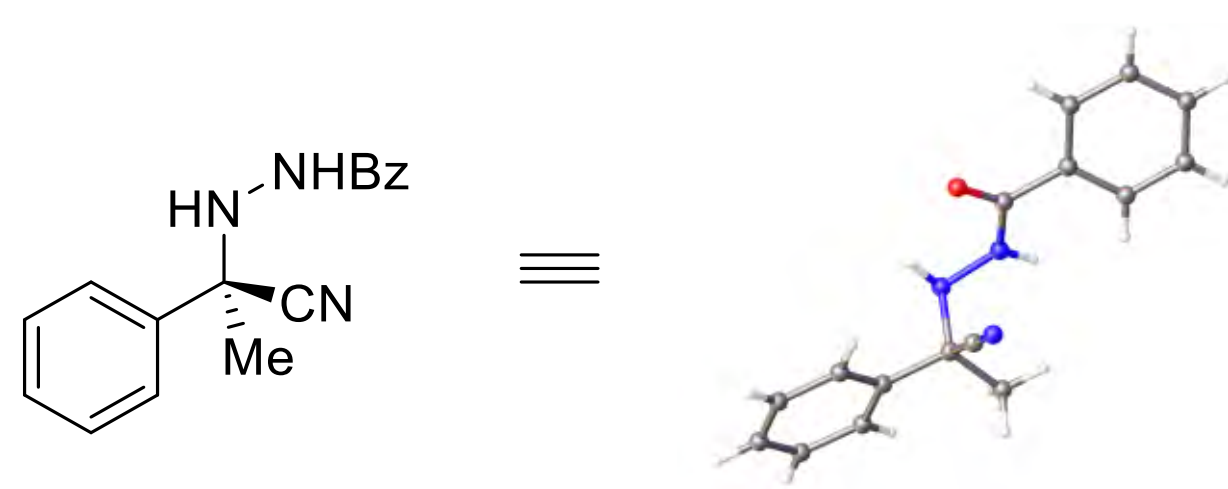

(*R*)-**3w** (CCDC: 2044037)

Table S17 Crystal data and structure refinement for (*R*)-3w.

Identification code	(*R*)-**3w**
Empirical formula	$C_{16}H_{15}N_3O$
Formula weight	265.31
Temperature/K	293(2)
Crystal system	monoclinic

Space group	$P2_1$
a/Å	6.58470(10)
b/Å	13.7845(2)
c/Å	8.58030(10)
α/°	90
β/°	109.693(2)
γ/°	90
Volume/Å^3	733.26(2)
Z	2
ρ_{calc}g/cm^3	1.202
μ/mm^{-1}	0.619
F(000)	280.0
Crystal size/mm^3	0.38 × 0.26 × 0.22
Radiation	CuKα (λ = 1.54184)
2Θ range for data collection/°	12.7 to 134.134
Index ranges	$-7 \leq h \leq 7$, $-16 \leq k \leq 16$, $-10 \leq l \leq 10$
Reflections collected	13640
Independent reflections	2582 [R_{int} = 0.0222, R_{sigma} = 0.0138]
Data/restraints/parameters	2582/1/187
Goodness-of-fit on F^2	1.060
Final R indexes [I>=2σ (I)]	R_1 = 0.0324, wR_2 = 0.0784
Final R indexes [all data]	R_1 = 0.0324, wR_2 = 0.0784
Largest diff. peak/hole / e Å^{-3}	0.39/-0.21
Flack parameter	0.35(5)

Table S18 Fractional Atomic Coordinates (×10^4) and Equivalent Isotropic Displacement Parameters (Å^2×10^3) for (*R*)-3w. U_{eq} is defined as 1/3 of of the trace of the orthogonalised U_{IJ} tensor.

Atom	*x*	*y*	*z*	U(eq)
O1	8741(3)	4605.9(13)	4121(2)	25.0(4)
N1	5156(3)	4433.0(14)	3505(2)	18.0(4)
N2	4745(3)	4753.1(14)	1862(2)	18.6(5)
N3	6572(4)	6877.1(18)	3931(3)	28.2(5)
C7	7194(4)	4380.7(17)	4558(3)	19.5(5)
C16	5583(4)	6516(2)	-307(3)	26.7(6)
C10	5402(4)	6407.5(18)	2921(3)	20.1(5)
C11	3786(4)	6104.1(17)	-75(3)	19.1(5)
C6	7464(4)	4057.8(17)	6279(3)	20.4(5)
C4	6135(5)	3866(2)	8556(3)	27.9(6)
C8	3897(4)	5766.4(17)	1656(3)	18.3(5)
C5	5811(4)	4143.9(19)	6939(3)	22.2(5)
C13	1876(5)	6277(2)	-2987(3)	29.6(6)
C9	1721(4)	5779.8(19)	1934(3)	23.9(6)
C1	9478(4)	3711.3(19)	7282(3)	26.2(6)
C12	1920(4)	5978.4(19)	-1433(3)	24.7(6)
C3	8123(5)	3515.2(19)	9536(3)	29.8(6)
C14	3659(5)	6705(2)	-3201(3)	29.4(6)
C2	9792(5)	3448(2)	8899(3)	30.6(6)
C15	5512(5)	6822(2)	-1867(3)	33.2(7)

Table S19 Anisotropic Displacement Parameters (Å^2×10^3) for (*R*)-3w. The Anisotropic displacement factor exponent takes the form: $-2\pi^2[h^2a^{*2}U_{11}+2hka^*b^*U_{12}+\ldots]$.

Atom	U_{11}	U_{22}	U_{33}	U_{23}	U_{13}	U_{12}
O1	21.1(9)	25.8(9)	30.1(10)	3.8(8)	11.0(8)	2.8(7)
N1	19.8(10)	19.1(10)	16.0(10)	2.6(8)	7.4(8)	-0.9(8)
N2	23.7(11)	17.4(10)	15.3(10)	2.1(8)	7.4(8)	1.9(8)
N3	35.0(13)	23.1(11)	23.6(11)	-1.8(10)	6.0(10)	-1.9(10)

C7	20.5(12)	16.0(12)	22.8(12)	0.3(10)	8.3(10)	1.2(9)
C16	26.2(13)	31.0(15)	20.9(13)	1.3(11)	5.2(10)	-5.0(11)
C10	25.6(13)	16.6(11)	18.9(12)	3.3(10)	8.6(10)	2.0(11)
C11	23.5(13)	16.0(12)	17.1(11)	1.2(9)	6.0(10)	1.6(10)
C6	25.4(13)	15.0(12)	18.3(12)	-3.8(10)	4.0(10)	-2.6(10)
C4	38.9(16)	24.5(14)	21.2(13)	-4.9(11)	11.6(12)	-5.8(12)
C8	18.1(12)	17.6(12)	17.9(12)	-0.2(10)	4.3(9)	-1.5(10)
C5	25.7(13)	20.4(12)	18.5(12)	-2.1(10)	5.0(10)	-3.0(10)
C13	34.0(15)	30.6(15)	18.2(13)	-0.7(12)	1.0(11)	5.2(12)
C9	24.7(13)	21.9(13)	27.8(13)	2.8(11)	12.6(11)	1.6(11)
C1	26.2(14)	22.6(13)	26.7(14)	0.8(11)	4.6(11)	2.4(11)
C12	23.0(13)	23.3(13)	24.7(13)	-0.7(11)	4.1(11)	-0.8(11)
C3	48.7(17)	20.8(14)	15.7(12)	-1.9(10)	5.4(11)	-2.7(12)
C14	48.9(18)	24.2(14)	15.2(12)	3.6(11)	10.7(12)	1.3(12)
C2	35.8(15)	22.2(13)	24.1(13)	2.7(11)	-2.8(11)	4.0(12)
C15	40.2(17)	35.3(15)	27.7(14)	3.4(13)	16.3(13)	-8.8(13)

Table S20 Bond Lengths for (*R*)-3w.

Atom	Atom	Length/Å	Atom	Atom	Length/Å
O1	C7	1.238(3)	C6	C5	1.391(4)
N1	N2	1.414(3)	C6	C1	1.399(4)
N1	C7	1.343(3)	C4	C5	1.385(4)
N2	C8	1.493(3)	C4	C3	1.383(4)
N3	C10	1.146(4)	C8	C9	1.530(3)
C7	C6	1.494(3)	C13	C12	1.386(4)
C16	C11	1.386(4)	C13	C14	1.381(4)
C16	C15	1.389(4)	C1	C2	1.381(4)
C10	C8	1.489(3)	C3	C2	1.385(4)
C11	C8	1.534(3)	C14	C15	1.372(4)
C11	C12	1.390(4)			

Table S21 Bond Angles for (*R*)-3w.

Atom	Atom	Atom	Angle/°	Atom	Atom	Atom	Angle/°
C7	N1	N2	120.0(2)	N2	C8	C11	106.99(19)
N1	N2	C8	110.38(18)	N2	C8	C9	109.00(19)
O1	C7	N1	121.3(2)	C10	C8	N2	109.79(19)
O1	C7	C6	122.5(2)	C10	C8	C11	109.2(2)
N1	C7	C6	116.1(2)	C10	C8	C9	107.9(2)
C11	C16	C15	120.6(3)	C9	C8	C11	113.9(2)
N3	C10	C8	177.8(3)	C4	C5	C6	120.5(2)
C16	C11	C8	119.9(2)	C14	C13	C12	120.7(3)
C16	C11	C12	119.1(2)	C2	C1	C6	119.9(3)
C12	C11	C8	120.9(2)	C13	C12	C11	119.7(3)
C5	C6	C7	121.9(2)	C4	C3	C2	119.9(2)
C5	C6	C1	119.2(2)	C15	C14	C13	119.8(2)
C1	C6	C7	118.8(2)	C1	C2	C3	120.6(3)
C3	C4	C5	120.0(3)	C14	C15	C16	120.0(3)

Table S22 Hydrogen Atom Coordinates (Å×10^4) and Isotropic Displacement Parameters (Å^2×10^3) for (*R*)-3w.

Atom	*x*	*y*	*z*	U(eq)
H1	4102.1	4271.89	3829.86	22
H16	6848.41	6848.41	6589.28	590.64
H4	5014.64	5014.64	3914.85	8983.14
H5	4479.77	4479.77	4389.89	6290.54
H13	630.35	630.35	6186.85	-3896.04
H9A	774.87	774.87	5311.35	1222.13
H9B	1092.13	1092.13	6414.12	1687.48
H9C	1929.44	1929.44	5622.27	3067.55
H1A	10602.42	10602.42	3658.44	6860.24
H12	707.32	707.32	5695.51	-1299.91
H3	8339.78	8339.78	3324.94	10620.07
H14	3604.31	3604.31	6913.89	-4244.6
H2A	11135.77	11135.77	3223.6	9566.82
H15	6719.62	6719.62	7105.87	-2007.44
H2	6140(50)	6140(50)	4770(20)	1740(40)

Data intensity of $Er(OTf)_3$/**L1**[19] was collected using a 'Bruker APEX-II CCD' diffractometer at 296(2) K. Data collection and reduction were done by using Olex2 and the structure was solved with the ShelXS structure solution program using direct methods and refined by full-matrix least-squares on F^2 with anisotropic displacement parameters for non-H atoms using SHELX-97. Hydrogen atoms were added at their geometrically idea positions and refined isotropically. The crystal was grown in CH_3CN/Et_2O system.

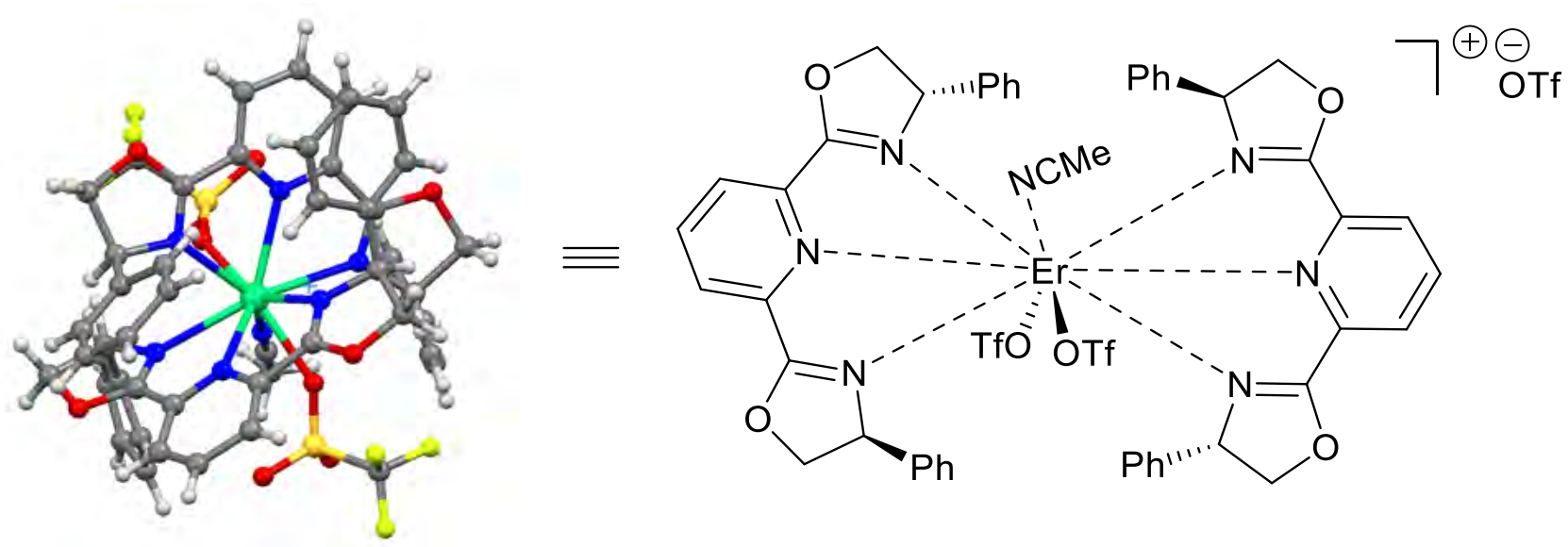

CCDC: 2390295

Table S23 Crystal data and structure refinement for $Er(OTf)_3$/L1.

Identification code	$Er(OTf)_3$/**L1**
Empirical formula	$C_{25.33}H_{20.5}Er_{0.5}F_4N_{3.5}O_6S_{1.33}$
Formula weight	672.31
Temperature/K	173.00(10)
Crystal system	trigonal
Space group	R32
a/Å	21.0624(13)
b/Å	21.0624(13)
c/Å	32.4402(18)
α/°	90
β/°	90
γ/°	120
Volume/Å^3	12463.2(17)
Z	18
ρ_{calc}g/cm^3	1.612
μ/mm^{-1}	4.576

F(000)	6054.0
Crystal size/mm^3	0.26 × 0.22 × 0.12
Radiation	Cu Kα (λ = 1.54184)
2Θ range for data collection/°	10.074 to 134.11
Index ranges	$-25 \le h \le 22$, $-19 \le k \le 25$, $-38 \le l \le 38$
Reflections collected	39981
Independent reflections	4974 [R_{int} = 0.1047, R_{sigma} = 0.0521]
Data/restraints/parameters	4974/112/417
Goodness-of-fit on F^2	1.019
Final R indexes [I>=2σ (I)]	R_1 = 0.0644, wR_2 = 0.1598
Final R indexes [all data]	R_1 = 0.0895, wR_2 = 0.1861
Largest diff. peak/hole / e Å^{-3}	0.92/-2.12
Flack parameter	-0.029(9)

Table S24 Fractional Atomic Coordinates (×10^4) and Equivalent Isotropic Displacement Parameters (Å^2×10^3) for Er(OTf)$_3$/L$_1$. U_{eq} is defined as 1/3 of the trace of the orthogonalised U_{IJ} tensor.

Atom	*x*	*y*	*z*	U(eq)
Er	7019.6(6)	6666.67	6666.67	61.3(3)
S(1)	5489(2)	5286(2)	7261.6(11)	72.4(10)
F(1)	5936(7)	4336(6)	7273(4)	125(4)
F(2)	4976(7)	4033(7)	6909(3)	117(4)
F(3)	4908(7)	3971(7)	7565(4)	129(4)
O(1)	6031(5)	5607(6)	6934(3)	76(3)
O(2)	5801(7)	5569(7)	7655(3)	92(4)
O(3)	4791(6)	5227(7)	7170(4)	97(4)
O(4)	7568(6)	4866(6)	6950(3)	74(3)
O(5)	7496(6)	7798(7)	7934(3)	86(3)
N(1)	7079(7)	7430(6)	7288(3)	70(3)
N(2)	7416(6)	6368(7)	7329(3)	58(2)
N(3)	7421(6)	5723(6)	6617(3)	59(3)
N(4)	5904(7)	6666.67	6666.67	55(3)
C(1)	5741(13)	7380(15)	7647(7)	134(10)
C(2)	5052(16)	7290(20)	7721(10)	187(15)
C(3)	4900(20)	7840(20)	7543(11)	177(13)

Table S24 Fractional Atomic Coordinates (×10^4) and Equivalent Isotropic Displacement Parameters (Å^2×10^3) for Er(OTf)$_3$/L$_1$. U_{eq} is defined as 1/3 of the trace of the orthogonalised U_{IJ} tensor.

**Atom**		***x***	***y z***	**U(eq)**
$C^{(4)}$	5365(17)	8304(17)	7274(7)	126(7)
$C^{(5)}$	6019(12)	8376(11)	7209(5)	92(5)
$C^{(6)}$	6235(11)	7930(10)	7392(5)	89(5)
$C^{(7)}$	7026(8)	8107(8)	7342(4)	69(4)
$C^{(8)}$	7458(12)	8415(11)	7761(5)	96(6)
$C^{(9)}$	7311(8)	7312(8)	7623(4)	66(3)
$C^{(10)}$	7426(7)	6698(8)	7682(4)	68(4)
$C^{(11)}$	7550(8)	6467(10)	8066(4)	78(4)
$C^{(12)}$	7630(10)	5883(11)	8081(4)	83(5)
$C^{(012)}$	5318(11)	4365(11)	7253(5)	92(5)
$C^{(13)}$	7617(9)	5502(10)	7721(4)	79(5)
$C^{(14)}$	7513(7)	5799(9)	7355(4)	68(4)
$C^{(15)}$	7502(7)	5469(7)	6949(4)	56(3)
$C^{(16)}$	7440(10)	4607(9)	6530(5)	76(4)
$C^{(17)}$	7452(8)	5246(8)	6279(4)	62(3)
$C^{(18)}$	8135(11)	5629(11)	6018(4)	75(3)
$C^{(19)}$	8827(10)	6083(10)	6209(6)	86(4)
$C^{(20)}$	9457(11)	6414(12)	5983(7)	99(4)
$C^{(21)}$	9426(12)	6304(13)	5546(7)	109(5)
$C^{(22)}$	8780(12)	5874(13)	5368(6)	105(5)
$C^{(23)}$	8135(12)	5540(12)	5599(5)	89(4)
$C^{(24)}$	5282(18)	6666.67	6666.67	129(11)
$C^{(25)}$	4610(20)	6666.67	6666.67	164(16)
$S^{(2)}$	9980(20)	10010(16)	7683(3)	103(4)
$F^{(4)}$	10460(30)	10290(19)	8440(11)	119(11)
$F^{(5)}$	9320(30)	10090(30)	8338(15)	131(11)
$F^{(6)}$	9480(30)	9210(20)	8343(13)	128(13)
$O^{(6)}$	9230(50)	9500(40)	7540(20)	103(11)
$O^{(7)}$	10400(40)	9670(30)	7560(20)	100(9)
$O^{(8)}$	10230(50)	10760(30)	7677(18)	116(12)
$C^{(26)}$	9830(20)	9910(20)	8244(14)	116(8)

Table S25 Anisotropic Displacement Parameters (Å^2×10^3) for Er(OTf)$_3$/L$_1$. The Anisotropic displacement factor exponent takes the form: $-2\pi^2[h^2a^{*2}U_{11}+2hka^*b^*U_{12}+\ldots]$.

**Atom**		**U_{11}**	**U_{22}**	**U_{33}**	**U_{23}**	**U_{13}**	**U_{12}**
Er 70.5(6) 64.6(8)		46.9(5)	-0.1(7)		-0.1(3)	32.3(4)	
$S^{(1)}$	61(2)	94(3)	54.2(18)	4.4(17)	5.6(15)	33.0(19)	

Table S25 Anisotropic Displacement Parameters (Å^2 × 10^3) for Er(OTf)$_3$/L$_1$. The Anisotropic displacement factor exponent takes the form: $-2\pi^2[h^2a^{*2}U_{11}+2hka^*b^*U_{12}+\ldots]$.

Atom	U_{11}	U_{22}	U_{33}	U_{23}	U_{13}	U_{12}
$F^{(1)}$	94(8)	100(8)	185(12)	-12(8)	-20(8)	52(7)
$F^{(2)}$	119(9)	110(8)	99(7)	-24(6)	-17(6)	38(7)
$F^{(3)}$	132(10)	118(9)	102(8)	18(7)	2(7)	37(8)
$O^{(1)}$	63(6)	102(7)	53(5)	3(5)	6(5)	34(6)
$O^{(2)}$	101(8)	106(8)	47(5)	-1(5)	18(5)	34(7)
$O^{(3)}$	64(6)	125(10)	95(8)	26(7)	24(6)	42(7)
$O^{(4)}$	90(7)	90(7)	61(5)	11(5)	6(5)	59(6)
$O^{(5)}$	97(8)	100(8)	59(6)	-9(5)	-2(5)	49(7)
$N^{(1)}$	79(8)	67(7)	48(6)	4(5)	9(5)	25(6)
$N^{(2)}$	60(6)	72(7)	49(5)	-6(6)	-3(5)	38(5)
$N^{(3)}$	62(6)	61(6)	52(6)	-2(5)	4(5)	29(5)
$N^{(4)}$	54(6)	59(9)	54(8)	18(8)	9(4)	30(5)
$C^{(1)}$	124(18)	190(20)	137(18)	76(17)	55(15)	111(19)
$C^{(2)}$	120(20)	270(40)	200(30)	120(30)	90(20)	130(30)
$C^{(3)}$	180(30)	240(40)	200(30)	30(30)	40(30)	180(30)
$C^{(4)}$	150(20)	160(20)	117(17)	15(18)	5(18)	110(19)
$C^{(5)}$	122(15)	100(13)	72(10)	4(9)	-1(10)	70(12)
$C^{(6)}$	113(14)	101(13)	69(9)	1(9)	14(9)	66(12)
$C^{(7)}$	94(11)	57(7)	65(8)	5(6)	19(7)	45(7)
$C^{(8)}$	102(14)	102(13)	79(10)	-21(9)	14(10)	46(12)
$C^{(9)}$	60(7)	69(8)	48(7)	-13(6)	7(6)	17(7)
$C^{(10)}$	54(7)	86(10)	51(7)	0(6)	3(5)	25(7)
$C^{(11)}$	78(10)	110(13)	58(8)	19(8)	10(7)	56(10)
$C^{(12)}$	91(12)	113(14)	46(7)	11(8)	5(7)	53(11)
$C^{(012)}$	65(10)	114(13)	92(11)	-18(10)	8(10)	41(10)
$C^{(13)}$	93(11)	125(14)	53(8)	12(8)	4(7)	81(11)
$C^{(14)}$	56(8)	88(10)	59(7)	2(7)	3(6)	35(8)
$C^{(15)}$	54(7)	64(8)	55(7)	5(6)	2(5)	33(6)
$C^{(16)}$	80(10)	68(9)	79(10)	3(8)	0(8)	37(8)
$C^{(17)}$	67(8)	66(8)	54(7)	-9(6)	-5(6)	33(7)
$C^{(18)}$	94(8)	84(8)	69(6)	9(7)	14(7)	60(6)
$C^{(19)}$	92(8)	85(8)	89(8)	8(7)	17(7)	50(7)
$C^{(20)}$	90(8)	106(9)	109(9)	10(8)	19(7)	54(7)
$C^{(21)}$	102(9)	116(10)	110(9)	12(9)	30(8)	56(8)
$C^{(22)}$	116(9)	119(10)	83(8)	15(8)	24(7)	61(8)
$C^{(23)}$	101(9)	105(9)	74(6)	16(7)	13(7)	62(7)
$C^{(24)}$	110(18)	120(30)	160(30)	0(20)	-1(11)	60(13)
$C^{(25)}$	140(30)	140(30)	210(50)	-20(30)	-10(16)	70(17)

Table S25 Anisotropic Displacement Parameters (Å^2 × 10^3) for Er(OTf)$_3$/L$_1$. The Anisotropic displacement factor exponent takes the form: -2π^2[h^{2}a*^{2}U$_{11}$+2hka*b*U$_{12}$+…].

Atom	U$_{11}$	U$_{22}$	U$_{33}$	U$_{23}$	U$_{13}$	U$_{12}$
$S^{(2)}$	183(18)	67(13)	93(6)	-25(9)	-48(11)	87(16)
$F^{(4)}$	220(30)	90(20)	89(17)	-34(15)	-75(18)	105(19)
$F^{(5)}$	200(30)	110(20)	96(19)	-3(18)	-10(20)	90(20)
$F^{(6)}$	220(30)	93(17)	67(16)	-10(13)	-20(20)	80(20)
$O^{(6)}$	170(20)	70(20)	80(20)	-31(19)	-20(20)	60(20)
$O^{(7)}$	180(30)	80(20)	88(19)	-16(17)	-62(18)	100(20)
$O^{(8)}$	190(30)	62(18)	120(20)	-19(18)	-60(20)	80(20)
$C^{(26)}$	200(20)	80(15)	92(10)	-24(12)	-48(12)	86(17)

Table S26 Bond Lengths for Er(OTf)$_3$/L$_1$.

Atom	Atom	Length/Å	Atom	Atom	Length/Å
Er	$O^{(1)}$	2.328(10)	$C^{(2)}$	$C^{(3)}$	1.46(4)
Er	$O^{(1)1}$	2.328(10)	$C^{(3)}$	$C^{(4)}$	1.31(4)
Er	$N^{(1)1}$	2.543(11)	$C^{(4)}$	$C^{(5)}$	1.33(3)
Er	$N^{(1)}$	2.543(11)	$C^{(5)}$	$C^{(6)}$	1.37(2)
Er	$N^{(2)1}$	2.496(9)	$C^{(6)}$	$C^{(7)}$	1.52(2)
Er	$N^{(2)}$	2.497(9)	$C^{(7)}$	$C^{(8)}$	1.58(2)
Er	$N^{(3)}$	2.525(11)	$C^{(9)}$	$C^{(10)}$	1.44(2)
Er	$N^{(3)1}$	2.525(11)	$C^{(10)}$	$C^{(11)}$	1.408(19)
Er	$N^{(4)}$	2.349(15)	$C^{(11)}$	$C^{(12)}$	1.32(2)
$S^{(1)}$	$O^{(1)}$	1.455(10)	$C^{(12)}$	$C^{(13)}$	1.41(2)
$S^{(1)}$	$O^{(2)}$	1.422(11)	$C^{(13)}$	$C^{(14)}$	1.408(19)
$S^{(1)}$	$O^{(3)}$	1.442(12)	$C^{(14)}$	$C^{(15)}$	1.483(19)
$S^{(1)}$	$C^{(012)}$	1.79(2)	$C^{(16)}$	$C^{(17)}$	1.56(2)
$F^{(1)}$	$C^{(012)}$	1.34(2)	$C^{(17)}$	$C^{(18)}$	1.51(2)
$F^{(2)}$	$C^{(012)}$	1.322(19)	$C^{(18)}$	$C^{(19)}$	1.42(3)
$F^{(3)}$	$C^{(012)}$	1.32(2)	$C^{(18)}$	$C^{(23)}$	1.37(2)
$O^{(4)}$	$C^{(15)}$	1.345(16)	$C^{(19)}$	$C^{(20)}$	1.36(2)
$O^{(4)}$	$C^{(16)}$	1.444(18)	$C^{(20)}$	$C^{(21)}$	1.43(3)
$O^{(5)}$	$C^{(8)}$	1.45(2)	$C^{(21)}$	$C^{(22)}$	1.33(3)
$O^{(5)}$	$C^{(9)}$	1.348(16)	$C^{(22)}$	$C^{(23)}$	1.39(3)
$N^{(1)}$	$C^{(7)}$	1.495(17)	$C^{(24)}$	$C^{(25)}$	1.43(5)
$N^{(1)}$	$C^{(9)}$	1.264(18)	$S^{(2)}$	$O^{(6)}$	1.48(10)
$N^{(2)}$	$C^{(10)}$	1.335(16)	$S^{(2)}$	$O^{(7)}$	1.44(9)
$N^{(2)}$	$C^{(14)}$	1.316(18)	$S^{(2)}$	$O^{(8)}$	1.40(6)
$N^{(3)}$	$C^{(15)}$	1.252(16)	$S^{(2)}$	$C^{(26)}$	1.85(5)
$N^{(3)}$	$C^{(17)}$	1.509(16)	$F^{(4)}$	$C^{(26)}$	1.330(14)
$N^{(4)}$	$C^{(24)}$	1.31(4)	$F^{(5)}$	$C^{(26)}$	1.327(15)

Table S26 Bond Lengths for Er(OTf)$_3$/L$_1$.

Atom	Atom	Length/Å	Atom	Atom	Length/Å
$C^{(1)}$	$C^{(2)}$	1.39(3)	$F^{(6)}$	$C^{(26)}$	1.327(15)
$C^{(1)}$	$C^{(6)}$	1.38(3)			

^{1}2/3-Y+X,4/3-Y,4/3-Z

Table S27 Bond Angles for Er(OTf)$_3$/L$_1$.

Atom	Atom	Atom	Angle/˚	Atom	Atom	Atom	Angle/˚
$O^{(1)}$	Er	$O^{(1)1}$	130.9(6)	$C^{(4)}$	$C^{(3)}$	$C^{(2)}$	119(2)
$O^{(1)}$	Er	$N^{(1)}$	92.8(3)	$C^{(3)}$	$C^{(4)}$	$C^{(5)}$	122(2)
$O^{(1)}$	Er	$N^{(1)1}$	74.3(3)	$C^{(4)}$	$C^{(5)}$	$C^{(6)}$	122(2)
$O^{(1)1}$	Er	$N^{(1)}$	74.3(3)	$C^{(1)}$	$C^{(6)}$	$C^{(7)}$	122.9(17)
$O^{(1)1}$	Er	$N^{(1)1}$	92.8(3)	$C^{(5)}$	$C^{(6)}$	$C^{(1)}$	117.8(19)
$O^{(1)}$	Er	$N^{(2)}$	71.9(4)	$C^{(5)}$	$C^{(6)}$	$C^{(7)}$	119.0(17)
$O^{(1)1}$	Er	$N^{(2)1}$	71.9(4)	$N^{(1)}$	$C^{(7)}$	$C^{(6)}$	111.9(13)
$O^{(1)1}$	Er	$N^{(2)}$	133.8(4)	$N^{(1)}$	$C^{(7)}$	$C^{(8)}$	101.0(12)
$O^{(1)}$	Er	$N^{(2)1}$	133.8(4)	$C^{(6)}$	$C^{(7)}$	$C^{(8)}$	110.0(13)
$O^{(1)}$	Er	$N^{(3)}$	76.1(4)	$O^{(5)}$	$C^{(8)}$	$C^{(7)}$	103.9(13)
$O^{(1)}$	Er	$N^{(3)1}$	147.7(3)	$O^{(5)}$	$C^{(9)}$	$C^{(10)}$	117.8(13)
$O^{(1)1}$	Er	$N^{(3)1}$	76.1(4)	$N^{(1)}$	$C^{(9)}$	$O^{(5)}$	119.1(15)
$O^{(1)1}$	Er	$N^{(3)}$	147.7(3)	$N^{(1)}$	$C^{(9)}$	$C^{(10)}$	123.0(12)
$O^{(1)}$	Er	$N^{(4)}$	65.5(3)	$N^{(2)}$	$C^{(10)}$	$C^{(9)}$	112.7(12)
$O^{(1)1}$	Er	$N^{(4)}$	65.5(3)	$N^{(2)}$	$C^{(10)}$	$C^{(11)}$	122.4(14)
$N^{(1)}$	Er	$N^{(1)1}$	149.1(6)	$C^{(11)}$	$C^{(10)}$	$C^{(9)}$	124.8(13)
$N^{(2)}$	Er	$N^{(1)1}$	133.3(4)	$C^{(12)}$	$C^{(11)}$	$C^{(10)}$	119.1(15)
$N^{(2)}$	Er	$N^{(1)}$	63.9(4)	$C^{(11)}$	$C^{(12)}$	$C^{(13)}$	121.6(14)
$N^{(2)1}$	Er	$N^{(1)1}$	63.9(4)	$F^{(1)}$	$C^{(012)}$	$S^{(1)}$	112.2(13)
$N^{(2)1}$	Er	$N^{(1)}$	133.3(4)	$F^{(2)}$	$C^{(012)}$	$S^{(1)}$	110.4(15)
$N^{(2)1}$	Er	$N^{(2)}$	125.2(5)	$F^{(2)}$	$C^{(012)}$	$F^{(1)}$	107.6(15)
$N^{(2)1}$	Er	$N^{(3)}$	76.0(4)	$F^{(3)}$	$C^{(012)}$	$S^{(1)}$	112.7(13)
$N^{(2)1}$	Er	$N^{(3)1}$	64.6(3)	$F^{(3)}$	$C^{(012)}$	$F^{(1)}$	106.3(18)
$N^{(2)}$	Er	$N^{(3)1}$	76.0(4)	$F^{(3)}$	$C^{(012)}$	$F^{(2)}$	107.5(15)
$N^{(2)}$	Er	$N^{(3)}$	64.6(3)	$C^{(14)}$	$C^{(13)}$	$C^{(12)}$	114.1(15)
$N^{(3)1}$	Er	$N^{(1)}$	76.7(4)	$N^{(2)}$	$C^{(14)}$	$C^{(13)}$	126.1(13)
$N^{(3)}$	Er	$N^{(1)1}$	76.7(4)	$N^{(2)}$	$C^{(14)}$	$C^{(15)}$	113.4(12)
$N^{(3)1}$	Er	$N^{(1)1}$	128.2(4)	$C^{(13)}$	$C^{(14)}$	$C^{(15)}$	120.6(14)
$N^{(3)}$	Er	$N^{(1)}$	128.2(4)	$O^{(4)}$	$C^{(15)}$	$C^{(14)}$	117.1(11)
$N^{(3)1}$	Er	$N^{(3)}$	86.5(5)	$N^{(3)}$	$C^{(15)}$	$O^{(4)}$	120.3(12)
$N^{(4)}$	Er	$N^{(1)}$	74.5(3)	$N^{(3)}$	$C^{(15)}$	$C^{(14)}$	122.6(12)
$N^{(4)}$	Er	$N^{(1)1}$	74.5(3)	$O^{(4)}$	$C^{(16)}$	$C^{(17)}$	104.3(11)
$N^{(4)}$	Er	$N^{(2)}$	117.4(2)	$N^{(3)}$	$C^{(17)}$	$C^{(16)}$	102.0(10)
$N^{(4)}$	Er	$N^{(2)1}$	117.4(2)	$N^{(3)}$	$C^{(17)}$	$C^{(18)}$	113.6(12)

Table S27 Bond Angles for Er(OTf)$_3$/L$_1$.

Atom	Atom	Atom	Angle/°	Atom	Atom	Atom	Angle/°
$N^{(4)}$	Er	$N^{(3)1}$	136.8(2)	$C^{(18)}$	$C^{(17)}$	$C^{(16)}$	110.7(12)
$N^{(4)}$	Er	$N^{(3)}$	136.8(3)	$C^{(19)}$	$C^{(18)}$	$C^{(17)}$	119.7(13)
$O^{(1)}$	$S^{(1)}$	$C^{(012)}$	100.3(7)	$C^{(23)}$	$C^{(18)}$	$C^{(17)}$	123.3(19)
$O^{(2)}$	$S^{(1)}$	$O^{(1)}$	111.7(6)	$C^{(23)}$	$C^{(18)}$	$C^{(19)}$	117.0(18)
$O^{(2)}$	$S^{(1)}$	$O^{(3)}$	117.2(8)	$C^{(20)}$	$C^{(19)}$	$C^{(18)}$	121.1(18)
$O^{(2)}$	$S^{(1)}$	$C^{(012)}$	105.6(8)	$C^{(19)}$	$C^{(20)}$	$C^{(21)}$	120(2)
$O^{(3)}$	$S^{(1)}$	$O^{(1)}$	114.7(7)	$C^{(22)}$	$C^{(21)}$	$C^{(20)}$	119(2)
$O^{(3)}$	$S^{(1)}$	$C^{(012)}$	105.0(9)	$C^{(21)}$	$C^{(22)}$	$C^{(23)}$	121(2)
$S^{(1)}$	$O^{(1)}$	Er	146.7(7)	$C^{(18)}$	$C^{(23)}$	$C^{(22)}$	122(2)
$C^{(15)}$	$O^{(4)}$	$C^{(16)}$	105.5(11)	$N^{(4)}$	$C^{(24)}$	$C^{(25)}$	180.00(3)
$C^{(9)}$	$O^{(5)}$	$C^{(8)}$	105.5(12)	$O^{(6)}$	$S^{(2)}$	$C^{(26)}$	99(4)
$C^{(7)}$	$N^{(1)}$	Er	133.8(8)	$O^{(7)}$	$S^{(2)}$	$O^{(6)}$	104(4)
$C^{(9)}$	$N^{(1)}$	Er	117.3(10)	$O^{(7)}$	$S^{(2)}$	$C^{(26)}$	111(3)
$C^{(9)}$	$N^{(1)}$	$C^{(7)}$	107.9(12)	$O^{(8)}$	$S^{(2)}$	$O^{(6)}$	118(6)
$C^{(10)}$	$N^{(2)}$	Er	121.9(9)	$O^{(8)}$	$S^{(2)}$	$O^{(7)}$	126(6)
$C^{(14)}$	$N^{(2)}$	Er	120.6(8)	$O^{(8)}$	$S^{(2)}$	$C^{(26)}$	95(3)
$C^{(14)}$	$N^{(2)}$	$C^{(10)}$	116.7(11)	$F^{(4)}$	$C^{(26)}$	$S^{(2)}$	109(4)
$C^{(15)}$	$N^{(3)}$	Er	116.8(9)	$F^{(5)}$	$C^{(26)}$	$S^{(2)}$	109(4)
$C^{(15)}$	$N^{(3)}$	$C^{(17)}$	106.3(11)	$F^{(5)}$	$C^{(26)}$	$F^{(4)}$	116(5)
$C^{(17)}$	$N^{(3)}$	Er	135.5(8)	$F^{(6)}$	$C^{(26)}$	$S^{(2)}$	109(4)
$C^{(24)}$	$N^{(4)}$	Er	180.000(13)	$F^{(6)}$	$C^{(26)}$	$F^{(4)}$	112(5)
$C^{(6)}$	$C^{(1)}$	$C^{(2)}$	121(2)	$F^{(6)}$	$C^{(26)}$	$F^{(5)}$	101(4)
$C^{(1)}$	$C^{(2)}$	$C^{(3)}$	116(3)				

[1]2/3-Y+X,4/3-Y,4/3-Z

Table S28 Torsion Angles for Er(OTf)$_3$/L$_1$.

A	B	C	D	Angle/°	A	B	C	D	Angle/°
Er	$N^{(1)}$	$C^{(7)}$	$C^{(6)}$	87.3(15)	$C^{(6)}$	$C^{(7)}$	$C^{(8)}$	$O^{(5)}$	102.7(15)
Er	$N^{(1)}$	$C^{(7)}$	$C^{(8)}$	-155.7(11)	$C^{(7)}$	$N^{(1)}$	$C^{(9)}$	$O^{(5)}$	-3.7(17)
Er	$N^{(1)}$	$C^{(9)}$	$O^{(5)}$	166.3(9)	$C^{(7)}$	$N^{(1)}$	$C^{(9)}$	$C^{(10)}$	179.8(13)
Er	$N^{(1)}$	$C^{(9)}$	$C^{(10)}$	-10.1(17)	$C^{(8)}$	$O^{(5)}$	$C^{(9)}$	$N^{(1)}$	-7.4(18)
Er	$N^{(2)}$	$C^{(10)}$	$C^{(9)}$	-10.0(15)	$C^{(8)}$	$O^{(5)}$	$C^{(9)}$	$C^{(10)}$	169.2(14)
Er	$N^{(2)}$	$C^{(10)}$	$C^{(11)}$	171.0(10)	$C^{(9)}$	$O^{(5)}$	$C^{(8)}$	$C^{(7)}$	14.2(16)
Er	$N^{(2)}$	$C^{(14)}$	$C^{(13)}$	-168.9(12)	$C^{(9)}$	$N^{(1)}$	$C^{(7)}$	$C^{(6)}$	-105.0(14)
Er	$N^{(2)}$	$C^{(14)}$	$C^{(15)}$	10.9(15)	$C^{(9)}$	$N^{(1)}$	$C^{(7)}$	$C^{(8)}$	12.0(15)
Er	$N^{(3)}$	$C^{(15)}$	$O^{(4)}$	166.5(9)	$C^{(9)}$	$C^{(10)}$	$C^{(11)}$	$C^{(12)}$	178.4(16)
Er	$N^{(3)}$	$C^{(15)}$	$C^{(14)}$	-12.0(16)	$C^{(10)}$	$N^{(2)}$	$C^{(14)}$	$C^{(13)}$	1(2)
Er	$N^{(3)}$	$C^{(17)}$	$C^{(16)}$	-156.7(10)	$C^{(10)}$	$N^{(2)}$	$C^{(14)}$	$C^{(15)}$	-179.4(11)
Er	$N^{(3)}$	$C^{(17)}$	$C^{(18)}$	84.2(14)	$C^{(10)}$	$C^{(11)}$	$C^{(12)}$	$C^{(13)}$	2(3)

Table S28 Torsion Angles for $Er(OTf)_3/L_1$.

A	B	C	D	Angle/˚	A	B	C	D	Angle/˚
$O^{(1)}$	$S^{(1)}$	$C^{(012)}$	$F^{(1)}$	51.5(14)	$C^{(11)}$	$C^{(12)}$	$C^{(13)}$	$C^{(14)}$	0(3)
$O^{(1)}$	$S^{(1)}$	$C^{(012)}$	$F^{(2)}$	-68.4(14)	$C^{(12)}$	$C^{(13)}$	$C^{(14)}$	$N^{(2)}$	-2(2)
$O^{(1)}$	$S^{(1)}$	$C^{(012)}$	$F^{(3)}$	171.4(13)	$C^{(12)}$	$C^{(13)}$	$C^{(14)}$	$C^{(15)}$	178.7(14)
$O^{(2)}$	$S^{(1)}$	$O^{(1)}$	Er	-41.7(15)	$C^{(012)}$	$S^{(1)}$	$O^{(1)}$	Er	-153.3(12)
$O^{(2)}$	$S^{(1)}$	$C^{(012)}$	$F^{(1)}$	-64.7(15)	$C^{(13)}$	$C^{(14)}$	$C^{(15)}$	$O^{(4)}$	2.4(19)
$O^{(2)}$	$S^{(1)}$	$C^{(012)}$	$F^{(2)}$	175.4(12)	$C^{(13)}$	$C^{(14)}$	$C^{(15)}$	$N^{(3)}$	-179.0(14)
$O^{(2)}$	$S^{(1)}$	$C^{(012)}$	$F^{(3)}$	55.2(16)	$C^{(14)}$	$N^{(2)}$	$C^{(10)}$	$C^{(9)}$	-179.6(12)
$O^{(3)}$	$S^{(1)}$	$O^{(1)}$	Er	94.8(13)	$C^{(14)}$	$N^{(2)}$	$C^{(10)}$	$C^{(11)}$	1(2)
$O^{(3)}$	$S^{(1)}$	$C^{(012)}$	$F^{(1)}$	170.8(13)	$C^{(15)}$	$O^{(4)}$	$C^{(16)}$	$C^{(17)}$	11.2(14)
$O^{(3)}$	$S^{(1)}$	$C^{(012)}$	$F^{(2)}$	50.9(14)	$C^{(15)}$	$N^{(3)}$	$C^{(17)}$	$C^{(16)}$	9.0(14)
$O^{(3)}$	$S^{(1)}$	$C^{(012)}$	$F^{(3)}$	-69.3(16)	$C^{(15)}$	$N^{(3)}$	$C^{(17)}$	$C^{(18)}$	-110.2(13)
$O^{(4)}$	$C^{(16)}$	$C^{(17)}$	$N^{(3)}$	-12.2(14)	$C^{(16)}$	$O^{(4)}$	$C^{(15)}$	$N^{(3)}$	-6.2(17)
$O^{(4)}$	$C^{(16)}$	$C^{(17)}$	$C^{(18)}$	109.0(13)	$C^{(16)}$	$O^{(4)}$	$C^{(15)}$	$C^{(14)}$	172.4(12)
$O^{(5)}$	$C^{(9)}$	$C^{(10)}$	$N^{(2)}$	-163.0(12)	$C^{(16)}$	$C^{(17)}$	$C^{(18)}$	$C^{(19)}$	-71.9(18)
$O^{(5)}$	$C^{(9)}$	$C^{(10)}$	$C^{(11)}$	16(2)	$C^{(16)}$	$C^{(17)}$	$C^{(18)}$	$C^{(23)}$	105.2(16)
$N^{(1)}$	$C^{(7)}$	$C^{(8)}$	$O^{(5)}$	-15.7(15)	$C^{(17)}$	$N^{(3)}$	$C^{(15)}$	$O^{(4)}$	-2.3(16)
$N^{(1)}$	$C^{(9)}$	$C^{(10)}$	$N^{(2)}$	13.5(19)	$C^{(17)}$	$N^{(3)}$	$C^{(15)}$	$C^{(14)}$	179.2(12)
$N^{(1)}$	$C^{(9)}$	$C^{(10)}$	$C^{(11)}$	-167.6(14)	$C^{(17)}$	$C^{(18)}$	$C^{(19)}$	$C^{(20)}$	177.5(15)
$N^{(2)}$	$C^{(10)}$	$C^{(11)}$	$C^{(12)}$	-3(2)	$C^{(17)}$	$C^{(18)}$	$C^{(23)}$	$C^{(22)}$	-176.9(16)
$N^{(2)}$	$C^{(14)}$	$C^{(15)}$	$O^{(4)}$	-177.4(12)	$C^{(18)}$	$C^{(19)}$	$C^{(20)}$	$C^{(21)}$	0(3)
$N^{(2)}$	$C^{(14)}$	$C^{(15)}$	$N^{(3)}$	1.1(19)	$C^{(19)}$	$C^{(18)}$	$C^{(23)}$	$C^{(22)}$	0(3)
$N^{(3)}$	$C^{(17)}$	$C^{(18)}$	$C^{(19)}$	42.1(18)	$C^{(19)}$	$C^{(20)}$	$C^{(21)}$	$C^{(22)}$	-1(3)
$N^{(3)}$	$C^{(17)}$	$C^{(18)}$	$C^{(23)}$	-140.7(15)	$C^{(20)}$	$C^{(21)}$	$C^{(22)}$	$C^{(23)}$	1(3)
$C^{(1)}$	$C^{(2)}$	$C^{(3)}$	$C^{(4)}$	11(6)	$C^{(21)}$	$C^{(22)}$	$C^{(23)}$	$C^{(18)}$	-1(3)
$C^{(1)}$	$C^{(6)}$	$C^{(7)}$	$N^{(1)}$	45(2)	$C^{(23)}$	$C^{(18)}$	$C^{(19)}$	$C^{(20)}$	0(3)
$C^{(1)}$	$C^{(6)}$	$C^{(7)}$	$C^{(8)}$	-66(2)	$O^{(6)}$	$S^{(2)}$	$C^{(26)}$	$F^{(4)}$	175(4)
$C^{(2)}$	$C^{(1)}$	$C^{(6)}$	$C^{(5)}$	-1(4)	$O^{(6)}$	$S^{(2)}$	$C^{(26)}$	$F^{(5)}$	-58(5)
$C^{(2)}$	$C^{(1)}$	$C^{(6)}$	$C^{(7)}$	173(3)	$O^{(6)}$	$S^{(2)}$	$C^{(26)}$	$F^{(6)}$	51(5)
$C^{(2)}$	$C^{(3)}$	$C^{(4)}$	$C^{(5)}$	-12(6)	$O^{(7)}$	$S^{(2)}$	$C^{(26)}$	$F^{(4)}$	65(5)
$C^{(3)}$	$C^{(4)}$	$C^{(5)}$	$C^{(6)}$	5(4)	$O^{(7)}$	$S^{(2)}$	$C^{(26)}$	$F^{(5)}$	-167(5)
$C^{(4)}$	$C^{(5)}$	$C^{(6)}$	$C^{(1)}$	1(3)	$O^{(7)}$	$S^{(2)}$	$C^{(26)}$	$F^{(6)}$	-58(6)
$C^{(4)}$	$C^{(5)}$	$C^{(6)}$	$C^{(7)}$	-173(2)	$O^{(8)}$	$S^{(2)}$	$C^{(26)}$	$F^{(4)}$	-66(5)
$C^{(5)}$	$C^{(6)}$	$C^{(7)}$	$N^{(1)}$	-141.2(15)	$O^{(8)}$	$S^{(2)}$	$C^{(26)}$	$F^{(5)}$	62(5)
$C^{(5)}$	$C^{(6)}$	$C^{(7)}$	$C^{(8)}$	107.4(18)	$O^{(8)}$	$S^{(2)}$	$C^{(26)}$	$F^{(6)}$	171(6)
$C^{(6)}$	$C^{(1)}$	$C^{(2)}$	$C^{(3)}$	-5(5)					

Table S29 Hydrogen Atom Coordinates (Å×10^4) and Isotropic Displacement Parameters (Å^2×10^3) for Er(OTf)$_3$/L$_1$.

Atom	*x*	*y*	*z*	U(eq)
H(1)	5875.41	7058.01	7774.25	161
H(2)	4695.09	6889.68	7878.82	225
H(3)	4479.25	7856.33	7621.99	212
H(4)	5225.75	8599.7	7121.22	151
H(5)	6350.22	8750.85	7028.27	110
H(7)	7273.63	8459.88	7110.91	83
H(8A)	7954.51	8836.05	7710.96	116
H(8B)	7190.53	8572.41	7948.45	116
H(11)	7574.38	6725.54	8311.09	93
H(12)	7698.95	5717.33	8340.57	99
H(13)	7672.81	5081.33	7724.14	95
H(16A)	6960.1	4152.18	6502.5	91
H(16B)	7830.57	4510.8	6434.25	91
H(17)	7006.08	5053.78	6101.5	75
H(19)	8848.96	6157.1	6498.6	104
H(20)	9914.42	6715.89	6114.92	119
H(21)	9860.54	6535.63	5384.55	131
H(22)	8757.65	5792.63	5079.25	126
H(23)	7681.86	5242.32	5461.89	107
H(25A)	4270.33	6307.3	6466	245
H(25B)	4694.43	7155.5	6592	245
H(25C)	4385.23	6537.2	6942	245

Table S30 Atomic Occupancy for Er(OTf)$_3$/L$_1$.

Atom	*Occupancy*	Atom	*Occupancy*	Atom	*Occupancy*
H(25A)	0.5	H(25B)	0.5	H(25C)	0.5
S(2)	0.3333	F(4)	0.3333	F(5)	0.3333
F(6)	0.3333	O(6)	0.3333	O(7)	0.3333
O(8)	0.3333	C(26)	0.3333		

Table S31 Solvent masks information for Er(OTf)$_3$/L$_1$.

Number	X	Y	Z	Volume	Electron count
1	0.000	0.000	0.058	15.3	1.7
2	0.000	0.000	0.500	218.7	79.2
3	0.000	0.000	0.942	15.3	1.7
4	0.099	0.223	0.133	13.8	0.0
5	0.333	0.667	0.167	218.7	79.2
6	0.110	0.542	0.800	13.8	0.0

Table S31 Solvent masks information for $Er(OTf)_3/L_1$.

Number	X	Y	Z	Volume	Electron count
7	0.124	0.901	0.133	13.8	0.0
8	0.209	0.443	0.534	13.8	0.6
9	0.223	0.099	0.867	13.8	0.6
10	0.234	0.791	0.534	13.8	0.6
11	0.333	0.667	0.609	15.3	1.7
12	0.333	0.667	0.724	15.3	1.7
13	0.432	0.890	0.800	13.8	0.0
14	0.667	0.333	0.833	218.7	79.2
15	0.443	0.209	0.466	13.8	0.0
16	0.458	0.568	0.800	13.8	0.0
17	0.542	0.110	0.200	13.8	0.6
18	0.568	0.458	0.200	13.8	0.6
19	0.557	0.766	0.534	13.8	0.6
20	0.667	0.333	0.276	15.3	1.7
21	0.667	0.333	0.391	15.3	1.7
22	0.766	0.557	0.466	13.8	0.0
23	0.777	0.876	0.133	13.8	0.0
24	0.791	0.234	0.466	13.8	0.0
25	0.876	0.777	0.867	13.8	0.6
26	0.901	0.124	0.867	13.8	0.6
27	0.890	0.432	0.200	13.8	0.6

Data intensity of $Er(OTf)_3$/**L₆**[20] was collected using a 'Bruker APEX-II CCD' diffractometer at 296(2) K. Data collection and reduction were done by using Olex2 and the structure was solved with the ShelXS structure solution program using direct methods and refined by full-matrix least-squares on F^2 with anisotropic displacement parameters for non-H atoms using SHELX-97. Hydrogen atoms were added at their geometrically idea positions and refined isotropically. The crystal was grown in CH_3CN/Et_2O system.

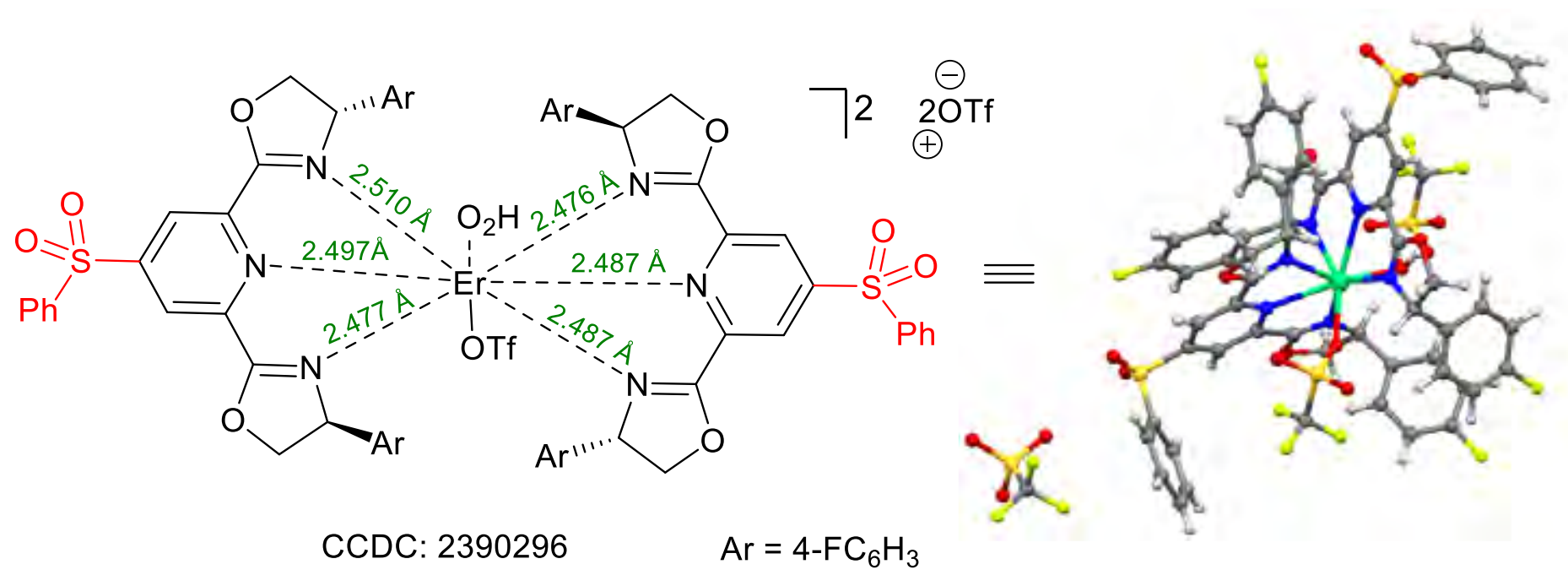

Table S32 Crystal data and structure refinement for $Er(OTf)_3$/L_6.

Identification code	$Er(OTf)_3$/**L_6**
Empirical formula	$C_{65}H_{50}ErF_{13}N_8O_{18}S_5$
Formula weight	1805.69
Temperature/K	173.00(10)
Crystal system	orthorhombic
Space group	$P2_12_12$
a/Å	44.2511(6)
b/Å	14.4061(2)
c/Å	11.4002(2)
α/°	90
β/°	90
γ/°	90
Volume/Å³	7267.46(19)
Z	4
ρ_{calc}g/cm³	1.650

μ/mm^{-1}	4.445
F(000)	3620.0
Crystal size/mm^3	0.26 × 0.22 × 0.12
Radiation	Cu Kα (λ = 1.54184)
2Θ range for data collection/°	7.322 to 134.138
Index ranges	$-50 \le h \le 52$, $-16 \le k \le 17$, $-13 \le l \le 13$
Reflections collected	86910
Independent reflections	12963 [R_{int} = 0.0778, R_{sigma} = 0.0446]
Data/restraints/parameters	12963/330/1083
Goodness-of-fit on F^2	1.048
Final R indexes [I>=2σ (I)]	R_1 = 0.0510, wR_2 = 0.1301
Final R indexes [all data]	R_1 = 0.0535, wR_2 = 0.1319
Largest diff. peak/hole / e Å^{-3}	1.08/-0.57
Flack parameter	-0.010(6)

Table S33 Fractional Atomic Coordinates (×10^4) and Equivalent Isotropic Displacement Parameters (Å^2×10^3) for Er(OTf)$_3$/L$_6$. U_{eq} is defined as 1/3 of the trace of the orthogonalised U_{IJ} tensor.

Atom	*x*	*y*	*z*	U(eq)
Er(1)	6202.0(2)	6610.6(3)	4020.0(4)	34.30(13)
S(1)	6882.3(5)	2197.8(14)	4061(3)	55.1(6)
S(3)	5518.4(5)	7442.3(17)	5463(2)	49.9(5)
S(2)	6351.7(6)	10400.9(16)	7820(2)	55.6(6)
S(100)	5805.6(8)	11787(2)	12323(4)	90.4(11)
S(006)	6889.0(9)	6741(3)	19(3)	69.9(12)
O(10)	6416.4(14)	9622(4)	3109(5)	44.3(13)
O(4)	7199.0(11)	5799(4)	3780(5)	42.6(13)
O(9)	5738.8(12)	7207(4)	4567(5)	44.3(13)
F(2)	7216.7(15)	10004(5)	7168(7)	87(2)
O(3)	5759.8(13)	3766(4)	4239(6)	52.0(16)
N(4)	6253.4(15)	6180(4)	6123(6)	38.9(15)
O(1W)	6237.6(15)	6189(4)	2073(5)	45.1(13)
N(2)	6768.9(12)	6630(4)	3937(5)	32.0(11)
F(4)	5162.4(14)	8581(6)	-552(6)	89(2)

Table S33 Fractional Atomic Coordinates (×10^4) and Equivalent Isotropic Displacement Parameters (Å^2×10^3) for Er(OTf)$_3$/L$_6$. U_{eq} is defined as 1/3 of the trace of the orthogonalised U_{IJ} tensor.

**Atom**	*x*	*y*	*z*	**U(eq)**
F(5)	5309.0(15)	8712(5)	4041(9)	90(2)
O(7)	5234.9(15)	6979(5)	5318(6)	61.9(18)
O(11)	6201.4(15)	6693(5)	7989(5)	51.1(14)
F(1)	7286.0(16)	3243(4)	7360(6)	80.7(19)
C(18)	7051.1(17)	8052(5)	4645(8)	37.4(17)
N(6)	6273.1(14)	8115(4)	3006(6)	36.1(15)
N(1)	6444.4(13)	5046(4)	4083(6)	36.6(13)
C(50)	6247.6(18)	6858(6)	6851(7)	40.4(18)
O(6)	6390(2)	10035(5)	8964(7)	80(2)
F(7)	5681.9(15)	9161(5)	5115(9)	102(3)
N(3)	5844.6(14)	5288(5)	3959(8)	46.1(16)
C(17)	7010.1(17)	7347(5)	3684(7)	37.9(18)
O(1)	6672(2)	1573(5)	4562(7)	76(2)
C(15)	6901.9(16)	5851(5)	3950(7)	34.5(15)
F(102)	5434(2)	10586(6)	13231(11)	126(4)
C(39)	6349.2(17)	8791(6)	4921(7)	36.2(18)
C(41)	6347.5(19)	9439(6)	6839(7)	42.4(19)
C(40)	6377.9(18)	9573(6)	5631(7)	39.9(18)
O(2)	7177.0(18)	2300(5)	4569(8)	78(3)
N(5)	6310.1(14)	7937(5)	5350(6)	35.8(15)
O(5)	6566(2)	11042(6)	7383(8)	86(3)
C(12)	6709.8(17)	3325(5)	4101(7)	39.5(15)
C(10)	6274.0(16)	4274(5)	4109(7)	36.3(16)
C(30)	5965.9(17)	8404(6)	1198(7)	41.6(17)
C(13)	6889.4(17)	4105(5)	4063(8)	37.3(15)
F(6)	5240.1(17)	8991(6)	5876(10)	118(3)
C(36)	6276.6(17)	8428(6)	1764(7)	40.5(17)
F(3)	5156(3)	6291(7)	-721(7)	134(4)
C(42)	6308.8(17)	8557(6)	7298(7)	40.1(18)
C(9)	5950.1(17)	4472(5)	4094(8)	41.9(17)
C(11)	6396.5(17)	3383(5)	4123(7)	40.4(16)
C(38)	6346.8(18)	8814(6)	3630(7)	37.2(18)
O(12)	6963(6)	6894(13)	-1205(13)	79(4)
C(21)	7163(2)	9369(7)	6329(10)	58(3)
O(8)	5647.8(17)	7476(7)	6608(6)	80(3)
C(37)	6401(2)	9436(6)	1839(8)	48(2)
C(52)	6222(2)	5317(6)	6851(8)	45.0(19)

Table S33 Fractional Atomic Coordinates (×10^4) and Equivalent Isotropic Displacement Parameters (Å^2×10^3) for Er(OTf)$_3$/L$_6$. U_{eq} is defined as 1/3 of the trace of the orthogonalised U_{IJ} tensor.

Atom	*x*	*y*	*z*	U(eq)
C(14)	6743.1(15)	4960(5)	4058(7)	33.6(14)
C(19)	7050.9(18)	7810(6)	5825(8)	45.8(19)
C(43)	6296.9(18)	7823(6)	6515(7)	38.4(18)
O(102)	5897(2)	11160(7)	11430(9)	96(3)
C(23)	7110.1(19)	8975(6)	4340(9)	49(2)
C(53)	6508(2)	4772(6)	6897(7)	42.9(18)
C(34)	5655(2)	7995(8)	-464(9)	58(2)
C(16)	7297.7(17)	6731(6)	3499(8)	43.8(19)
C(55)	6762(3)	3280(7)	7112(8)	61(3)
C(33)	5432(2)	8519(9)	14(9)	62(3)
C(57)	7054(2)	4692(7)	7131(10)	57(2)
F(100)	5324(2)	11998(6)	13586(10)	123(4)
C(20)	7102(2)	8467(8)	6672(9)	57(2)
C(56)	7031(3)	3753(7)	7202(9)	60(3)
C(54)	6496(3)	3807(6)	6962(8)	55(2)
C(58)	6791(2)	5204(6)	7001(9)	52(2)
F(01Y)	6949(3)	5303(9)	1451(9)	109(4)
C(24)	6917(2)	1985(7)	2558(10)	57(3)
O(101)	5969(2)	11662(8)	13493(10)	111(4)
C(7)	5516.7(19)	5189(6)	3802(10)	56(3)
C(51)	6123(2)	5696(6)	8057(9)	55(2)
C(2)	5254(2)	6301(8)	2418(11)	66(3)
F(101)	5261(2)	11393(11)	11841(13)	181(6)
C(32)	5457(2)	8957(8)	1097(10)	63(3)
C(31)	5731(2)	8910(7)	1659(9)	56(2)
C(1)	5421(2)	5490(8)	2593(10)	58(3)
O(13)	6562(2)	6621(10)	213(9)	78(3)
C(35)	5929(2)	7944(7)	155(8)	48(2)
C(22)	7167(2)	9635(7)	5224(11)	60(3)
C(3)	5166(3)	6563(10)	1305(10)	78(3)
C(4)	5247(4)	6041(12)	390(13)	93(5)
O(100)	5769(3)	12711(7)	11957(9)	123(4)
C(8)	5458(2)	4152(7)	4079(13)	65(3)
C(02F)	5428(2)	8662(9)	5107(14)	79(4)
C(100)	5436(3)	11426(13)	12780(18)	108(6)
C(25)	6672(2)	1683(8)	1935(10)	68(3)
C(26)	6688(3)	1552(10)	737(10)	83(4)

Table S33 Fractional Atomic Coordinates (×10^4) and Equivalent Isotropic Displacement Parameters (Å^2×10^3) for Er(OTf)$_3$/L$_6$. U$_{eq}$ is defined as 1/3 of the trace of the orthogonalised U$_{IJ}$ tensor.

**Atom**	*x*	*y*	*z*	**U(eq)**
C$^{(5)}$	5417(3)	5245(14)	507(15)	101(5)
O$^{(16)}$	7012(3)	7282(10)	823(13)	107(4)
C$^{(6)}$	5502(3)	4946(10)	1636(12)	77(4)
C$^{(29)}$	7189(3)	2164(14)	1968(16)	120(7)
F$^{(02Q)}$	6883(4)	4873(9)	-295(10)	134(5)
C$^{(28)}$	7205(4)	2010(17)	794(18)	147(9)
C$^{(27)}$	6955(4)	1710(14)	150(15)	118(6)
F$^{(9)}$	7311(3)	5491(13)	302(16)	164(5)
N$^{(200)}$	6219(3)	4402(7)	1235(10)	84(3)
C$^{(200)}$	6201(5)	3811(10)	546(13)	106(5)
C$^{(201)}$	6118(9)	3098(16)	-360(20)	236(18)
C$^{(47)}$	5399(2)	11409(10)	7412(13)	168(6)
C$^{(46)}$	5481(2)	10862(10)	8365(12)	154(5)
C$^{(45)}$	5778(2)	10572(7)	8495(8)	112(4)
C$^{(44)}$	5994.6(18)	10829(6)	7672(8)	76(3)
C$^{(49)}$	5913(3)	11377(7)	6719(8)	109(4)
C$^{(48)}$	5615(3)	11666(9)	6589(10)	143(5)
C$^{(63)}$	7008(4)	5549(15)	358(16)	90(4)
N$^{(7)}$	5580(13)	4240(30)	7260(40)	112(9)
C$^{(59)}$	5593(9)	3550(30)	8270(40)	122(9)
C$^{(60)}$	5530(10)	2920(30)	9240(30)	139(11)
N$^{(7A)}$	5023(14)	4870(40)	7040(20)	106(9)
C$^{(59A)}$	5341(8)	4500(20)	7160(30)	98(7)
C$^{(60A)}$	5614(14)	3980(40)	7510(60)	106(9)
C$^{(63A)}$	6972(9)	7100(20)	0(40)	87(4)
F$^{(9A)}$	7180(7)	6630(30)	700(30)	103(6)
F$^{(02R)}$	6984(17)	6720(40)	-1080(40)	91(8)
F$^{(01Z)}$	6714(7)	6950(20)	570(30)	91(6)
S$^{(0)}$	7069(4)	8295(14)	106(16)	118(4)
O$^{(16A)}$	7073(7)	8573(19)	1230(18)	66(6)
O$^{(13A)}$	6839(7)	8810(20)	-490(20)	77(7)
O$^{(12A)}$	7351(6)	8460(20)	-400(20)	76(7)

Table S34 Anisotropic Displacement Parameters (Å^2×10^3) for Er(OTf)$_3$/L$_6$. The Anisotropic displacement factor exponent takes the form: $-2\pi^2[h^2a^{*2}U_{11}+2hka^*b^*U_{12}+\dots]$.

**Atom**	**U$_{11}$**	**U$_{22}$**	**U$_{33}$**	**U$_{23}$**	**U$_{13}$**	**U$_{12}$**
Er$^{(1)}$	32.7(2)	28.6(2)	41.6(2)	-5.7(2)	1.9(2)	-1.71(18)

Table S34 Anisotropic Displacement Parameters (Å^2×10^3) for Er(OTf)$_3$/L$_6$. The Anisotropic displacement factor exponent takes the form: -2π^2[h^{2}a*^{2}U$_{11}$+2hka*b*U$_{12}$+…].

Atom	U_{11}	U_{22}	U_{33}	U_{23}	U_{13}	U_{12}
S$^{(1)}$	62.5(13)	30.3(9)	72.5(14)	-6.1(11)	-25.6(13)	7.0(8)
S$^{(3)}$	39.1(11)	55.3(13)	55.2(12)	-10.8(11)	6.2(9)	2.3(9)
S$^{(2)}$	69.5(15)	43.0(12)	54.2(13)	-18.7(10)	8.9(11)	-17.7(11)
S$^{(100)}$	80(2)	72(2)	119(3)	-41(2)	27.0(19)	-12.7(15)
S$^{(006)}$	67(2)	107(3)	34.9(15)	-15.3(18)	2.0(14)	33(2)
O$^{(10)}$	54(3)	35(3)	44(3)	1(3)	1(3)	-9(3)
O$^{(4)}$	30(3)	38(3)	59(4)	2(3)	1(2)	-2(2)
O$^{(9)}$	35(3)	45(3)	53(3)	-12(3)	5(3)	6(2)
F$^{(2)}$	68(4)	83(5)	109(6)	-50(4)	2(4)	-14(3)
O$^{(3)}$	41(3)	40(3)	75(5)	-11(3)	8(3)	-12(2)
N$^{(4)}$	47(4)	31(3)	39(4)	-1(3)	7(3)	-1(3)
O$^{(1W)}$	56(4)	38(3)	41(3)	-12(2)	-7(3)	-4(3)
N$^{(2)}$	30(3)	26(3)	40(3)	0(3)	-3(2)	-5(2)
F$^{(4)}$	54(3)	125(6)	87(4)	-5(4)	-25(3)	11(4)
F$^{(5)}$	66(4)	76(4)	129(6)	19(5)	-12(5)	6(3)
O$^{(7)}$	48(4)	66(4)	72(4)	-12(4)	10(3)	-10(3)
O$^{(11)}$	56(3)	53(3)	44(3)	-6(3)	12(3)	-1(4)
F$^{(1)}$	91(4)	58(4)	93(5)	-8(3)	-19(4)	34(3)
C$^{(18)}$	31(4)	29(4)	52(5)	-3(3)	-4(3)	1(3)
N$^{(6)}$	37(4)	31(3)	40(3)	-6(3)	-1(3)	-1(2)
N$^{(1)}$	37(3)	34(3)	39(3)	-12(3)	7(3)	-5(2)
C$^{(50)}$	41(4)	43(5)	37(4)	-3(3)	4(3)	-6(3)
O$^{(6)}$	108(6)	73(5)	58(4)	-21(4)	-6(5)	-14(4)
F$^{(7)}$	60(4)	55(4)	191(9)	-29(5)	2(5)	-4(3)
N$^{(3)}$	30(3)	44(4)	64(4)	-16(4)	6(4)	-8(3)
C$^{(17)}$	31(4)	36(4)	46(5)	4(3)	-2(3)	-5(3)
O$^{(1)}$	113(6)	34(3)	81(5)	16(4)	-17(4)	-7(4)
C$^{(15)}$	31(3)	34(4)	37(4)	-1(4)	1(3)	-4(3)
F$^{(102)}$	100(6)	74(5)	205(11)	-42(6)	41(7)	-15(5)
C$^{(39)}$	31(4)	34(4)	44(5)	-14(3)	3(3)	-4(3)
C$^{(41)}$	42(4)	49(5)	36(4)	-12(4)	7(4)	-4(4)
C$^{(40)}$	43(4)	34(4)	43(4)	-5(3)	2(3)	-4(3)
O$^{(2)}$	76(5)	46(4)	112(6)	-10(4)	-58(5)	15(3)
N$^{(5)}$	34(3)	30(3)	43(4)	-7(3)	2(3)	1(3)
O$^{(5)}$	99(6)	60(5)	100(6)	-35(4)	21(5)	-39(4)
C$^{(12)}$	51(4)	32(3)	36(4)	-1(4)	-7(3)	0(3)
C$^{(10)}$	40(4)	28(3)	40(4)	-4(3)	9(3)	-7(3)
C$^{(30)}$	44(4)	41(4)	39(4)	-3(4)	-1(3)	1(4)

Table S34 Anisotropic Displacement Parameters (Å^2×10^3) for Er(OTf)$_3$/L$_6$. The Anisotropic displacement factor exponent takes the form: -2π^2[h^{2}a*^{2}U$_{11}$+2hka*b*U$_{12}$+…].

Atom	U_{11}	U_{22}	U_{33}	U_{23}	U_{13}	U_{12}
$C^{(13)}$	39(4)	32(4)	40(4)	-9(4)	-7(4)	4(3)
$F^{(6)}$	79(5)	94(5)	181(9)	-61(6)	34(6)	23(4)
$C^{(36)}$	41(4)	42(4)	39(4)	-5(4)	0(3)	-8(4)
$F^{(3)}$	187(10)	140(8)	76(5)	4(5)	-32(6)	-59(7)
$C^{(42)}$	39(4)	38(5)	43(4)	-9(4)	6(3)	-3(3)
$C^{(9)}$	38(4)	35(4)	53(5)	-10(4)	2(4)	-12(3)
$C^{(11)}$	47(4)	33(3)	41(4)	1(4)	1(3)	-6(3)
$C^{(38)}$	34(4)	39(4)	39(4)	0(3)	2(3)	-1(3)
$O^{(12)}$	92(8)	89(9)	56(6)	-5(6)	20(6)	-5(7)
$C^{(21)}$	37(5)	56(6)	80(7)	-30(5)	1(4)	-6(4)
$O^{(8)}$	56(4)	136(8)	47(4)	-18(5)	2(3)	12(5)
$C^{(37)}$	60(6)	42(5)	43(5)	3(4)	4(4)	-11(4)
$C^{(52)}$	55(5)	34(4)	46(4)	-1(3)	9(4)	-7(4)
$C^{(14)}$	35(3)	31(3)	35(4)	-5(3)	1(3)	0(3)
$C^{(19)}$	41(4)	43(4)	53(5)	1(4)	-7(4)	-3(3)
$C^{(43)}$	38(4)	35(4)	42(4)	-9(4)	1(3)	2(3)
$O^{(102)}$	85(6)	87(6)	115(7)	-51(5)	15(5)	4(5)
$C^{(23)}$	39(4)	33(4)	74(7)	4(4)	0(4)	-7(3)
$C^{(53)}$	58(5)	35(4)	36(4)	2(3)	2(4)	-4(4)
$C^{(34)}$	53(5)	76(7)	43(5)	-10(5)	-10(4)	-5(5)
$C^{(16)}$	29(4)	46(5)	56(5)	3(4)	1(3)	-7(3)
$C^{(55)}$	106(8)	30(4)	47(5)	0(4)	-3(5)	8(5)
$C^{(33)}$	48(5)	78(7)	61(6)	-6(6)	-16(4)	-3(5)
$C^{(57)}$	55(6)	54(6)	63(6)	-12(5)	-8(5)	10(4)
$F^{(100)}$	111(6)	83(5)	177(10)	-27(6)	52(6)	13(5)
$C^{(20)}$	45(5)	62(6)	63(6)	-13(5)	-2(4)	-8(5)
$C^{(56)}$	75(7)	49(5)	55(6)	-2(4)	-10(5)	17(5)
$C^{(54)}$	81(7)	33(4)	51(5)	-2(4)	10(5)	-6(4)
$C^{(58)}$	61(6)	38(5)	55(5)	-4(4)	1(4)	-5(4)
$F^{(01Y)}$	128(9)	135(10)	62(5)	12(6)	-2(6)	19(7)
$C^{(24)}$	41(5)	51(5)	79(7)	-24(5)	-5(5)	7(4)
$O^{(101)}$	81(6)	113(7)	139(9)	-41(7)	-53(6)	-2(6)
$C^{(7)}$	34(4)	48(5)	85(8)	-19(5)	7(5)	-8(4)
$C^{(51)}$	69(6)	41(5)	54(5)	4(4)	17(5)	-6(4)
$C^{(2)}$	54(6)	68(7)	76(7)	-20(6)	5(5)	-21(5)
$F^{(101)}$	109(7)	232(15)	204(13)	-74(12)	-69(8)	10(9)
$C^{(32)}$	51(5)	77(7)	61(6)	-12(6)	-3(5)	7(5)
$C^{(31)}$	54(5)	57(6)	58(6)	-13(5)	3(4)	3(4)

Table S34 Anisotropic Displacement Parameters (Å^2×10^3) for Er(OTf)$_3$/L$_6$. The Anisotropic displacement factor exponent takes the form: -2π^2[h^{2}a*^{2}U$_{11}$+2hka*b*U$_{12}$+…].

Atom	U11	U22	U33	U23	U13	U12
$C^{(1)}$	34(4)	66(6)	74(7)	-20(5)	-4(4)	-16(4)
$O^{(13)}$	58(5)	120(8)	55(5)	12(6)	8(4)	26(6)
$C^{(35)}$	48(5)	51(5)	44(5)	0(4)	2(4)	-5(4)
$C^{(22)}$	52(6)	31(5)	98(9)	-5(5)	-4(6)	-6(4)
$C^{(3)}$	93(8)	77(8)	65(7)	2(7)	-15(6)	-23(7)
$C^{(4)}$	93(10)	109(12)	78(9)	-16(9)	-5(8)	-46(9)
$O^{(100)}$	211(13)	69(6)	87(7)	-7(5)	49(8)	27(7)
$C^{(8)}$	38(4)	51(5)	106(8)	-10(7)	8(6)	-10(4)
$C^{(02F)}$	41(5)	73(8)	122(11)	-30(7)	12(6)	4(5)
$C^{(100)}$	69(8)	105(13)	148(15)	-52(12)	-1(9)	10(8)
$C^{(25)}$	62(6)	58(6)	85(7)	-36(6)	-10(5)	-5(5)
$C^{(26)}$	102(9)	81(8)	65(7)	-33(7)	1(6)	-15(7)
$C^{(5)}$	75(9)	138(15)	90(10)	-48(10)	10(8)	-25(9)
$O^{(16)}$	120(8)	112(8)	89(7)	-6(7)	-3(7)	-15(7)
$C^{(6)}$	48(6)	93(9)	91(9)	-28(7)	7(6)	-2(6)
$C^{(29)}$	48(7)	182(17)	131(13)	-87(13)	7(8)	-7(8)
$F^{(02Q)}$	230(13)	100(8)	70(6)	-14(6)	7(8)	25(8)
$C^{(28)}$	88(11)	220(20)	129(15)	-80(16)	46(11)	-22(13)
$C^{(27)}$	115(13)	143(16)	96(11)	-51(12)	17(10)	-15(12)
$F^{(9)}$	93(7)	203(13)	196(12)	61(11)	36(8)	52(8)
$N^{(200)}$	91(7)	57(5)	103(8)	-24(5)	21(7)	-19(5)
$C^{(200)}$	168(16)	69(8)	81(9)	-2(7)	15(11)	9(10)
$C^{(201)}$	470(60)	115(17)	119(16)	-30(13)	110(30)	-40(20)
$C^{(47)}$	149(10)	171(11)	184(12)	21(10)	10(9)	69(10)
$C^{(46)}$	125(9)	160(11)	176(11)	24(9)	40(9)	58(9)
$C^{(45)}$	102(8)	101(8)	131(9)	10(7)	43(7)	36(7)
$C^{(44)}$	101(7)	50(5)	77(6)	-21(5)	17(5)	18(5)
$C^{(49)}$	144(9)	83(8)	102(8)	-10(6)	2(7)	51(7)
$C^{(48)}$	164(10)	130(10)	134(10)	2(9)	-6(8)	70(9)
$C^{(63)}$	93(7)	110(8)	67(7)	-7(6)	21(7)	30(7)
$N^{(7)}$	100(15)	110(20)	122(17)	-33(13)	20(16)	-38(16)
$C^{(59)}$	138(17)	110(20)	117(18)	-41(14)	6(17)	-2(16)
$C^{(60)}$	180(30)	130(20)	110(20)	-29(16)	10(20)	40(20)
$N^{(7A)}$	105(14)	110(20)	106(12)	-33(16)	6(18)	-23(13)
$C^{(59A)}$	95(14)	101(18)	97(13)	-39(13)	6(15)	-29(14)
$C^{(60A)}$	102(14)	100(20)	113(19)	-35(16)	18(16)	-25(16)
$C^{(63A)}$	83(8)	115(8)	62(8)	-11(8)	4(8)	10(8)
$F^{(9A)}$	89(11)	133(12)	87(11)	11(11)	1(10)	13(11)

Table S34 Anisotropic Displacement Parameters (Å^2 × 10^3) for Er(OTf)$_3$/L$_6$. The Anisotropic displacement factor exponent takes the form: -2π^2[h^{2}a*^{2}U$_{11}$+2hka*b*U$_{12}$+…].

Atom	U$_{11}$	U$_{22}$	U$_{33}$	U$_{23}$	U$_{13}$	U$_{12}$
F$^{(02R)}$	90(14)	109(15)	72(12)	-14(13)	5(12)	6(14)
F$^{(01Z)}$	79(11)	113(13)	82(12)	-8(12)	-3(10)	6(11)
S$^{(0)}$	124(9)	131(9)	98(8)	-2(8)	3(7)	-1(8)
O$^{(16A)}$	86(14)	66(13)	45(10)	34(9)	7(10)	-5(12)
O$^{(13A)}$	94(16)	98(17)	39(13)	-13(13)	-6(12)	-9(14)
O$^{(12A)}$	95(15)	77(17)	57(14)	-3(15)	12(12)	2(15)

Table S35 Bond Lengths for Er(OTf)$_3$/L$_6$.

Atom	Atom	Length/Å	Atom	Atom	Length/Å
Er$^{(1)}$	O$^{(9)}$	2.308(5)	C$^{(10)}$	C$^{(9)}$	1.461(11)
Er$^{(1)}$	N$^{(4)}$	2.487(7)	C$^{(10)}$	C$^{(11)}$	1.394(11)
Er$^{(1)}$	O$^{(1W)}$	2.307(5)	C$^{(30)}$	C$^{(36)}$	1.519(10)
Er$^{(1)}$	N$^{(2)}$	2.510(5)	C$^{(30)}$	C$^{(31)}$	1.372(12)
Er$^{(1)}$	N$^{(6)}$	2.476(7)	C$^{(30)}$	C$^{(35)}$	1.371(12)
Er$^{(1)}$	N$^{(1)}$	2.497(6)	C$^{(13)}$	C$^{(14)}$	1.391(10)
Er$^{(1)}$	N$^{(3)}$	2.477(6)	F$^{(6)}$	C$^{(02F)}$	1.297(14)
Er$^{(1)}$	N$^{(5)}$	2.487(7)	C$^{(36)}$	C$^{(37)}$	1.554(12)
S$^{(1)}$	O$^{(1)}$	1.416(8)	F$^{(3)}$	C$^{(4)}$	1.377(17)
S$^{(1)}$	O$^{(2)}$	1.434(7)	C$^{(42)}$	C$^{(43)}$	1.385(11)
S$^{(1)}$	C$^{(12)}$	1.795(8)	C$^{(21)}$	C$^{(20)}$	1.384(16)
S$^{(1)}$	C$^{(24)}$	1.747(11)	C$^{(21)}$	C$^{(22)}$	1.316(16)
S$^{(3)}$	O$^{(9)}$	1.452(6)	C$^{(52)}$	C$^{(53)}$	1.490(13)
S$^{(3)}$	O$^{(7)}$	1.430(7)	C$^{(52)}$	C$^{(51)}$	1.543(12)
S$^{(3)}$	O$^{(8)}$	1.426(8)	C$^{(19)}$	C$^{(20)}$	1.372(13)
S$^{(3)}$	C$^{(02F)}$	1.847(14)	C$^{(23)}$	C$^{(22)}$	1.409(14)
S$^{(2)}$	O$^{(6)}$	1.416(9)	C$^{(53)}$	C$^{(54)}$	1.394(12)
S$^{(2)}$	C$^{(41)}$	1.781(9)	C$^{(53)}$	C$^{(58)}$	1.401(13)
S$^{(2)}$	O$^{(5)}$	1.413(8)	C$^{(34)}$	C$^{(33)}$	1.358(15)
S$^{(2)}$	C$^{(44)}$	1.705(8)	C$^{(34)}$	C$^{(35)}$	1.405(13)
S$^{(100)}$	O$^{(102)}$	1.420(8)	C$^{(55)}$	C$^{(56)}$	1.374(16)
S$^{(100)}$	O$^{(101)}$	1.527(10)	C$^{(55)}$	C$^{(54)}$	1.408(15)
S$^{(100)}$	O$^{(100)}$	1.405(11)	C$^{(33)}$	C$^{(32)}$	1.391(15)
S$^{(100)}$	C$^{(100)}$	1.792(17)	C$^{(57)}$	C$^{(56)}$	1.359(15)
S$^{(006)}$	O$^{(12)}$	1.451(16)	C$^{(57)}$	C$^{(58)}$	1.385(13)
S$^{(006)}$	O$^{(13)}$	1.472(11)	F$^{(100)}$	C$^{(100)}$	1.331(17)
S$^{(006)}$	O$^{(16)}$	1.322(14)	F$^{(01Y)}$	C$^{(63)}$	1.32(2)
S$^{(006)}$	C$^{(63)}$	1.84(2)	C$^{(24)}$	C$^{(25)}$	1.366(13)
O$^{(10)}$	C$^{(38)}$	1.343(10)	C$^{(24)}$	C$^{(29)}$	1.402(18)

Table S35 Bond Lengths for Er(OTf)$_3$/L$_6$.

Atom	Atom	Length/Å	Atom	Atom	Length/Å
O$^{(10)}$	C$^{(37)}$	1.474(11)	C$^{(7)}$	C$^{(1)}$	1.506(16)
O$^{(4)}$	C$^{(15)}$	1.331(9)	C$^{(7)}$	C$^{(8)}$	1.548(13)
O$^{(4)}$	C$^{(16)}$	1.447(10)	C$^{(2)}$	C$^{(1)}$	1.396(16)
F$^{(2)}$	C$^{(21)}$	1.343(11)	C$^{(2)}$	C$^{(3)}$	1.380(16)
O$^{(3)}$	C$^{(9)}$	1.331(9)	F$^{(101)}$	C$^{(100)}$	1.32(2)
O$^{(3)}$	C$^{(8)}$	1.457(11)	C$^{(32)}$	C$^{(31)}$	1.375(14)
N$^{(4)}$	C$^{(50)}$	1.282(10)	C$^{(1)}$	C$^{(6)}$	1.390(16)
N$^{(4)}$	C$^{(52)}$	1.501(10)	C$^{(3)}$	C$^{(4)}$	1.335(19)
N$^{(2)}$	C$^{(17)}$	1.513(9)	C$^{(4)}$	C$^{(5)}$	1.38(2)
N$^{(2)}$	C$^{(15)}$	1.267(9)	C$^{(25)}$	C$^{(26)}$	1.381(16)
F$^{(4)}$	C$^{(33)}$	1.358(11)	C$^{(26)}$	C$^{(27)}$	1.38(2)
F$^{(5)}$	C$^{(02F)}$	1.326(17)	C$^{(5)}$	C$^{(6)}$	1.41(2)
O$^{(11)}$	C$^{(50)}$	1.334(10)	C$^{(29)}$	C$^{(28)}$	1.36(2)
O$^{(11)}$	C$^{(51)}$	1.480(11)	F$^{(02Q)}$	C$^{(63)}$	1.35(2)
F$^{(1)}$	C$^{(56)}$	1.360(11)	C$^{(28)}$	C$^{(27)}$	1.40(2)
C$^{(18)}$	C$^{(17)}$	1.505(11)	F$^{(9)}$	C$^{(63)}$	1.35(2)
C$^{(18)}$	C$^{(19)}$	1.390(13)	N$^{(200)}$	C$^{(200)}$	1.161(16)
C$^{(18)}$	C$^{(23)}$	1.398(11)	C$^{(200)}$	C$^{(201)}$	1.50(3)
N$^{(6)}$	C$^{(36)}$	1.486(10)	C$^{(47)}$	C$^{(46)}$	1.3900
N$^{(6)}$	C$^{(38)}$	1.276(10)	C$^{(47)}$	C$^{(48)}$	1.3900
N$^{(1)}$	C$^{(10)}$	1.344(9)	C$^{(46)}$	C$^{(45)}$	1.3900
N$^{(1)}$	C$^{(14)}$	1.328(9)	C$^{(45)}$	C$^{(44)}$	1.3900
C$^{(50)}$	C$^{(43)}$	1.459(12)	C$^{(44)}$	C$^{(49)}$	1.3900
F$^{(7)}$	C$^{(02F)}$	1.335(13)	C$^{(49)}$	C$^{(48)}$	1.3900
N$^{(3)}$	C$^{(9)}$	1.275(10)	N$^{(7)}$	C$^{(59)}$	1.52(5)
N$^{(3)}$	C$^{(7)}$	1.469(10)	C$^{(59)}$	C$^{(60)}$	1.46(5)
C$^{(17)}$	C$^{(16)}$	1.566(11)	N$^{(7A)}$	N$^{(7A)1}$	0.41(10)
C$^{(15)}$	C$^{(14)}$	1.469(10)	N$^{(7A)}$	C$^{(59A)}$	1.52(6)
F$^{(102)}$	C$^{(100)}$	1.31(2)	N$^{(7A)}$	C$^{(59A)1}$	1.85(7)
C$^{(39)}$	C$^{(40)}$	1.392(11)	C$^{(59A)}$	C$^{(60A)}$	1.48(5)
C$^{(39)}$	N$^{(5)}$	1.335(10)	C$^{(63A)}$	F$^{(9A)}$	1.40(3)
C$^{(39)}$	C$^{(38)}$	1.472(12)	C$^{(63A)}$	F$^{(02R)}$	1.34(3)
C$^{(41)}$	C$^{(40)}$	1.397(12)	C$^{(63A)}$	F$^{(01Z)}$	1.33(3)
C$^{(41)}$	C$^{(42)}$	1.385(12)	C$^{(63A)}$	S$^{(0)}$	1.78(3)
N$^{(5)}$	C$^{(43)}$	1.339(10)	S$^{(0)}$	O$^{(16A)}$	1.34(2)
C$^{(12)}$	C$^{(13)}$	1.377(11)	S$^{(0)}$	O$^{(13A)}$	1.43(2)
C$^{(12)}$	C$^{(11)}$	1.389(11)	S$^{(0)}$	O$^{(12A)}$	1.40(2)

[1] 1-X,1-Y,+Z

Table S36 Bond Angles for Er(OTf)$_3$/L$_6$.

Atom	Atom	Atom	Angle/˚	Atom	Atom	Atom	Angle/˚
O(9)	Er(1)	N(4)	85.0(2)	C(30)	C(36)	C(37)	111.3(8)
O(9)	Er(1)	N(2)	153.30(19)	C(43)	C(42)	C(41)	117.5(8)
O(9)	Er(1)	N(6)	85.0(2)	O(3)	C(9)	C(10)	118.0(7)
O(9)	Er(1)	N(1)	135.2(2)	N(3)	C(9)	O(3)	119.3(7)
O(9)	Er(1)	N(3)	74.1(2)	N(3)	C(9)	C(10)	122.7(6)
O(9)	Er(1)	N(5)	73.7(2)	C(12)	C(11)	C(10)	116.3(7)
N(4)	Er(1)	N(2)	87.0(2)	O(10)	C(38)	C(39)	117.4(7)
N(4)	Er(1)	N(1)	73.0(2)	N(6)	C(38)	O(10)	119.8(7)
O(1W)	Er(1)	O(9)	114.7(2)	N(6)	C(38)	C(39)	122.8(8)
O(1W)	Er(1)	N(4)	148.84(19)	F(2)	C(21)	C(20)	118.2(11)
O(1W)	Er(1)	N(2)	84.2(2)	C(22)	C(21)	F(2)	118.7(10)
O(1W)	Er(1)	N(6)	76.9(2)	C(22)	C(21)	C(20)	123.1(9)
O(1W)	Er(1)	N(1)	76.1(2)	O(10)	C(37)	C(36)	104.0(6)
O(1W)	Er(1)	N(3)	79.3(3)	N(4)	C(52)	C(51)	103.0(6)
O(1W)	Er(1)	N(5)	140.9(2)	C(53)	C(52)	N(4)	112.2(7)
N(6)	Er(1)	N(4)	131.0(2)	C(53)	C(52)	C(51)	113.5(8)
N(6)	Er(1)	N(2)	81.1(2)	N(1)	C(14)	C(15)	113.3(6)
N(6)	Er(1)	N(1)	138.5(2)	N(1)	C(14)	C(13)	123.1(6)
N(6)	Er(1)	N(3)	137.8(2)	C(13)	C(14)	C(15)	123.5(6)
N(6)	Er(1)	N(5)	65.6(2)	C(20)	C(19)	C(18)	120.5(9)
N(1)	Er(1)	N(2)	65.28(18)	N(5)	C(43)	C(50)	112.6(7)
N(3)	Er(1)	N(4)	83.9(3)	N(5)	C(43)	C(42)	123.0(8)
N(3)	Er(1)	N(2)	130.2(2)	C(42)	C(43)	C(50)	124.3(8)
N(3)	Er(1)	N(1)	65.2(2)	C(18)	C(23)	C(22)	119.8(9)
N(3)	Er(1)	N(5)	137.0(2)	C(54)	C(53)	C(52)	119.7(8)
N(5)	Er(1)	N(4)	65.5(2)	C(54)	C(53)	C(58)	118.2(9)
N(5)	Er(1)	N(2)	79.8(2)	C(58)	C(53)	C(52)	121.8(8)
N(5)	Er(1)	N(1)	126.4(2)	C(33)	C(34)	C(35)	117.2(9)
O(1)	S(1)	O(2)	120.0(5)	O(4)	C(16)	C(17)	104.5(6)
O(1)	S(1)	C(12)	106.5(4)	C(56)	C(55)	C(54)	117.6(9)
O(1)	S(1)	C(24)	110.0(5)	F(4)	C(33)	C(34)	119.0(9)
O(2)	S(1)	C(12)	106.5(4)	F(4)	C(33)	C(32)	117.5(10)
O(2)	S(1)	C(24)	109.5(5)	C(34)	C(33)	C(32)	123.3(9)
C(24)	S(1)	C(12)	102.8(4)	C(56)	C(57)	C(58)	118.3(10)
O(9)	S(3)	C(02F)	102.4(5)	C(19)	C(20)	C(21)	118.8(10)
O(7)	S(3)	O(9)	113.5(4)	F(1)	C(56)	C(55)	117.5(9)
O(7)	S(3)	C(02F)	103.2(5)	C(57)	C(56)	F(1)	118.8(10)
O(8)	S(3)	O(9)	112.5(4)	C(57)	C(56)	C(55)	123.6(10)
O(8)	S(3)	O(7)	118.2(5)	C(53)	C(54)	C(55)	120.9(10)
O(8)	S(3)	C(02F)	104.8(6)	C(57)	C(58)	C(53)	121.4(9)

Table S36 Bond Angles for Er(OTf)$_3$/L$_6$.

Atom	Atom	Atom	Angle/°	Atom	Atom	Atom	Angle/°
O$^{(6)}$	S$^{(2)}$	C$^{(41)}$	106.9(5)	C$^{(25)}$	C$^{(24)}$	S$^{(1)}$	119.7(8)
O$^{(6)}$	S$^{(2)}$	C$^{(44)}$	109.6(5)	C$^{(25)}$	C$^{(24)}$	C$^{(29)}$	119.3(11)
O$^{(5)}$	S$^{(2)}$	O$^{(6)}$	119.2(6)	C$^{(29)}$	C$^{(24)}$	S$^{(1)}$	120.9(9)
O$^{(5)}$	S$^{(2)}$	C$^{(41)}$	107.1(4)	N$^{(3)}$	C$^{(7)}$	C$^{(1)}$	111.2(8)
O$^{(5)}$	S$^{(2)}$	C$^{(44)}$	110.4(6)	N$^{(3)}$	C$^{(7)}$	C$^{(8)}$	103.5(7)
C$^{(44)}$	S$^{(2)}$	C$^{(41)}$	102.1(4)	C$^{(1)}$	C$^{(7)}$	C$^{(8)}$	114.7(9)
O$^{(102)}$	S$^{(100)}$	O$^{(101)}$	114.6(7)	O$^{(11)}$	C$^{(51)}$	C$^{(52)}$	103.2(7)
O$^{(102)}$	S$^{(100)}$	C$^{(100)}$	106.4(7)	C$^{(3)}$	C$^{(2)}$	C$^{(1)}$	120.6(11)
O$^{(101)}$	S$^{(100)}$	C$^{(100)}$	98.2(8)	C$^{(31)}$	C$^{(32)}$	C$^{(33)}$	117.5(9)
O$^{(100)}$	S$^{(100)}$	O$^{(102)}$	115.0(7)	C$^{(30)}$	C$^{(31)}$	C$^{(32)}$	121.1(9)
O$^{(100)}$	S$^{(100)}$	O$^{(101)}$	115.2(7)	C$^{(2)}$	C$^{(1)}$	C$^{(7)}$	121.4(9)
O$^{(100)}$	S$^{(100)}$	C$^{(100)}$	104.9(9)	C$^{(6)}$	C$^{(1)}$	C$^{(7)}$	118.9(11)
O$^{(12)}$	S$^{(006)}$	O$^{(13)}$	112.6(11)	C$^{(6)}$	C$^{(1)}$	C$^{(2)}$	119.8(12)
O$^{(12)}$	S$^{(006)}$	C$^{(63)}$	106.3(10)	C$^{(30)}$	C$^{(35)}$	C$^{(34)}$	120.8(9)
O$^{(13)}$	S$^{(006)}$	C$^{(63)}$	98.1(8)	C$^{(21)}$	C$^{(22)}$	C$^{(23)}$	119.1(9)
O$^{(16)}$	S$^{(006)}$	O$^{(12)}$	119.0(11)	C$^{(4)}$	C$^{(3)}$	C$^{(2)}$	119.2(14)
O$^{(16)}$	S$^{(006)}$	O$^{(13)}$	111.7(8)	F$^{(3)}$	C$^{(4)}$	C$^{(5)}$	117.8(15)
O$^{(16)}$	S$^{(006)}$	C$^{(63)}$	106.6(10)	C$^{(3)}$	C$^{(4)}$	F$^{(3)}$	119.5(17)
C$^{(38)}$	O$^{(10)}$	C$^{(37)}$	105.4(6)	C$^{(3)}$	C$^{(4)}$	C$^{(5)}$	122.7(15)
C$^{(15)}$	O$^{(4)}$	C$^{(16)}$	106.2(6)	O$^{(3)}$	C$^{(8)}$	C$^{(7)}$	104.0(6)
S$^{(3)}$	O$^{(9)}$	Er$^{(1)}$	150.8(4)	F$^{(5)}$	C$^{(02F)}$	S$^{(3)}$	109.8(8)
C$^{(9)}$	O$^{(3)}$	C$^{(8)}$	105.8(6)	F$^{(5)}$	C$^{(02F)}$	F$^{(7)}$	108.1(12)
C$^{(50)}$	N$^{(4)}$	Er$^{(1)}$	115.6(5)	F$^{(7)}$	C$^{(02F)}$	S$^{(3)}$	109.1(8)
C$^{(50)}$	N$^{(4)}$	C$^{(52)}$	105.8(7)	F$^{(6)}$	C$^{(02F)}$	S$^{(3)}$	109.8(11)
C$^{(52)}$	N$^{(4)}$	Er$^{(1)}$	137.0(5)	F$^{(6)}$	C$^{(02F)}$	F$^{(5)}$	110.3(11)
C$^{(17)}$	N$^{(2)}$	Er$^{(1)}$	136.0(4)	F$^{(6)}$	C$^{(02F)}$	F$^{(7)}$	109.7(10)
C$^{(15)}$	N$^{(2)}$	Er$^{(1)}$	117.0(4)	F$^{(102)}$	C$^{(100)}$	S$^{(100)}$	112.8(11)
C$^{(15)}$	N$^{(2)}$	C$^{(17)}$	106.2(6)	F$^{(102)}$	C$^{(100)}$	F$^{(100)}$	107.3(16)
C$^{(50)}$	O$^{(11)}$	C$^{(51)}$	105.1(6)	F$^{(102)}$	C$^{(100)}$	F$^{(101)}$	106.2(15)
C$^{(19)}$	C$^{(18)}$	C$^{(17)}$	122.3(7)	F$^{(100)}$	C$^{(100)}$	S$^{(100)}$	111.2(11)
C$^{(19)}$	C$^{(18)}$	C$^{(23)}$	118.7(8)	F$^{(101)}$	C$^{(100)}$	S$^{(100)}$	108.2(15)
C$^{(23)}$	C$^{(18)}$	C$^{(17)}$	118.9(8)	F$^{(101)}$	C$^{(100)}$	F$^{(100)}$	111.2(14)
C$^{(36)}$	N$^{(6)}$	Er$^{(1)}$	135.4(5)	C$^{(24)}$	C$^{(25)}$	C$^{(26)}$	121.2(11)
C$^{(38)}$	N$^{(6)}$	Er$^{(1)}$	117.6(5)	C$^{(27)}$	C$^{(26)}$	C$^{(25)}$	120.0(13)
C$^{(38)}$	N$^{(6)}$	C$^{(36)}$	106.8(7)	C$^{(4)}$	C$^{(5)}$	C$^{(6)}$	119.2(14)
C$^{(10)}$	N$^{(1)}$	Er$^{(1)}$	120.4(5)	C$^{(1)}$	C$^{(6)}$	C$^{(5)}$	118.4(13)
C$^{(14)}$	N$^{(1)}$	Er$^{(1)}$	120.8(4)	C$^{(28)}$	C$^{(29)}$	C$^{(24)}$	119.3(13)

Table S36 Bond Angles for Er(OTf)$_3$/L$_6$.

Atom	Atom	Atom	Angle/˚	Atom	Atom	Atom	Angle/˚
$C^{(14)}$	$N^{(1)}$	$C^{(10)}$	118.8(6)	$C^{(29)}$	$C^{(28)}$	$C^{(27)}$	121.6(15)
$N^{(4)}$	$C^{(50)}$	$O^{(11)}$	119.7(7)	$C^{(26)}$	$C^{(27)}$	$C^{(28)}$	118.5(15)
$N^{(4)}$	$C^{(50)}$	$C^{(43)}$	123.6(7)	$N^{(200)}$	$C^{(200)}$	$C^{(201)}$	169(2)
$O^{(11)}$	$C^{(50)}$	$C^{(43)}$	116.7(7)	$C^{(46)}$	$C^{(47)}$	$C^{(48)}$	120.0
$C^{(9)}$	$N^{(3)}$	$Er^{(1)}$	118.2(5)	$C^{(47)}$	$C^{(46)}$	$C^{(45)}$	120.0
$C^{(9)}$	$N^{(3)}$	$C^{(7)}$	106.6(7)	$C^{(44)}$	$C^{(45)}$	$C^{(46)}$	120.0
$C^{(7)}$	$N^{(3)}$	$Er^{(1)}$	135.2(5)	$C^{(45)}$	$C^{(44)}$	$S^{(2)}$	118.3(6)
$N^{(2)}$	$C^{(17)}$	$C^{(16)}$	102.2(6)	$C^{(45)}$	$C^{(44)}$	$C^{(49)}$	120.0
$C^{(18)}$	$C^{(17)}$	$N^{(2)}$	114.0(6)	$C^{(49)}$	$C^{(44)}$	$S^{(2)}$	121.5(6)
$C^{(18)}$	$C^{(17)}$	$C^{(16)}$	112.5(6)	$C^{(48)}$	$C^{(49)}$	$C^{(44)}$	120.0
$O^{(4)}$	$C^{(15)}$	$C^{(14)}$	115.8(6)	$C^{(49)}$	$C^{(48)}$	$C^{(47)}$	120.0
$N^{(2)}$	$C^{(15)}$	$O^{(4)}$	120.5(6)	$F^{(01Y)}$	$C^{(63)}$	$S^{(006)}$	113.2(13)
$N^{(2)}$	$C^{(15)}$	$C^{(14)}$	123.6(6)	$F^{(01Y)}$	$C^{(63)}$	$F^{(02Q)}$	104.3(18)
$C^{(40)}$	$C^{(39)}$	$C^{(38)}$	124.3(8)	$F^{(01Y)}$	$C^{(63)}$	$F^{(9)}$	103.0(17)
$N^{(5)}$	$C^{(39)}$	$C^{(40)}$	123.0(8)	$F^{(02Q)}$	$C^{(63)}$	$S^{(006)}$	116.2(14)
$N^{(5)}$	$C^{(39)}$	$C^{(38)}$	112.7(7)	$F^{(02Q)}$	$C^{(63)}$	$F^{(9)}$	109.9(17)
$C^{(40)}$	$C^{(41)}$	$S^{(2)}$	120.7(7)	$F^{(9)}$	$C^{(63)}$	$S^{(006)}$	109.5(16)
$C^{(42)}$	$C^{(41)}$	$S^{(2)}$	118.6(6)	$C^{(60)}$	$C^{(59)}$	$N^{(7)}$	166(4)
$C^{(42)}$	$C^{(41)}$	$C^{(40)}$	120.7(8)	$N^{(7A)1}$	$N^{(7A)}$	$C^{(59A)}$	139(10)
$C^{(39)}$	$C^{(40)}$	$C^{(41)}$	116.9(8)	$N^{(7A)1}$	$N^{(7A)}$	$C^{(59A)1}$	32(10)
$C^{(39)}$	$N^{(5)}$	$Er^{(1)}$	120.6(5)	$C^{(59A)}$	$N^{(7A)}$	$C^{(59A)1}$	167(3)
$C^{(39)}$	$N^{(5)}$	$C^{(43)}$	118.8(7)	$C^{(60A)}$	$C^{(59A)}$	$N^{(7A)}$	165(5)
$C^{(43)}$	$N^{(5)}$	$Er^{(1)}$	120.1(6)	$F^{(9A)}$	$C^{(63A)}$	$S^{(0)}$	105(3)
$C^{(13)}$	$C^{(12)}$	$S^{(1)}$	119.5(6)	$F^{(02R)}$	$C^{(63A)}$	$F^{(9A)}$	108(4)
$C^{(13)}$	$C^{(12)}$	$C^{(11)}$	121.8(7)	$F^{(02R)}$	$C^{(63A)}$	$S^{(0)}$	117(3)
$C^{(11)}$	$C^{(12)}$	$S^{(1)}$	118.7(6)	$F^{(01Z)}$	$C^{(63A)}$	$F^{(9A)}$	102(3)
$N^{(1)}$	$C^{(10)}$	$C^{(9)}$	112.9(6)	$F^{(01Z)}$	$C^{(63A)}$	$F^{(02R)}$	114(4)
$N^{(1)}$	$C^{(10)}$	$C^{(11)}$	123.0(7)	$F^{(01Z)}$	$C^{(63A)}$	$S^{(0)}$	109(3)
$C^{(11)}$	$C^{(10)}$	$C^{(9)}$	124.1(6)	$O^{(16A)}$	$S^{(0)}$	$C^{(63A)}$	111(2)
$C^{(31)}$	$C^{(30)}$	$C^{(36)}$	120.7(8)	$O^{(16A)}$	$S^{(0)}$	$O^{(13A)}$	108(2)
$C^{(35)}$	$C^{(30)}$	$C^{(36)}$	119.1(7)	$O^{(16A)}$	$S^{(0)}$	$O^{(12A)}$	109(2)
$C^{(35)}$	$C^{(30)}$	$C^{(31)}$	119.9(8)	$O^{(13A)}$	$S^{(0)}$	$C^{(63A)}$	107(2)
$C^{(12)}$	$C^{(13)}$	$C^{(14)}$	117.0(7)	$O^{(12A)}$	$S^{(0)}$	$C^{(63A)}$	111(2)
$N^{(6)}$	$C^{(36)}$	$C^{(30)}$	112.9(6)	$O^{(12A)}$	$S^{(0)}$	$O^{(13A)}$	111(2)
$N^{(6)}$	$C^{(36)}$	$C^{(37)}$	103.6(6)				

[1] 1-X,1-Y,+Z

Table S37 Torsion Angles for Er(OTf)$_3$/L$_6$.

A	B	C	D	Angle/˚	A	B	C	D	Angle/˚
Er$^{(1)}$	N$^{(4)}$	C$^{(50)}$	O$^{(11)}$	164.1(6)	C$^{(11)}$	C$^{(12)}$	C$^{(13)}$	C$^{(14)}$	0.6(13)
Er$^{(1)}$	N$^{(4)}$	C$^{(50)}$	C$^{(43)}$	-17.5(10)	C$^{(11)}$	C$^{(10)}$	C$^{(9)}$	O$^{(3)}$	8.1(14)
Er$^{(1)}$	N$^{(4)}$	C$^{(52)}$	C$^{(53)}$	86.9(9)	C$^{(11)}$	C$^{(10)}$	C$^{(9)}$	N$^{(3)}$	-172.5(9)
Er$^{(1)}$	N$^{(4)}$	C$^{(52)}$	C$^{(51)}$	-150.7(6)	C$^{(38)}$	O$^{(10)}$	C$^{(37)}$	C$^{(36)}$	6.1(9)
Er$^{(1)}$	N$^{(2)}$	C$^{(17)}$	C$^{(18)}$	72.3(9)	C$^{(38)}$	N$^{(6)}$	C$^{(36)}$	C$^{(30)}$	-115.0(8)
Er$^{(1)}$	N$^{(2)}$	C$^{(17)}$	C$^{(16)}$	-166.0(5)	C$^{(38)}$	N$^{(6)}$	C$^{(36)}$	C$^{(37)}$	5.5(8)
Er$^{(1)}$	N$^{(2)}$	C$^{(15)}$	O$^{(4)}$	172.6(6)	C$^{(38)}$	C$^{(39)}$	C$^{(40)}$	C$^{(41)}$	174.2(8)
Er$^{(1)}$	N$^{(2)}$	C$^{(15)}$	C$^{(14)}$	-2.5(10)	C$^{(38)}$	C$^{(39)}$	N$^{(5)}$	Er$^{(1)}$	-4.9(9)
Er$^{(1)}$	N$^{(6)}$	C$^{(36)}$	C$^{(30)}$	70.8(9)	C$^{(38)}$	C$^{(39)}$	N$^{(5)}$	C$^{(43)}$	-177.6(7)
Er$^{(1)}$	N$^{(6)}$	C$^{(36)}$	C$^{(37)}$	-168.7(6)	O$^{(12)}$	S$^{(006)}$	C$^{(63)}$	F$^{(01Y)}$	-178.9(17)
Er$^{(1)}$	N$^{(6)}$	C$^{(38)}$	O$^{(10)}$	173.6(5)	O$^{(12)}$	S$^{(006)}$	C$^{(63)}$	F$^{(02Q)}$	-58.3(19)
Er$^{(1)}$	N$^{(6)}$	C$^{(38)}$	C$^{(39)}$	-8.7(11)	O$^{(12)}$	S$^{(006)}$	C$^{(63)}$	F$^{(9)}$	66.9(18)
Er$^{(1)}$	N$^{(1)}$	C$^{(10)}$	C$^{(9)}$	0.1(10)	O$^{(8)}$	S$^{(3)}$	O$^{(9)}$	Er$^{(1)}$	17.3(10)
Er$^{(1)}$	N$^{(1)}$	C$^{(10)}$	C$^{(11)}$	178.8(6)	O$^{(8)}$	S$^{(3)}$	C$^{(02F)}$	F$^{(5)}$	179.0(7)
Er$^{(1)}$	N$^{(1)}$	C$^{(14)}$	C$^{(15)}$	-2.0(10)	O$^{(8)}$	S$^{(3)}$	C$^{(02F)}$	F$^{(7)}$	60.8(11)
Er$^{(1)}$	N$^{(1)}$	C$^{(14)}$	C$^{(13)}$	-178.5(6)	O$^{(8)}$	S$^{(3)}$	C$^{(02F)}$	F$^{(6)}$	-59.5(10)
Er$^{(1)}$	N$^{(3)}$	C$^{(9)}$	O$^{(3)}$	170.1(6)	C$^{(37)}$	O$^{(10)}$	C$^{(38)}$	N$^{(6)}$	-3.0(10)
Er$^{(1)}$	N$^{(3)}$	C$^{(9)}$	C$^{(10)}$	-9.3(13)	C$^{(37)}$	O$^{(10)}$	C$^{(38)}$	C$^{(39)}$	179.1(7)
Er$^{(1)}$	N$^{(3)}$	C$^{(7)}$	C$^{(1)}$	65.5(11)	C$^{(52)}$	N$^{(4)}$	C$^{(50)}$	O$^{(11)}$	-3.7(10)
Er$^{(1)}$	N$^{(3)}$	C$^{(7)}$	C$^{(8)}$	-170.9(8)	C$^{(52)}$	N$^{(4)}$	C$^{(50)}$	C$^{(43)}$	174.6(8)
Er$^{(1)}$	N$^{(5)}$	C$^{(43)}$	C$^{(50)}$	6.1(9)	C$^{(52)}$	C$^{(53)}$	C$^{(54)}$	C$^{(55)}$	-174.3(8)
Er$^{(1)}$	N$^{(5)}$	C$^{(43)}$	C$^{(42)}$	-169.9(6)	C$^{(52)}$	C$^{(53)}$	C$^{(58)}$	C$^{(57)}$	175.1(9)
S$^{(1)}$	C$^{(12)}$	C$^{(13)}$	C$^{(14)}$	177.9(7)	C$^{(14)}$	N$^{(1)}$	C$^{(10)}$	C$^{(9)}$	-178.1(8)
S$^{(1)}$	C$^{(12)}$	C$^{(11)}$	C$^{(10)}$	-177.6(6)	C$^{(14)}$	N$^{(1)}$	C$^{(10)}$	C$^{(11)}$	0.7(13)
S$^{(1)}$	C$^{(24)}$	C$^{(25)}$	C$^{(26)}$	176.8(11)	C$^{(19)}$	C$^{(18)}$	C$^{(17)}$	N$^{(2)}$	41.9(10)
S$^{(1)}$	C$^{(24)}$	C$^{(29)}$	C$^{(28)}$	-178.6(17)	C$^{(19)}$	C$^{(18)}$	C$^{(17)}$	C$^{(16)}$	-73.9(9)
S$^{(2)}$	C$^{(41)}$	C$^{(40)}$	C$^{(39)}$	-175.3(6)	C$^{(19)}$	C$^{(18)}$	C$^{(23)}$	C$^{(22)}$	-0.1(12)
S$^{(2)}$	C$^{(41)}$	C$^{(42)}$	C$^{(43)}$	178.0(6)	O$^{(102)}$	S$^{(100)}$	C$^{(100)}$	F$^{(102)}$	62.2(15)
S$^{(2)}$	C$^{(44)}$	C$^{(49)}$	C$^{(48)}$	176.0(8)	O$^{(102)}$	S$^{(100)}$	C$^{(100)}$	F$^{(100)}$	-177.3(13)
O$^{(4)}$	C$^{(15)}$	C$^{(14)}$	N$^{(1)}$	-172.3(7)	O$^{(102)}$	S$^{(100)}$	C$^{(100)}$	F$^{(101)}$	-54.9(14)
O$^{(4)}$	C$^{(15)}$	C$^{(14)}$	C$^{(13)}$	4.1(12)	C$^{(23)}$	C$^{(18)}$	C$^{(17)}$	N$^{(2)}$	-142.1(7)
O$^{(9)}$	S$^{(3)}$	C$^{(02F)}$	F$^{(5)}$	61.5(8)	C$^{(23)}$	C$^{(18)}$	C$^{(17)}$	C$^{(16)}$	102.0(9)
O$^{(9)}$	S$^{(3)}$	C$^{(02F)}$	F$^{(7)}$	-56.8(10)	C$^{(23)}$	C$^{(18)}$	C$^{(19)}$	C$^{(20)}$	1.2(12)
O$^{(9)}$	S$^{(3)}$	C$^{(02F)}$	F$^{(6)}$	-177.1(9)	C$^{(53)}$	C$^{(52)}$	C$^{(51)}$	O$^{(11)}$	104.4(8)
F$^{(2)}$	C$^{(21)}$	C$^{(20)}$	C$^{(19)}$	-179.2(8)	C$^{(34)}$	C$^{(33)}$	C$^{(32)}$	C$^{(31)}$	5.3(18)
F$^{(2)}$	C$^{(21)}$	C$^{(22)}$	C$^{(23)}$	-179.7(8)	C$^{(16)}$	O$^{(4)}$	C$^{(15)}$	N$^{(2)}$	-5.2(10)
N$^{(4)}$	C$^{(50)}$	C$^{(43)}$	N$^{(5)}$	8.0(12)	C$^{(16)}$	O$^{(4)}$	C$^{(15)}$	C$^{(14)}$	170.3(7)
N$^{(4)}$	C$^{(50)}$	C$^{(43)}$	C$^{(42)}$	-176.1(8)	C$^{(33)}$	C$^{(34)}$	C$^{(35)}$	C$^{(30)}$	0.2(15)

Table S37 Torsion Angles for Er(OTf)3/L6.

A	B	C	D	Angle/°	A	B	C	D	Angle/°
$N^{(4)}$	$C^{(52)}$	$C^{(53)}$	$C^{(54)}$	-146.7(8)	$C^{(33)}$	$C^{(32)}$	$C^{(31)}$	$C^{(30)}$	-3.7(16)
$N^{(4)}$	$C^{(52)}$	$C^{(53)}$	$C^{(58)}$	40.2(11)	$C^{(20)}$	$C^{(21)}$	$C^{(22)}$	$C^{(23)}$	-0.7(15)
$N^{(4)}$	$C^{(52)}$	$C^{(51)}$	$O^{(11)}$	-17.2(9)	$C^{(56)}$	$C^{(55)}$	$C^{(54)}$	$C^{(53)}$	0.7(14)
$N^{(2)}$	$C^{(17)}$	$C^{(16)}$	$O^{(4)}$	-5.8(8)	$C^{(56)}$	$C^{(57)}$	$C^{(58)}$	$C^{(53)}$	-2.6(16)
$N^{(2)}$	$C^{(15)}$	$C^{(14)}$	$N^{(1)}$	3.0(12)	$C^{(54)}$	$C^{(53)}$	$C^{(58)}$	$C^{(57)}$	1.9(15)
$N^{(2)}$	$C^{(15)}$	$C^{(14)}$	$C^{(13)}$	179.5(8)	$C^{(54)}$	$C^{(55)}$	$C^{(56)}$	$F^{(1)}$	179.2(9)
$F^{(4)}$	$C^{(33)}$	$C^{(32)}$	$C^{(31)}$	-178.7(10)	$C^{(54)}$	$C^{(55)}$	$C^{(56)}$	$C^{(57)}$	-1.4(16)
$O^{(7)}$	$S^{(3)}$	$O^{(9)}$	$Er^{(1)}$	-120.3(8)	$C^{(58)}$	$C^{(53)}$	$C^{(54)}$	$C^{(55)}$	-0.9(14)
$O^{(7)}$	$S^{(3)}$	$C^{(02F)}$	$F^{(5)}$	-56.6(9)	$C^{(58)}$	$C^{(57)}$	$C^{(56)}$	$F^{(1)}$	-178.3(9)
$O^{(7)}$	$S^{(3)}$	$C^{(02F)}$	$F^{(7)}$	-174.8(9)	$C^{(58)}$	$C^{(57)}$	$C^{(56)}$	$C^{(55)}$	2.3(17)
$O^{(7)}$	$S^{(3)}$	$C^{(02F)}$	$F^{(6)}$	64.8(10)	$C^{(24)}$	$S^{(1)}$	$C^{(12)}$	$C^{(13)}$	-86.8(7)
$O^{(11)}$	$C^{(50)}$	$C^{(43)}$	$N^{(5)}$	-173.6(7)	$C^{(24)}$	$S^{(1)}$	$C^{(12)}$	$C^{(11)}$	90.6(7)
$O^{(11)}$	$C^{(50)}$	$C^{(43)}$	$C^{(42)}$	2.4(12)	$C^{(24)}$	$C^{(25)}$	$C^{(26)}$	$C^{(27)}$	1(2)
$C^{(18)}$	$C^{(17)}$	$C^{(16)}$	$O^{(4)}$	116.9(7)	$C^{(24)}$	$C^{(29)}$	$C^{(28)}$	$C^{(27)}$	2(4)
$C^{(18)}$	$C^{(19)}$	$C^{(20)}$	$C^{(21)}$	-2.0(13)	$O^{(101)}$	$S^{(100)}$	$C^{(100)}$	$F^{(102)}$	-56.6(13)
$C^{(18)}$	$C^{(23)}$	$C^{(22)}$	$C^{(21)}$	-0.2(14)	$O^{(101)}$	$S^{(100)}$	$C^{(100)}$	$F^{(100)}$	63.9(15)
$N^{(6)}$	$C^{(36)}$	$C^{(37)}$	$O^{(10)}$	-7.0(8)	$O^{(101)}$	$S^{(100)}$	$C^{(100)}$	$F^{(101)}$	-173.7(12)
$N^{(1)}$	$C^{(10)}$	$C^{(9)}$	$O^{(3)}$	-173.2(8)	$C^{(7)}$	$N^{(3)}$	$C^{(9)}$	$O^{(3)}$	-9.6(12)
$N^{(1)}$	$C^{(10)}$	$C^{(9)}$	$N^{(3)}$	6.2(14)	$C^{(7)}$	$N^{(3)}$	$C^{(9)}$	$C^{(10)}$	171.0(9)
$N^{(1)}$	$C^{(10)}$	$C^{(11)}$	$C^{(12)}$	-0.4(12)	$C^{(7)}$	$C^{(1)}$	$C^{(6)}$	$C^{(5)}$	-178.6(11)
$C^{(50)}$	$N^{(4)}$	$C^{(52)}$	$C^{(53)}$	-109.2(8)	$C^{(51)}$	$O^{(11)}$	$C^{(50)}$	$N^{(4)}$	-8.1(11)
$C^{(50)}$	$N^{(4)}$	$C^{(52)}$	$C^{(51)}$	13.2(9)	$C^{(51)}$	$O^{(11)}$	$C^{(50)}$	$C^{(43)}$	173.4(7)
$C^{(50)}$	$O^{(11)}$	$C^{(51)}$	$C^{(52)}$	15.4(10)	$C^{(51)}$	$C^{(52)}$	$C^{(53)}$	$C^{(54)}$	97.0(10)
$O^{(6)}$	$S^{(2)}$	$C^{(41)}$	$C^{(40)}$	-166.2(7)	$C^{(51)}$	$C^{(52)}$	$C^{(53)}$	$C^{(58)}$	-76.1(11)
$O^{(6)}$	$S^{(2)}$	$C^{(41)}$	$C^{(42)}$	14.8(8)	$C^{(2)}$	$C^{(1)}$	$C^{(6)}$	$C^{(5)}$	1.3(17)
$O^{(6)}$	$S^{(2)}$	$C^{(44)}$	$C^{(45)}$	-15.8(7)	$C^{(2)}$	$C^{(3)}$	$C^{(4)}$	$F^{(3)}$	179.0(11)
$O^{(6)}$	$S^{(2)}$	$C^{(44)}$	$C^{(49)}$	168.1(6)	$C^{(2)}$	$C^{(3)}$	$C^{(4)}$	$C^{(5)}$	0(2)
$N^{(3)}$	$C^{(7)}$	$C^{(1)}$	$C^{(2)}$	-109.8(9)	$C^{(31)}$	$C^{(30)}$	$C^{(36)}$	$N^{(6)}$	58.9(11)
$N^{(3)}$	$C^{(7)}$	$C^{(1)}$	$C^{(6)}$	70.1(11)	$C^{(31)}$	$C^{(30)}$	$C^{(36)}$	$C^{(37)}$	-57.1(11)
$N^{(3)}$	$C^{(7)}$	$C^{(8)}$	$O^{(3)}$	-5.7(12)	$C^{(31)}$	$C^{(30)}$	$C^{(35)}$	$C^{(34)}$	1.1(14)
$C^{(17)}$	$N^{(2)}$	$C^{(15)}$	$O^{(4)}$	1.1(10)	$C^{(1)}$	$C^{(7)}$	$C^{(8)}$	$O^{(3)}$	115.6(10)
$C^{(17)}$	$N^{(2)}$	$C^{(15)}$	$C^{(14)}$	-174.0(8)	$C^{(1)}$	$C^{(2)}$	$C^{(3)}$	$C^{(4)}$	-1.2(17)
$C^{(17)}$	$C^{(18)}$	$C^{(19)}$	$C^{(20)}$	177.1(8)	$O^{(13)}$	$S^{(006)}$	$C^{(63)}$	$F^{(01Y)}$	-62.5(17)
$C^{(17)}$	$C^{(18)}$	$C^{(23)}$	$C^{(22)}$	-176.2(8)	$O^{(13)}$	$S^{(006)}$	$C^{(63)}$	$F^{(02Q)}$	58.2(14)
$O^{(1)}$	$S^{(1)}$	$C^{(12)}$	$C^{(13)}$	157.5(7)	$O^{(13)}$	$S^{(006)}$	$C^{(63)}$	$F^{(9)}$	-176.7(14)
$O^{(1)}$	$S^{(1)}$	$C^{(12)}$	$C^{(11)}$	-25.0(8)	$C^{(35)}$	$C^{(30)}$	$C^{(36)}$	$N^{(6)}$	-127.4(8)
$O^{(1)}$	$S^{(1)}$	$C^{(24)}$	$C^{(25)}$	33.0(11)	$C^{(35)}$	$C^{(30)}$	$C^{(36)}$	$C^{(37)}$	116.6(9)

Table S37 Torsion Angles for Er(OTf)3/L6.

A	B	C	D	Angle/˚	A	B	C	D	Angle/˚
$O^{(1)}$	$S^{(1)}$	$C^{(24)}$	$C^{(29)}$	-150.1(12)	$C^{(35)}$	$C^{(30)}$	$C^{(31)}$	$C^{(32)}$	0.7(15)
$C^{(15)}$	$O^{(4)}$	$C^{(16)}$	$C^{(17)}$	6.5(8)	$C^{(35)}$	$C^{(34)}$	$C^{(33)}$	$F^{(4)}$	-179.4(10)
$C^{(15)}$	$N^{(2)}$	$C^{(17)}$	$C^{(18)}$	-118.5(8)	$C^{(35)}$	$C^{(34)}$	$C^{(33)}$	$C^{(32)}$	-3.5(17)
$C^{(15)}$	$N^{(2)}$	$C^{(17)}$	$C^{(16)}$	3.2(8)	$C^{(22)}$	$C^{(21)}$	$C^{(20)}$	$C^{(19)}$	1.7(15)
$C^{(39)}$	$N^{(5)}$	$C^{(43)}$	$C^{(50)}$	178.8(7)	$C^{(3)}$	$C^{(2)}$	$C^{(1)}$	$C^{(7)}$	-179.5(9)
$C^{(39)}$	$N^{(5)}$	$C^{(43)}$	$C^{(42)}$	2.9(13)	$C^{(3)}$	$C^{(2)}$	$C^{(1)}$	$C^{(6)}$	0.6(15)
$C^{(41)}$	$S^{(2)}$	$C^{(44)}$	$C^{(45)}$	97.3(7)	$C^{(3)}$	$C^{(4)}$	$C^{(5)}$	$C^{(6)}$	2(2)
$C^{(41)}$	$S^{(2)}$	$C^{(44)}$	$C^{(49)}$	-78.8(7)	$C^{(4)}$	$C^{(5)}$	$C^{(6)}$	$C^{(1)}$	-3(2)
$C^{(41)}$	$C^{(42)}$	$C^{(43)}$	$C^{(50)}$	-177.9(8)	$O^{(100)}$	$S^{(100)}$	$C^{(100)}$	$F^{(102)}$	-175.5(12)
$C^{(41)}$	$C^{(42)}$	$C^{(43)}$	$N^{(5)}$	-2.4(13)	$O^{(100)}$	$S^{(100)}$	$C^{(100)}$	$F^{(100)}$	-55.0(16)
$C^{(40)}$	$C^{(39)}$	$N^{(5)}$	$Er^{(1)}$	172.9(6)	$O^{(100)}$	$S^{(100)}$	$C^{(100)}$	$F^{(101)}$	67.4(13)
$C^{(40)}$	$C^{(39)}$	$N^{(5)}$	$C^{(43)}$	0.2(12)	$C^{(8)}$	$O^{(3)}$	$C^{(9)}$	$N^{(3)}$	5.6(13)
$C^{(40)}$	$C^{(39)}$	$C^{(38)}$	$O^{(10)}$	9.2(13)	$C^{(8)}$	$O^{(3)}$	$C^{(9)}$	$C^{(10)}$	-175.0(9)
$C^{(40)}$	$C^{(39)}$	$C^{(38)}$	$N^{(6)}$	-168.6(8)	$C^{(8)}$	$C^{(7)}$	$C^{(1)}$	$C^{(2)}$	133.2(9)
$C^{(40)}$	$C^{(41)}$	$C^{(42)}$	$C^{(43)}$	-1.0(12)	$C^{(8)}$	$C^{(7)}$	$C^{(1)}$	$C^{(6)}$	-47.0(12)
$O^{(2)}$	$S^{(1)}$	$C^{(12)}$	$C^{(13)}$	28.3(9)	$C^{(02F)}$	$S^{(3)}$	$O^{(9)}$	$Er^{(1)}$	129.2(8)
$O^{(2)}$	$S^{(1)}$	$C^{(12)}$	$C^{(11)}$	-154.3(7)	$C^{(25)}$	$C^{(24)}$	$C^{(29)}$	$C^{(28)}$	-2(3)
$O^{(2)}$	$S^{(1)}$	$C^{(24)}$	$C^{(25)}$	166.9(9)	$C^{(25)}$	$C^{(26)}$	$C^{(27)}$	$C^{(28)}$	0(3)
$O^{(2)}$	$S^{(1)}$	$C^{(24)}$	$C^{(29)}$	-16.2(14)	$O^{(16)}$	$S^{(006)}$	$C^{(63)}$	$F^{(01Y)}$	53.2(17)
$N^{(5)}$	$C^{(39)}$	$C^{(40)}$	$C^{(41)}$	-3.4(12)	$O^{(16)}$	$S^{(006)}$	$C^{(63)}$	$F^{(02Q)}$	173.8(14)
$N^{(5)}$	$C^{(39)}$	$C^{(38)}$	$O^{(10)}$	-173.0(7)	$O^{(16)}$	$S^{(006)}$	$C^{(63)}$	$F^{(9)}$	-61.0(16)
$N^{(5)}$	$C^{(39)}$	$C^{(38)}$	$N^{(6)}$	9.1(12)	$C^{(29)}$	$C^{(24)}$	$C^{(25)}$	$C^{(26)}$	0(2)
$O^{(5)}$	$S^{(2)}$	$C^{(41)}$	$C^{(40)}$	-37.4(9)	$C^{(29)}$	$C^{(28)}$	$C^{(27)}$	$C^{(26)}$	-1(4)
$O^{(5)}$	$S^{(2)}$	$C^{(41)}$	$C^{(42)}$	143.5(8)	$C^{(47)}$	$C^{(46)}$	$C^{(45)}$	$C^{(44)}$	0.0
$O^{(5)}$	$S^{(2)}$	$C^{(44)}$	$C^{(45)}$	-149.0(7)	$C^{(46)}$	$C^{(47)}$	$C^{(48)}$	$C^{(49)}$	0.0
$O^{(5)}$	$S^{(2)}$	$C^{(44)}$	$C^{(49)}$	34.9(8)	$C^{(46)}$	$C^{(45)}$	$C^{(44)}$	$S^{(2)}$	-176.1(8)
$C^{(12)}$	$S^{(1)}$	$C^{(24)}$	$C^{(25)}$	-80.2(10)	$C^{(46)}$	$C^{(45)}$	$C^{(44)}$	$C^{(49)}$	0.0
$C^{(12)}$	$S^{(1)}$	$C^{(24)}$	$C^{(29)}$	96.7(13)	$C^{(45)}$	$C^{(44)}$	$C^{(49)}$	$C^{(48)}$	0.0
$C^{(12)}$	$C^{(13)}$	$C^{(14)}$	$N^{(1)}$	-0.3(13)	$C^{(44)}$	$S^{(2)}$	$C^{(41)}$	$C^{(40)}$	78.7(8)
$C^{(12)}$	$C^{(13)}$	$C^{(14)}$	$C^{(15)}$	-176.4(8)	$C^{(44)}$	$S^{(2)}$	$C^{(41)}$	$C^{(42)}$	-100.4(8)
$C^{(10)}$	$N^{(1)}$	$C^{(14)}$	$C^{(15)}$	176.1(7)	$C^{(44)}$	$C^{(49)}$	$C^{(48)}$	$C^{(47)}$	0.0
$C^{(10)}$	$N^{(1)}$	$C^{(14)}$	$C^{(13)}$	-0.3(13)	$C^{(48)}$	$C^{(47)}$	$C^{(46)}$	$C^{(45)}$	0.0
$C^{(30)}$	$C^{(36)}$	$C^{(37)}$	$O^{(10)}$	114.6(7)	$N^{(7A)1}$	$N^{(7A)}$	$C^{(59A)}$	$C^{(60A)}$	129(17)
$C^{(13)}$	$C^{(12)}$	$C^{(11)}$	$C^{(10)}$	-0.3(12)	$C^{(59A)1}$	$N^{(7A)}$	$C^{(59A)}$	$N^{(7A)1}$	-42(14)
$C^{(36)}$	$N^{(6)}$	$C^{(38)}$	$O^{(10)}$	-1.8(10)	$C^{(59A)1}$	$N^{(7A)}$	$C^{(59A)}$	$C^{(60A)}$	86(26)
$C^{(36)}$	$N^{(6)}$	$C^{(38)}$	$C^{(39)}$	175.9(7)	$F^{(9A)}$	$C^{(63A)}$	$S^{(0)}$	$O^{(16A)}$	-55(3)
$C^{(36)}$	$C^{(30)}$	$C^{(31)}$	$C^{(32)}$	174.3(9)	$F^{(9A)}$	$C^{(63A)}$	$S^{(0)}$	$O^{(13A)}$	-173(3)
$C^{(36)}$	$C^{(30)}$	$C^{(35)}$	$C^{(34)}$	-172.6(9)	$F^{(9A)}$	$C^{(63A)}$	$S^{(0)}$	$O^{(12A)}$	66(3)

Table S37 Torsion Angles for $Er(OTf)_3/L_6$.

A	B	C	D	Angle/˚	A	B	C	D	Angle/˚
$F^{(3)}$	$C^{(4)}$	$C^{(5)}$	$C^{(6)}$	-177.0(12)	$F^{(02R)}$	$C^{(63A)}$	$S^{(0)}$	$O^{(16A)}$	-175(4)
$C^{(42)}$	$C^{(41)}$	$C^{(40)}$	$C^{(39)}$	3.7(13)	$F^{(02R)}$	$C^{(63A)}$	$S^{(0)}$	$O^{(13A)}$	67(4)
$C^{(9)}$	$O^{(3)}$	$C^{(8)}$	$C^{(7)}$	0.9(12)	$F^{(02R)}$	$C^{(63A)}$	$S^{(0)}$	$O^{(12A)}$	-54(5)
$C^{(9)}$	$N^{(3)}$	$C^{(7)}$	$C^{(1)}$	-114.9(9)	$F^{(01Z)}$	$C^{(63A)}$	$S^{(0)}$	$O^{(16A)}$	54(3)
$C^{(9)}$	$N^{(3)}$	$C^{(7)}$	$C^{(8)}$	8.8(12)	$F^{(01Z)}$	$C^{(63A)}$	$S^{(0)}$	$O^{(13A)}$	-64(3)
$C^{(9)}$	$C^{(10)}$	$C^{(11)}$	$C^{(12)}$	178.2(8)	$F^{(01Z)}$	$C^{(63A)}$	$S^{(0)}$	$O^{(12A)}$	175(3)

^{1}1-X,1-Y,+Z

Table S38 Hydrogen Atom Coordinates (Å×10⁴) and Isotropic Displacement Parameters (Å²×10³) for $Er(OTf)_3/L_6$.

Atom	*x*	*y*	*z*	U(eq)
$H^{(1WA)}$	6422.65	6287.67	1817.32	68
$H^{(1WB)}$	6213.5	5584.09	2000.86	68
$H^{(17)}$	6960.58	7674.35	2934.78	46
$H^{(40)}$	6416.28	10169.11	5308.8	48
$H^{(13)}$	7103.5	4061.64	4040.87	45
$H^{(36)}$	6419.56	8033.54	1302.37	49
$H^{(42)}$	6291.18	8458.78	8119.24	48
$H^{(11)}$	6272.57	2845.14	4146.35	48
$H^{(37A)}$	6603.06	9480.74	1474.23	58
$H^{(37B)}$	6262.58	9877.31	1444.85	58
$H^{(52)}$	6057.87	4920.54	6519.36	54
$H^{(19)}$	7014.96	7183.73	6046.22	55
$H^{(23)}$	7111.71	9155.85	3538.25	58
$H^{(34)}$	5627.45	7676.84	-1185.93	69
$H^{(16A)}$	7368.87	6767.7	2676.66	53
$H^{(16B)}$	7463.27	6928.93	4026.09	53
$H^{(55)}$	6755.31	2621.29	7148.41	73
$H^{(57)}$	7244.92	4989.24	7169.11	69
$H^{(20)}$	7096	8306.15	7479.92	68
$H^{(54)}$	6307	3499.82	6905.39	66
$H^{(58)}$	6802.17	5862.24	6982.84	62
$H^{(7)}$	5410.4	5580.08	4397.77	67
$H^{(51A)}$	5902.86	5609.7	8177.35	66
$H^{(51B)}$	6233.39	5385.94	8701.83	66
$H^{(2)}$	5200.8	6675.32	3071.87	79
$H^{(32)}$	5290.48	9276.06	1436.06	75

Table S38 Hydrogen Atom Coordinates (Å×10^4) and Isotropic Displacement Parameters (Å^2×10^3) for Er(OTf)$_3$/L$_6$.

Atom	*x*	*y*	*z*	U(eq)
$H^{(31)}$	5759.17	9232.56	2377.13	68
$H^{(35)}$	6090.99	7585.33	-152.82	57
$H^{(22)}$	7208.13	10263.07	5026.42	72
$H^{(3)}$	5049.5	7109.47	1191.24	94
$H^{(8A)}$	5336.14	4082.98	4801.53	78
$H^{(8B)}$	5352.14	3843.52	3421.98	78
$H^{(25)}$	6487.86	1559.89	2334.4	82
$H^{(26)}$	6513.91	1351.92	316.94	99
$H^{(5)}$	5475.99	4901.1	-166.75	121
$H^{(6)}$	5611.68	4385.27	1741.57	93
$H^{(29)}$	7359.27	2390.26	2384.83	144
$H^{(28)}$	7392.07	2108.69	400.89	176
$H^{(27)}$	6968.6	1618.13	-673.2	141
$H^{(20A)}$	6284.59	3025.91	-919.48	354
$H^{(20B)}$	5936.04	3301.34	-781.45	354
$H^{(20C)}$	6077.9	2502.34	23.49	354
$H^{(47)}$	5195.67	11607	7322.98	201
$H^{(46)}$	5333.01	10685.57	8927.08	185
$H^{(45)}$	5834.22	10197.7	9146.25	134
$H^{(49)}$	6060.77	11552.71	6157.24	131
$H^{(48)}$	5559.56	12040.58	5938.04	171
$H^{(60A)}$	5338.64	2591.18	9121.11	209
$H^{(60B)}$	5521.64	3266.68	9980.51	209
$H^{(60C)}$	5696.54	2475.78	9266.01	209
$H^{(60D)}$	5794.83	4314.99	7255.99	159
$H^{(60E)}$	5610.37	3362.87	7154.39	159
$H^{(60F)}$	5617.27	3917.02	8370.74	159

Table S39 Atomic Occupancy for Er(OTf)$_3$/L$_6$.

Atom	*Occupancy*	Atom	*Occupancy*	Atom	*Occupancy*
$S^{(006)}$	0.760(6)	$O^{(12)}$	0.760(6)	$F^{(01Y)}$	0.760(6)
$O^{(13)}$	0.760(6)	$O^{(16)}$	0.760(6)	$F^{(02Q)}$	0.760(6)
$F^{(9)}$	0.760(6)	$C^{(63)}$	0.760(6)	$N^{(7)}$	0.512(18)
$C^{(59)}$	0.512(18)	$C^{(60)}$	0.512(18)	$H^{(60A)}$	0.512(18)
$H^{(60B)}$	0.512(18)	$H^{(60C)}$	0.512(18)	$N^{(7A)}$	0.488(18)
$C^{(59A)}$	0.488(18)	$C^{(60A)}$	0.488(18)	$H^{(60D)}$	0.488(18)
$H^{(60E)}$	0.488(18)	$H^{(60F)}$	0.488(18)	$C^{(63A)}$	0.240(6)
$F^{(9A)}$	0.240(6)	$F^{(02R)}$	0.240(6)	$F^{(01Z)}$	0.240(6)
$S^{(0)}$	0.240(6)	$O^{(16A)}$	0.240(6)	$O^{(13A)}$	0.240(6)

Table S39 Atomic Occupancy for $Er(OTf)_3/L_6$.

Atom	*Occupancy*	Atom	*Occupancy*	Atom	*Occupancy*
$O^{(12A)}$	0.240(6)				

12. ^{1}H, ^{13}C, and ^{19}F NMR spectra

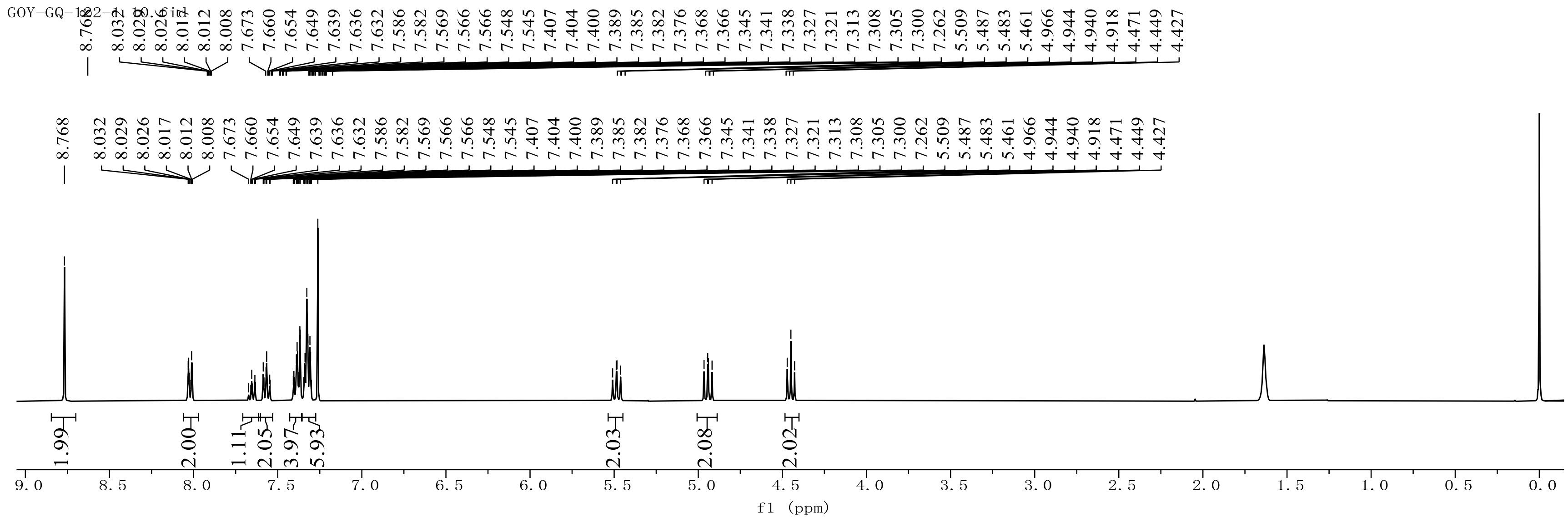

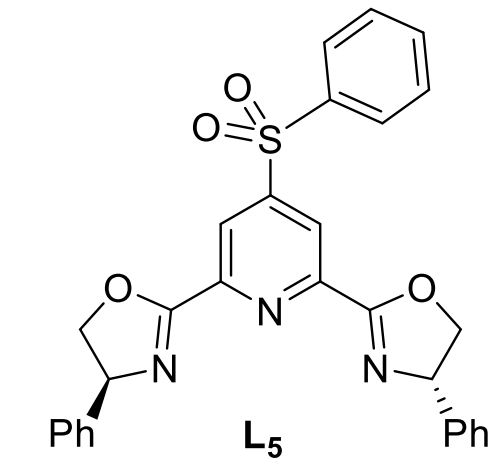

^{1}H NMR of compound $\mathbf{L_5}$ (400 MHz, $CDCl_3$)

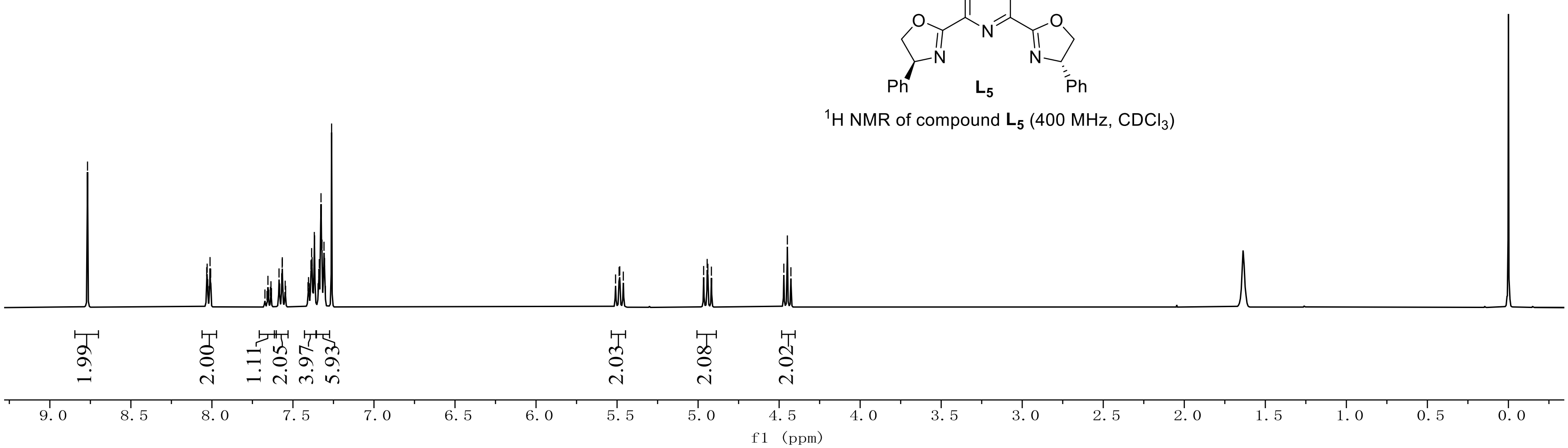

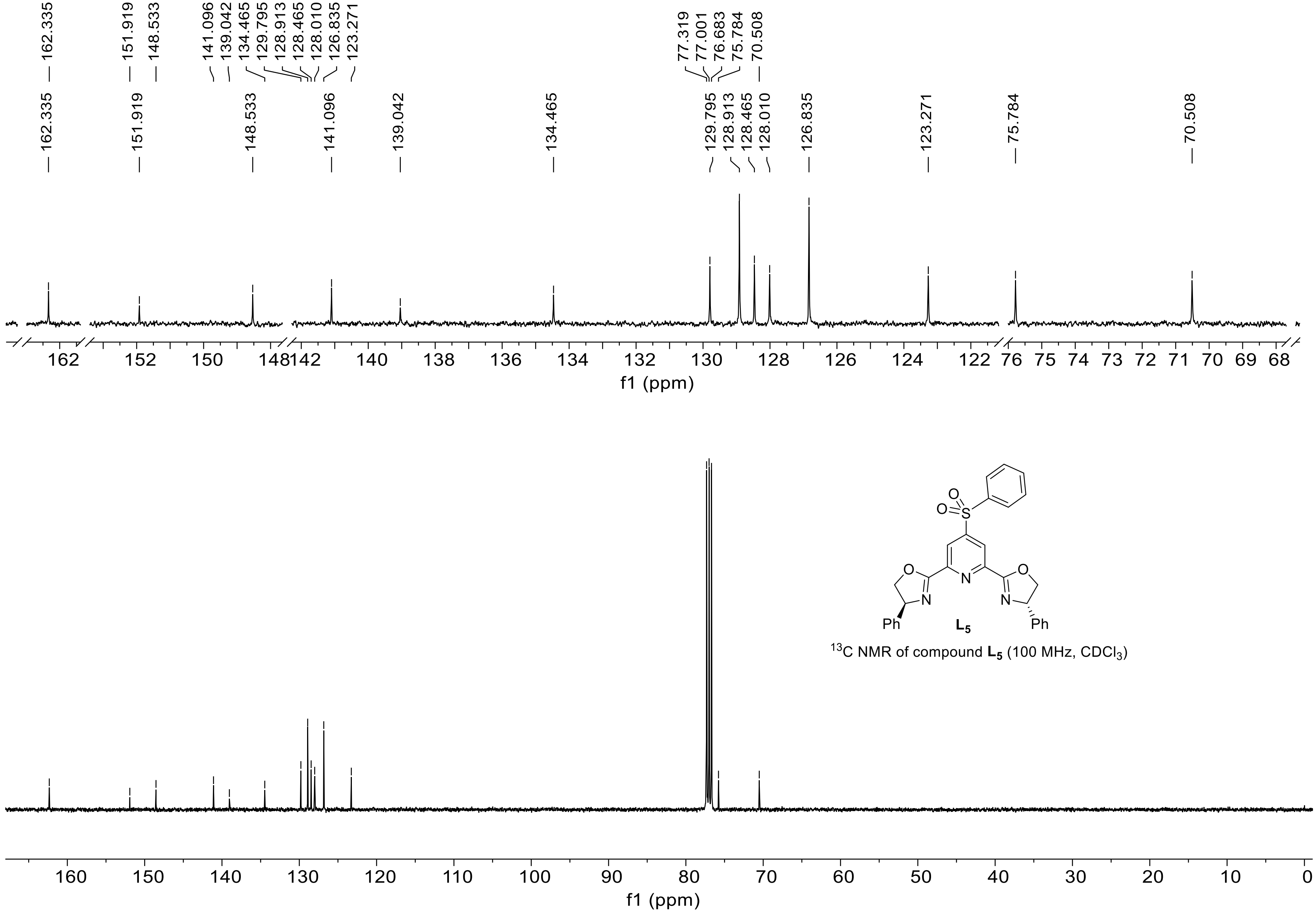

$\mathbf{L_5}$

^{13}C NMR of compound $\mathbf{L_5}$ (100 MHz, $CDCl_3$)

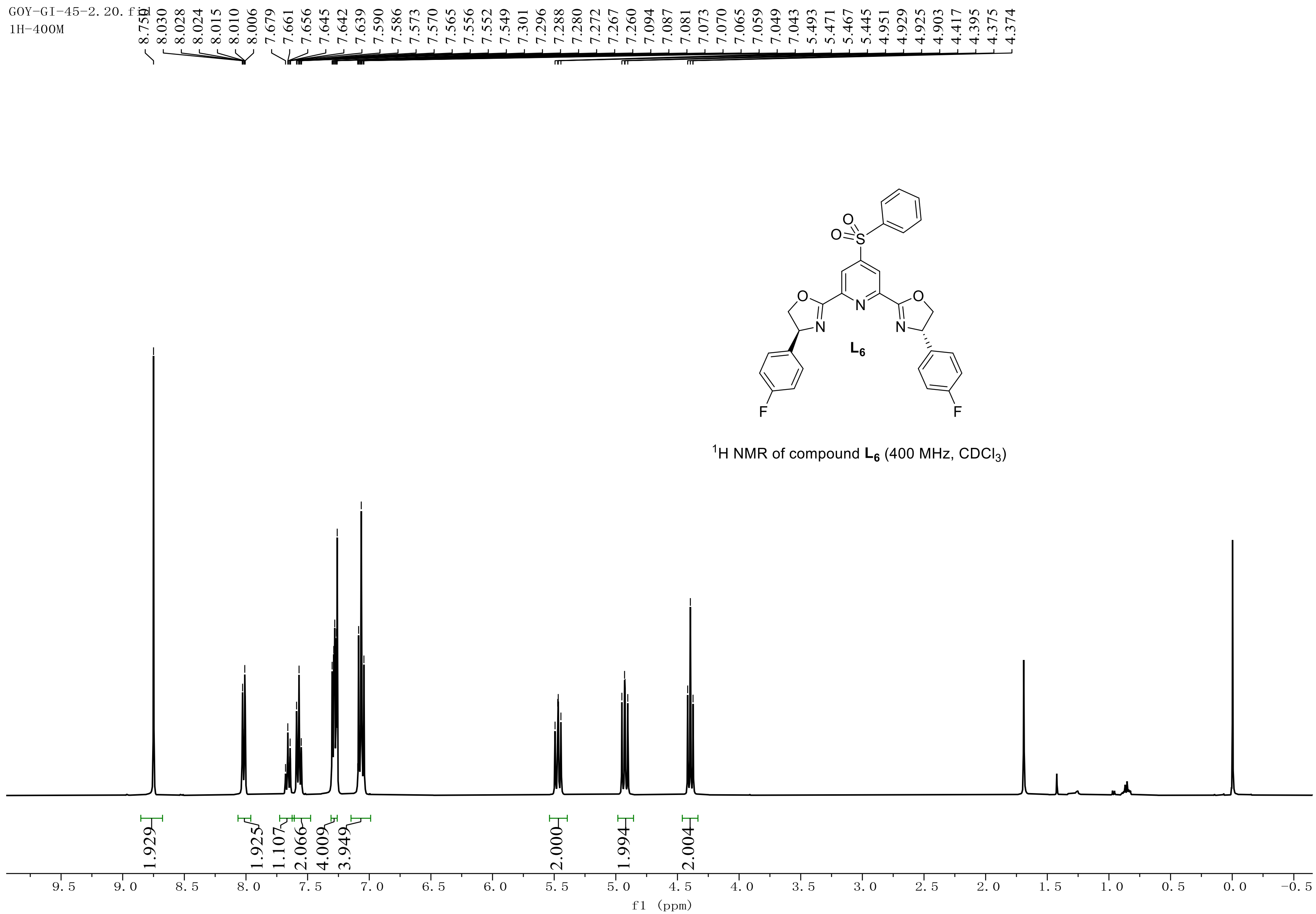

^{1}H NMR of compound **L_6** (400 MHz, $CDCl_3$)

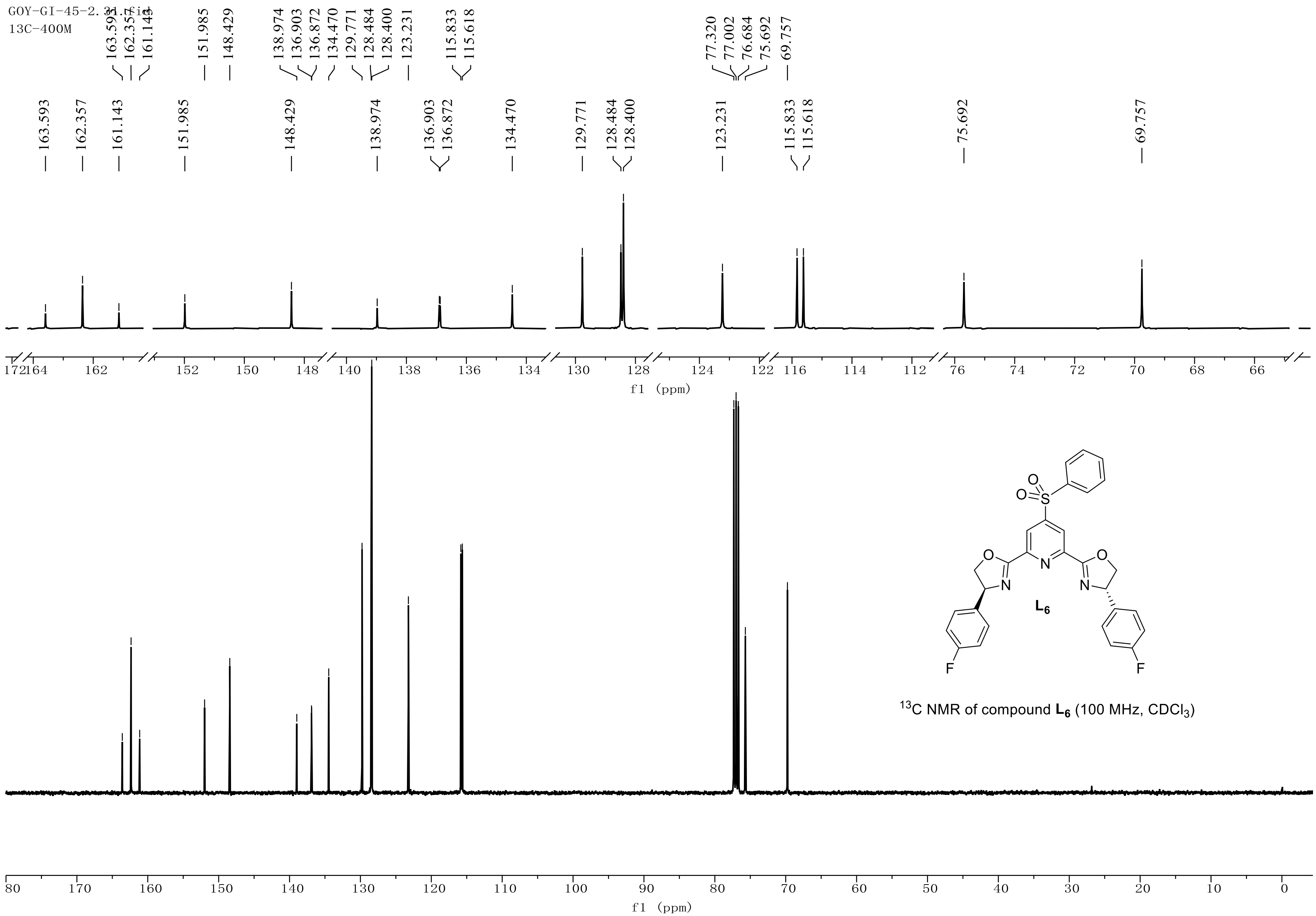

^{13}C NMR of compound **L_6** (100 MHz, $CDCl_3$)

GOY-GI-45-2.21.fid
19F-400M

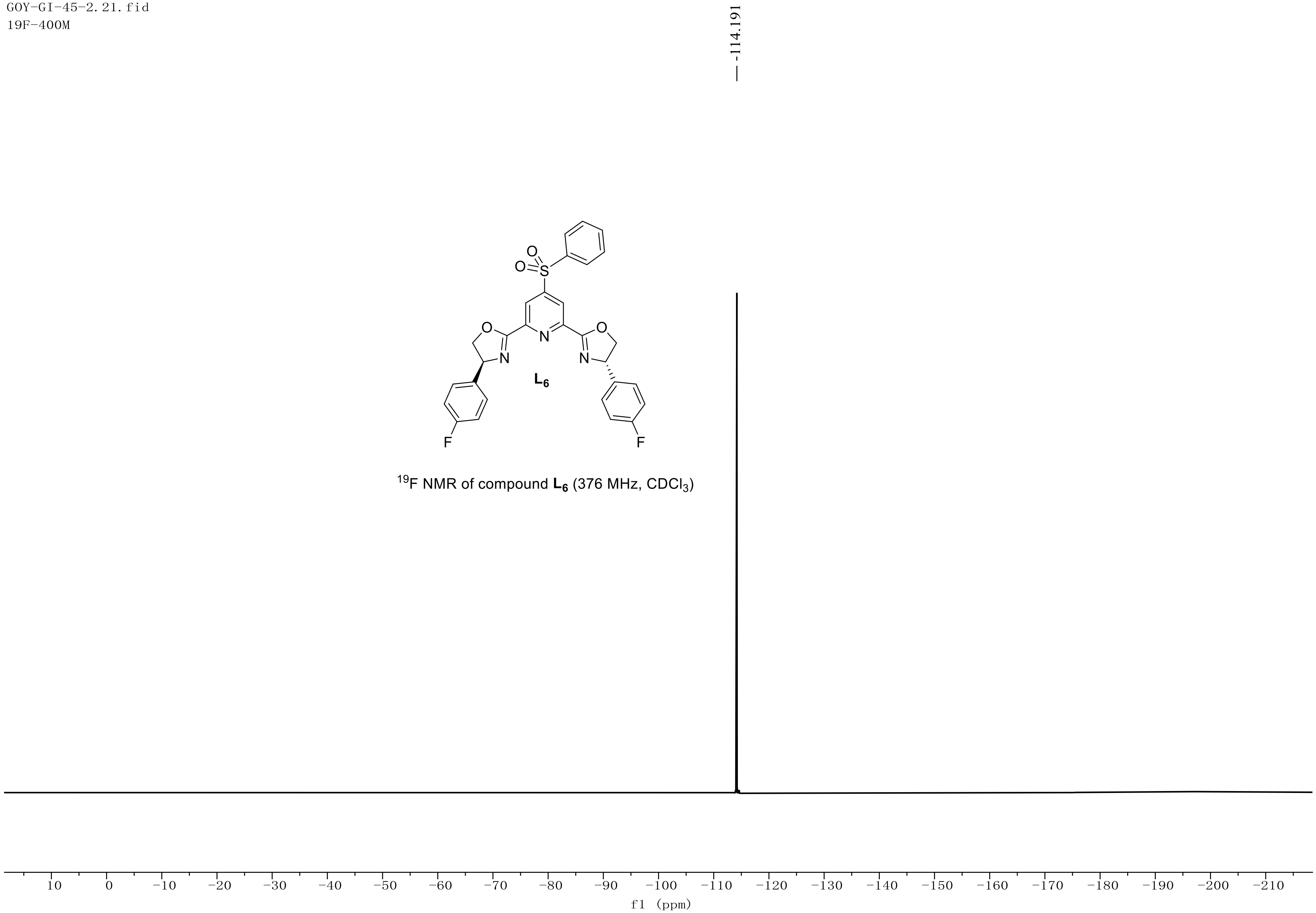

^{19}F NMR of compound **L_6** (376 MHz, $CDCl_3$)

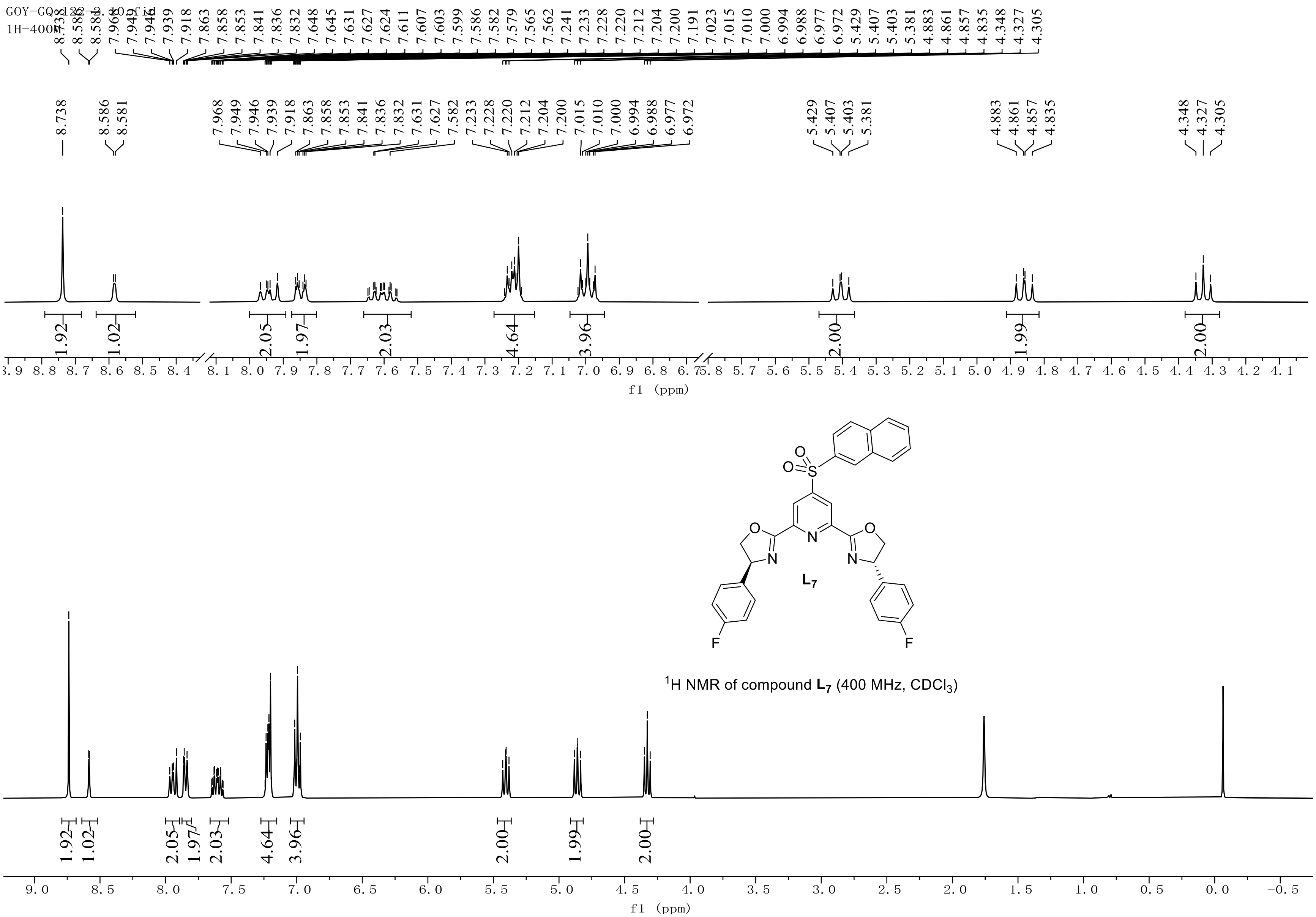

^{1}H NMR of compound **L_7** (400 MHz, $CDCl_3$)

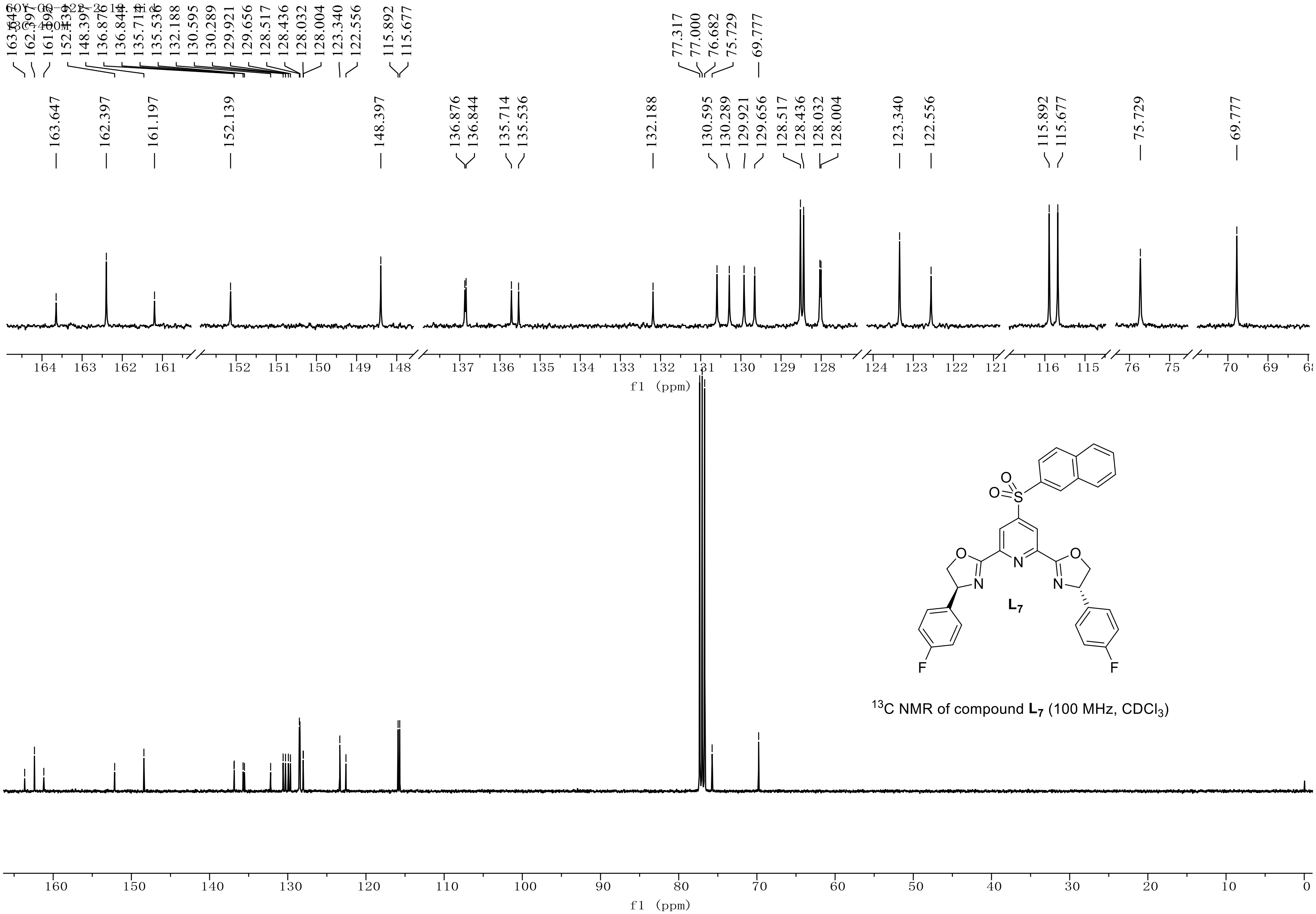

^{13}C NMR of compound $\mathbf{L_7}$ (100 MHz, $CDCl_3$)

GOY-GQ-122-2.13.fid
19F-400M

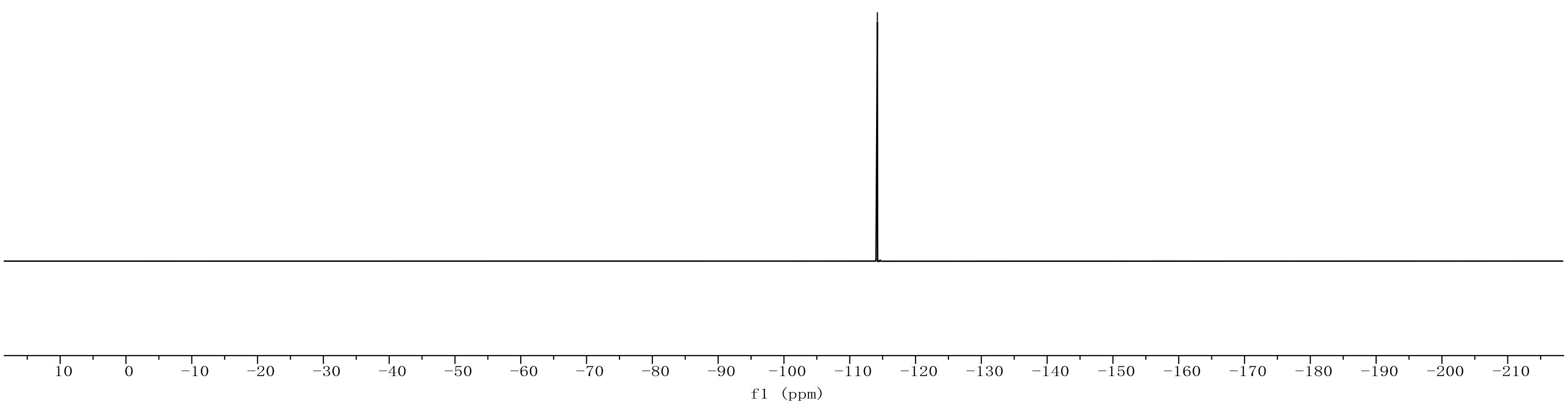

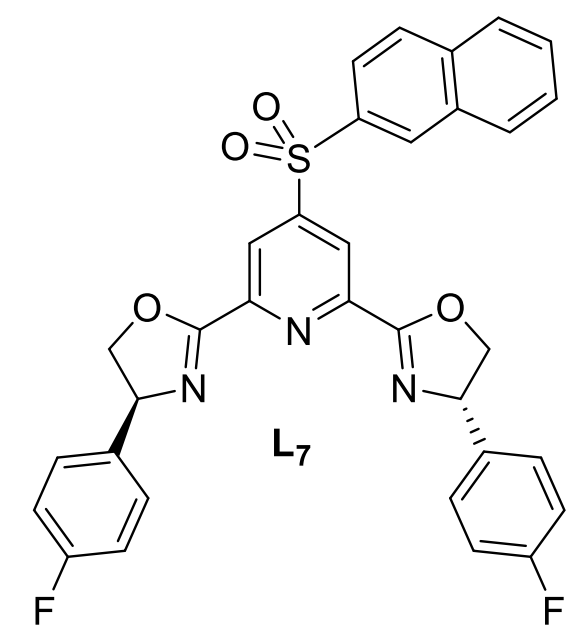

L_7

^{19}F NMR of compound **L_7** (376 MHz, $CDCl_3$)

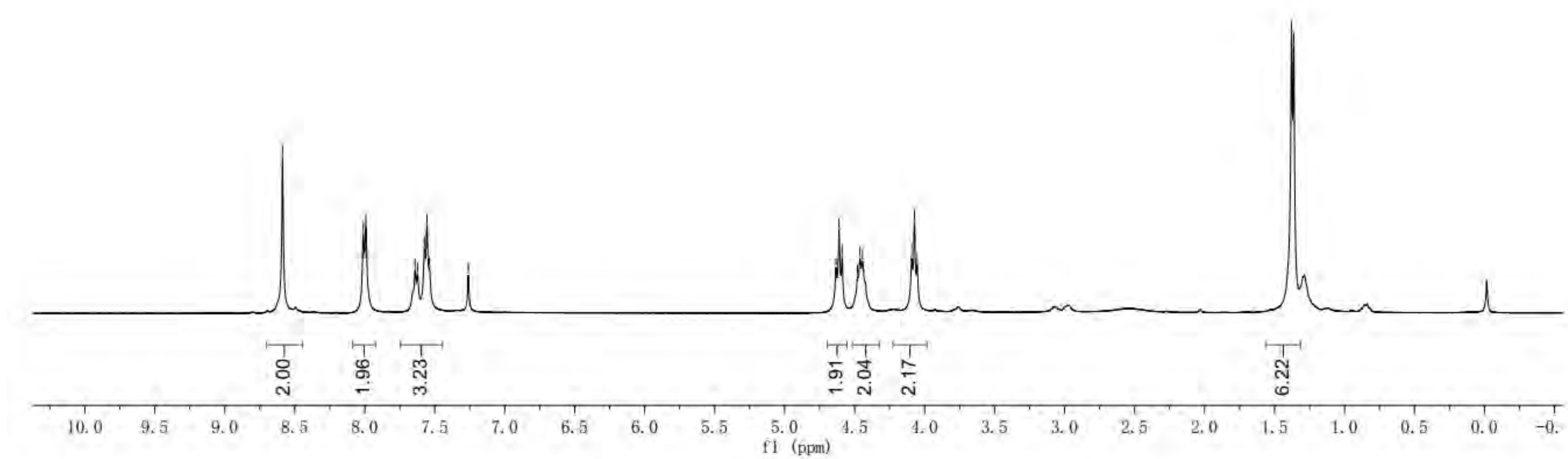

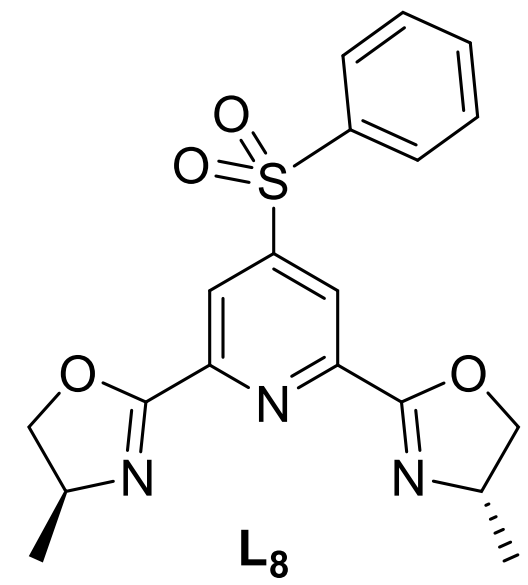

L_8

^{1}H NMR of compound **L_8** (400 MHz, $CDCl_3$)

ZHZ-ZAD-ME-L-C

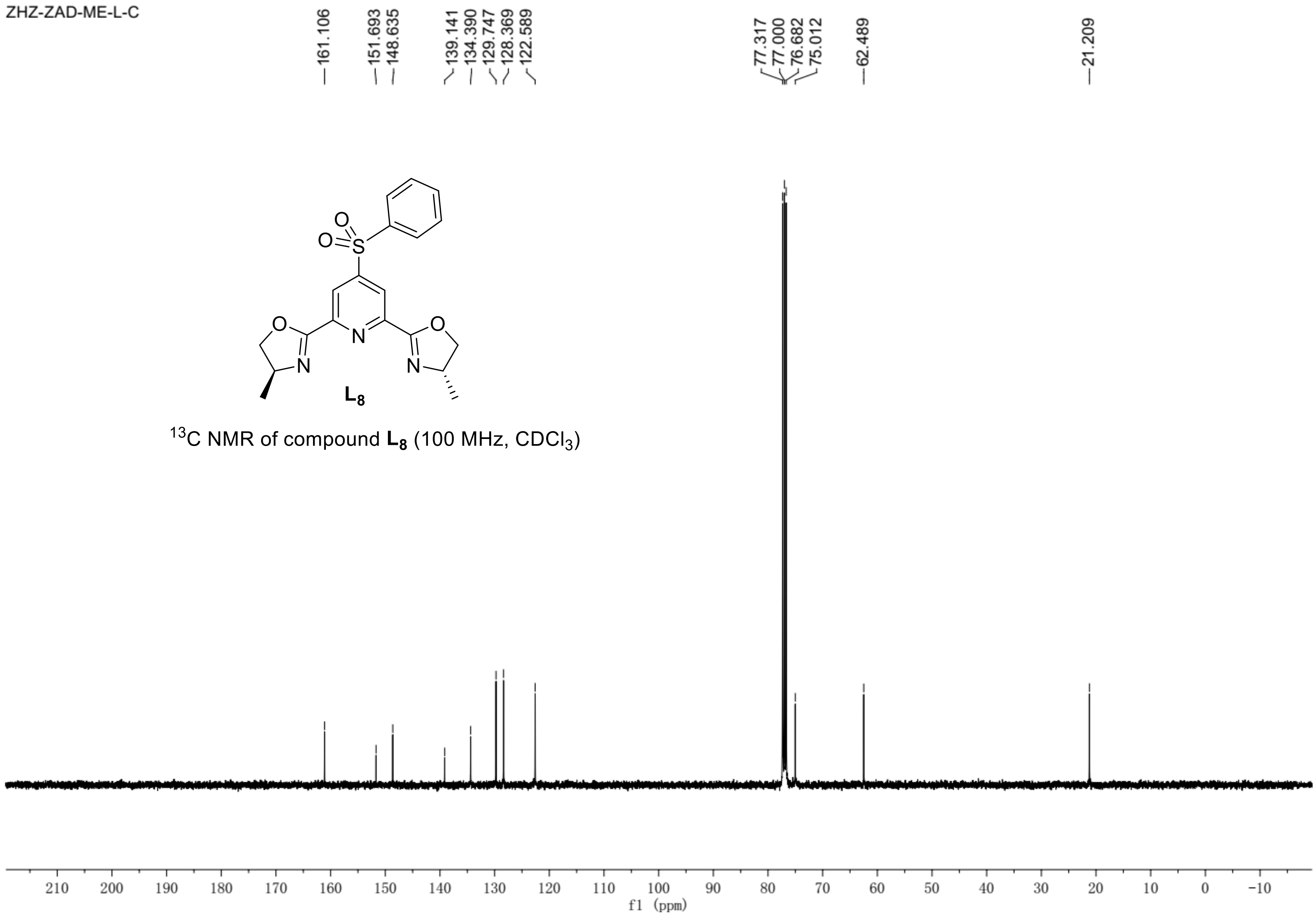

L$_8$

^{13}C NMR of compound **L$_8$** (100 MHz, $CDCl_3$)

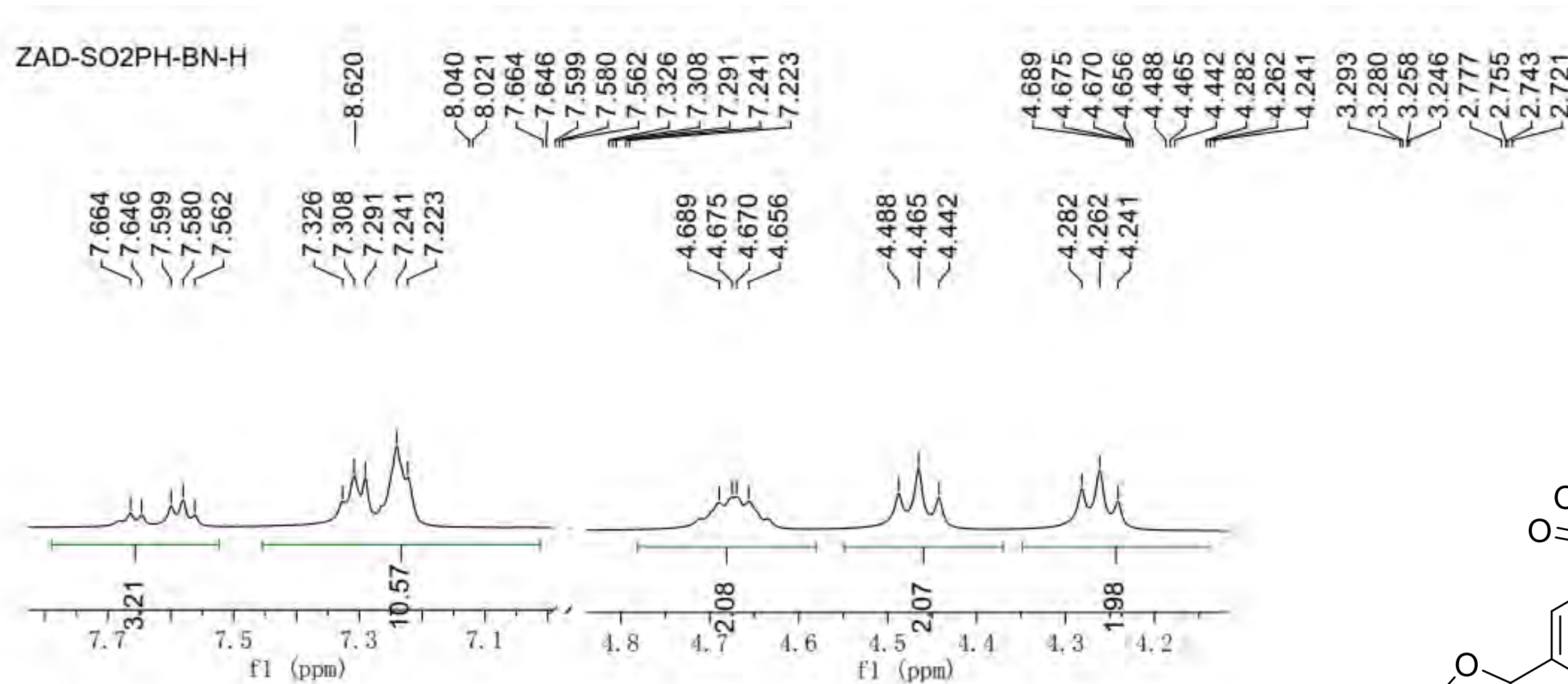

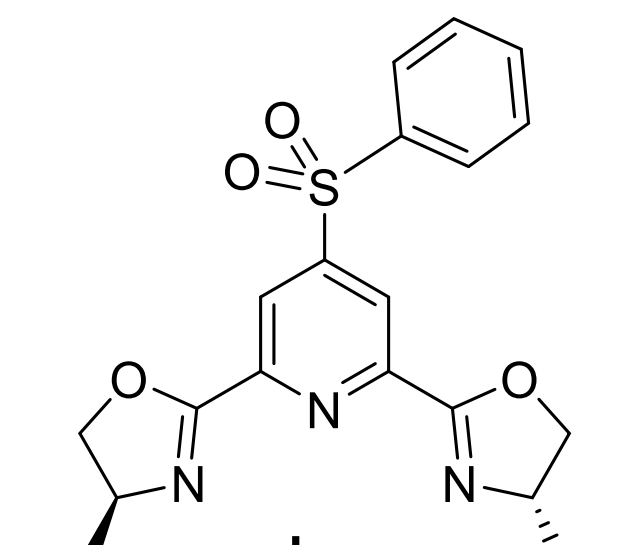

L₉

^{1}H NMR of compound **L$_9$** (400 MHz, $CDCl_3$)

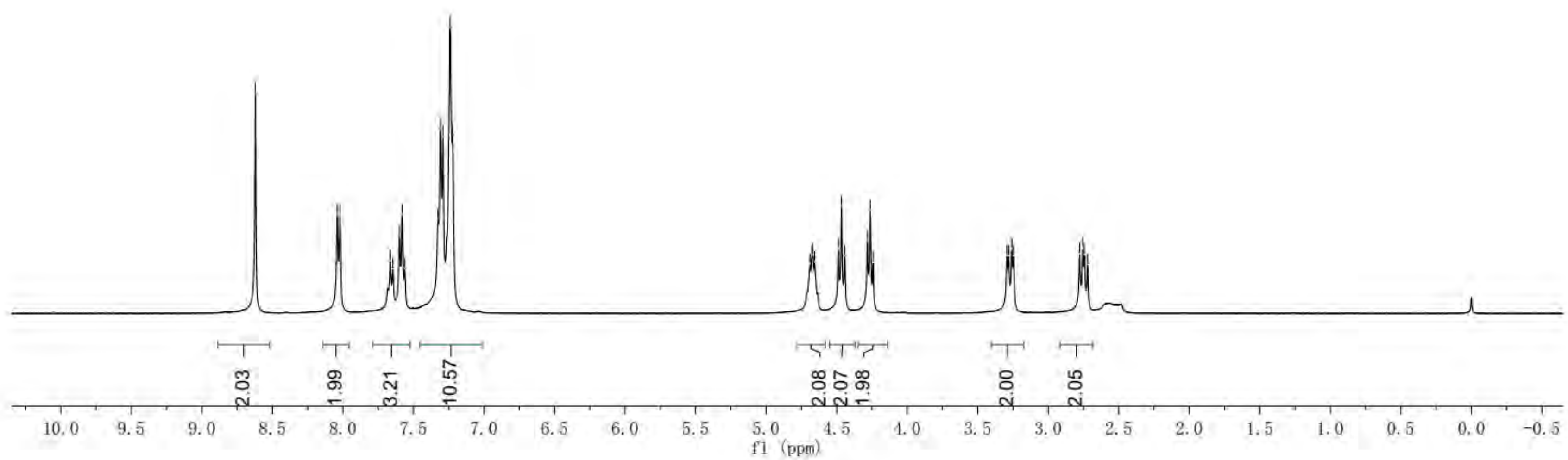

ZAD-SO2PH-BN-C

161.526, 151.710, 148.465, 139.027, 137.269, 134.388, 129.715, 129.105, 128.567, 128.319, 126.623, 122.643, 77.318, 77.000, 76.682, 72.816, 68.145, 41.373

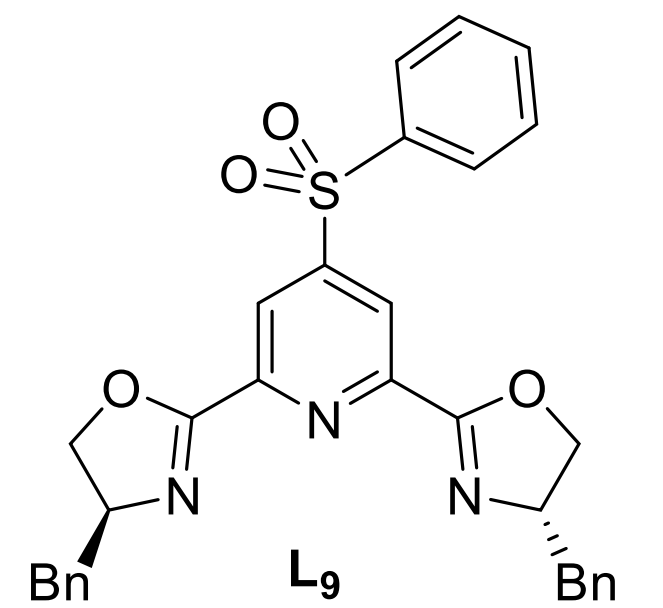

L_9

^{13}C NMR of compound $\mathbf{L_9}$ (100 MHz, $CDCl_3$)

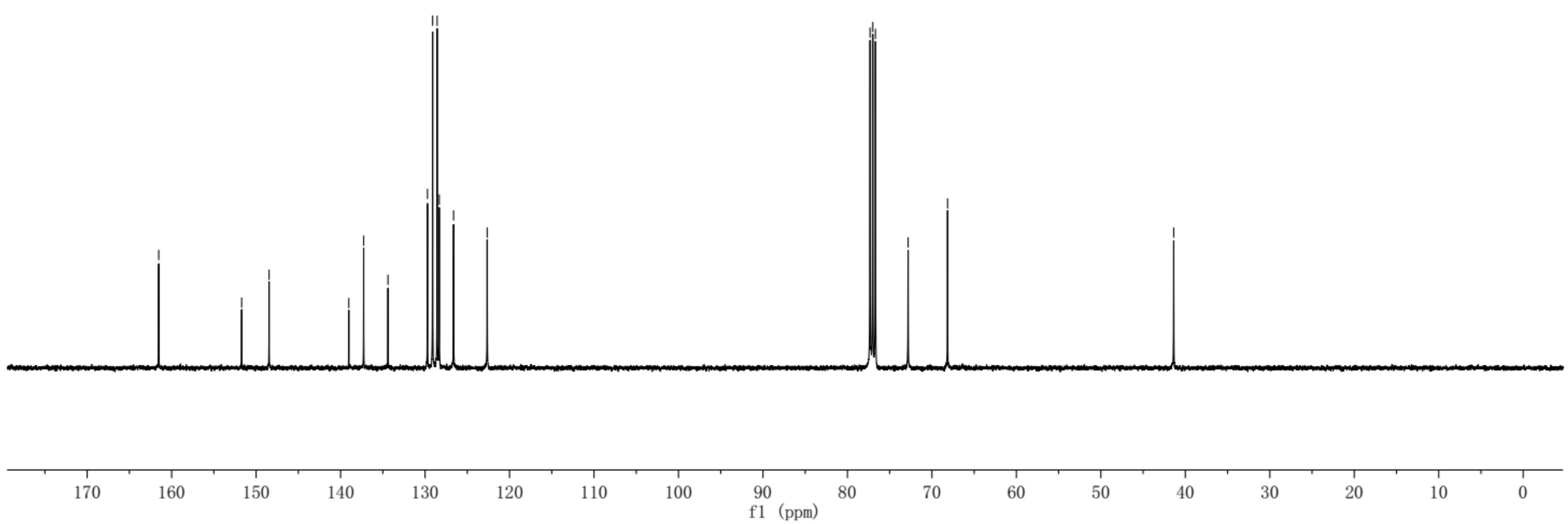

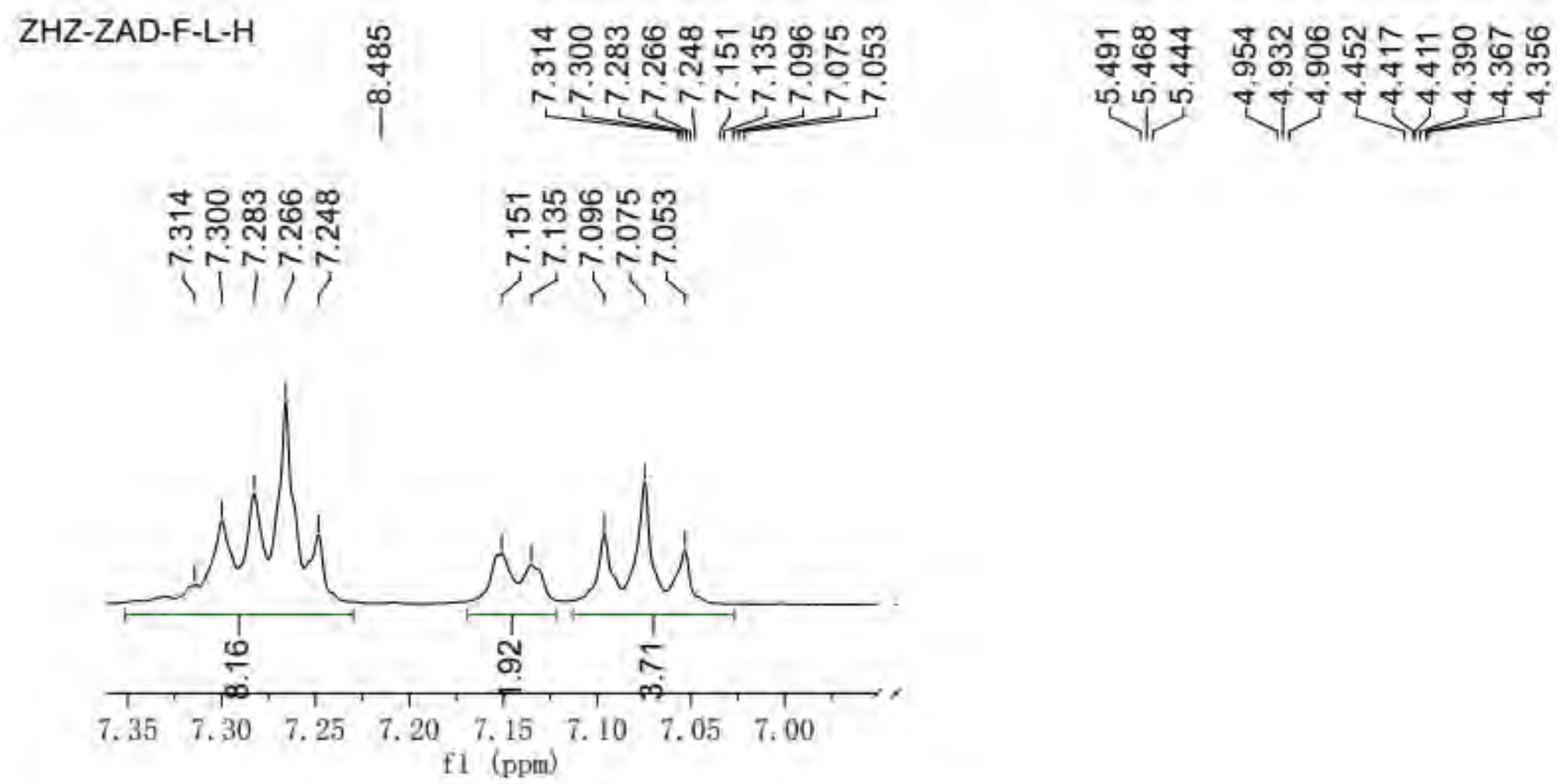

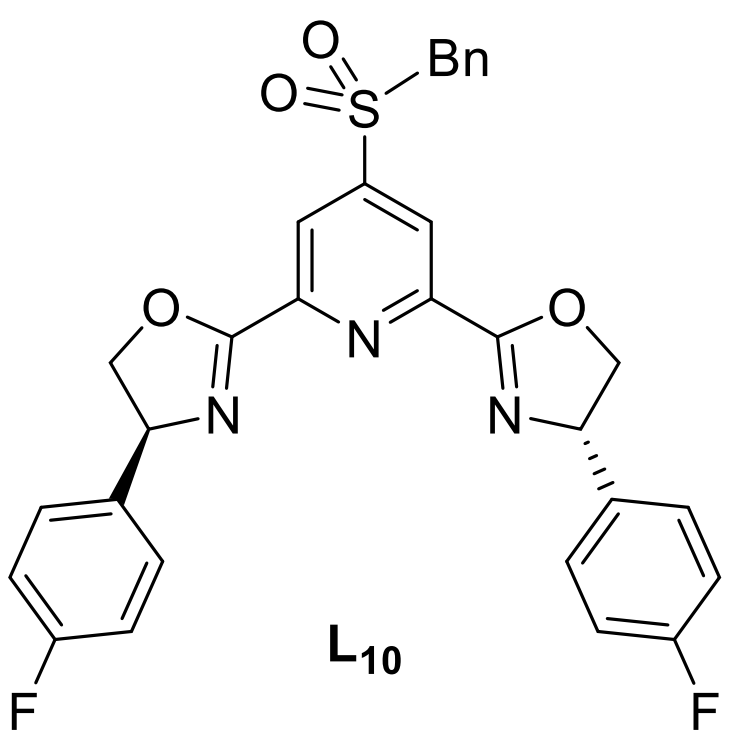

L_{10}

^{1}H NMR of compound **L_{10}** (400 MHz, $CDCl_3$)

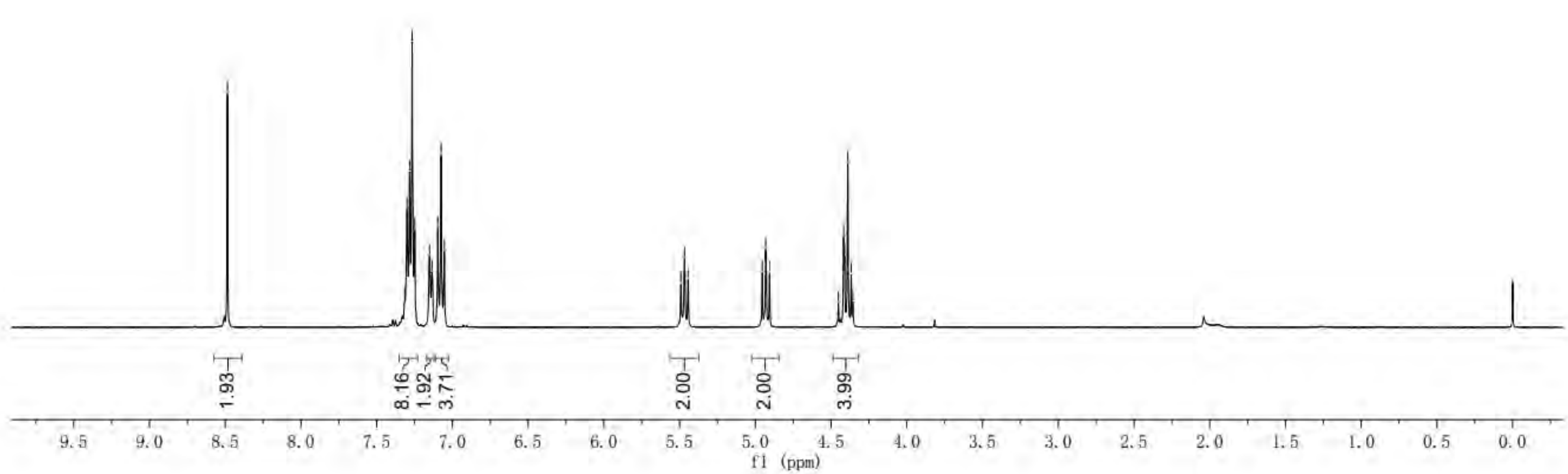

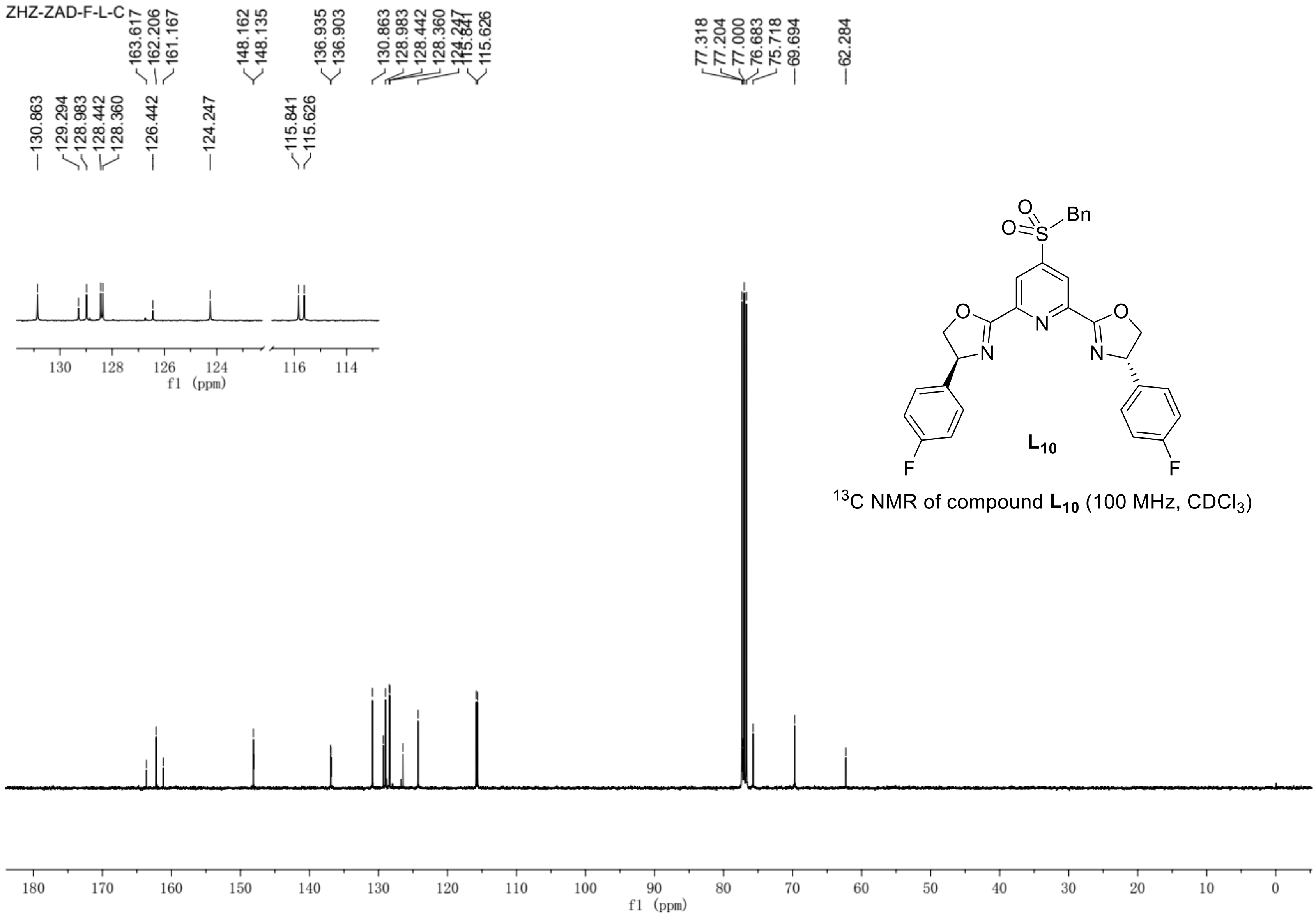

L_{10}

^{13}C NMR of compound $\mathbf{L_{10}}$ (100 MHz, $CDCl_3$)

ZHZ-ZAD-F-L-F

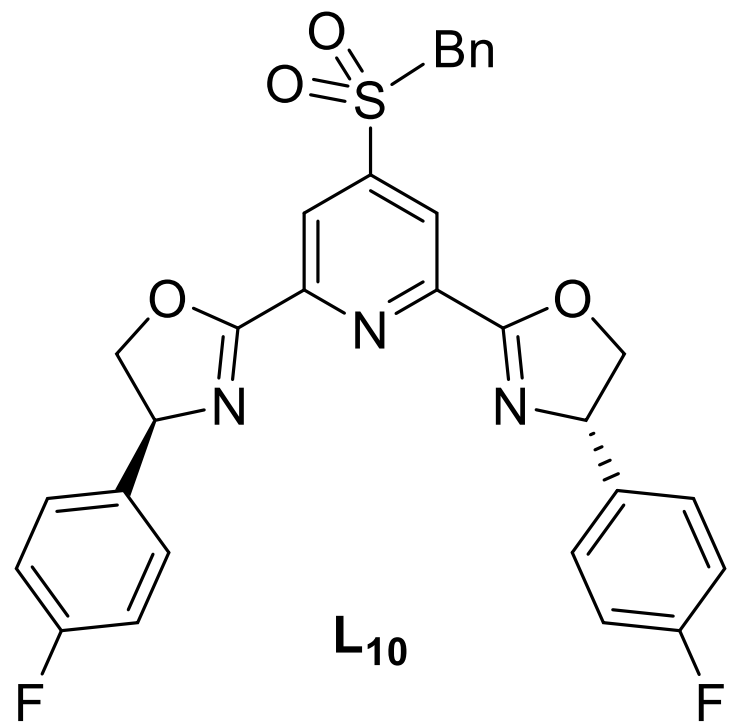

^{19}F NMR of compound $\mathbf{L_{10}}$ (376 MHz, $CDCl_3$)

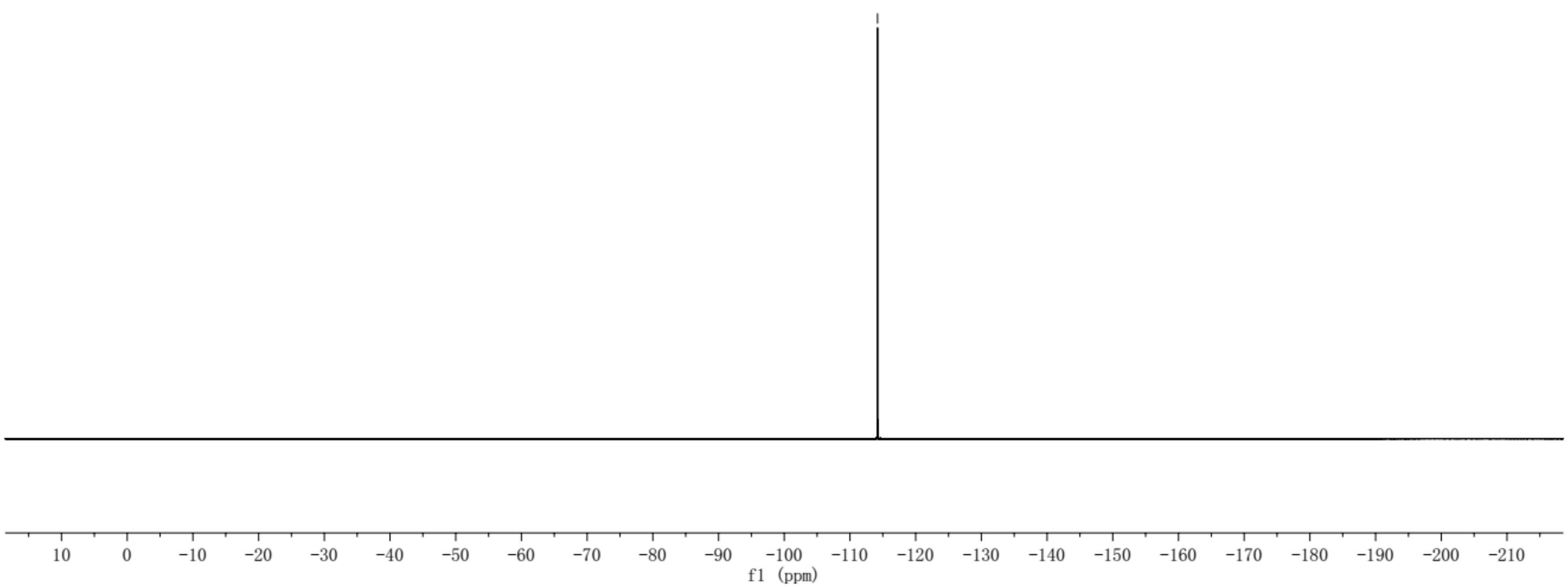

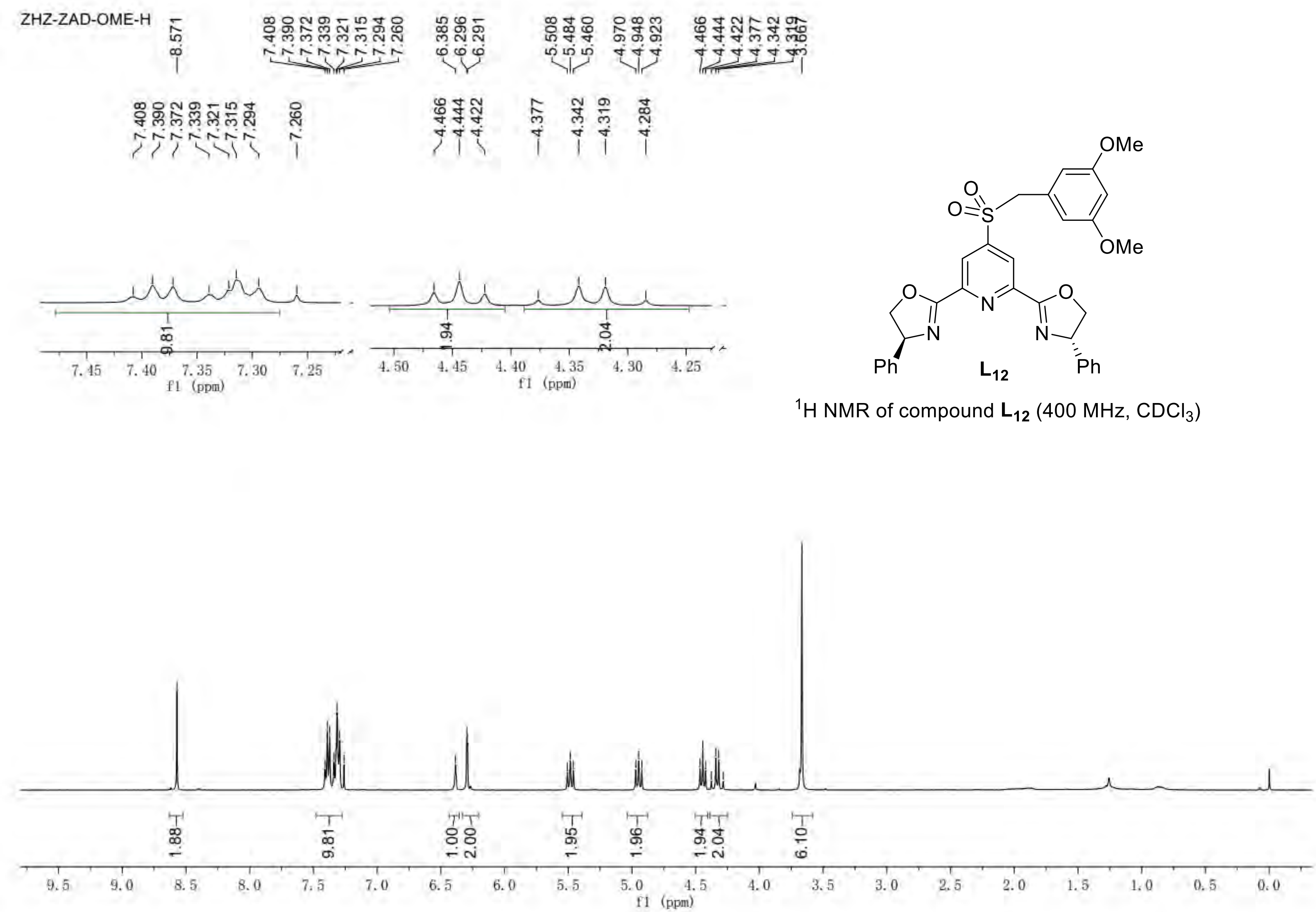

^{1}H NMR of compound **L_{12}** (400 MHz, $CDCl_3$)

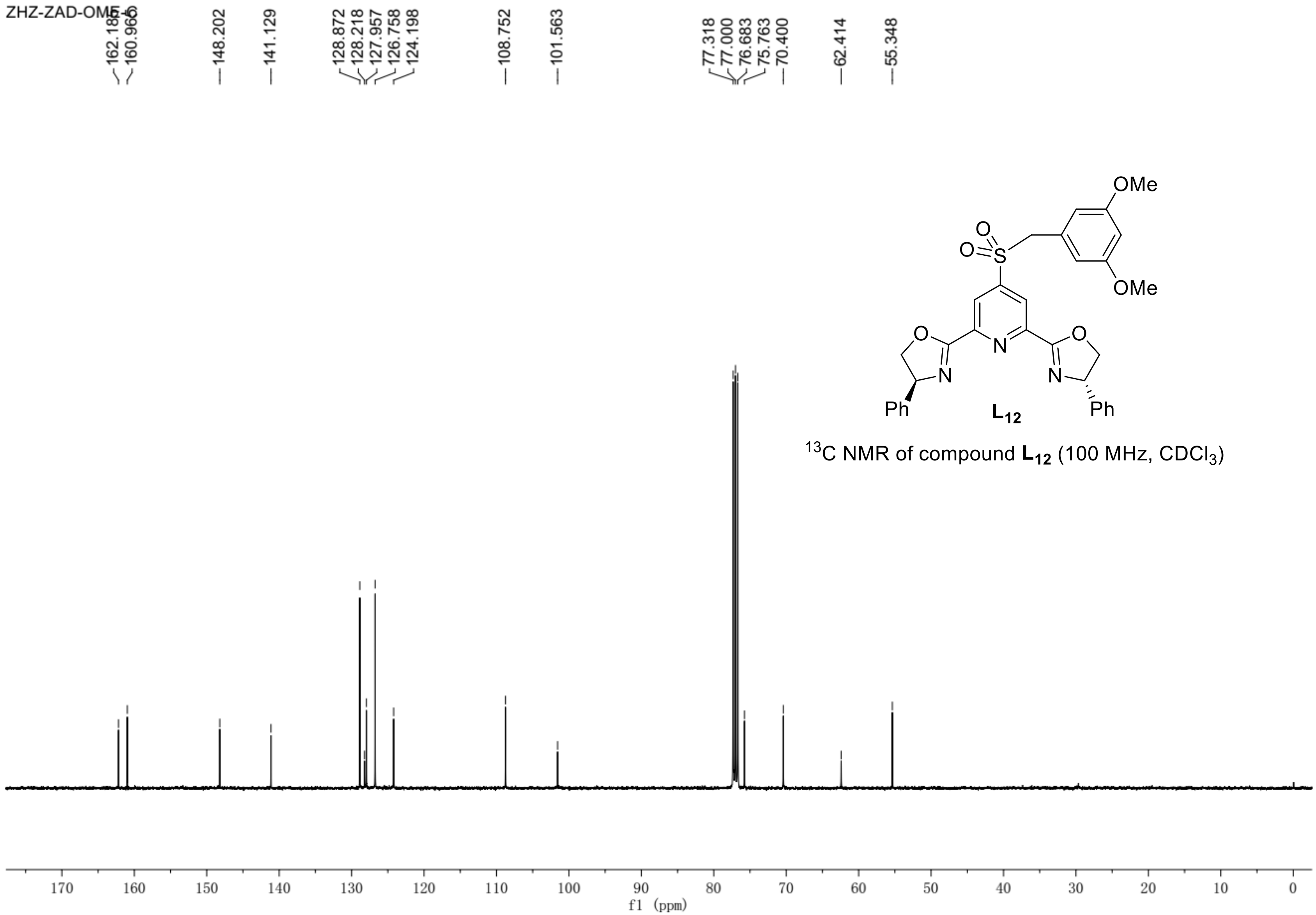

^{13}C NMR of compound **L_{12}** (100 MHz, $CDCl_3$)

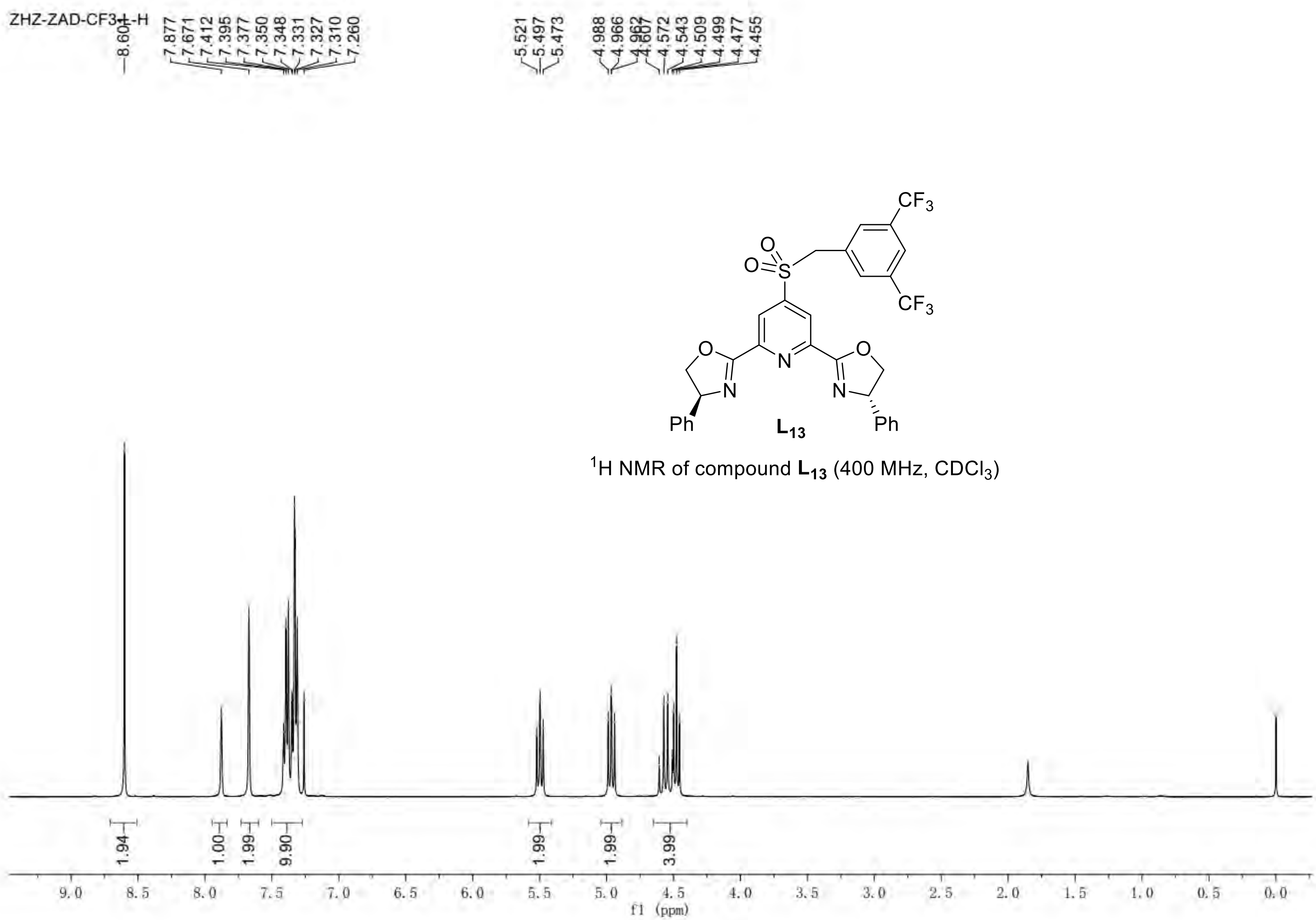

^{1}H NMR of compound **L_{13}** (400 MHz, $CDCl_3$)

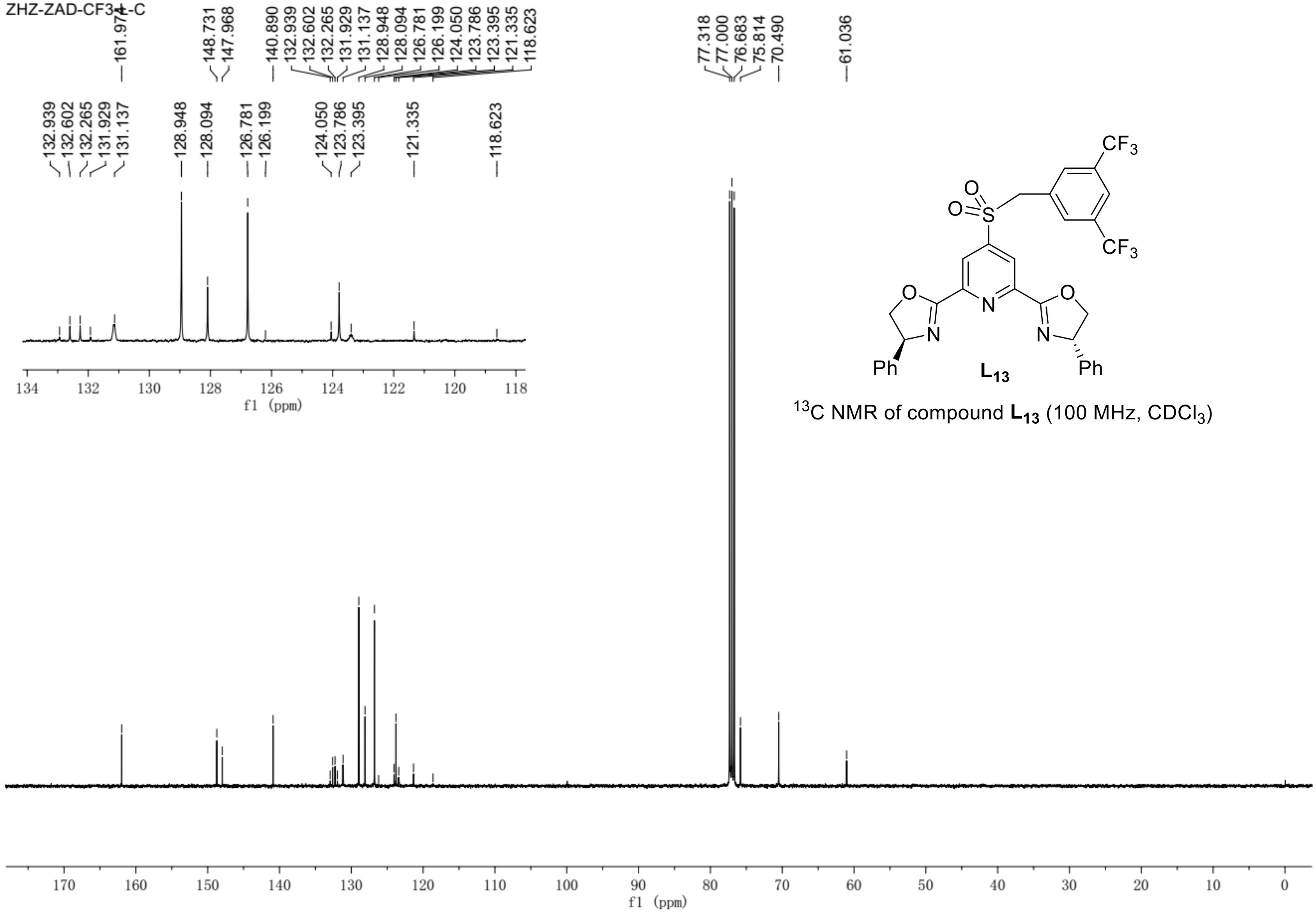

^{13}C NMR of compound $\mathbf{L_{13}}$ (100 MHz, $CDCl_3$)

ZHZ-ZAD-CF3-L-F

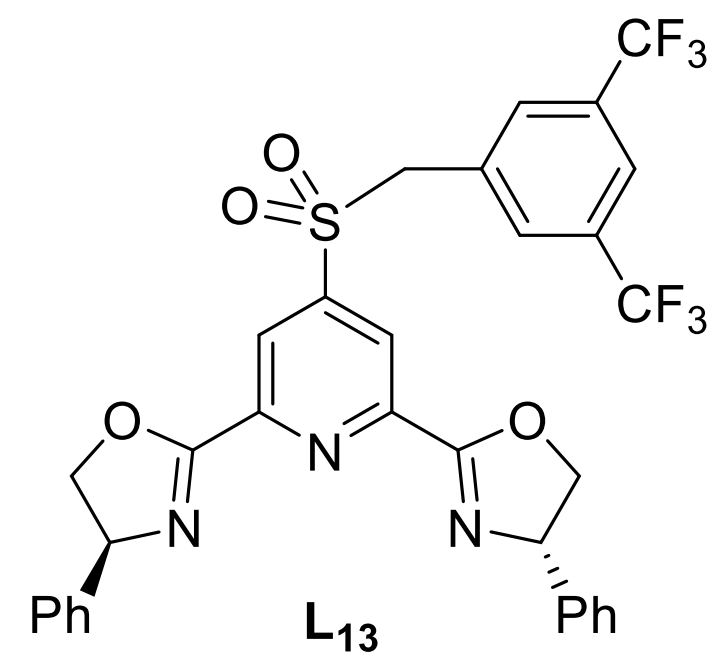

L_{13}

^{19}F NMR of compound **L_{13}** (376 MHz, $CDCl_3$)

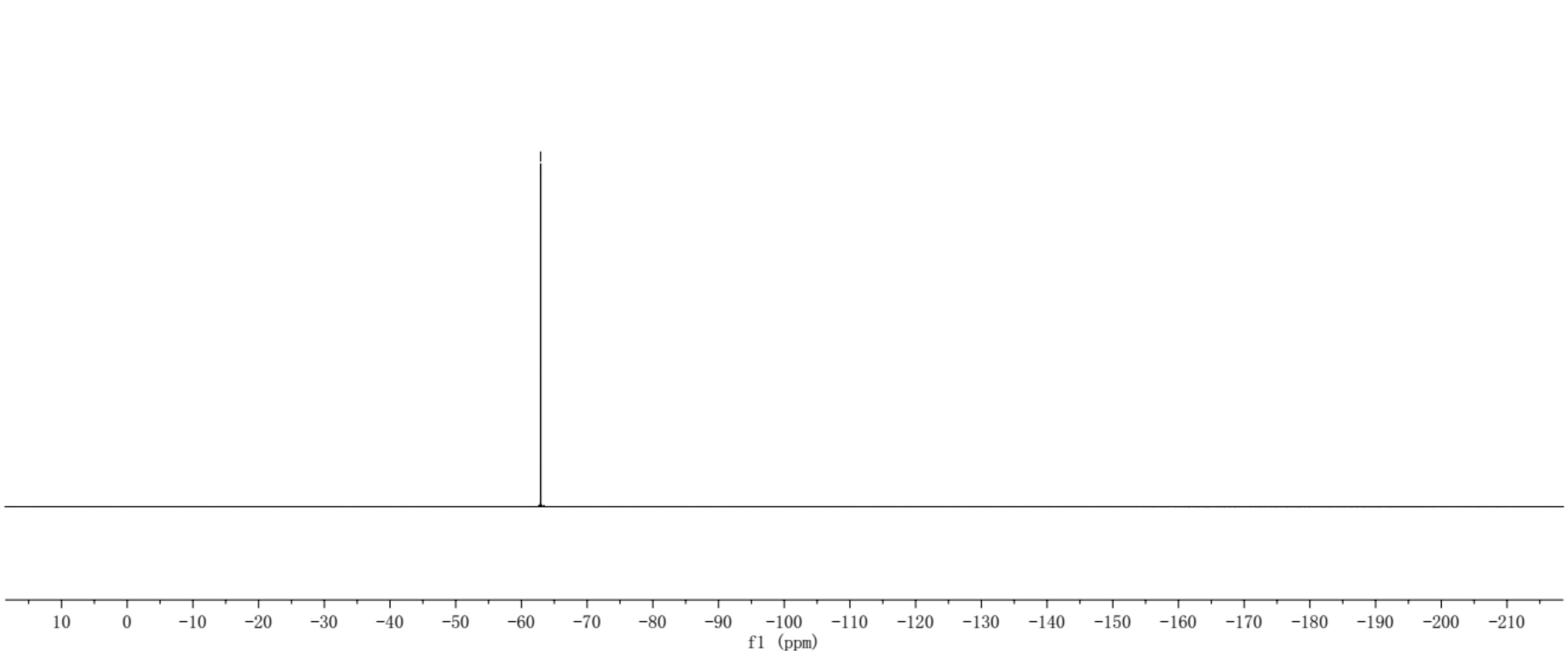

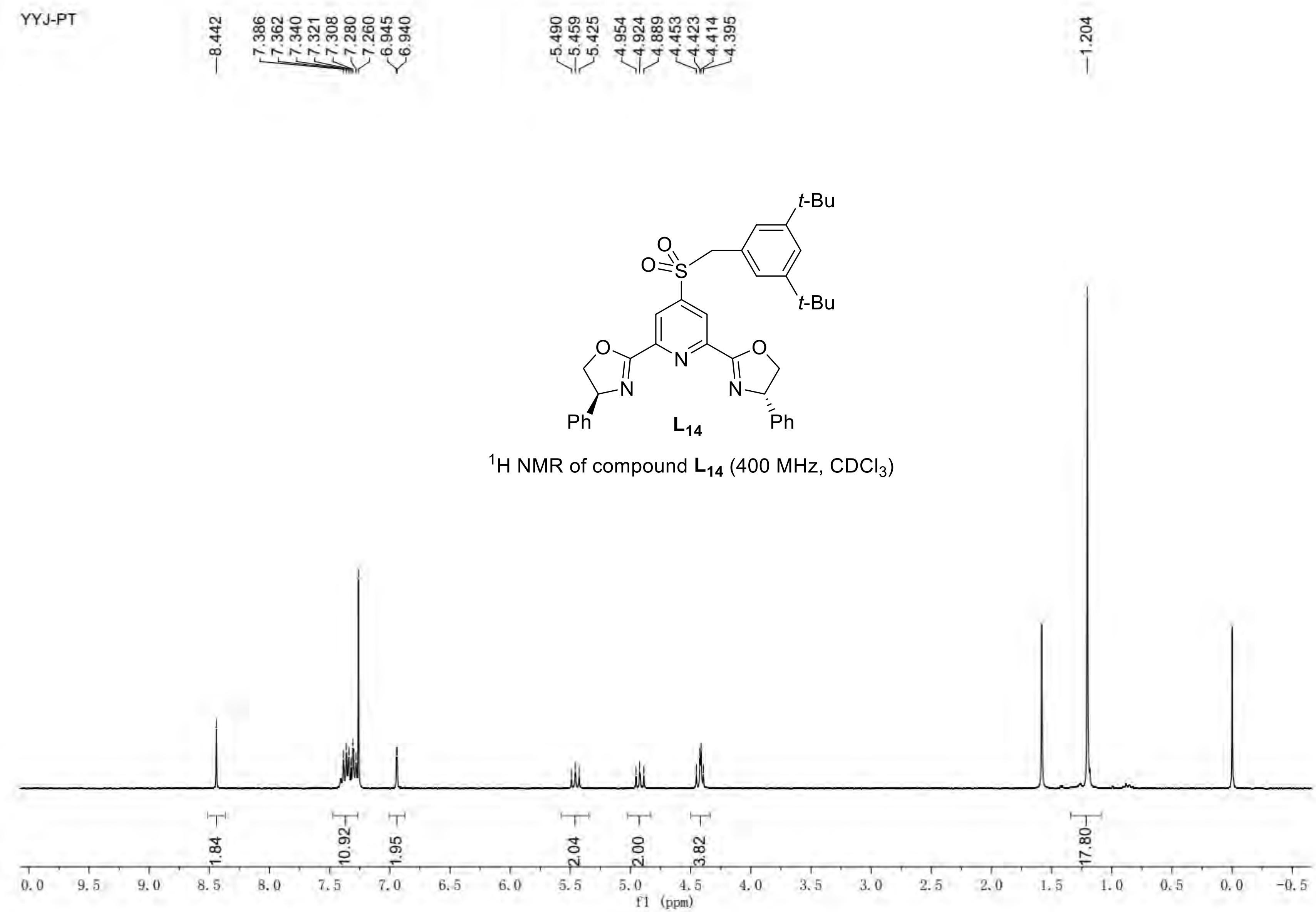

^{1}H NMR of compound **L_{14}** (400 MHz, $CDCl_3$)

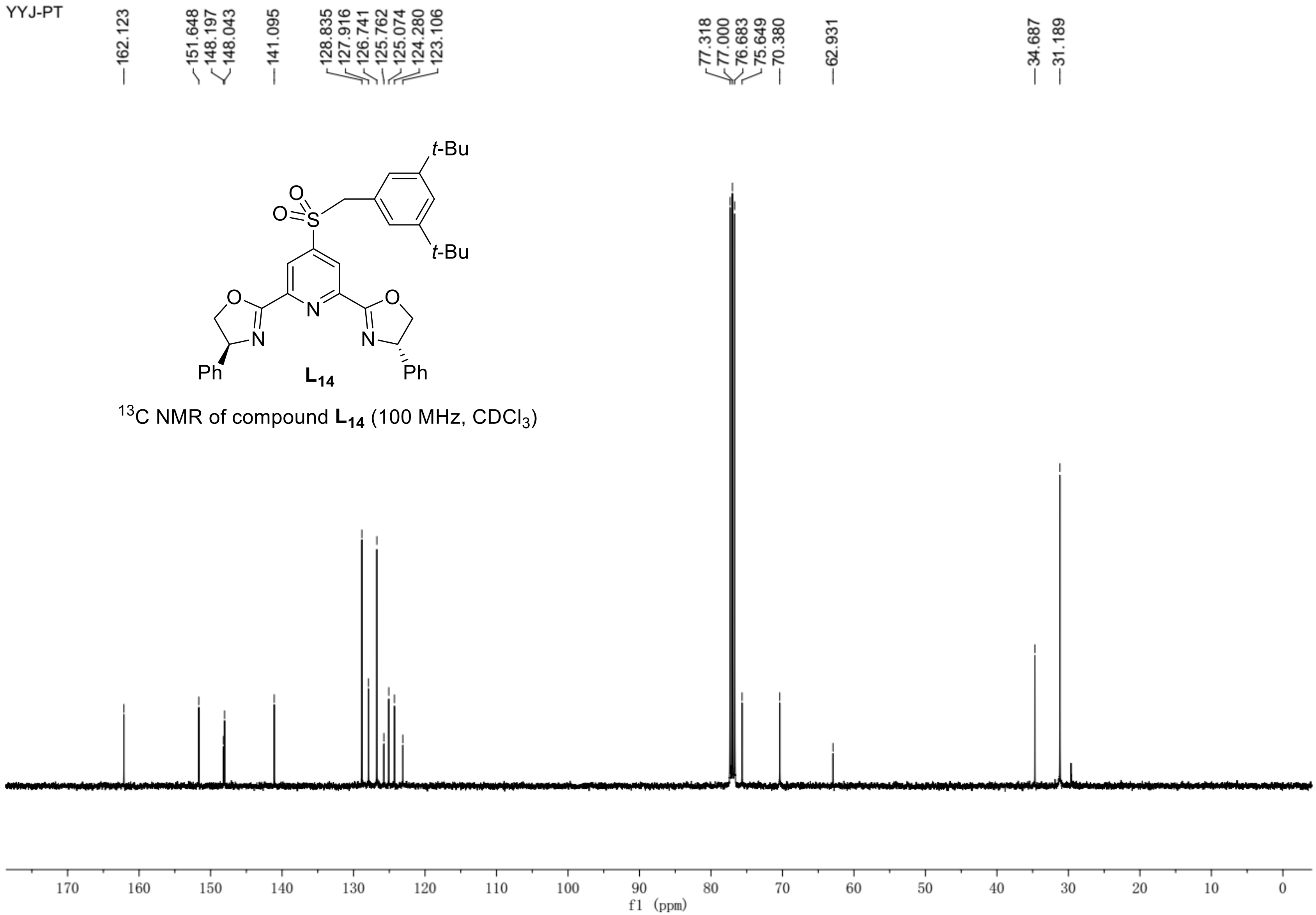

^{13}C NMR of compound **L$_{14}$** (100 MHz, $CDCl_3$)

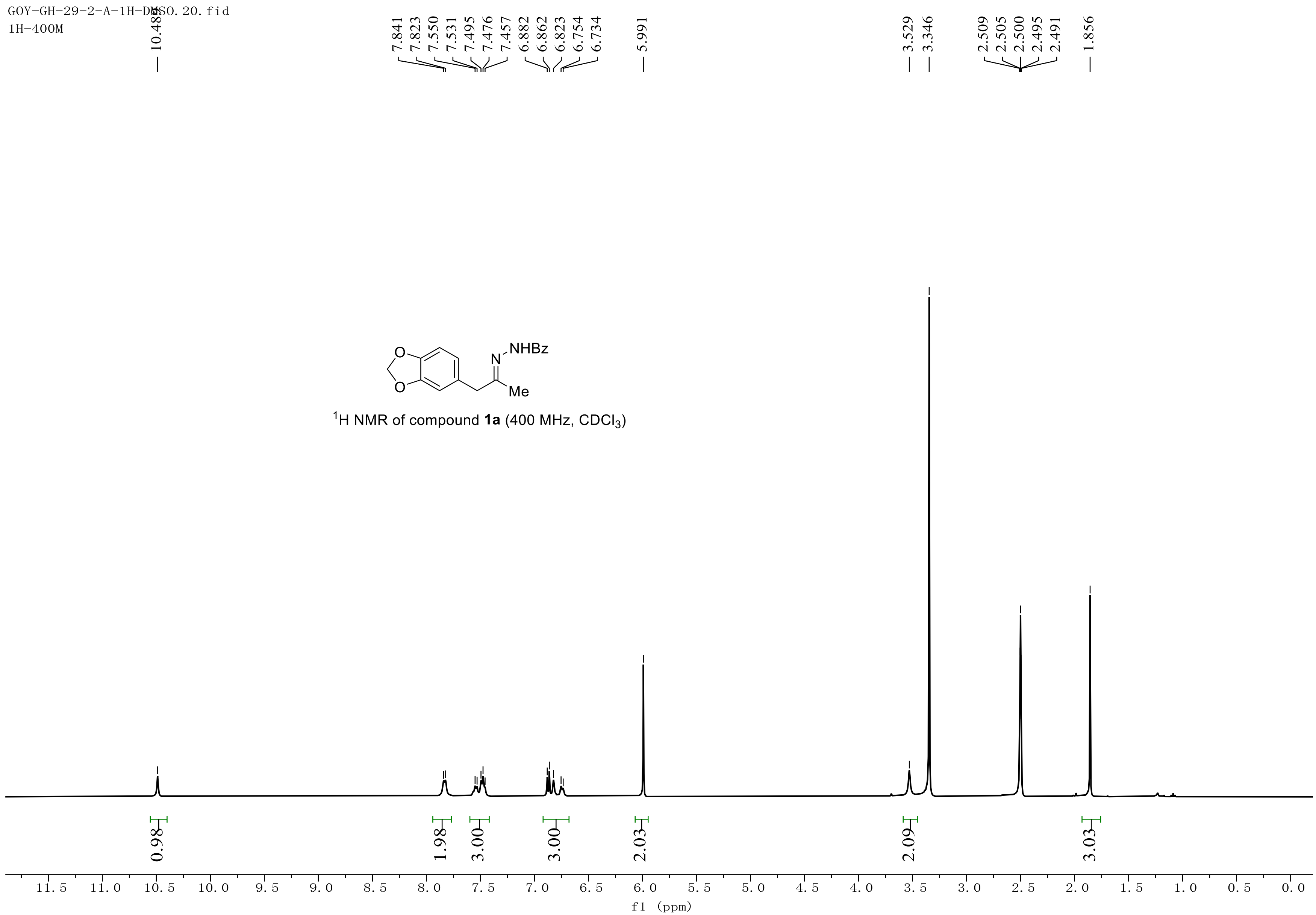

^{1}H NMR of compound **1a** (400 MHz, $CDCl_3$)

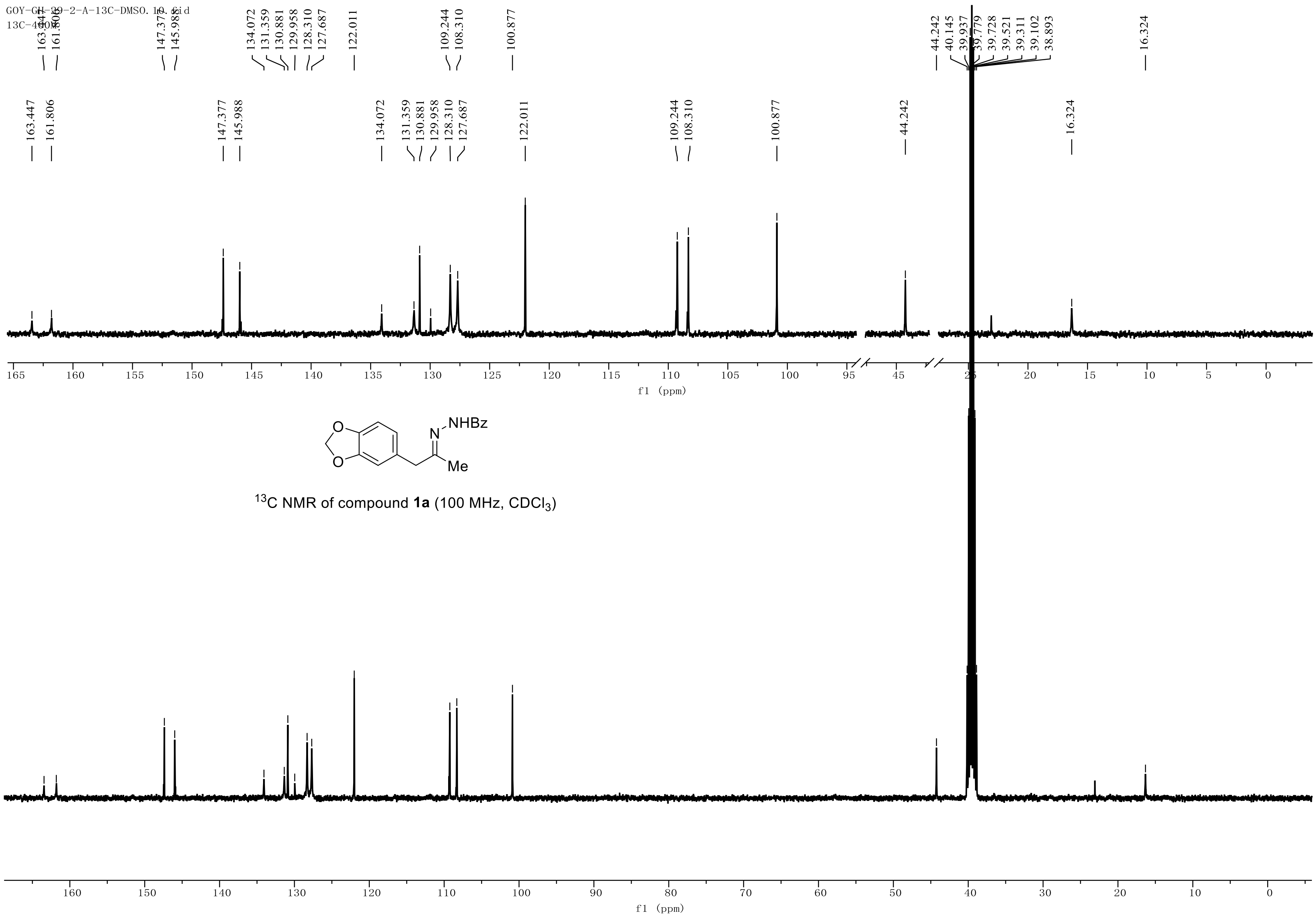

^{13}C NMR of compound **1a** (100 MHz, $CDCl_3$)

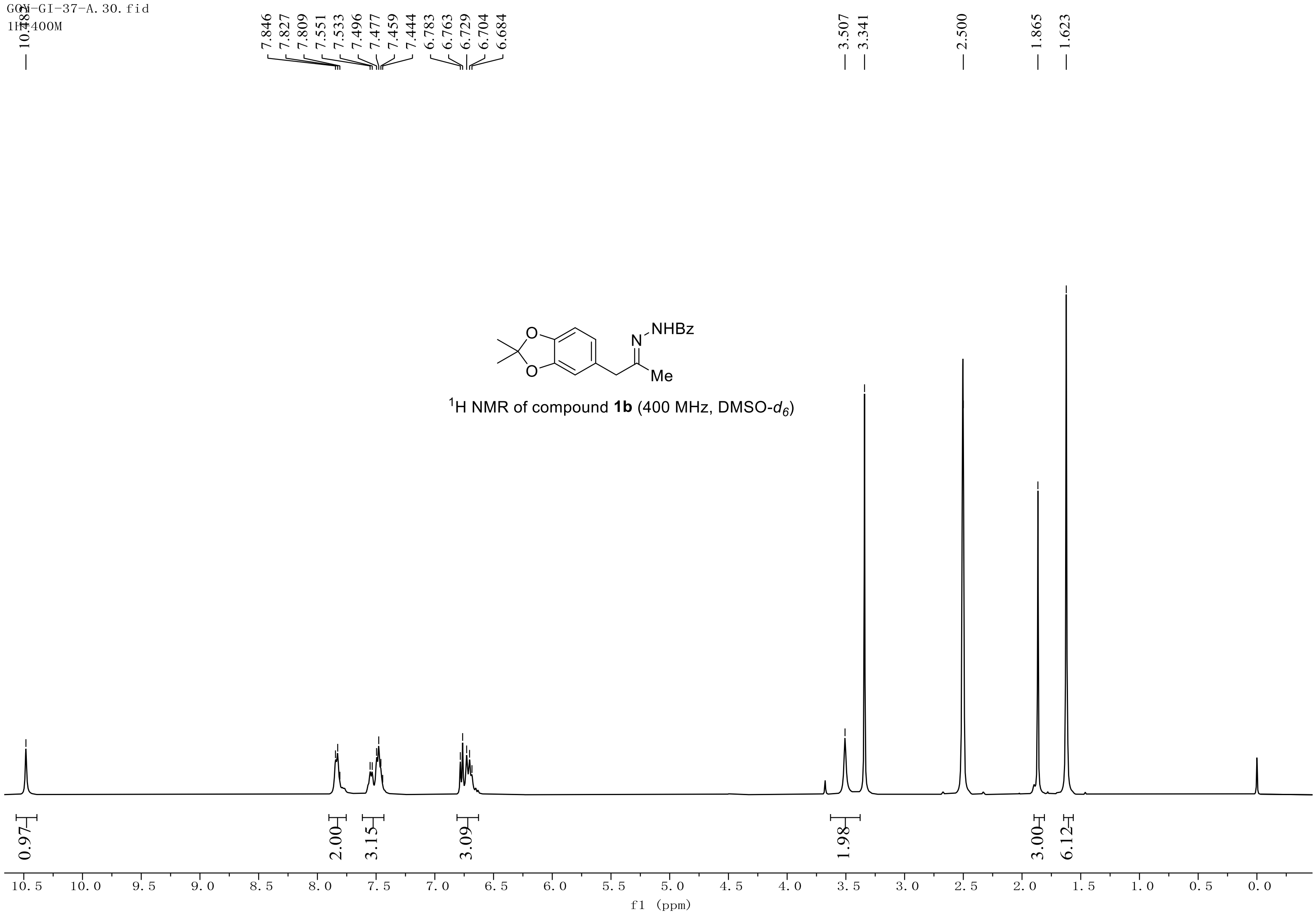

^{1}H NMR of compound **1b** (400 MHz, DMSO-d_6)

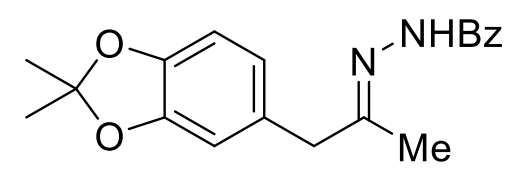

^{13}C NMR of compound **1b** (100 MHz, DMSO-d_6)

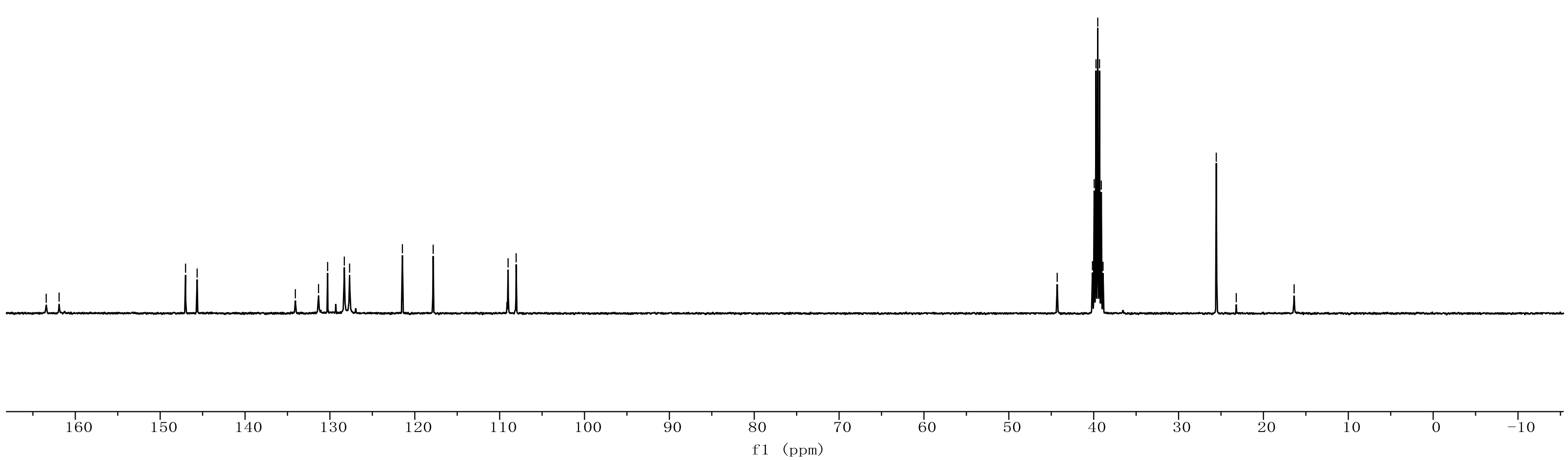

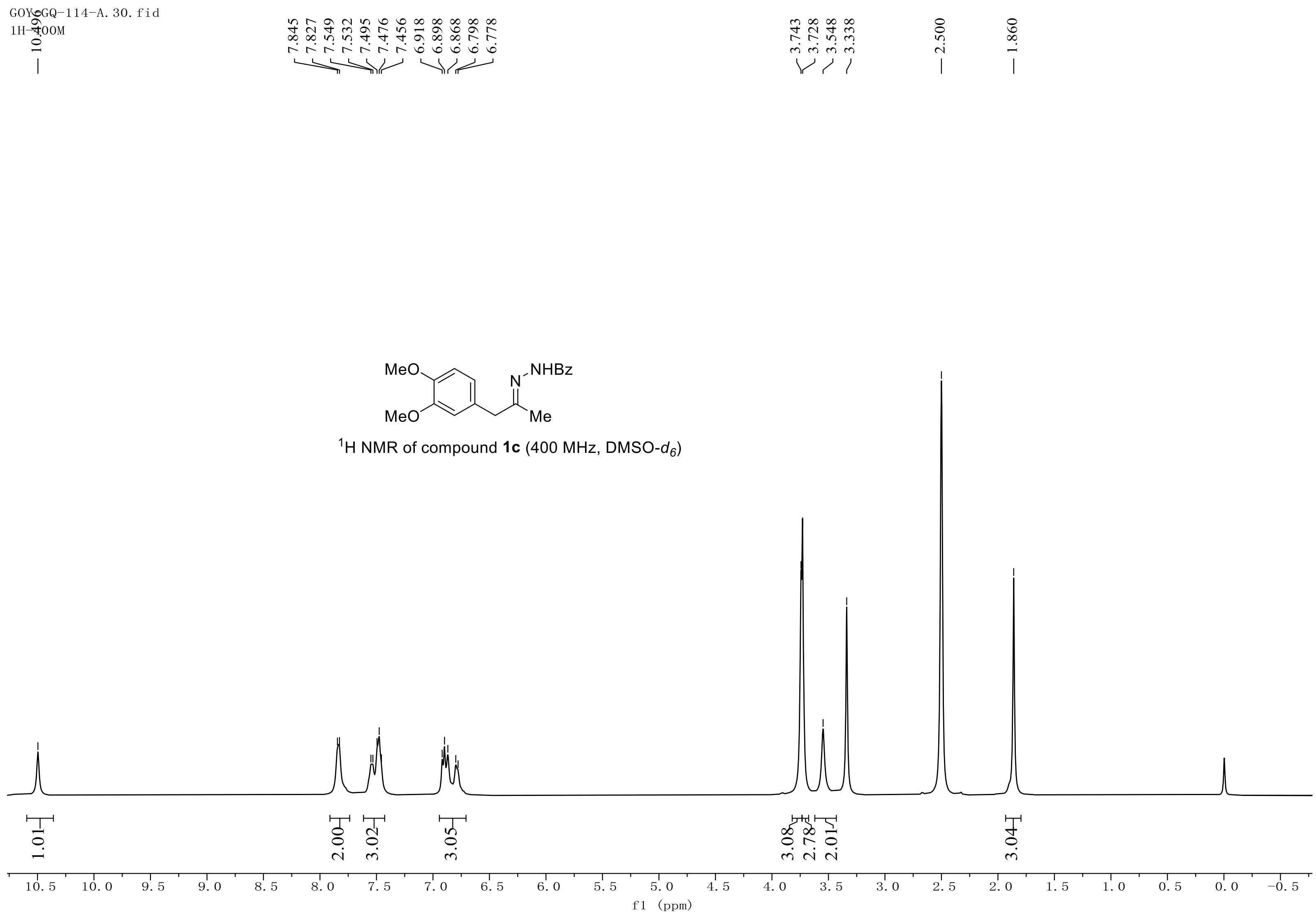

^{1}H NMR of compound **1c** (400 MHz, DMSO-d_6)

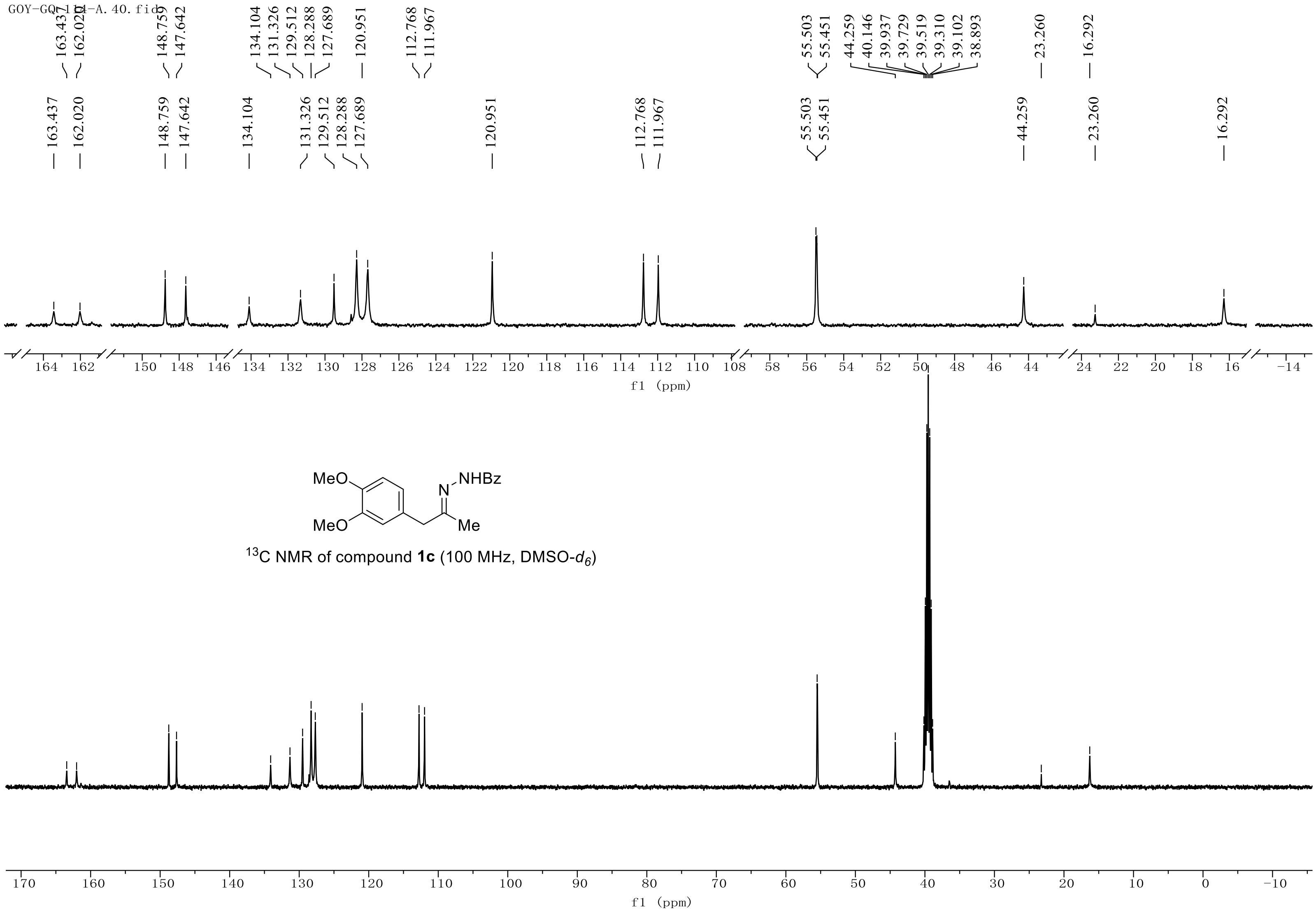

^{13}C NMR of compound **1c** (100 MHz, DMSO-d_6)

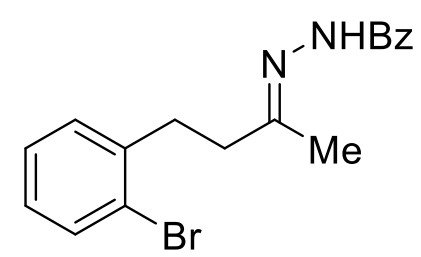

^{1}H NMR of compound **1d** (400 MHz, DMSO-d_6)

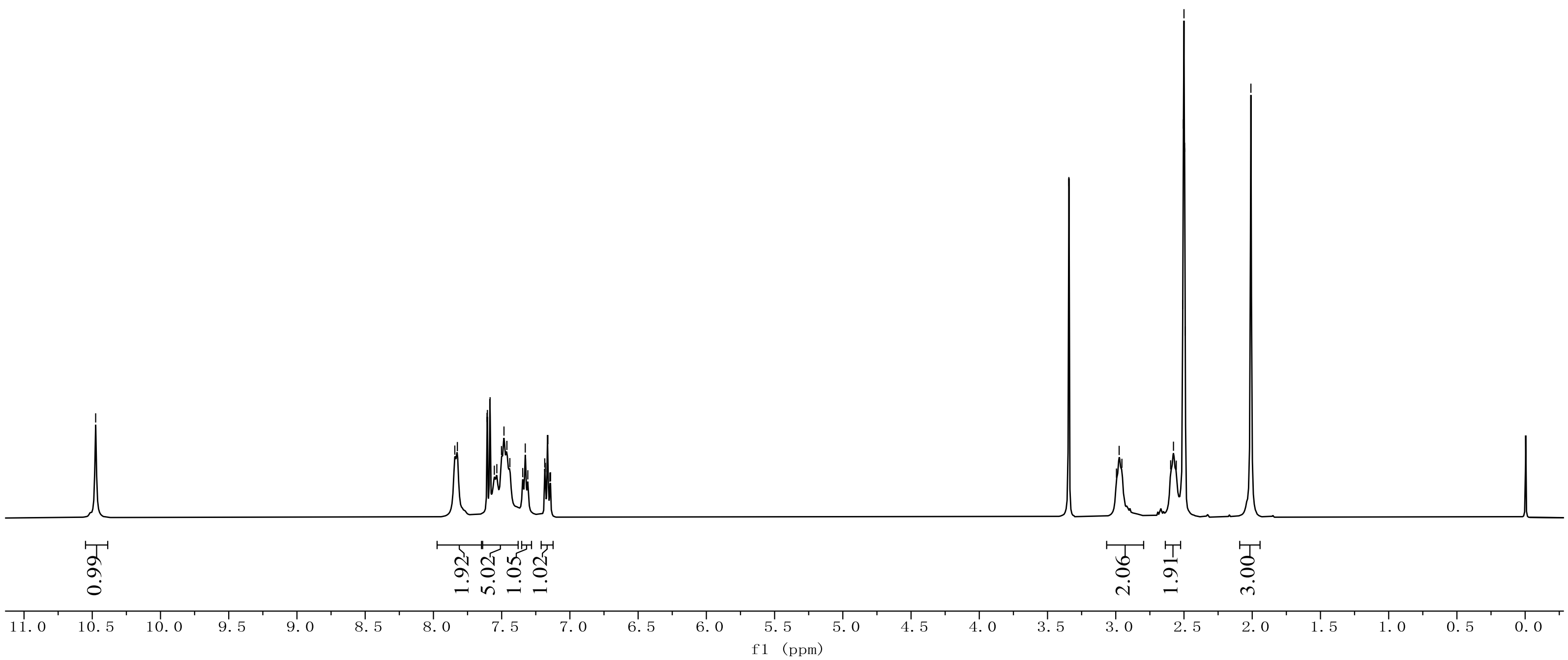

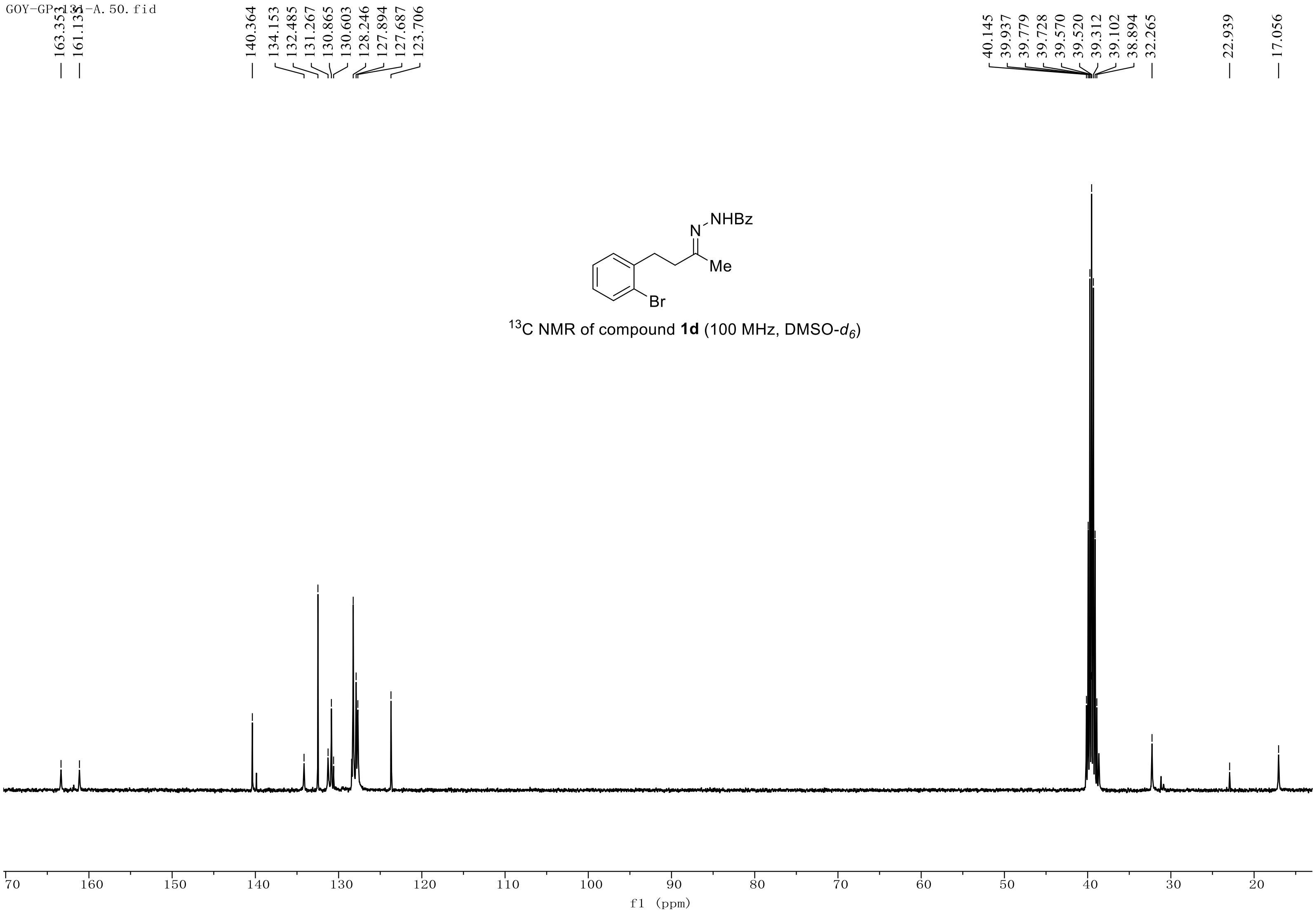

^{13}C NMR of compound **1d** (100 MHz, DMSO-d_6)

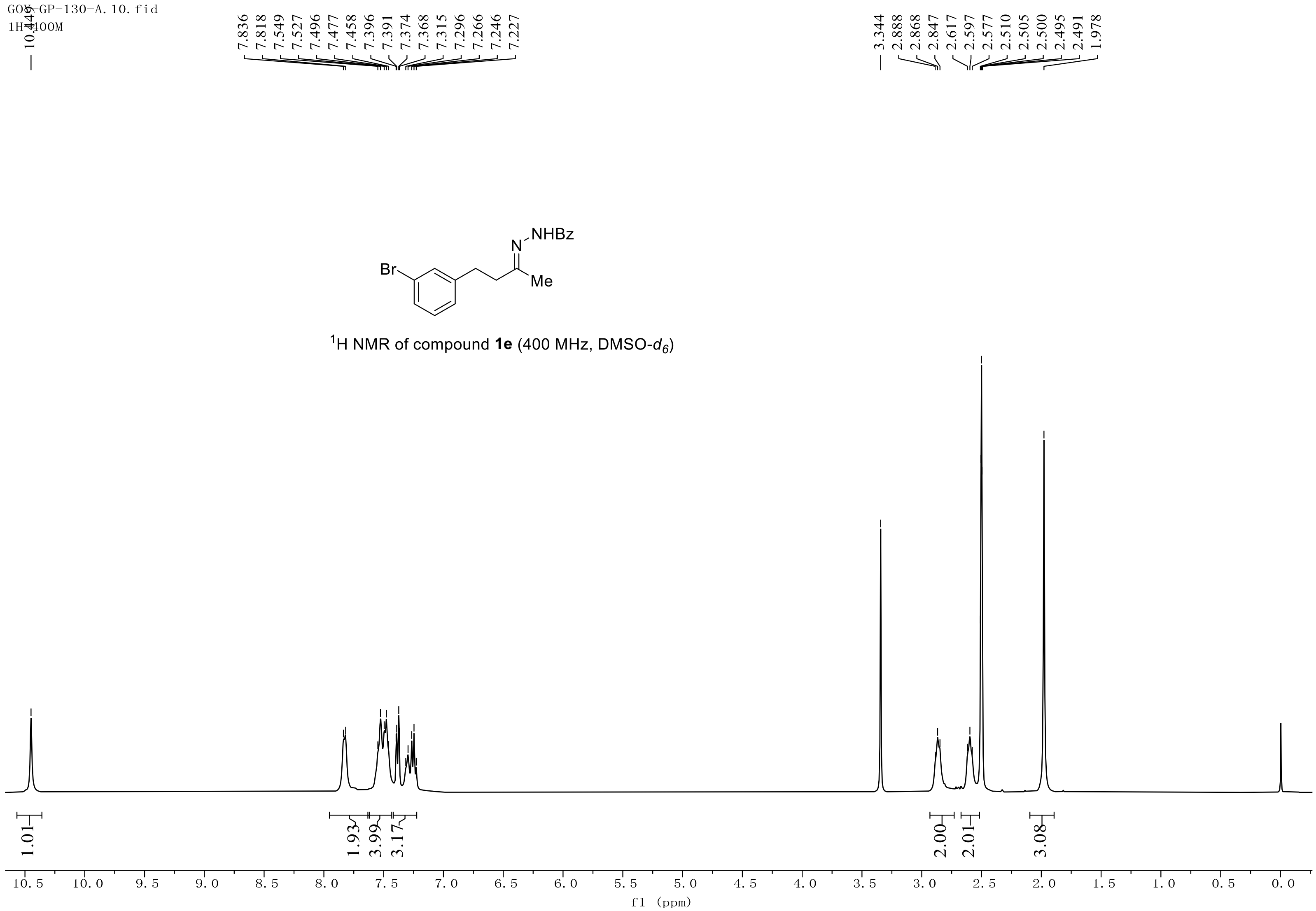

^{1}H NMR of compound **1e** (400 MHz, DMSO-d_6)

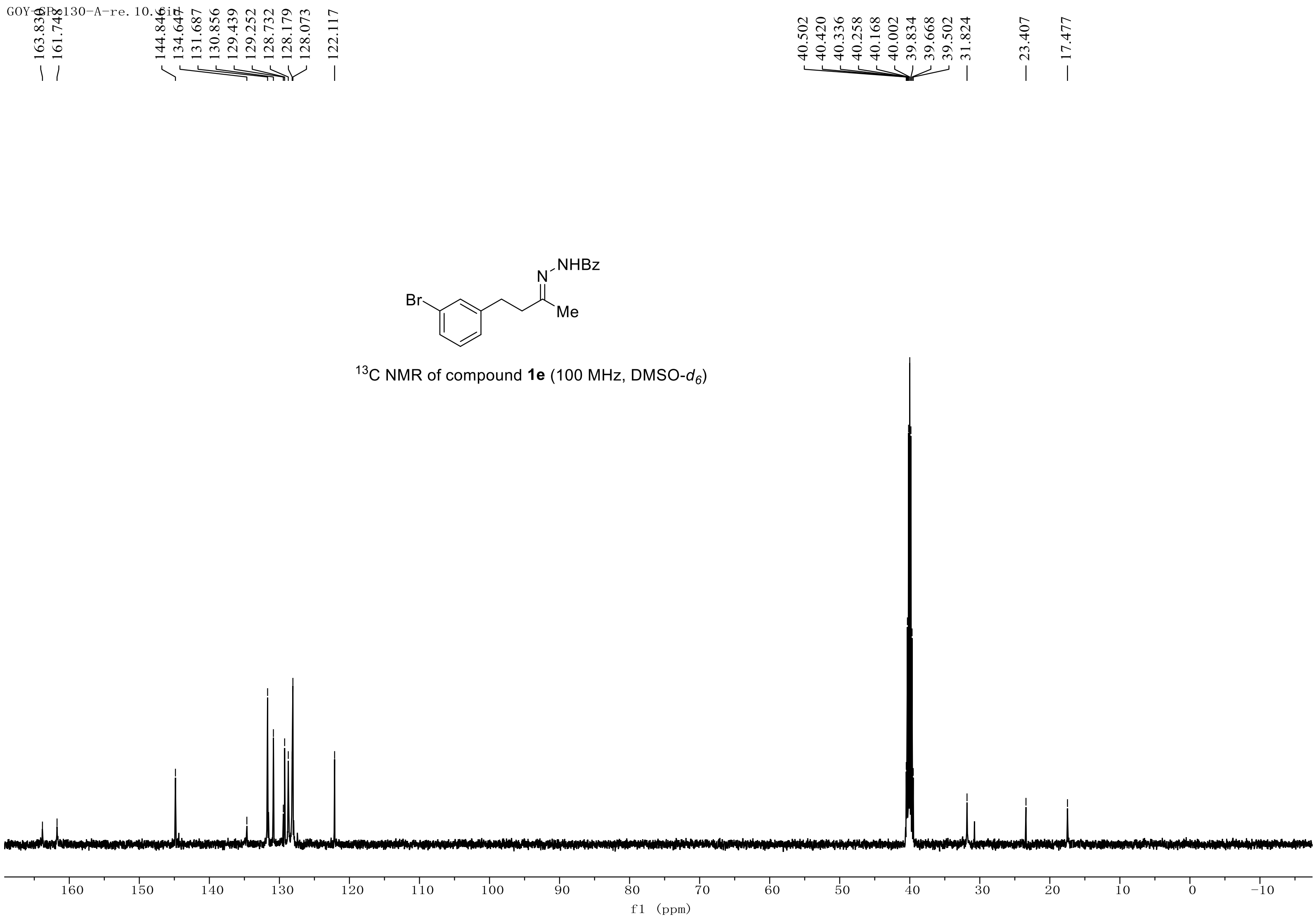

^{13}C NMR of compound **1e** (100 MHz, DMSO-d_6)

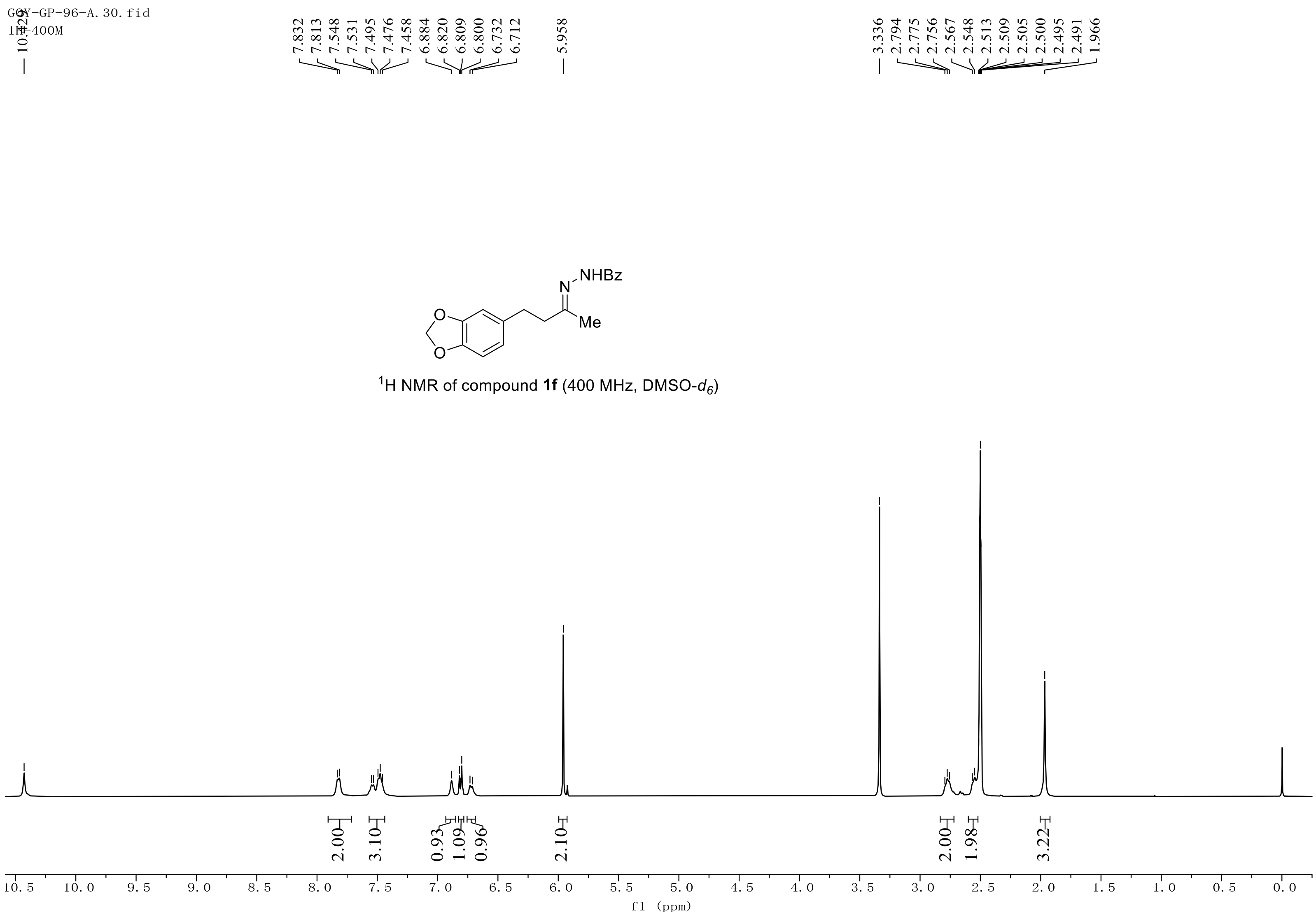

^{1}H NMR of compound **1f** (400 MHz, DMSO-d_6)

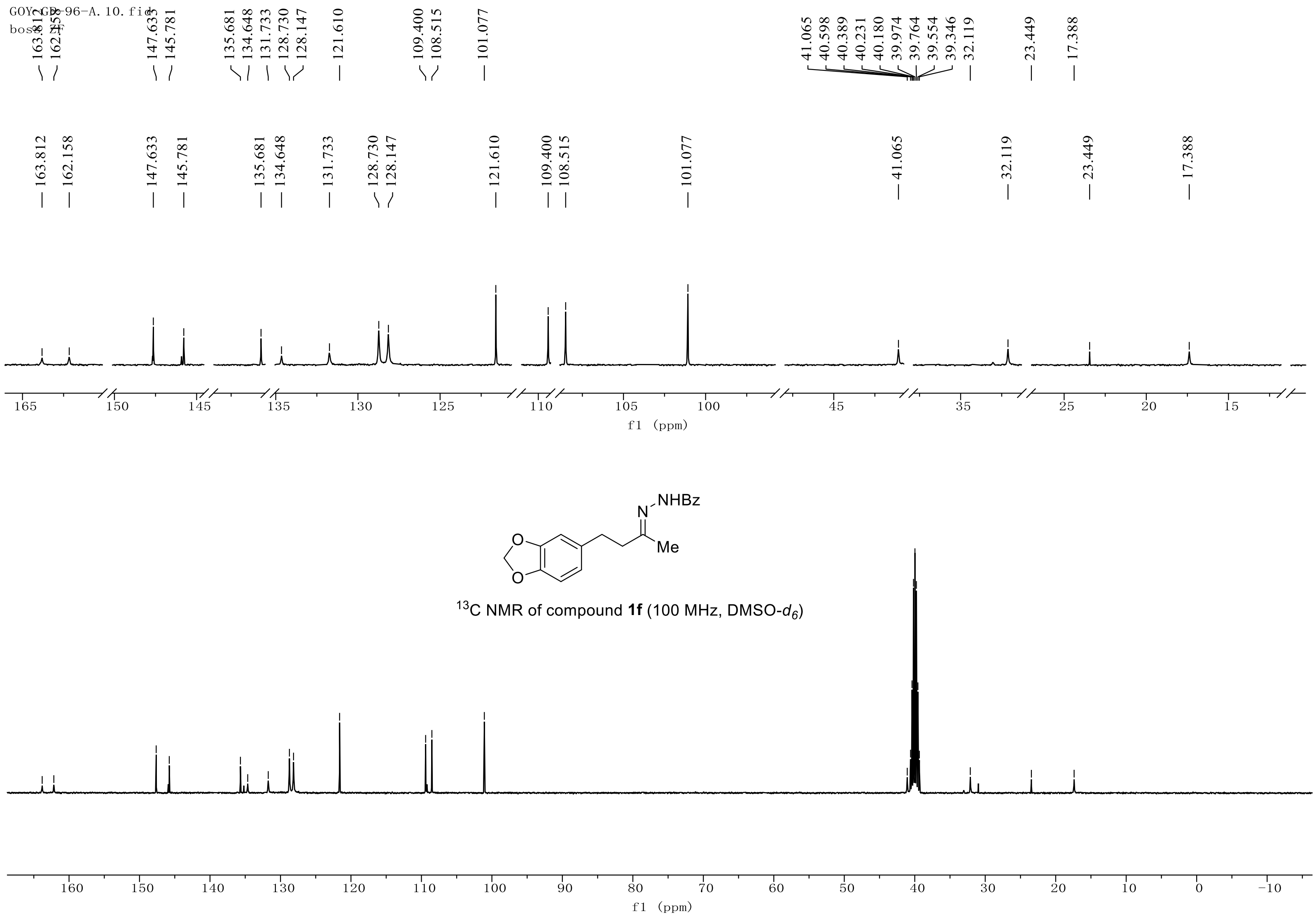

^{13}C NMR of compound **1f** (100 MHz, DMSO-d_6)

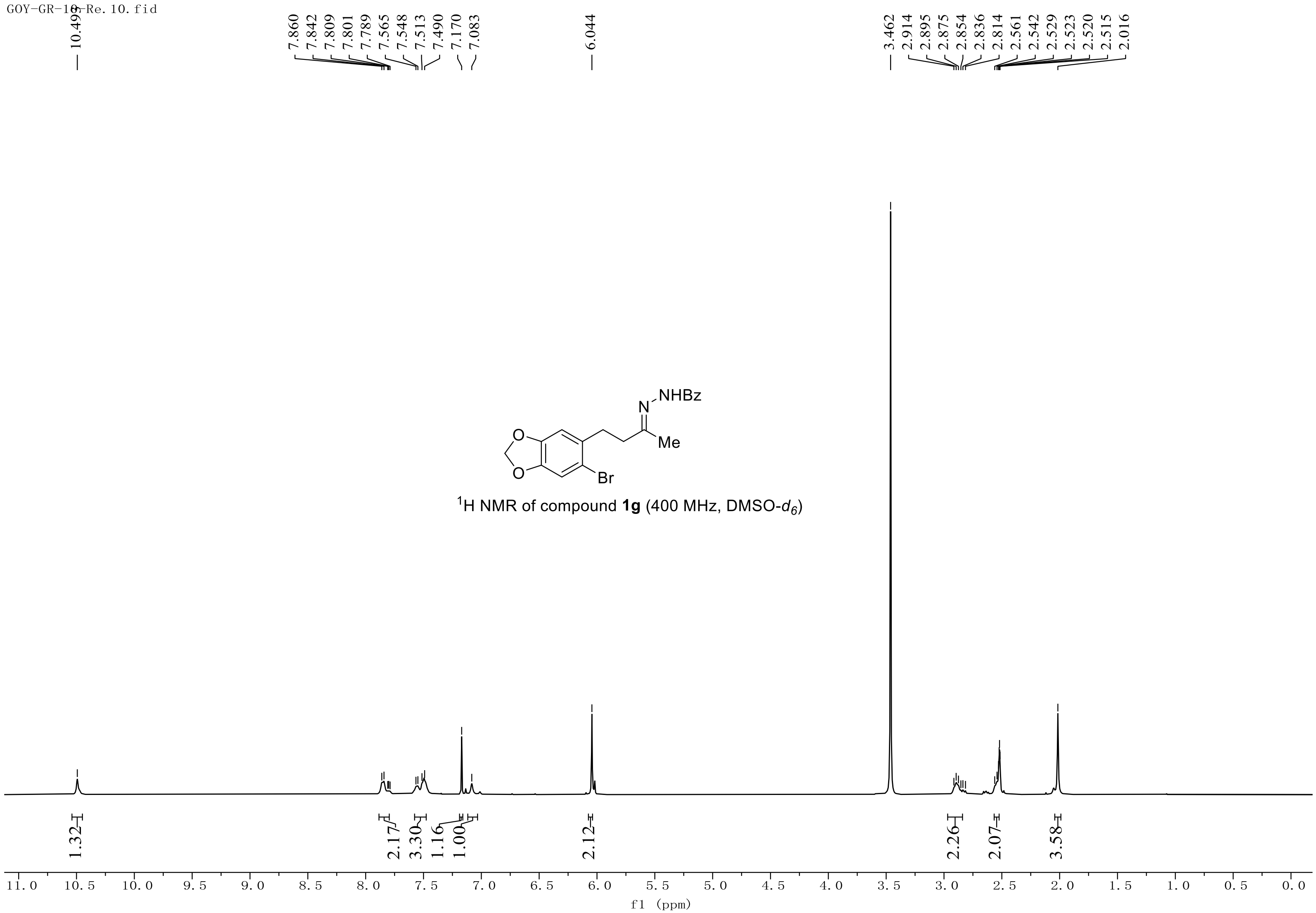

^{1}H NMR of compound **1g** (400 MHz, DMSO-d_6)

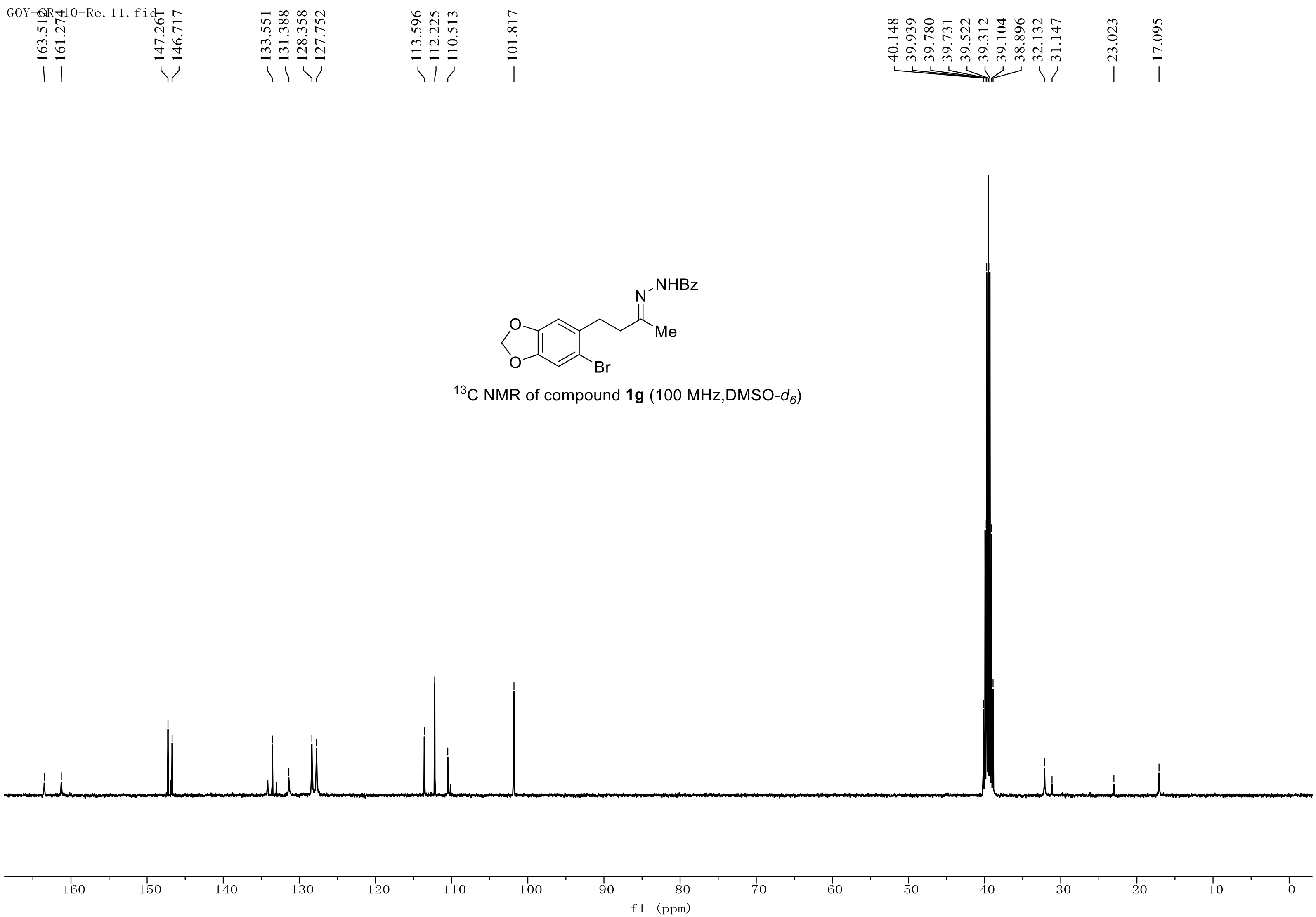

^{13}C NMR of compound **1g** (100 MHz,DMSO-d_6)

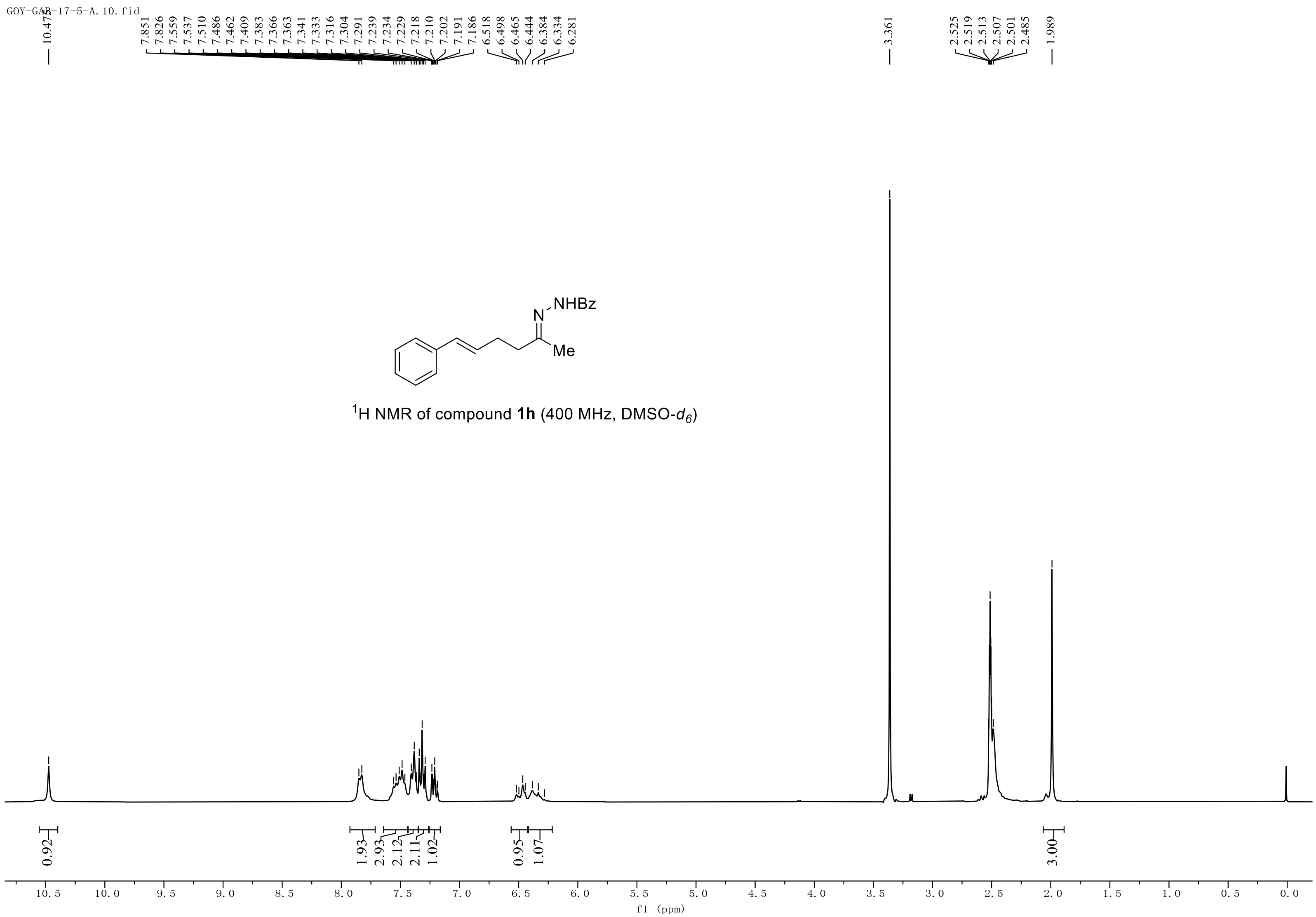

^{1}H NMR of compound **1h** (400 MHz, DMSO-d_6)

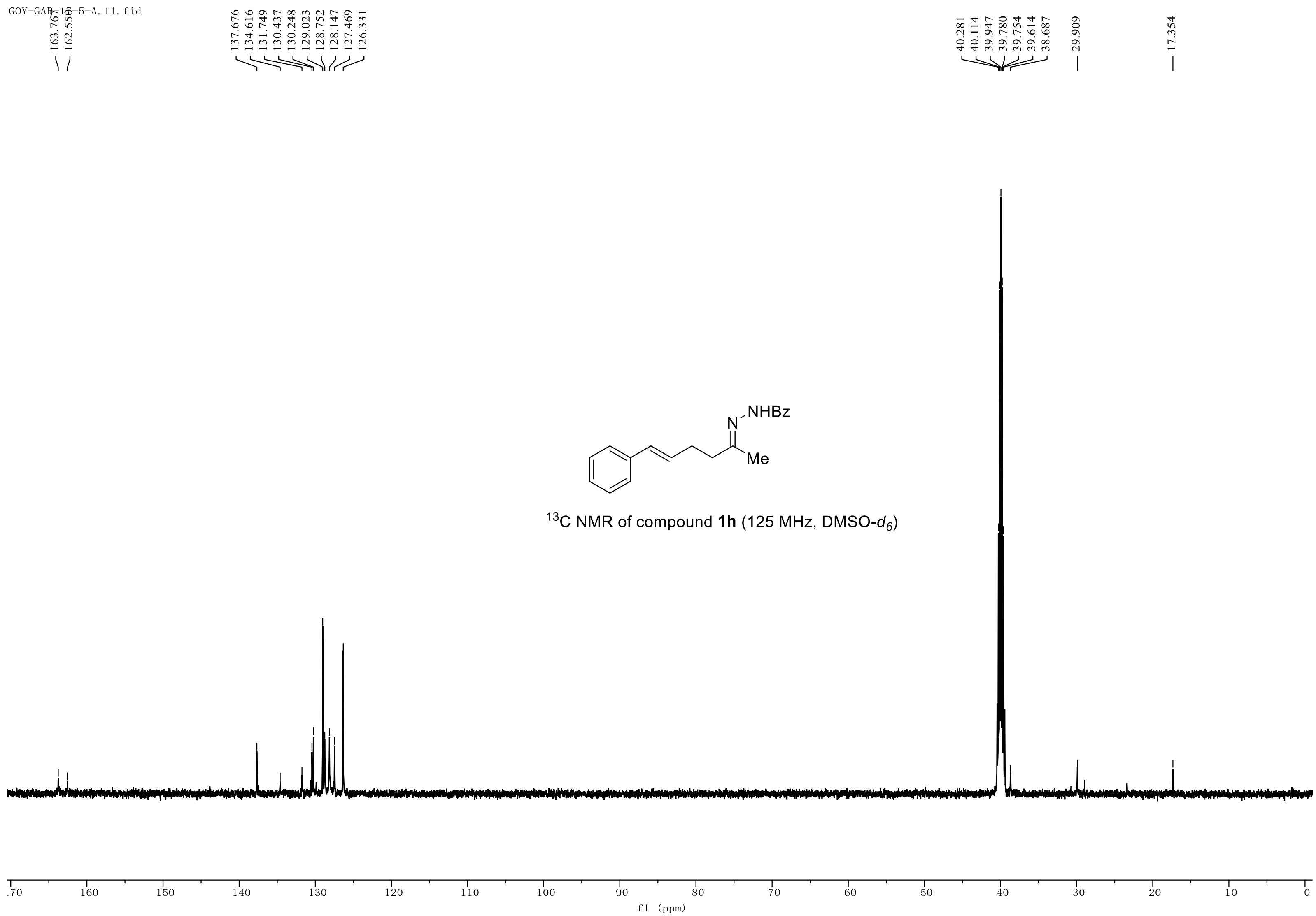

^{13}C NMR of compound **1h** (125 MHz, DMSO-d_6)

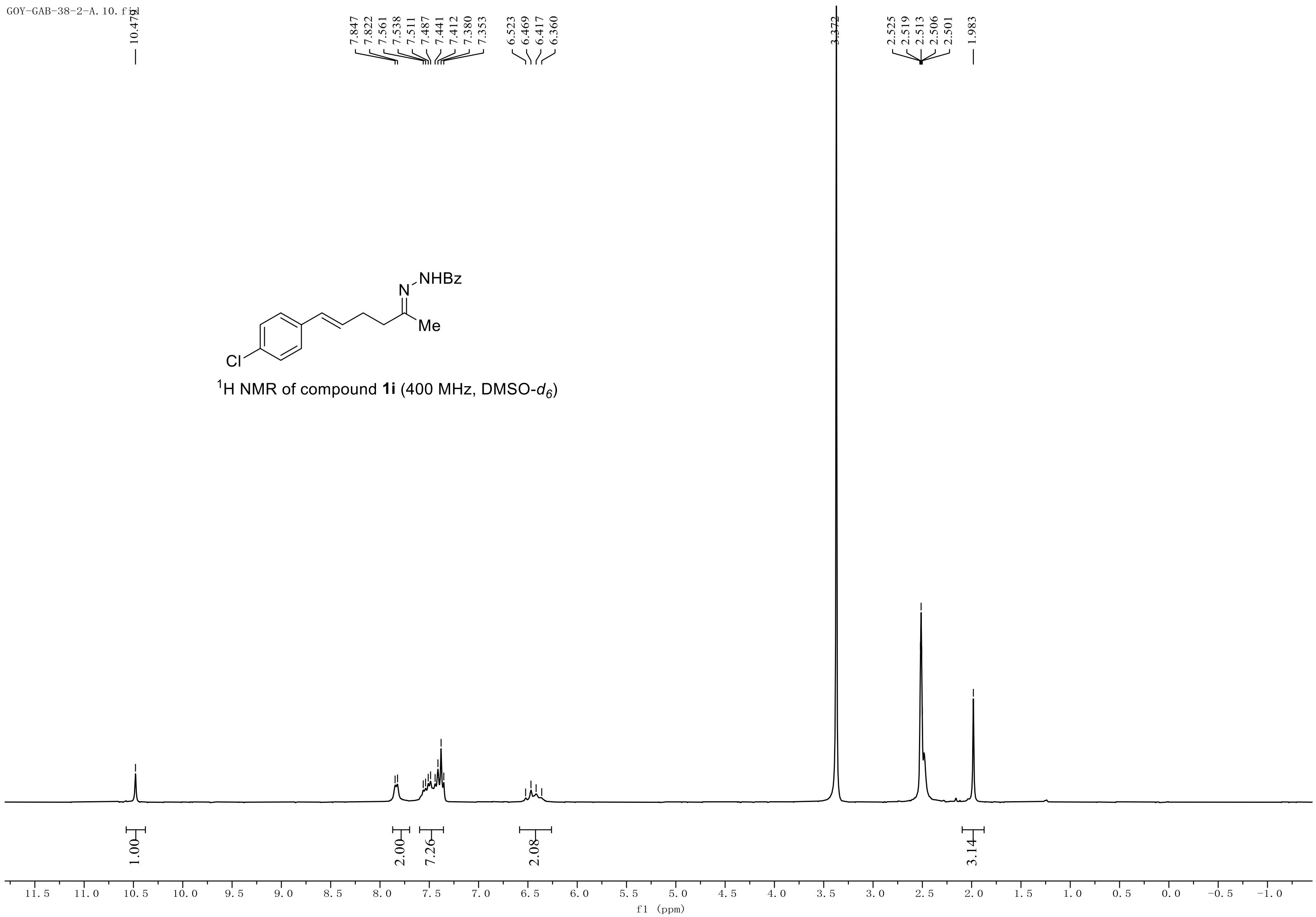

^{1}H NMR of compound **1i** (400 MHz, DMSO-d_6)

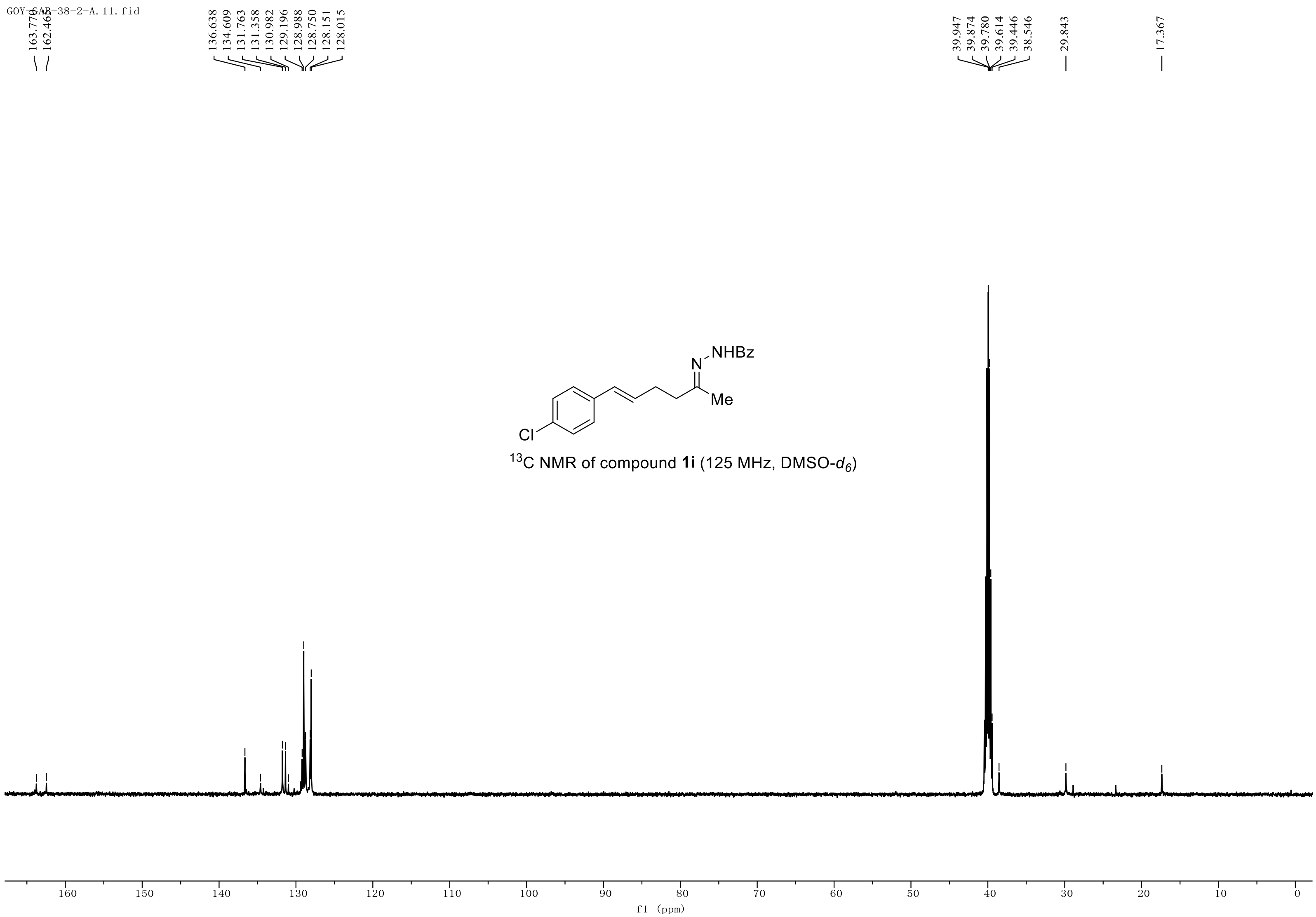

^{13}C NMR of compound **1i** (125 MHz, DMSO-d_6)

GOY-GAB-38-1-A.40.fid

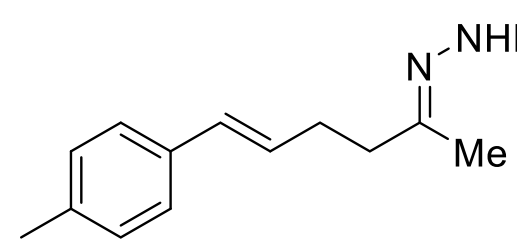

^{1}H NMR of compound **1j** (500 MHz, DMSO-d_6)

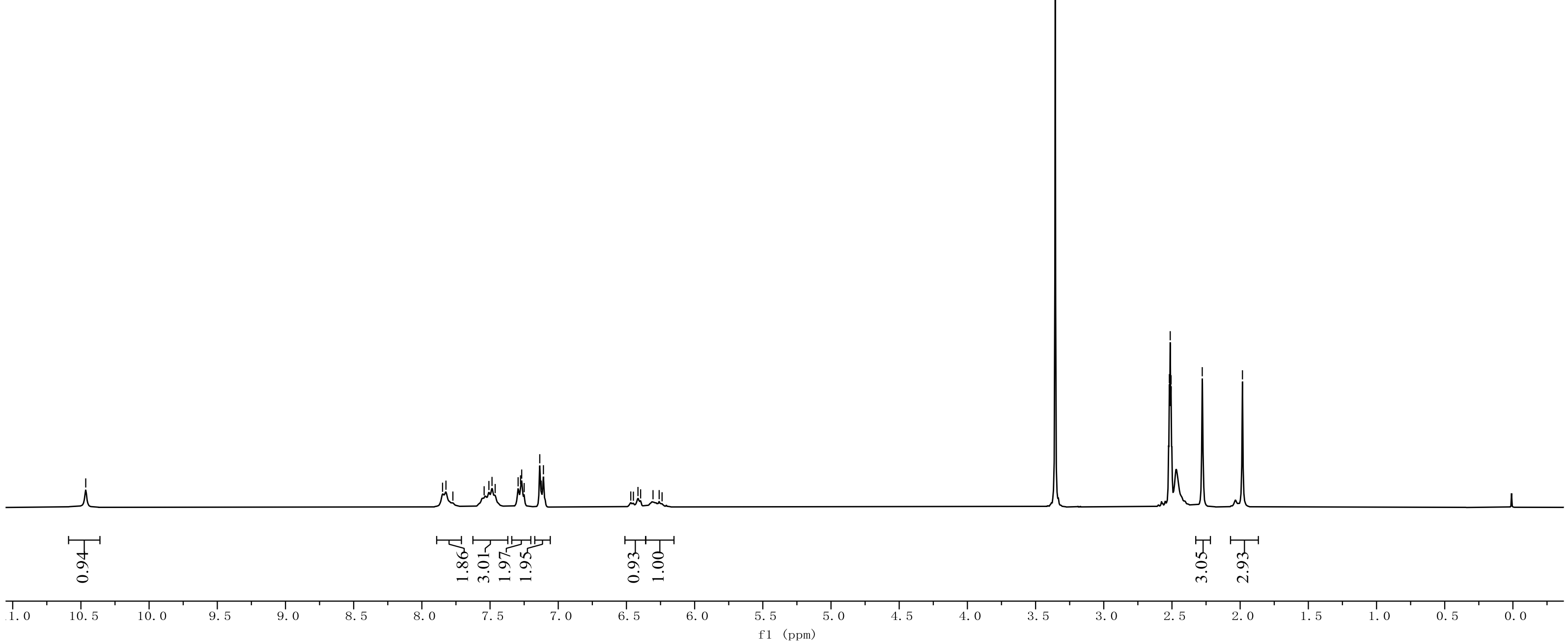

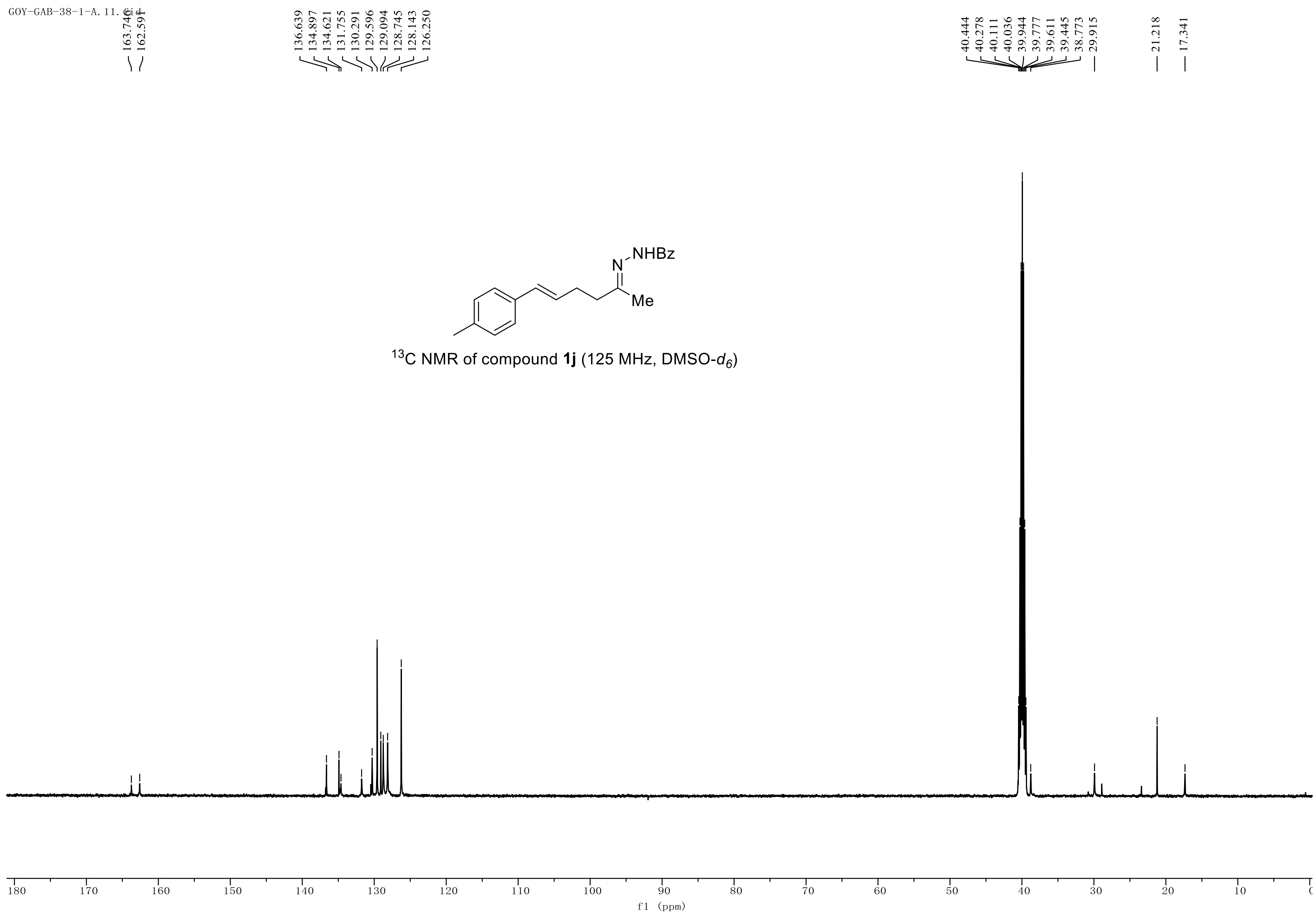

^{13}C NMR of compound **1j** (125 MHz, DMSO-d_6)

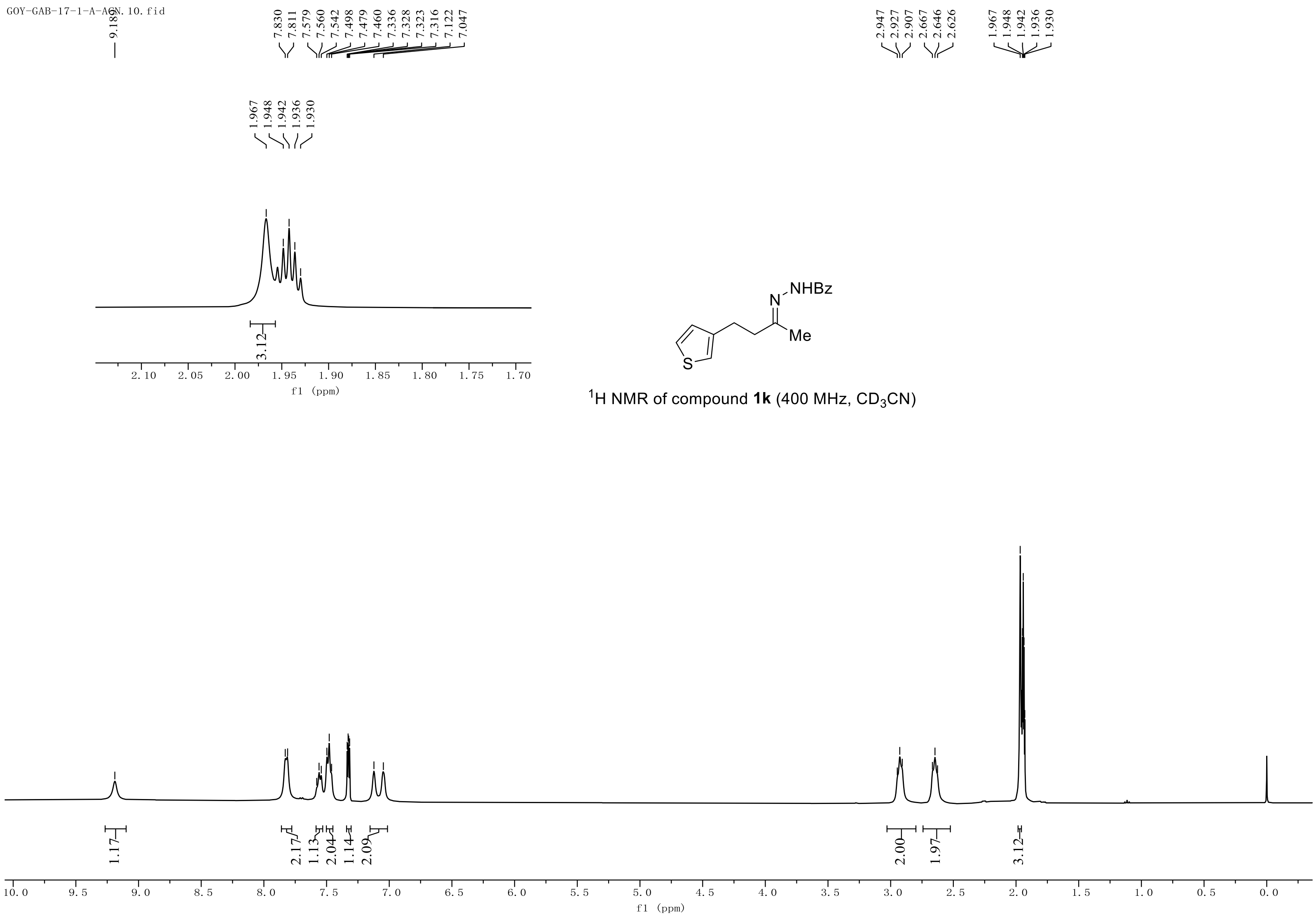

^{1}H NMR of compound **1k** (400 MHz, CD_3CN)

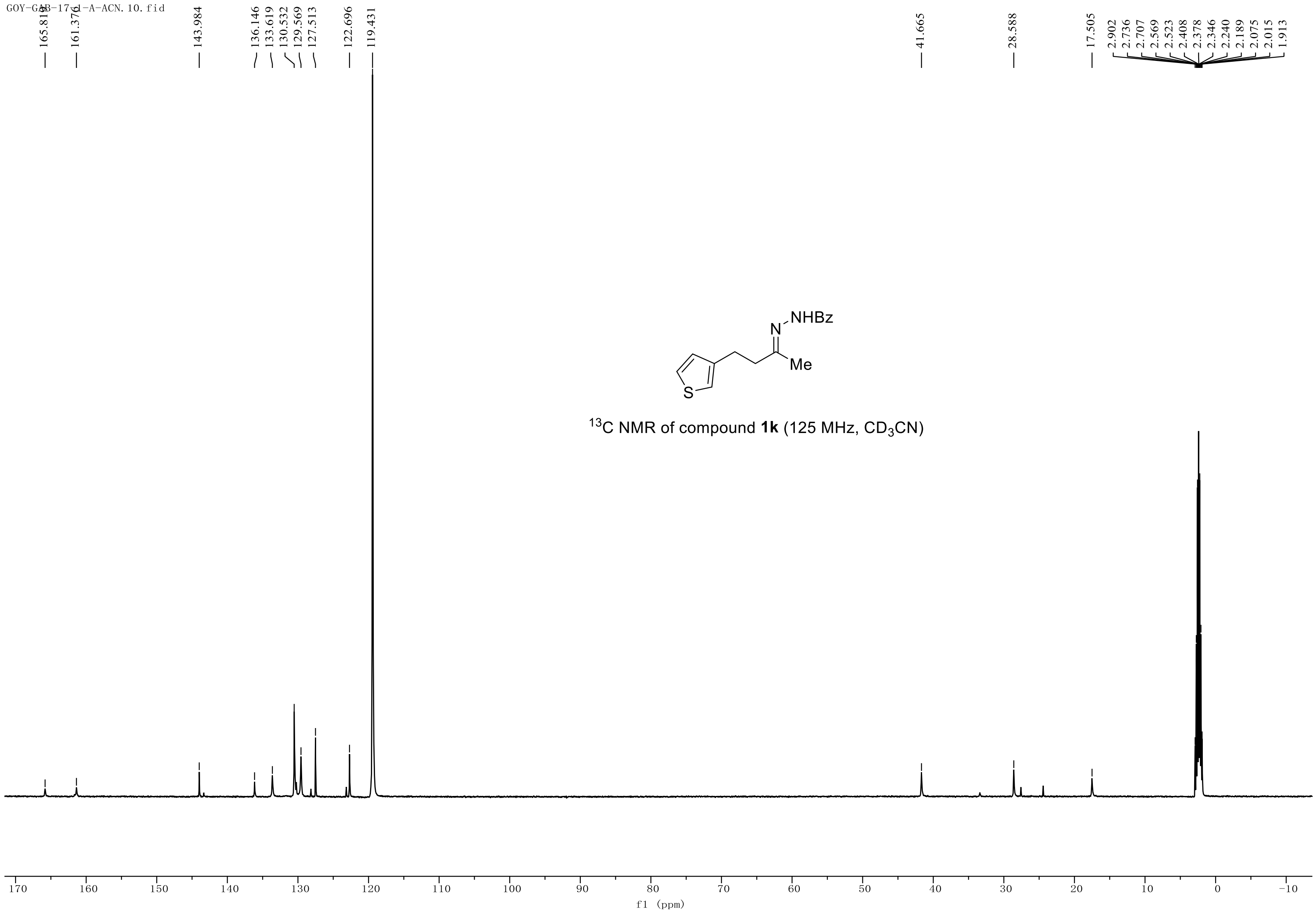

^{13}C NMR of compound **1k** (125 MHz, CD_3CN)

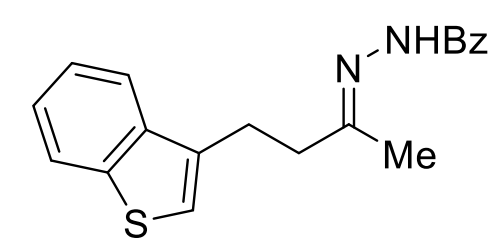

^{1}H NMR of compound **1l** (400 MHz, DMSO-d_6)

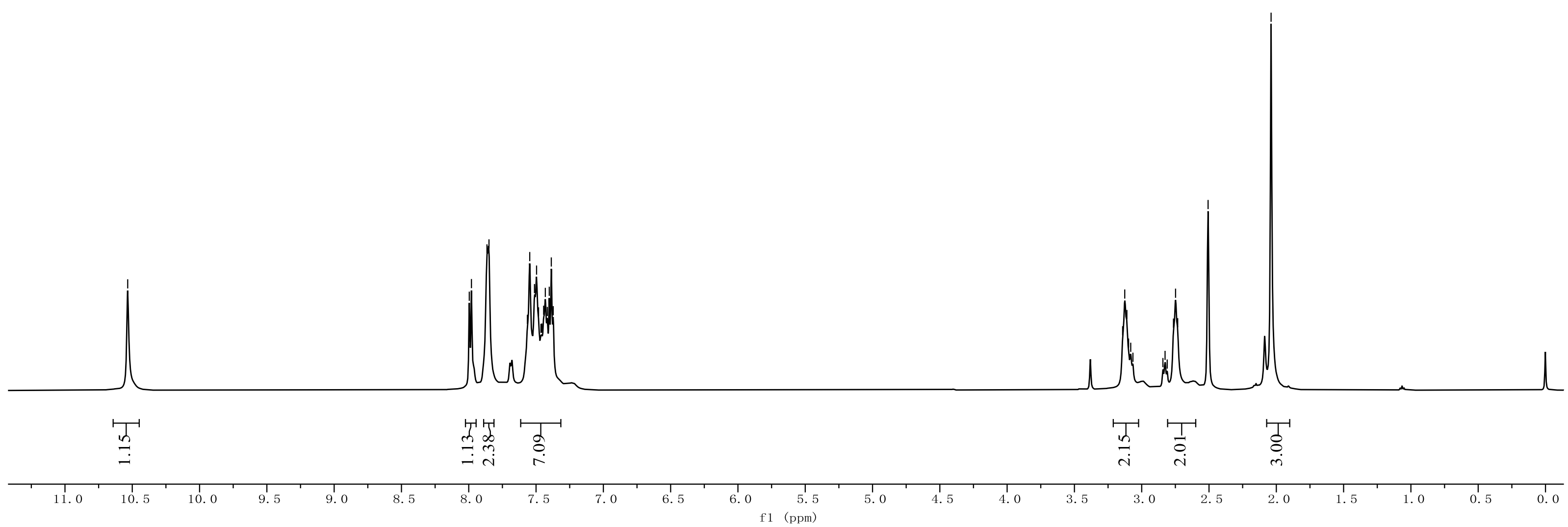

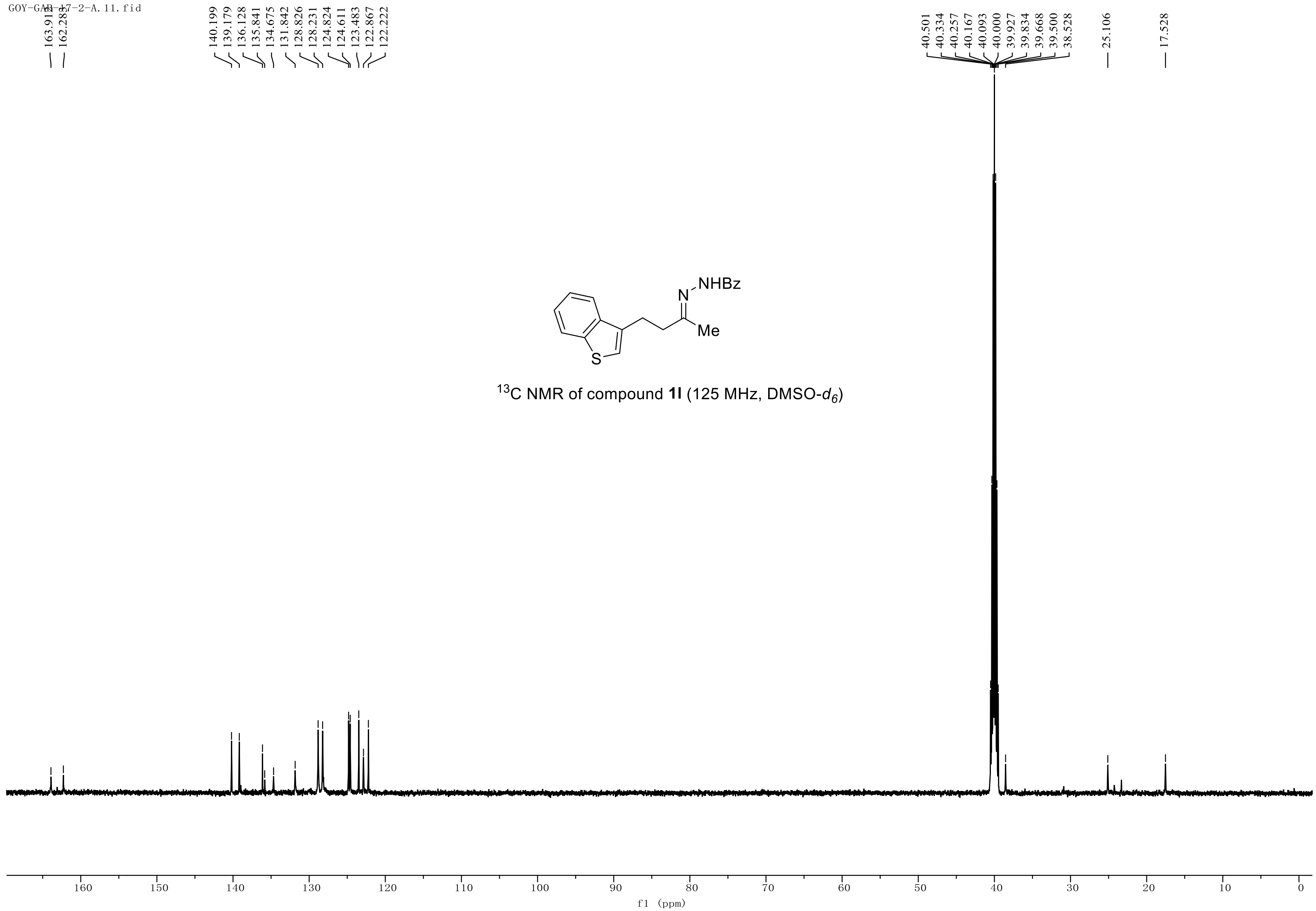

^{13}C NMR of compound **1l** (125 MHz, DMSO-d_6)

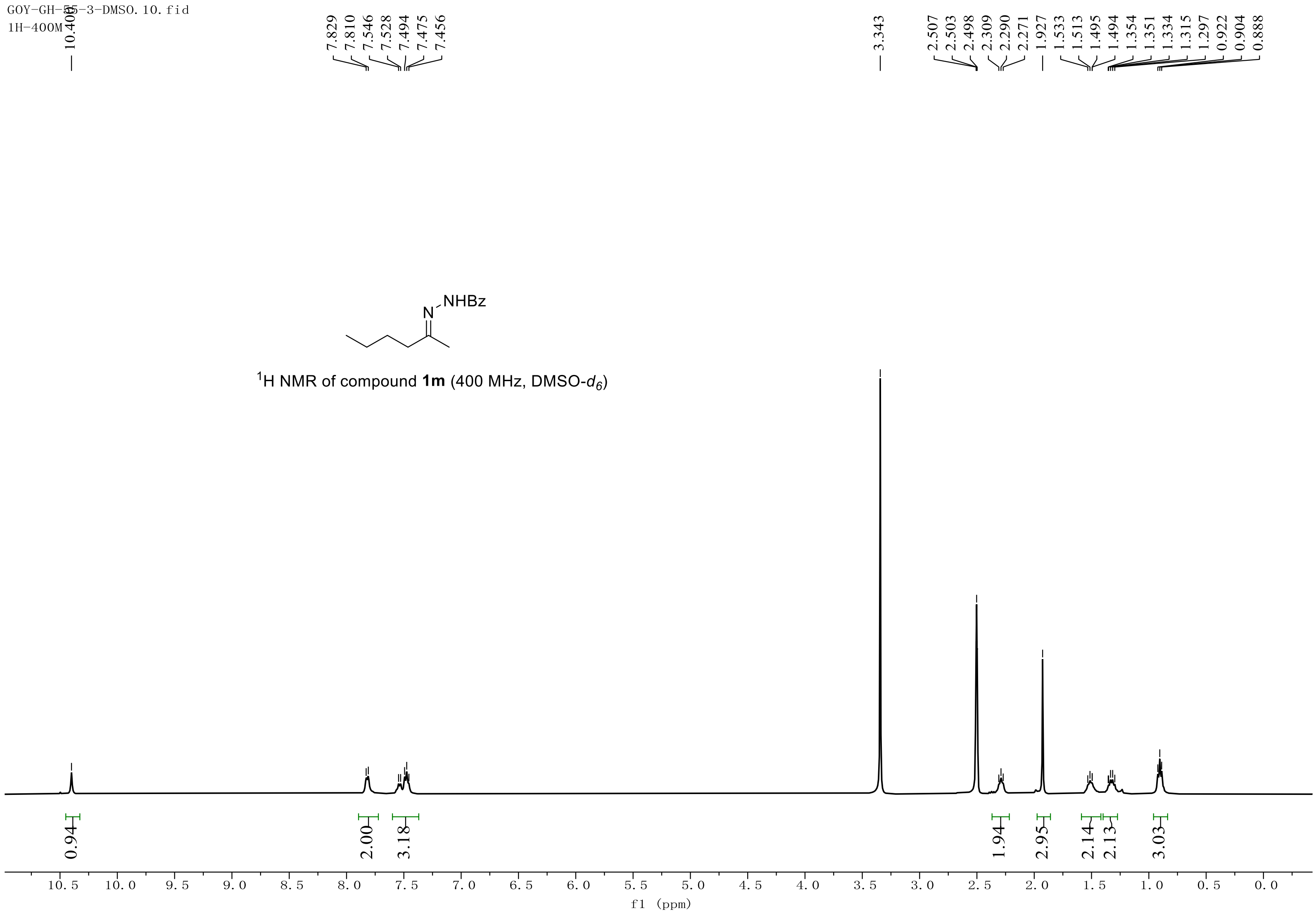

^{1}H NMR of compound **1m** (400 MHz, DMSO-d_6)

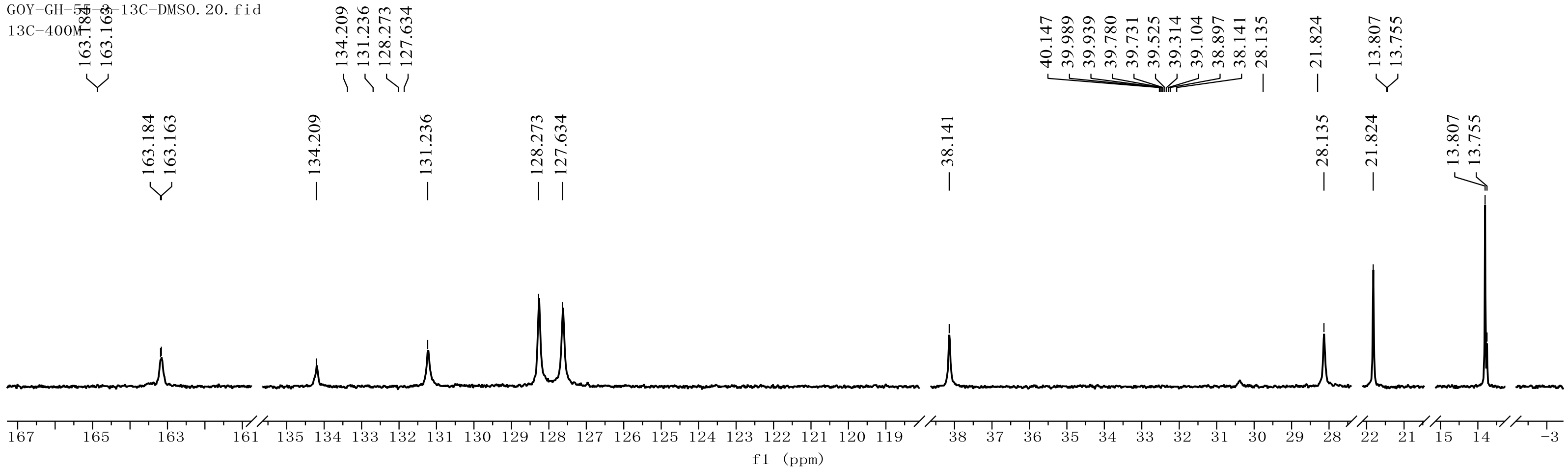

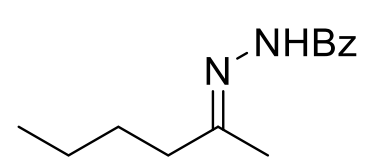

^{13}C NMR of compound **1m** (100 MHz, DMSO-d_6)

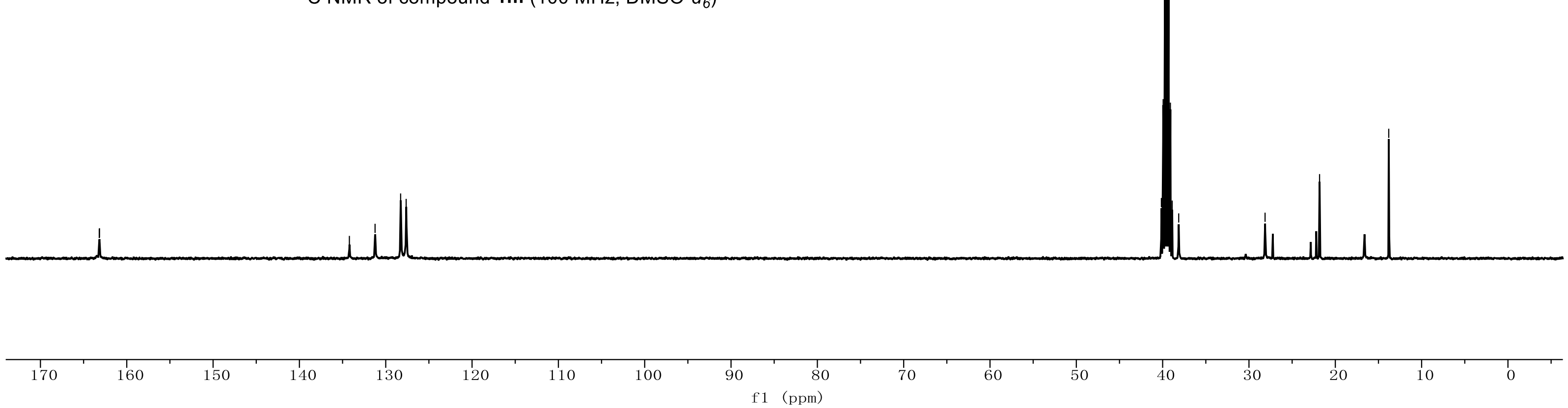

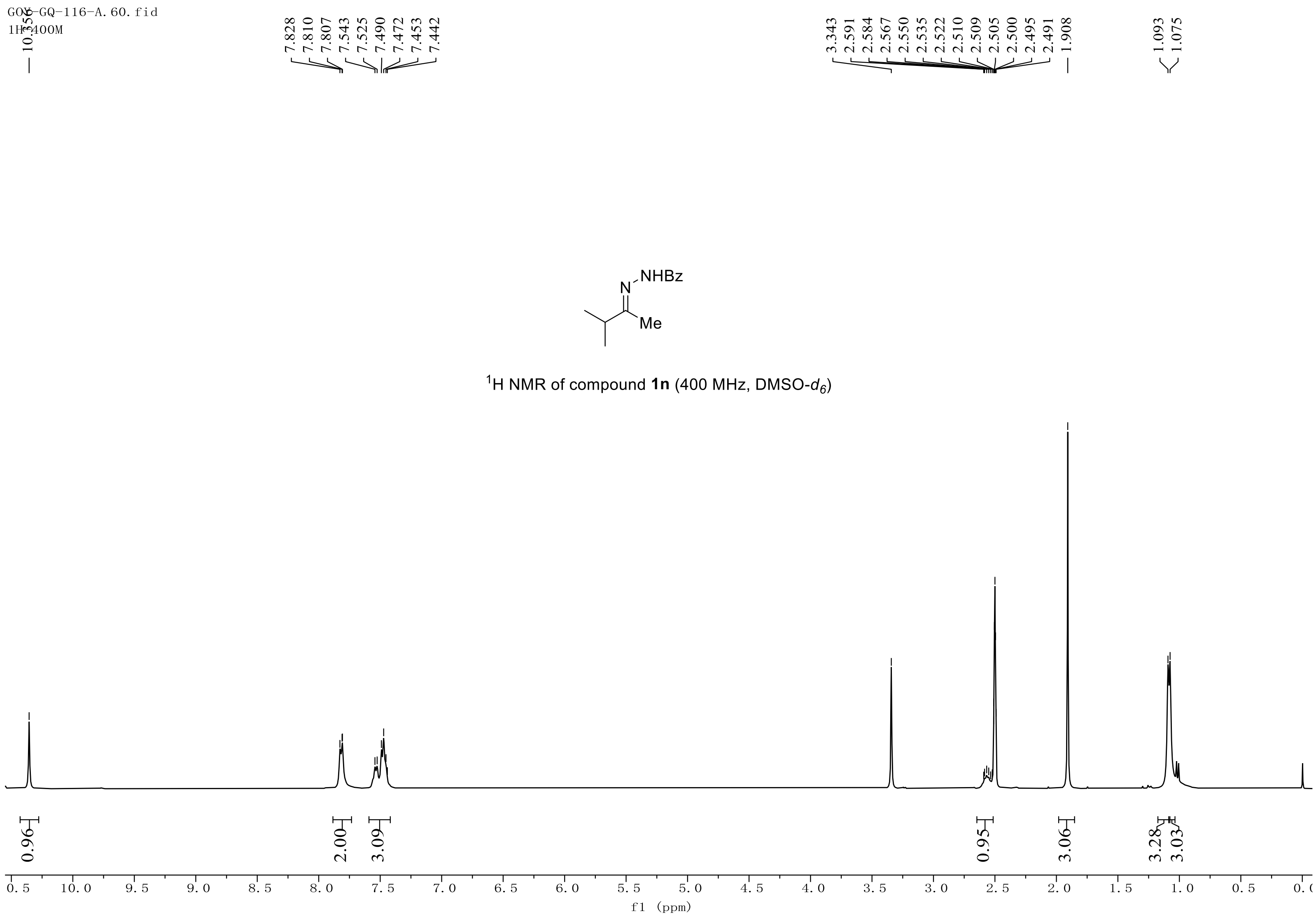

^{1}H NMR of compound **1n** (400 MHz, DMSO-d_6)

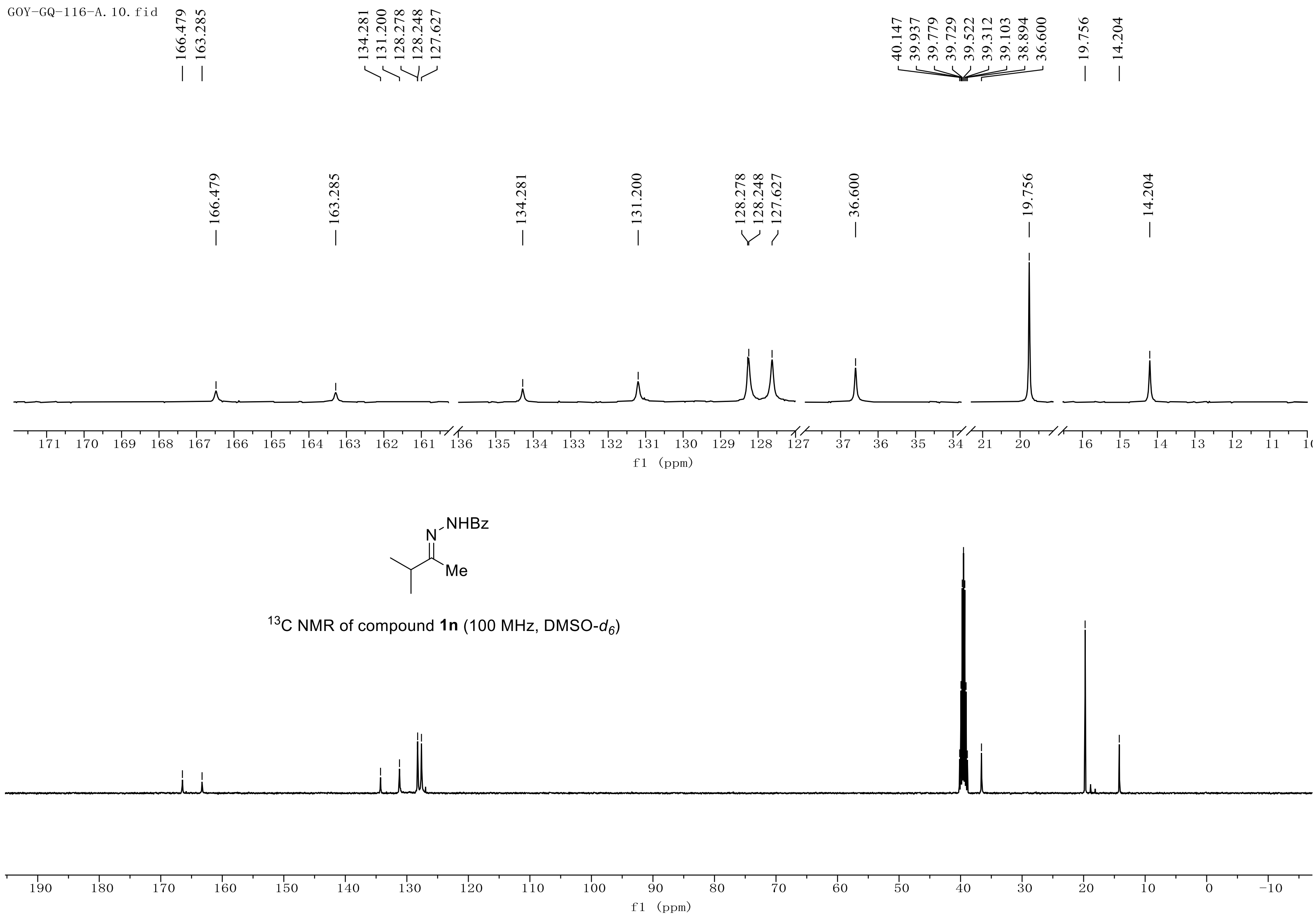

^{13}C NMR of compound **1n** (100 MHz, DMSO-d_6)

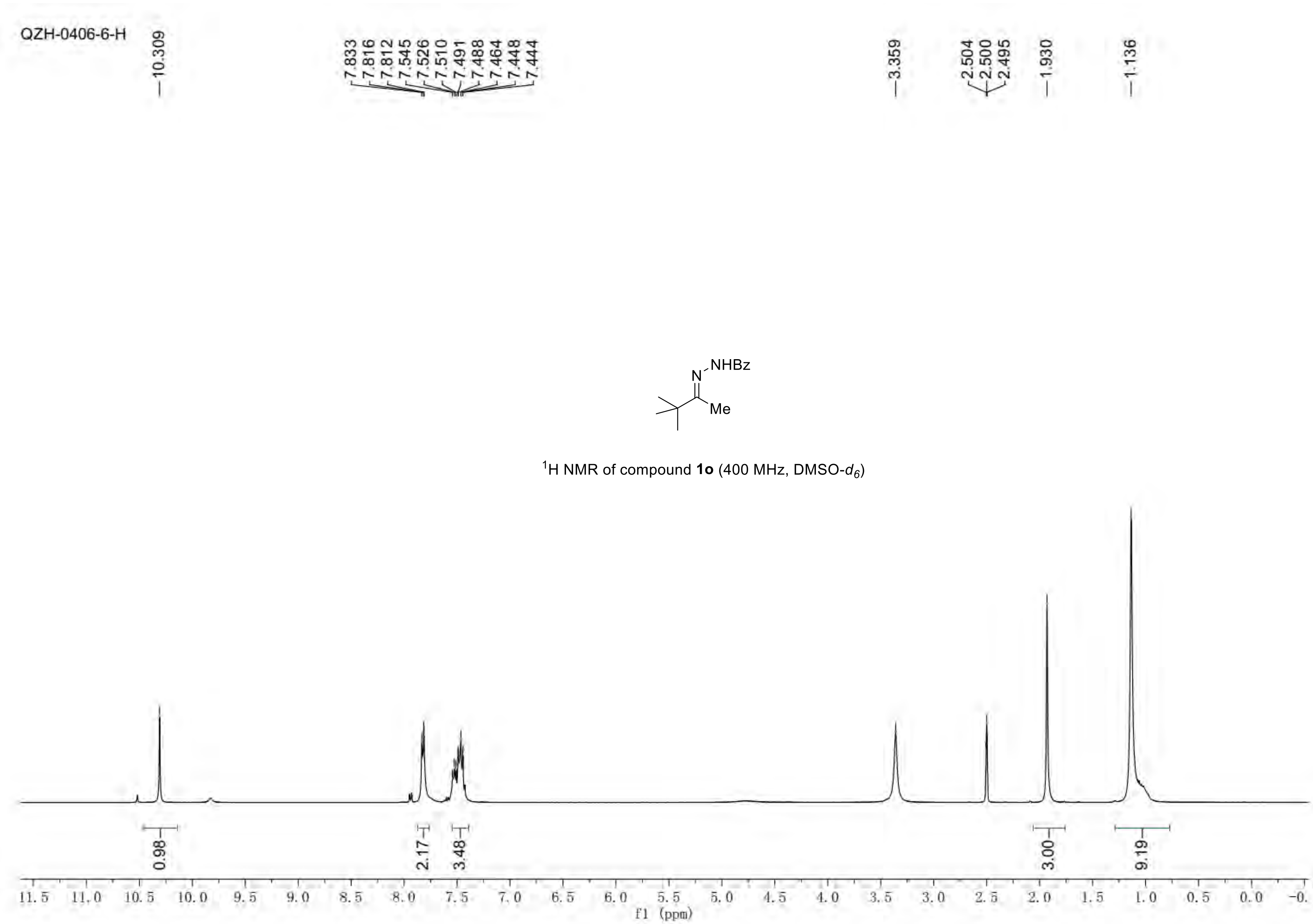

^{1}H NMR of compound **1o** (400 MHz, DMSO-d_6)

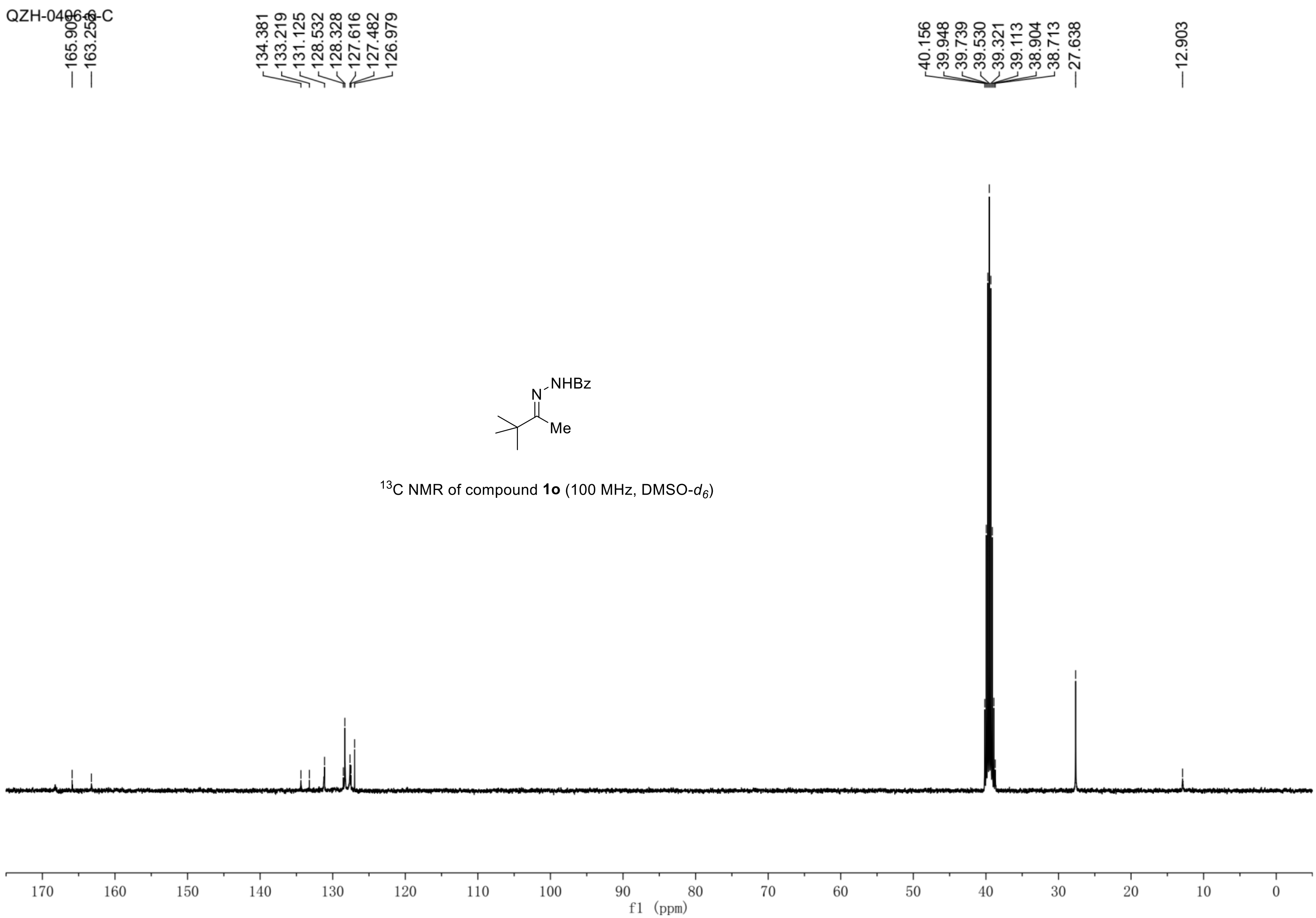

^{13}C NMR of compound **1o** (100 MHz, DMSO-d_6)

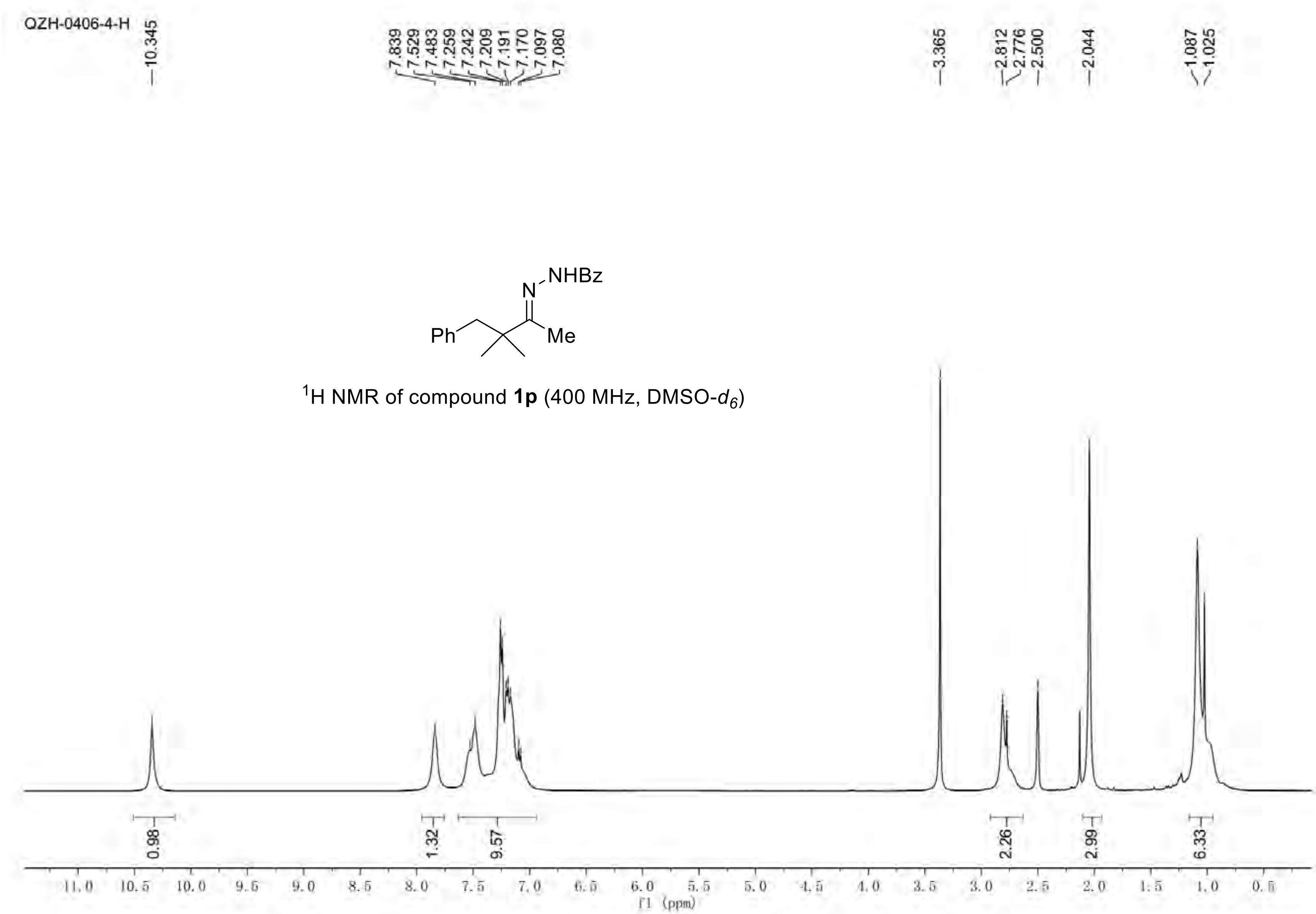

^{1}H NMR of compound **1p** (400 MHz, DMSO-d_6)

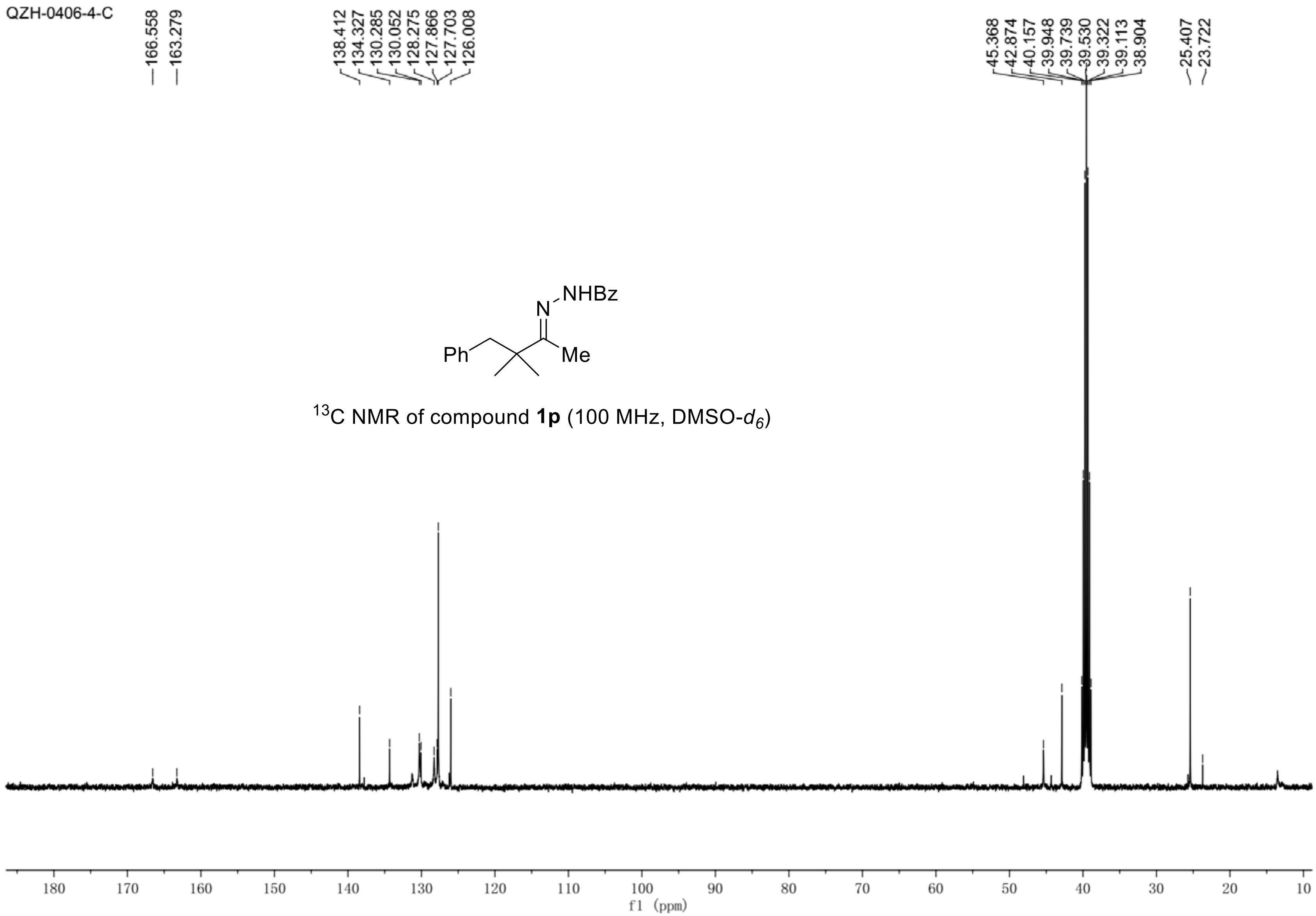

^{13}C NMR of compound **1p** (100 MHz, DMSO-d_6)

LXJ-LD-10-1-H

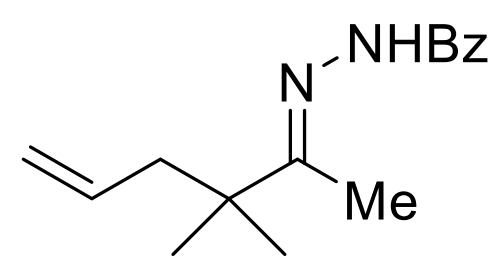

^{1}H NMR of compound **1q** (400 MHz, DMSO-d_6)

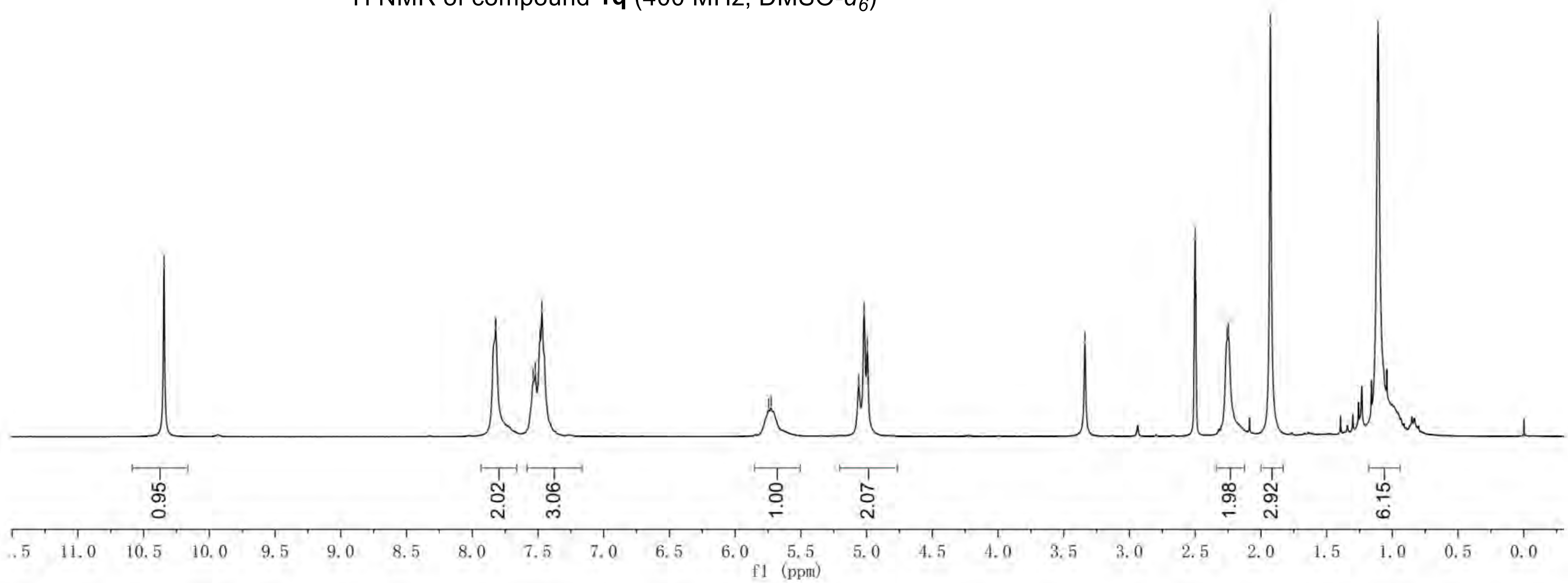

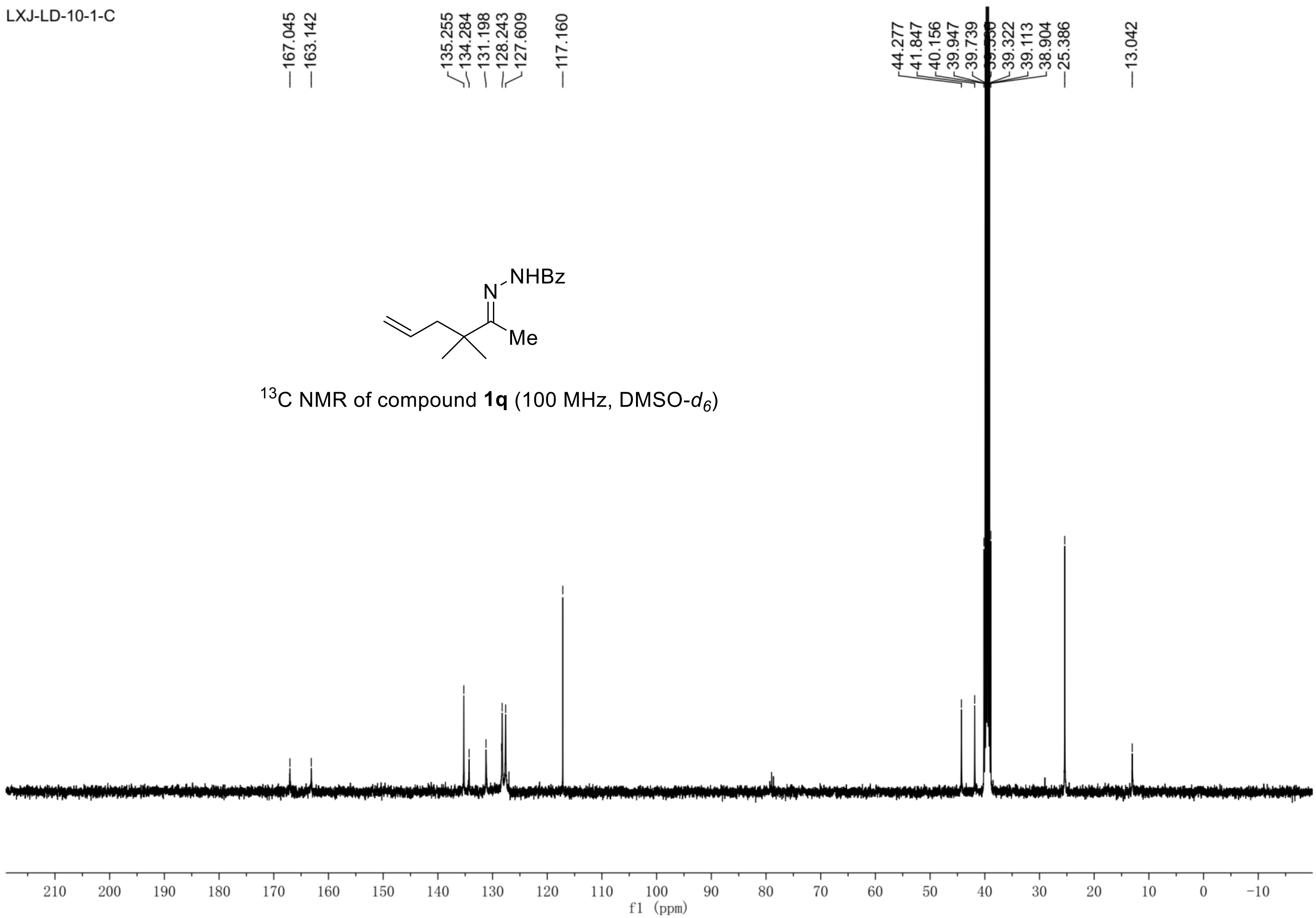

^{13}C NMR of compound **1q** (100 MHz, DMSO-d_6)

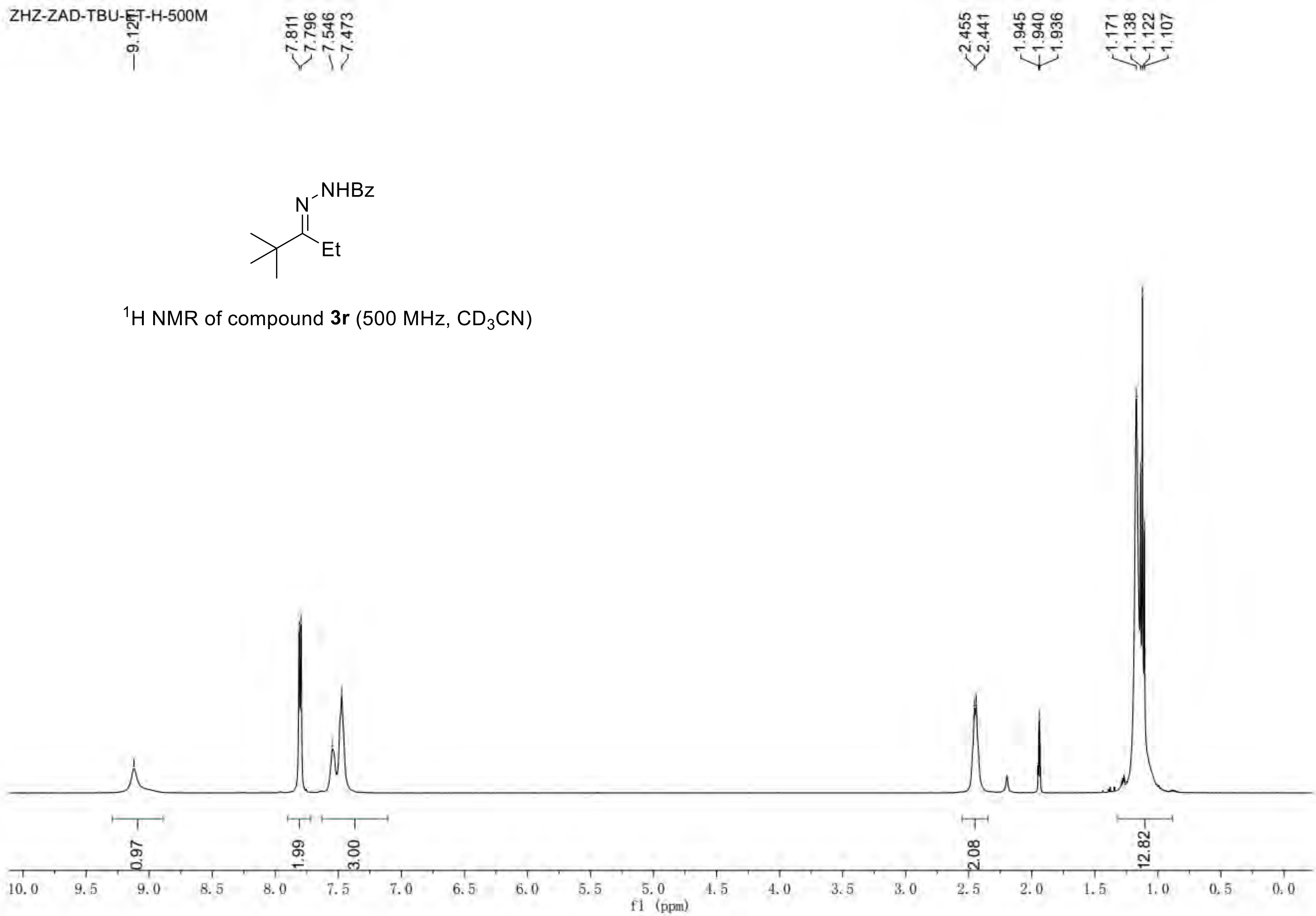

^{1}H NMR of compound **3r** (500 MHz, CD_3CN)

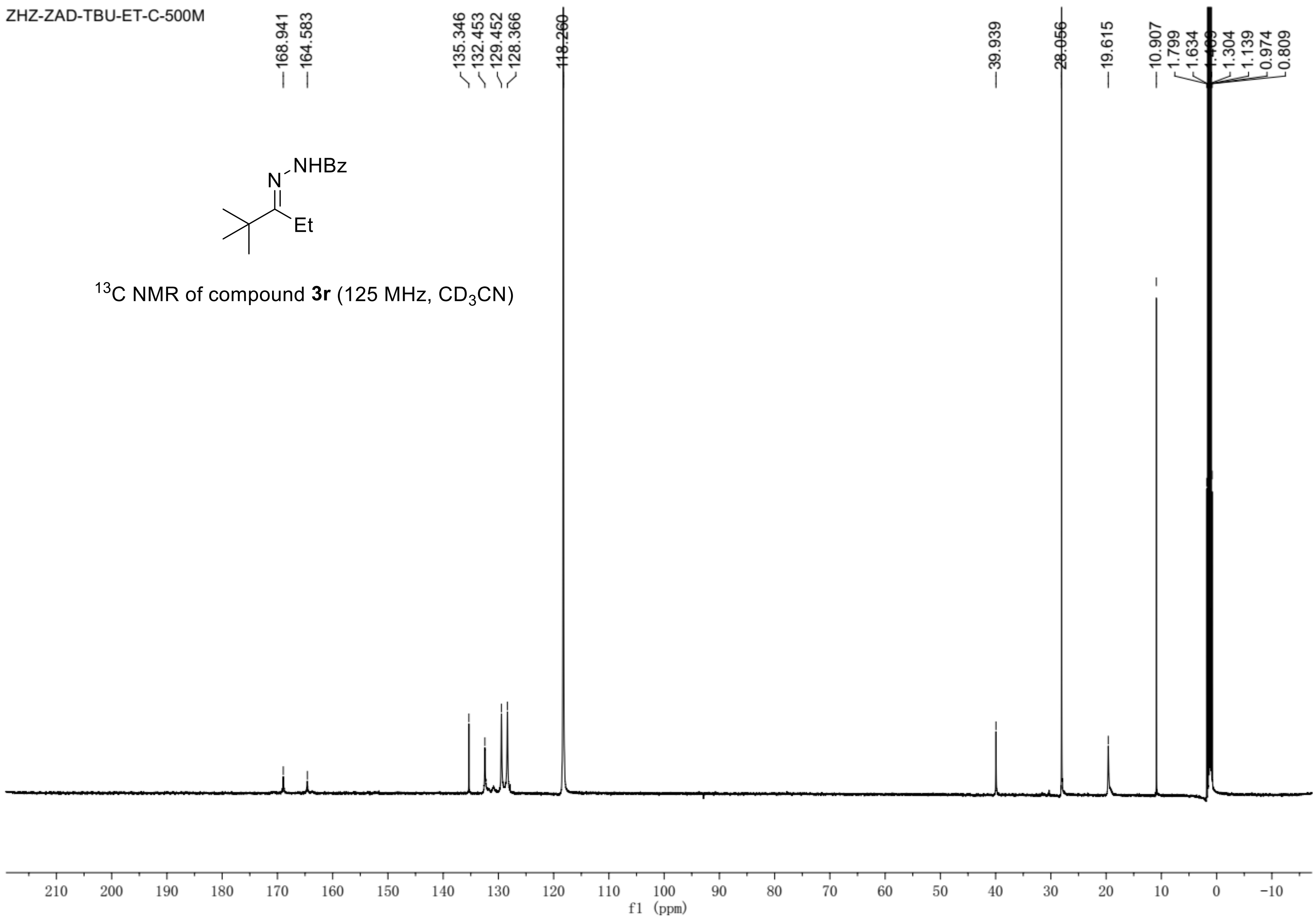

^{13}C NMR of compound **3r** (125 MHz, CD_3CN)

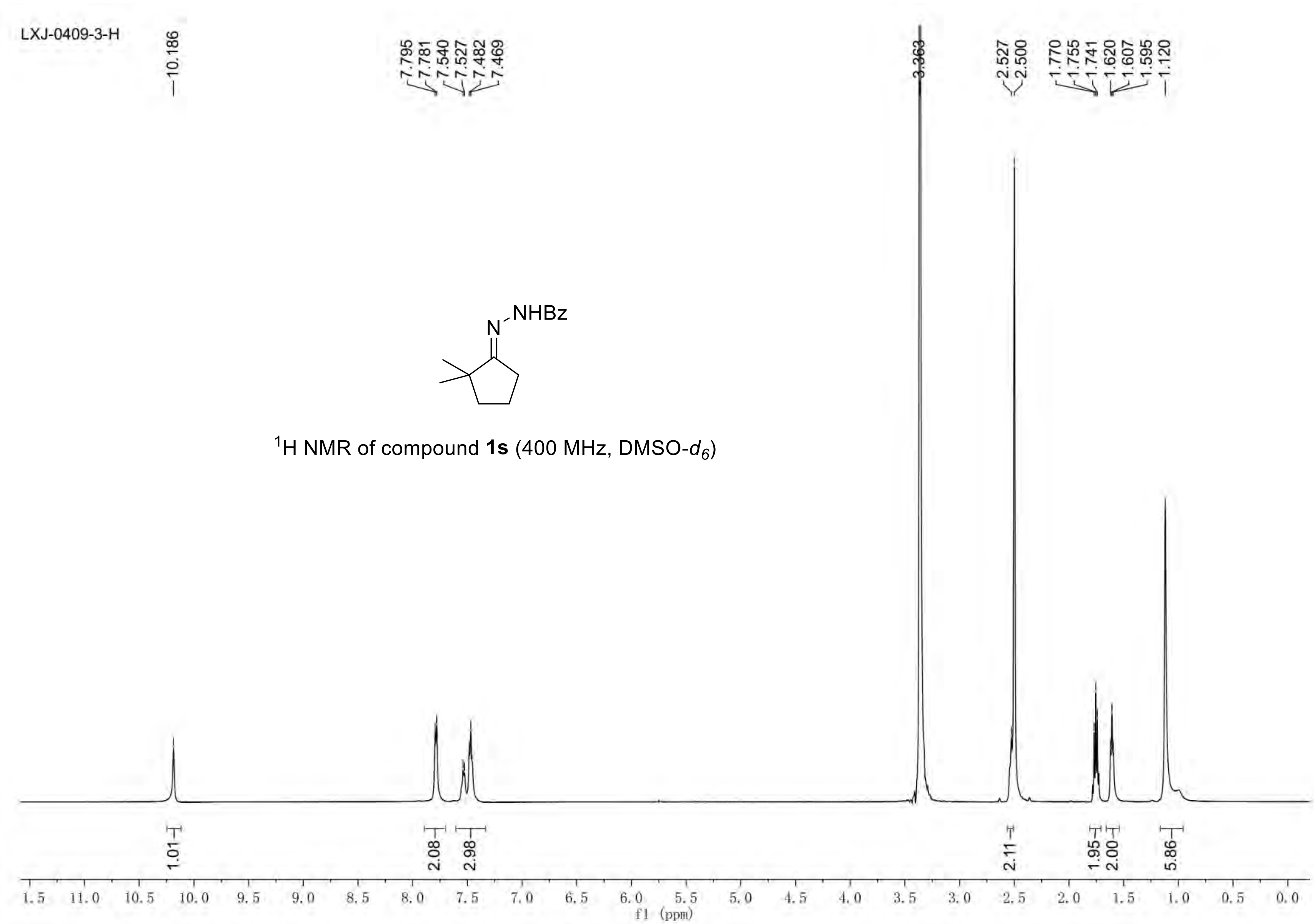

^{1}H NMR of compound **1s** (400 MHz, DMSO-d_6)

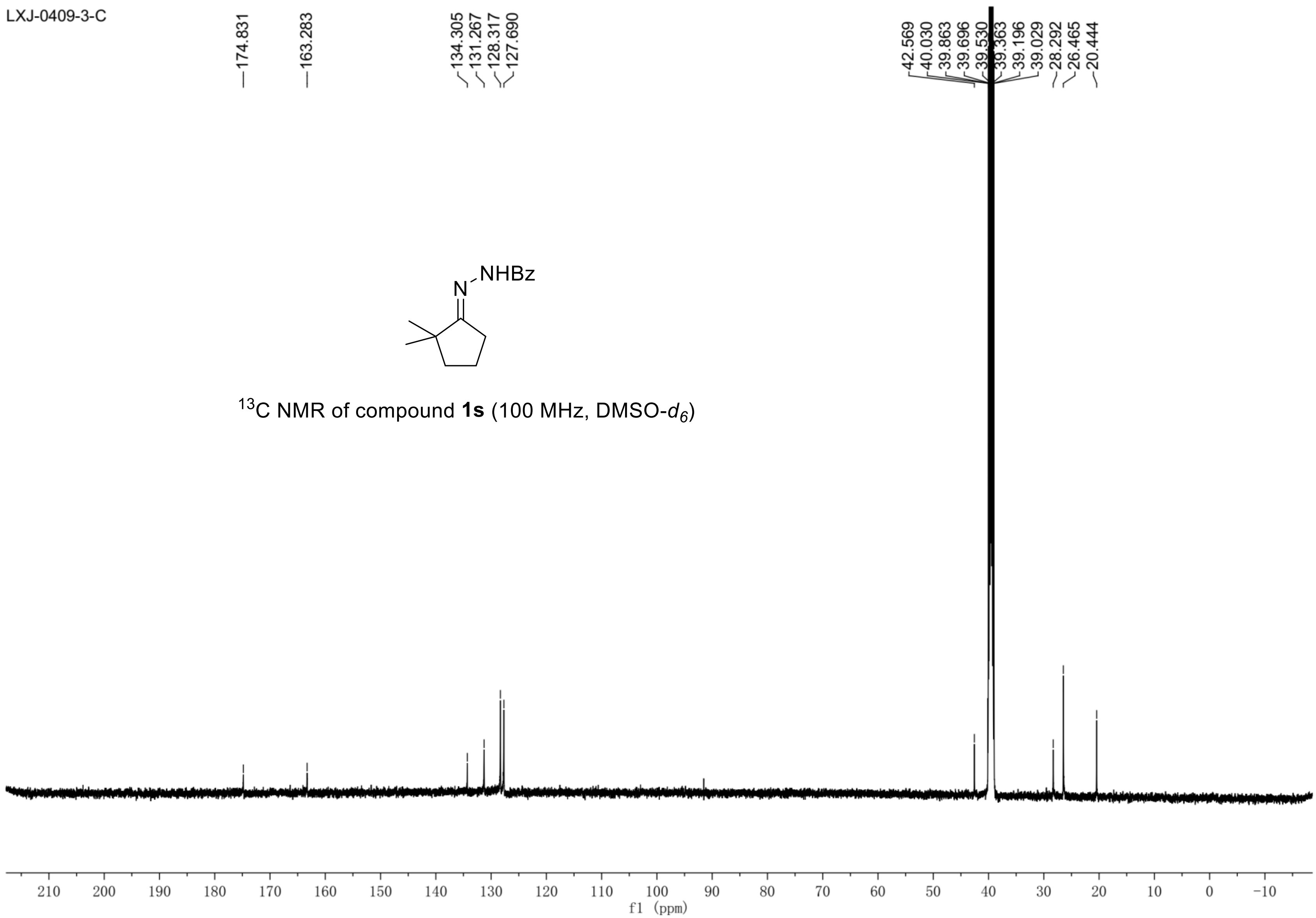

^{13}C NMR of compound **1s** (100 MHz, DMSO-d_6)

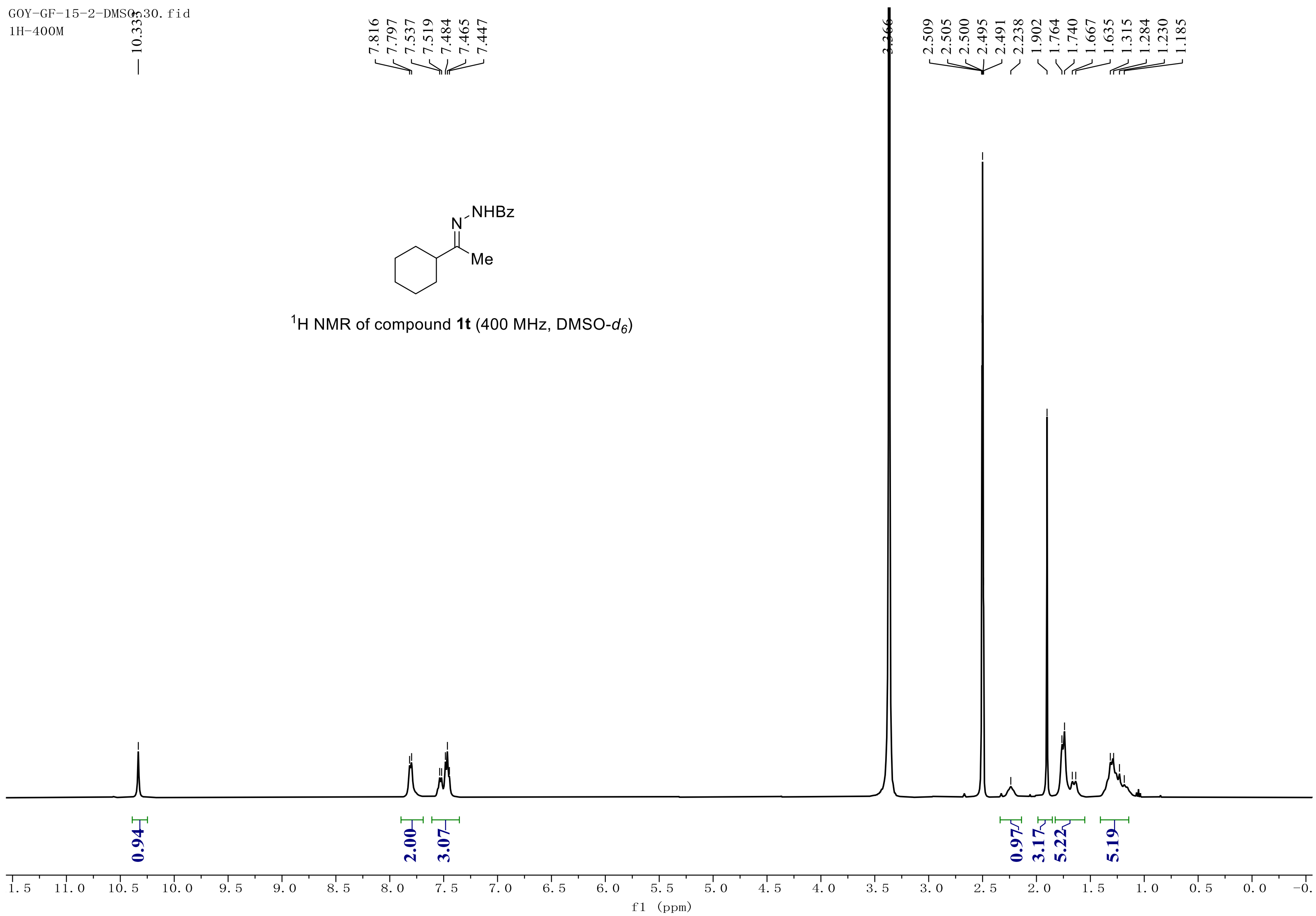

^{1}H NMR of compound **1t** (400 MHz, DMSO-d_6)

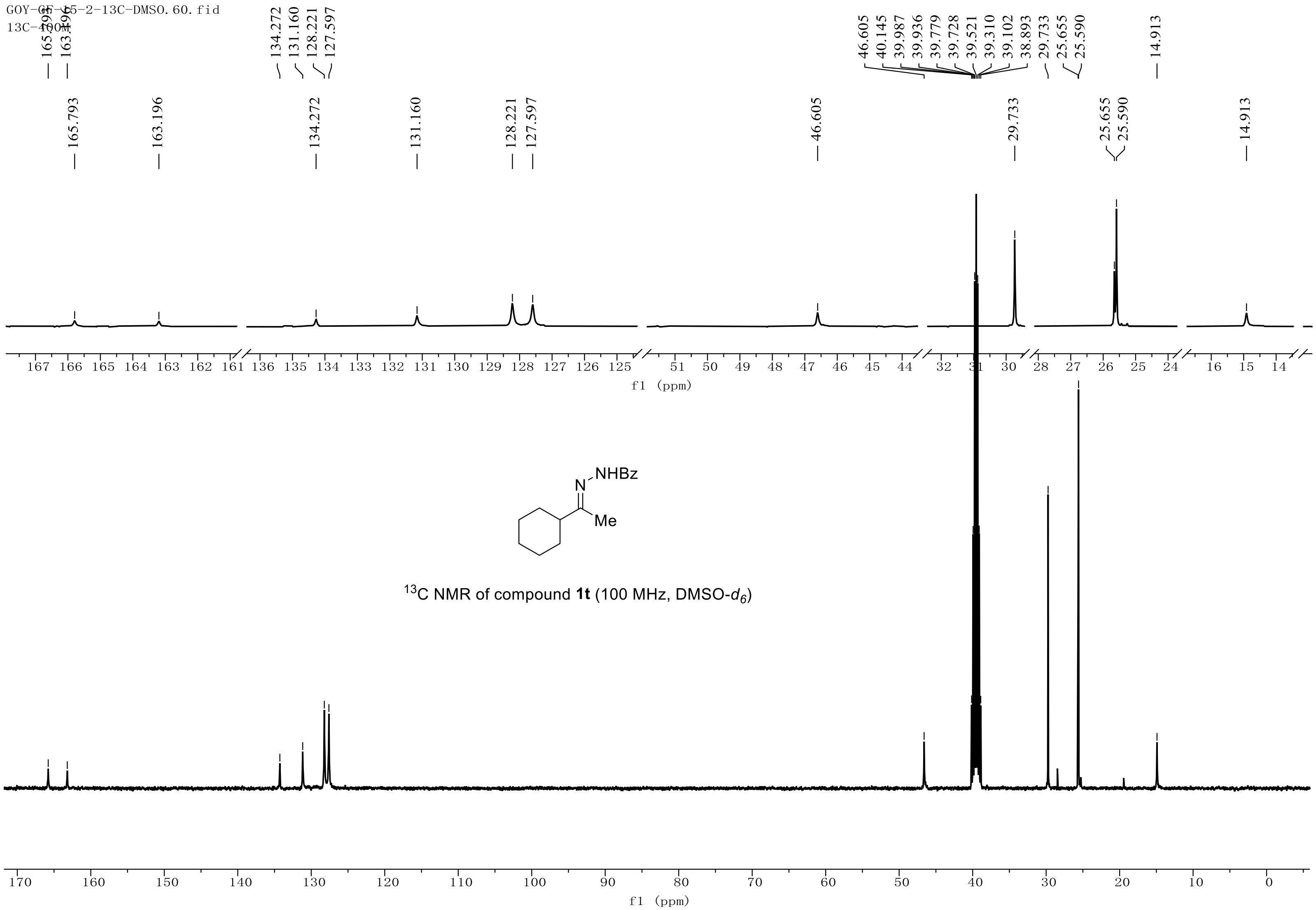

^{13}C NMR of compound **1t** (100 MHz, DMSO-d_6)

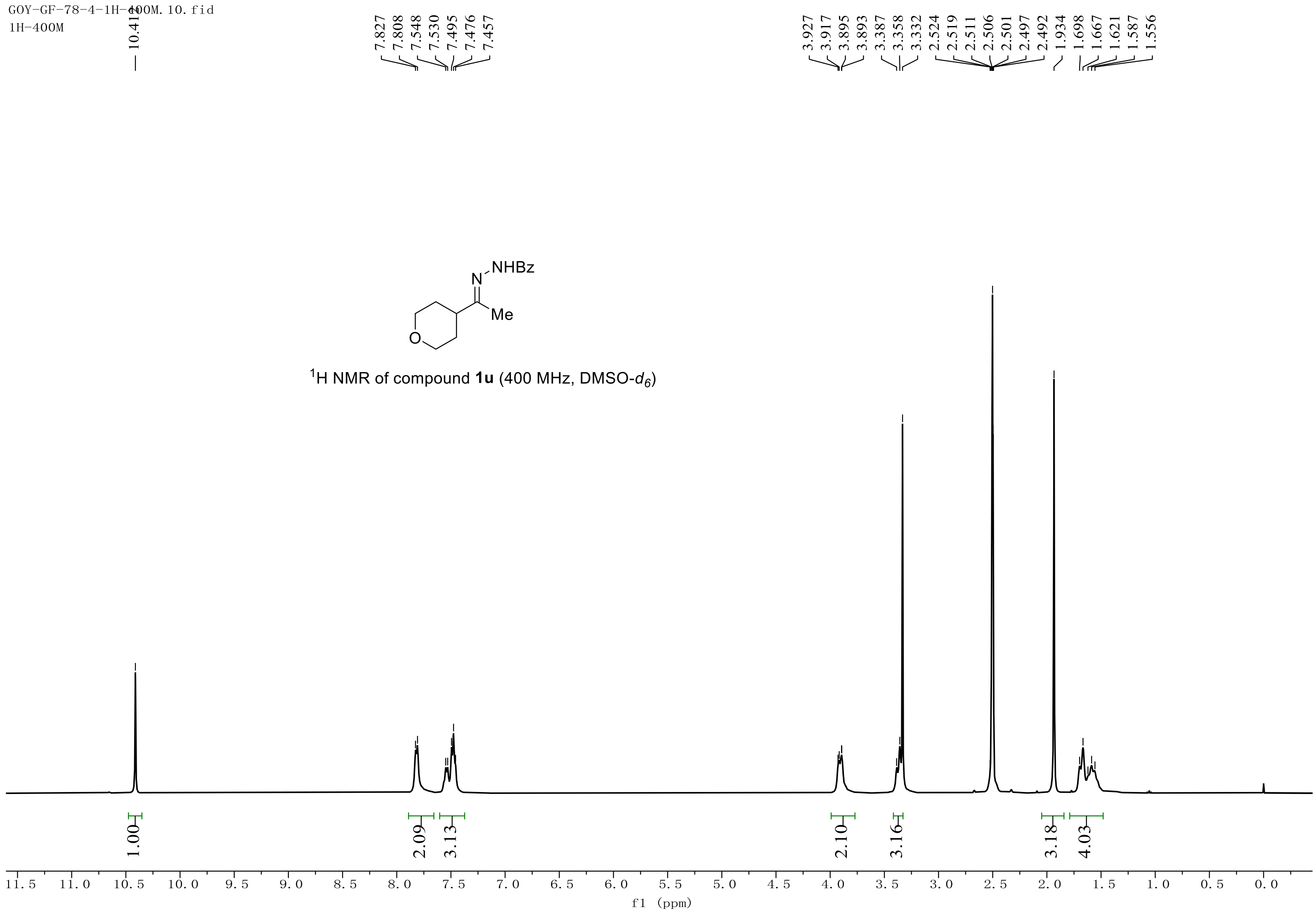

^{1}H NMR of compound **1u** (400 MHz, DMSO-d_6)

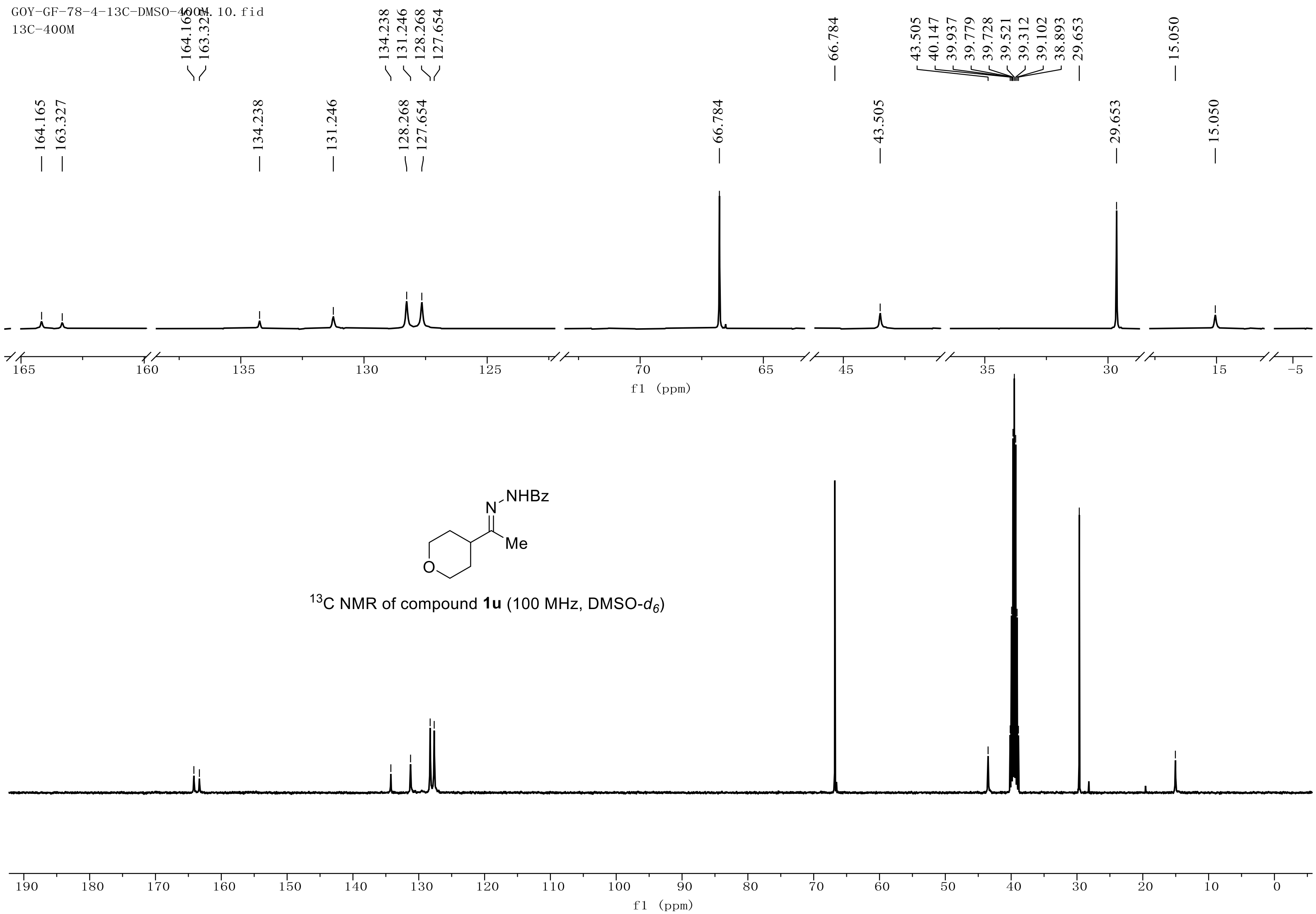

^{13}C NMR of compound **1u** (100 MHz, DMSO-d_6)

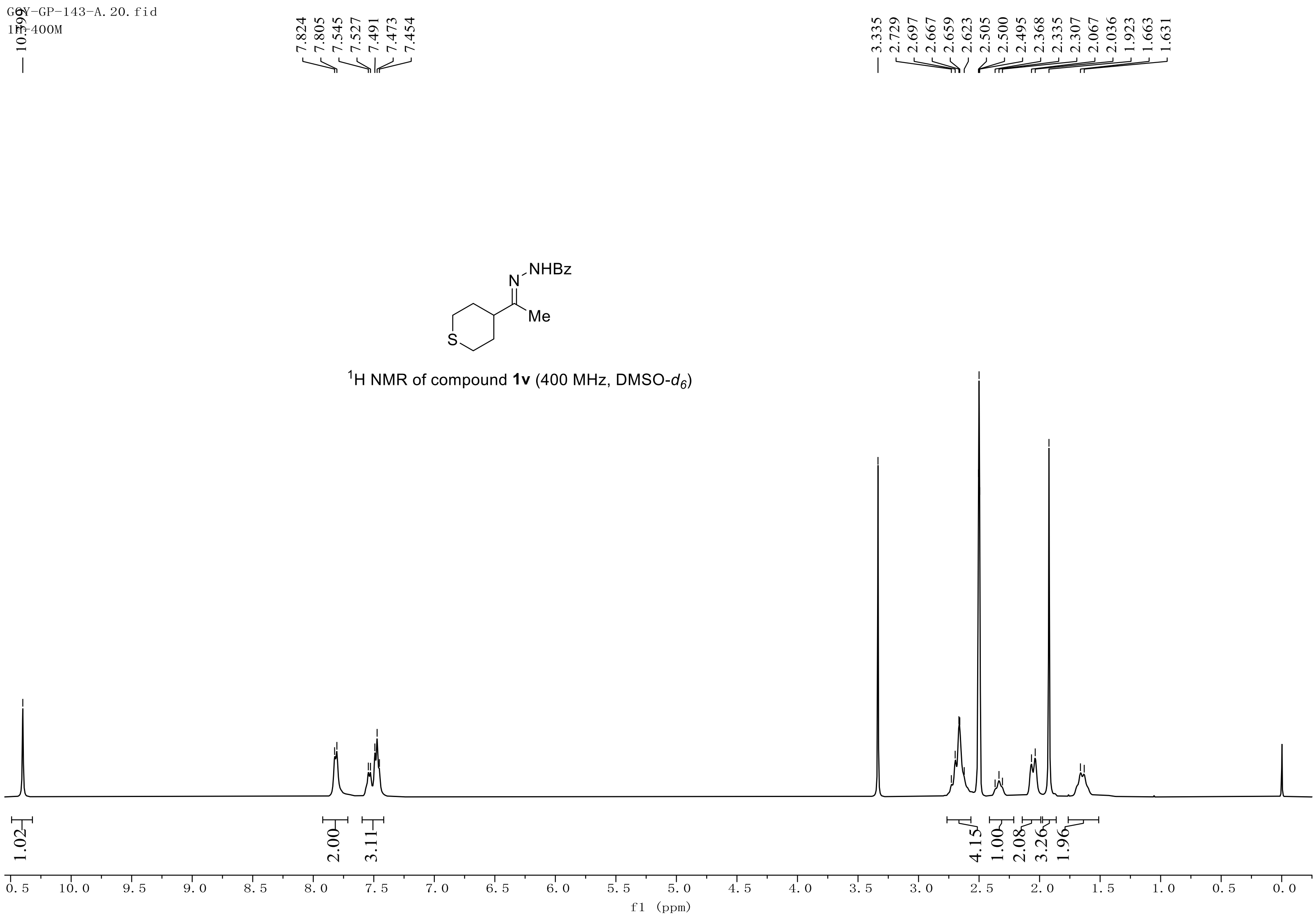

^{1}H NMR of compound **1v** (400 MHz, DMSO-d_6)

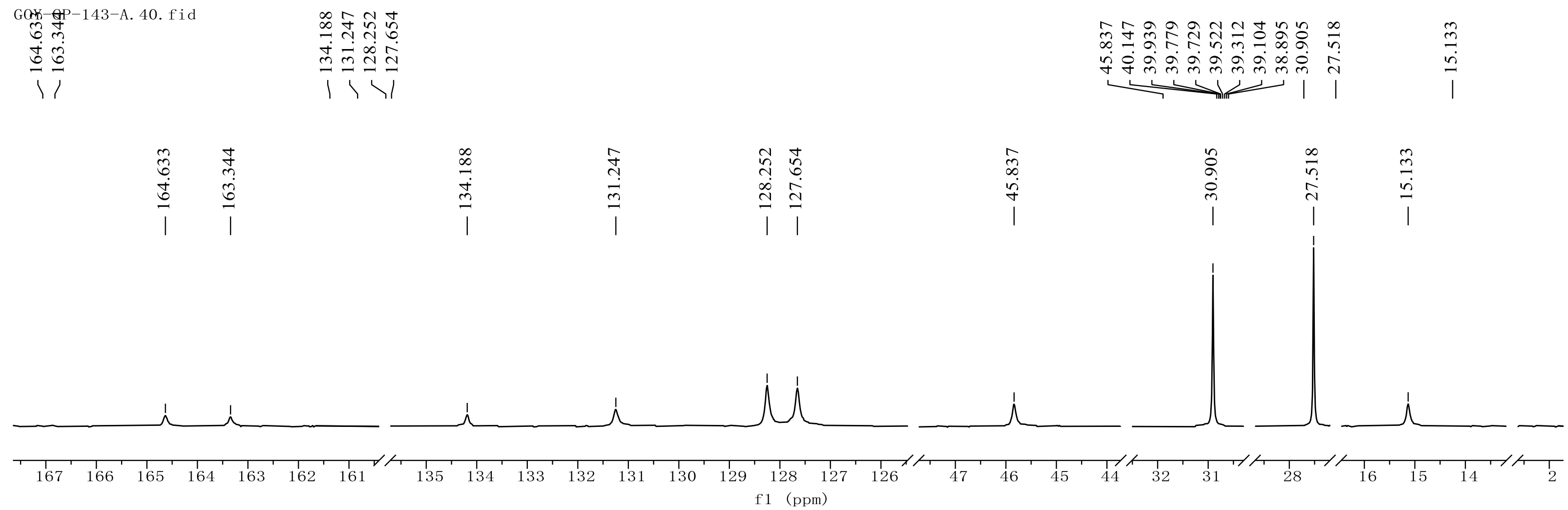

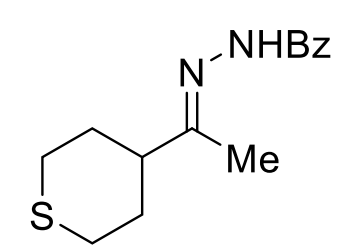

^{13}C NMR of compound **1v** (100 MHz, DMSO-d_6)

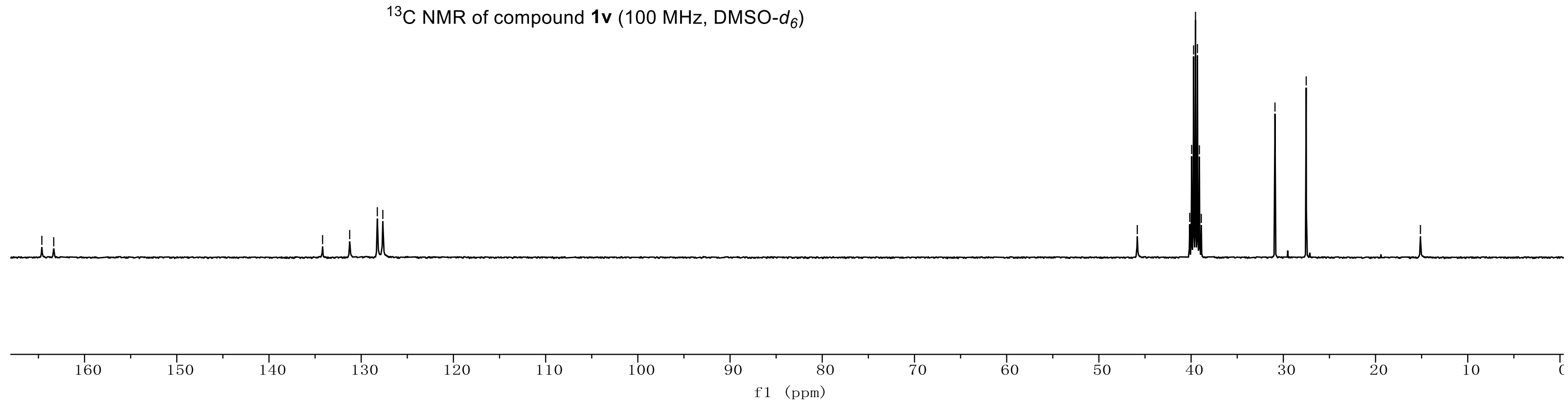

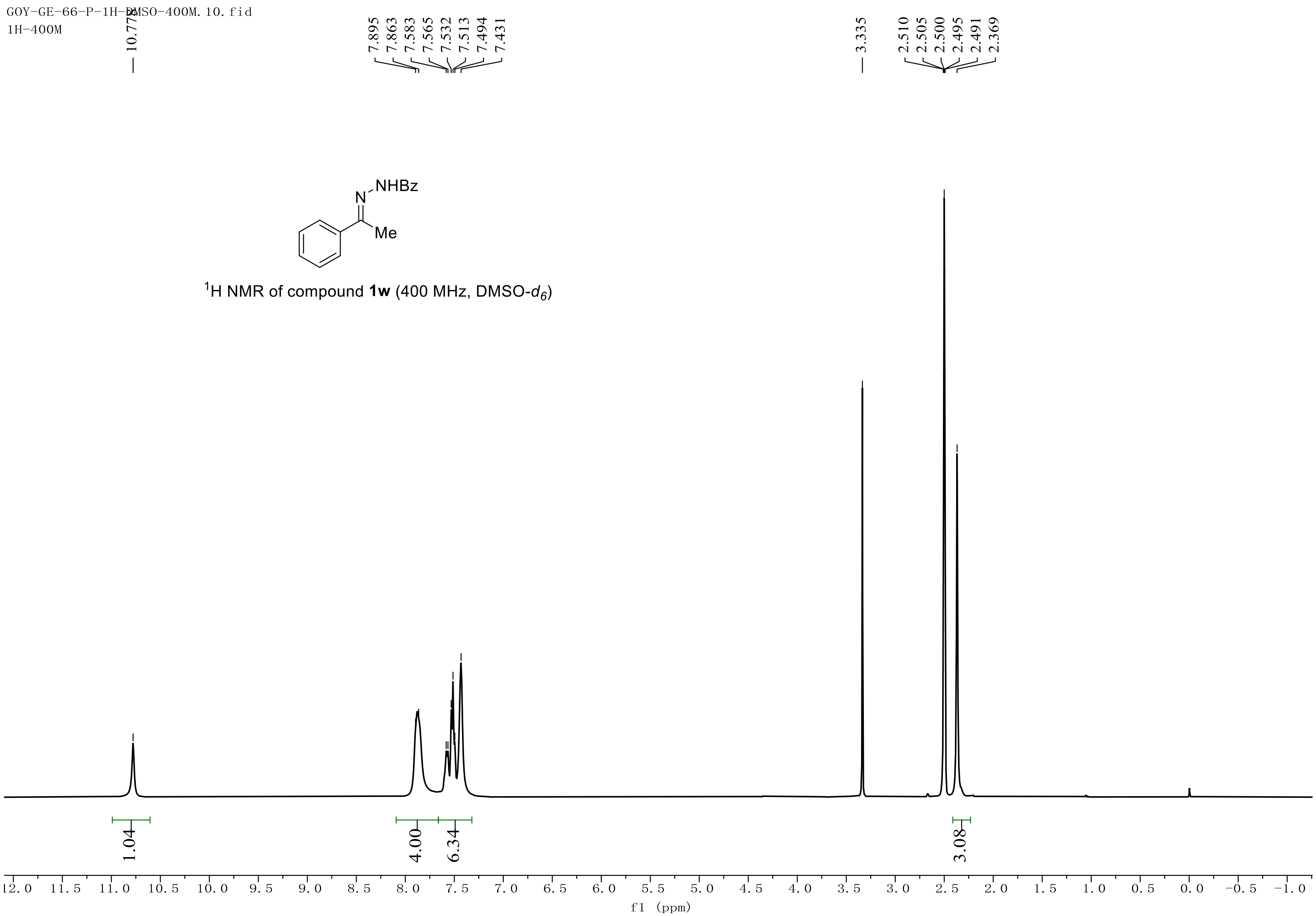

^{1}H NMR of compound **1w** (400 MHz, DMSO-d_6)

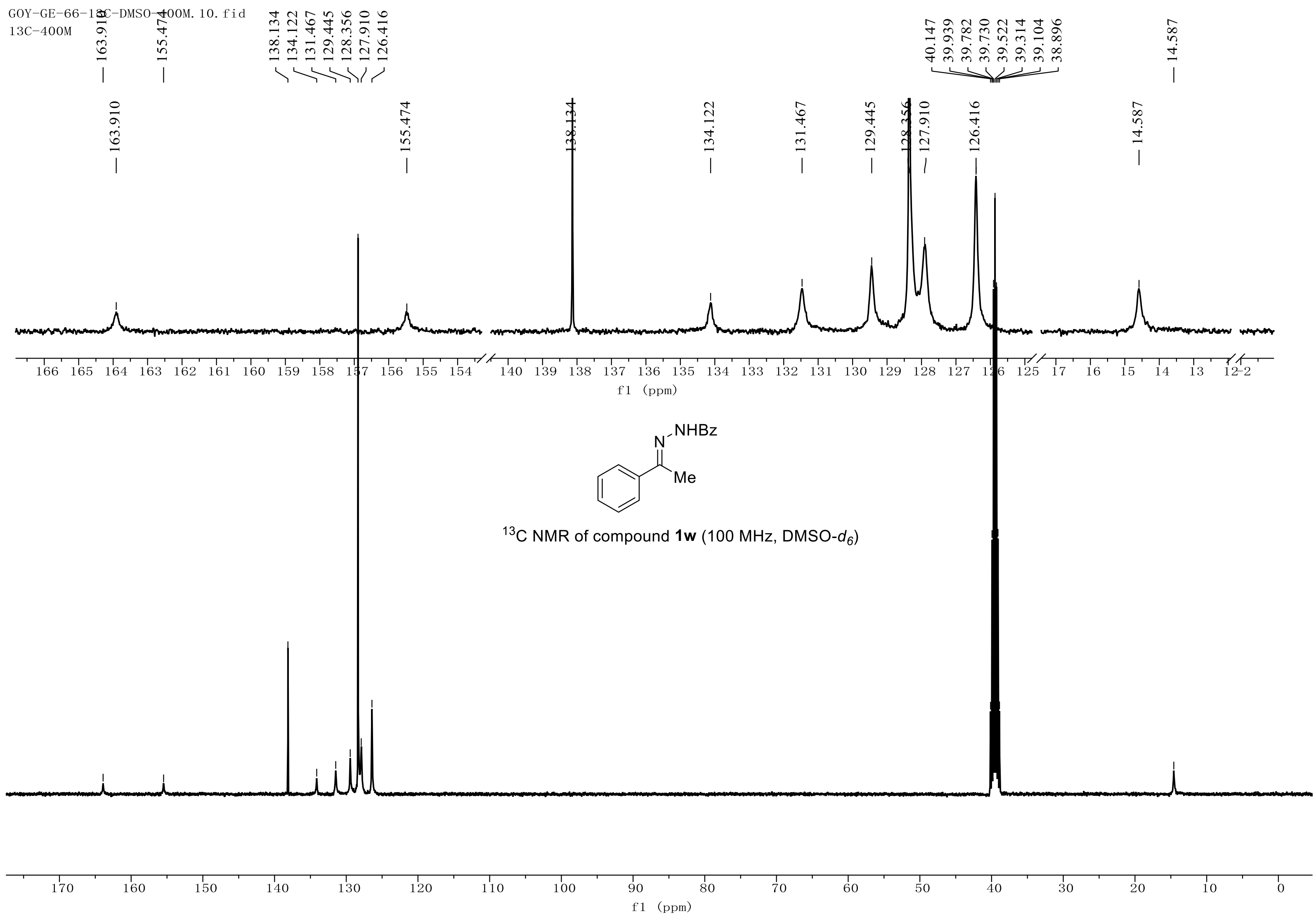

^{13}C NMR of compound **1w** (100 MHz, DMSO-d_6)

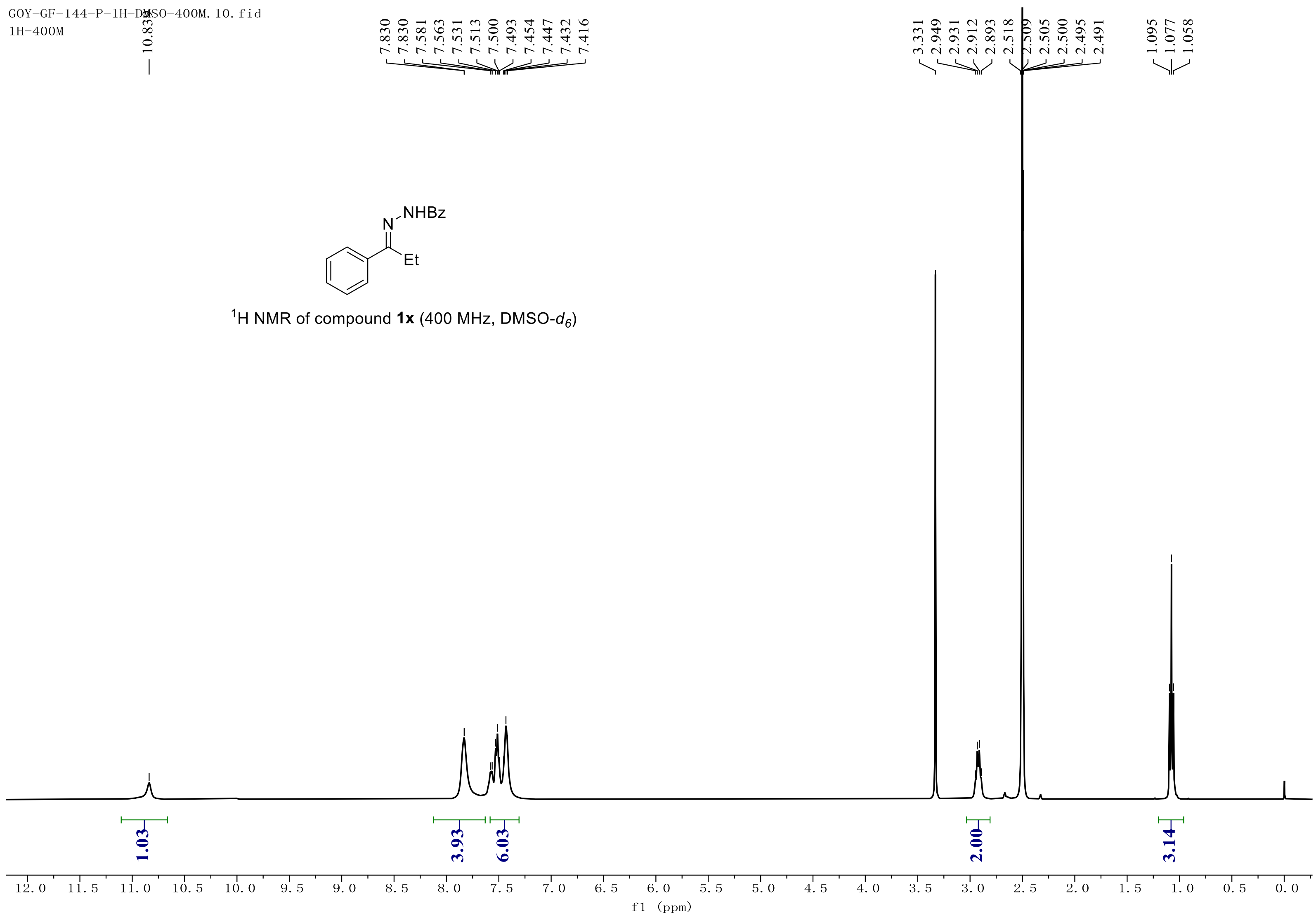

^{1}H NMR of compound **1x** (400 MHz, DMSO-d_6)

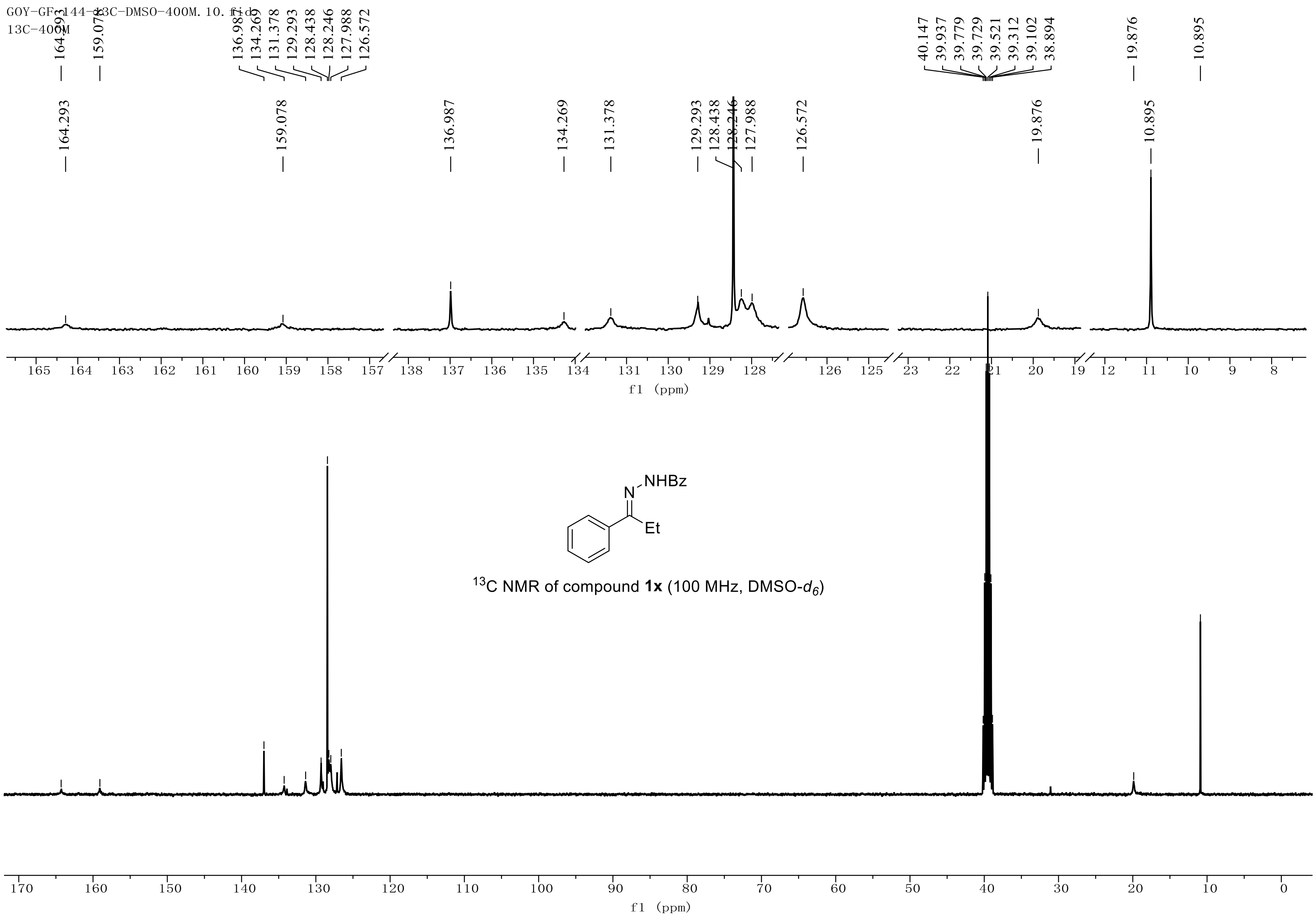

^{13}C NMR of compound **1x** (100 MHz, DMSO-d_6)

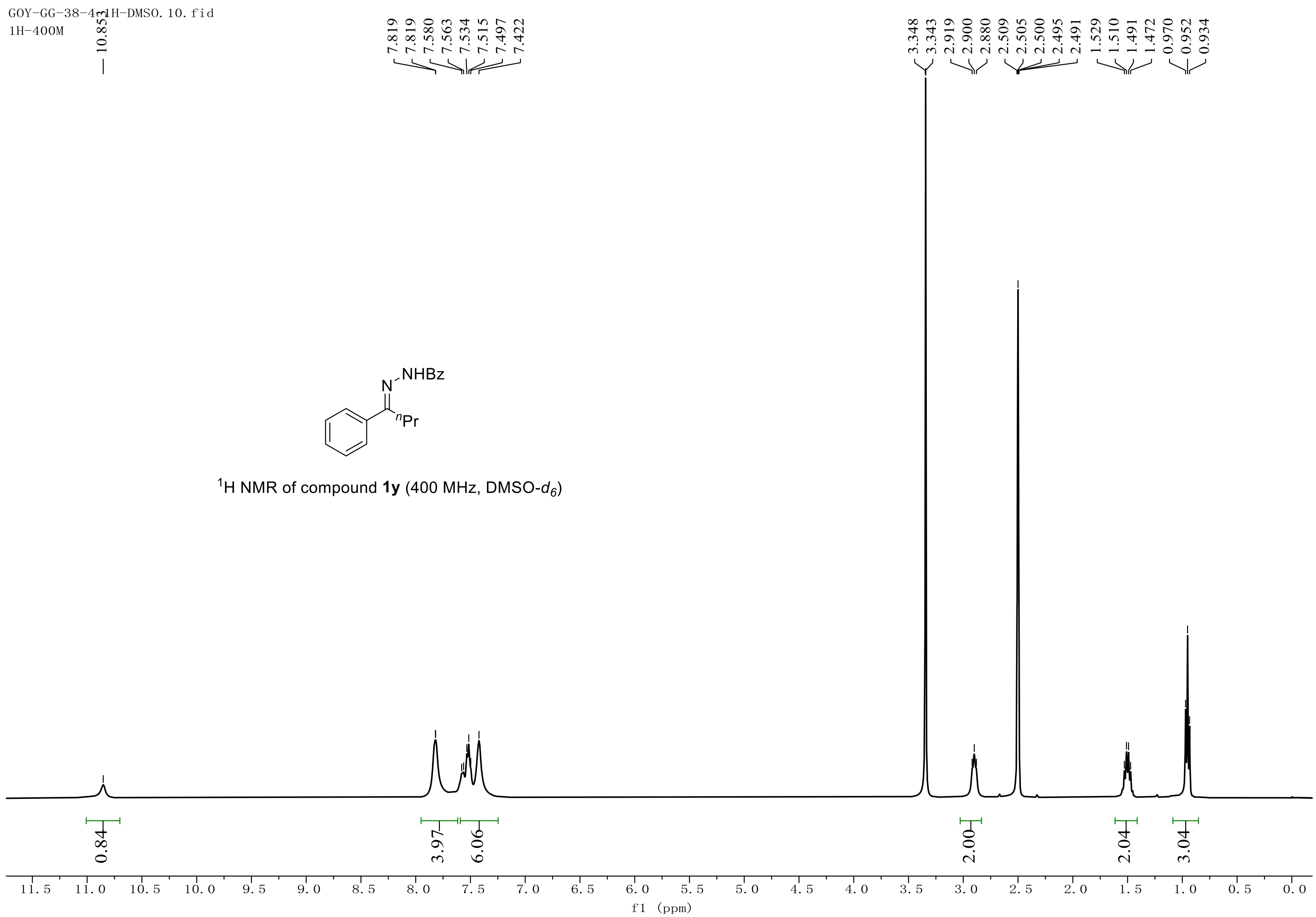

^{1}H NMR of compound **1y** (400 MHz, DMSO-d_6)

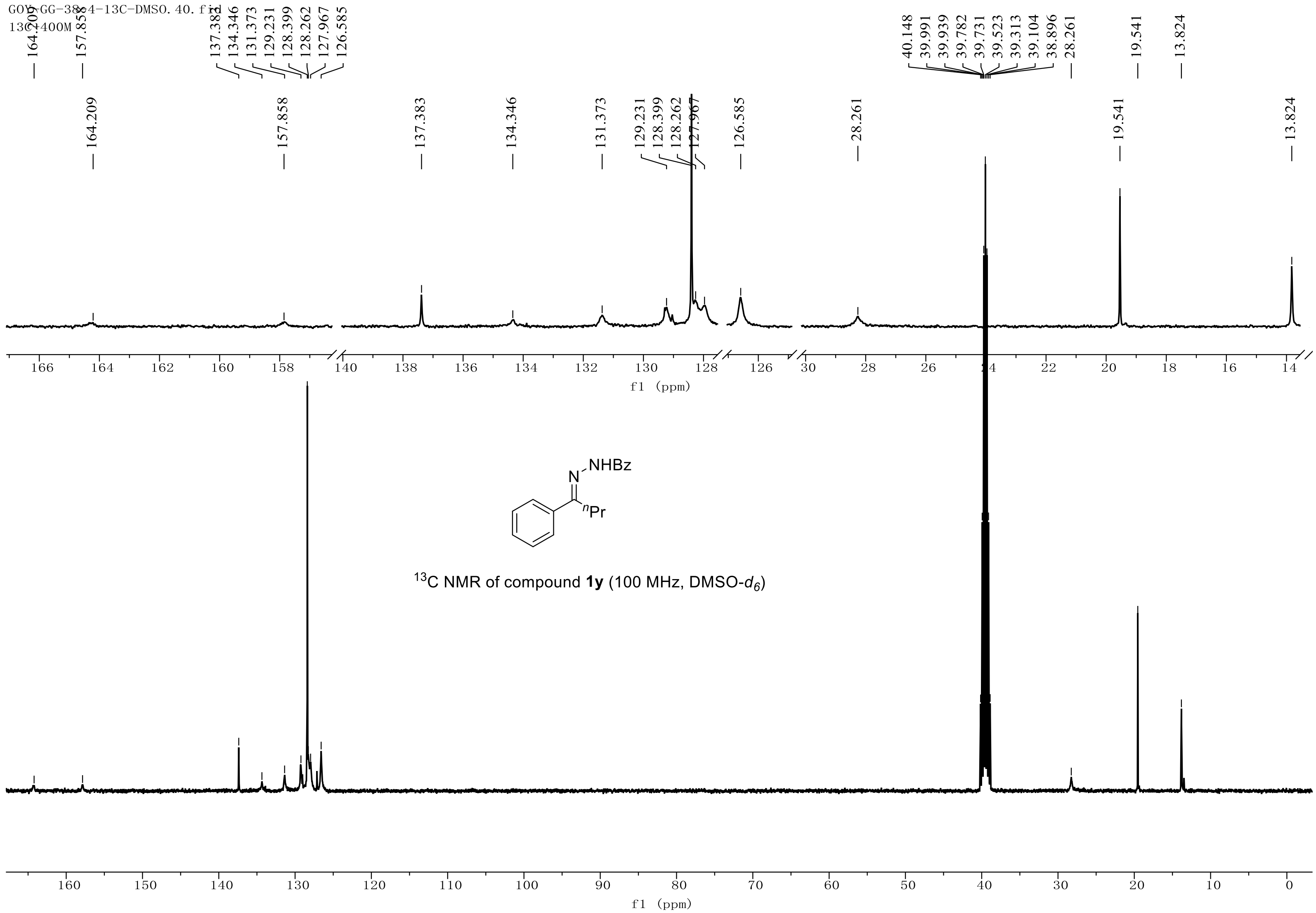

^{13}C NMR of compound **1y** (100 MHz, DMSO-d_6)

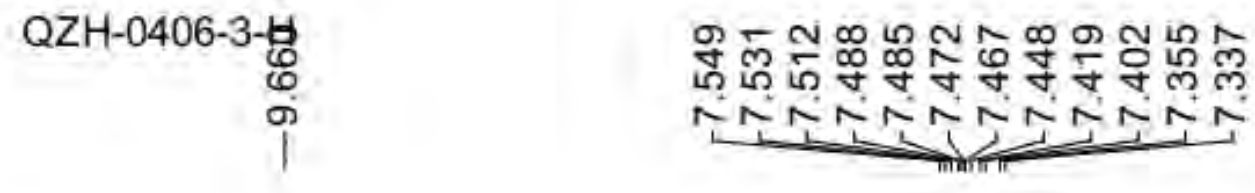

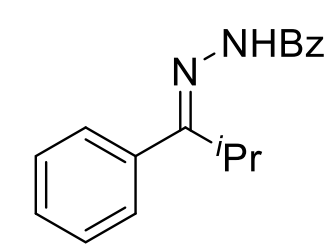

^{1}H NMR of compound **1z** (400 MHz, DMSO-d_6)

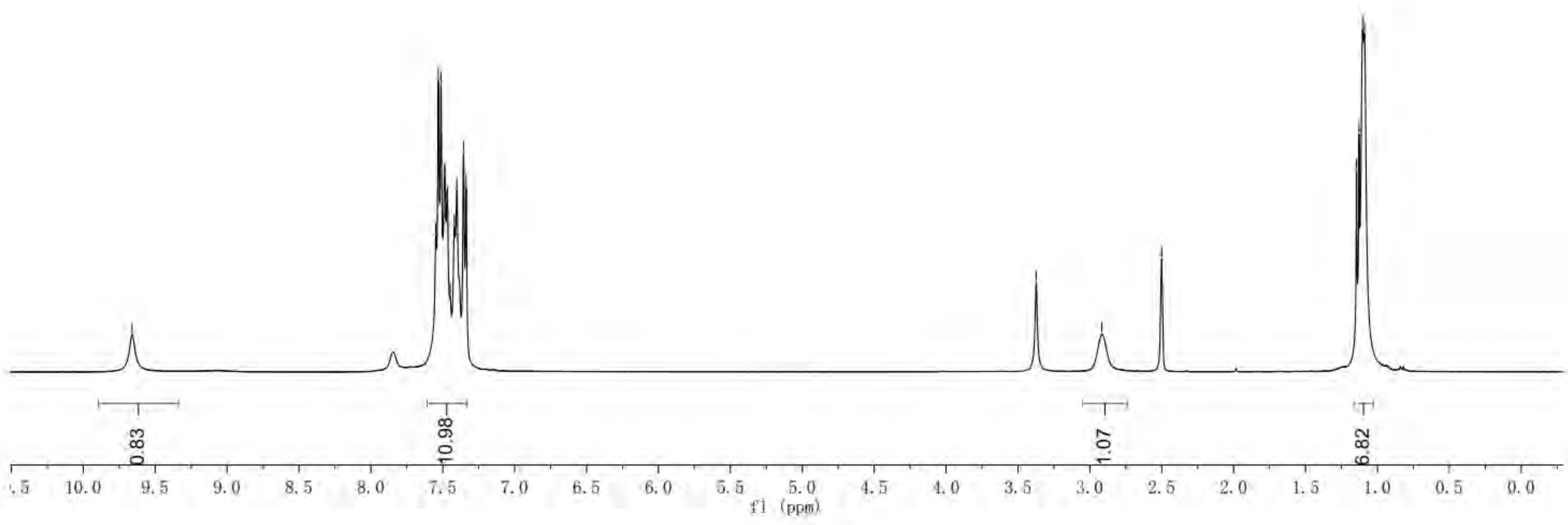

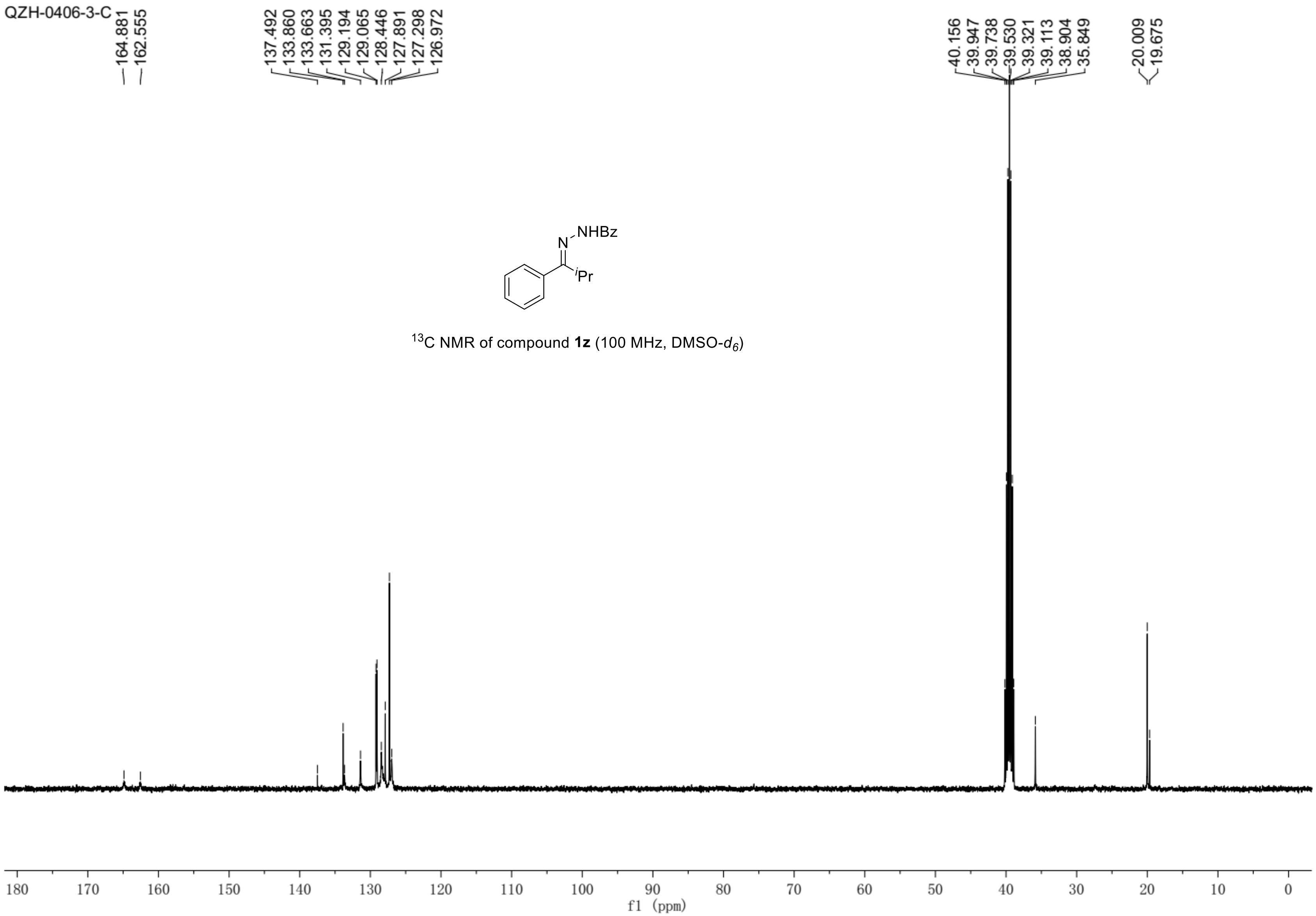

^{13}C NMR of compound **1z** (100 MHz, DMSO-d_6)

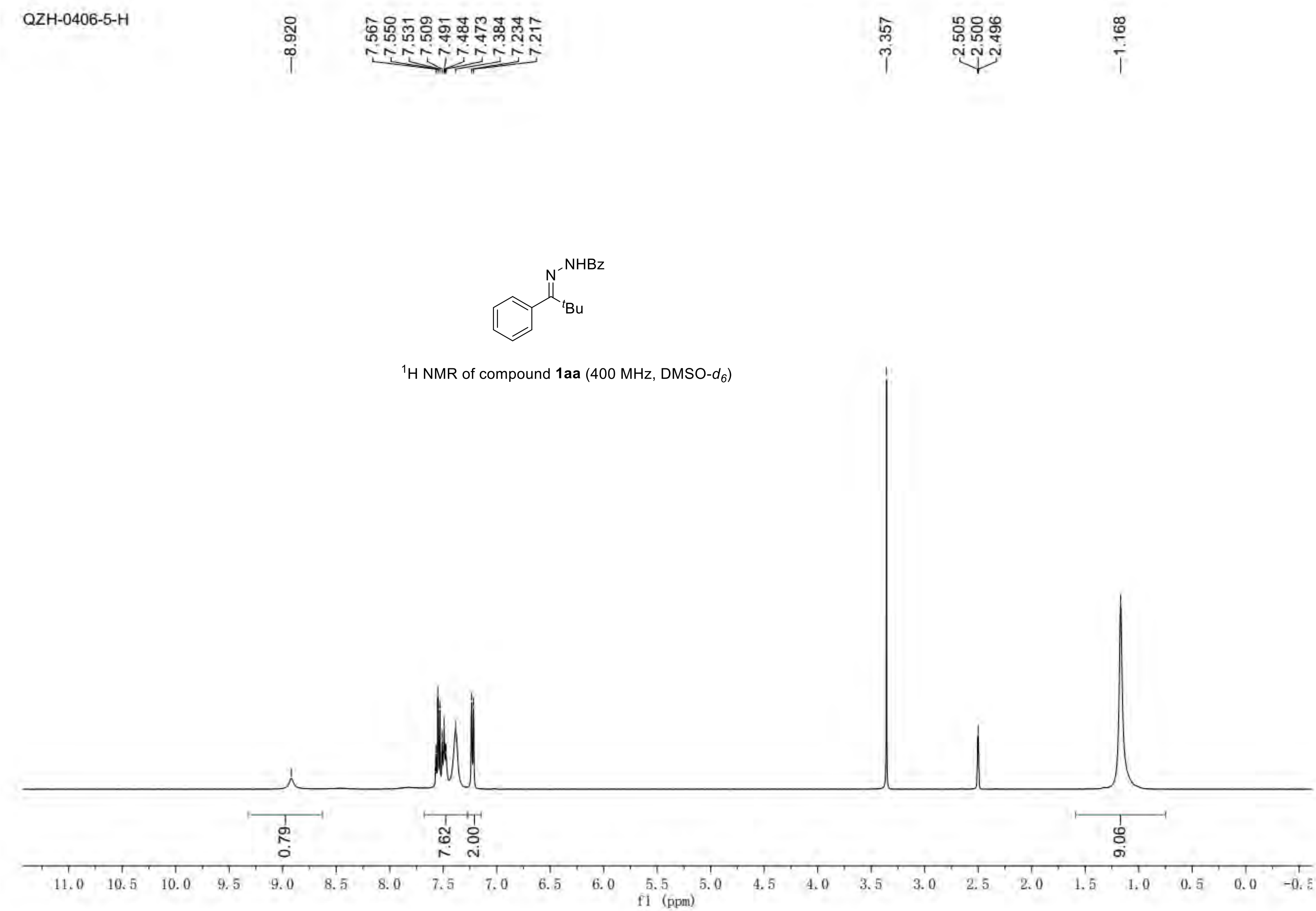

^{1}H NMR of compound **1aa** (400 MHz, DMSO-d_6)

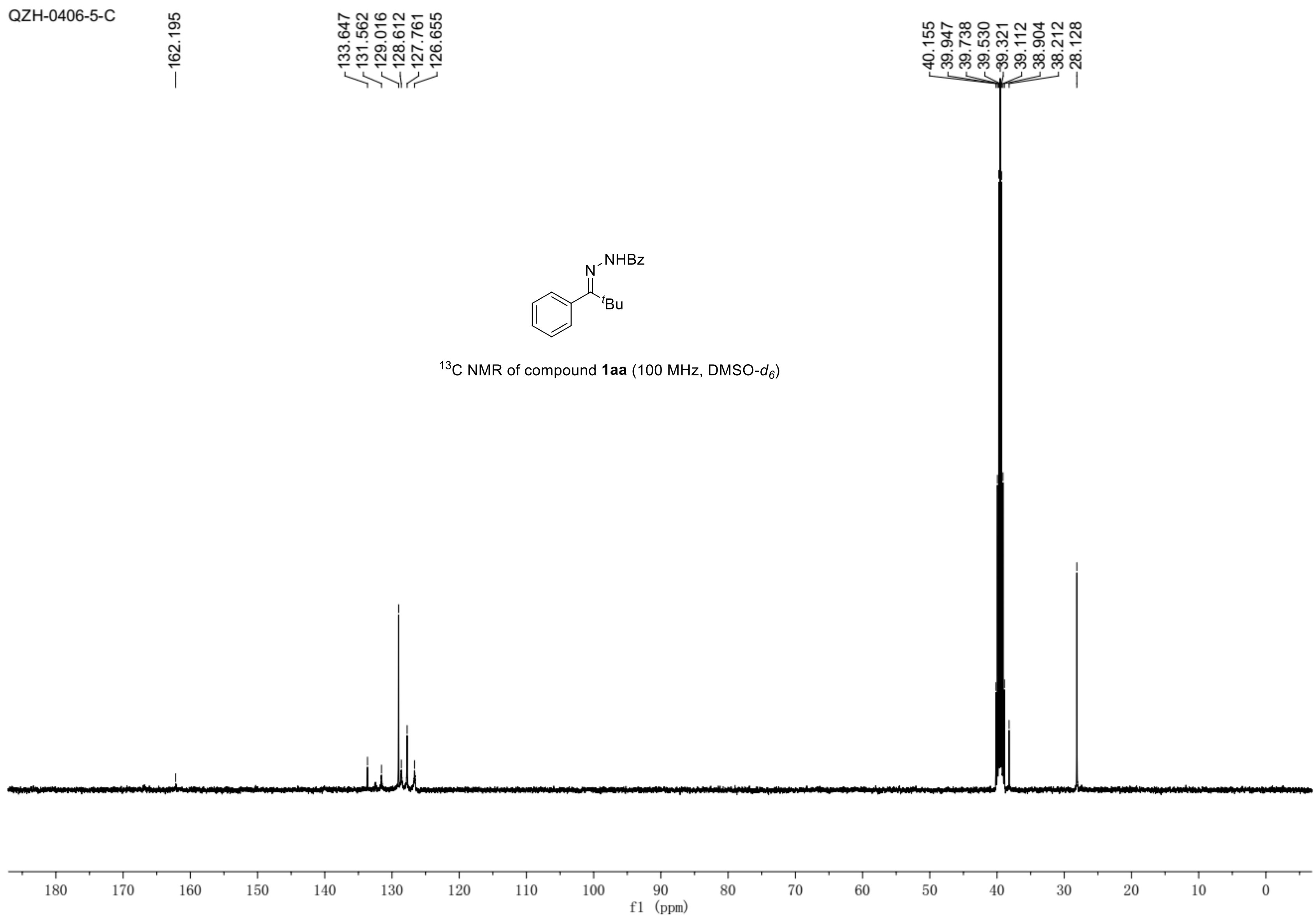

^{13}C NMR of compound **1aa** (100 MHz, DMSO-d_6)

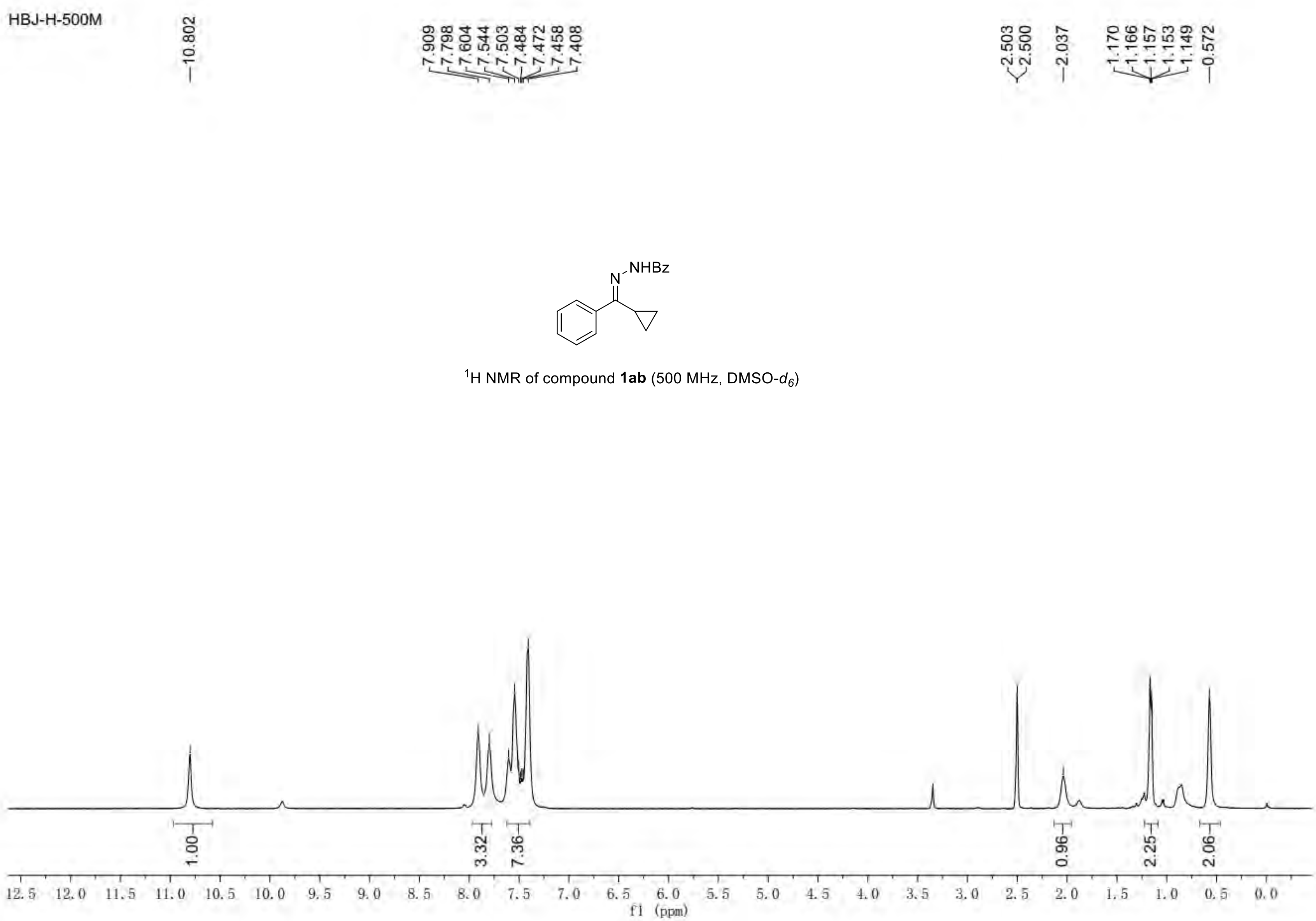

^1H NMR of compound **1ab** (500 MHz, DMSO-d_6)

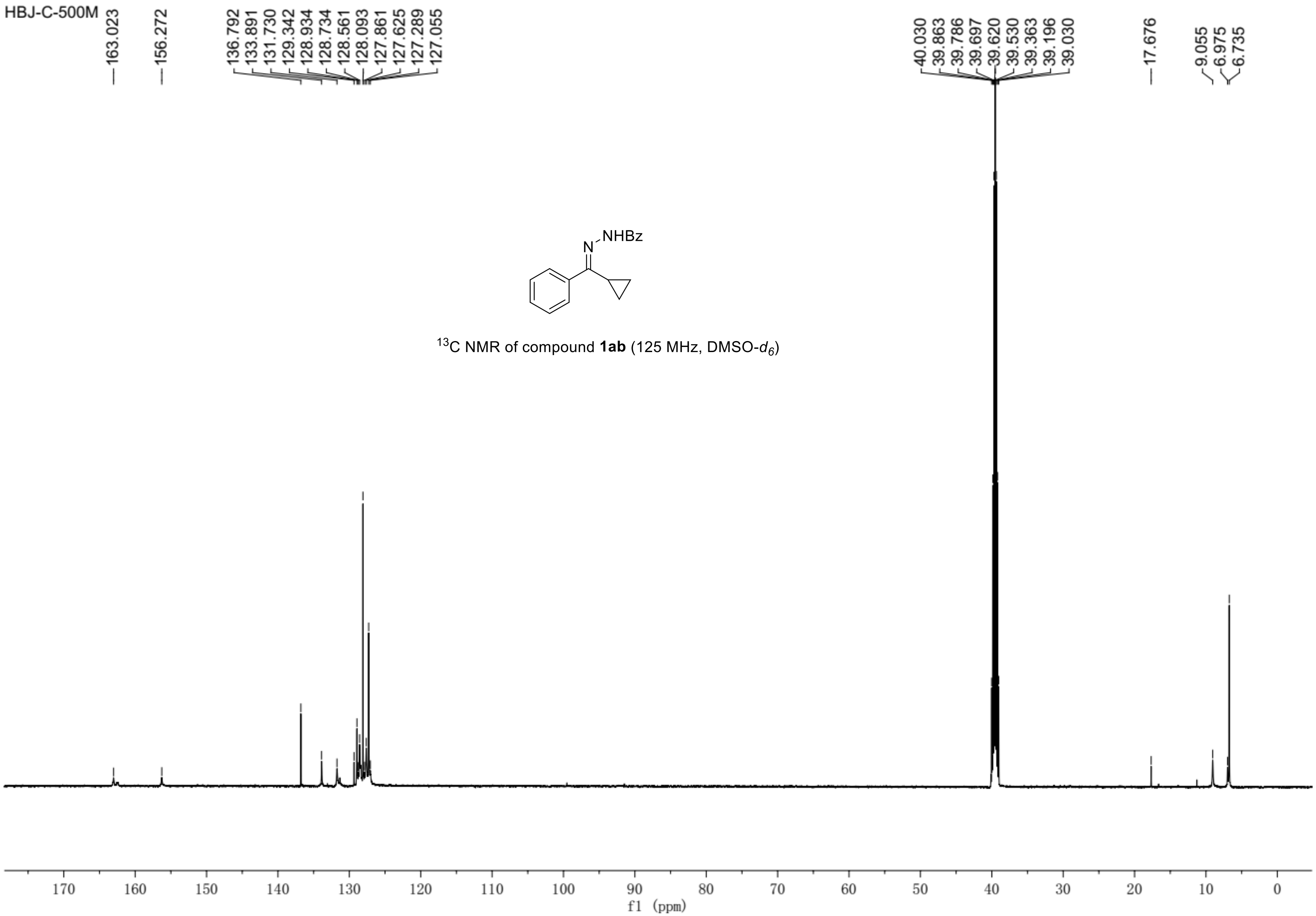

^{13}C NMR of compound **1ab** (125 MHz, DMSO-d_6)

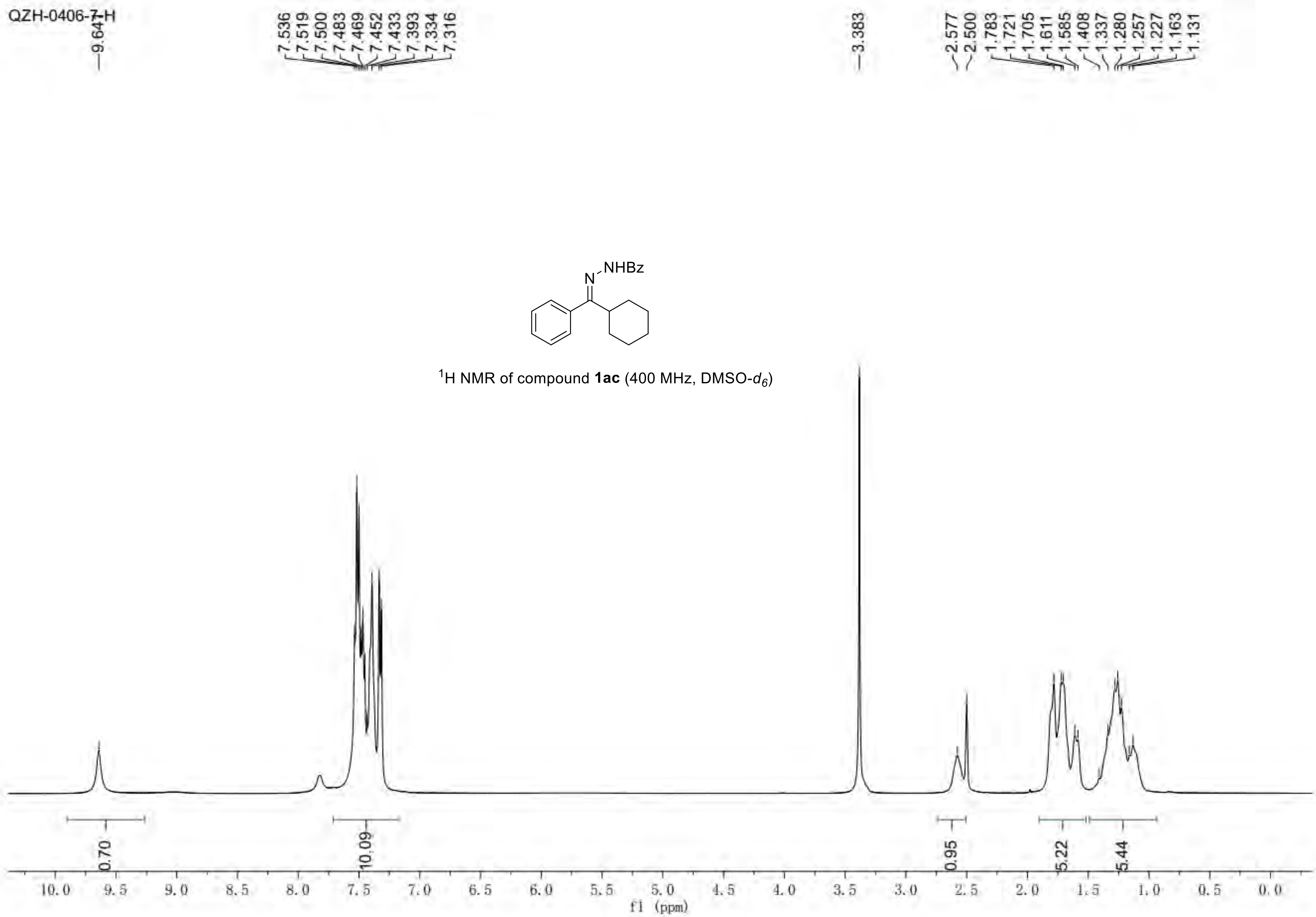

^{1}H NMR of compound **1ac** (400 MHz, DMSO-d_6)

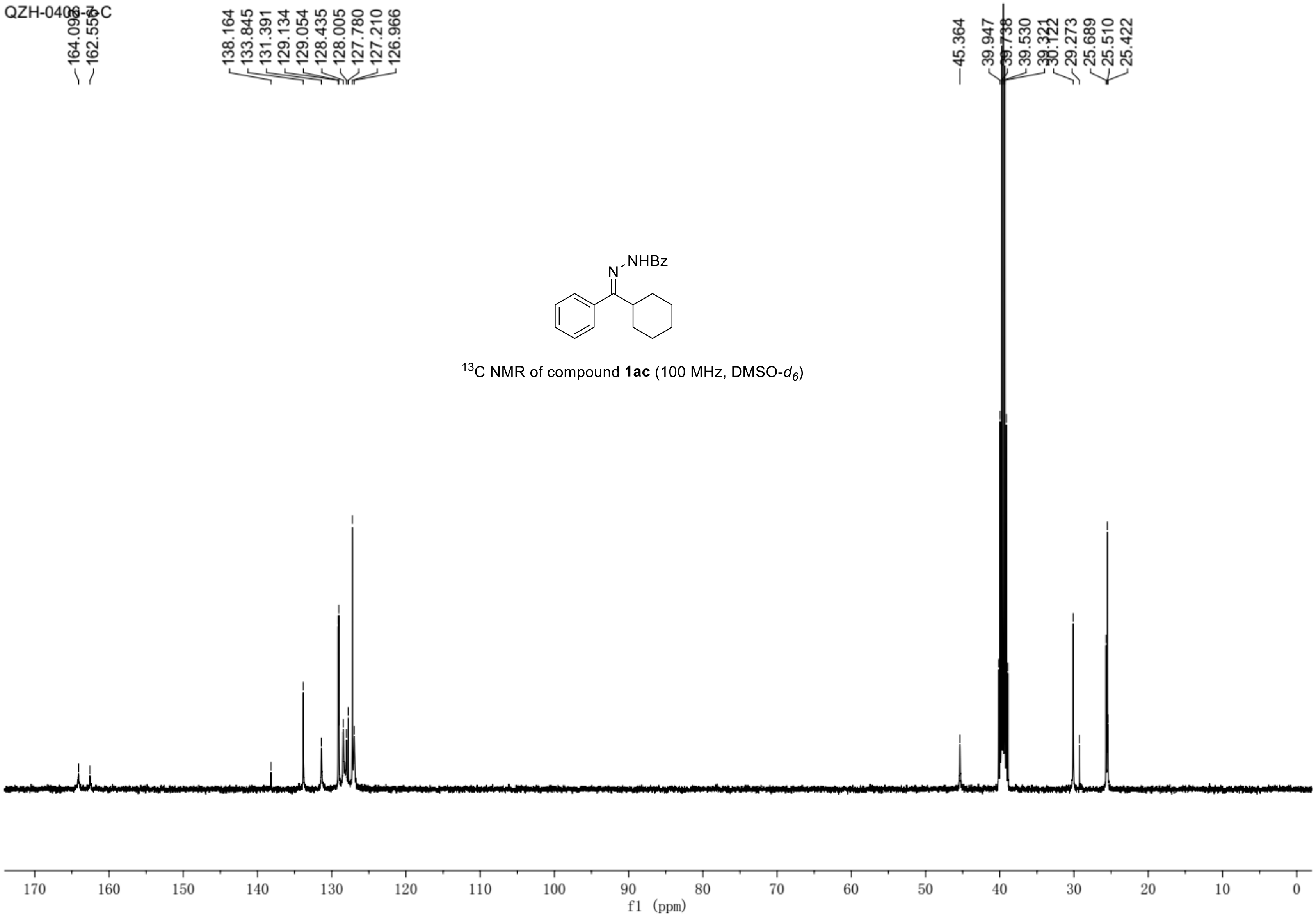

^{13}C NMR of compound **1ac** (100 MHz, DMSO-d_6)

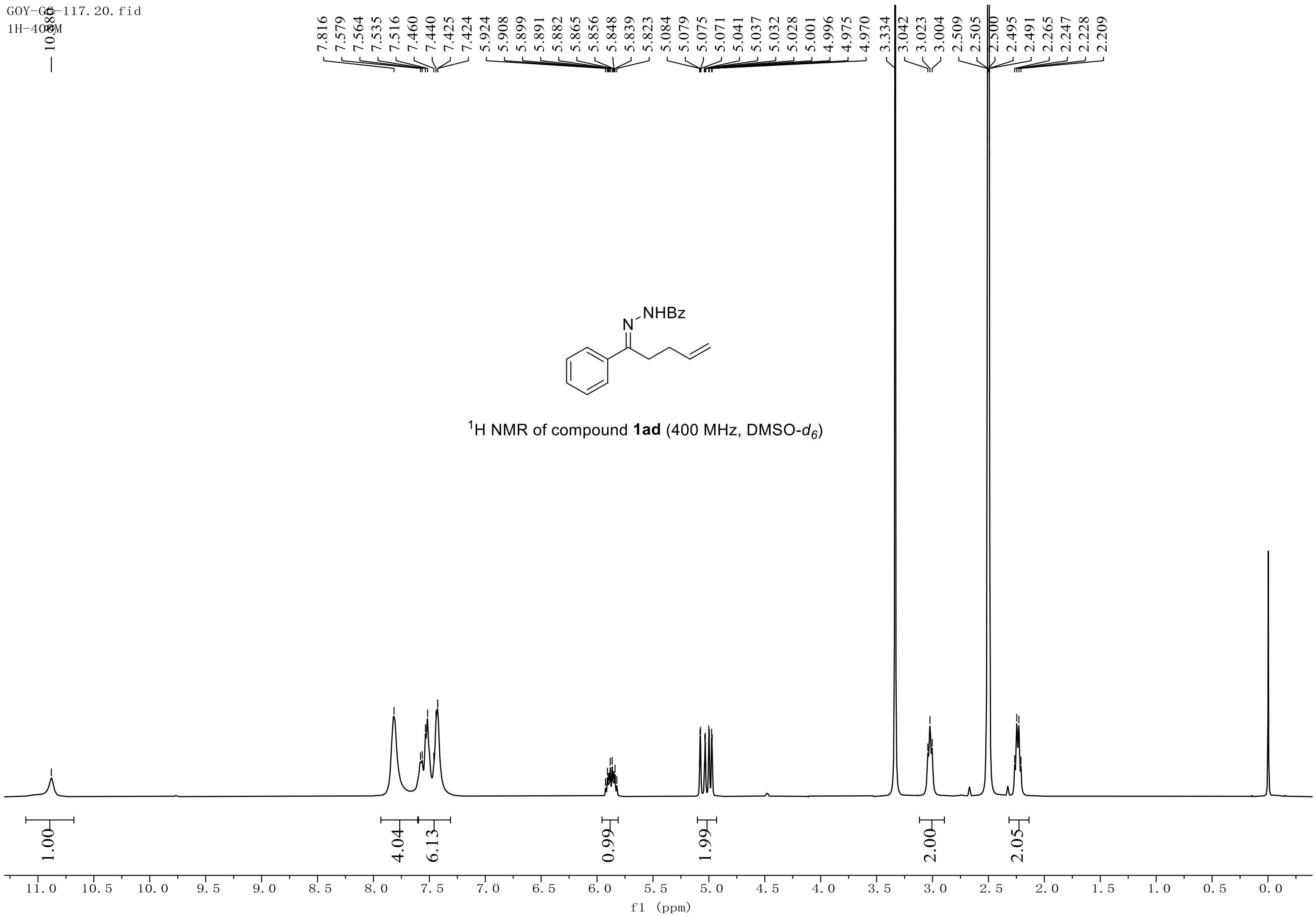

^{1}H NMR of compound **1ad** (400 MHz, DMSO-d_6)

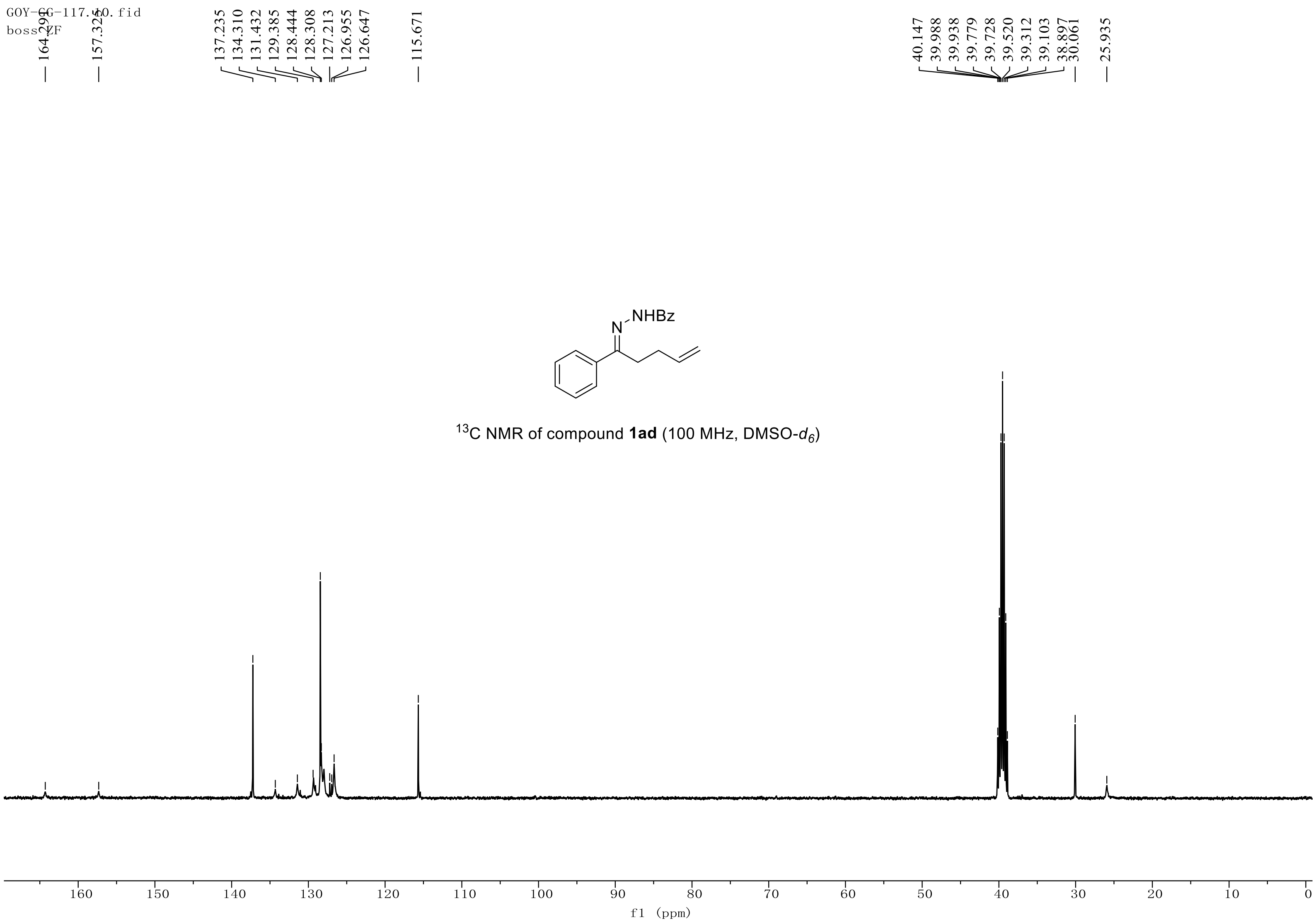

^{13}C NMR of compound **1ad** (100 MHz, DMSO-d_6)

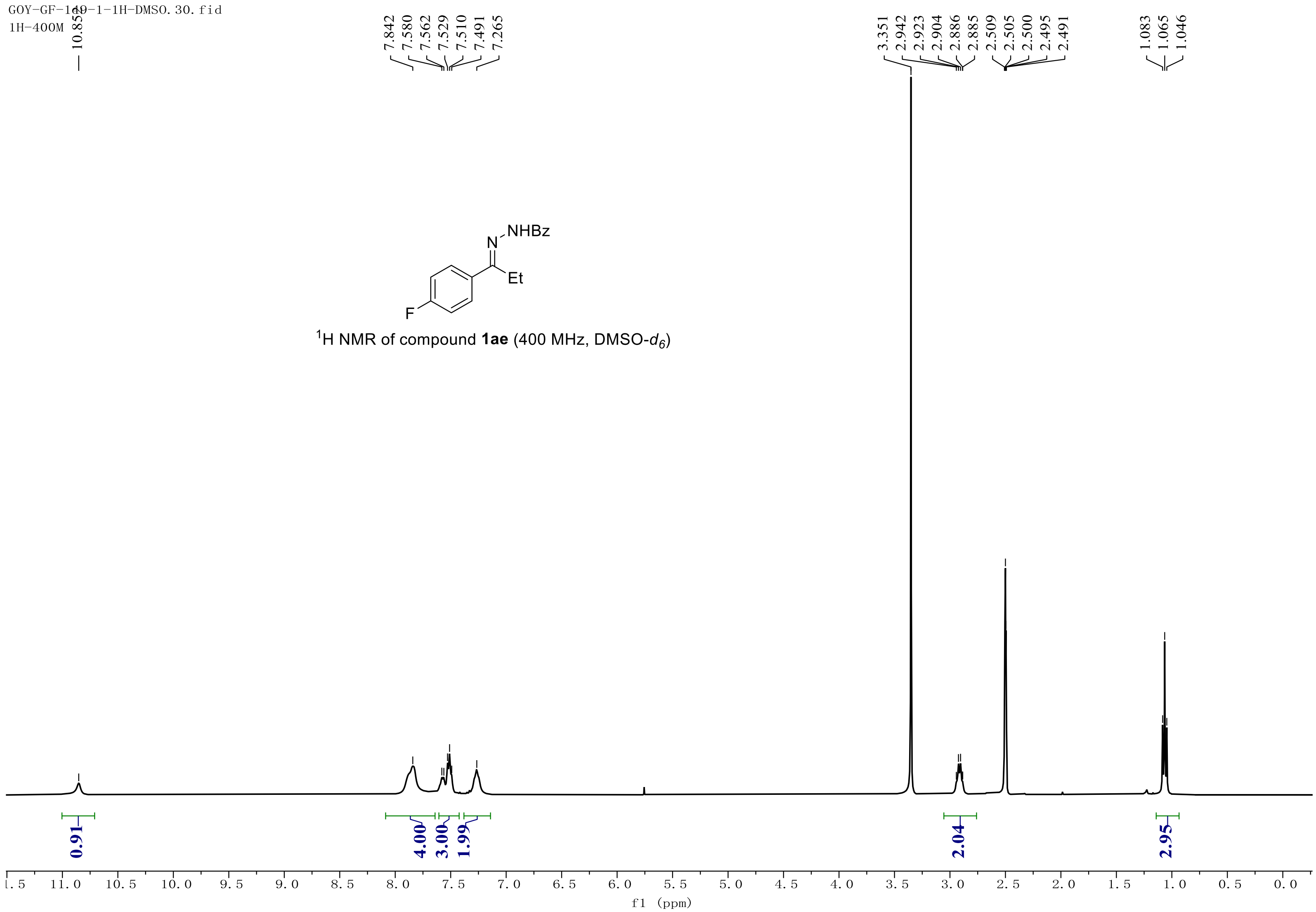

^{1}H NMR of compound **1ae** (400 MHz, DMSO-d_6)

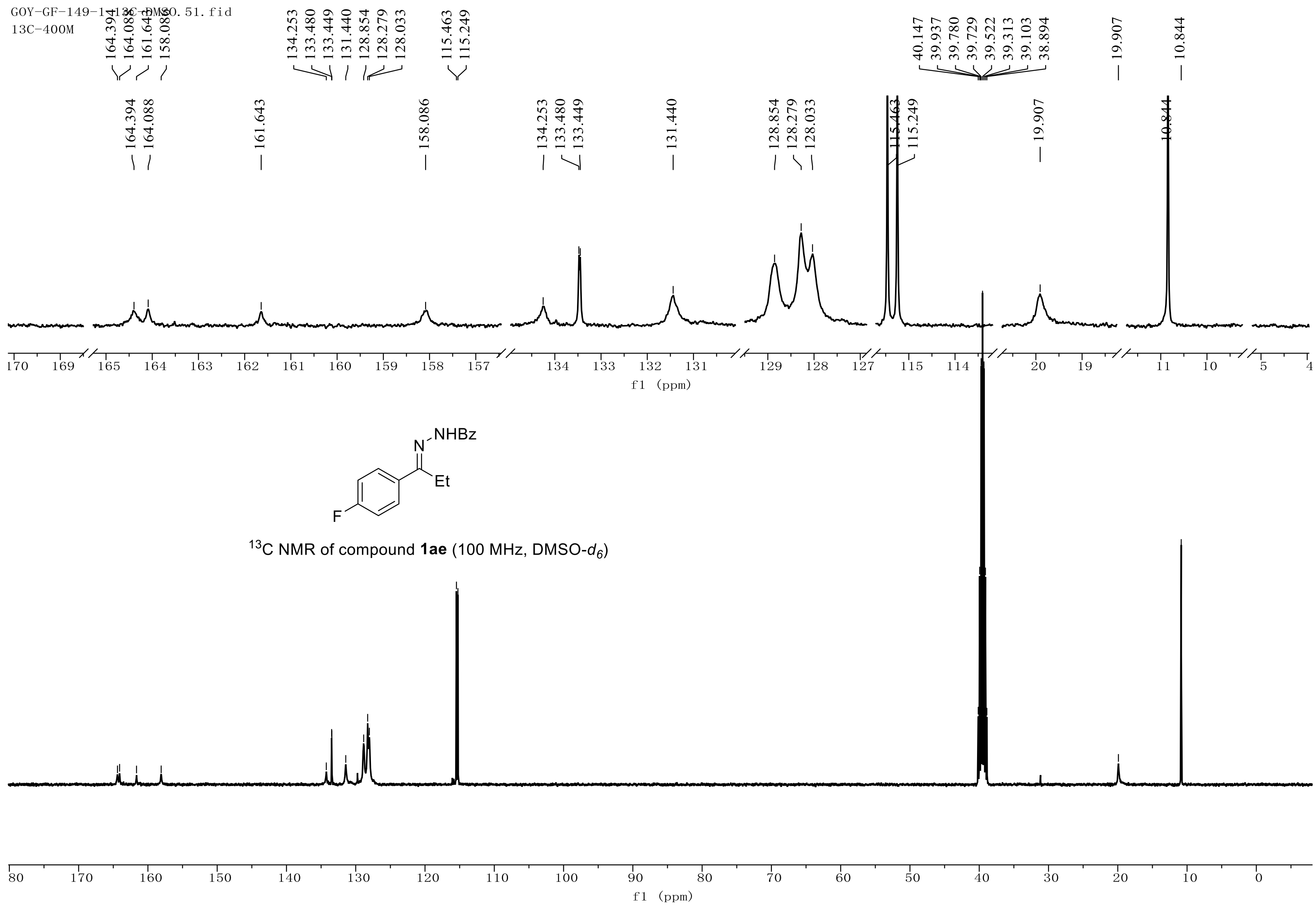

^{13}C NMR of compound **1ae** (100 MHz, DMSO-d_6)

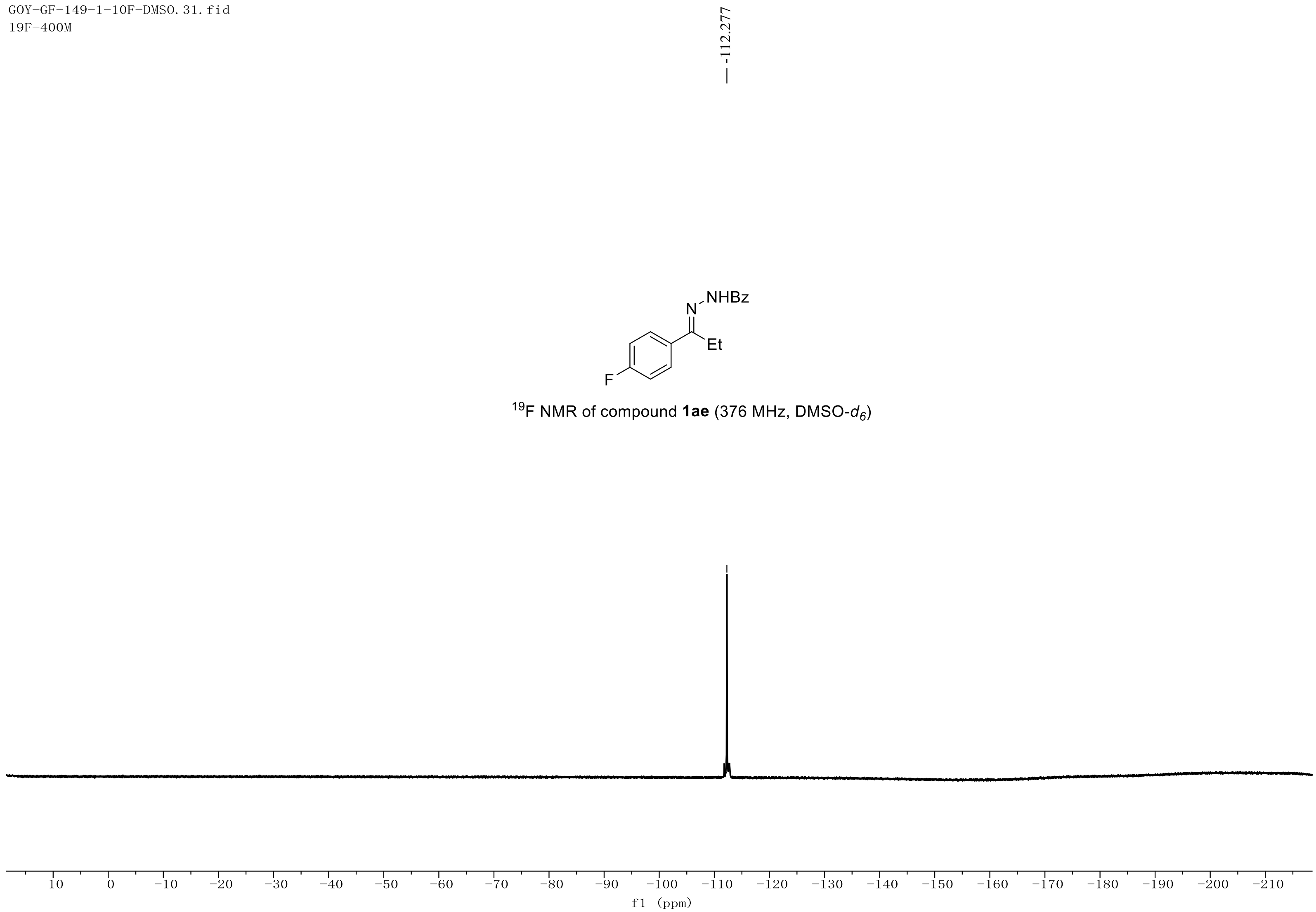

^{19}F NMR of compound **1ae** (376 MHz, DMSO-d_6)

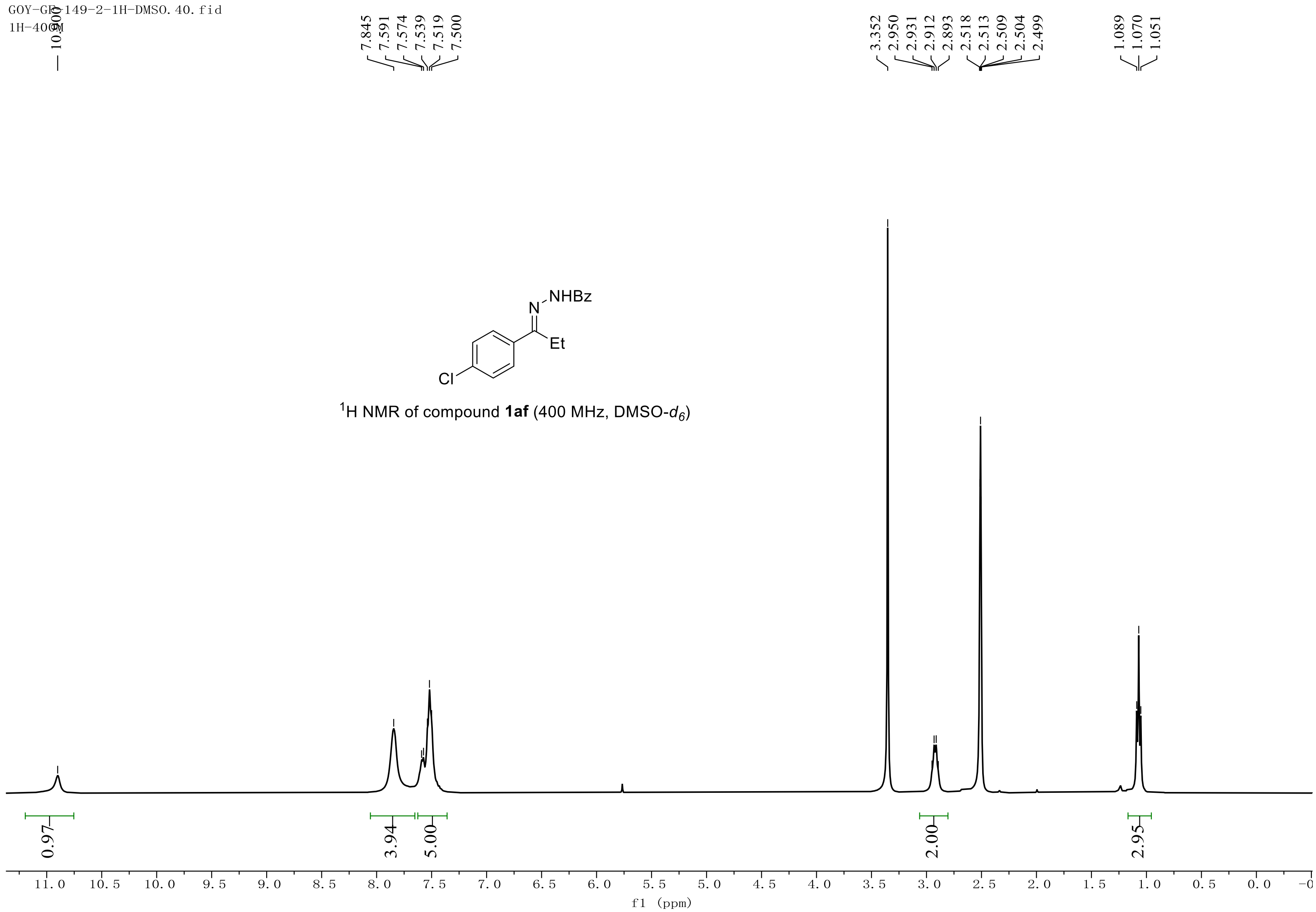

^{1}H NMR of compound **1af** (400 MHz, DMSO-d_6)

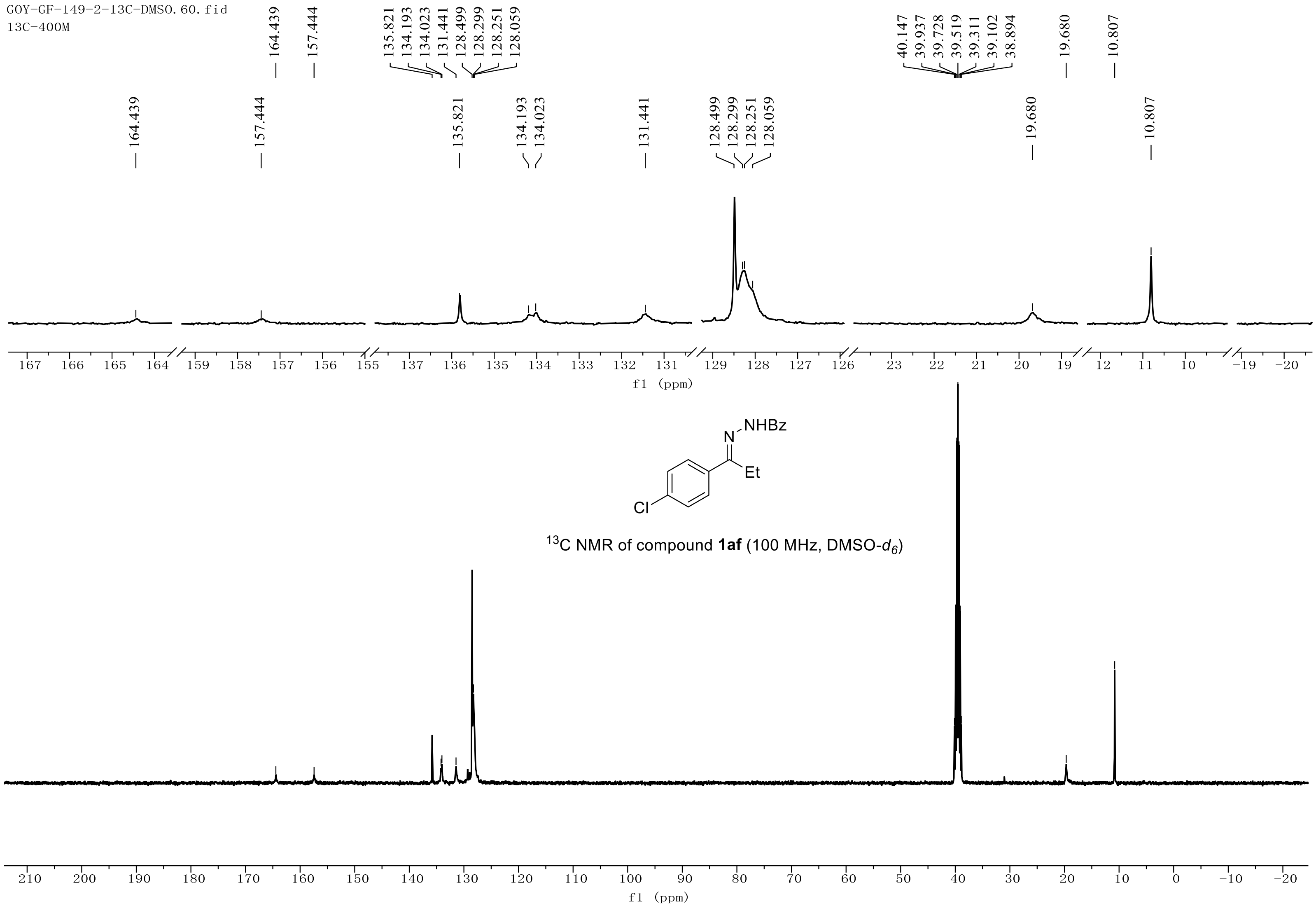

^{13}C NMR of compound **1af** (100 MHz, DMSO-d_6)

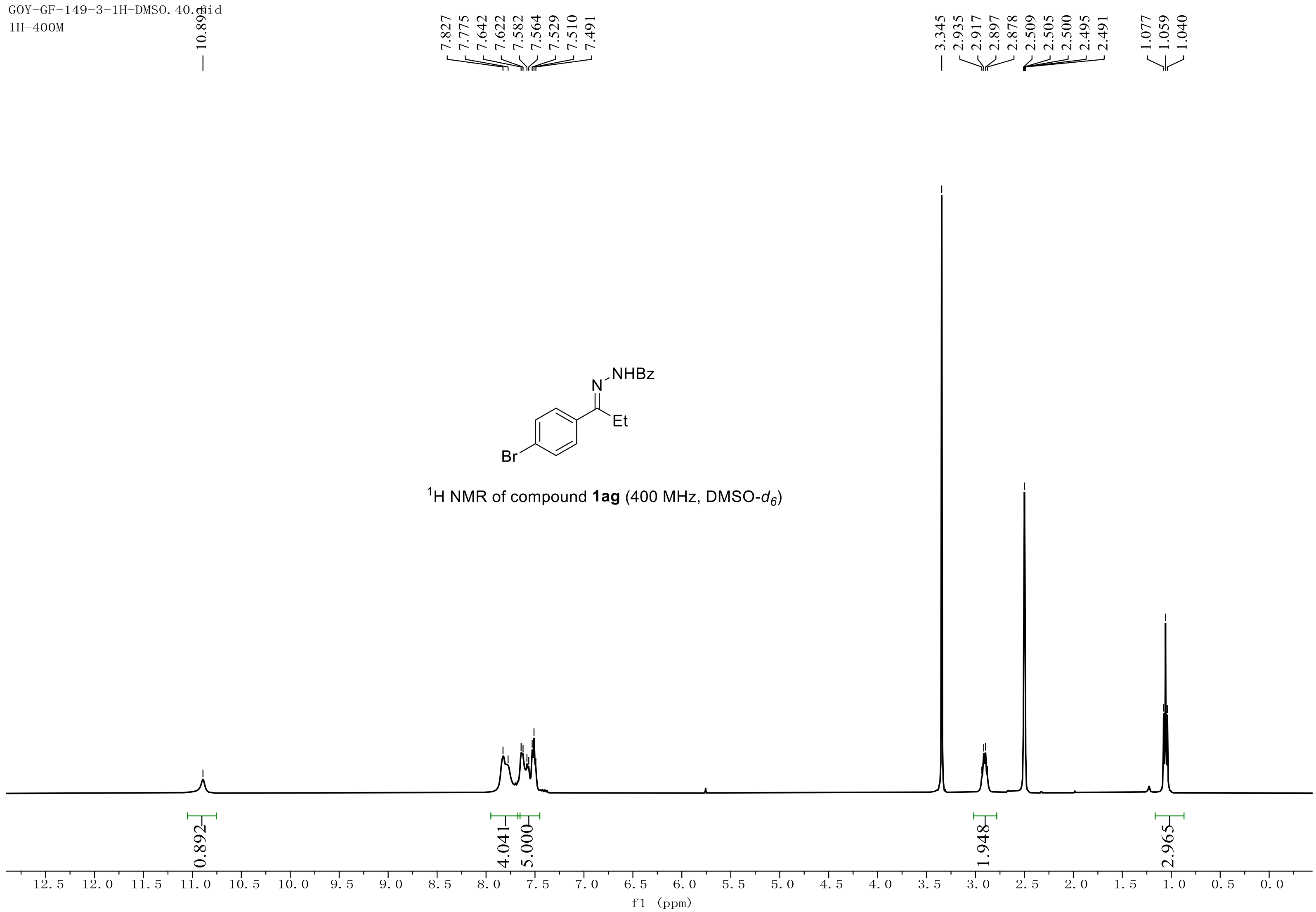

^{1}H NMR of compound **1ag** (400 MHz, DMSO-d_6)

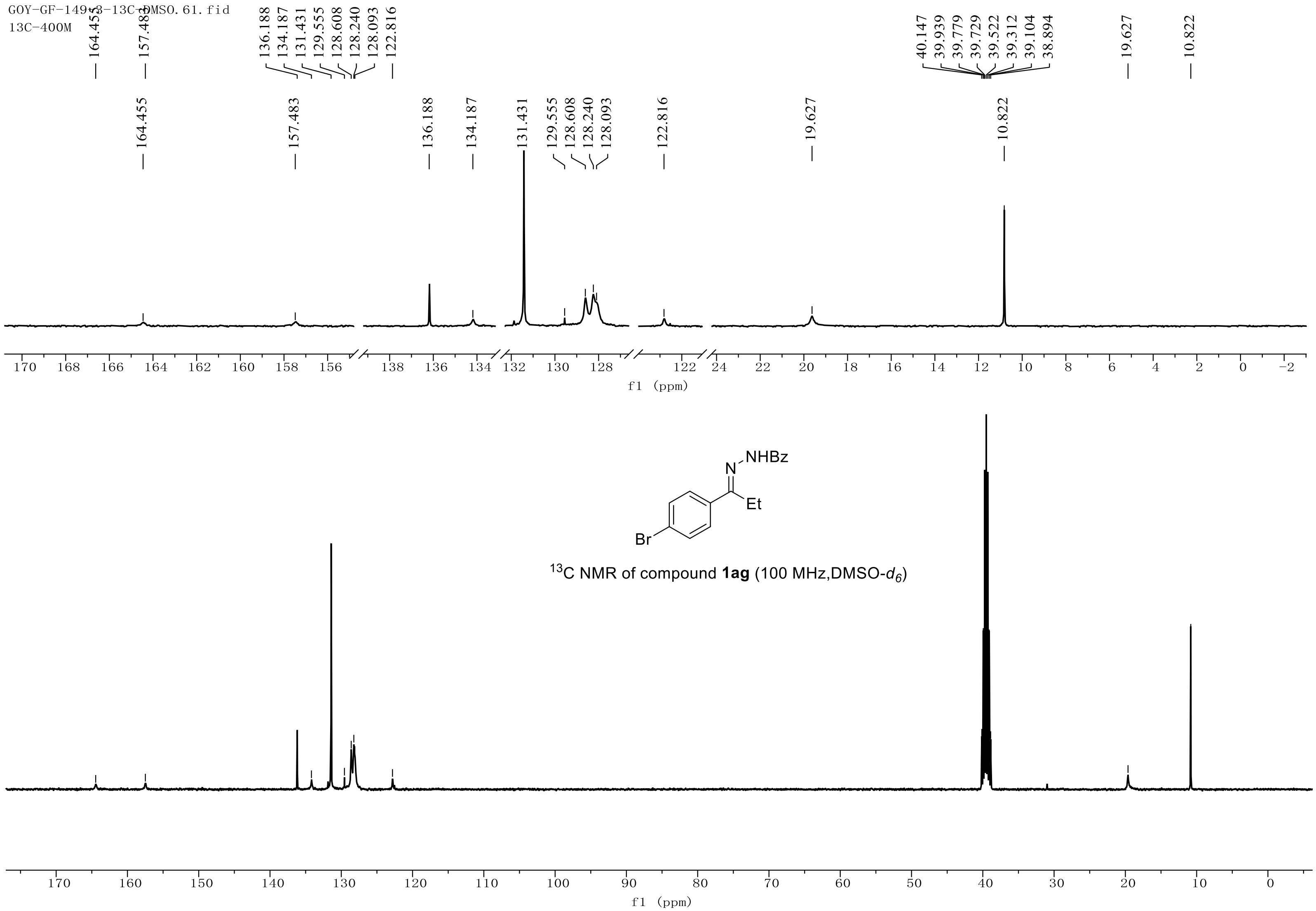

^{13}C NMR of compound **1ag** (100 MHz,DMSO-d_6)

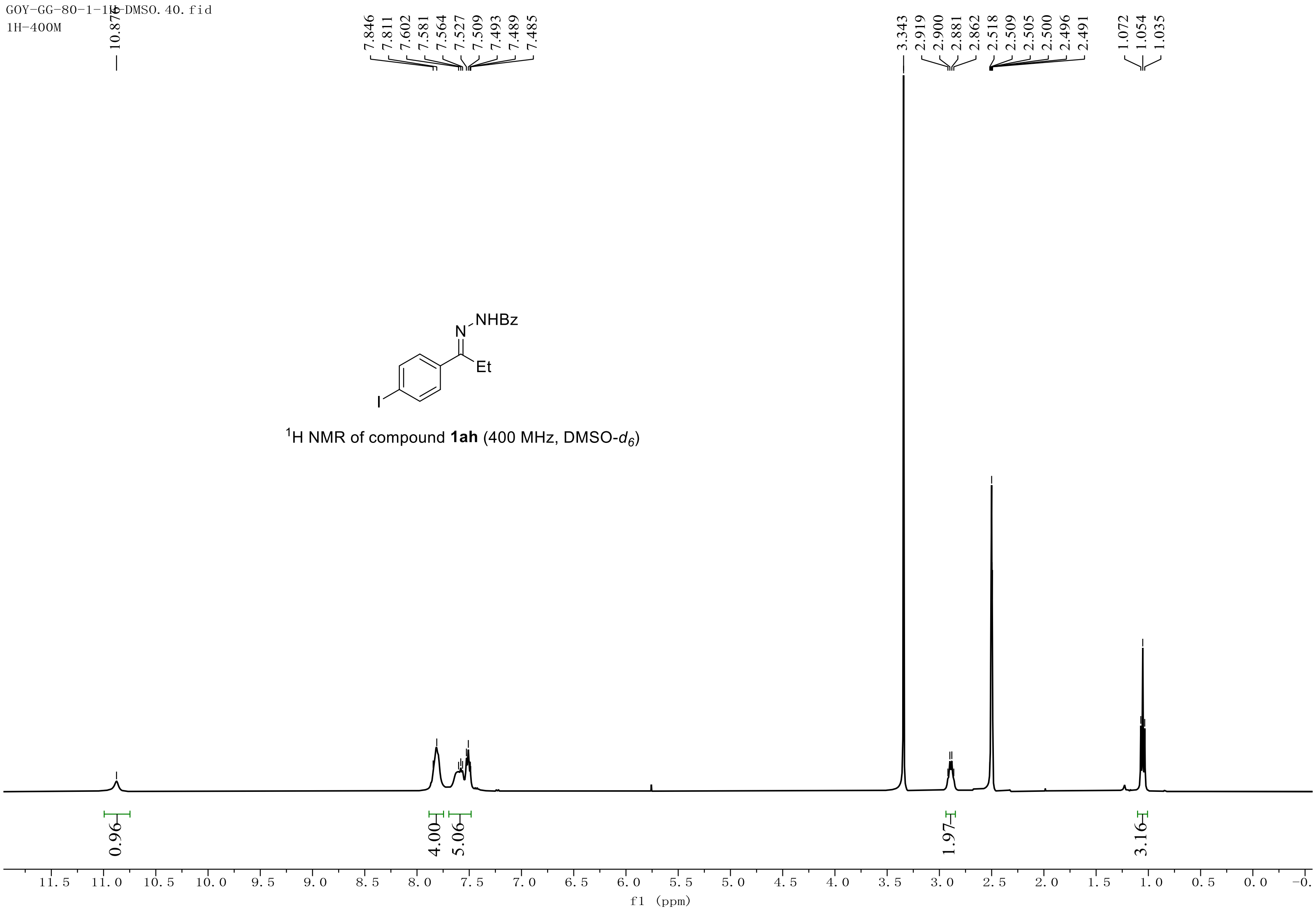

^{1}H NMR of compound **1ah** (400 MHz, DMSO-d_6)

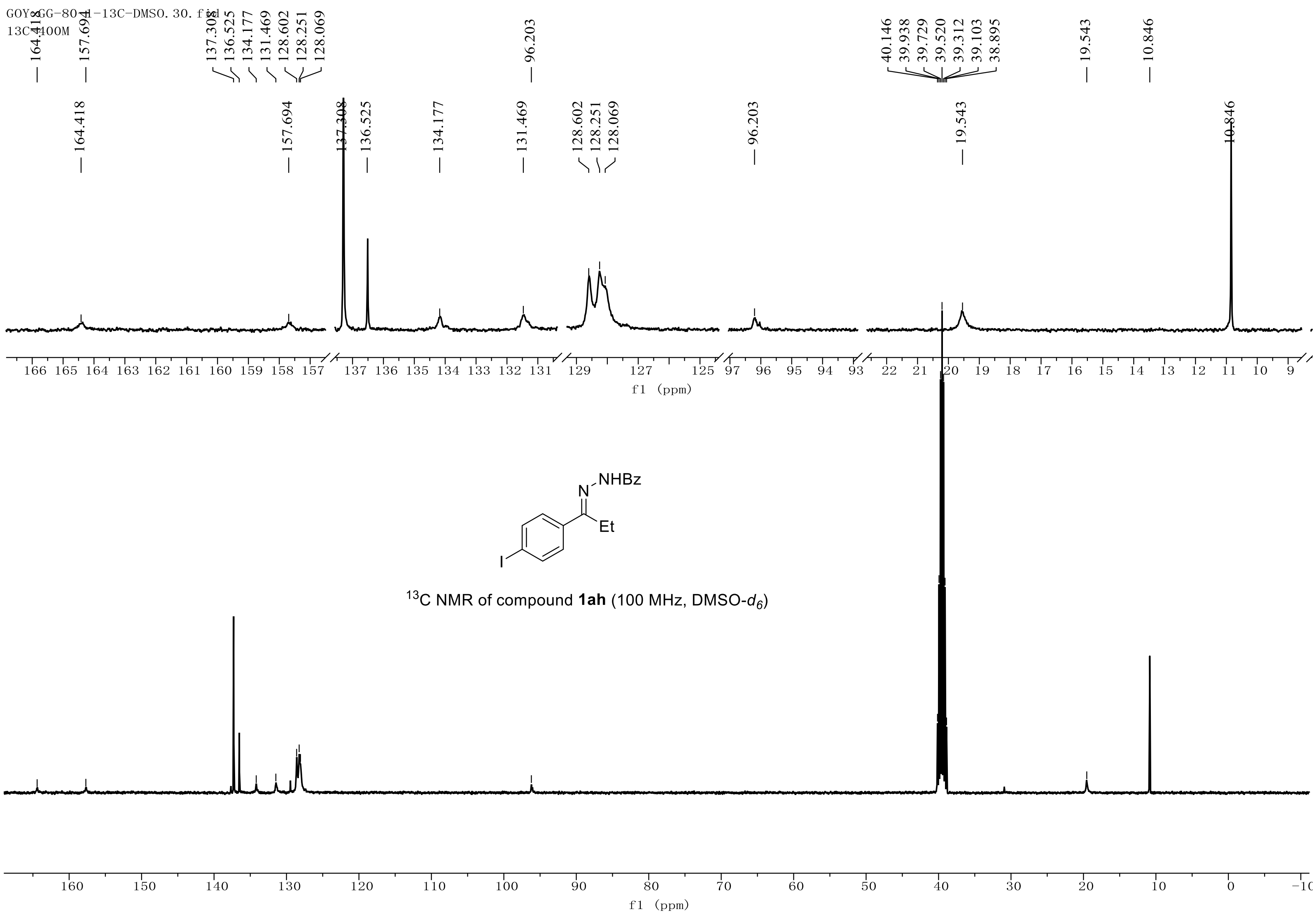

^{13}C NMR of compound **1ah** (100 MHz, DMSO-d_6)

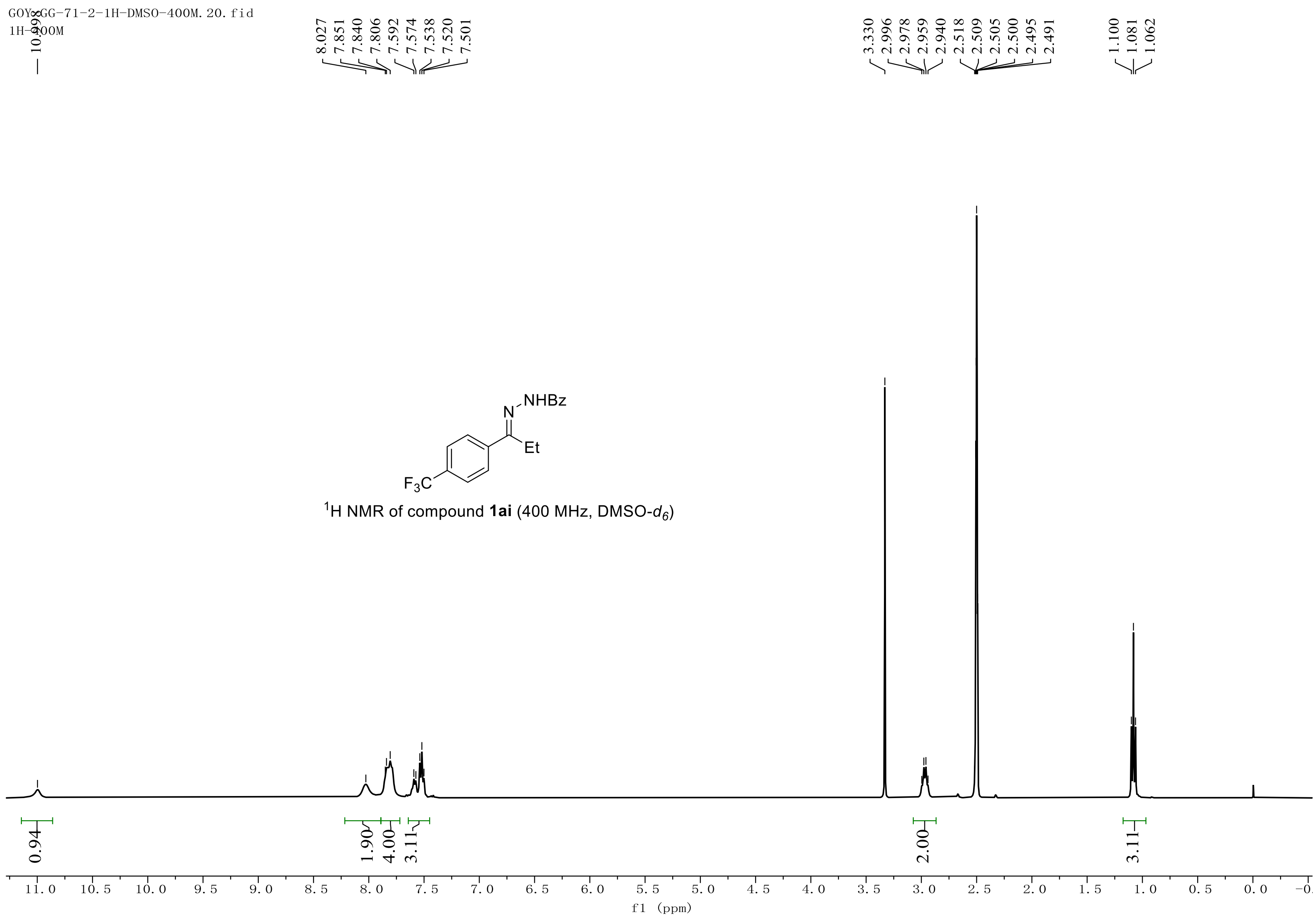

^{1}H NMR of compound **1ai** (400 MHz, DMSO-d_6)

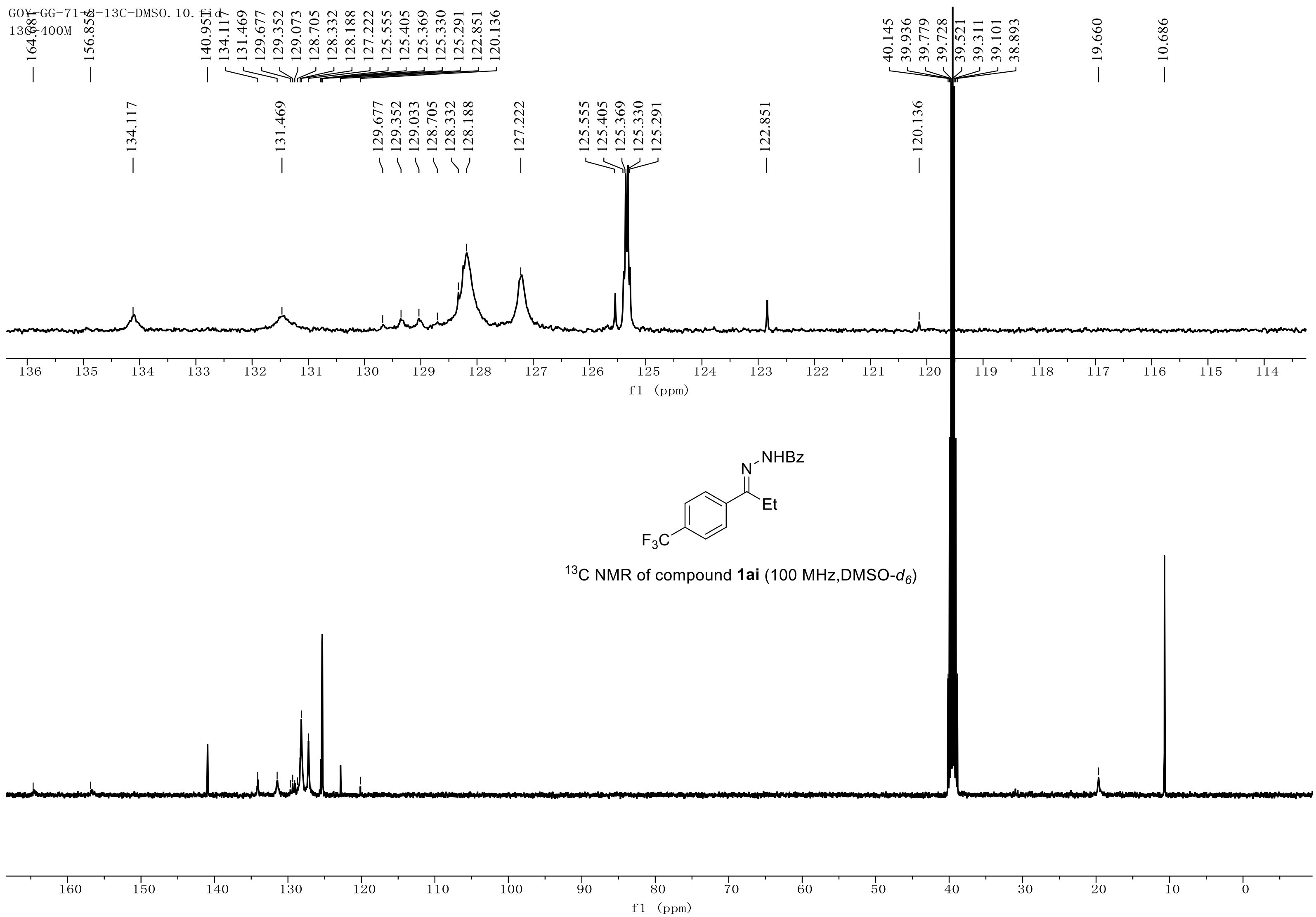

^{13}C NMR of compound **1ai** (100 MHz,DMSO-d_6)

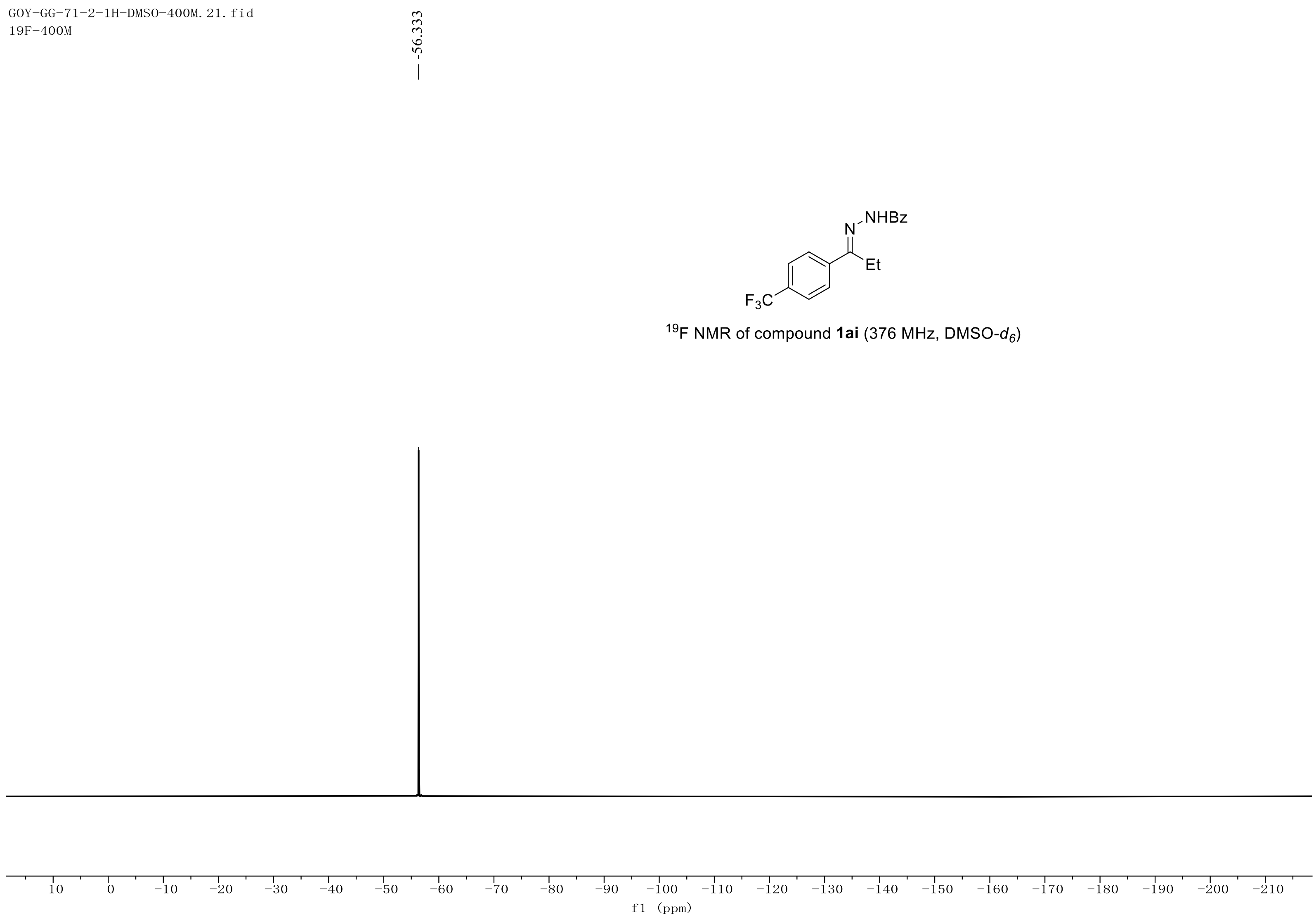

^{19}F NMR of compound **1ai** (376 MHz, DMSO-d_6)

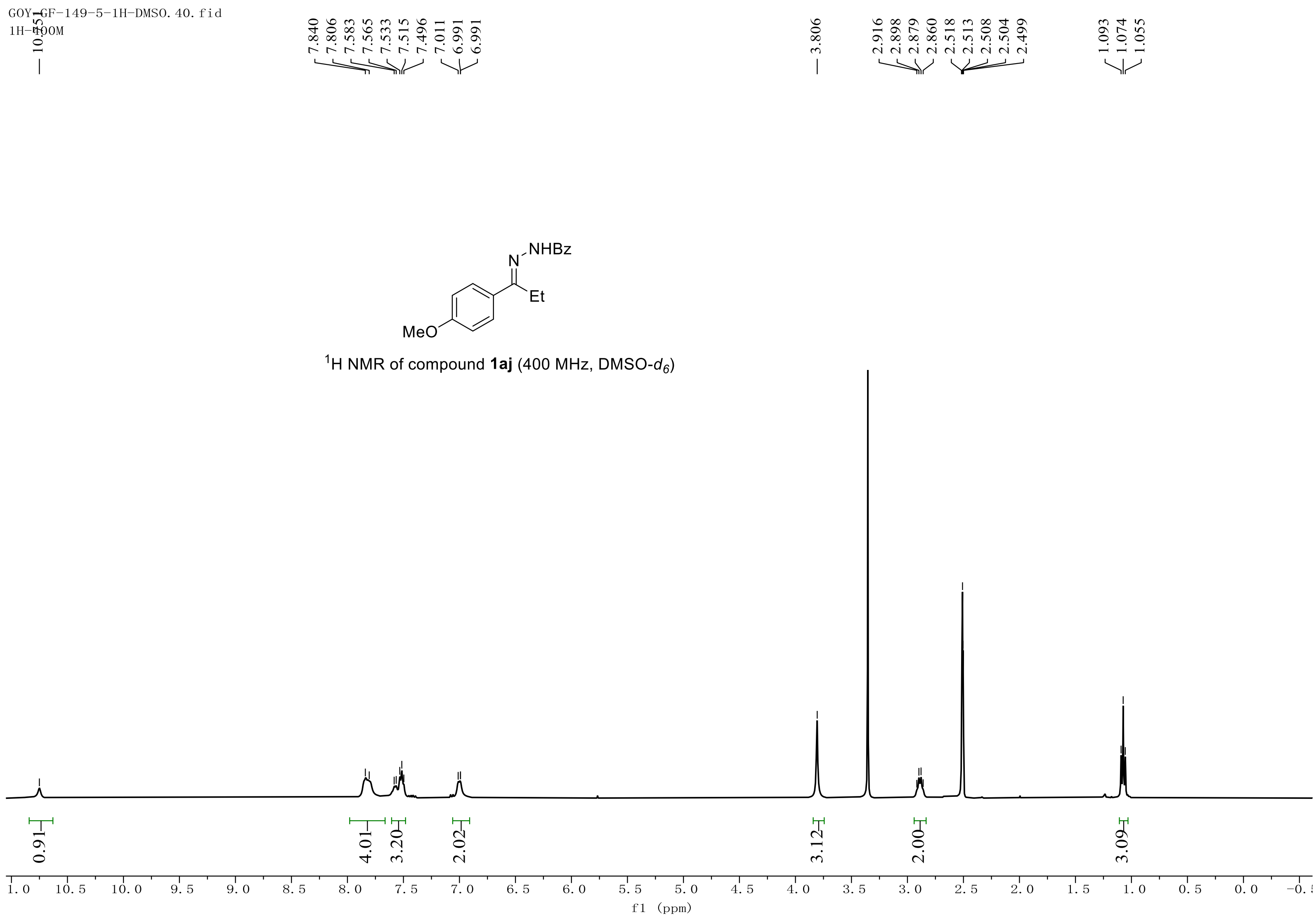

^{1}H NMR of compound **1aj** (400 MHz, DMSO-d_6)

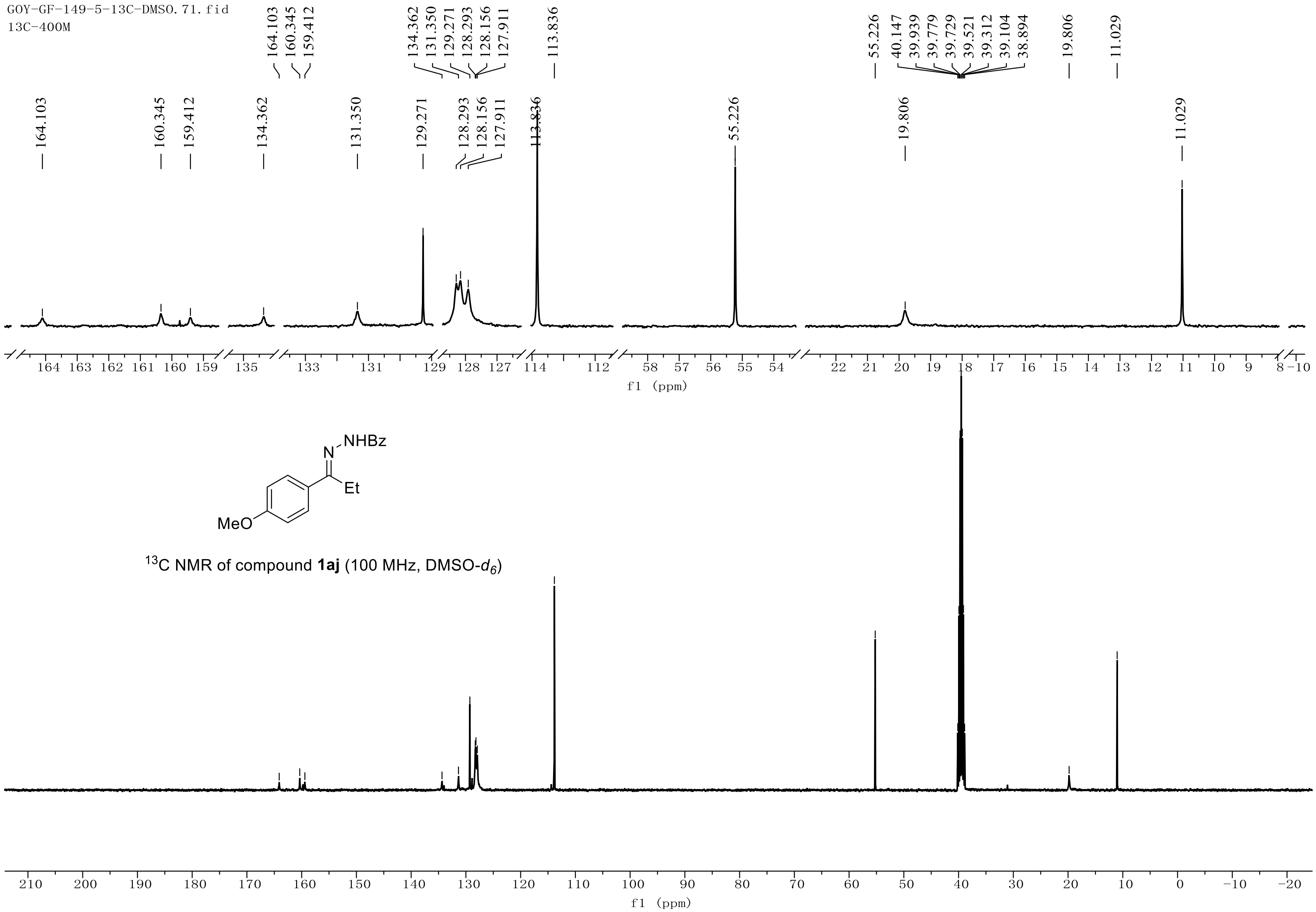

^{13}C NMR of compound **1aj** (100 MHz, DMSO-d_6)

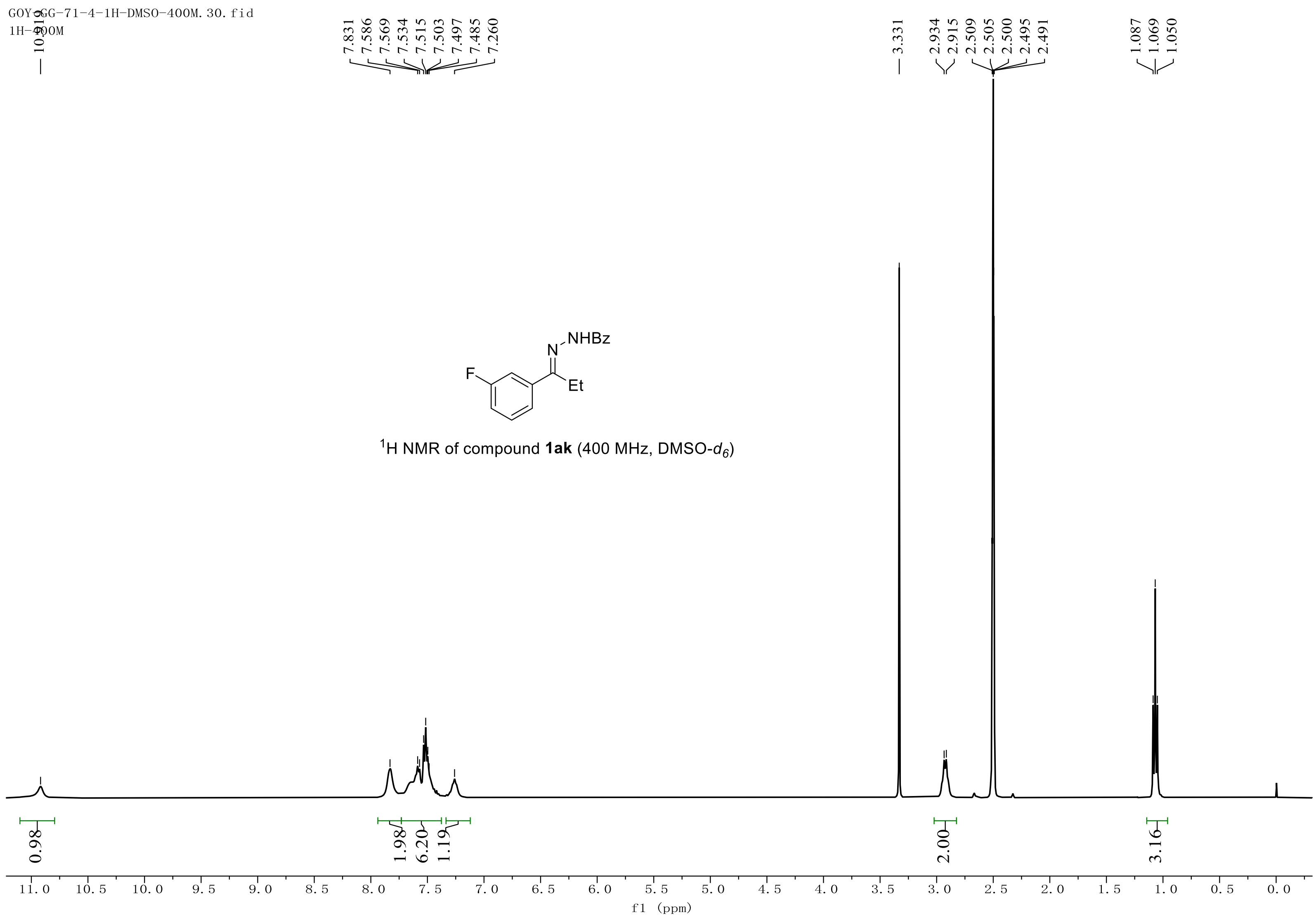

^{1}H NMR of compound **1ak** (400 MHz, DMSO-d_6)

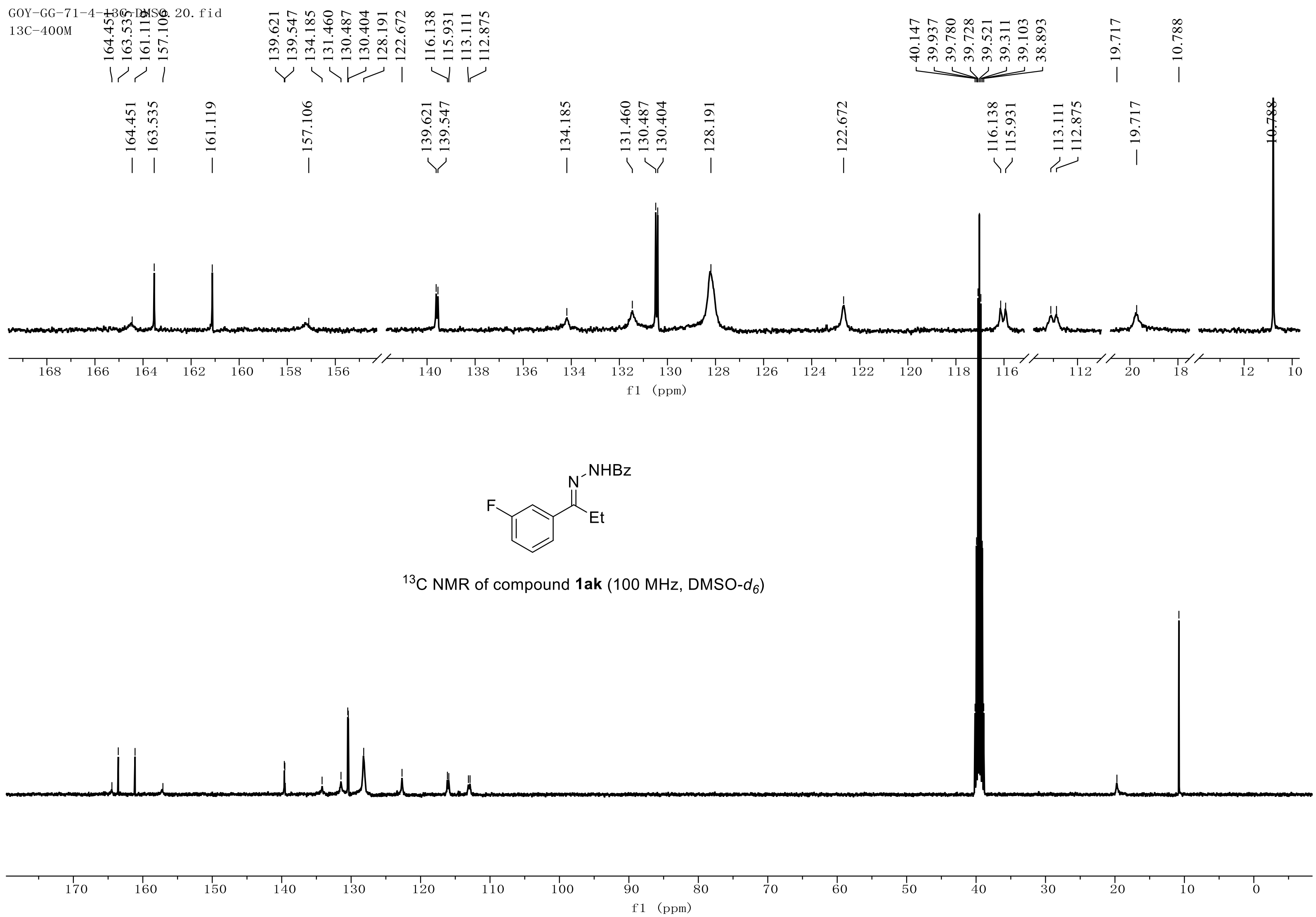

^{13}C NMR of compound **1ak** (100 MHz, DMSO-d_6)

GOY-GG-71-4-1H-DMSO-400M.31.fid
19F-400M

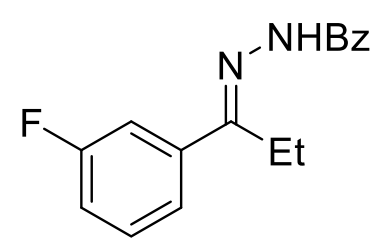

^{19}F NMR of compound **1ak** (376 MHz, DMSO-d_6)

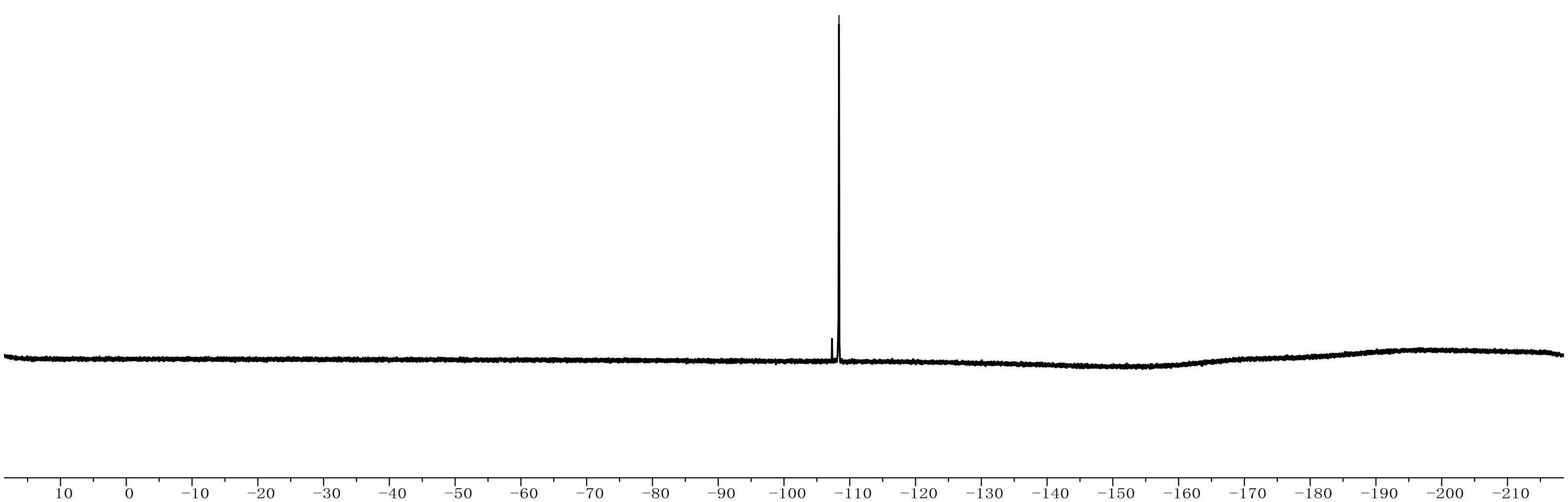

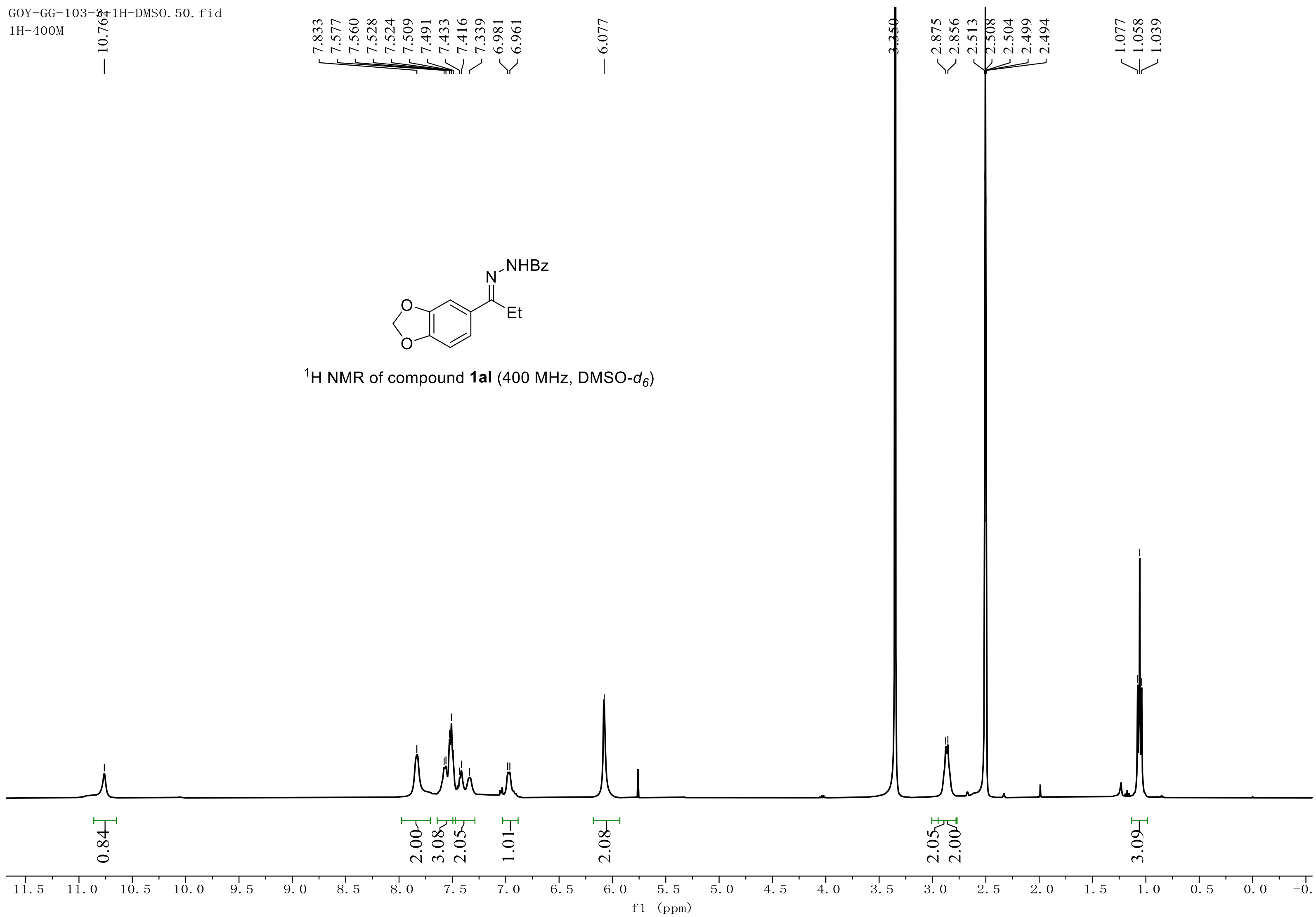

^{1}H NMR of compound **1al** (400 MHz, DMSO-d_6)

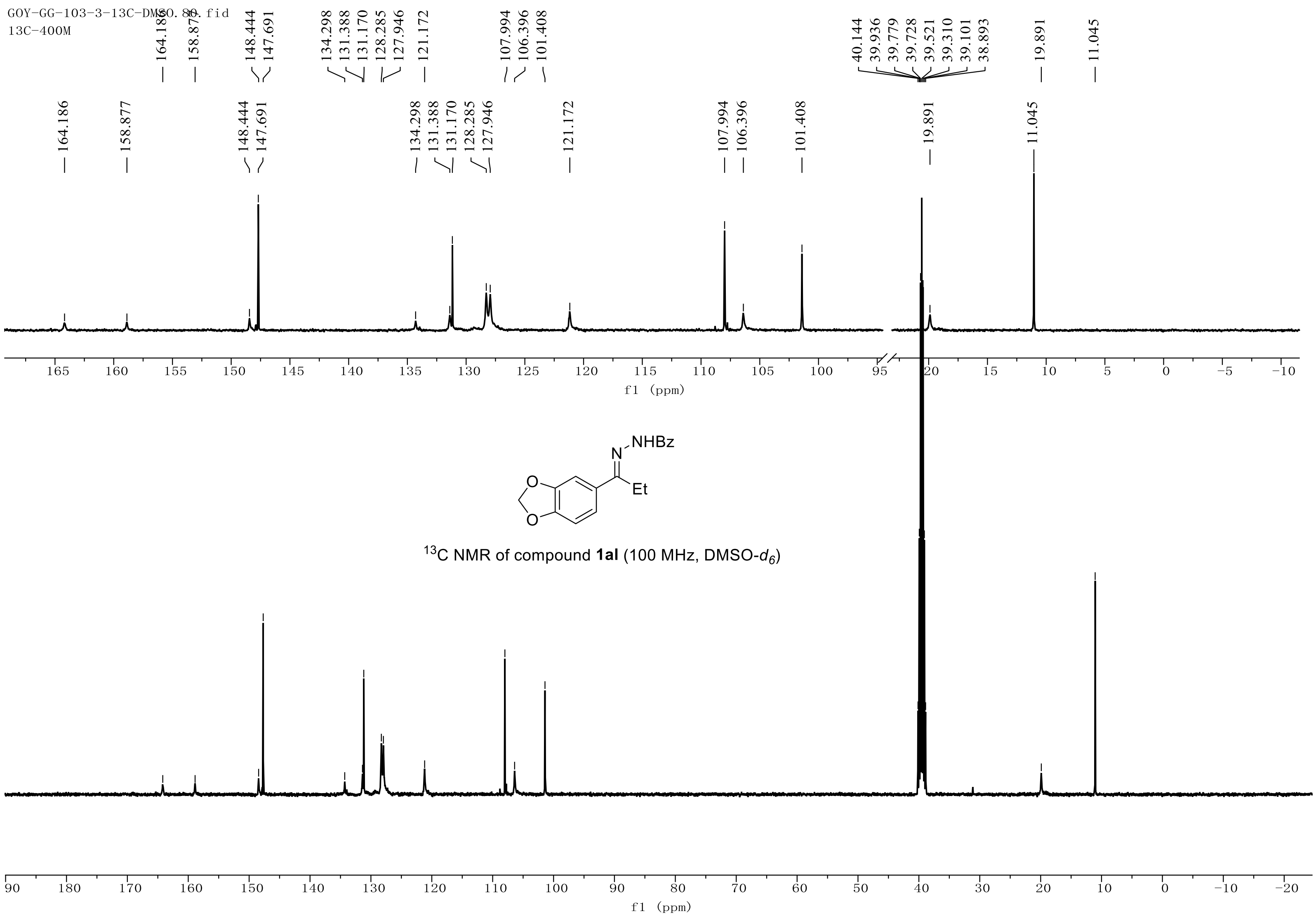

^{13}C NMR of compound **1al** (100 MHz, DMSO-d_6)

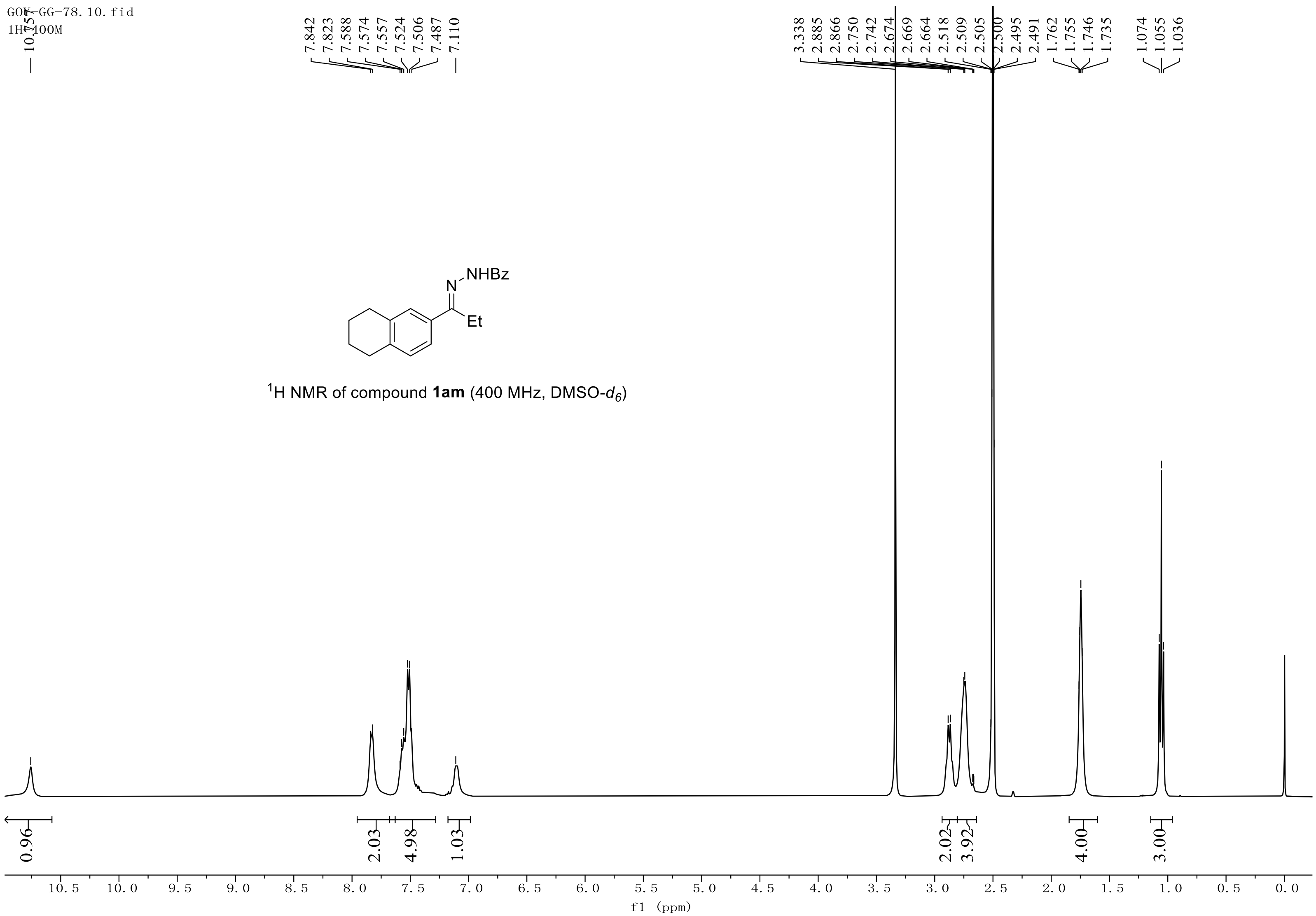

^{1}H NMR of compound **1am** (400 MHz, DMSO-d_6)

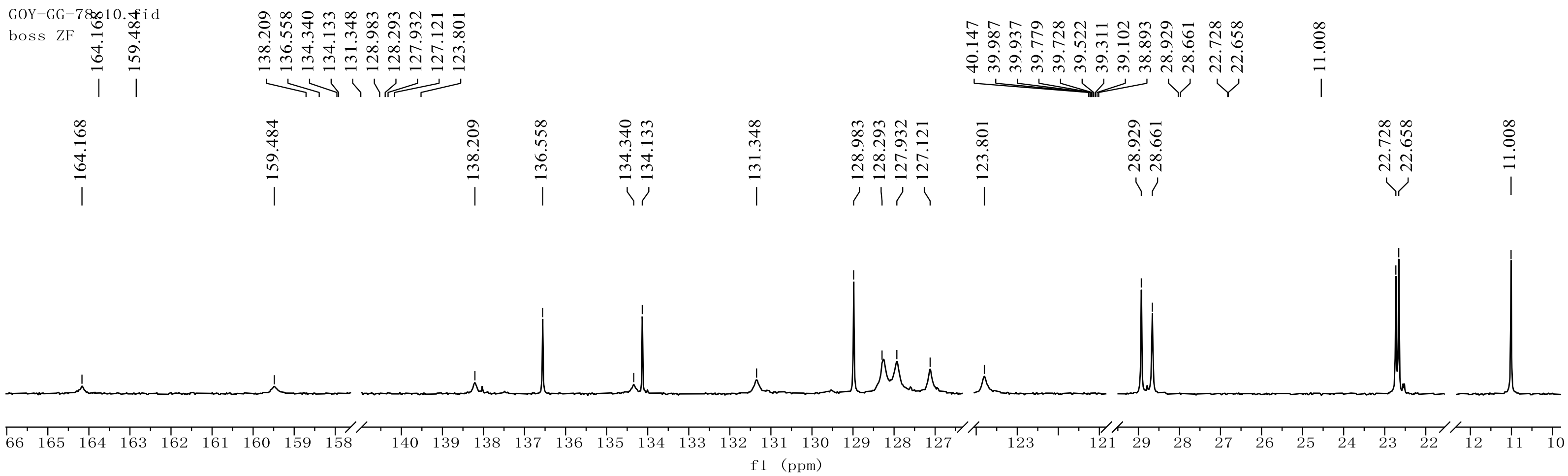

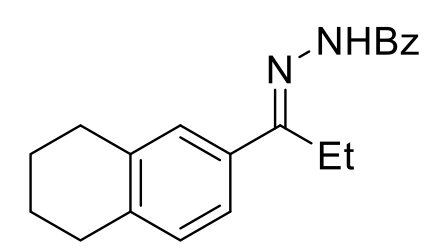

^{13}C NMR of compound **1am** (100 MHz,DMSO-d_6)

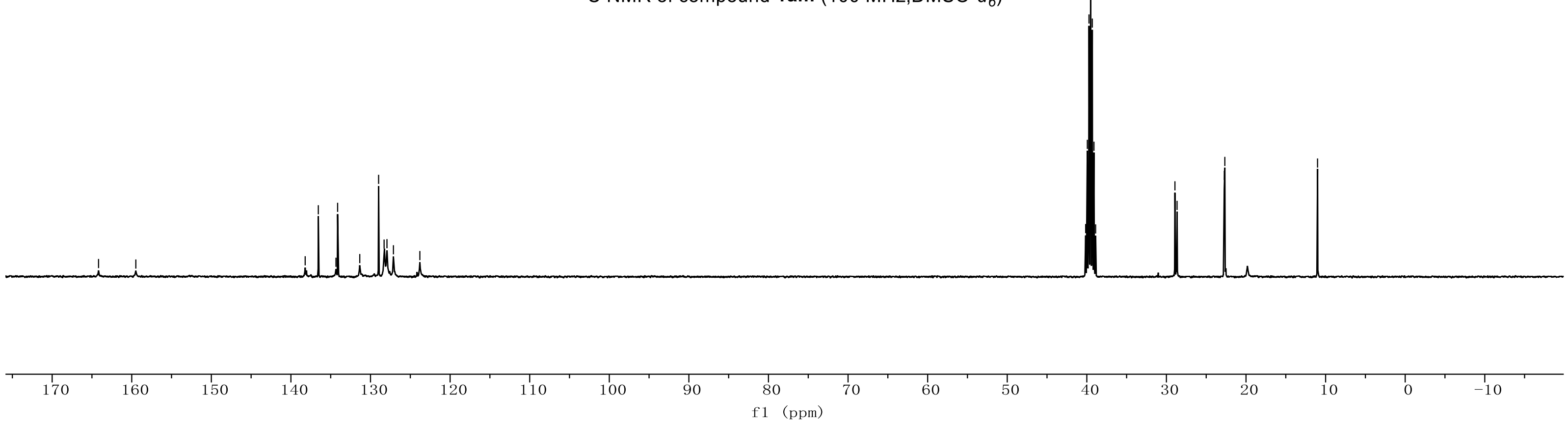

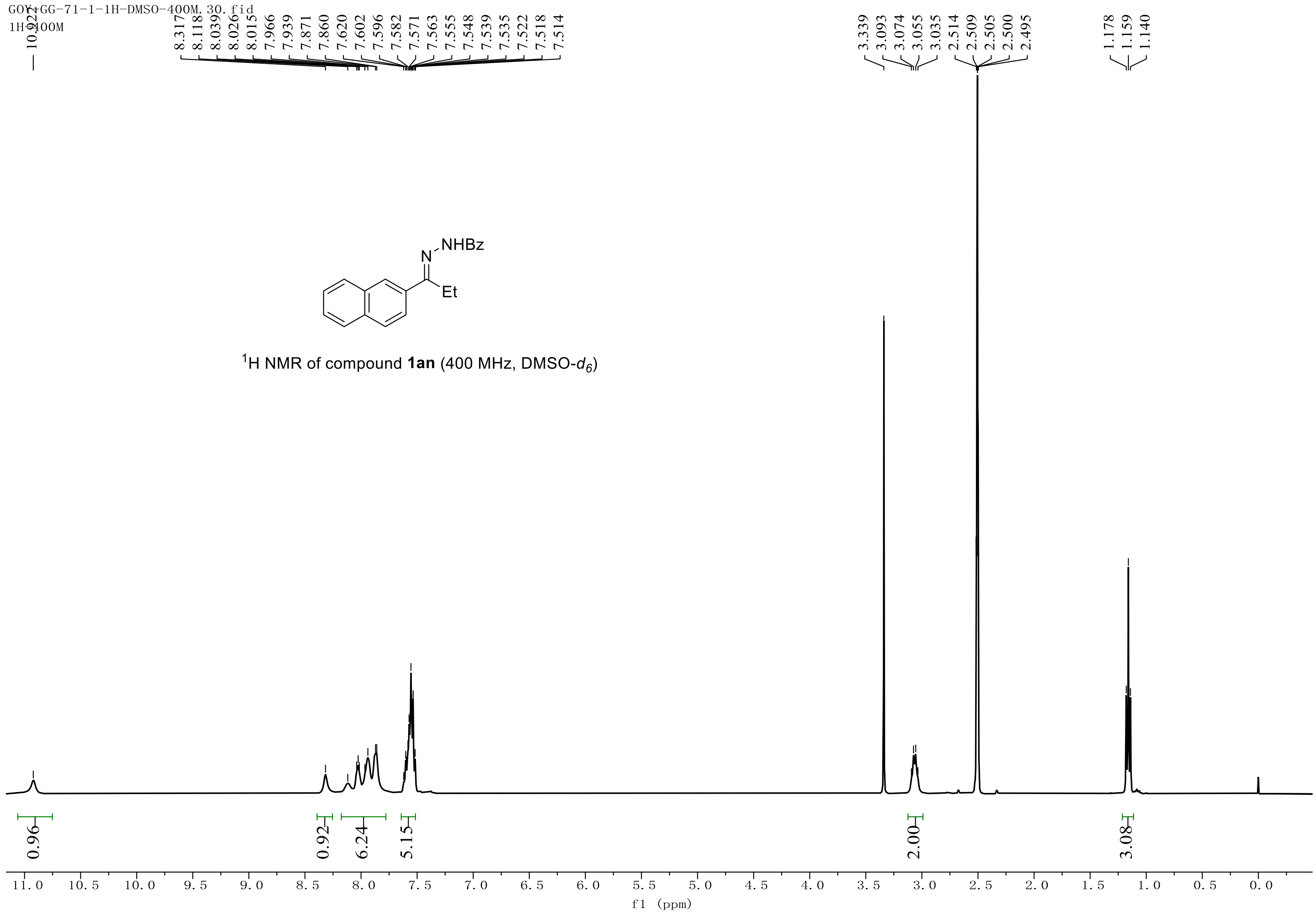

^{1}H NMR of compound **1an** (400 MHz, DMSO-d_6)

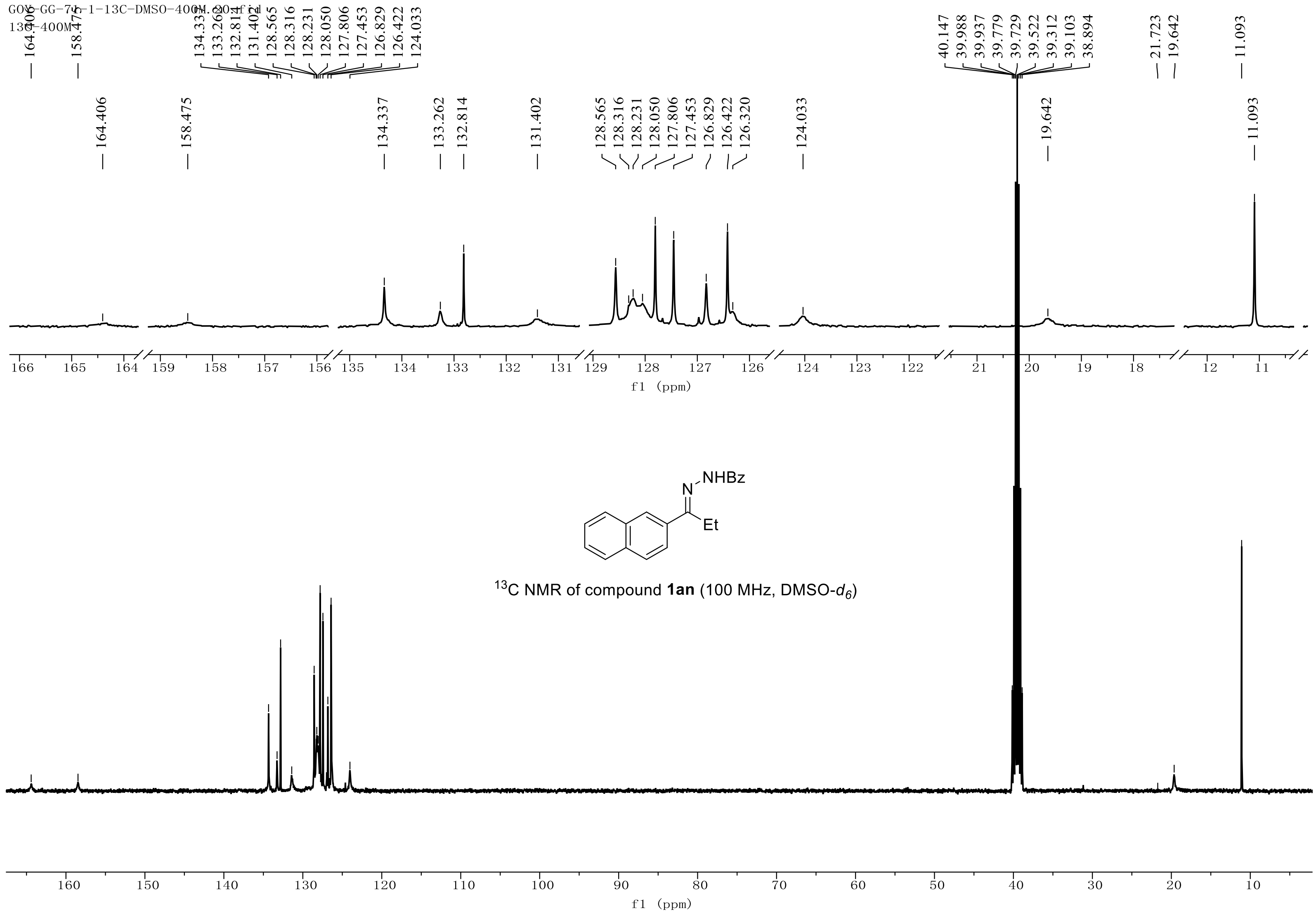

^{13}C NMR of compound **1an** (100 MHz, DMSO-d_6)

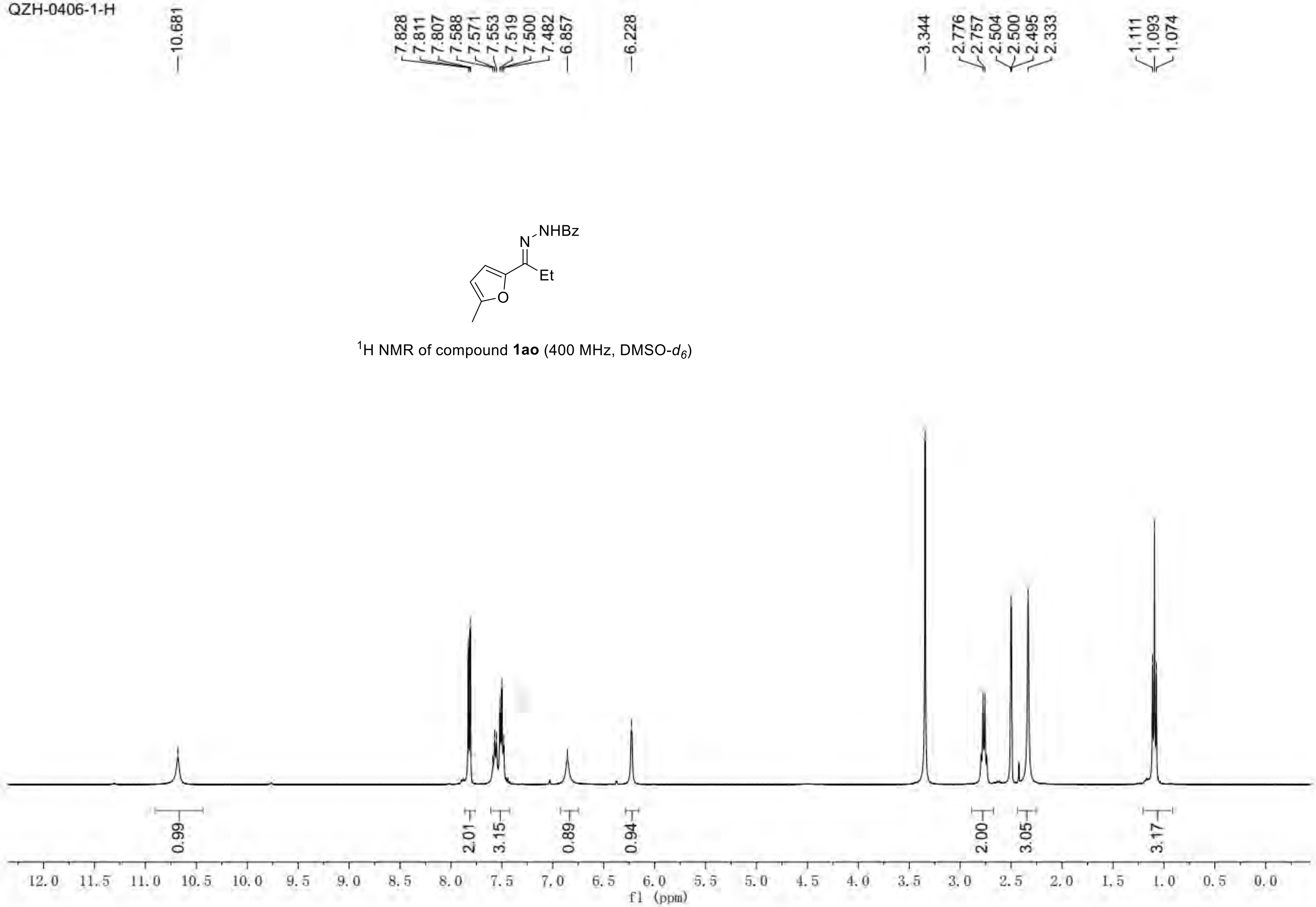

^{1}H NMR of compound **1ao** (400 MHz, DMSO-d_6)

QZH-0406-1-C

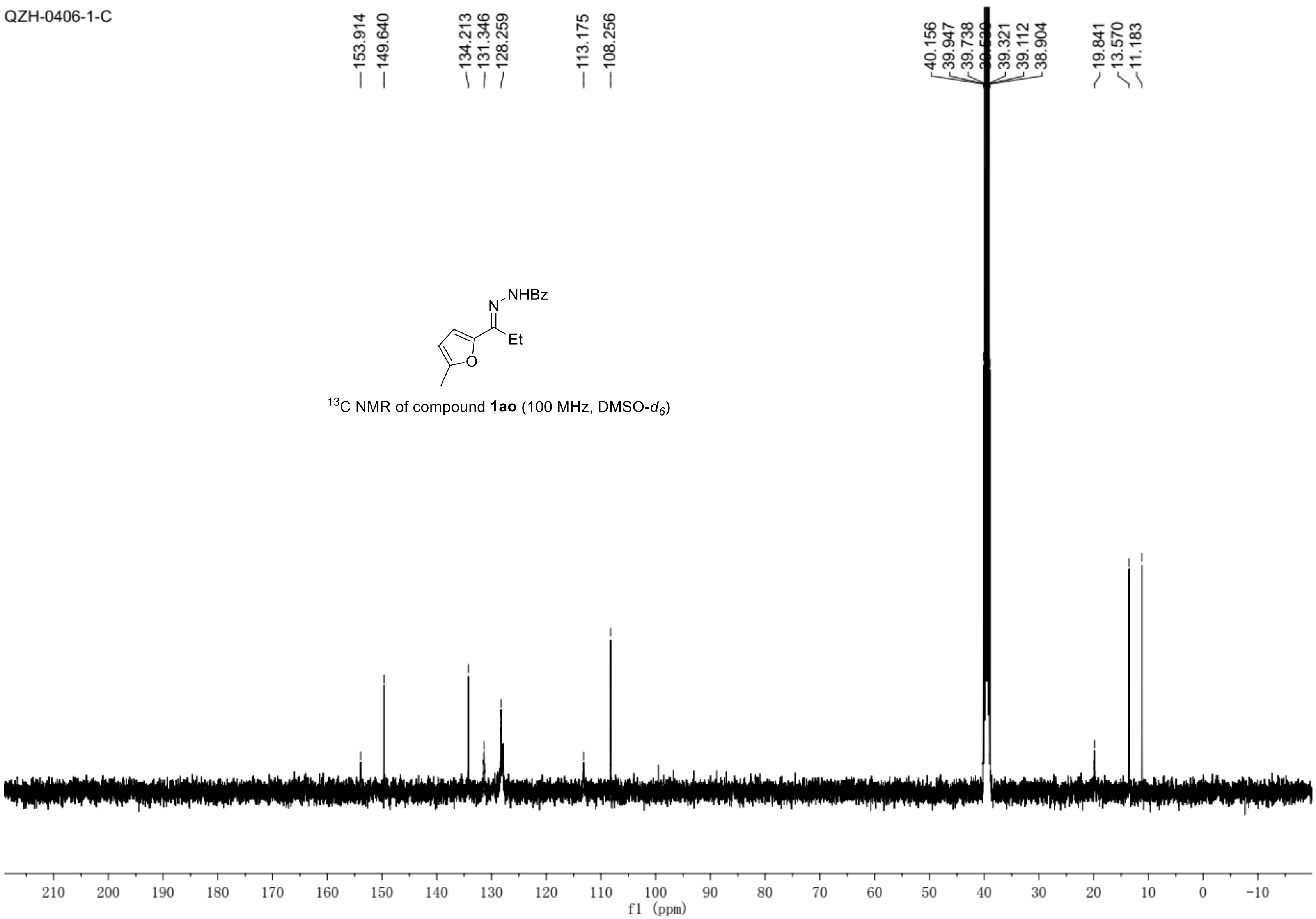

^{13}C NMR of compound **1ao** (100 MHz, DMSO-d_6)

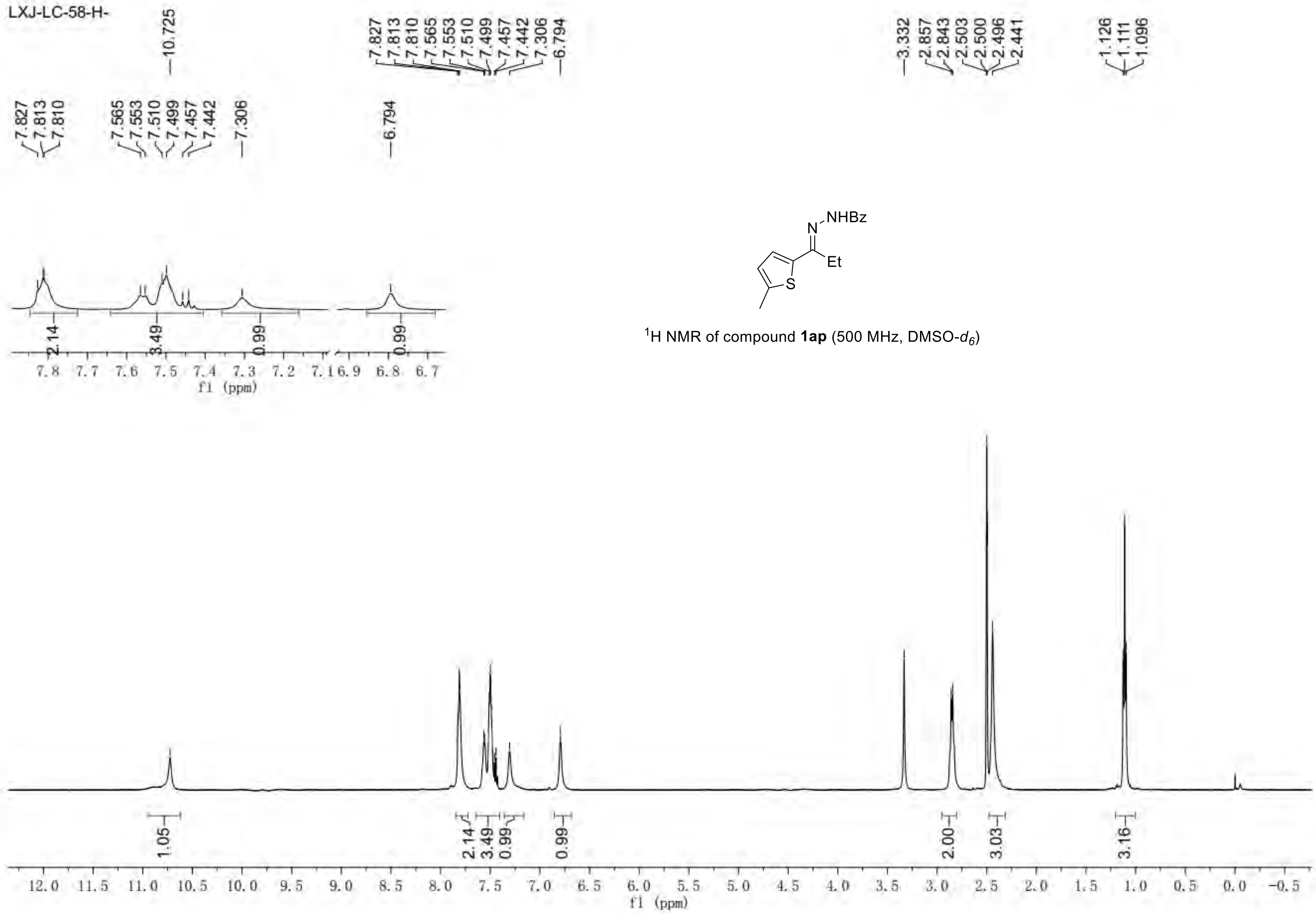

^{1}H NMR of compound **1ap** (500 MHz, DMSO-d_6)

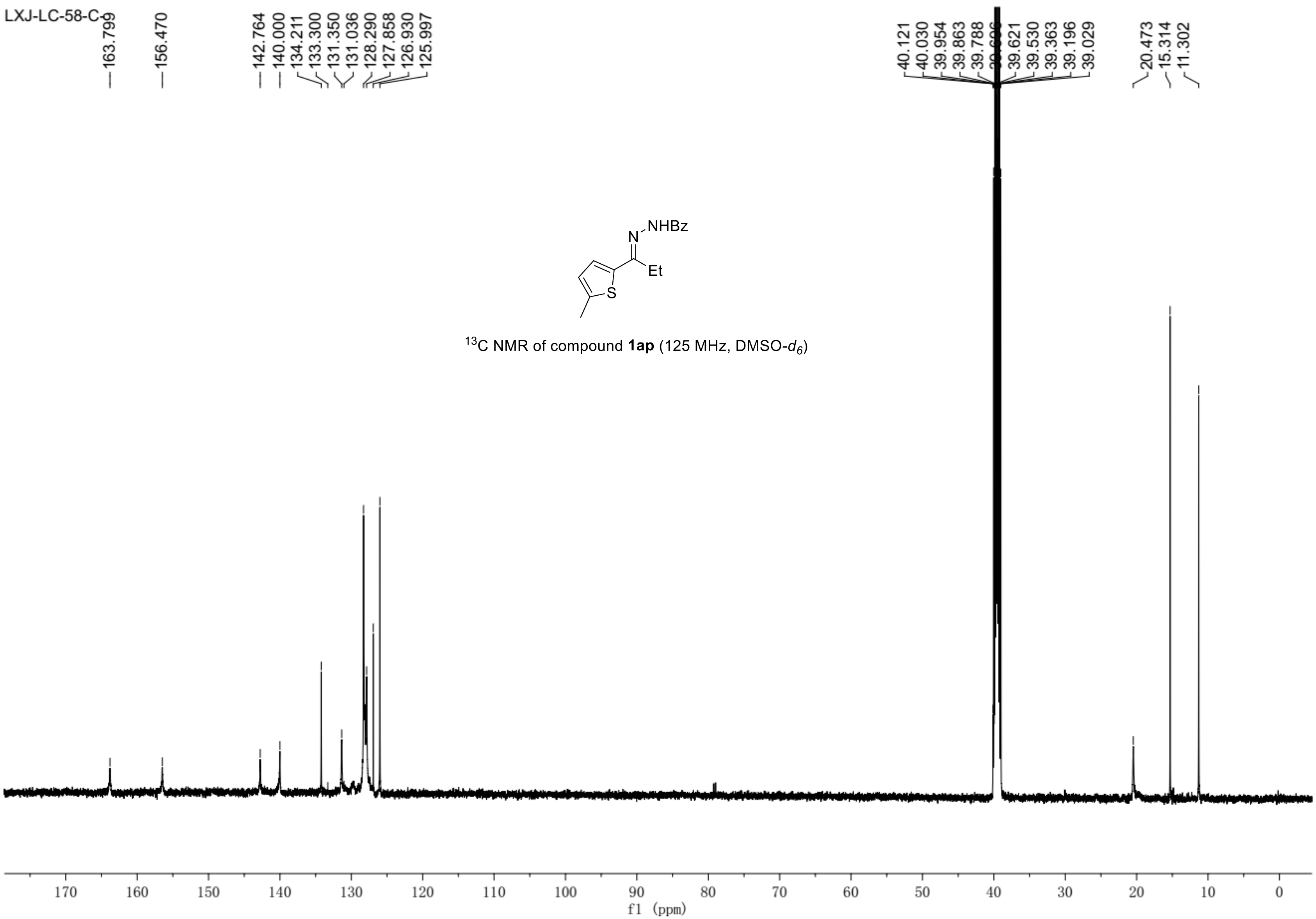

^{13}C NMR of compound **1ap** (125 MHz, DMSO-d_6)

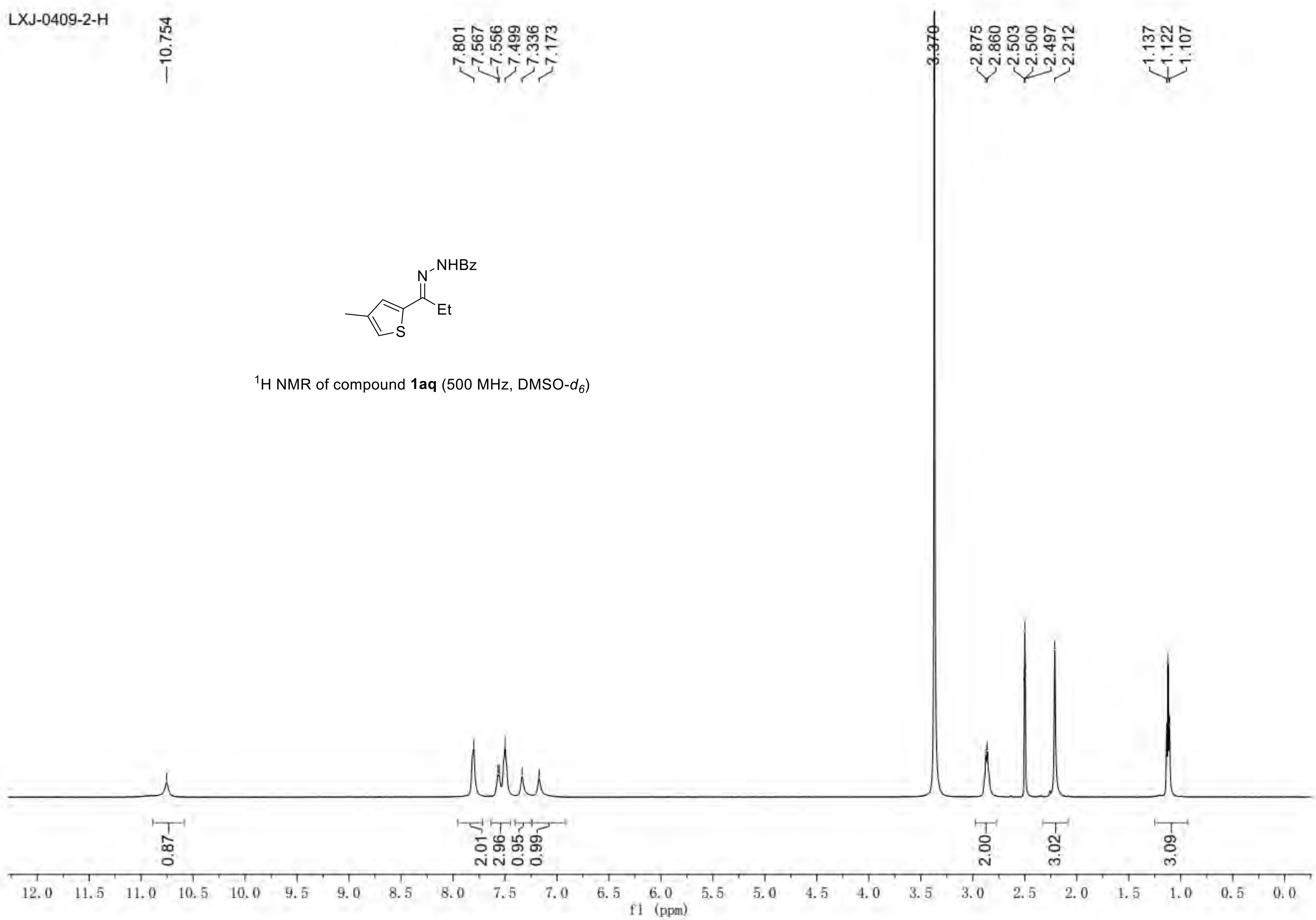

^{1}H NMR of compound **1aq** (500 MHz, DMSO-d_6)

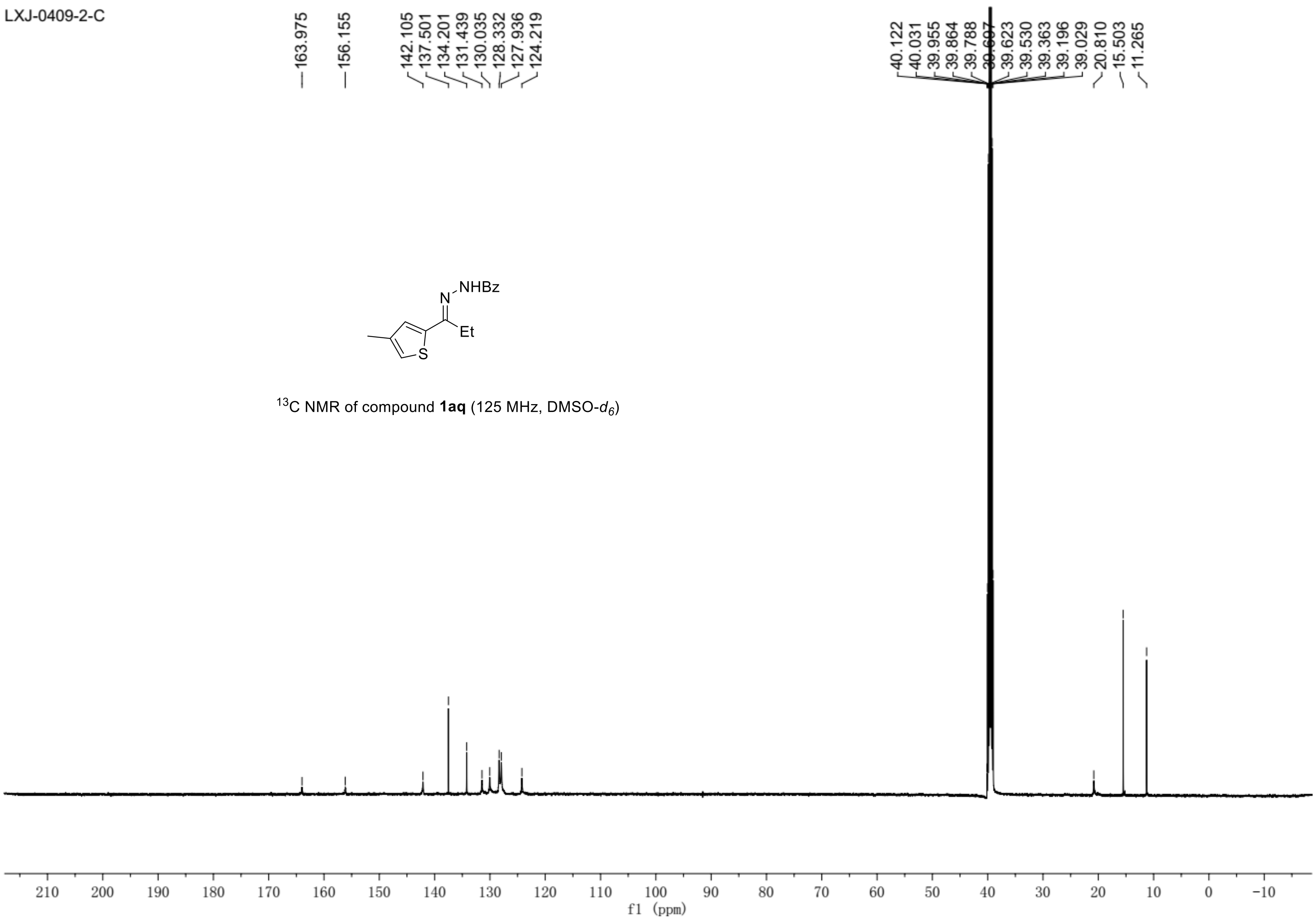

^{13}C NMR of compound **1aq** (125 MHz, DMSO-d_6)

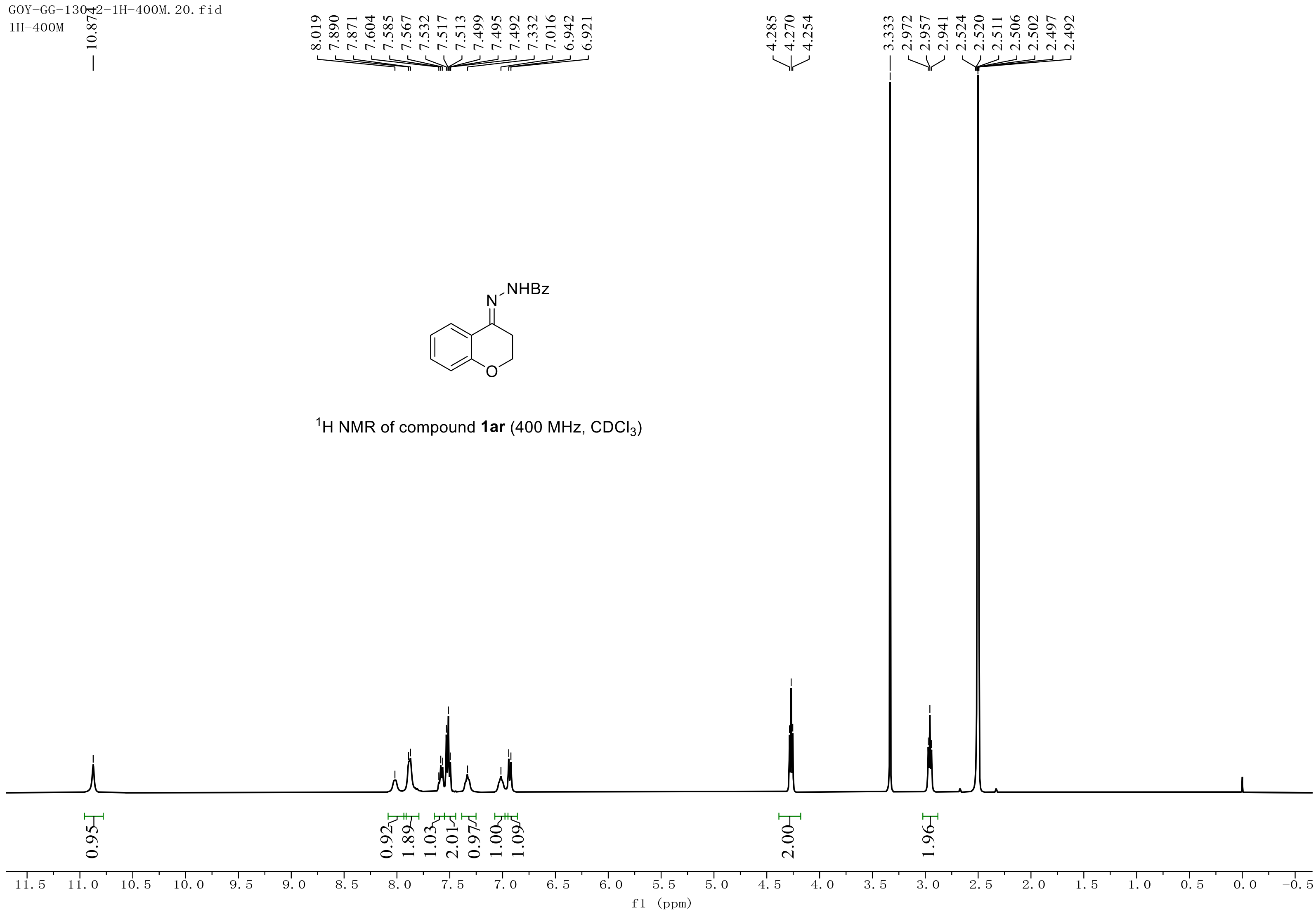

^{1}H NMR of compound **1ar** (400 MHz, $CDCl_3$)

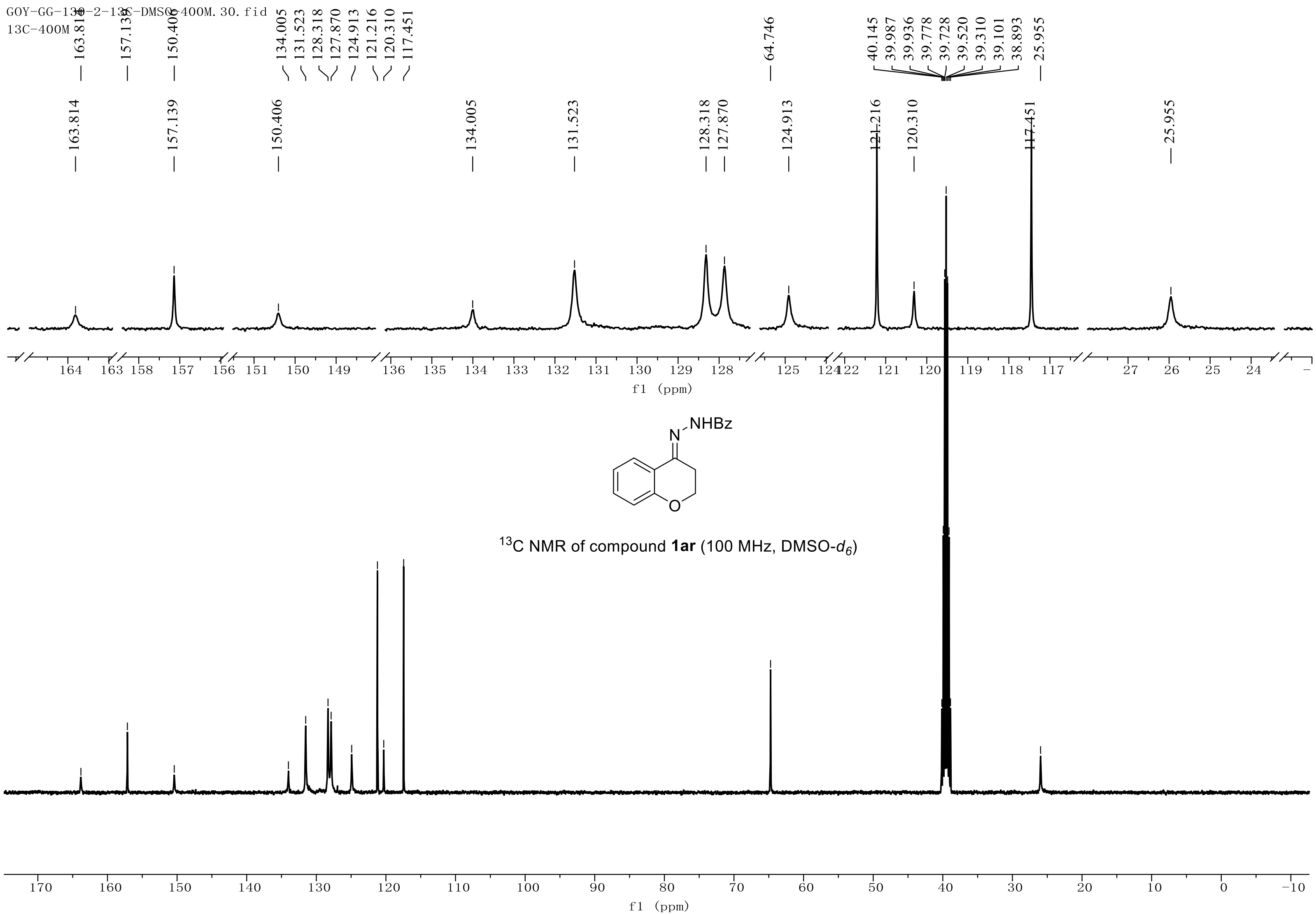

^{13}C NMR of compound **1ar** (100 MHz, DMSO-d_6)

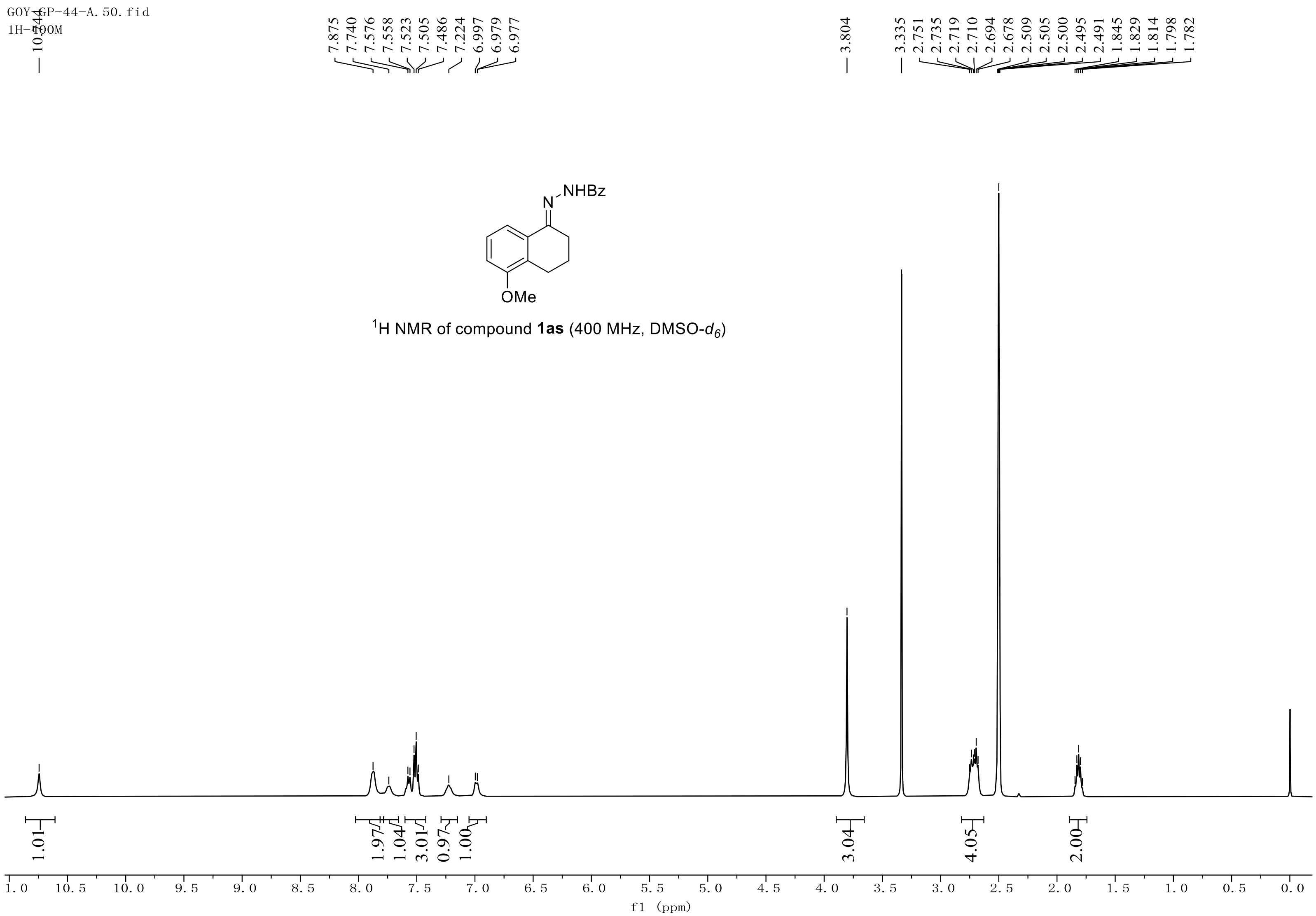

^{1}H NMR of compound **1as** (400 MHz, DMSO-d_6)

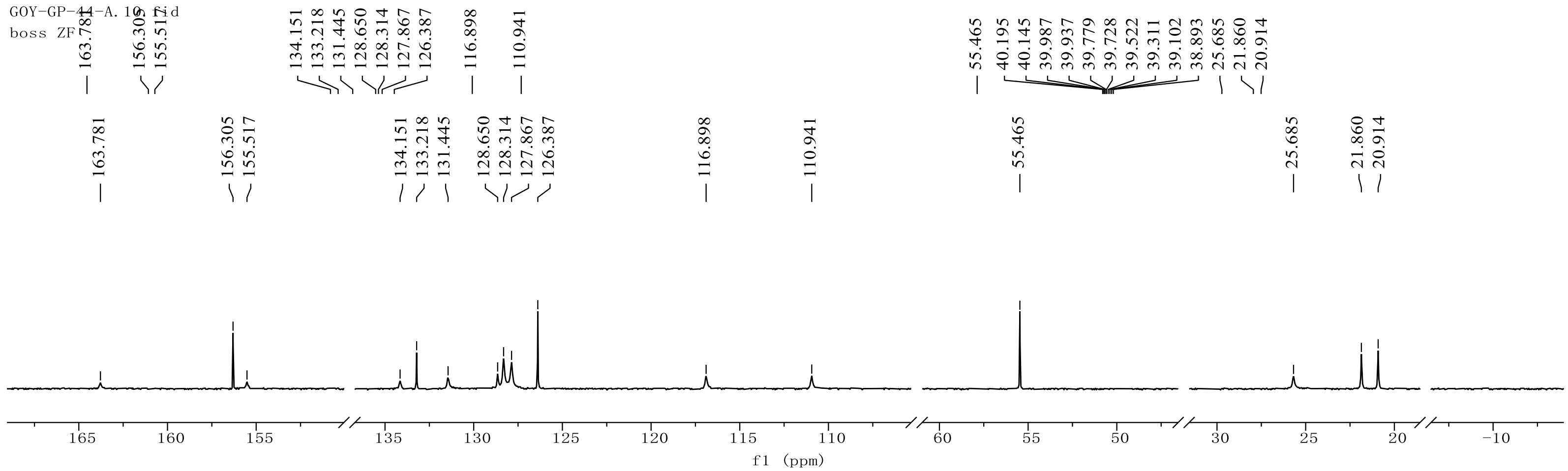

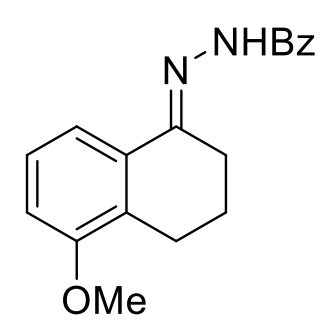

^{13}C NMR of compound **1as** (100 MHz, DMSO-d_6)

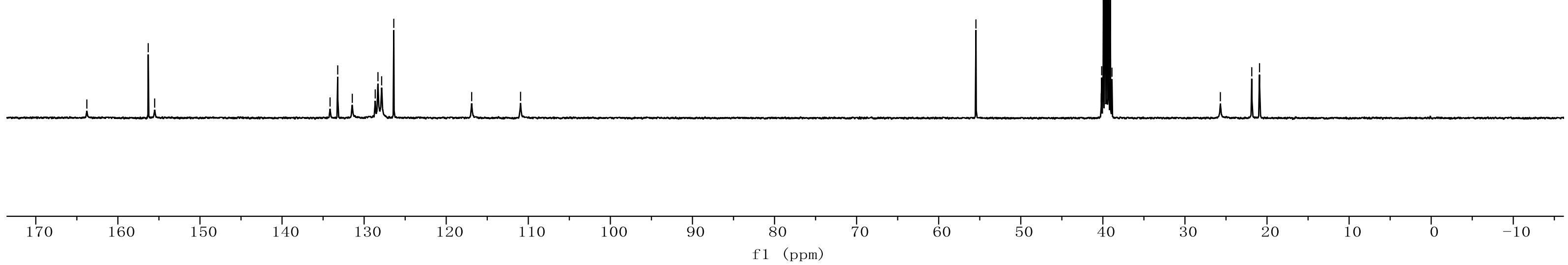

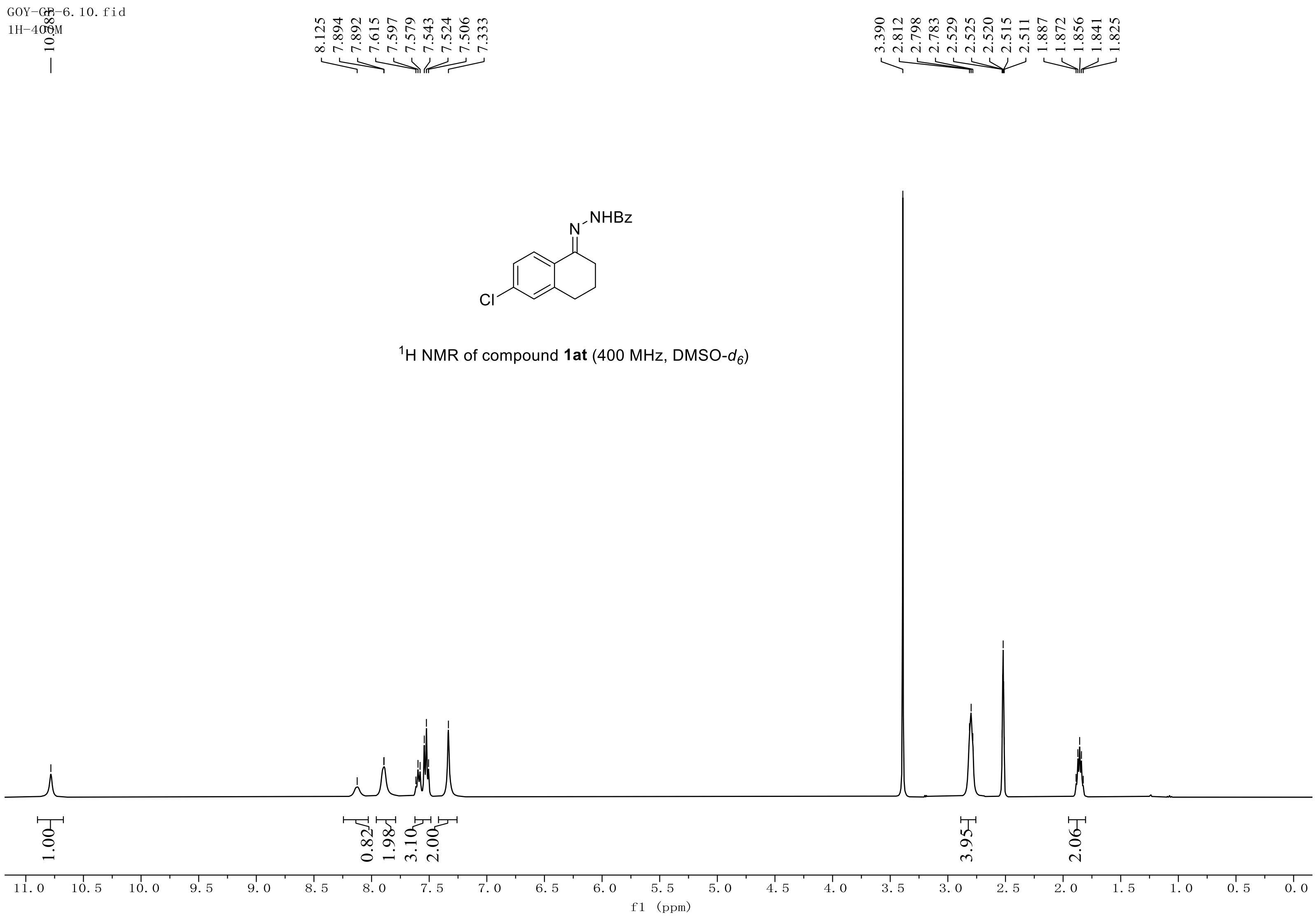

^{1}H NMR of compound **1at** (400 MHz, DMSO-d_6)

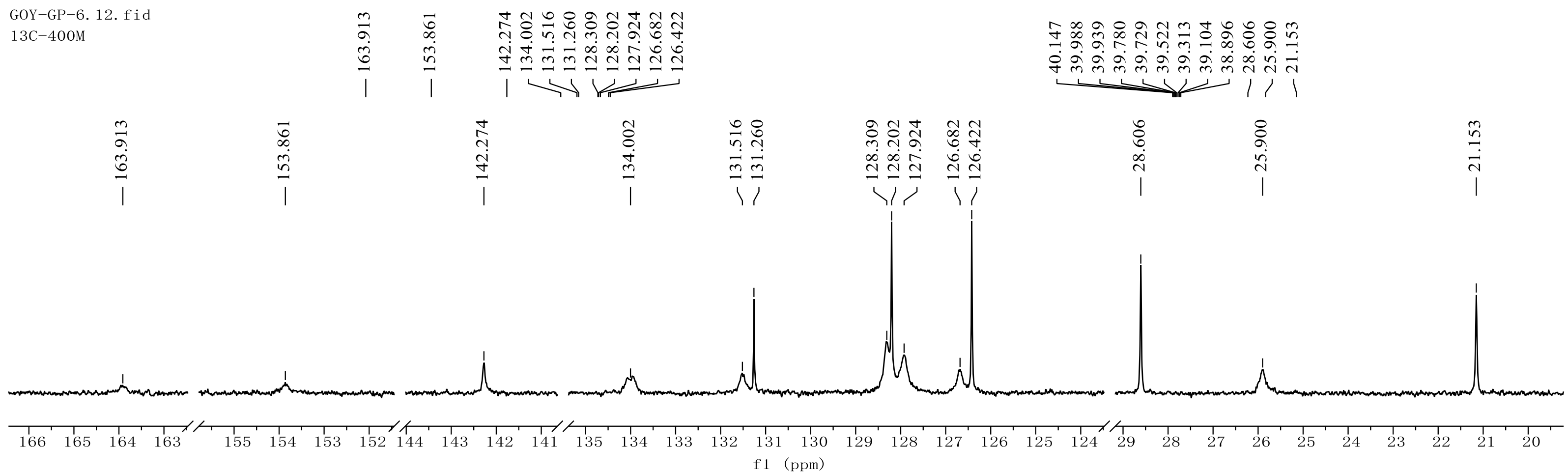

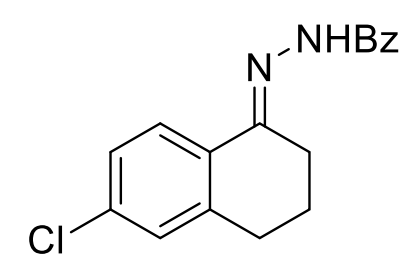

^{13}C NMR of compound **1at** (100 MHz, DMSO-d_6)

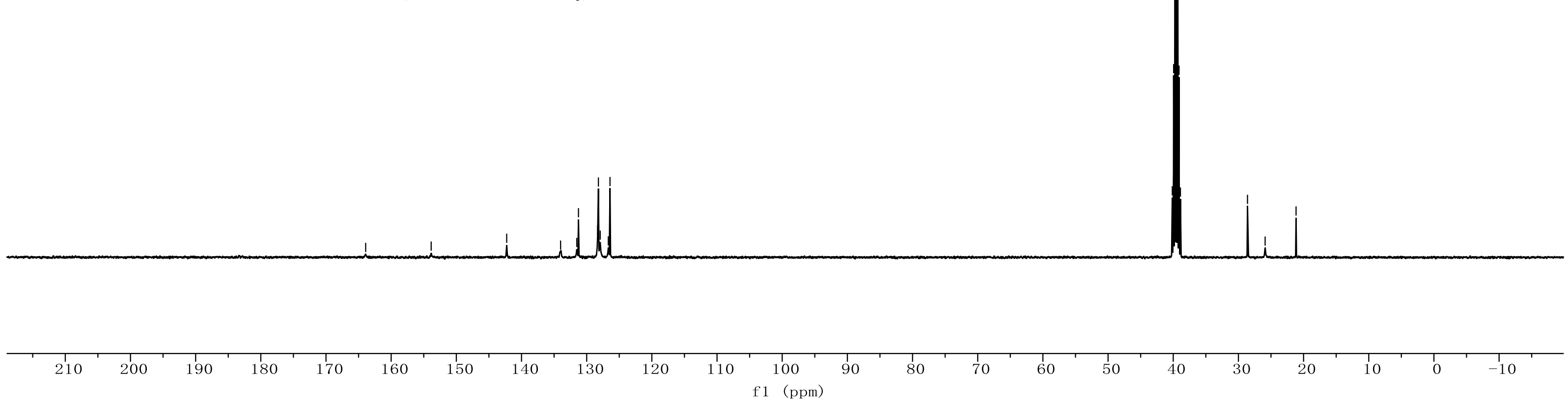

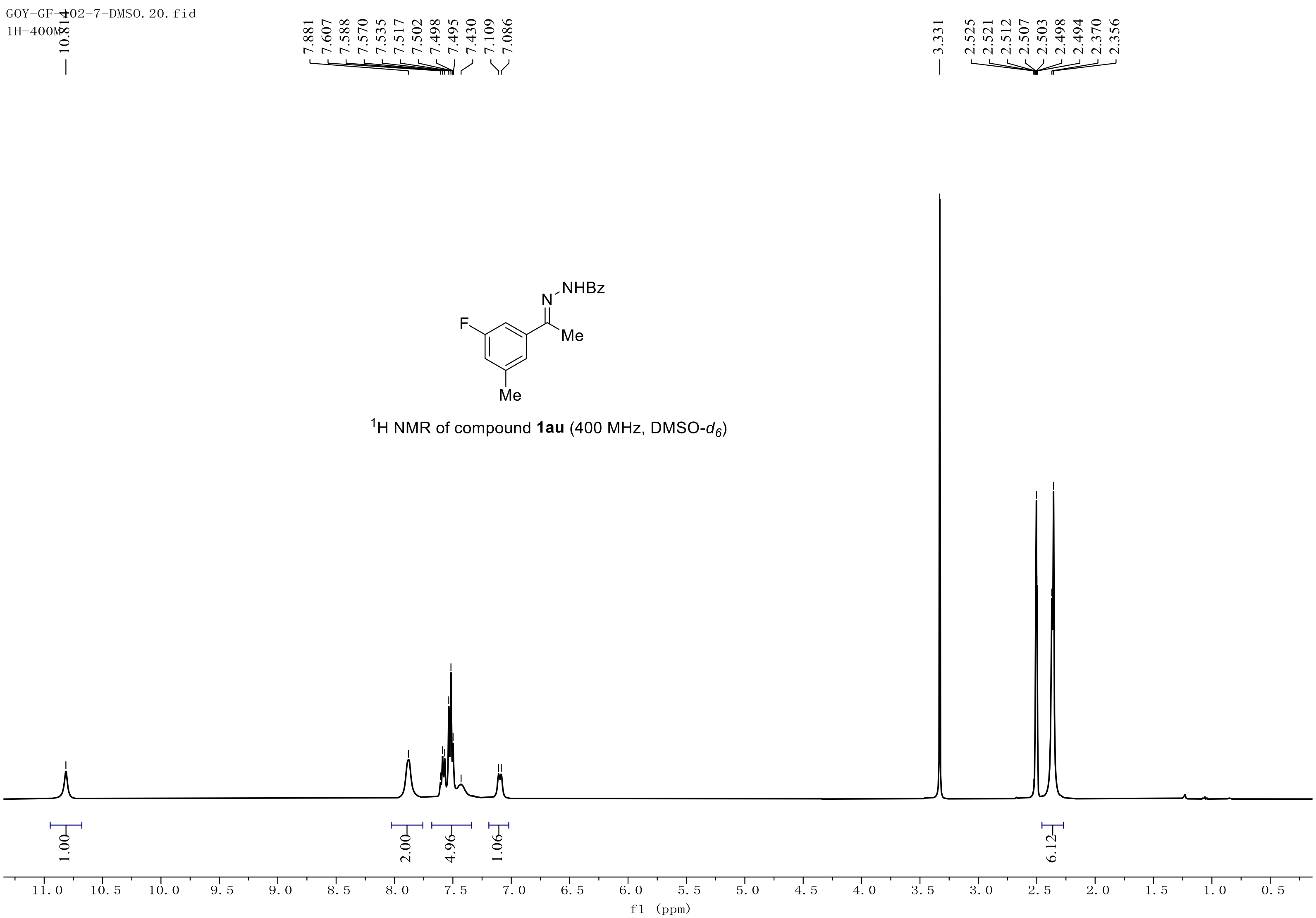

^{1}H NMR of compound **1au** (400 MHz, DMSO-d_6)

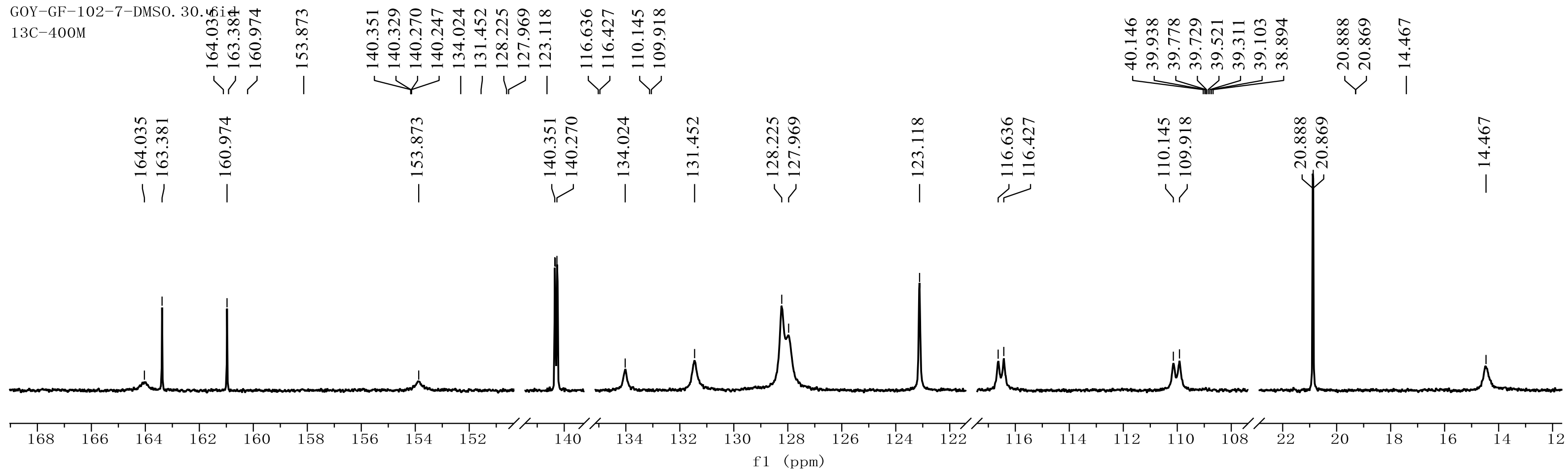

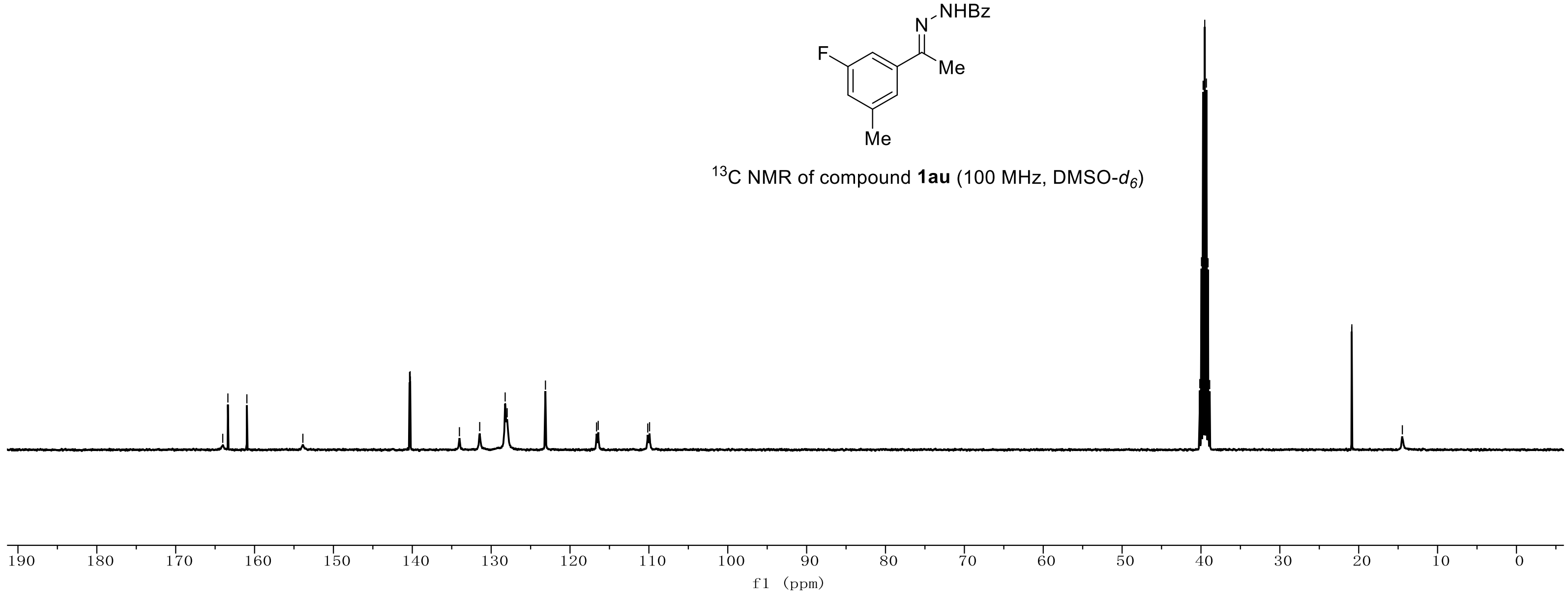

^{13}C NMR of compound **1au** (100 MHz, DMSO-d_6)

GOY-GF-102-7-DMSO.10.fid
19F-400M

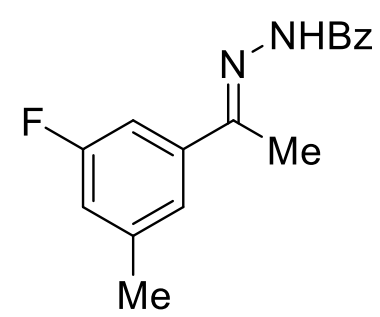

^{19}F NMR of compound **1au** (376 MHz,DMSO-d_6)

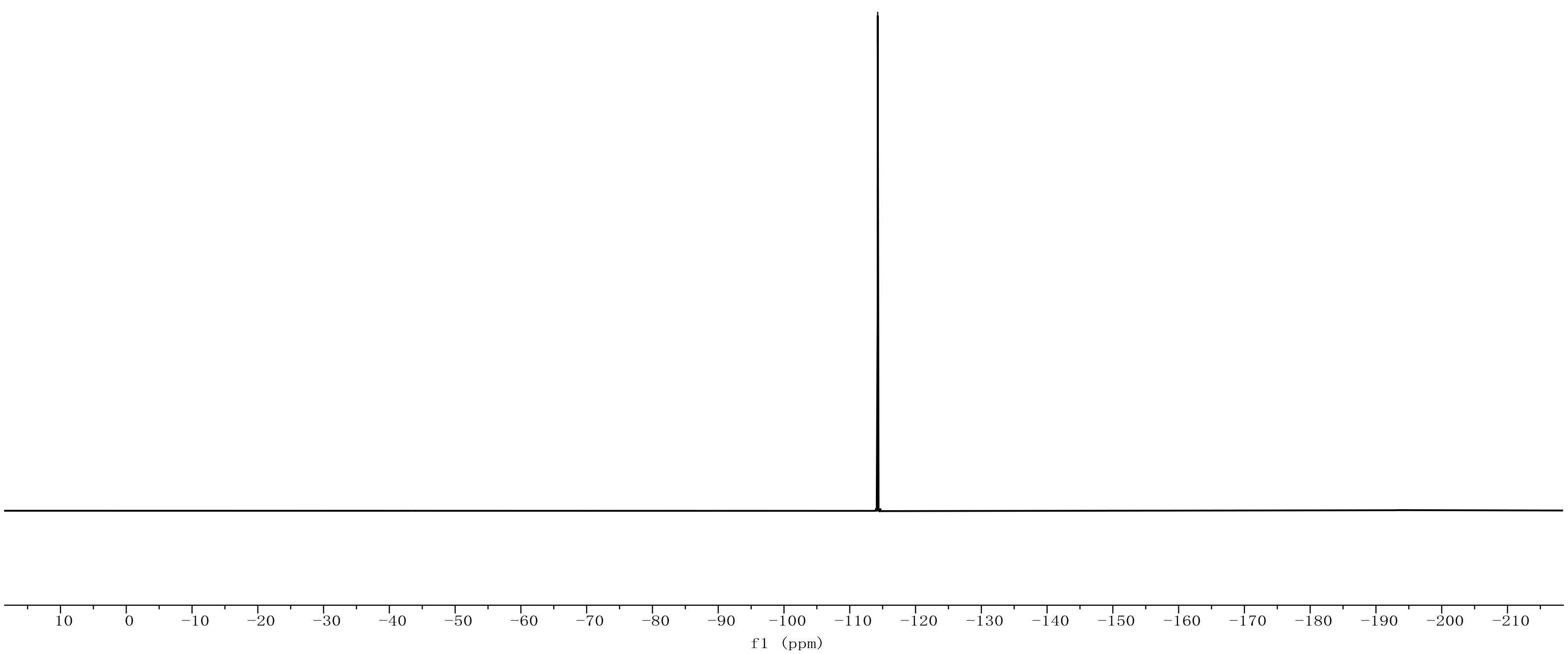

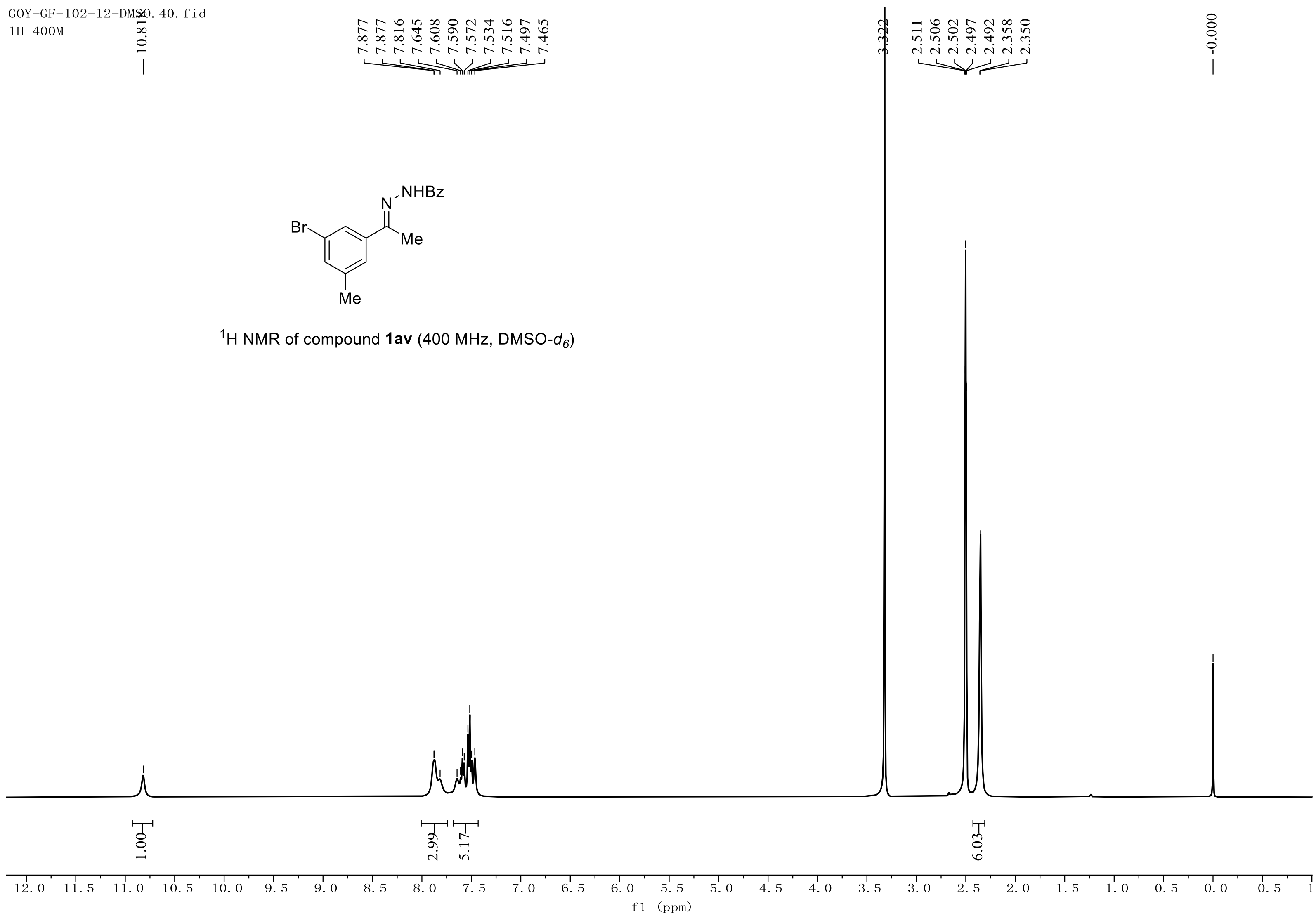

^{1}H NMR of compound **1av** (400 MHz, DMSO-d_6)

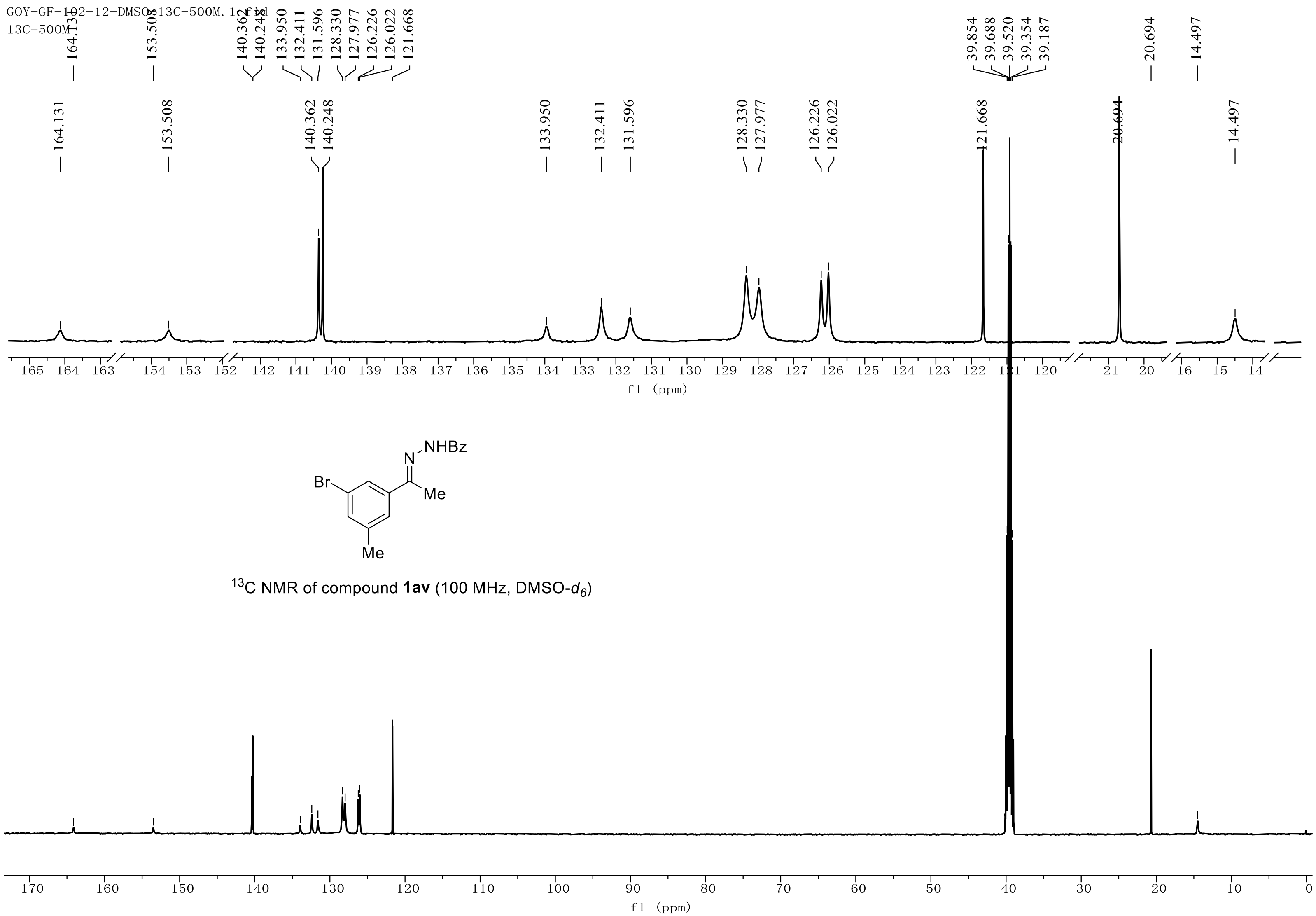

^{13}C NMR of compound **1av** (100 MHz, DMSO-d_6)

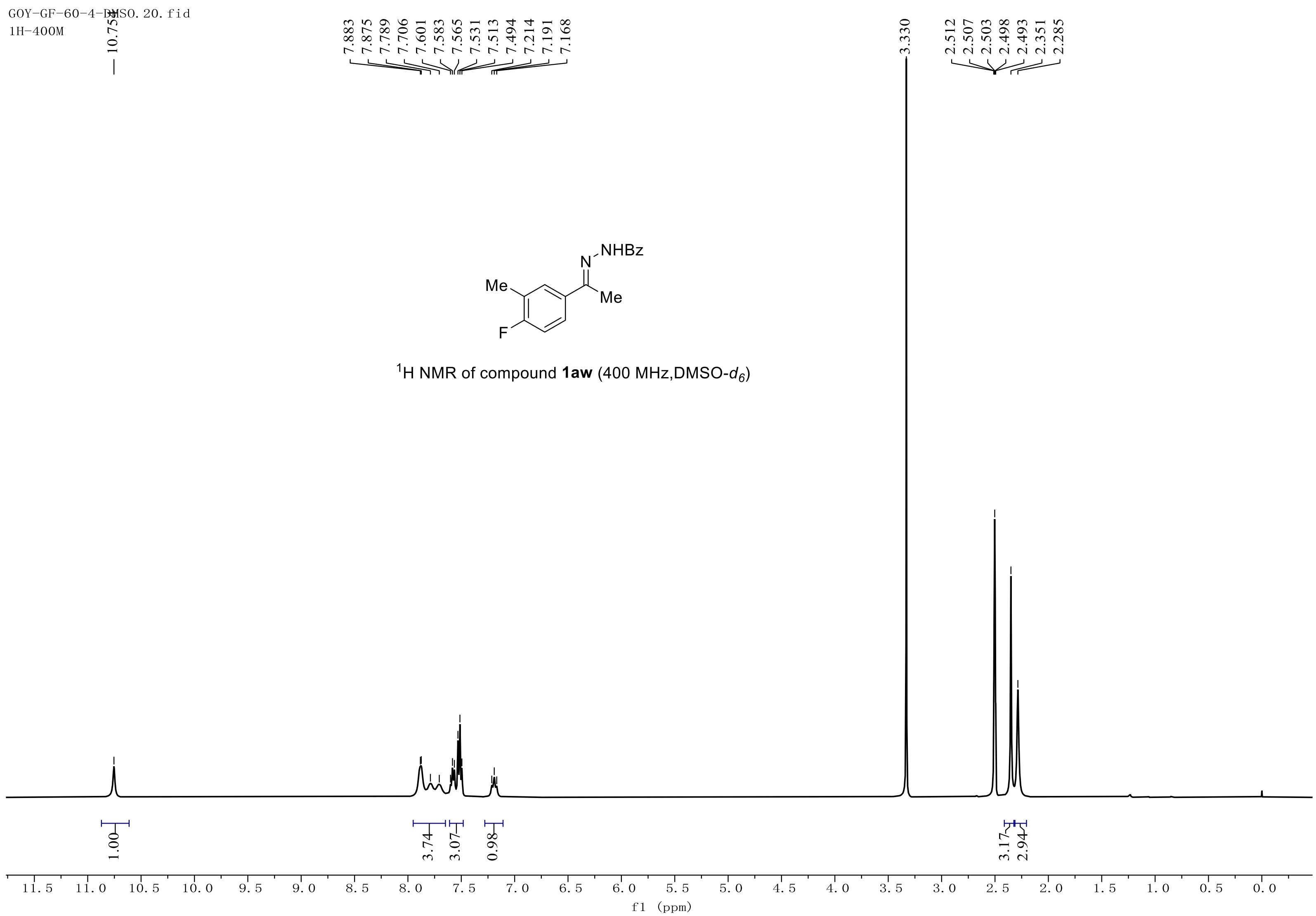

^{1}H NMR of compound **1aw** (400 MHz,DMSO-d_6)

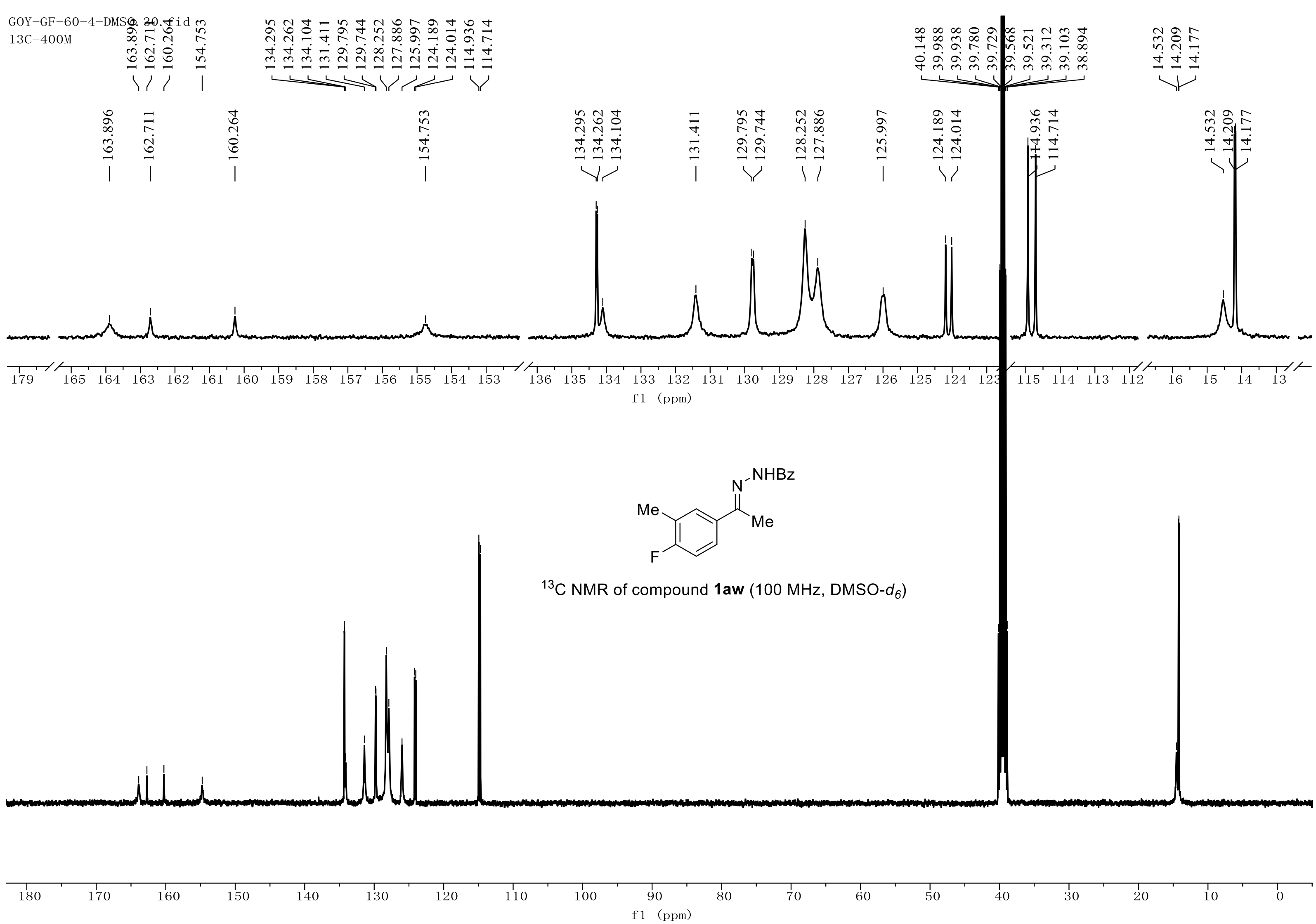

^{13}C NMR of compound **1aw** (100 MHz, DMSO-d_6)

GOY-GF-60-4-DMSO.22.fid
19F-400M

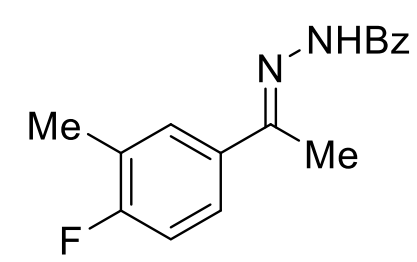

^{19}F NMR of compound **1aw** (376 MHz, DMSO-d_6)

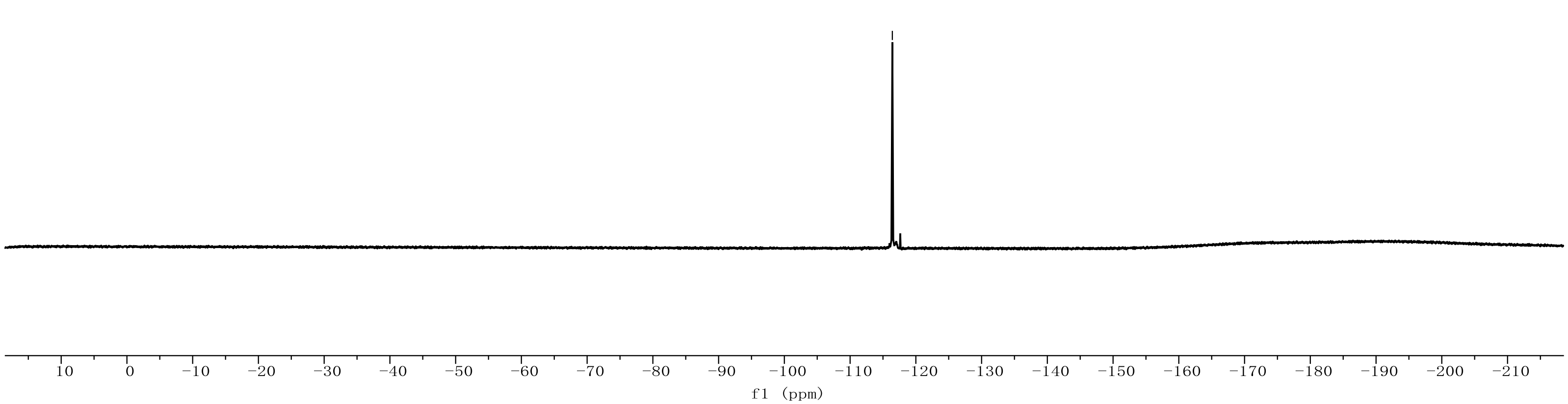

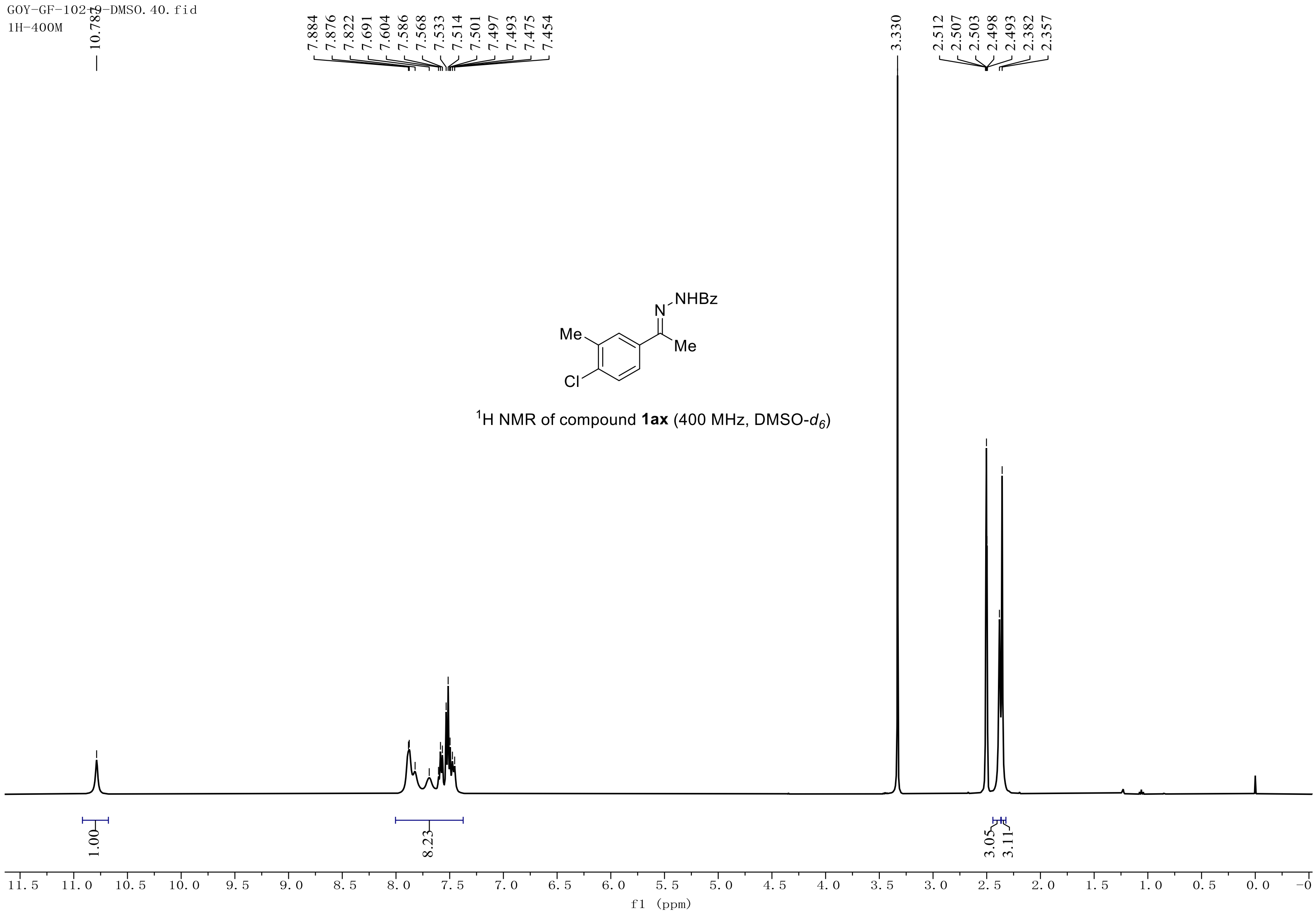

^{1}H NMR of compound **1ax** (400 MHz, DMSO-d_6)

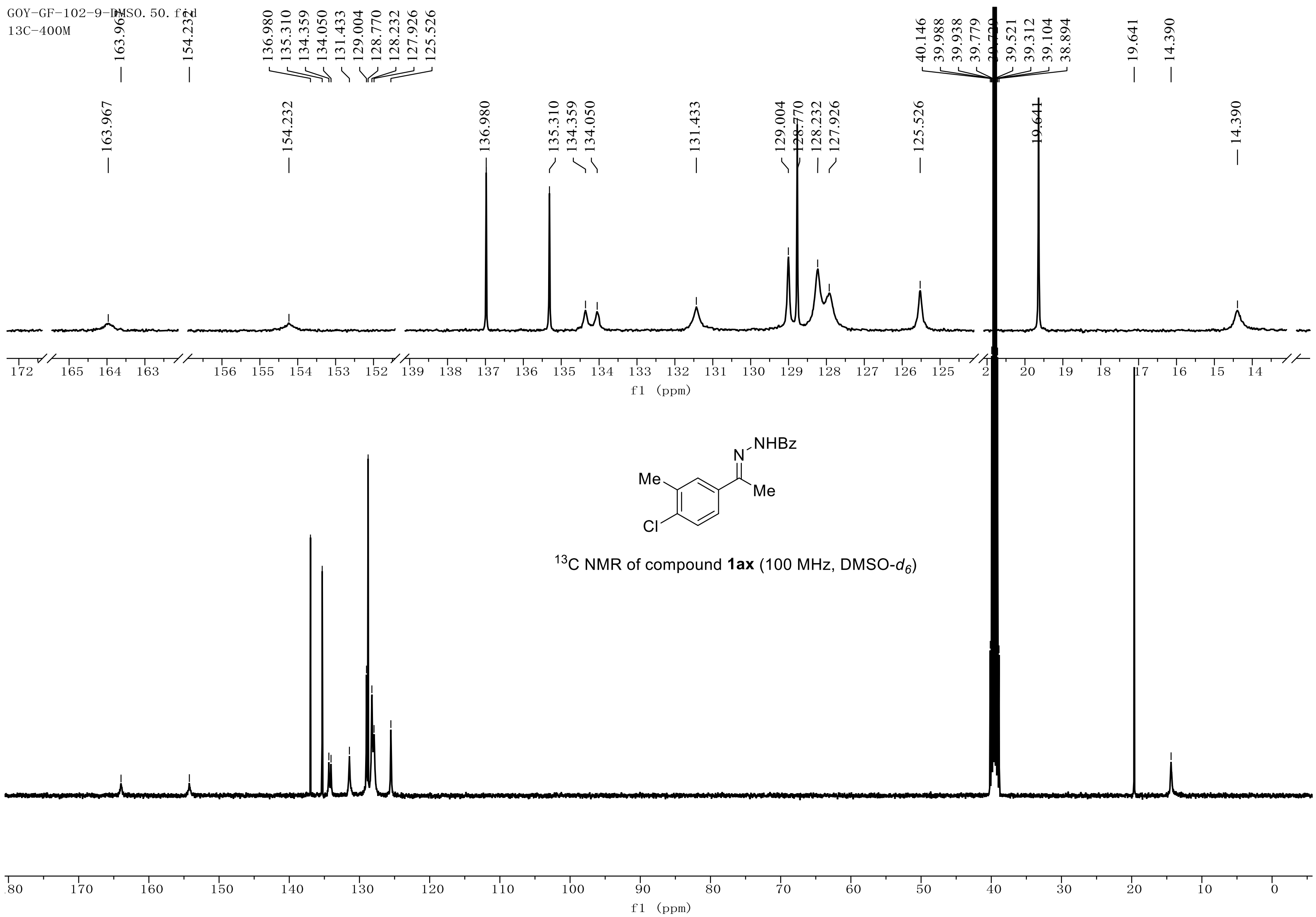

^{13}C NMR of compound **1ax** (100 MHz, DMSO-d_6)

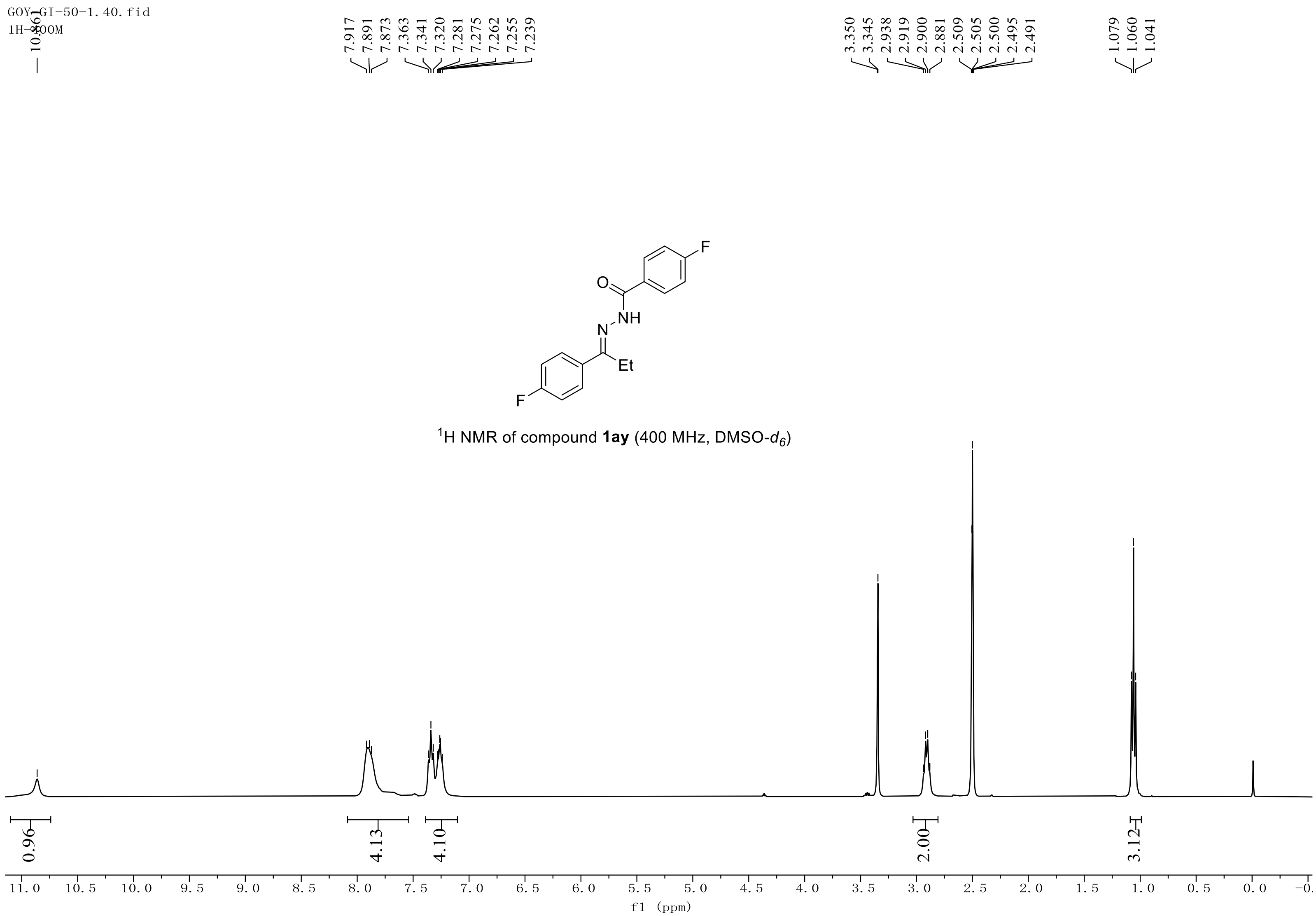

^{1}H NMR of compound **1ay** (400 MHz, DMSO-d_6)

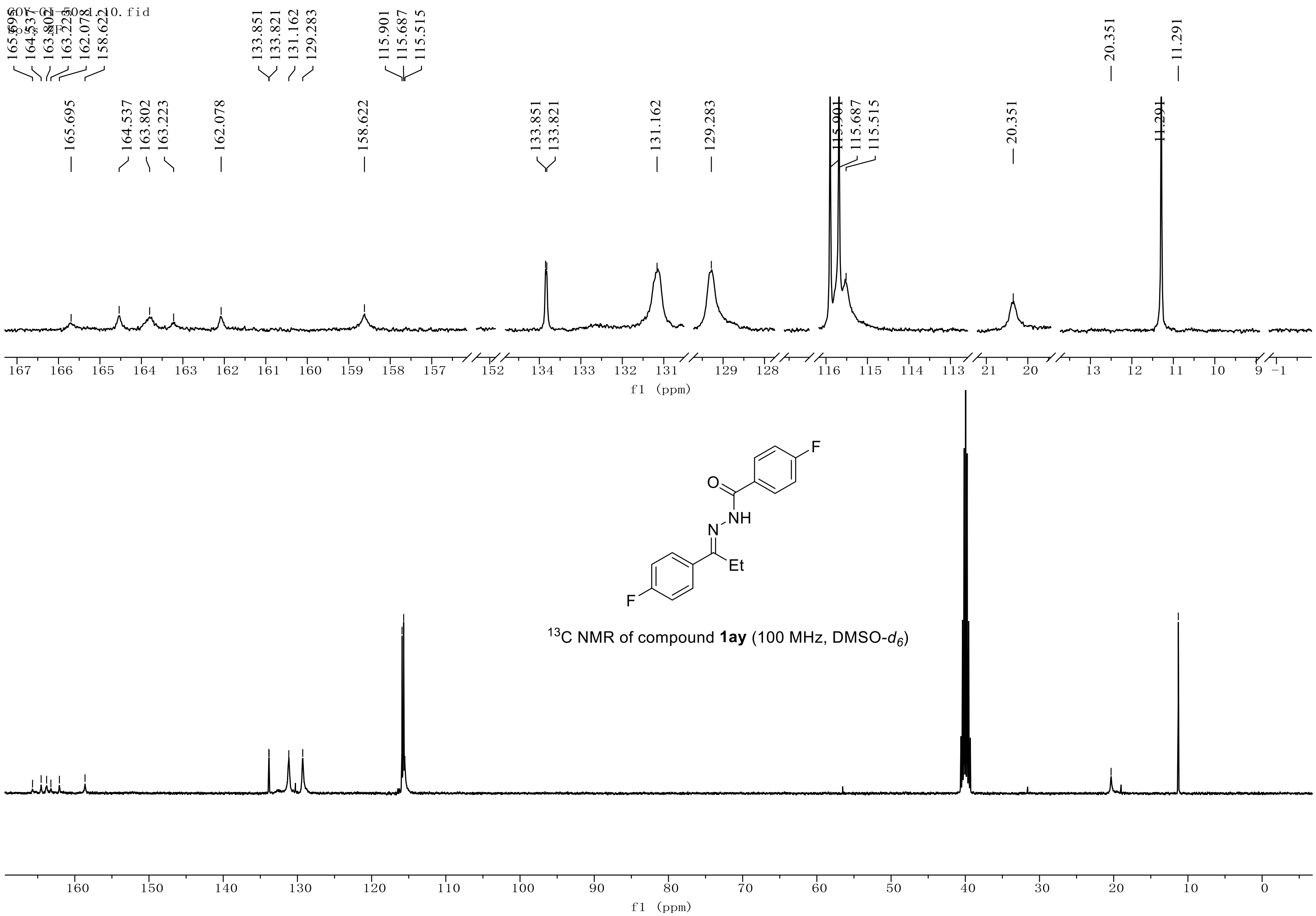

^{13}C NMR of compound **1ay** (100 MHz, DMSO-d_6)

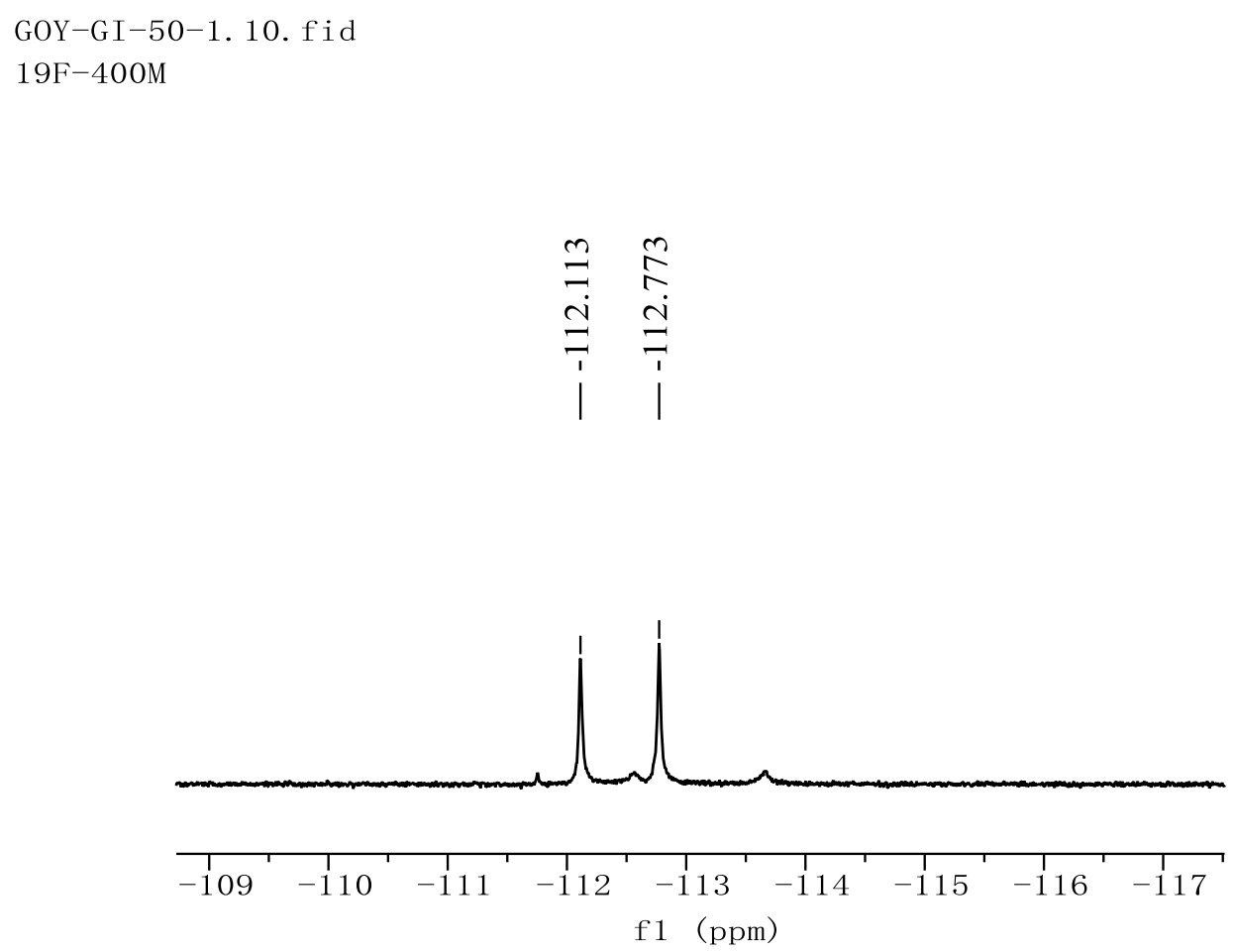

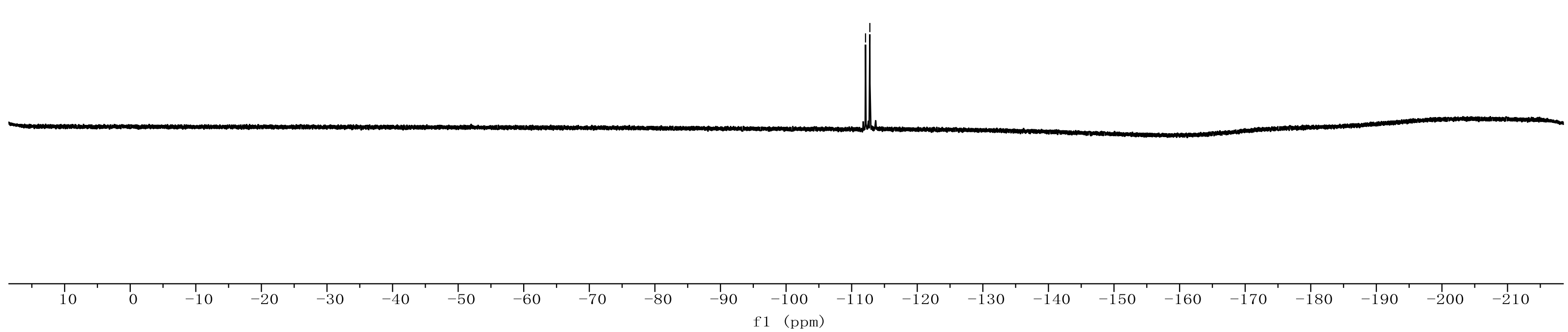

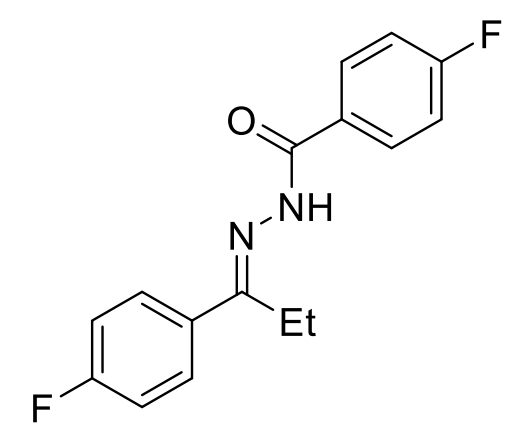

^{19}F NMR of compound **1ay** (100 MHz, DMSO-d_6)

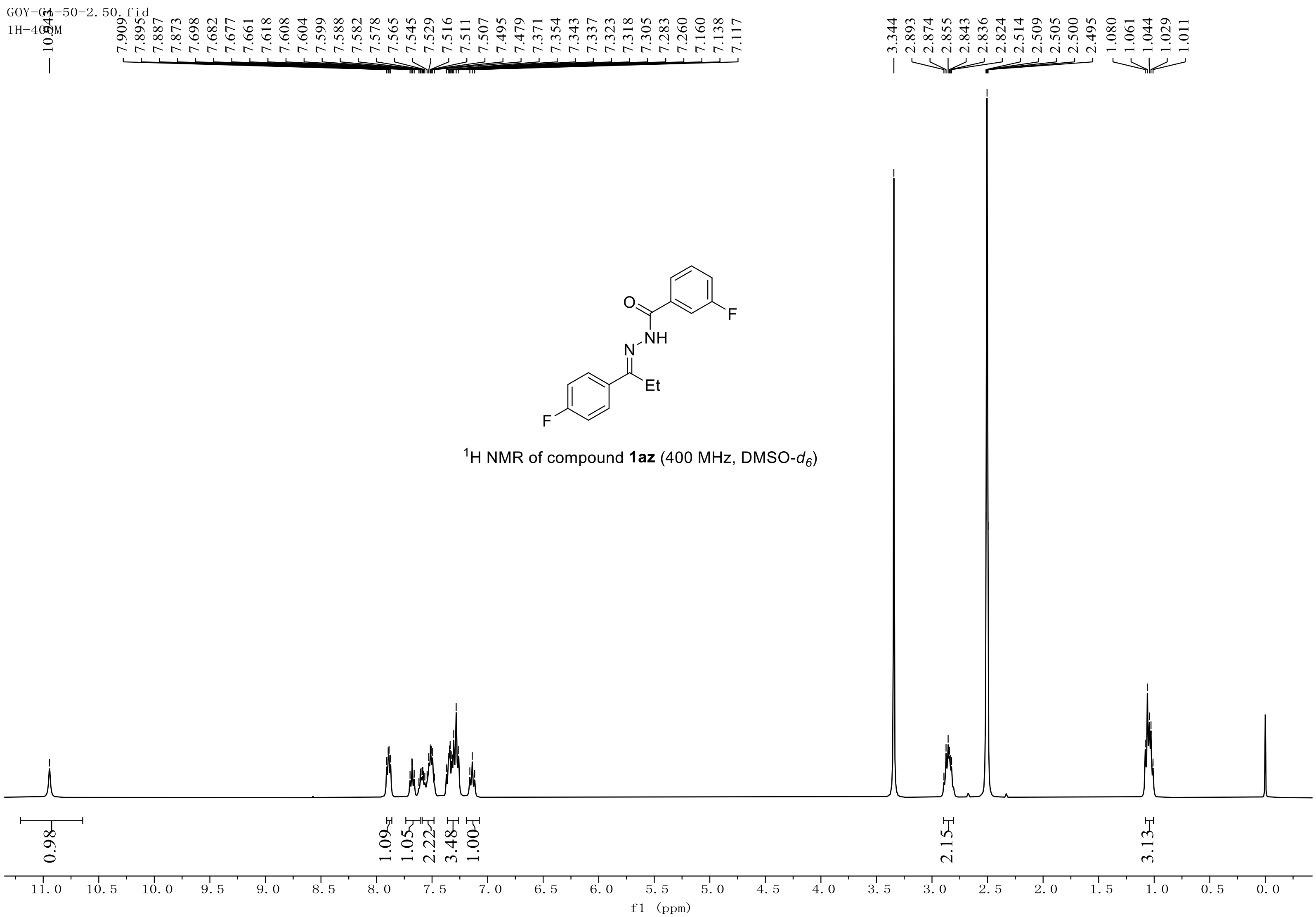

^{1}H NMR of compound **1az** (400 MHz, DMSO-d_6)

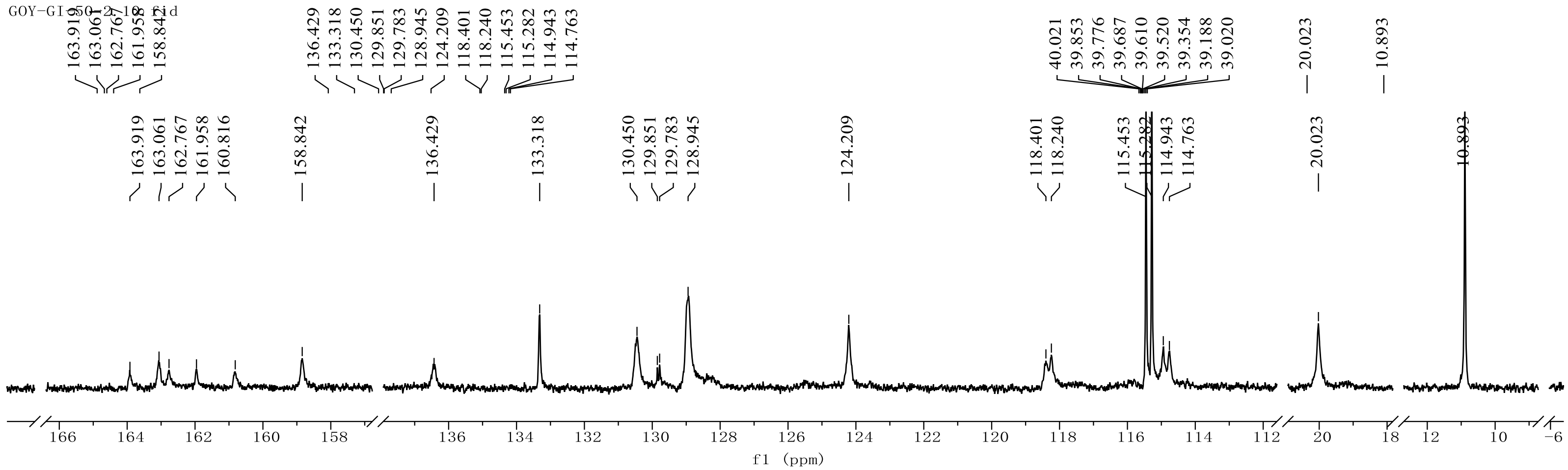

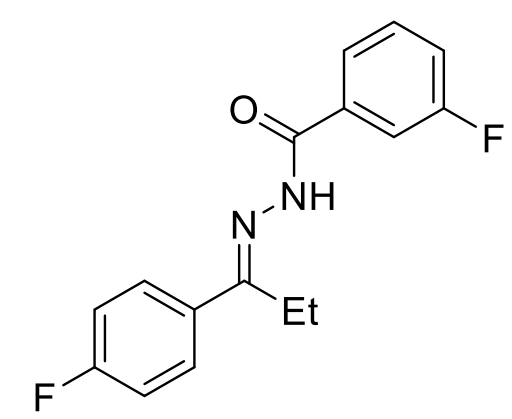

^{13}C NMR of compound **1az** (100 MHz, DMSO-d_6)

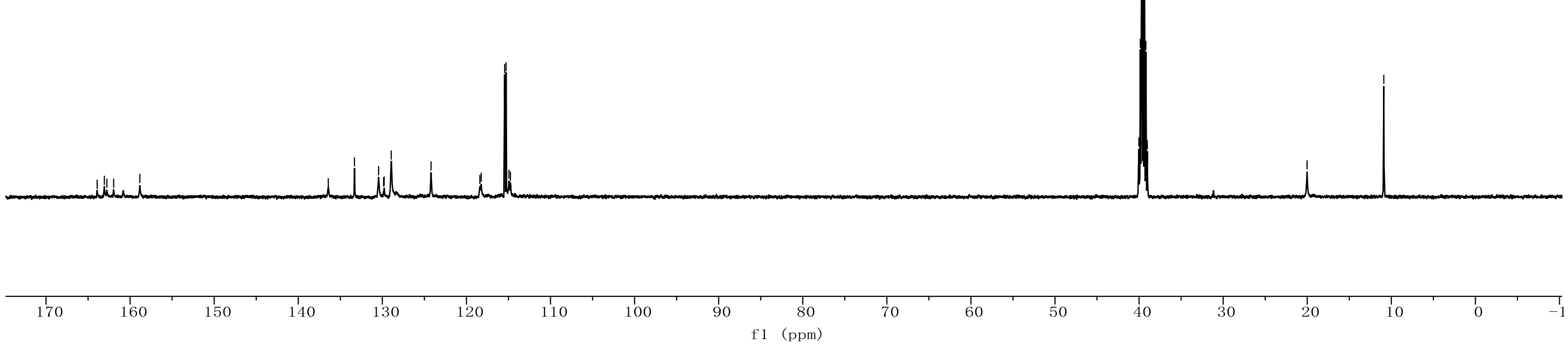

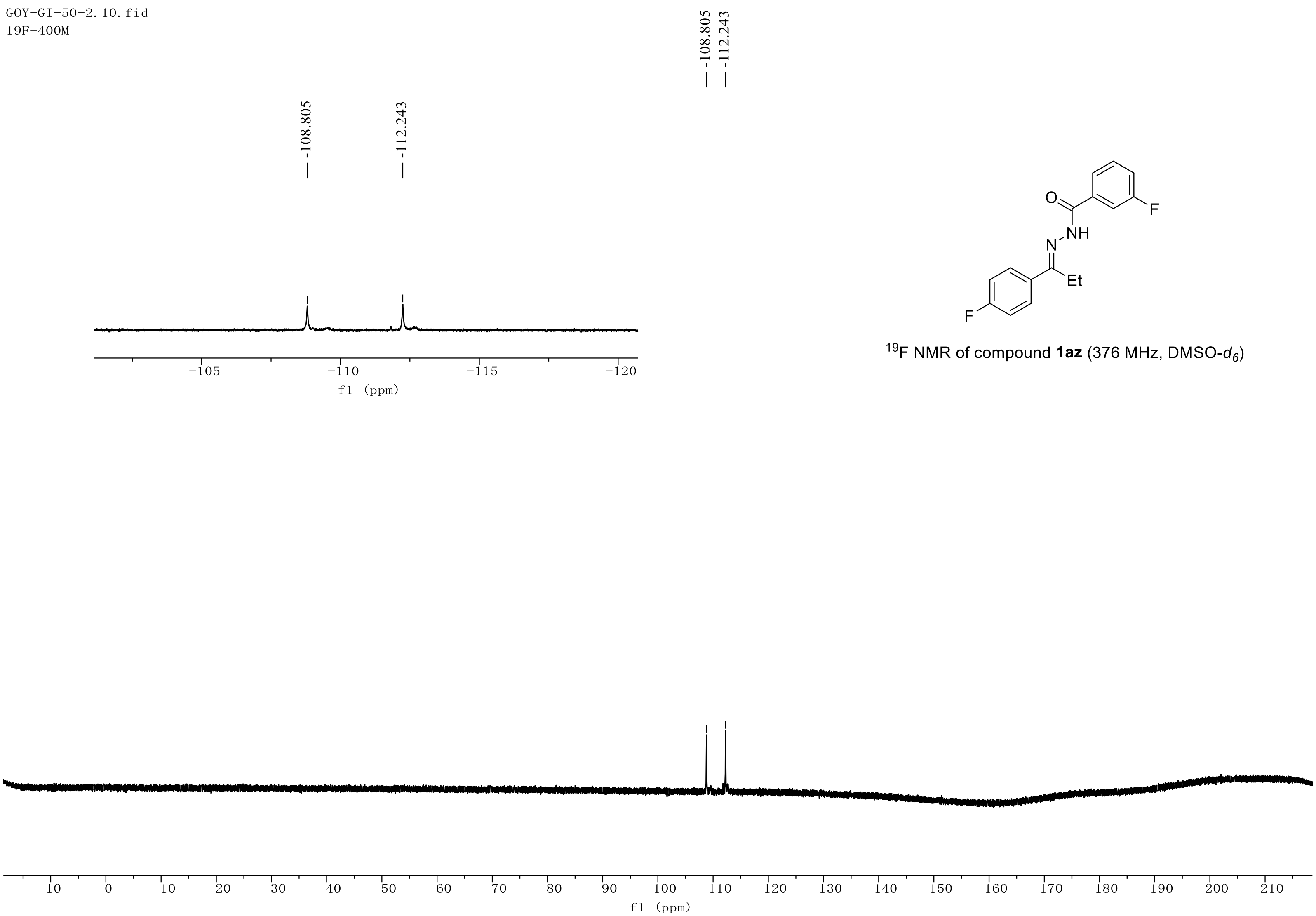

^{19}F NMR of compound **1az** (376 MHz, DMSO-d_6)

GOY-GE-142-1H 10.fid
1H-400M

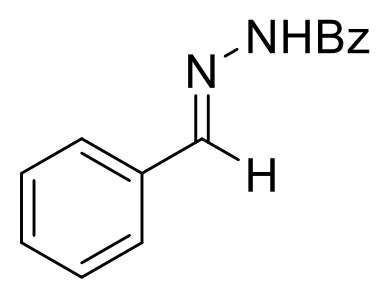

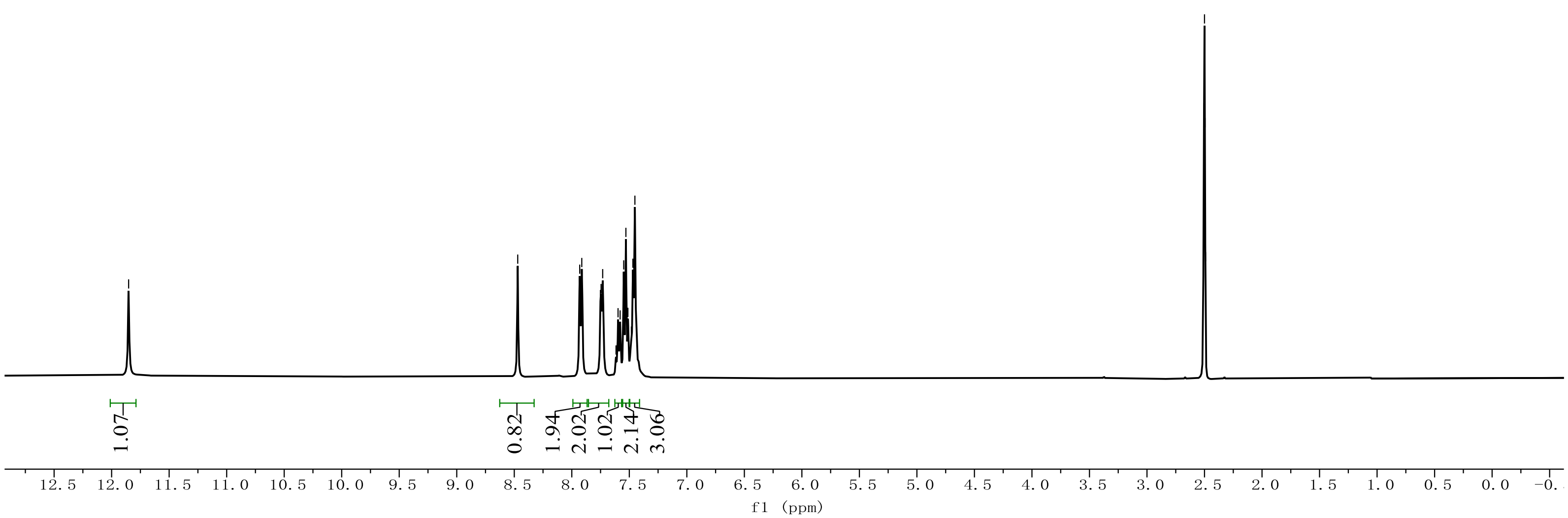

^{1}H NMR of compound **1ba** (400 MHz, DMSO-d_6)

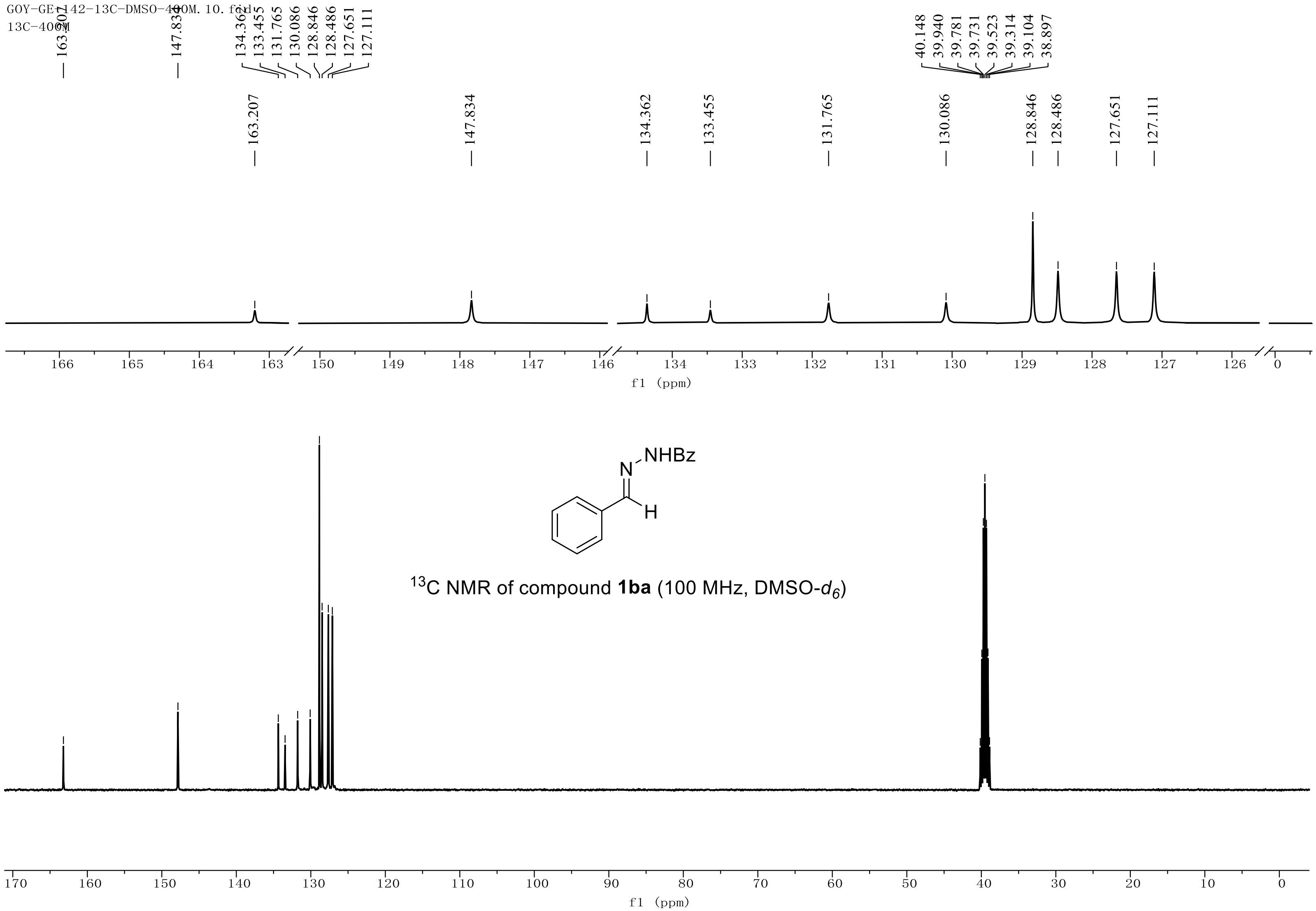

^{13}C NMR of compound **1ba** (100 MHz, DMSO-d_6)

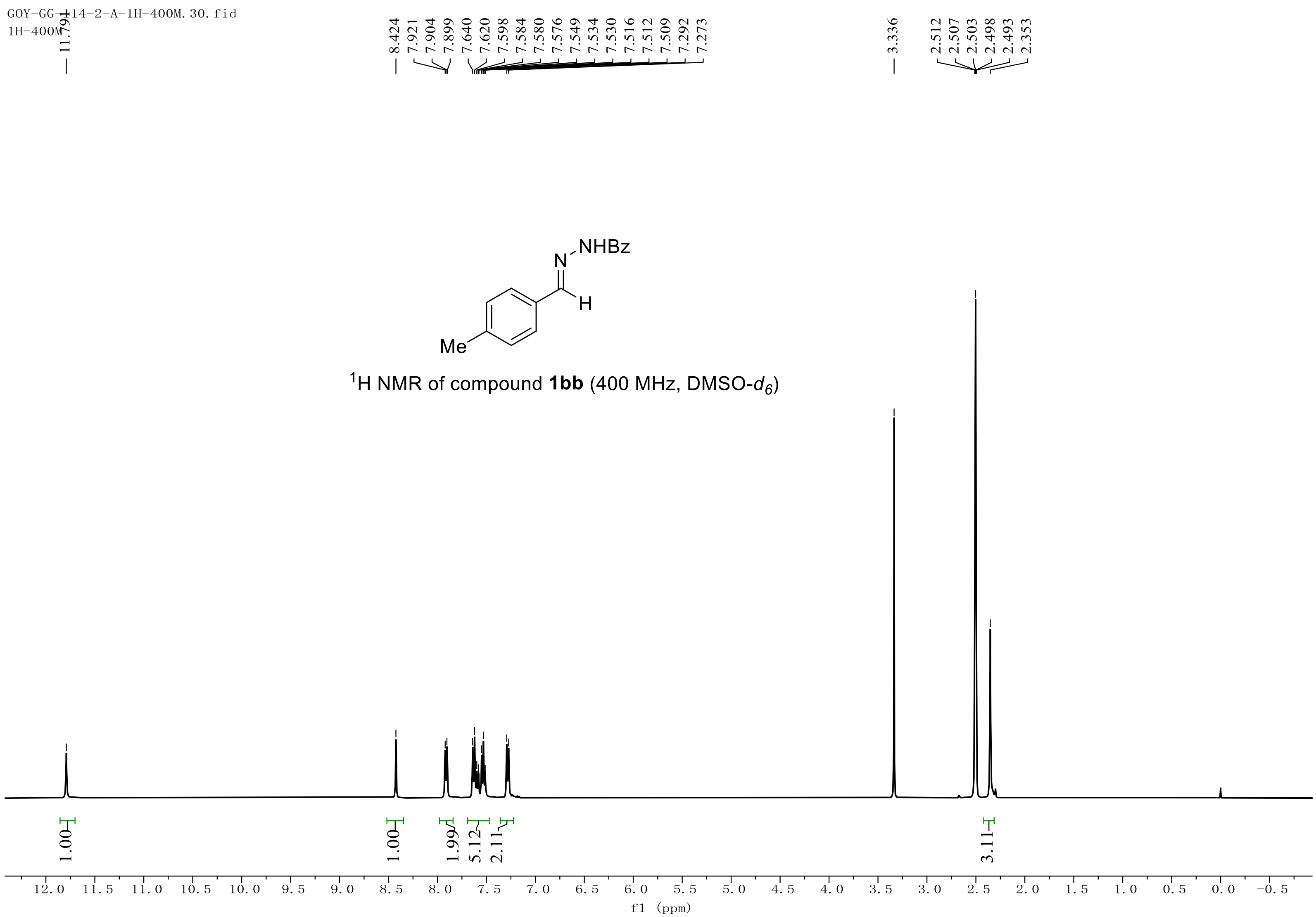

^{1}H NMR of compound **1bb** (400 MHz, DMSO-d_6)

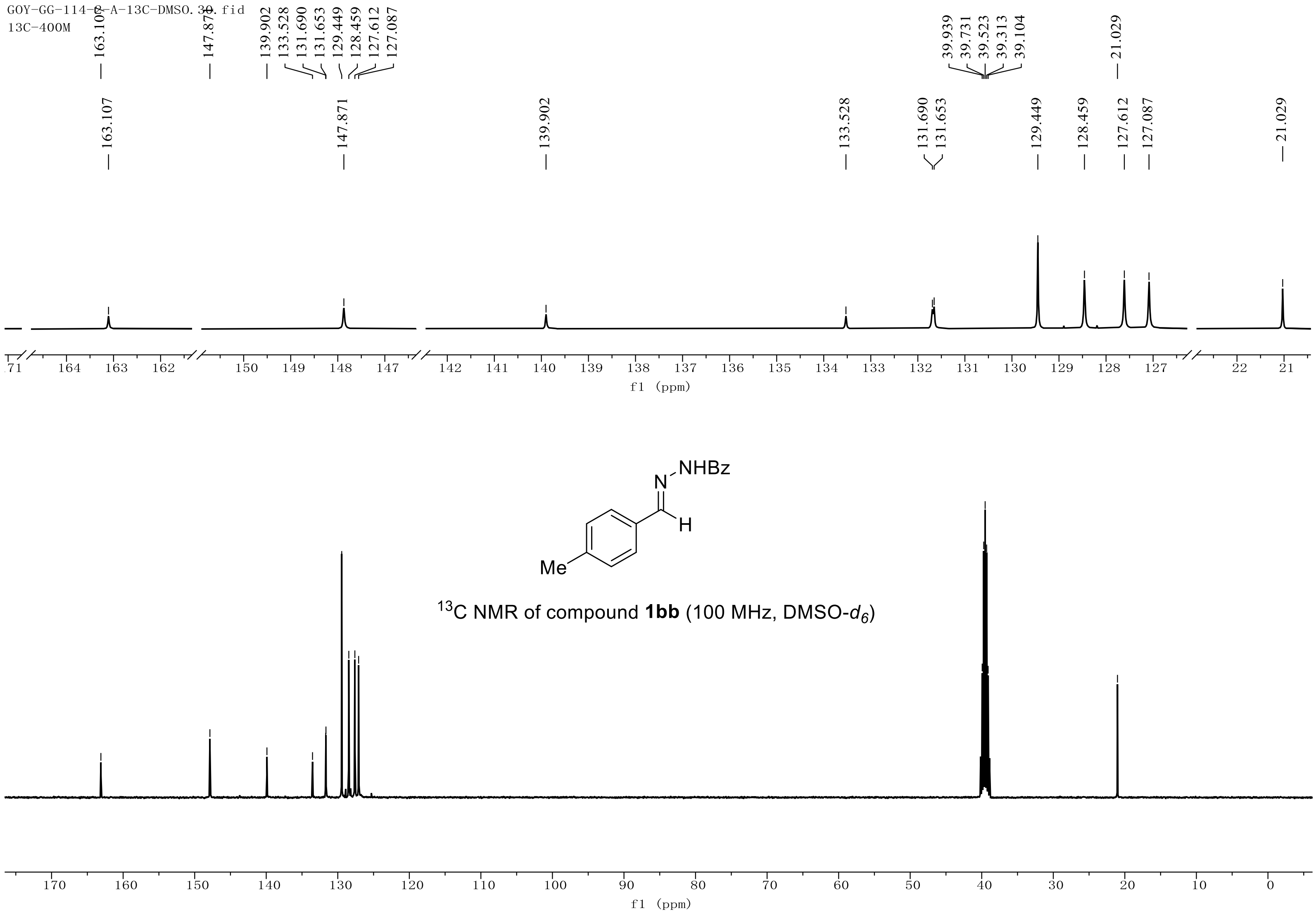

^{13}C NMR of compound **1bb** (100 MHz, DMSO-d_6)

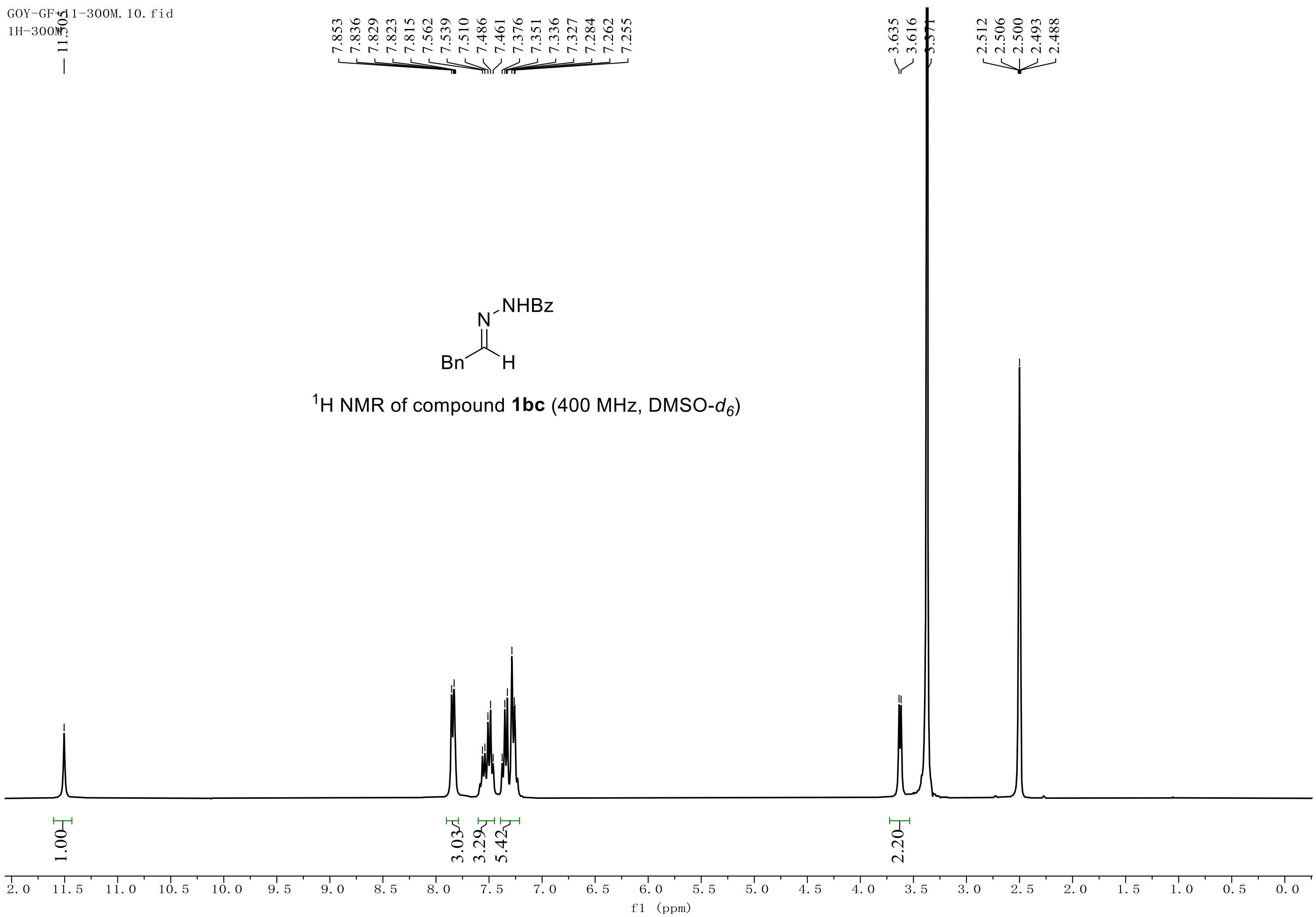

^{1}H NMR of compound **1bc** (400 MHz, DMSO-d_6)

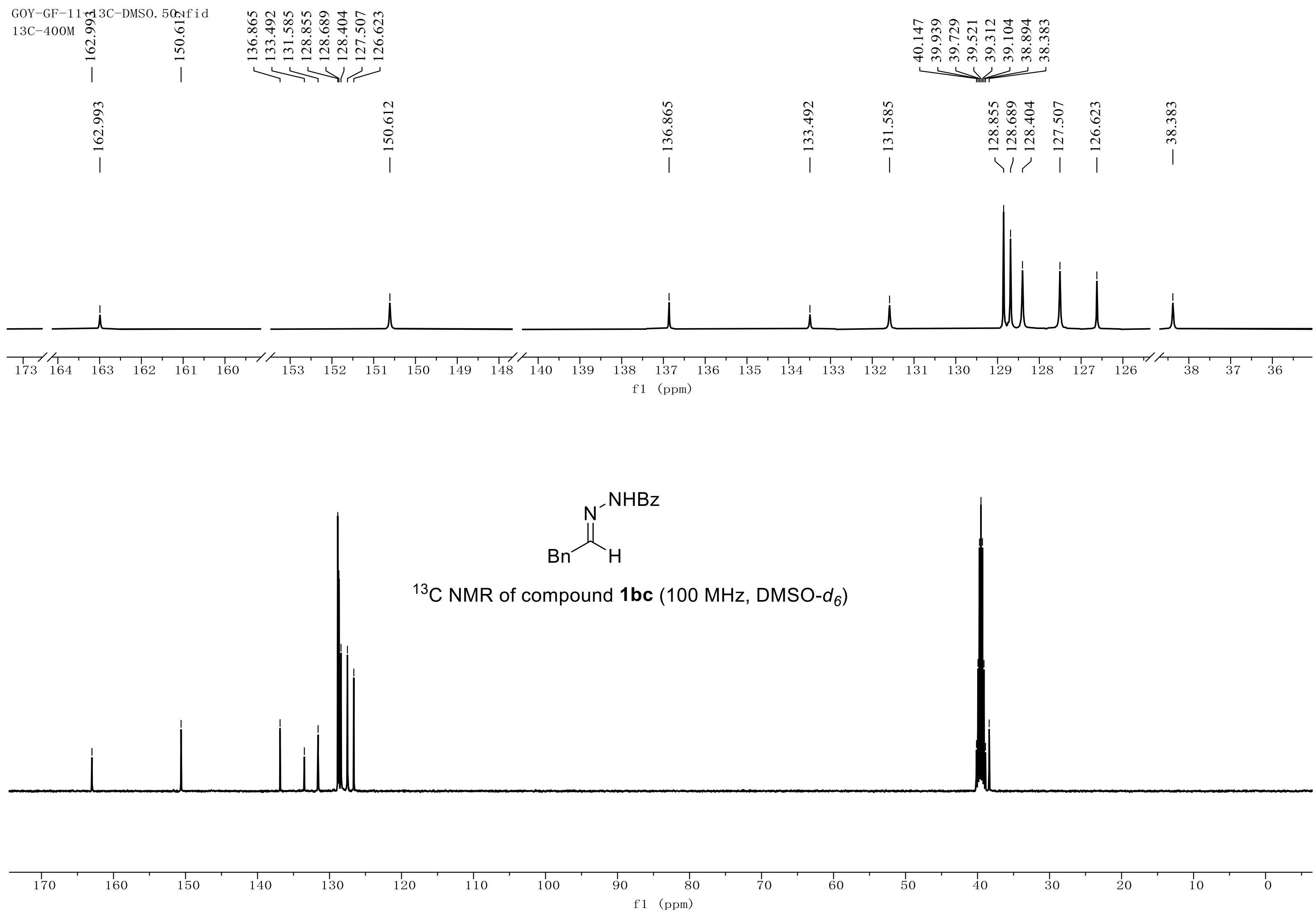

^{13}C NMR of compound **1bc** (100 MHz, DMSO-d_6)

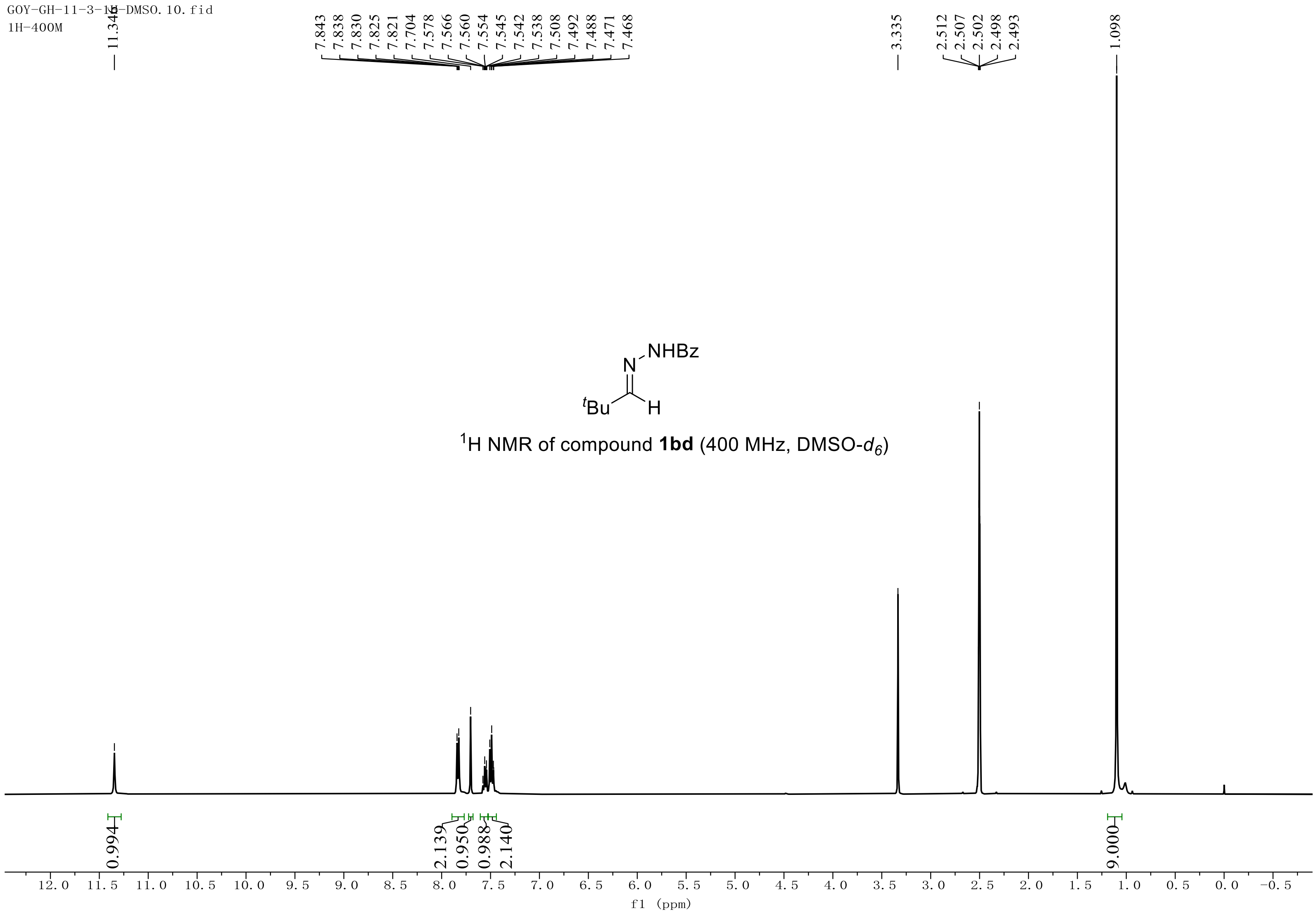

^{1}H NMR of compound **1bd** (400 MHz, DMSO-d_6)

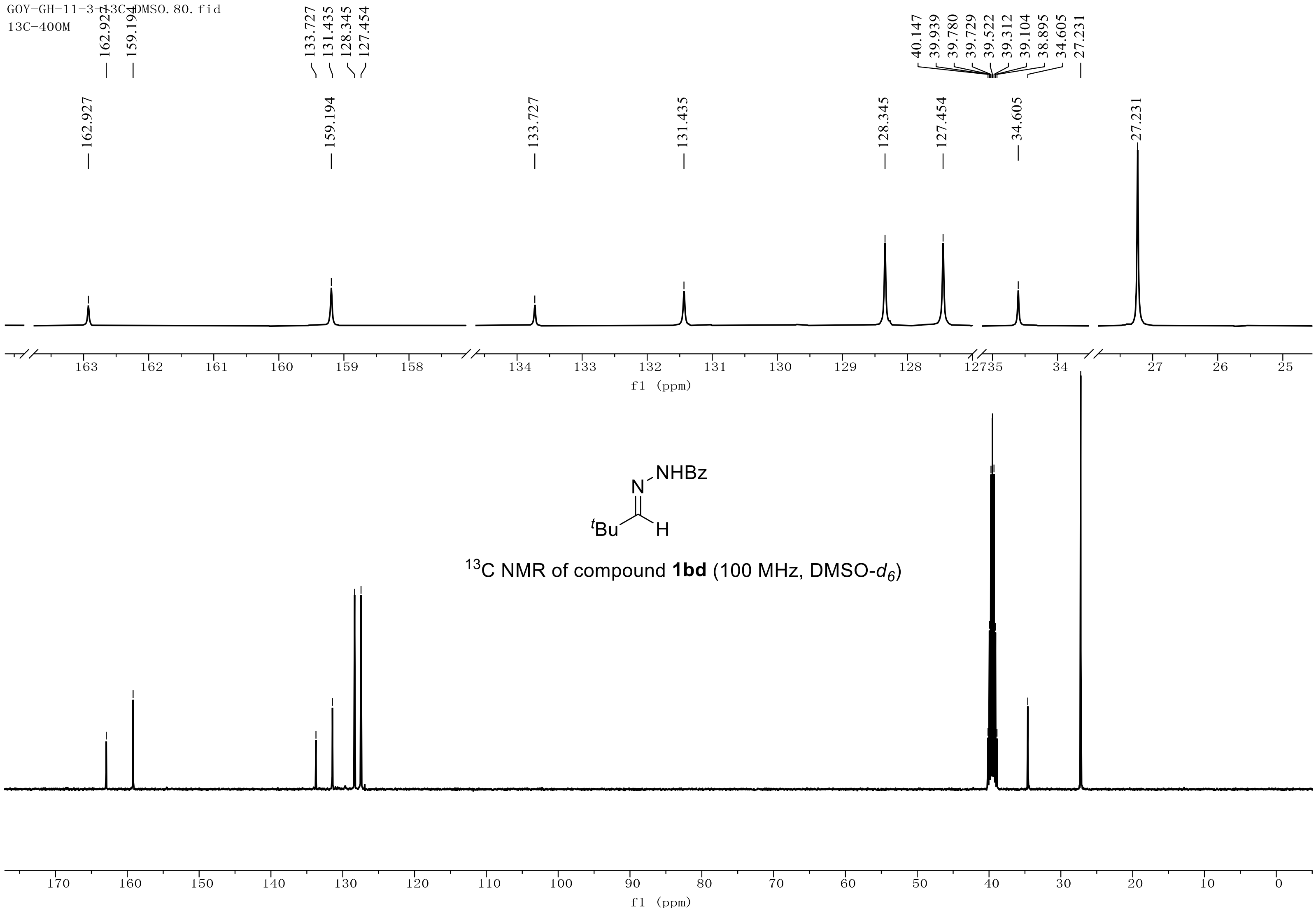

^{13}C NMR of compound **1bd** (100 MHz, DMSO-d_6)

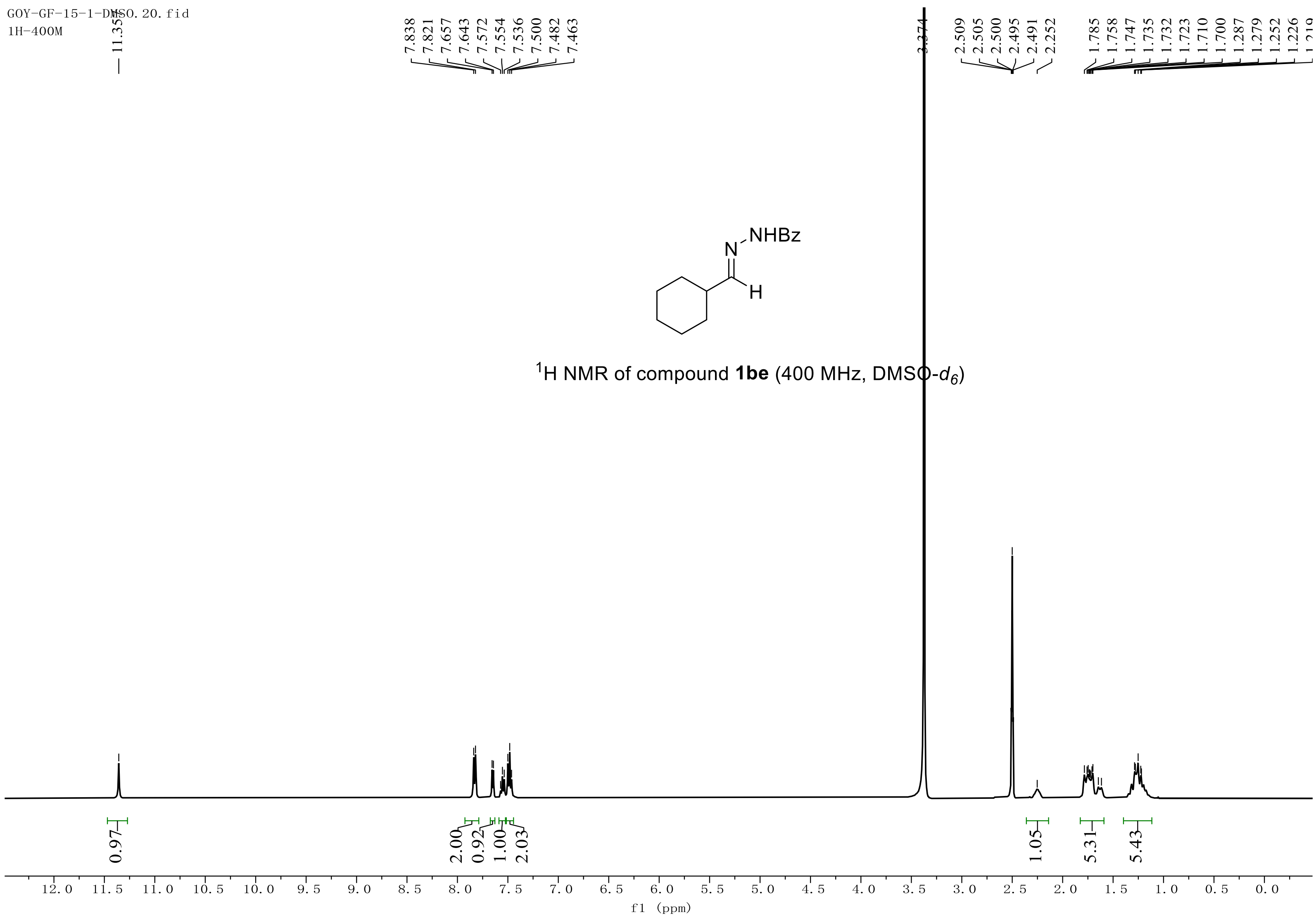

^{1}H NMR of compound **1be** (400 MHz, DMSO-d_6)

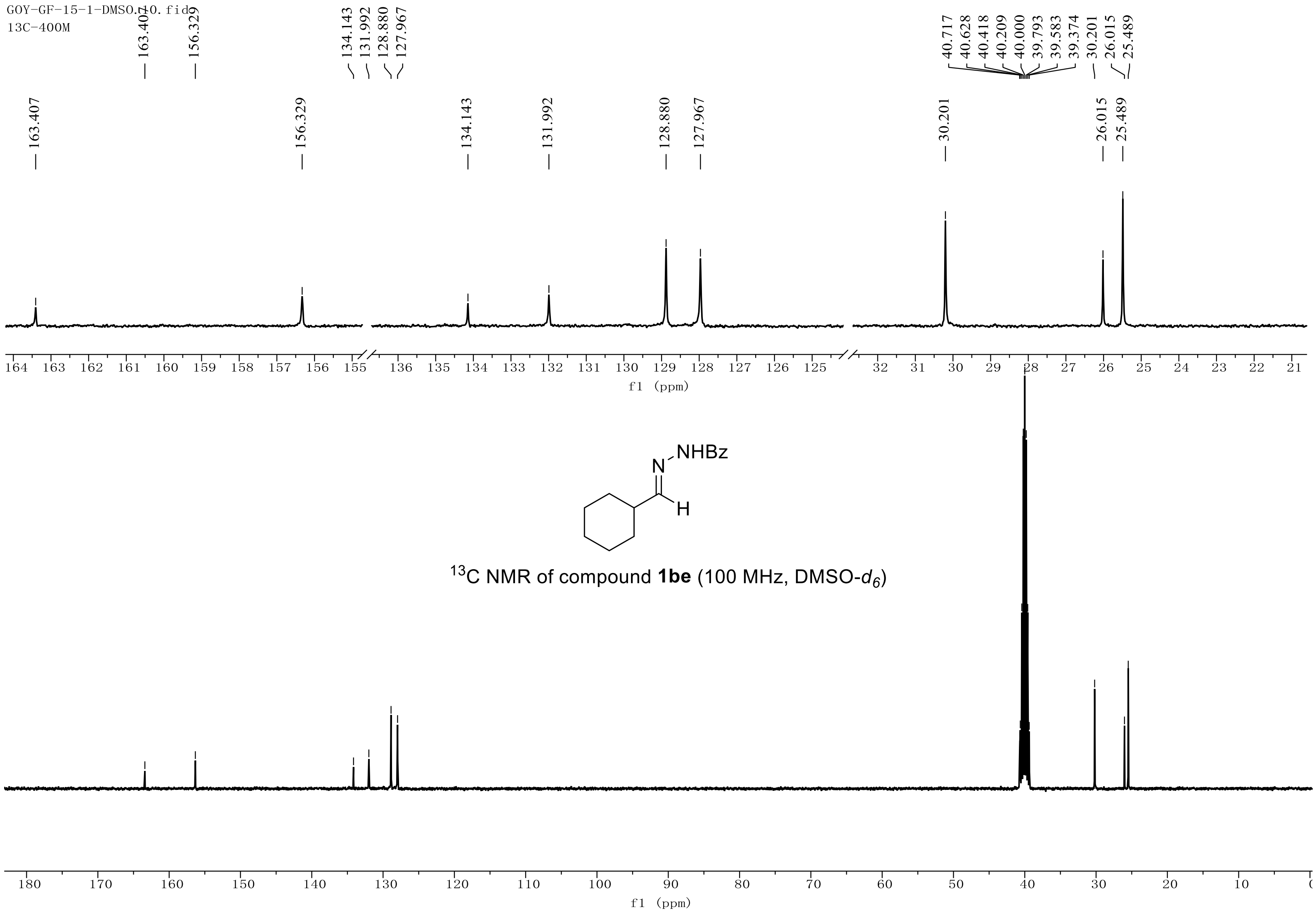

^{13}C NMR of compound **1be** (100 MHz, DMSO-d_6)

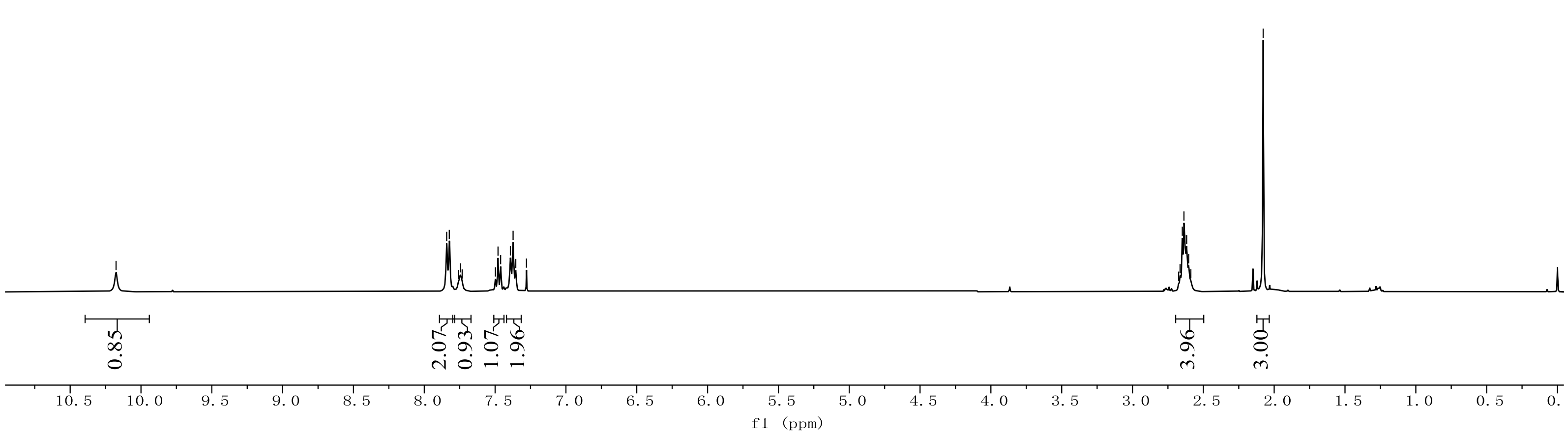

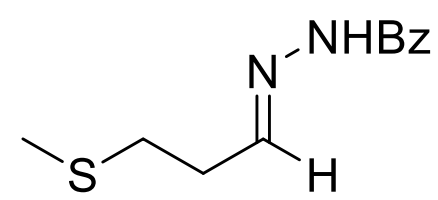

^{1}H NMR of compound **1bf** (400 MHz, DMSO-d_6)

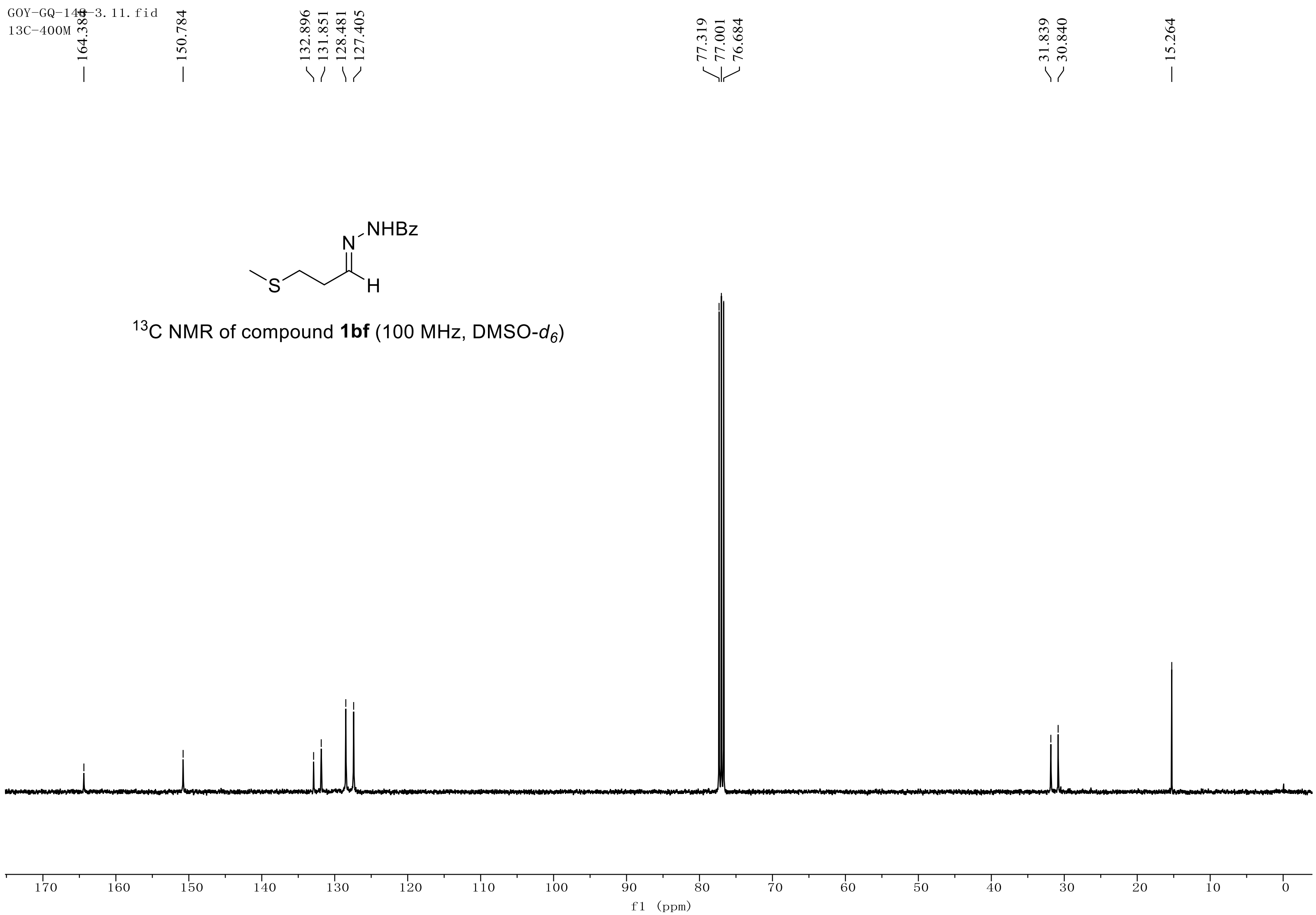

^{13}C NMR of compound **1bf** (100 MHz, DMSO-d_6)

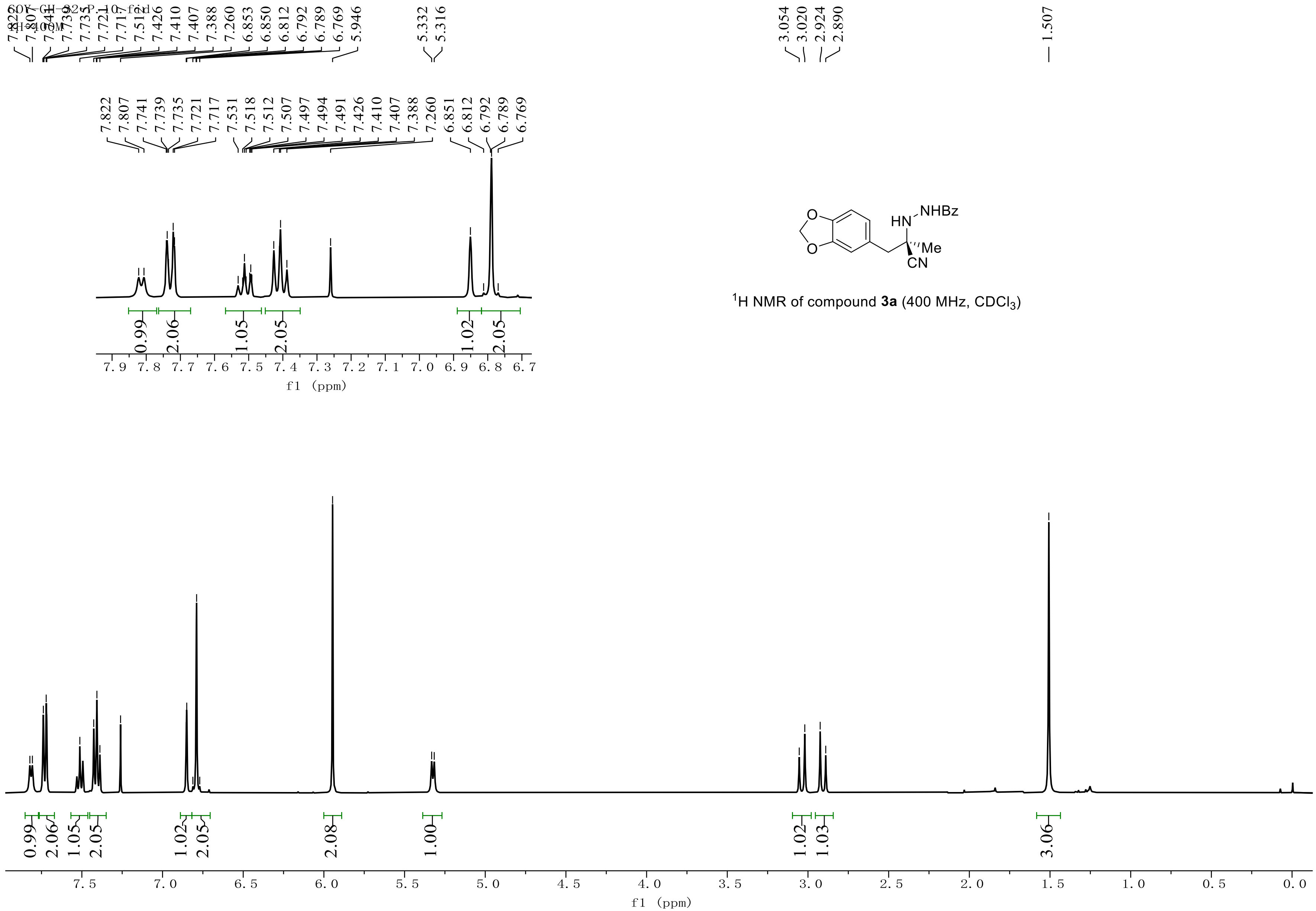

^{1}H NMR of compound **3a** (400 MHz, $CDCl_3$)

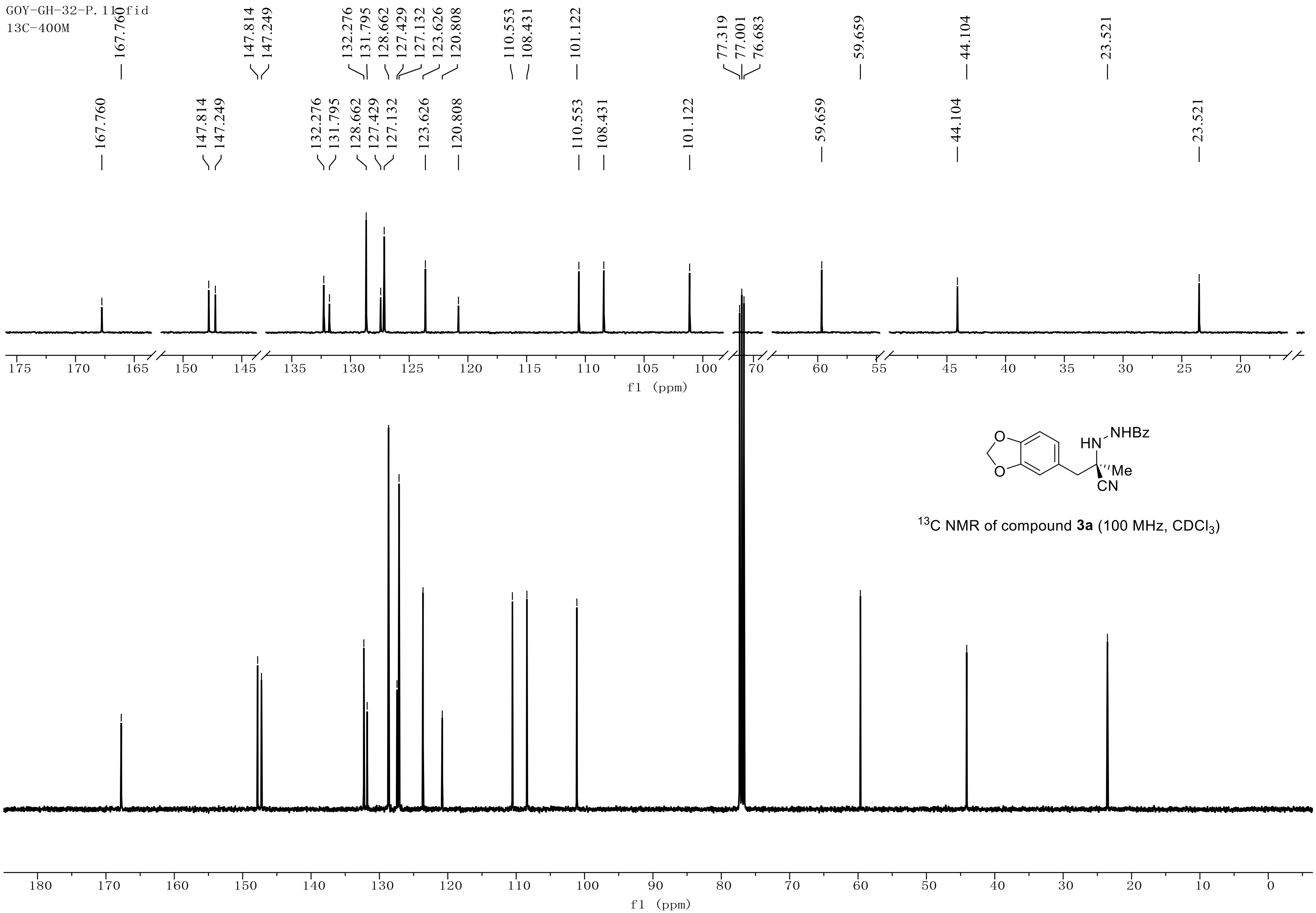

^{13}C NMR of compound **3a** (100 MHz, $CDCl_3$)

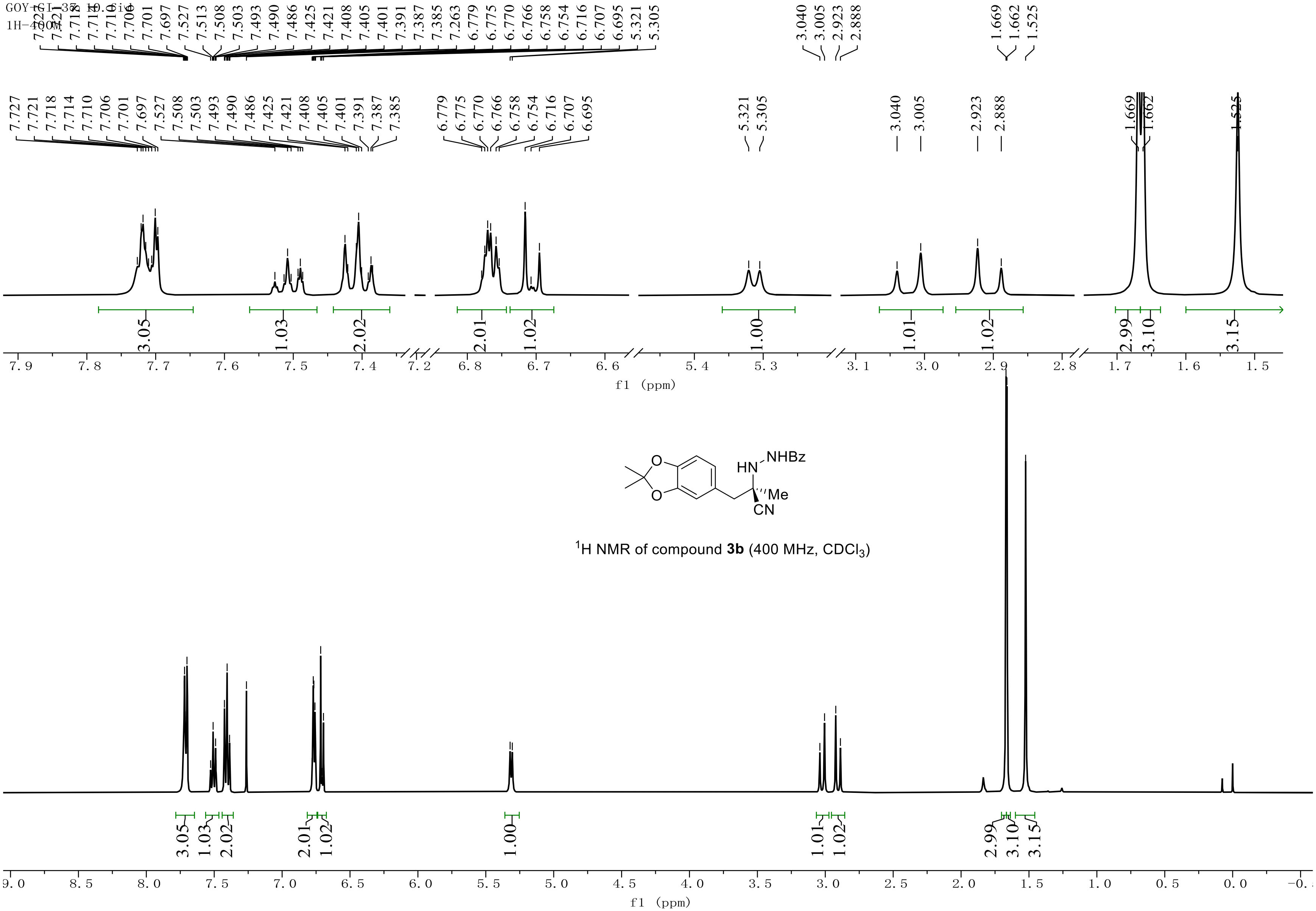

^{1}H NMR of compound **3b** (400 MHz, $CDCl_3$)

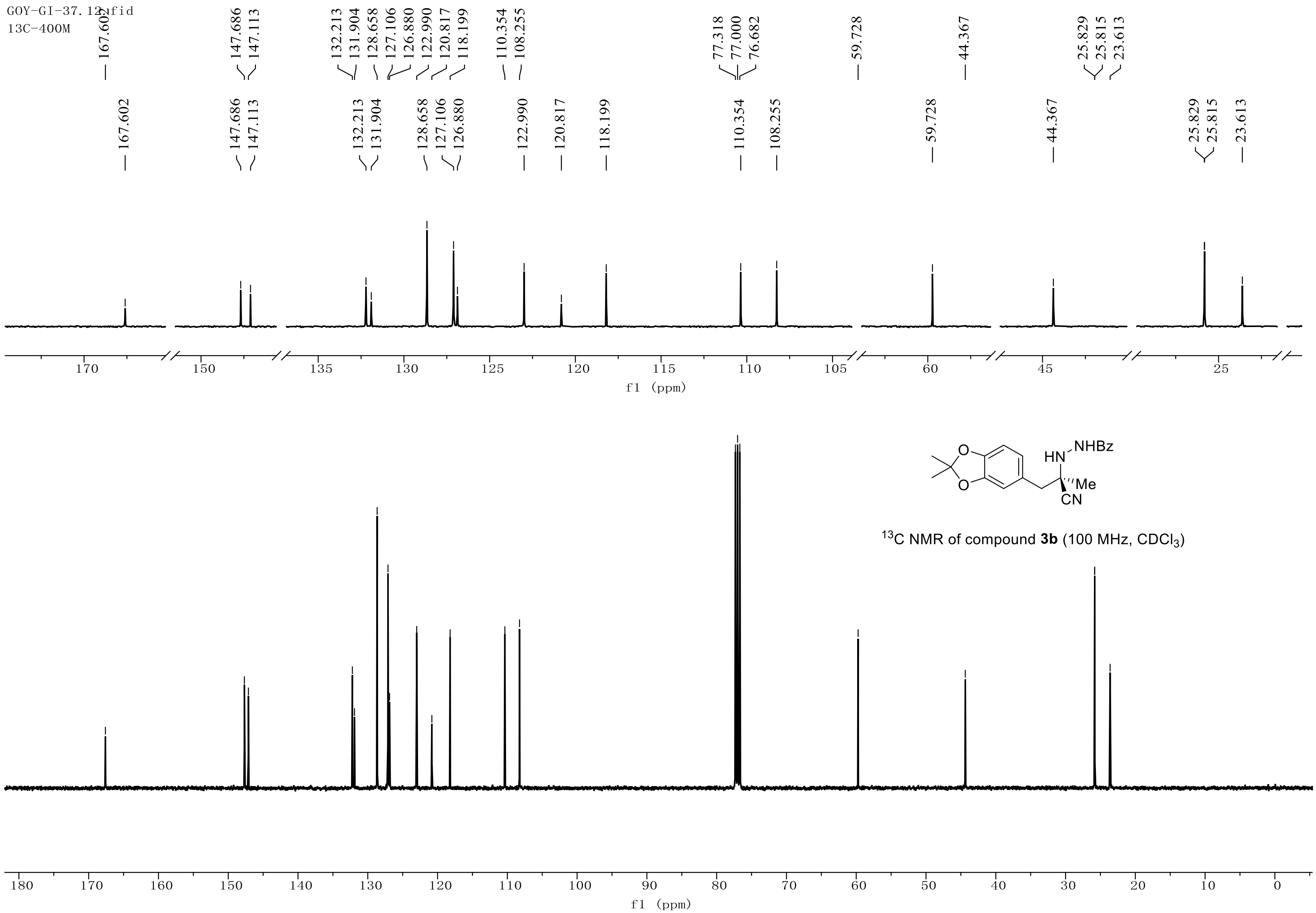

^{13}C NMR of compound **3b** (100 MHz, $CDCl_3$)

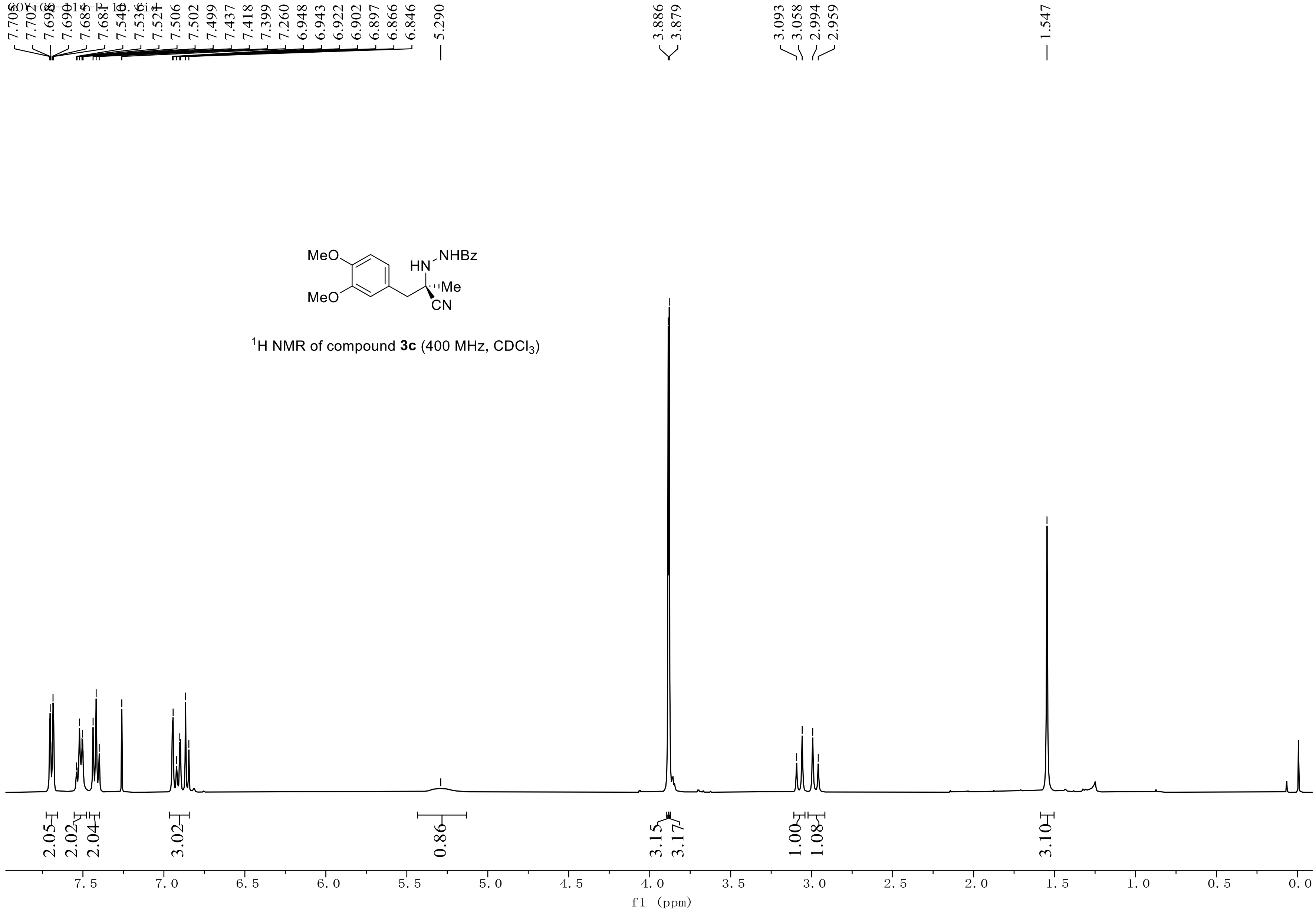

^1H NMR of compound **3c** (400 MHz, $CDCl_3$)

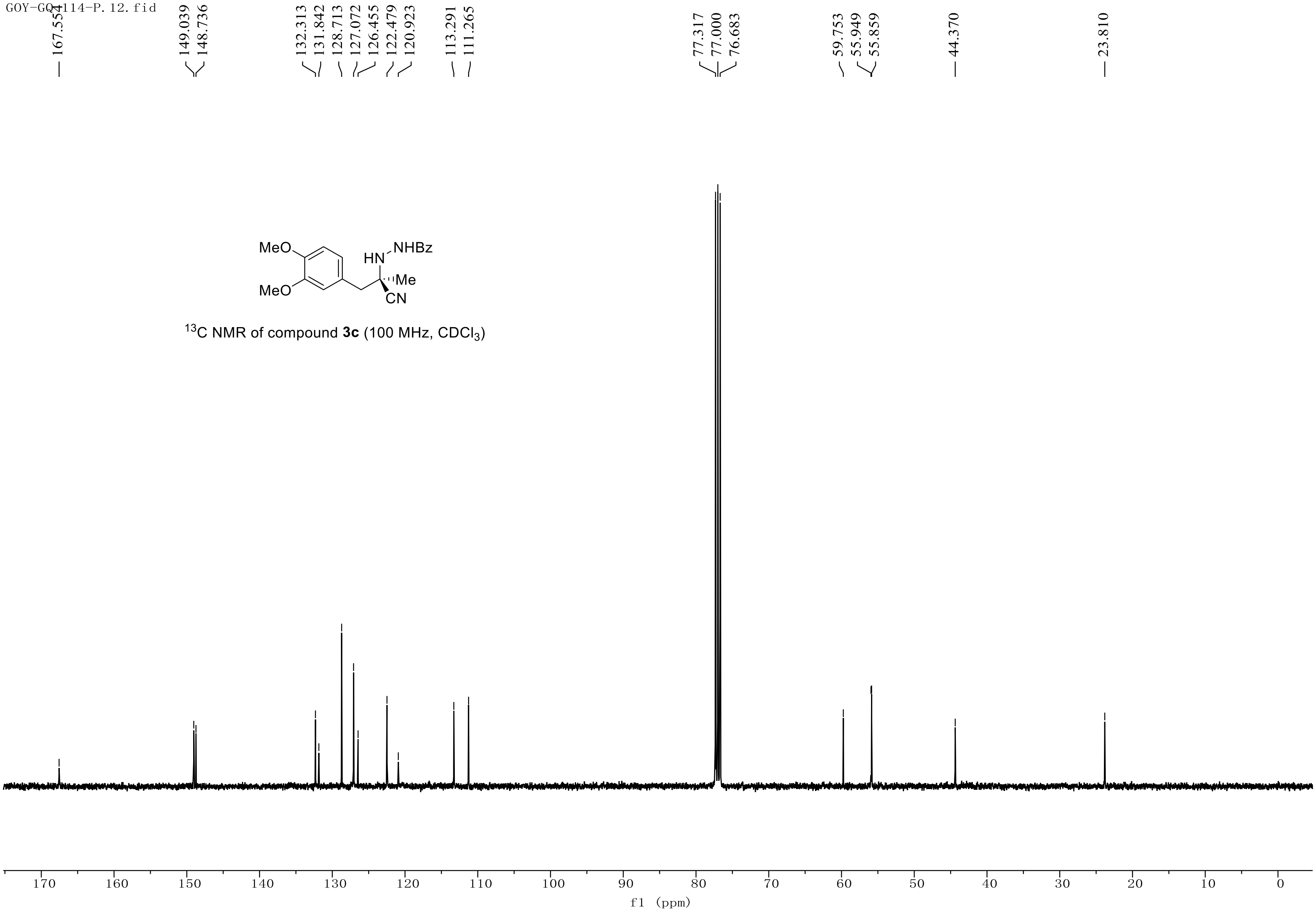

^{13}C NMR of compound **3c** (100 MHz, $CDCl_3$)

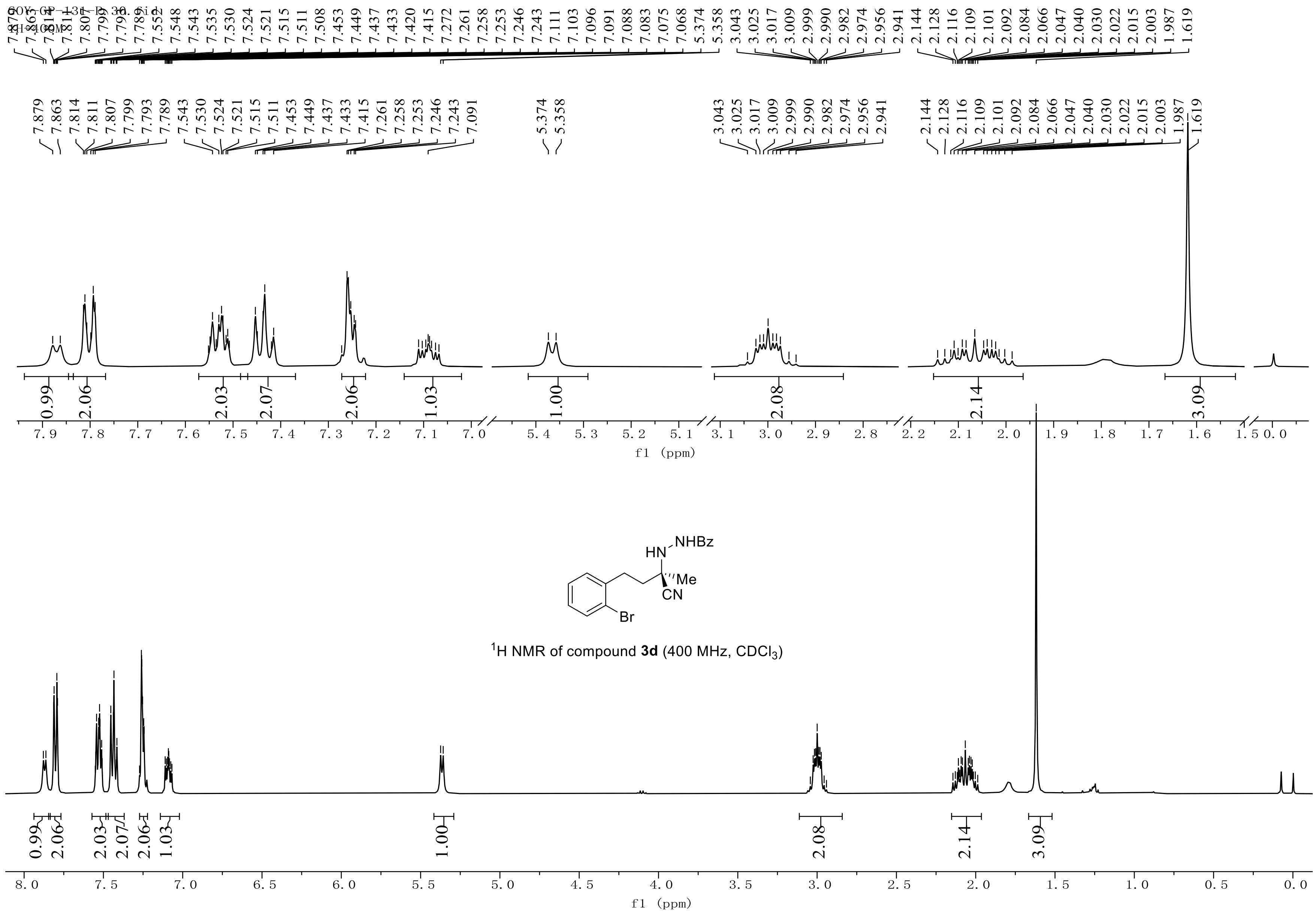

^{1}H NMR of compound **3d** (400 MHz, $CDCl_3$)

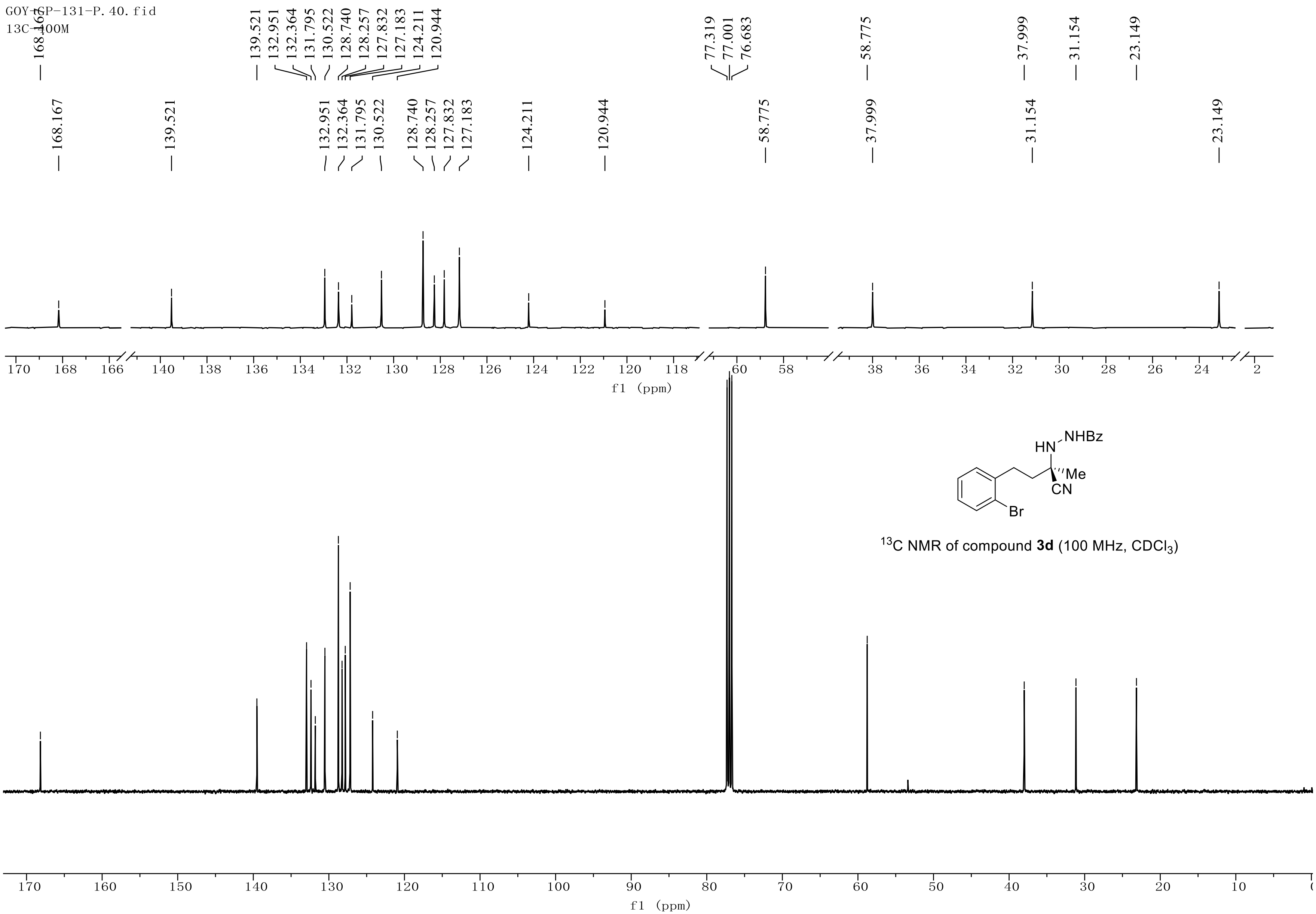

^{13}C NMR of compound **3d** (100 MHz, $CDCl_3$)

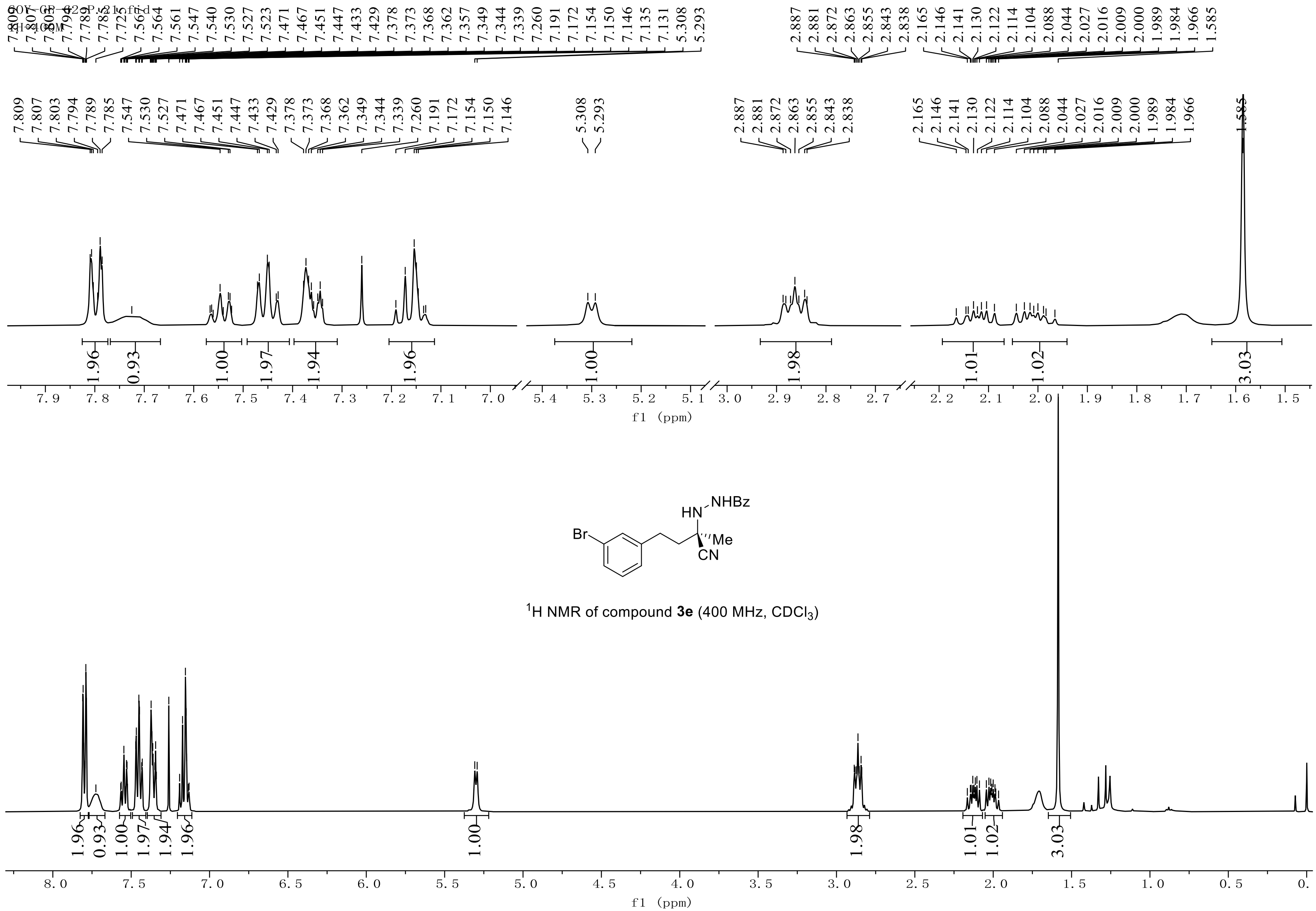

^{1}H NMR of compound **3e** (400 MHz, $CDCl_3$)

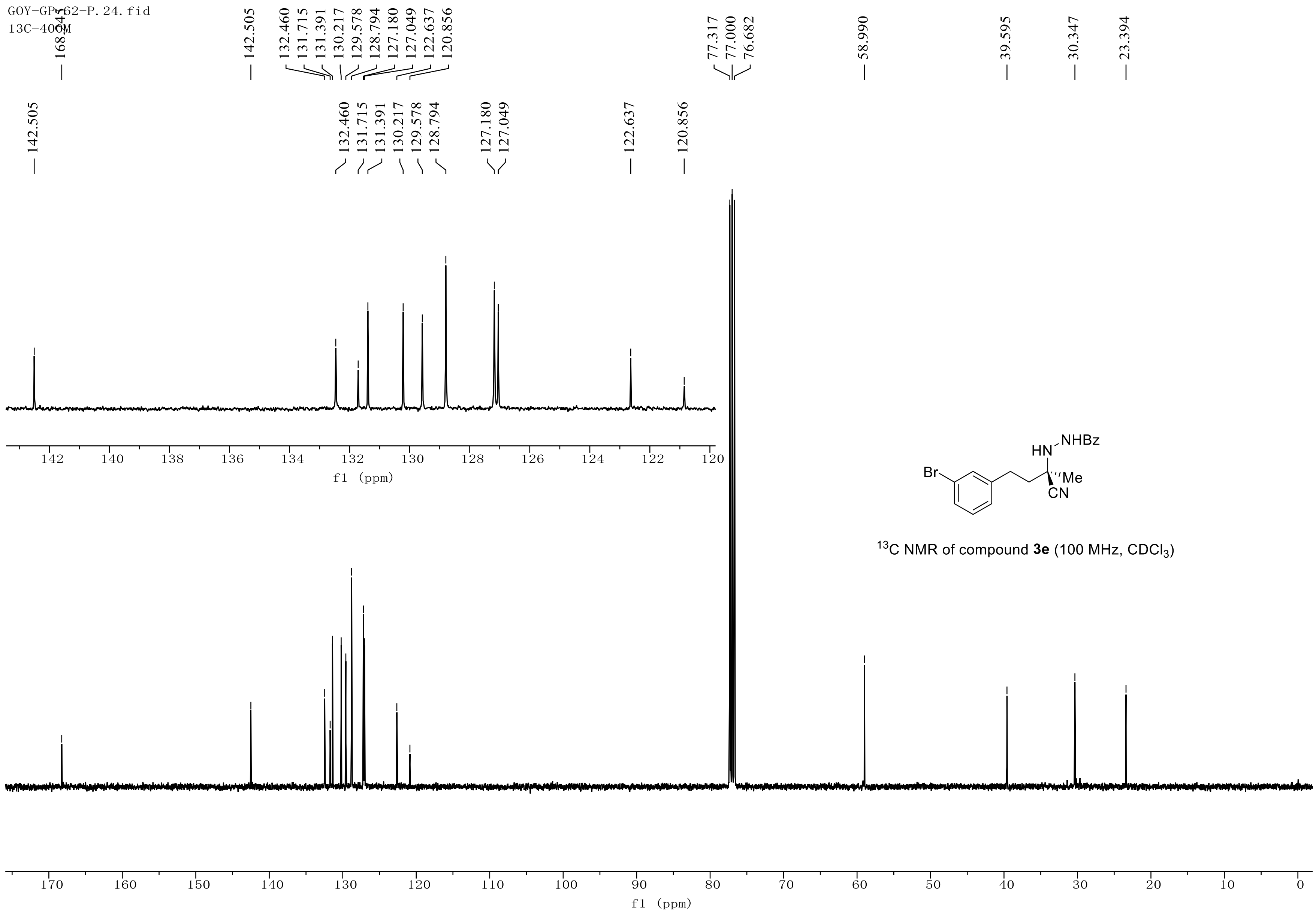

^{13}C NMR of compound **3e** (100 MHz, $CDCl_3$)

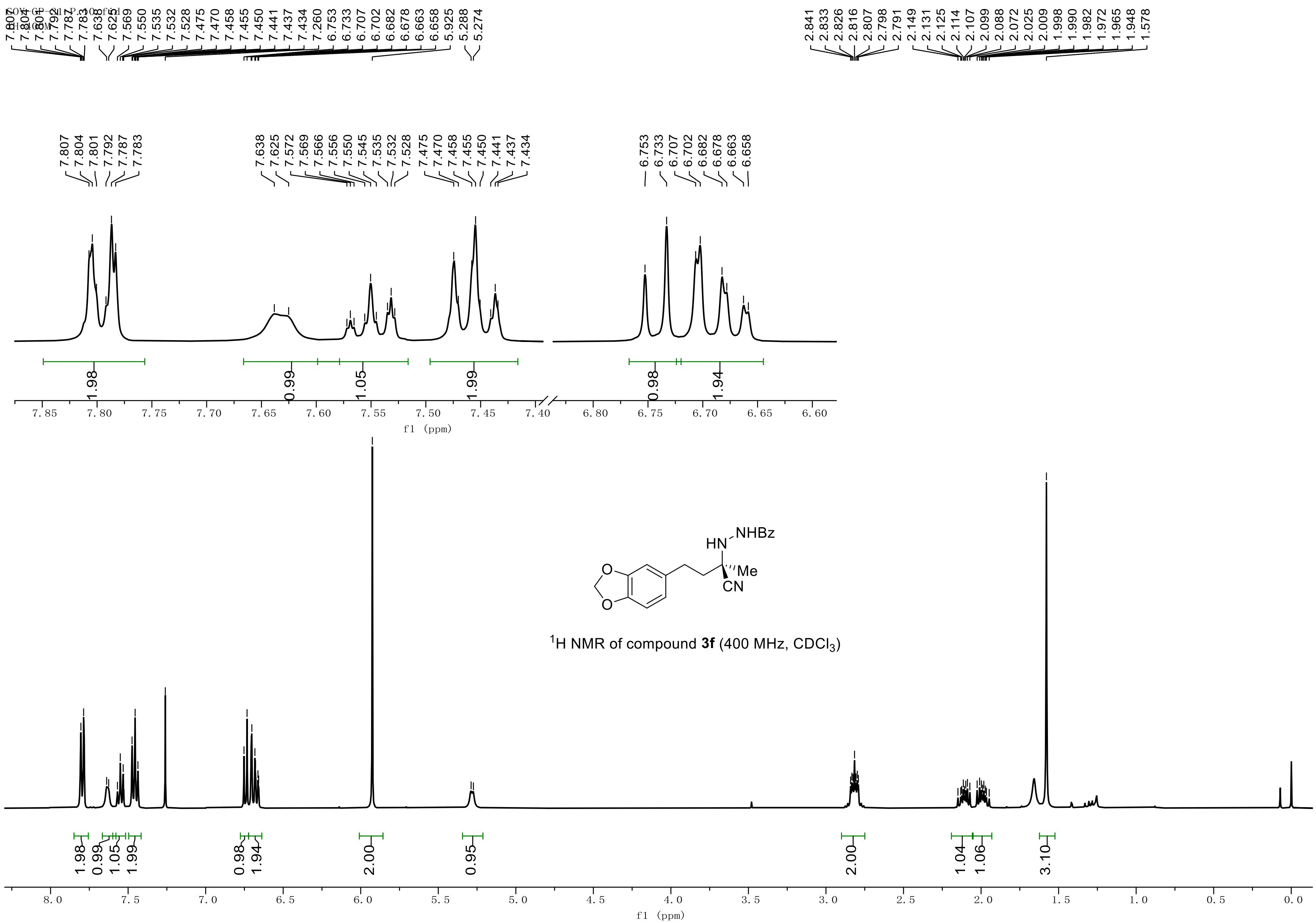

^{1}H NMR of compound **3f** (400 MHz, $CDCl_3$)

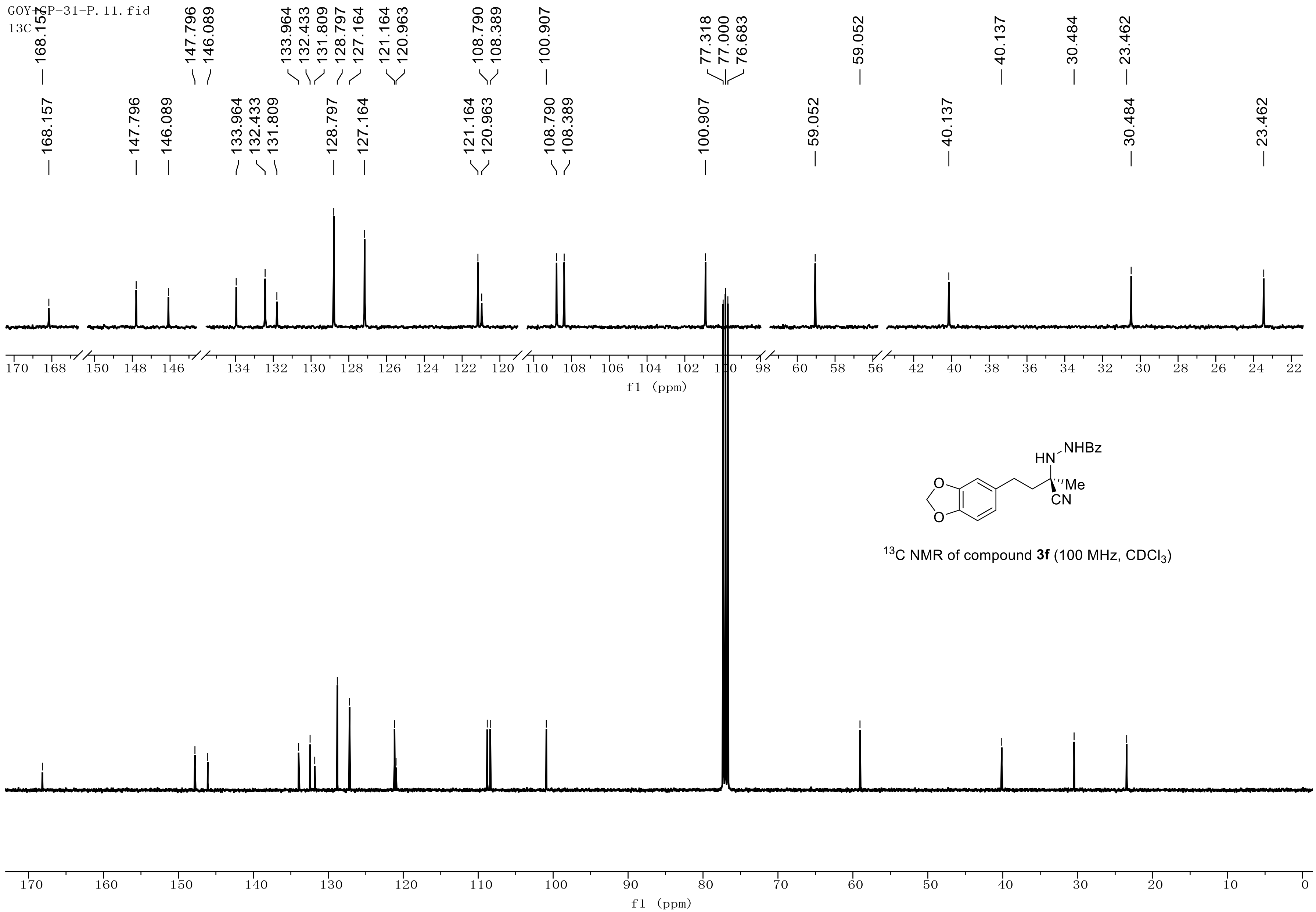

^{13}C NMR of compound **3f** (100 MHz, $CDCl_3$)

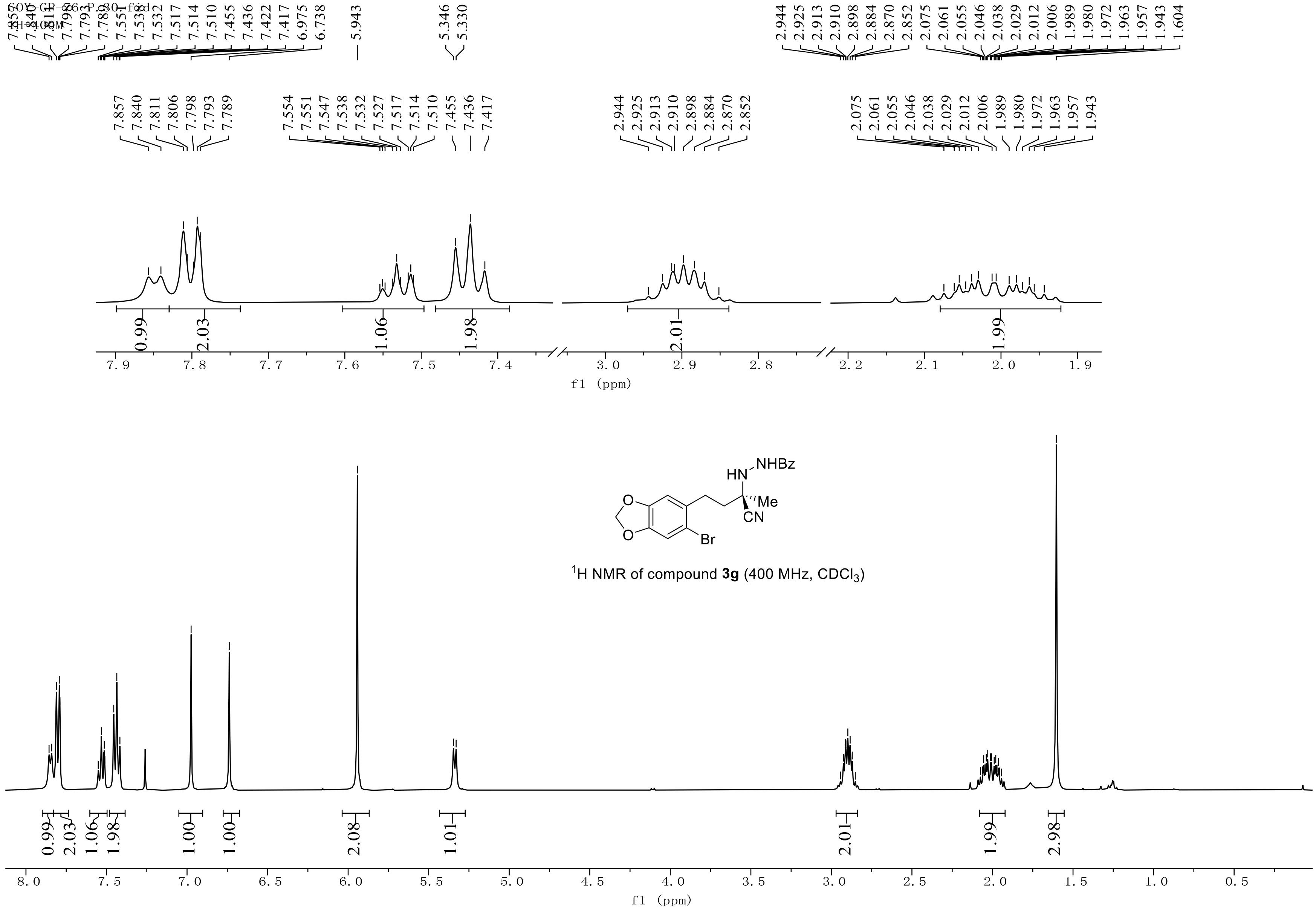

^{1}H NMR of compound **3g** (400 MHz, $CDCl_3$)

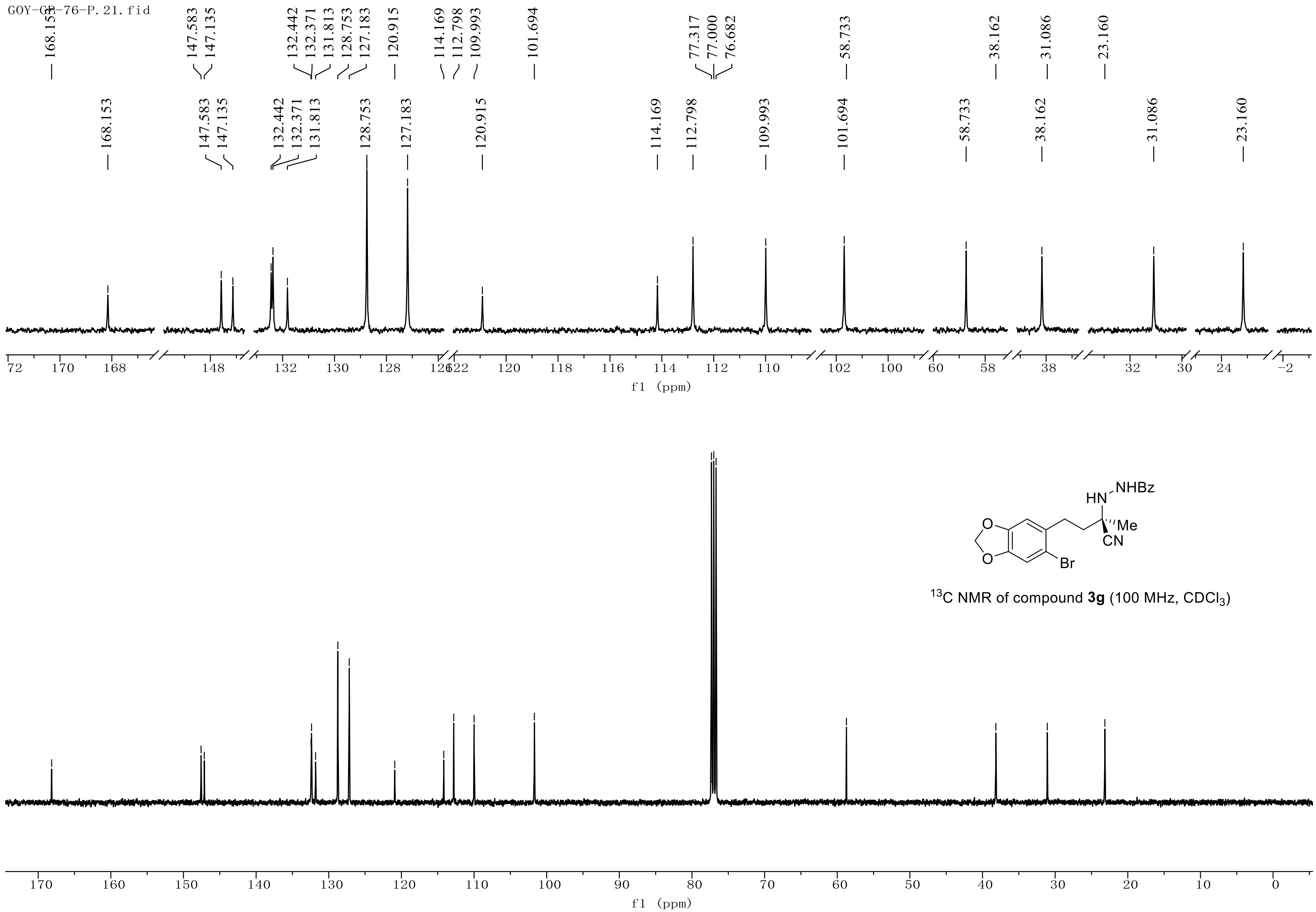

^{13}C NMR of compound **3g** (100 MHz, $CDCl_3$)

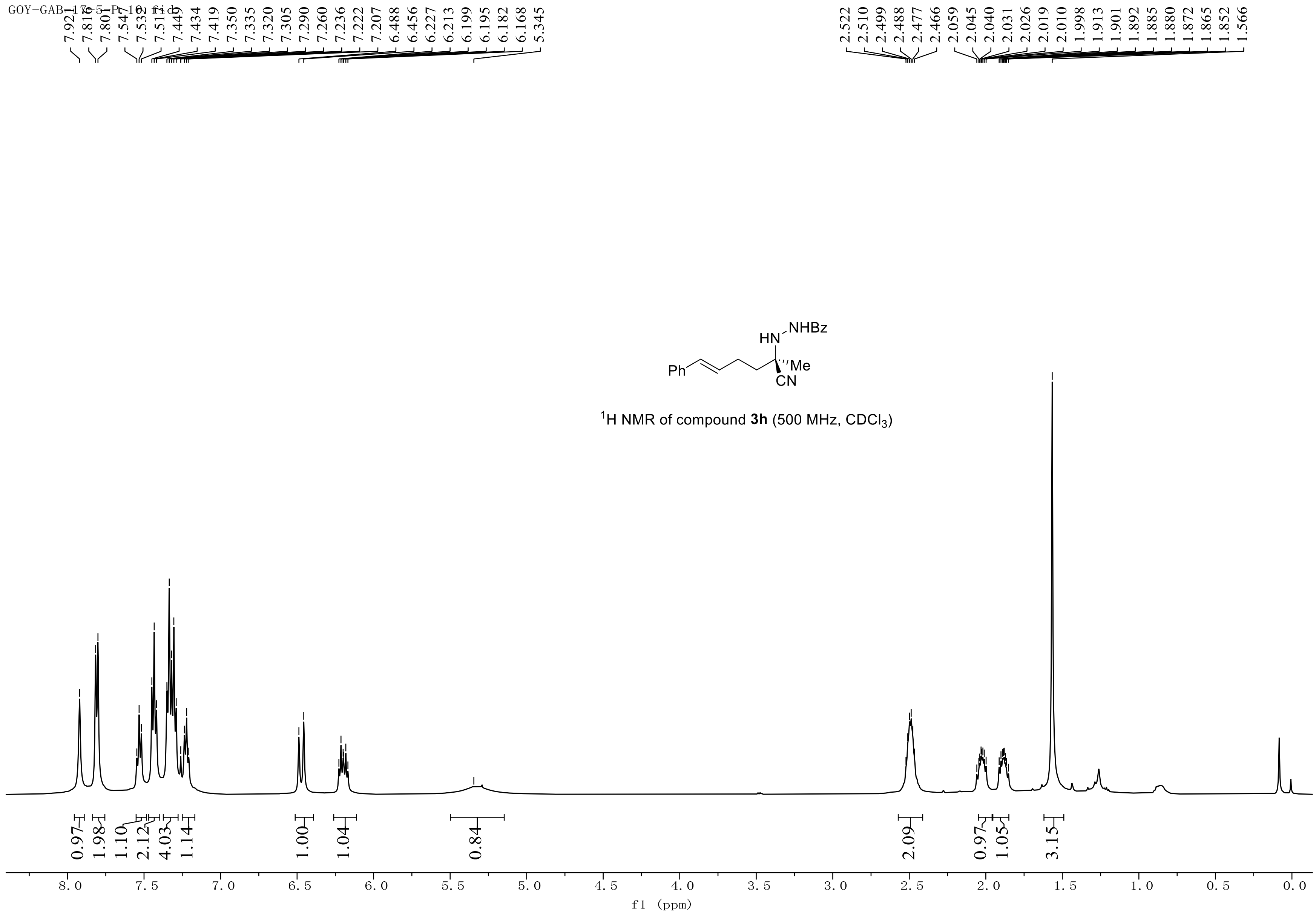

^{1}H NMR of compound **3h** (500 MHz, $CDCl_3$)

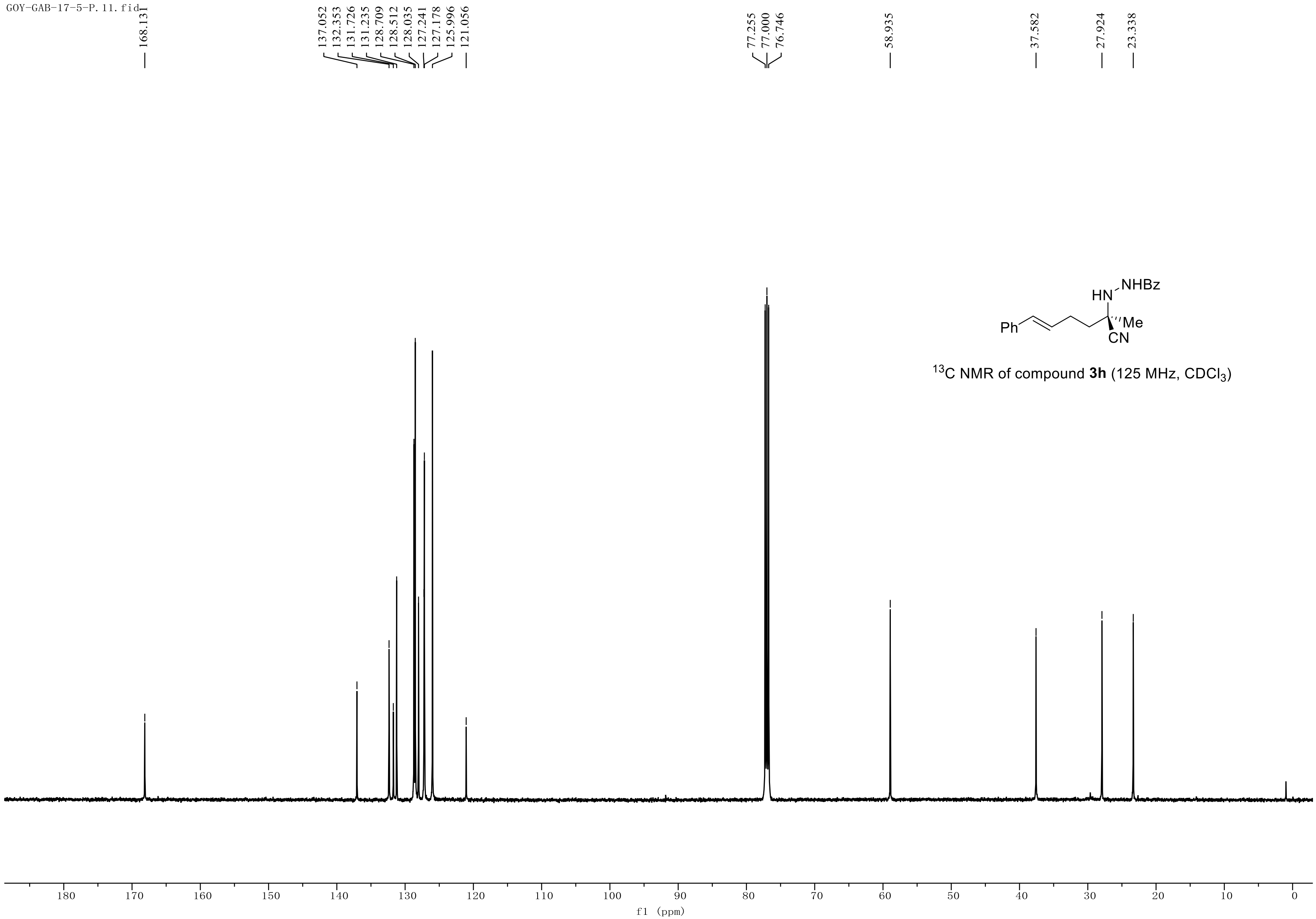
GOY-GAB-17-5-P.11.fid
168.131
137.052
132.353
131.726
131.235
128.709
128.512
128.035
127.241
127.178
125.996
121.056
77.255
77.000
76.746
58.935
37.582
27.924
23.338
HN
NHBz
Ph
Me
CN
13C NMR of compound 3h (125 MHz, CDCl3)
180
170
160
150
140
130
120
110
100
90
80
70
60
50
40
30
20
10
0
f1 (ppm)

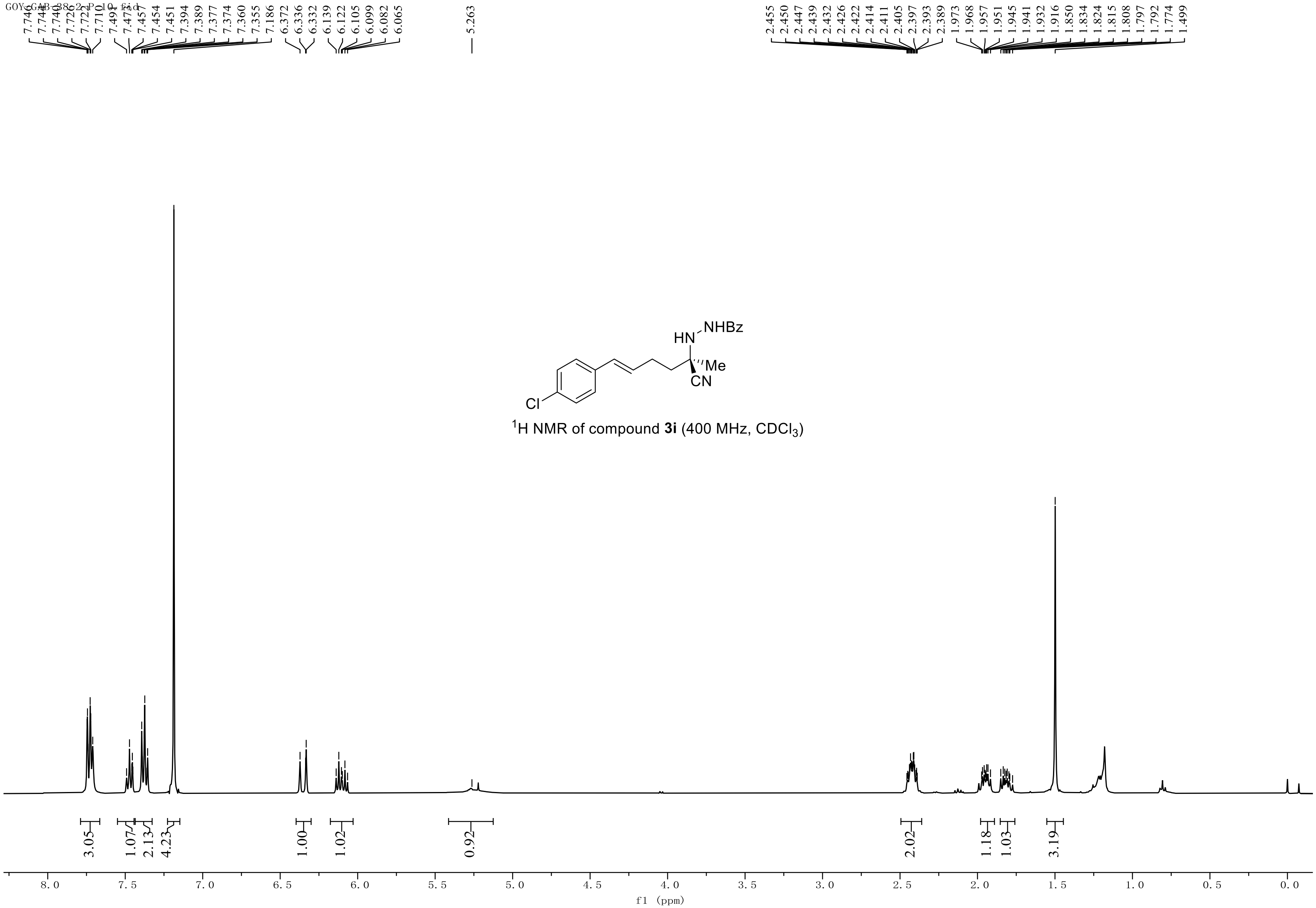

^{1}H NMR of compound **3i** (400 MHz, $CDCl_3$)

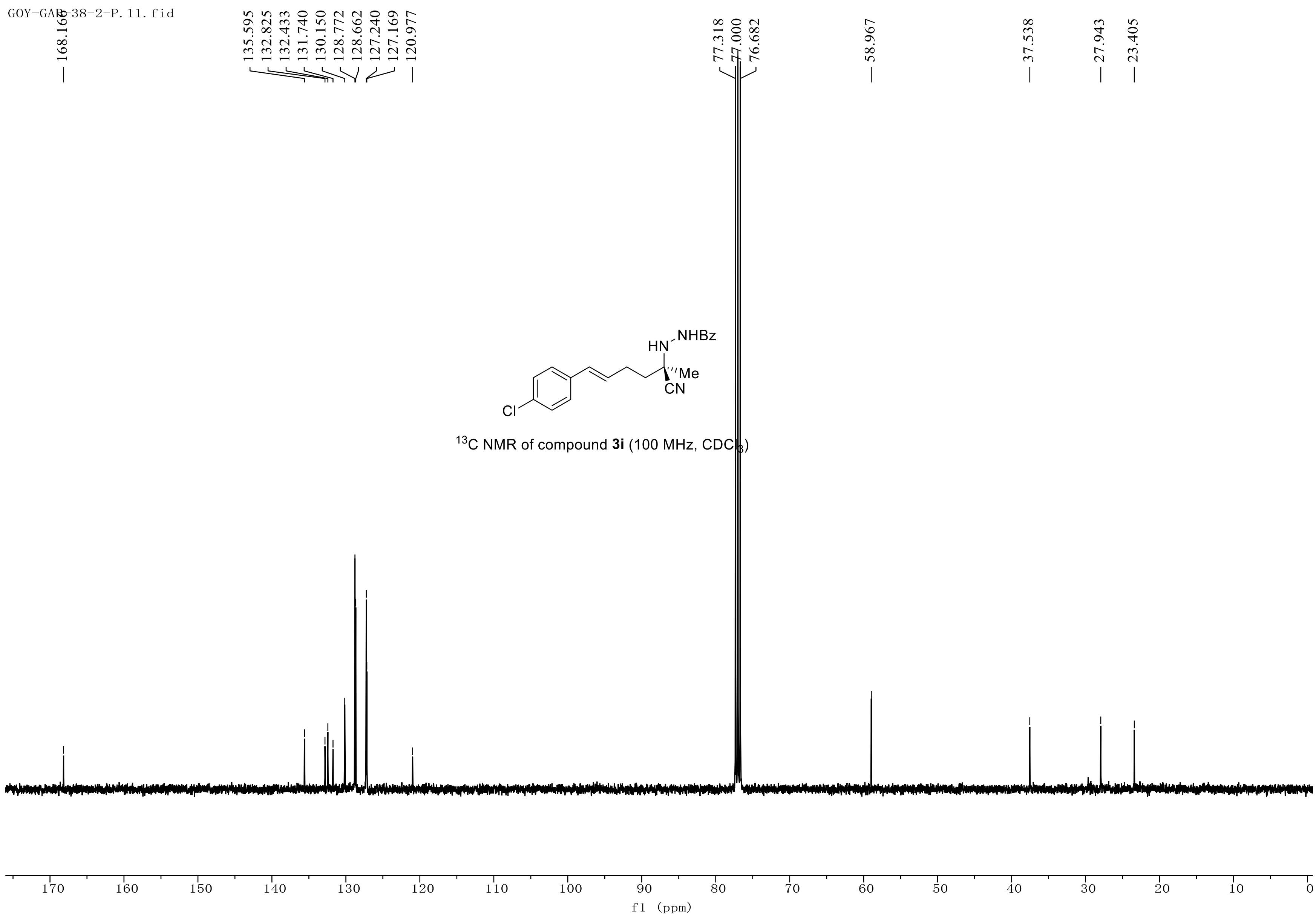

^{13}C NMR of compound **3i** (100 MHz, $CDCl_3$)

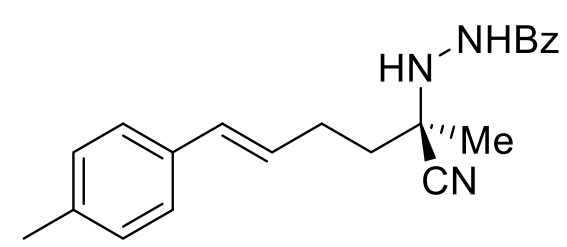

^{1}H NMR of compound **3j** (400 MHz, $CDCl_3$)

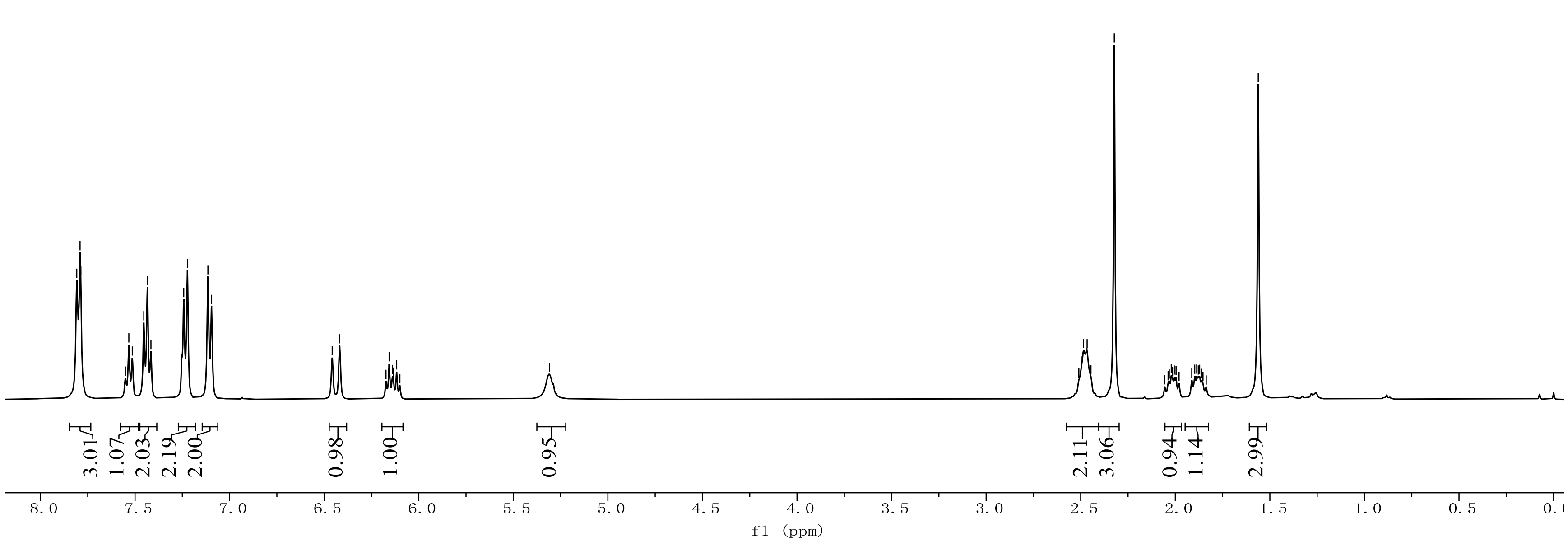

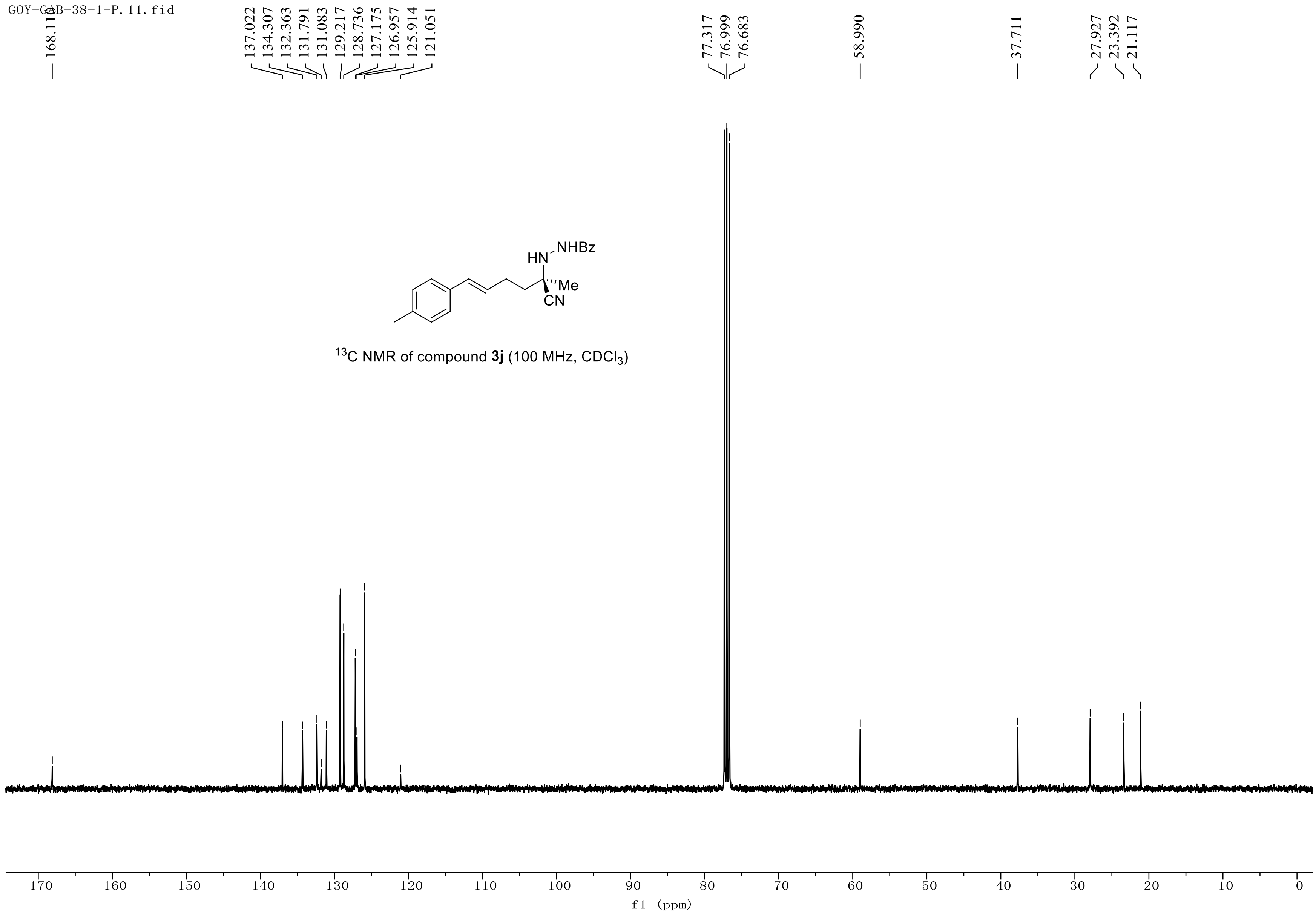

^{13}C NMR of compound **3j** (100 MHz, $CDCl_3$)

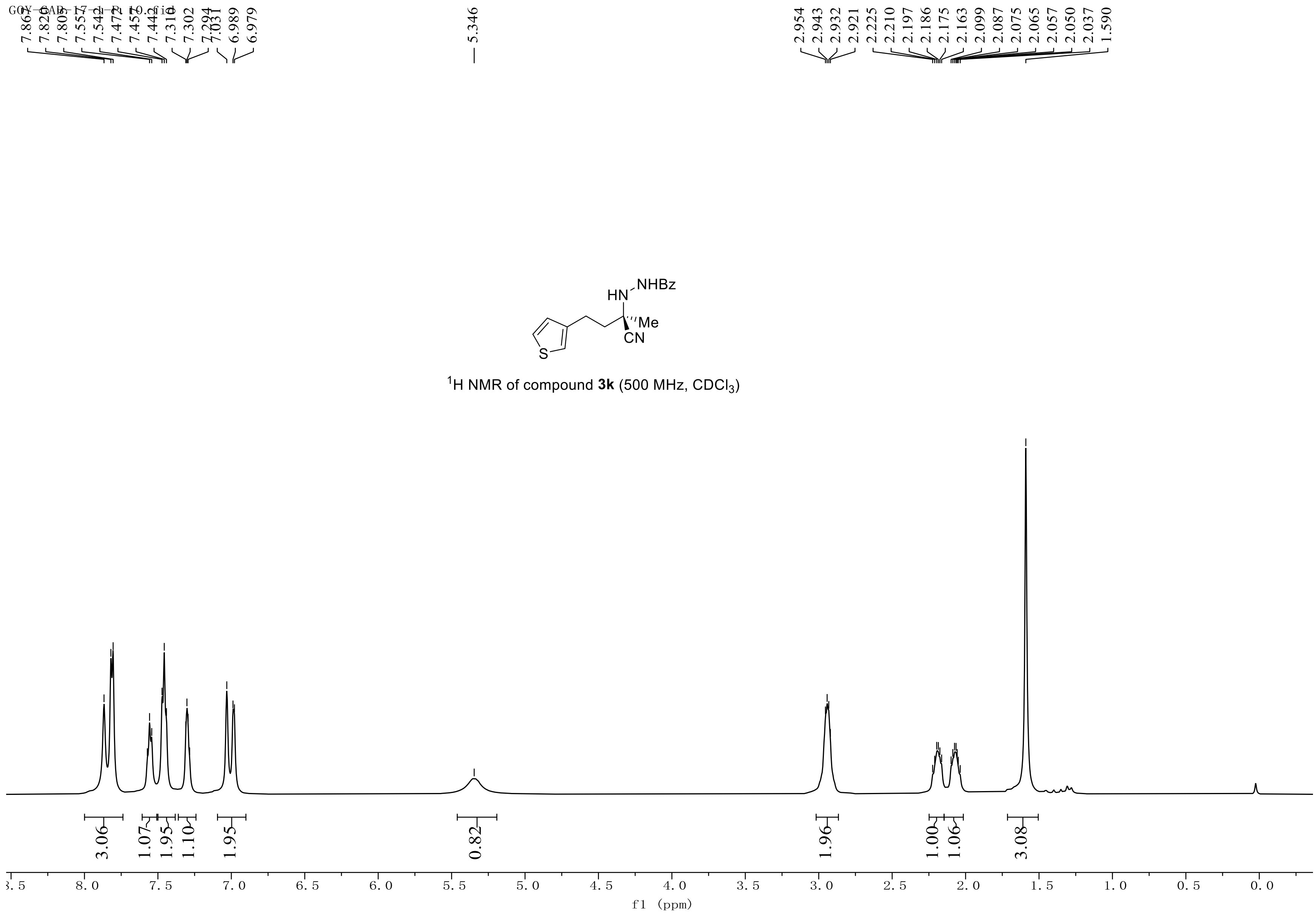

^{1}H NMR of compound **3k** (500 MHz, $CDCl_3$)

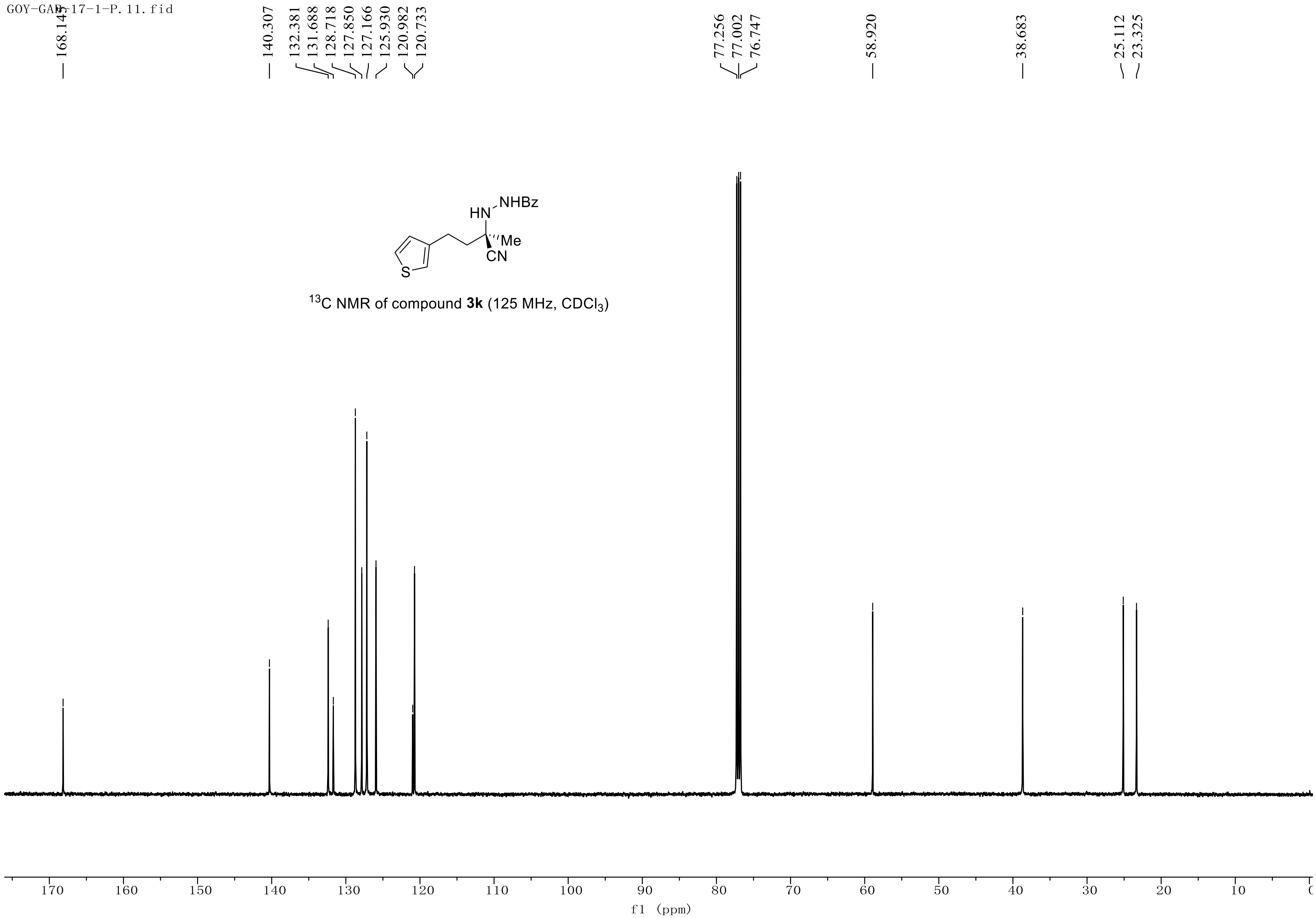

^{13}C NMR of compound **3k** (125 MHz, $CDCl_3$)

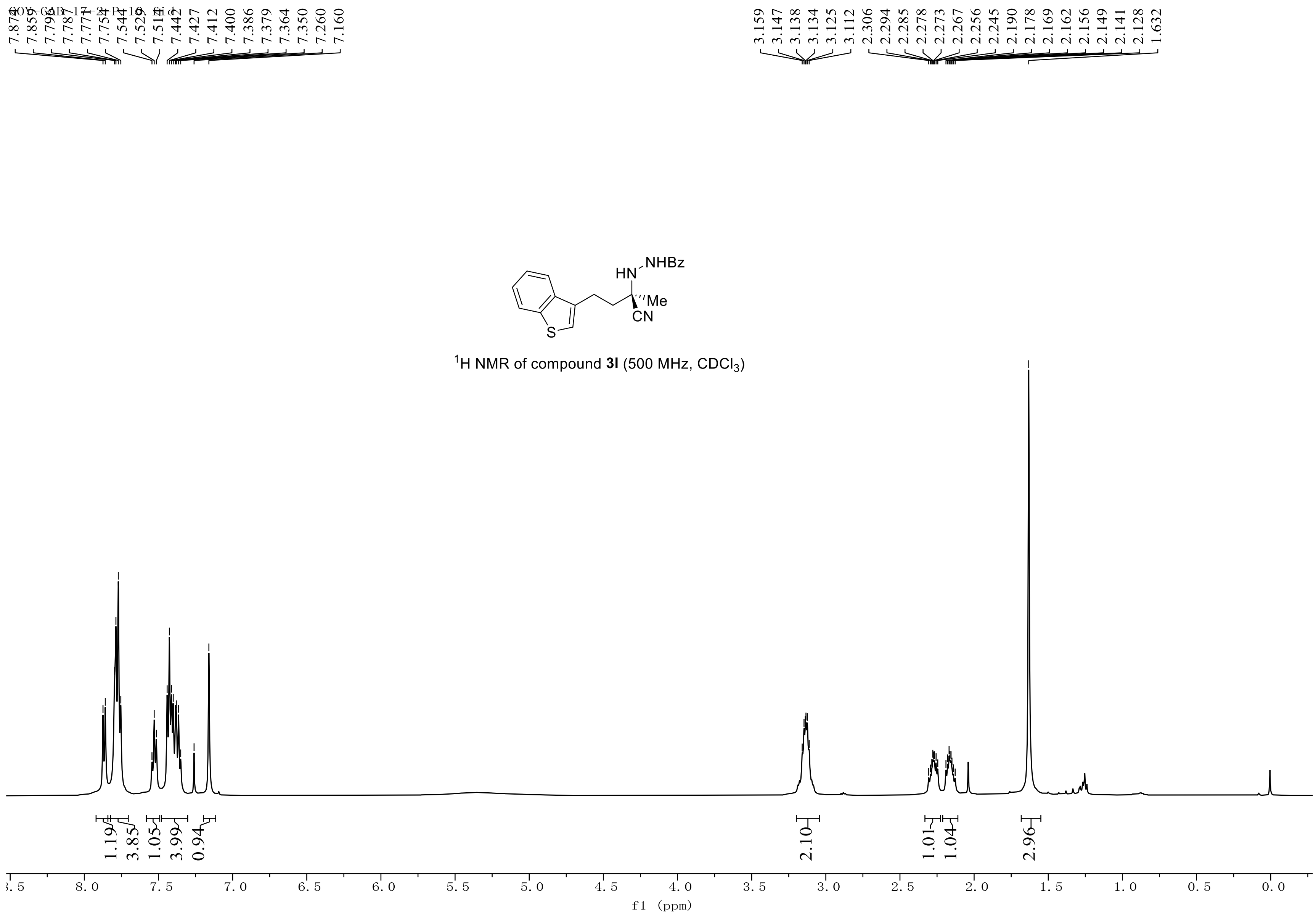

^{1}H NMR of compound **3l** (500 MHz, $CDCl_3$)

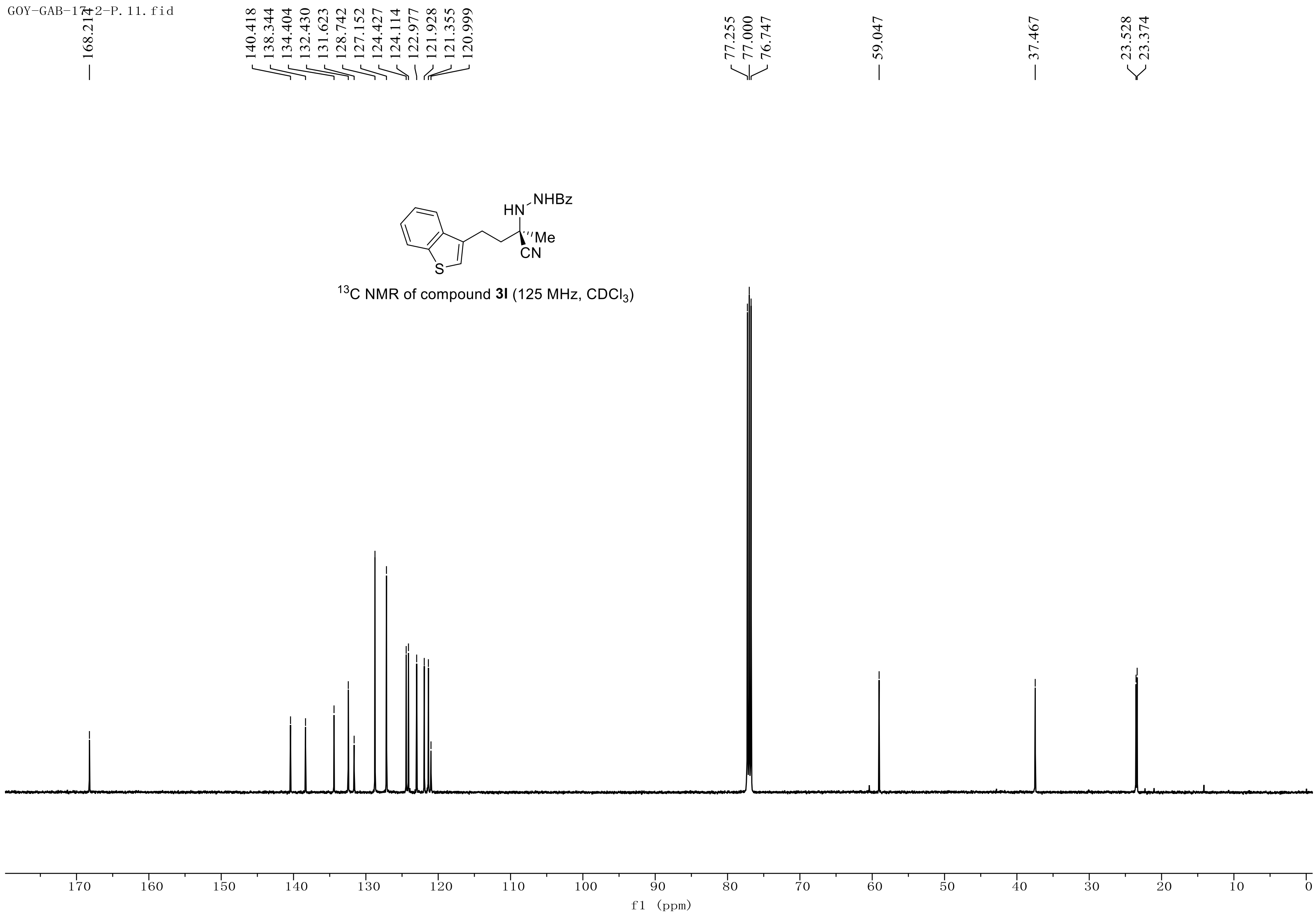

^{13}C NMR of compound **3l** (125 MHz, $CDCl_3$)

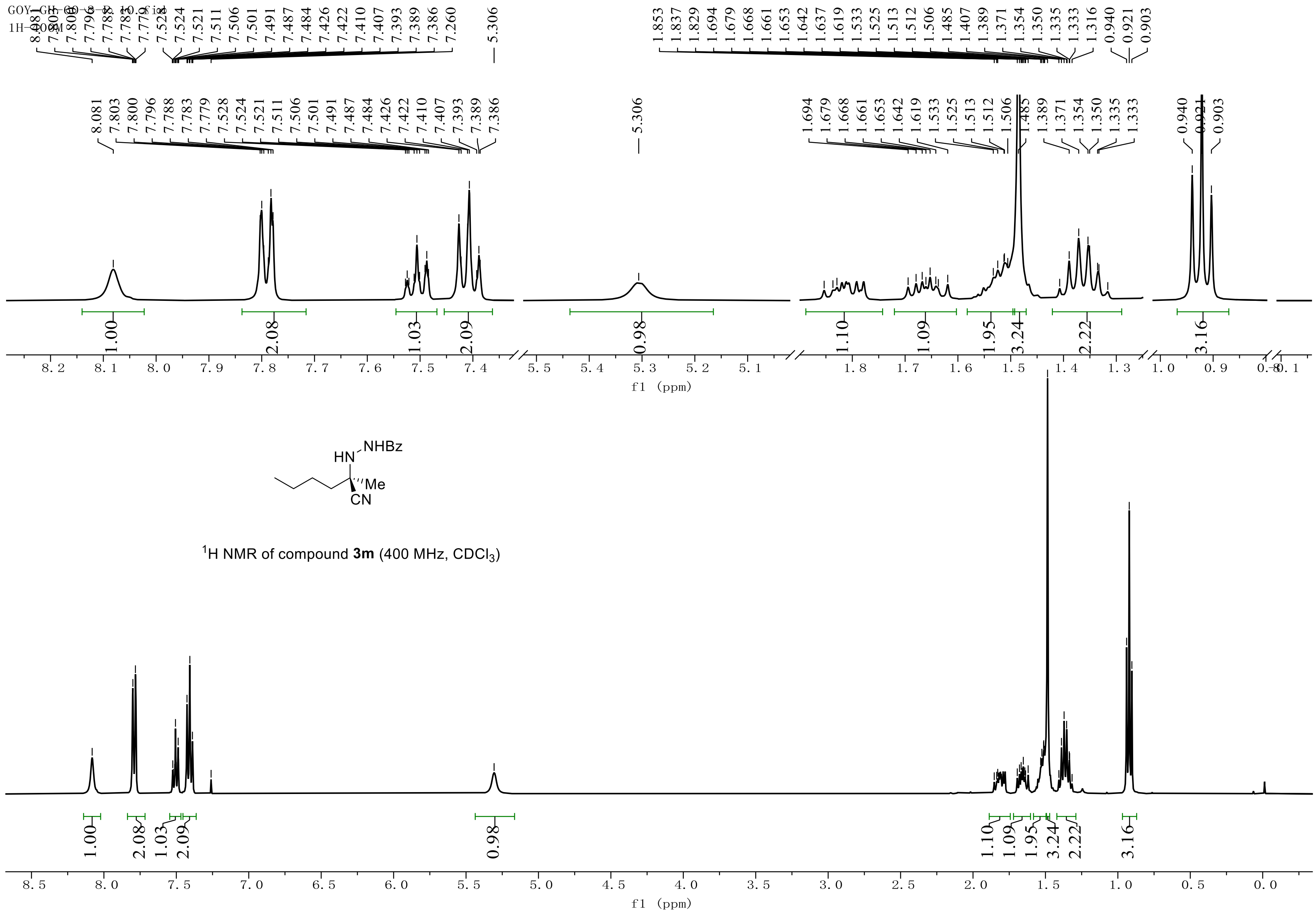

^{1}H NMR of compound **3m** (400 MHz, $CDCl_3$)

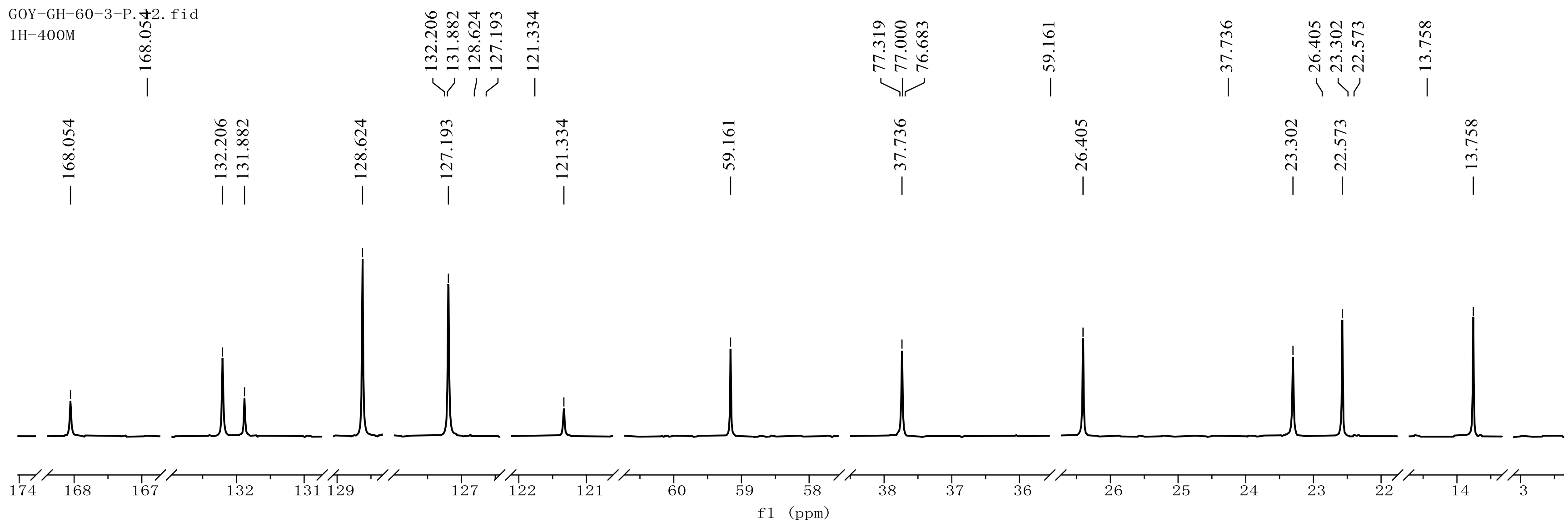

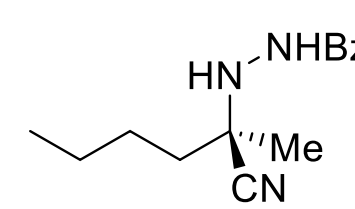

^{13}C NMR of compound **3m** (100 MHz, $CDCl_3$)

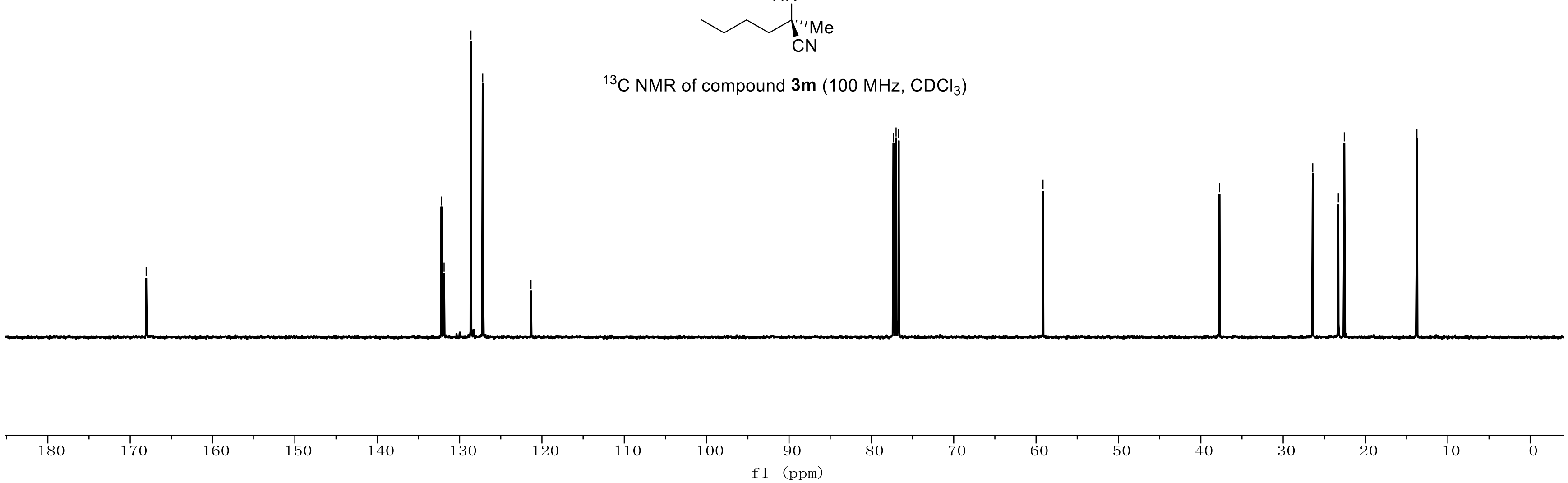

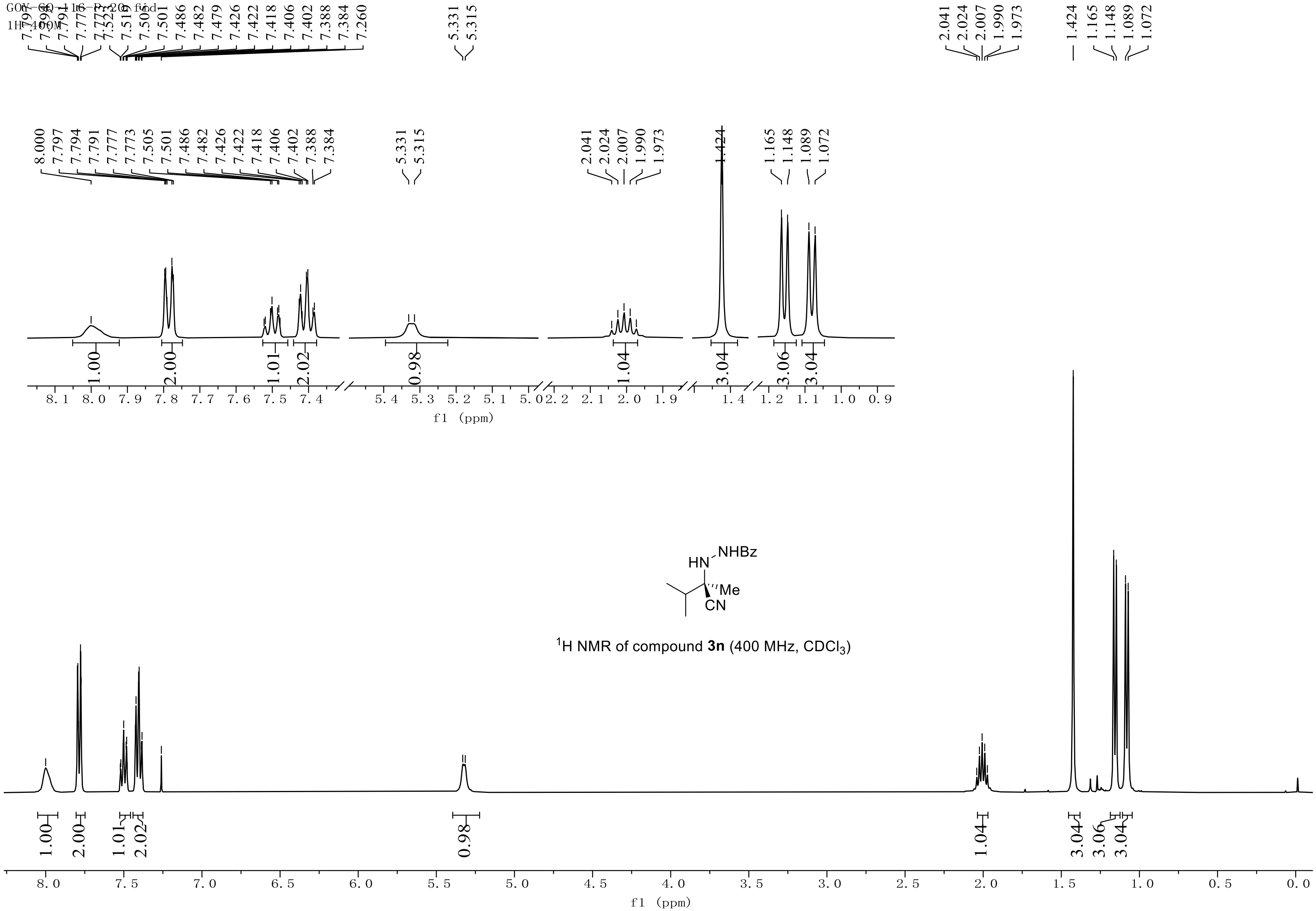

^{1}H NMR of compound **3n** (400 MHz, $CDCl_3$)

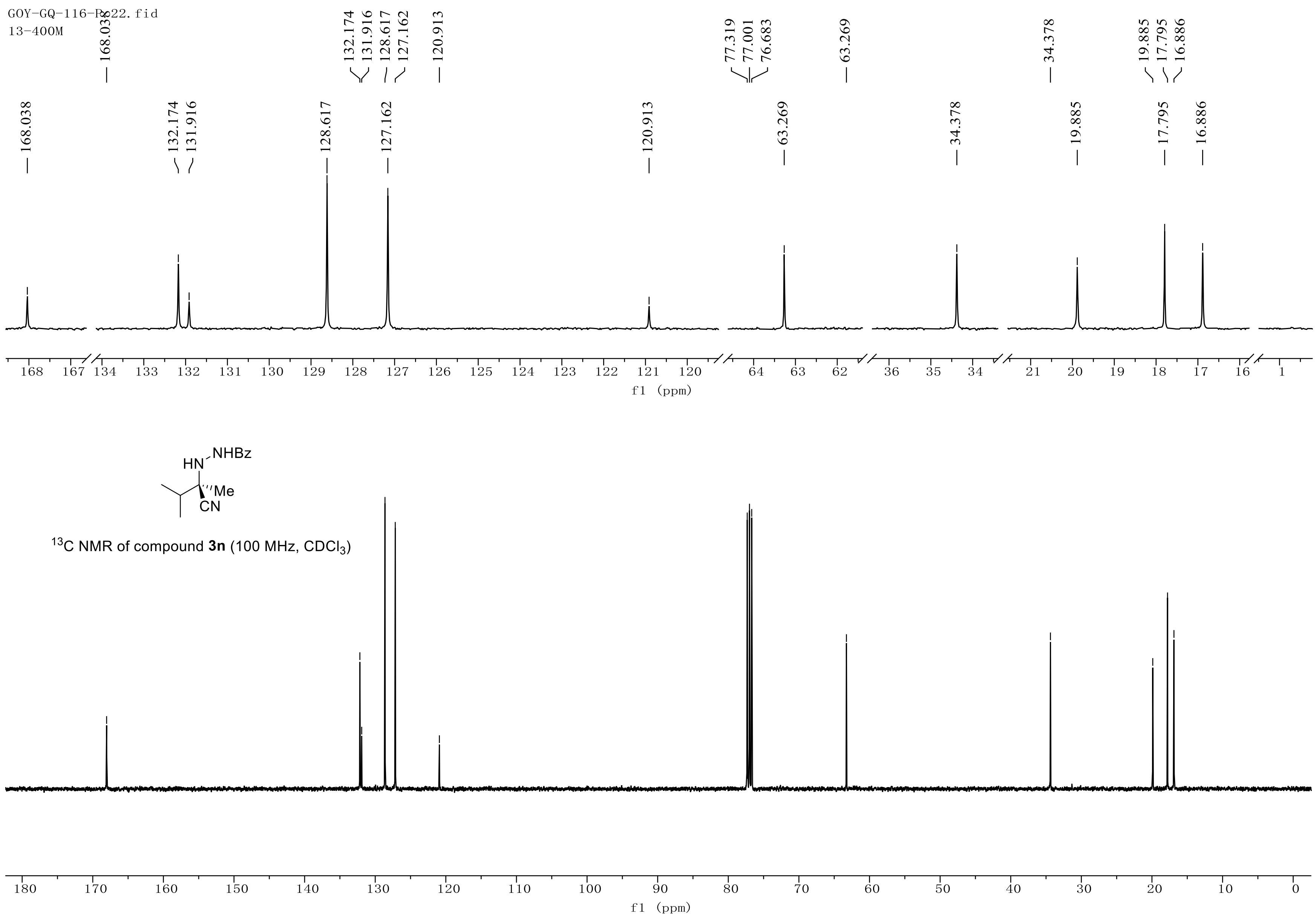

^{13}C NMR of compound **3n** (100 MHz, $CDCl_3$)

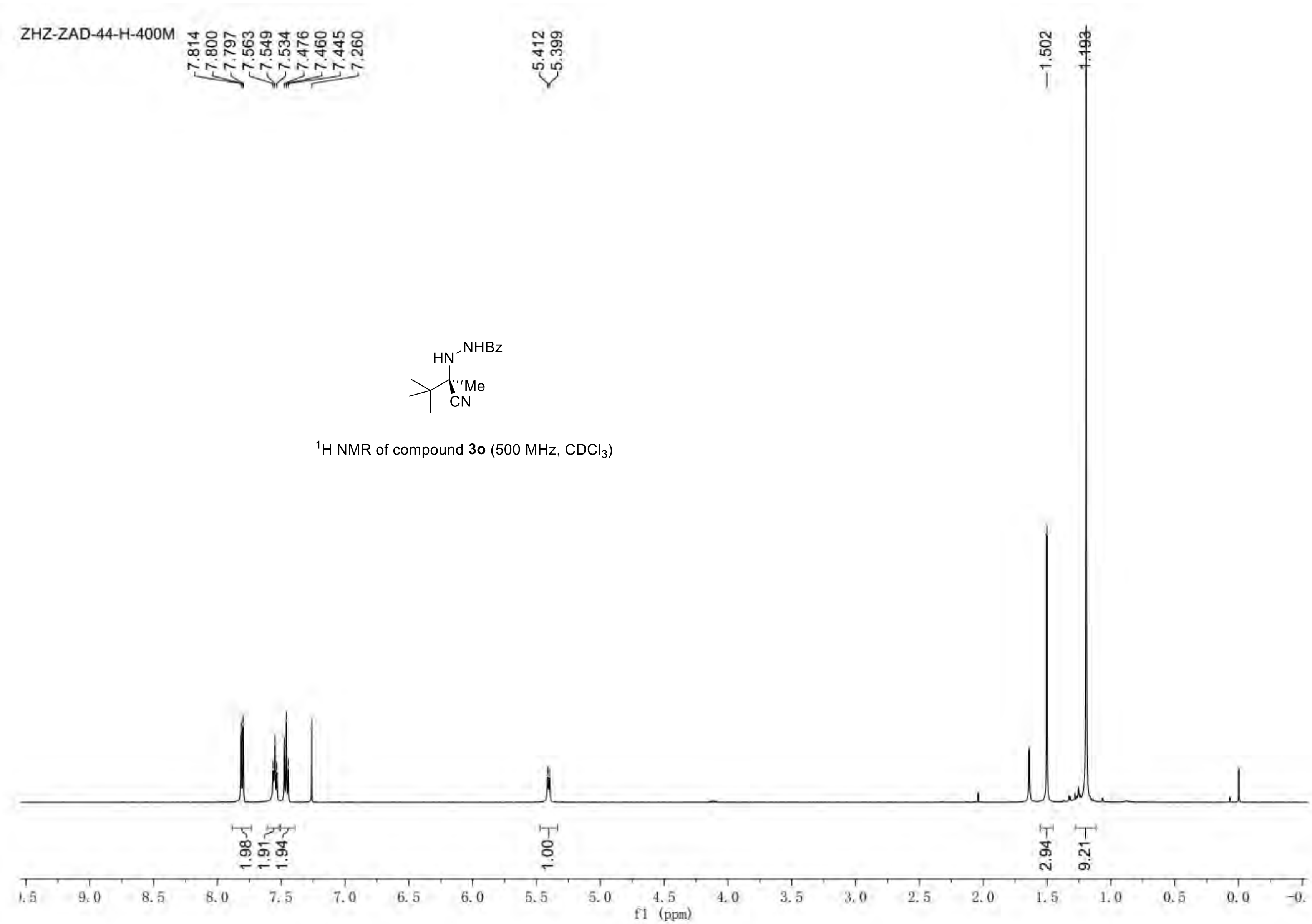

^{1}H NMR of compound **3o** (500 MHz, $CDCl_3$)

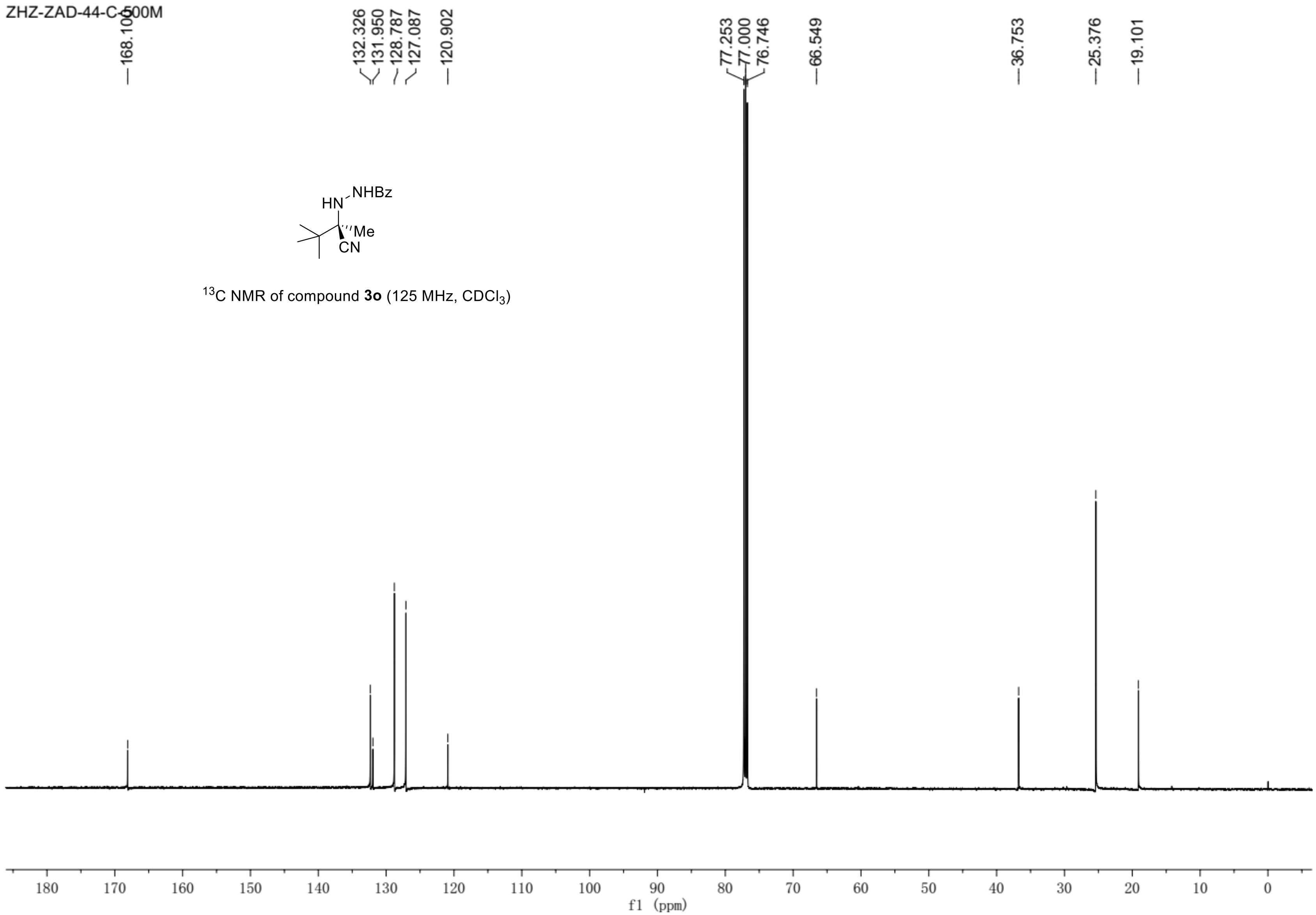
ZHZ-ZAD-44-C-500M
168.100
132.326
131.950
128.787
127.087
120.902
77.253
77.000
76.746
66.549
36.753
25.376
19.101
HN
NHBz
Me
CN
^{13}C NMR of compound **3o** (125 MHz, $CDCl_3$)
180
170
160
150
140
130
120
110
100
90
80
70
60
50
40
30
20
10
0
f1 (ppm)

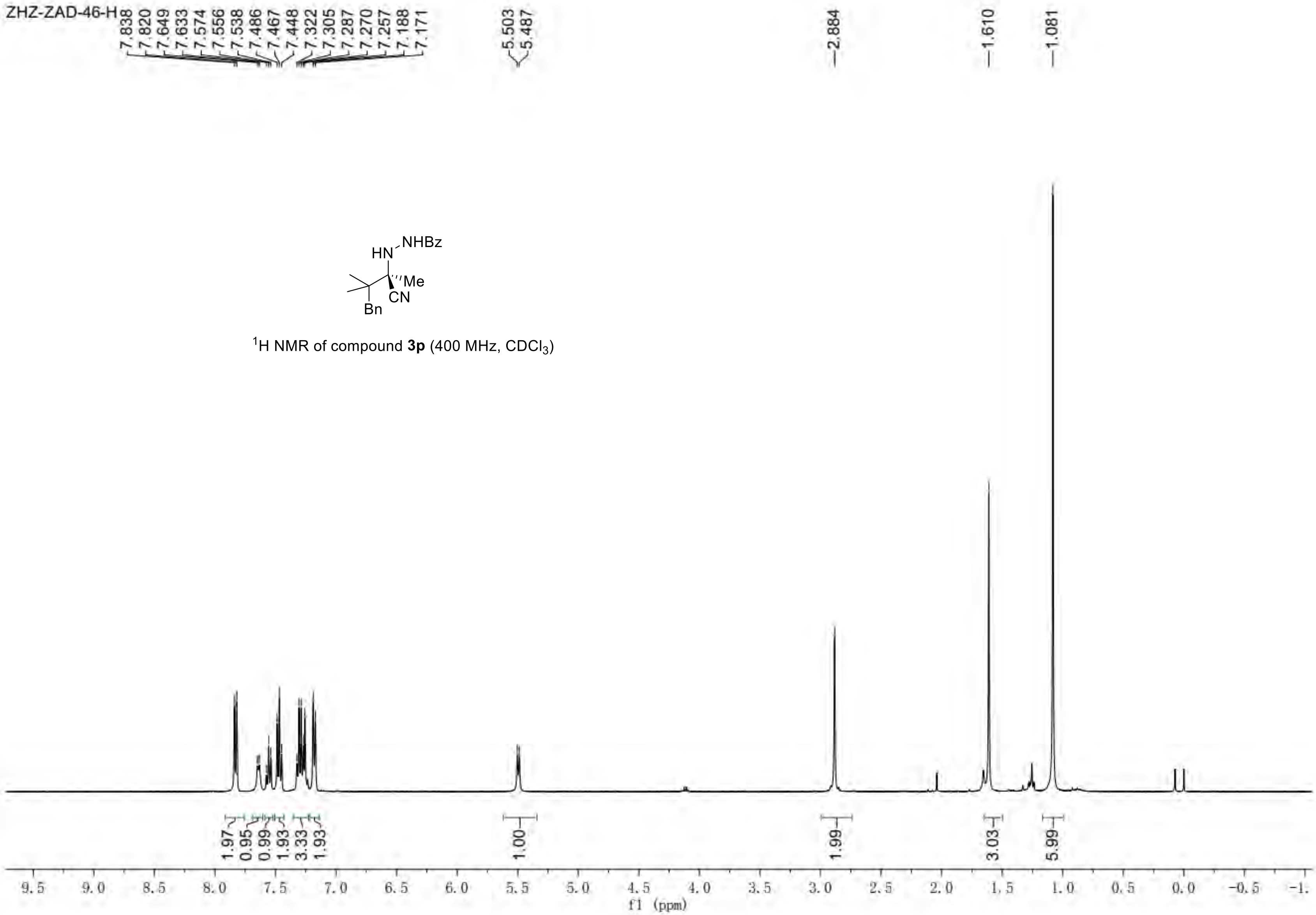

^{1}H NMR of compound **3p** (400 MHz, $CDCl_3$)

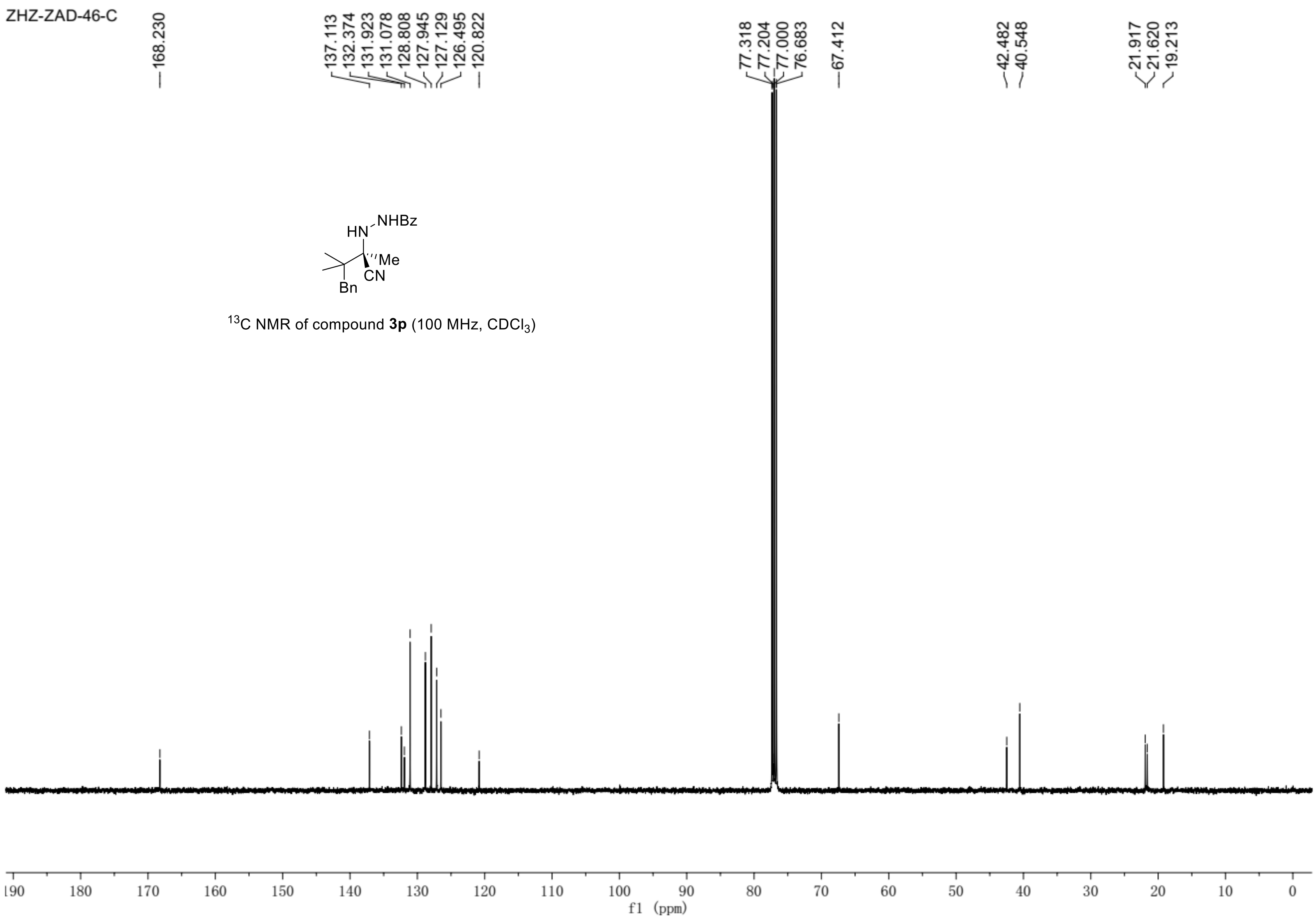

^{13}C NMR of compound **3p** (100 MHz, $CDCl_3$)

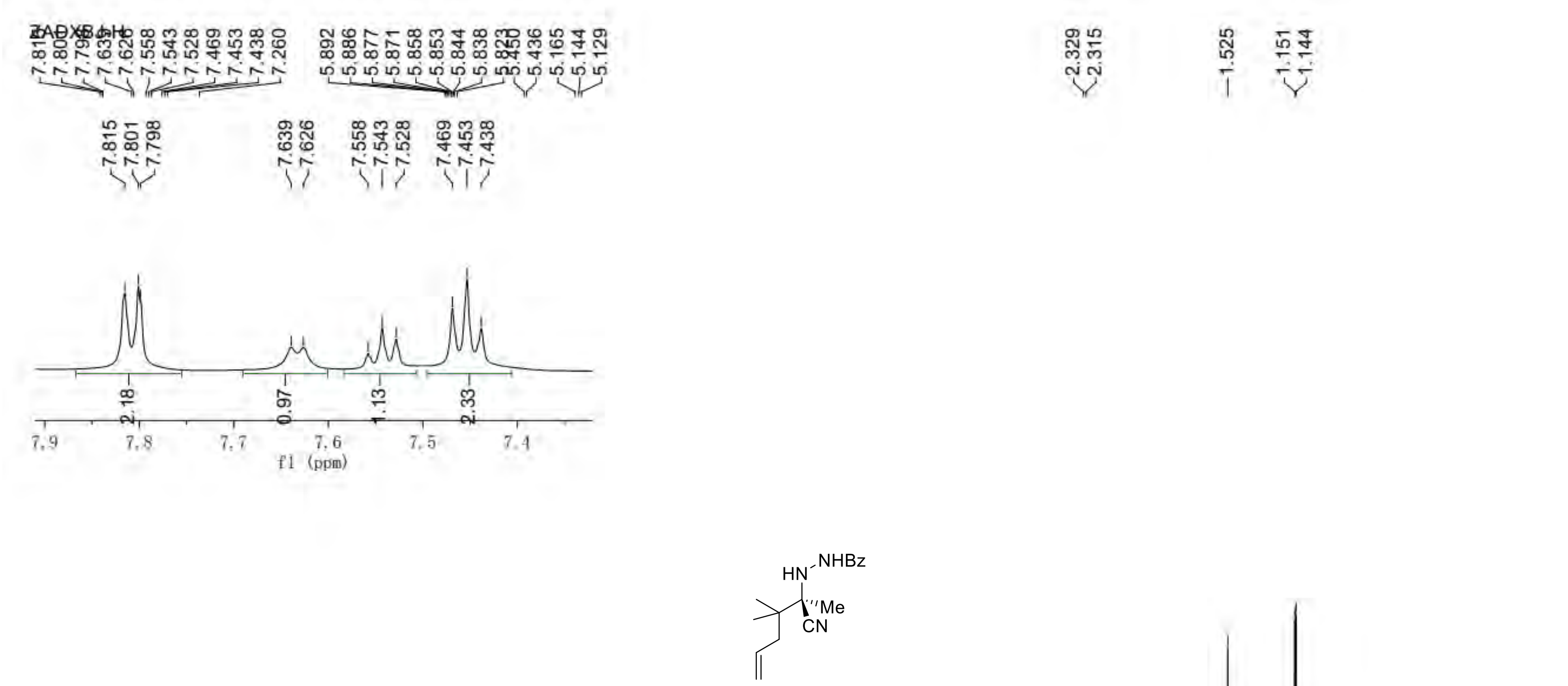

^{1}H NMR of compound **3q** (500 MHz, $CDCl_3$)

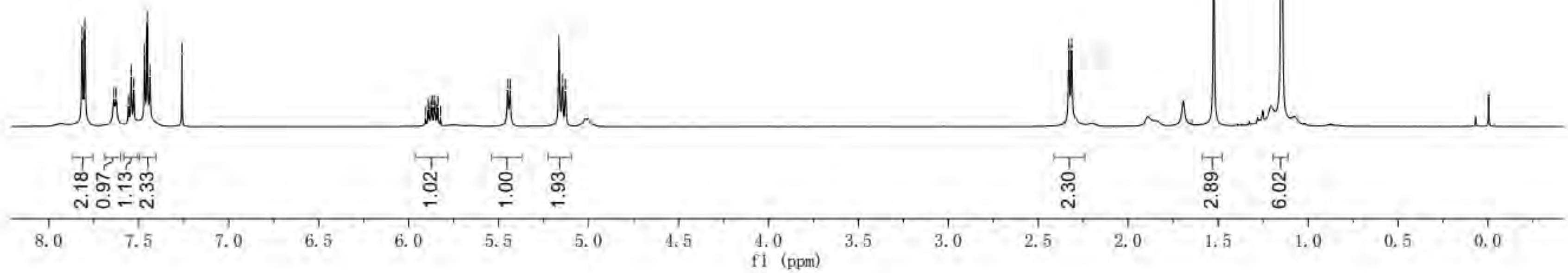

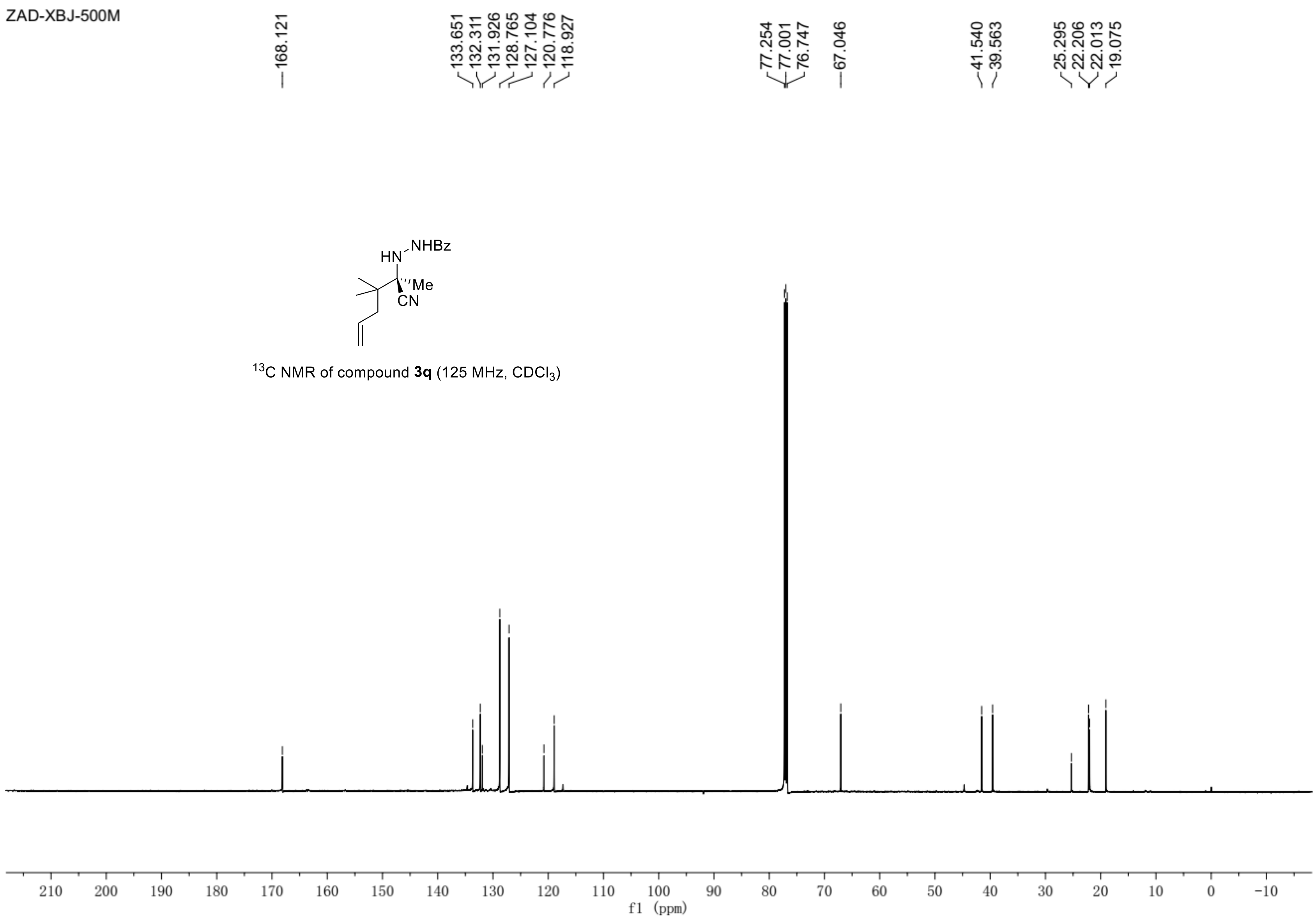

^{13}C NMR of compound **3q** (125 MHz, $CDCl_3$)

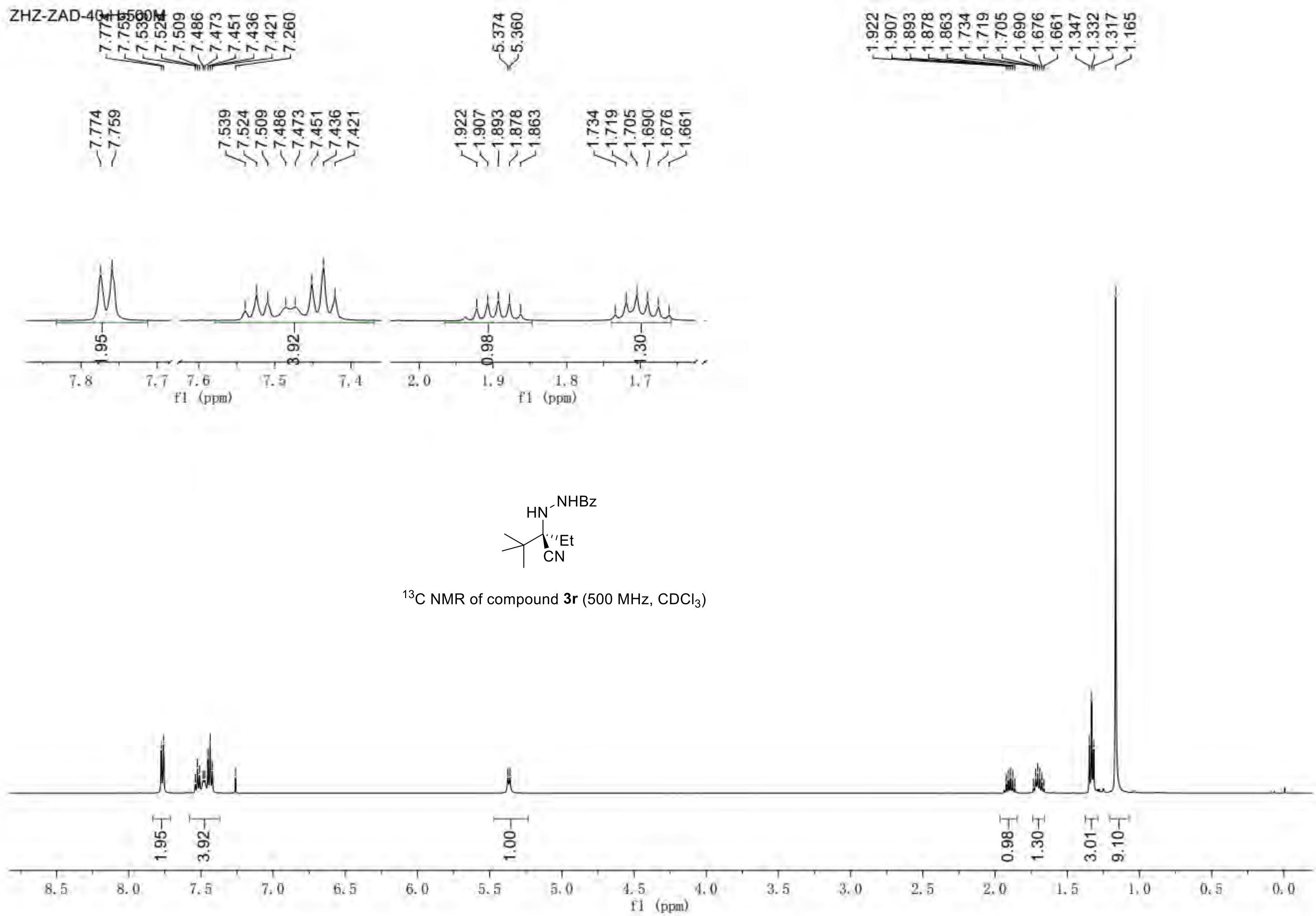

^{13}C NMR of compound **3r** (500 MHz, $CDCl_3$)

ZHZ-ZAD-40-C-500M

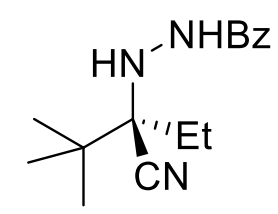

^{13}C NMR of compound **3r** (125 MHz, $CDCl_3$)

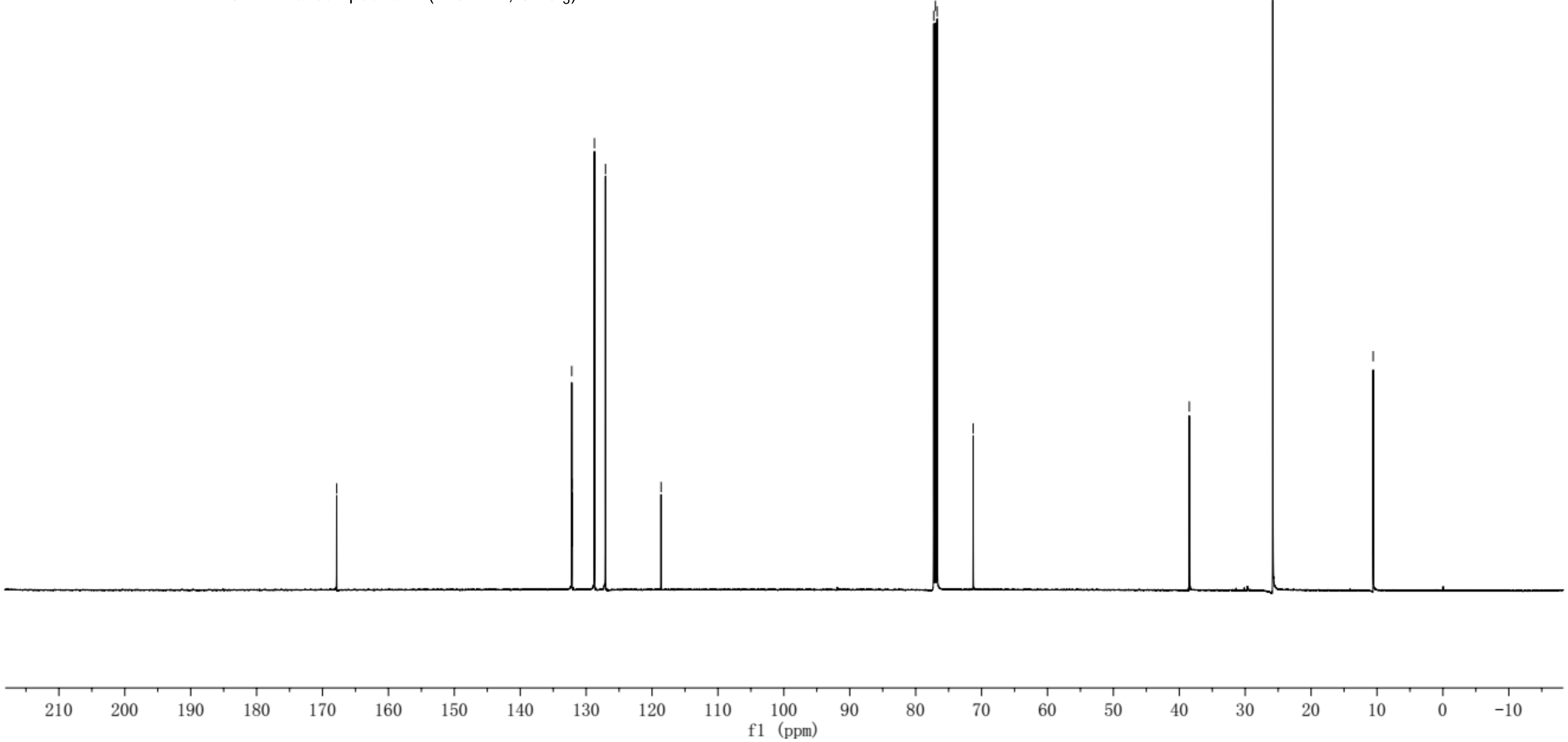

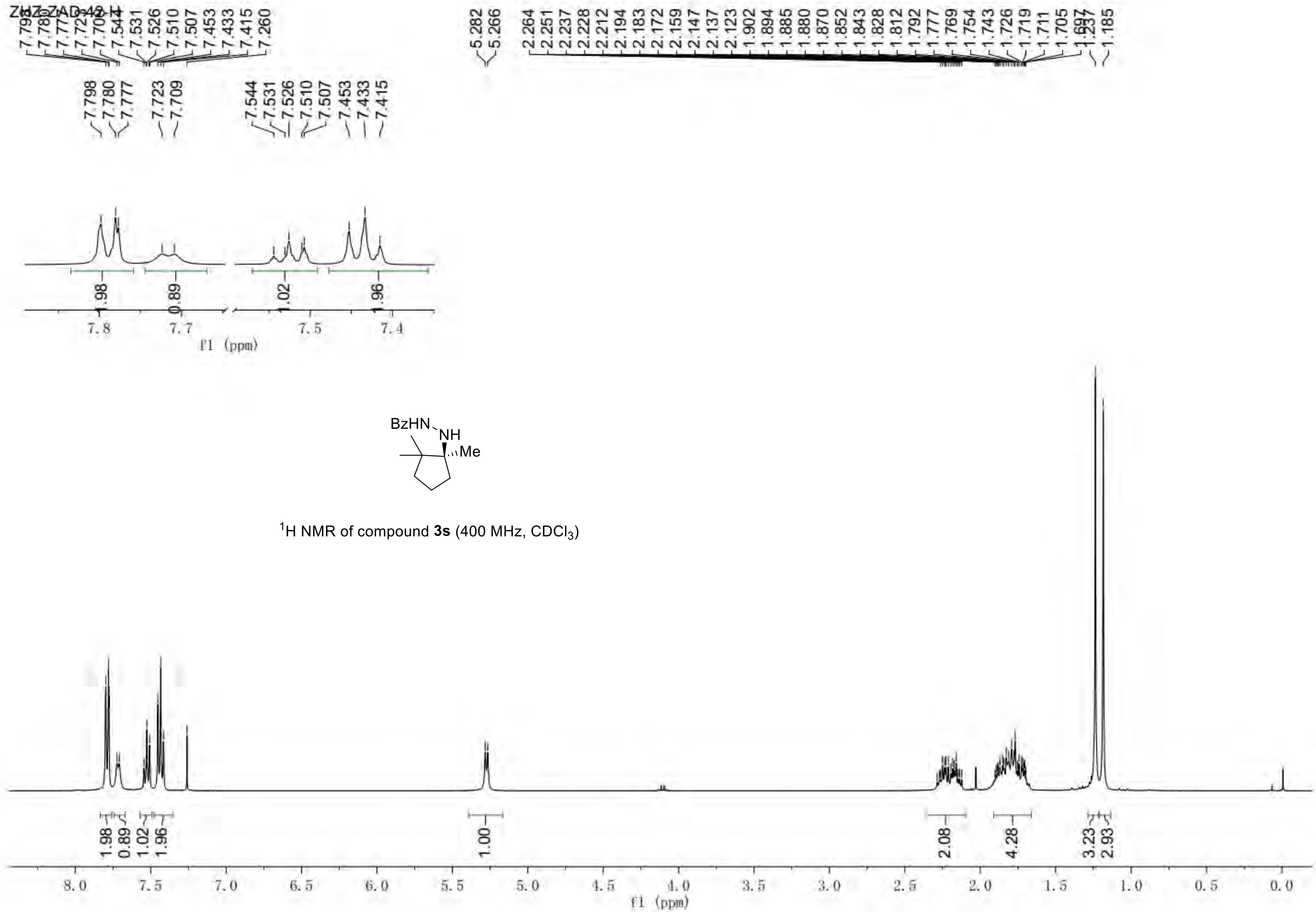

^{1}H NMR of compound **3s** (400 MHz, $CDCl_3$)

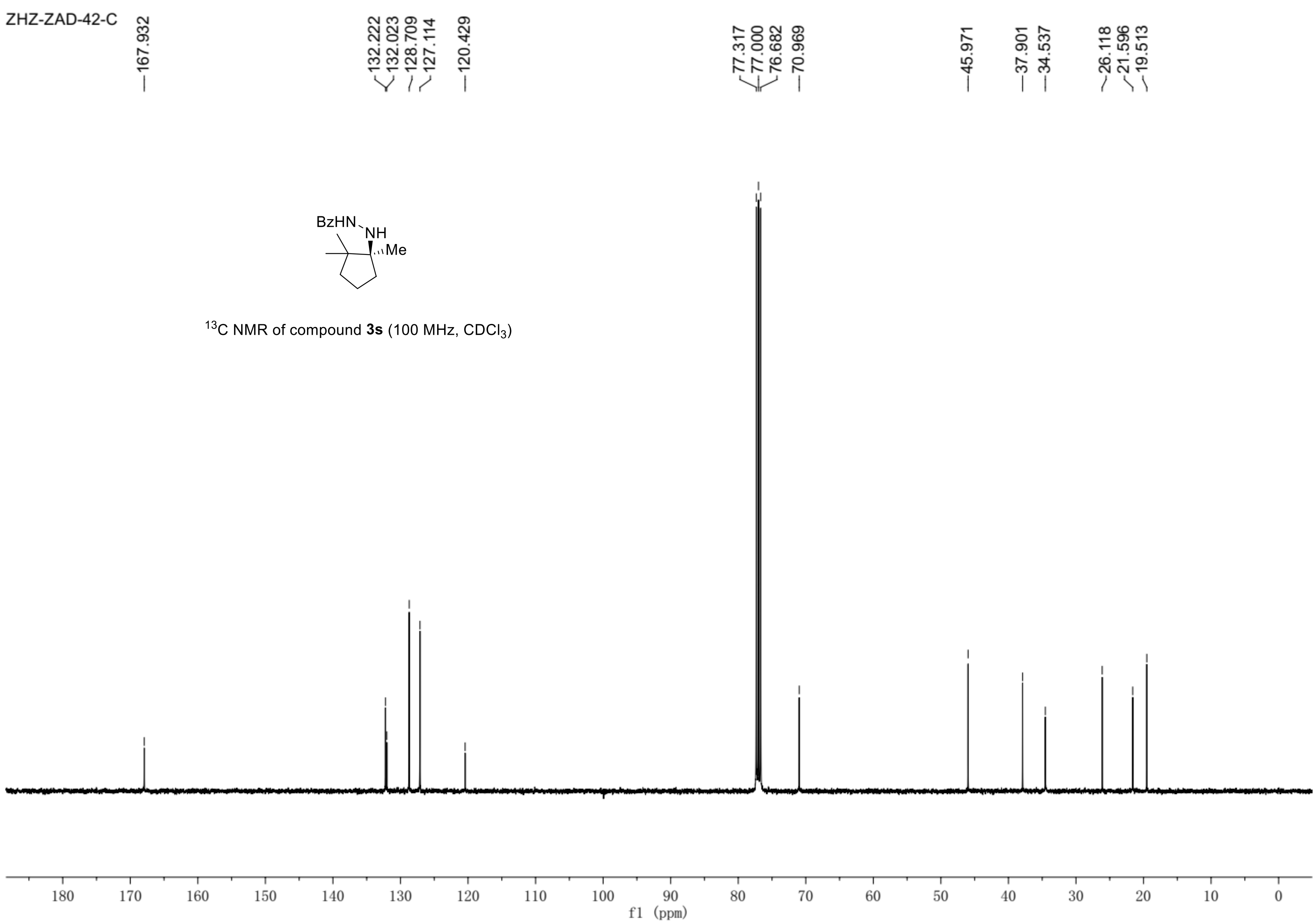

^{13}C NMR of compound **3s** (100 MHz, $CDCl_3$)

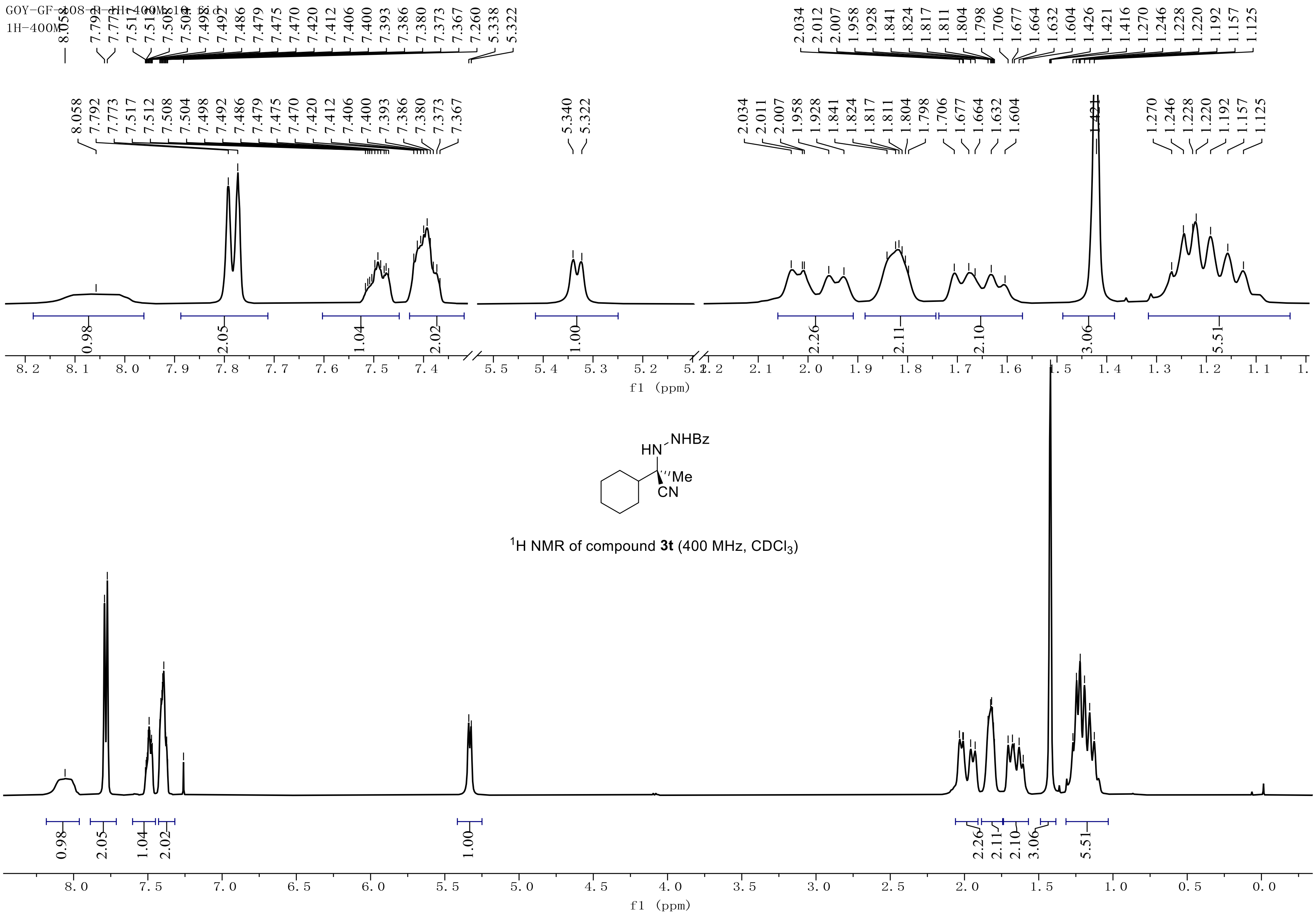

^{1}H NMR of compound **3t** (400 MHz, $CDCl_3$)

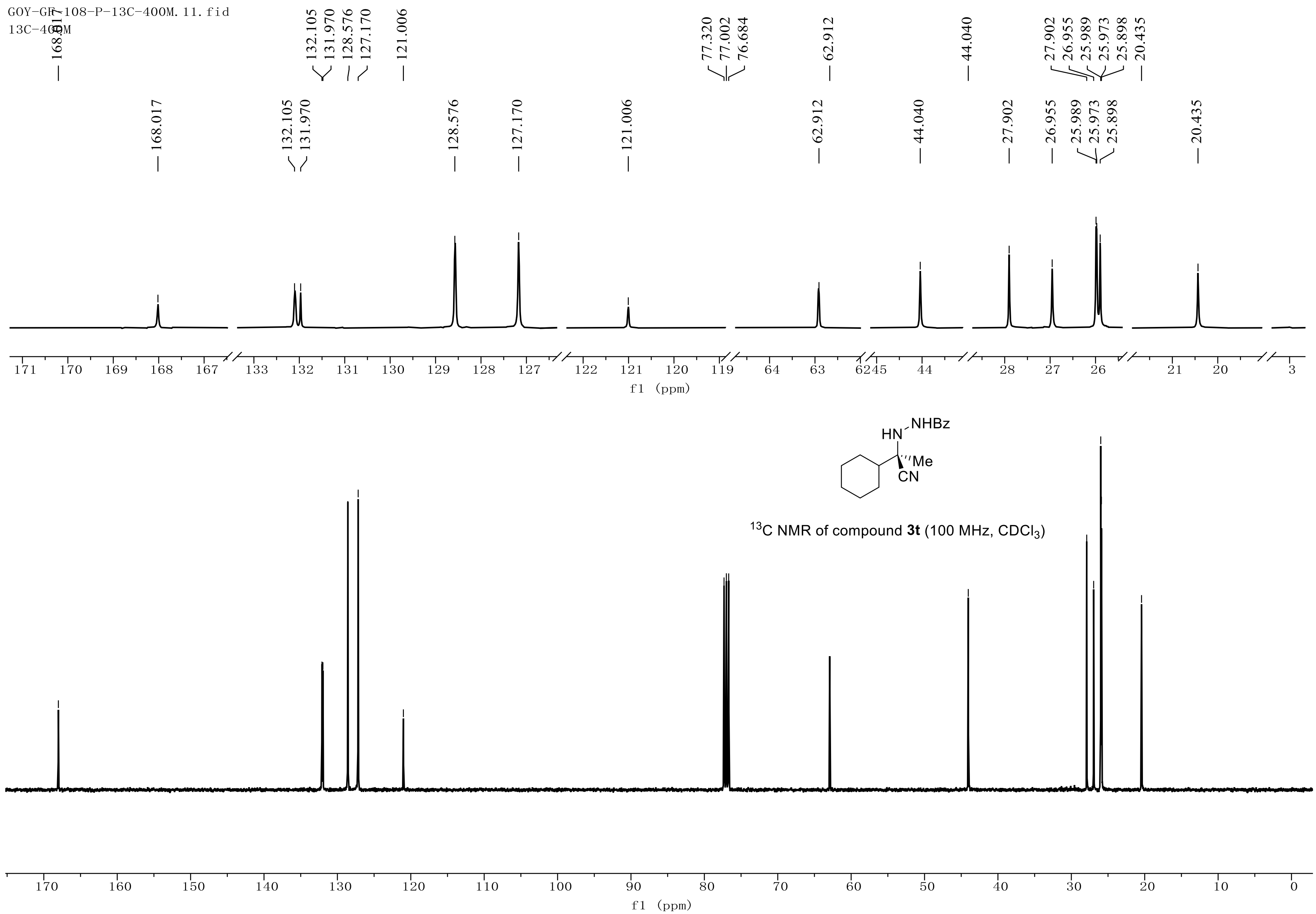

^{13}C NMR of compound **3t** (100 MHz, $CDCl_3$)

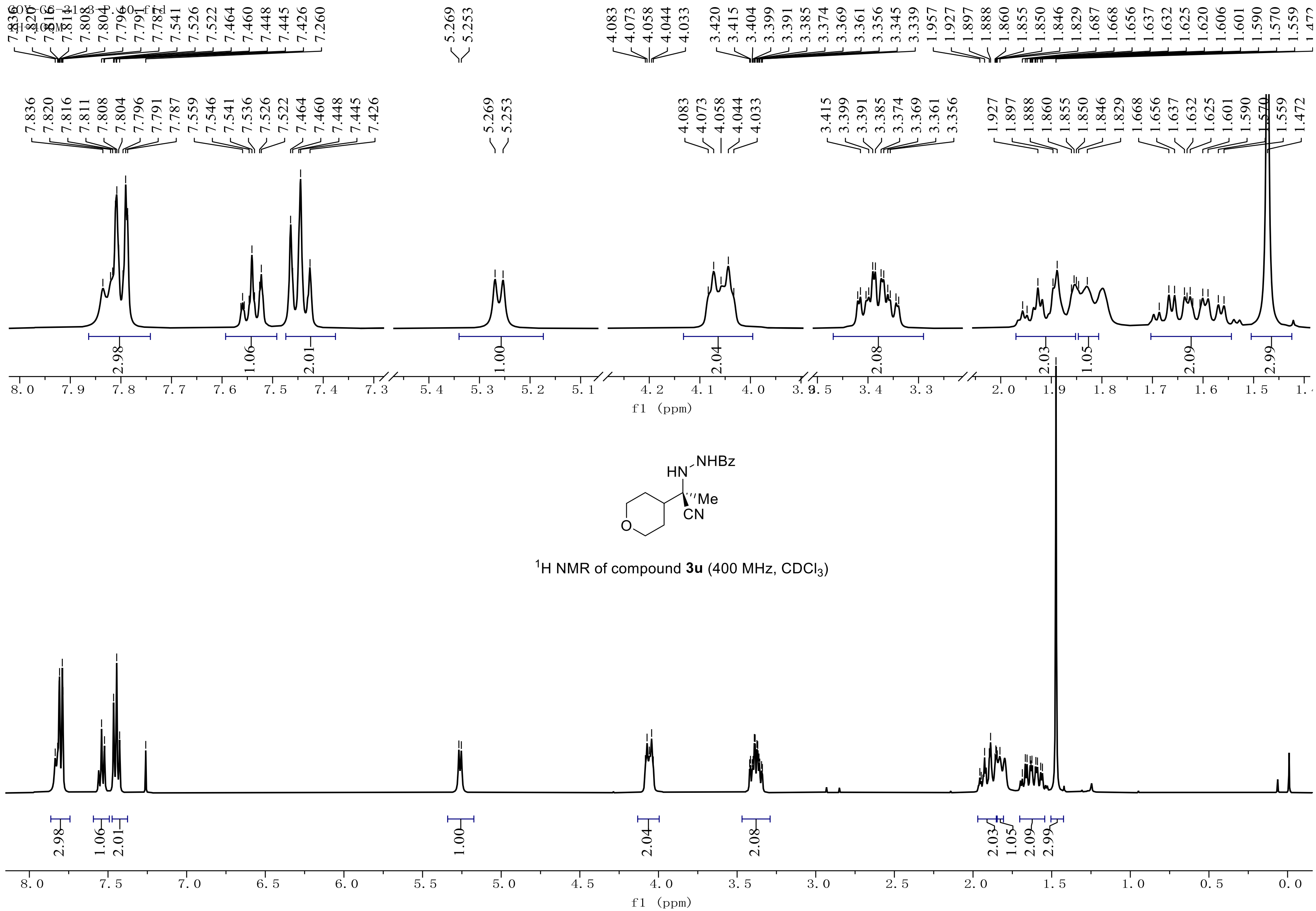

^{1}H NMR of compound **3u** (400 MHz, $CDCl_3$)

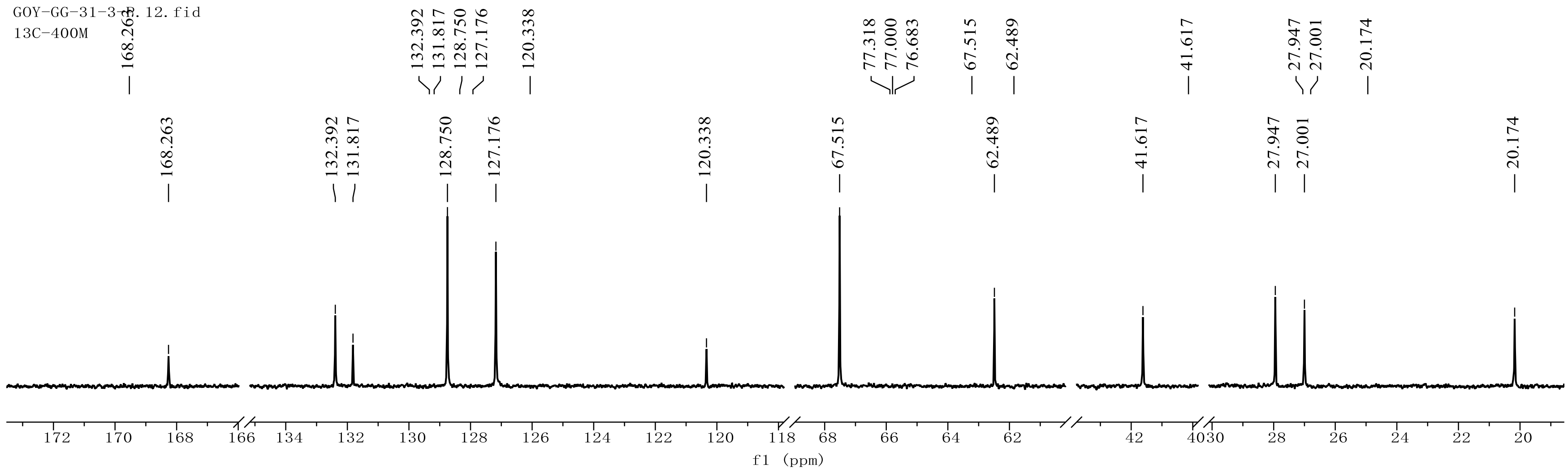

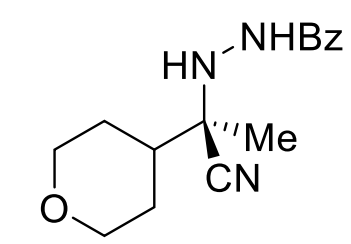

^{13}C NMR of compound **3u** (100 MHz, $CDCl_3$)

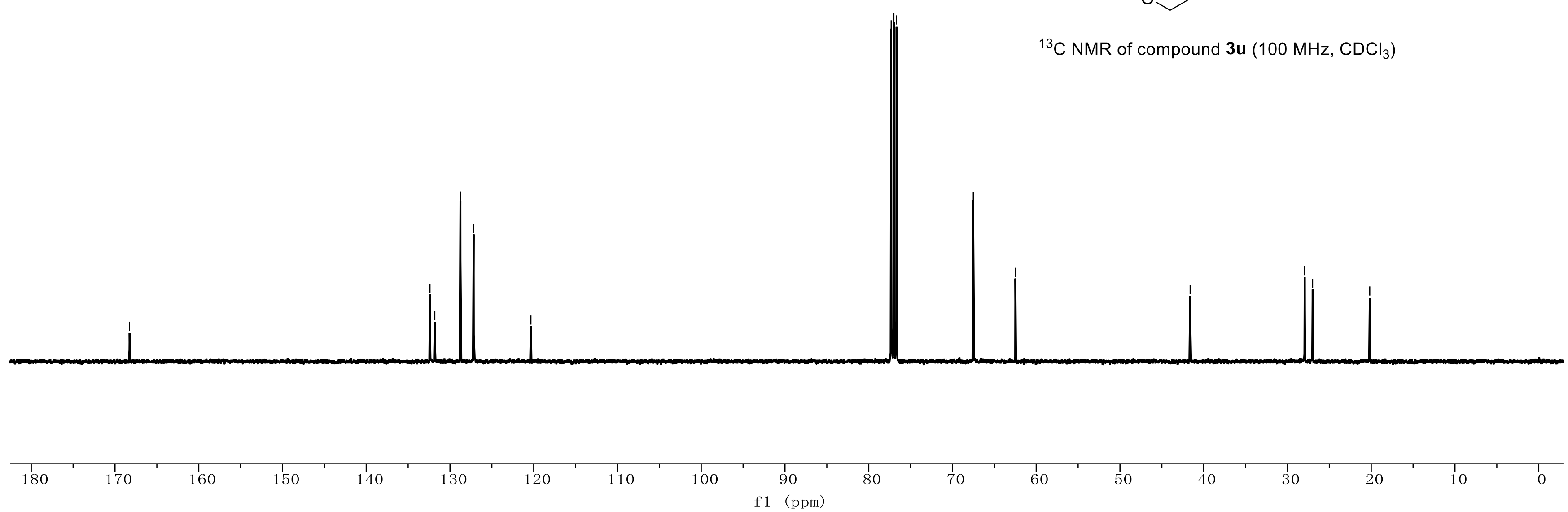

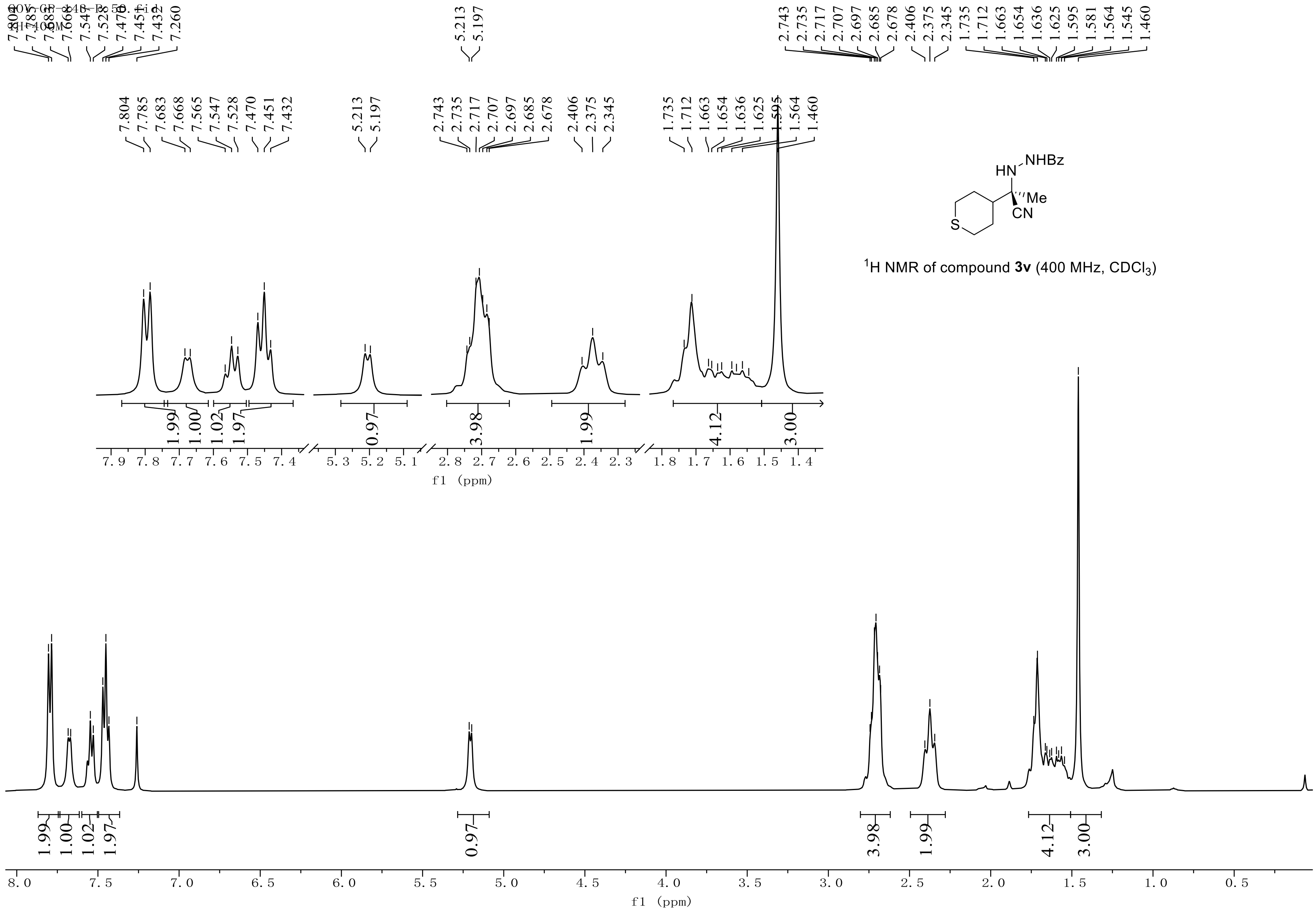

^{1}H NMR of compound **3v** (400 MHz, $CDCl_3$)

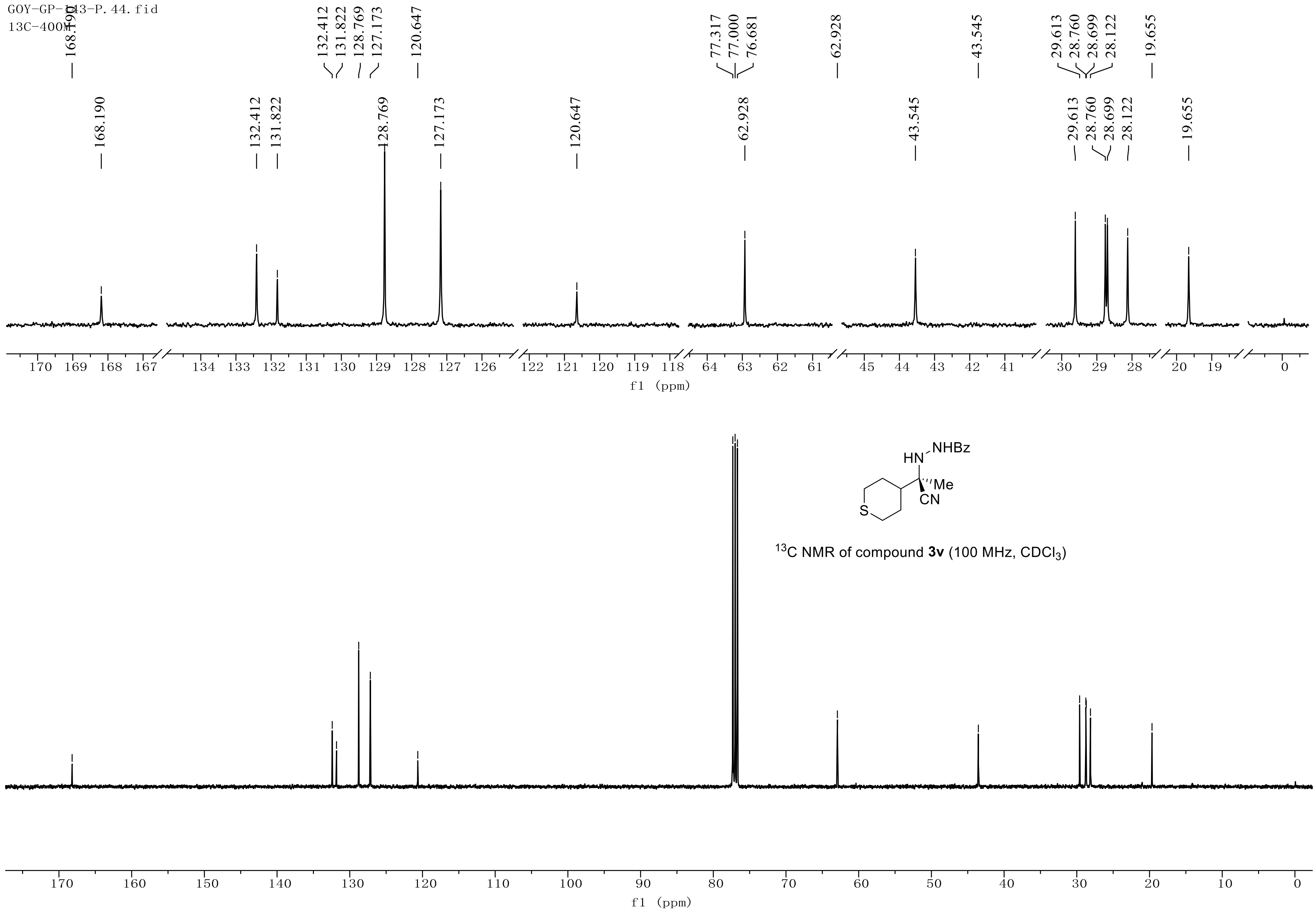

^{13}C NMR of compound **3v** (100 MHz, $CDCl_3$)

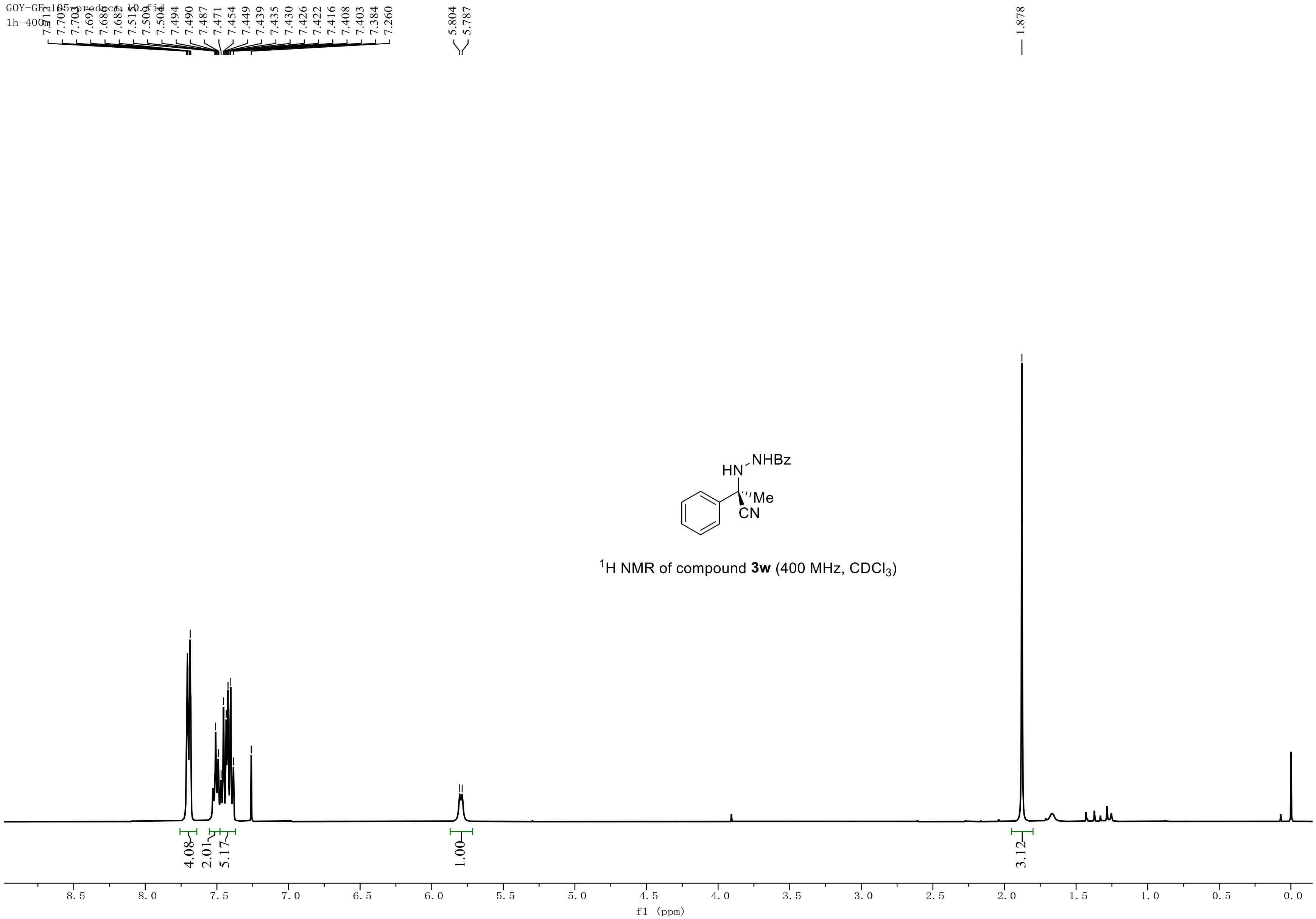

^{1}H NMR of compound **3w** (400 MHz, $CDCl_3$)

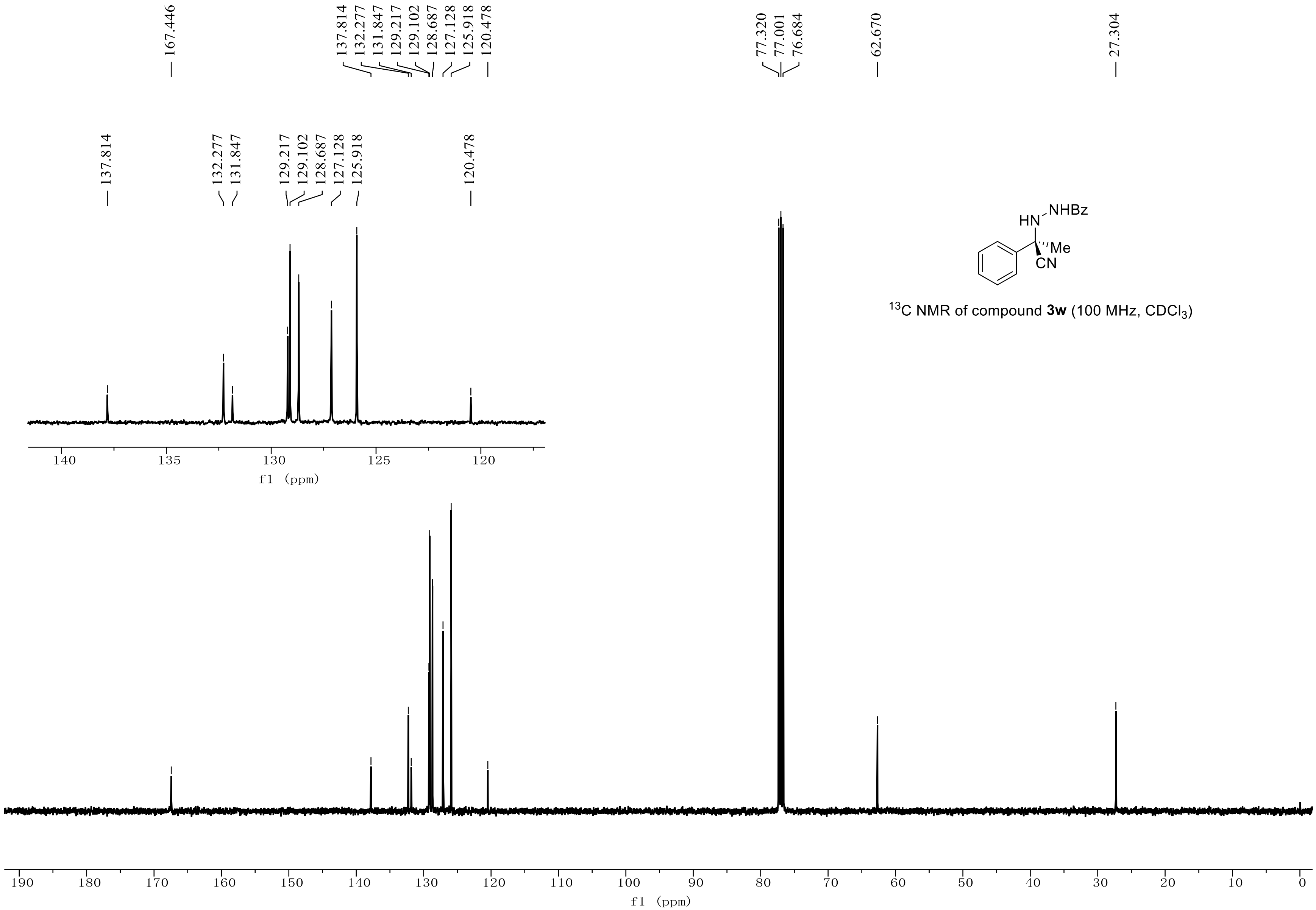

^{13}C NMR of compound **3w** (100 MHz, $CDCl_3$)

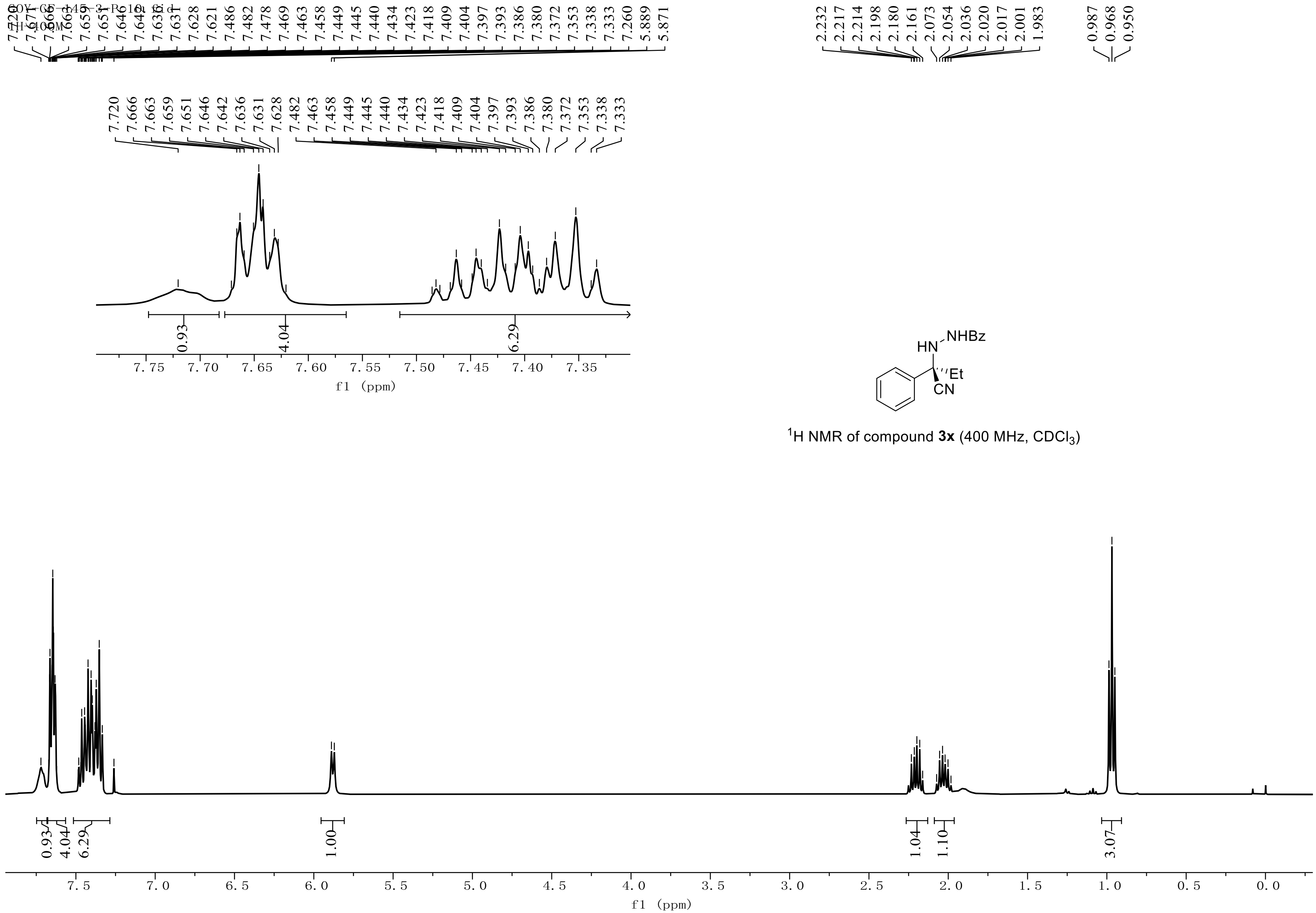

^{1}H NMR of compound **3x** (400 MHz, $CDCl_3$)

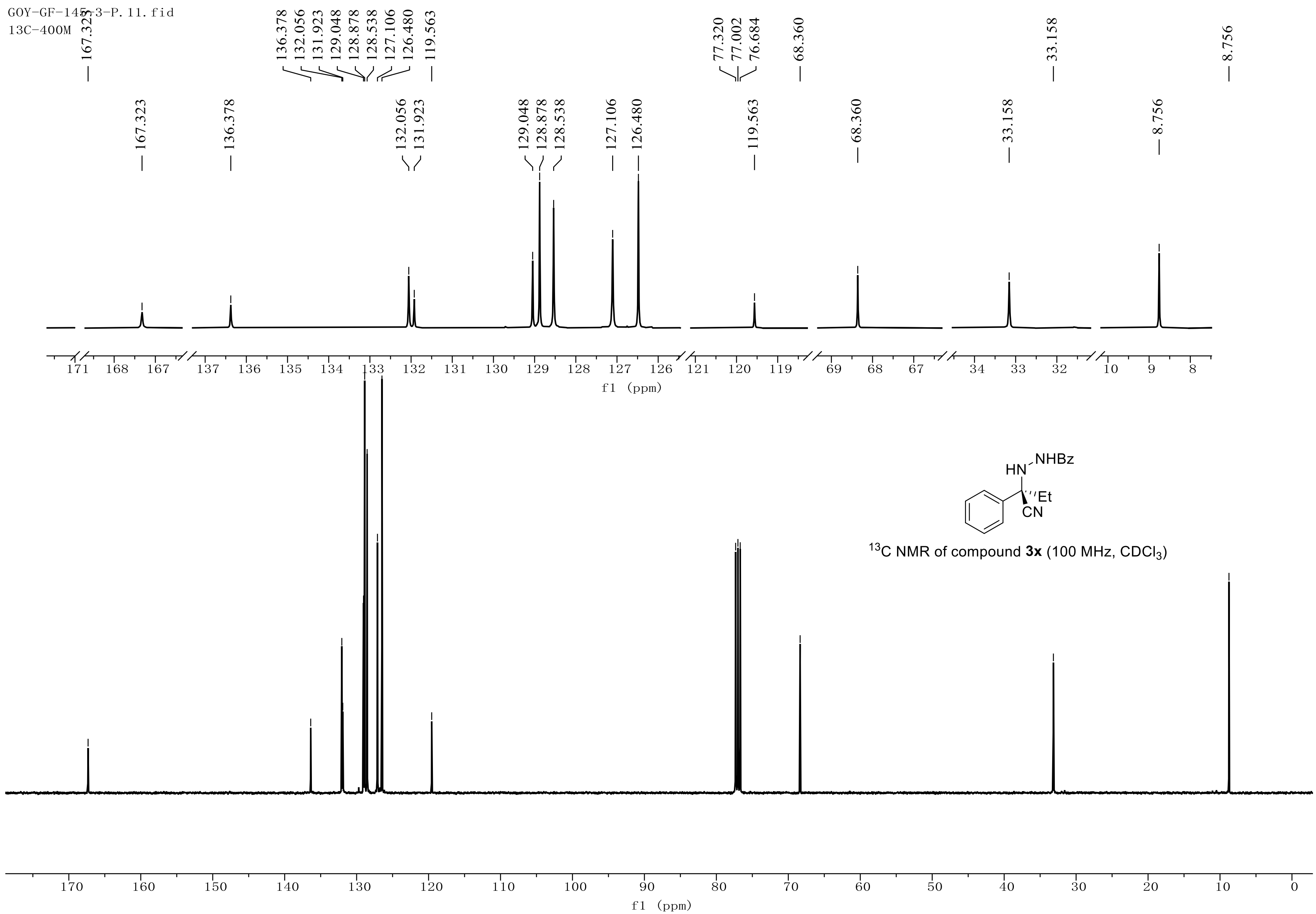

^{13}C NMR of compound **3x** (100 MHz, $CDCl_3$)

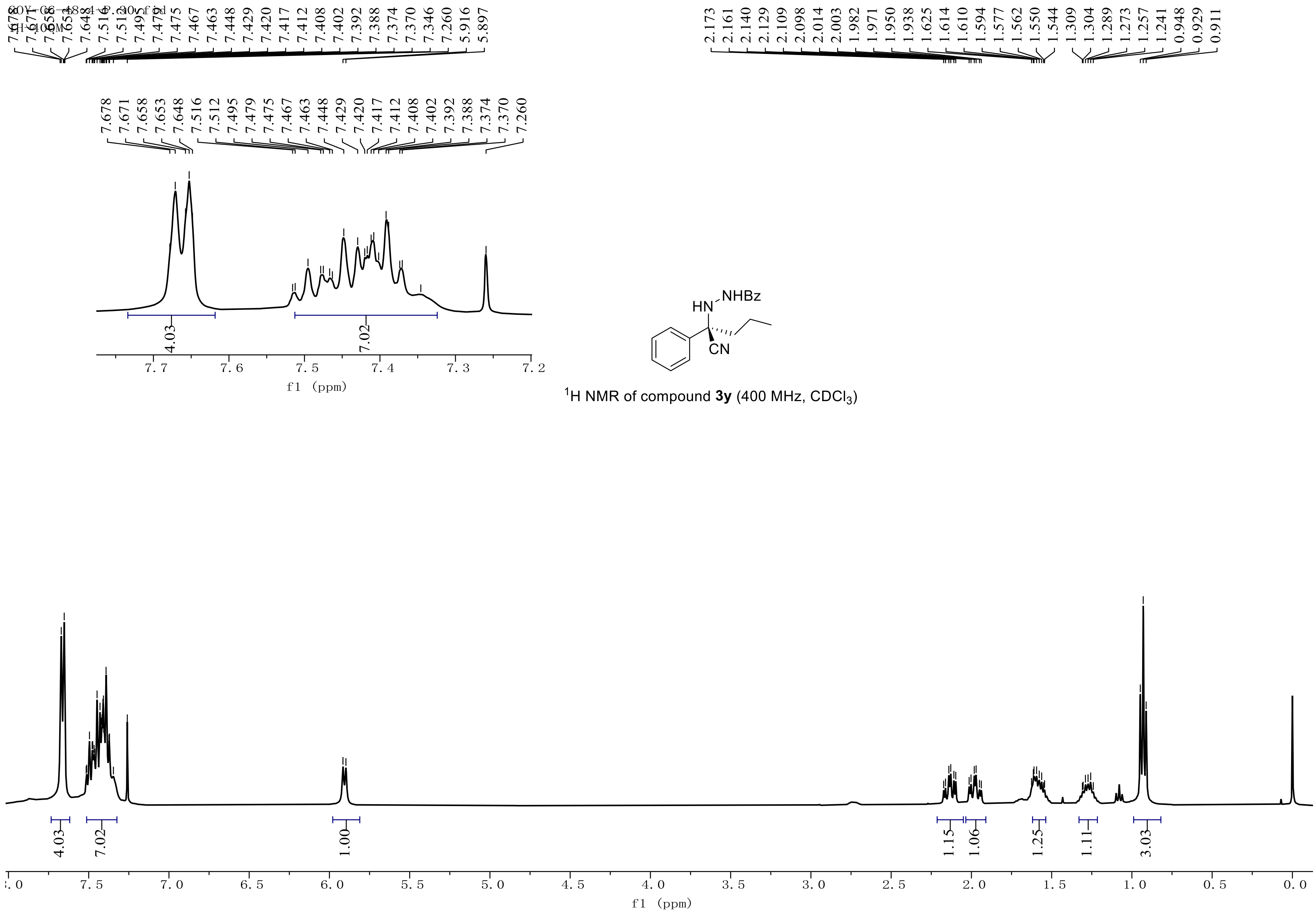

^{1}H NMR of compound **3y** (400 MHz, $CDCl_3$)

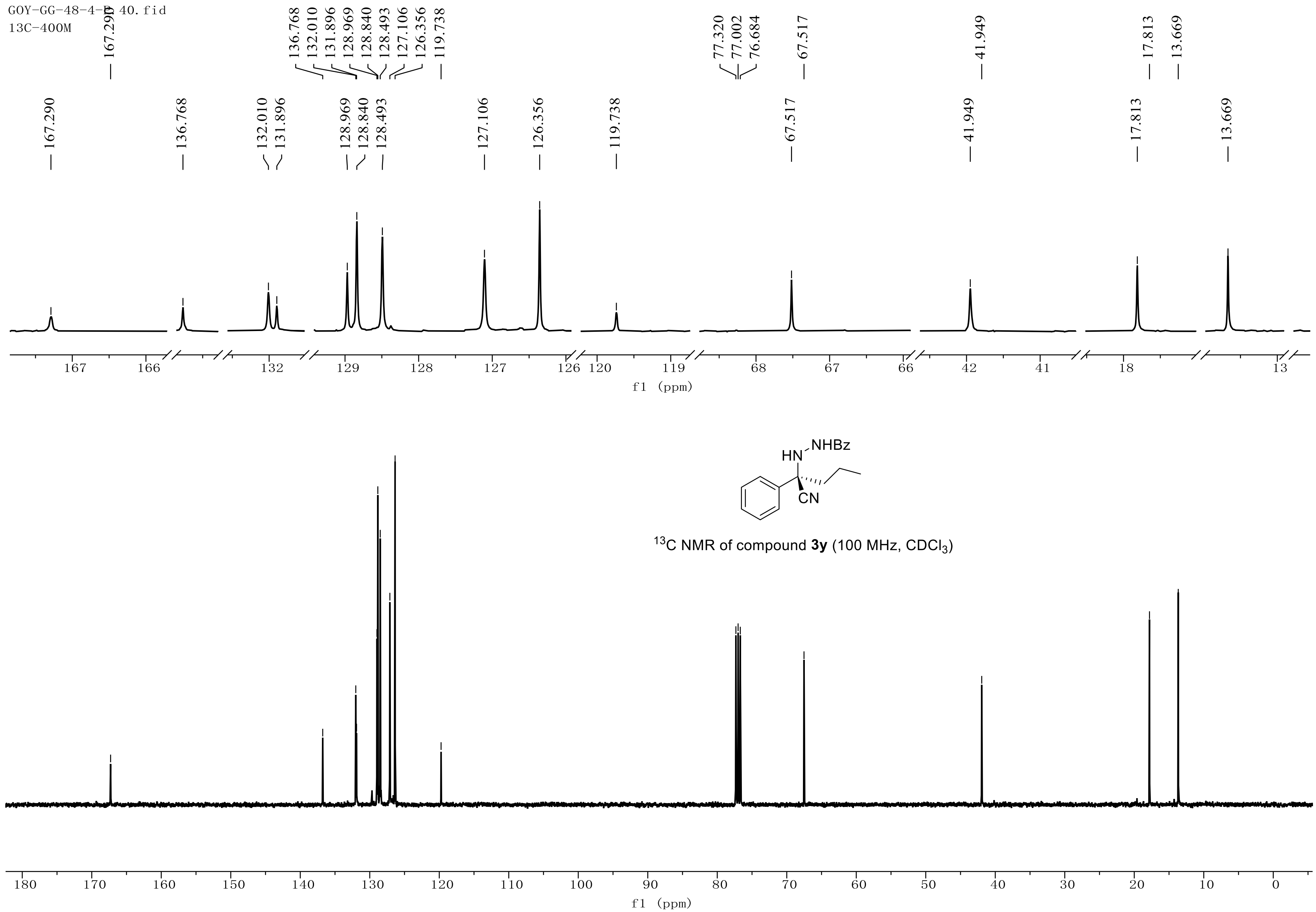

^{13}C NMR of compound **3y** (100 MHz, $CDCl_3$)

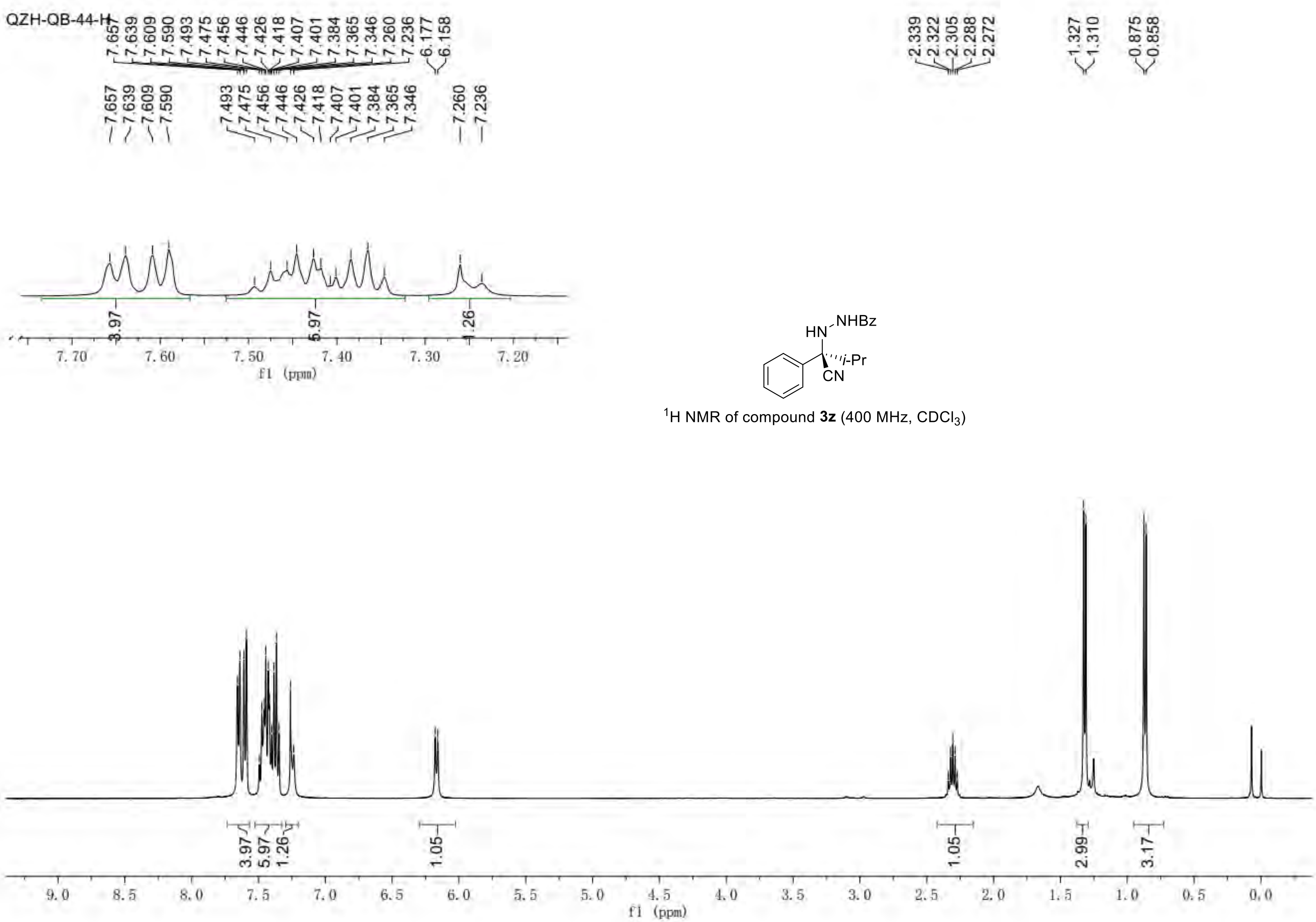

^{1}H NMR of compound **3z** (400 MHz, $CDCl_3$)

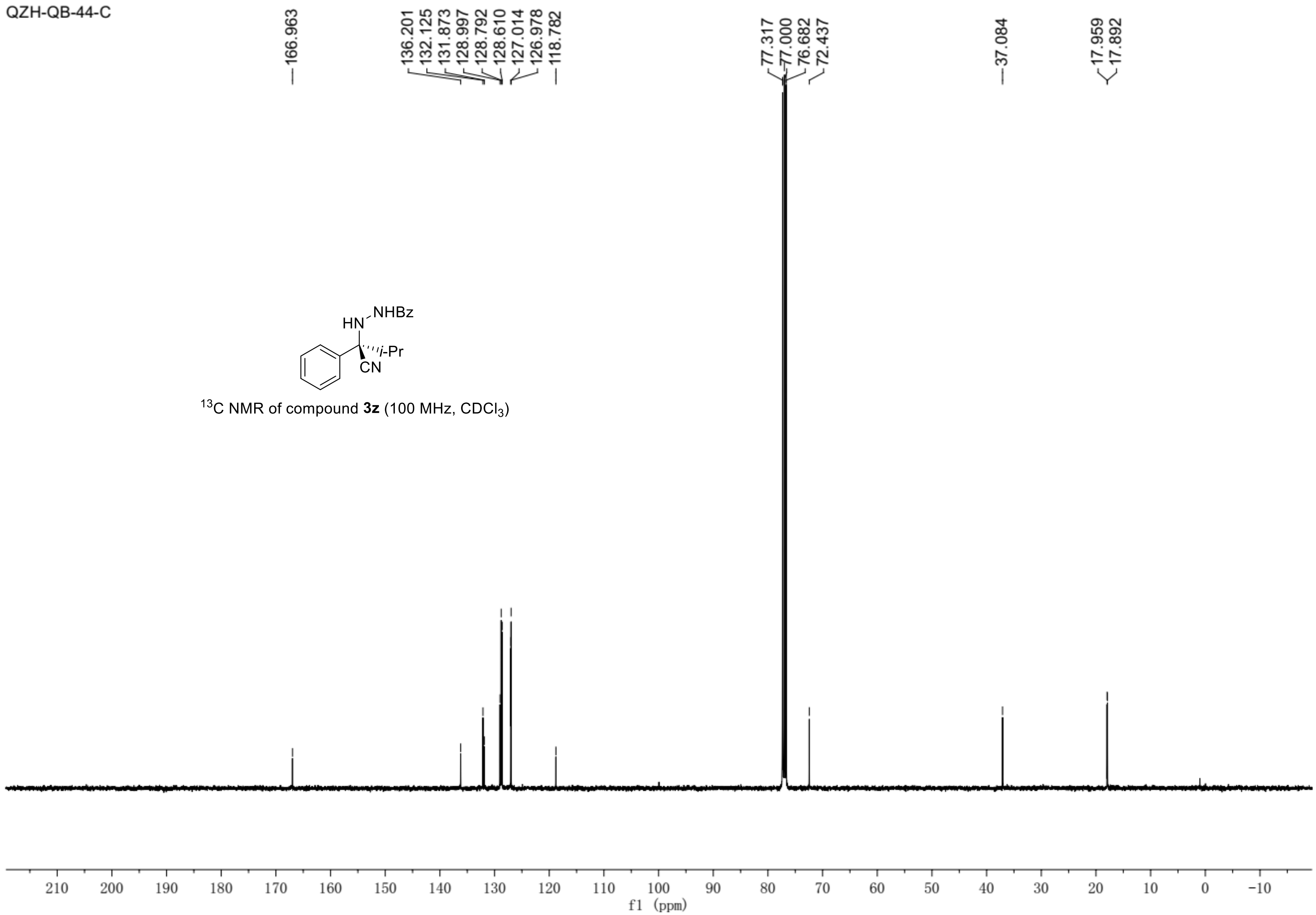

^{13}C NMR of compound **3z** (100 MHz, $CDCl_3$)

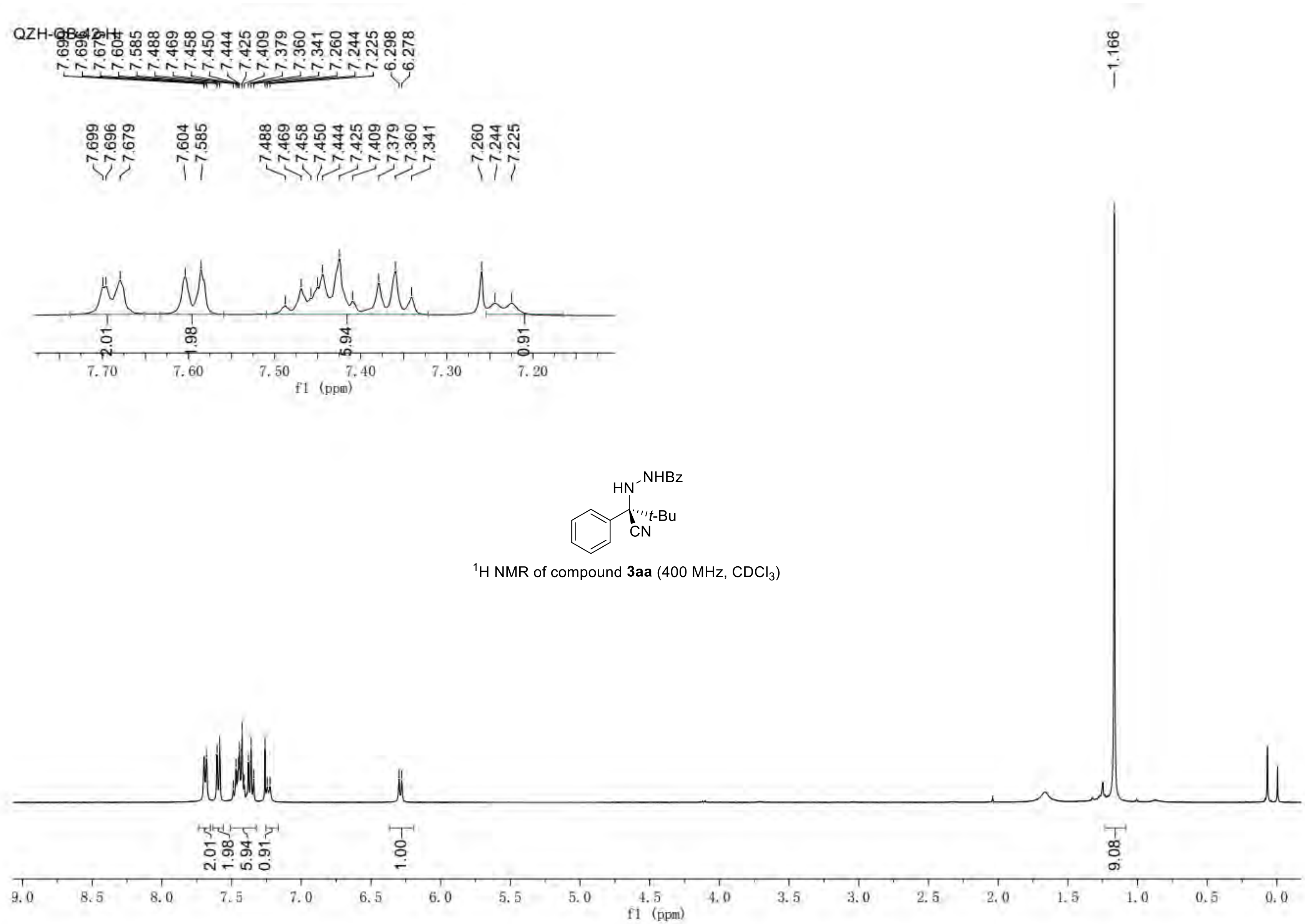

^{1}H NMR of compound **3aa** (400 MHz, $CDCl_3$)

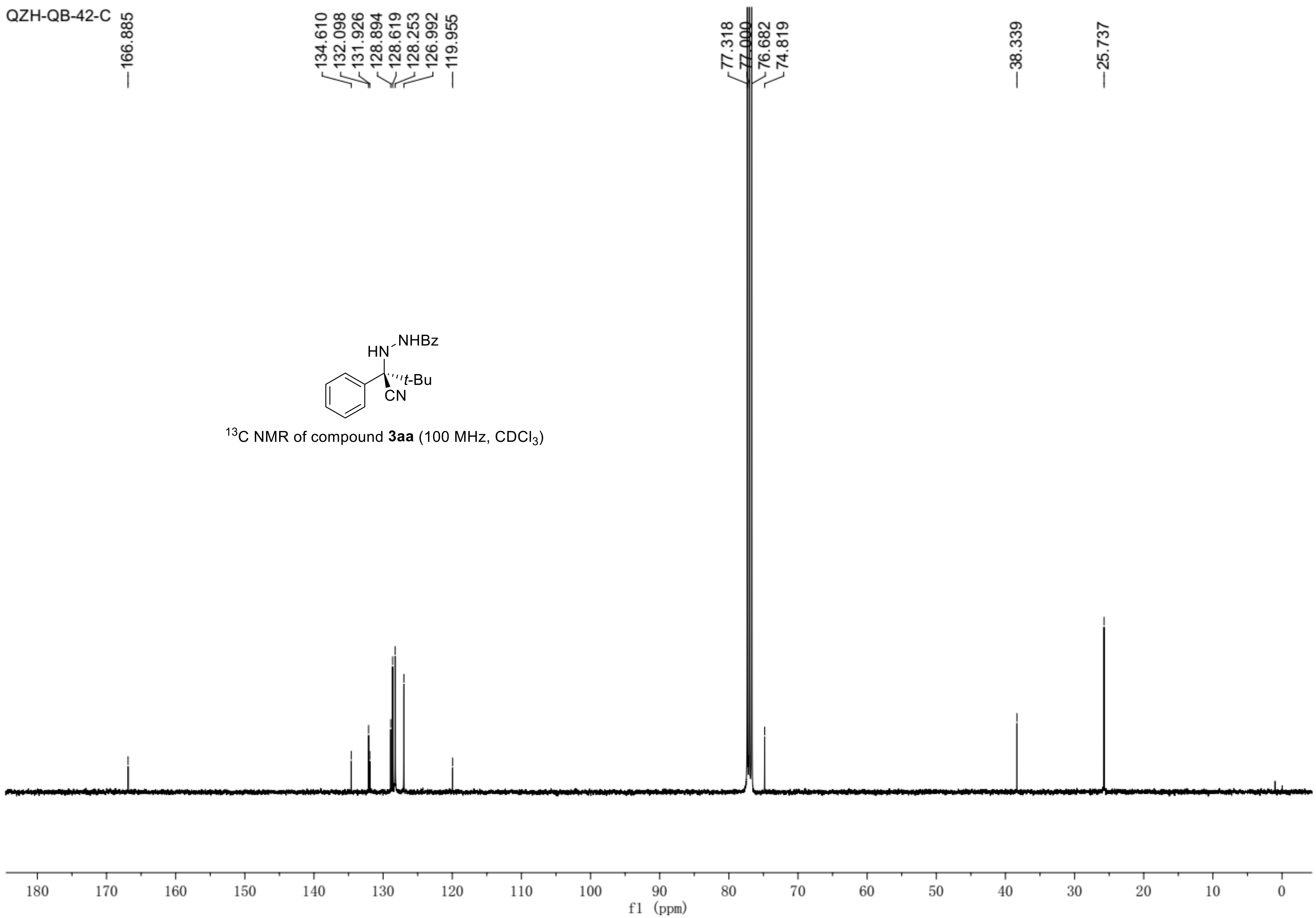

^{13}C NMR of compound **3aa** (100 MHz, $CDCl_3$)

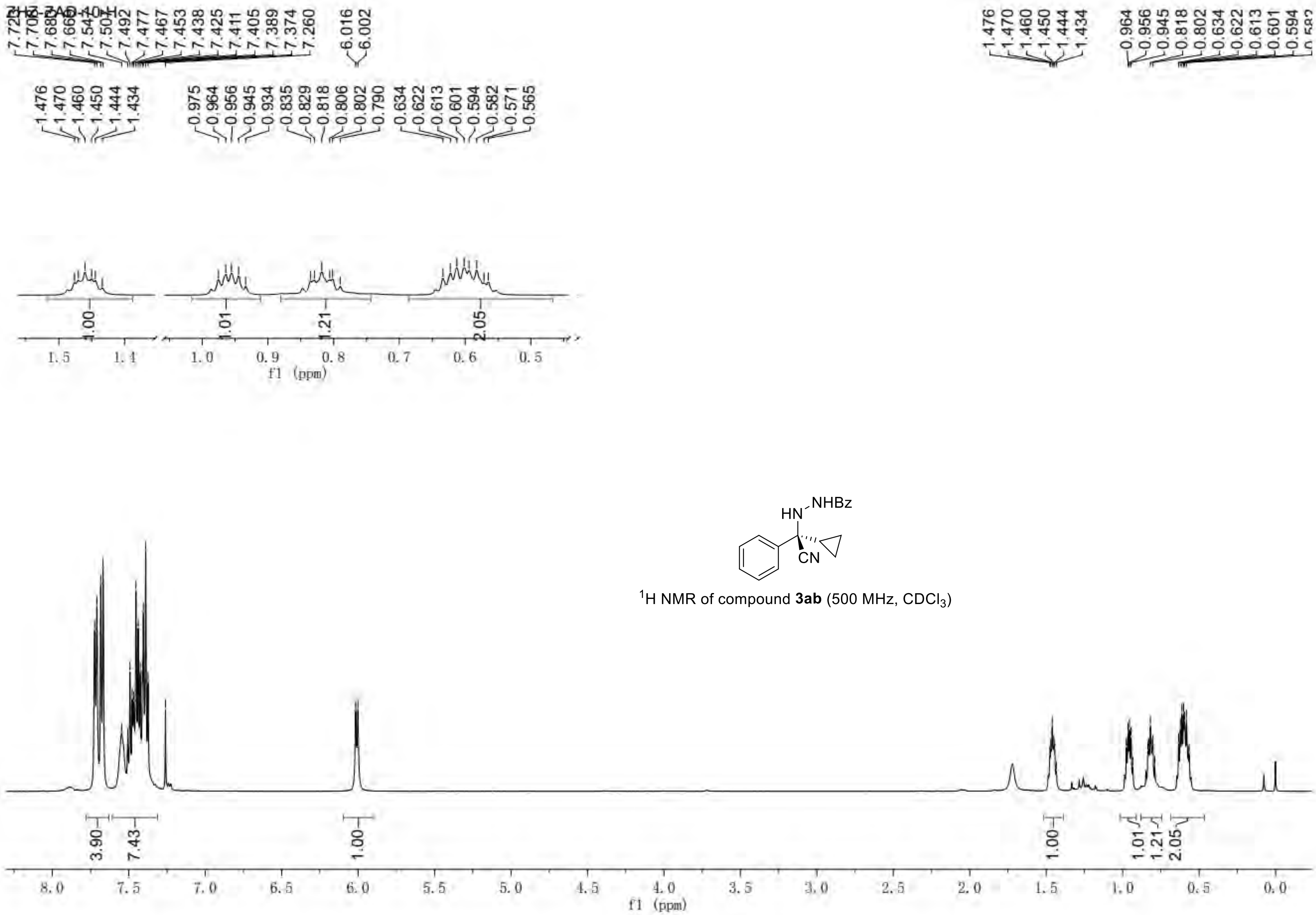

^{1}H NMR of compound **3ab** (500 MHz, $CDCl_3$)

ZHZ-ZAD-10-C

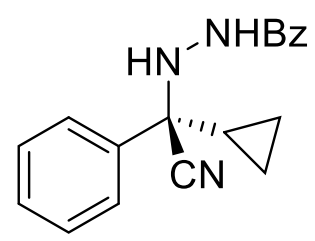

^{13}C NMR of compound **3ab** (125 MHz, $CDCl_3$)

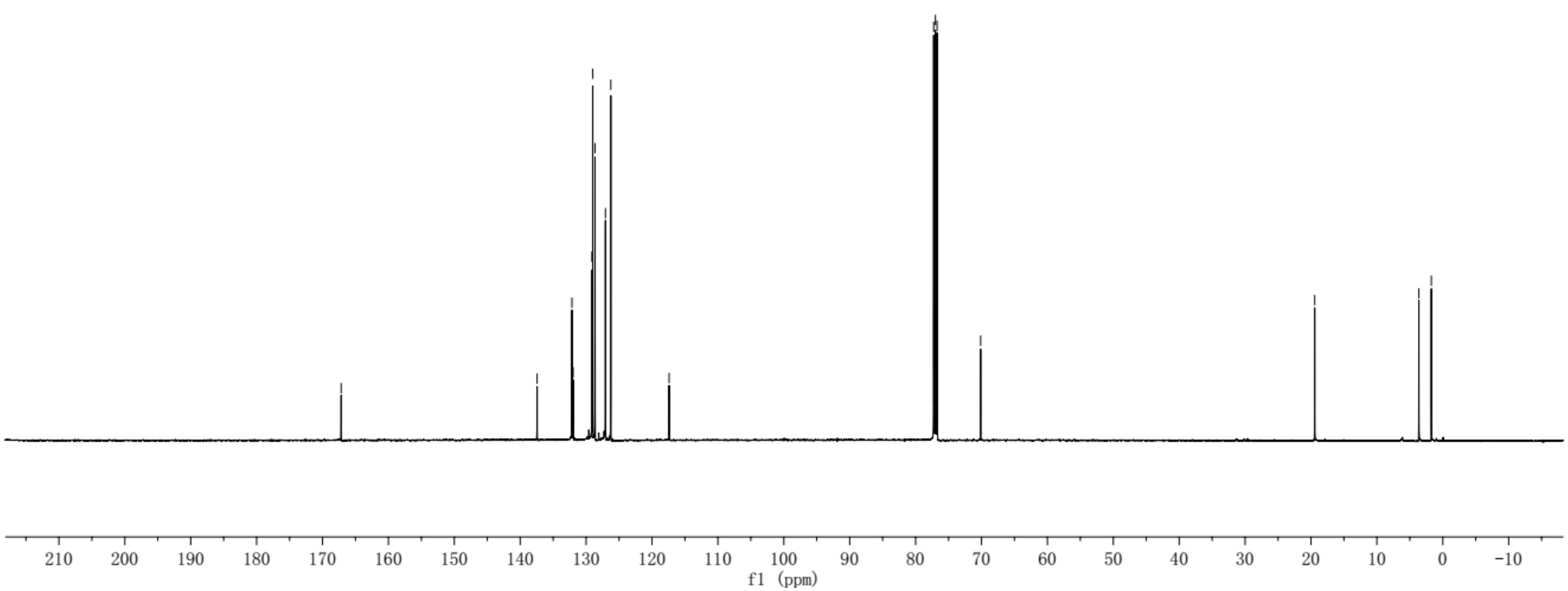

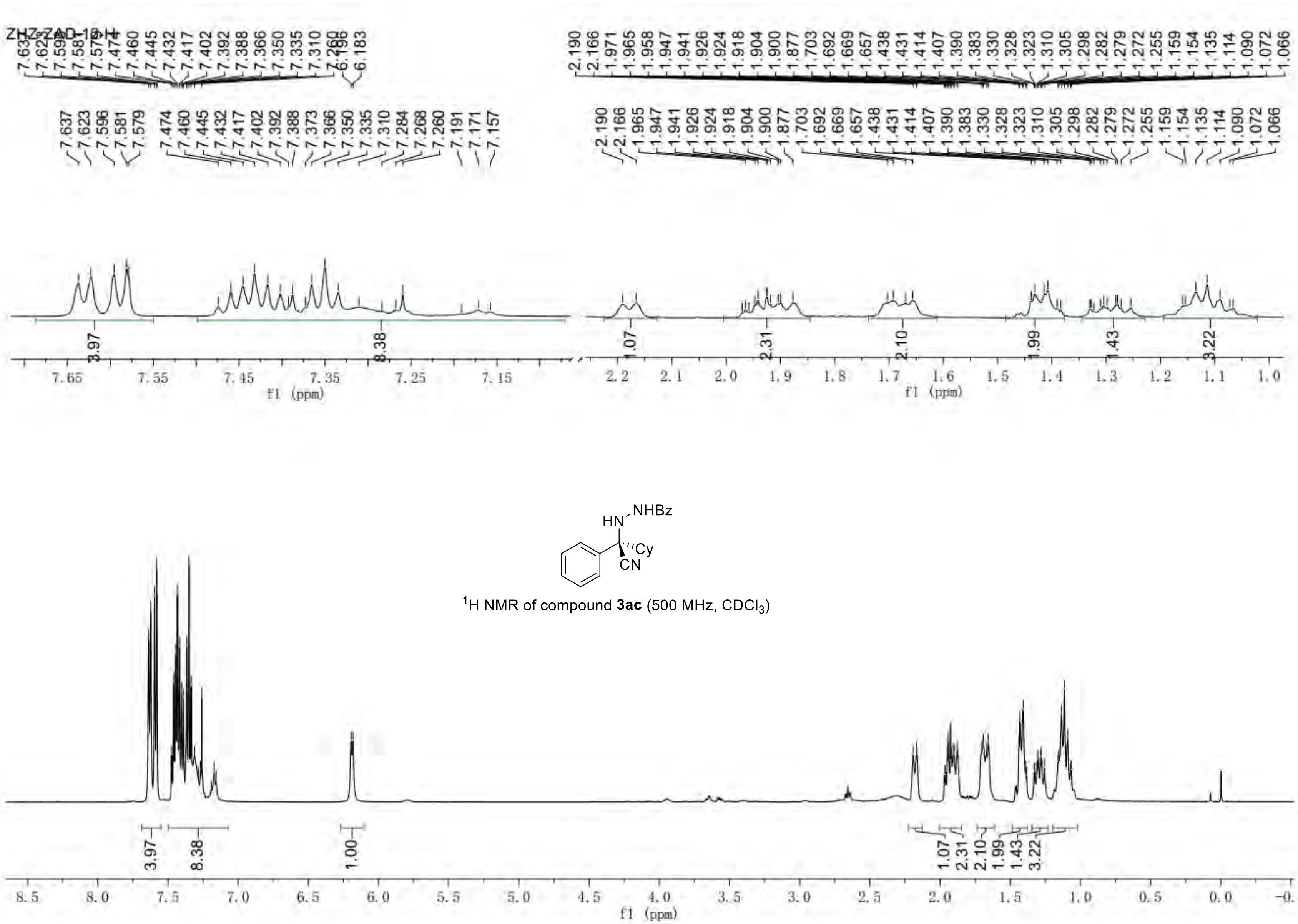

^{1}H NMR of compound **3ac** (500 MHz, $CDCl_3$)

ZHZ-ZAD-12-C

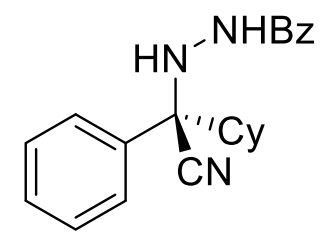

^{13}C NMR of compound **3ac** (125 MHz, $CDCl_3$)

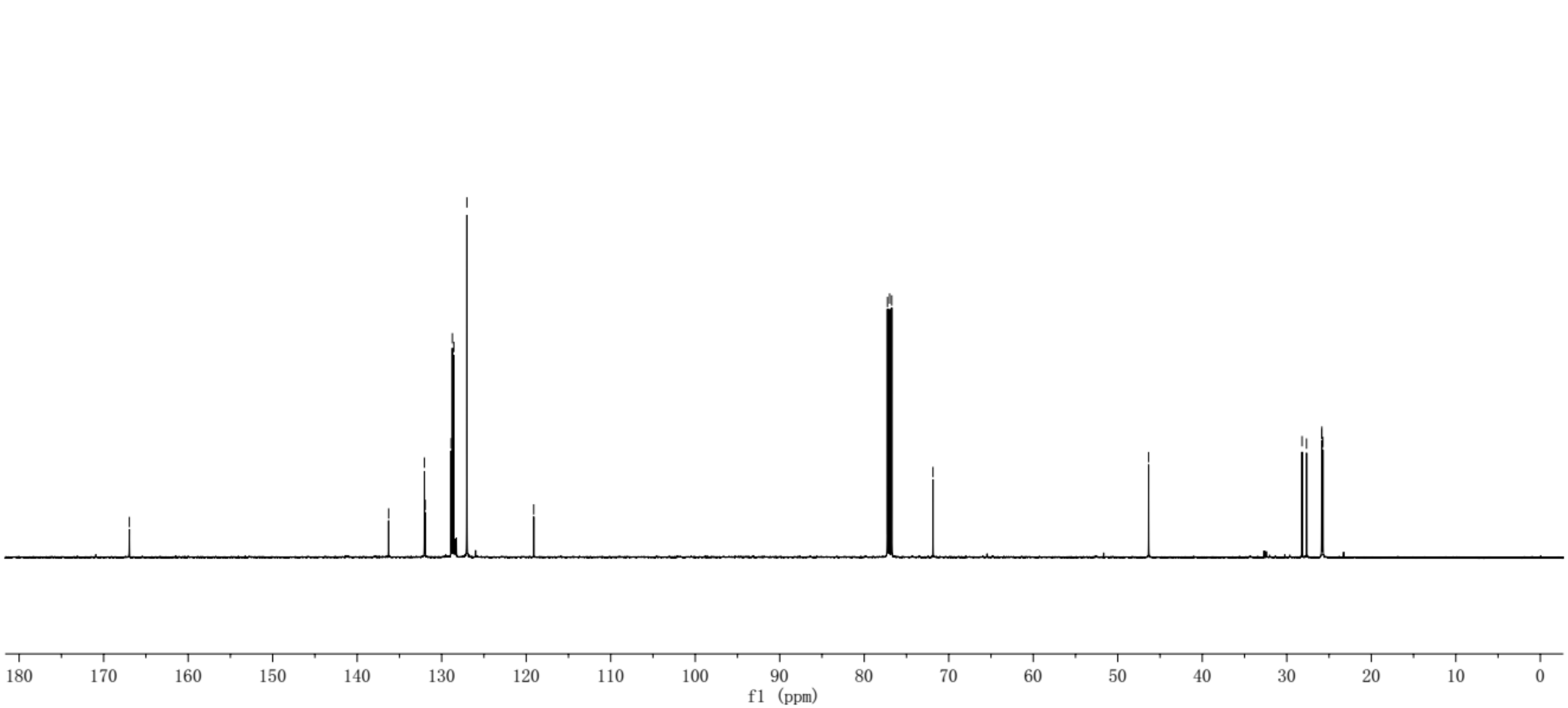

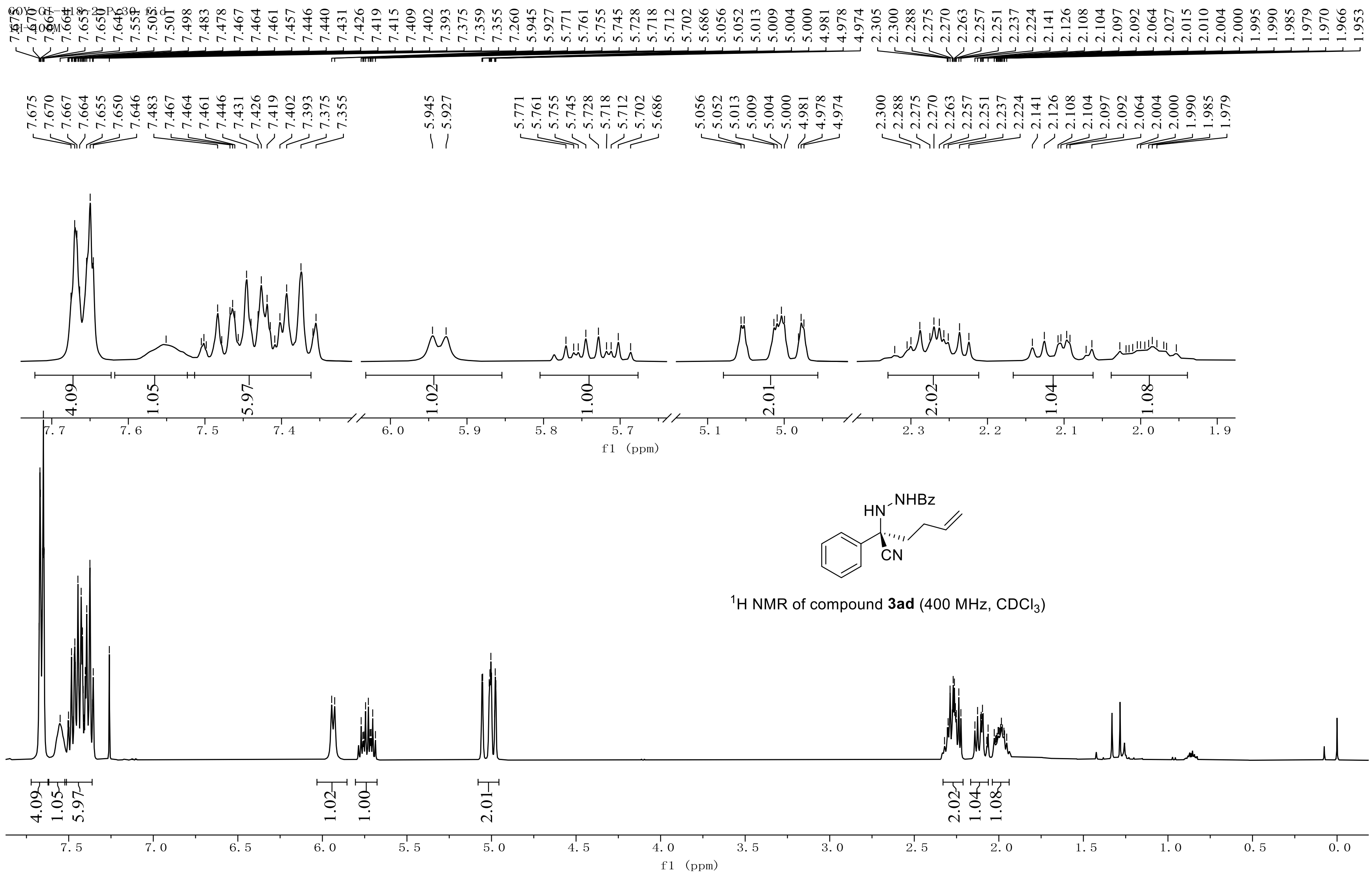

^{1}H NMR of compound **3ad** (400 MHz, $CDCl_3$)

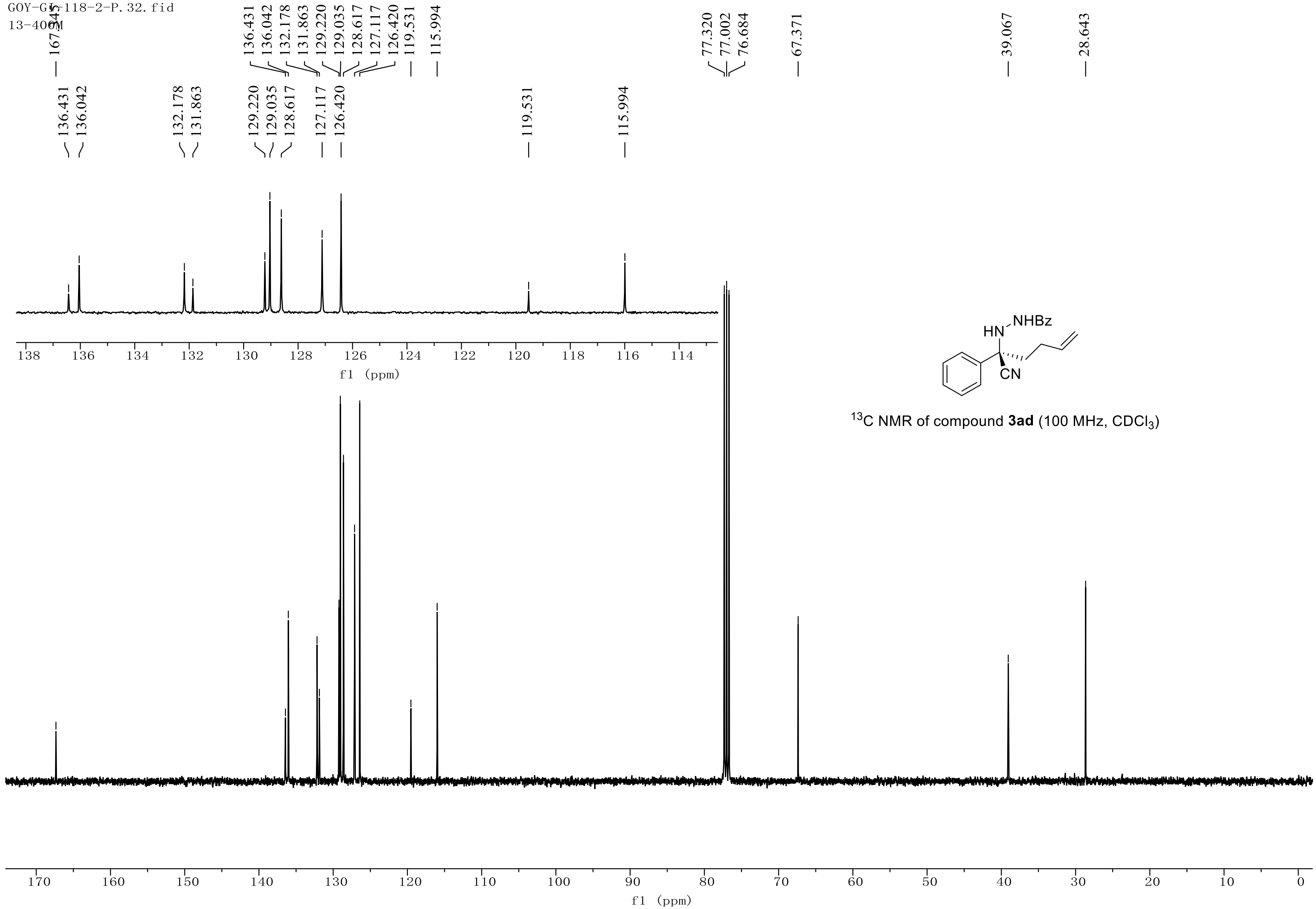

^{13}C NMR of compound **3ad** (100 MHz, $CDCl_3$)

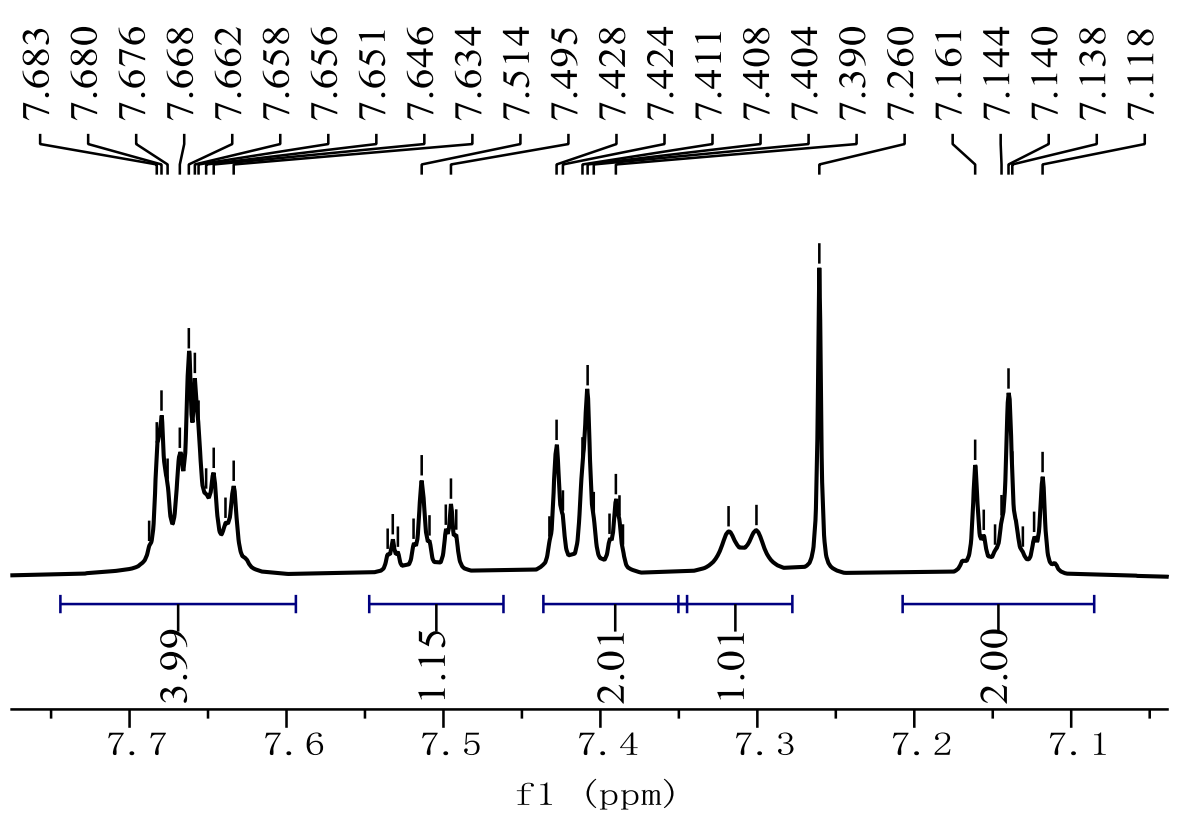

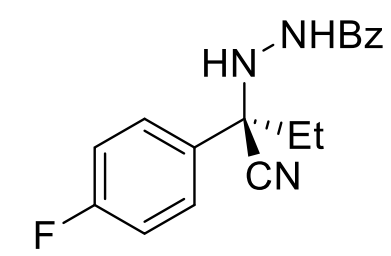

^{1}H NMR of compound **3ae** (400 MHz, $CDCl_3$)

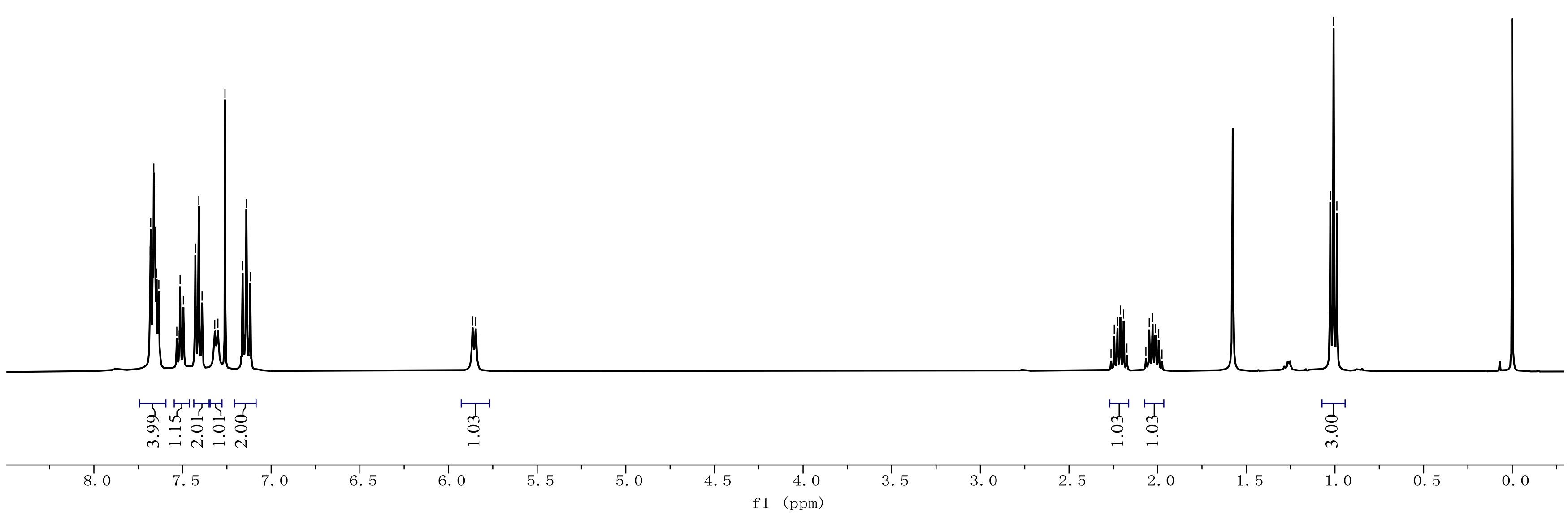

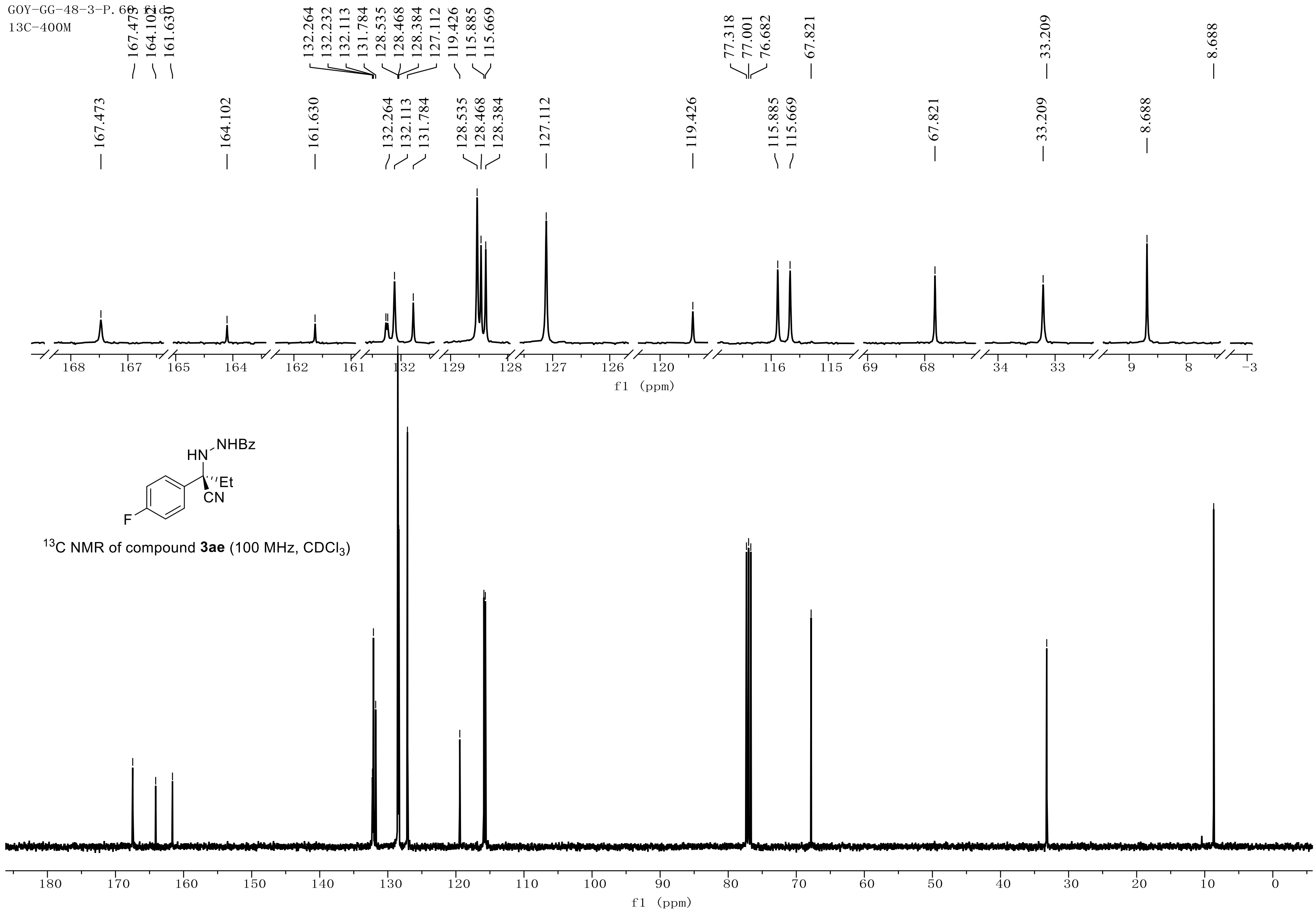

^{13}C NMR of compound **3ae** (100 MHz, $CDCl_3$)

GOY-GG-48-3-P.41.fid
19F-400M

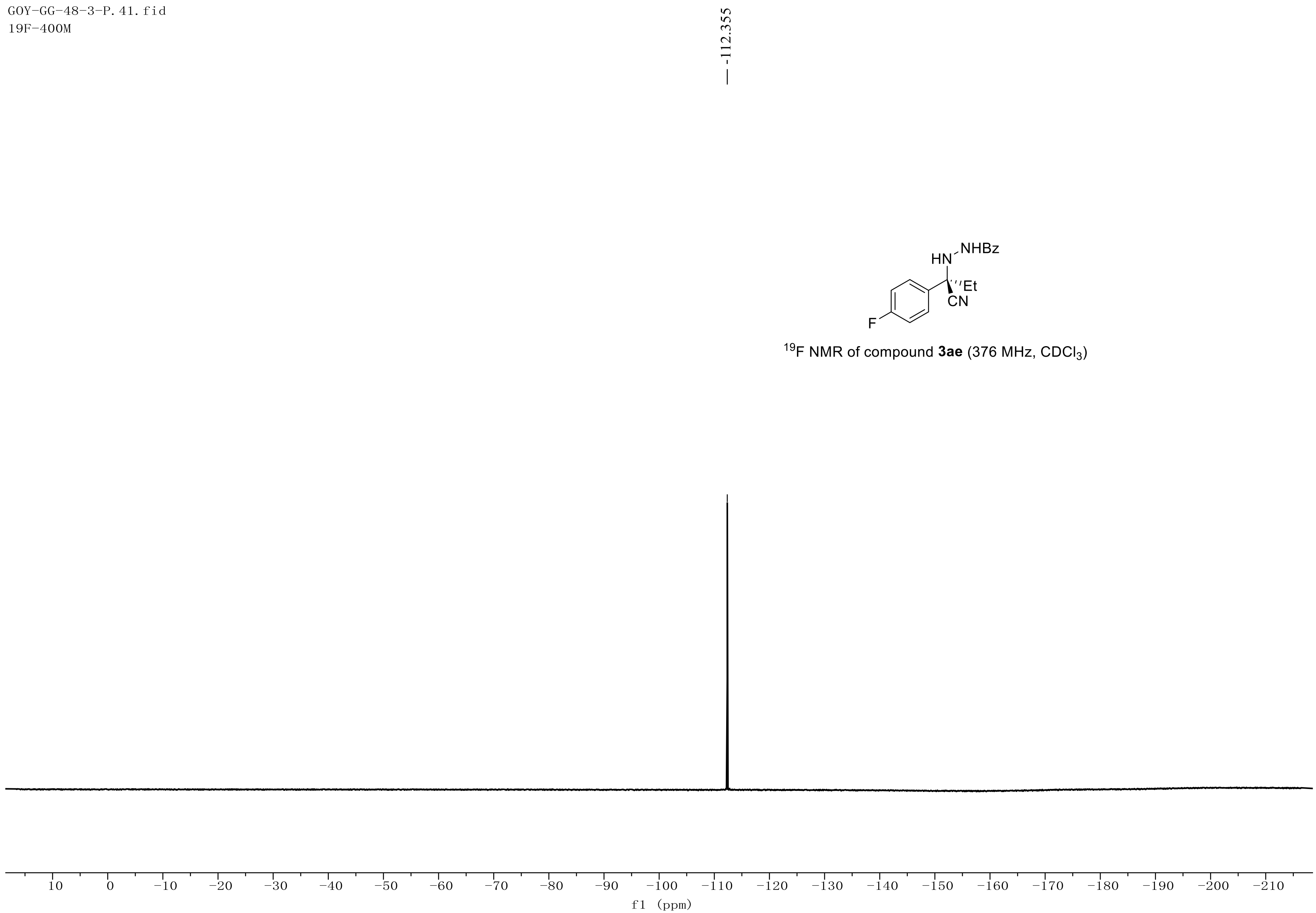

^{19}F NMR of compound **3ae** (376 MHz, $CDCl_3$)

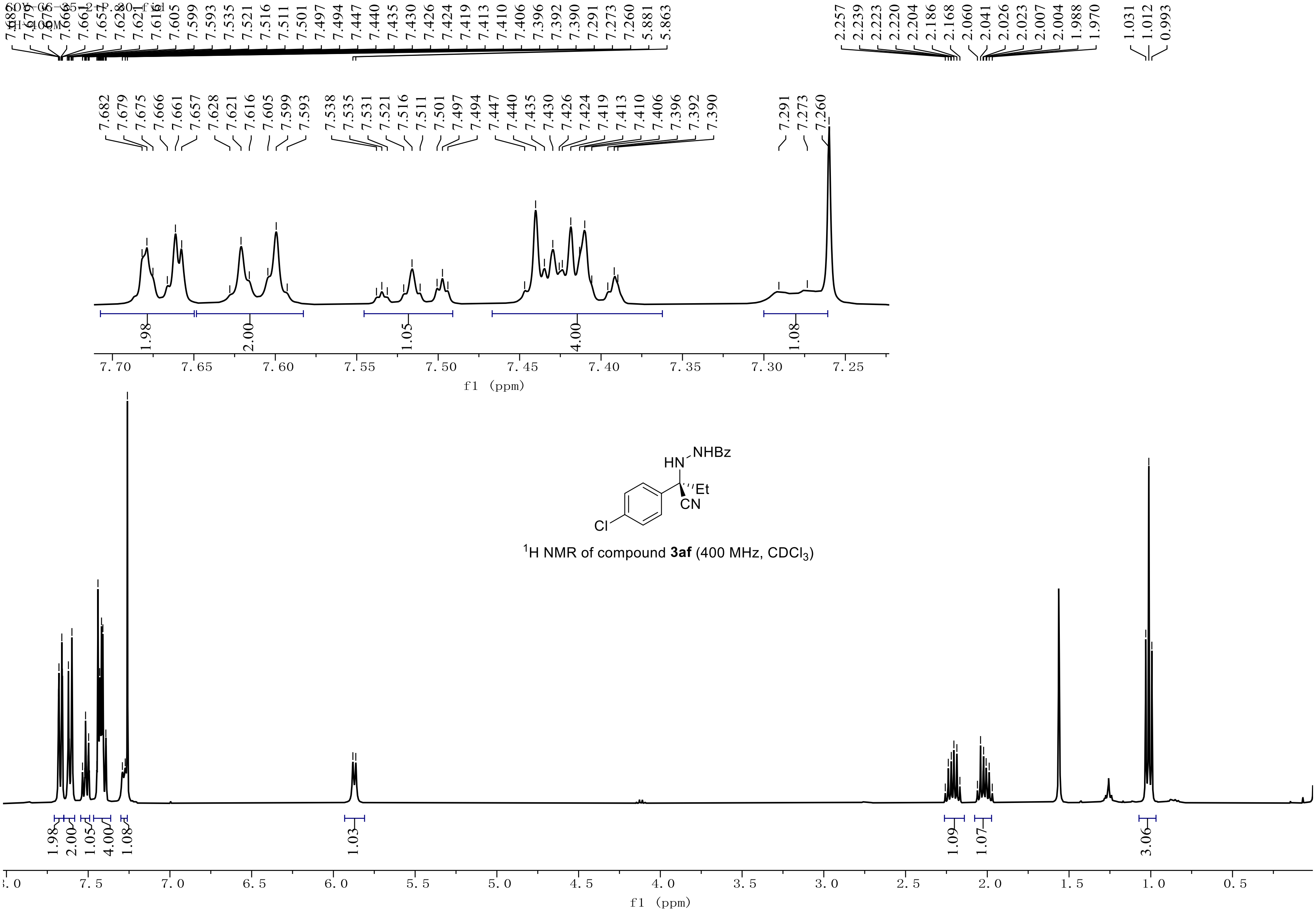
HN
NHBz
Et
CN
Cl
^{1}H NMR of compound 3af (400 MHz, CDCl$_3$)
f1 (ppm)

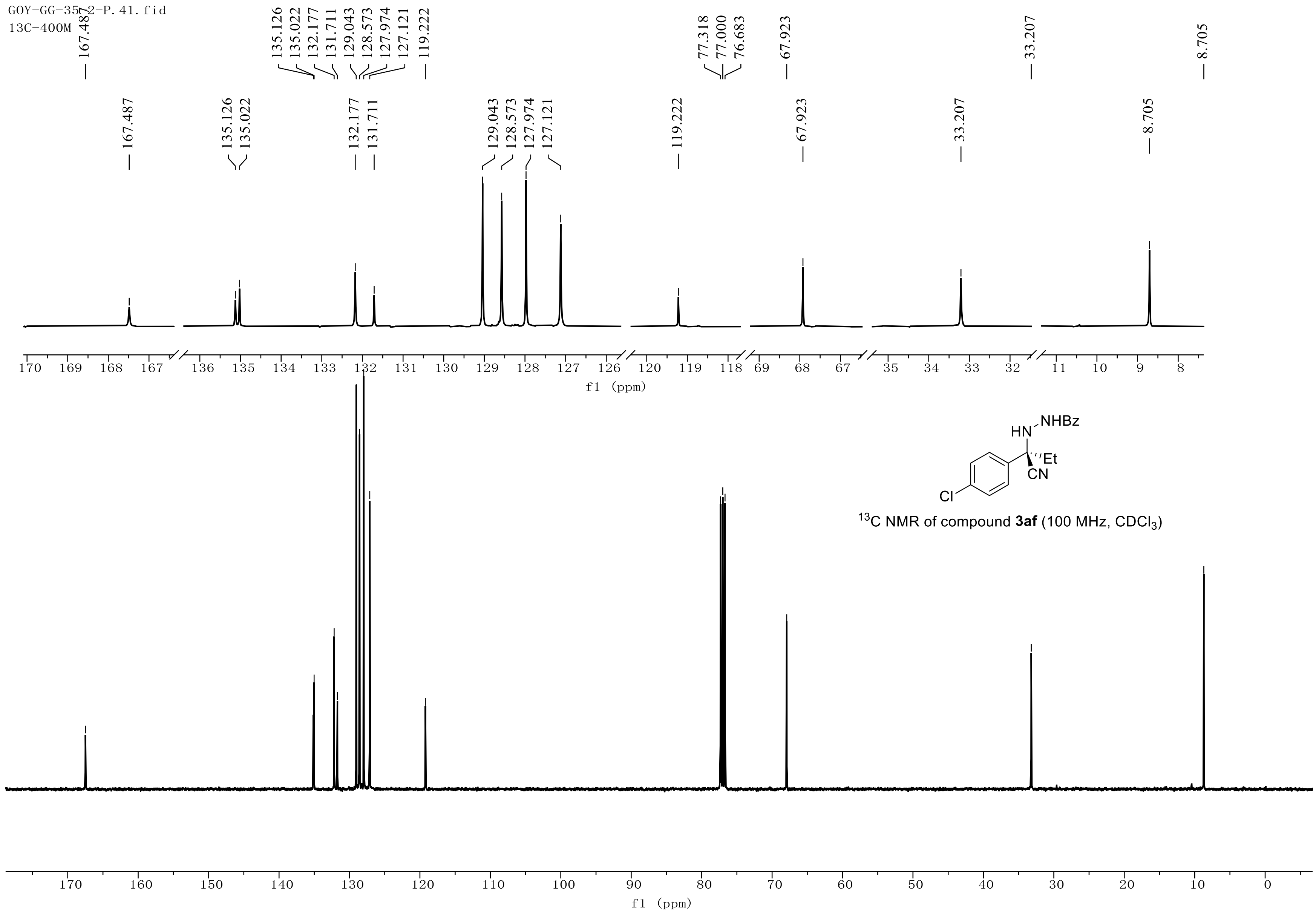

^{13}C NMR of compound **3af** (100 MHz, $CDCl_3$)

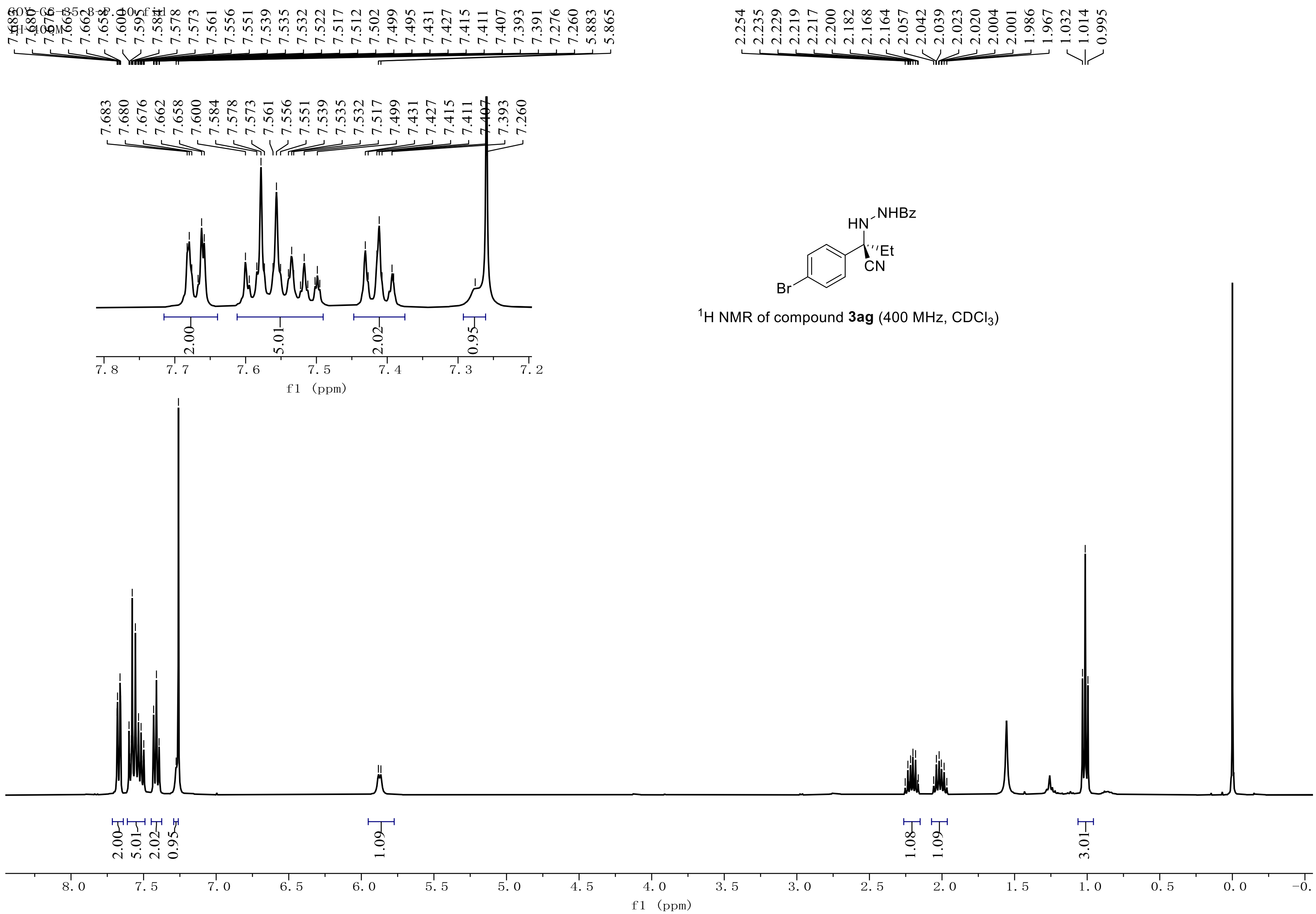

^{1}H NMR of compound **3ag** (400 MHz, $CDCl_3$)

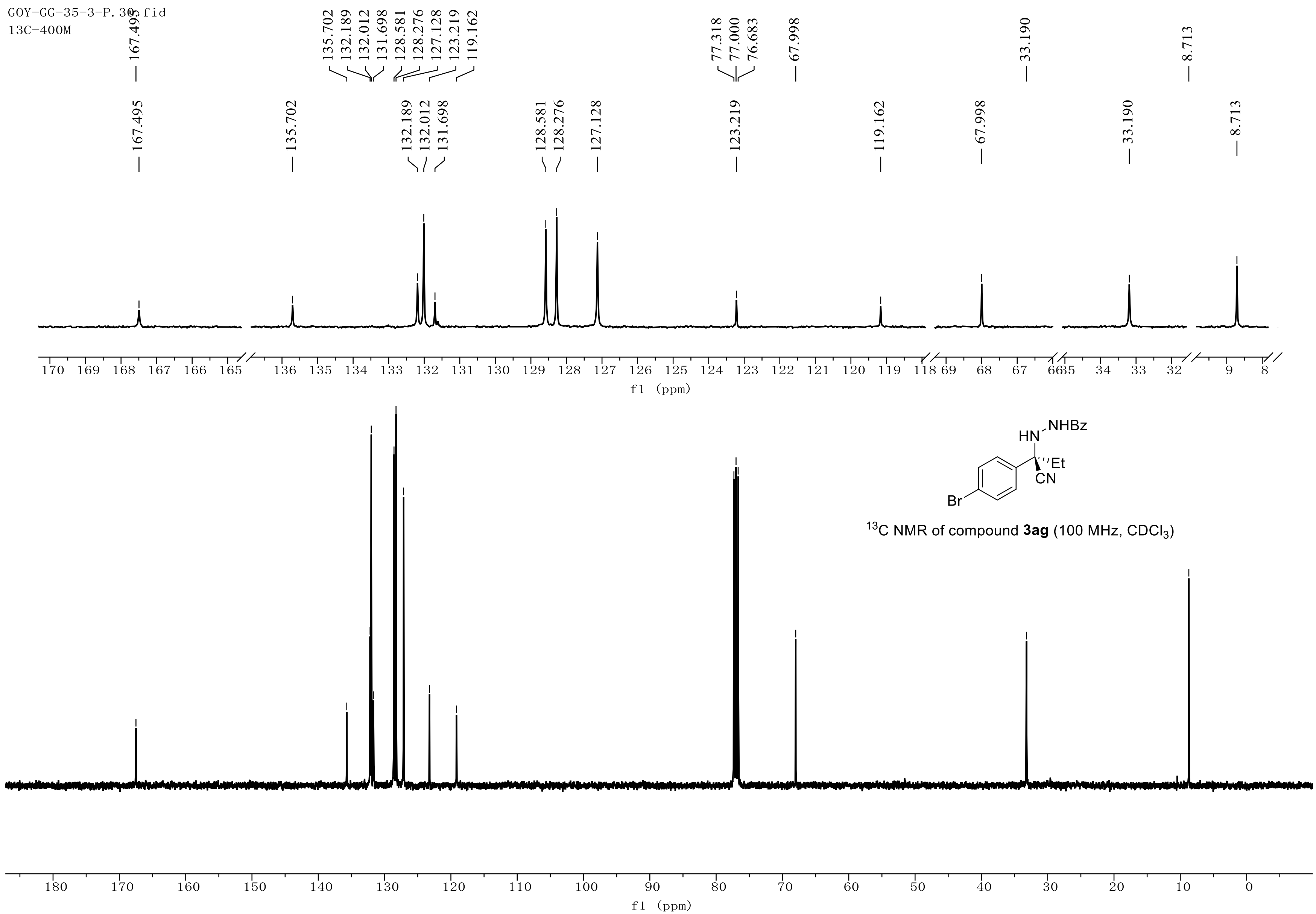

^{13}C NMR of compound **3ag** (100 MHz, $CDCl_3$)

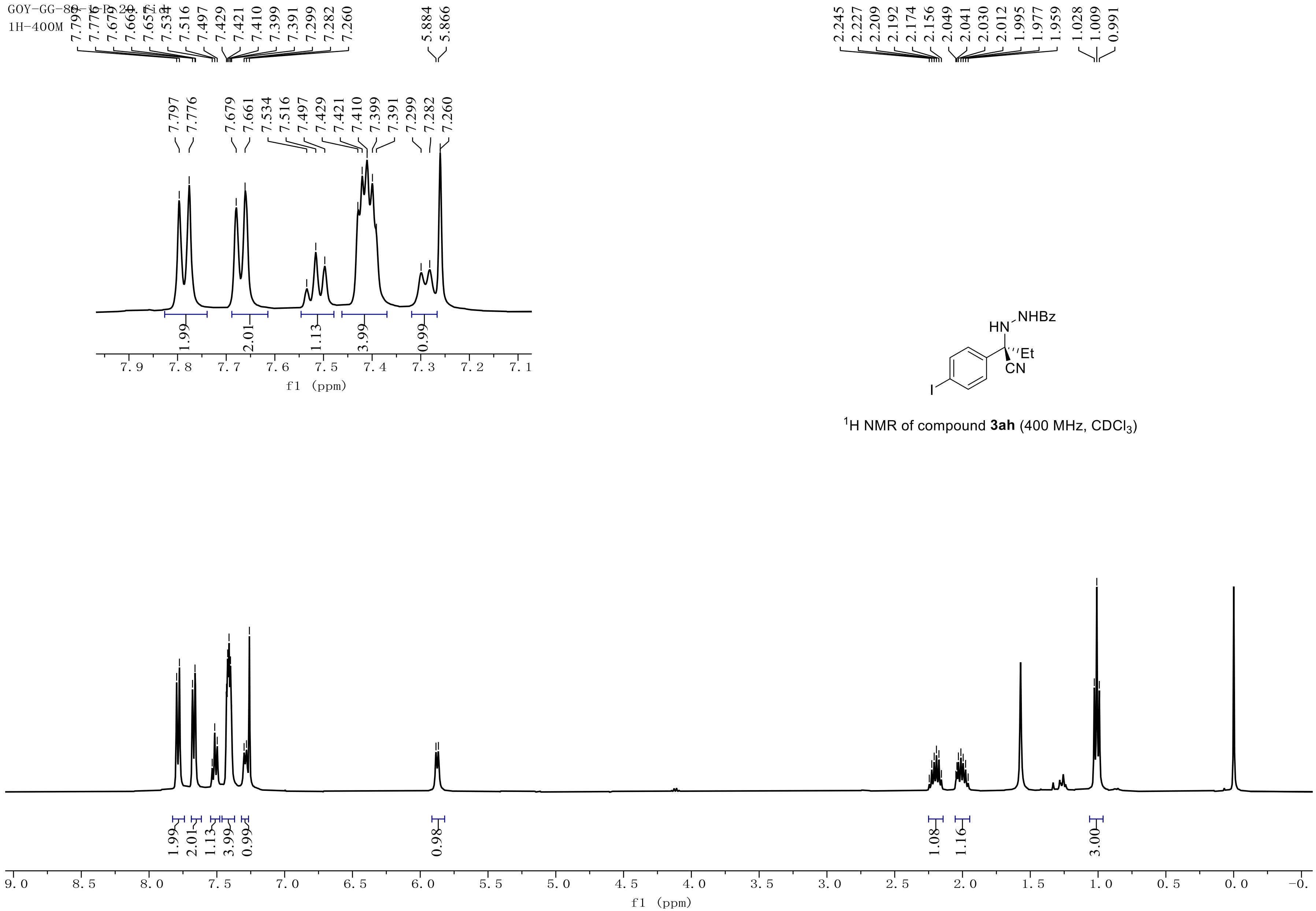

^1H NMR of compound **3ah** (400 MHz, $CDCl_3$)

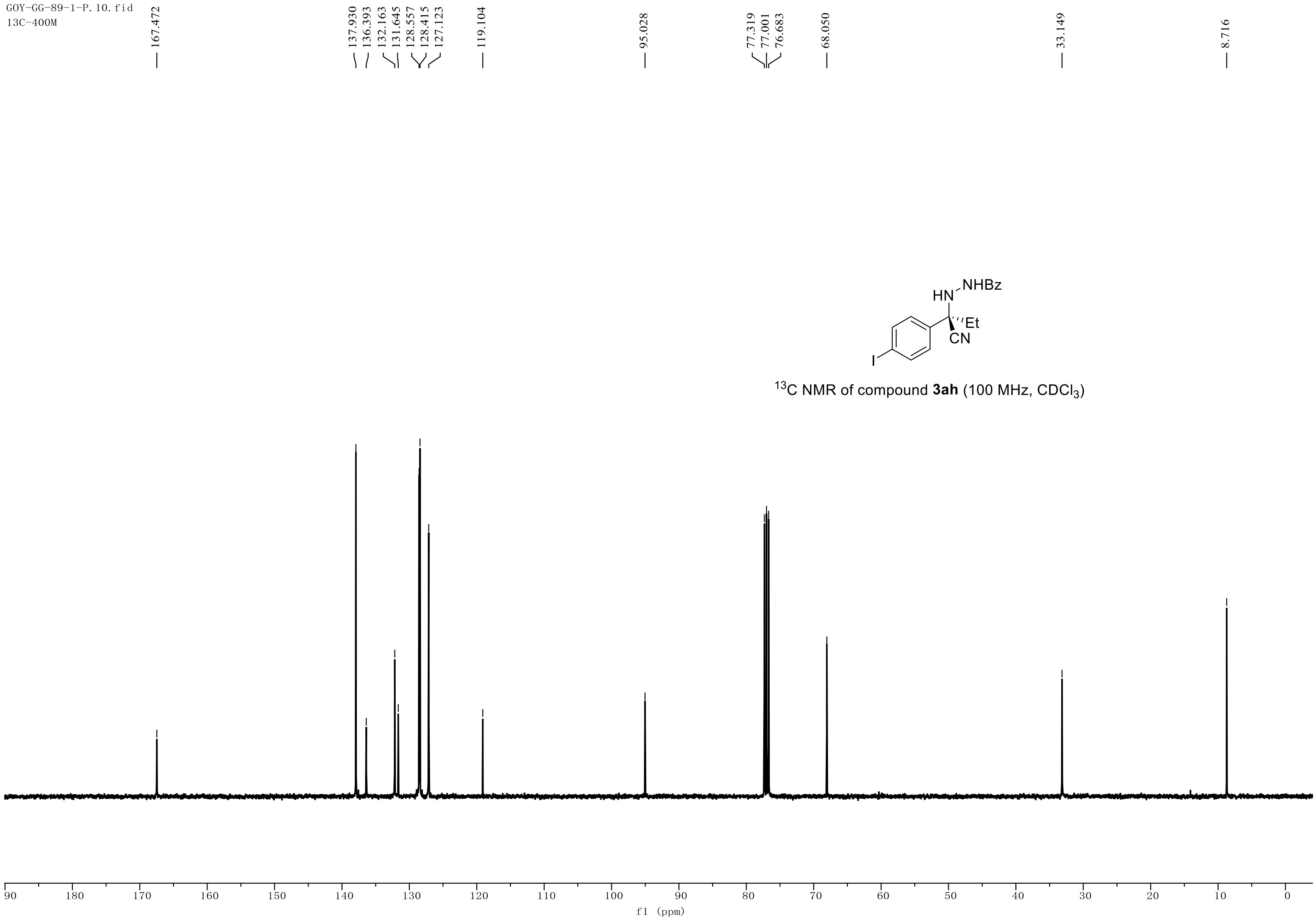

^{13}C NMR of compound **3ah** (100 MHz, $CDCl_3$)

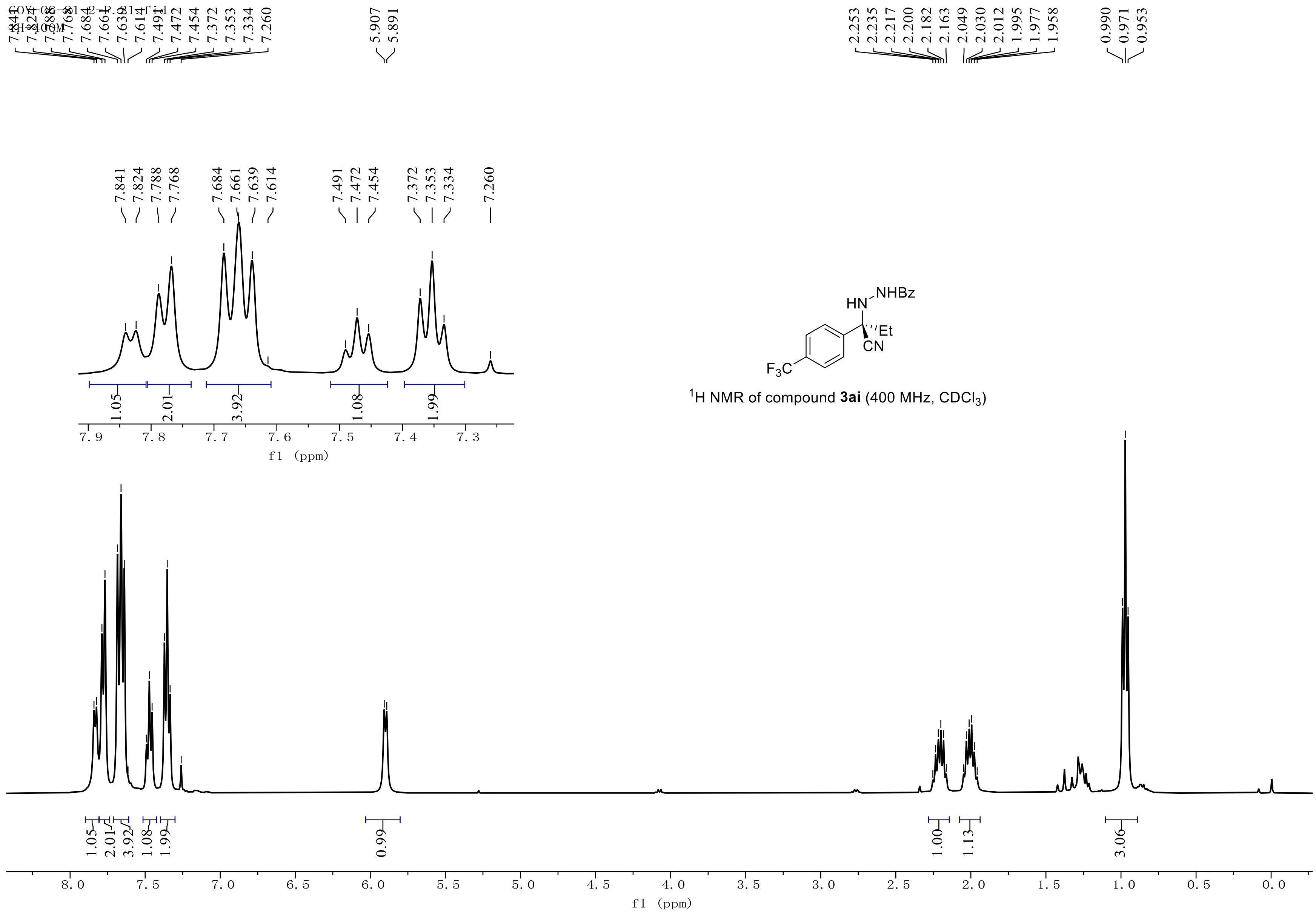

^{1}H NMR of compound **3ai** (400 MHz, $CDCl_3$)

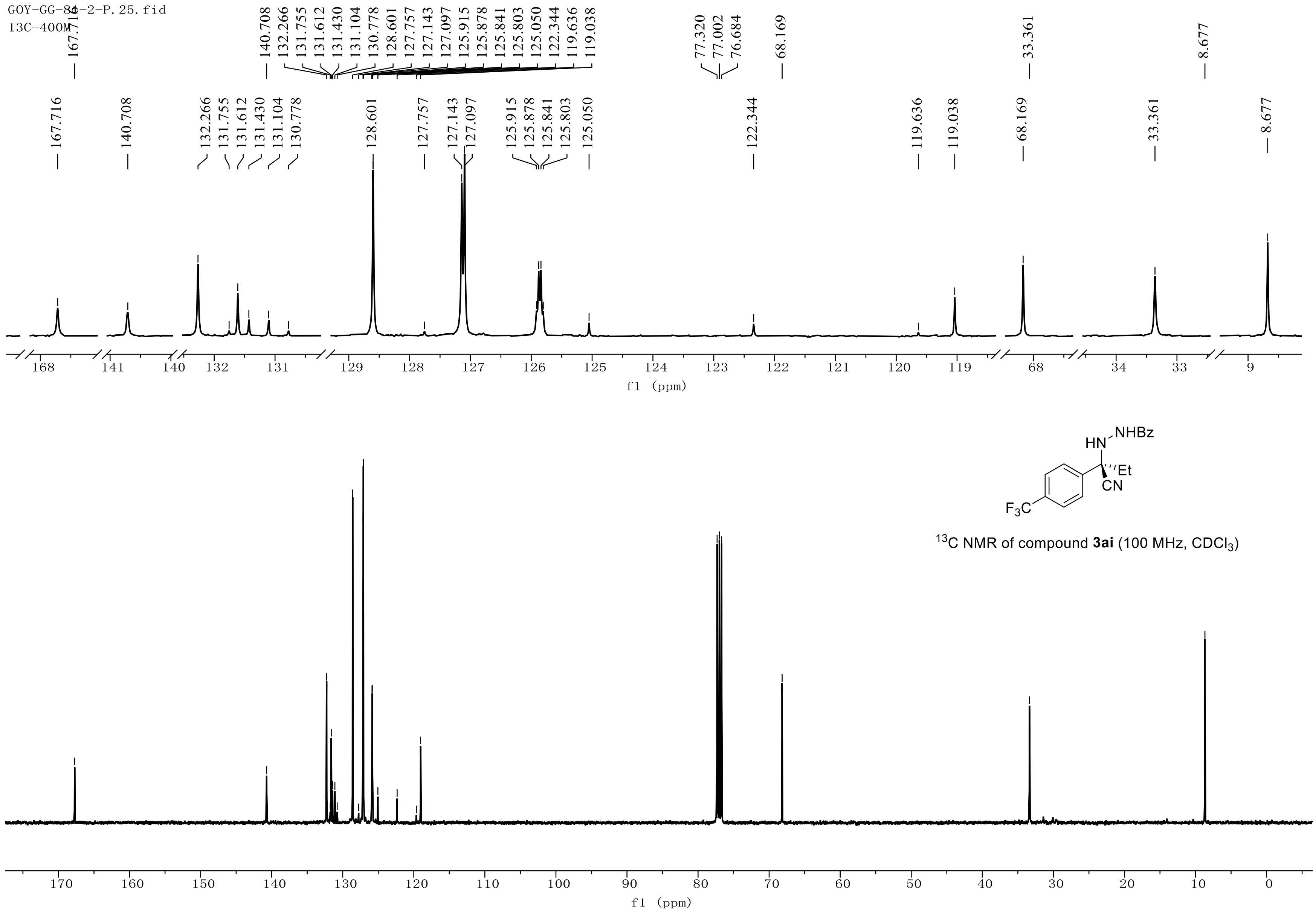

^{13}C NMR of compound **3ai** (100 MHz, $CDCl_3$)

GOY-GG-81-2-P.24.fid
19F-400M

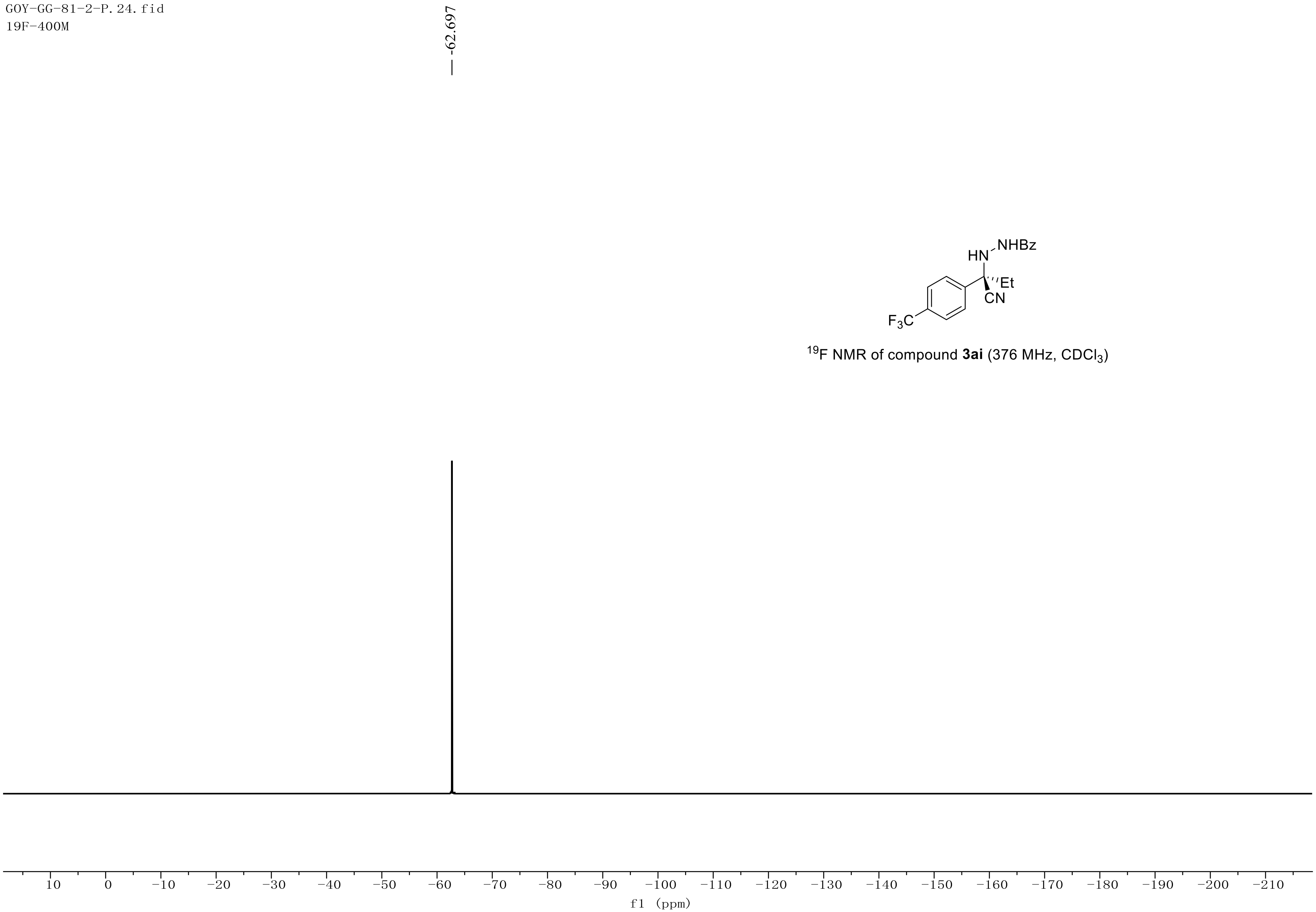

^{19}F NMR of compound **3ai** (376 MHz, $CDCl_3$)

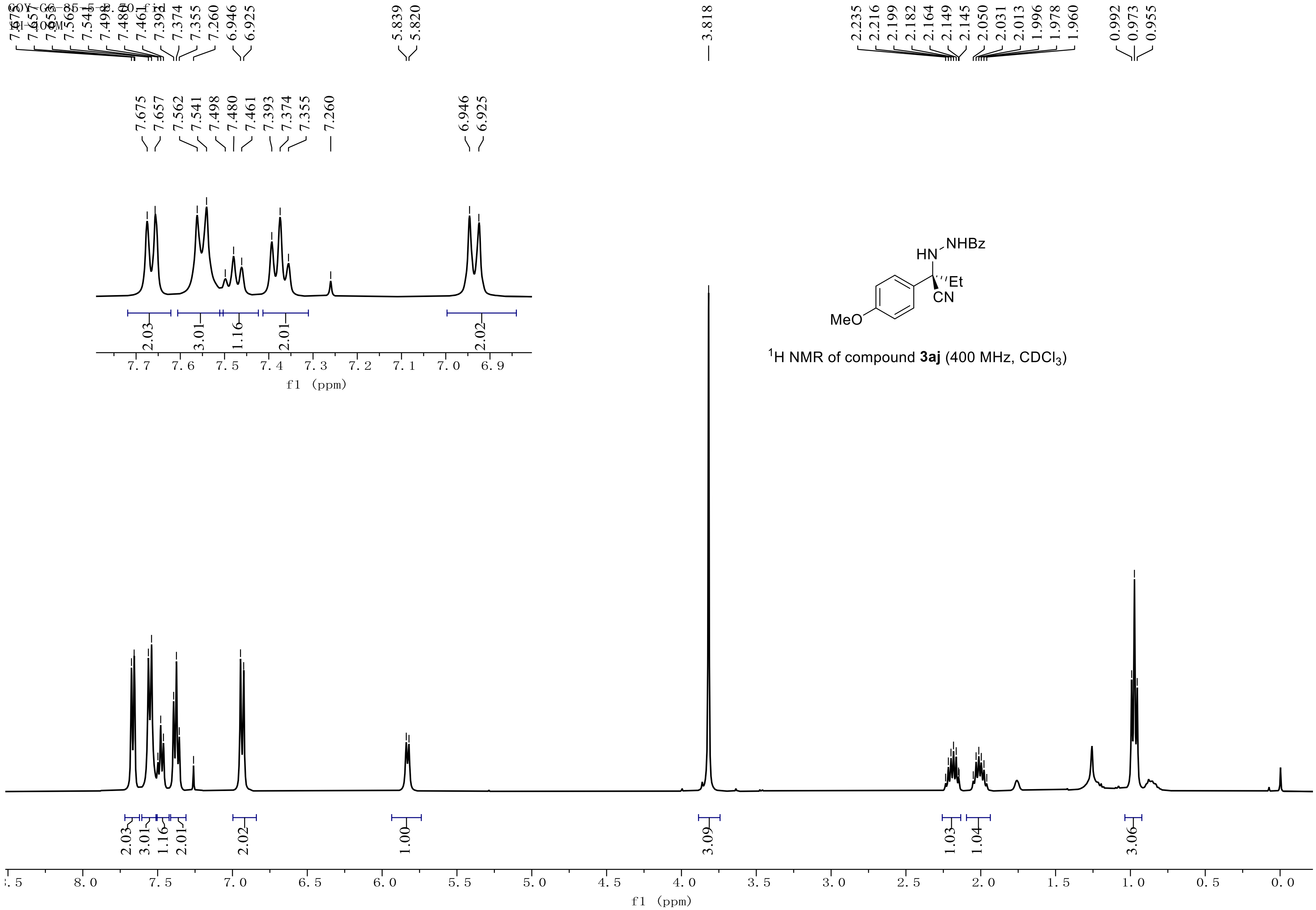

^{1}H NMR of compound **3aj** (400 MHz, $CDCl_3$)

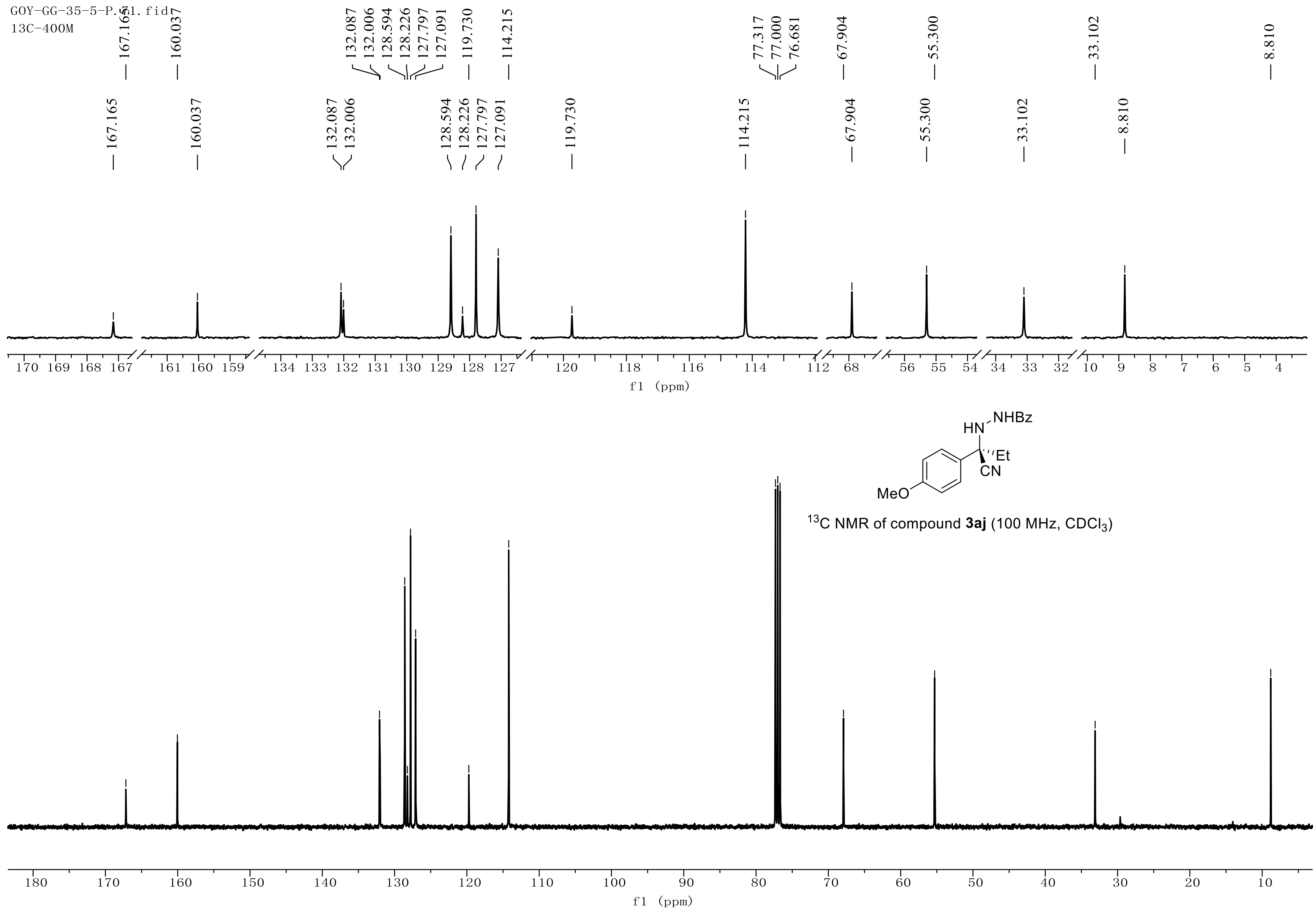

^{13}C NMR of compound **3aj** (100 MHz, $CDCl_3$)

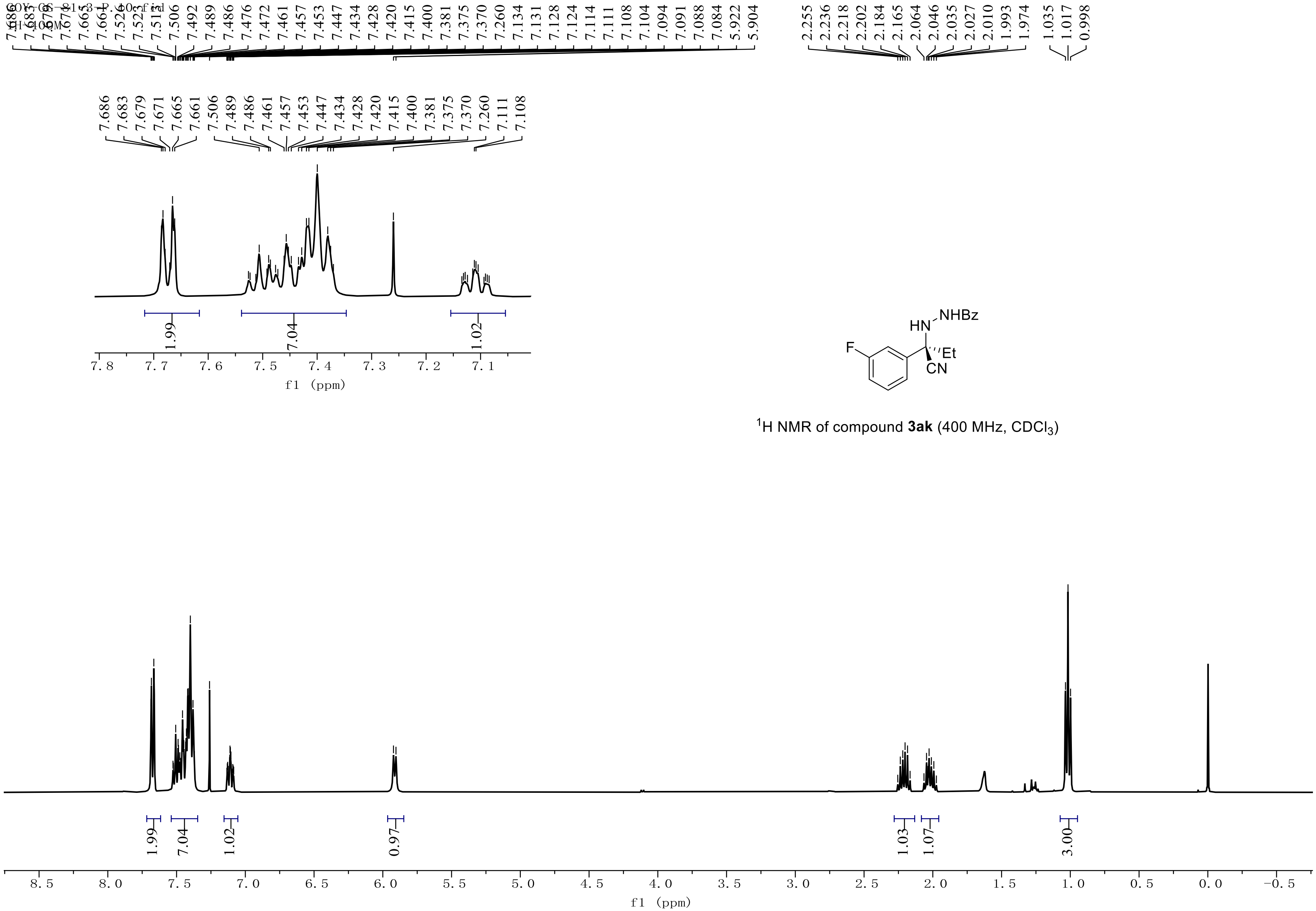

^{1}H NMR of compound **3ak** (400 MHz, $CDCl_3$)

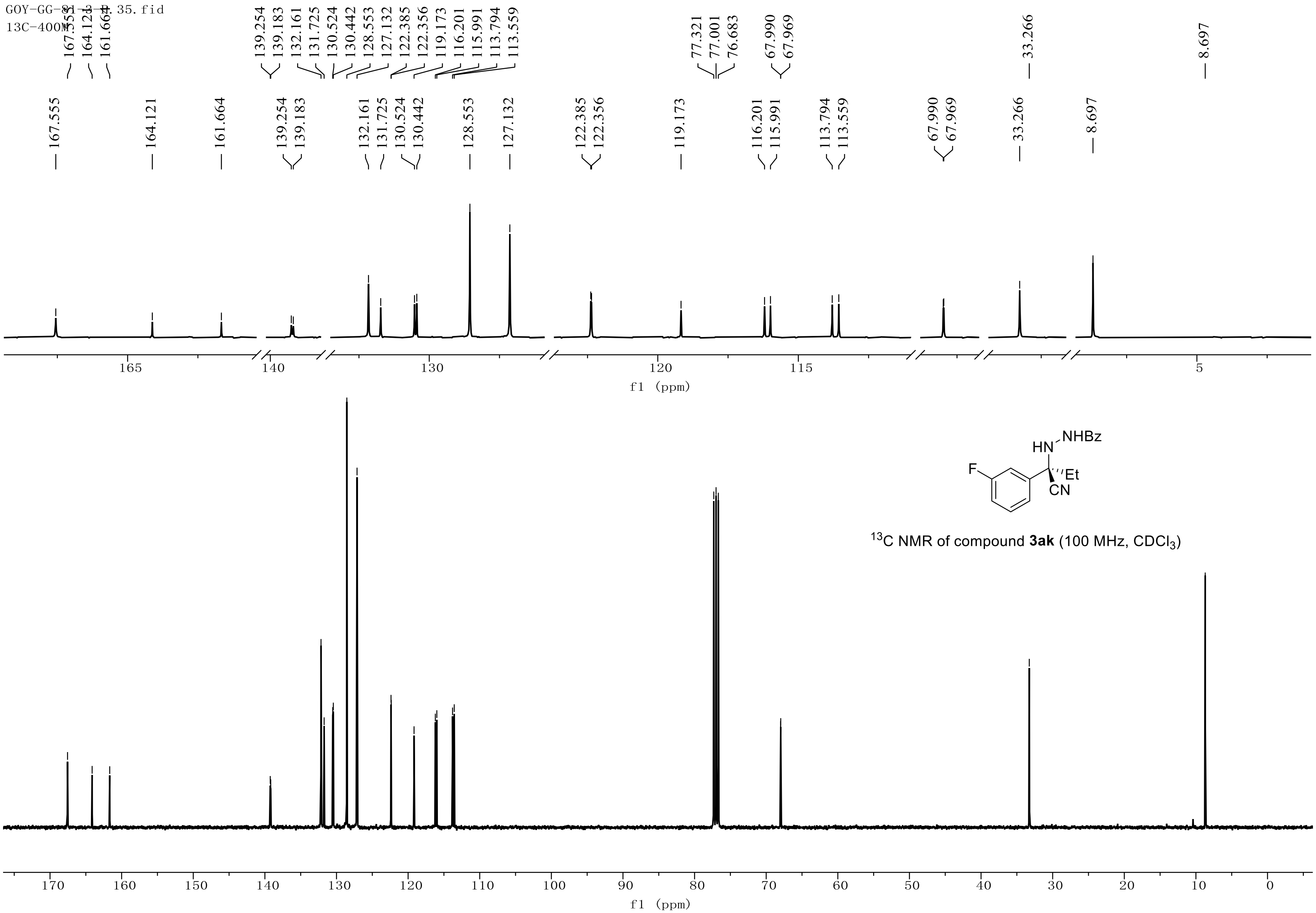

^{13}C NMR of compound **3ak** (100 MHz, $CDCl_3$)

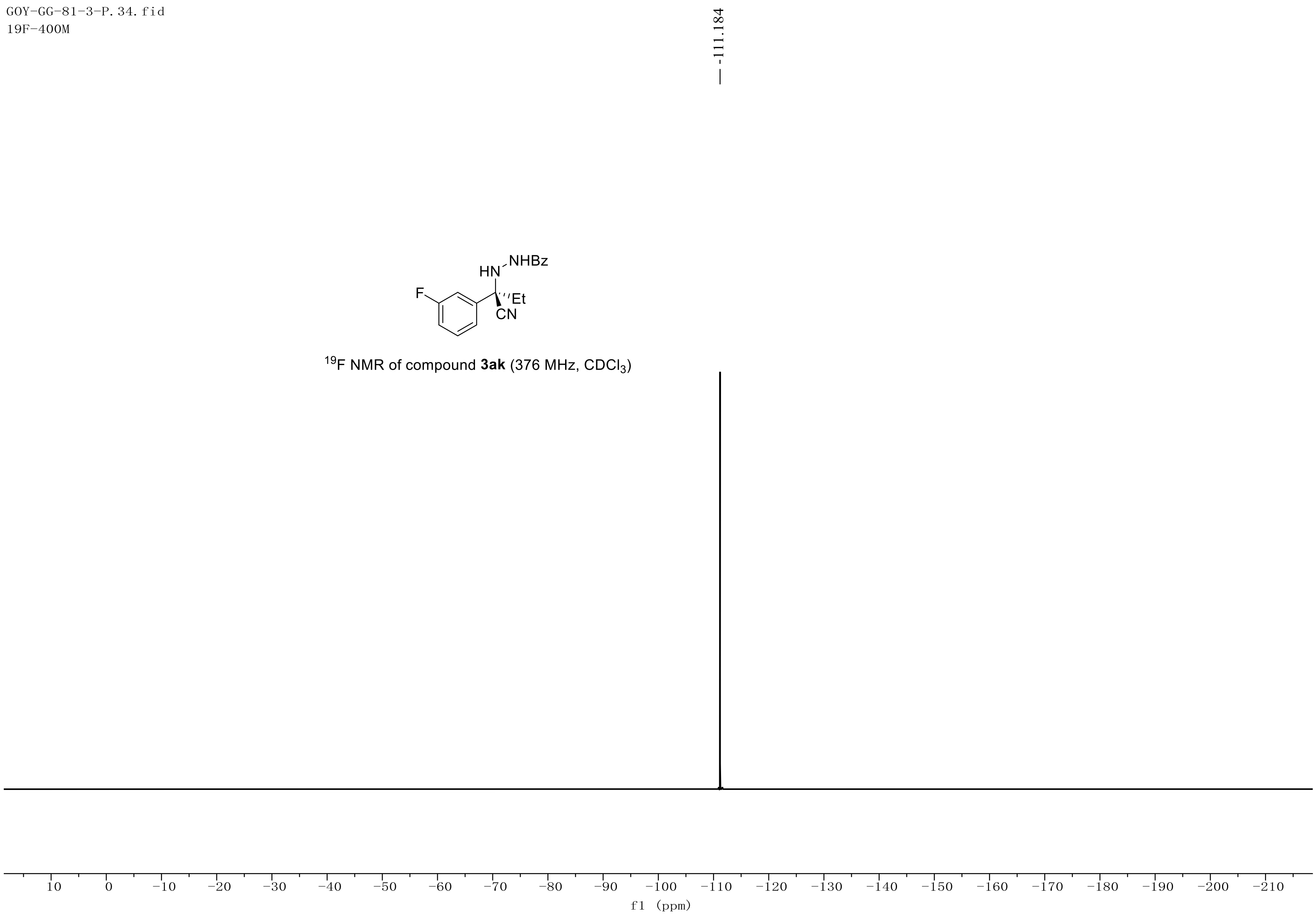

^{19}F NMR of compound **3ak** (376 MHz, $CDCl_3$)

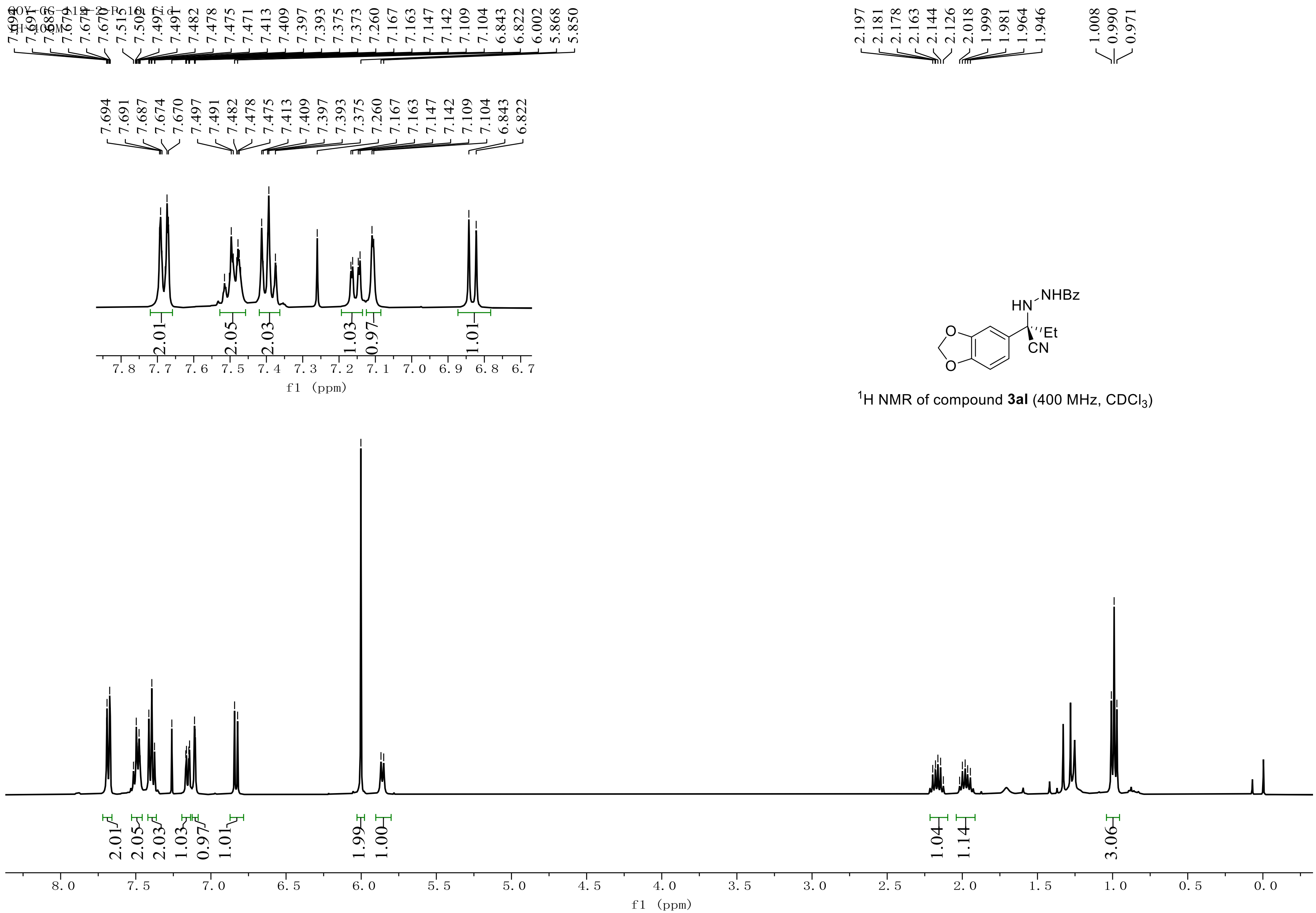

^{1}H NMR of compound **3al** (400 MHz, $CDCl_3$)

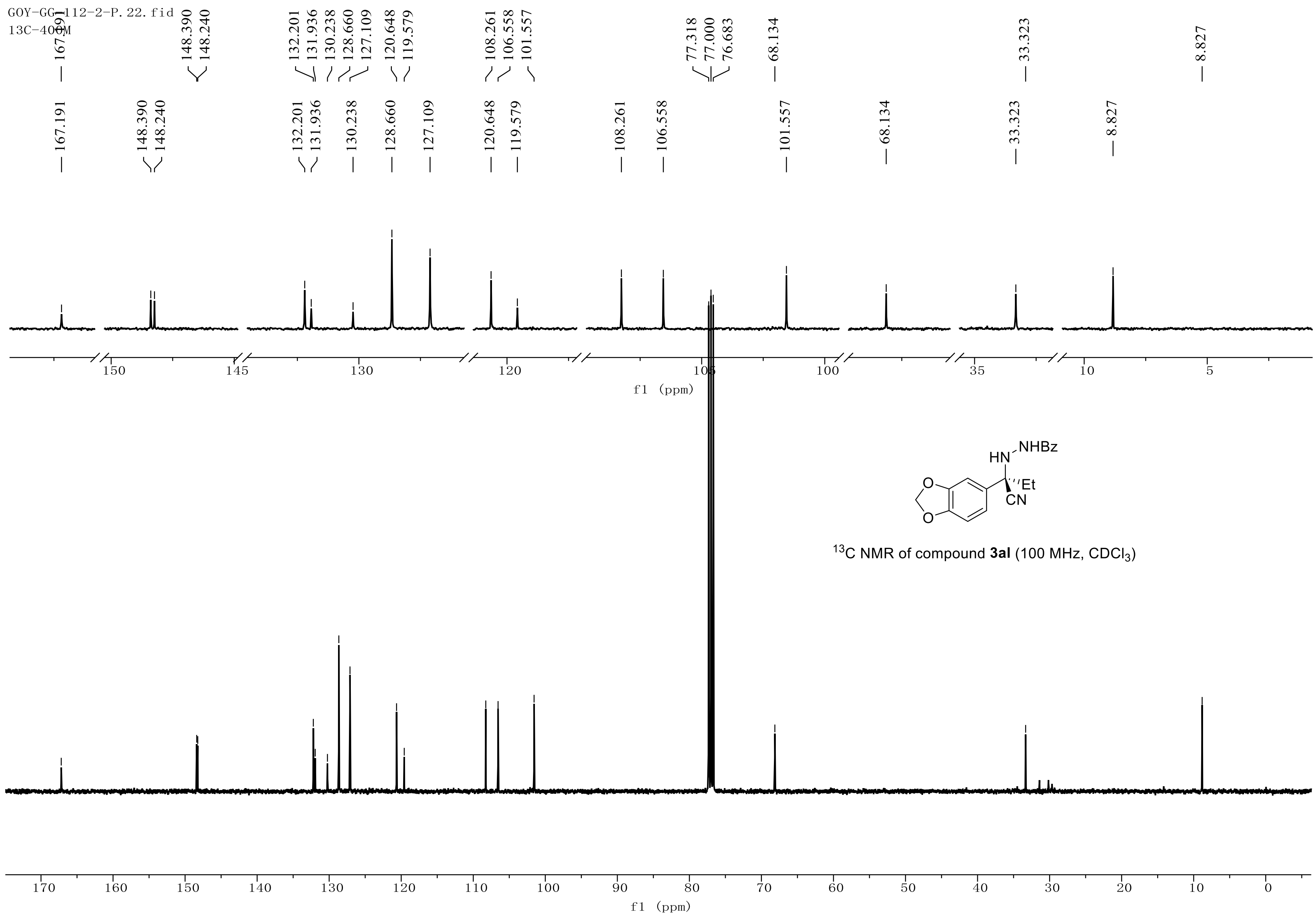

^{13}C NMR of compound **3al** (100 MHz, $CDCl_3$)

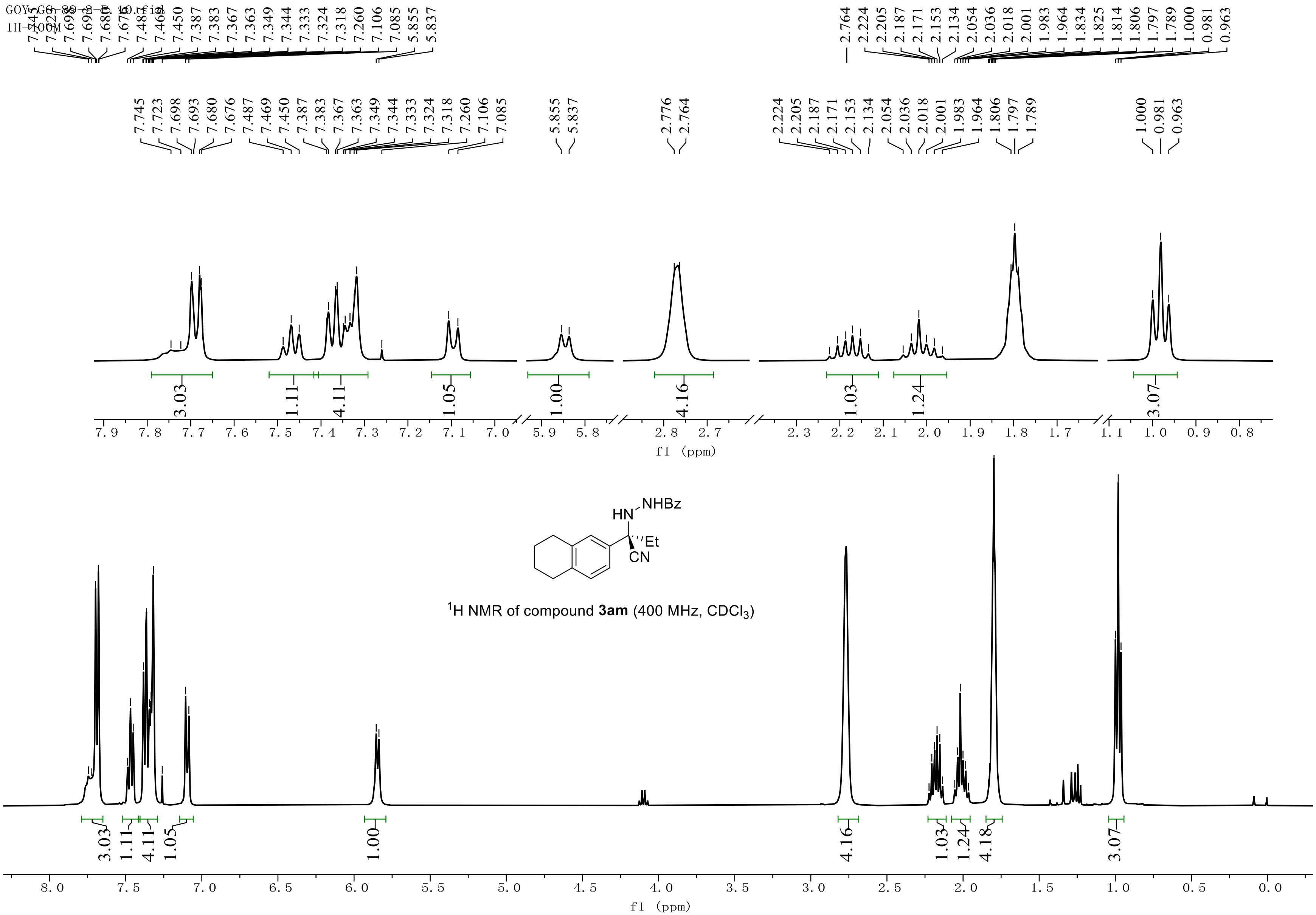

^{1}H NMR of compound **3am** (400 MHz, $CDCl_3$)

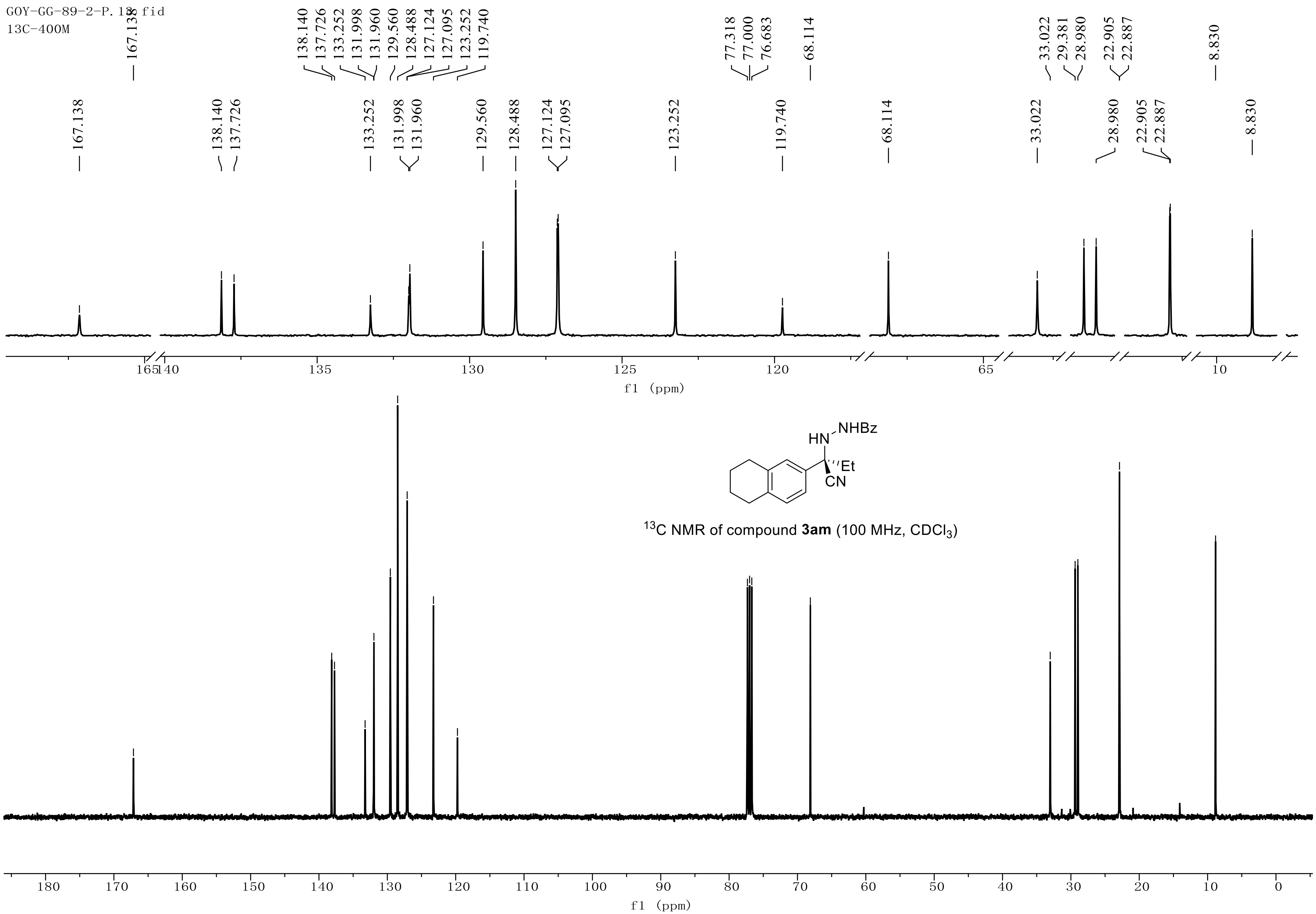

^{13}C NMR of compound **3am** (100 MHz, $CDCl_3$)

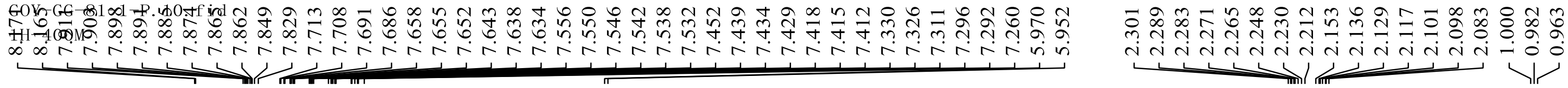

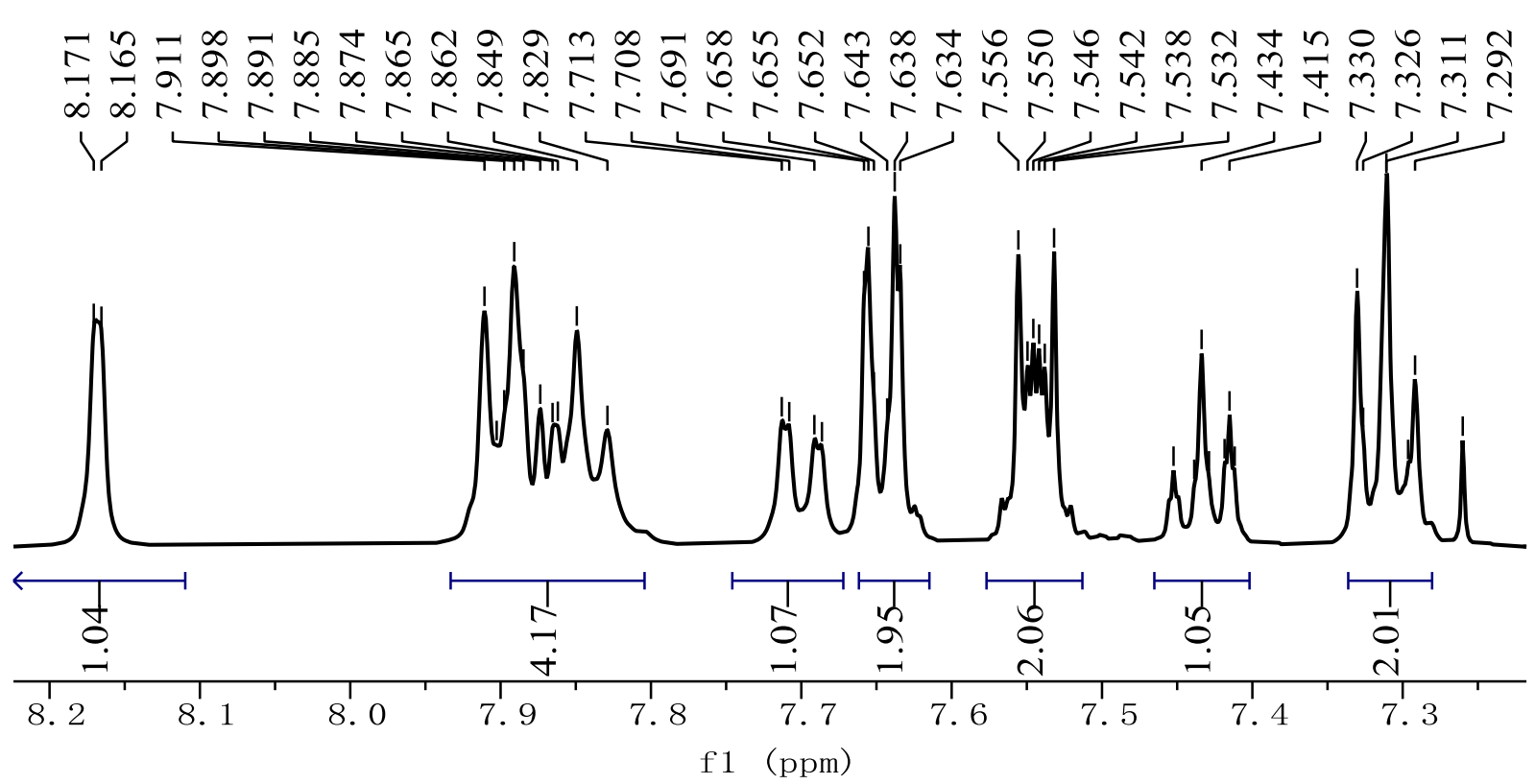

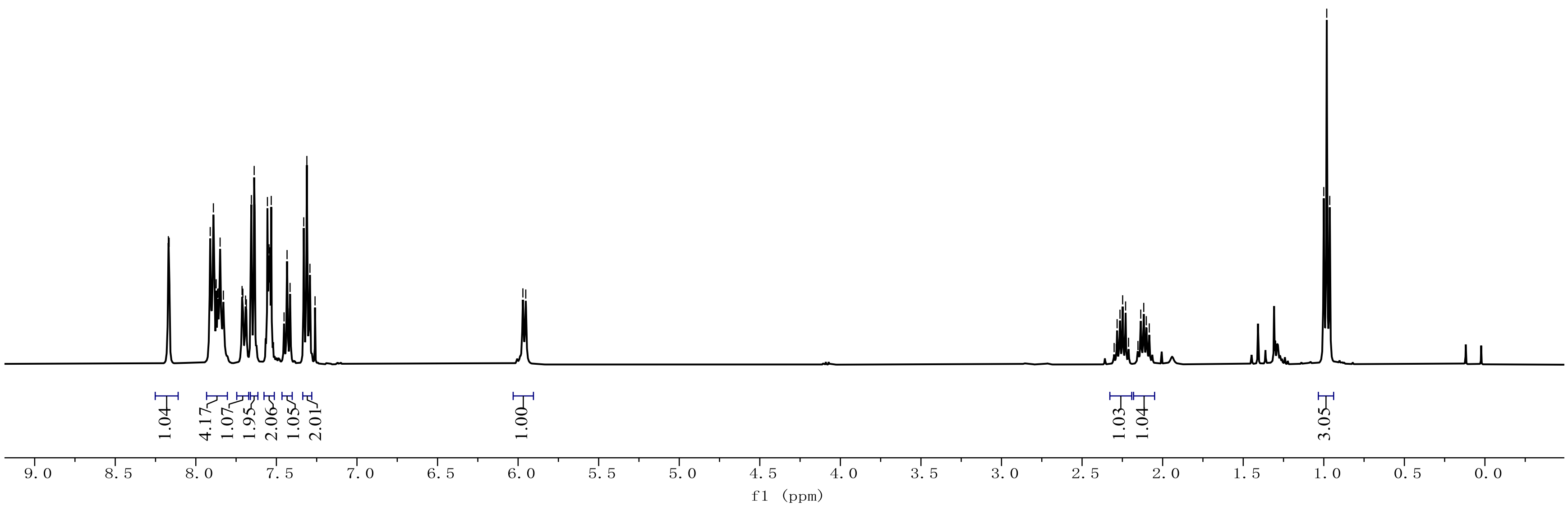

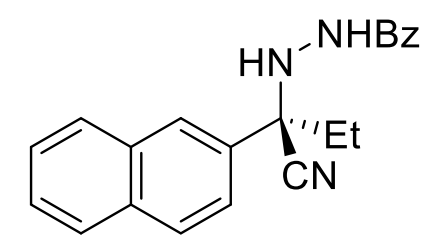

^{1}H NMR of compound **3an** (400 MHz, $CDCl_3$)

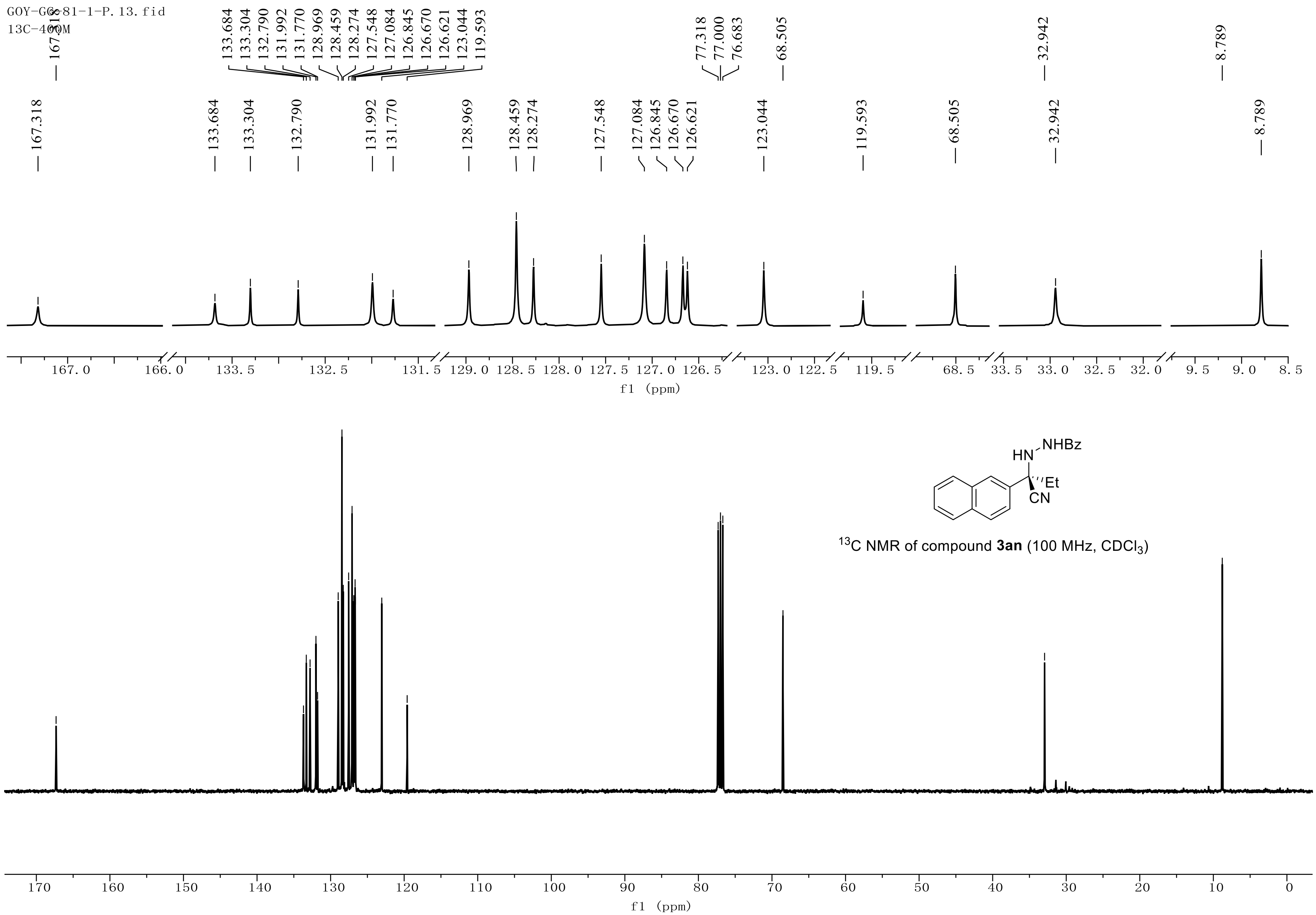
GOY-GG-81-1-P.13.fid
13C-400M
167.318
133.684
133.304
132.790
131.992
131.770
128.969
128.459
128.274
127.548
127.084
126.845
126.670
126.621
123.044
119.593
77.318
77.000
76.683
68.505
32.942
8.789
f1 (ppm)
HN
NHBz
Et
CN
^{13}C NMR of compound **3an** (100 MHz, $CDCl_3$)
f1 (ppm)

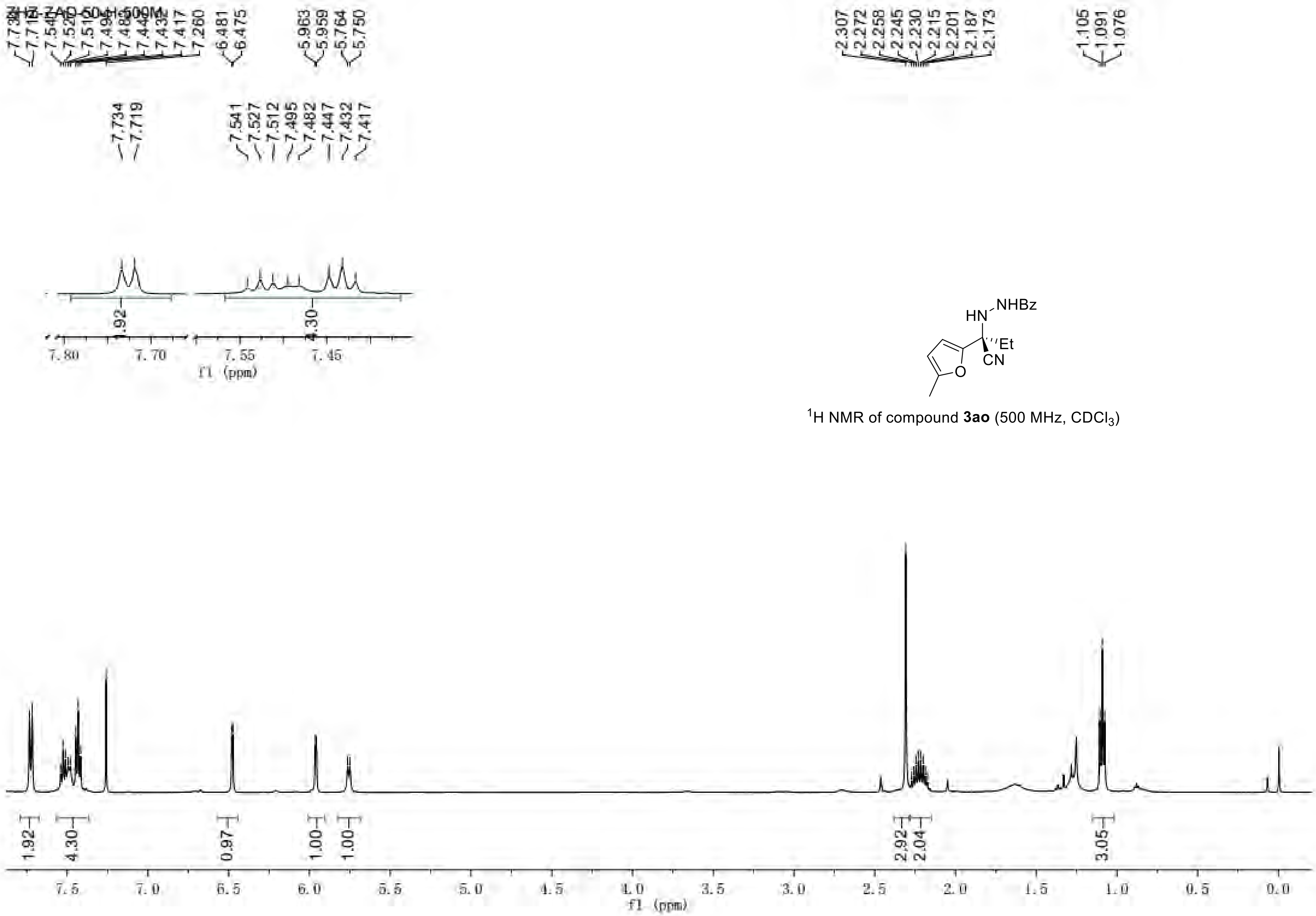

^{1}H NMR of compound **3ao** (500 MHz, $CDCl_3$)

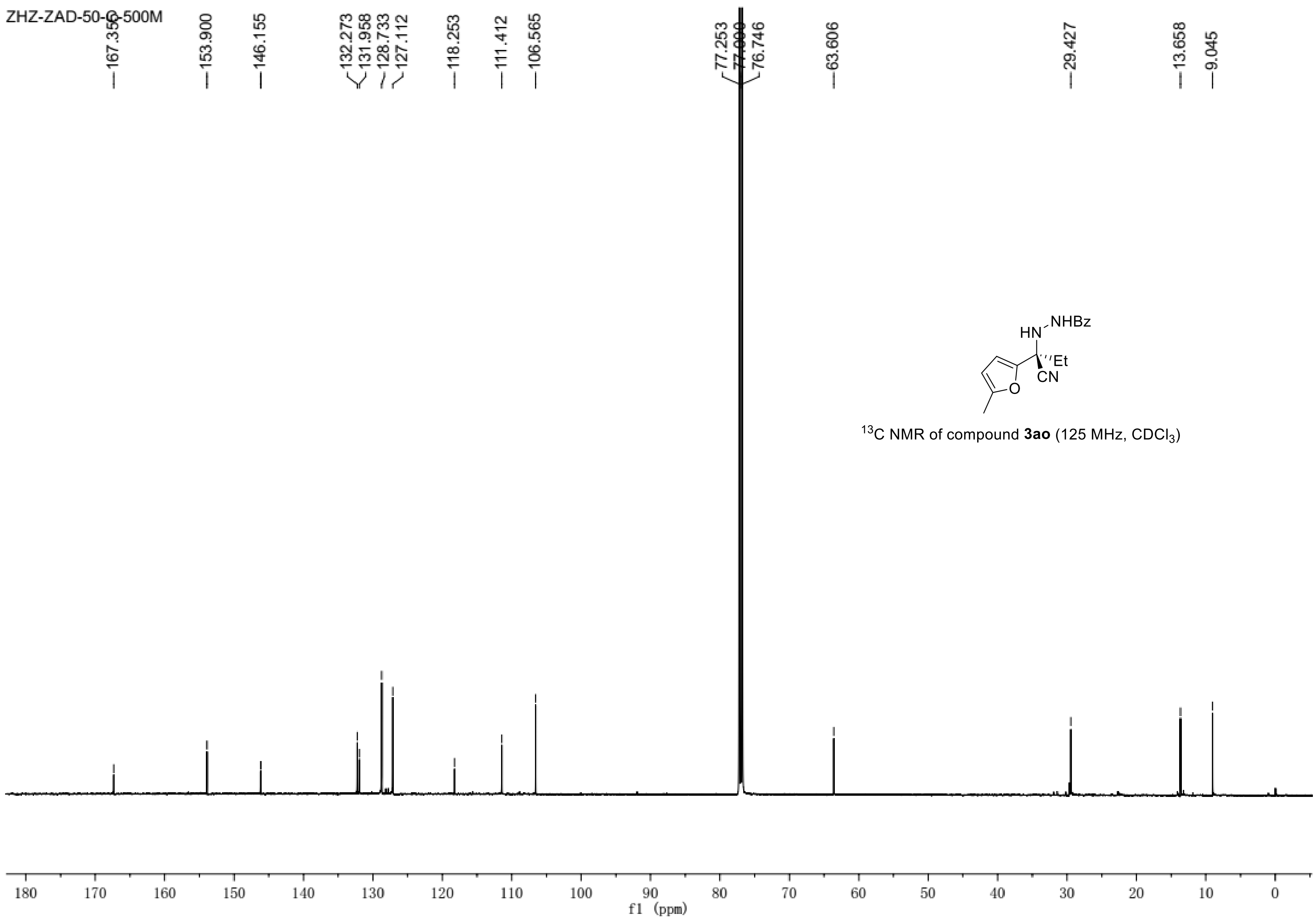

^{13}C NMR of compound **3ao** (125 MHz, $CDCl_3$)

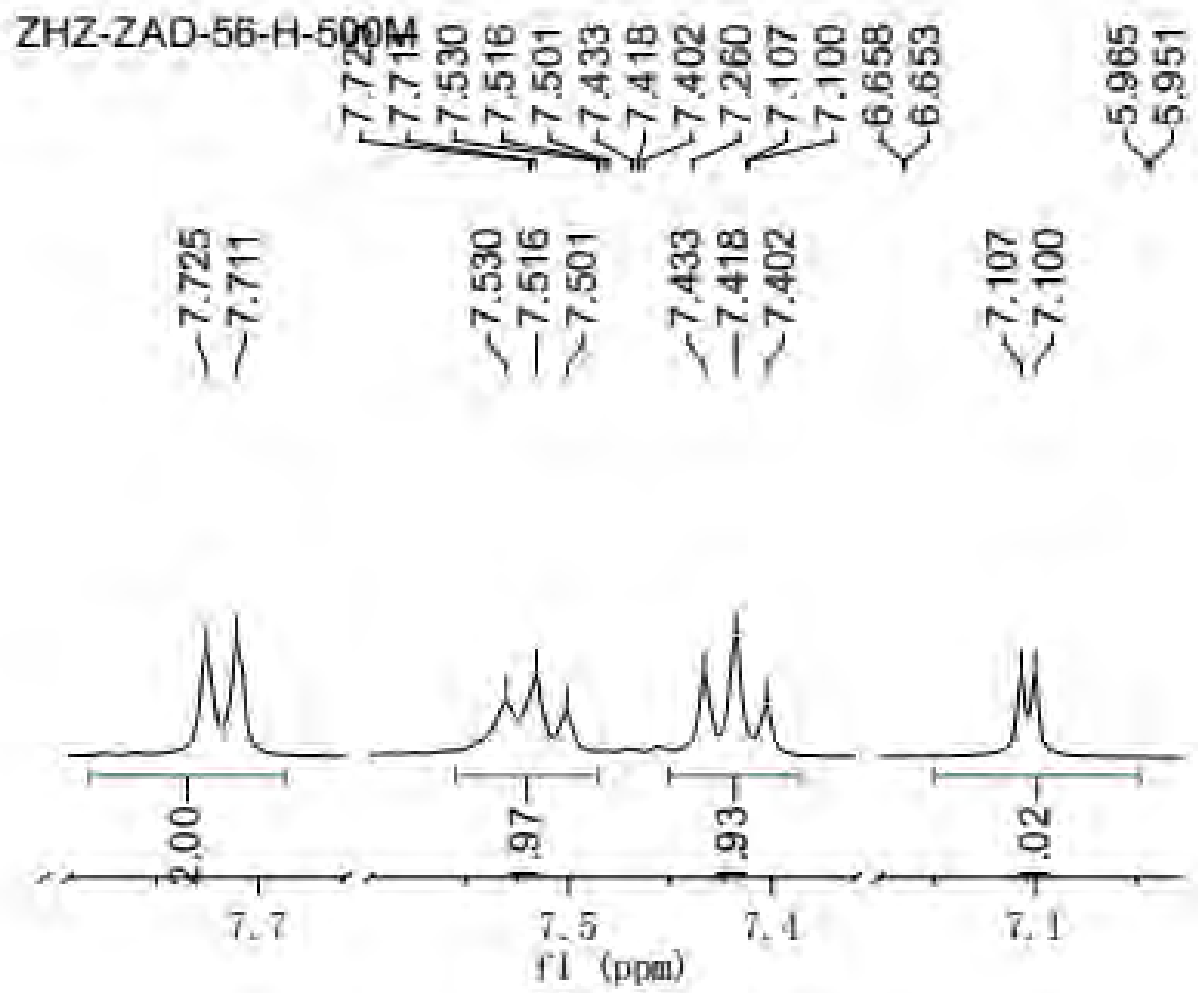

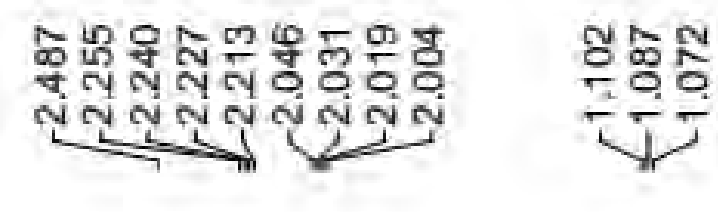

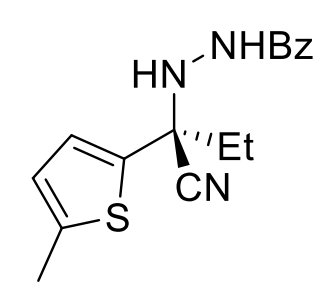

^{1}H NMR of compound **3ap** (500 MHz, $CDCl_3$)

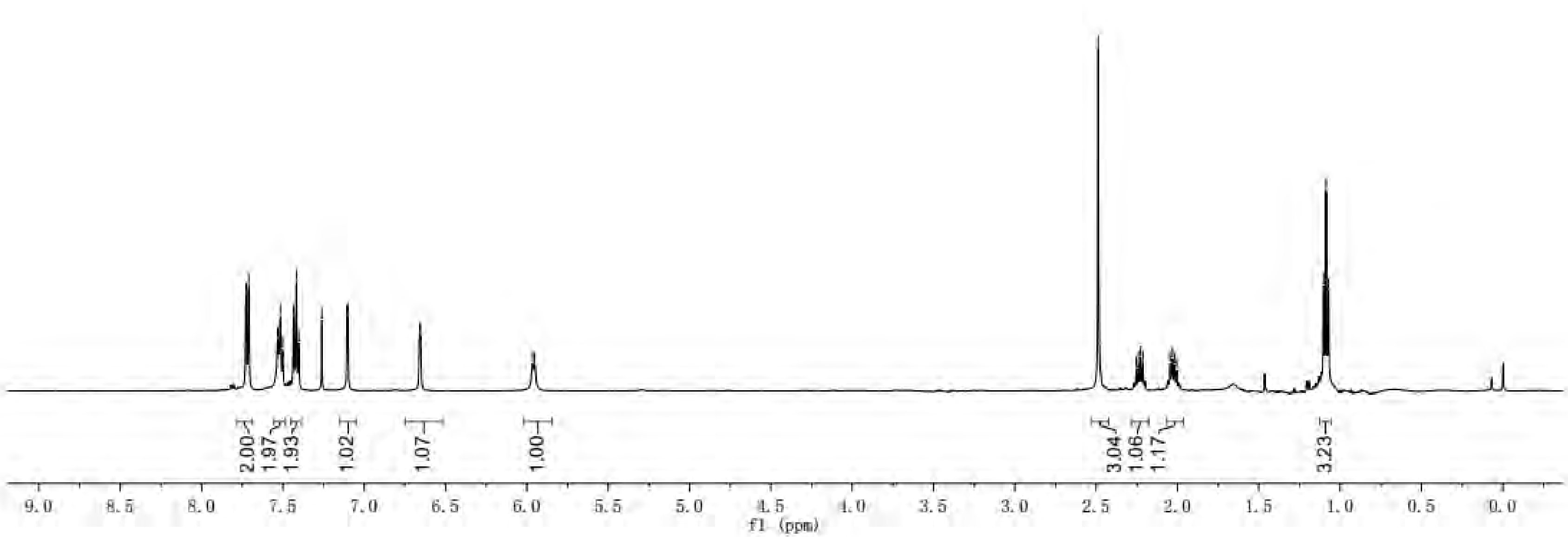

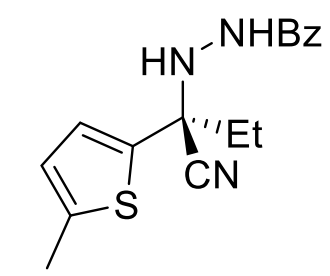

^{13}C NMR of compound **3ap** (125 MHz, $CDCl_3$)

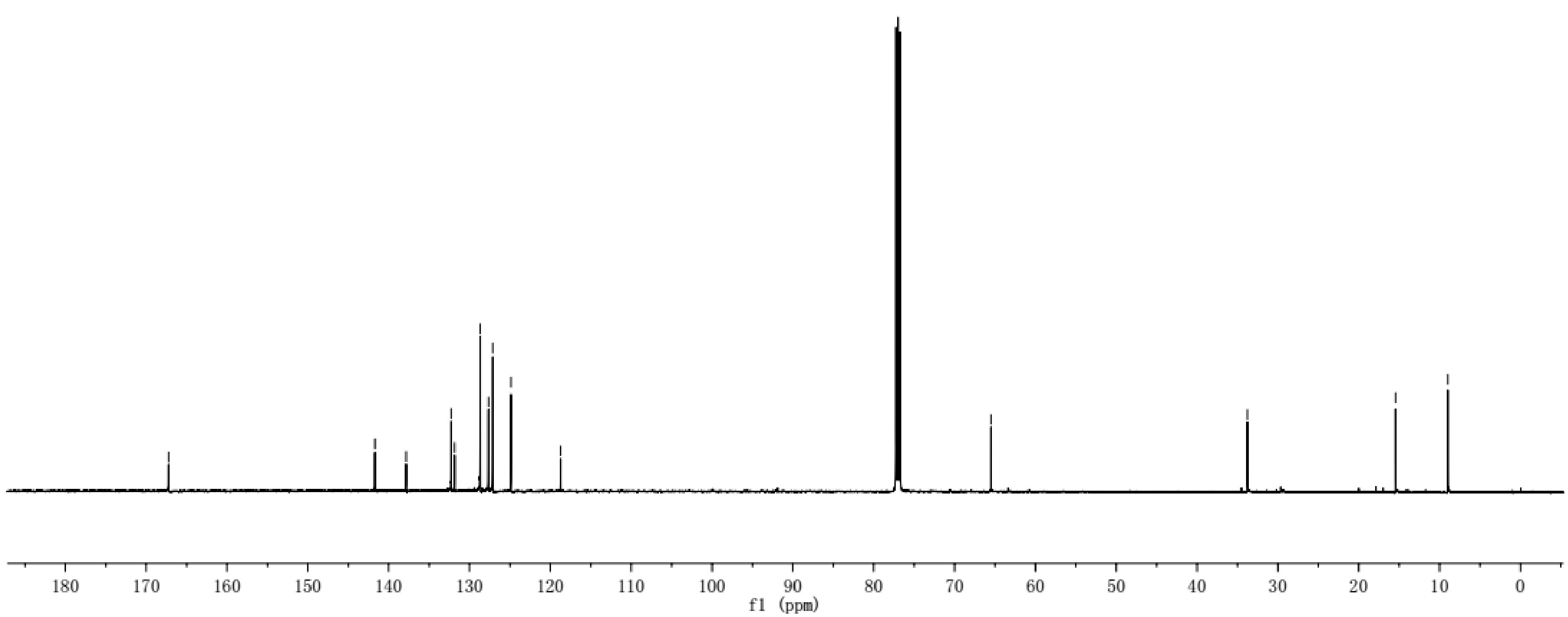

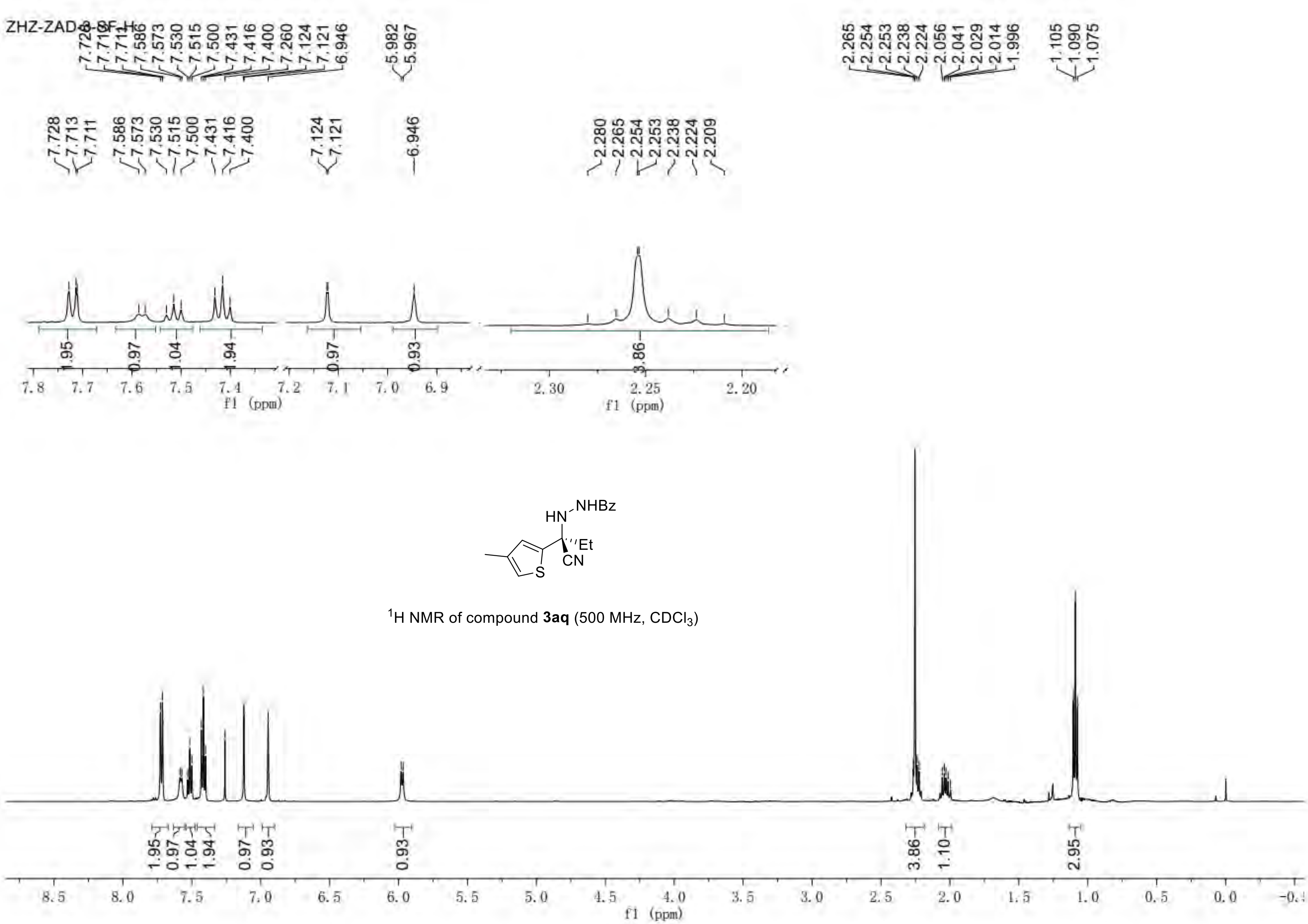

^{1}H NMR of compound **3aq** (500 MHz, $CDCl_3$)

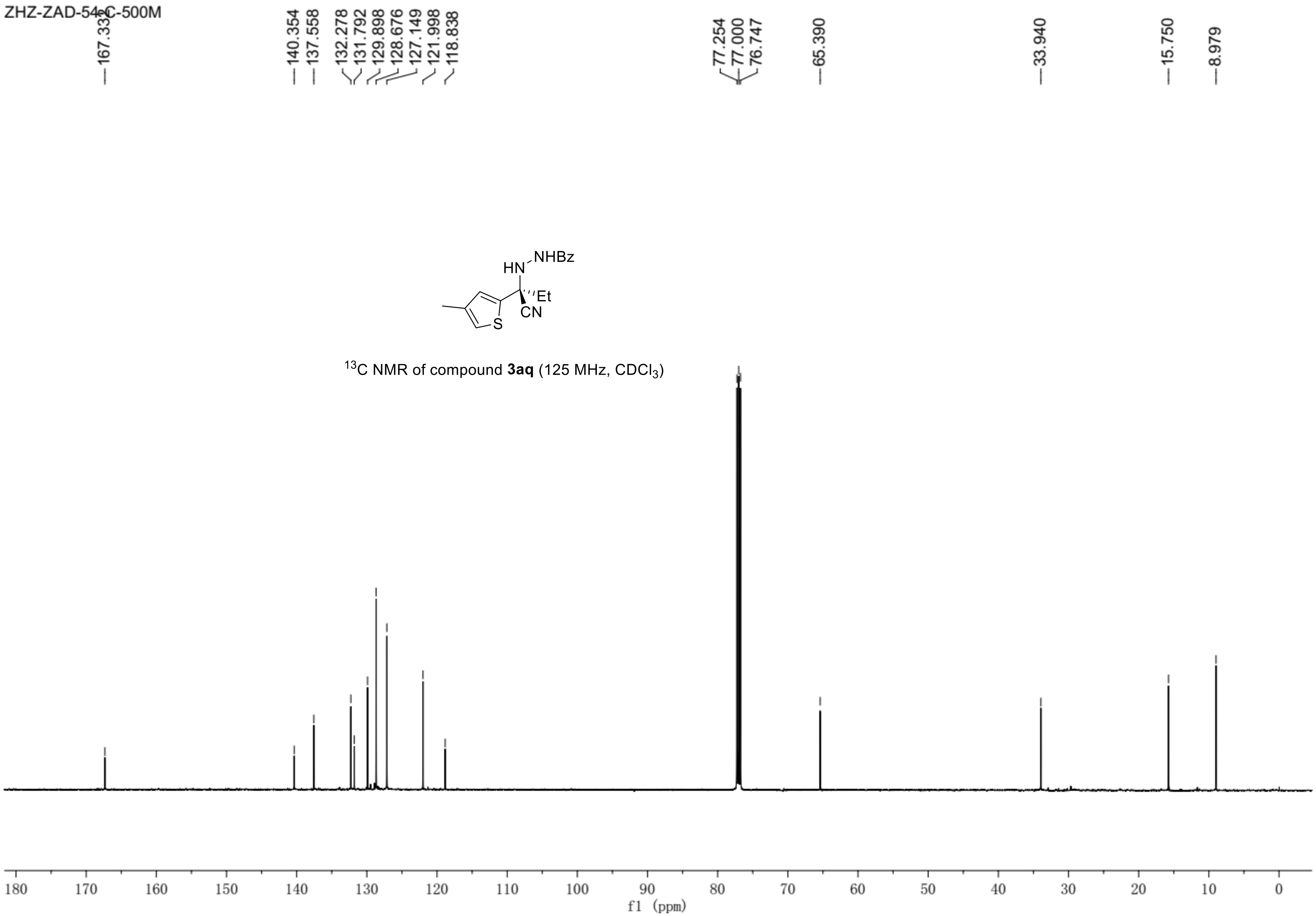

^{13}C NMR of compound **3aq** (125 MHz, $CDCl_3$)

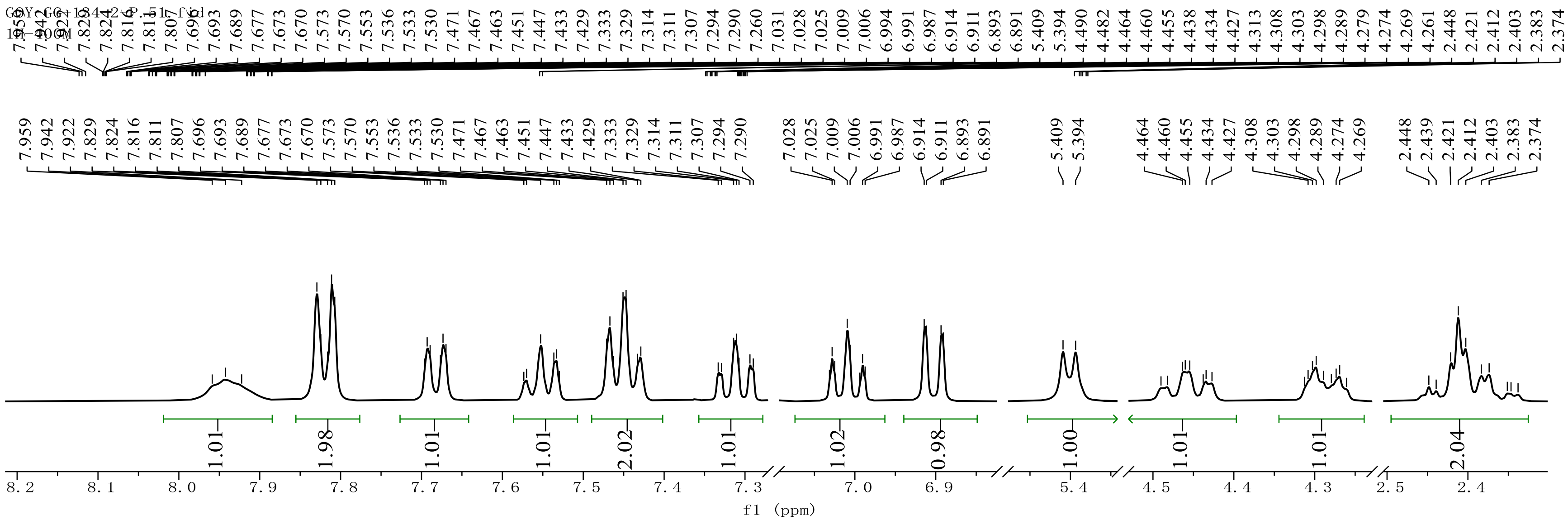

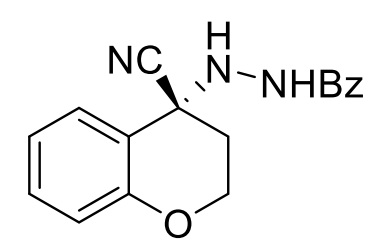

^{1}H NMR of compound **3ar** (400 MHz, $CDCl_3$)

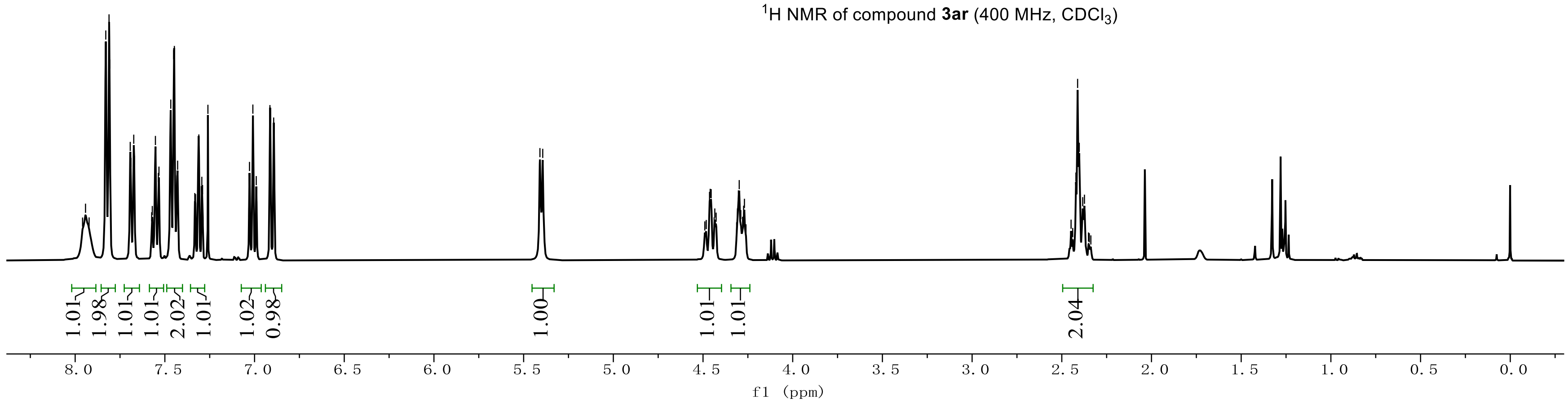

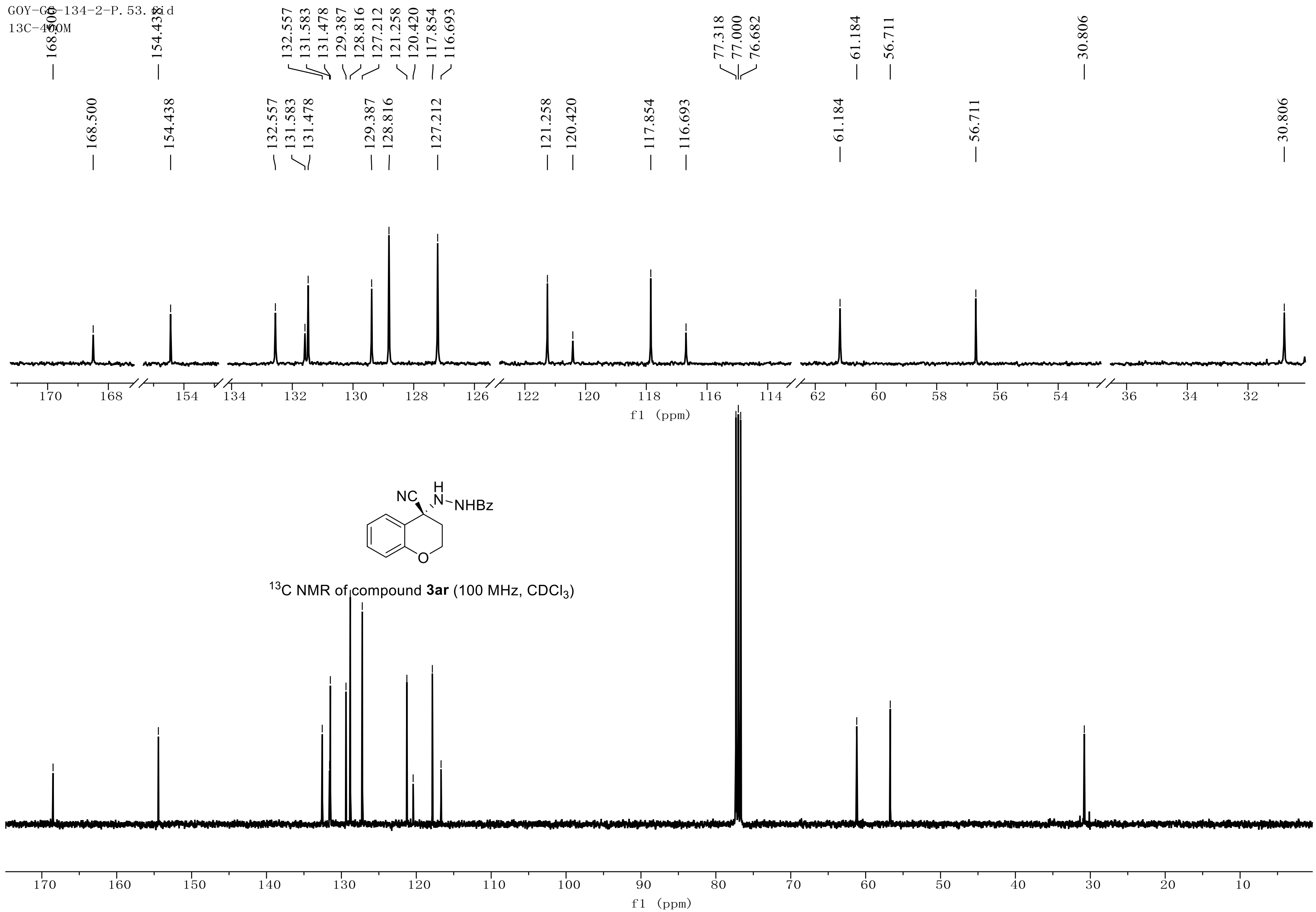

^{13}C NMR of compound **3ar** (100 MHz, $CDCl_3$)

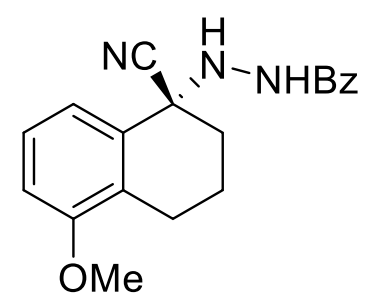

^{1}H NMR of compound **3as** (400 MHz, $CDCl_3$)

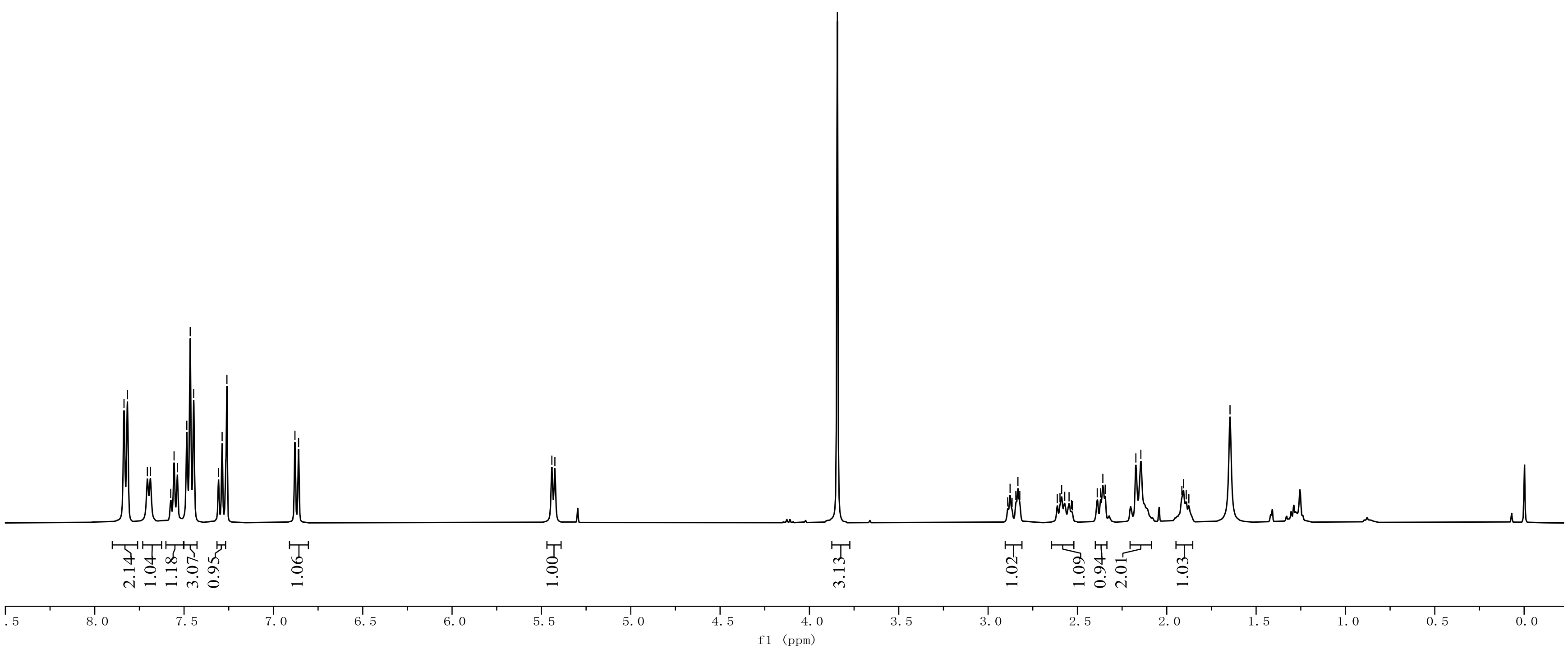

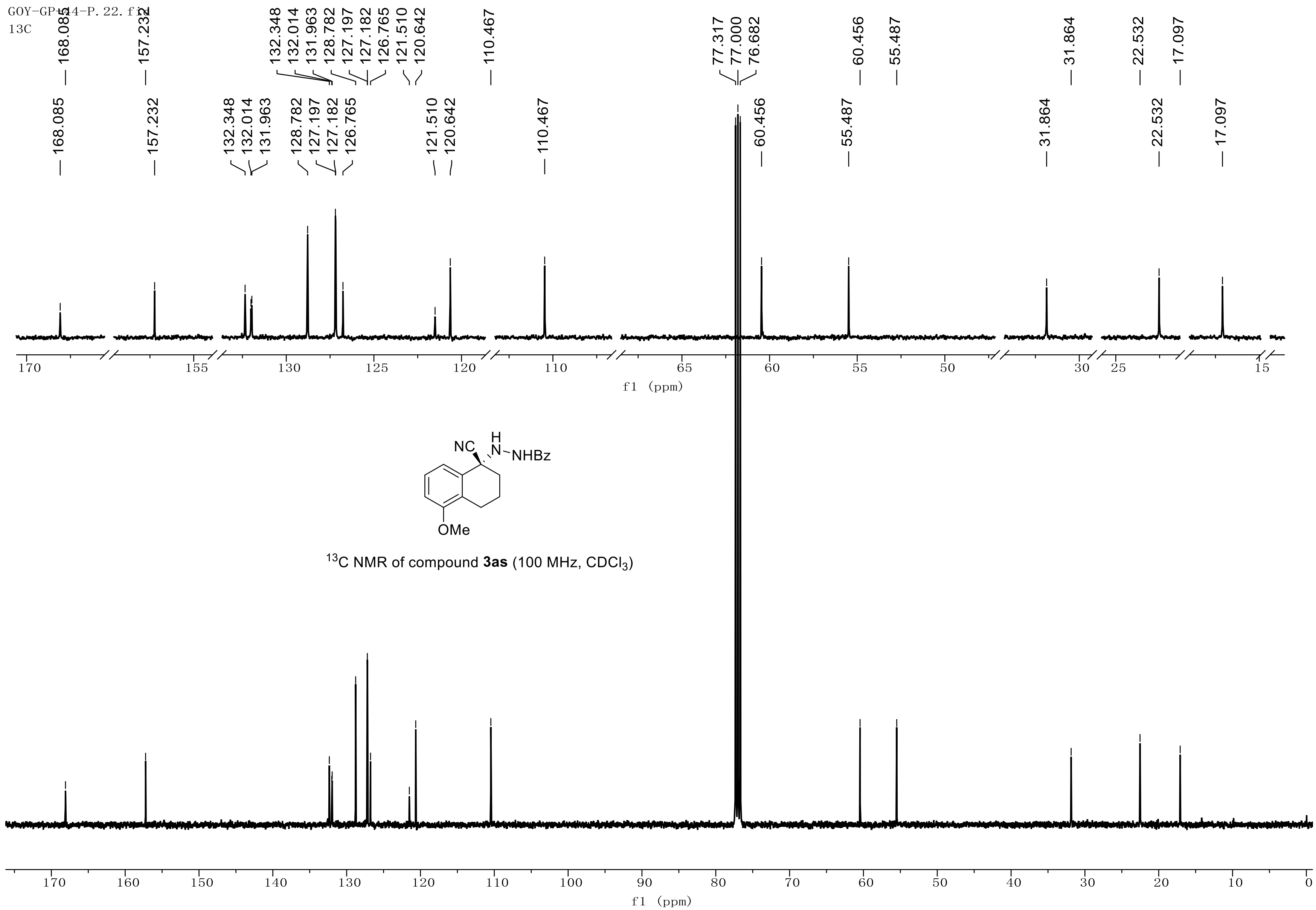

^{13}C NMR of compound **3as** (100 MHz, $CDCl_3$)

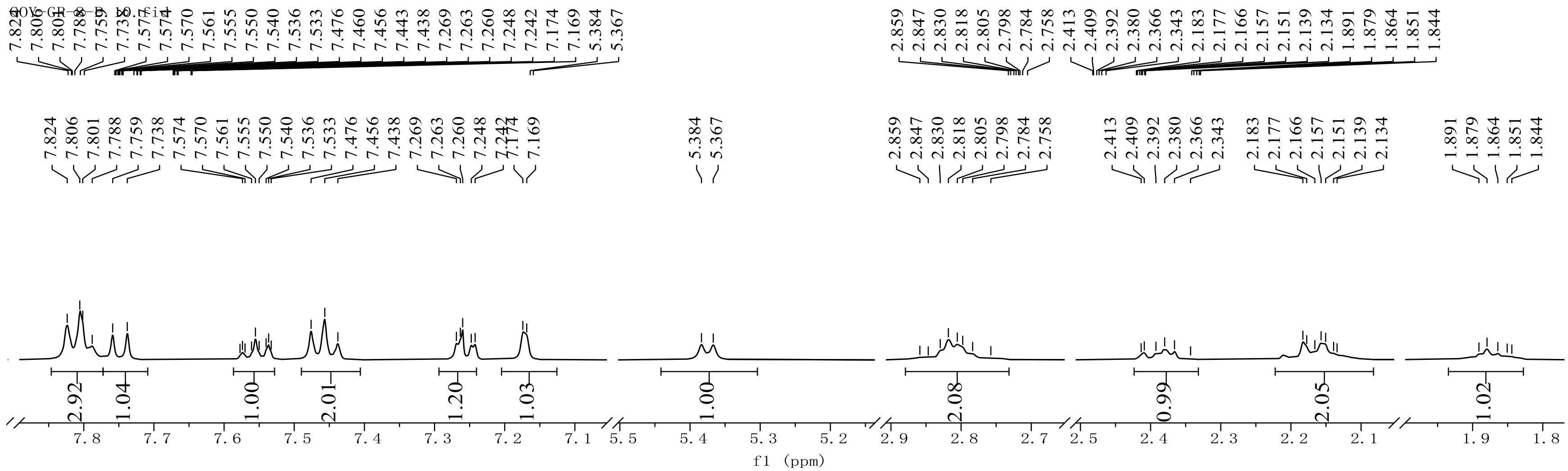

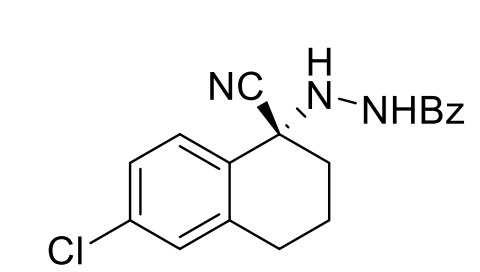

^{1}H NMR of compound **3at** (400 MHz, $CDCl_3$)

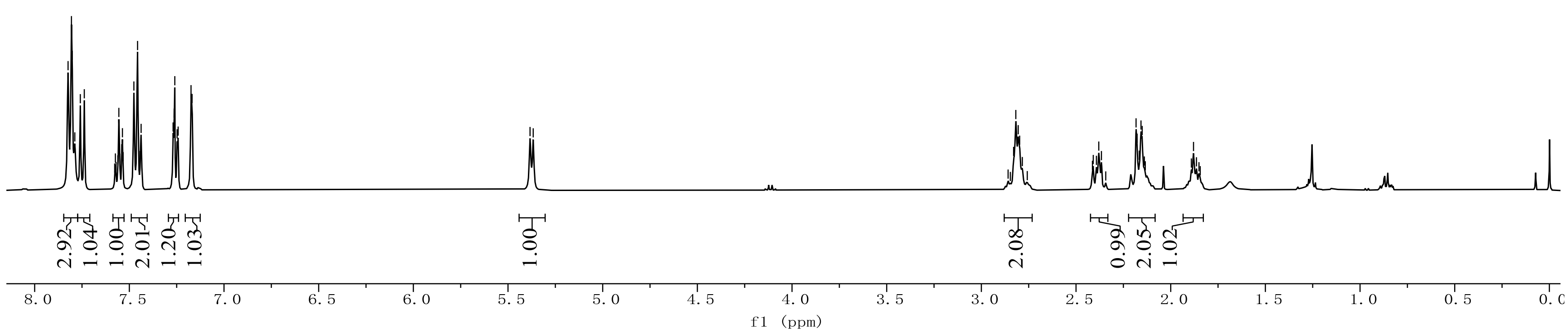

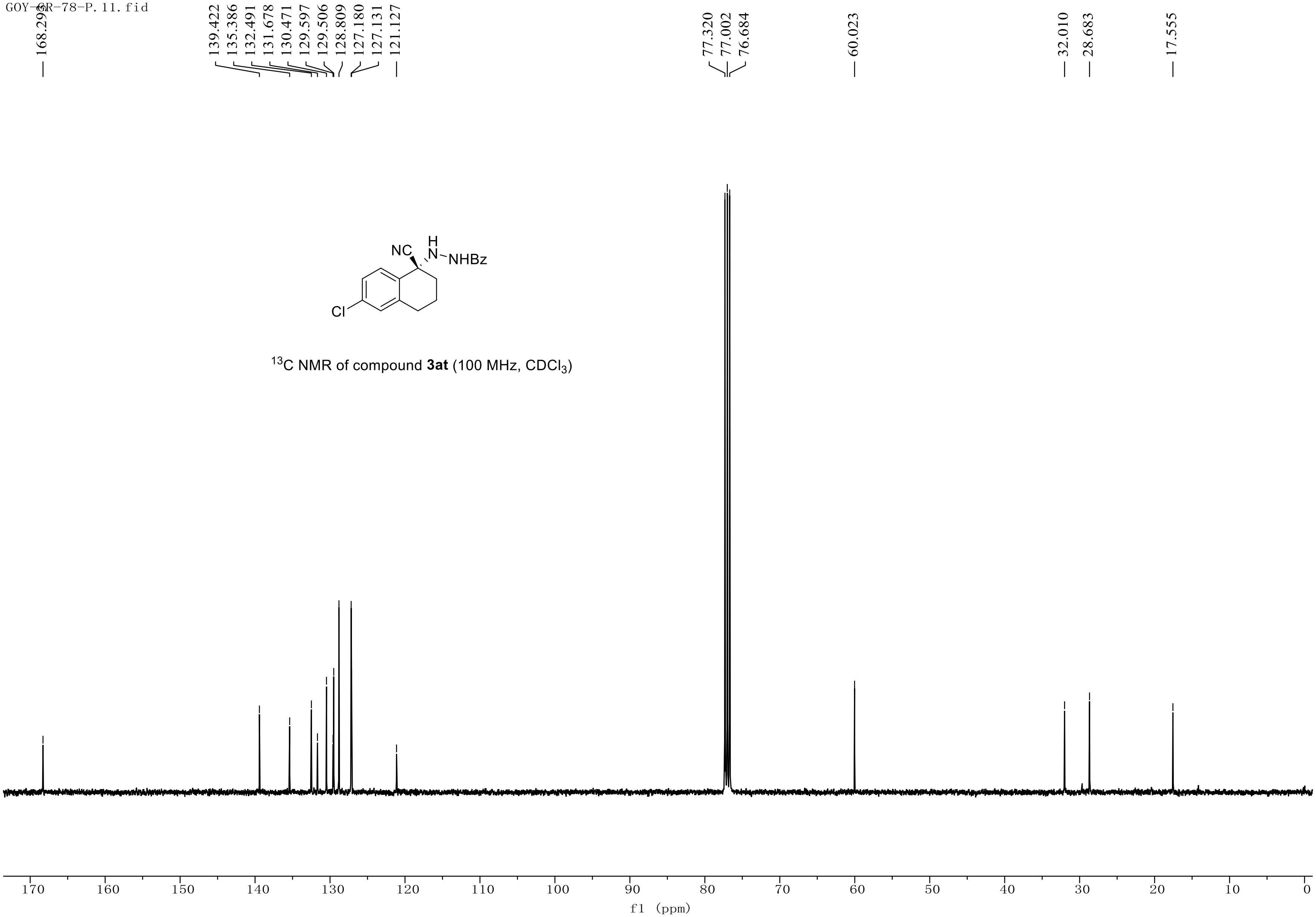

^{13}C NMR of compound **3at** (100 MHz, $CDCl_3$)

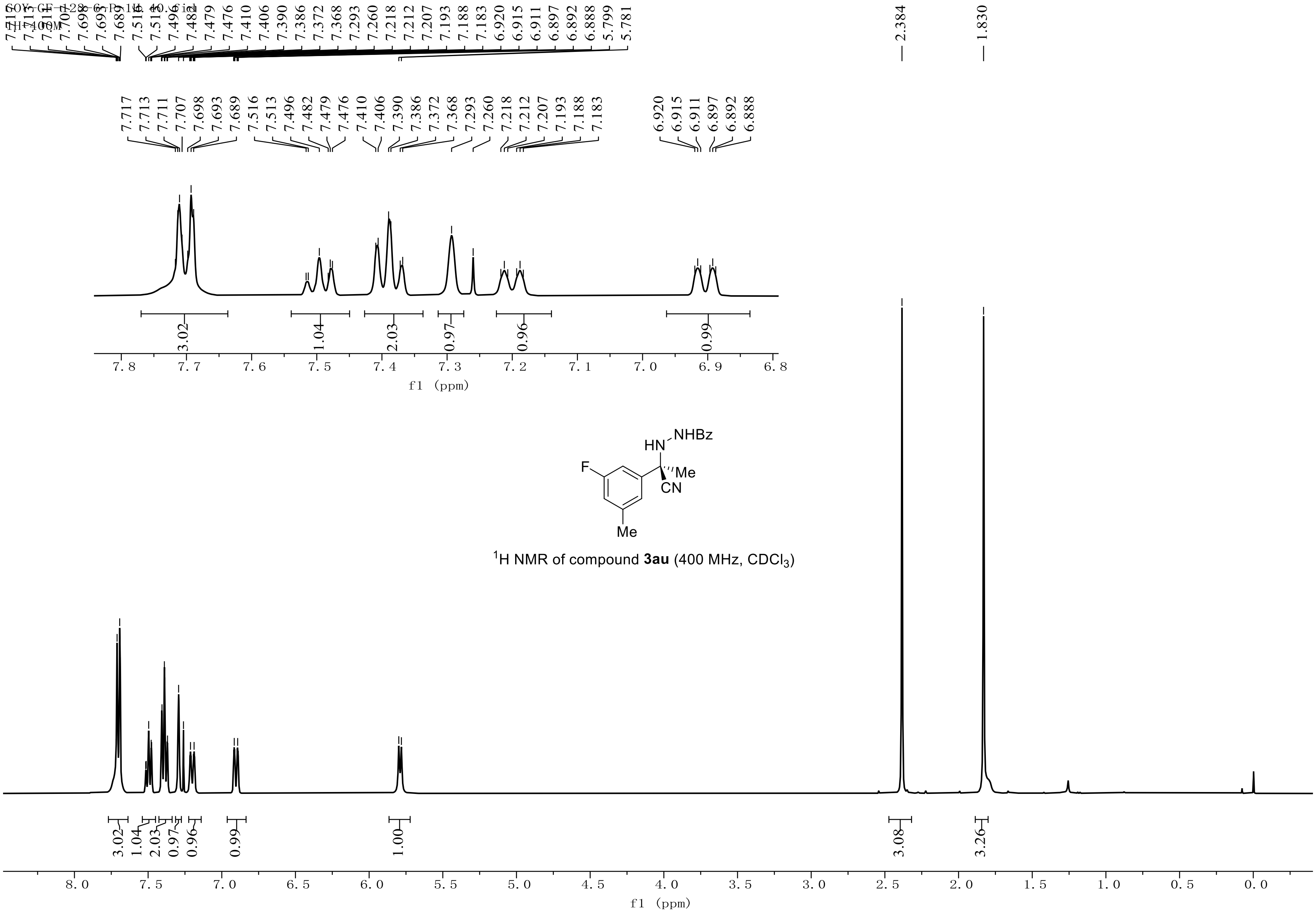

^{1}H NMR of compound **3au** (400 MHz, $CDCl_3$)

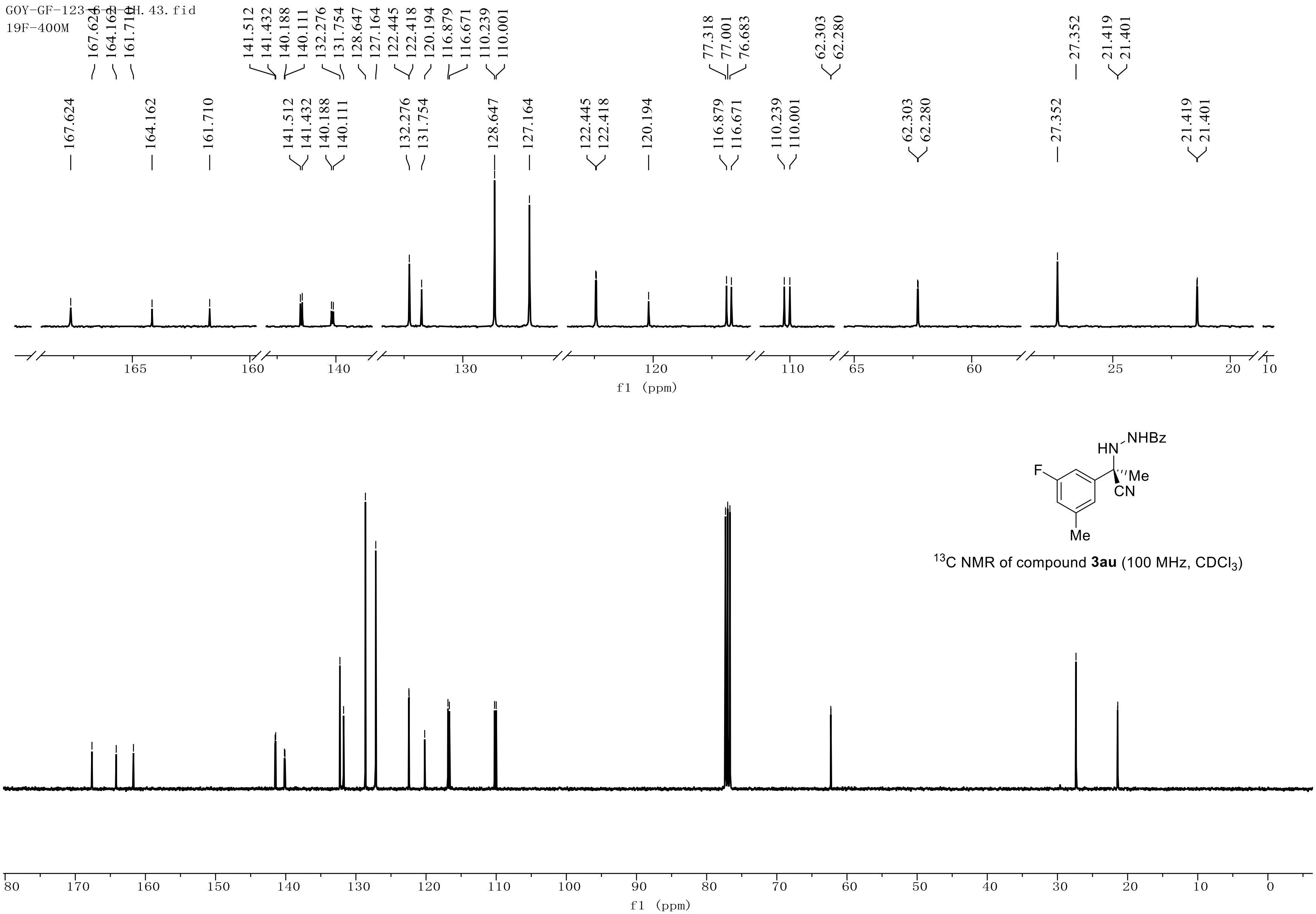

^{13}C NMR of compound **3au** (100 MHz, $CDCl_3$)

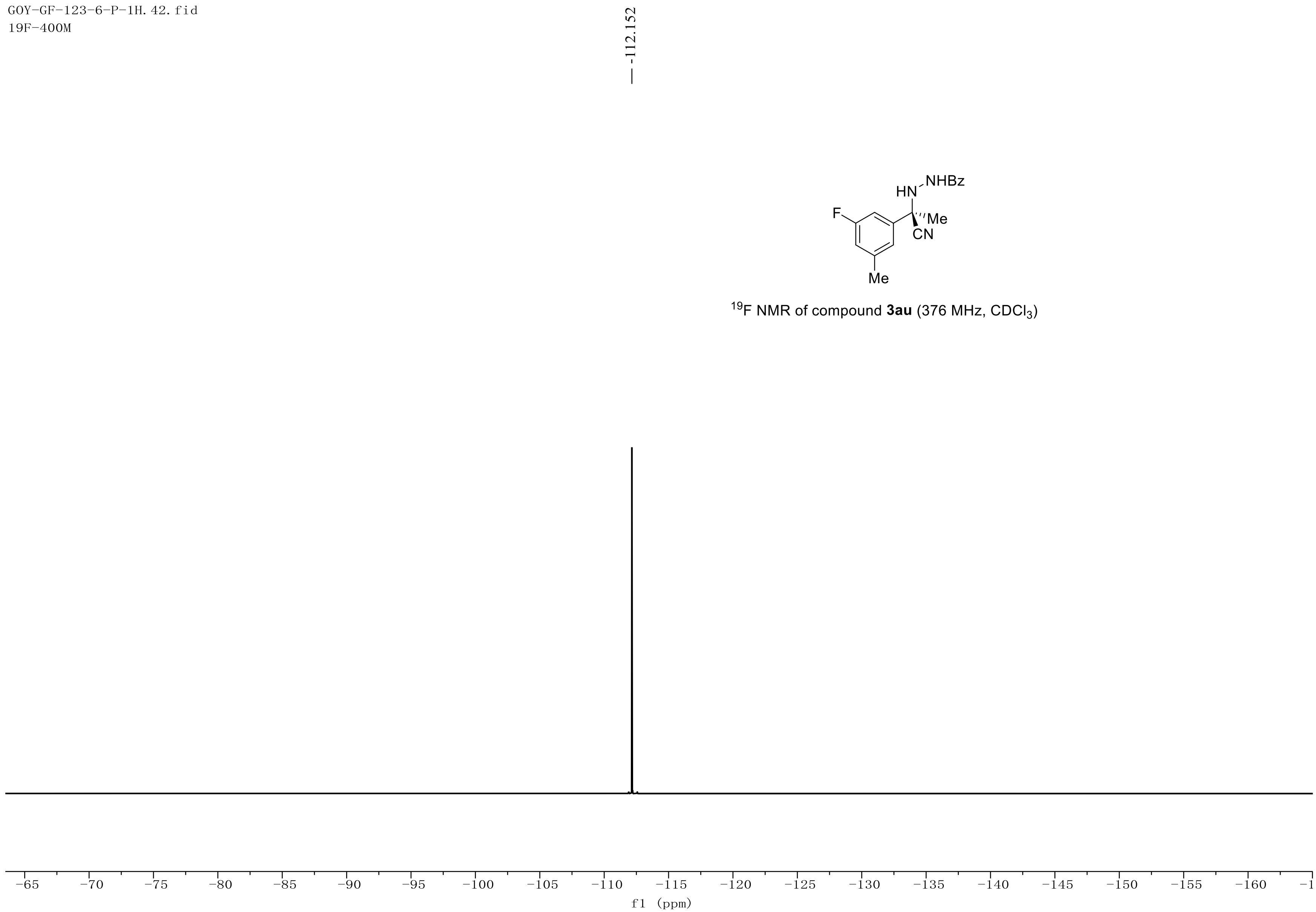

^{19}F NMR of compound **3au** (376 MHz, $CDCl_3$)

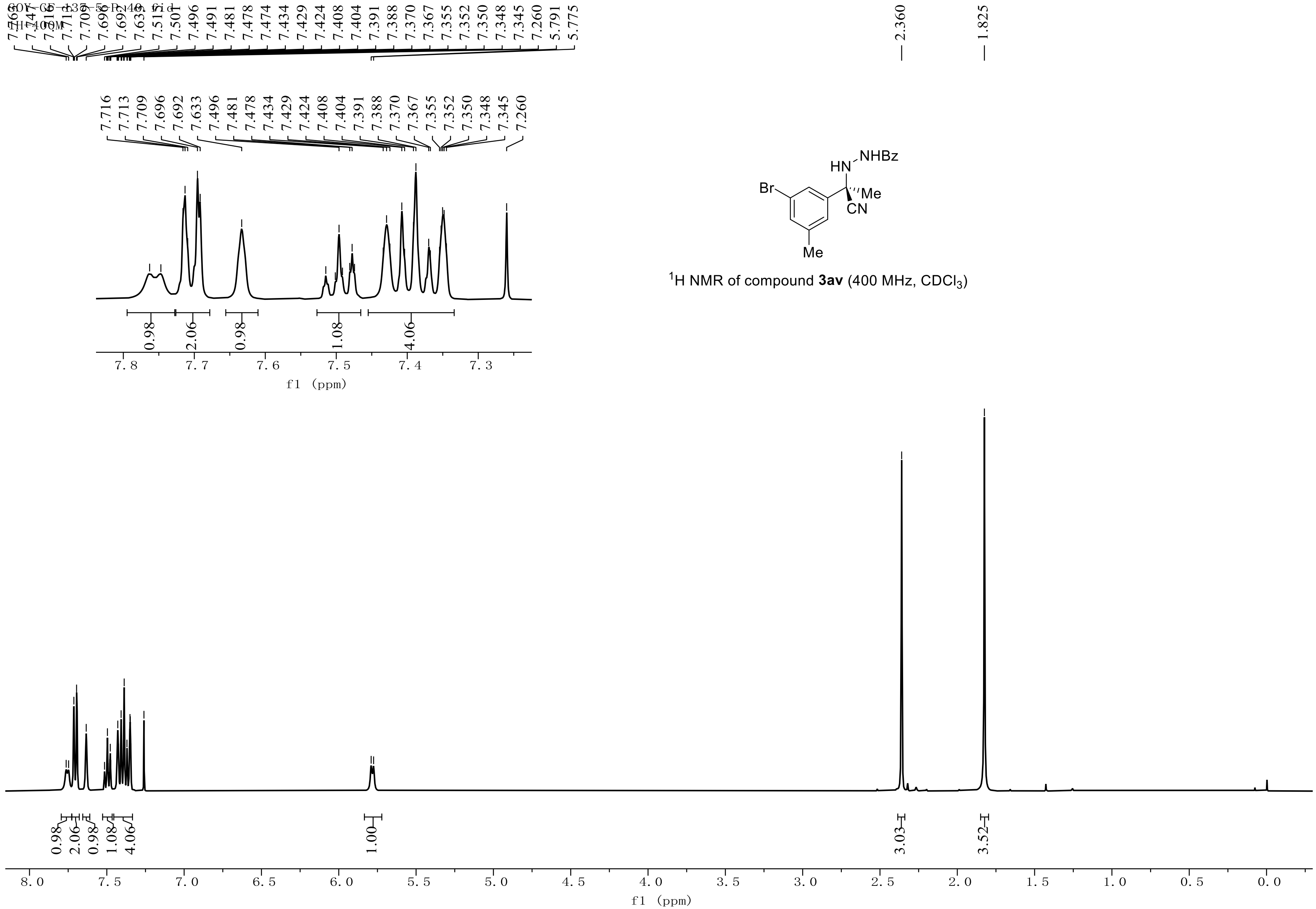

^{1}H NMR of compound **3av** (400 MHz, $CDCl_3$)

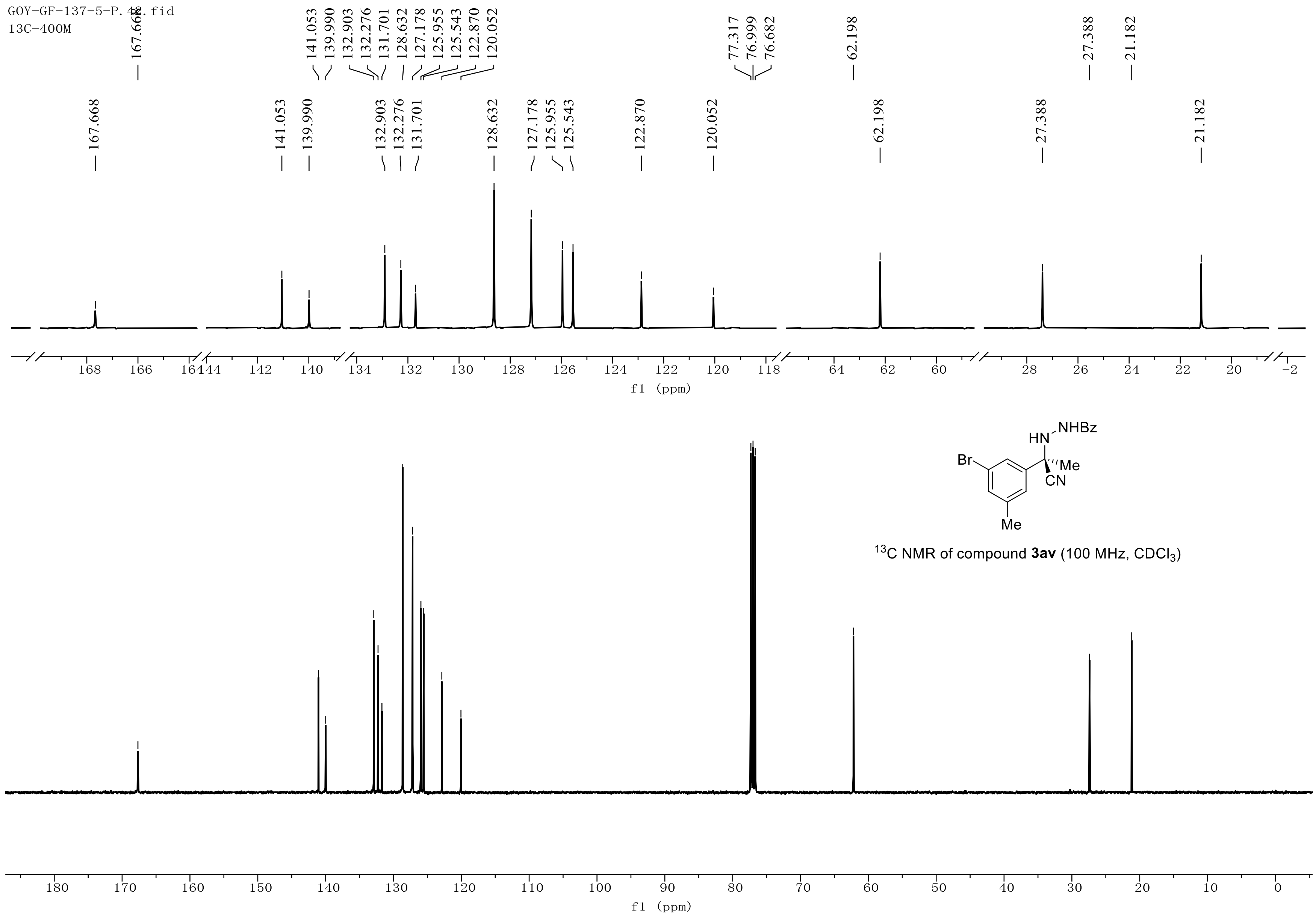

^{13}C NMR of compound **3av** (100 MHz, $CDCl_3$)

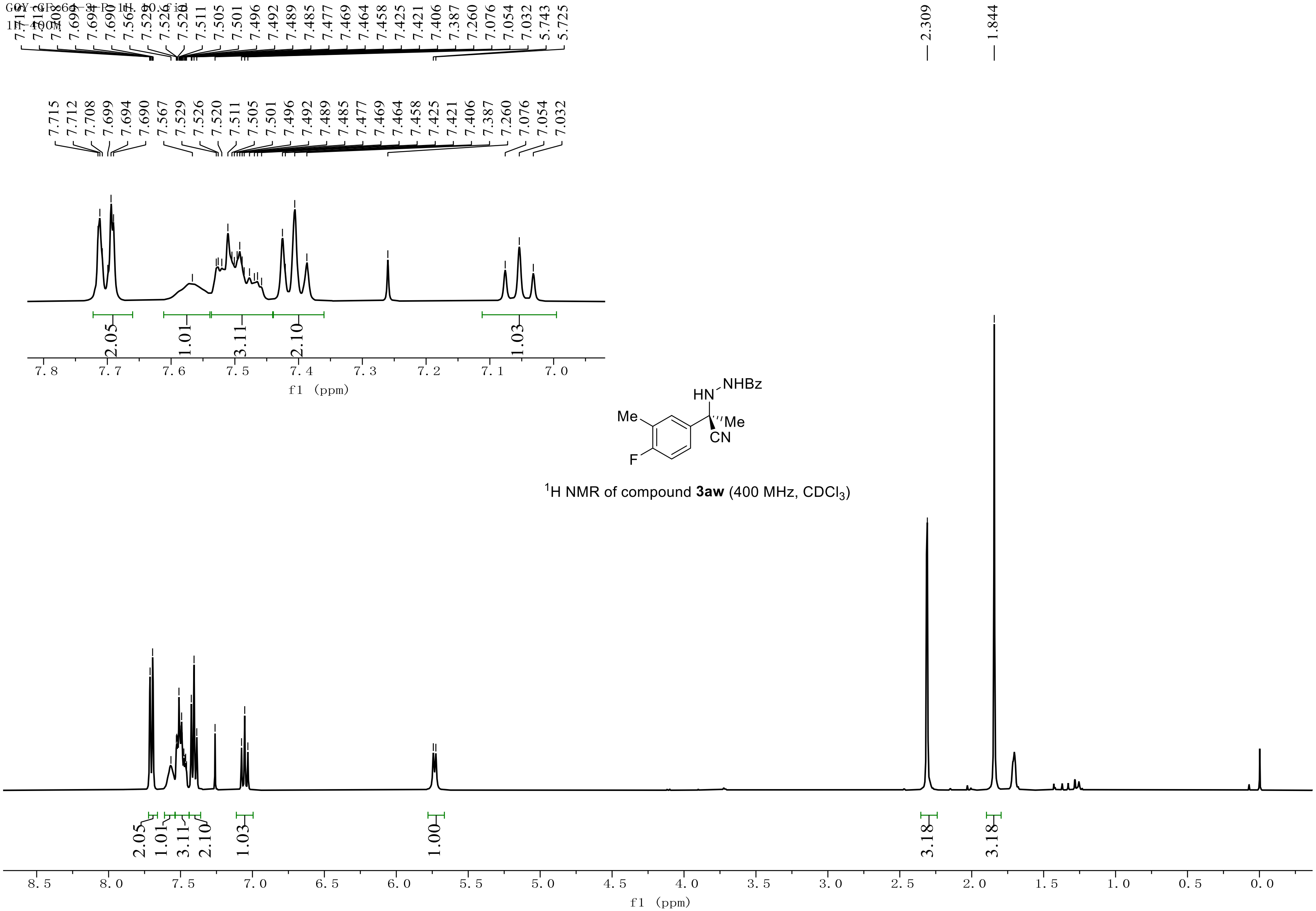

^{1}H NMR of compound **3aw** (400 MHz, $CDCl_3$)

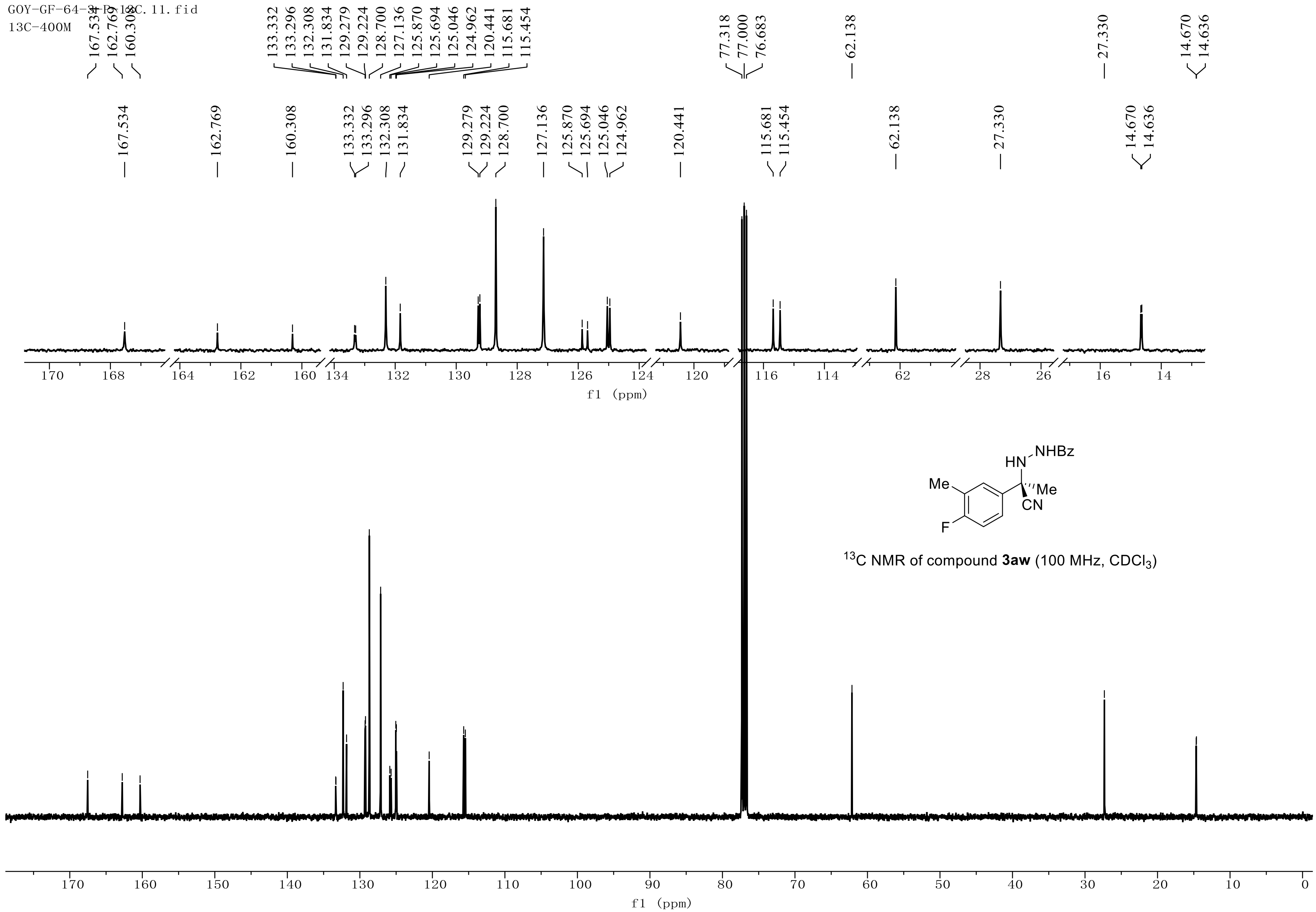

^{13}C NMR of compound **3aw** (100 MHz, $CDCl_3$)

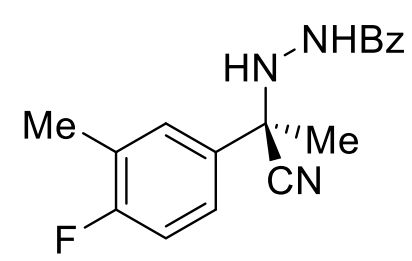

^{19}F NMR of compound **3aw** (376 MHz, $CDCl_3$)

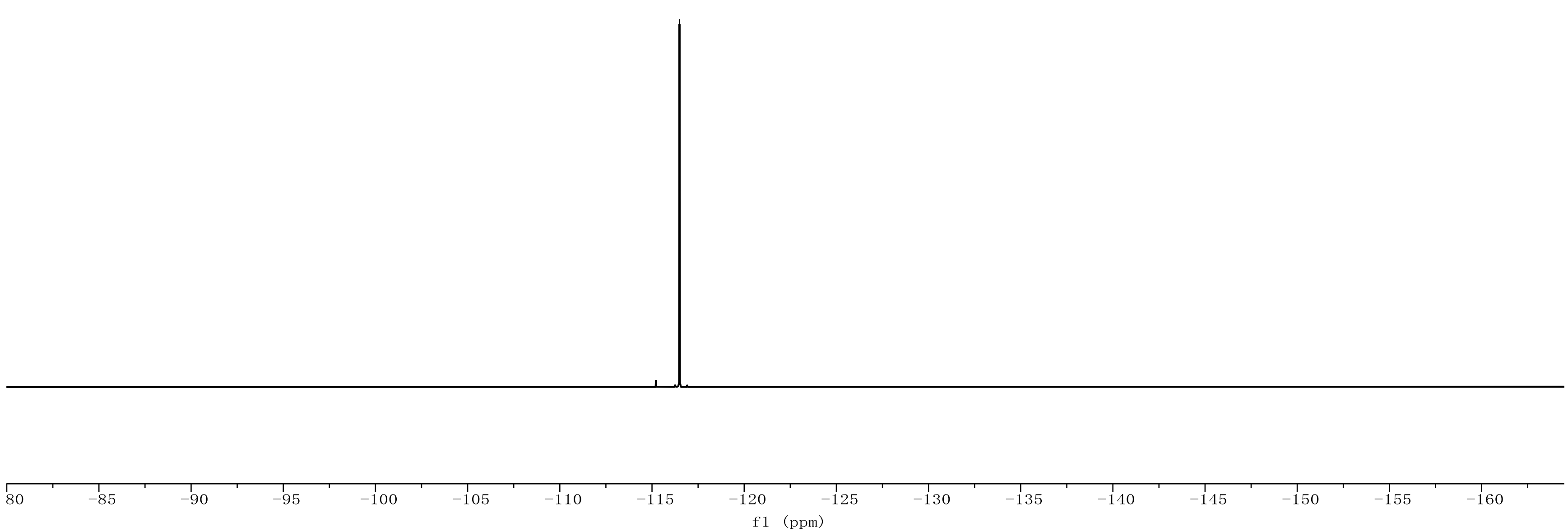

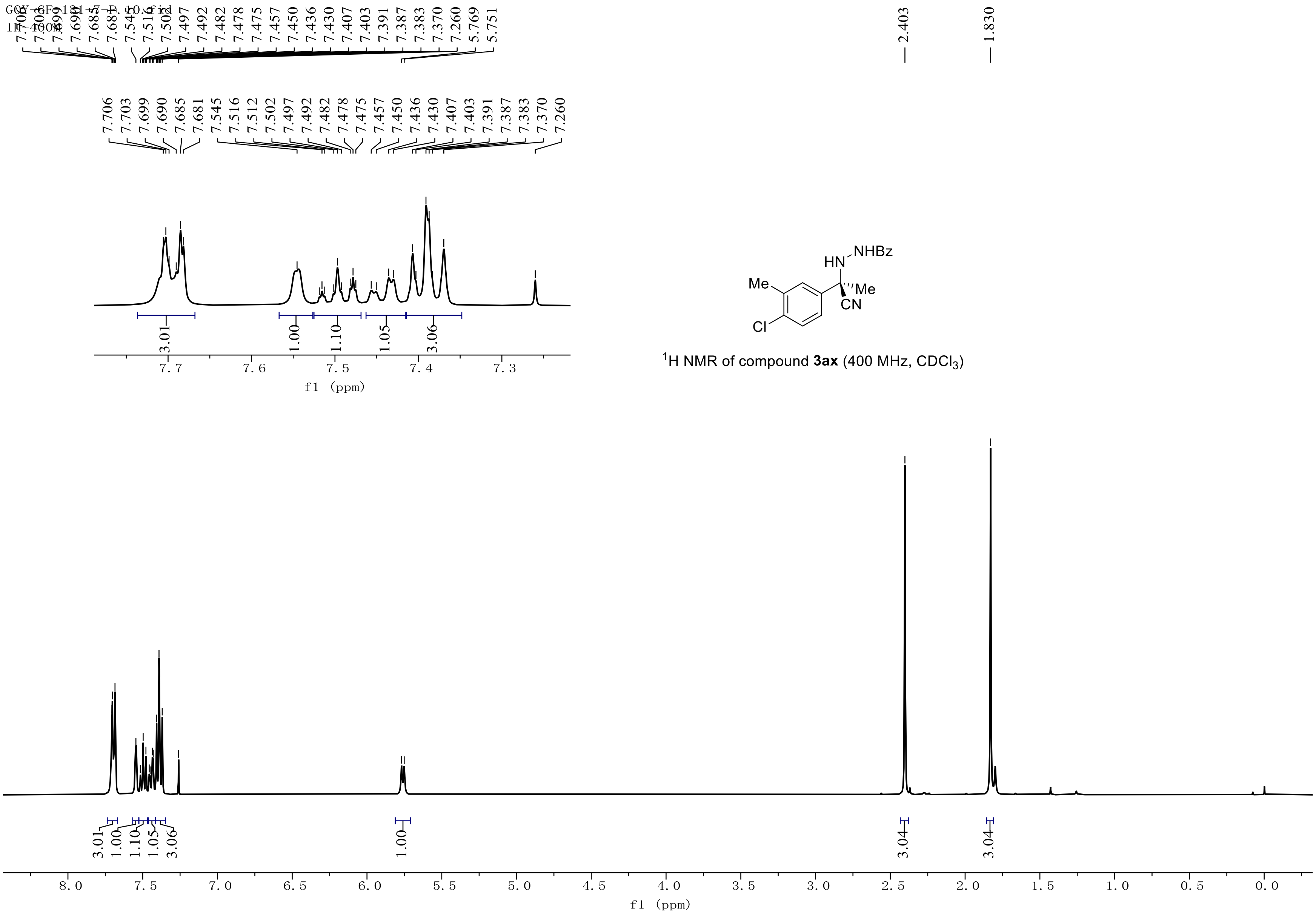

^{1}H NMR of compound **3ax** (400 MHz, $CDCl_3$)

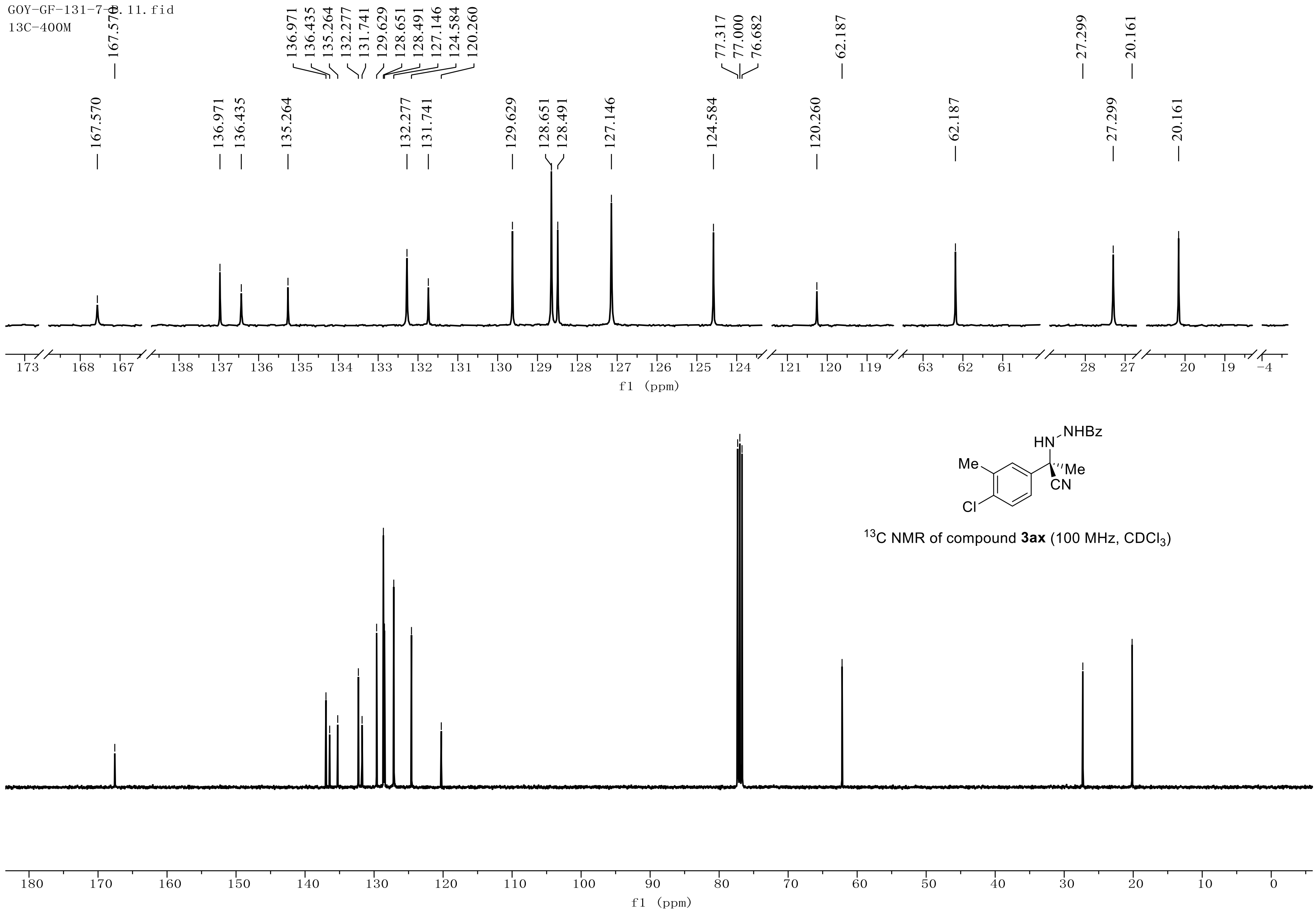

^{13}C NMR of compound **3ax** (100 MHz, $CDCl_3$)

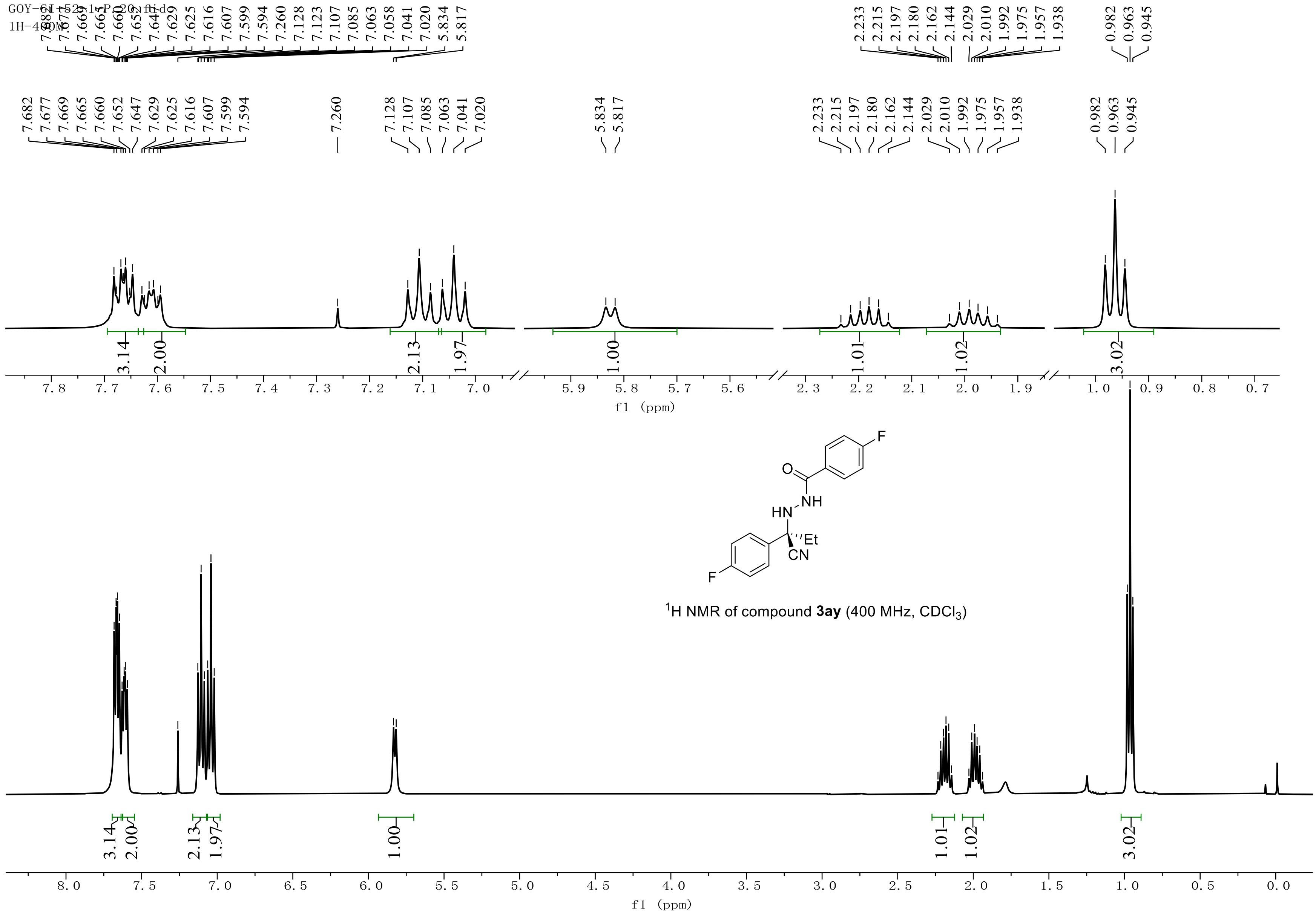

^{1}H NMR of compound **3ay** (400 MHz, $CDCl_3$)

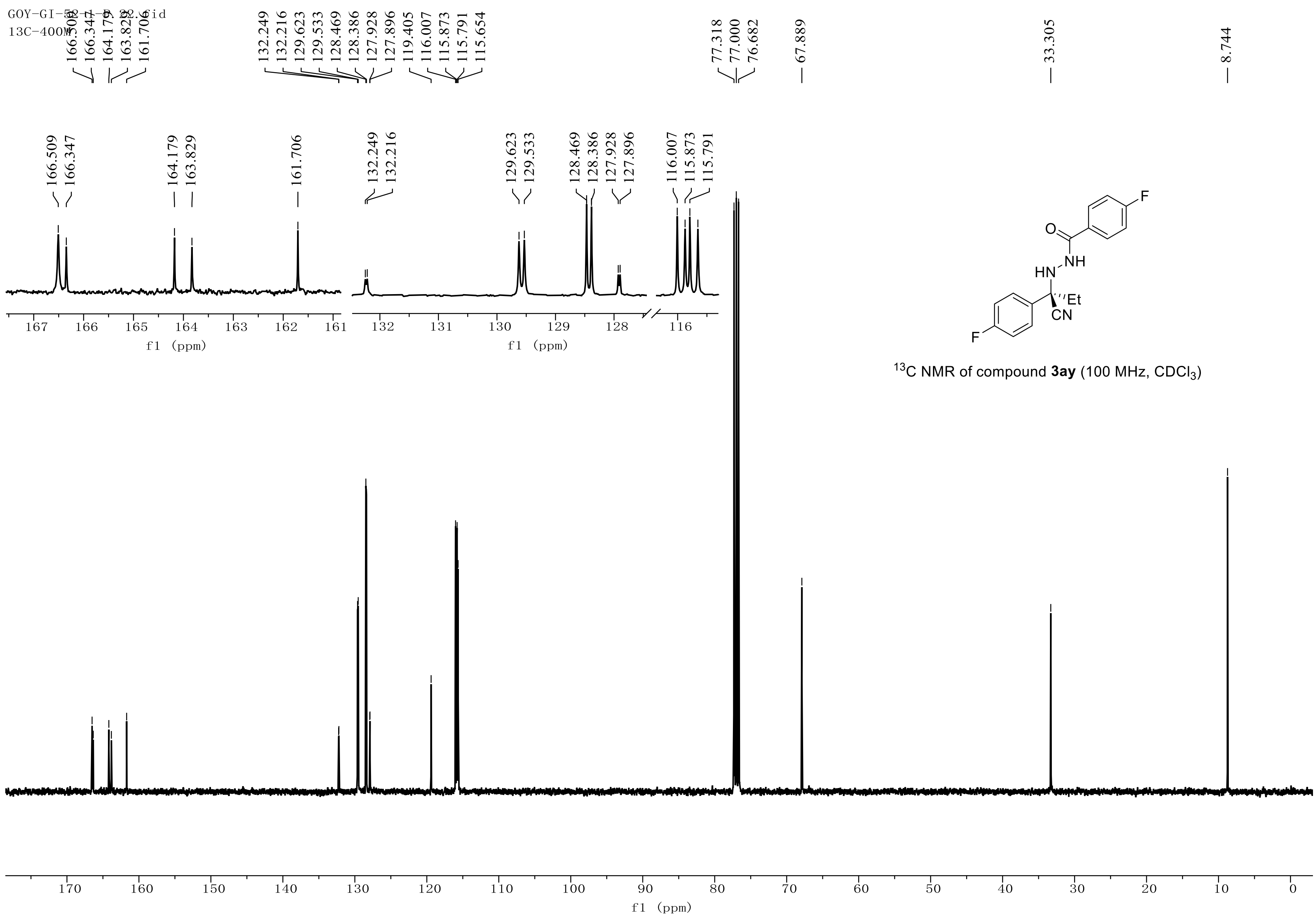

^{13}C NMR of compound **3ay** (100 MHz, $CDCl_3$)

GOY-GI-52-1-P.21.fid
19F-400M

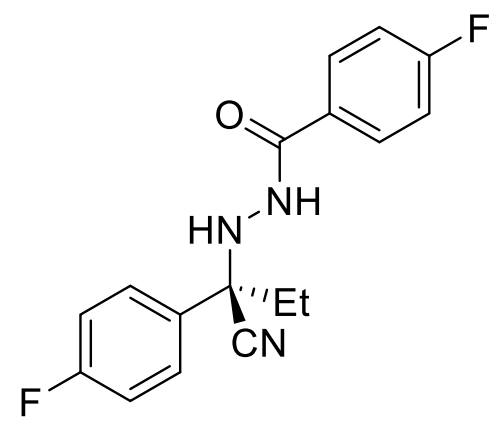

^{19}F NMR of compound **3ay** (376 MHz, $CDCl_3$)

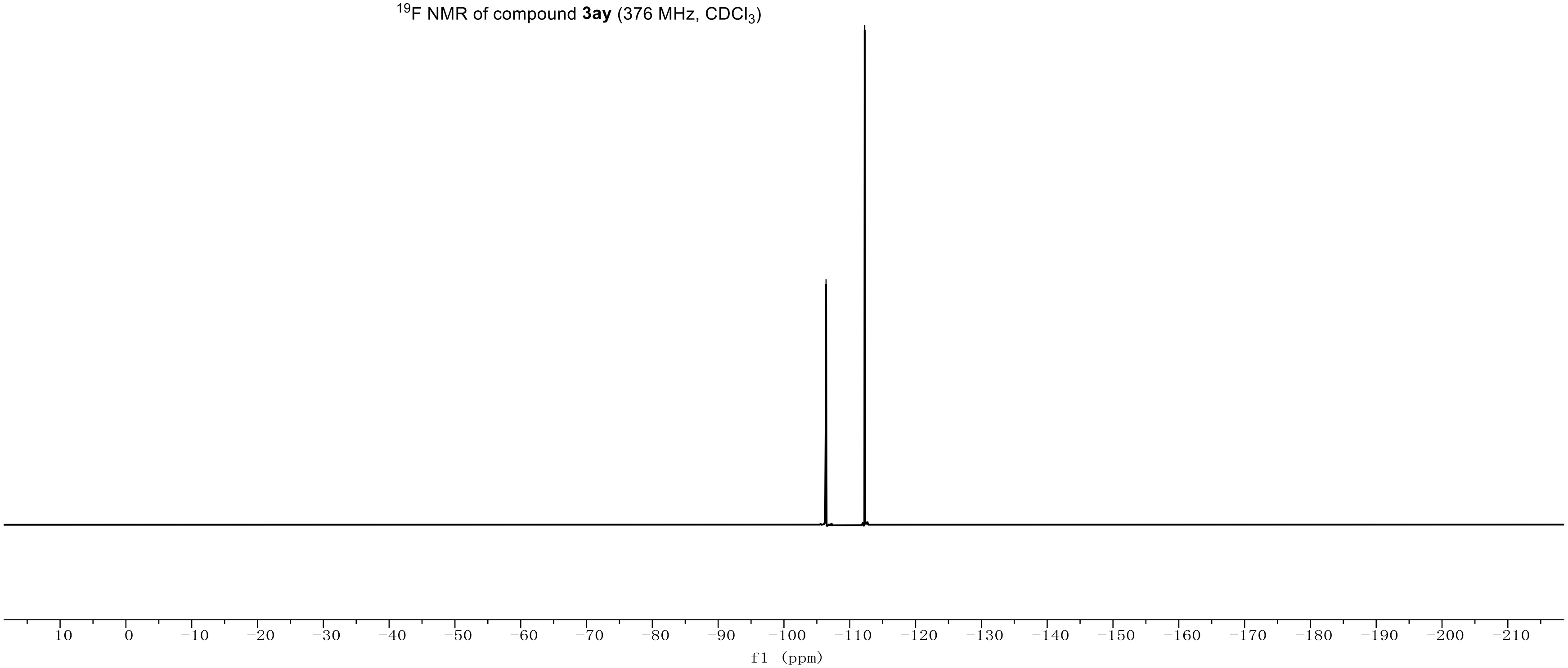

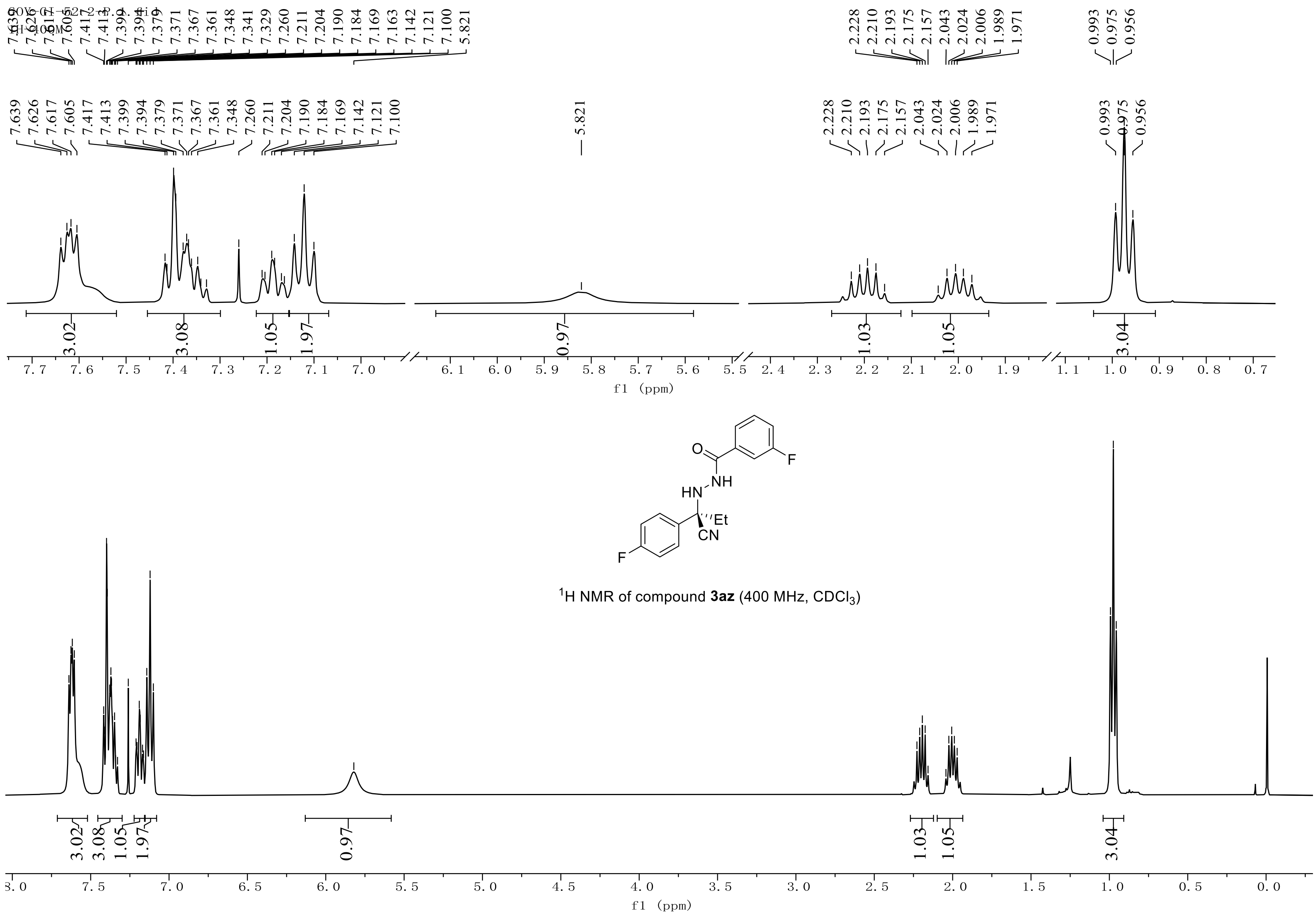

^{1}H NMR of compound **3az** (400 MHz, $CDCl_3$)

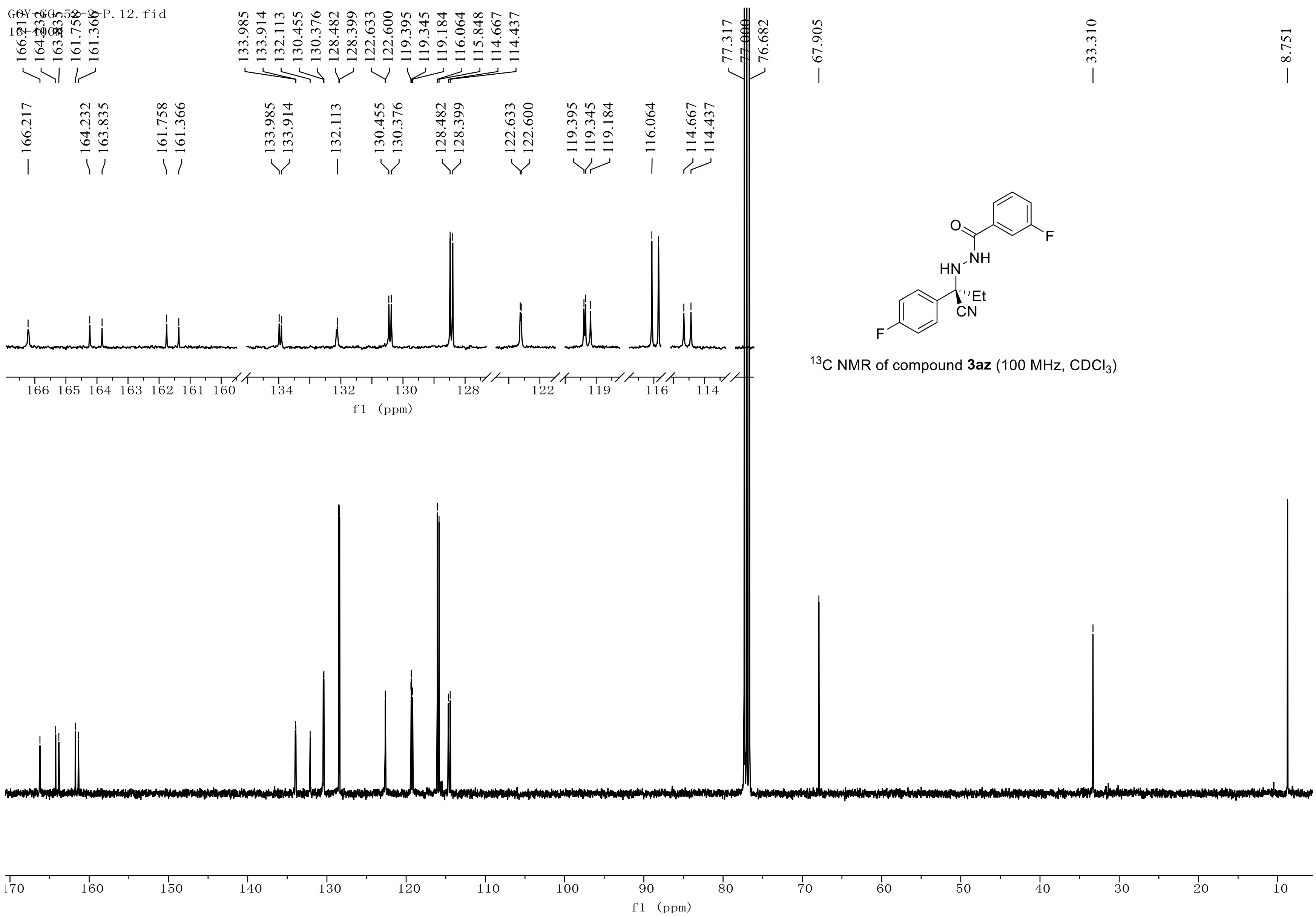

^{13}C NMR of compound **3az** (100 MHz, $CDCl_3$)

GOY-GI-52-2-P.100.fid
19F-400M

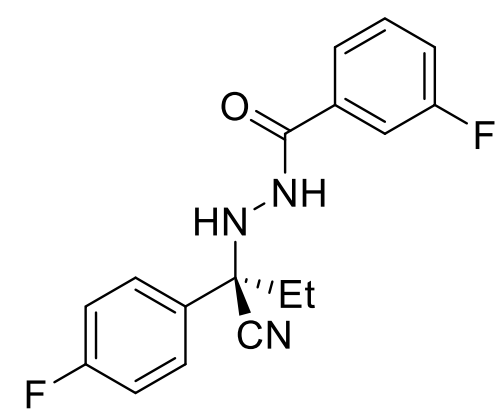

^{19}F NMR of compound **3az** (376 MHz, $CDCl_3$)

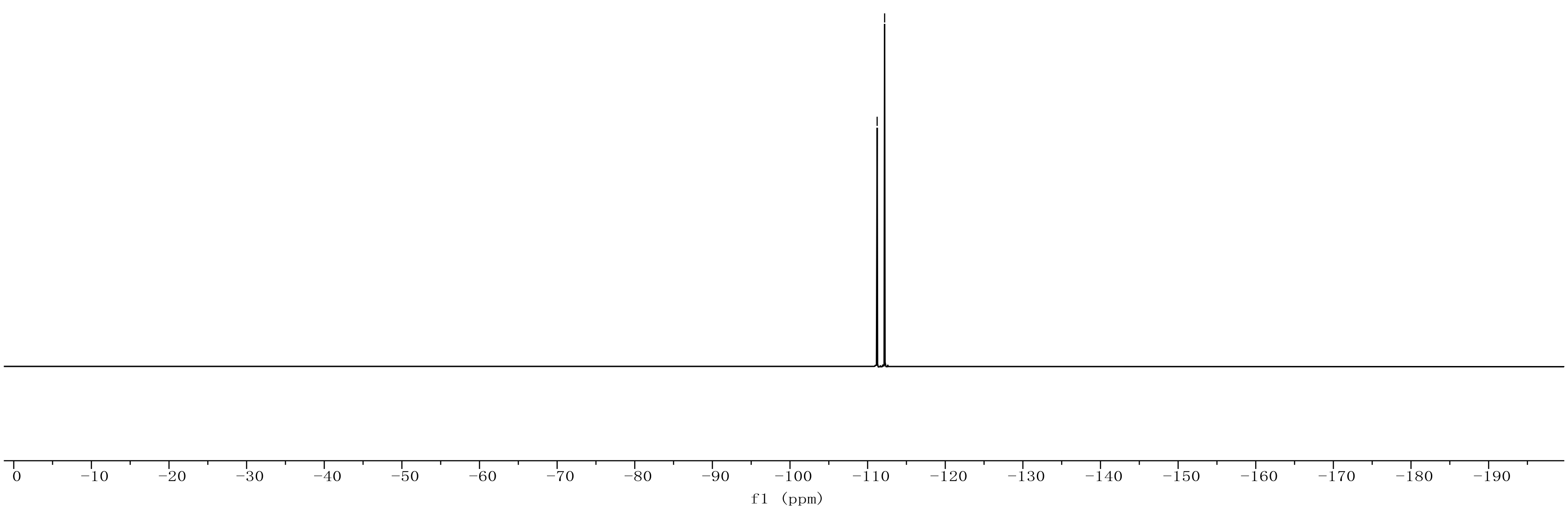

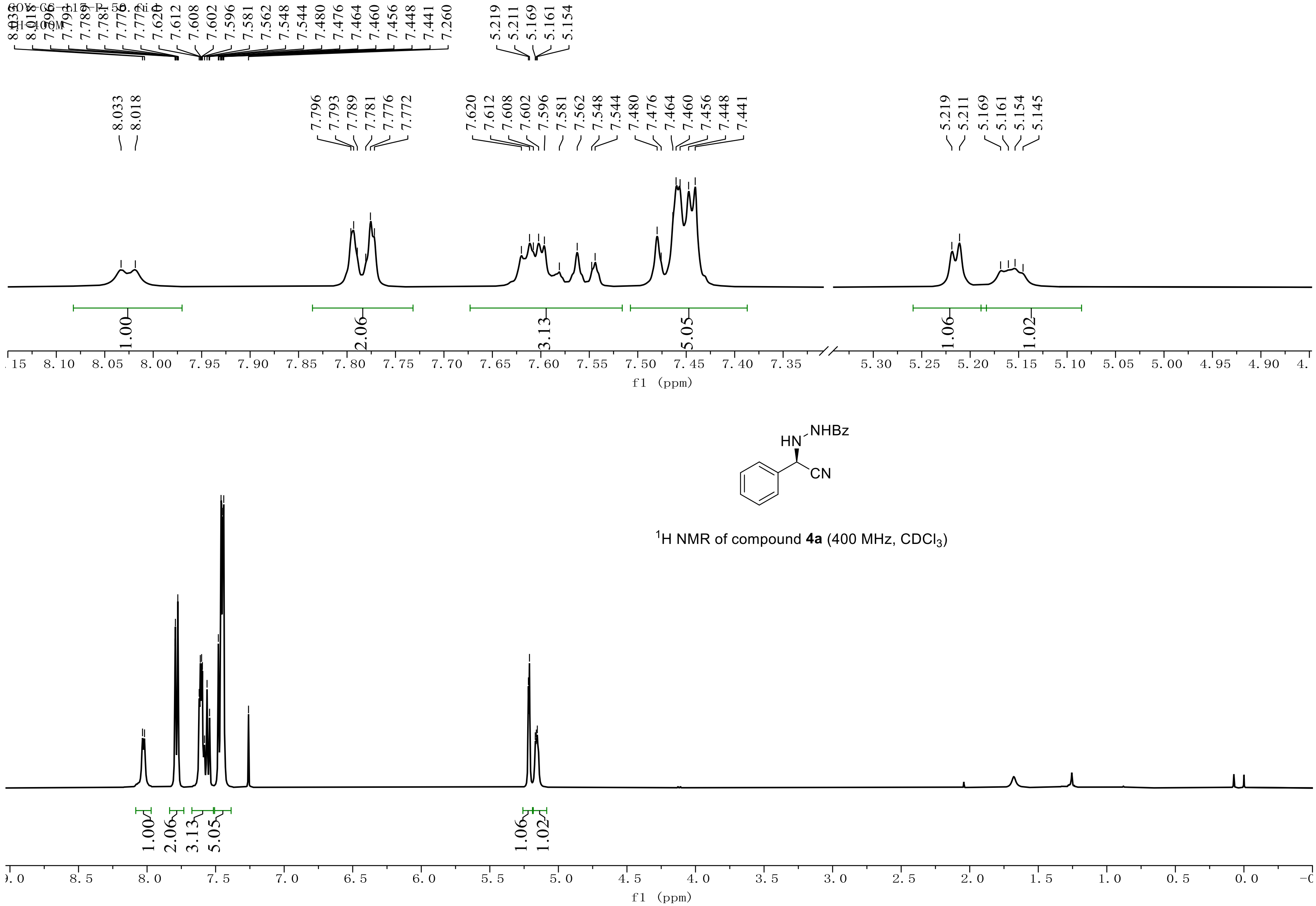

^{1}H NMR of compound **4a** (400 MHz, $CDCl_3$)

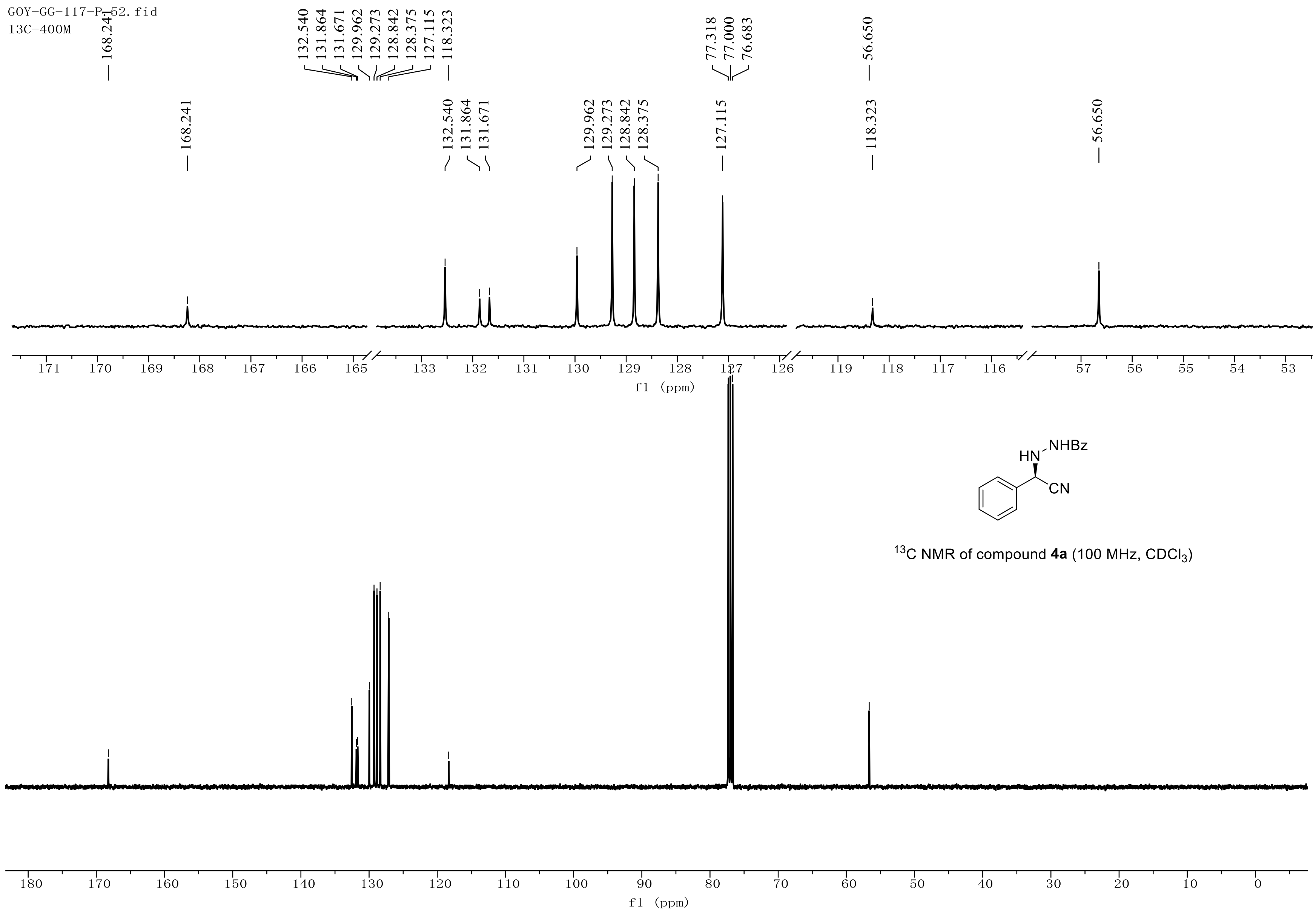

^{13}C NMR of compound **4a** (100 MHz, $CDCl_3$)

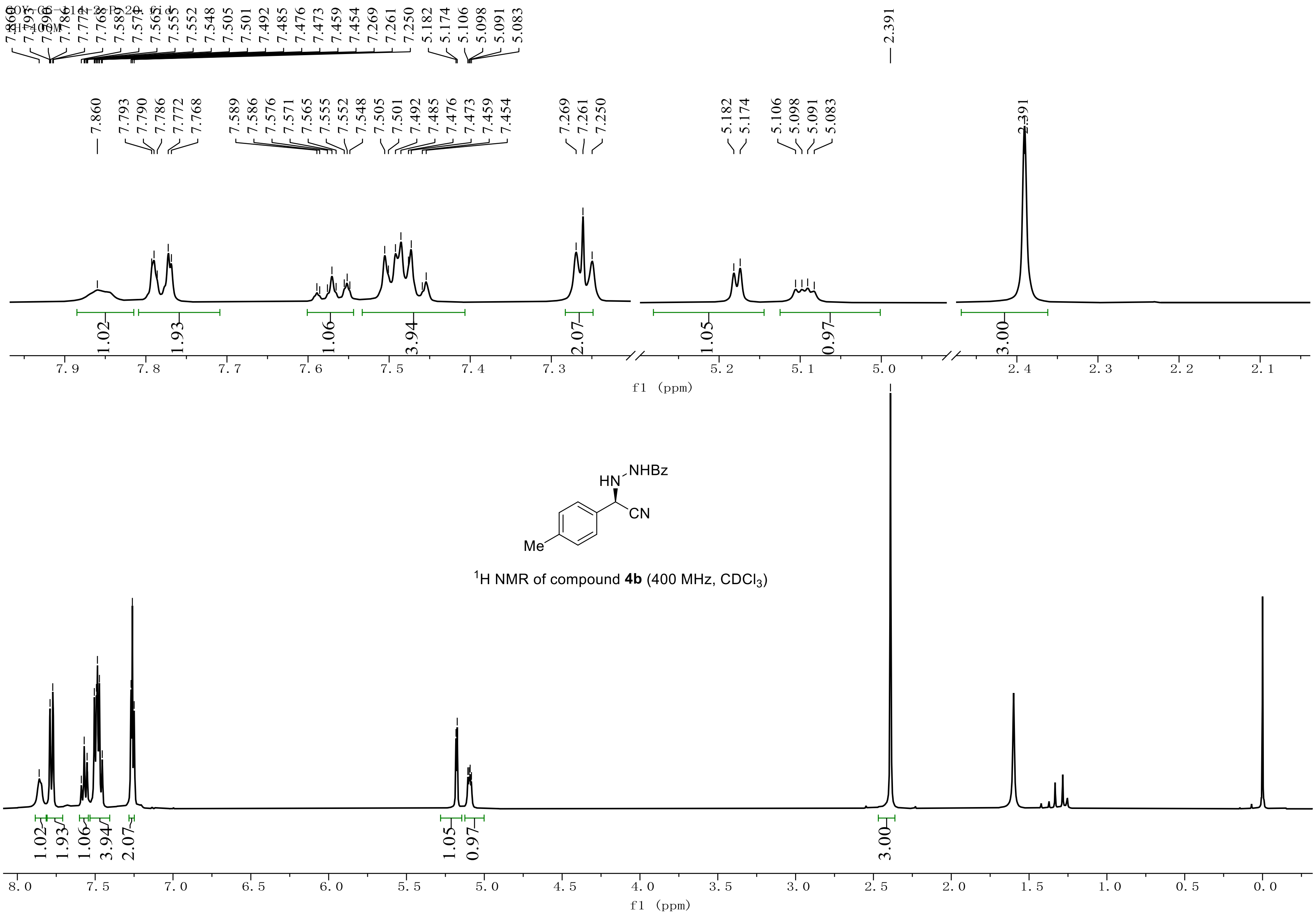

^{1}H NMR of compound **4b** (400 MHz, $CDCl_3$)

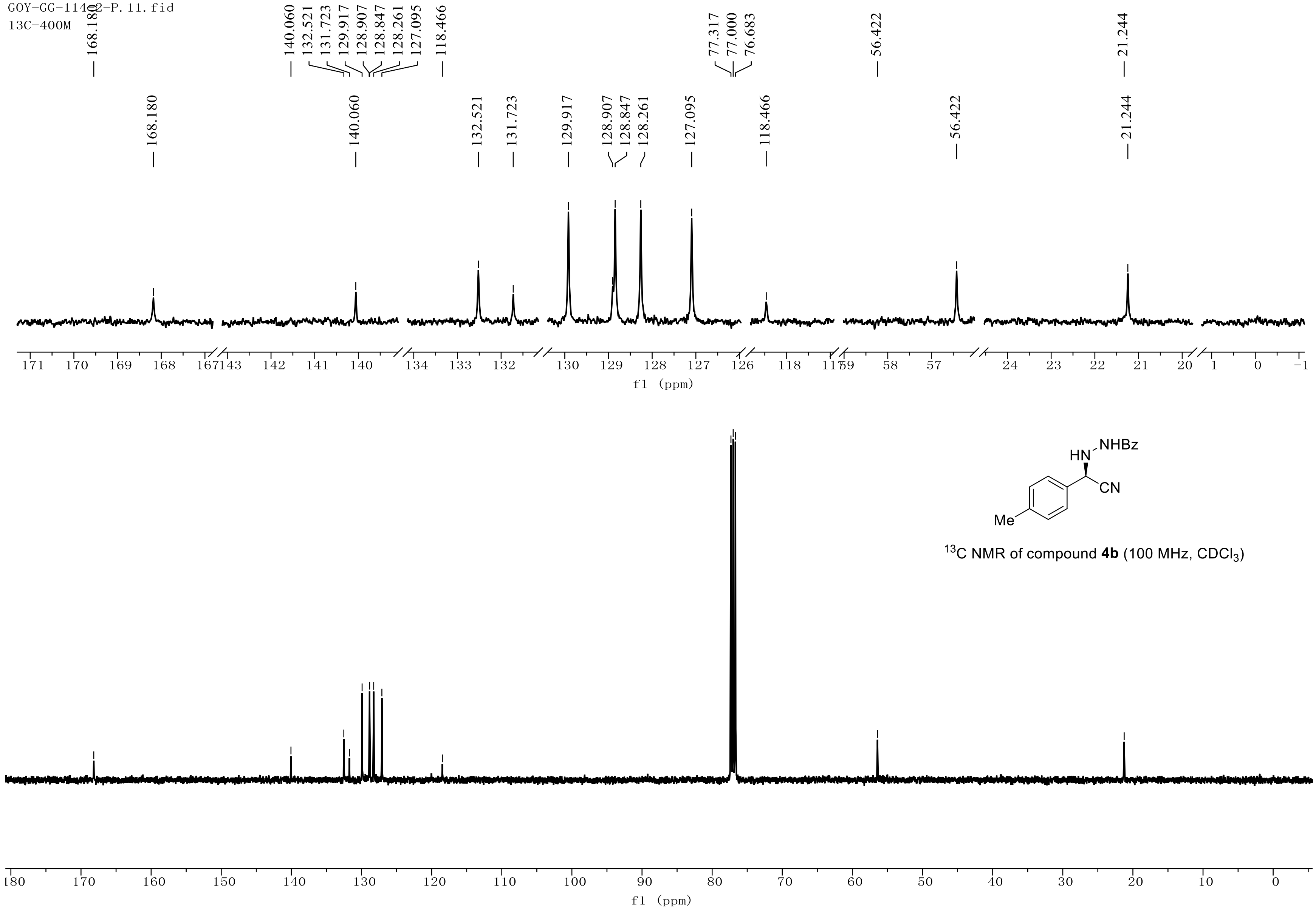

^{13}C NMR of compound **4b** (100 MHz, $CDCl_3$)

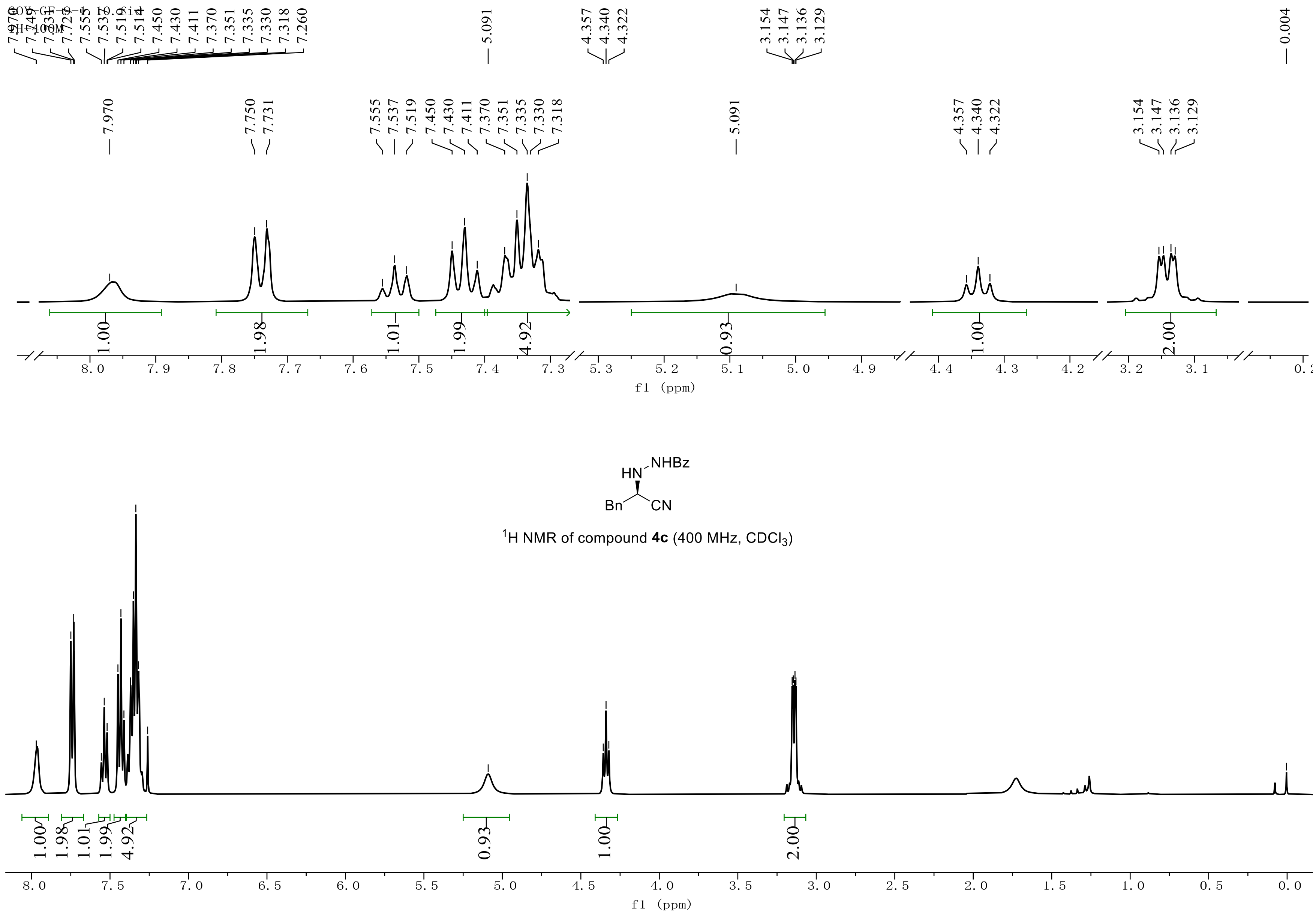

^{1}H NMR of compound **4c** (400 MHz, $CDCl_3$)

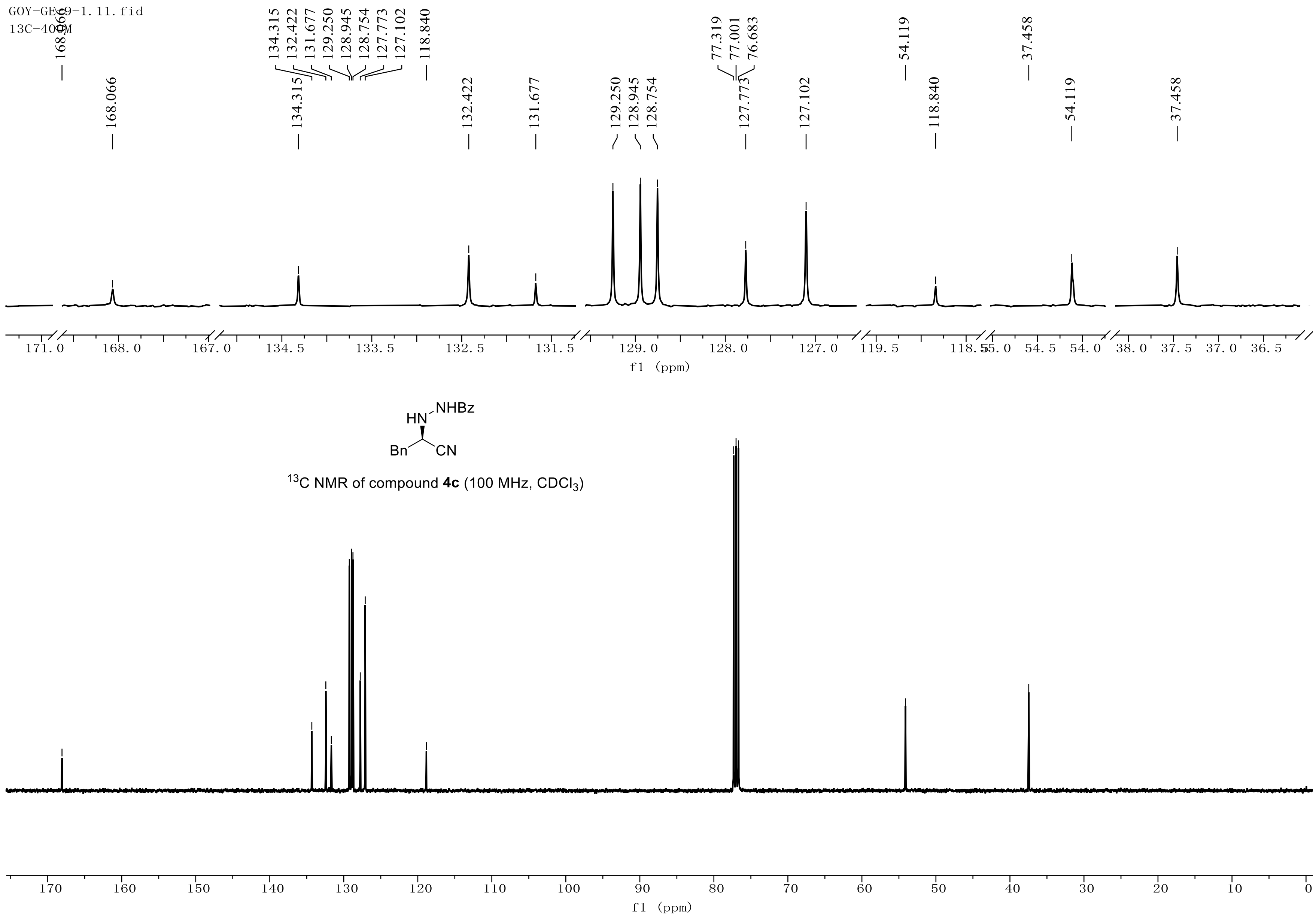

^{13}C NMR of compound **4c** (100 MHz, $CDCl_3$)

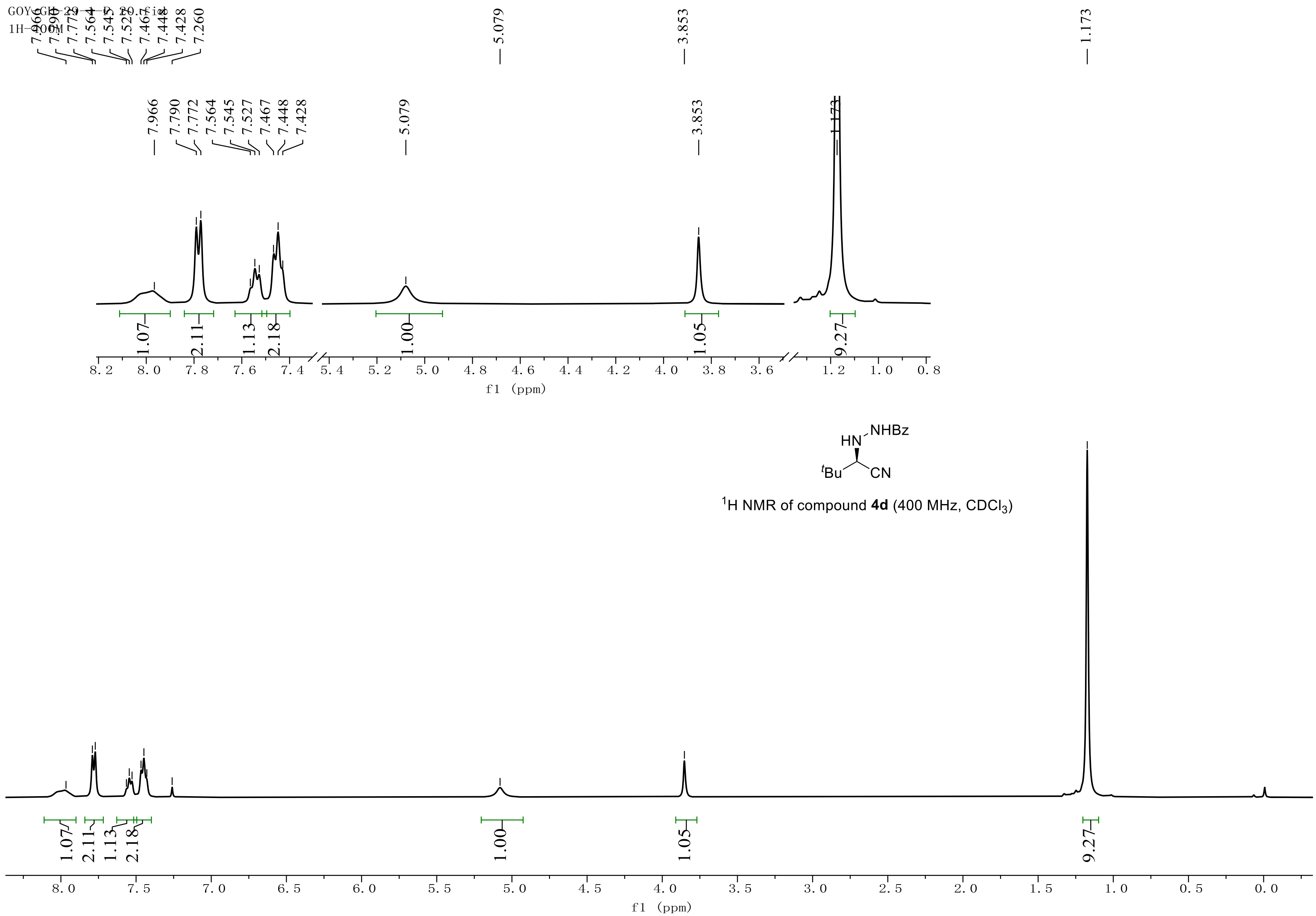

^{1}H NMR of compound **4d** (400 MHz, $CDCl_3$)

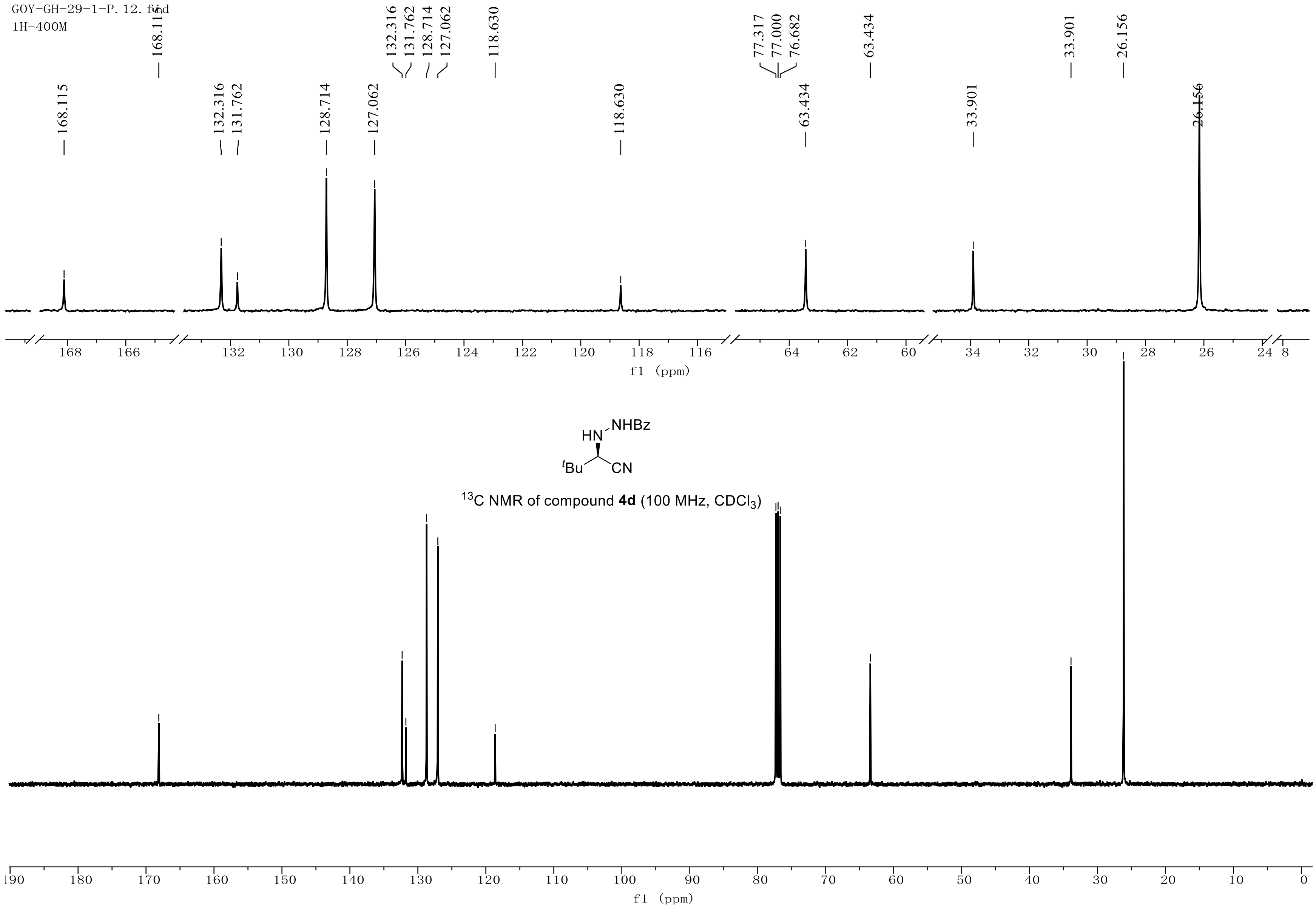

^{13}C NMR of compound **4d** (100 MHz, $CDCl_3$)

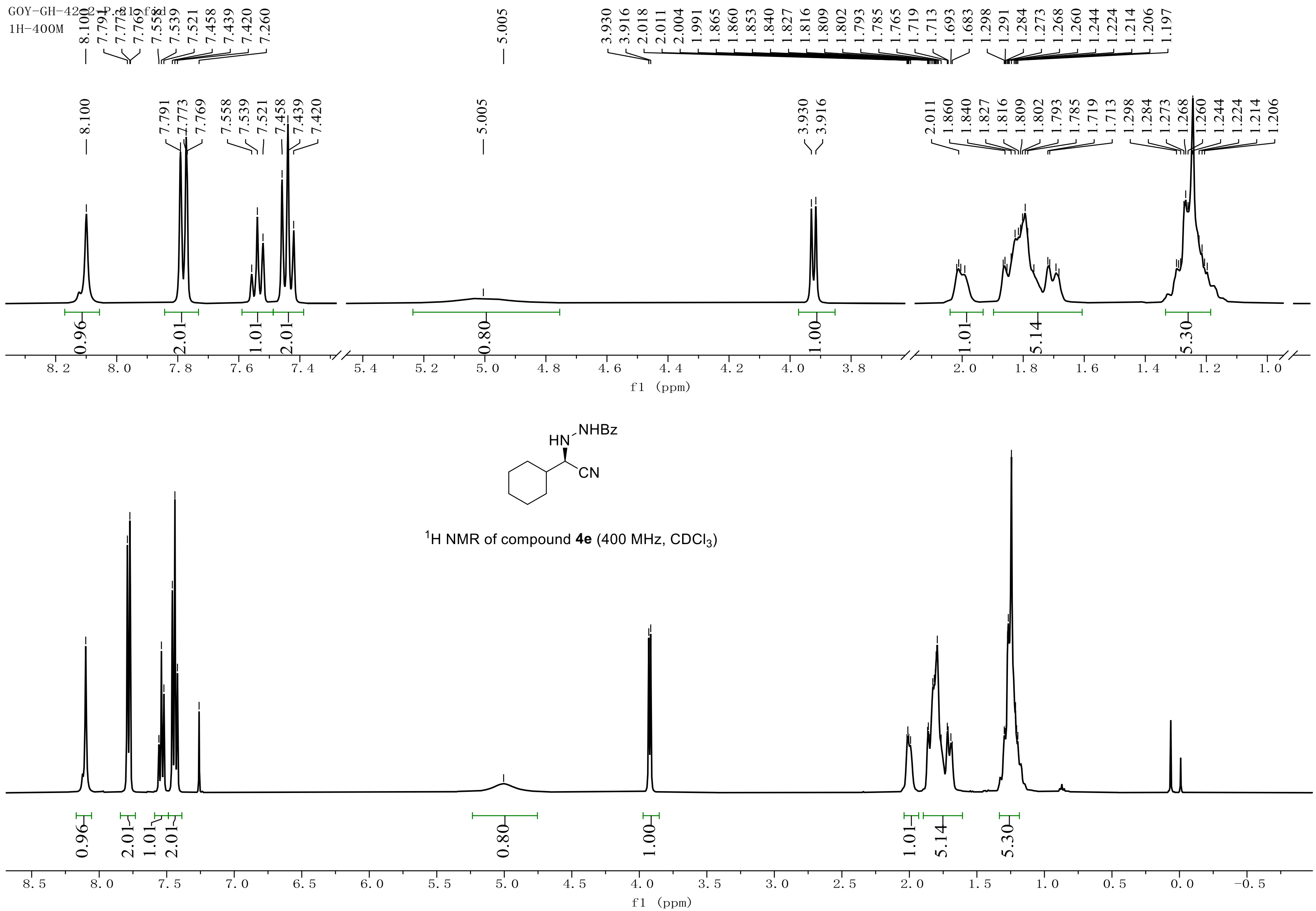

^{1}H NMR of compound **4e** (400 MHz, $CDCl_3$)

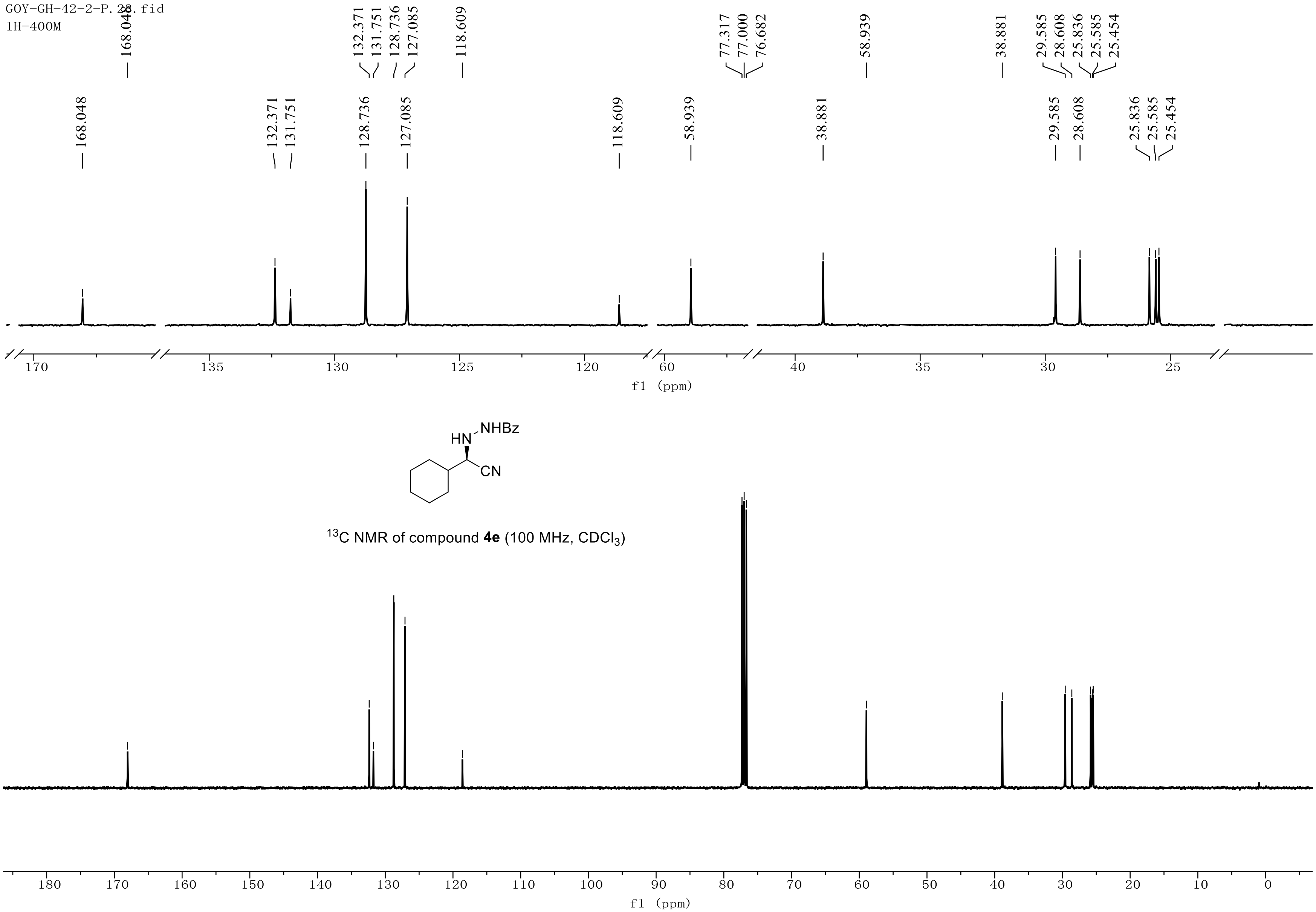

^{13}C NMR of compound **4e** (100 MHz, $CDCl_3$)

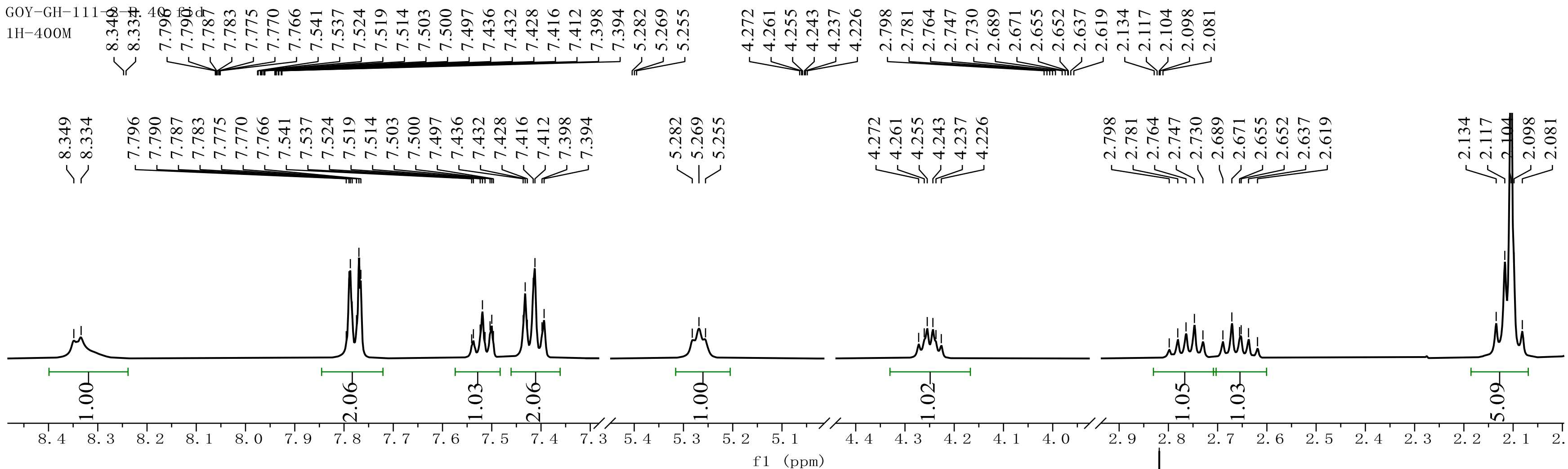

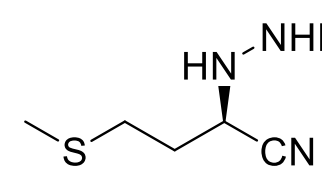

^{1}H NMR of compound **4f** (400 MHz, $CDCl_3$)

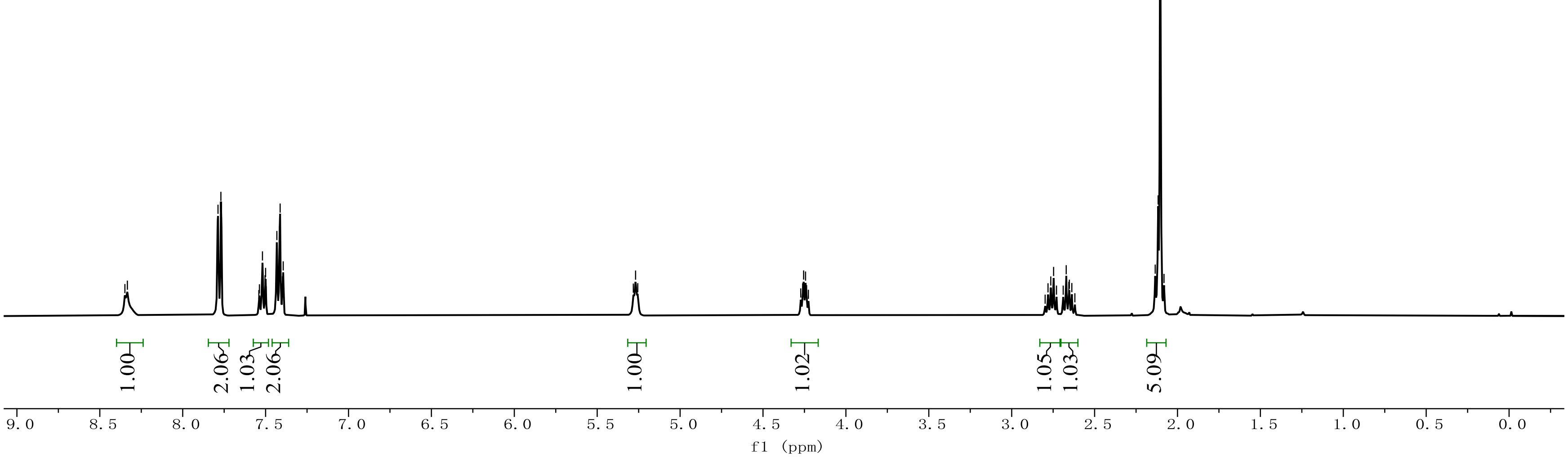

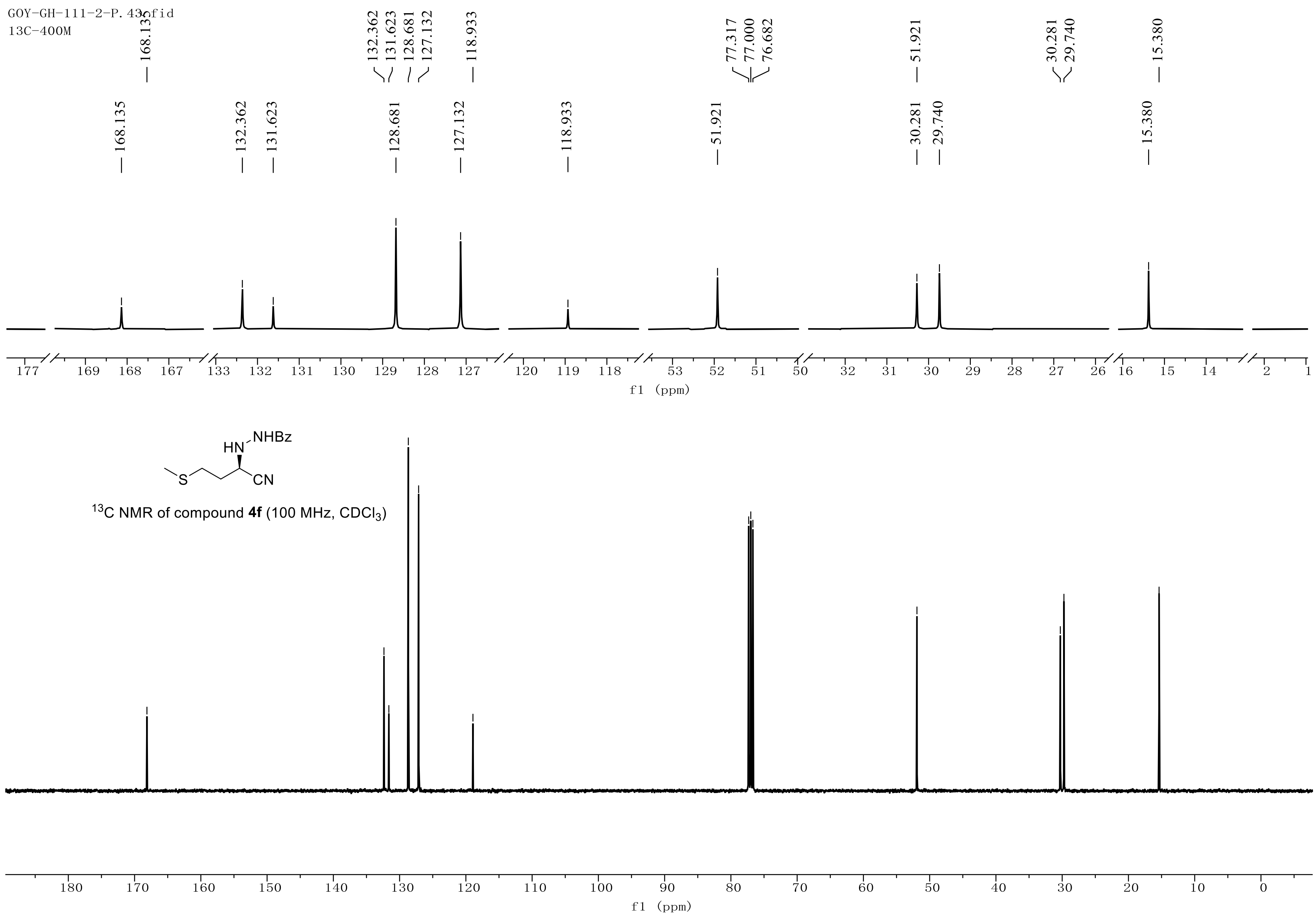

^{13}C NMR of compound **4f** (100 MHz, $CDCl_3$)

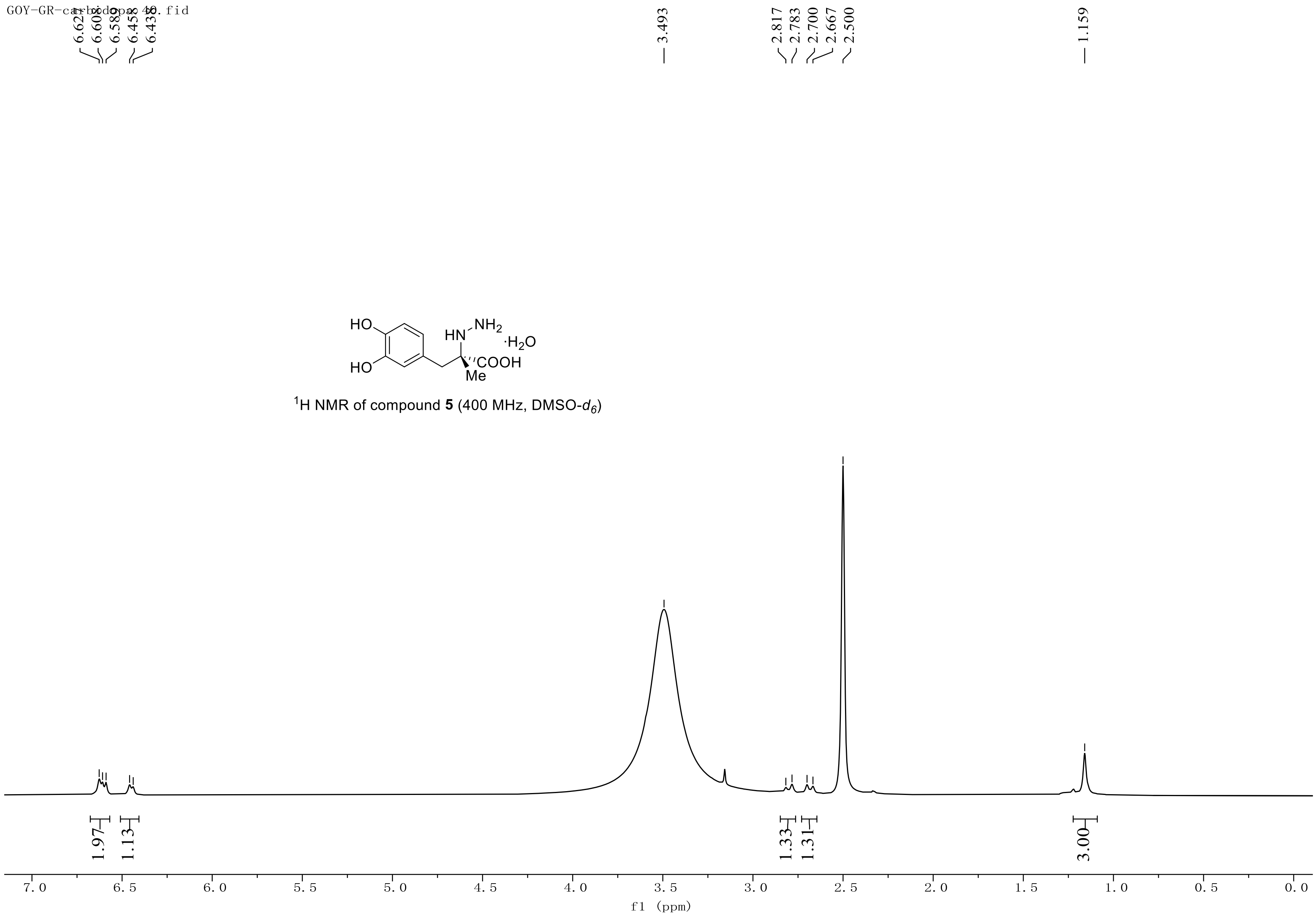

^{1}H NMR of compound **5** (400 MHz, DMSO-d_6)

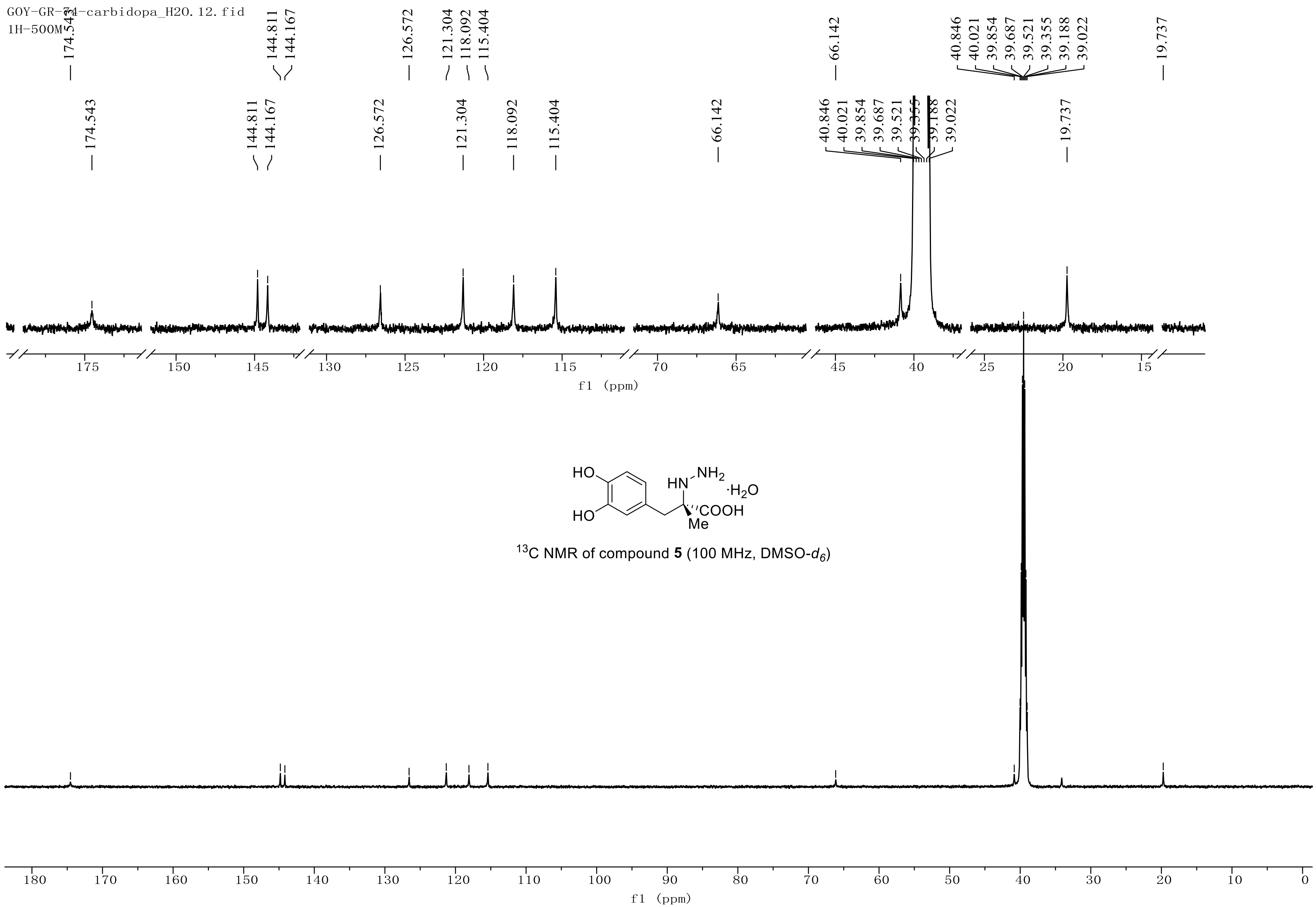

^{13}C NMR of compound **5** (100 MHz, DMSO-d_6)

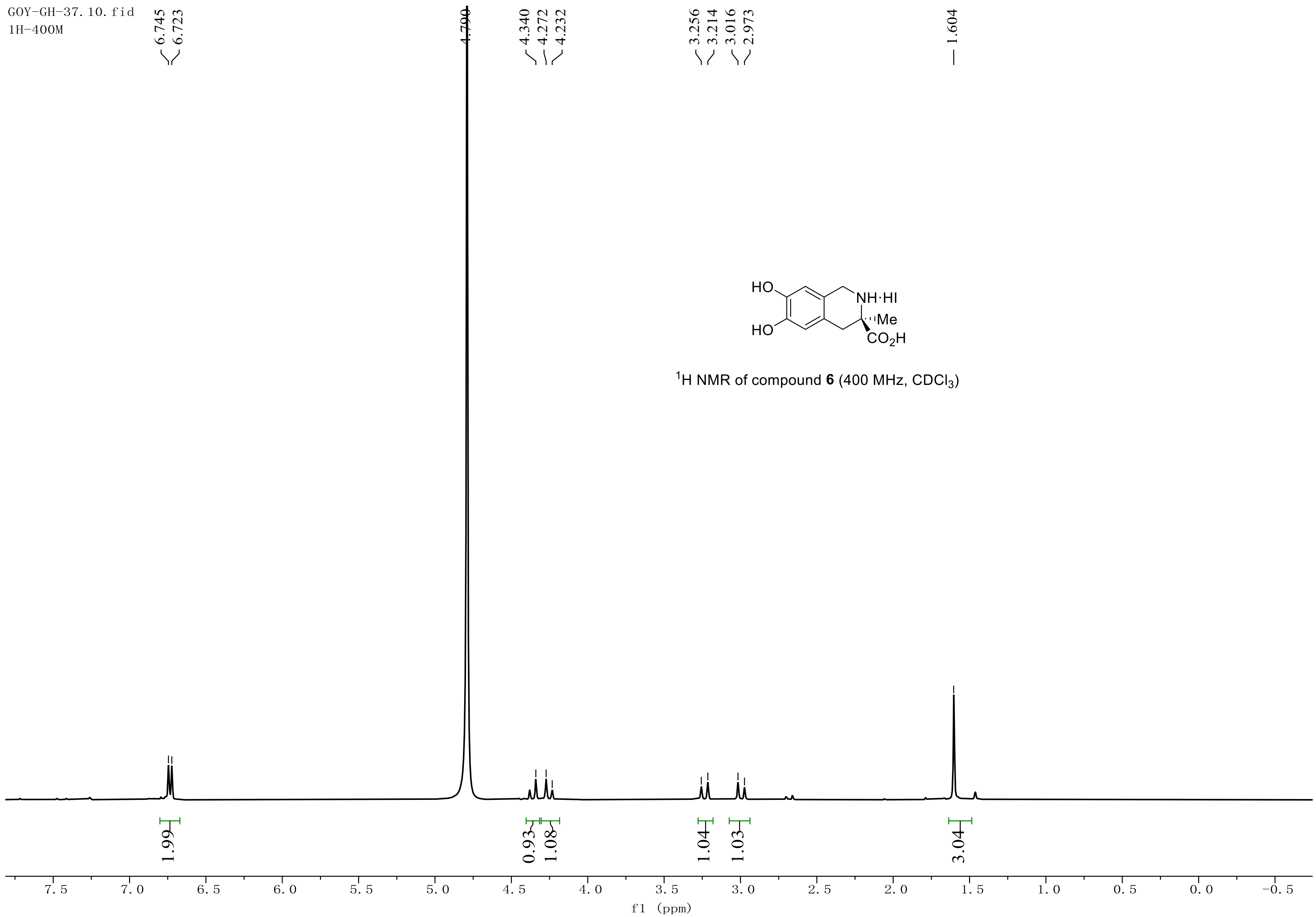

^{1}H NMR of compound **6** (400 MHz, $CDCl_3$)

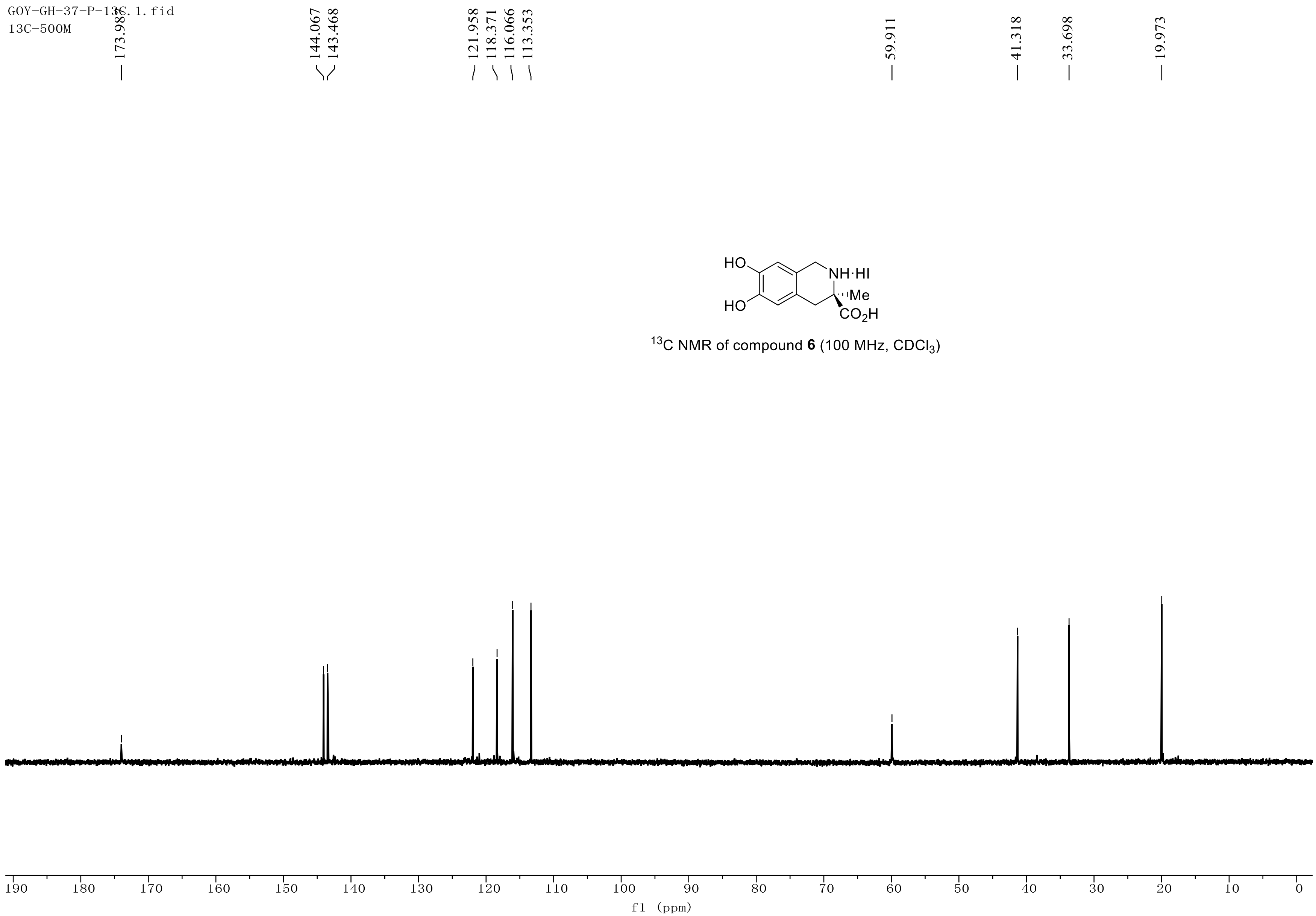

^{13}C NMR of compound **6** (100 MHz, $CDCl_3$)

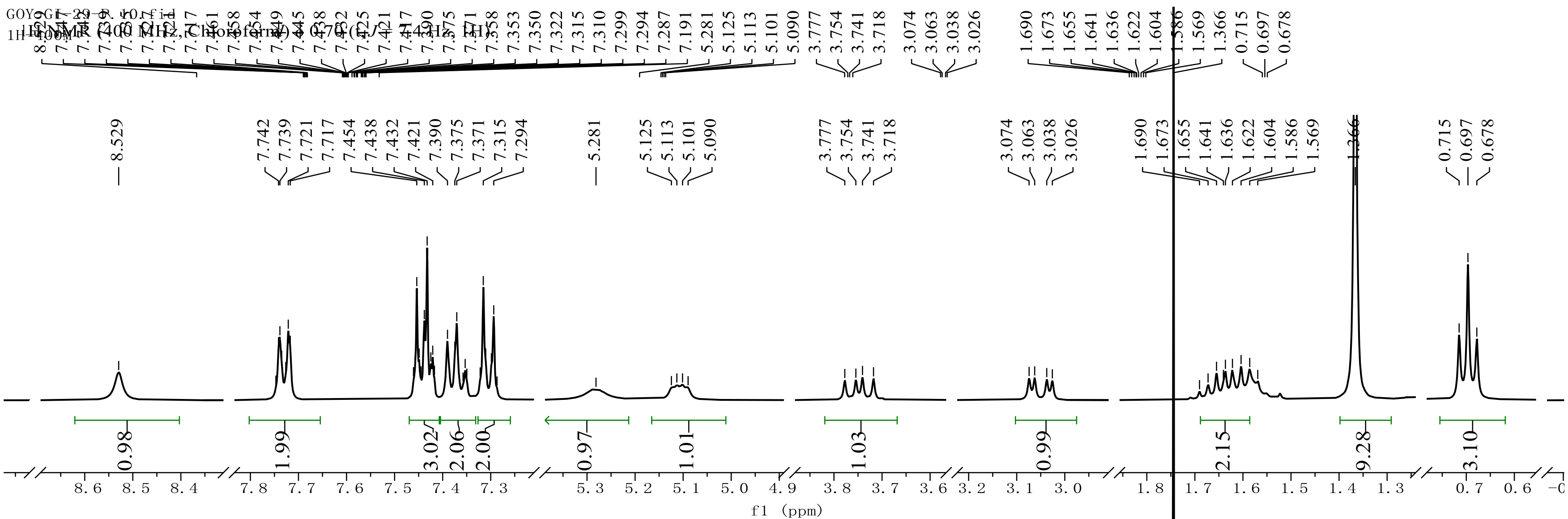

^{1}H NMR of compound **7** (400 MHz, $CDCl_3$)

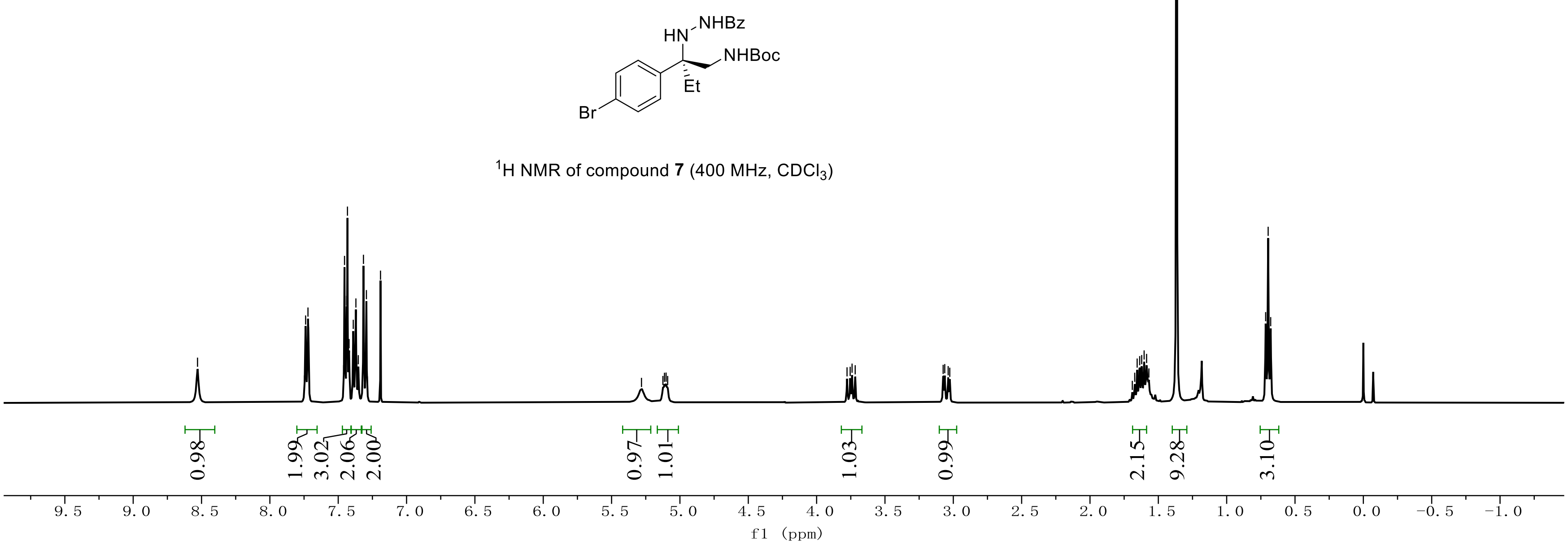

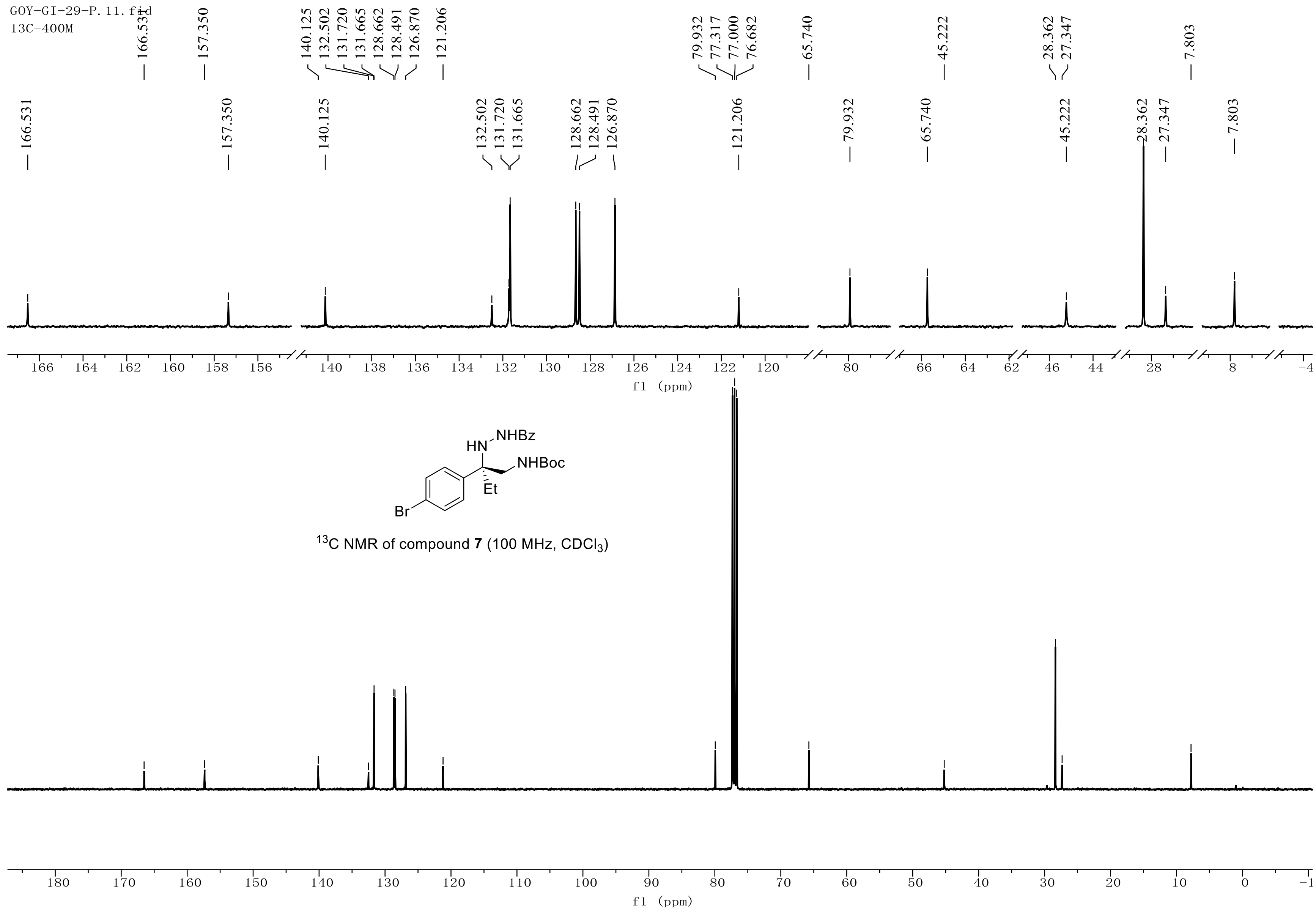

^{13}C NMR of compound **7** (100 MHz, $CDCl_3$)

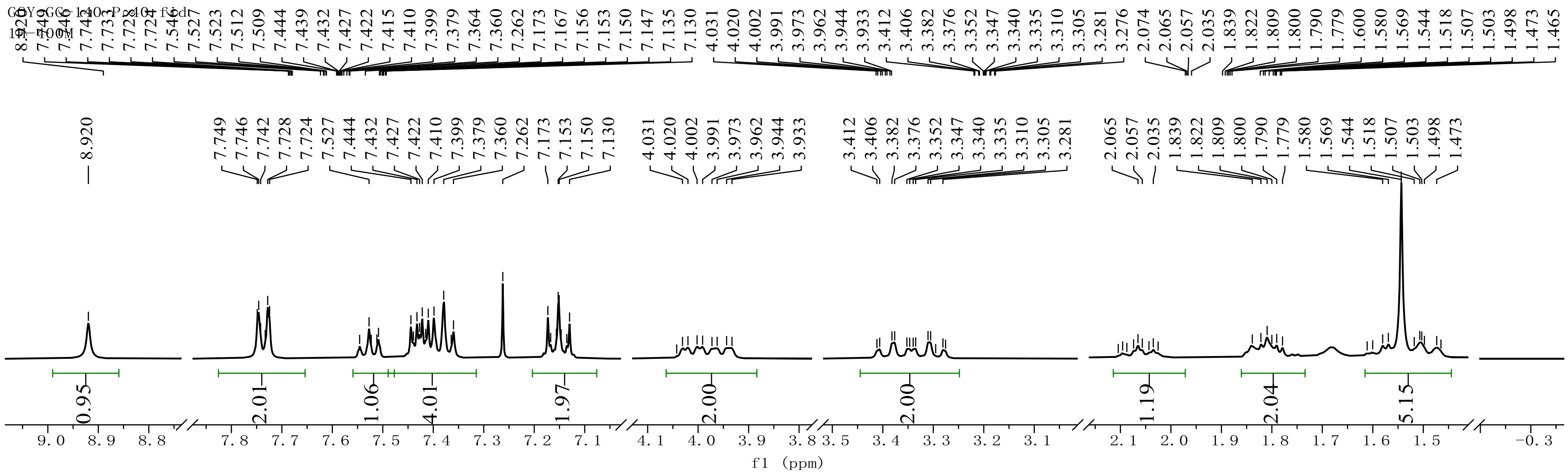

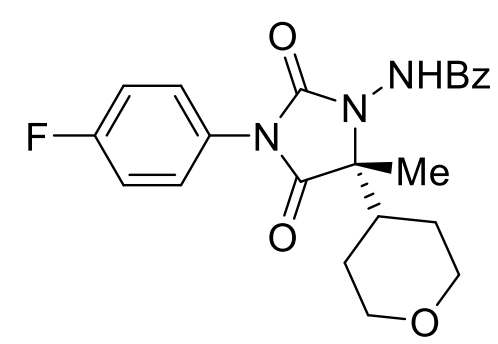

^{1}H NMR of compound **8** (400 MHz, $CDCl_3$)

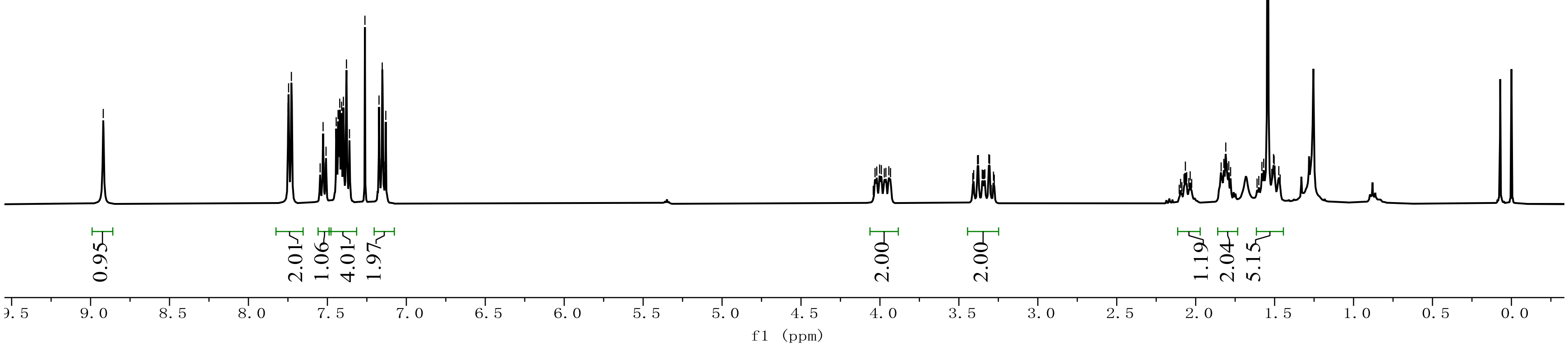

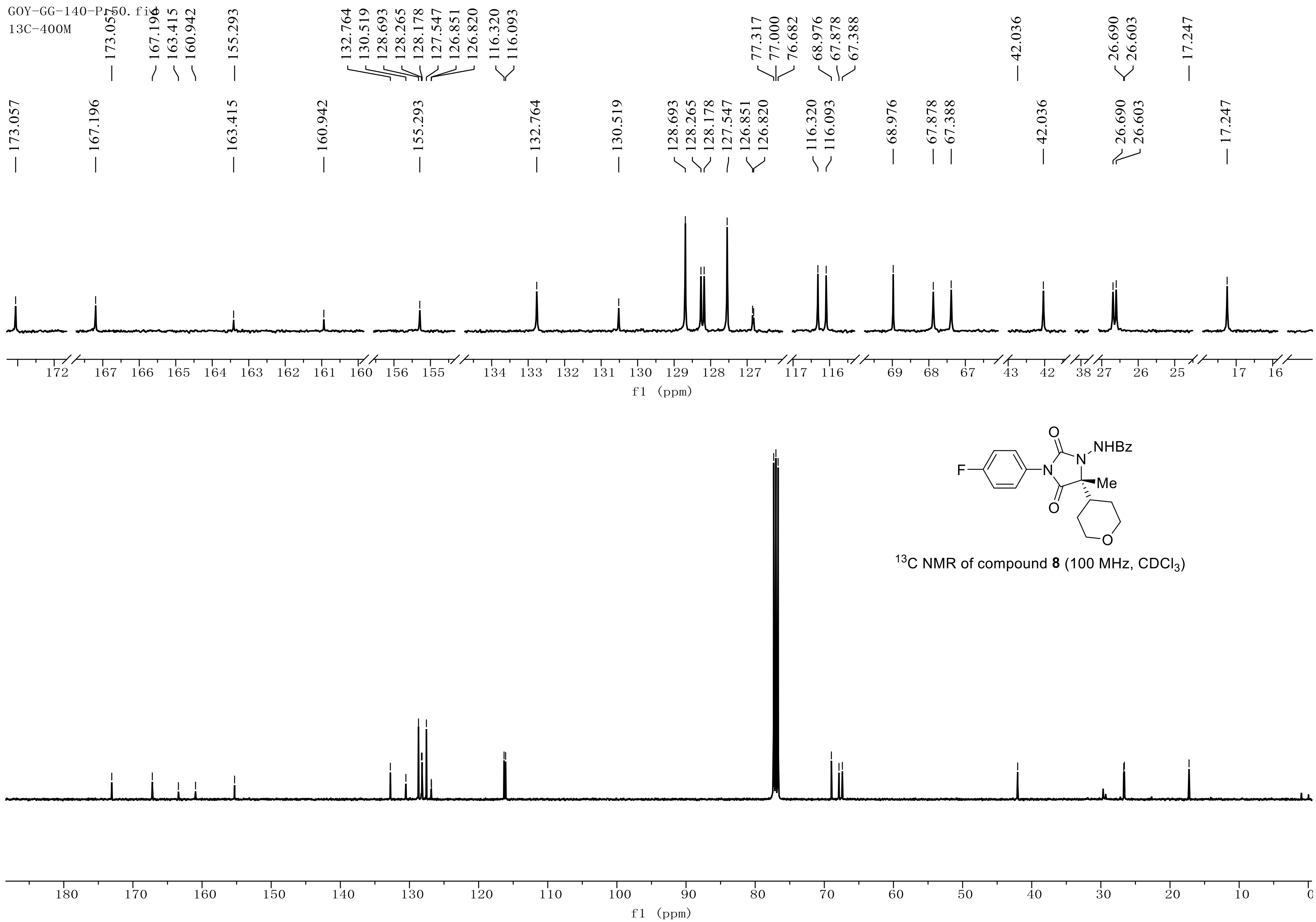

^{13}C NMR of compound **8** (100 MHz, $CDCl_3$)

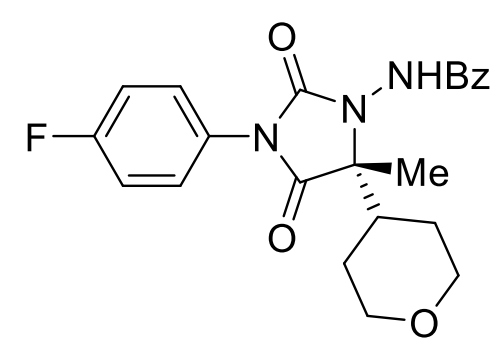

^{19}F NMR of compound **8** (376 MHz, $CDCl_3$)

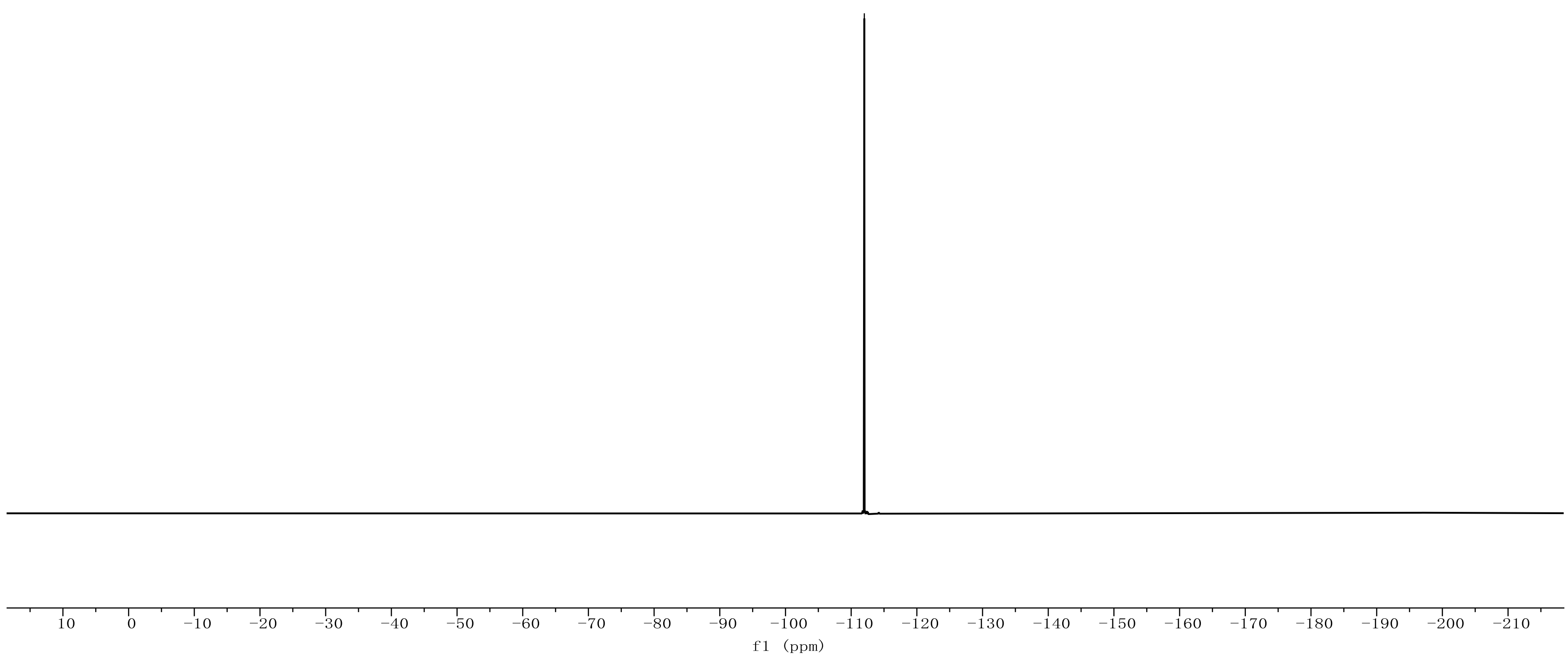

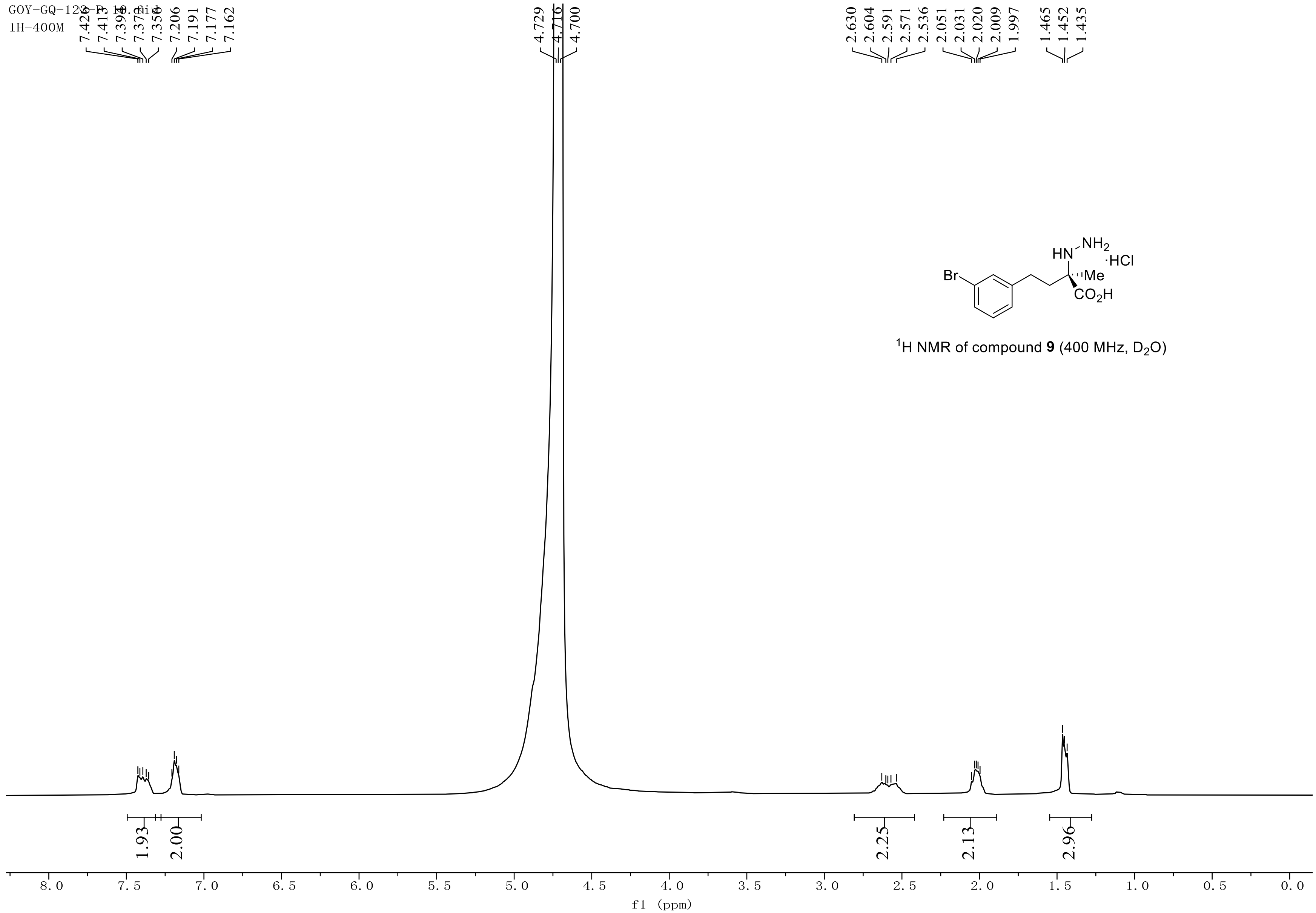

^{1}H NMR of compound **9** (400 MHz, D_2O)

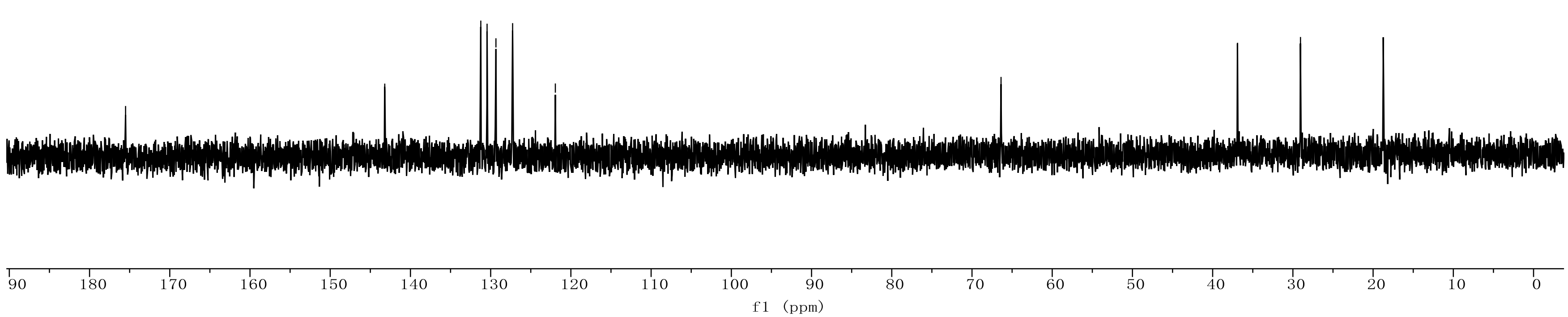

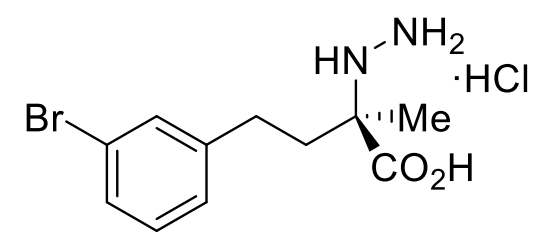

^{13}C NMR of compound **9** (100 MHz, D_2O)

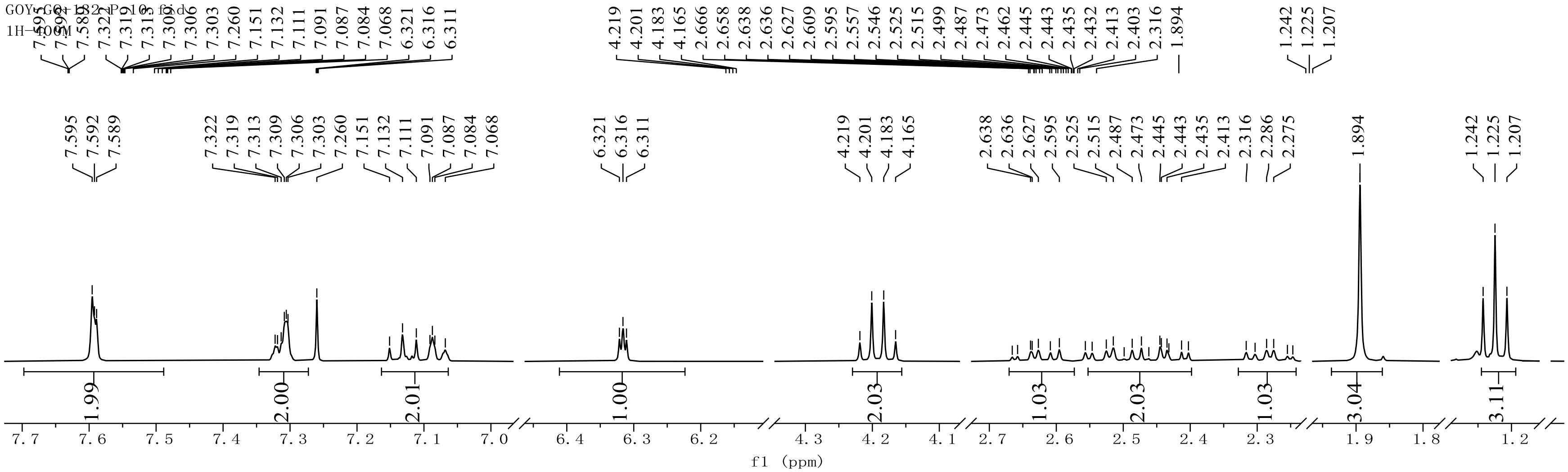

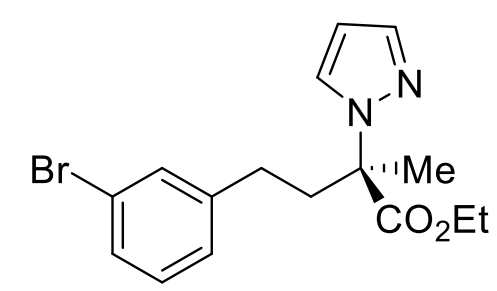

^1H NMR of compound **10** (400 MHz, $CDCl_3$)

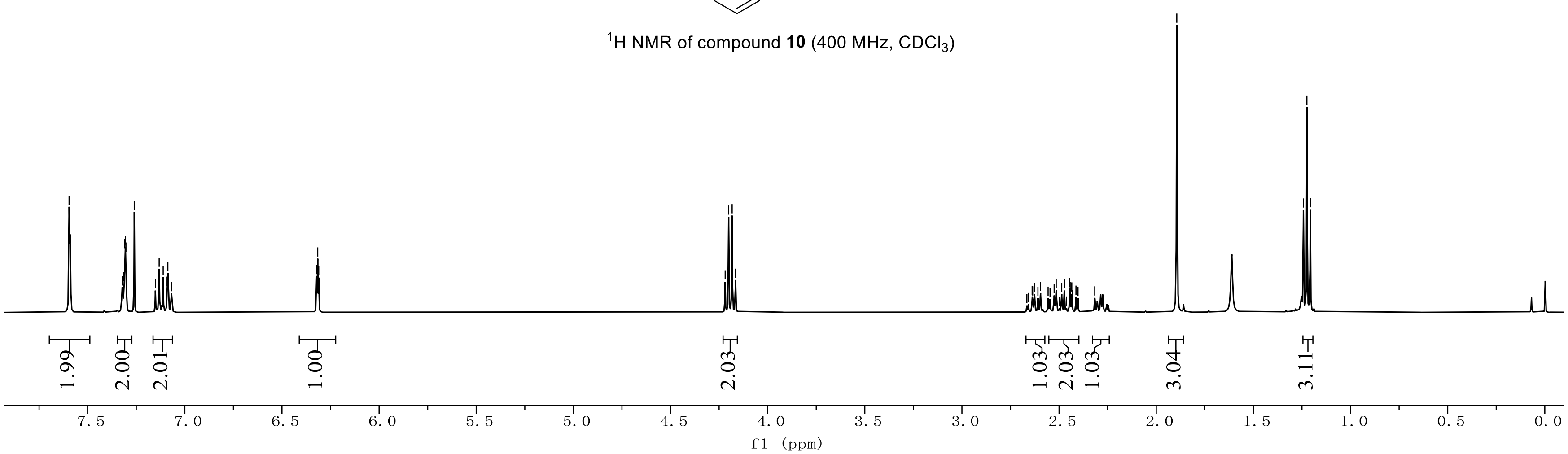

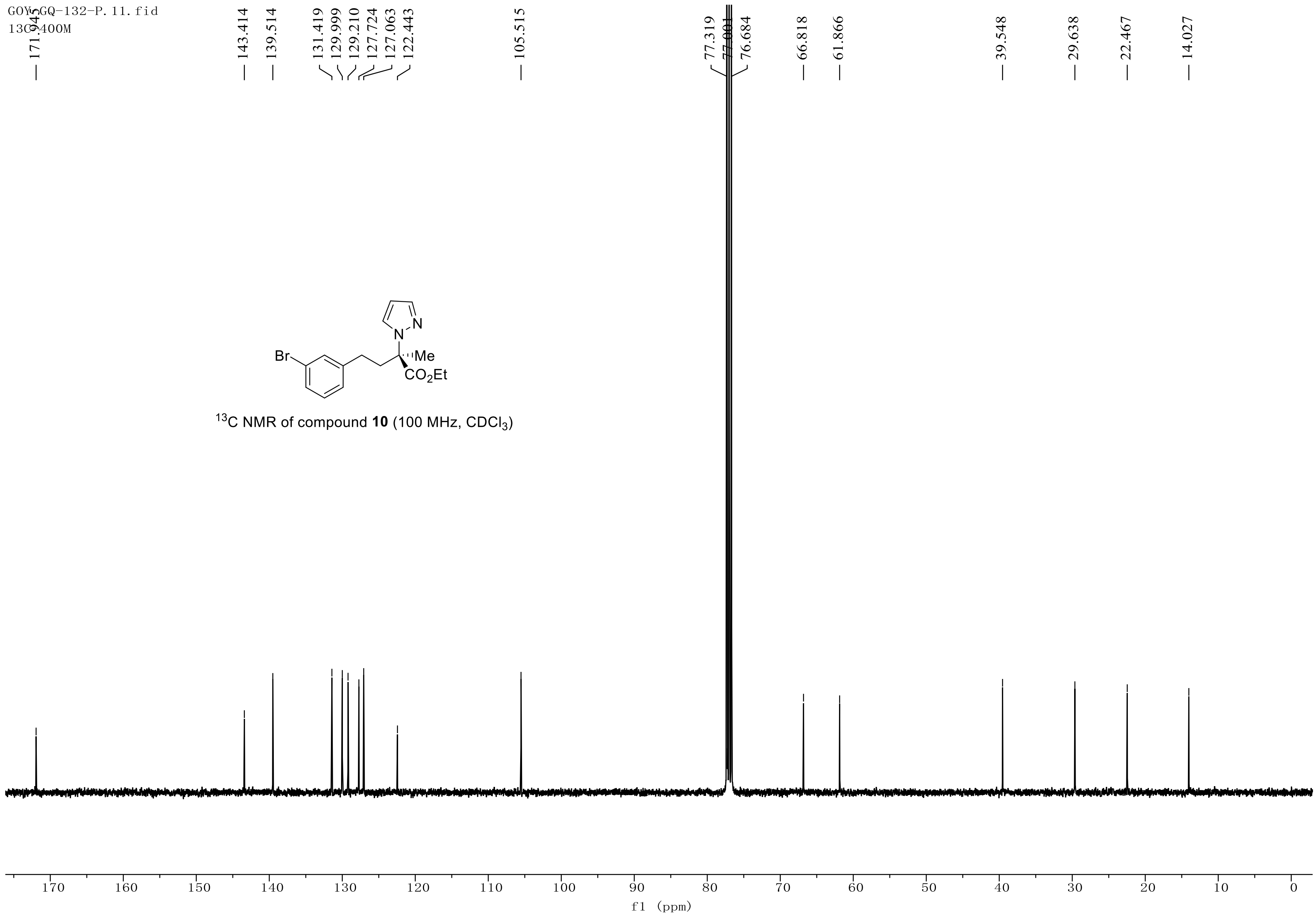

^{13}C NMR of compound **10** (100 MHz, $CDCl_3$)

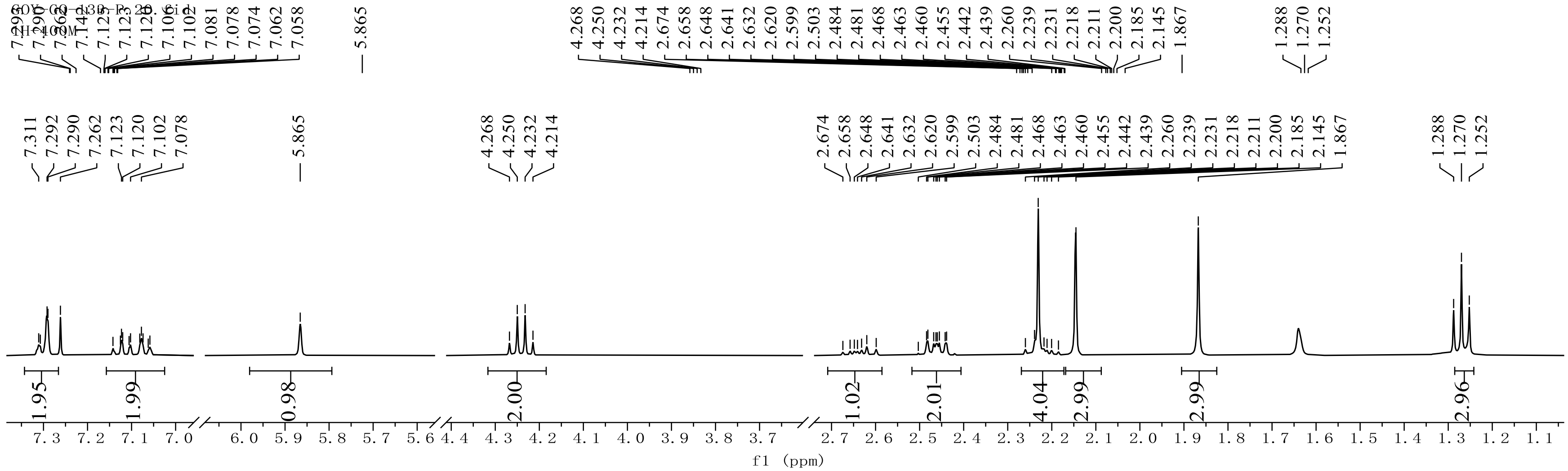

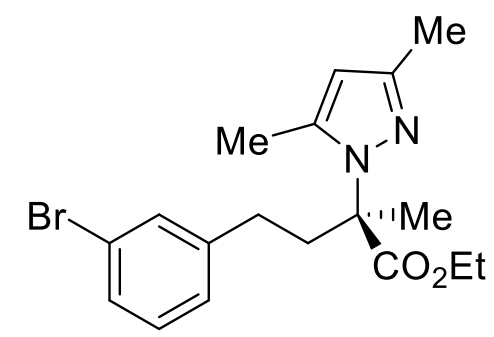

^{1}H NMR of compound **11** (400 MHz, $CDCl_3$)

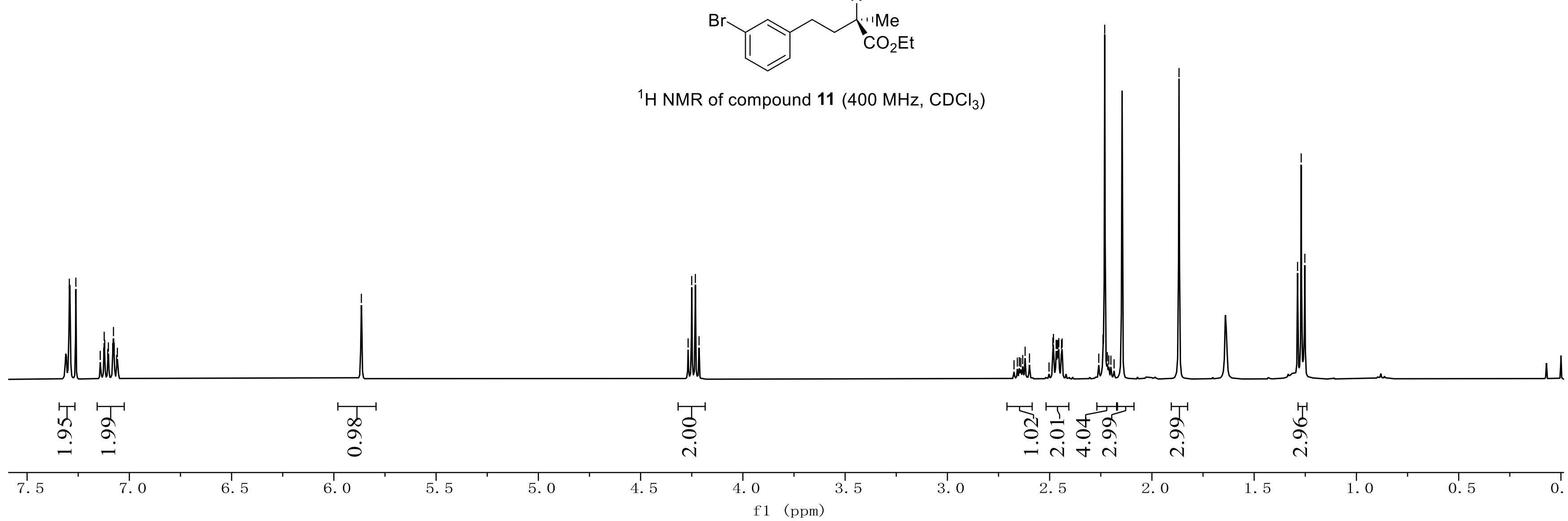

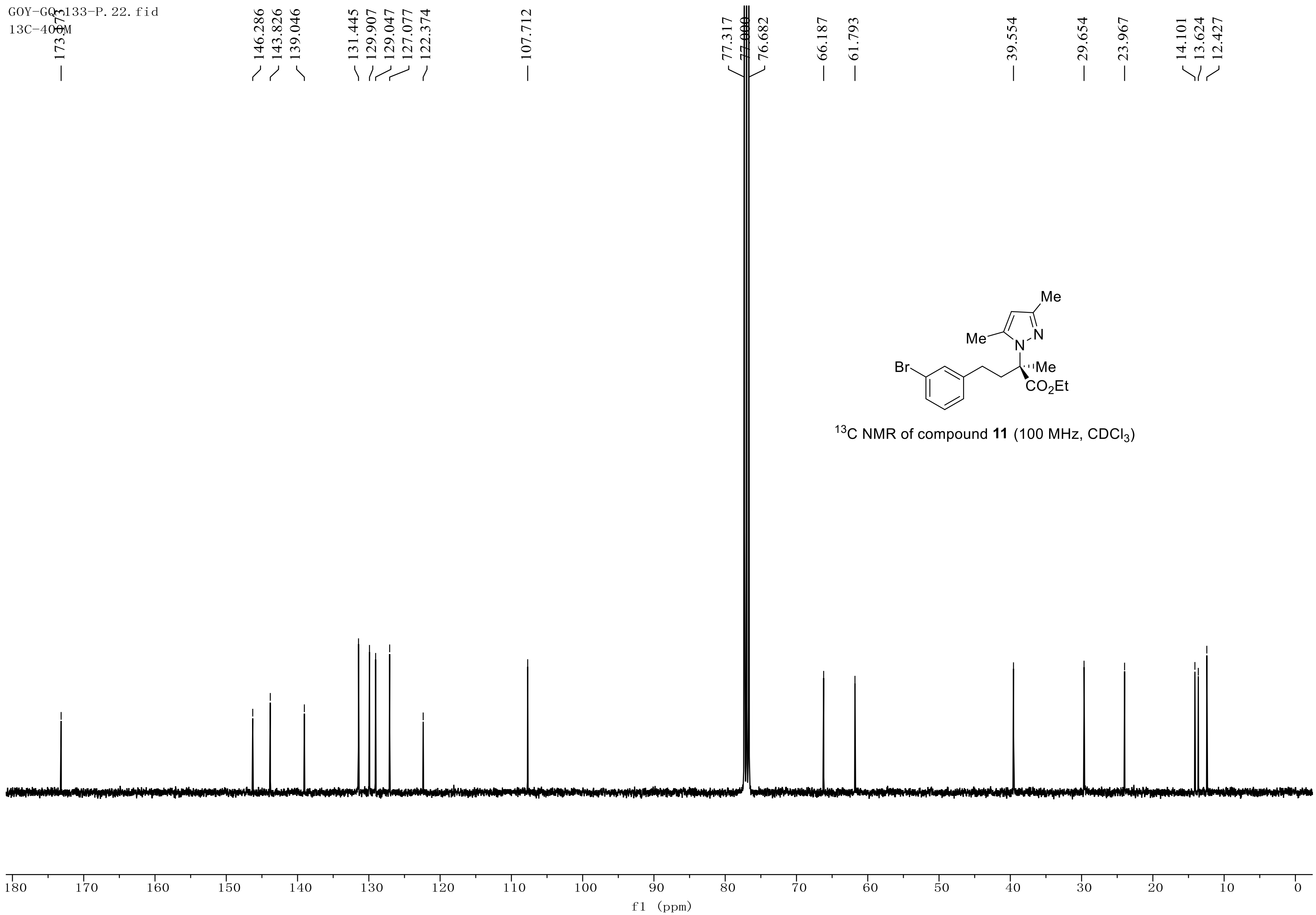

^{13}C NMR of compound **11** (100 MHz, $CDCl_3$)

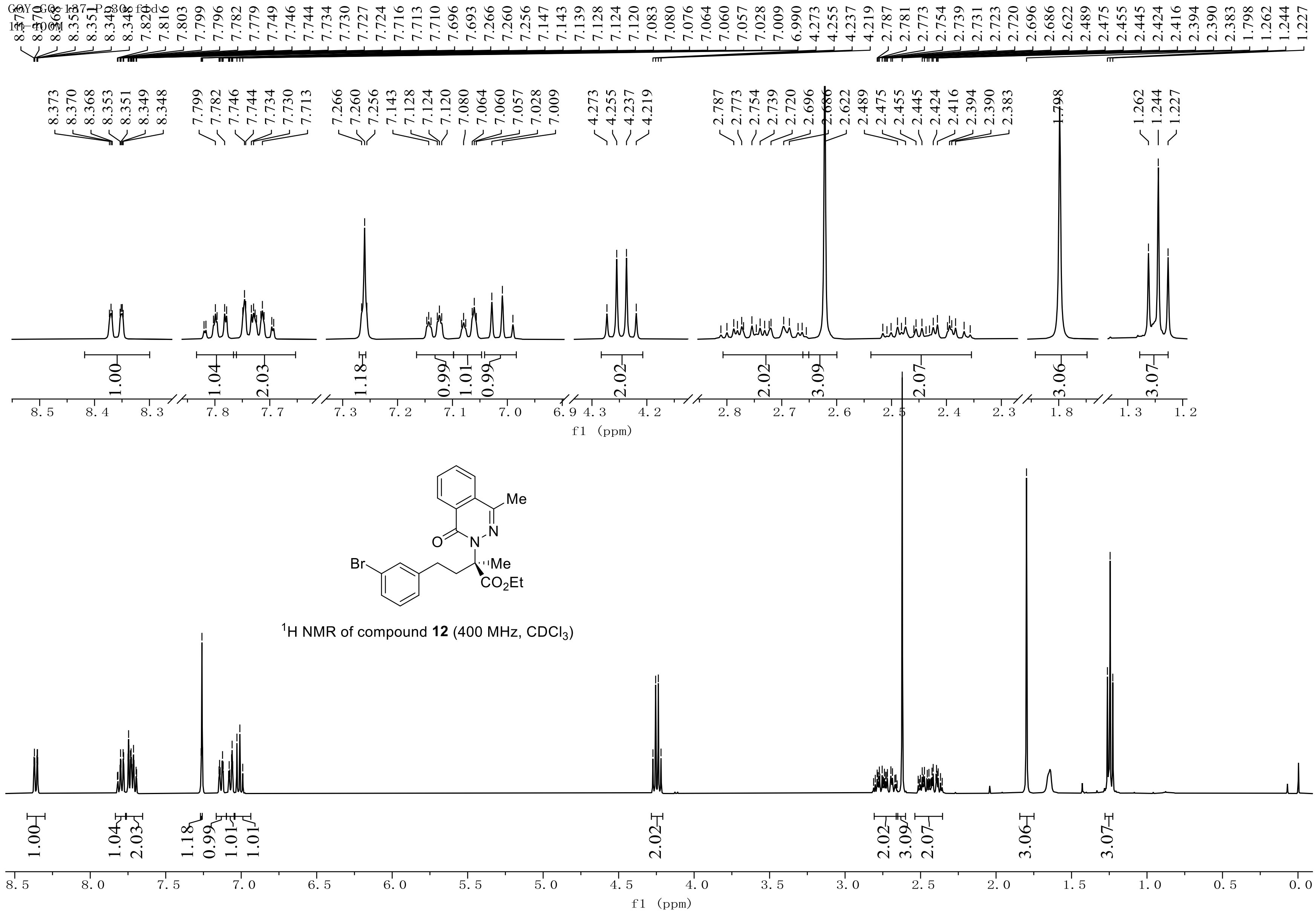

^{1}H NMR of compound **12** (400 MHz, $CDCl_3$)

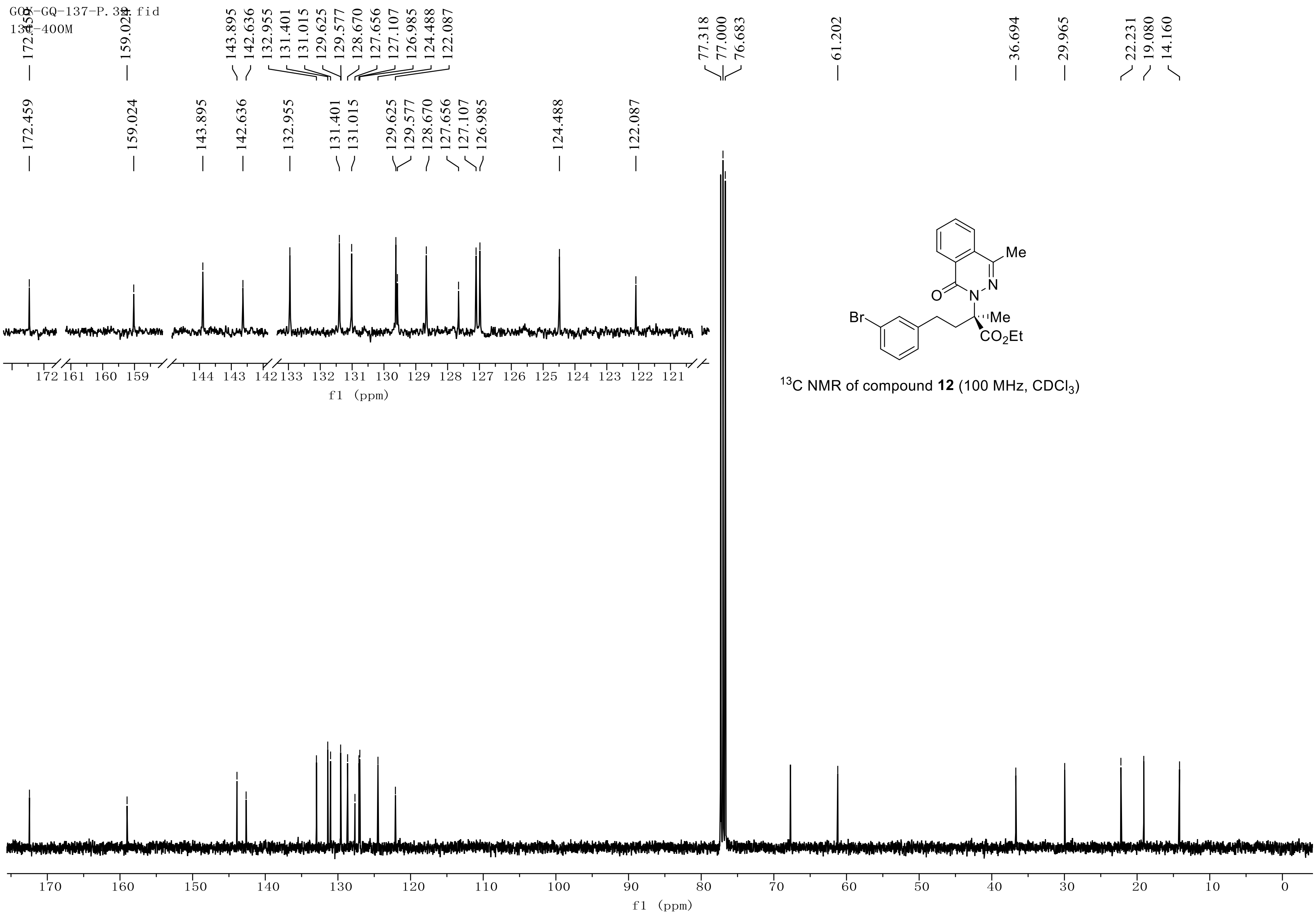

^{13}C NMR of compound **12** (100 MHz, $CDCl_3$)

13. HPLC spectra

<Sample Information>

Sample Name : GOY-GH-29-2-rac-adh-8020-1.0-230
Sample ID :
Data Filename : GOY-GH-29-2-rac-adh-8020-1.0-230.lcd
Method Filename : GY-1.0.lcm
Batch Filename :
Vial # : 1-1 Sample Type : Unknown
Injection Volume : 20 uL
Date Acquired : 12/18/2020 10:08:35 AM Acquired by : System Administrator
Date Processed : 12/18/2020 11:11:44 AM Processed by : System Administrator

<Chromatogram>

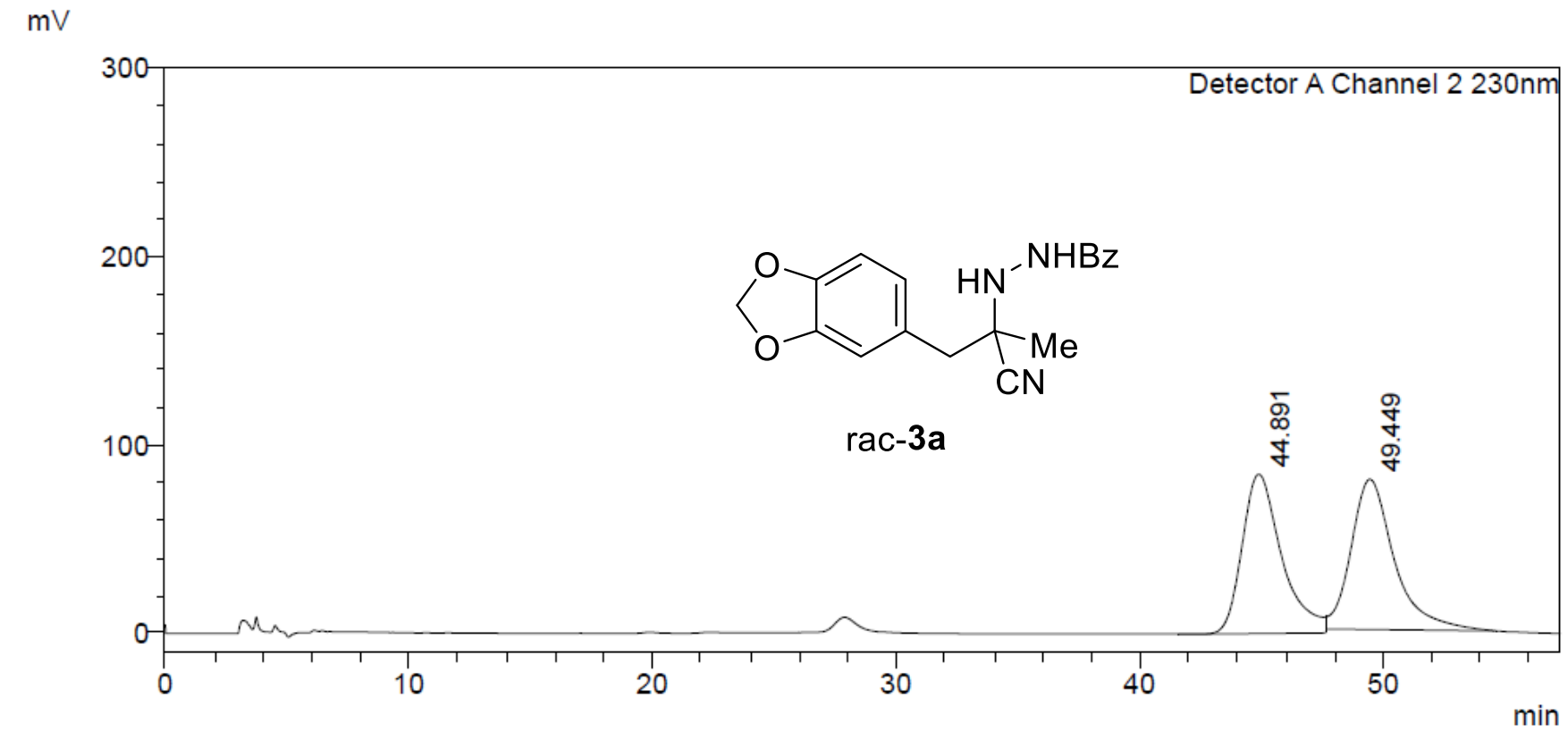

<Peak Table>

Detector A Channel 2 230nm

Peak#	Ret. Time	Area	Height	Conc.
1	44.891	9561248	84619	49.158
2	49.449	9888952	79783	50.842
Total		19450201	164402	

<Sample Information>

Sample Name : GOY-GH-29-2-adh-8020-1.0-230
Sample ID :
Data Filename : GOY-GH-29-2-asy-adh-8020-1.0-230.lcd
Method Filename : GY-1.0.lcm
Batch Filename :
Vial # : 1-1 Sample Type : Unknown
Injection Volume : 20 uL
Date Acquired : 12/18/2020 9:02:39 AM Acquired by : System Administrator
Date Processed : 12/18/2020 7:51:06 PM Processed by : System Administrator

<Chromatogram>

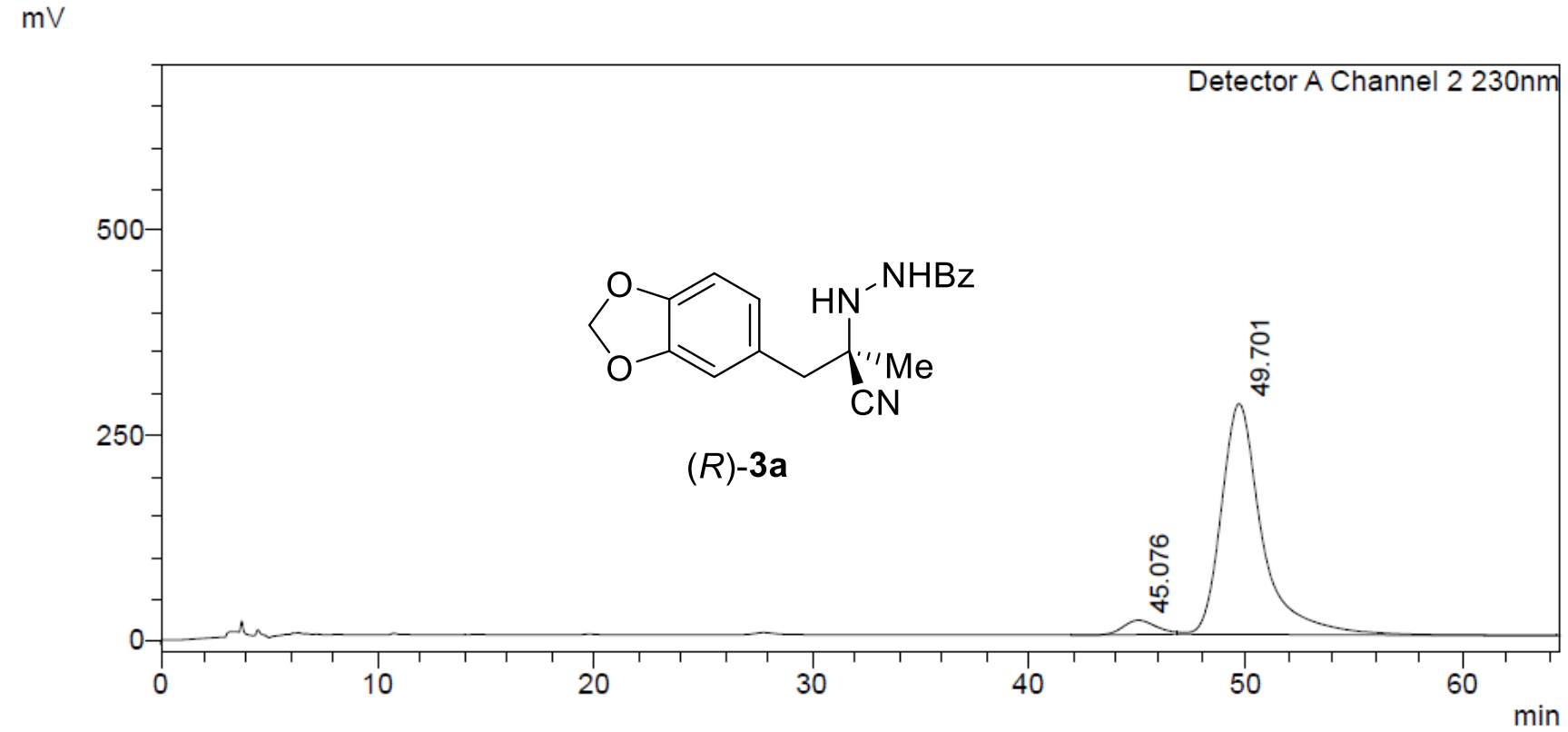

<Peak Table>

Detector A Channel 2 230nm

Peak#	Ret. Time	Area	Height	Conc.
1	45.076	1892886	17522	4.872
2	49.701	36957223	281322	95.128
Total		38850110	298844	

<Sample Information>

Sample Name : GOY-GAA-121-1-rac-AD-3-8020-1.0-230
Sample ID :
Data Filename : GOY-GAA-121-1-rac-AD-3-8020-1.0-230.lcd
Method Filename : GY-1.0.lcm
Batch Filename :
Vial # : 1-1
Sample Type : Unknown
Injection Volume : 20 uL
Date Acquired : 11/14/2024 8:07:33 PM
Acquired by : System Administrator
Date Processed : 2/8/2025 9:24:22 AM
Processed by : System Administrator

<Chromatogram>

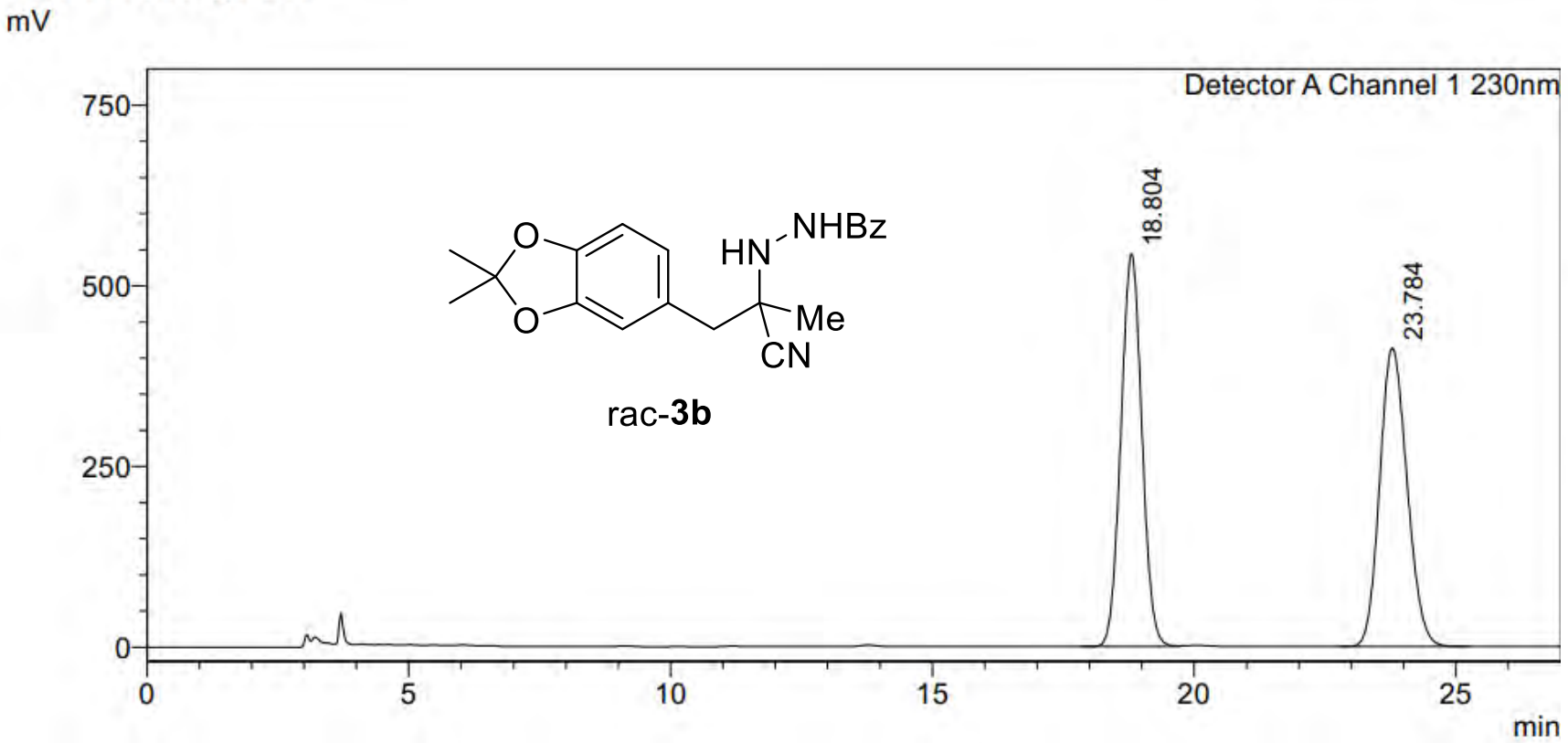

<Peak Table>

Detector A Channel 1 230nm

Peak#	Ret. Time	Area	Height	Conc.
1	18.804	15189338	543341	50.253
2	23.784	15036510	413354	49.747
Total		30225847	956695	

<Sample Information>

Sample Name : GOY-GAA-121-1-asy-AD-3-8020-1.0-230
Sample ID :
Data Filename : GOY-GAA-121-1-asy-AD-3-8020-1.0-230-DATA.lcd
Method Filename : GY-1.0.lcm
Batch Filename :
Vial # : 1-1
Sample Type : Unknown
Injection Volume : 20 uL
Date Acquired : 11/14/2024 4:55:48 PM
Acquired by : System Administrator
Date Processed : 2/8/2025 9:23:41 AM
Processed by : System Administrator

<Chromatogram>

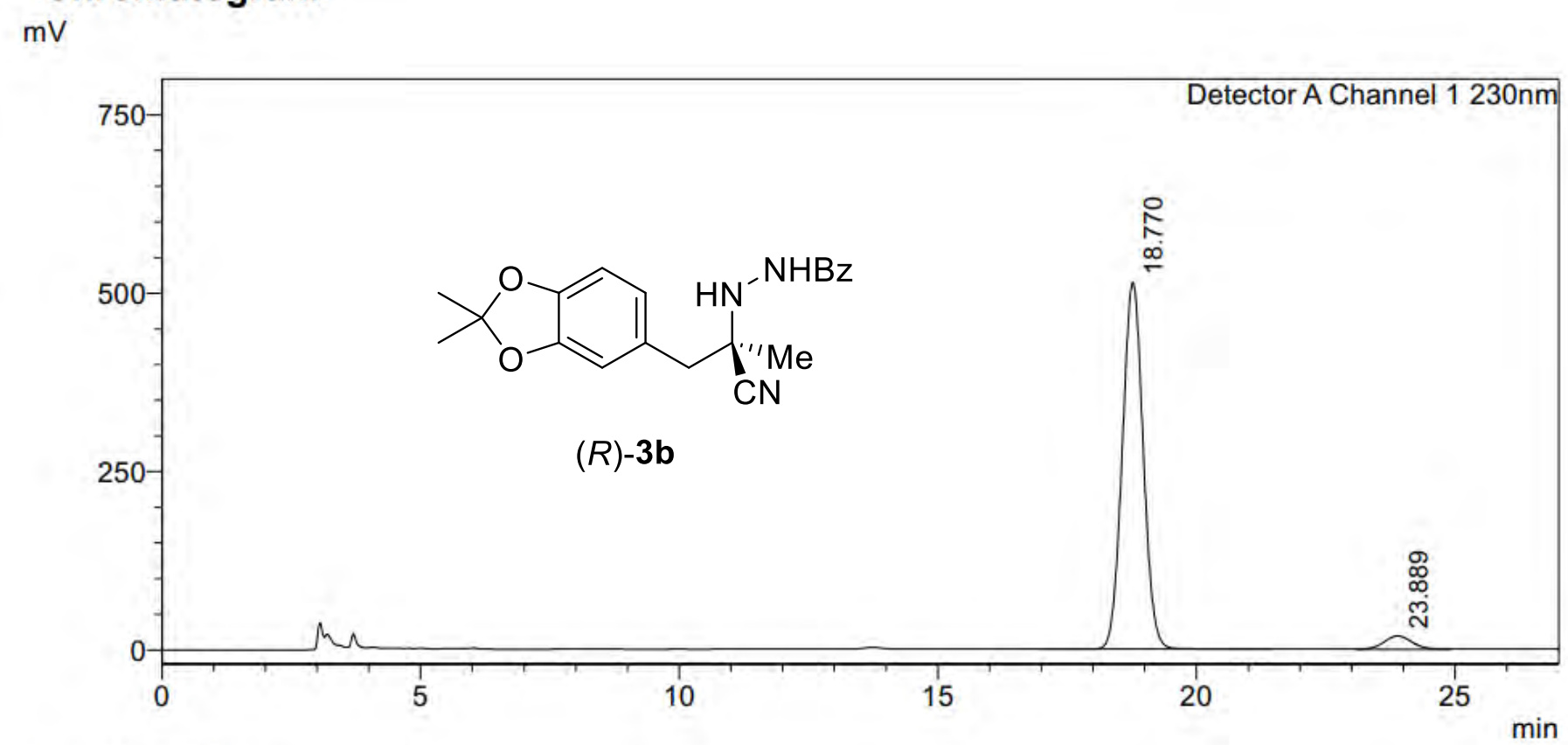

<Peak Table>

Detector A Channel 1 230nm

Peak#	Ret. Time	Area	Height	Conc.
1	18.770	14414064	513785	95.287
2	23.889	712867	18784	4.713
Total		15126931	532569	

<Sample Information>

Sample Name	: GOY-GQ-114-rac-ADH-8020-230-1.0-data		
Sample ID	:		
Data Filename	: GOY-GQ-114-rac-ADH-8020-230-1.0-data.lcd		
Method Filename	: GY-1.0.lcm		
Batch Filename	:		
Vial #	: 1-1	Sample Type	: Unknown
Injection Volume	: 10 uL		
Date Acquired	: 10/19/2022 3:20:48 PM	Acquired by	: System Administrator
Date Processed	: 10/19/2022 4:22:58 PM	Processed by	: System Administrator

<Chromatogram>

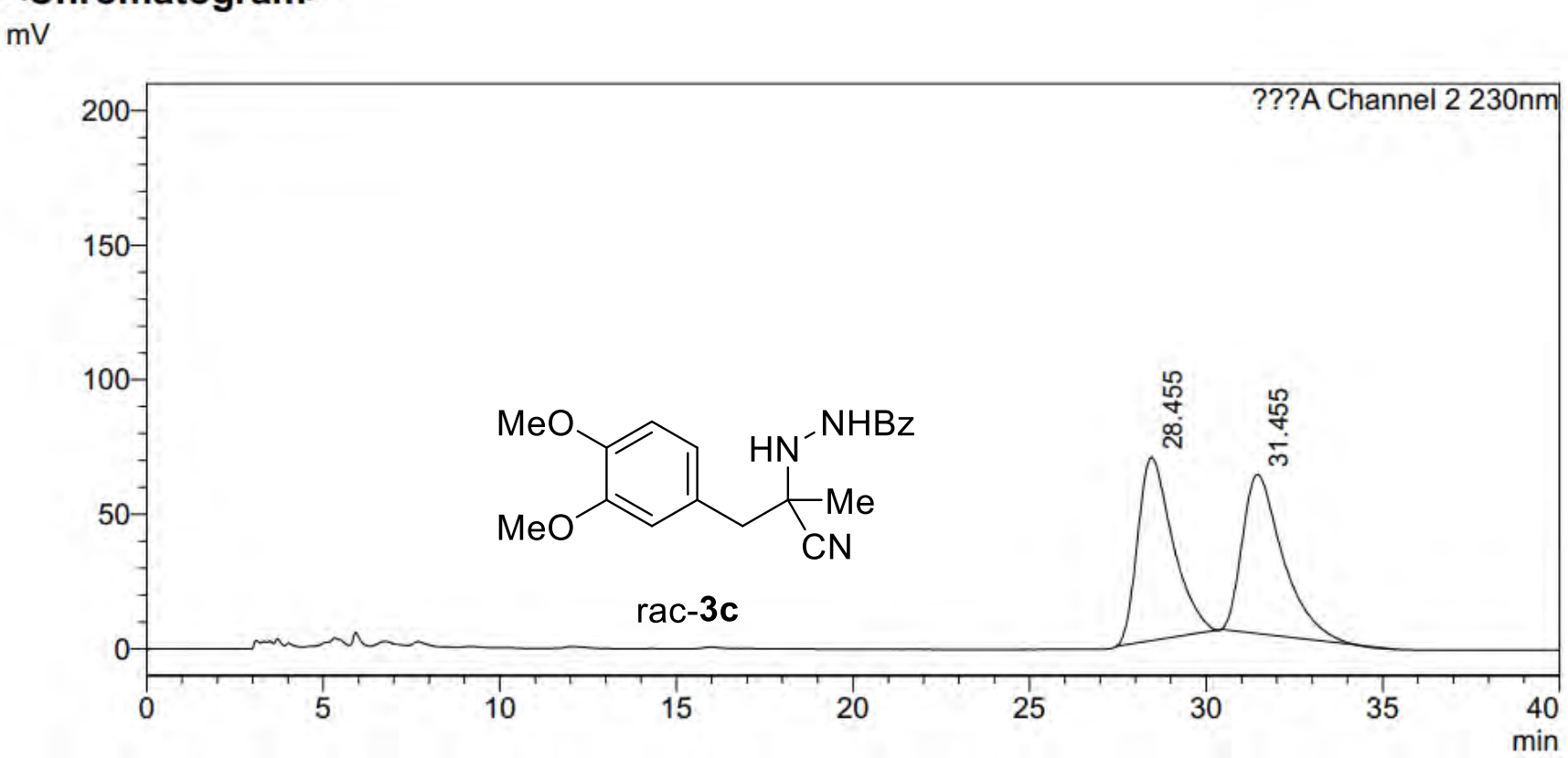

<Peak Table>

???A Channel 2 230nm

Peak#	Ret. Time	Area	Height	Conc.
1	28.455	4709500	68010	49.808
2	31.455	4745806	59188	50.192
Total		9455306	127198	

<Sample Information>

Sample Name	: GOY-GQ-114-asy-ADH-8020-230-1.0		
Sample ID	:		
Data Filename	: GOY-GQ-114-asy-ADH-8020-230-1.0-data.lcd		
Method Filename	: GY-1.0.lcm		
Batch Filename	:		
Vial #	: 1-1	Sample Type	: Unknown
Injection Volume	: 10 uL		
Date Acquired	: 10/19/2022 2:38:31 PM	Acquired by	: System Administrator
Date Processed	: 10/26/2022 6:32:07 PM	Processed by	: System Administrator

<Chromatogram>

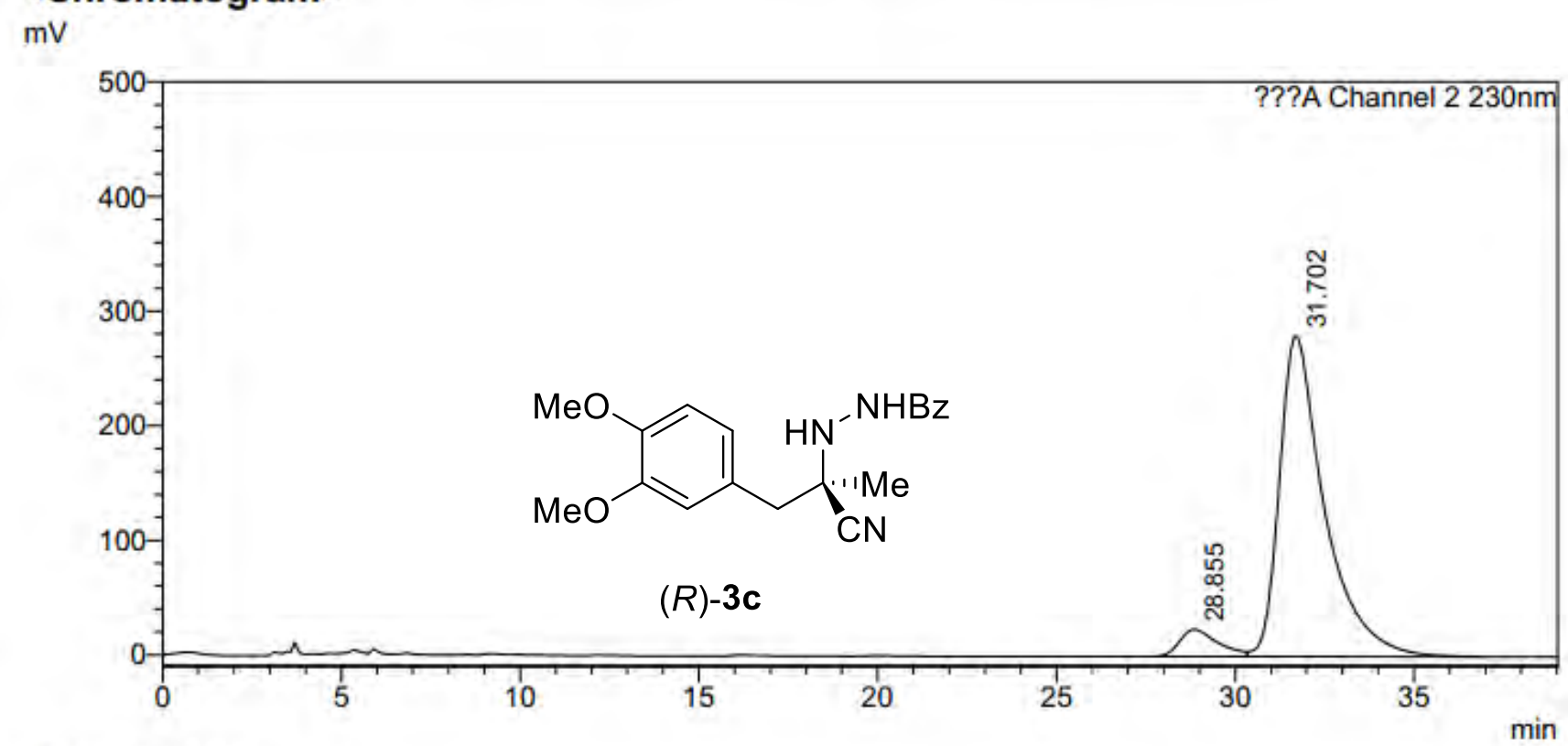

<Peak Table>

???A Channel 2 230nm

Peak#	Ret. Time	Area	Height	Conc.
1	28.855	1763871	23741	6.717
2	31.702	24497148	280096	93.283
Total		26261019	303836	

<Sample Information>

Sample Name	: GOY-GAA-121-2-rac-IE-3-8020-1.0-230
Sample ID	:
Data Filename	: GOY-GAA-121-2-rac-IE-3-8020-1.0-230.lcd
Method Filename	: GY-1.0.lcm
Batch Filename	:
Vial #	: 1-1
Injection Volume	: 20 uL
Date Acquired	: 11/14/2024 10:25:30 PM
Date Processed	: 2/8/2025 9:31:10 AM
Sample Type	: Unknown
Acquired by	: System Administrator
Processed by	: System Administrator

<Chromatogram>

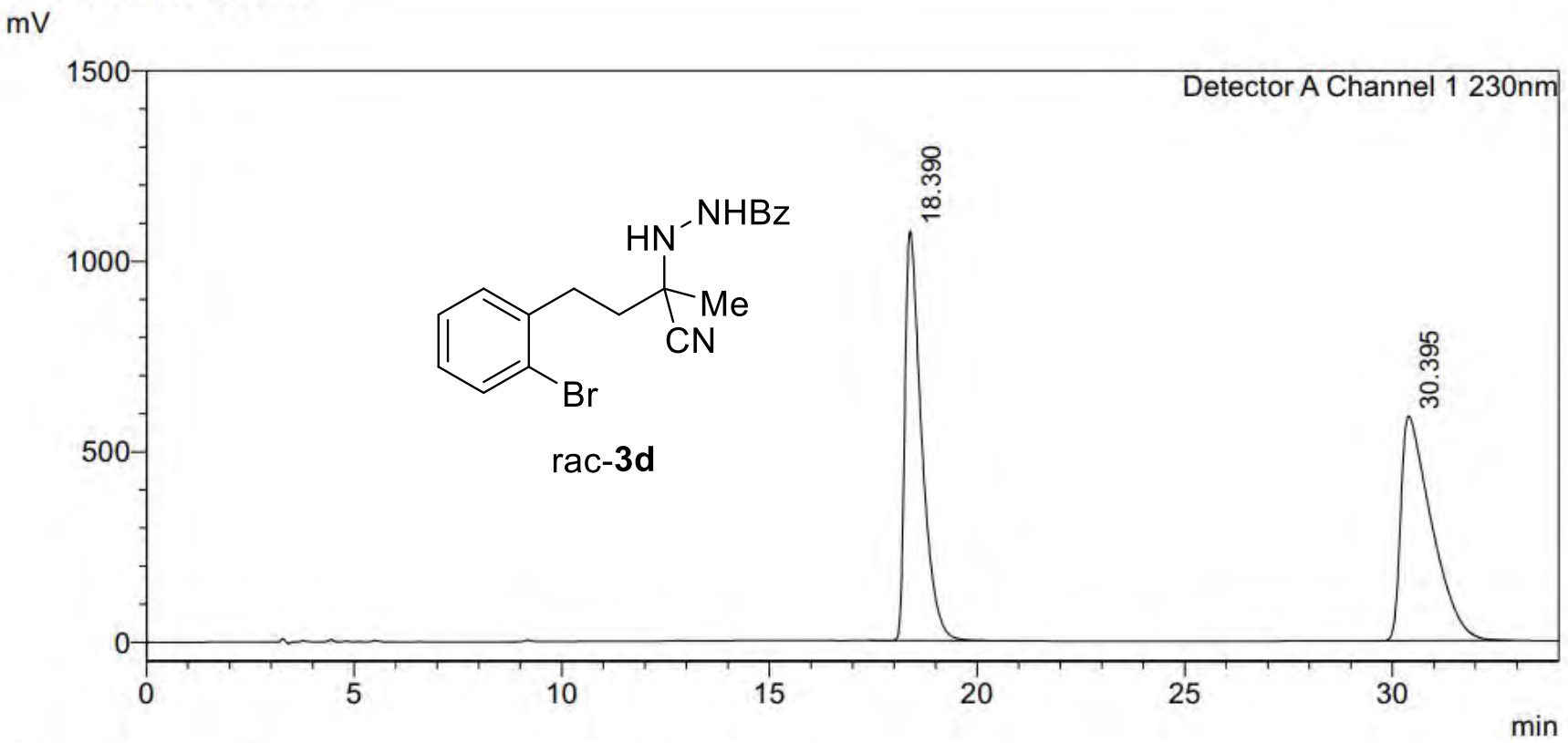

<Peak Table>

Detector A Channel 1 230nm

Peak#	Ret. Time	Area	Height	Conc.
1	18.390	30118529	1075431	49.732
2	30.395	30442778	589517	50.268
Total		60561308	1664948	

<Sample Information>

Sample Name	: GOY-GAA-121-2-asy-IE-3-8020-1.0-230
Sample ID	:
Data Filename	: GOY-GAA-121-2-asy-IE-3-8020-1.0-231.lcd
Method Filename	: GY-1.0.lcm
Batch Filename	:
Vial #	: 1-1
Injection Volume	: 20 uL
Date Acquired	: 11/14/2024 9:50:27 PM
Date Processed	: 11/15/2024 8:39:32 AM
Sample Type	: Unknown
Acquired by	: System Administrator
Processed by	: System Administrator

<Chromatogram>

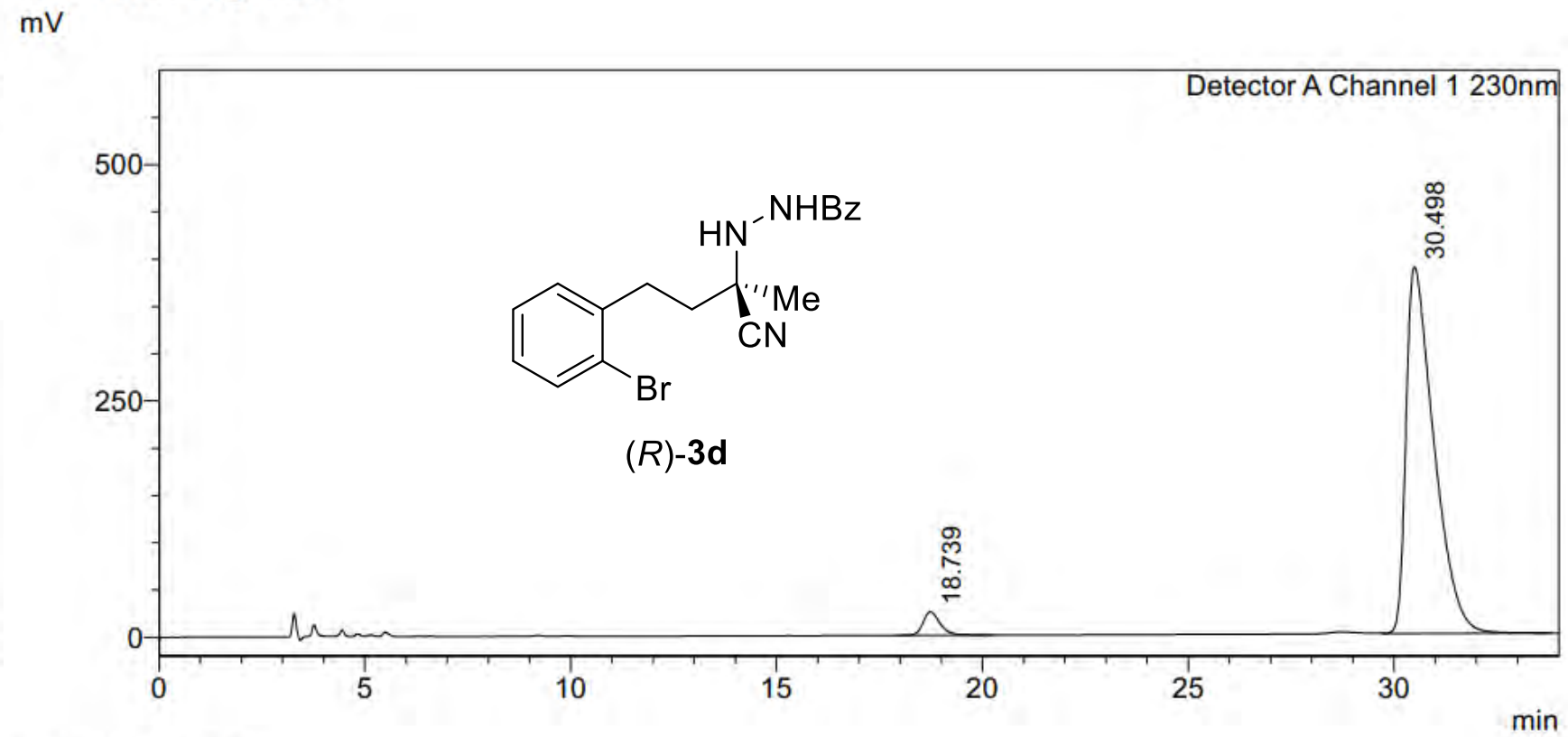

<Peak Table>

Detector A Channel 1 230nm

Peak#	Ret. Time	Area	Height	Conc.
1	18.739	719827	24904	3.782
2	30.498	18313509	388274	96.218
Total		19033336	413178	

<Sample Information>

Sample Name	: GOY-GP-130-rac-IE-8020-230-1.0-DATA		
Sample ID	:		
Data Filename	: GOY-GP-130-rac-IE-8020-230-1.0-DATA.lcd		
Method Filename	: GY-1.0.lcm		
Batch Filename	:		
Vial #	: 1-1	Sample Type	: Unknown
Injection Volume	: 20 uL		
Date Acquired	: 8/11/2022 4:31:17 PM	Acquired by	: System Administrator
Date Processed	: 8/18/2022 11:13:09 AM	Processed by	: System Administrator

<Chromatogram>

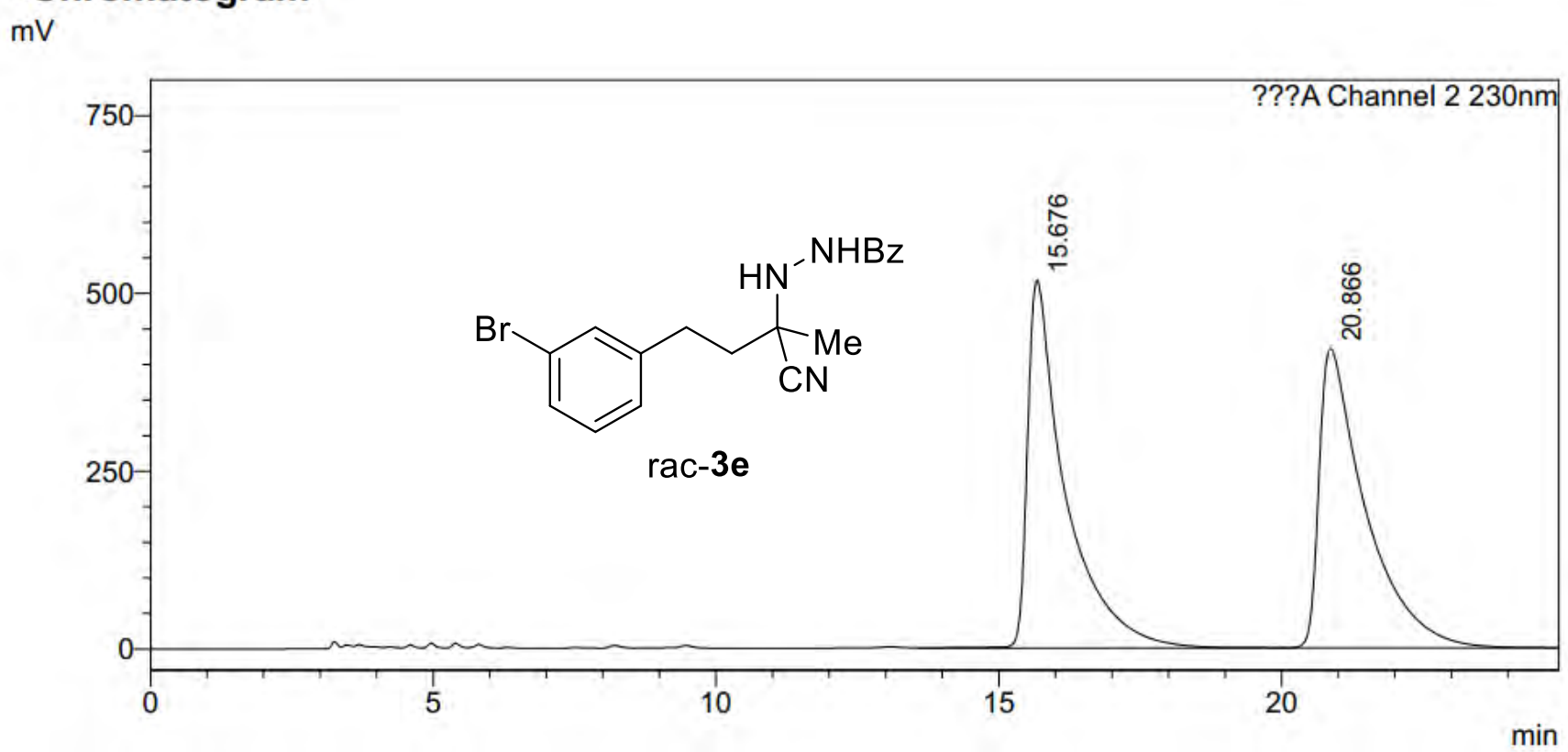

<Peak Table>

???A Channel 2 230nm

Peak#	Ret. Time	Area	Height	Conc.
1	15.676	24045229	517473	50.137
2	20.866	23913756	420712	49.863
Total		47958985	938184	

<Sample Information>

Sample Name	: GOY-GP-130-asy-IE-8020-230-1.0-DATA		
Sample ID	:		
Data Filename	: GOY-GP-130-asy-IE-8020-230-1.0-DATA.lcd		
Method Filename	: GY-1.0.lcm		
Batch Filename	:		
Vial #	: 1-1	Sample Type	: Unknown
Injection Volume	: 20 uL		
Date Acquired	: 8/11/2022 3:34:36 PM	Acquired by	: System Administrator
Date Processed	: 10/11/2022 6:39:19 PM	Processed by	: System Administrator

<Chromatogram>

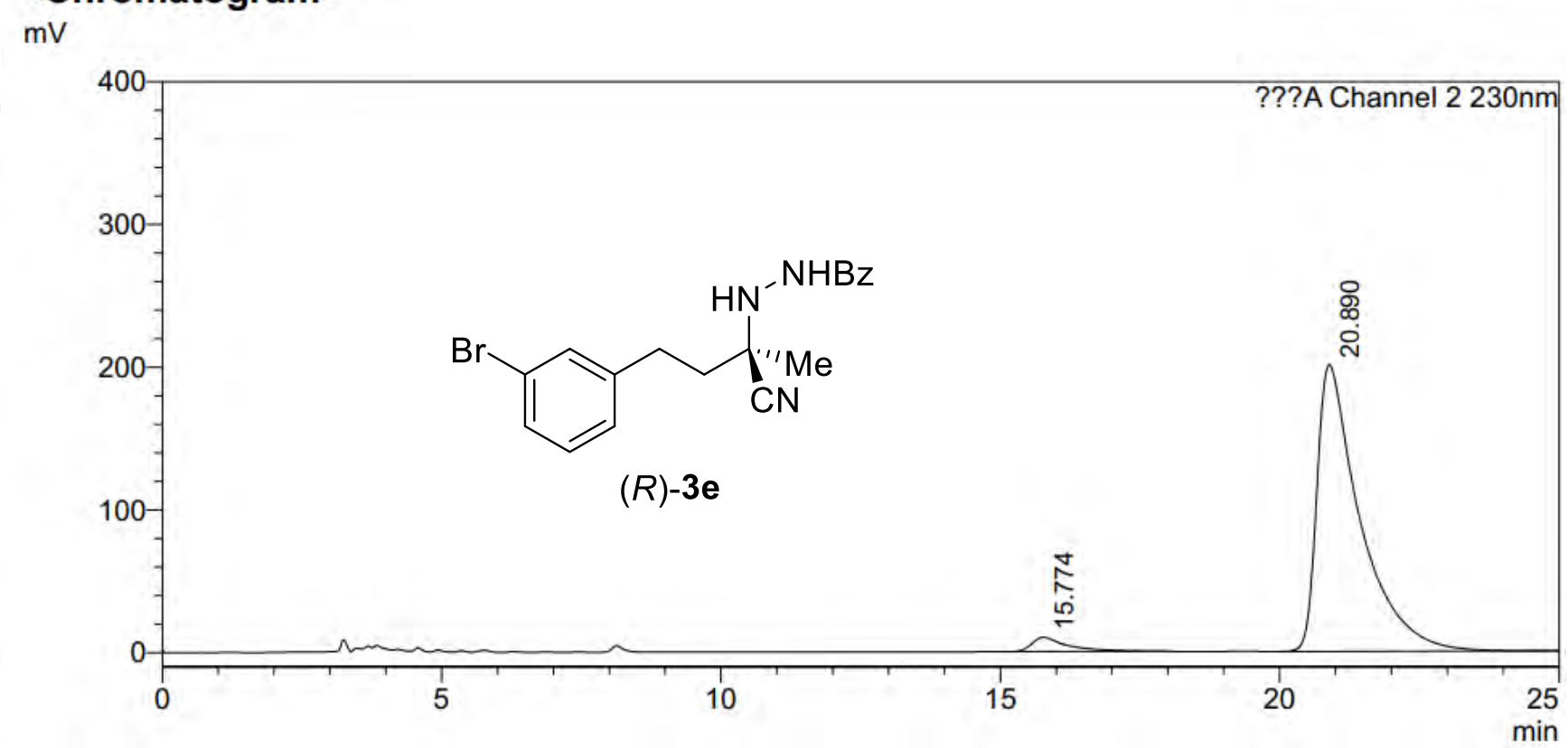

<Peak Table>

???A Channel 2 230nm

Peak#	Ret. Time	Area	Height	Conc.
1	15.774	449162	9988	4.017
2	20.890	10732750	200736	95.983
Total		11181913	210724	

<Sample Information>

Sample Name : GOY-GP-96-RAC-IF-7030-230-1.0
Sample ID :
Data Filename : GOY-GP-96-RAC-IF-7030-230-1.0.lcd
Method Filename : GY-1.0-OVEN.lcm
Batch Filename :
Vial # : 1-1
Injection Volume : 20 uL
Date Acquired : 7/28/2022 10:57:46 AM
Date Processed : 7/28/2022 11:48:26 AM
Sample Type : Unknown
Acquired by : System Administrator
Processed by : System Administrator

<Chromatogram>

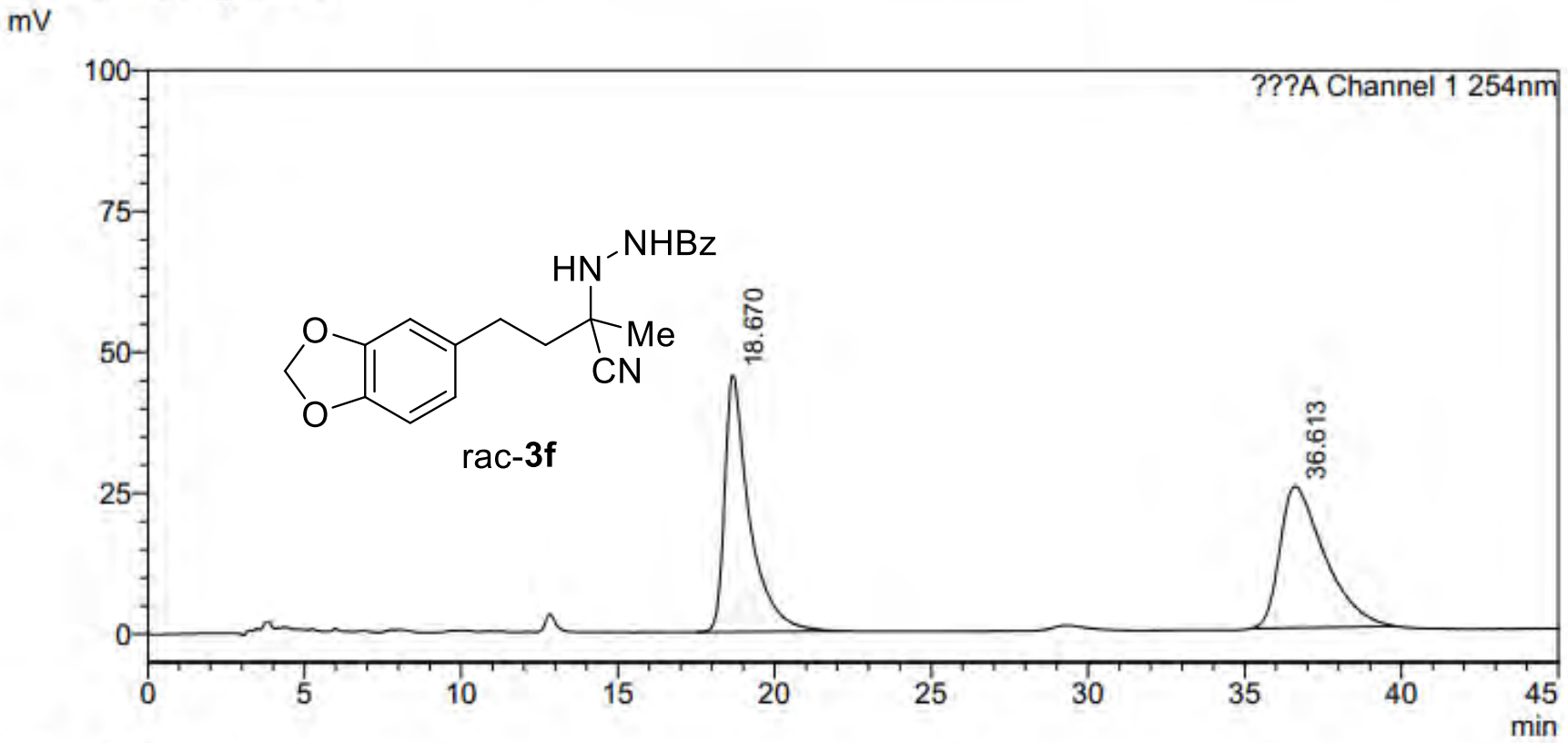

<Peak Table>

???A Channel 1 254nm

Peak#	Ret. Time	Area	Height	Conc.
1	18.670	2514952	45587	50.141
2	36.613	2500823	25111	49.859
Total		5015775	70697	

<Sample Information>

Sample Name : GOY-GP-96-asy-IF-7030-230-1.0
Sample ID :
Data Filename : GOY-GP-96-asy-IF-7030-230-1.0.lcd
Method Filename : GY-1.0-OVEN.lcm
Batch Filename :
Vial # : 1-1
Injection Volume : 20 uL
Date Acquired : 7/28/2022 9:21:54 AM
Date Processed : 7/28/2022 11:47:15 AM
Sample Type : Unknown
Acquired by : System Administrator
Processed by : System Administrator

<Chromatogram>

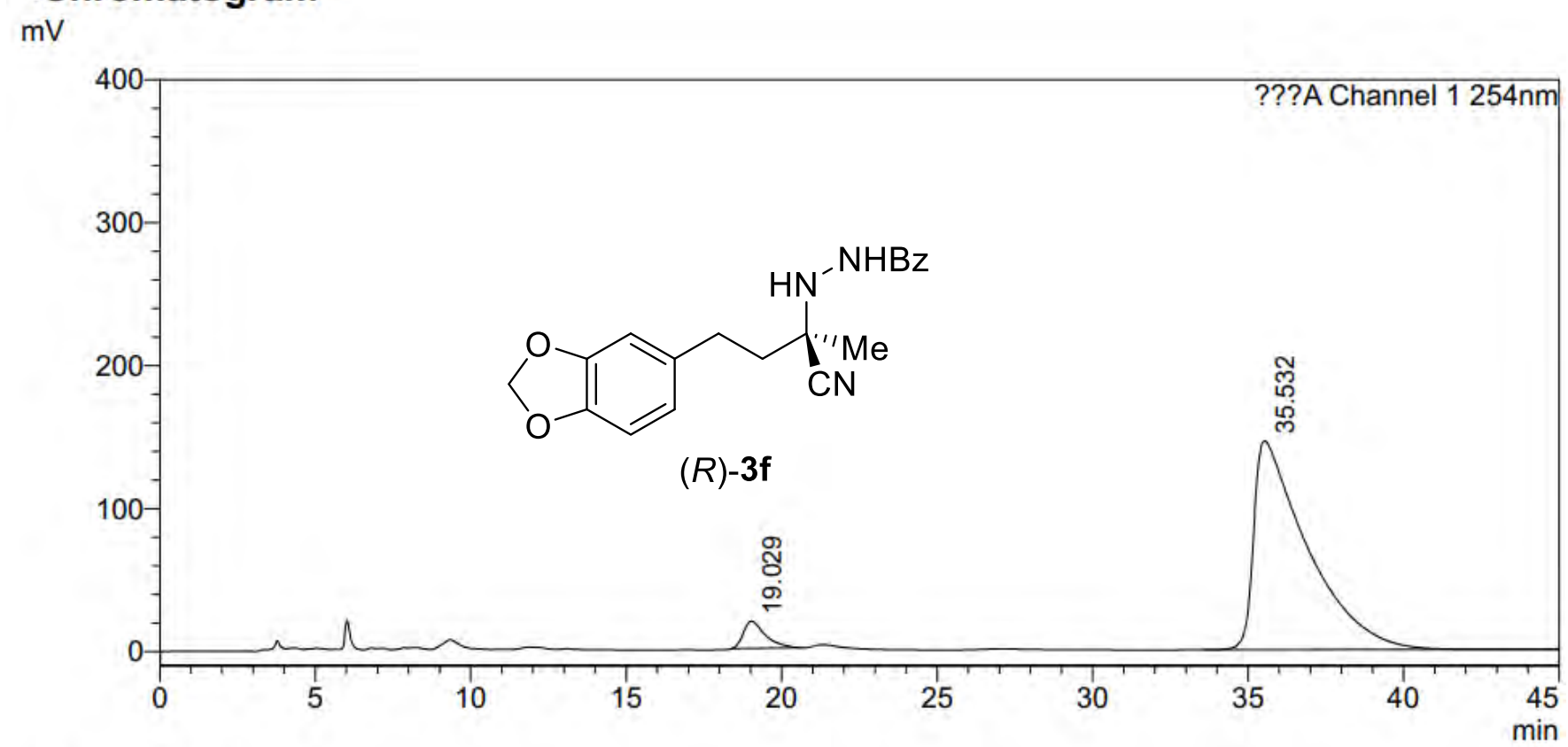

<Peak Table>

???A Channel 1 254nm

Peak#	Ret. Time	Area	Height	Conc.
1	19.029	925079	18990	4.995
2	35.532	17595126	146201	95.005
Total		18520205	165191	

<Sample Information>

Sample Name : GOY-GP-76-rac-IE-6040-230-1.0
Sample ID :
Data Filename : GOY-GP-76-rac-IE-6040-230-1.1.lcd
Method Filename : GY-1.0.lcm
Batch Filename :
Vial # : 1-1
Injection Volume : 20 uL
Date Acquired : 7/22/2022 3:11:20 PM
Date Processed : 7/22/2022 3:37:07 PM
Sample Type : Unknown
Acquired by : System Administrator
Processed by : System Administrator

<Chromatogram>

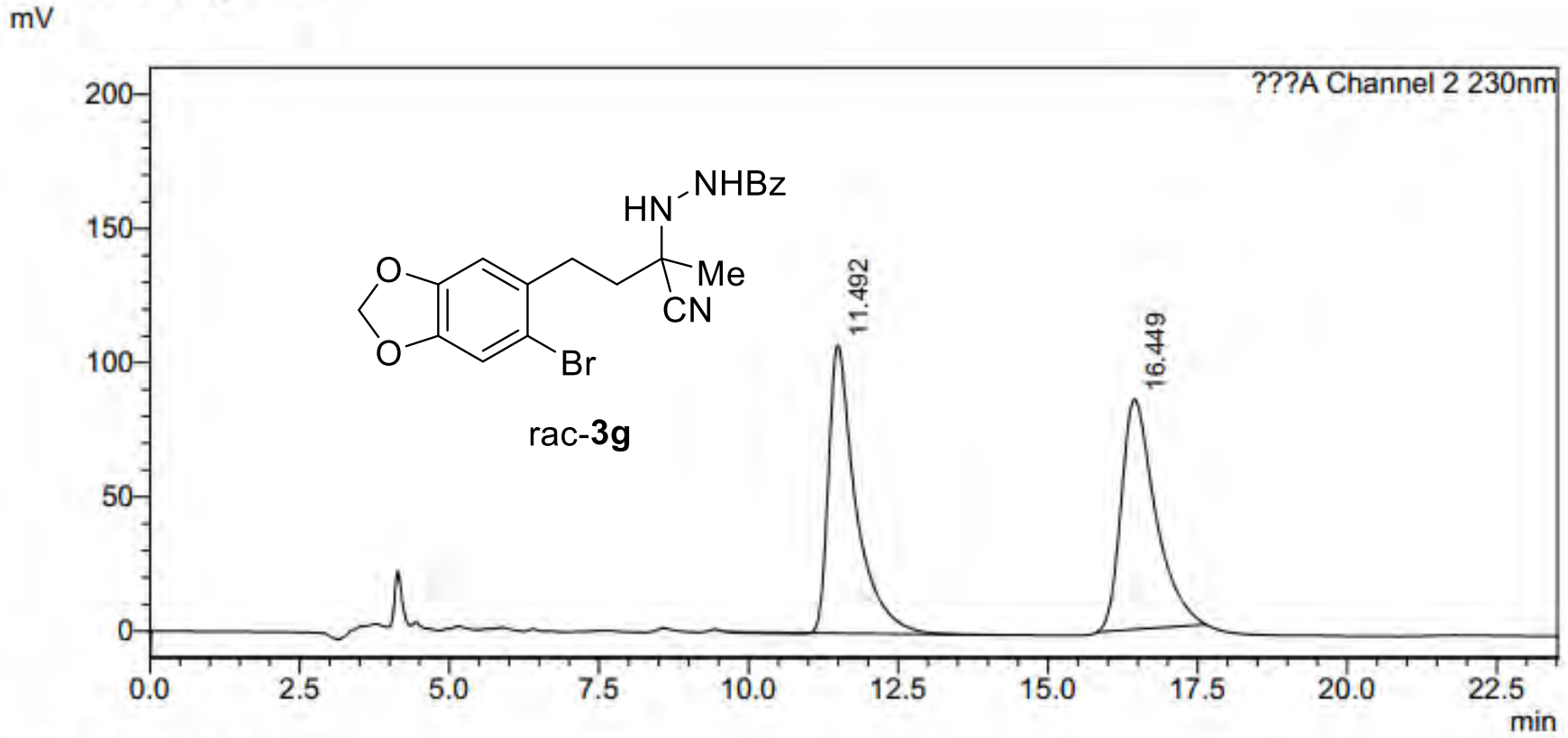

<Peak Table>

???A Channel 2 230nm

Peak#	Ret. Time	Area	Height	Conc.
1	11.492	3379935	107466	49.690
2	16.449	3422096	85765	50.310
Total		6802030	193231	

<Sample Information>

Sample Name : GOY-GP-76-asy-IE-6040-230-1.0
Sample ID :
Data Filename : GOY-GP-76-asy-IE-6040-230-1.0.lcd
Method Filename : GY-1.0.lcm
Batch Filename :
Vial # : 1-1
Injection Volume : 20 uL
Date Acquired : 7/22/2022 2:35:58 PM
Date Processed : 7/22/2022 3:09:21 PM
Sample Type : Unknown
Acquired by : System Administrator
Processed by : System Administrator

<Chromatogram>

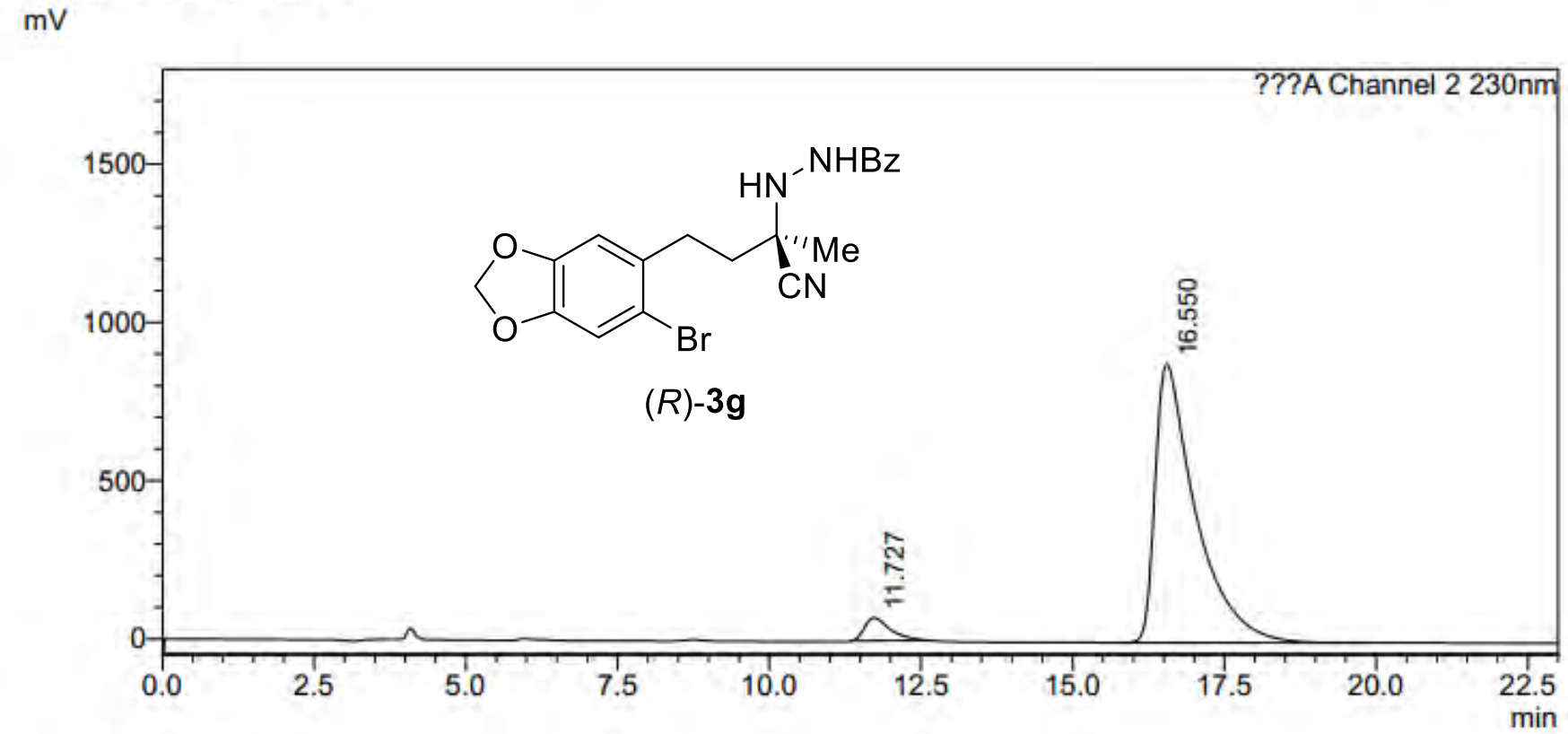

<Peak Table>

???A Channel 2 230nm

Peak#	Ret. Time	Area	Height	Conc.
1	11.727	2121835	72457	4.935
2	16.550	40874970	880045	95.065
Total		42996805	952502	

<Sample Information>

Sample Name	: GOY-GAB-17-5-rac-IB-3-8020-1.0-230		
Sample ID	:		
Data Filename	: GOY-GAB-17-5-rac-IB-3-8020-1.0-230-DATA.lcd		
Method Filename	: GY-1.0-OVEN.lcm		
Batch Filename	:		
Vial #	: 1-1	Sample Type	: Unknown
Injection Volume	: 20 uL		
Date Acquired	: 12/18/2024 5:08:48 PM	Acquired by	: System Administrator
Date Processed	: 1/22/2025 2:19:39 AM	Processed by	: System Administrator

<Chromatogram>

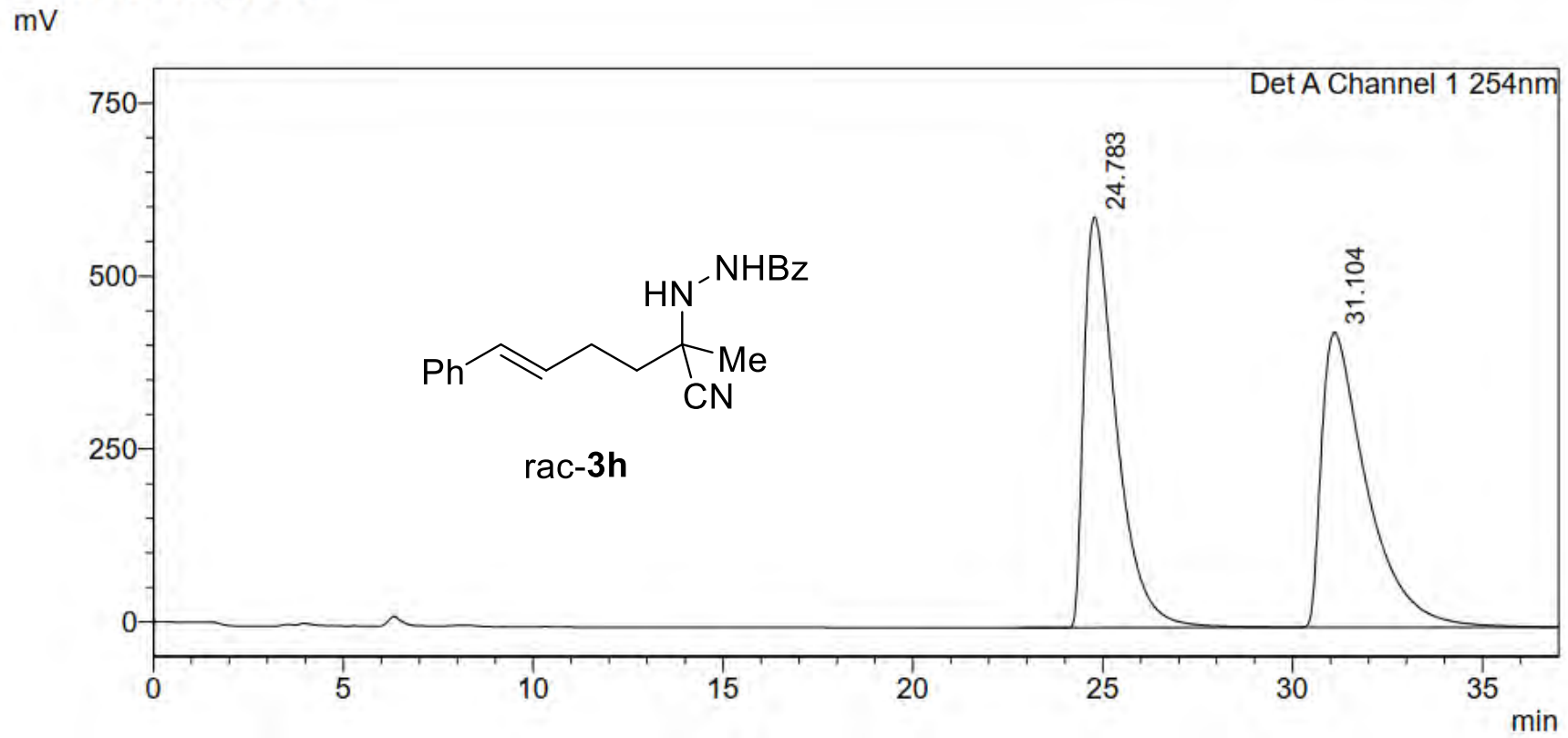

<Peak Table>

Det A Channel 1 254nm

Peak#	Ret. Time	Area	Height	Conc.
1	24.783	35306693	593446	50.004
2	31.104	35301113	425770	49.996
Total		70607806	1019216	

<Sample Information>

Sample Name	: GOY-GAB-17-5-asy-IB-3-8020-1.0-230-DATA		
Sample ID	:		
Data Filename	: GOY-GAB-17-5-asy-IB-3-8020-1.0-230-DATA.lcd		
Method Filename	: GY-1.0-OVEN.lcm		
Batch Filename	:		
Vial #	: 1-1	Sample Type	: Unknown
Injection Volume	: 20 uL		
Date Acquired	: 12/18/2024 5:58:17 PM	Acquired by	: System Administrator
Date Processed	: 12/18/2024 11:34:43 PM	Processed by	: System Administrator

<Chromatogram>

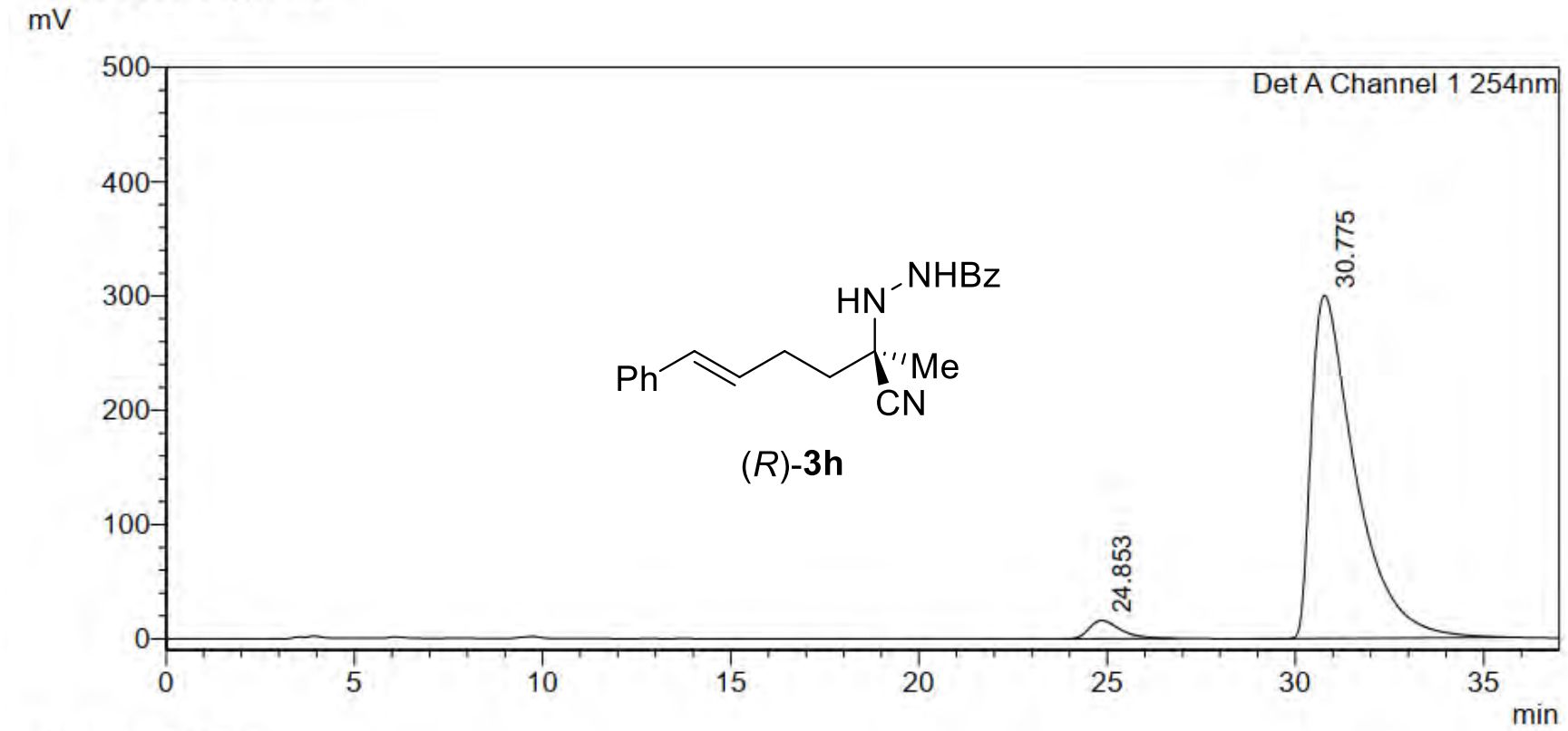

<Peak Table>

Det A Channel 1 254nm

Peak#	Ret. Time	Area	Height	Conc.
1	24.853	916570	15960	3.605
2	30.775	24506587	300262	96.395
Total		25423157	316222	

<Sample Information>

Sample Name	: GOY-GAB-38-2-rac-IB-3-8020-1.0-230		
Sample ID	:		
Data Filename	: GOY-GAB-38-2-rac-IB-3-8020-1.0-230.lcd		
Method Filename	: GY-1.0-OVEN.lcm		
Batch Filename	:		
Vial #	: 1-1	Sample Type	: Unknown
Injection Volume	: 20 uL		
Date Acquired	: 1/16/2025 8:57:47 PM	Acquired by	: System Administrator
Date Processed	: 1/22/2025 2:24:20 AM	Processed by	: System Administrator

<Chromatogram>

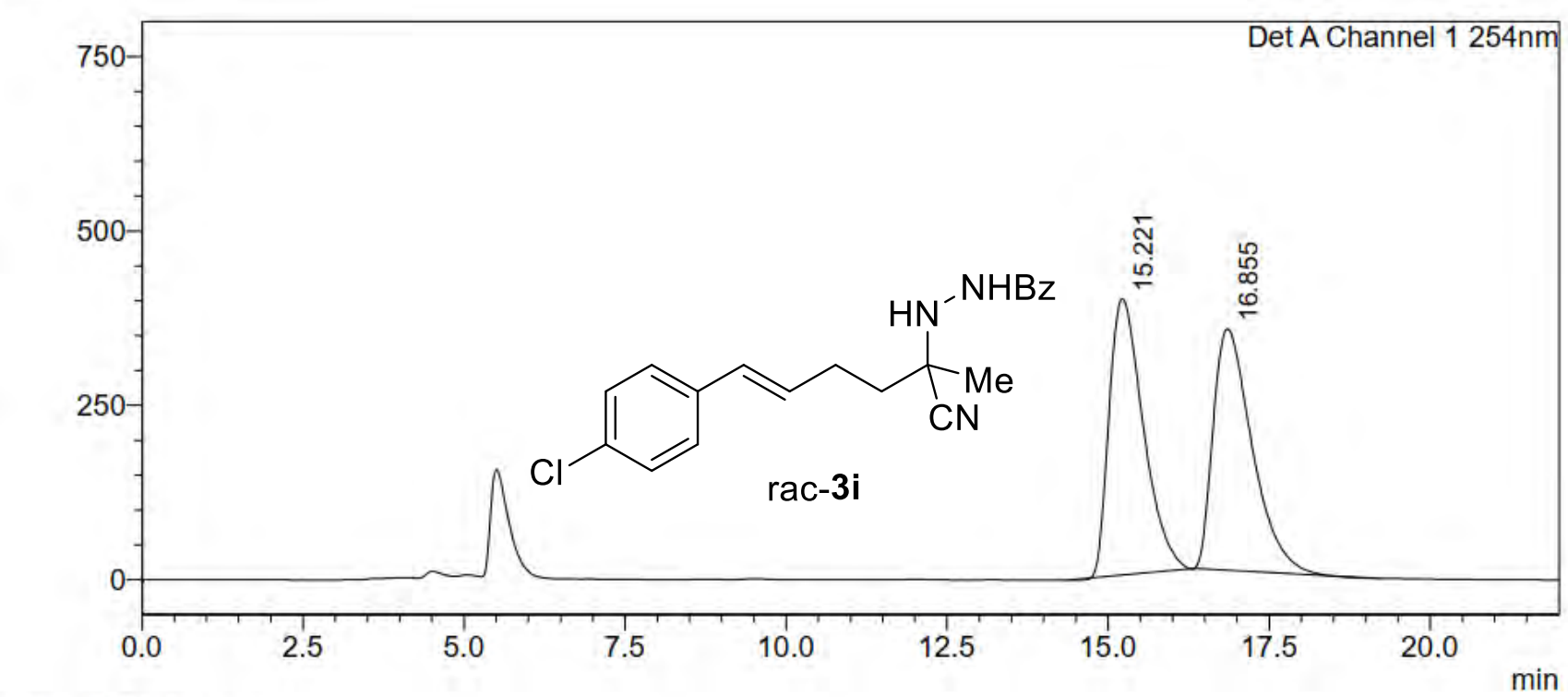

<Peak Table>

Det A Channel 1 254nm

Peak#	Ret. Time	Area	Height	Conc.
1	15.221	14567200	395844	50.068
2	16.855	14527513	345483	49.932
Total		29094713	741327	

<Sample Information>

Sample Name	: GOY-GAB-38-2-asy-IB-3-8020-1.0-230		
Sample ID	:		
Data Filename	: GOY-GAB-38-2-asy-IB-3-8020-1.0-230.lcd		
Method Filename	: GY-1.0-OVEN.lcm		
Batch Filename	:		
Vial #	: 1-1	Sample Type	: Unknown
Injection Volume	: 20 uL		
Date Acquired	: 1/16/2025 8:17:30 PM	Acquired by	: System Administrator
Date Processed	: 1/22/2025 2:24:48 AM	Processed by	: System Administrator

<Chromatogram>

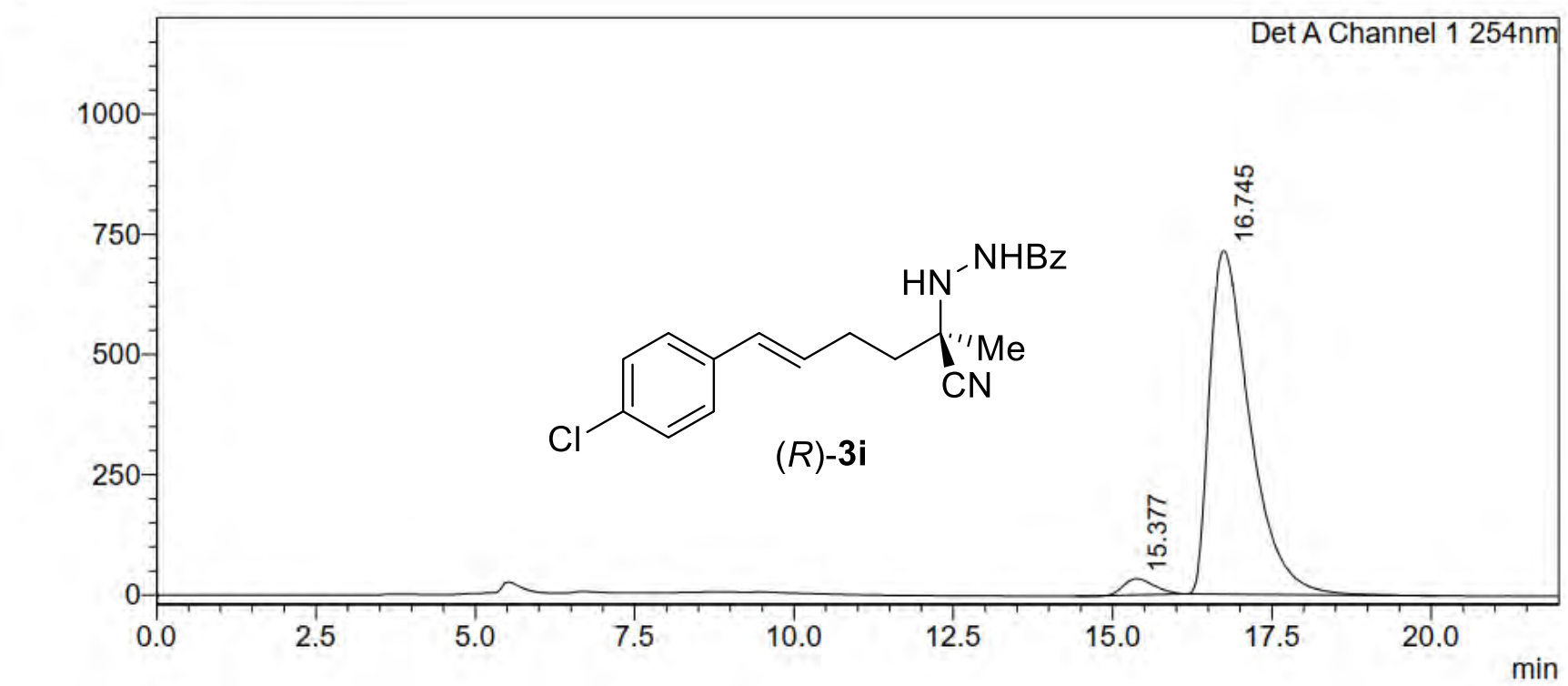

<Peak Table>

Det A Channel 1 254nm

Peak#	Ret. Time	Area	Height	Conc.
1	15.377	1132248	33824	3.454
2	16.745	31651944	714967	96.546
Total		32784192	748791	

<Sample Information>

Sample Name : GOY-GAB-38-1-rac-IB-3-8020-1.0-230
Sample ID :
Data Filename : GOY-GAB-38-1-rac-IB-3-8020-1.0-230-DATA.lcd
Method Filename : GY-1.0-OVEN.lcm
Batch Filename :
Vial # : 1-1
Sample Type : Unknown
Injection Volume : 20 uL
Date Acquired : 1/13/2025 4:54:39 PM
Acquired by : System Administrator
Date Processed : 1/22/2025 2:22:00 AM
Processed by : System Administrator

<Chromatogram>

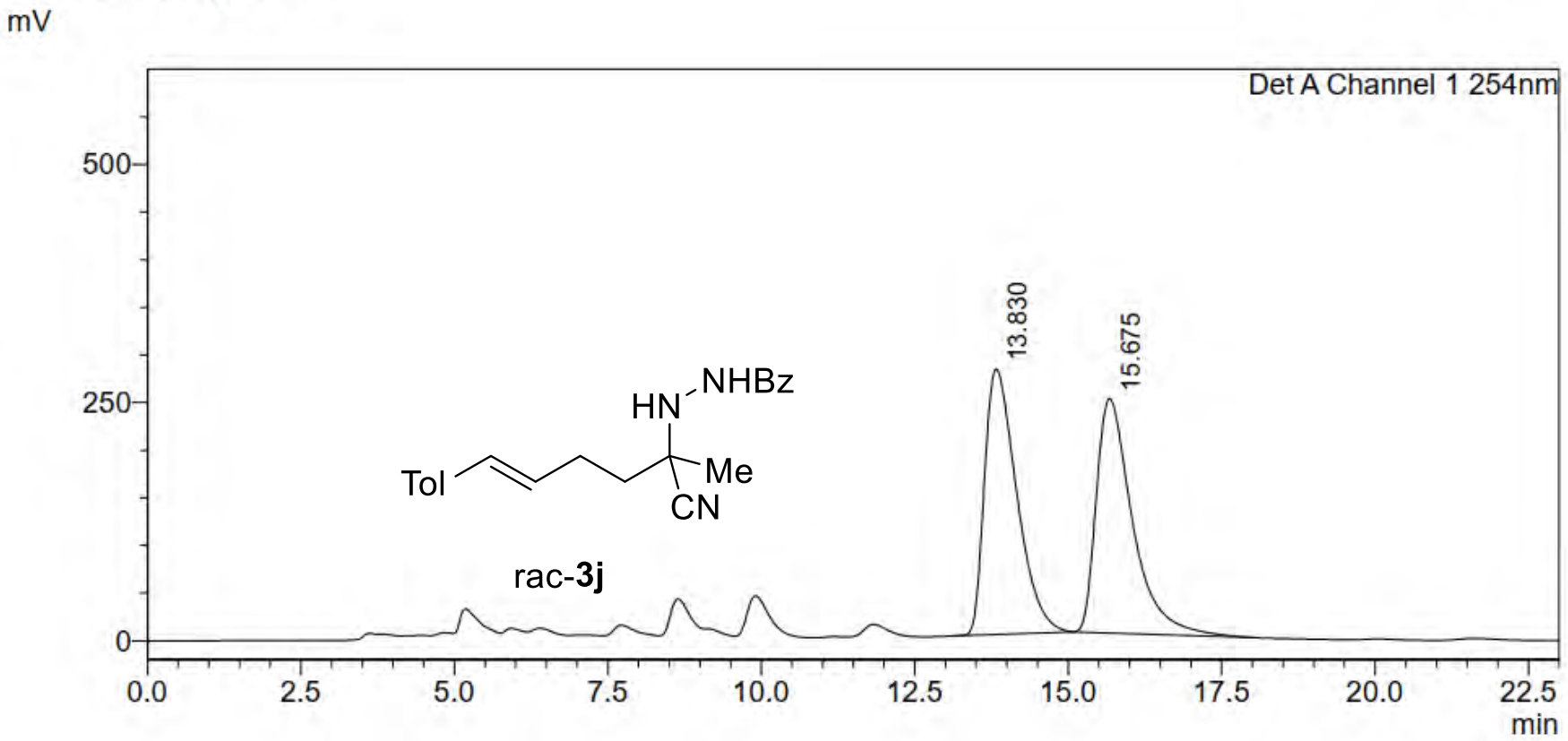

<Peak Table>

Det A Channel 1 254nm

Peak#	Ret. Time	Area	Height	Conc.
1	13.830	10176040	278634	50.101
2	15.675	10135159	246036	49.899
Total		20311199	524671	

<Sample Information>

Sample Name : GOY-GAB-38-1-asy-IB-3-8020-1.0-230
Sample ID :
Data Filename : GOY-GAB-38-1-asy-IB-3-8020-1.0-230-DATA.lcd
Method Filename : GY-1.0-OVEN.lcm
Batch Filename :
Vial # : 1-1
Sample Type : Unknown
Injection Volume : 20 uL
Date Acquired : 1/13/2025 4:13:59 PM
Acquired by : System Administrator
Date Processed : 1/22/2025 2:21:43 AM
Processed by : System Administrator

<Chromatogram>

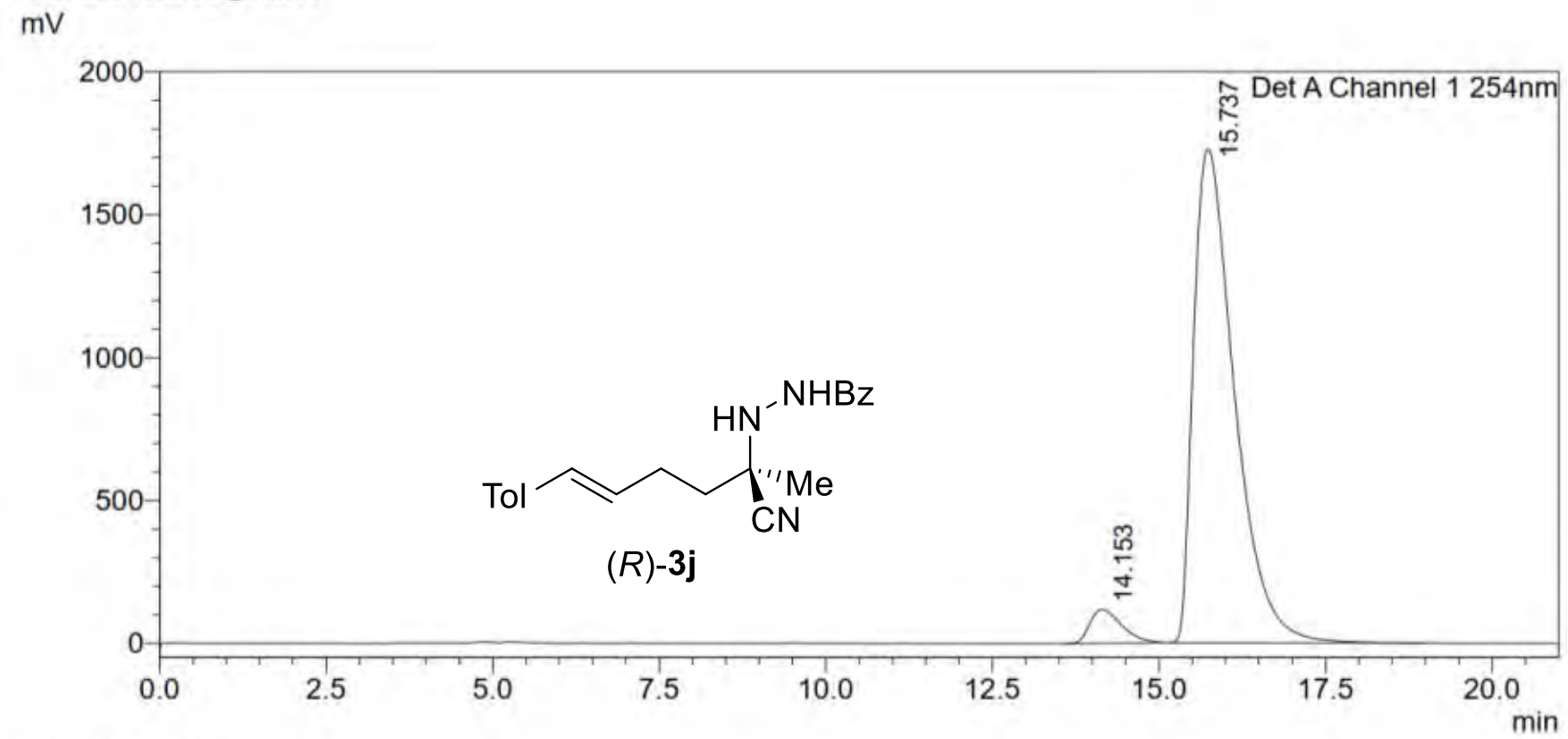

<Peak Table>

Det A Channel 1 254nm

Peak#	Ret. Time	Area	Height	Conc.
1	14.153	3899793	118572	5.002
2	15.737	74065500	1728555	94.998
Total		77965293	1847127	

<Sample Information>

Sample Name : GOY-GAB-17-1-rac-IF-3-8020-1.0-230
Sample ID :
Data Filename : GOY-GAB-17-1-rac-IF-3-8020-1.0-230-DATA.lcd
Method Filename : GY-1.0-OVEN.lcm
Batch Filename :
Vial # : 1-1 Sample Type : Unknown
Injection Volume : 20 uL
Date Acquired : 12/18/2024 7:46:35 PM Acquired by : System Administrator
Date Processed : 1/22/2025 2:07:40 AM Processed by : System Administrator

<Chromatogram>

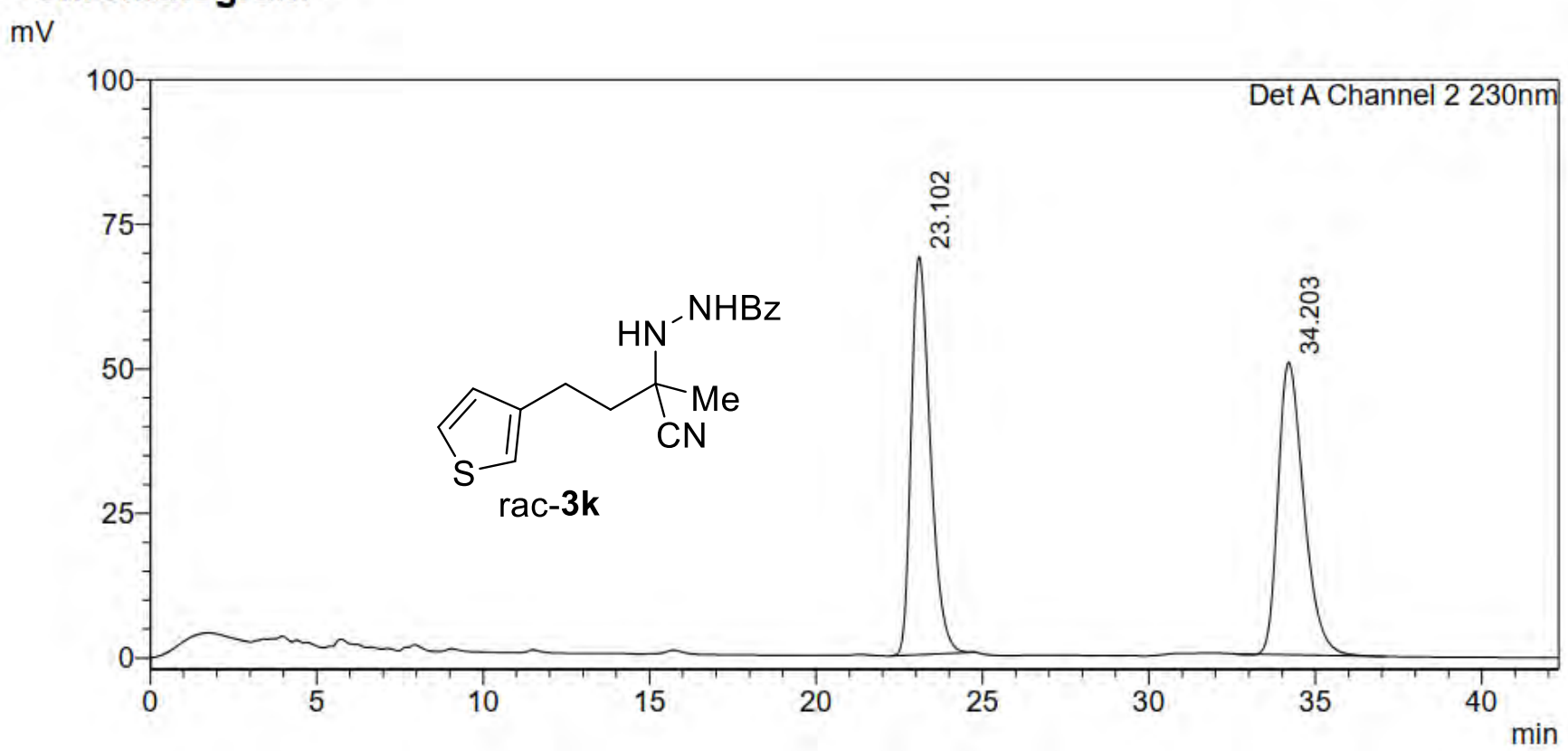

<Peak Table>

Det A Channel 2 230nm

Peak#	Ret. Time	Area	Height	Conc.
1	23.102	2753699	68861	50.314
2	34.203	2719349	50562	49.686
Total		5473048	119424	

<Sample Information>

Sample Name : GOY-GAB-17-1-asy-IF-3-8020-1.0-230
Sample ID :
Data Filename : GOY-GAB-17-1-asy-IF-3-8020-1.0-231-DATA.lcd
Method Filename : GY-1.0-OVEN.lcm
Batch Filename :
Vial # : 1-1 Sample Type : Unknown
Injection Volume : 20 uL
Date Acquired : 12/18/2024 10:53:25 PM Acquired by : System Administrator
Date Processed : 1/22/2025 5:04:21 AM Processed by : System Administrator

<Chromatogram>

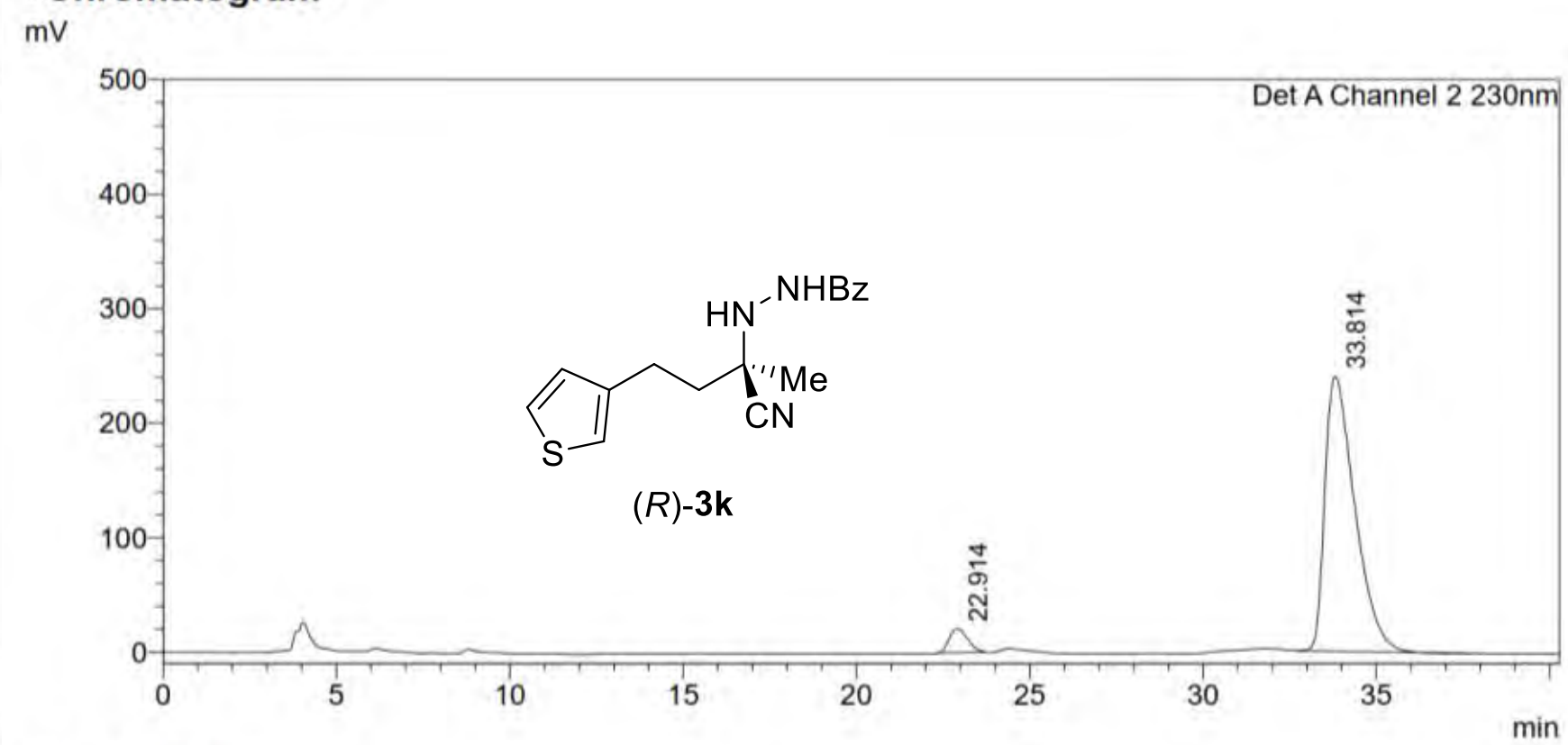

<Peak Table>

Det A Channel 2 230nm

Peak#	Ret. Time	Area	Height	Conc.
1	22.914	784064	21124	5.139
2	33.814	14471854	240089	94.861
Total		15255918	261213	

<Sample Information>

Sample Name	: GOY-GAB-17-2-rac-IA-3-8020-1.0-230
Sample ID	:
Data Filename	: GOY-GAB-17-2-rac-IA-3-8020-1.0-230-DATA.lcd
Method Filename	: GY-1.0-OVEN.lcm
Batch Filename	:
Vial #	: 1-1
Injection Volume	: 20 uL
Date Acquired	: 12/19/2024 1:32:38 PM
Date Processed	: 1/22/2025 2:13:47 AM
Sample Type	: Unknown
Acquired by	: System Administrator
Processed by	: System Administrator

<Chromatogram>

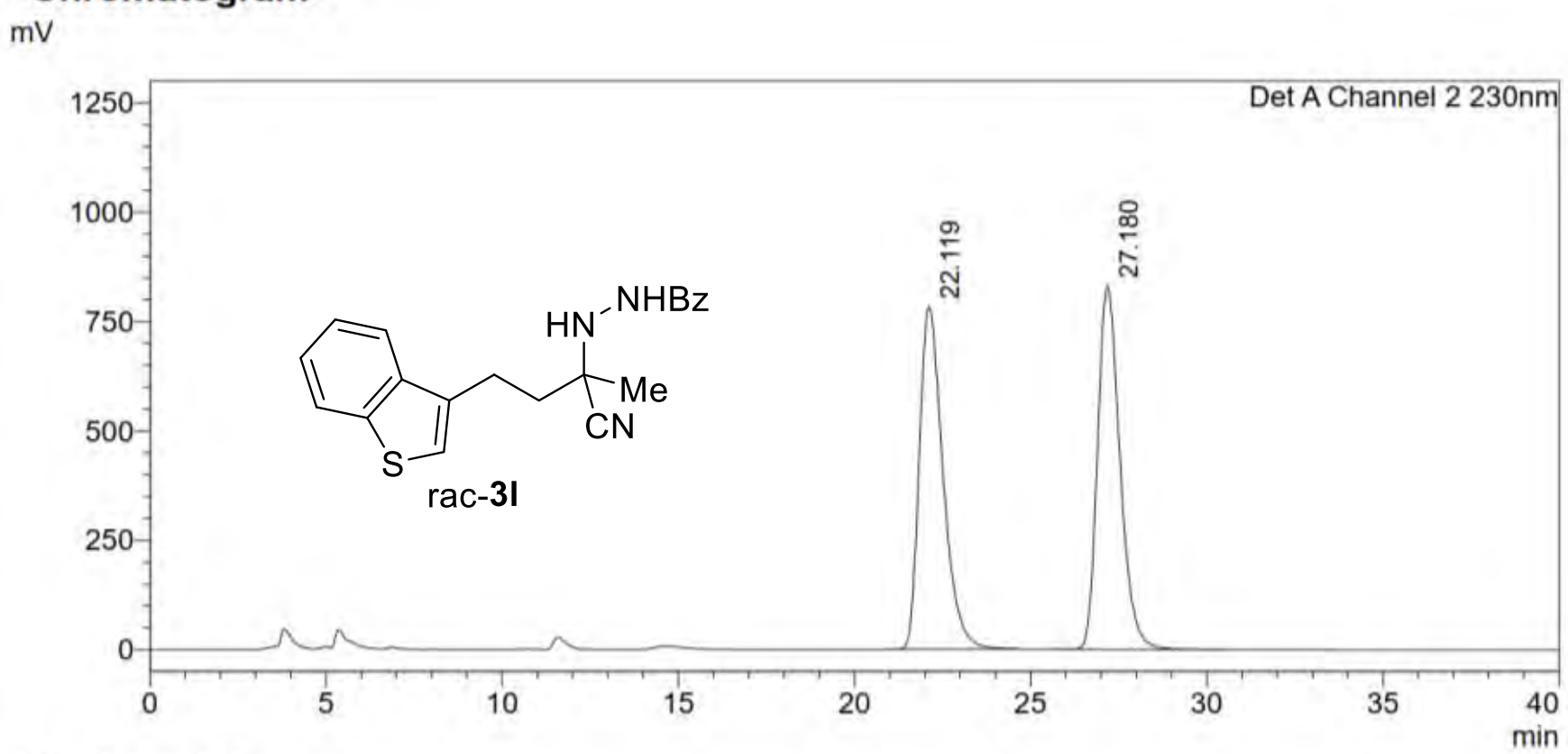

<Peak Table>

Det A Channel 2 230nm

Peak#	Ret. Time	Area	Height	Conc.
1	22.119	36635429	782523	49.932
2	27.180	36734854	830748	50.068
Total		73370284	1613271	

<Sample Information>

Sample Name	: GOY-GAB-17-2-asy-IA-3-8020-1.0-230
Sample ID	:
Data Filename	: GOY-GAB-17-2-asy-IA-3-8020-1.0-230-DATA.lcd
Method Filename	: GY-1.0-OVEN.lcm
Batch Filename	:
Vial #	: 1-1
Injection Volume	: 20 uL
Date Acquired	: 12/19/2024 12:37:57 PM
Date Processed	: 1/22/2025 2:15:14 AM
Sample Type	: Unknown
Acquired by	: System Administrator
Processed by	: System Administrator

<Chromatogram>

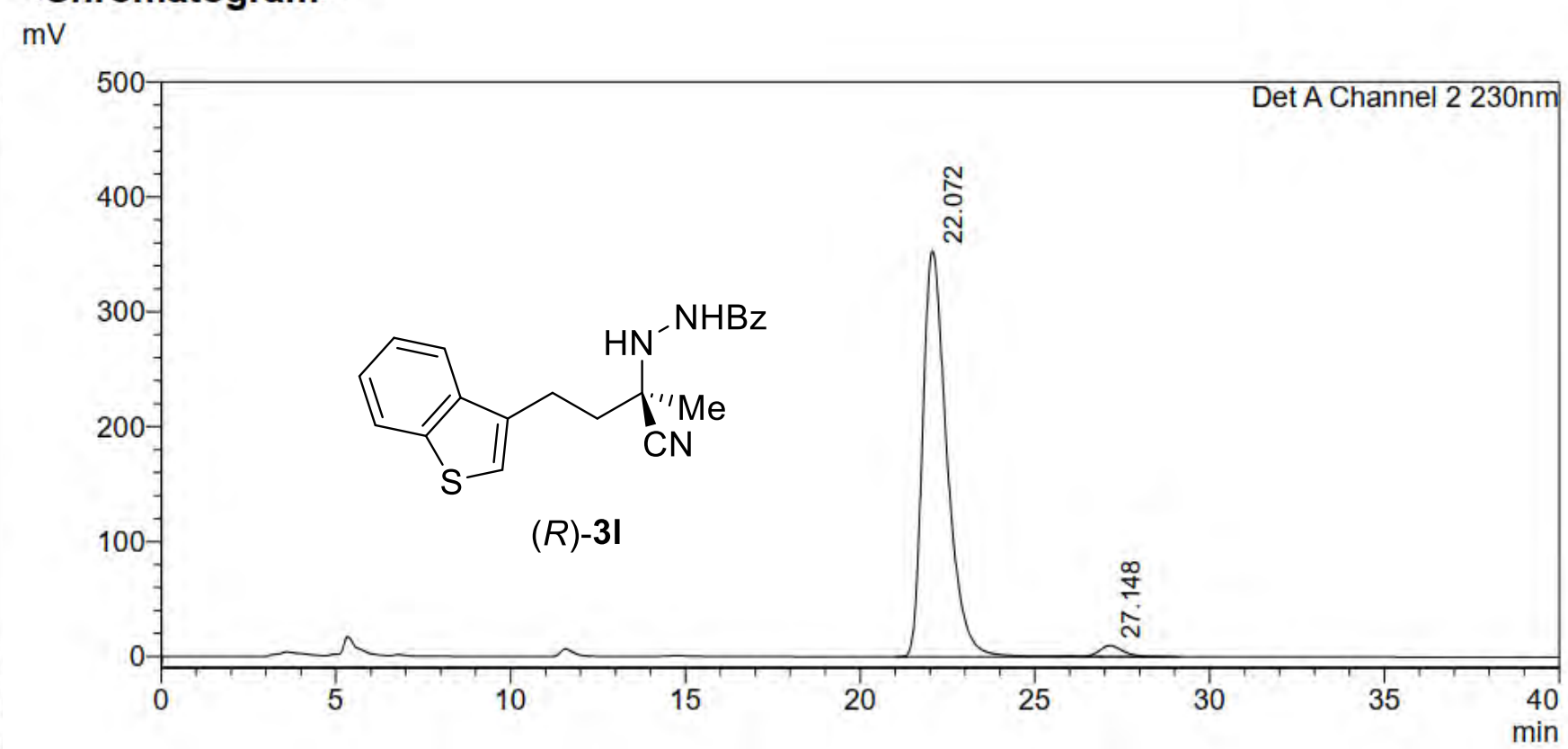

<Peak Table>

Det A Channel 2 230nm

Peak#	Ret. Time	Area	Height	Conc.
1	22.072	17092514	353584	97.518
2	27.148	434973	9424	2.482
Total		17527487	363008	

<Sample Information>

Sample Name	: GOY-GAA-70-7-rac-IF-8515-1.0-230
Sample ID	:
Data Filename	: GOY-GAA-70-7-rac-IF-8515-1.0-230.lcd
Method Filename	: GY-1.0.lcm
Batch Filename	:
Vial #	: 1-1
Sample Type	: Unknown
Injection Volume	: 20 uL
Date Acquired	: 10/8/2024 9:56:51 AM
Acquired by	: System Administrator
Date Processed	: 10/22/2024 9:47:28 PM
Processed by	: System Administrator

<Chromatogram>

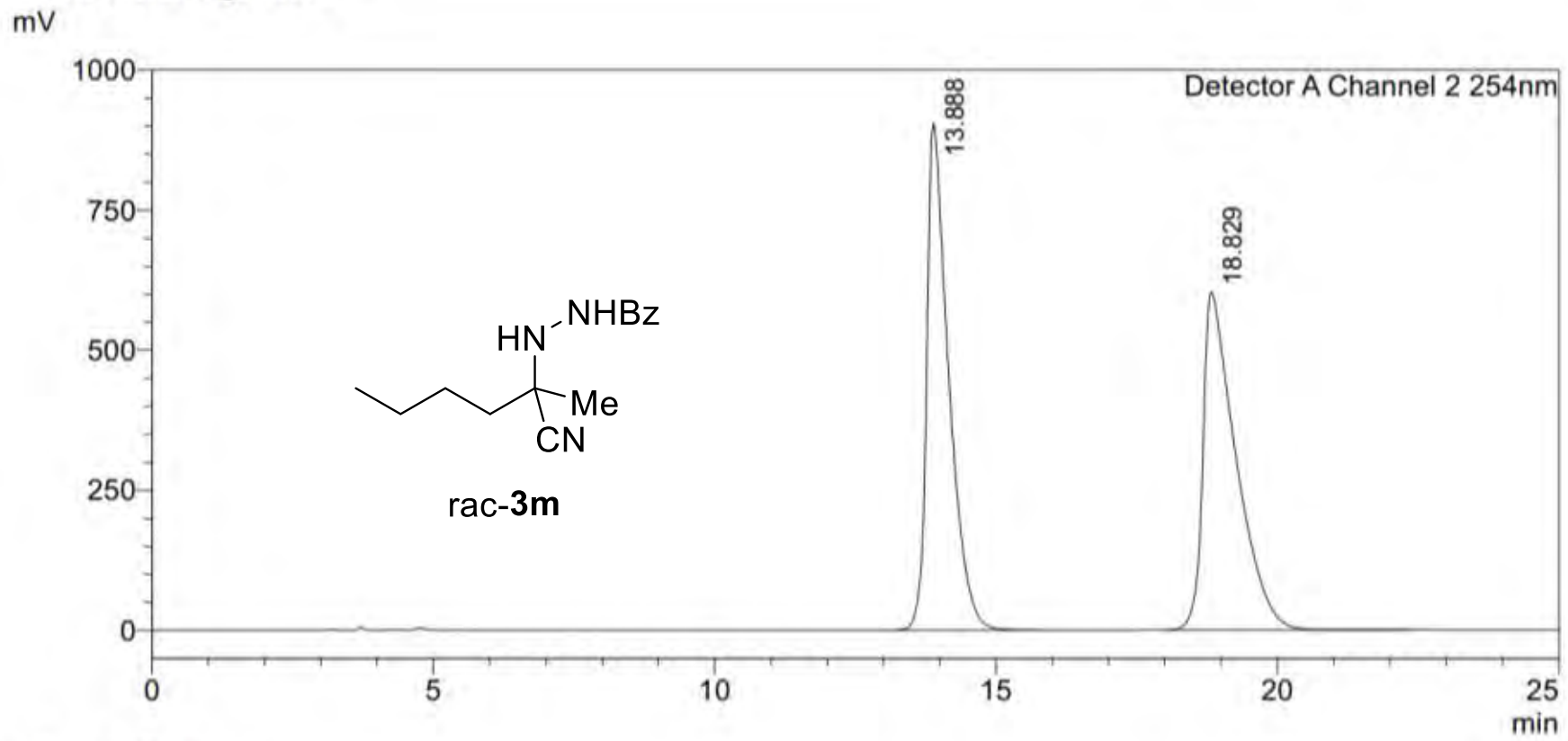

<Peak Table>

Detector A Channel 2 254nm

Peak#	Ret. Time	Area	Height	Conc.
1	13.888	24623014	904265	50.136
2	18.829	24489458	602178	49.864
Total		49112472	1506443	

<Sample Information>

Sample Name	: GOY-GAA-70-7-IF-8515-1.0-230
Sample ID	:
Data Filename	: GOY-GAA-70-7-IF-8515-1.0-230.lcd
Method Filename	: GY-1.0.lcm
Batch Filename	:
Vial #	: 1-1
Sample Type	: Unknown
Injection Volume	: 20 uL
Date Acquired	: 10/8/2024 9:25:51 AM
Acquired by	: System Administrator
Date Processed	: 10/22/2024 9:49:11 PM
Processed by	: System Administrator

<Chromatogram>

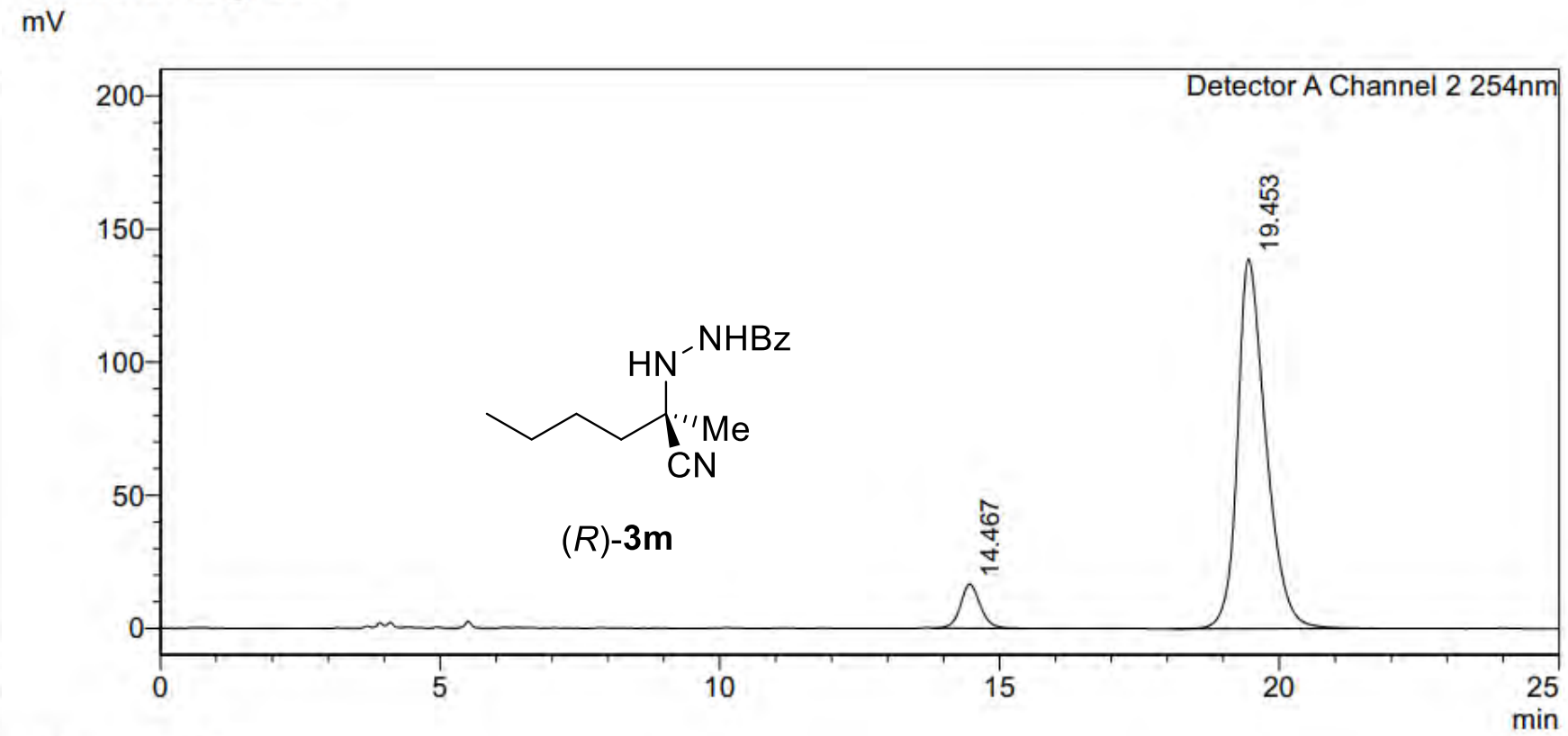

<Peak Table>

Detector A Channel 2 254nm

Peak#	Ret. Time	Area	Height	Conc.
1	14.467	405090	16512	7.631
2	19.453	4903712	138730	92.369
Total		5308802	155242	

<Sample Information>

Sample Name	: GY-IPR-ME--RAC-IA-3-95-5-1.0-230-254		
Sample ID	:		
Data Filename	: GY-IPR-ME--RAC-IA-3-95-5-1.0-230-254.lcd		
Method Filename	: GY-1.0.lcm		
Batch Filename	:		
Vial #	: 1-1	Sample Type	: Unknown
Injection Volume	: 20 uL		
Date Acquired	: 4/16/2026 10:24:04 AM	Acquired by	: System Administrator
Date Processed	: 4/17/2026 12:56:51 AM	Processed by	: System Administrator

<Chromatogram>

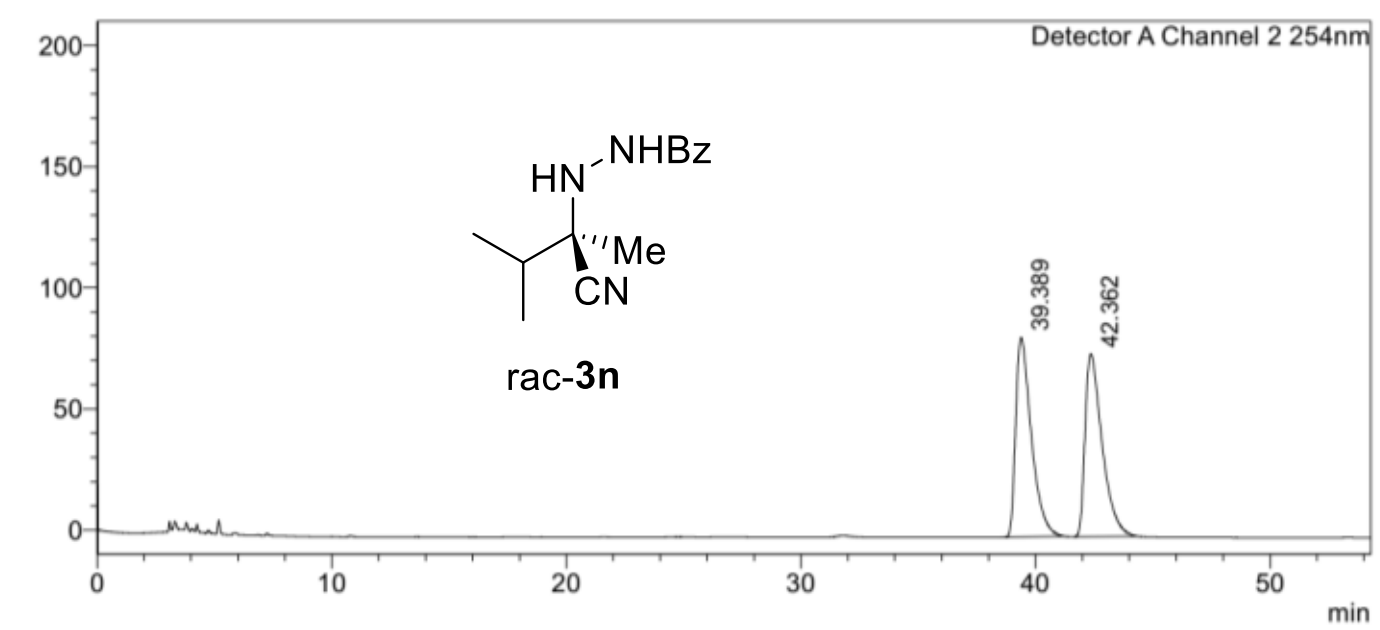

<Peak Table>

Detector A Channel 2 254nm

Peak#	Ret. Time	Area	Height	Conc.
1	39.389	3848584	82413	50.178
2	42.362	3821294	75450	49.822
Total		7669878	157862	

C:\Data\ZHZ\GY-IPR-ME--RAC-IA-3-95-5-1.0-230-254.lcd

<Sample Information>

Sample Name	: GY-IPR-ME--ASY-IA-3-95-5-1.0-230-254		
Sample ID	:		
Data Filename	: GY-IPR-ME--ASY-IA-3-95-5-1.0-230-254.lcd		
Method Filename	: GY-1.0.lcm		
Batch Filename	:		
Vial #	: 1-1	Sample Type	: Unknown
Injection Volume	: 20 uL		
Date Acquired	: 4/16/2026 11:19:39 AM	Acquired by	: System Administrator
Date Processed	: 4/16/2026 12:13:37 PM	Processed by	: System Administrator

<Chromatogram>

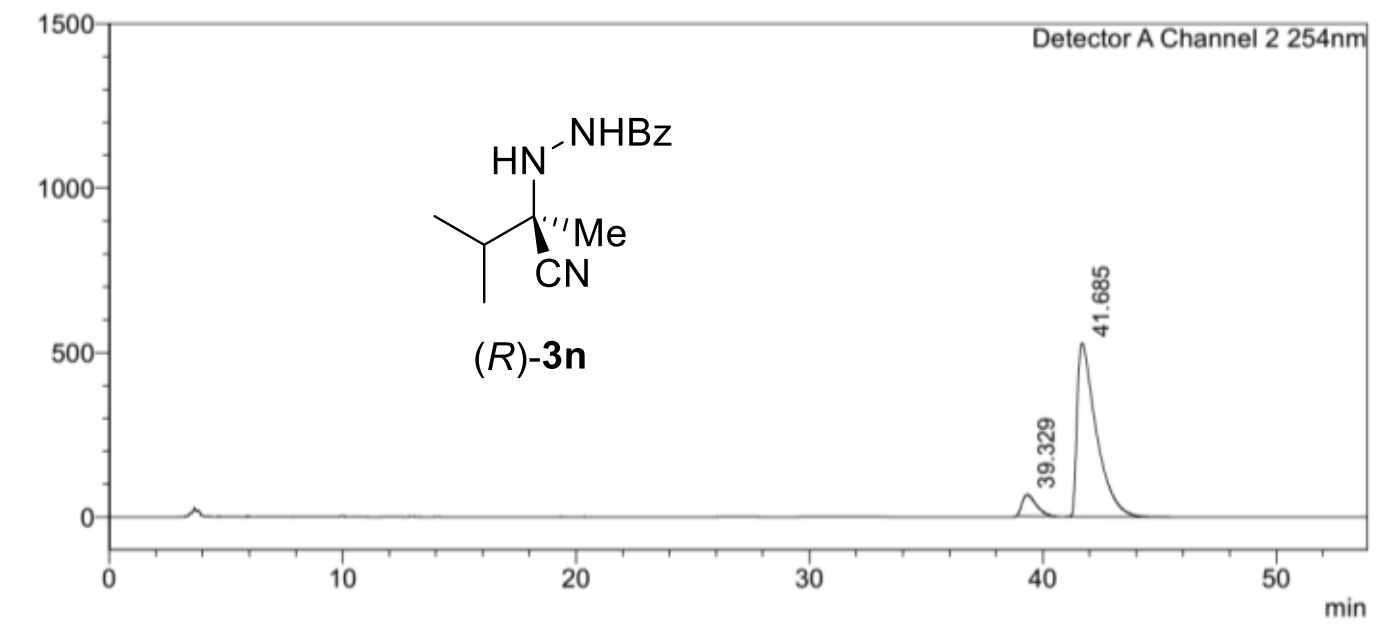

<Peak Table>

Detector A Channel 2 254nm

Peak#	Ret. Time	Area	Height	Conc.
1	39.329	2772791	65157	8.398
2	41.685	30243266	528340	91.602
Total		33016057	593498	

C:\Data\ZHZ\GY-IPR-ME--ASY-IA-3-95-5-1.0-230-254.lcd

<Sample Information>

Sample Name	: GY-TBU-ME-RAC-IE-70-30-1.0-230-254-1-		
Sample ID	:		
Data Filename	: GY-TBU-ME-RAC-IE-70-30-1.0-230-254--1.lcd		
Method Filename	: ZHZ-1.0-254-230.lcm		
Batch Filename	:		
Vial #	: 1-1	Sample Type	: 未知
Injection Volume	: 20 uL		
Date Acquired	: 2026/1/30 21:49:40	Acquired by	: System Administrator
Date Processed	: 2026/1/30 22:03:08	Processed by	: System Administrator

<Chromatogram>

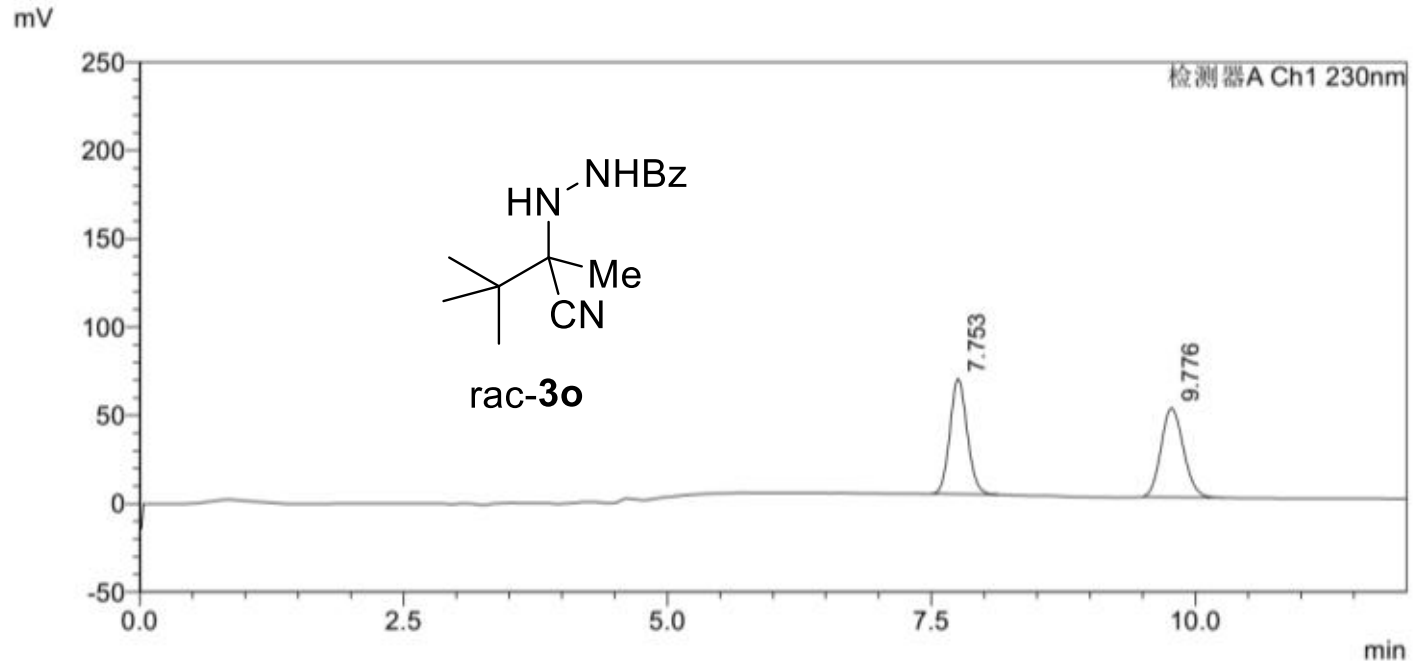

<Peak Table>

检测器A Ch1 230nm

Peak#	Ret. Time	Area	Height	Conc.
1	7.753	747294	65389	50.272
2	9.776	739200	50371	49.728
总计		1486494	115760	

E:\Data\ZHZ\GY-TBU-ME-RAC-IE-70-30-1.0-230-254--1.lcd

<Sample Information>

Sample Name	: GY-tBu-Me-L-Me-ASY-IE-70-30-254-230-1.0		
Sample ID	:		
Data Filename	: GY-tBu-Me-L-Me-ASY-IE-70-30-254-230-1.0.lcd		
Method Filename	: ZHZ-1.0-254-230.lcm		
Batch Filename	:		
Vial #	: 1-1	Sample Type	: 未知
Injection Volume	: 20 uL		
Date Acquired	: 2026/1/30 20:43:46	Acquired by	: System Administrator
Date Processed	: 2026/1/30 21:08:04	Processed by	: System Administrator

<Chromatogram>

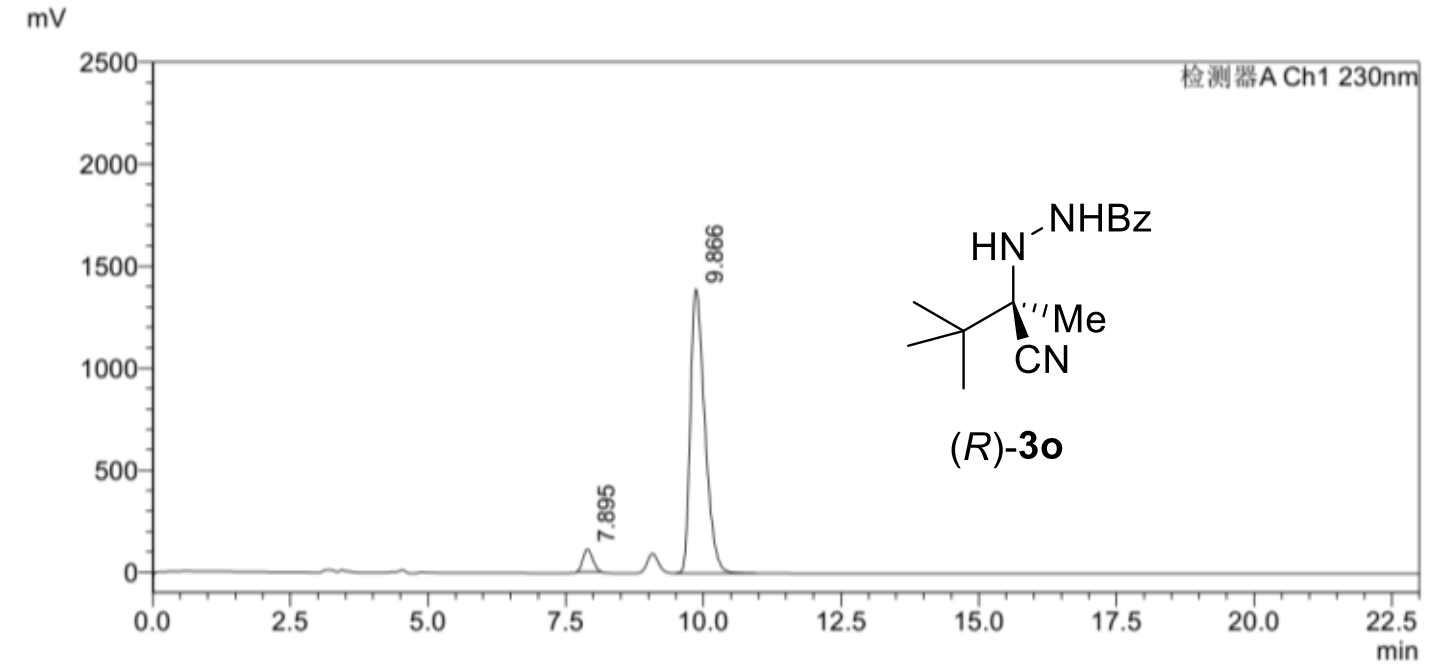

<Peak Table>

检测器A Ch1 230nm

Peak#	Ret. Time	Area	Height	Conc.
1	7.895	1375741	111020	5.132
2	9.866	25430406	1390702	94.868
总计		26806147	1501722	

E:\Data\ZHZ\GY-tBu-Me-L-Me-ASY-IE-70-30-254-230-1.0.lcd

<Sample Information>

Sample Name : ZHZ-ZAD-14-RAC--IA-80-20-1.0-254-230
Sample ID :
Data Filename : ZHZ-ZAD-14-RAC-IA-80-20-1.0-254-230.lcd
Method Filename : ZHZ-1.0-254-230.lcm
Batch Filename :
Vial # : 1-1 Sample Type : 未知
Injection Volume : 20 uL
Date Acquired : 2026/3/31 0:51:19 Acquired by : System Administrator
Date Processed : 2026/3/31 1:09:13 Processed by : System Administrator

<Chromatogram>

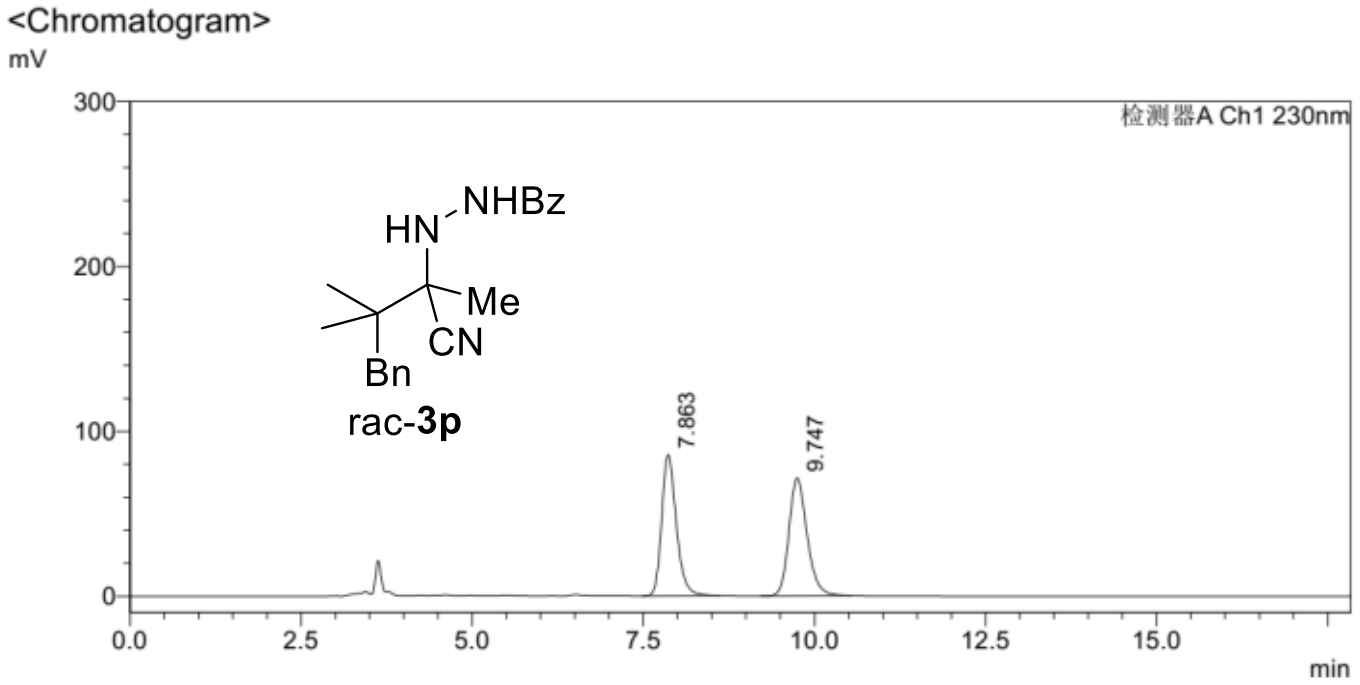

<Peak Table>

检测器A Ch1 230nm

Peak#	Ret. Time	Area	Height	Conc.
1	7.863	1278023	85693	49.204
2	9.747	1319358	71411	50.796
总计		2597381	157104	

E:\Data\ZHZ\ZHZ-ZAD-14-RAC-IA-80-20-1.0-254-230.lcd

<Sample Information>

Sample Name : ZHZ-ZAD-14-ASY--IA-80-20-1.0-254-230
Sample ID :
Data Filename : ZHZ-ZAD-14-ASY--IA-80-20-1.0-254-230.lcd
Method Filename : ZHZ-1.0-254-230.lcm
Batch Filename :
Vial # : 1-1 Sample Type : 未知
Injection Volume : 20 uL
Date Acquired : 2026/3/31 0:35:38 Acquired by : System Administrator
Date Processed : 2026/3/31 0:48:17 Processed by : System Administrator

<Chromatogram>

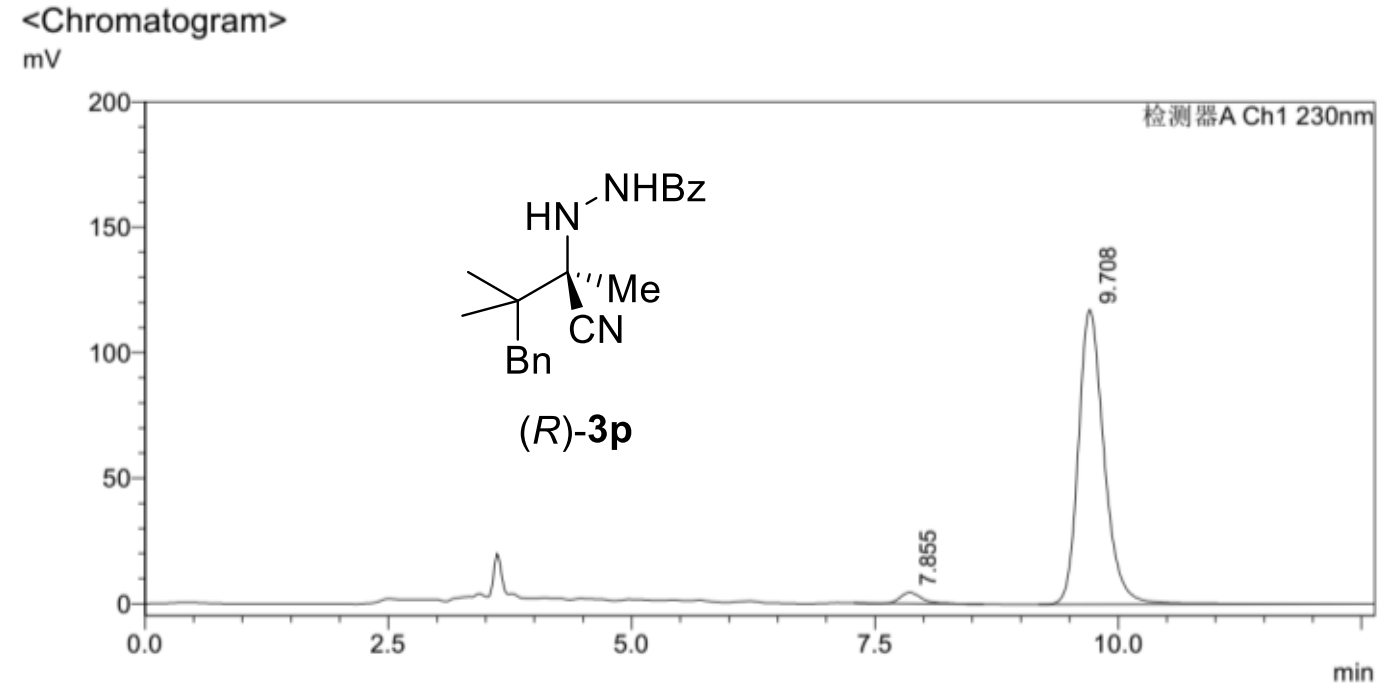

<Peak Table>

检测器A Ch1 230nm

Peak#	Ret. Time	Area	Height	Conc.
1	7.855	63612	4364	2.857
2	9.708	2163163	117357	97.143
总计		2226775	121720	

E:\Data\ZHZ\ZHZ-ZAD-14-ASY--IA-80-20-1.0-254-230.lcd

<Sample Information>

Sample Name	: ZHZ-ZAD-60-RAC-IE-80-20-230-254-1.0		
Sample ID	:		
Data Filename	: ZHZ-ZAD-60-RAC-IE-80-20-230-254-1.0.lcd		
Method Filename	: GY-1.0.lcm		
Batch Filename	:		
Vial #	: 1-1	Sample Type	: Unknown
Injection Volume	: 20 uL		
Date Acquired	: 4/14/2026 11:39:52 PM	Acquired by	: System Administrator
Date Processed	: 4/15/2026 12:04:52 AM	Processed by	: System Administrator

<Chromatogram>

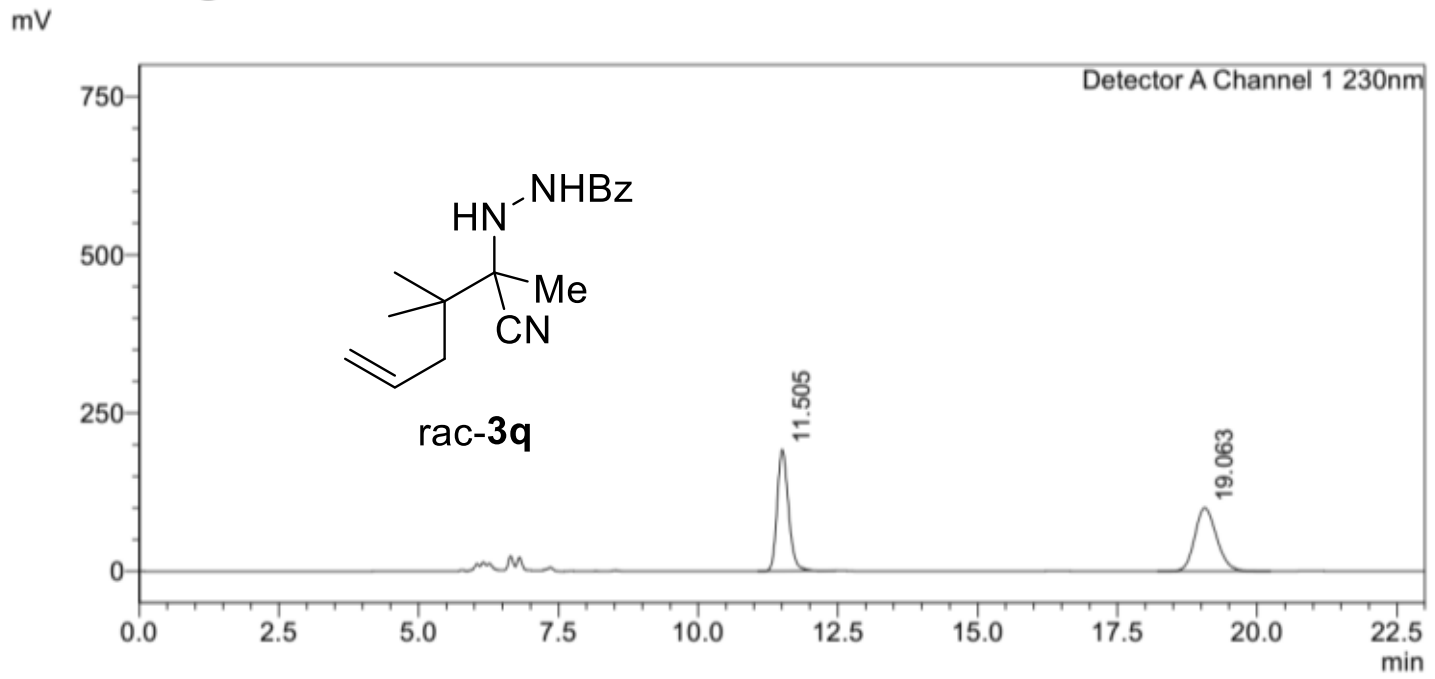

<Peak Table>

Detector A Channel 1 230nm

Peak#	Ret. Time	Area	Height	Conc.
1	11.505	2693657	191430	49.774
2	19.063	2718084	99818	50.226
Total		5411741	291249	

C:\Data\ZHZ\ZHZ-ZAD-60-RAC-IE-80-20-230-254-1.0.lcd

<Sample Information>

Sample Name	: ZHZ-ZAD-60-ASY-IE-80-20-230-254-1.0		
Sample ID	:		
Data Filename	: ZHZ-ZAD-60-ASY-IE-80-20-230-254-1.0.lcd		
Method Filename	: GY-1.0.lcm		
Batch Filename	:		
Vial #	: 1-1	Sample Type	: Unknown
Injection Volume	: 20 uL		
Date Acquired	: 4/15/2026 12:06:49 AM	Acquired by	: System Administrator
Date Processed	: 4/15/2026 12:34:05 AM	Processed by	: System Administrator

<Chromatogram>

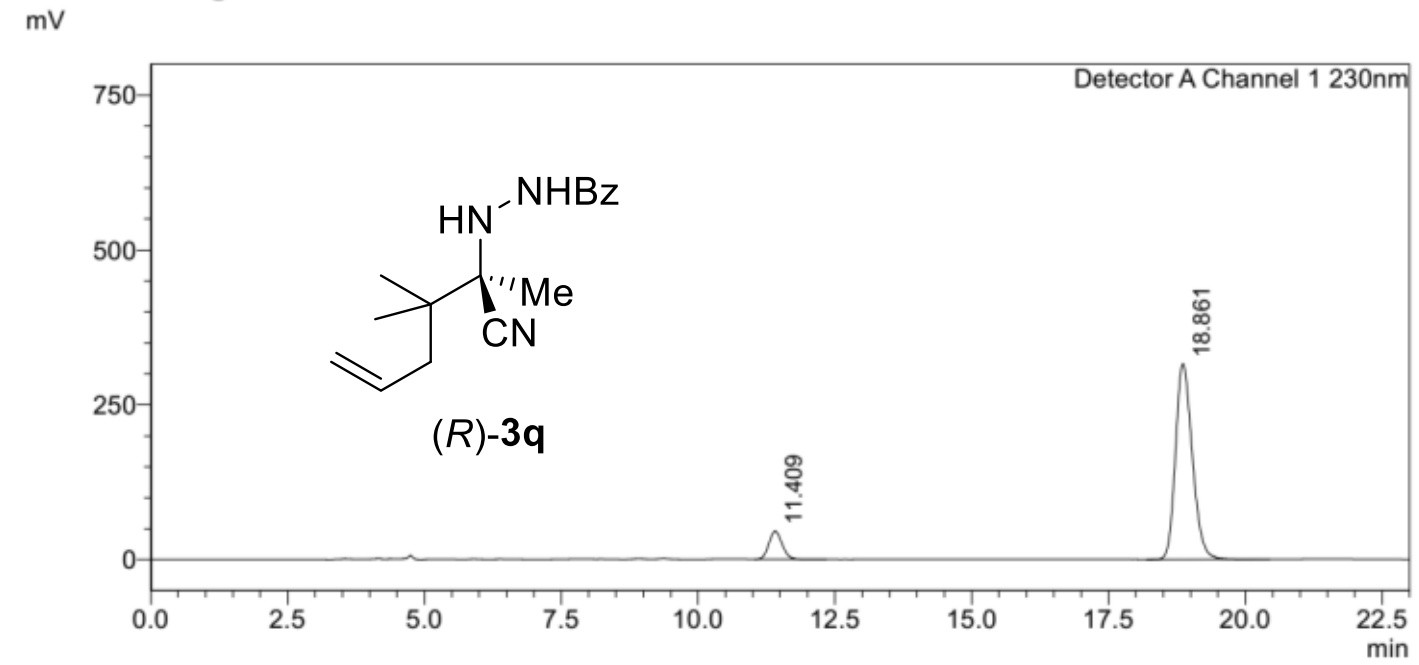

<Peak Table>

Detector A Channel 1 230nm

Peak#	Ret. Time	Area	Height	Conc.
1	11.409	756949	45159	10.053
2	18.861	6772606	315892	89.947
Total		7529555	361051	

C:\Data\ZHZ\ZHZ-ZAD-60-ASY-IE-80-20-230-254-1.0.lcd

<Sample Information>

Sample Name	: ZHZ-ZAD-TBU-ET-RAC-IE-70-30-1.0-230-254		
Sample ID	:		
Data Filename	: ZHZ-ZAD-TBU-ET-RAC-IE-70-30-1.0-230-254.lcd		
Method Filename	: GY-1.0.lcm		
Batch Filename	:		
Vial #	: 1-1	Sample Type	: Unknown
Injection Volume	: 20 uL		
Date Acquired	: 4/16/2026 4:59:51 PM	Acquired by	: System Administrator
Date Processed	: 4/16/2026 5:18:11 PM	Processed by	: System Administrator

<Chromatogram>

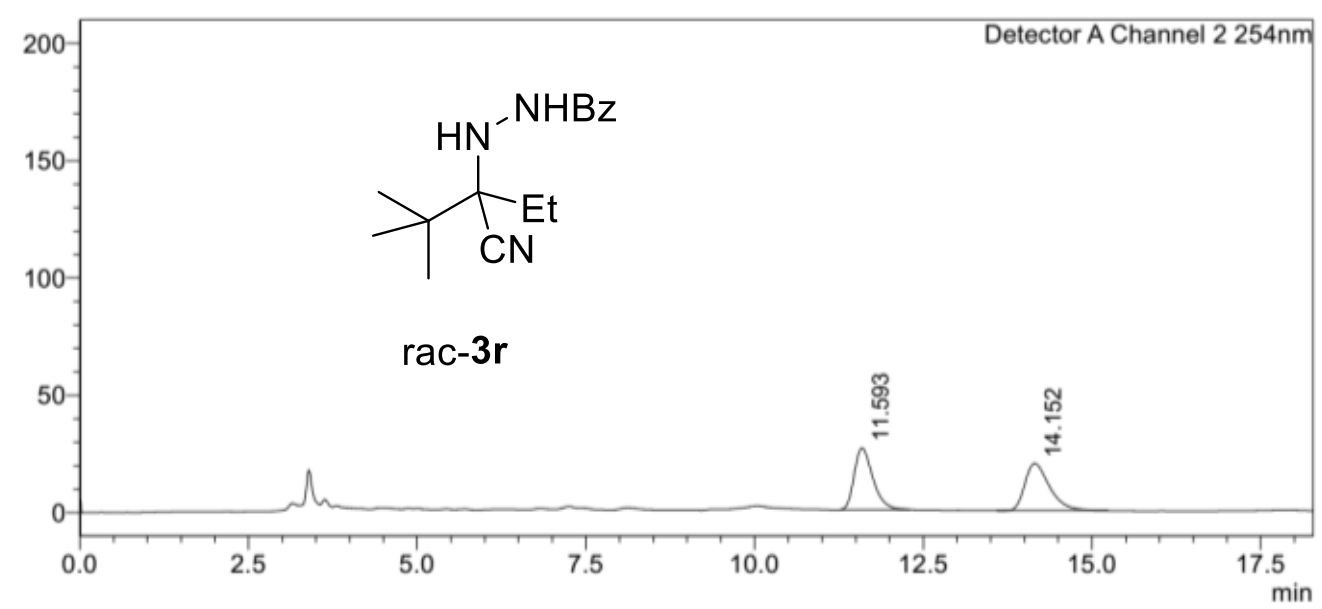

<Peak Table>

Detector A Channel 2 254nm

Peak#	Ret. Time	Area	Height	Conc.
1	11.593	505540	26262	49.794
2	14.152	509714	20030	50.206
Total		1015254	46292	

C:\Data\ZHZ\ZHZ-ZAD-TBU-ET-RAC-IE-70-30-1.0-230-254.lcd

<Sample Information>

Sample Name	: ZHZ-ZAD-TBU-ET-ASY-IE-70-30-1.0-230-254		
Sample ID	:		
Data Filename	: ZHZ-ZAD-TBU-ET-ASY-IE-70-30-1.0-230-254.lcd		
Method Filename	: GY-1.0.lcm		
Batch Filename	:		
Vial #	: 1-1	Sample Type	: Unknown
Injection Volume	: 20 uL		
Date Acquired	: 4/16/2026 5:37:45 PM	Acquired by	: System Administrator
Date Processed	: 4/16/2026 6:07:58 PM	Processed by	: System Administrator

<Chromatogram>

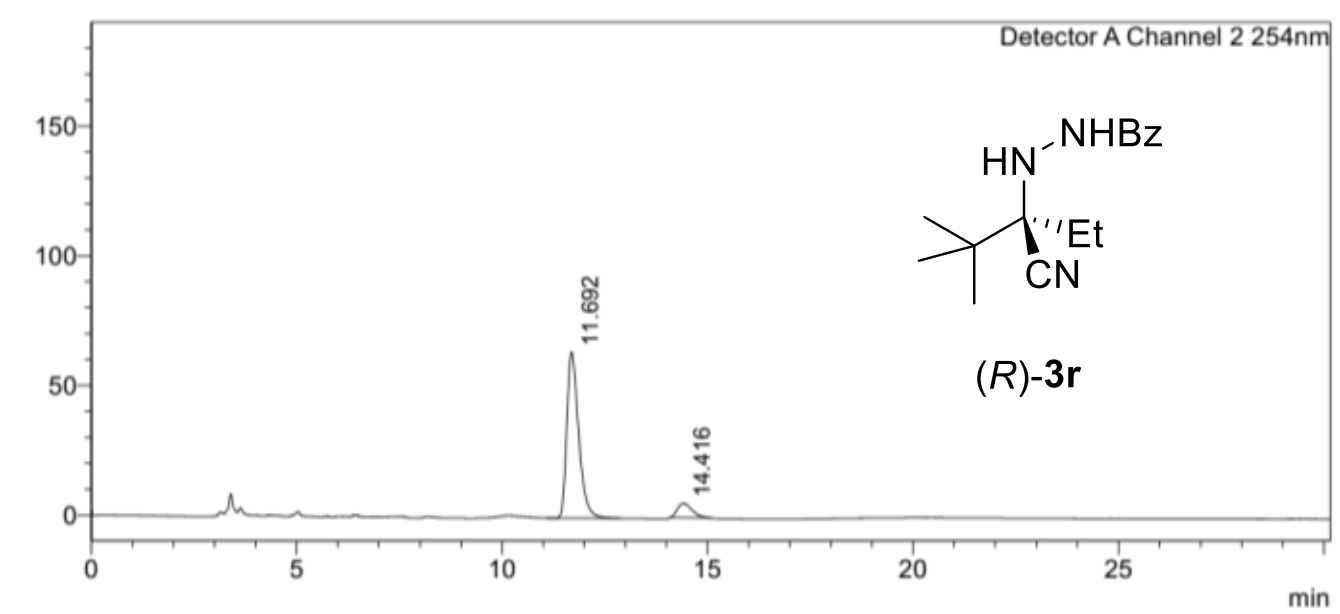

<Peak Table>

Detector A Channel 2 254nm

Peak#	Ret. Time	Area	Height	Conc.
1	11.692	1286610	64002	90.247
2	14.416	139049	5579	9.753
Total		1425660	69581	

C:\Data\ZHZ\ZHZ-ZAD-TBU-ET-ASY-IE-70-30-1.0-230-254.lcd

<Sample Information>

Sample Name	: ZHZ-ZAD-42-RAC-IE-70-30-230-254		
Sample ID	:		
Data Filename	: ZHZ-ZAD-42-RAC-IE-70-30-230-254.lcd		
Method Filename	: GY-1.0.lcm		
Batch Filename	:		
Vial #	: 1-1	Sample Type	: Unknown
Injection Volume	: 20 uL		
Date Acquired	: 4/16/2026 12:33:51 AM	Acquired by	: System Administrator
Date Processed	: 4/16/2026 12:53:36 AM	Processed by	: System Administrator

<Chromatogram>

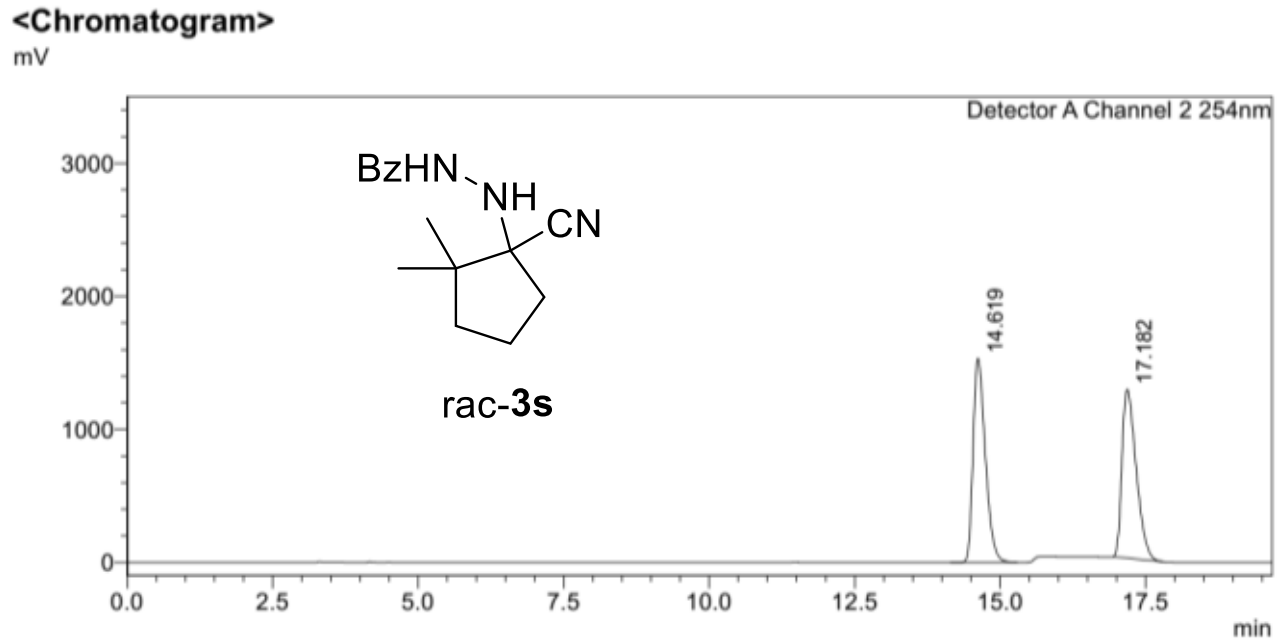

<Peak Table>

Detector A Channel 2 254nm

Peak#	Ret. Time	Area	Height	Conc.
1	14.619	22435156	1531487	51.065
2	17.182	21499012	1266725	48.935
Total		43934169	2798212	

C:\Data\ZHZ\ZHZ-ZAD-42-RAC-IE-70-30-230-254.lcd

<Sample Information>

Sample Name	: zhz-zad-HWJ--ASY-IE-70-30-230-210-1.0		
Sample ID	:		
Data Filename	: zhz-zad-HWJ--ASY-IE-70-30-230-210-1.0.lcd		
Method Filename	: GY-1.0.lcm		
Batch Filename	:		
Vial #	: 1-1	Sample Type	: Unknown
Injection Volume	: 20 uL		
Date Acquired	: 4/7/2026 8:59:43 PM	Acquired by	: System Administrator
Date Processed	: 4/7/2026 9:32:15 PM	Processed by	: System Administrator

<Chromatogram>

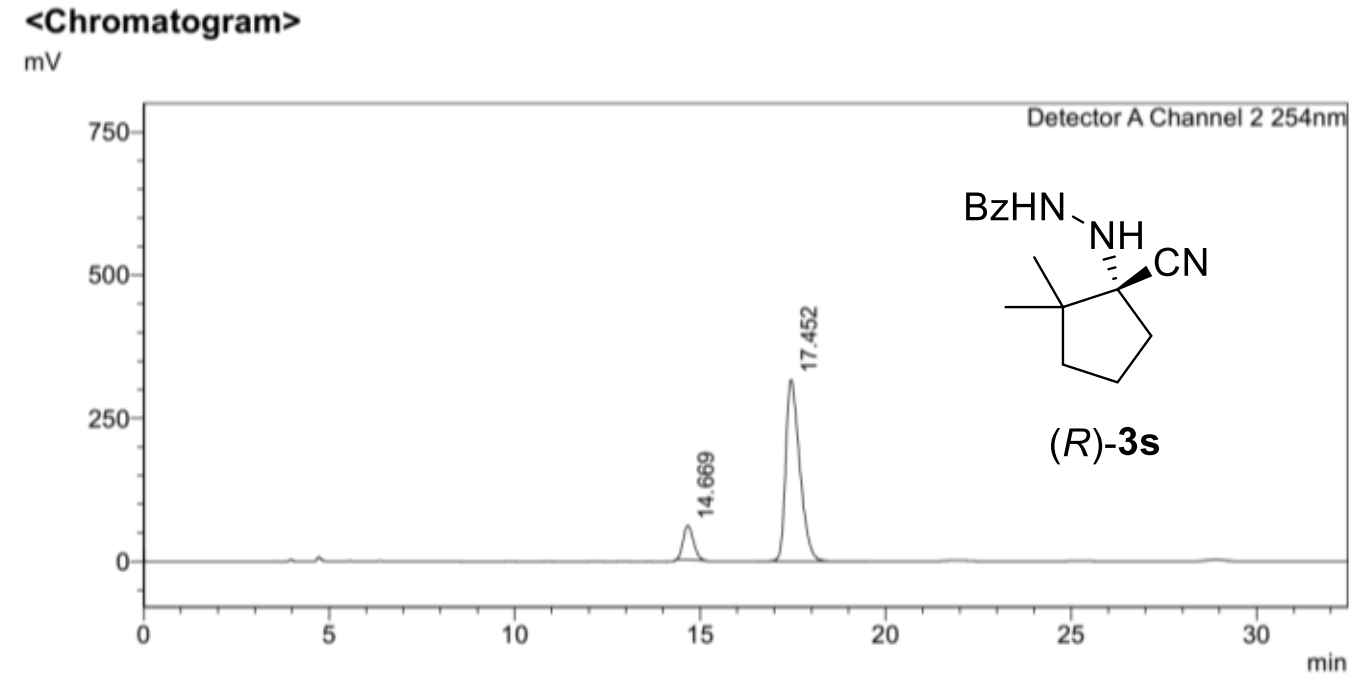

<Peak Table>

Detector A Channel 2 254nm

Peak#	Ret. Time	Area	Height	Conc.
1	14.669	1160041	60096	12.191
2	17.452	8355316	317299	87.809
Total		9515357	377395	

C:\Data\ZHZ\zhz-zad-HWJ--ASY-IE-70-30-230-210-1.0.lcd

<Sample Information>

Sample Name	: GOY-GAA-113-rac-IA-3-8515-1.0-230-DATA		
Sample ID	:		
Data Filename	: GOY-GAA-113-rac-IA-3-8515-1.0-230-DATA.lcd		
Method Filename	: GY-1.0.lcm		
Batch Filename	:		
Vial #	: 1-1	Sample Type	: Unknown
Injection Volume	: 20 uL		
Date Acquired	: 11/12/2024 10:02:12 AM	Acquired by	: System Administrator
Date Processed	: 2/8/2025 9:15:33 AM	Processed by	: System Administrator

<Chromatogram>

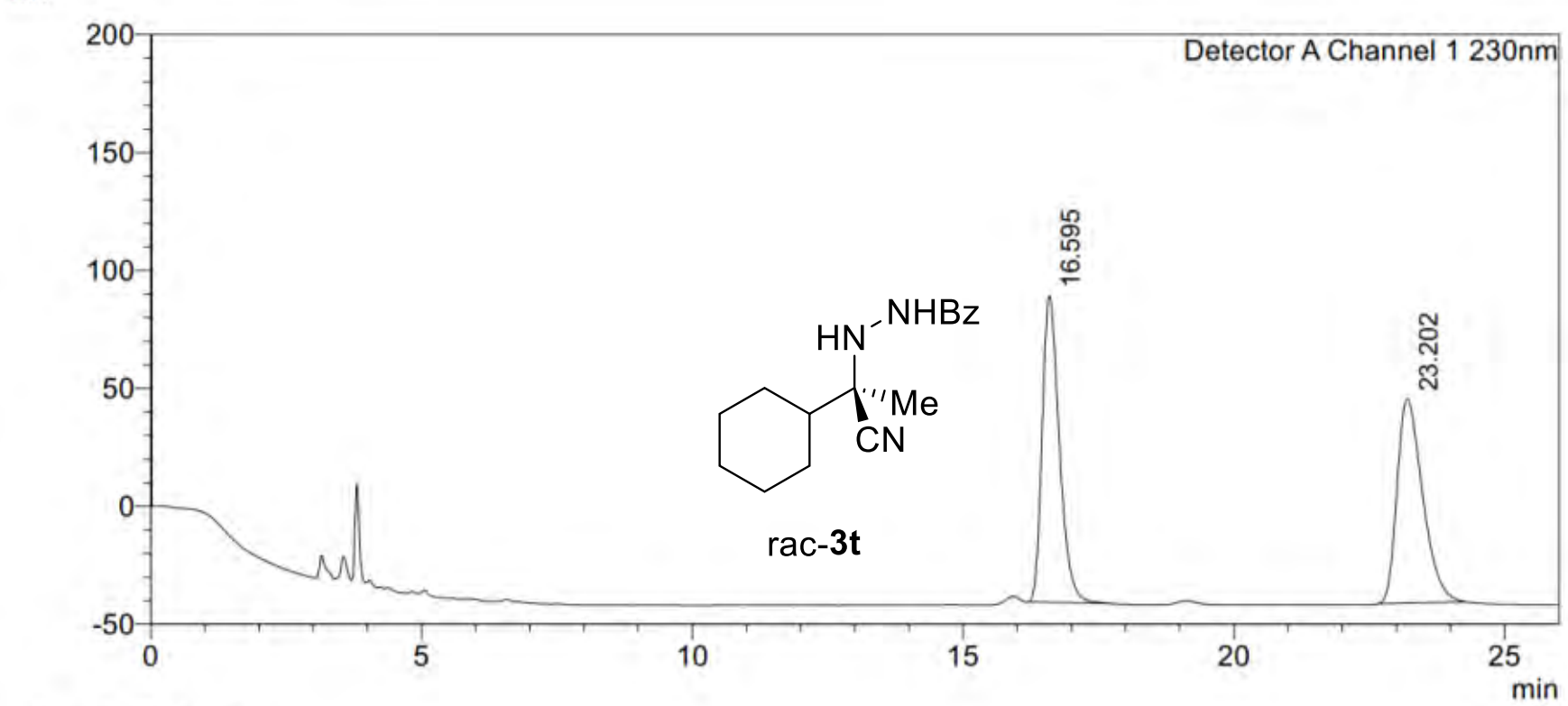

<Peak Table>

Detector A Channel 1 230nm

Peak#	Ret. Time	Area	Height	Conc.
1	16.595	2877459	130224	50.242
2	23.202	2849712	86702	49.758
Total		5727171	216926	

<Sample Information>

Sample Name	: GOY-GAA-113-asy-IA-3-8515-1.0-230-DATA		
Sample ID	:		
Data Filename	: GOY-GAA-113-asy-IA-3-8515-1.0-230-DATA.lcd		
Method Filename	: GY-1.0.lcm		
Batch Filename	:		
Vial #	: 1-1	Sample Type	: Unknown
Injection Volume	: 20 uL		
Date Acquired	: 11/12/2024 10:30:27 AM	Acquired by	: System Administrator
Date Processed	: 11/12/2024 10:58:57 AM	Processed by	: System Administrator

<Chromatogram>

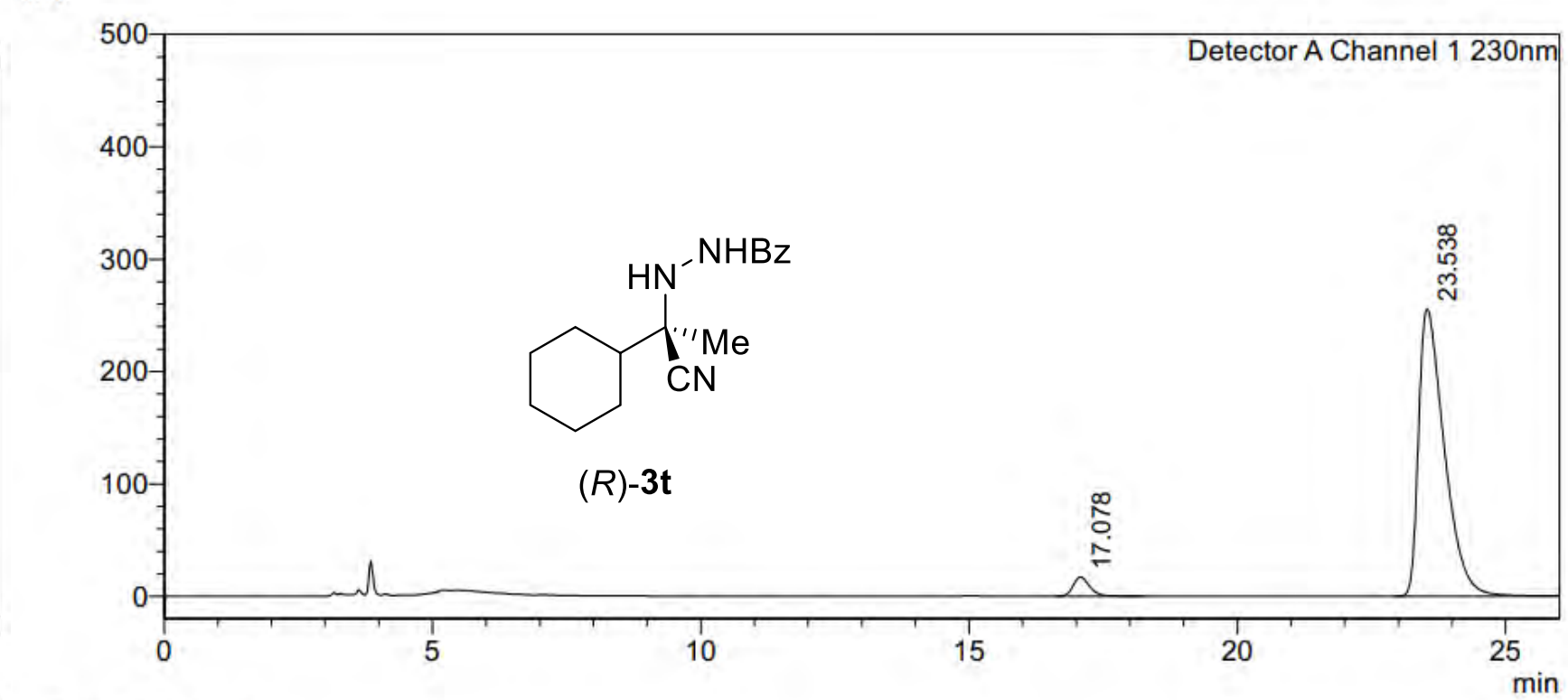

<Peak Table>

Detector A Channel 1 230nm

Peak#	Ret. Time	Area	Height	Conc.
1	17.078	370128	17173	4.156
2	23.538	8535456	255864	95.844
Total		8905584	273037	

<Sample Information>

Sample Name	: GOY-GG-31-3-rac-ia-8515-1.0-230		
Sample ID	:		
Data Filename	: GOY-GG-31-3-rac-ia-8515-1.0-231.lcd		
Method Filename	: GY-1.0.lcm		
Batch Filename	:		
Vial #	: 1-1	Sample Type	: Unknown
Injection Volume	: 20 uL		
Date Acquired	: 10/12/2020 2:16:42 PM	Acquired by	: System Administrator
Date Processed	: 10/12/2020 3:19:25 PM	Processed by	: System Administrator

<Chromatogram>

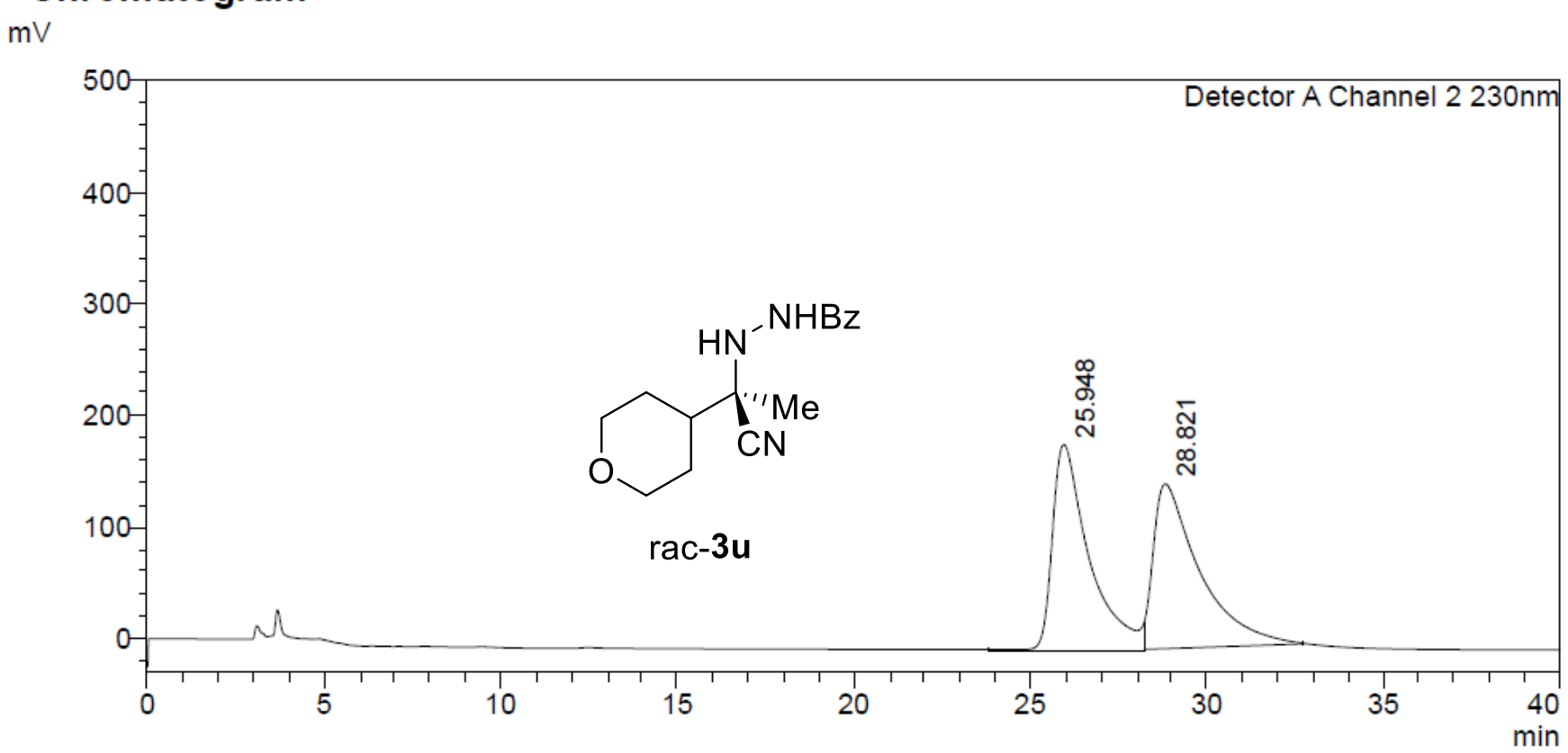

<Peak Table>

Detector A Channel 2 230nm

Peak#	Ret. Time	Area	Height	Conc.
1	25.948	13512531	184949	50.120
2	28.821	13447939	147751	49.880
Total		26960470	332700	

<Sample Information>

Sample Name	: GOY-GG-31-3-asy-ia-8515-1.0-230		
Sample ID	:		
Data Filename	: GOY-GG-31-3-asy-ia-8515-1.0-231.lcd		
Method Filename	: GY-1.0.lcm		
Batch Filename	:		
Vial #	: 1-1	Sample Type	: Unknown
Injection Volume	: 20 uL		
Date Acquired	: 10/12/2020 3:17:57 PM	Acquired by	: System Administrator
Date Processed	: 10/12/2020 4:05:56 PM	Processed by	: System Administrator

<Chromatogram>

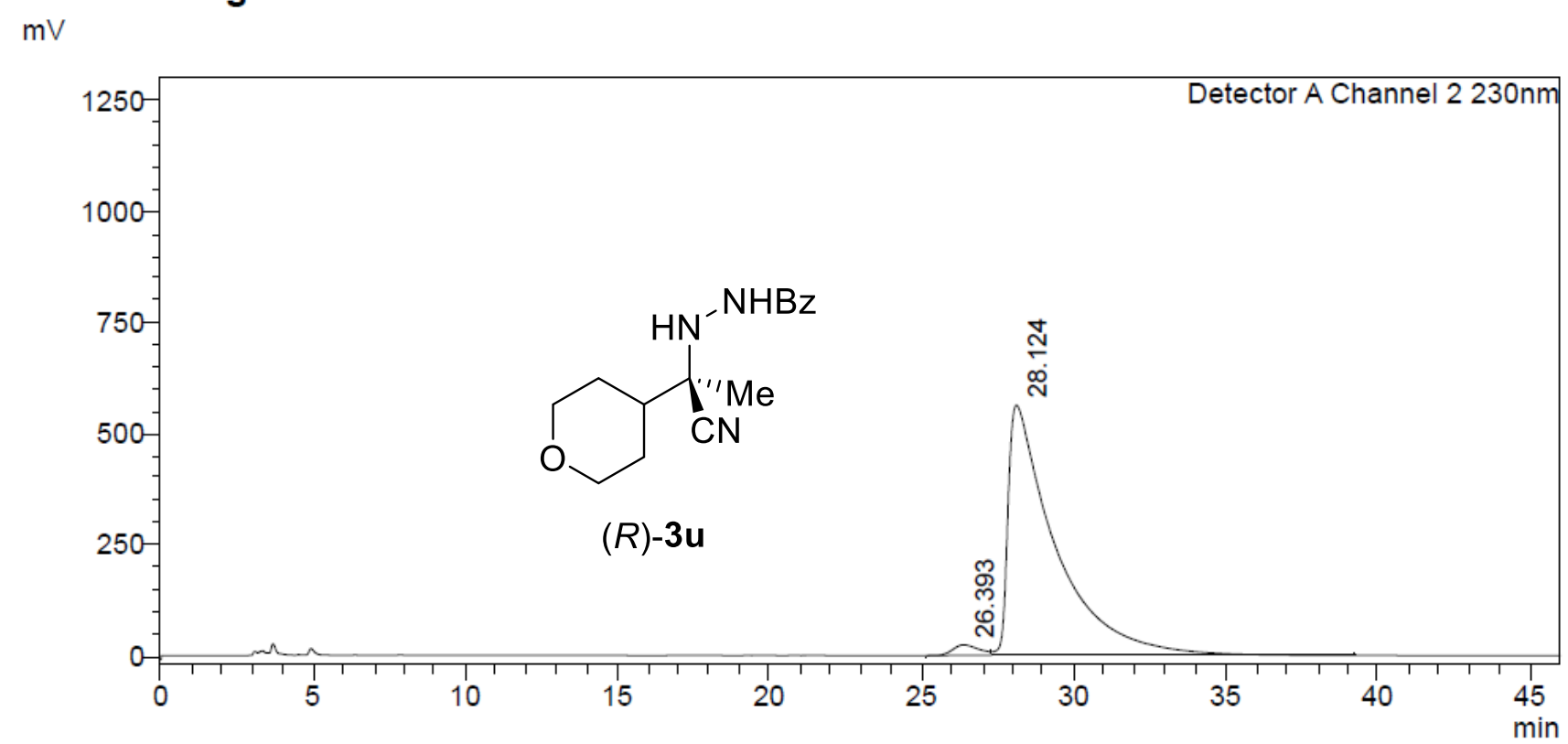

<Peak Table>

Detector A Channel 2 230nm

Peak#	Ret. Time	Area	Height	Conc.
1	26.393	1420922	23516	2.278
2	28.124	60956884	563604	97.722
Total		62377806	587120	

<Sample Information>

Sample Name	: GOY-GO-143-rac-IA-7030-230-1.0
Sample ID	:
Data Filename	: GOY-GP-143-rac-IA-7030-230-1.1-DATA.lcd
Method Filename	: GY-1.0.lcm
Batch Filename	:
Vial #	: 1-1
Sample Type	: Unknown
Injection Volume	: 20 uL
Date Acquired	: 8/17/2022 4:07:07 PM
Acquired by	: System Administrator
Date Processed	: 8/18/2022 11:11:47 AM
Processed by	: System Administrator

<Chromatogram>

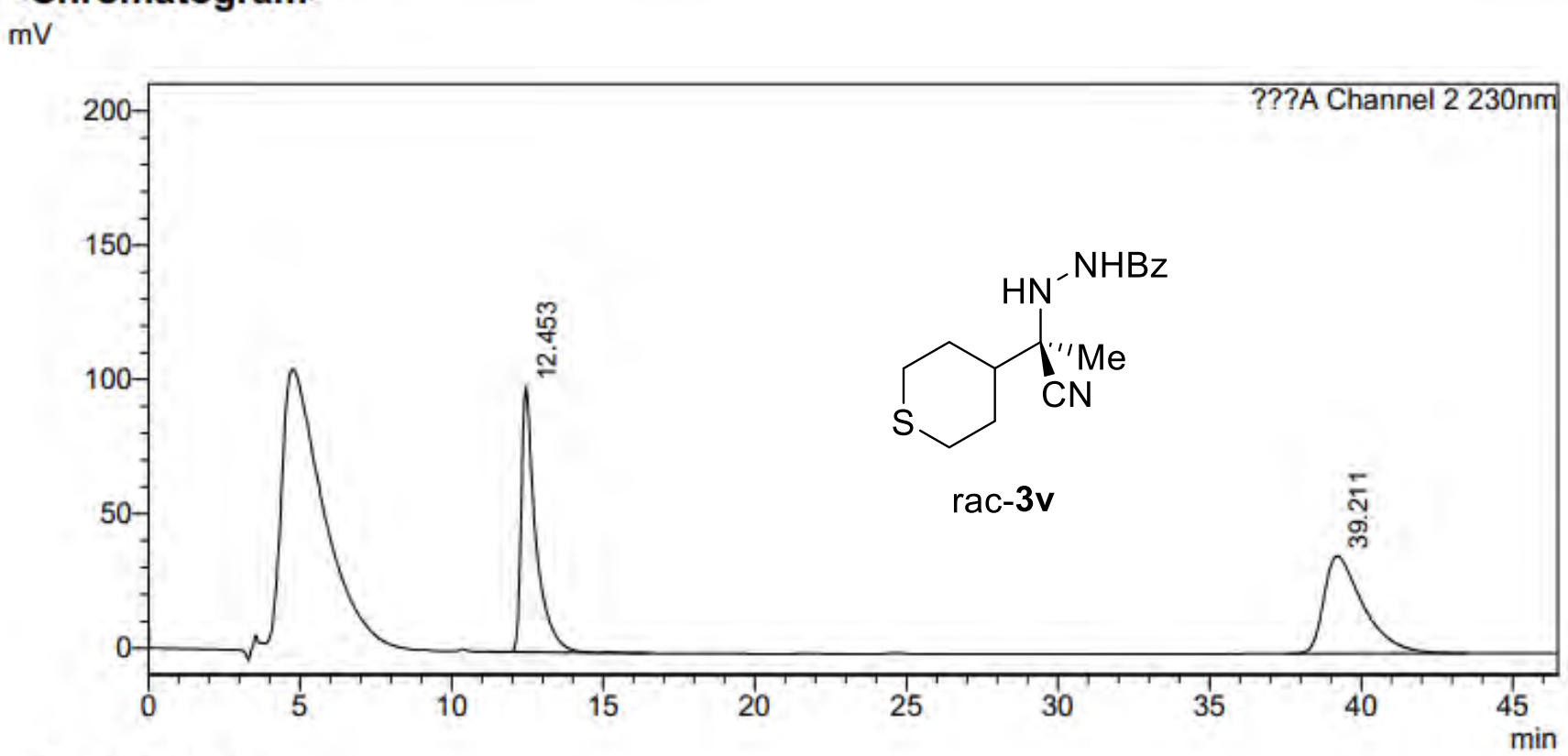

<Peak Table>

???A Channel 2 230nm

Peak#	Ret. Time	Area	Height	Conc.
1	12.453	3274892	98420	49.993
2	39.211	3275840	36269	50.007
Total		6550731	134689	

<Sample Information>

Sample Name	: GOY-GO-143-asy-IA-7030-230-1.0
Sample ID	:
Data Filename	: GOY-GP-143-asy-IA-7030-230-1.0-DATA.lcd
Method Filename	: GY-1.0.lcm
Batch Filename	:
Vial #	: 1-1
Sample Type	: Unknown
Injection Volume	: 20 uL
Date Acquired	: 8/17/2022 4:54:34 PM
Acquired by	: System Administrator
Date Processed	: 8/18/2022 11:10:40 AM
Processed by	: System Administrator

<Chromatogram>

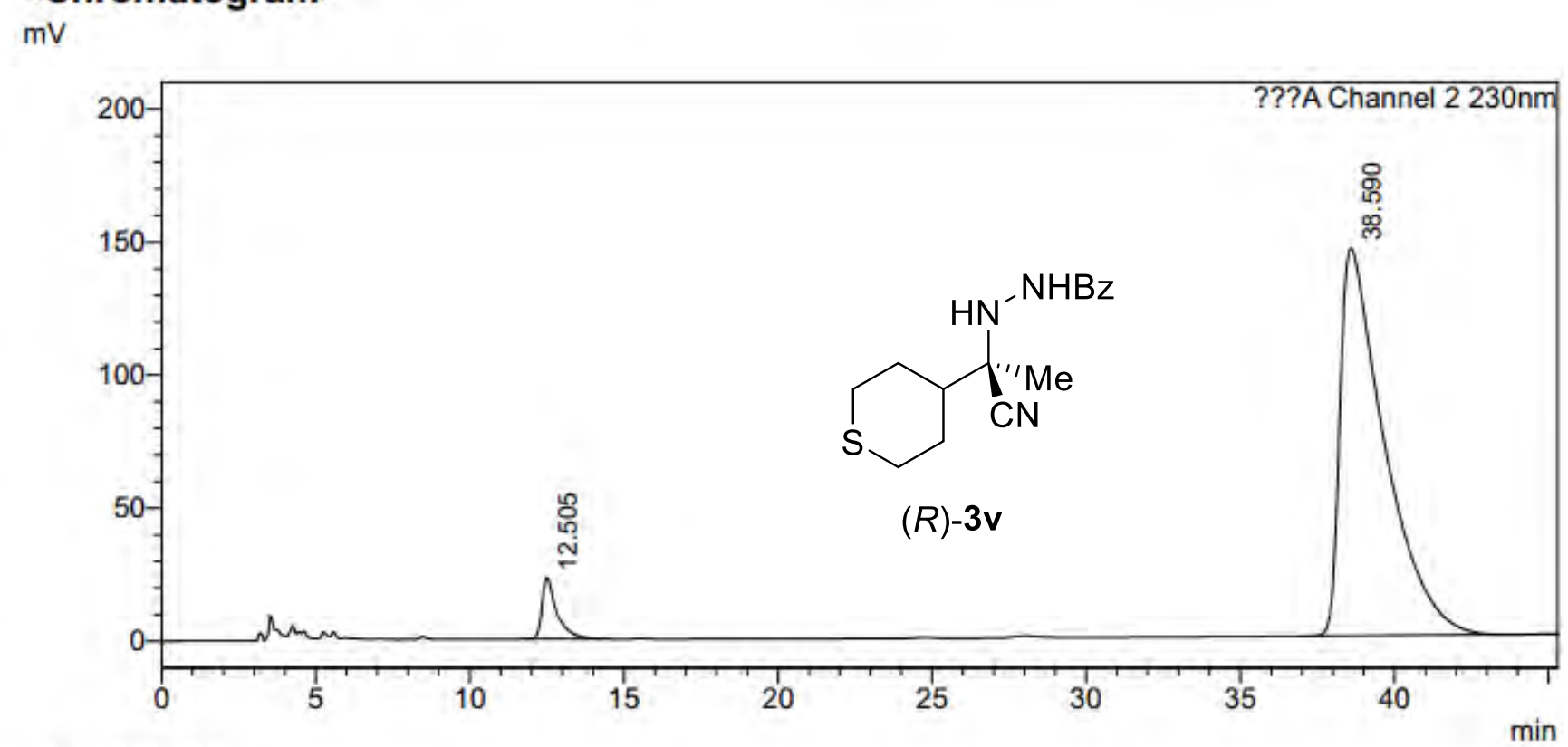

<Peak Table>

???A Channel 2 230nm

Peak#	Ret. Time	Area	Height	Conc.
1	12.505	763591	23004	4.971
2	38.590	14597626	145627	95.029
Total		15361216	168631	

<Sample Information>

Sample Name	: GOY-GG-99-1-rac-odh-9010-1.0-230		
Sample ID	:		
Data Filename	: GOY-GG-99-1-rac-odh-9010-1.0-231.lcd		
Method Filename	: GY-1.0.lcm		
Batch Filename	:		
Vial #	: 1-1	Sample Type	: Unknown
Injection Volume	: 20 uL		
Date Acquired	: 11/9/2020 3:23:00 PM	Acquired by	: System Administrator
Date Processed	: 11/9/2020 4:43:55 PM	Processed by	: System Administrator

<Chromatogram>

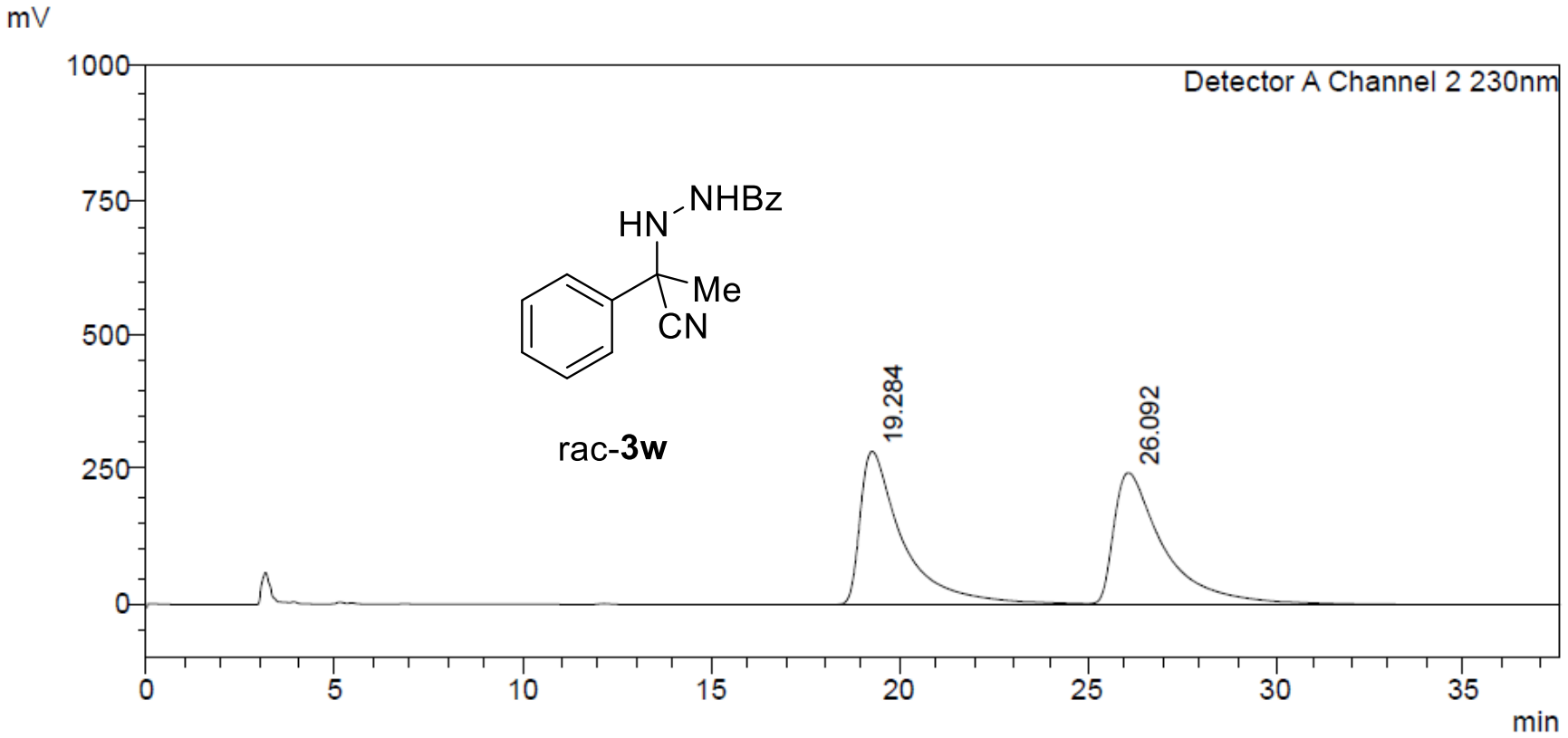

<Peak Table>

Detector A Channel 2 230nm

Peak#	Ret. Time	Area	Height	Conc.
1	19.284	22848144	285592	49.832
2	26.092	23002522	245535	50.168
Total		45850666	531127	

<Sample Information>

Sample Name	: GOY-GG-99-1-asy-odh-9010-1.0-230		
Sample ID	:		
Data Filename	: GOY-GG-99-1-rac-odh-9010-1.0-232.lcd		
Method Filename	: GY-1.0.lcm		
Batch Filename	:		
Vial #	: 1-1	Sample Type	: Unknown
Injection Volume	: 20 uL		
Date Acquired	: 11/9/2020 4:01:15 PM	Acquired by	: System Administrator
Date Processed	: 11/9/2020 4:42:14 PM	Processed by	: System Administrator

<Chromatogram>

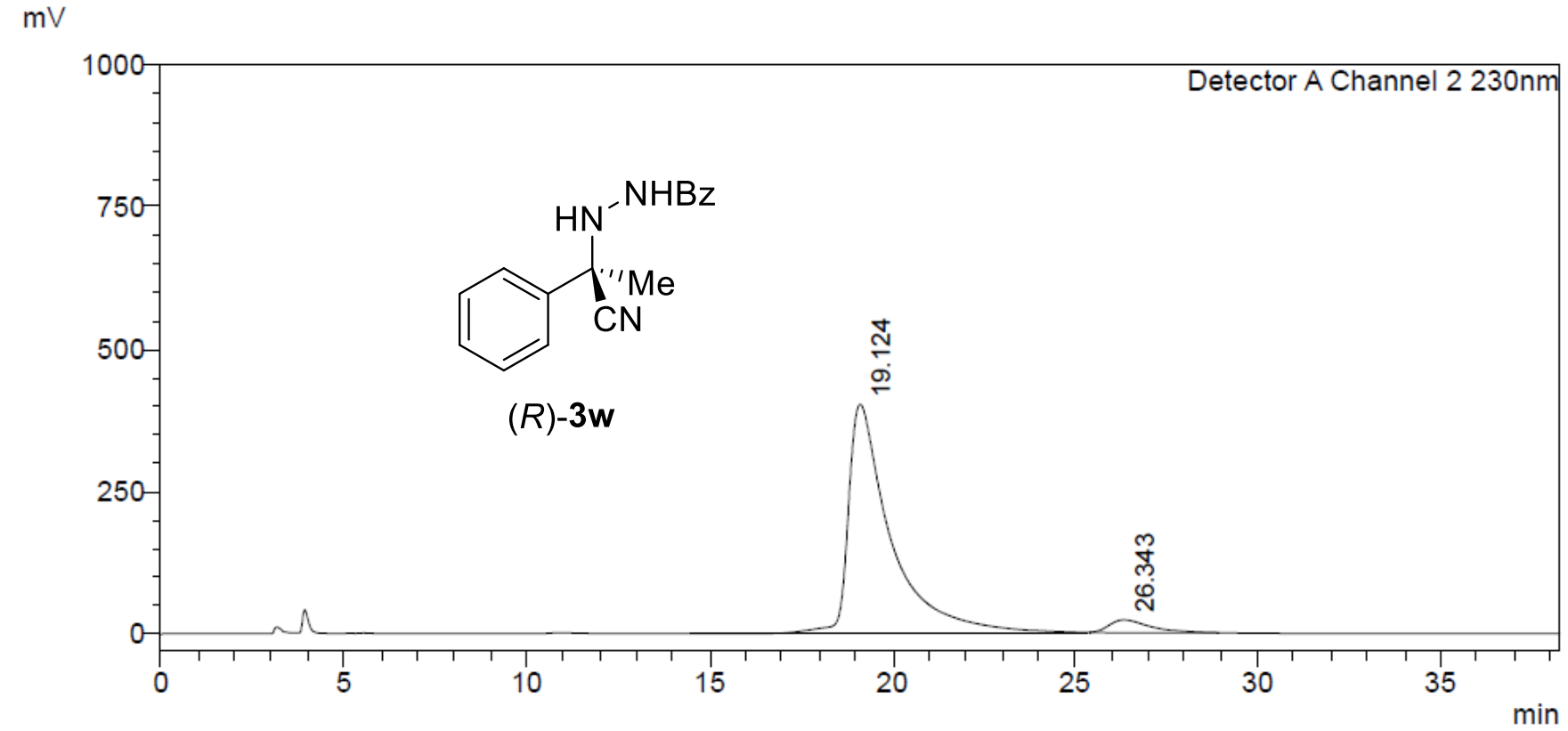

<Peak Table>

Detector A Channel 2 230nm

Peak#	Ret. Time	Area	Height	Conc.
1	19.124	33138382	403507	95.085
2	26.343	1712894	22051	4.915
Total		34851276	425558	

<Sample Information>

Sample Name	: GOY-GF-145-3-rac-ie-7525-1.0-230		
Sample ID	:		
Data Filename	: GOY-GF-145-3-rac-ie-7525-1.0-230.lcd		
Method Filename	: GY-1.0.lcm		
Batch Filename	:		
Vial #	: 1-1	Sample Type	: Unknown
Injection Volume	: 20 uL		
Date Acquired	: 8/31/2020 9:17:34 PM	Acquired by	: System Administrator
Date Processed	: 8/31/2020 9:39:38 PM	Processed by	: System Administrator

<Chromatogram>

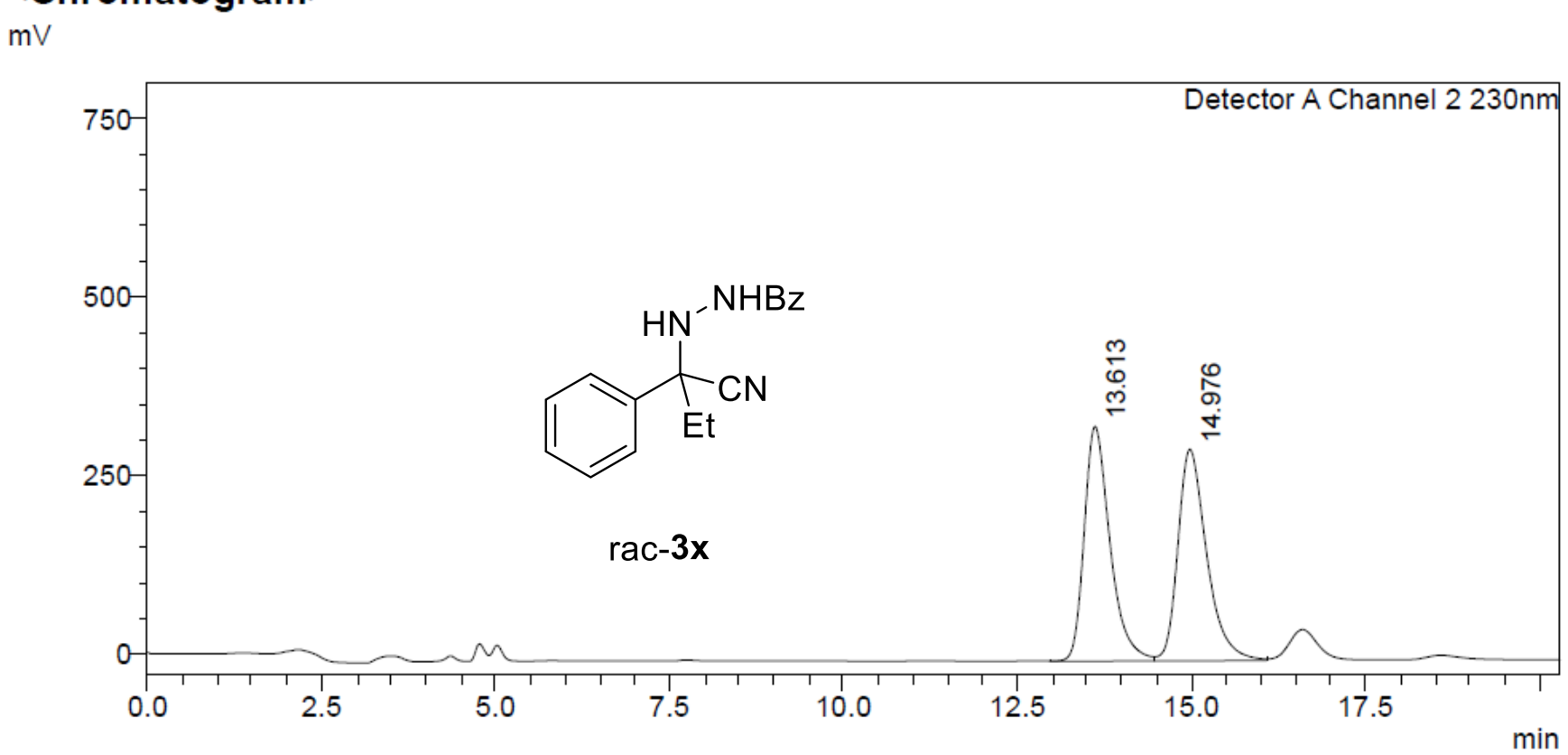

<Peak Table>

Detector A Channel 2 230nm

Peak#	Ret. Time	Area	Height	Conc.
1	13.613	8506836	329525	49.818
2	14.976	8568866	297230	50.182
Total		17075702	626755	

<Sample Information>

Sample Name	: GOY-GF-145-3-asy-ie-7525-1.0-230		
Sample ID	:		
Data Filename	: GOY-GF-145-3-asy-ie-7525-1.0-230.lcd		
Method Filename	: GY-1.0.lcm		
Batch Filename	:		
Vial #	: 1-1	Sample Type	: Unknown
Injection Volume	: 20 uL		
Date Acquired	: 8/31/2020 9:38:33 PM	Acquired by	: System Administrator
Date Processed	: 8/31/2020 9:58:29 PM	Processed by	: System Administrator

<Chromatogram>

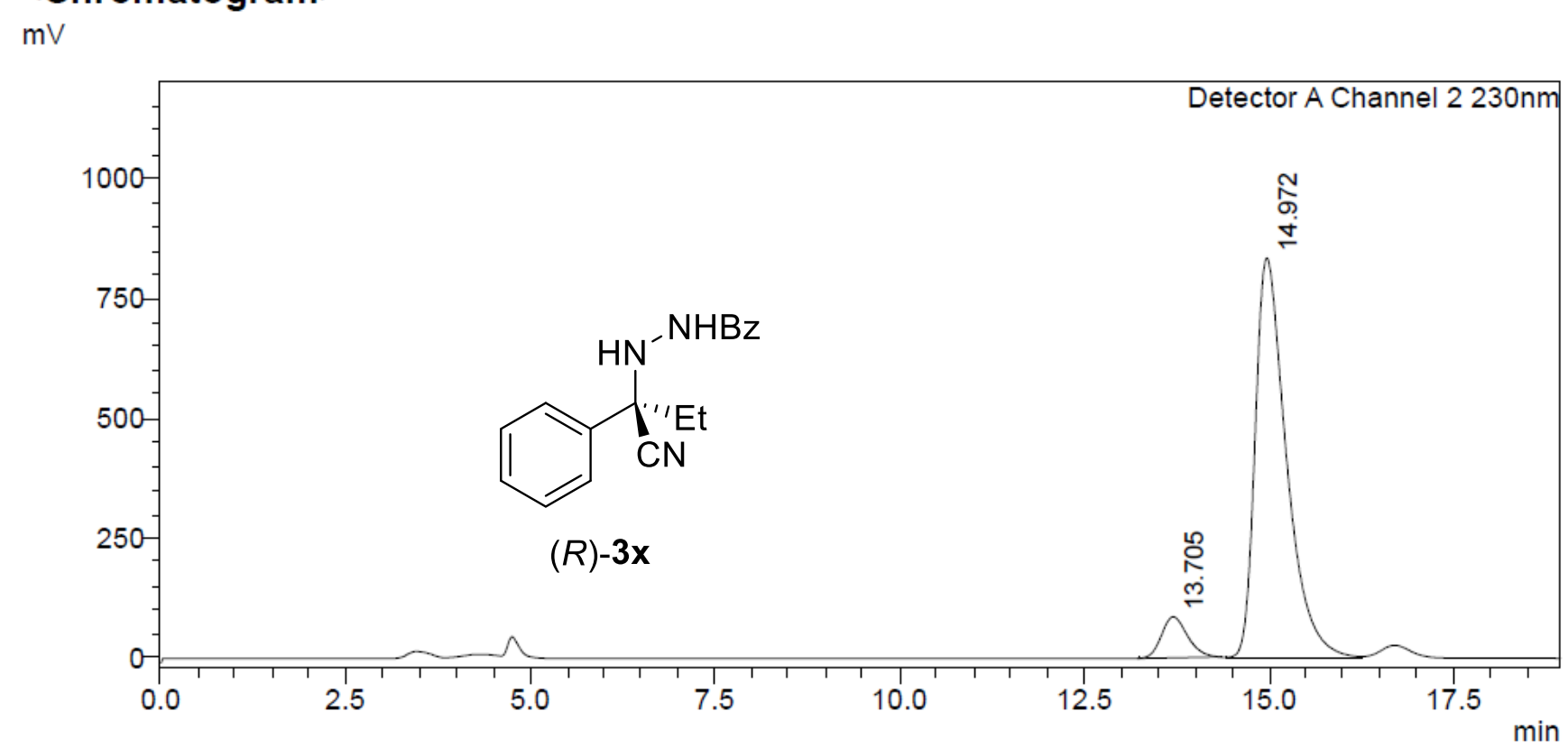

<Peak Table>

Detector A Channel 2 230nm

Peak#	Ret. Time	Area	Height	Conc.
1	13.705	2095880	85322	7.829
2	14.972	24673568	834314	92.171
Total		26769448	919636	

<Sample Information>

Sample Name : GOY-GG-48-4-rac-ia-9010-1.0-230
Sample ID :
Data Filename : GOY-GG-48-4-rac-ia-9010-1.0-231.lcd
Method Filename : GY-1.0.lcm
Batch Filename :
Vial # : 1-1
Sample Type : Unknown
Injection Volume : 20 uL
Date Acquired : 10/11/2020 7:07:49 PM
Acquired by : System Administrator
Date Processed : 10/11/2020 7:48:25 PM
Processed by : System Administrator

<Chromatogram>

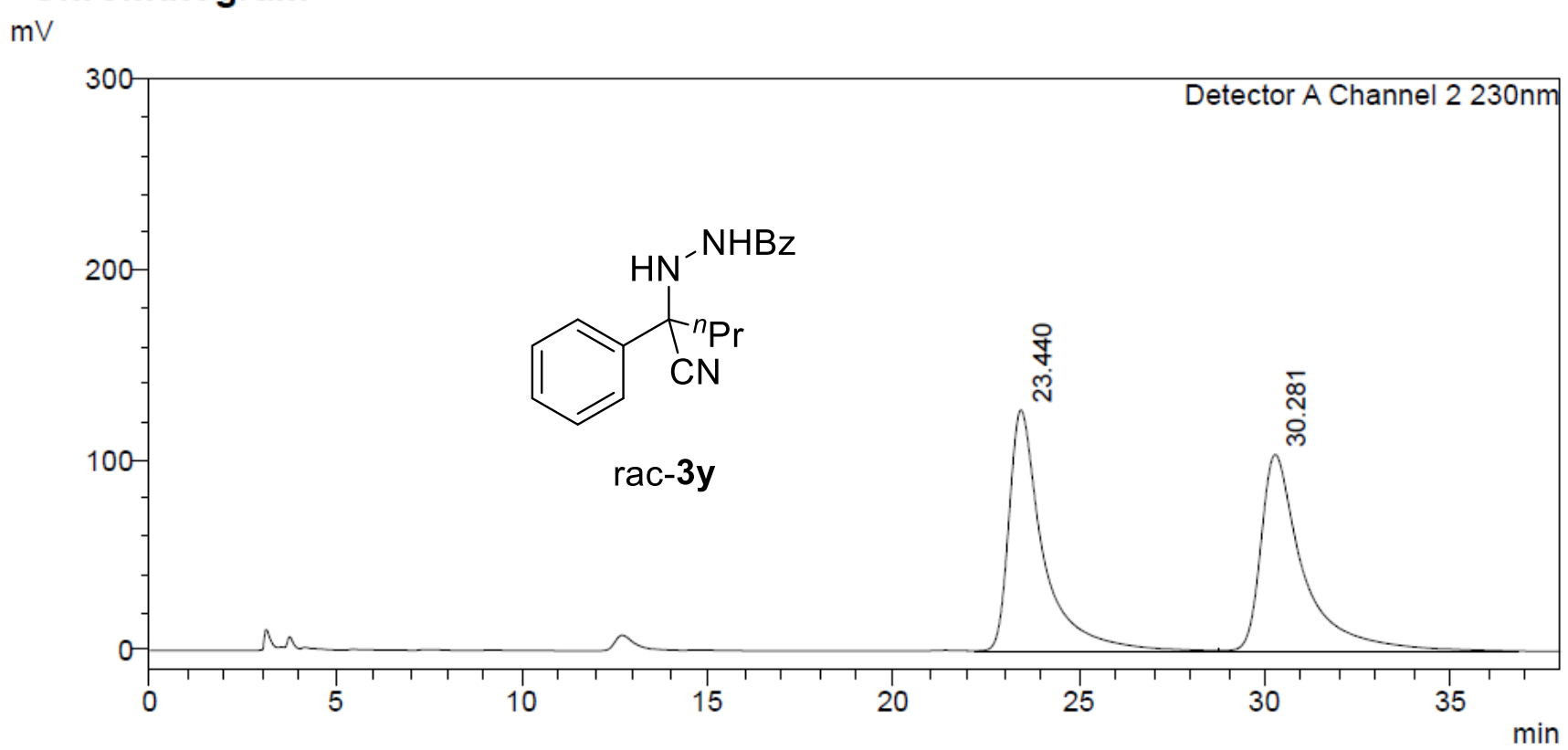

<Peak Table>

Detector A Channel 2 230nm

Peak#	Ret. Time	Area	Height	Conc.
1	23.440	8138103	126583	49.770
2	30.281	8213242	103255	50.230
Total		16351345	229838	

<Sample Information>

Sample Name : GOY-GG-48-4-asy-ia-9010-1.0-230
Sample ID :
Data Filename : GOY-GG-48-4-asy-ia-9010-1.0-231.lcd
Method Filename : GY-1.0.lcm
Batch Filename :
Vial # : 1-1
Sample Type : Unknown
Injection Volume : 20 uL
Date Acquired : 10/11/2020 7:46:45 PM
Acquired by : System Administrator
Date Processed : 10/11/2020 8:26:51 PM
Processed by : System Administrator

<Chromatogram>

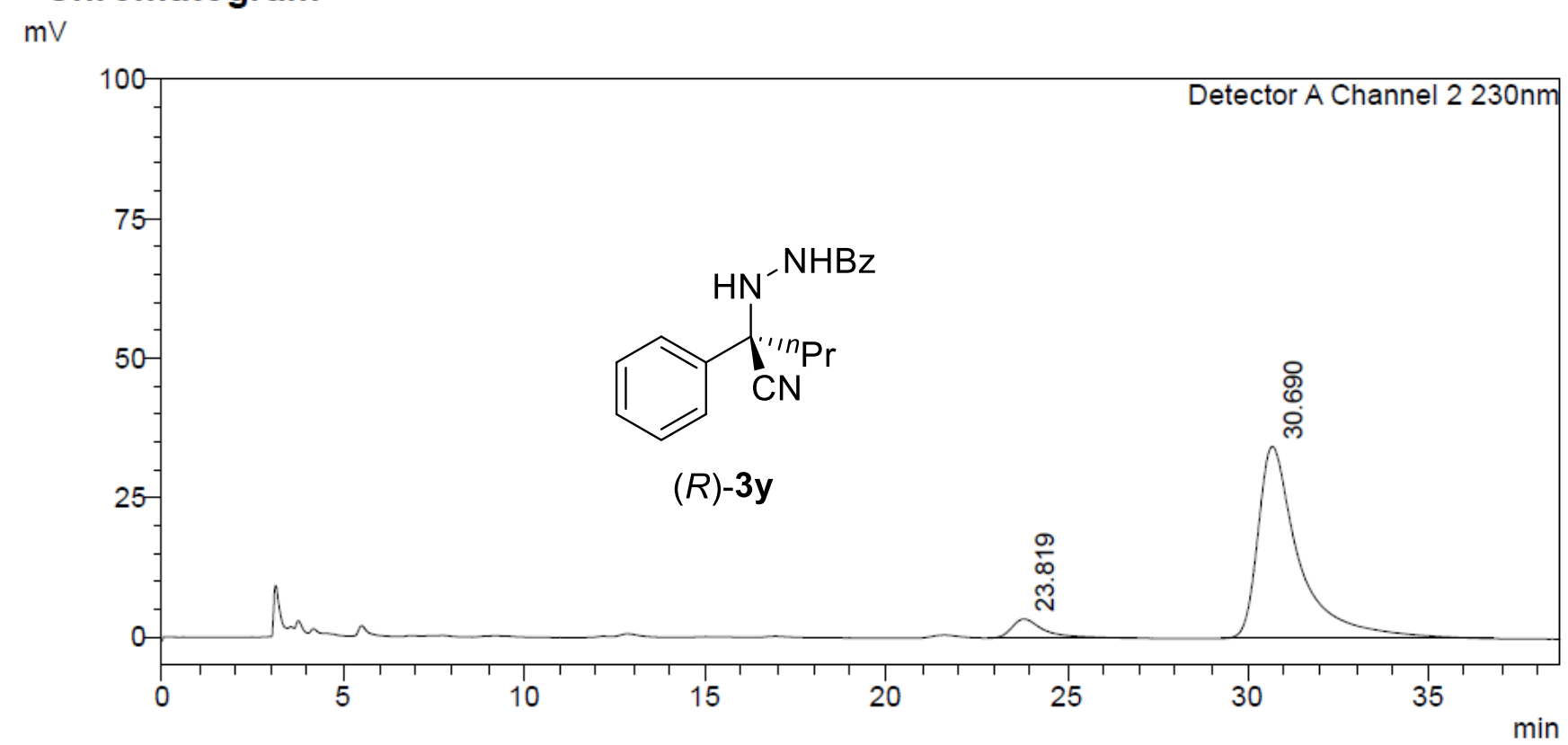

<Peak Table>

Detector A Channel 2 230nm

Peak#	Ret. Time	Area	Height	Conc.
1	23.819	213450	3425	7.199
2	30.690	2751533	34461	92.801
Total		2964984	37886	

<Sample Information>

Sample Name	: ZHZ-ZAC-126-RAC-IE-85-15-1.0-254-230		
Sample ID	:		
Data Filename	: ZHZ-ZAC-126-RAC-IE-85-15-1.0-254-230.lcd		
Method Filename	: GY-1.0.lcm		
Batch Filename	:		
Vial #	: 1-1	Sample Type	: Unknown
Injection Volume	: 20 uL		
Date Acquired	: 4/13/2026 12:39:24 AM	Acquired by	: System Administrator
Date Processed	: 4/13/2026 1:11:22 AM	Processed by	: System Administrator

<Chromatogram>

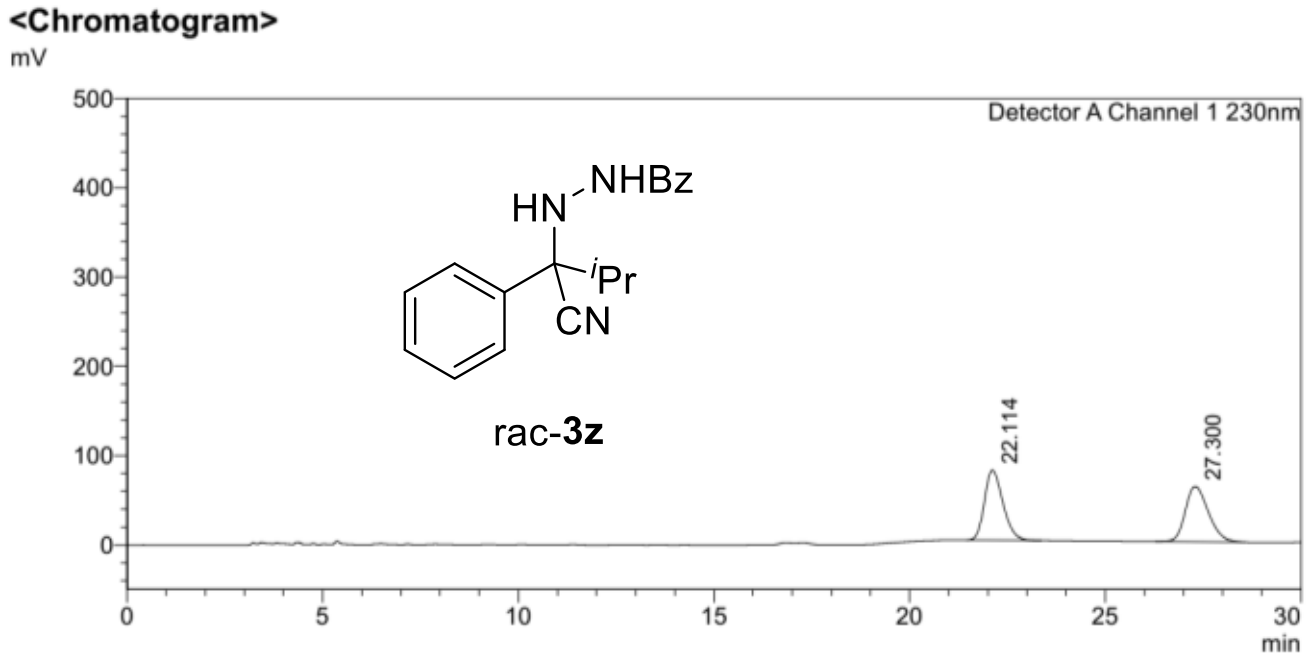

<Peak Table>

Detector A Channel 1 230nm

Peak#	Ret. Time	Area	Height	Conc.
1	22.114	2587800	78399	50.653
2	27.300	2521028	61914	49.347
Total		5108829	140313	

C:\Data\ZHZ\ZHZ-ZAC-126-RAC-IE-85-15-1.0-254-230.lcd

<Sample Information>

Sample Name	: ZHZ-ZAC-126-ASY-IE-85-15-1.0-254-230		
Sample ID	:		
Data Filename	: ZHZ-ZAC-126-ASY-IE-85-15-1.0-254-230.lcd		
Method Filename	: GY-1.0.lcm		
Batch Filename	:		
Vial #	: 1-1	Sample Type	: Unknown
Injection Volume	: 20 uL		
Date Acquired	: 4/13/2026 12:01:16 AM	Acquired by	: System Administrator
Date Processed	: 4/13/2026 12:36:51 AM	Processed by	: System Administrator

<Chromatogram>

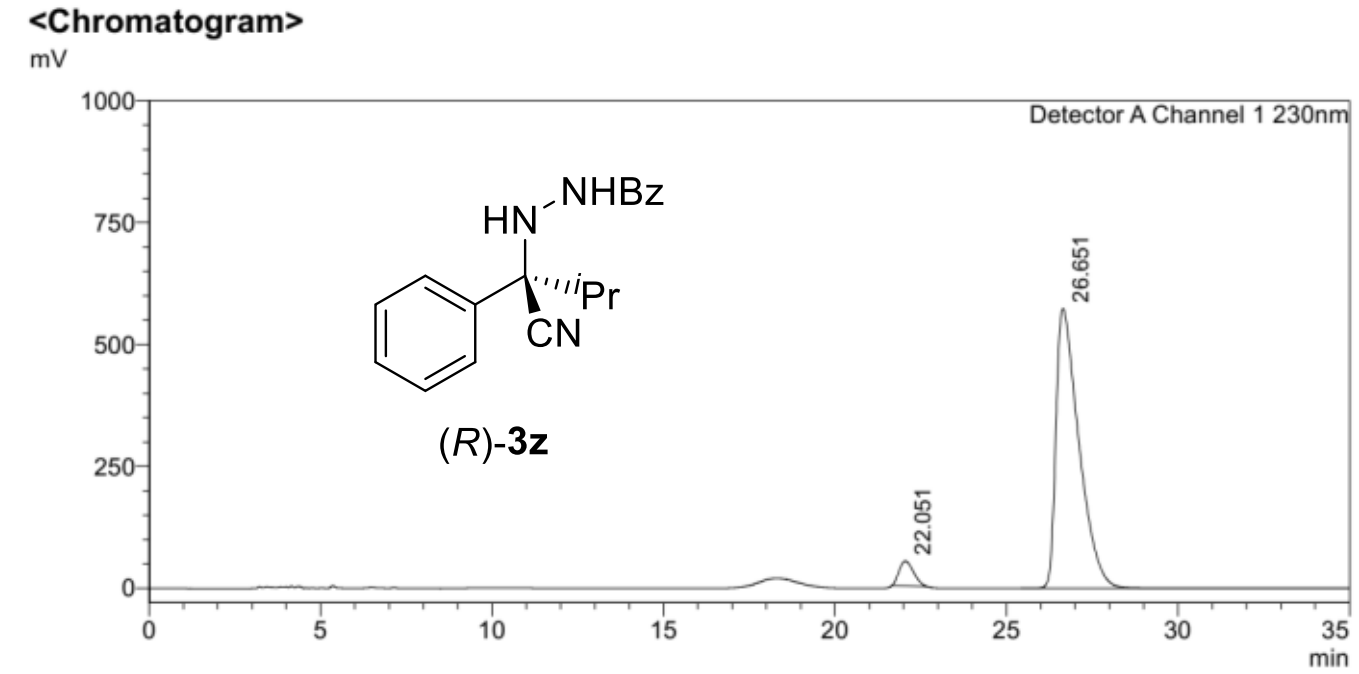

<Peak Table>

Detector A Channel 1 230nm

Peak#	Ret. Time	Area	Height	Conc.
1	22.051	1485278	50853	5.268
2	26.651	26710579	573829	94.732
Total		28195857	624682	

C:\Data\ZHZ\ZHZ-ZAC-126-ASY-IE-85-15-1.0-254-230.lcd

<Sample Information>

Sample Name : gy-0121-tbu-IA-70-30-254-230-1.0
Sample ID :
Data Filename : gy-0121-tbu-IA-70-30-254-230-1.0.lcd
Method Filename : ZHZ-1.0-254-230.lcm
Batch Filename :
Vial # : 1-1
Sample Type : 未知
Injection Volume : 20 uL
Date Acquired : 2026/1/15 22:36:15
Acquired by : System Administrator
Date Processed : 2026/1/15 22:53:49
Processed by : System Administrator

<Chromatogram>

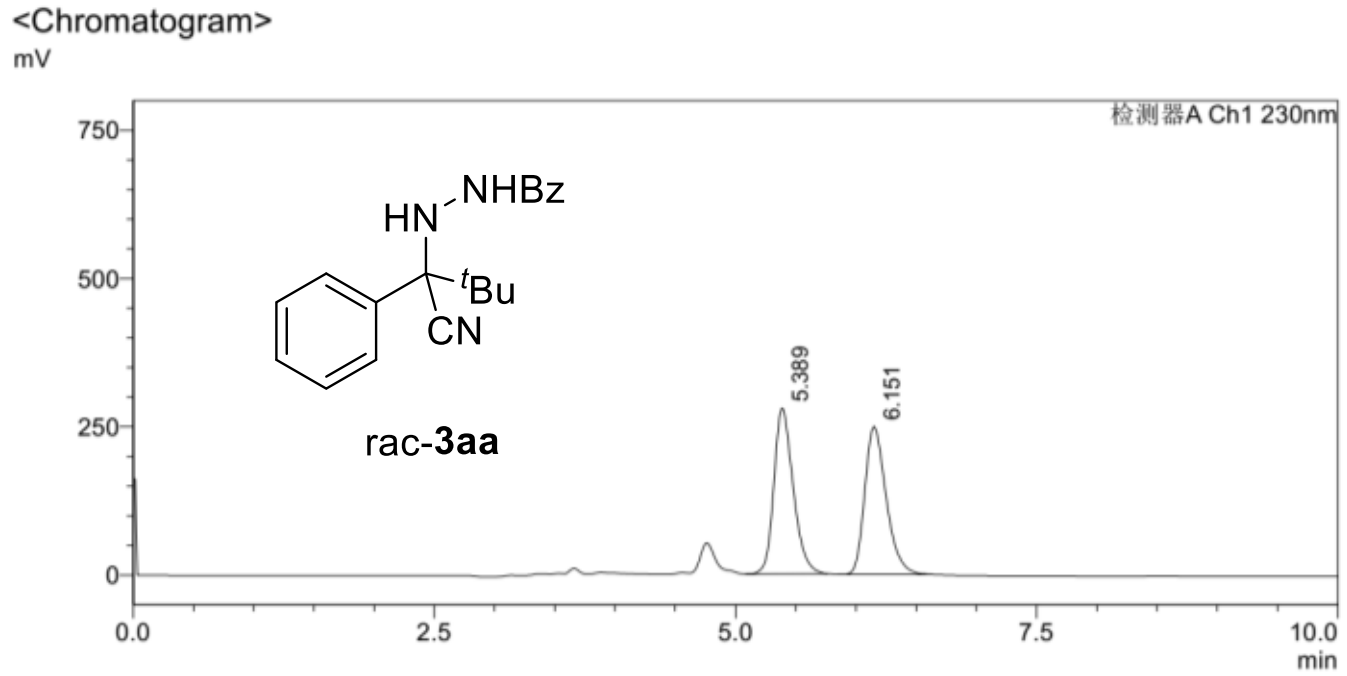

<Peak Table>

检测器A Ch1 230nm

Peak#	Ret. Time	Area	Height	Conc.
1	5.389	3118100	279757	49.997
2	6.151	3118462	249373	50.003
总计		6236561	529131	

E:\Data\ZHZ\gy-0121-tbu-IA-70-30-254-230-1.0.lcd

<Sample Information>

Sample Name : ZHZ-ZAC-130-ASY-IA-75-25-210-220-1.0-
Sample ID :
Data Filename : ZHZ-ZAC-130-IA-ASY-75-25-210-220-1.0-.lcd
Method Filename : ZHZ-1.0-254-230.lcm
Batch Filename :
Vial # : 1-1
Sample Type : 未知
Injection Volume : 20 uL
Date Acquired : 2026/3/10 0:57:37
Acquired by : System Administrator
Date Processed : 2026/4/6 17:07:29
Processed by : System Administrator

<Chromatogram>

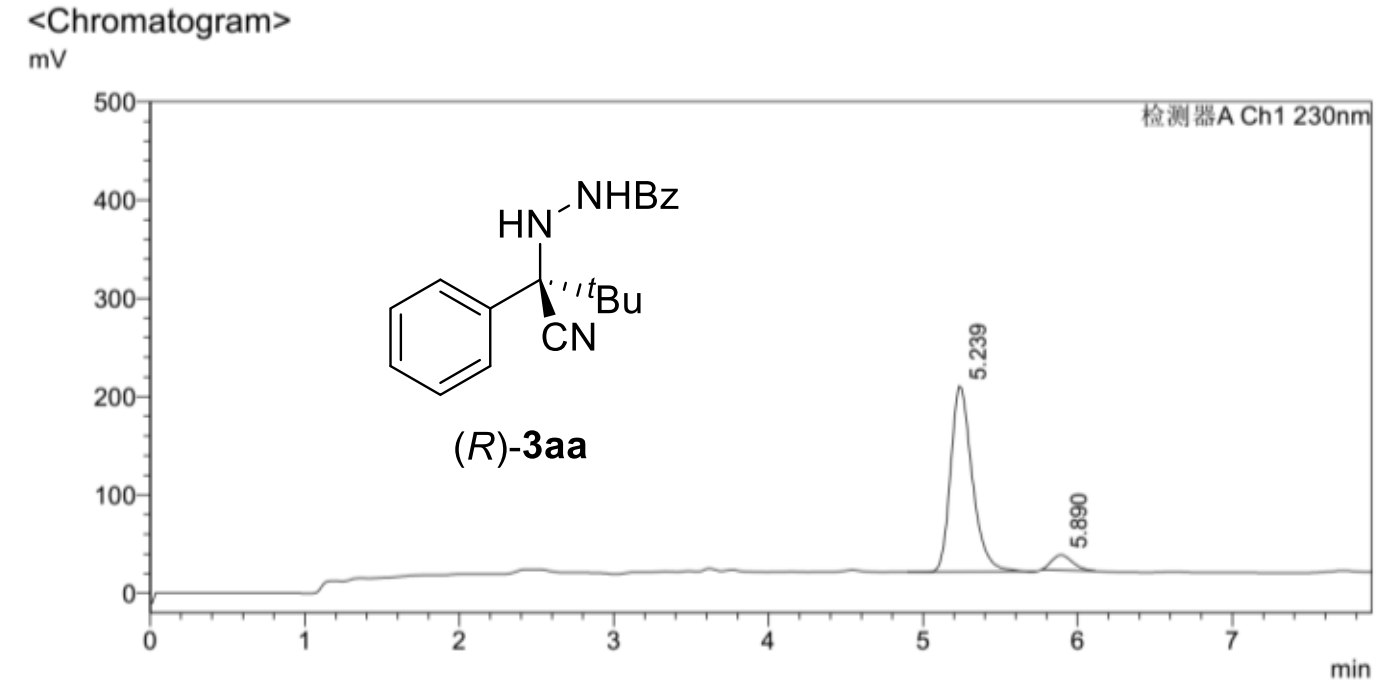

<Peak Table>

检测器A Ch1 230nm

Peak#	Ret. Time	Area	Height	Conc.
1	5.239	1837493	188745	92.747
2	5.890	143692	15222	7.253
总计		1981186	203967	

E:\Data\ZHZ\ZHZ-ZAC-130-IA-ASY-75-25-210-220-1.0-.lcd

<Sample Information>

Sample Name	: ZHZ-ZAC-150-RAC-IE-80-20-1.0-254-230		
Sample ID	:		
Data Filename	: ZHZ-ZAC-150-RAC-IE-80-20-1.0-254-230.lcd		
Method Filename	: ZHZ-1.0-254-230.lcm		
Batch Filename	:		
Vial #	: 1-1	Sample Type	: 未知
Injection Volume	: 20 uL		
Date Acquired	: 2026/3/30 22:07:29	Acquired by	: System Administrator
Date Processed	: 2026/3/30 22:28:06	Processed by	: System Administrator

<Chromatogram>

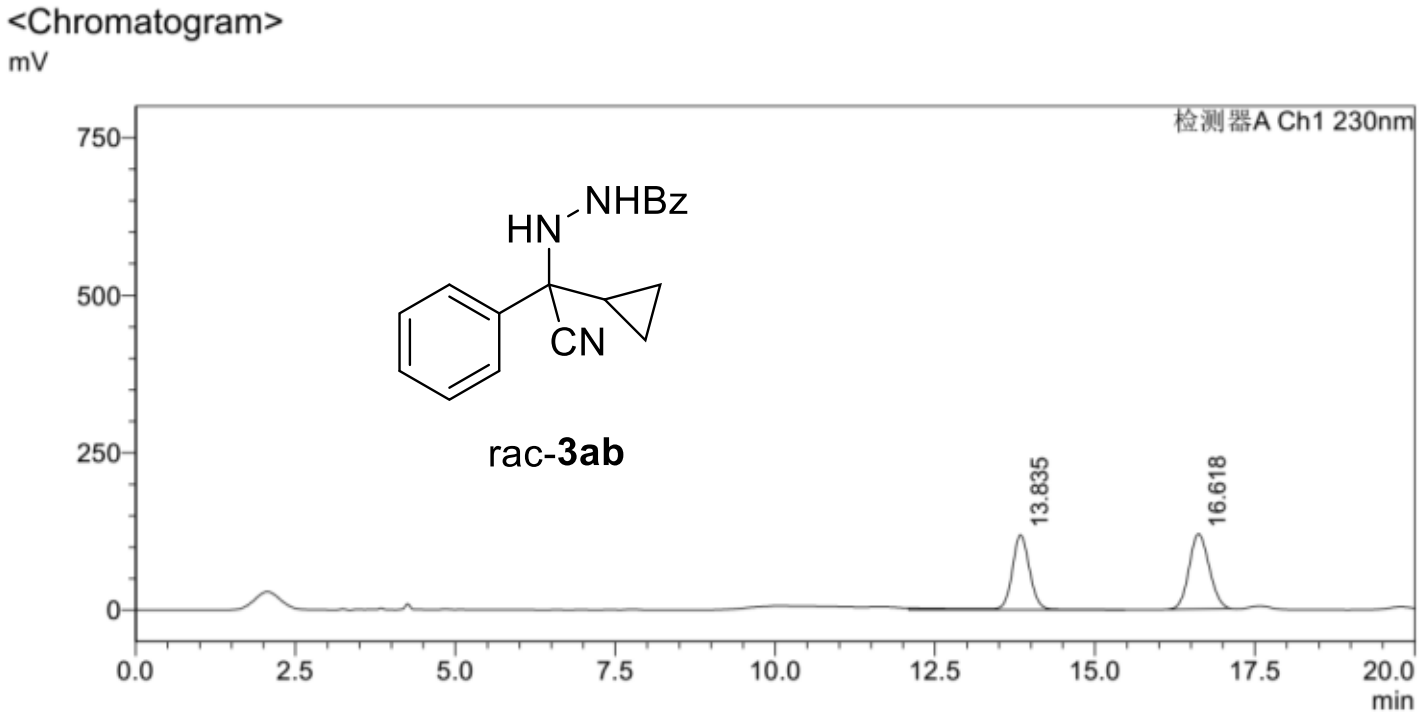

<Peak Table>

检测器A Ch1 230nm

Peak#	Ret. Time	Area	Height	Conc.
1	13.835	2412381	118928	47.813
2	16.618	2633020	119274	52.187
总计		5045401	238202	

E:\Data\ZHZ\ZHZ-ZAC-150-RAC-IE-80-20-1.0-254-230.lcd

<Sample Information>

Sample Name	: ZHZ-ZAC-150-ASY-IE-80-20-1.0-254-230		
Sample ID	:		
Data Filename	: ZHZ-ZAC-150-ASY-IE-80-20-1.0-254-230.lcd		
Method Filename	: ZHZ-1.0-254-230.lcm		
Batch Filename	:		
Vial #	: 1-1	Sample Type	: 未知
Injection Volume	: 20 uL		
Date Acquired	: 2026/3/30 21:47:15	Acquired by	: System Administrator
Date Processed	: 2026/3/30 22:07:00	Processed by	: System Administrator

<Chromatogram>

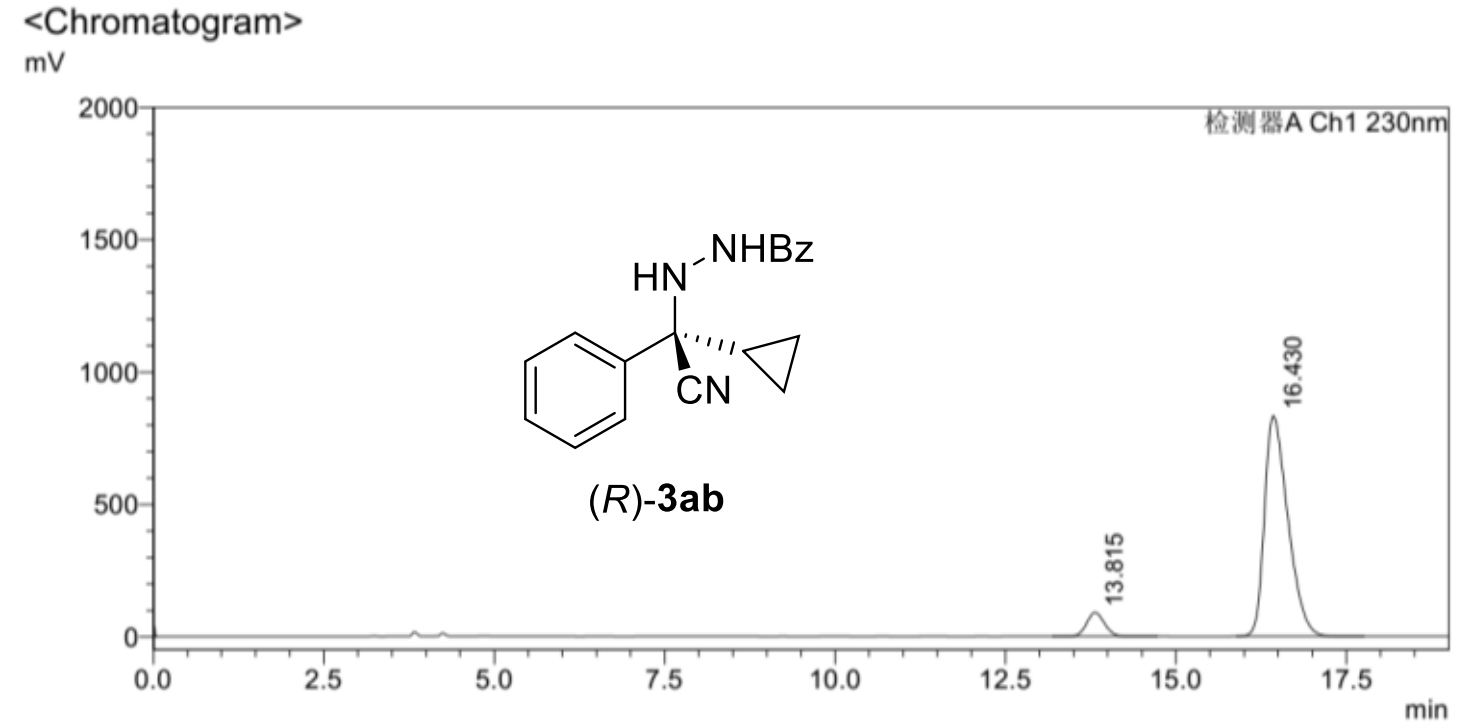

<Peak Table>

检测器A Ch1 230nm

Peak#	Ret. Time	Area	Height	Conc.
1	13.815	1660495	90184	7.665
2	16.430	20001601	833040	92.335
总计		21662096	923224	

E:\Data\ZHZ\ZHZ-ZAC-150-ASY-IE-80-20-1.0-254-230.lcd

<Sample Information>

Sample Name	: ZHZ-ZAC-phcy-rac-IE-85-15-1.0-254-230		
Sample ID	:		
Data Filename	: ZHZ-ZAC-phcy-rac-IE-85-15-1.0-254-230.lcd		
Method Filename	: GY-1.0.lcm		
Batch Filename	:		
Vial #	: 1-1	Sample Type	: Unknown
Injection Volume	: 20 uL		
Date Acquired	: 4/13/2026 9:46:59 AM	Acquired by	: System Administrator
Date Processed	: 4/13/2026 10:28:23 AM	Processed by	: System Administrator

<Chromatogram>

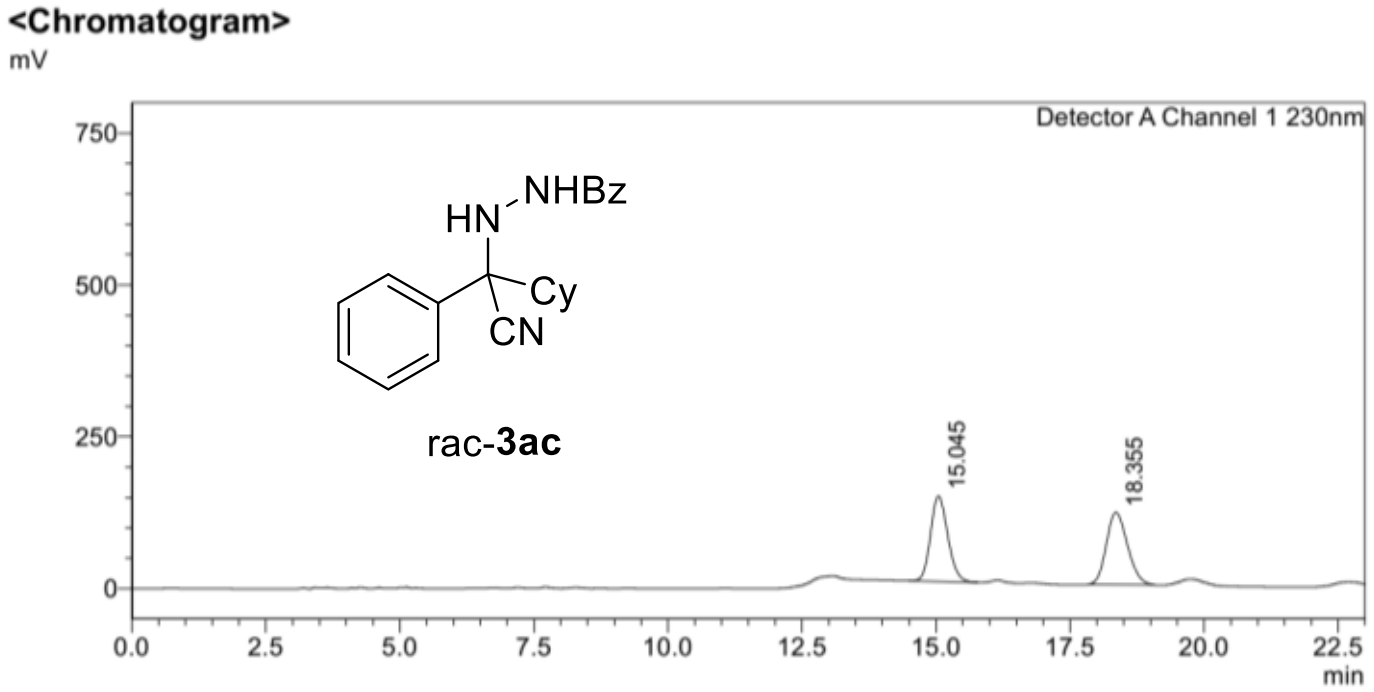

<Peak Table>

Detector A Channel 1 230nm

Peak#	Ret. Time	Area	Height	Conc.
1	15.045	3153215	140579	49.197
2	18.355	3256143	118805	50.803
Total		6409358	259384	

C:\Data\ZHZ\ZHZ-ZAC-phcy-rac-IE-85-15-1.0-254-230.lcd

<Sample Information>

Sample Name	: ZHZ-ZAC-phcy-IE-85-15-1.0-254-230		
Sample ID	:		
Data Filename	: ZHZ-ZAC-phcy-IE-85-15-1.0-254-230.lcd		
Method Filename	: GY-1.0.lcm		
Batch Filename	:		
Vial #	: 1-1	Sample Type	: Unknown
Injection Volume	: 20 uL		
Date Acquired	: 4/13/2026 9:16:22 AM	Acquired by	: System Administrator
Date Processed	: 4/13/2026 9:46:28 AM	Processed by	: System Administrator

<Chromatogram>

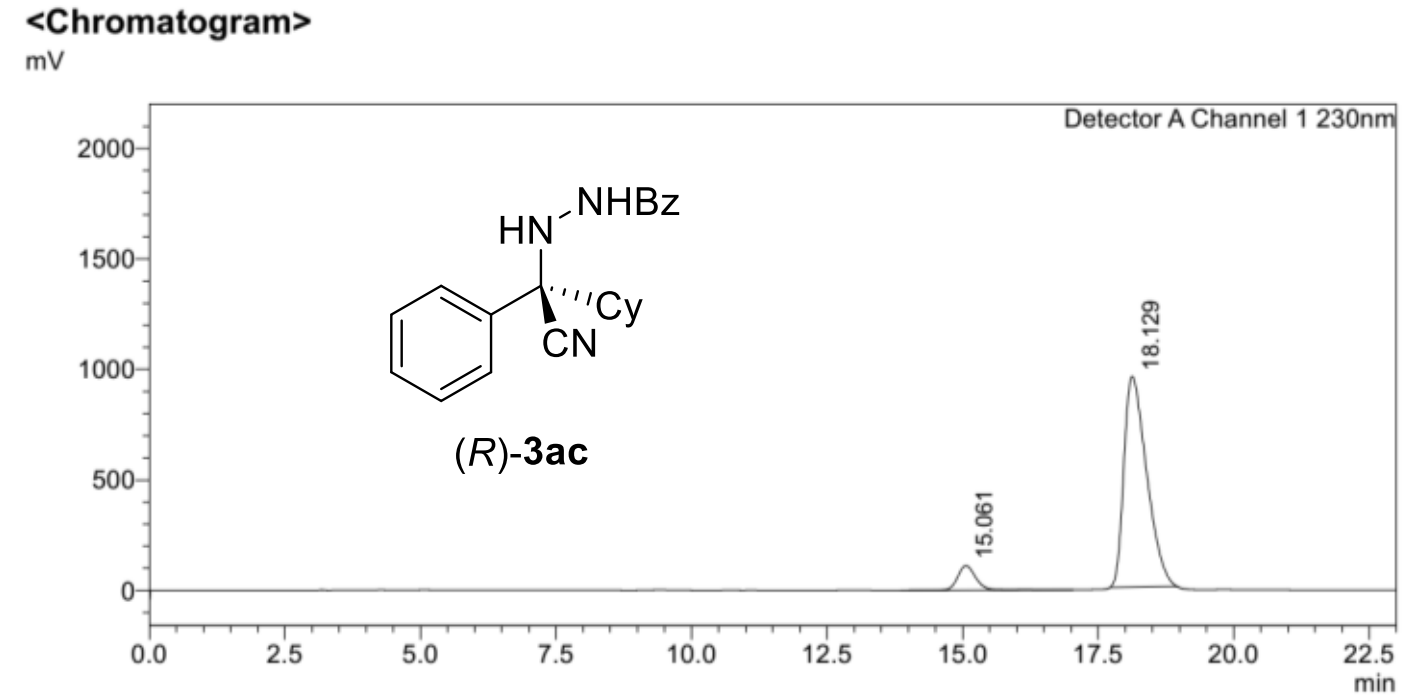

<Peak Table>

Detector A Channel 1 230nm

Peak#	Ret. Time	Area	Height	Conc.
1	15.061	2626033	111039	8.599
2	18.129	27911179	953291	91.401
Total		30537213	1064330	

C:\Data\ZHZ\ZHZ-ZAC-phcy-IE-85-15-1.0-254-230.lcd

<Sample Information>

Sample Name	: GOY-GG-118-1-rac-ie-8020-1.0-230		
Sample ID	:		
Data Filename	: GOY-GG-118-1-rac-ie-8020-1.0-231.lcd		
Method Filename	: GY-1.0.lcm		
Batch Filename	:		
Vial #	: 1-1	Sample Type	: Unknown
Injection Volume	: 20 uL		
Date Acquired	: 11/21/2020 11:07:24 AM	Acquired by	: System Administrator
Date Processed	: 11/21/2020 2:09:28 PM	Processed by	: System Administrator

<Chromatogram>

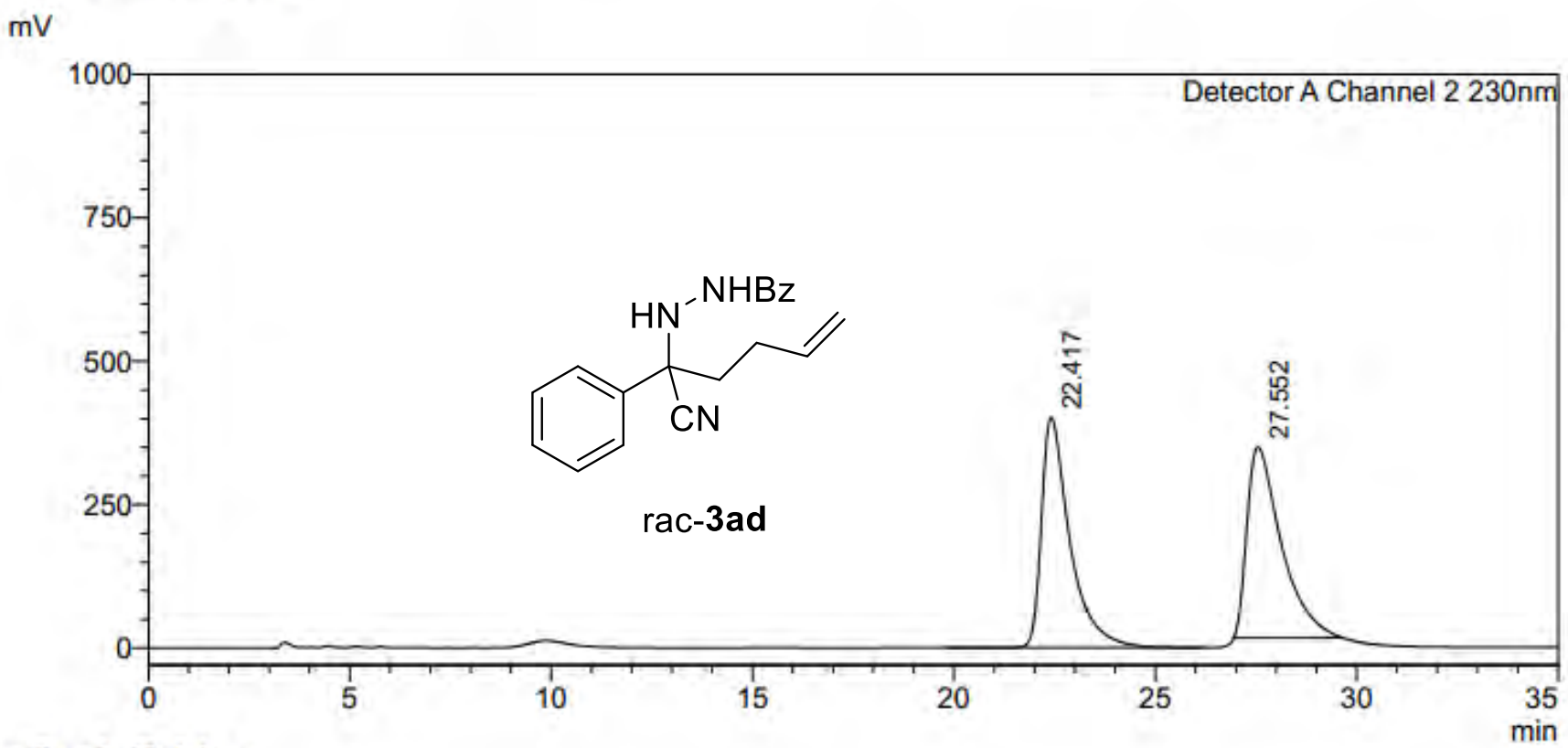

<Peak Table>

Detector A Channel 2 230nm

Peak#	Ret. Time	Area	Height	Conc.
1	22.417	19736529	401959	50.022
2	27.552	19719076	332636	49.978
Total		39455605	734595	

<Sample Information>

Sample Name	: GOY-GG-118-1-asy-ie-8020-1.0-230		
Sample ID	:		
Data Filename	: GOY-GG-118-1-asy-ie-8020-1.0-231.lcd		
Method Filename	: GY-1.0.lcm		
Batch Filename	:		
Vial #	: 1-1	Sample Type	: Unknown
Injection Volume	: 20 uL		
Date Acquired	: 11/21/2020 2:04:44 PM	Acquired by	: System Administrator
Date Processed	: 11/21/2020 2:45:37 PM	Processed by	: System Administrator

<Chromatogram>

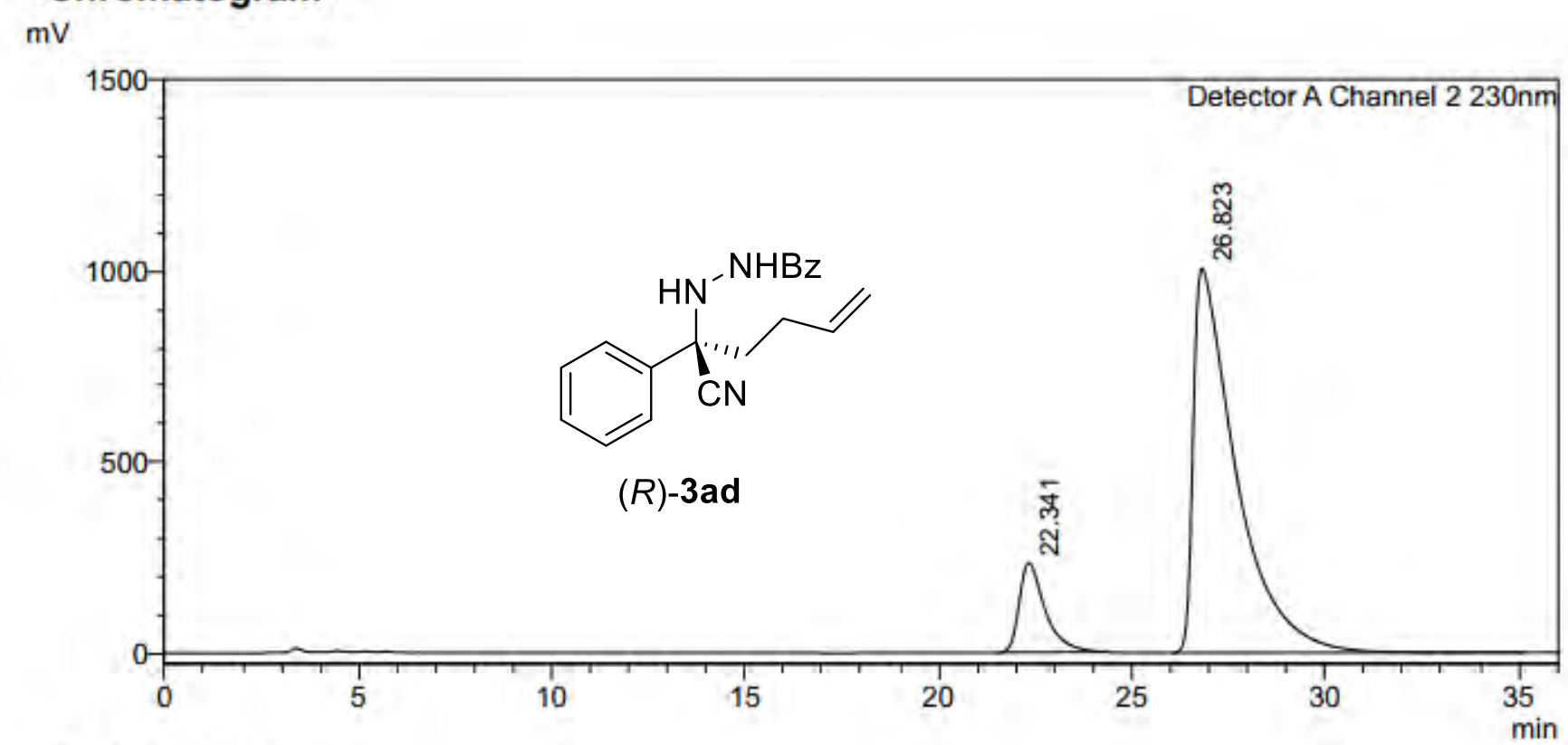

<Peak Table>

Detector A Channel 2 230nm

Peak#	Ret. Time	Area	Height	Conc.
1	22.341	10875900	234232	12.339
2	26.823	77265338	1006527	87.661
Total		88141238	1240759	

<Sample Information>

Sample Name	: GOY-GG-48-3-rac-ia-8020-1.0-230		
Sample ID	:		
Data Filename	: GOY-GG-48-3-rac-ia-8020-1.0-231.lcd		
Method Filename	: GY-1.0.lcm		
Batch Filename	:		
Vial #	: 1-1	Sample Type	: Unknown
Injection Volume	: 20 uL		
Date Acquired	: 10/11/2020 9:49:56 AM	Acquired by	: System Administrator
Date Processed	: 10/11/2020 10:23:47 AM	Processed by	: System Administrator

<Chromatogram>

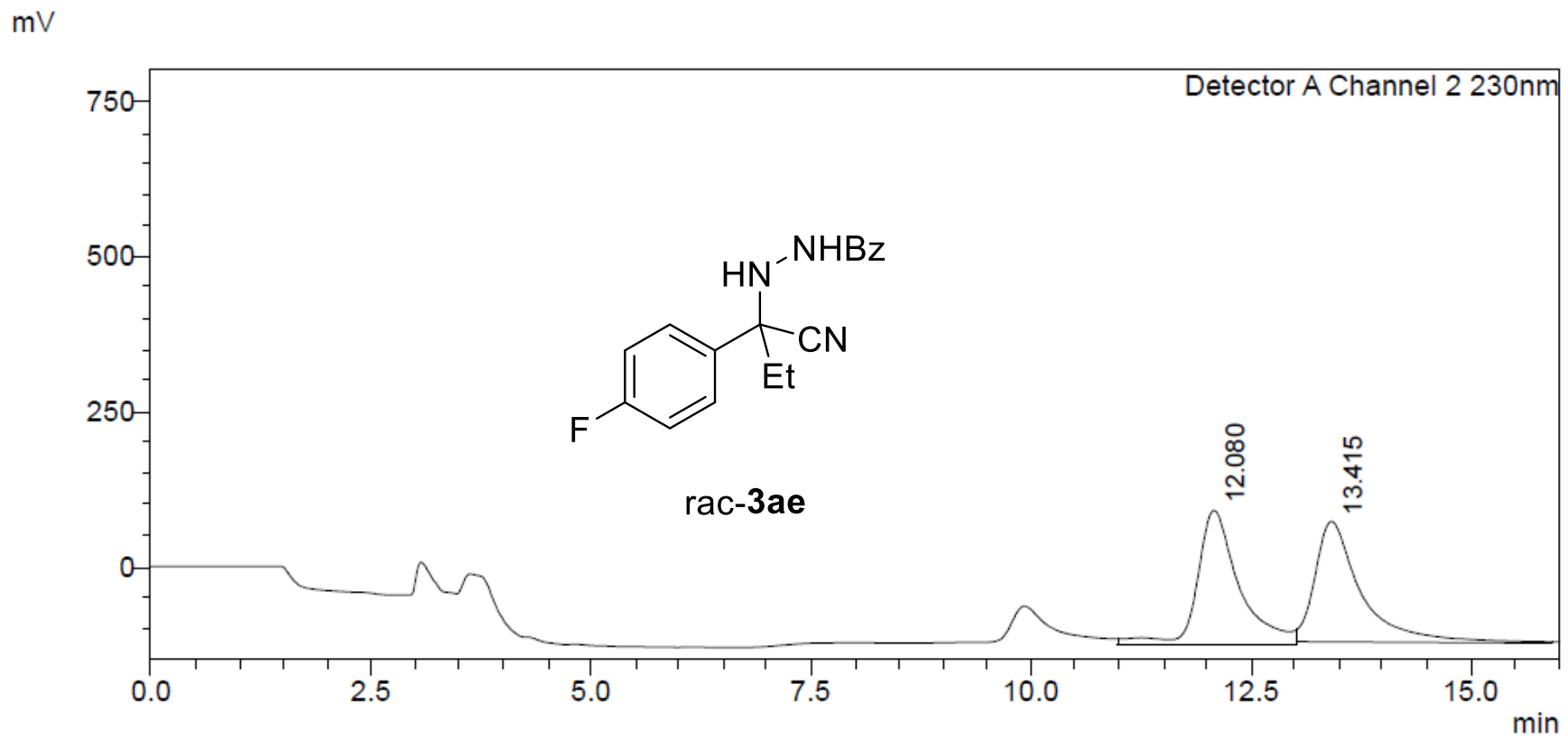

<Peak Table>

Detector A Channel 2 230nm

Peak#	Ret. Time	Area	Height	Conc.
1	12.080	7166899	215849	49.995
2	13.415	7168373	194480	50.005
Total		14335272	410329	

<Sample Information>

Sample Name	: GOY-GG-48-3-asy-ia-8020-1.0-230		
Sample ID	:		
Data Filename	: GOY-GG-48-3-asy-ia-8020-1.0-231.lcd		
Method Filename	: GY-1.0.lcm		
Batch Filename	:		
Vial #	: 1-1	Sample Type	: Unknown
Injection Volume	: 20 uL		
Date Acquired	: 10/11/2020 10:07:20 AM	Acquired by	: System Administrator
Date Processed	: 10/11/2020 10:27:47 AM	Processed by	: System Administrator

<Chromatogram>

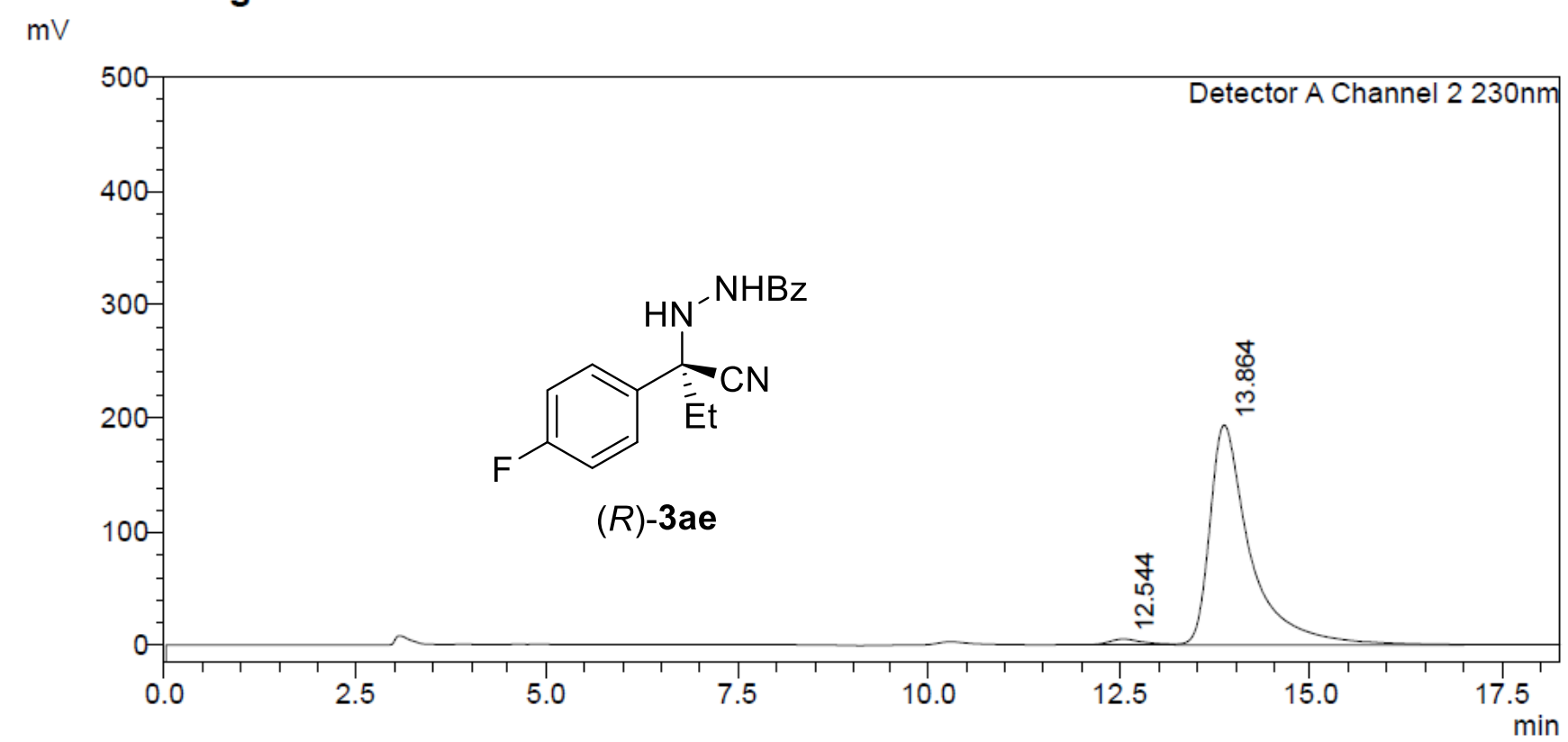

<Peak Table>

Detector A Channel 2 230nm

Peak#	Ret. Time	Area	Height	Conc.
1	12.544	161617	5295	2.203
2	13.864	7176280	193688	97.797
Total		7337898	198982	

<Sample Information>

Sample Name : GOY-GG-35-2-rac-ia-9010-1.0-230
Sample ID :
Data Filename : GOY-GG-35-2-rac-ia-9010-1.0-231.lcd
Method Filename : GY-1.0.lcm
Batch Filename :
Vial # : 1-1 Sample Type : Unknown
Injection Volume : 20 uL
Date Acquired : 10/11/2020 2:13:06 PM Acquired by : System Administrator
Date Processed : 10/11/2020 3:08:35 PM Processed by : System Administrator

<Chromatogram>

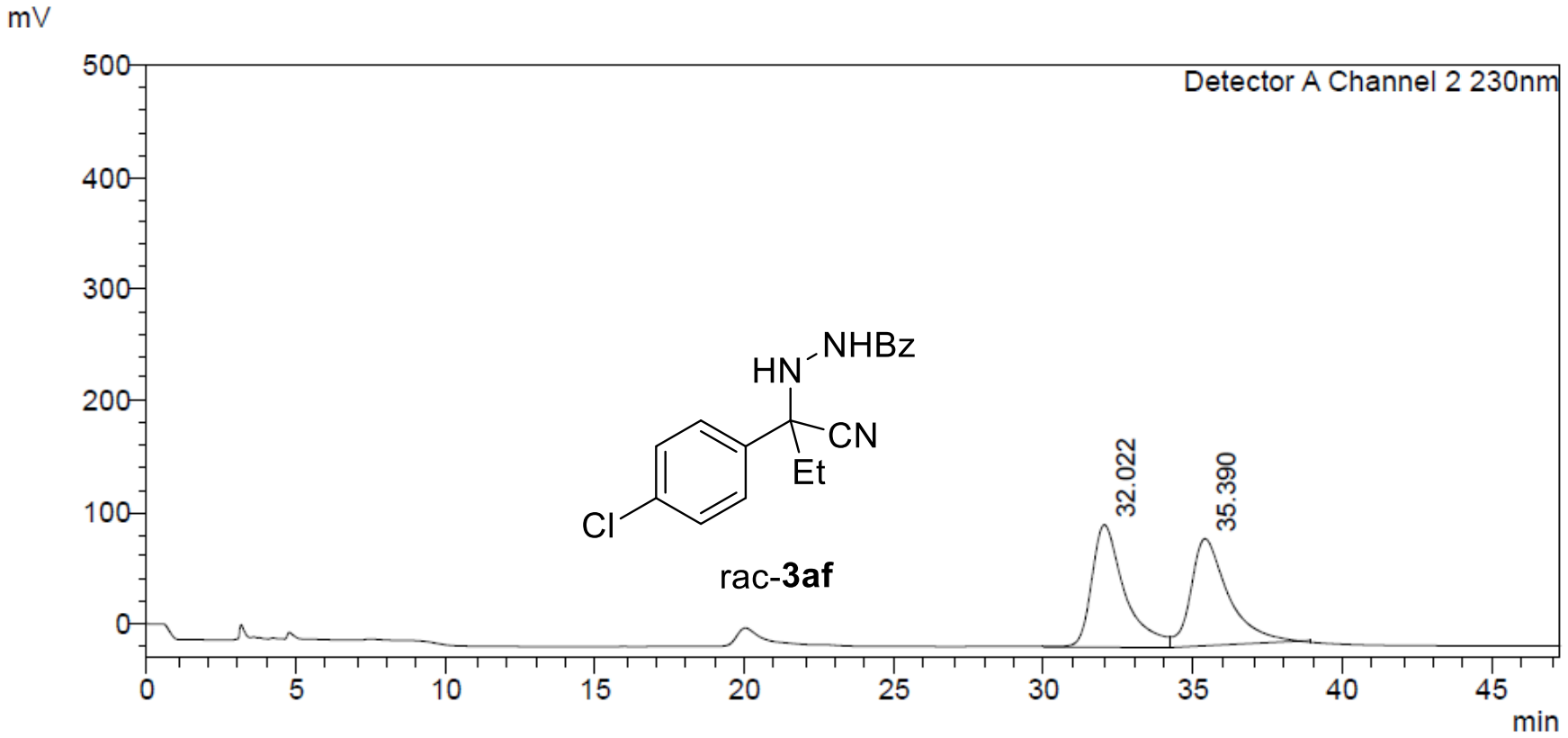

<Peak Table>

Detector A Channel 2 230nm

Peak#	Ret. Time	Area	Height	Conc.
1	32.022	8461107	109828	50.302
2	35.390	8359566	96063	49.698
Total		16820673	205891	

<Sample Information>

Sample Name : GOY-GG-35-2-asy-ia-9010-1.0-230
Sample ID :
Data Filename : GOY-GG-35-2-asy-ia-9010-1.0-232.lcd
Method Filename : GY-1.0.lcm
Batch Filename :
Vial # : 1-1 Sample Type : Unknown
Injection Volume : 20 uL
Date Acquired : 10/11/2020 3:01:35 PM Acquired by : System Administrator
Date Processed : 10/11/2020 3:58:19 PM Processed by : System Administrator

<Chromatogram>

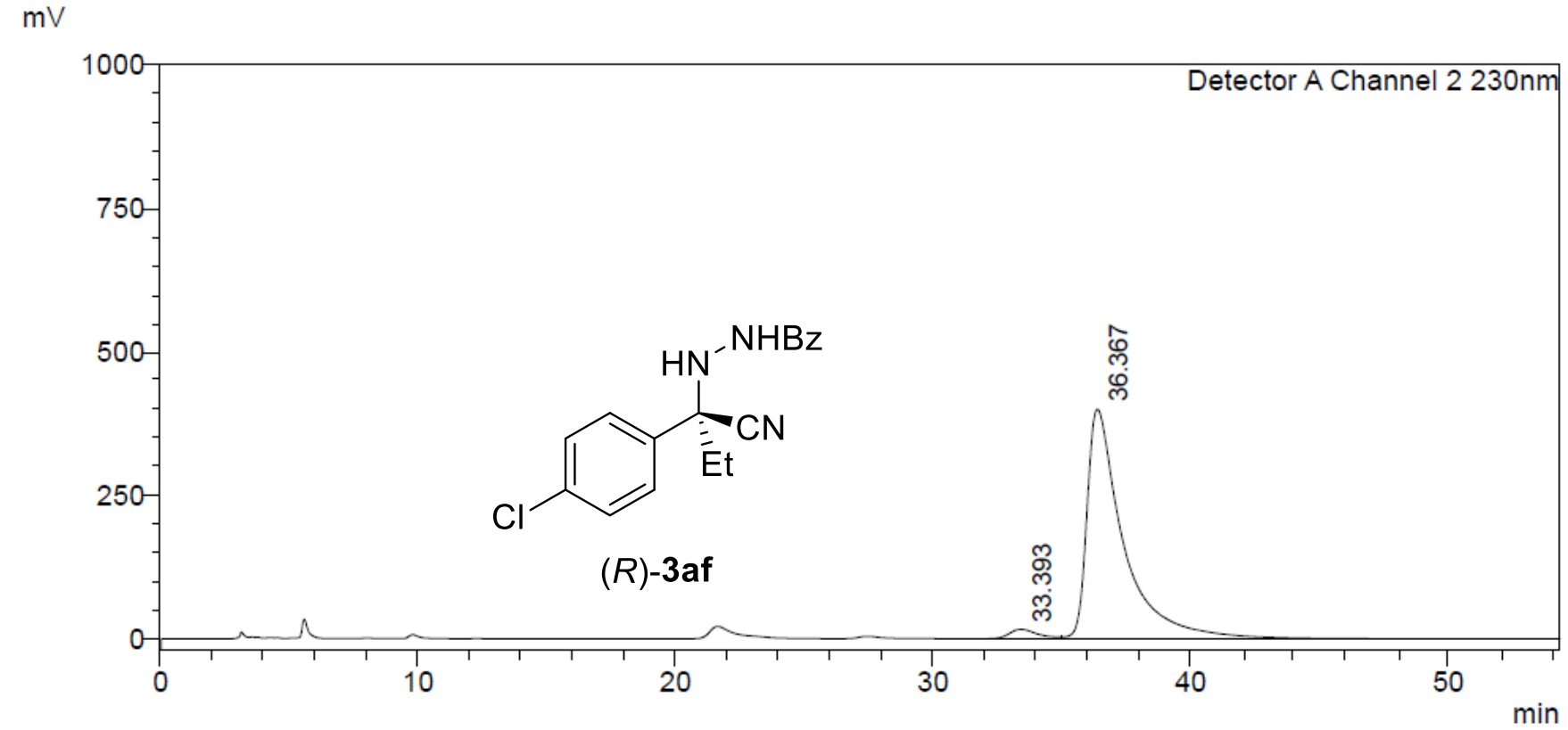

<Peak Table>

Detector A Channel 2 230nm

Peak#	Ret. Time	Area	Height	Conc.
1	33.393	1242282	16494	3.048
2	36.367	39509544	400252	96.952
Total		40751825	416746	

<Sample Information>

Sample Name	: GOY-GG-35-3-rac-ia-9010-1.0-230		
Sample ID	:		
Data Filename	: GOY-GG-35-3-rac-ia-9010-1.0-231.lcd		
Method Filename	: GY-1.0.lcm		
Batch Filename	:		
Vial #	: 1-1	Sample Type	: Unknown
Injection Volume	: 20 uL		
Date Acquired	: 10/11/2020 3:57:07 PM	Acquired by	: System Administrator
Date Processed	: 10/11/2020 4:53:06 PM	Processed by	: System Administrator

<Chromatogram>

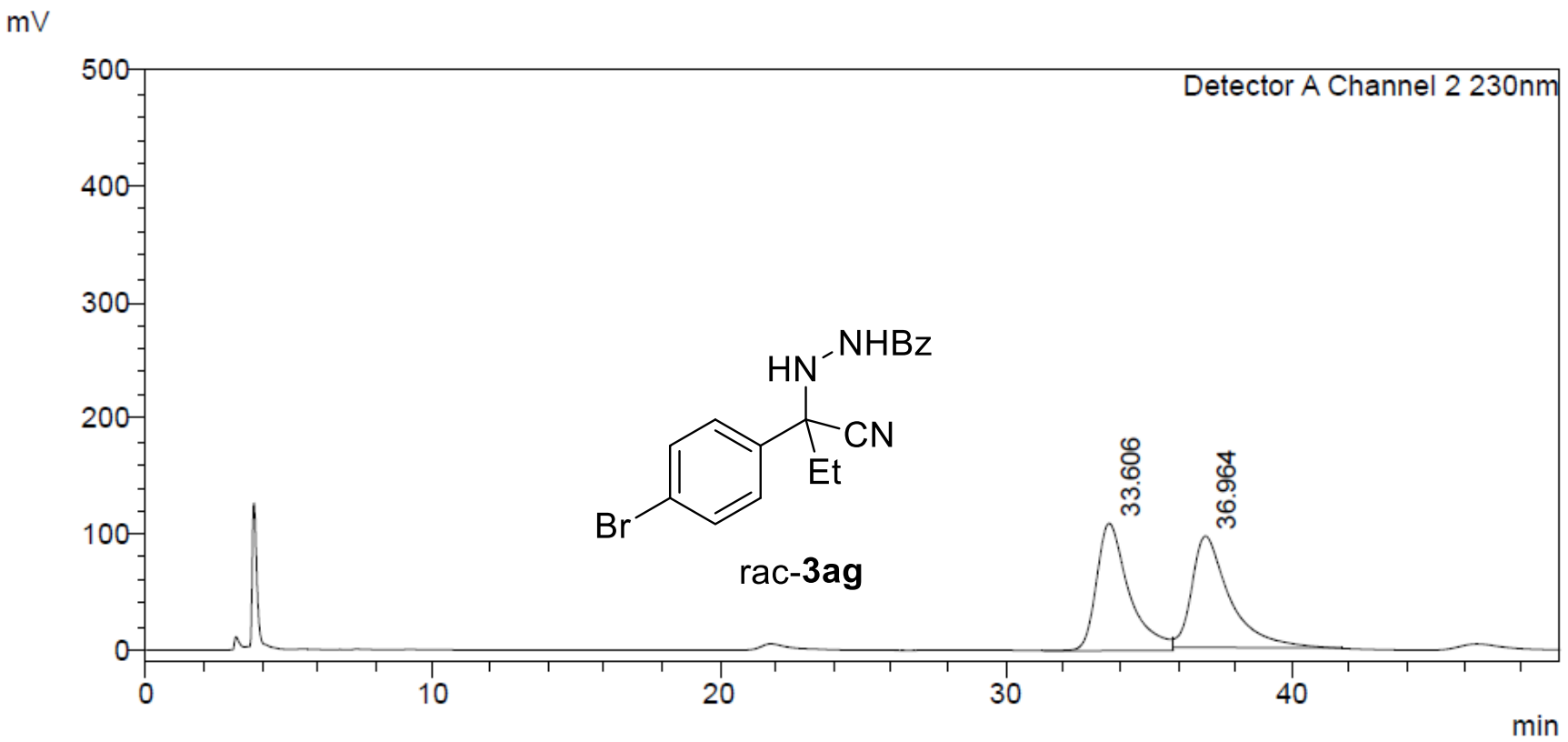

<Peak Table>

Detector A Channel 2 230nm

Peak#	Ret. Time	Area	Height	Conc.
1	33.606	8719234	109652	49.601
2	36.964	8859606	96057	50.399
Total		17578840	205709	

<Sample Information>

Sample Name	: GOY-GG-35-3-asy-ia-9010-1.0-230		
Sample ID	:		
Data Filename	: GOY-GG-35-3-asy-ia-9010-1.0-232.lcd		
Method Filename	: GY-1.0.lcm		
Batch Filename	:		
Vial #	: 1-1	Sample Type	: Unknown
Injection Volume	: 20 uL		
Date Acquired	: 10/11/2020 4:47:41 PM	Acquired by	: System Administrator
Date Processed	: 10/13/2020 8:53:30 AM	Processed by	: System Administrator

<Chromatogram>

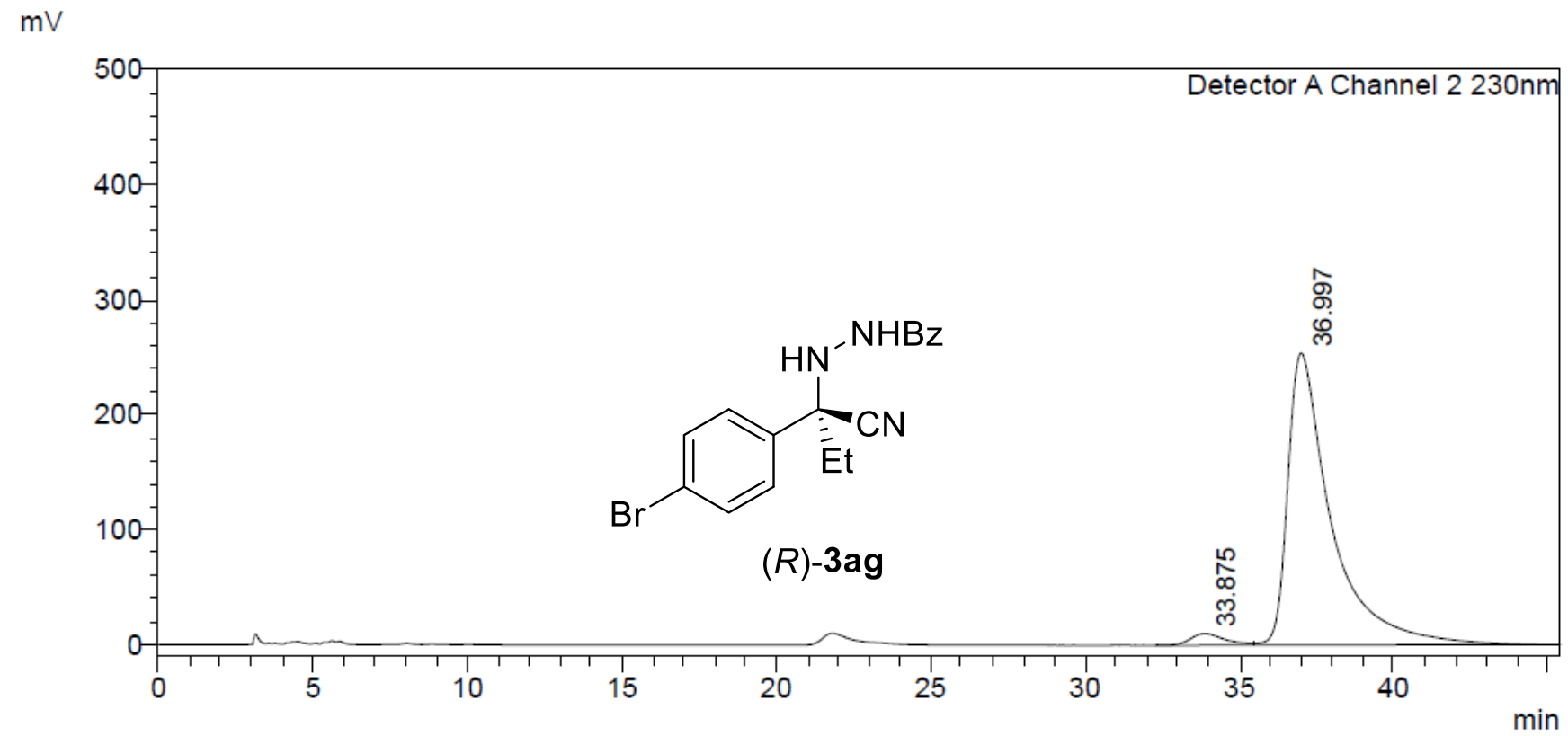

<Peak Table>

Detector A Channel 2 230nm

Peak#	Ret. Time	Area	Height	Conc.
1	33.875	793517	10290	3.079
2	36.997	24978908	254073	96.921
Total		25772425	264363	

<Sample Information>

Sample Name : GOY-GG-89-1-rac-ia-9010-1.0-230
Sample ID :
Data Filename : GOY-GG-89-1-rac-ia-9010-1.0-231.lcd
Method Filename : GY-1.0.lcm
Batch Filename :
Vial # : 1-1 Sample Type : Unknown
Injection Volume : 20 uL
Date Acquired : 10/29/2020 9:31:39 AM Acquired by : System Administrator
Date Processed : 10/29/2020 10:31:22 AM Processed by : System Administrator

<Chromatogram>

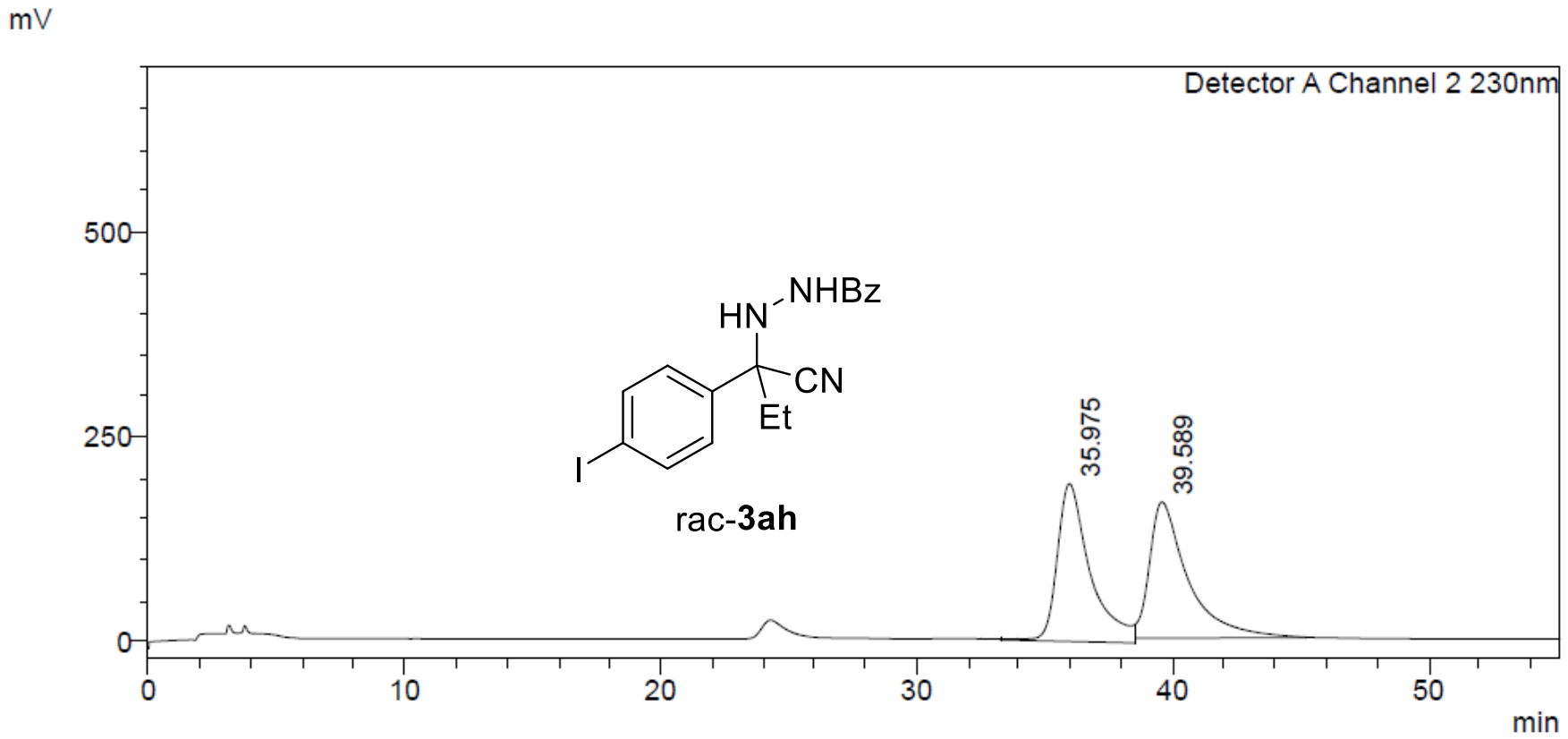

<Peak Table>

Detector A Channel 2 230nm

Peak#	Ret. Time	Area	Height	Conc.
1	35.975	17252247	192397	49.748
2	39.589	17426888	165939	50.252
Total		34679135	358336	

<Sample Information>

Sample Name : GOY-GG-89-1-asy-ia-9010-1.0-230
Sample ID :
Data Filename : GOY-GG-89-1-asy-ia-9010-1.0-232.lcd
Method Filename : GY-1.0.lcm
Batch Filename :
Vial # : 1-1 Sample Type : Unknown
Injection Volume : 20 uL
Date Acquired : 10/29/2020 10:27:38 AM Acquired by : System Administrator
Date Processed : 10/29/2020 11:27:23 AM Processed by : System Administrator

<Chromatogram>

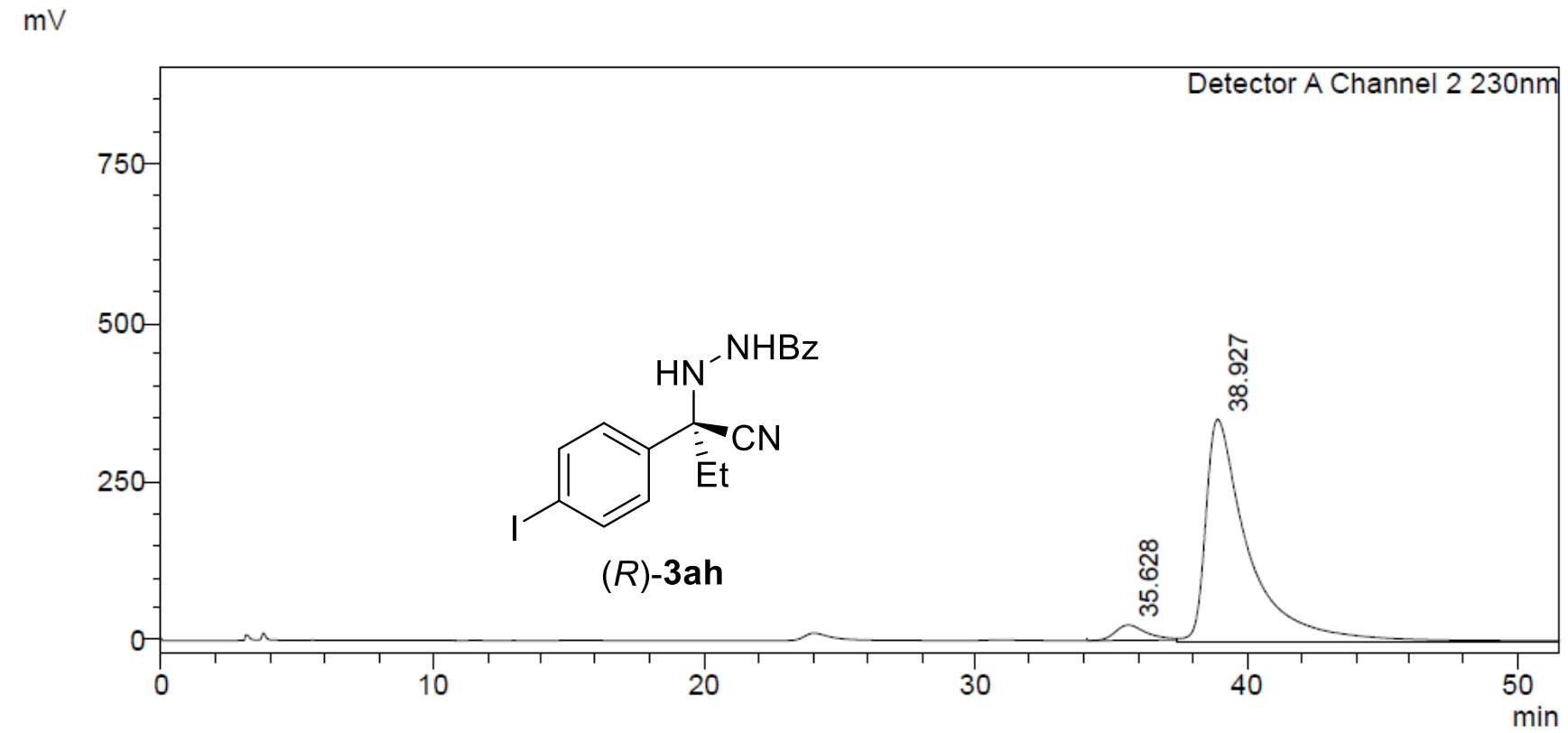

<Peak Table>

Detector A Channel 2 230nm

Peak#	Ret. Time	Area	Height	Conc.
1	35.628	1943947	24232	4.852
2	38.927	38122957	348918	95.148
Total		40066904	373150	

<Sample Information>

Sample Name : GOY-GG-81-2-rac-ia-9010-1.0-230
Sample ID :
Data Filename : GOY-GG-81-2-rac-ia-9010-1.0-231.lcd
Method Filename : GY-1.0.lcm
Batch Filename :
Vial # : 1-1 Sample Type : Unknown
Injection Volume : 20 uL
Date Acquired : 10/28/2020 9:34:59 AM Acquired by : System Administrator
Date Processed : 10/28/2020 10:21:29 AM Processed by : System Administrator

<Chromatogram>

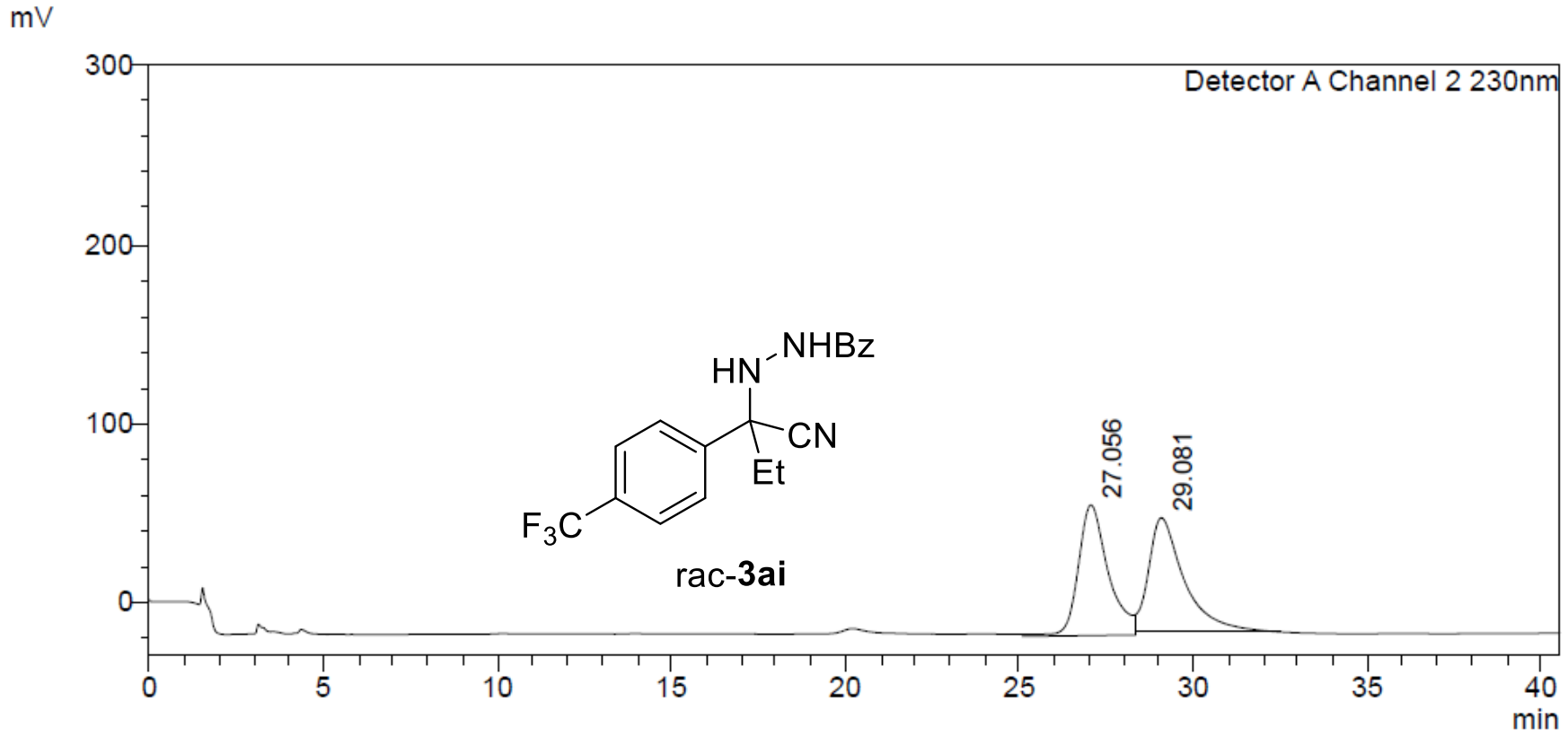

<Peak Table>

Detector A Channel 2 230nm

Peak#	Ret. Time	Area	Height	Conc.
1	27.056	4406527	73013	49.513
2	29.081	4493143	63540	50.487
Total		8899670	136554	

<Sample Information>

Sample Name : GOY-GG-81-2-asy-ia-9010-1.0-230
Sample ID :
Data Filename : GOY-GG-81-2-asy-ia-9010-1.0-231.lcd
Method Filename : GY-1.0.lcm
Batch Filename :
Vial # : 1-1 Sample Type : Unknown
Injection Volume : 20 uL
Date Acquired : 10/28/2020 10:17:02 AM Acquired by : System Administrator
Date Processed : 10/28/2020 10:58:55 AM Processed by : System Administrator

<Chromatogram>

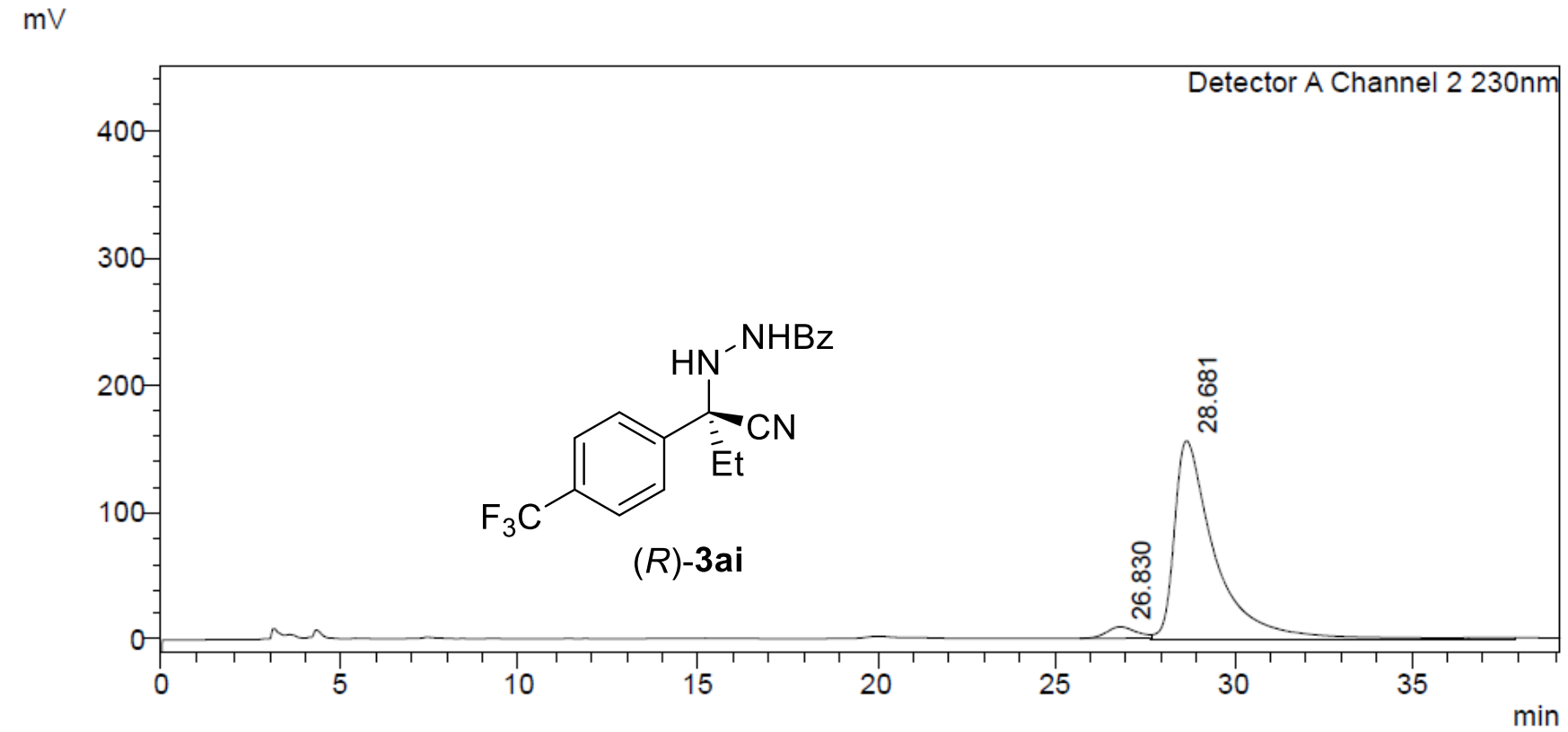

<Peak Table>

Detector A Channel 2 230nm

Peak#	Ret. Time	Area	Height	Conc.
1	26.830	516536	9055	4.032
2	28.681	12295198	155497	95.968
Total		12811734	164552	

<Sample Information>

Sample Name	: GOY-GG-35-5-rac-ia-8020-1.0-230		
Sample ID	:		
Data Filename	: GOY-GG-35-5-rac-ia-8020-1.0-234.lcd		
Method Filename	: GY-1.0.lcm		
Batch Filename	:		
Vial #	: 1-1	Sample Type	: Unknown
Injection Volume	: 20 uL		
Date Acquired	: 10/11/2020 11:25:14 AM	Acquired by	: System Administrator
Date Processed	: 10/11/2020 11:59:04 AM	Processed by	: System Administrator

<Chromatogram>

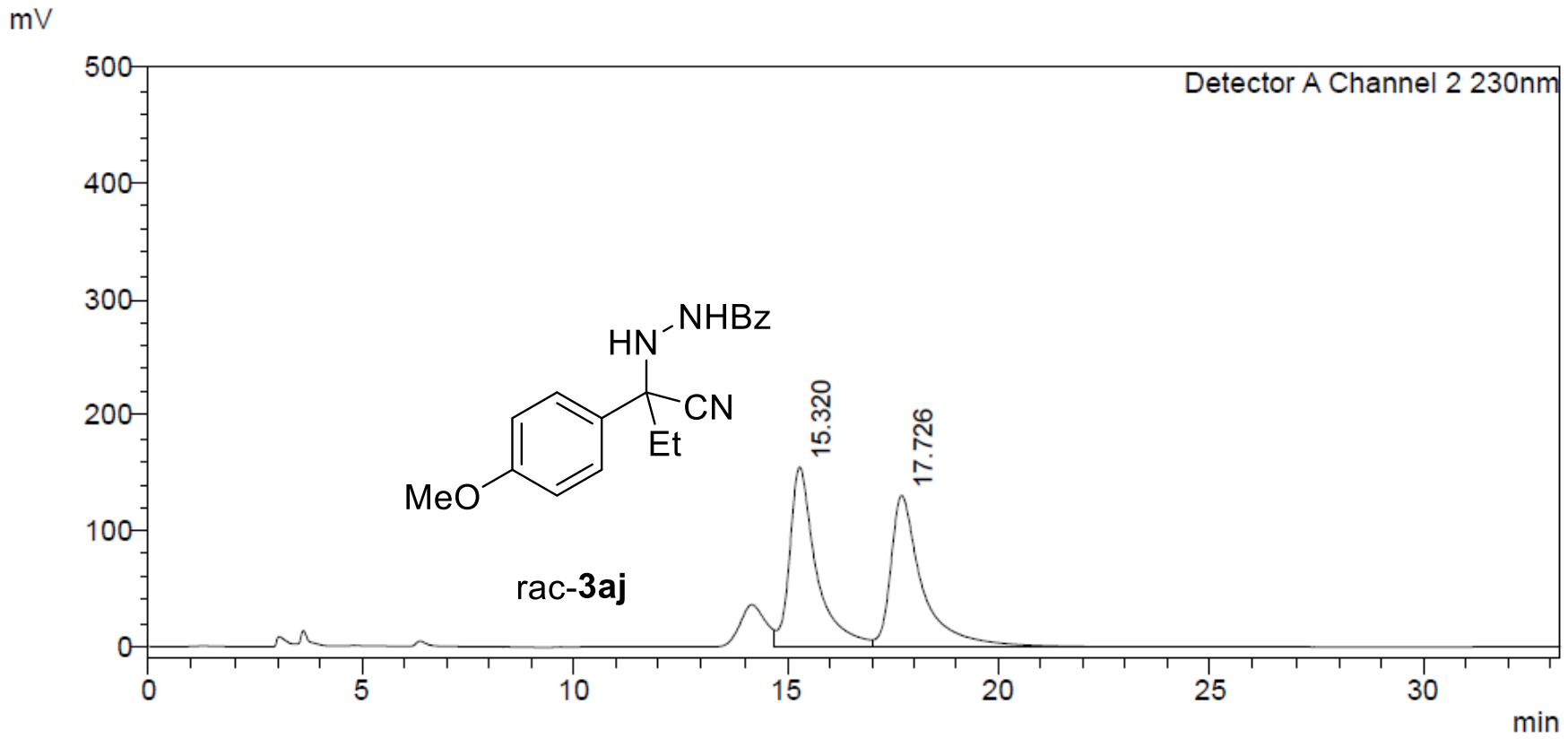

<Peak Table>

Detector A Channel 2 230nm

Peak#	Ret. Time	Area	Height	Conc.
1	15.320	6731035	154994	49.908
2	17.726	6755745	130501	50.092
Total		13486780	285496	

<Sample Information>

Sample Name	: GOY-GG-35-5-asy-ia-8020-1.0-230		
Sample ID	:		
Data Filename	: GOY-GG-35-5-asy-ia-8020-1.0-231.lcd		
Method Filename	: GY-1.0.lcm		
Batch Filename	:		
Vial #	: 1-1	Sample Type	: Unknown
Injection Volume	: 20 uL		
Date Acquired	: 10/11/2020 12:00:14 PM	Acquired by	: System Administrator
Date Processed	: 10/11/2020 12:23:52 PM	Processed by	: System Administrator

<Chromatogram>

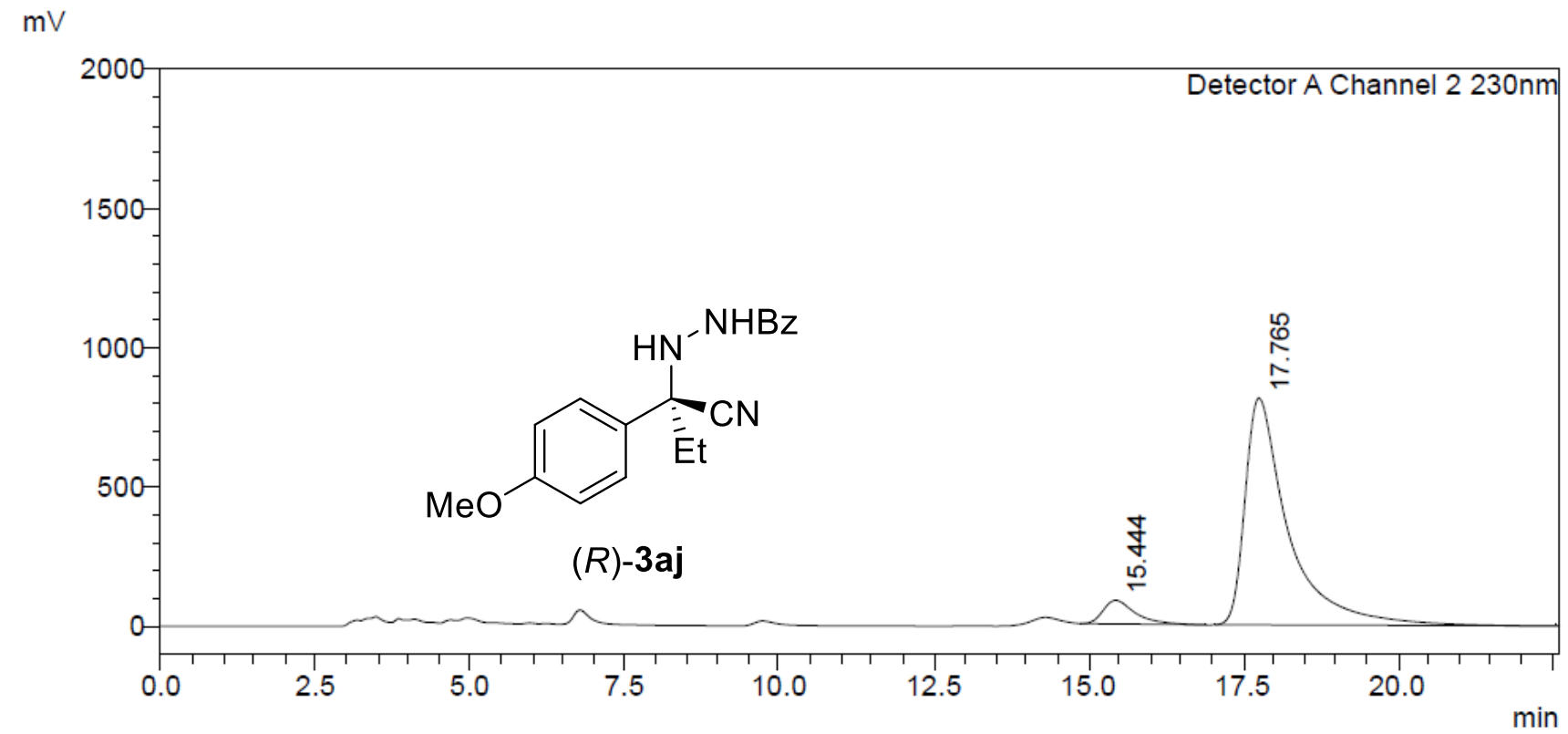

<Peak Table>

Detector A Channel 2 230nm

Peak#	Ret. Time	Area	Height	Conc.
1	15.444	3082197	84543	7.168
2	17.765	39916529	814584	92.832
Total		42998726	899127	

<Sample Information>

Sample Name	: GOY-GG-81-3-rac-ie-8020-1.0-230		
Sample ID	:		
Data Filename	: GOY-GG-81-3-rac-ie-8020-1.0-231.lcd		
Method Filename	: GY-1.0.lcm		
Batch Filename	:		
Vial #	: 1-1	Sample Type	: Unknown
Injection Volume	: 10 uL		
Date Acquired	: 10/27/2020 9:17:06 PM	Acquired by	: System Administrator
Date Processed	: 10/27/2020 9:49:41 PM	Processed by	: System Administrator

<Chromatogram>

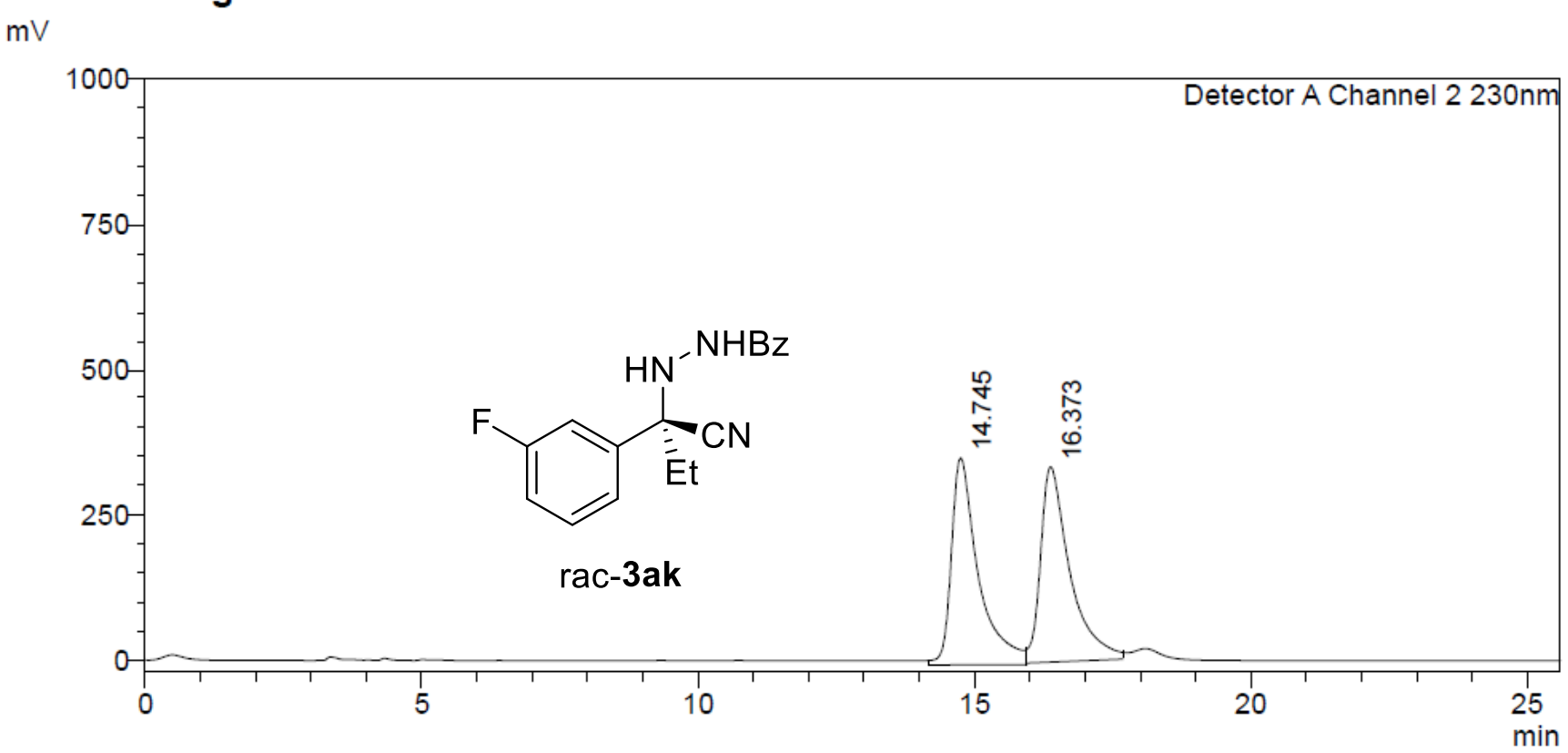

<Peak Table>

Detector A Channel 2 230nm

Peak#	Ret. Time	Area	Height	Conc.
1	14.745	12158221	356405	49.512
2	16.373	12397891	335985	50.488
Total		24556113	692389	

<Sample Information>

Sample Name	: GOY-GG-81-3-asy-ie-8020-1.0-230		
Sample ID	:		
Data Filename	: GOY-GG-81-3-asy-ie-8020-1.0-231.lcd		
Method Filename	: GY-1.0.lcm		
Batch Filename	:		
Vial #	: 1-1	Sample Type	: Unknown
Injection Volume	: 20 uL		
Date Acquired	: 10/27/2020 9:43:58 PM	Acquired by	: System Administrator
Date Processed	: 10/27/2020 10:06:46 PM	Processed by	: System Administrator

<Chromatogram>

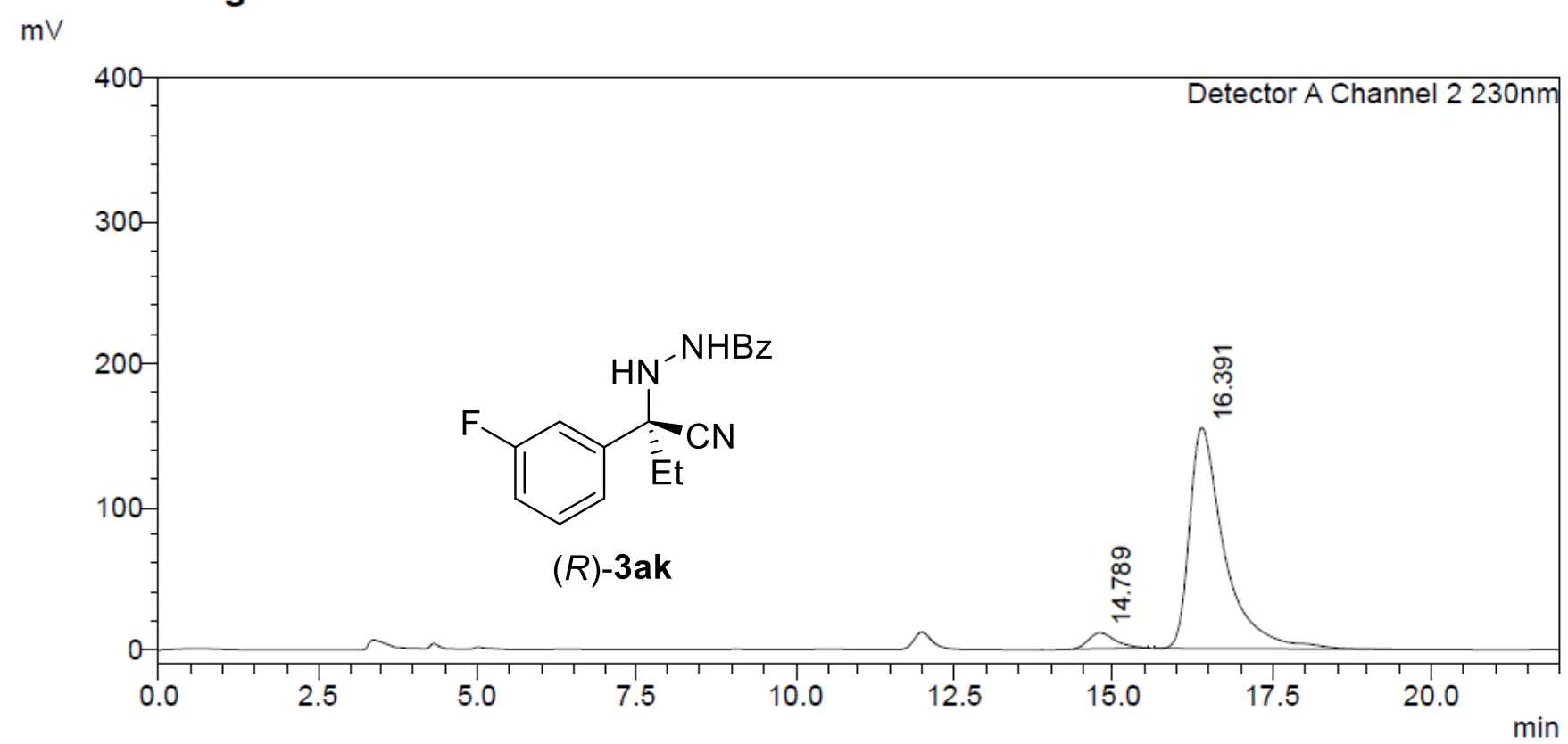

<Peak Table>

Detector A Channel 2 230nm

Peak#	Ret. Time	Area	Height	Conc.
1	14.789	317853	10944	5.108
2	16.391	5904340	154604	94.892
Total		6222193	165548	

<Sample Information>

Sample Name : GOY-GG-112-2-rac-ia-8020-1.0-230
Sample ID :
Data Filename : GOY-GG-112-2-rac-ia-8020-1.0-231.lcd
Method Filename : GY-1.0.lcm
Batch Filename :
Vial # : 1-1 Sample Type : Unknown
Injection Volume : 20 uL
Date Acquired : 11/12/2020 4:08:22 PM Acquired by : System Administrator
Date Processed : 11/12/2020 4:42:10 PM Processed by : System Administrator

<Chromatogram>

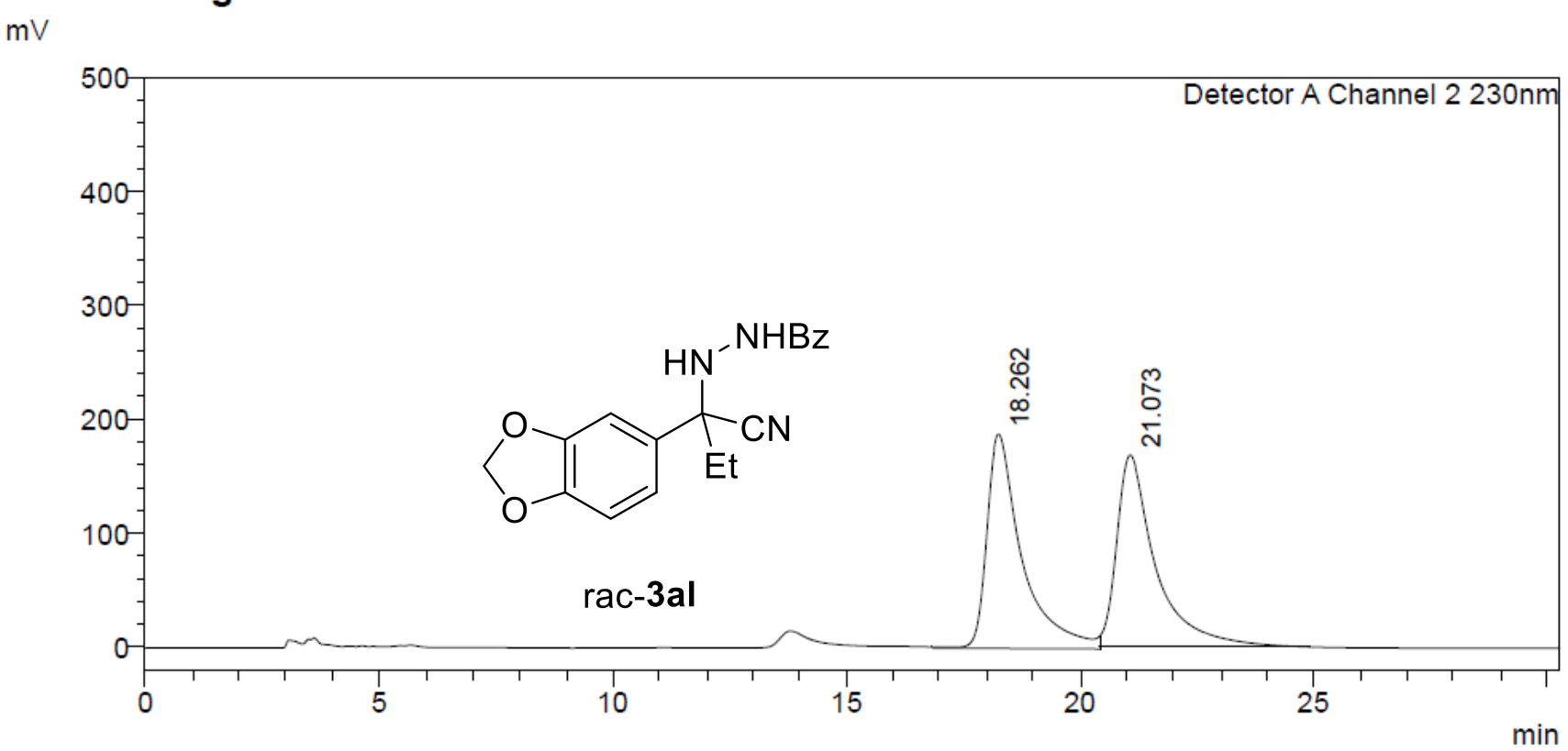

rac-**3al**

<Peak Table>

Detector A Channel 2 230nm

Peak#	Ret. Time	Area	Height	Conc.
1	18.262	9886967	188341	49.981
2	21.073	9894620	168787	50.019
Total		19781587	357128	

<Sample Information>

Sample Name : GOY-GG-112-2-asy-ia-8020-1.0-230
Sample ID :
Data Filename : GOY-GG-112-2-asy-ia-8020-1.0-231.lcd
Method Filename : GY-1.0.lcm
Batch Filename :
Vial # : 1-1 Sample Type : Unknown
Injection Volume : 20 uL
Date Acquired : 11/12/2020 4:40:02 PM Acquired by : System Administrator
Date Processed : 11/12/2020 5:13:01 PM Processed by : System Administrator

<Chromatogram>

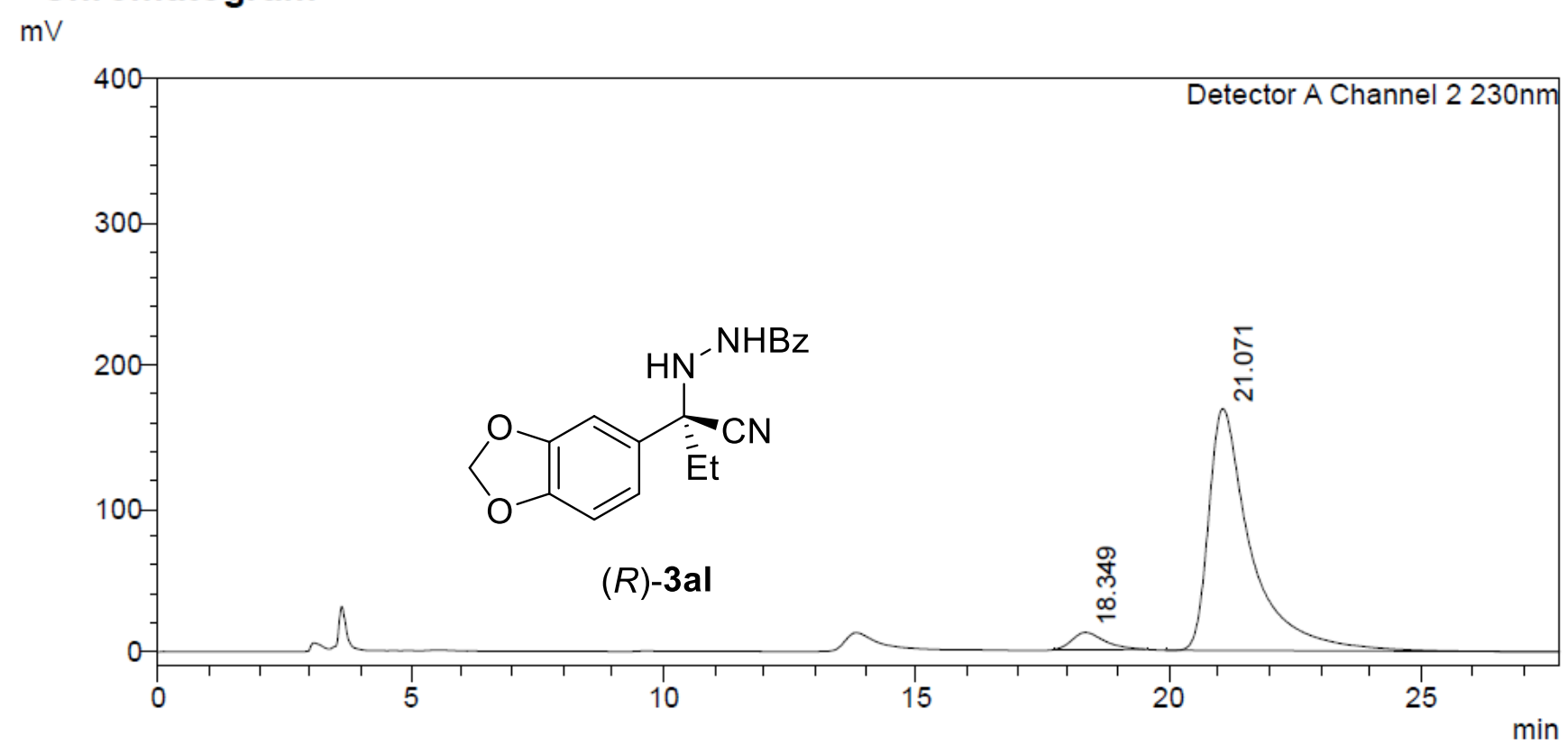

(*R*)-**3al**

<Peak Table>

Detector A Channel 2 230nm

Peak#	Ret. Time	Area	Height	Conc.
1	18.349	528506	11903	5.076
2	21.071	9882369	169071	94.924
Total		10410875	180974	

<Sample Information>

Sample Name	: GOY-GG-89-2-rac-ie-8515-1.0-230		
Sample ID	:		
Data Filename	: GOY-GG-89-2-rac-ie-8515-1.0-231.lcd		
Method Filename	: GY-1.0.lcm		
Batch Filename	:		
Vial #	: 1-1	Sample Type	: Unknown
Injection Volume	: 20 uL		
Date Acquired	: 10/29/2020 3:16:12 PM	Acquired by	: System Administrator
Date Processed	: 10/29/2020 3:55:32 PM	Processed by	: System Administrator

<Chromatogram>

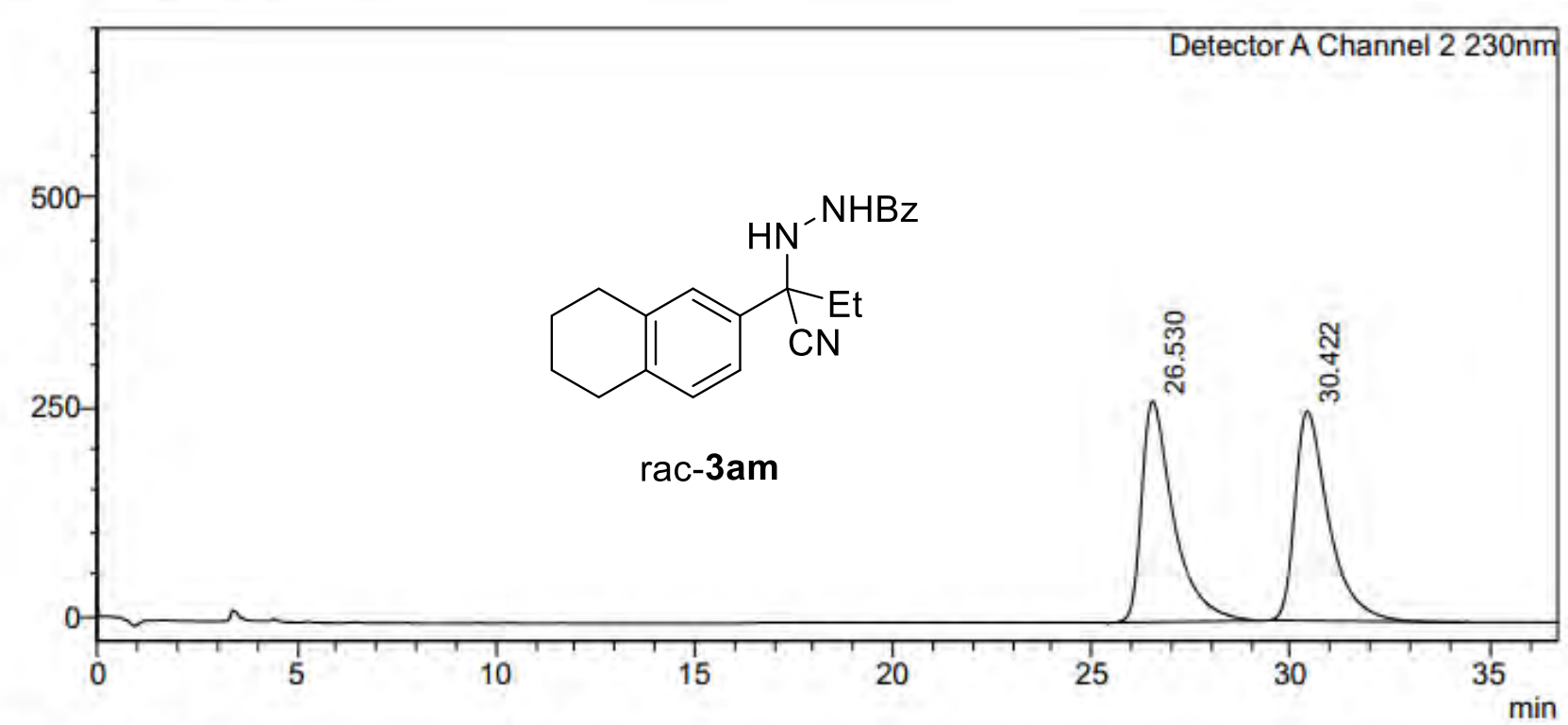

<Peak Table>

Detector A Channel 2 230nm

Peak#	Ret. Time	Area	Height	Conc.
1	26.530	14773717	263069	50.021
2	30.422	14761394	249827	49.979
Total		29535112	512896	

<Sample Information>

Sample Name	: GOY-GP-32-asy-IE-8515-230-1.0		
Sample ID	:		
Data Filename	: GOY-GP-32-asy-IE-8515-230-1.0.lcd		
Method Filename	: GY-1.0.lcm		
Batch Filename	:		
Vial #	: 1-1	Sample Type	: Unknown
Injection Volume	: 20 uL		
Date Acquired	: 7/10/2022 8:49:19 AM	Acquired by	: System Administrator
Date Processed	: 7/10/2022 9:33:52 AM	Processed by	: System Administrator

<Chromatogram>

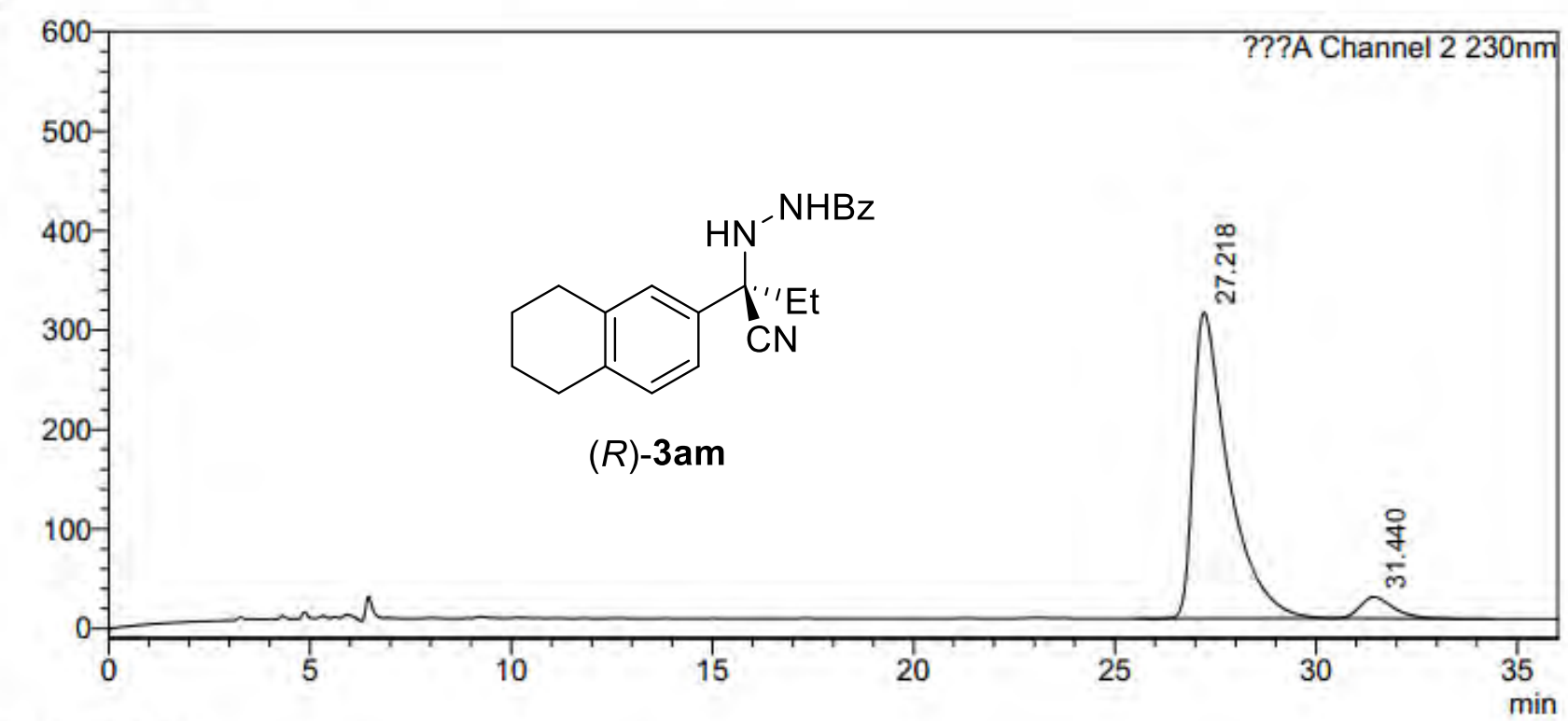

<Peak Table>

???A Channel 2 230nm

Peak#	Ret. Time	Area	Height	Conc.
1	27.218	18723075	308054	93.731
2	31.440	1252329	21569	6.269
Total		19975404	329623	

<Sample Information>

Sample Name	: GOY-GG-81-1-rac-ie-8020-1.0-230		
Sample ID	:		
Data Filename	: GOY-GG-81-1-rac-ie-8020-1.0-231.lcd		
Method Filename	: GY-1.0.lcm		
Batch Filename	:		
Vial #	: 1-1	Sample Type	: Unknown
Injection Volume	: 10 uL		
Date Acquired	: 10/27/2020 4:27:36 PM	Acquired by	: System Administrator
Date Processed	: 10/27/2020 8:31:41 PM	Processed by	: System Administrator

<Chromatogram>

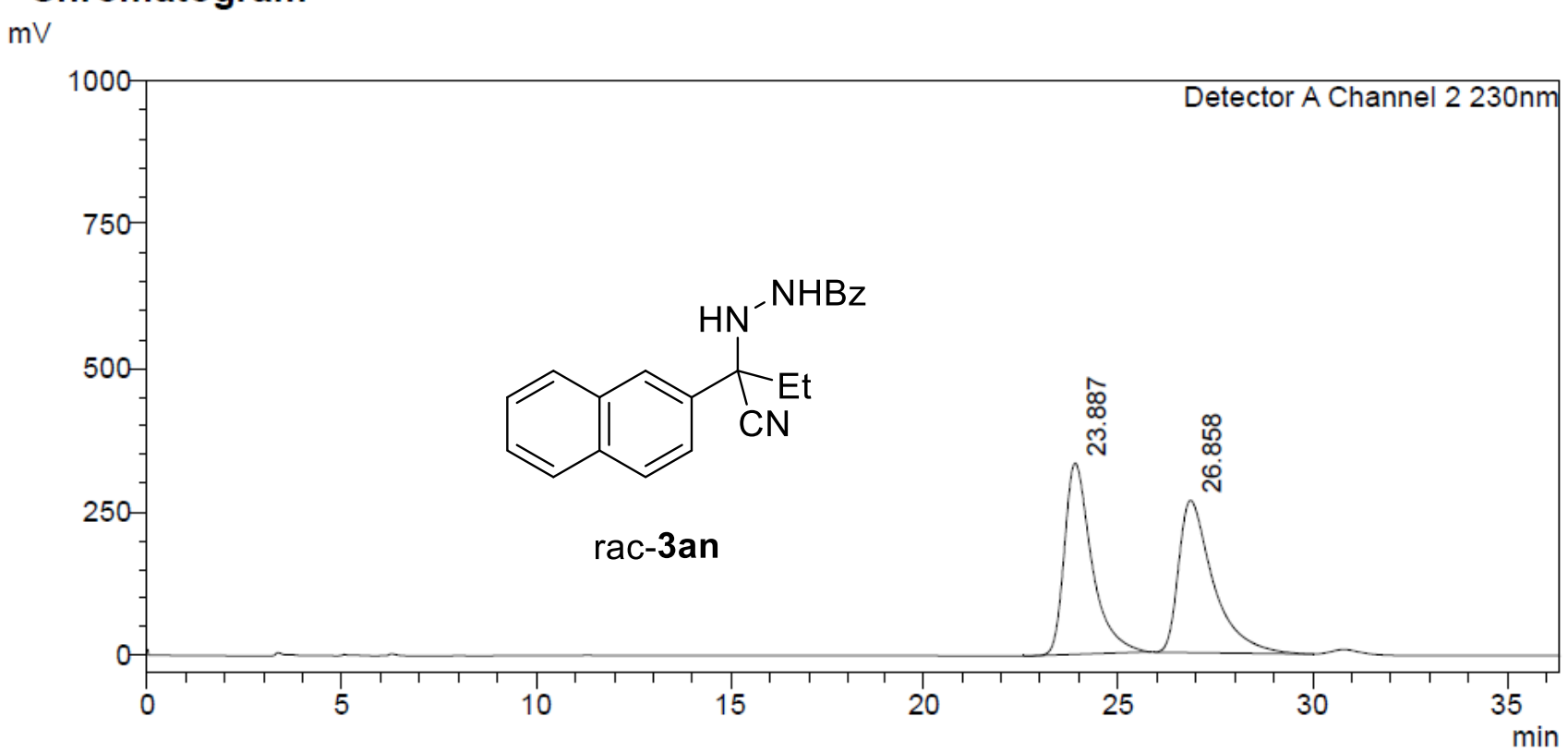

<Peak Table>

Detector A Channel 2 230nm

Peak#	Ret. Time	Area	Height	Conc.
1	23.887	16401933	333043	49.861
2	26.858	16493573	264983	50.139
Total		32895506	598026	

<Sample Information>

Sample Name	: GOY-GG-81-1-asy-ie-8020-1.0-230		
Sample ID	:		
Data Filename	: GOY-GG-81-1-asy-ie-8020-1.0-231.lcd		
Method Filename	: GY-1.0.lcm		
Batch Filename	:		
Vial #	: 1-1	Sample Type	: Unknown
Injection Volume	: 20 uL		
Date Acquired	: 10/27/2020 8:30:26 PM	Acquired by	: System Administrator
Date Processed	: 10/27/2020 9:05:24 PM	Processed by	: System Administrator

<Chromatogram>

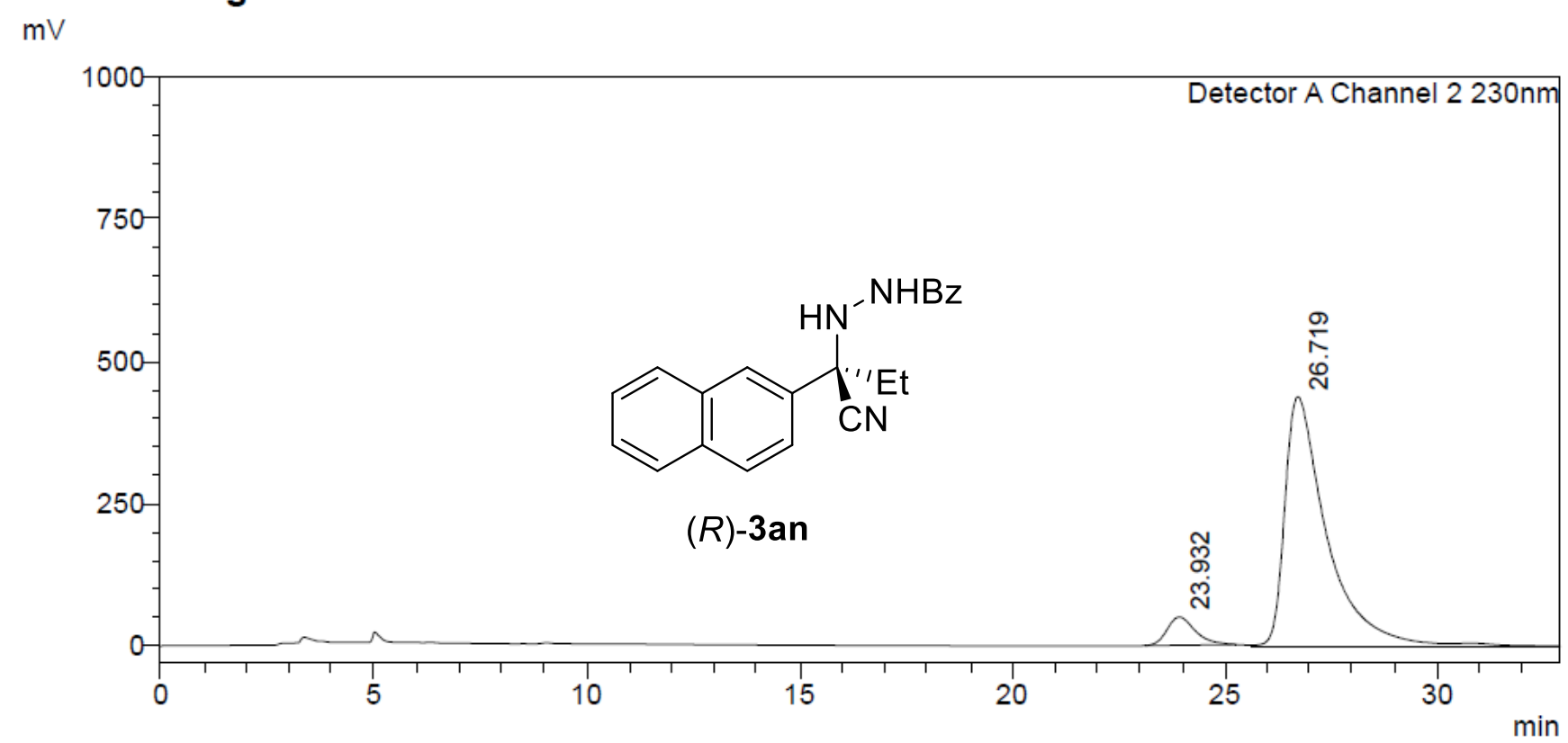

<Peak Table>

Detector A Channel 2 230nm

Peak#	Ret. Time	Area	Height	Conc.
1	23.932	2291864	49714	7.079
2	26.719	30083759	438703	92.921
Total		32375623	488417	

<Sample Information>

Sample Name	: ZHZ-ZAD-FN-ADH-RAC-70-30-1.0-230-254-		
Sample ID	:		
Data Filename	: ZHZ-ZAD-FN-ADH-RAC-70-30-1.0-230--254.lcd		
Method Filename	: GY-1.0.lcm		
Batch Filename	:		
Vial #	: 1-1	Sample Type	: Unknown
Injection Volume	: 20 uL		
Date Acquired	: 4/17/2026 12:28:11 AM	Acquired by	: System Administrator
Date Processed	: 4/17/2026 12:50:48 AM	Processed by	: System Administrator

<Chromatogram>

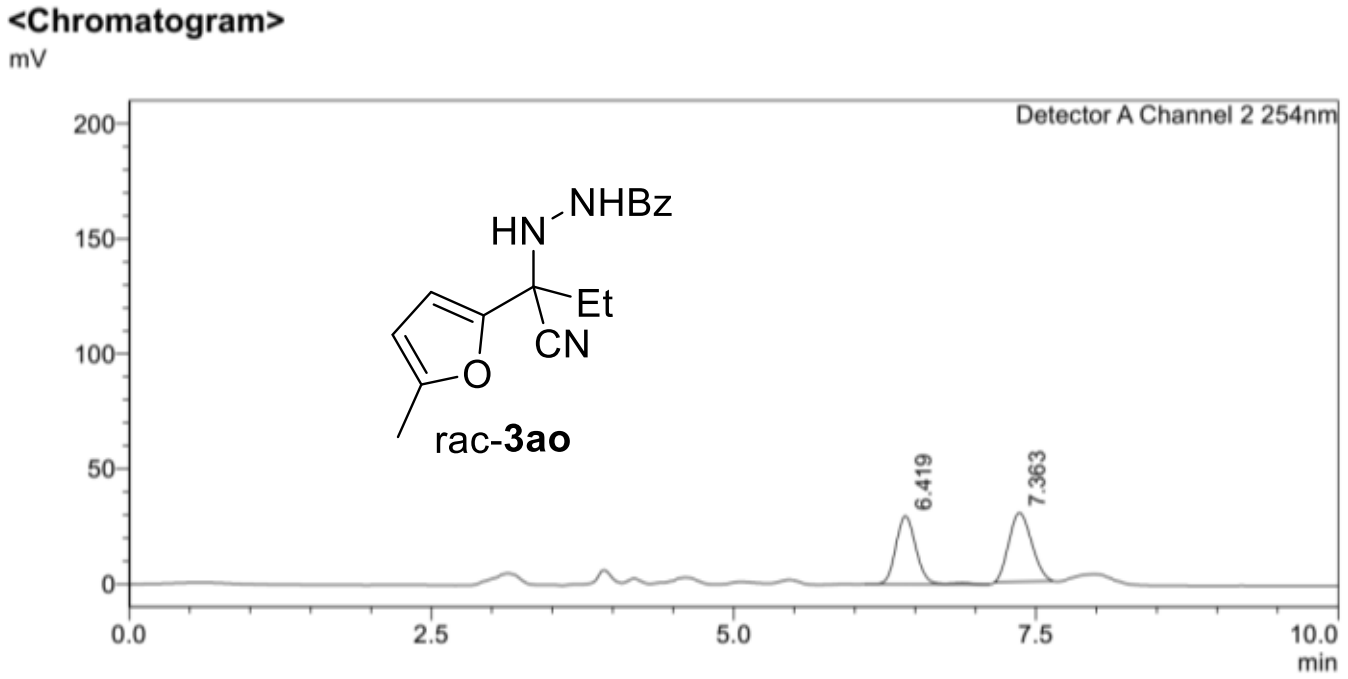

<Peak Table>

Detector A Channel 2 254nm

Peak#	Ret. Time	Area	Height	Conc.
1	6.419	341908	29590	46.527
2	7.363	392945	29922	53.473
Total		734854	59511	

C:\Data\ZHZ\ZHZ-ZAD-FN-ADH-RAC-70-30-1.0-230--254.lcd

<Sample Information>

Sample Name	: ZHZ-ZAD-FN-ADH-ASY-70-30-1.0-230-254-		
Sample ID	:		
Data Filename	: ZHZ-ZAD-FN-ADH-ASY-70-30-1.0-230--254.lcd		
Method Filename	: GY-1.0.lcm		
Batch Filename	:		
Vial #	: 1-1	Sample Type	: Unknown
Injection Volume	: 20 uL		
Date Acquired	: 4/17/2026 12:16:34 AM	Acquired by	: System Administrator
Date Processed	: 4/17/2026 12:26:05 AM	Processed by	: System Administrator

<Chromatogram>

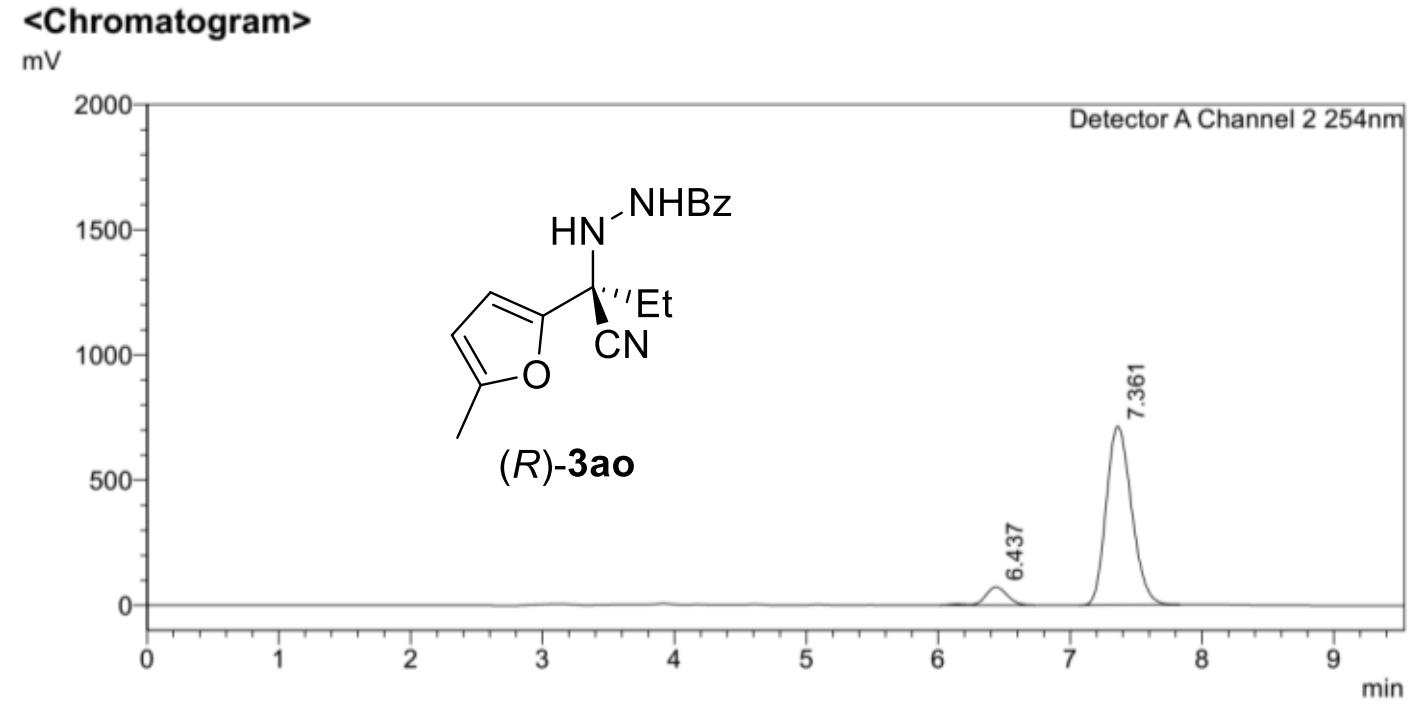

<Peak Table>

Detector A Channel 2 254nm

Peak#	Ret. Time	Area	Height	Conc.
1	6.437	860914	72569	8.270
2	7.361	9548631	713663	91.730
Total		10409546	786232	

C:\Data\ZHZ\ZHZ-ZAD-FN-ADH-ASY-70-30-1.0-230--254.lcd

<Sample Information>

Sample Name	: ZHZ-ZAD-56-RAC-ADH-80-20--1.0-254-230-
Sample ID	:
Data Filename	: ZHZ-ZAD--56-RAC--ADH-80-20--1.0-254-230.lcd
Method Filename	: GY-1.0.lcm
Batch Filename	:
Vial #	: 1-1
Sample Type	: Unknown
Injection Volume	: 20 uL
Date Acquired	: 4/13/2026 10:03:50 PM
Acquired by	: System Administrator
Date Processed	: 4/13/2026 10:31:41 PM
Processed by	: System Administrator

<Chromatogram>

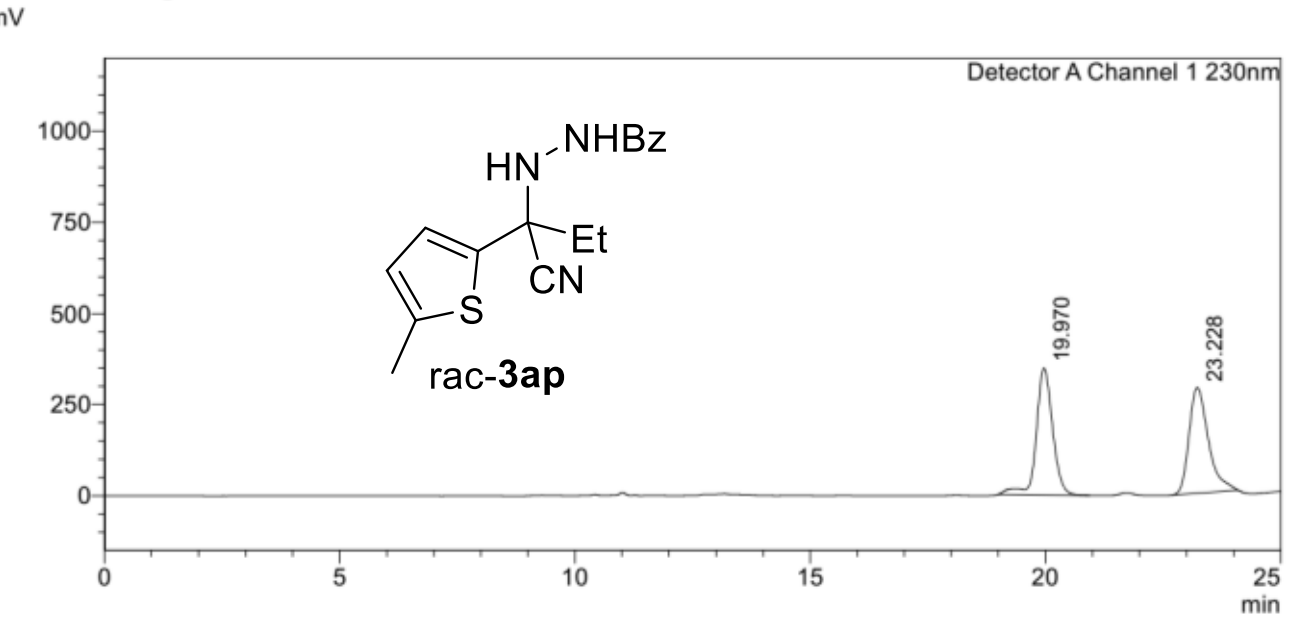

<Peak Table>

Detector A Channel 1 230nm

Peak#	Ret. Time	Area	Height	Conc.
1	19.970	8461111	347303	50.477
2	23.228	8301174	289942	49.523
Total		16762285	637245	

C:\Data\ZHZ\ZHZ-ZAD--56-RAC--ADH-80-20--1.0-254-230.lcd

<Sample Information>

Sample Name	: ZHZ-ZAD-56-ASY-ADH-80-20--1.0-254-230-
Sample ID	:
Data Filename	: ZHZ-ZAD--56-ASY--ADH-80-20--1.0-254-230.lcd
Method Filename	: GY-1.0.lcm
Batch Filename	:
Vial #	: 1-1
Sample Type	: Unknown
Injection Volume	: 20 uL
Date Acquired	: 4/13/2026 9:25:22 PM
Acquired by	: System Administrator
Date Processed	: 4/13/2026 10:10:04 PM
Processed by	: System Administrator

<Chromatogram>

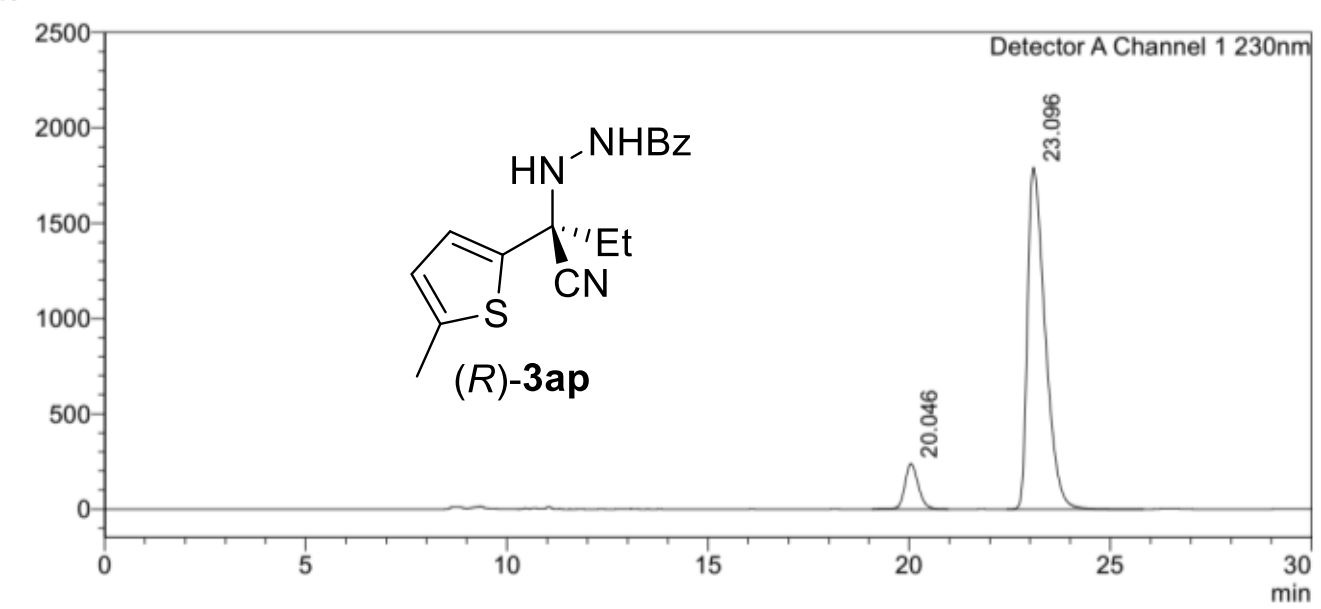

<Peak Table>

Detector A Channel 1 230nm

Peak#	Ret. Time	Area	Height	Conc.
1	20.046	5536113	237028	9.304
2	23.096	53968189	1792558	90.696
Total		59504302	2029586	

C:\Data\ZHZ\ZHZ-ZAD--56-ASY--ADH-80-20--1.0-254-230.lcd

<Sample Information>

Sample Name	: ZHZ-ZAD-54-RAC-ADH-80-20--1.0-254-230		
Sample ID	:		
Data Filename	: ZHZ-ZAD-54-RAC-ADH-80-20--1.0-254-230.lcd		
Method Filename	: GY-1.0.lcm		
Batch Filename	:		
Vial #	: 1-1	Sample Type	: Unknown
Injection Volume	: 20 uL		
Date Acquired	: 4/13/2026 2:51:14 PM	Acquired by	: System Administrator
Date Processed	: 4/13/2026 3:08:36 PM	Processed by	: System Administrator

<Chromatogram>

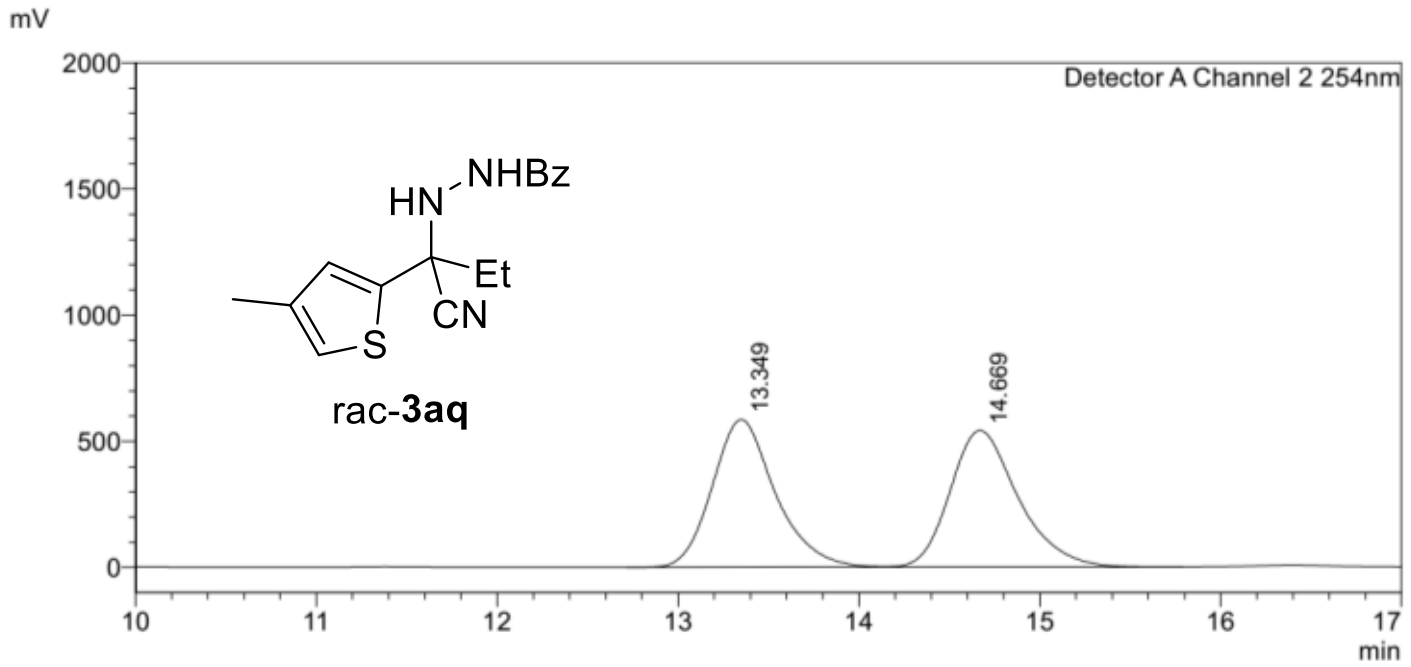

<Peak Table>

Detector A Channel 2 254nm

Peak#	Ret. Time	Area	Height	Conc.
1	13.349	14230240	585657	50.023
2	14.669	14216948	541971	49.977
Total		28447188	1127628	

C:\Data\ZHZ\ZHZ-ZAD-54-RAC-ADH-80-20--1.0-254-230.lcd

<Sample Information>

Sample Name	: zhz-zad-54-ASY-ADH-80-20-230-254-1.0		
Sample ID	:		
Data Filename	: zhz-zad-54-ASY-ADH-80-20-230-254-1.0.lcd		
Method Filename	: GY-1.0.lcm		
Batch Filename	:		
Vial #	: 1-1	Sample Type	: Unknown
Injection Volume	: 20 uL		
Date Acquired	: 4/8/2026 10:00:23 AM	Acquired by	: System Administrator
Date Processed	: 4/8/2026 10:17:27 AM	Processed by	: System Administrator

<Chromatogram>

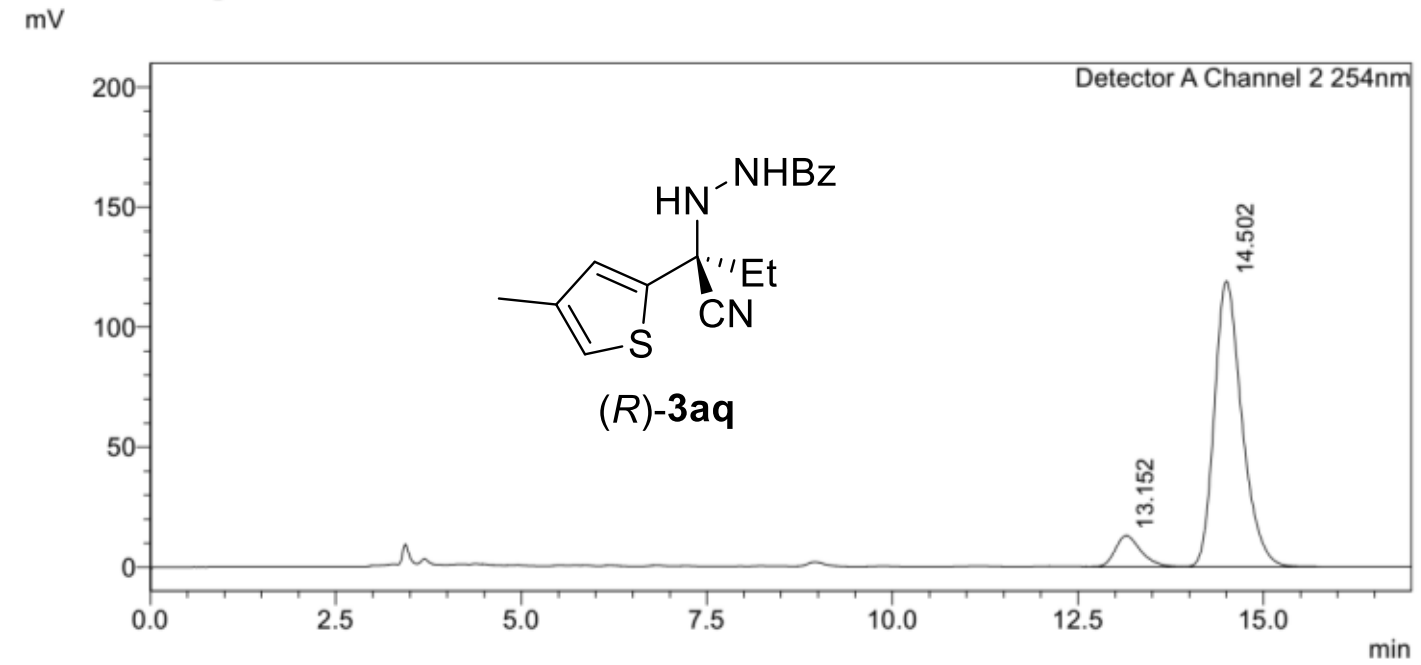

<Peak Table>

Detector A Channel 2 254nm

Peak#	Ret. Time	Area	Height	Conc.
1	13.152	315020	12959	9.265
2	14.502	3085000	119061	90.735
Total		3400020	132020	

C:\Data\ZHZ\zhz-zad-54-ASY-ADH-80-20-230-254-1.0.lcd

<Sample Information>

Sample Name	: GOY-GG-134-2-rac-ia-8020-1.0-231.lcd
Sample ID	:
Data Filename	: GOY-GG-134-2-rac-ia-8020-1.0-233.lcd
Method Filename	: ZXX-ZD-133-2.lcm
Batch Filename	:
Vial #	: 1-1
Sample Type	: Unknown
Injection Volume	: 20 uL
Date Acquired	: 11/28/2020 8:44:52 AM
Acquired by	: System Administrator
Date Processed	: 11/28/2020 9:25:33 AM
Processed by	: System Administrator

<Chromatogram>

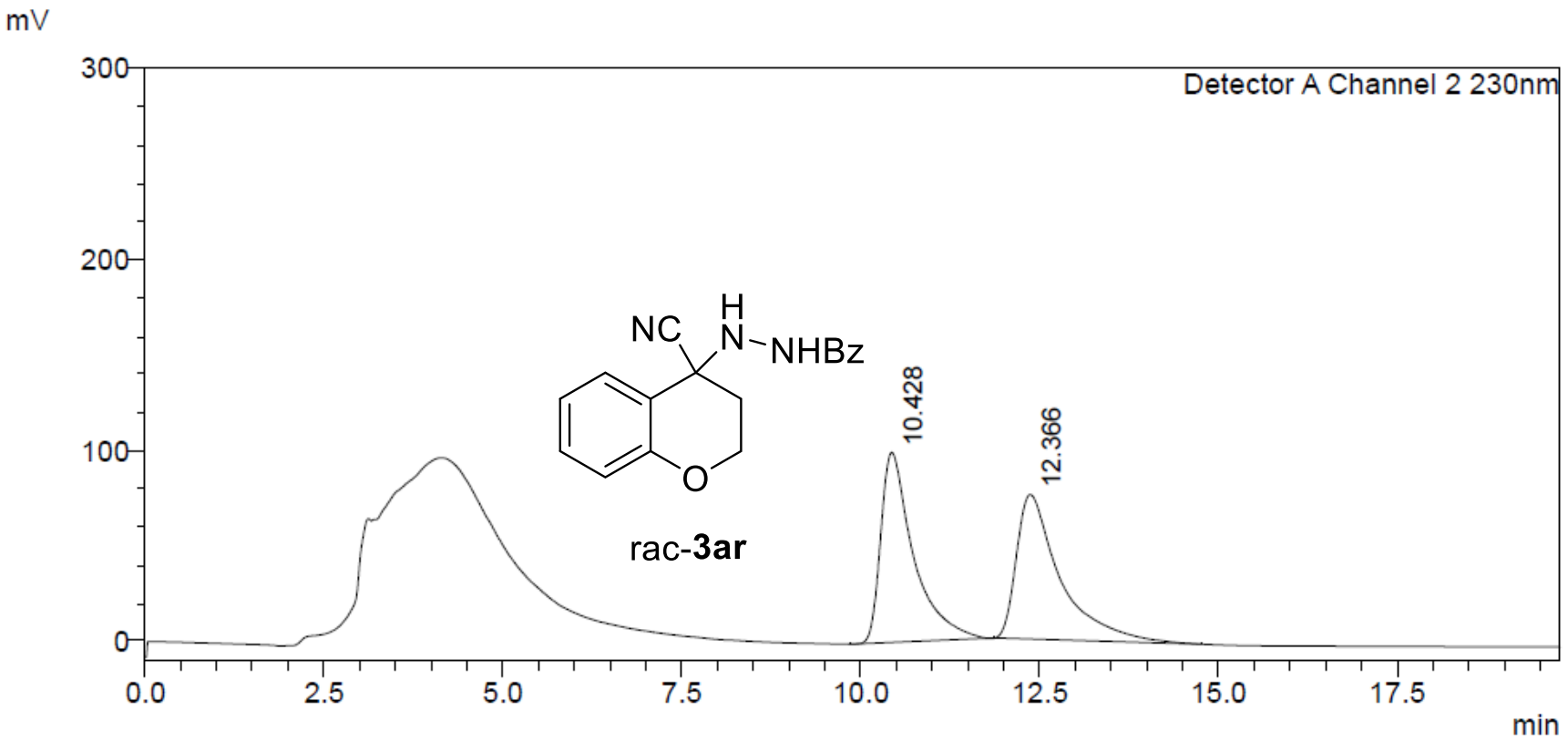

<Peak Table>

Detector A Channel 2 230nm

Peak#	Ret. Time	Area	Height	Conc.
1	10.428	3171234	99506	49.645
2	12.366	3216637	75648	50.355
Total		6387871	175153	

<Sample Information>

Sample Name	: GOY-GG-134-2-asy-ia-8020-1.0-230
Sample ID	:
Data Filename	: GOY-GG-134-2-asy-ia-8020-1.0-231.lcd
Method Filename	: ZXX-ZD-133-2.lcm
Batch Filename	:
Vial #	: 1-1
Sample Type	: Unknown
Injection Volume	: 20 uL
Date Acquired	: 11/28/2020 9:06:20 AM
Acquired by	: System Administrator
Date Processed	: 11/28/2020 9:34:34 AM
Processed by	: System Administrator

<Chromatogram>

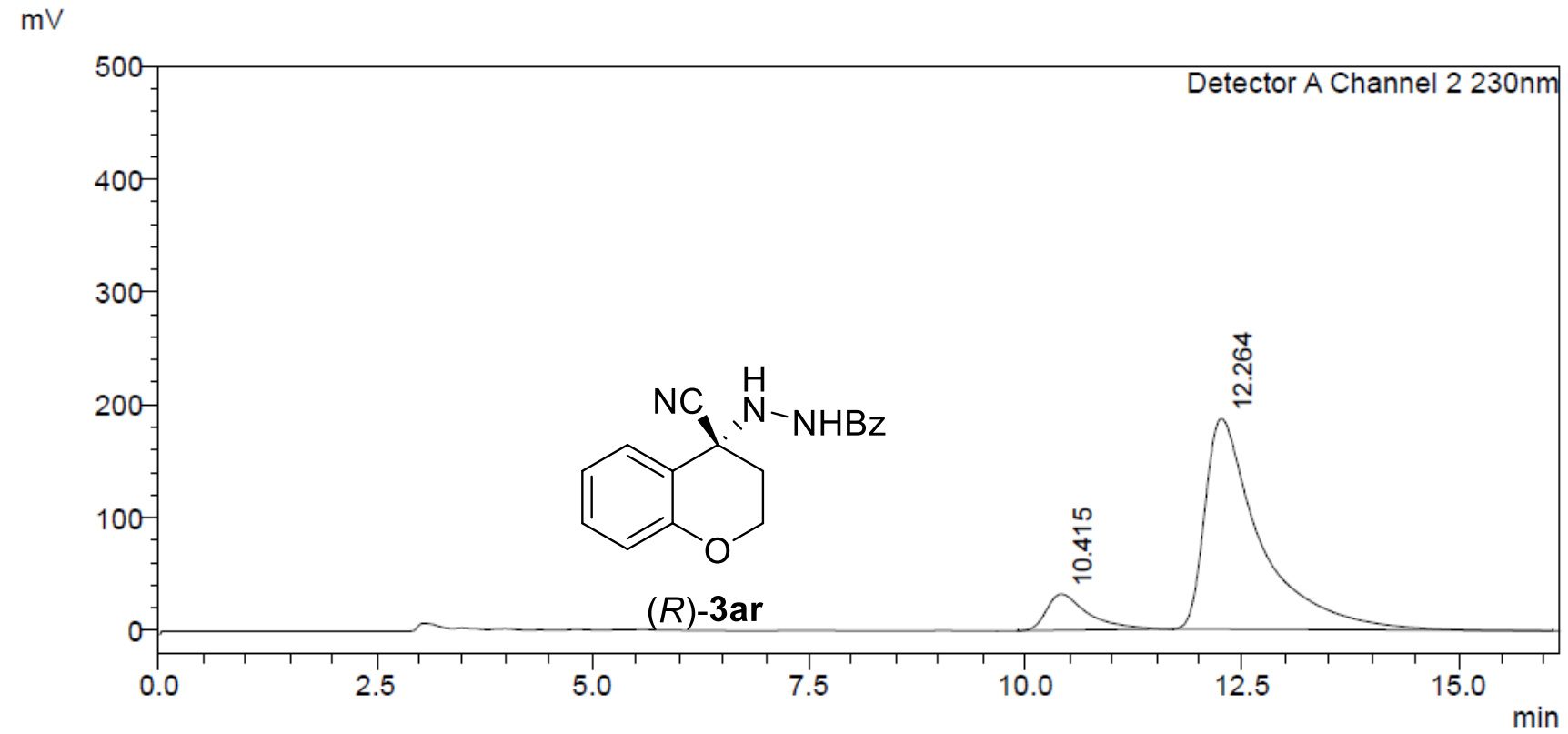

<Peak Table>

Detector A Channel 2 230nm

Peak#	Ret. Time	Area	Height	Conc.
1	10.415	1067289	32150	11.297
2	12.264	8380415	186782	88.703
Total		9447704	218932	

<Sample Information>

Sample Name : GOY-GP-44-rac-IA-8020-230-1.0-Data
Sample ID :
Data Filename : GOY-GP-44-rac-IE-8020-230-1.0-Data.lcd
Method Filename : GY-1.0.lcm
Batch Filename :
Vial # : 1-1
Injection Volume : 20 uL
Date Acquired : 7/21/2022 2:39:03 PM
Date Processed : 7/21/2022 5:16:40 PM
Sample Type : Unknown
Acquired by : System Administrator
Processed by : System Administrator

<Chromatogram>

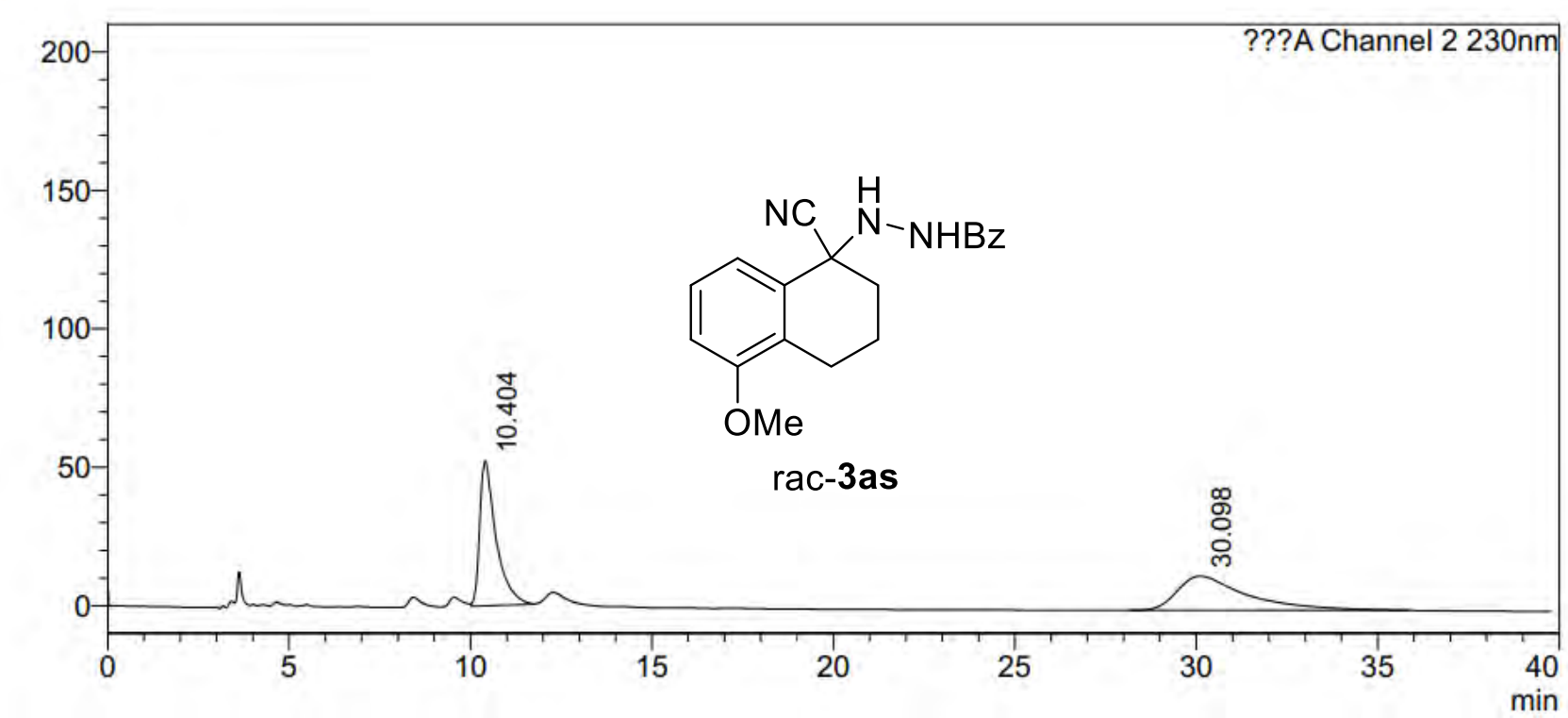

<Peak Table>

???A Channel 2 230nm

Peak#	Ret. Time	Area	Height	Conc.
1	10.404	1641511	52399	49.872
2	30.098	1649940	12353	50.128
Total		3291451	64752	

<Sample Information>

Sample Name : GOY-GP-44-asy-IA-8020-230-1.0-Data
Sample ID :
Data Filename : GOY-GP-44-asy-IE-8020-230-1.0-Data.lcd
Method Filename : GY-1.0.lcm
Batch Filename :
Vial # : 1-1
Injection Volume : 20 uL
Date Acquired : 7/21/2022 4:14:06 PM
Date Processed : 7/21/2022 5:19:32 PM
Sample Type : Unknown
Acquired by : System Administrator
Processed by : System Administrator

<Chromatogram>

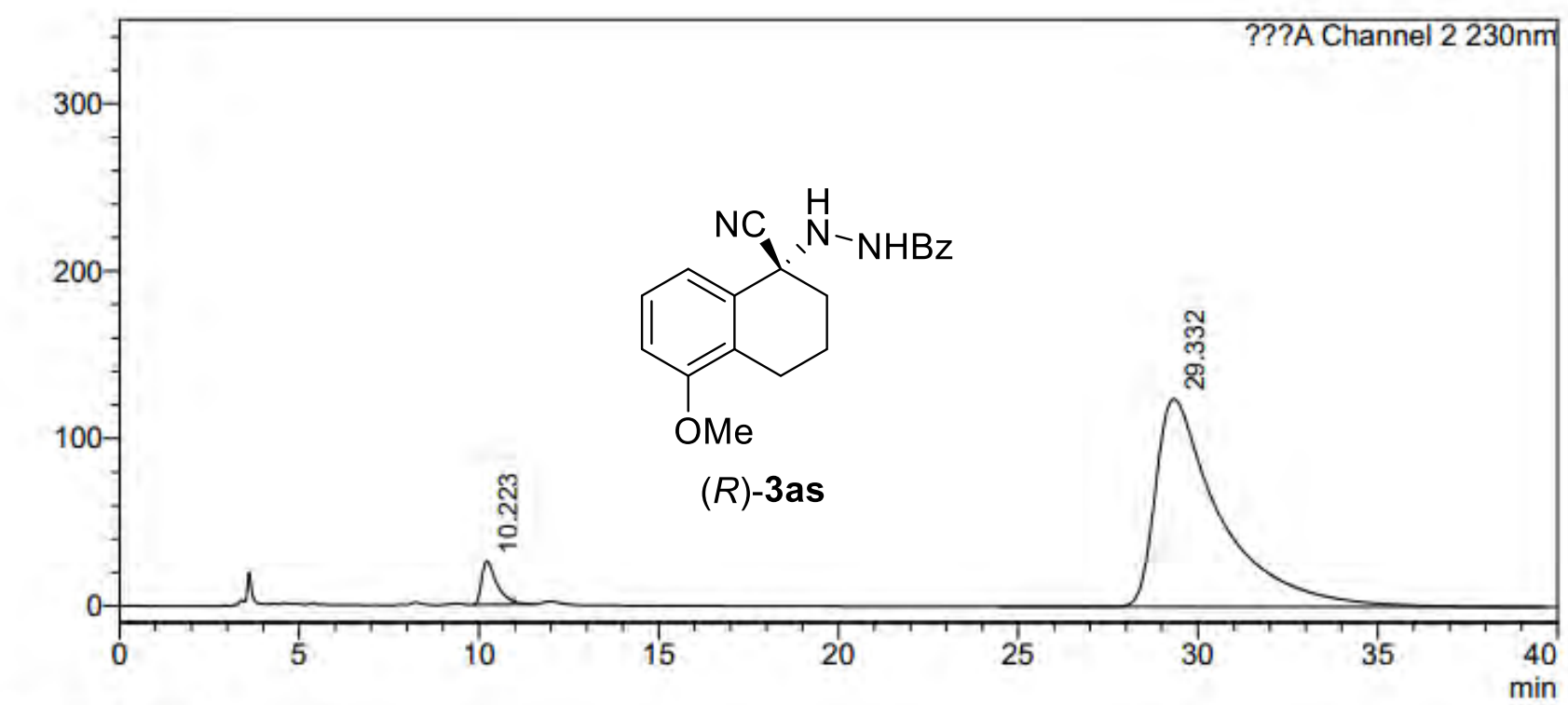

<Peak Table>

???A Channel 2 230nm

Peak#	Ret. Time	Area	Height	Conc.
1	10.223	767440	26173	4.692
2	29.332	15589796	124211	95.308
Total		16357235	150384	

<Sample Information>

Sample Name : GOY-GP-46-rac-IA-8020-230-1.0-DATA
Sample ID :
Data Filename : GOY-GP-46-rac-IA-8020-230-1.0-DATA.lcd
Method Filename : GY-1.0.lcm
Batch Filename :
Vial # : 1-1
Injection Volume : 20 uL
Date Acquired : 10/24/2022 8:04:24 PM
Date Processed : 10/24/2022 8:26:00 PM
Sample Type : Unknown
Acquired by : System Administrator
Processed by : System Administrator

<Chromatogram>

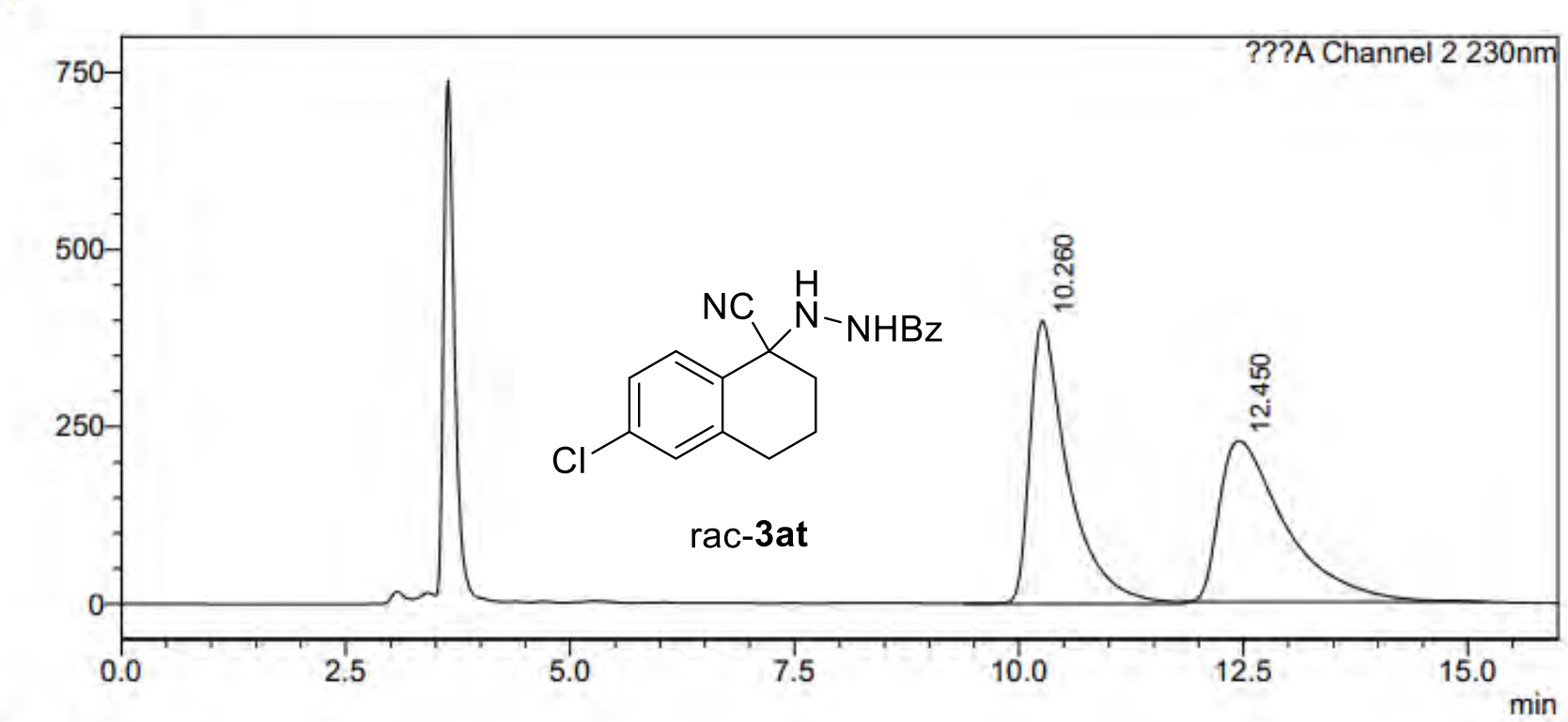

<Peak Table>

???A Channel 2 230nm

Peak#	Ret. Time	Area	Height	Conc.
1	10.260	11893338	398003	50.257
2	12.450	11771907	227399	49.743
Total		23665245	625402	

<Sample Information>

Sample Name : GOY-GP-46-asy-IA-8020-230-1.0.lcd
Sample ID :
Data Filename : GOY-GP-46-asy-IA-8020-230-1.0-DATA.lcd
Method Filename : GY-1.0.lcm
Batch Filename :
Vial # : 1-1
Injection Volume : 20 uL
Date Acquired : 7/16/2022 2:25:17 PM
Date Processed : 7/16/2022 2:44:13 PM
Sample Type : Unknown
Acquired by : System Administrator
Processed by : System Administrator

<Chromatogram>

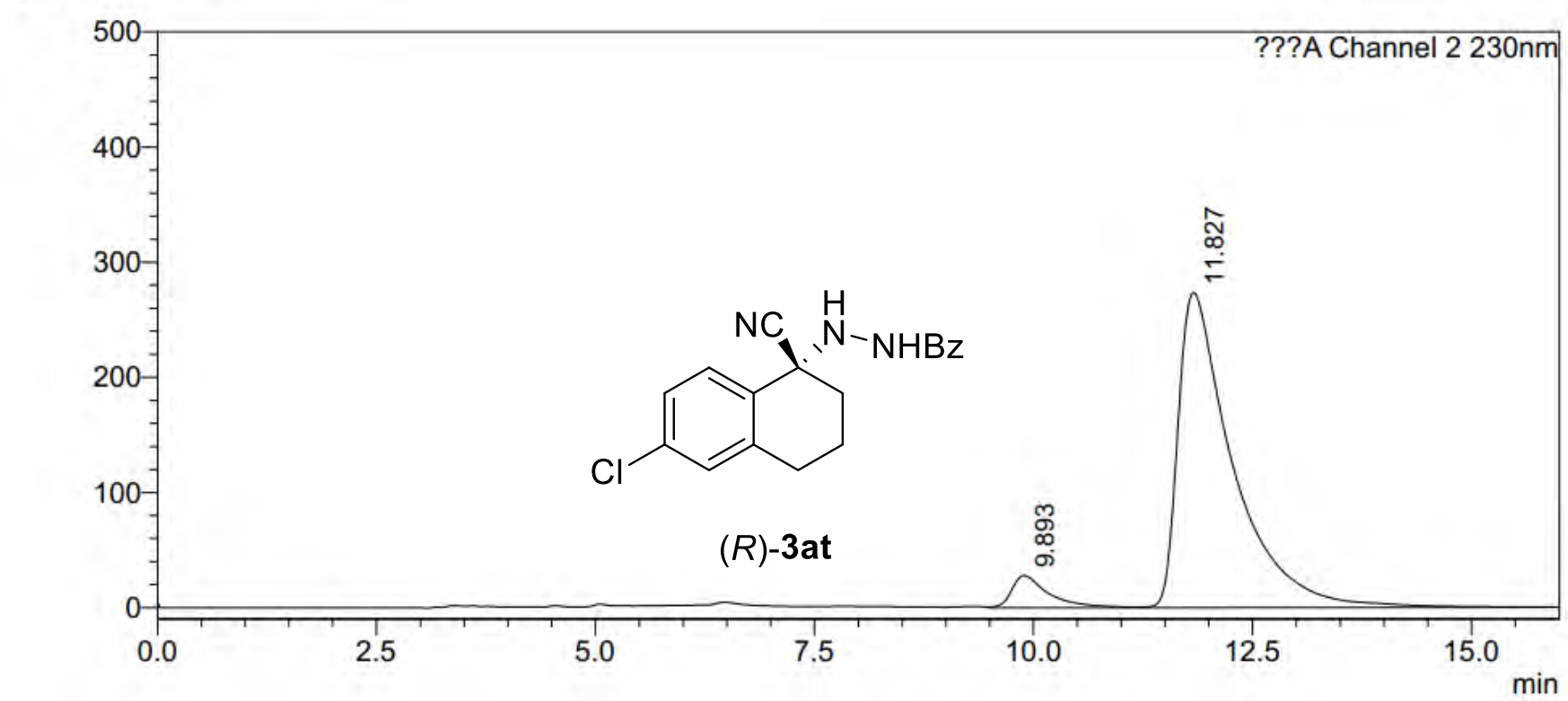

<Peak Table>

???A Channel 2 230nm

Peak#	Ret. Time	Area	Height	Conc.
1	9.893	819793	27976	6.433
2	11.827	11924064	273445	93.567
Total		12743857	301421	

<Sample Information>

Sample Name : GOY-GF-123-6-rac-ie-6040-1.0-230
Sample ID :
Data Filename : GOY-GF-117-6-rac-ie-6040-1.0-230.lcd
Method Filename : GY-1.0.lcm
Batch Filename :
Vial # : 1-1 Sample Type : Unknown
Injection Volume : 20 uL
Date Acquired : 8/13/2020 10:00:35 PM Acquired by : System Administrator
Date Processed : 8/14/2020 10:42:28 AM Processed by : System Administrator

<Chromatogram>

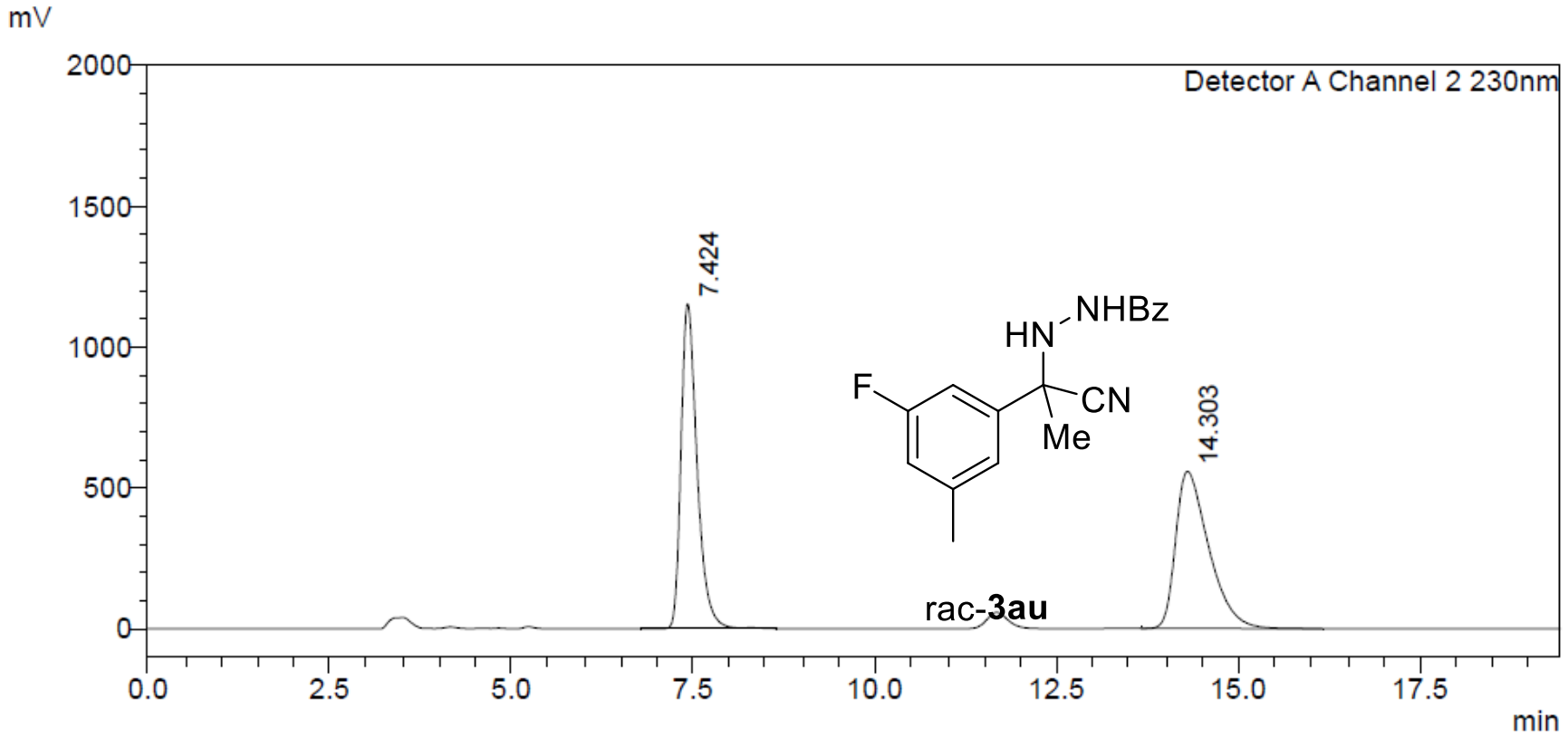

<Peak Table>

Detector A Channel 2 230nm

Peak#	Ret. Time	Area	Height	Conc.
1	7.424	17725774	1153150	50.058
2	14.303	17684910	558234	49.942
Total		35410683	1711384	

<Sample Information>

Sample Name : GOY-GF-123-6-asy-ie-6040-1.0-230
Sample ID :
Data Filename : GOY-GF-117-6-asy-ie-6040-1.0-230.lcd
Method Filename : GY-1.0.lcm
Batch Filename :
Vial # : 1-1 Sample Type : Unknown
Injection Volume : 20 uL
Date Acquired : 8/13/2020 10:20:50 PM Acquired by : System Administrator
Date Processed : 8/14/2020 10:43:51 AM Processed by : System Administrator

<Chromatogram>

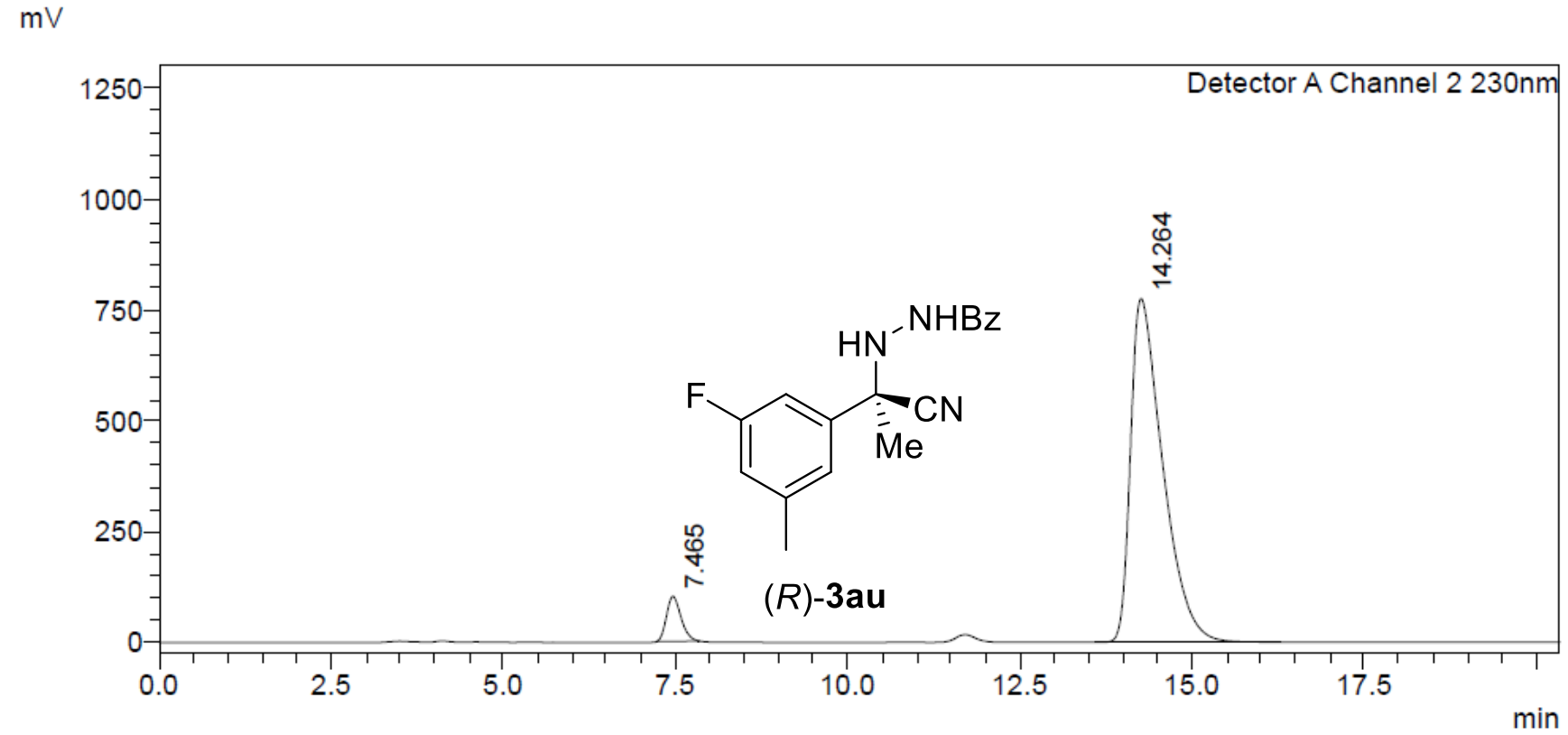

<Peak Table>

Detector A Channel 2 230nm

Peak#	Ret. Time	Area	Height	Conc.
1	7.465	1491487	101734	5.575
2	14.264	25259398	775596	94.425
Total		26750885	877330	

<Sample Information>

Sample Name	: GOY-GF-131-5-rac-ie-8020-1.0-230
Sample ID	:
Data Filename	: GOY-GF-131-5-rac-ie-8020-1.0-230.lcd
Method Filename	: GY-1.0.lcm
Batch Filename	:
Vial #	: 1-1
Injection Volume	: 20 uL
Date Acquired	: 8/29/2020 1:51:25 PM
Date Processed	: 8/29/2020 2:24:57 PM
Sample Type	: Unknown
Acquired by	: System Administrator
Processed by	: System Administrator

<Chromatogram>

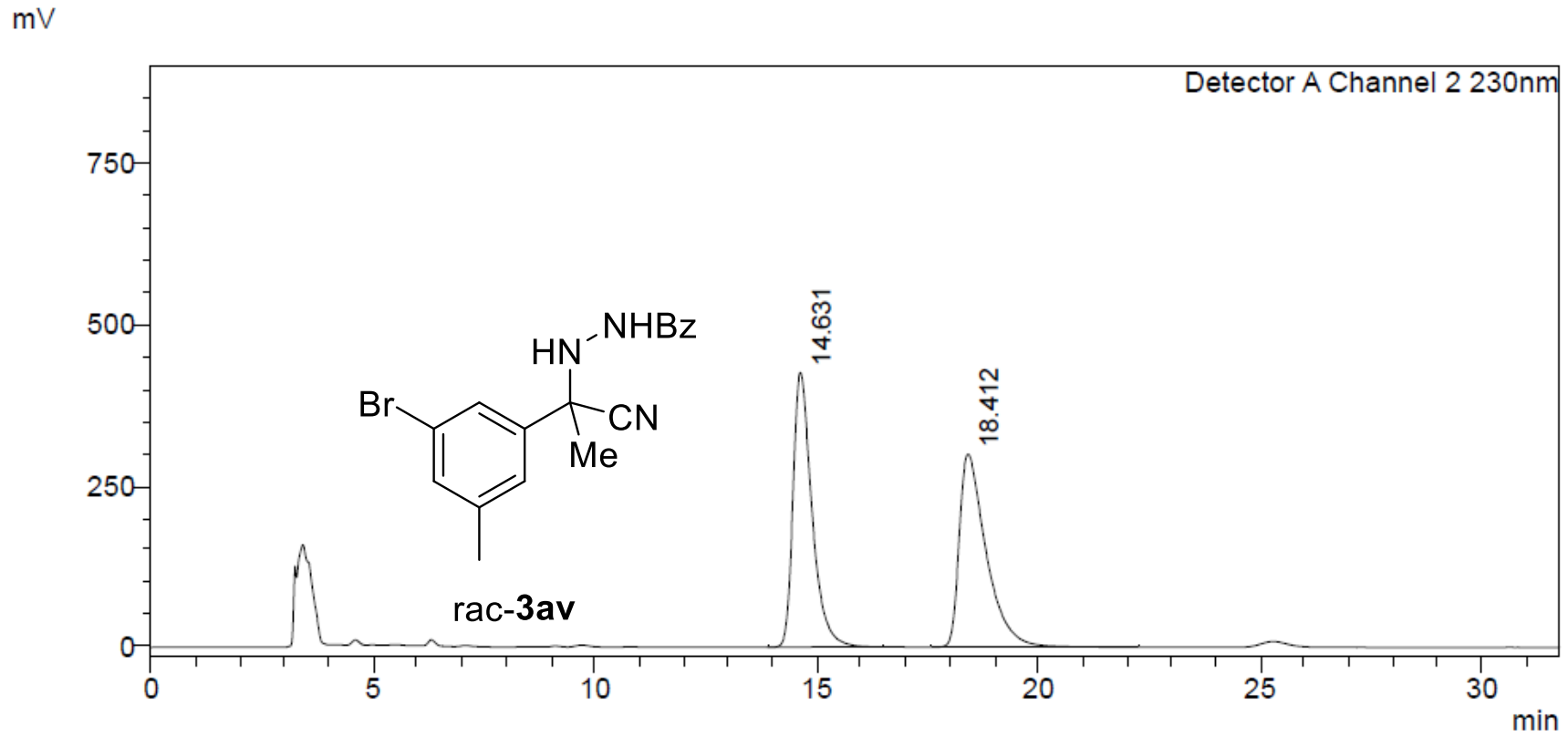

<Peak Table>

Detector A Channel 2 230nm

Peak#	Ret. Time	Area	Height	Conc.
1	14.631	12649095	426110	49.967
2	18.412	12666013	299241	50.033
Total		25315108	725351	

<Sample Information>

Sample Name	: GOY-GF-131-5-asy-ie-8020-1.0-230
Sample ID	:
Data Filename	: GOY-GF-131-5-asy-ie-8020-1.0-230.lcd
Method Filename	: GY-1.0.lcm
Batch Filename	:
Vial #	: 1-1
Injection Volume	: 20 uL
Date Acquired	: 8/29/2020 2:23:49 PM
Date Processed	: 8/29/2020 2:49:24 PM
Sample Type	: Unknown
Acquired by	: System Administrator
Processed by	: System Administrator

<Chromatogram>

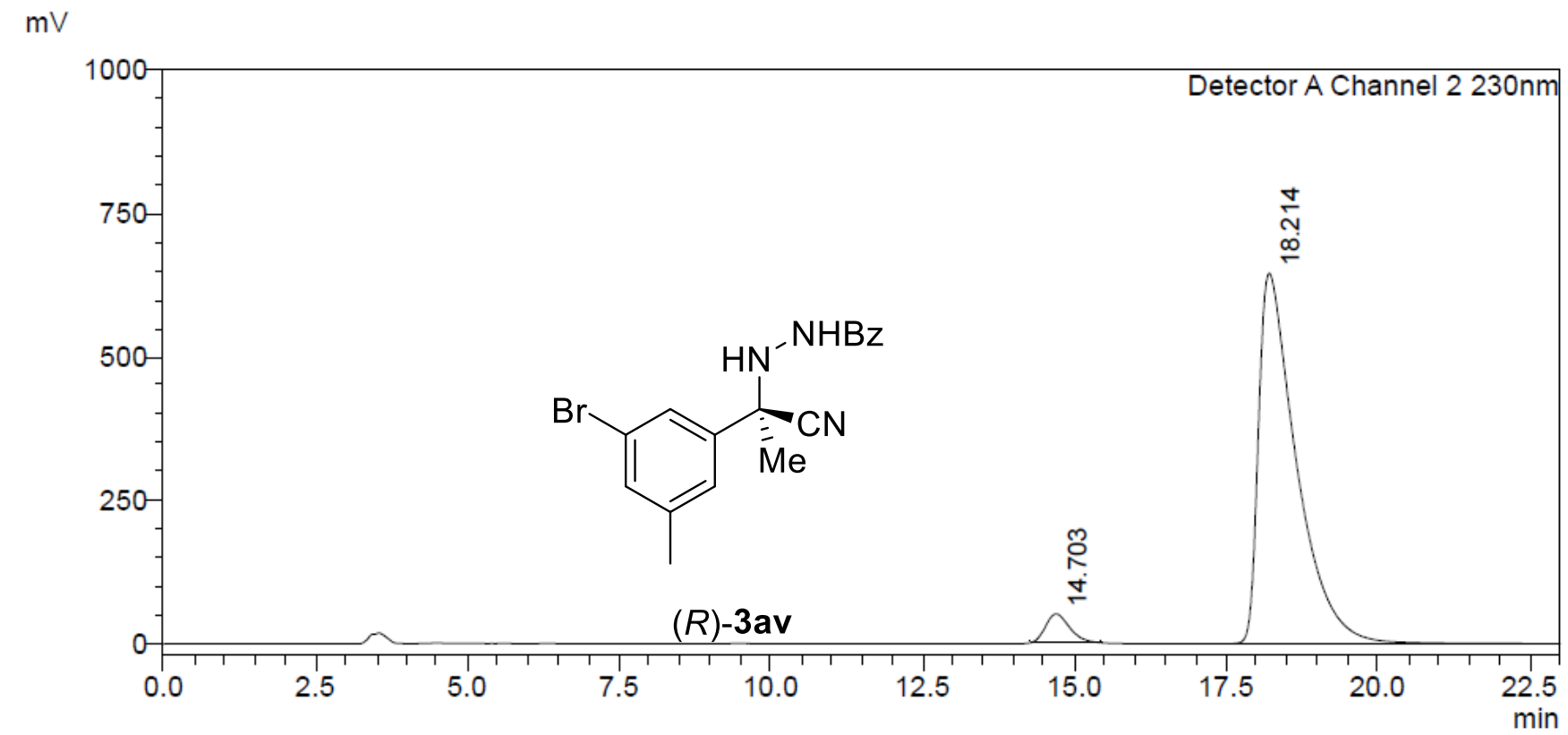

<Peak Table>

Detector A Channel 2 230nm

Peak#	Ret. Time	Area	Height	Conc.
1	14.703	1379769	50230	4.687
2	18.214	28057500	646002	95.313
Total		29437269	696232	

<Sample Information>

Sample Name	: GOY-GF-64-3-rac-ie-8020-1.0-230		
Sample ID	:		
Data Filename	: GOY-GF-64-3-rac-ie-8020-1.0-230.lcd		
Method Filename	: GY-1.0.lcm		
Batch Filename	:		
Vial #	: 1-1	Sample Type	: Unknown
Injection Volume	: 20 uL		
Date Acquired	: 7/10/2020 2:19:22 PM	Acquired by	: System Administrator
Date Processed	: 8/6/2020 4:46:35 PM	Processed by	: System Administrator

<Chromatogram>

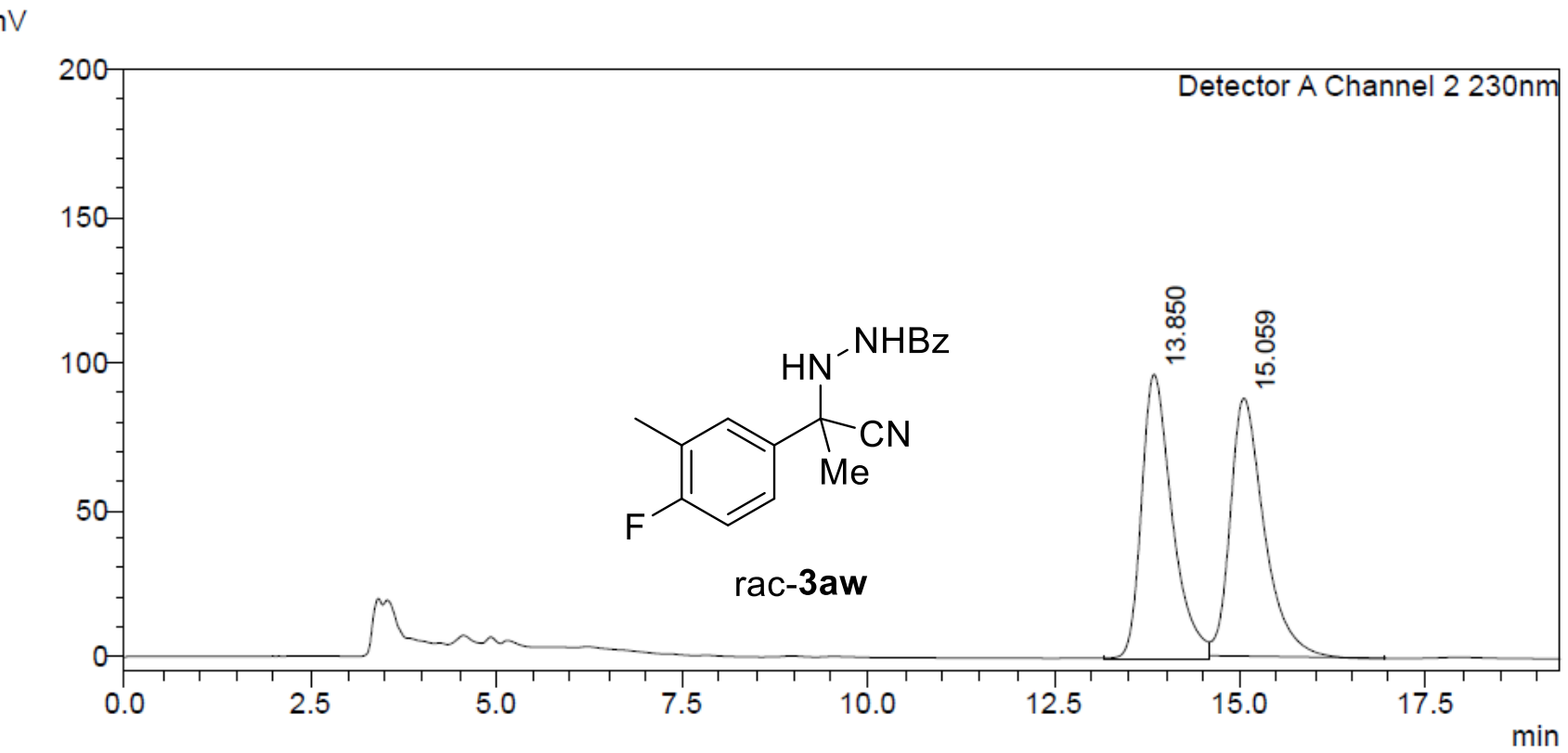

<Peak Table>

Detector A Channel 2 230nm

Peak#	Ret. Time	Area	Height	Conc.
1	13.850	2780639	96856	49.760
2	15.059	2807465	88070	50.240
Total		5588103	184926	

<Sample Information>

Sample Name	: GOY-GF-64-3-asy-ie-8020-1.0-230		
Sample ID	:		
Data Filename	: GOY-GF-64-3-asy-ie-8020-1.0-230.lcd		
Method Filename	: GY-1.0.lcm		
Batch Filename	:		
Vial #	: 1-1	Sample Type	: Unknown
Injection Volume	: 20 uL		
Date Acquired	: 7/10/2020 11:01:03 AM	Acquired by	: System Administrator
Date Processed	: 7/10/2020 11:41:14 AM	Processed by	: System Administrator

<Chromatogram>

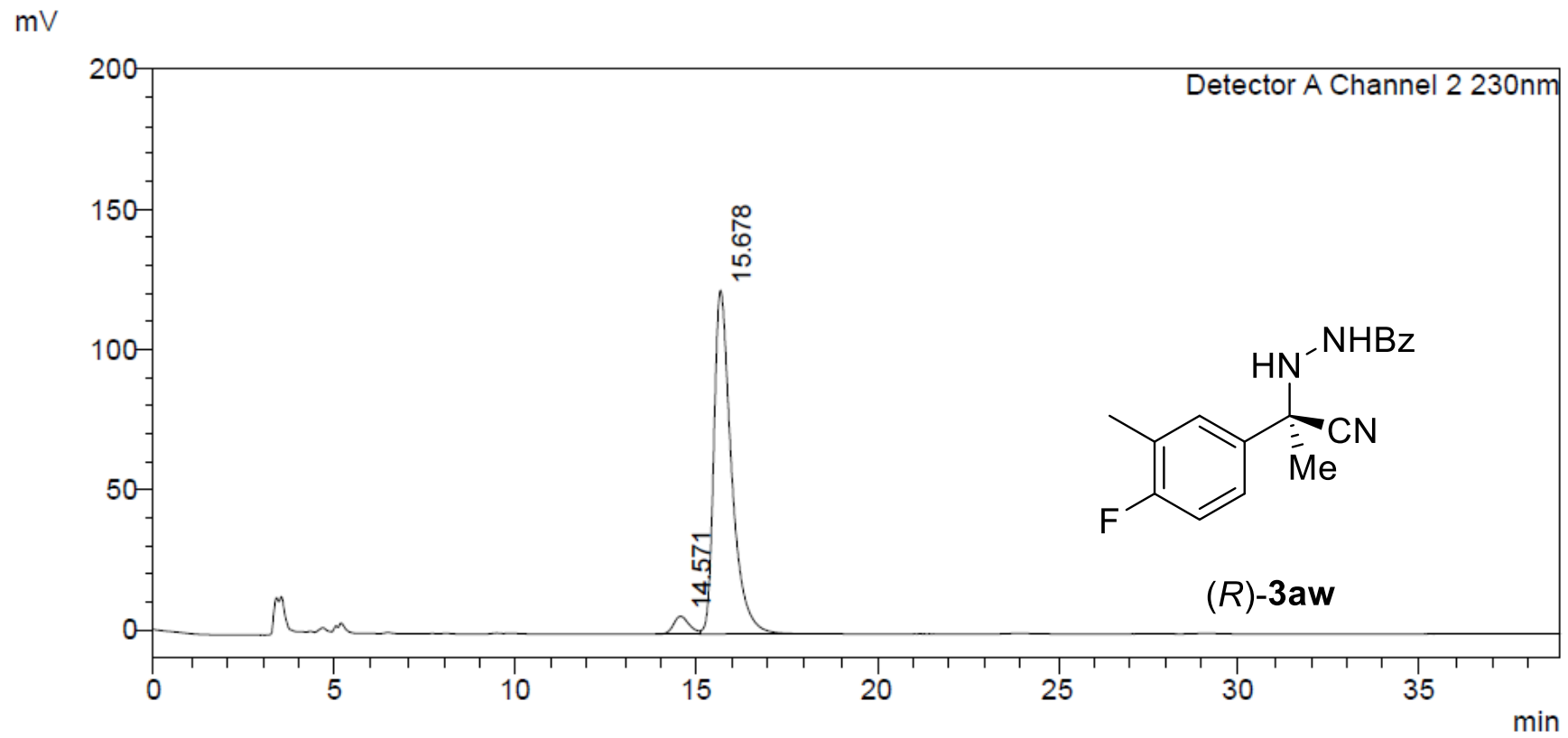

<Peak Table>

Detector A Channel 2 230nm

Peak#	Ret. Time	Area	Height	Conc.
1	14.571	189307	6341	4.360
2	15.678	4152434	122659	95.640
Total		4341741	129001	

<Sample Information>

Sample Name : GOY-GF-131-7-rac-ie-8515-1.0-230
Sample ID :
Data Filename : GOY-GF-131-7-rac-ie-8515-1.0-230.lcd
Method Filename : GY-1.0.lcm
Batch Filename :
Vial # : 1-1 Sample Type : Unknown
Injection Volume : 20 uL
Date Acquired : 8/30/2020 10:58:31 AM Acquired by : System Administrator
Date Processed : 1/28/2021 11:46:37 AM Processed by : System Administrator

<Chromatogram>

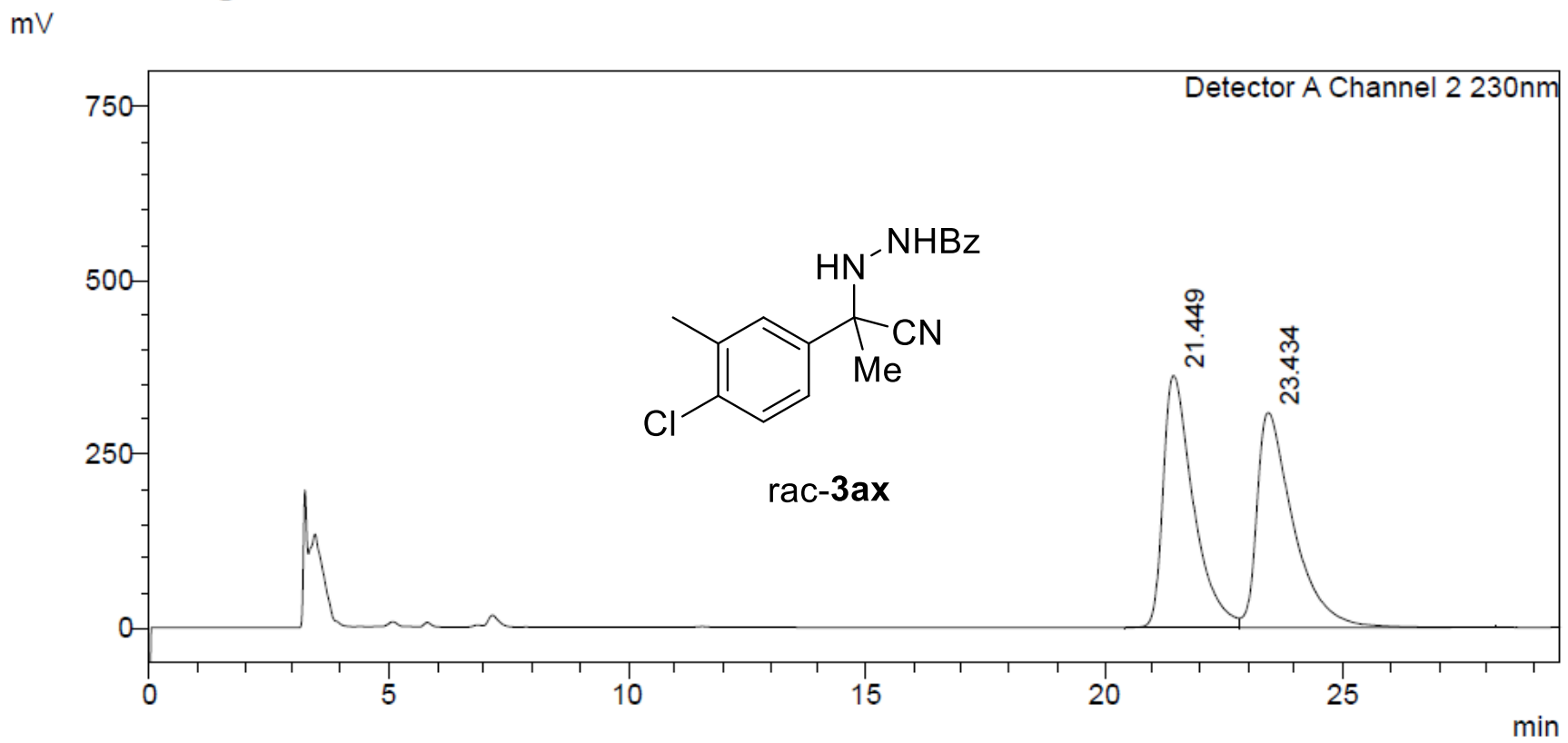

<Peak Table>

Detector A Channel 2 230nm

Peak#	Ret. Time	Area	Height	Conc.
1	21.449	15923810	362592	49.259
2	23.434	16403162	309300	50.741
Total		32326972	671892	

<Sample Information>

Sample Name : GOY-GF-131-7-asy-ie-8515-1.0-230
Sample ID :
Data Filename : GOY-GF-131-7-asy-ie-8515-1.0-230.lcd
Method Filename : GY-1.0.lcm
Batch Filename :
Vial # : 1-1 Sample Type : Unknown
Injection Volume : 20 uL
Date Acquired : 8/30/2020 11:28:55 AM Acquired by : System Administrator
Date Processed : 8/30/2020 11:58:40 AM Processed by : System Administrator

<Chromatogram>

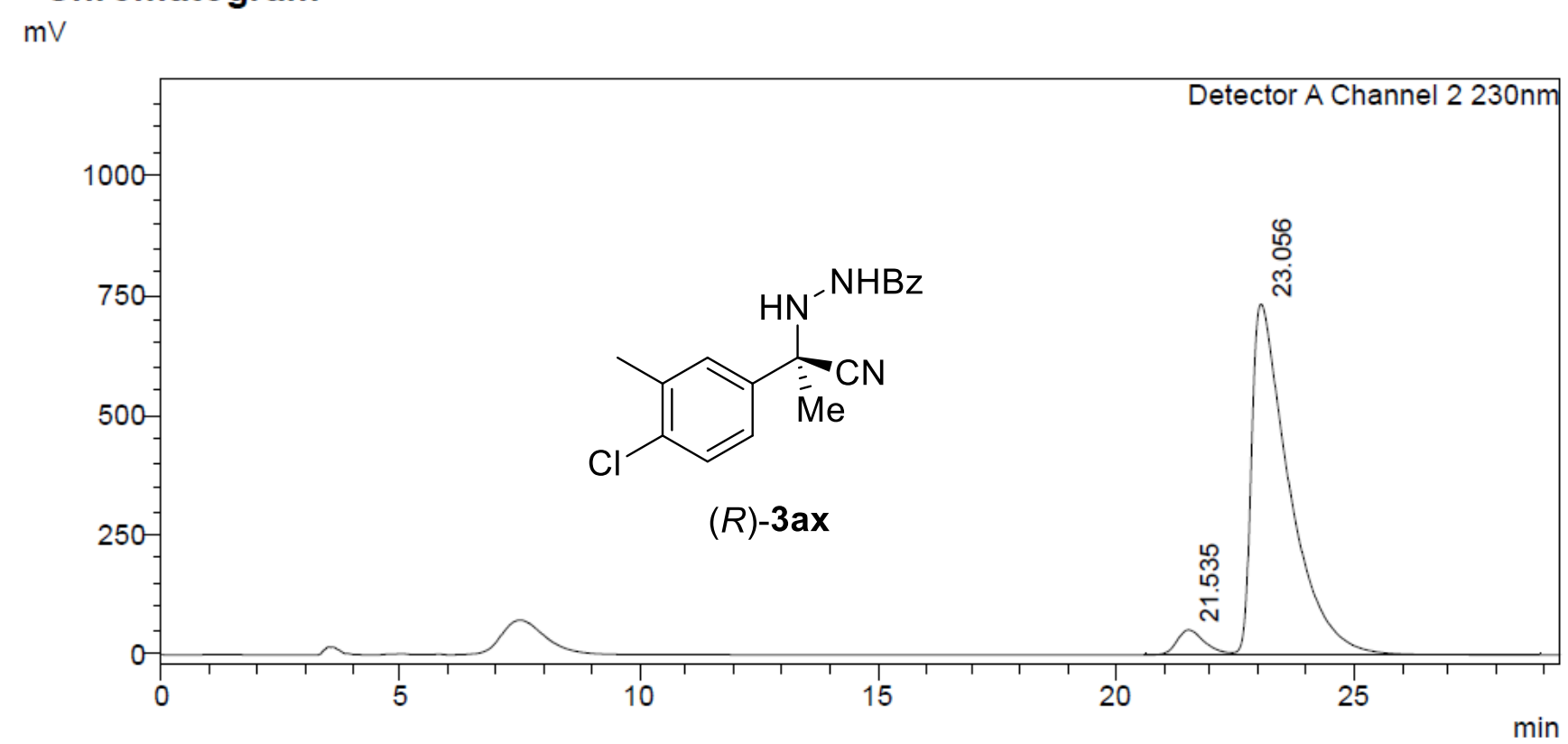

<Peak Table>

Detector A Channel 2 230nm

Peak#	Ret. Time	Area	Height	Conc.
1	21.535	2126445	51989	5.029
2	23.056	40155488	731813	94.971
Total		42281933	783802	

<Sample Information>

Sample Name : GOY-GI-52-1-rac-ia-8515-1.0-230
Sample ID :
Data Filename : GOY-GI-52-1-rac-ia-8515-1.0-230.lcd
Method Filename : GY-1.0.lcm
Batch Filename :
Vial # : 1-1 Sample Type : Unknown
Injection Volume : 20 uL
Date Acquired : 4/1/2021 10:32:58 AM Acquired by : System Administrator
Date Processed : 4/1/2021 11:16:44 AM Processed by : System Administrator

<Chromatogram>

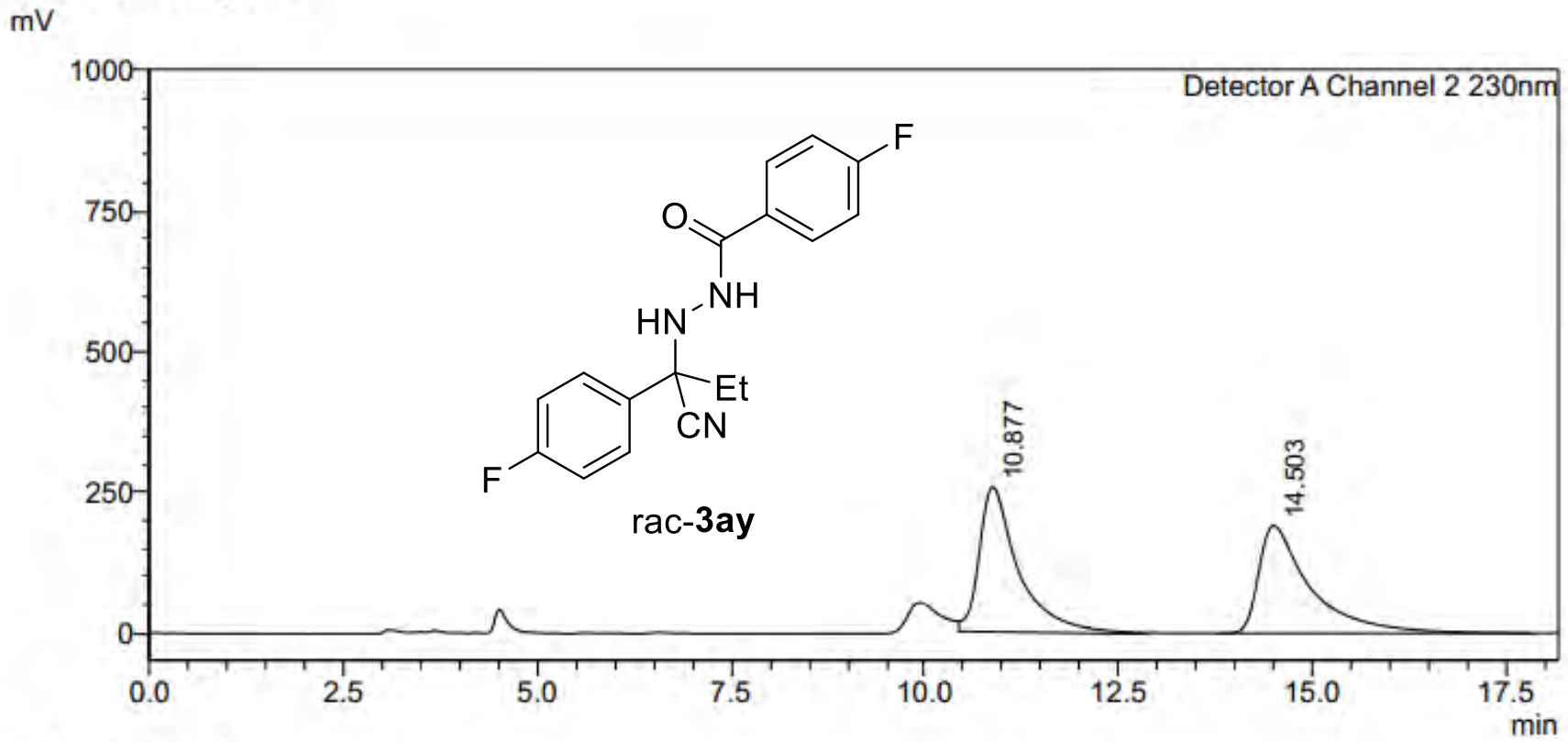

<Peak Table>

Detector A Channel 2 230nm

Peak#	Ret. Time	Area	Height	Conc.
1	10.877	9148404	257611	50.644
2	14.503	8915824	191655	49.356
Total		18064229	449266	

<Sample Information>

Sample Name : GOY-GI-52-1-asy-ia-8515-1.0-230
Sample ID :
Data Filename : GOY-GI-52-1-asy-ia-8515-1.0-230.lcd
Method Filename : GY-1.0.lcm
Batch Filename :
Vial # : 1-1 Sample Type : Unknown
Injection Volume : 20 uL
Date Acquired : 4/1/2021 10:52:21 AM Acquired by : System Administrator
Date Processed : 4/1/2021 11:19:37 AM Processed by : System Administrator

<Chromatogram>

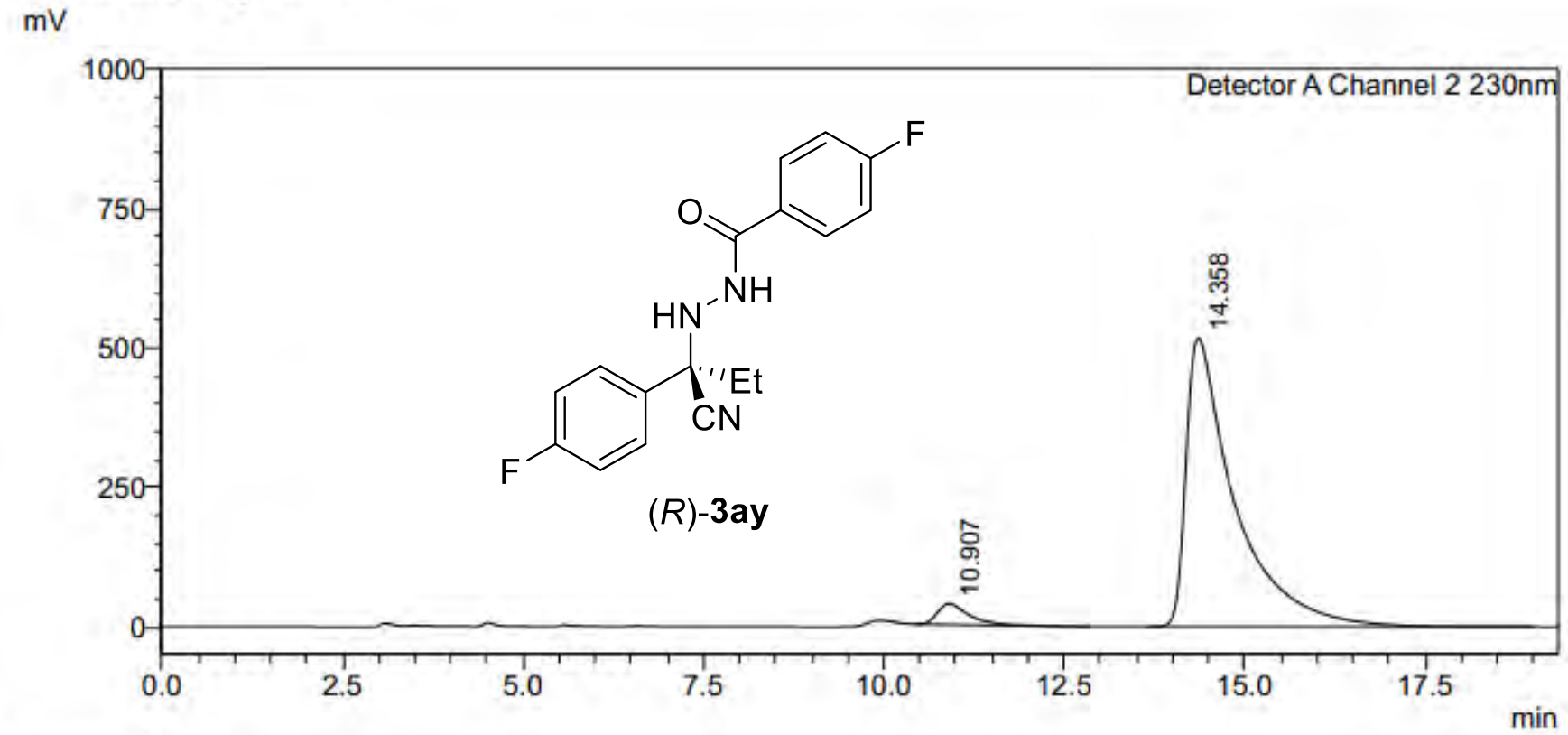

<Peak Table>

Detector A Channel 2 230nm

Peak#	Ret. Time	Area	Height	Conc.
1	10.907	1161669	37551	4.552
2	14.358	24358125	518238	95.448
Total		25519795	555789	

<Sample Information>

Sample Name	: GOY-GI-52-2-rac-ia-8515-1.0-230		
Sample ID	:		
Data Filename	: GOY-GI-52-2-rac-ia-8515-1.0-230.lcd		
Method Filename	: GY-1.0.lcm		
Batch Filename	:		
Vial #	: 1-1	Sample Type	: Unknown
Injection Volume	: 20 uL		
Date Acquired	: 4/1/2021 11:14:44 AM	Acquired by	: System Administrator
Date Processed	: 4/1/2021 9:30:07 PM	Processed by	: System Administrator

<Chromatogram>

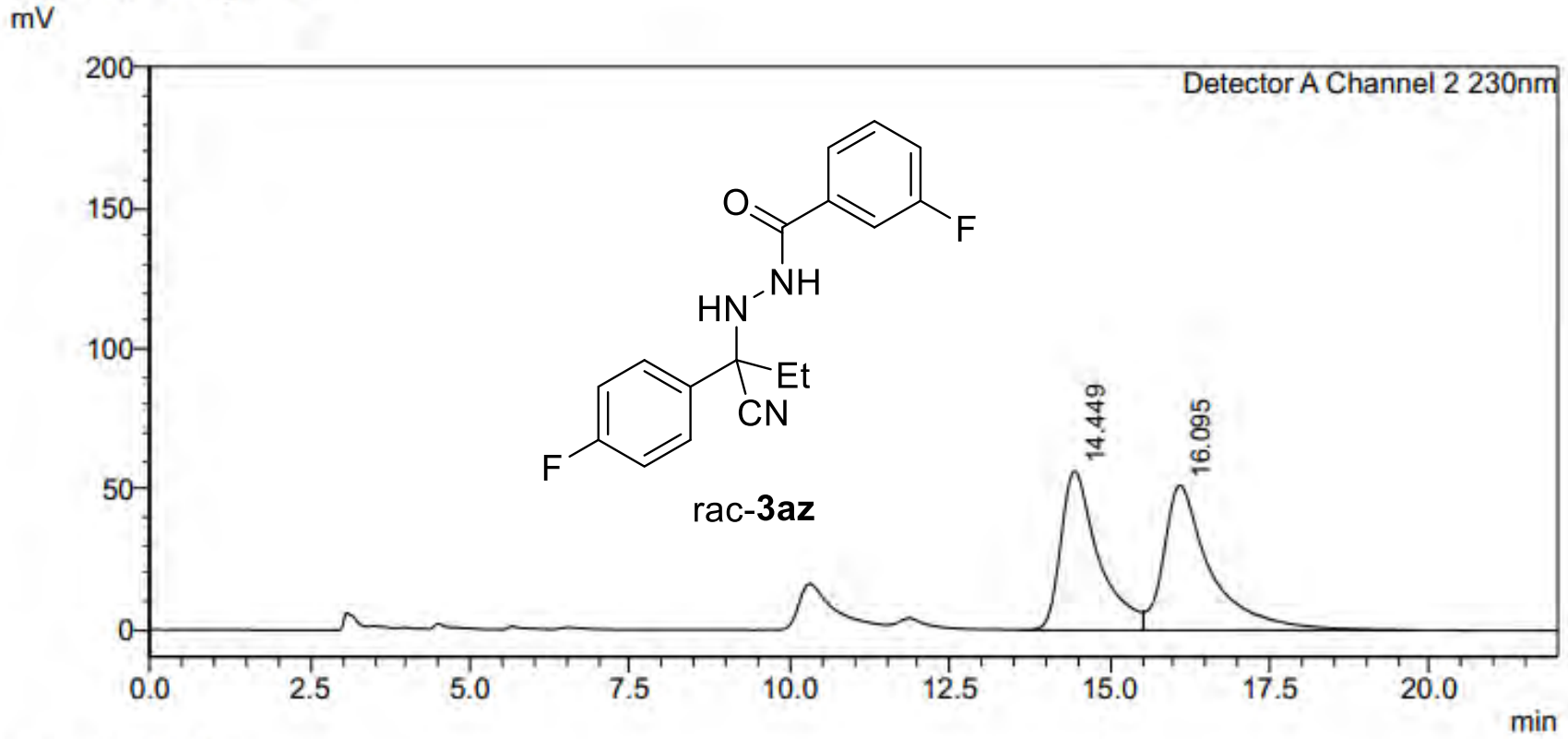

<Peak Table>

Detector A Channel 2 230nm

Peak#	Ret. Time	Area	Height	Conc.
1	14.449	2311253	56632	46.853
2	16.095	2621773	51503	53.147
Total		4933025	108135	

<Sample Information>

Sample Name	: GOY-GI-52-2-asy-ia-8515-1.0-230		
Sample ID	:		
Data Filename	: GOY-GI-52-2-asy-ia-8515-1.0-231.lcd		
Method Filename	: zzh-1.0-254-230.lcm		
Batch Filename	:		
Vial #	: 1-1	Sample Type	: Unknown
Injection Volume	: 20 uL		
Date Acquired	: 4/1/2021 8:37:25 PM	Acquired by	: System Administrator
Date Processed	: 4/1/2021 9:36:26 PM	Processed by	: System Administrator

<Chromatogram>

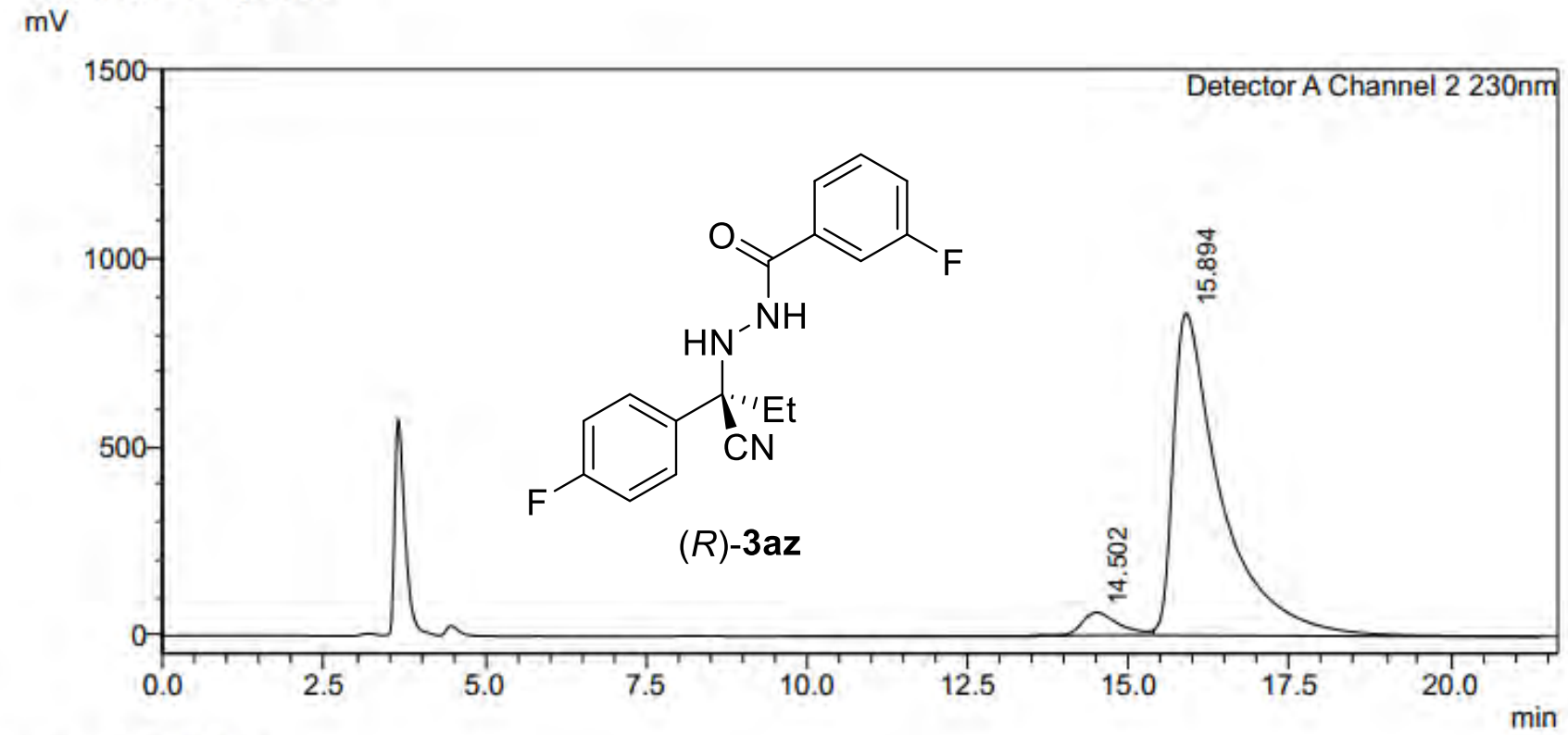

<Peak Table>

Detector A Channel 2 230nm

Peak#	Ret. Time	Area	Height	Conc.
1	14.502	2413763	60879	5.156
2	15.894	44402975	855724	94.844
Total		46816737	916603	

<Sample Information>

Sample Name	: gy-4a-rac-od-3-90-10-230-254-1.0		
Sample ID	:		
Data Filename	: gy-4a-rac-od-3-90-10-230-254-1.0.lcd		
Method Filename	: ZHZ-1.0-254-230.lcm		
Batch Filename	:		
Vial #	: 1-1	Sample Type	: 未知
Injection Volume	: 20 uL		
Date Acquired	: 2026/4/16 11:34:25	Acquired by	: System Administrator
Date Processed	: 2026/4/16 12:10:42	Processed by	: System Administrator

<Chromatogram>

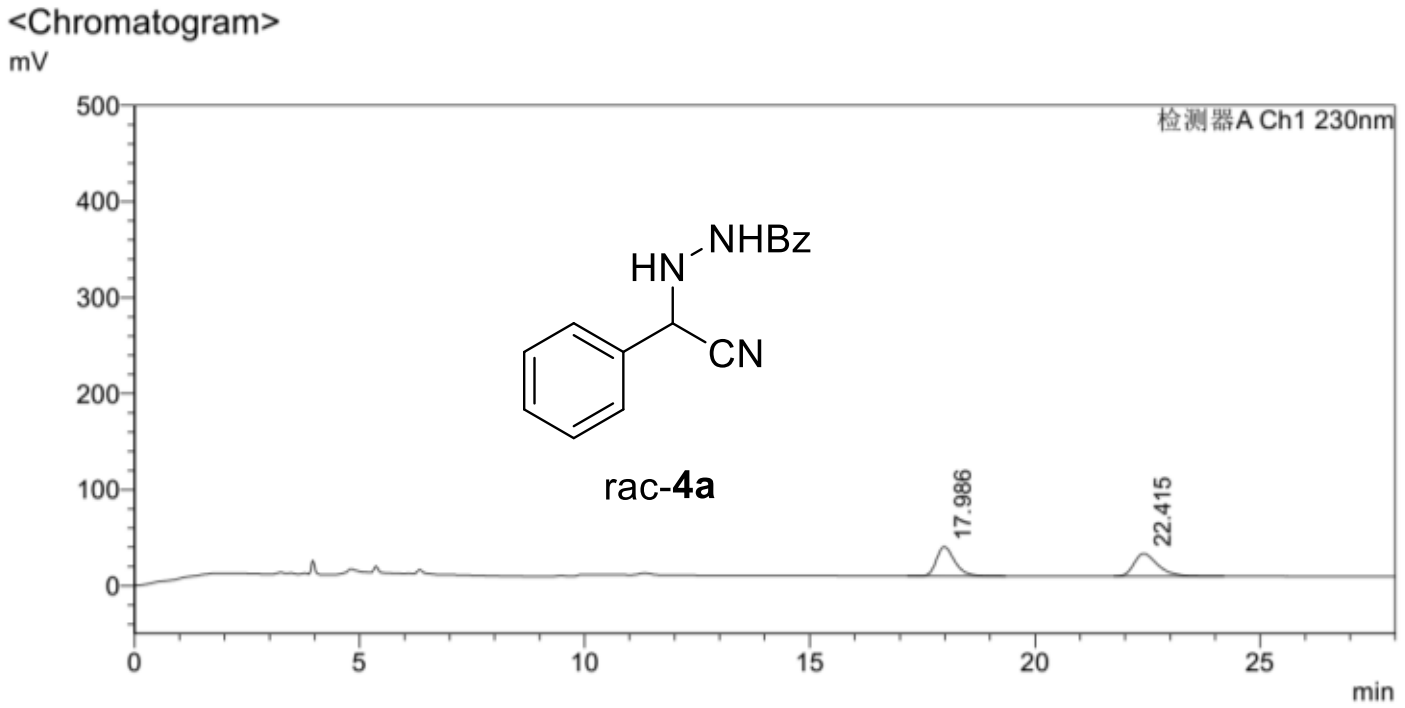

<Peak Table>

检测器A Ch1 230nm

Peak#	Ret. Time	Area	Height	Conc.
1	17.986	853587	30327	49.949
2	22.415	855336	23275	50.051
总计		1708924	53602	

E:\Data\ZHZ\gy-4a-rac-od-3-90-10-230-254-1.0.lcd

<Sample Information>

Sample Name	: gy-4a-asy-od-3-90-10-230-254-1.0		
Sample ID	:		
Data Filename	: gy-4a-asy-od-3-90-10-230-254-1.0.lcd		
Method Filename	: ZHZ-1.0-254-230.lcm		
Batch Filename	:		
Vial #	: 1-1	Sample Type	: 未知
Injection Volume	: 20 uL		
Date Acquired	: 2026/4/16 12:11:09	Acquired by	: System Administrator
Date Processed	: 2026/4/16 13:01:56	Processed by	: System Administrator

<Chromatogram>

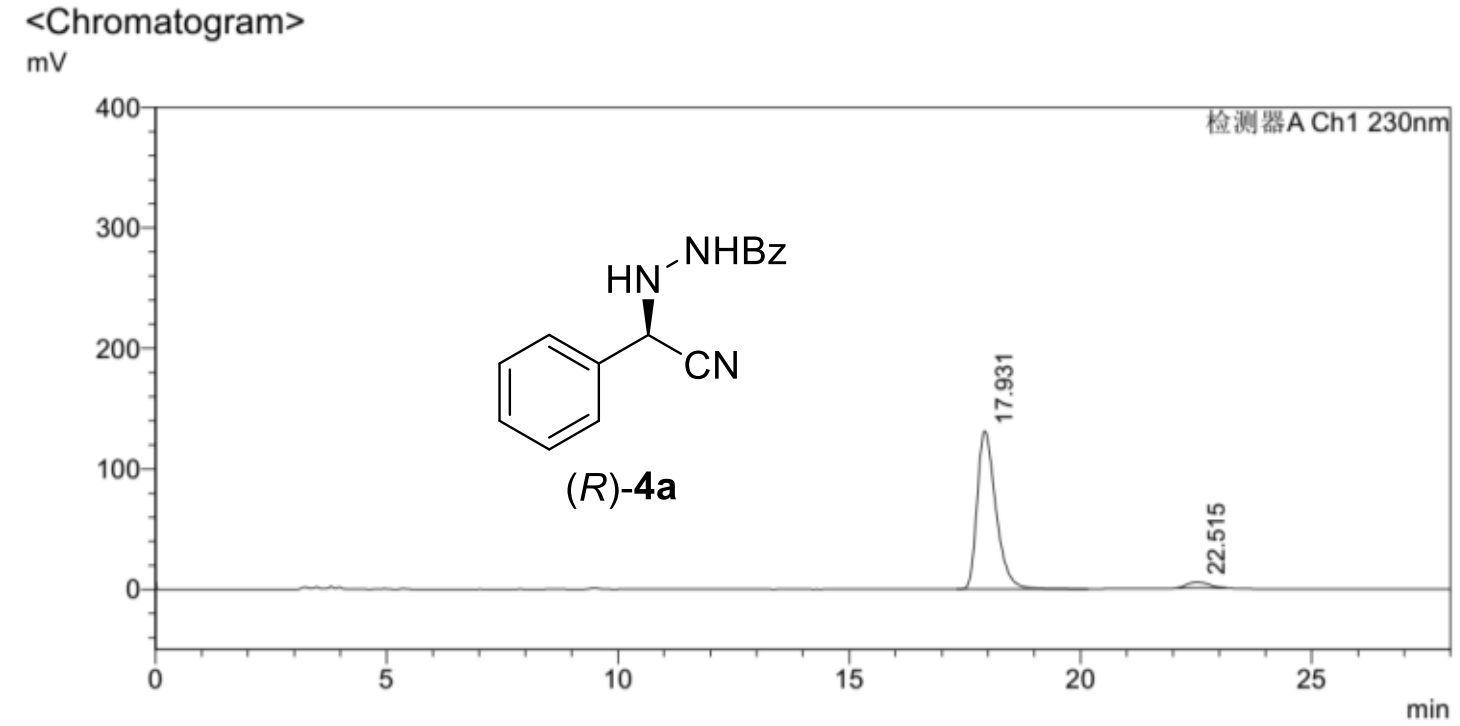

<Peak Table>

检测器A Ch1 230nm

Peak#	Ret. Time	Area	Height	Conc.
1	17.931	3702456	131323	95.792
2	22.515	162644	5135	4.208
总计		3865100	136458	

E:\Data\ZHZ\gy-4a-asy-od-3-90-10-230-254-1.0.lcd

<Sample Information>

Sample Name : GOY-GG-114-2-rac-odh-8020-1.0-230
Sample ID :
Data Filename : GOY-GG-114-2-rac-odh-8020-1.0-232.lcd
Method Filename : GY-1.0.lcm
Batch Filename :
Vial # : 1-1 Sample Type : Unknown
Injection Volume : 20 uL
Date Acquired : 11/21/2020 3:18:21 PM Acquired by : System Administrator
Date Processed : 11/21/2020 3:38:05 PM Processed by : System Administrator

<Chromatogram>

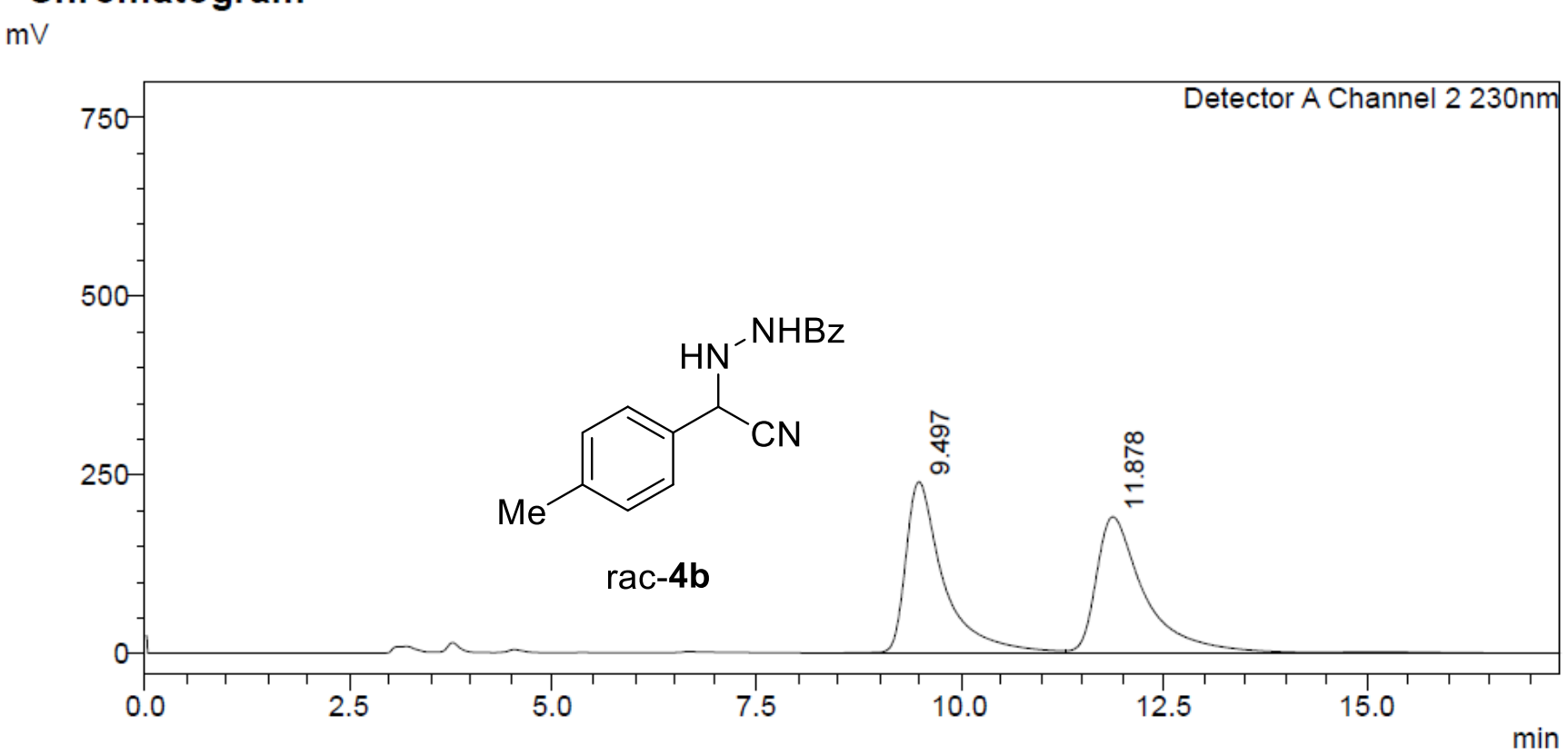

<Peak Table>

Detector A Channel 2 230nm

Peak#	Ret. Time	Area	Height	Conc.
1	9.497	7815017	240172	49.637
2	11.878	7929363	190548	50.363
Total		15744380	430720	

<Sample Information>

Sample Name : GOY-GG-114-2-asy-odh-8020-1.0-230
Sample ID :
Data Filename : GOY-GG-114-2-asy-odh-8020-1.0-231.lcd
Method Filename : GY-1.0.lcm
Batch Filename :
Vial # : 1-1 Sample Type : Unknown
Injection Volume : 20 uL
Date Acquired : 11/21/2020 3:36:42 PM Acquired by : System Administrator
Date Processed : 11/21/2020 4:31:56 PM Processed by : System Administrator

<Chromatogram>

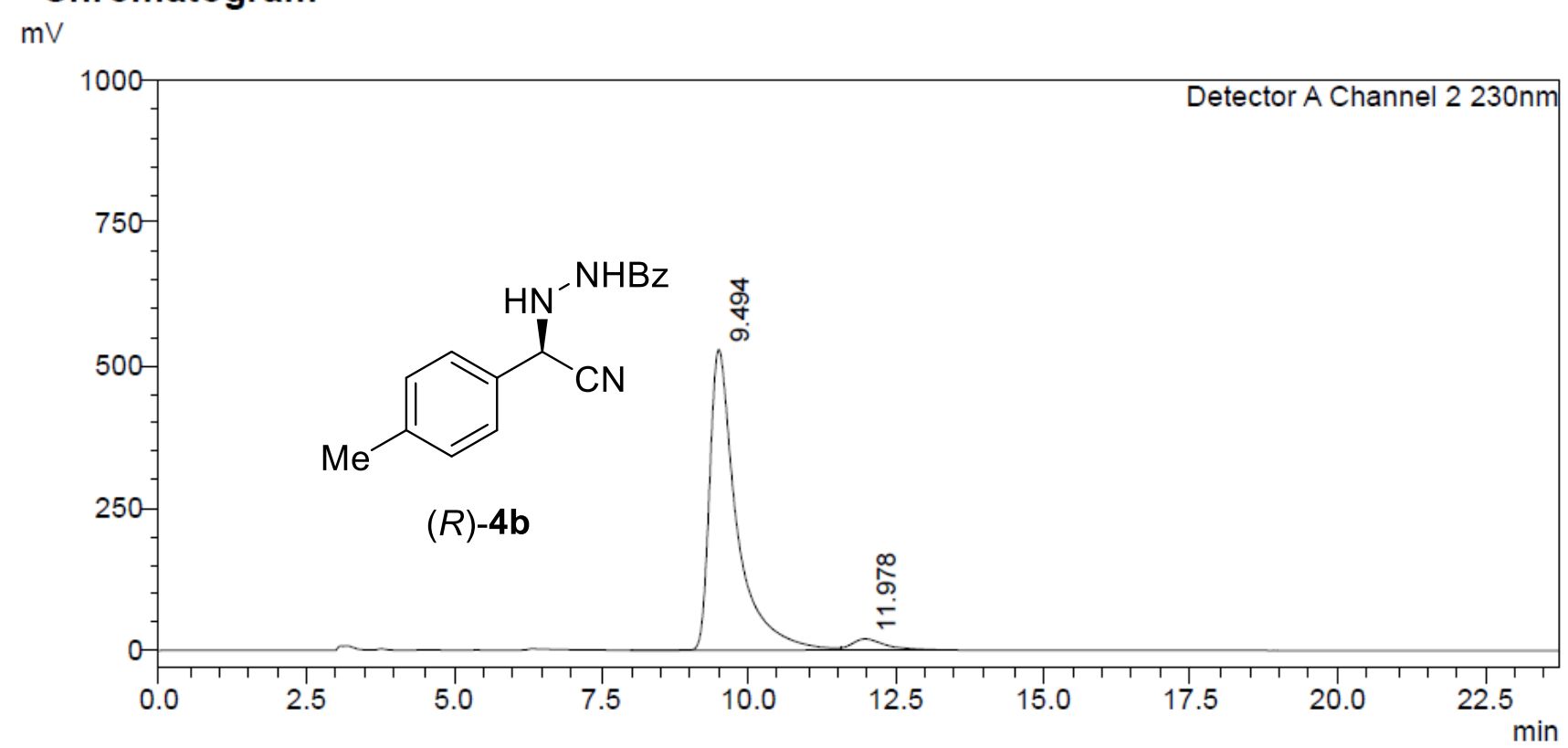

<Peak Table>

Detector A Channel 2 230nm

Peak#	Ret. Time	Area	Height	Conc.
1	9.494	16954341	528146	95.632
2	11.978	774476	19766	4.368
Total		17728816	547912	

<Sample Information>

Sample Name : GOY-GG-136-2-rac-odh-9010-1.0-230
Sample ID :
Data Filename : GOY-GG-136-2-rac-odh-9010-1.0-231.lcd
Method Filename : GY-1.0.lcm
Batch Filename :
Vial # : 1-1 Sample Type : Unknown
Injection Volume : 20 uL
Date Acquired : 3/31/2021 11:04:38 AM Acquired by : System Administrator
Date Processed : 3/31/2021 11:46:01 AM Processed by : System Administrator

<Chromatogram>

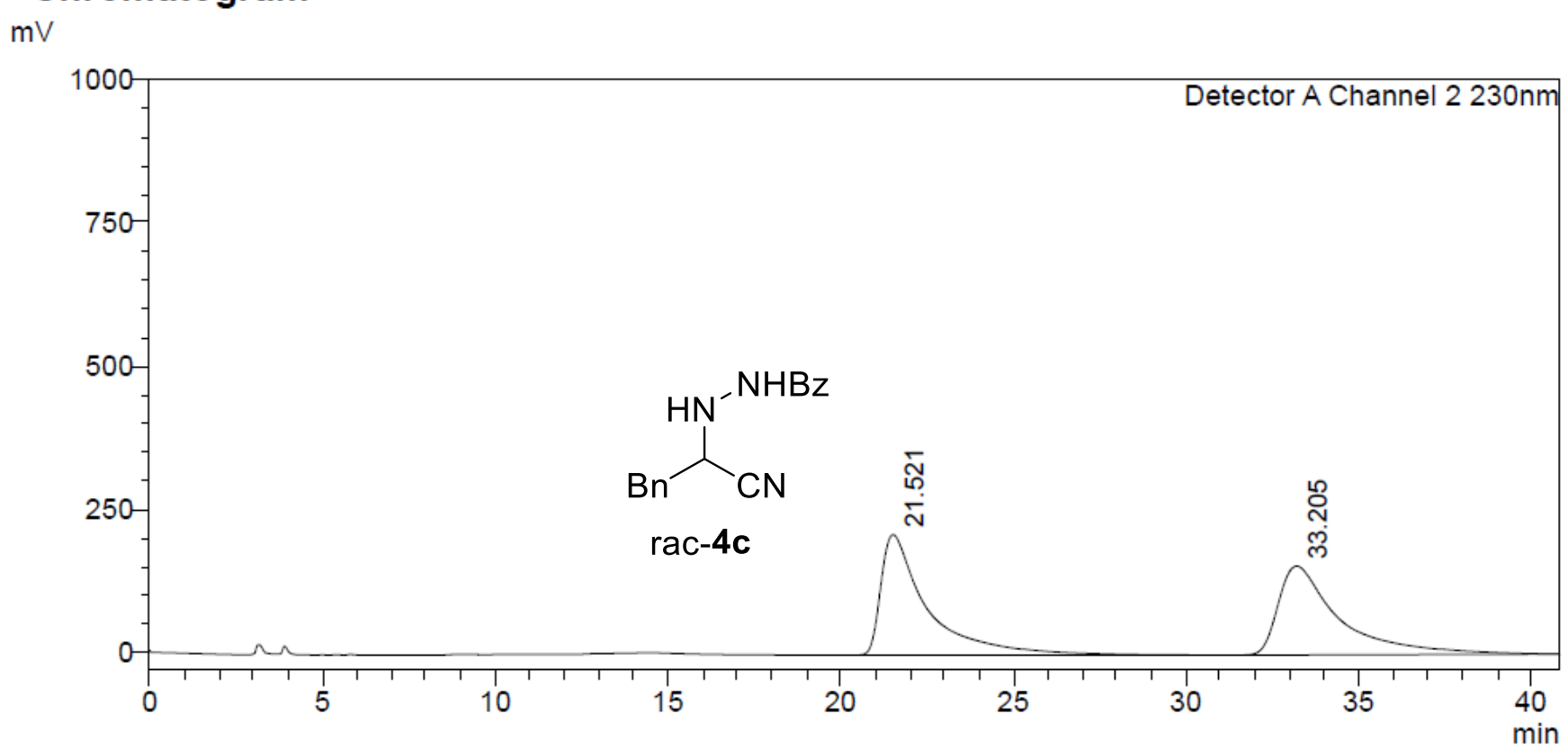

<Peak Table>

Detector A Channel 2 230nm

Peak#	Ret. Time	Area	Height	Conc.
1	21.521	19755414	210369	50.777
2	33.205	19150980	155233	49.223
Total		38906394	365602	

<Sample Information>

Sample Name : GOY-GG-136-2-asy-odh-9010-1.0-230
Sample ID :
Data Filename : GOY-GG-136-2-asy-odh-9010-1.0-230.lcd
Method Filename : GY-1.0.lcm
Batch Filename :
Vial # : 1-1 Sample Type : Unknown
Injection Volume : 20 uL
Date Acquired : 3/31/2021 10:23:21 AM Acquired by : System Administrator
Date Processed : 3/31/2021 3:01:35 PM Processed by : System Administrator

<Chromatogram>

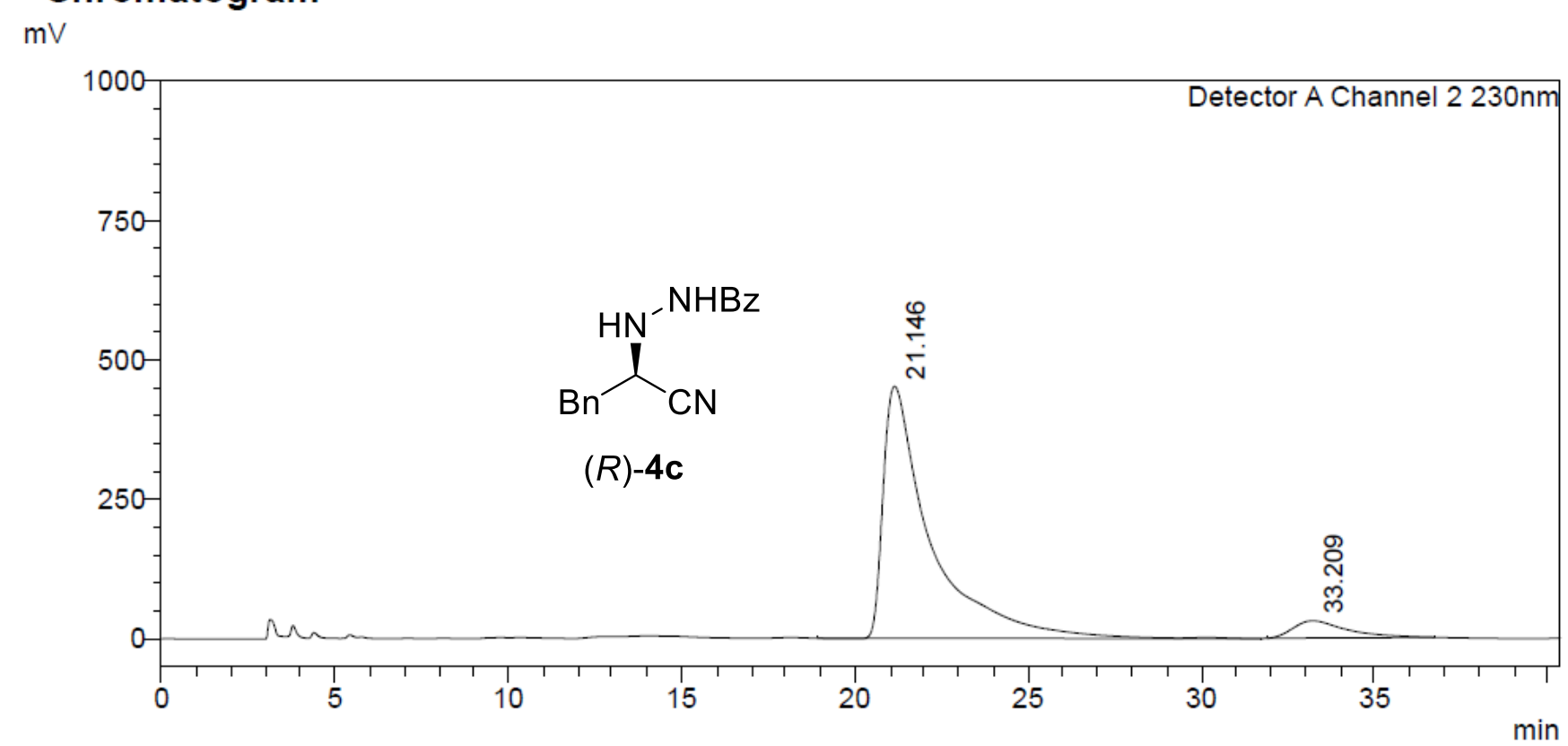

<Peak Table>

Detector A Channel 2 230nm

Peak#	Ret. Time	Area	Height	Conc.
1	21.146	43232221	452291	92.899
2	33.209	3304568	30875	7.101
Total		46536790	483167	

<Sample Information>

Sample Name	: GOY-GH-29-1-rac-odh-9010-1.0-230		
Sample ID	:		
Data Filename	: GOY-GH-29-1-rac-odh-9010-1.0-230.lcd		
Method Filename	: GY-1.0.lcm		
Batch Filename	:		
Vial #	: 1-1	Sample Type	: Unknown
Injection Volume	: 20 uL		
Date Acquired	: 1/28/2021 6:39:47 PM	Acquired by	: System Administrator
Date Processed	: 1/28/2021 6:58:29 PM	Processed by	: System Administrator

<Chromatogram>

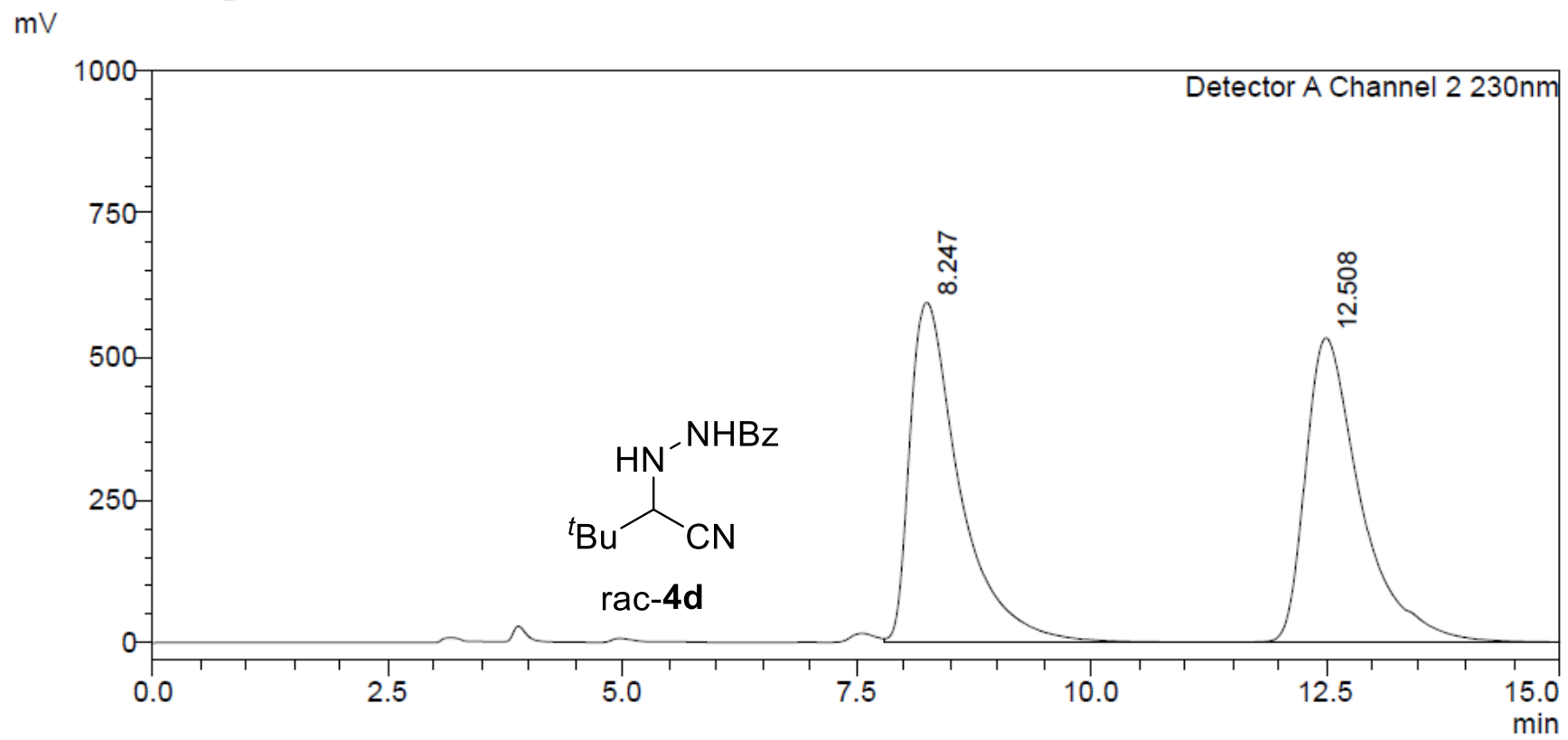

<Peak Table>

Detector A Channel 2 230nm

Peak#	Ret. Time	Area	Height	Conc.
1	8.247	22100474	595531	50.125
2	12.508	21990237	533239	49.875
Total		44090711	1128769	

<Sample Information>

Sample Name	: GOY-GH-29-1-asy-odh-9010-1.0-230		
Sample ID	:		
Data Filename	: GOY-GH-29-1-asy-odh-9010-1.0-231.lcd		
Method Filename	: GY-1.0.lcm		
Batch Filename	:		
Vial #	: 1-1	Sample Type	: Unknown
Injection Volume	: 20 uL		
Date Acquired	: 1/28/2021 6:15:48 PM	Acquired by	: System Administrator
Date Processed	: 1/28/2021 6:41:37 PM	Processed by	: System Administrator

<Chromatogram>

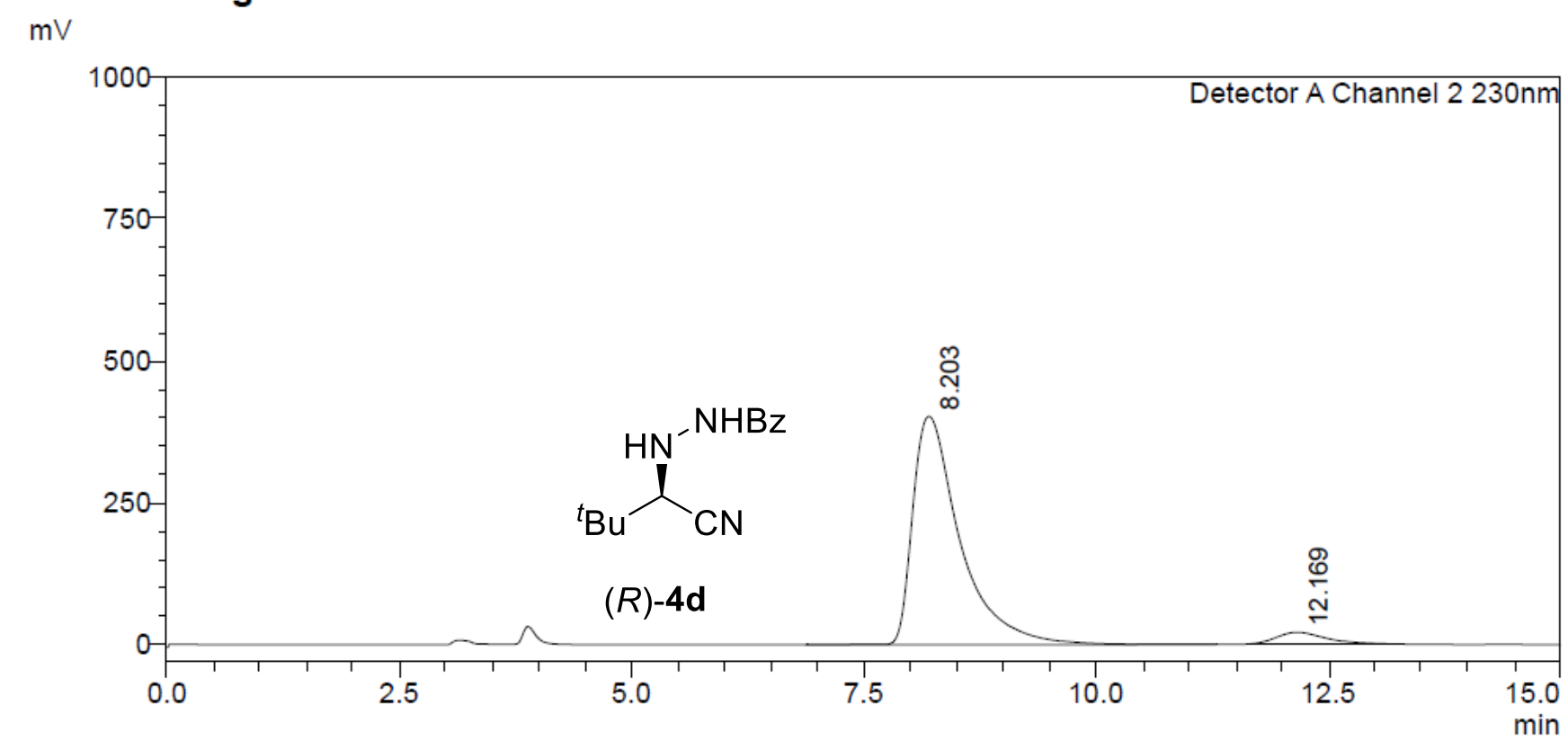

<Peak Table>

Detector A Channel 2 230nm

Peak#	Ret. Time	Area	Height	Conc.
1	8.203	14296026	403075	94.884
2	12.169	770801	20671	5.116
Total		15066826	423745	

<Sample Information>

Sample Name : GOY-GH-42-2-rac-odh-9010-1.0-230
Sample ID :
Data Filename : GOY-GH-42-2-rac-odh-9010-1.0-230.lcd
Method Filename : GY-1.0.lcm
Batch Filename :
Vial # : 1-1 Sample Type : Unknown
Injection Volume : 20 uL
Date Acquired : 3/30/2021 2:22:23 PM Acquired by : System Administrator
Date Processed : 3/30/2021 2:51:06 PM Processed by : System Administrator

<Chromatogram>

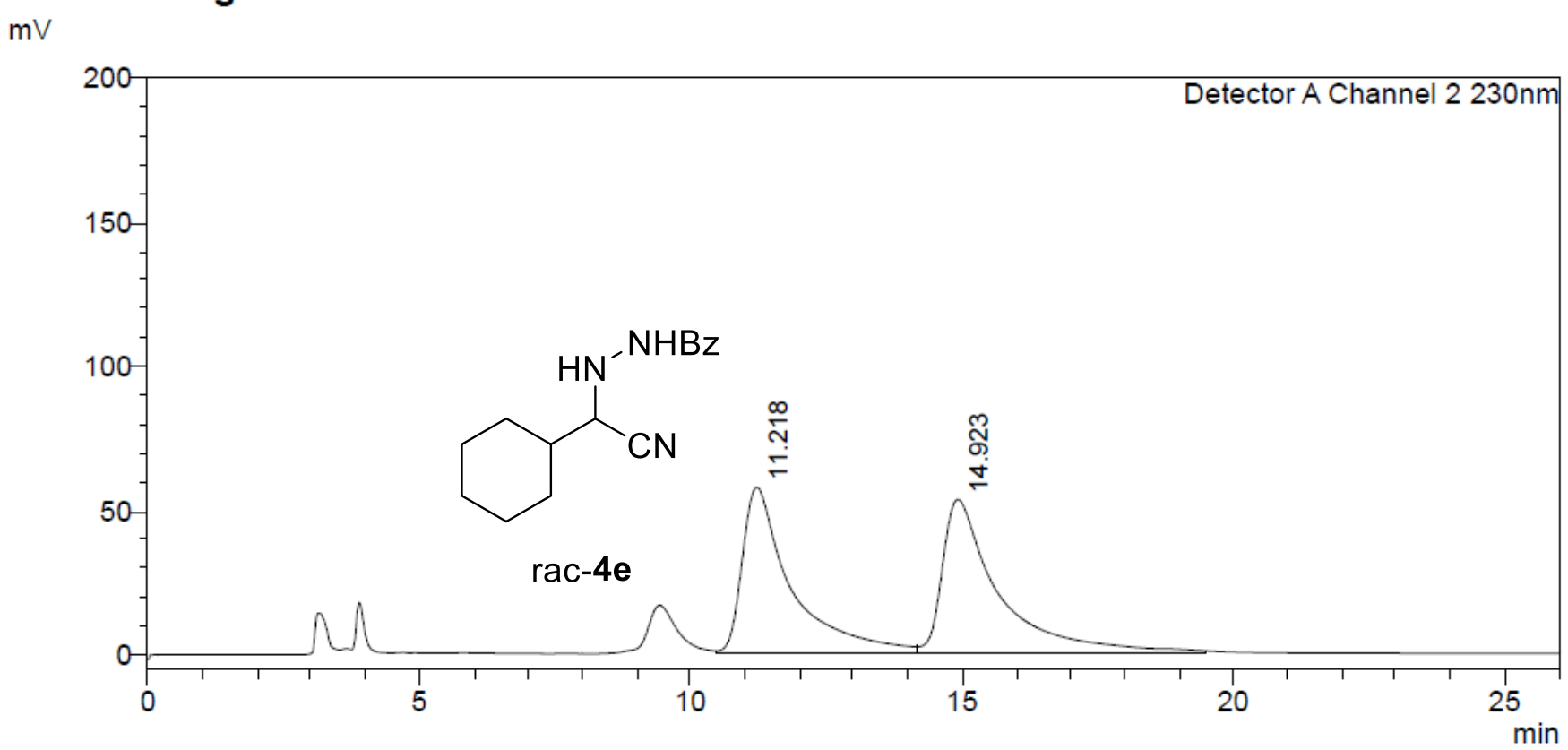

<Peak Table>

Detector A Channel 2 230nm

Peak#	Ret. Time	Area	Height	Conc.
1	11.218	3668031	57728	49.278
2	14.923	3775464	53048	50.722
Total		7443495	110776	

<Sample Information>

Sample Name : GOY-GH-42-2-asy-odh-9010-1.0-230
Sample ID :
Data Filename : GOY-GH-42-2-asy-odh-9010-1.0-230.lcd
Method Filename : GY-1.0.lcm
Batch Filename :
Vial # : 1-1 Sample Type : Unknown
Injection Volume : 20 uL
Date Acquired : 3/30/2021 2:49:56 PM Acquired by : System Administrator
Date Processed : 3/30/2021 3:14:31 PM Processed by : System Administrator

<Chromatogram>

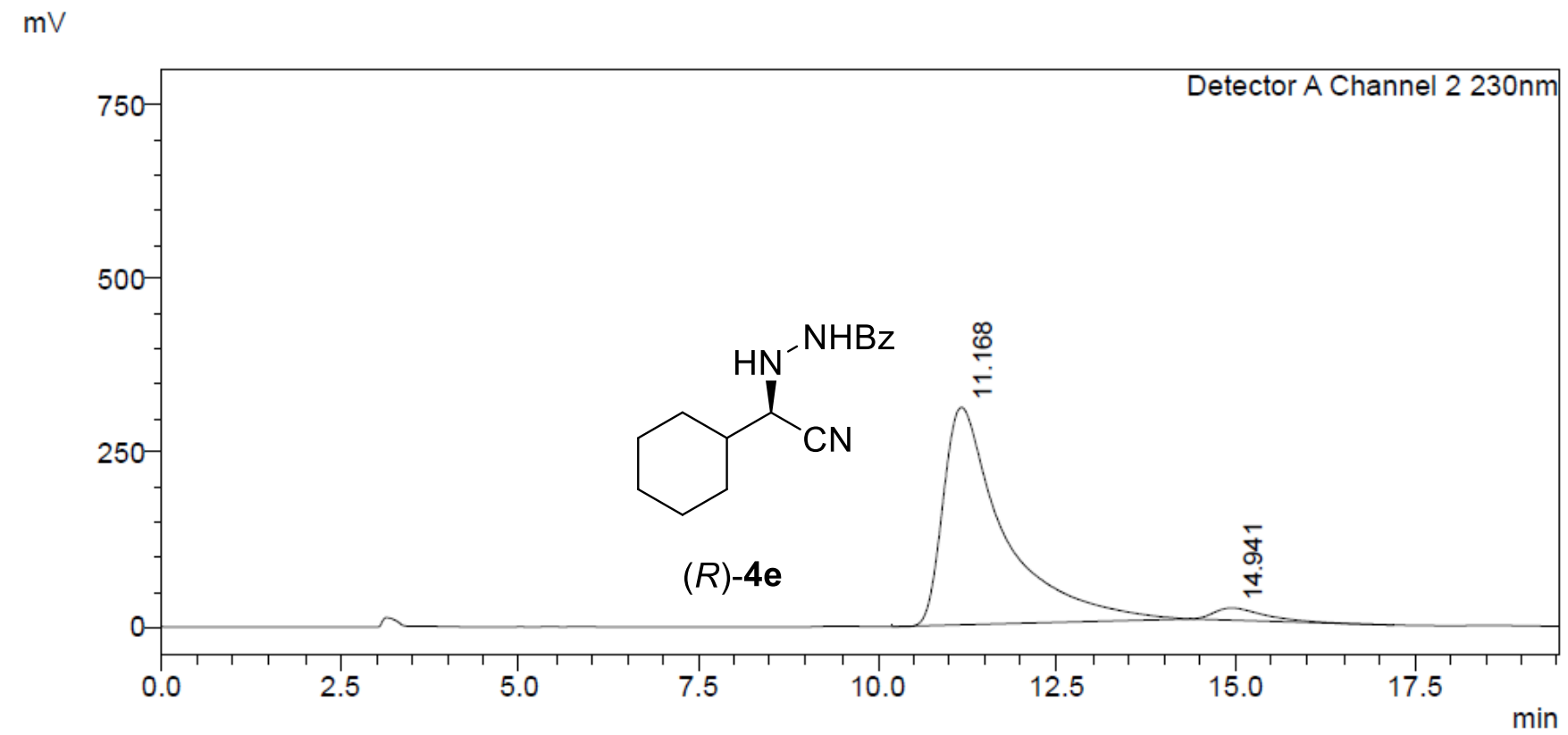

<Peak Table>

Detector A Channel 2 230nm

Peak#	Ret. Time	Area	Height	Conc.
1	11.168	18712529	312817	95.604
2	14.941	860355	17485	4.396
Total		19572884	330302	

<Sample Information>

Sample Name : GOY-GH-111-1-rac-odh-9010-1.0-230
Sample ID :
Data Filename : GOY-GH-111-1-rac-odh-9010-1.0-231.lcd
Method Filename : GY-1.0.lcm
Batch Filename :
Vial # : 1-1 Sample Type : Unknown
Injection Volume : 20 uL
Date Acquired : 2/23/2021 8:19:21 PM Acquired by : System Administrator
Date Processed : 2/23/2021 9:25:29 PM Processed by : System Administrator

<Chromatogram>

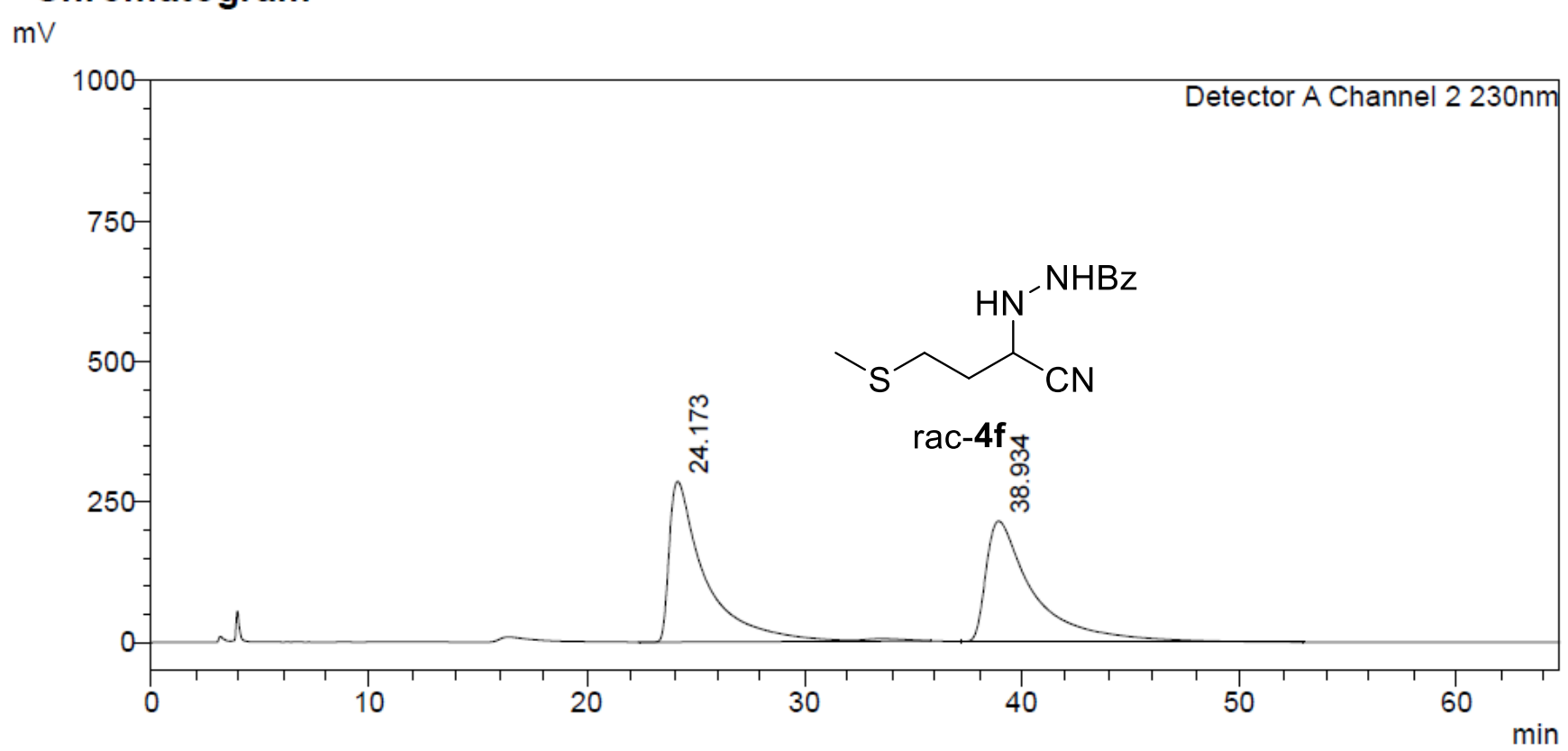

<Peak Table>

Detector A Channel 2 230nm

Peak#	Ret. Time	Area	Height	Conc.
1	24.173	33248542	285921	50.094
2	38.934	33124080	215657	49.906
Total		66372623	501579	

<Sample Information>

Sample Name : GOY-GH-111-1-asy-odh-9010-1.0-230
Sample ID :
Data Filename : GOY-GH-111-1-asy-odh-9010-1.0-231.lcd
Method Filename : GY-1.0.lcm
Batch Filename :
Vial # : 1-1 Sample Type : Unknown
Injection Volume : 20 uL
Date Acquired : 2/23/2021 7:14:16 PM Acquired by : System Administrator
Date Processed : 2/23/2021 9:26:58 PM Processed by : System Administrator

<Chromatogram>

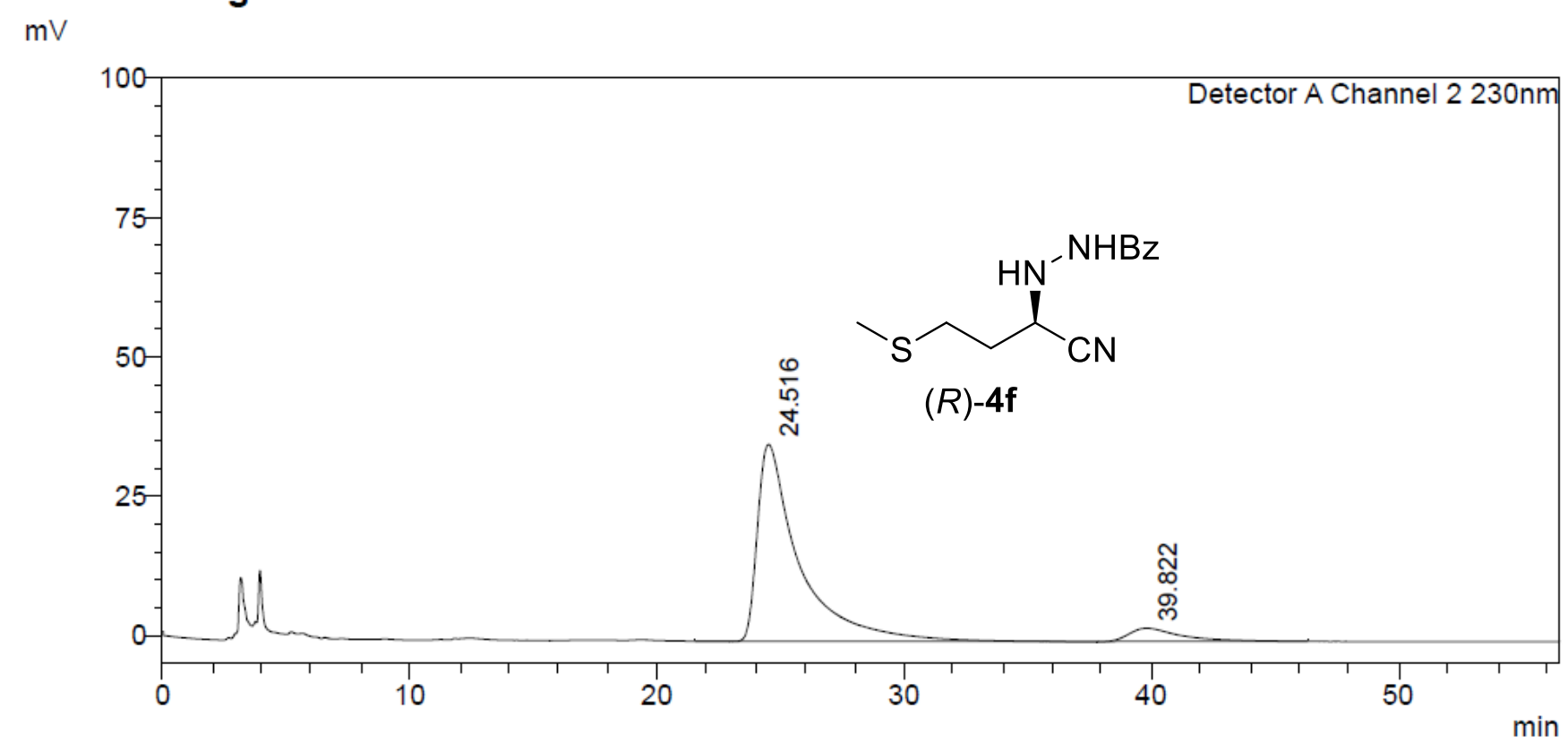

<Peak Table>

Detector A Channel 2 230nm

Peak#	Ret. Time	Area	Height	Conc.
1	24.516	4192487	35330	92.227
2	39.822	353348	2376	7.773
Total		4545836	37706	

<Sample Information>

Sample Name : GOY-GV-22-RLigand-rac-ADH-8020-1.0-230-DATA
Sample ID :
Data Filename : GOY-GV-22-RLigand-rac-ADH-8020-1.0-230-DATA1.lcd
Method Filename : GY-1.0.lcm
Batch Filename :
Vial # : 1-1 Sample Type : Unknown
Injection Volume : 20 uL
Date Acquired : 11/2/2023 9:34:19 PM Acquired by : System Administrator
Date Processed : 11/2/2023 10:22:52 PM Processed by : System Administrator

<Chromatogram>

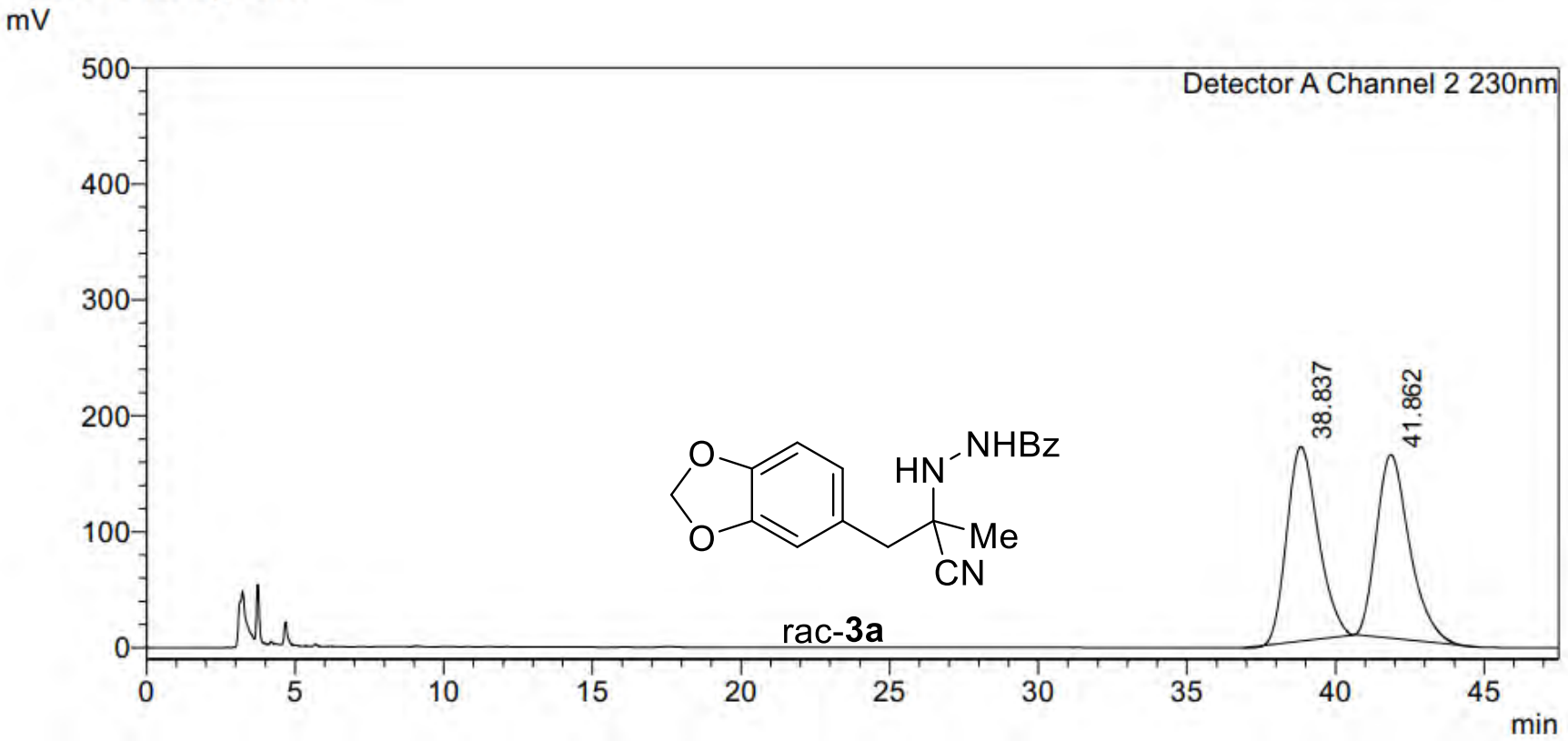

<Peak Table>

Detector A Channel 2 230nm

Peak#	Ret. Time	Area	Height	Conc.
1	38.837	12315310	167703	49.998
2	41.862	12316350	158310	50.002
Total		24631660	326013	

<Sample Information>

Sample Name : GOY-GV-22-RLigand-asy-ADH-8020-1.0-230
Sample ID :
Data Filename : GOY-GV-22-RLigand-asy-ADH-8020-1.0-231-DATA.lcd
Method Filename : GY-1.0.lcm
Batch Filename :
Vial # : 1-1 Sample Type : Unknown
Injection Volume : 20 uL
Date Acquired : 11/2/2023 8:15:04 PM Acquired by : System Administrator
Date Processed : 11/2/2023 9:23:46 PM Processed by : System Administrator

<Chromatogram>

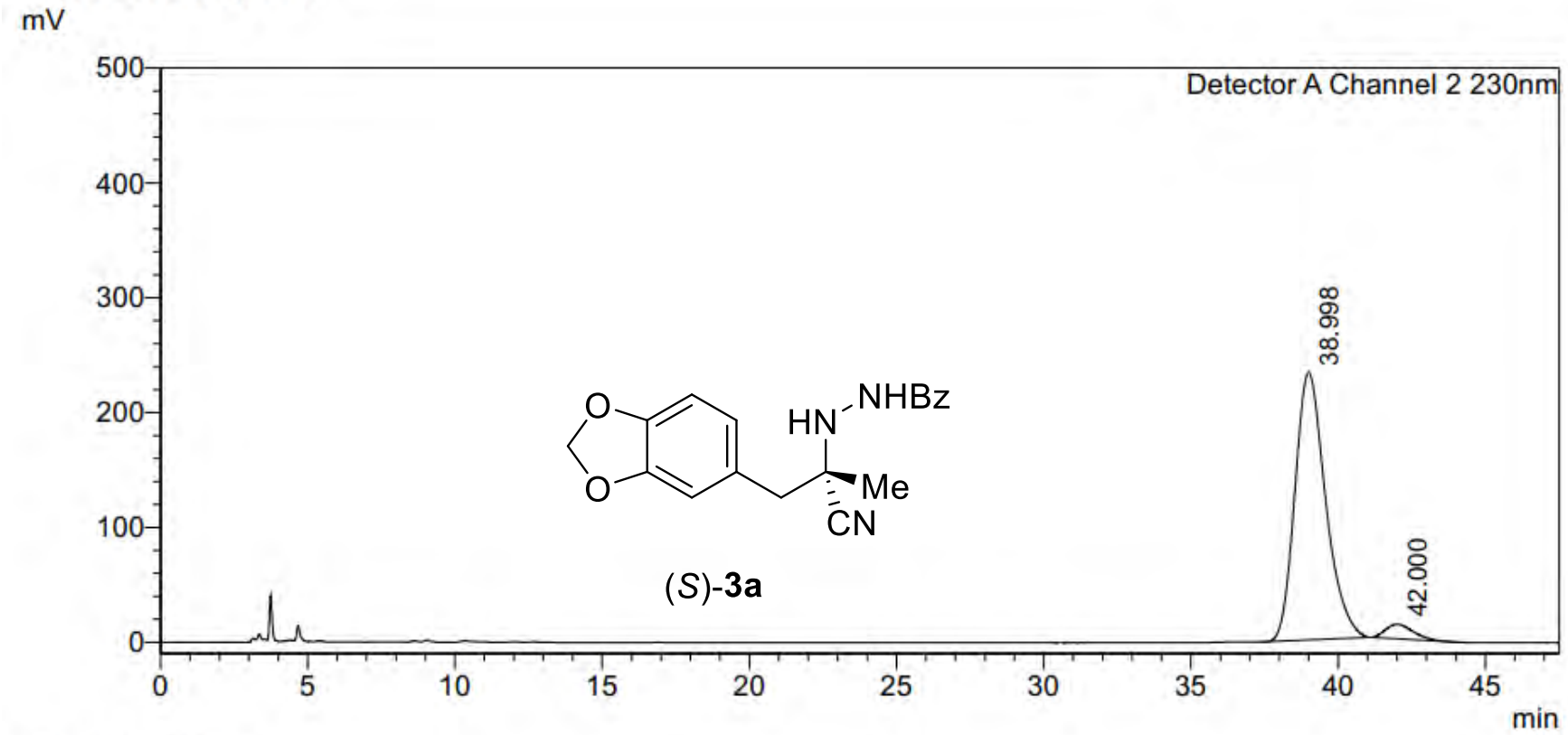

<Peak Table>

Detector A Channel 2 230nm

Peak#	Ret. Time	Area	Height	Conc.
1	38.998	16889211	232889	95.352
2	42.000	823267	12603	4.648
Total		17712478	245492	

<Sample Information>

Sample Name	: GOY-GI-54-rac-ia-8515-1.0-230		
Sample ID	:		
Data Filename	: GOY-GI-54-rac-ia-8515-1.0-231.lcd		
Method Filename	: GY-1.0.lcm		
Batch Filename	:		
Vial #	: 1-1	Sample Type	: Unknown
Injection Volume	: 20 uL		
Date Acquired	: 3/31/2021 9:16:12 AM	Acquired by	: System Administrator
Date Processed	: 3/31/2021 11:06:14 AM	Processed by	: System Administrator

<Chromatogram>

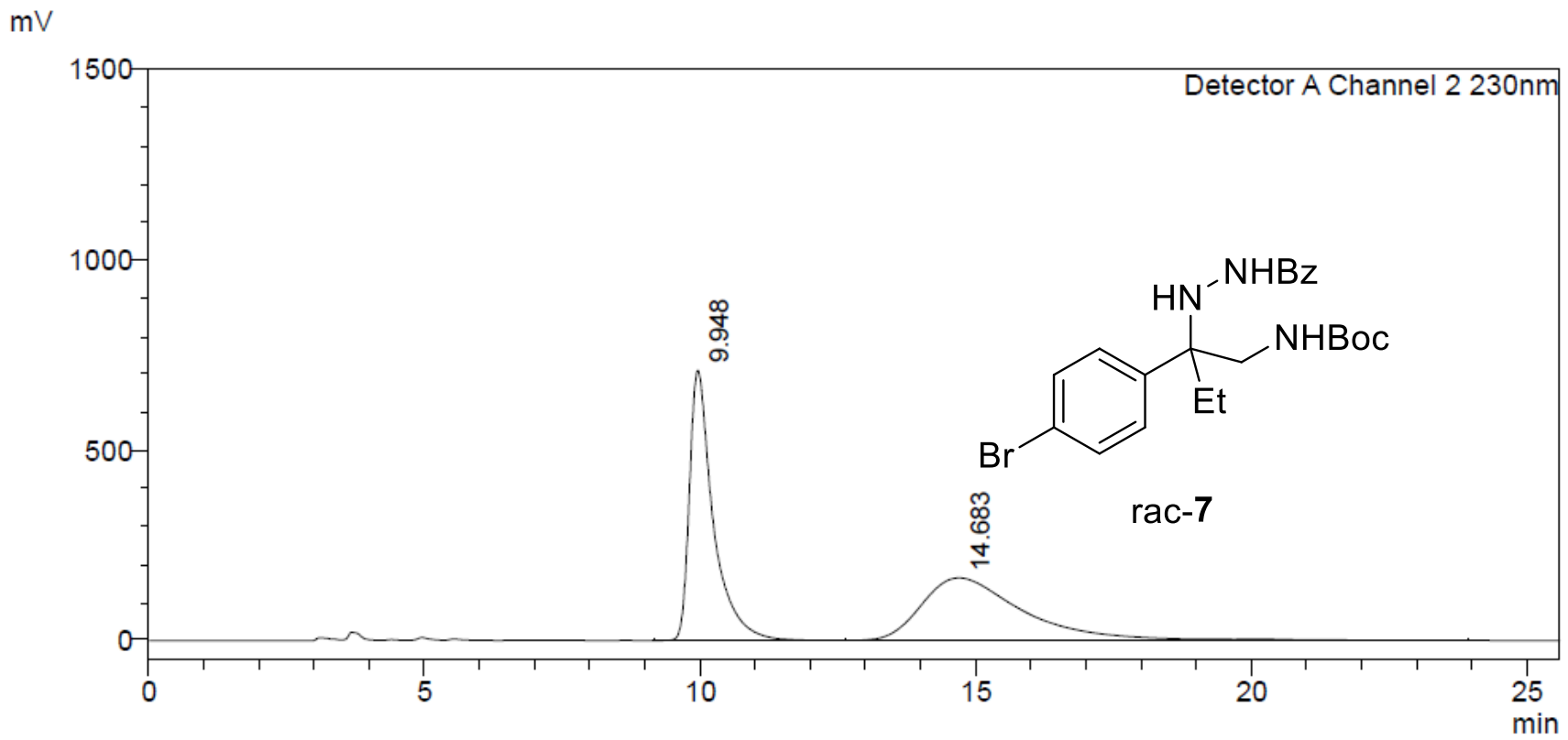

<Peak Table>

Detector A Channel 2 230nm

Peak#	Ret. Time	Area	Height	Conc.
1	9.948	21456089	710105	50.118
2	14.683	21355337	164799	49.882
Total		42811425	874904	

<Sample Information>

Sample Name	: GOY-GI-54-asy-ia-8515-1.0-230		
Sample ID	:		
Data Filename	: GOY-GI-54-asy-ia-8515-1.0-233.lcd		
Method Filename	: GY-1.0.lcm		
Batch Filename	:		
Vial #	: 1-1	Sample Type	: Unknown
Injection Volume	: 20 uL		
Date Acquired	: 3/31/2021 8:47:16 AM	Acquired by	: System Administrator
Date Processed	: 3/31/2021 11:07:19 AM	Processed by	: System Administrator

<Chromatogram>

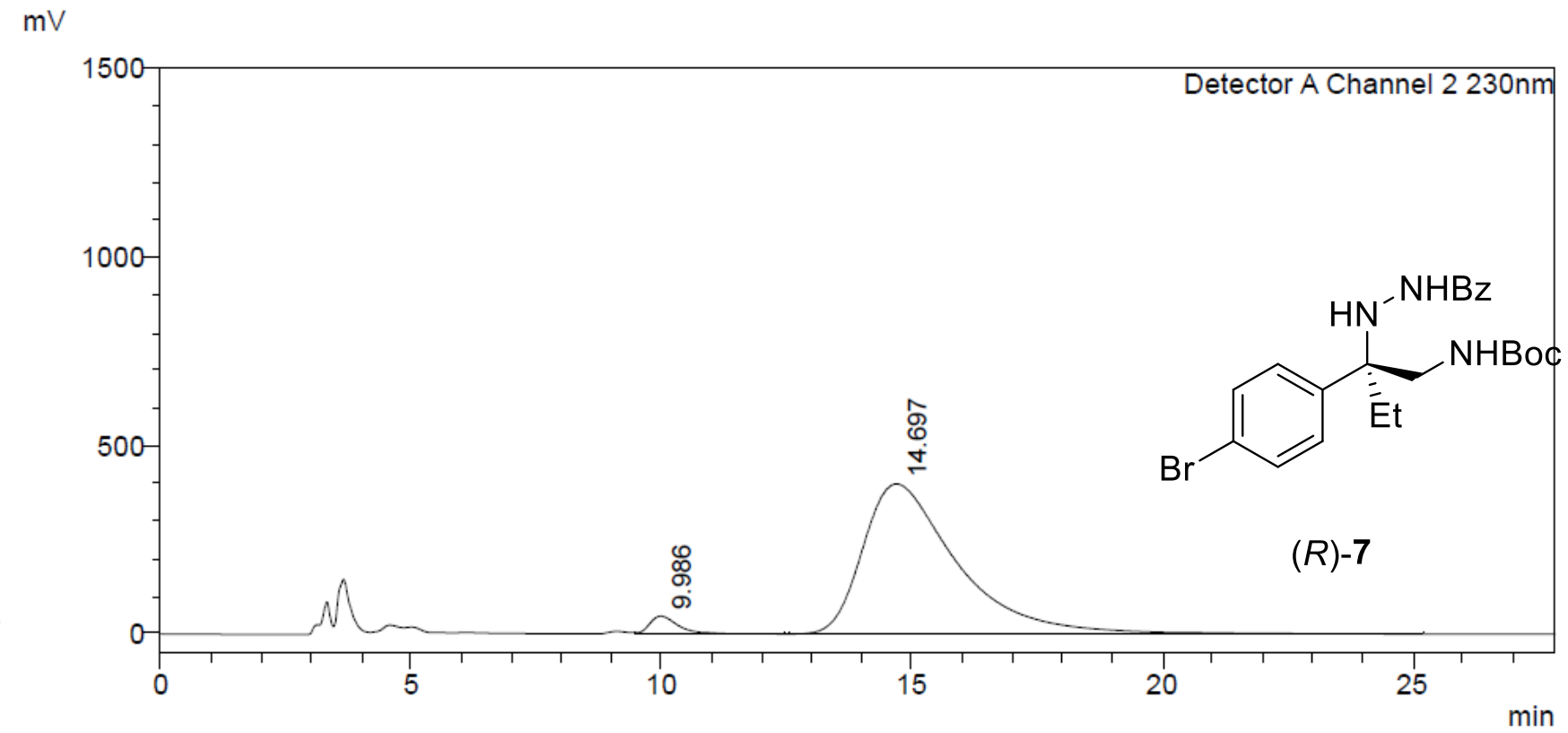

<Peak Table>

Detector A Channel 2 230nm

Peak#	Ret. Time	Area	Height	Conc.
1	9.986	1936069	47270	3.543
2	14.697	52707331	398541	96.457
Total		54643400	445810	

<Sample Information>

Sample Name	: GOY-GH-27-rac-ia-8515-1.0-230		
Sample ID	:		
Data Filename	: GOY-GH-27-rac-ia-8515-1.0-231.lcd		
Method Filename	: GY-1.0.lcm		
Batch Filename	:		
Vial #	: 1-1	Sample Type	: Unknown
Injection Volume	: 20 uL		
Date Acquired	: 12/12/2020 2:44:05 PM	Acquired by	: System Administrator
Date Processed	: 12/12/2020 3:44:42 PM	Processed by	: System Administrator

<Chromatogram>

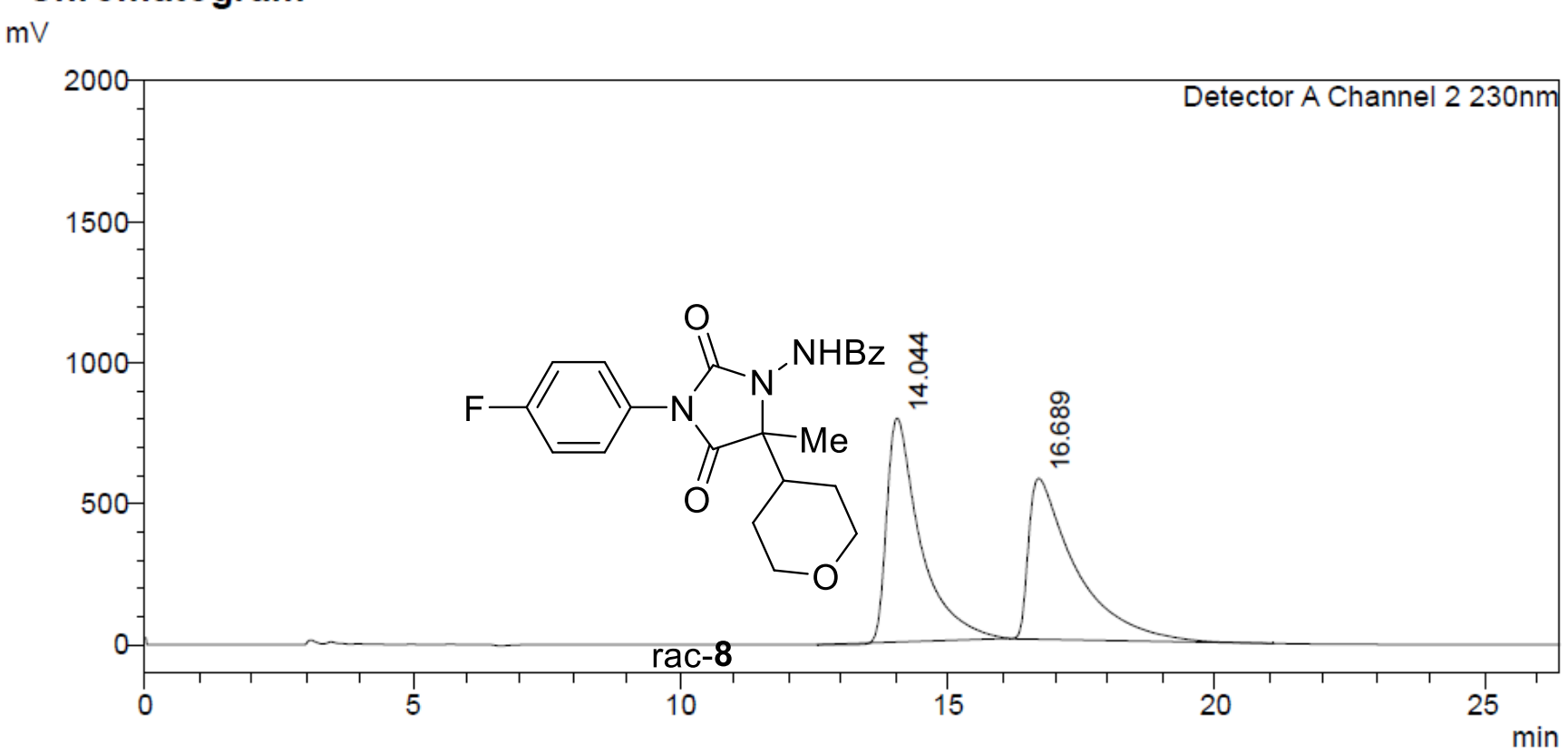

<Peak Table>

Detector A Channel 2 230nm

Peak#	Ret. Time	Area	Height	Conc.
1	14.044	34743658	795130	49.960
2	16.689	34798738	570144	50.040
Total		69542396	1365275	

<Sample Information>

Sample Name	: GOY-GH-27-asy-ia-8515-1.0-230		
Sample ID	:		
Data Filename	: GOY-GH-27-asy-ia-8515-1.0-232.lcd		
Method Filename	: GY-1.0.lcm		
Batch Filename	:		
Vial #	: 1-1	Sample Type	: Unknown
Injection Volume	: 20 uL		
Date Acquired	: 12/12/2020 3:13:20 PM	Acquired by	: System Administrator
Date Processed	: 12/12/2020 3:48:35 PM	Processed by	: System Administrator

<Chromatogram>

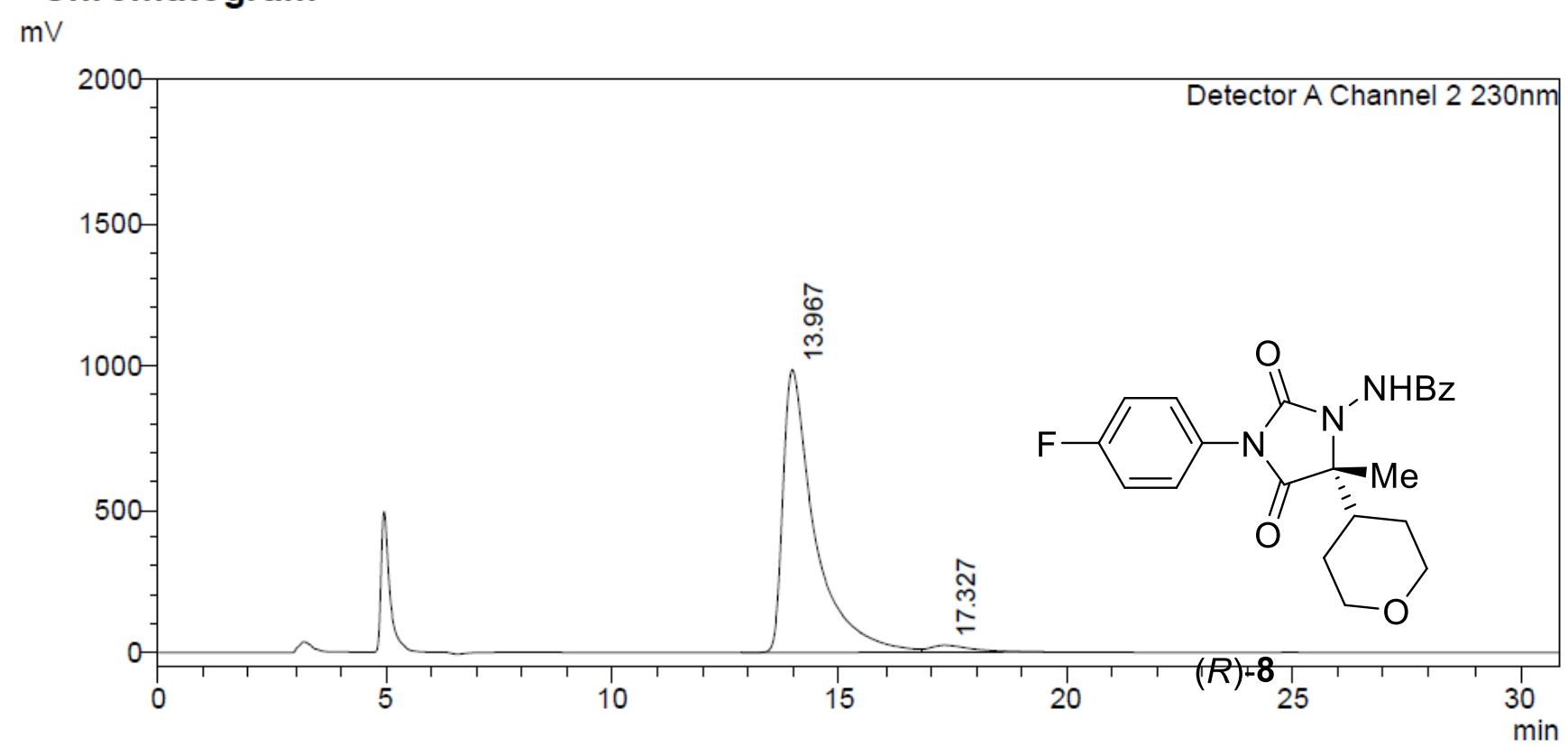

<Peak Table>

Detector A Channel 2 230nm

Peak#	Ret. Time	Area	Height	Conc.
1	13.967	48342559	989232	96.905
2	17.327	1543926	24896	3.095
Total		49886485	1014128	

<Sample Information>

Sample Name	: GOY-GQ-132-rac-IC-9703-230205-1.0-DATA		
Sample ID	:		
Data Filename	: GOY-GQ-132-rac-IC-9703-230205-1.0-DATA.lcd		
Method Filename	: GY-1.0-205.lcm		
Batch Filename	:		
Vial #	: 1-1	Sample Type	: Unknown
Injection Volume	: 10 uL		
Date Acquired	: 10/11/2022 4:32:35 PM	Acquired by	: System Administrator
Date Processed	: 10/13/2022 9:20:37 PM	Processed by	: System Administrator

<Chromatogram>

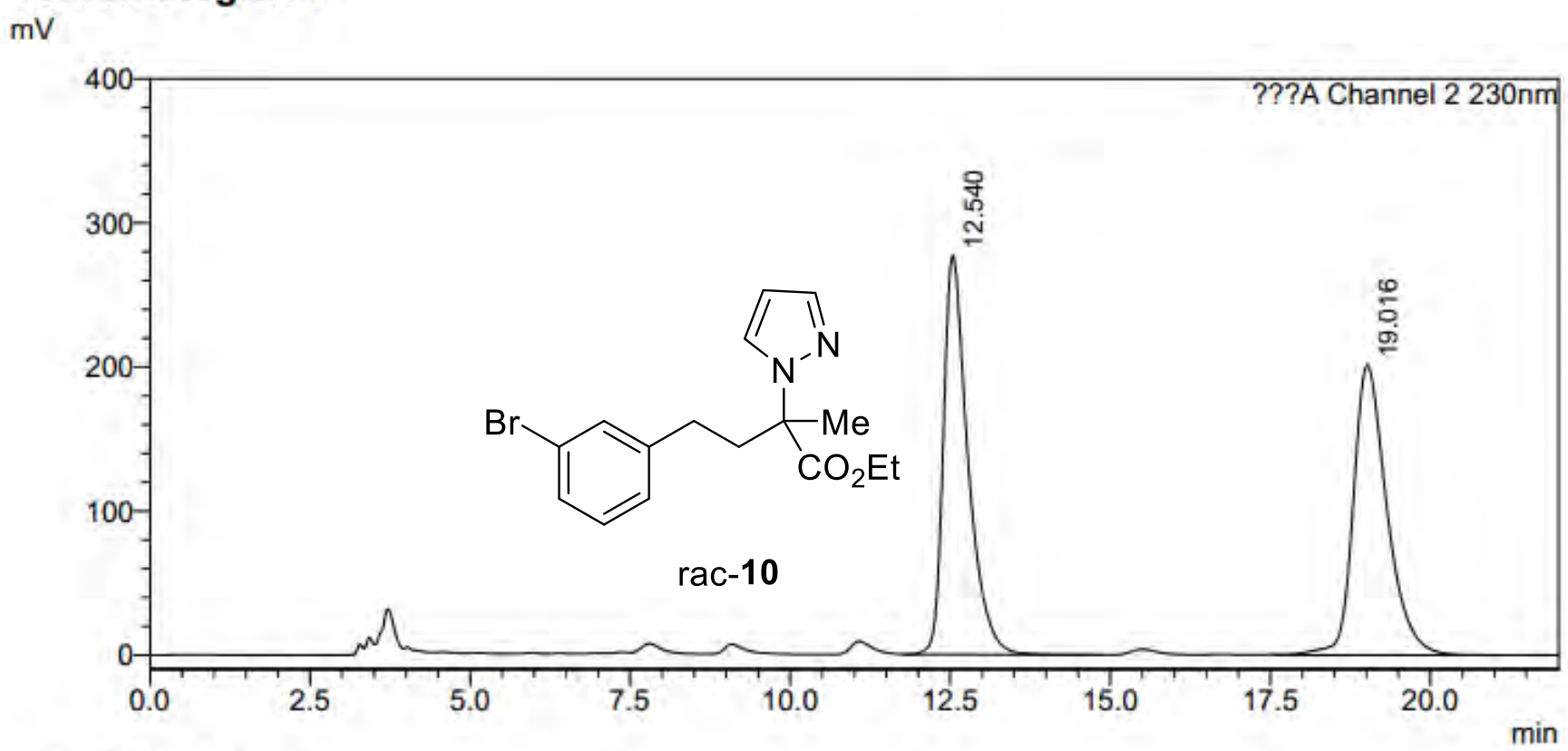

<Peak Table>

???A Channel 2 230nm

Peak#	Ret. Time	Area	Height	Conc.
1	12.540	7391505	277078	50.057
2	19.016	7374731	201262	49.943
Total		14766235	478340	

<Sample Information>

Sample Name	: GOY-GQ-132-asy-IC-9703-230205-1.0-DATA		
Sample ID	:		
Data Filename	: GOY-GQ-132-asy-IC-9703-230205-1.0-DATA.lcd		
Method Filename	: GY-1.0-205.lcm		
Batch Filename	:		
Vial #	: 1-1	Sample Type	: Unknown
Injection Volume	: 10 uL		
Date Acquired	: 10/11/2022 4:10:40 PM	Acquired by	: System Administrator
Date Processed	: 10/11/2022 4:32:02 PM	Processed by	: System Administrator

<Chromatogram>

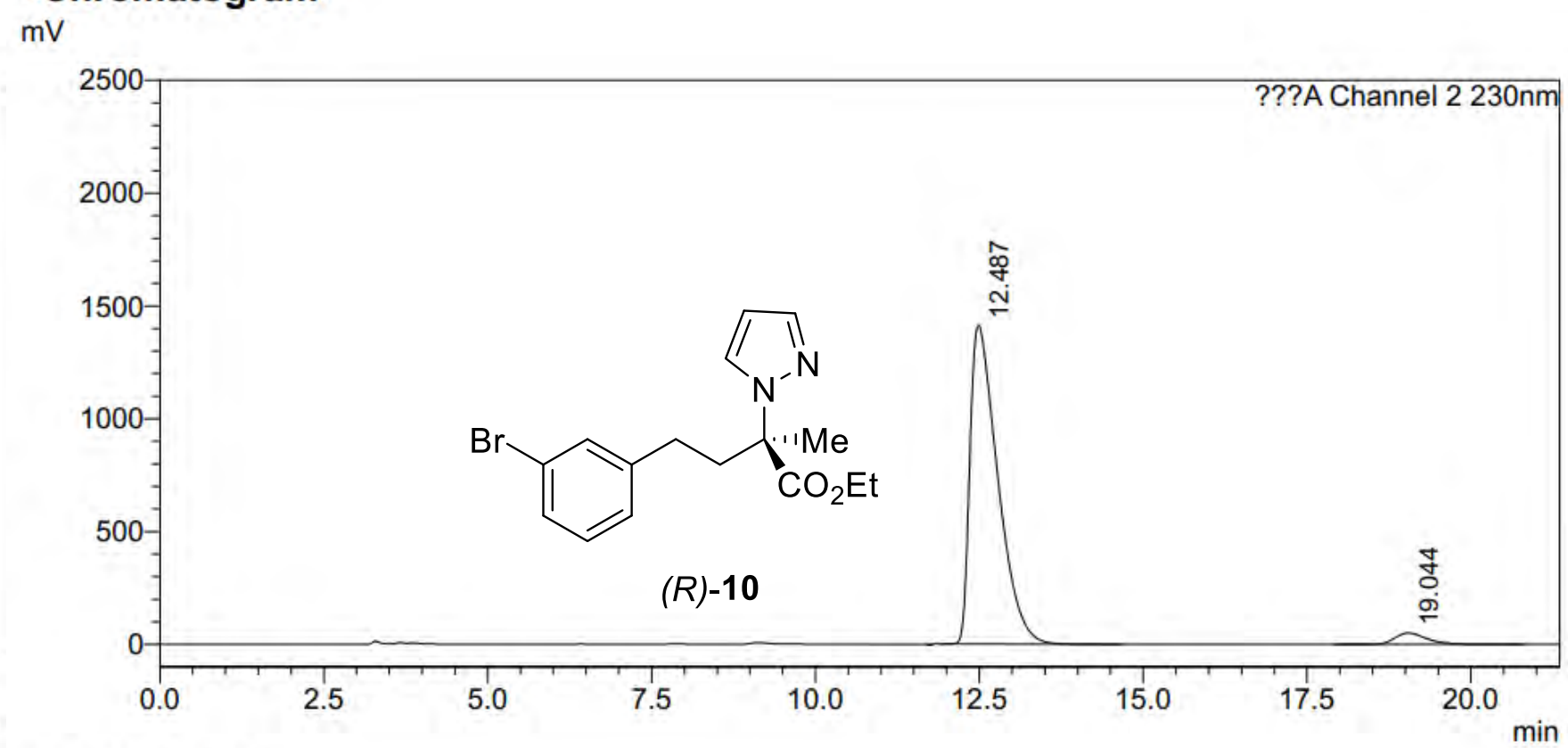

<Peak Table>

???A Channel 2 230nm

Peak#	Ret. Time	Area	Height	Conc.
1	12.487	41698942	1411914	96.005
2	19.044	1735213	51077	3.995
Total		43434155	1462991	

<Sample Information>

Sample Name	: GOY-GQ-133-rac-IC-9703-230205-1.0-DATA		
Sample ID	:		
Data Filename	: GOY-GQ-133-rac-IC-9703-230205-1.0-DATA.lcd		
Method Filename	: GY-1.0-205.lcm		
Batch Filename	:		
Vial #	: 1-1	Sample Type	: Unknown
Injection Volume	: 10 uL		
Date Acquired	: 10/11/2022 5:30:36 PM	Acquired by	: System Administrator
Date Processed	: 10/11/2022 6:14:59 PM	Processed by	: System Administrator

<Chromatogram>

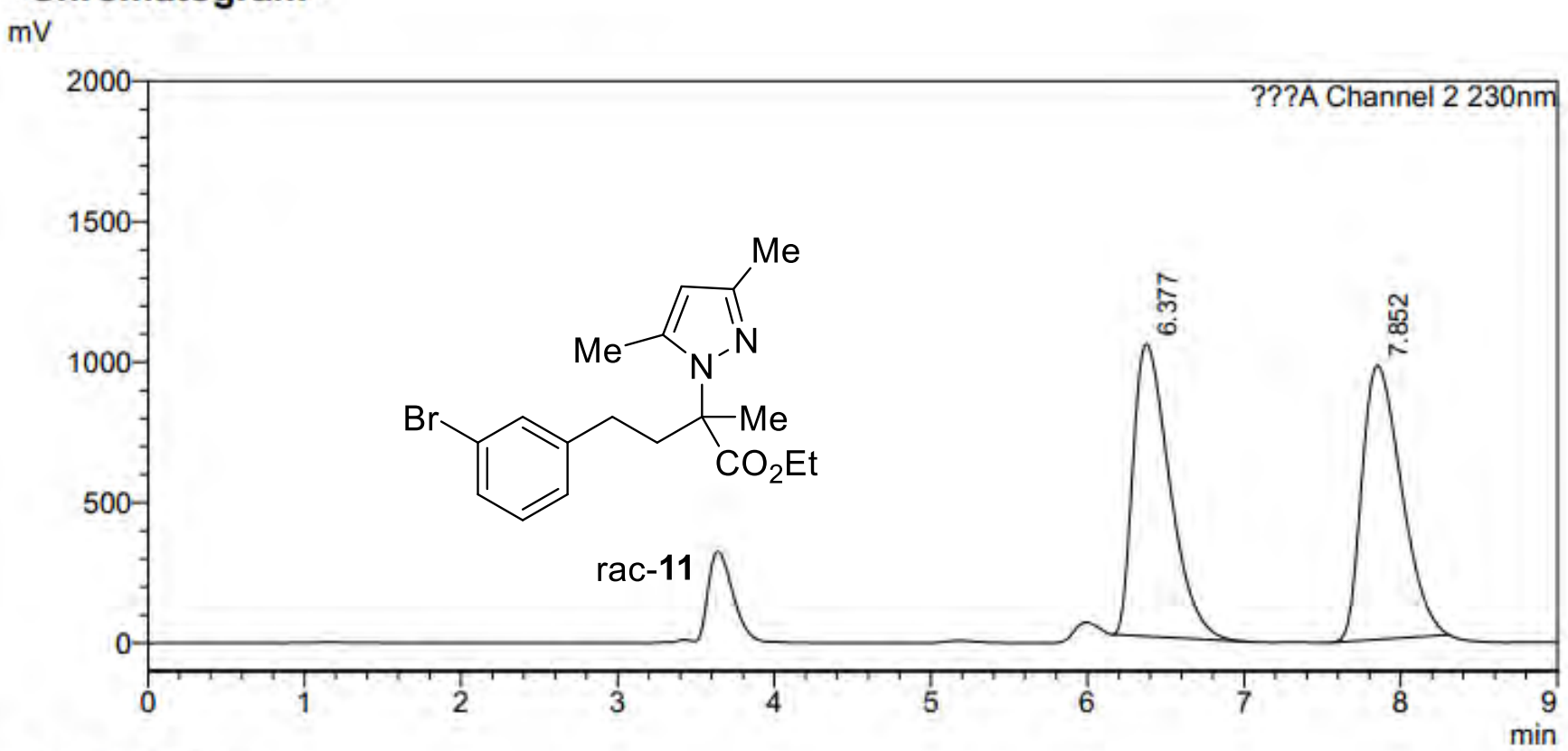

<Peak Table>

???A Channel 2 230nm

Peak#	Ret. Time	Area	Height	Conc.
1	6.377	16956283	1041266	49.707
2	7.852	17156306	974861	50.293
Total		34112589	2016127	

<Sample Information>

Sample Name	: GOY-GQ-133-asy-IC-9703-230205-1.0-DATA		
Sample ID	:		
Data Filename	: GOY-GQ-133-asy-IC-9703-230205-1.0-DATA.lcd		
Method Filename	: GY-1.0-205.lcm		
Batch Filename	:		
Vial #	: 1-1	Sample Type	: Unknown
Injection Volume	: 10 uL		
Date Acquired	: 10/11/2022 5:12:38 PM	Acquired by	: System Administrator
Date Processed	: 10/13/2022 9:27:19 PM	Processed by	: System Administrator

<Chromatogram>

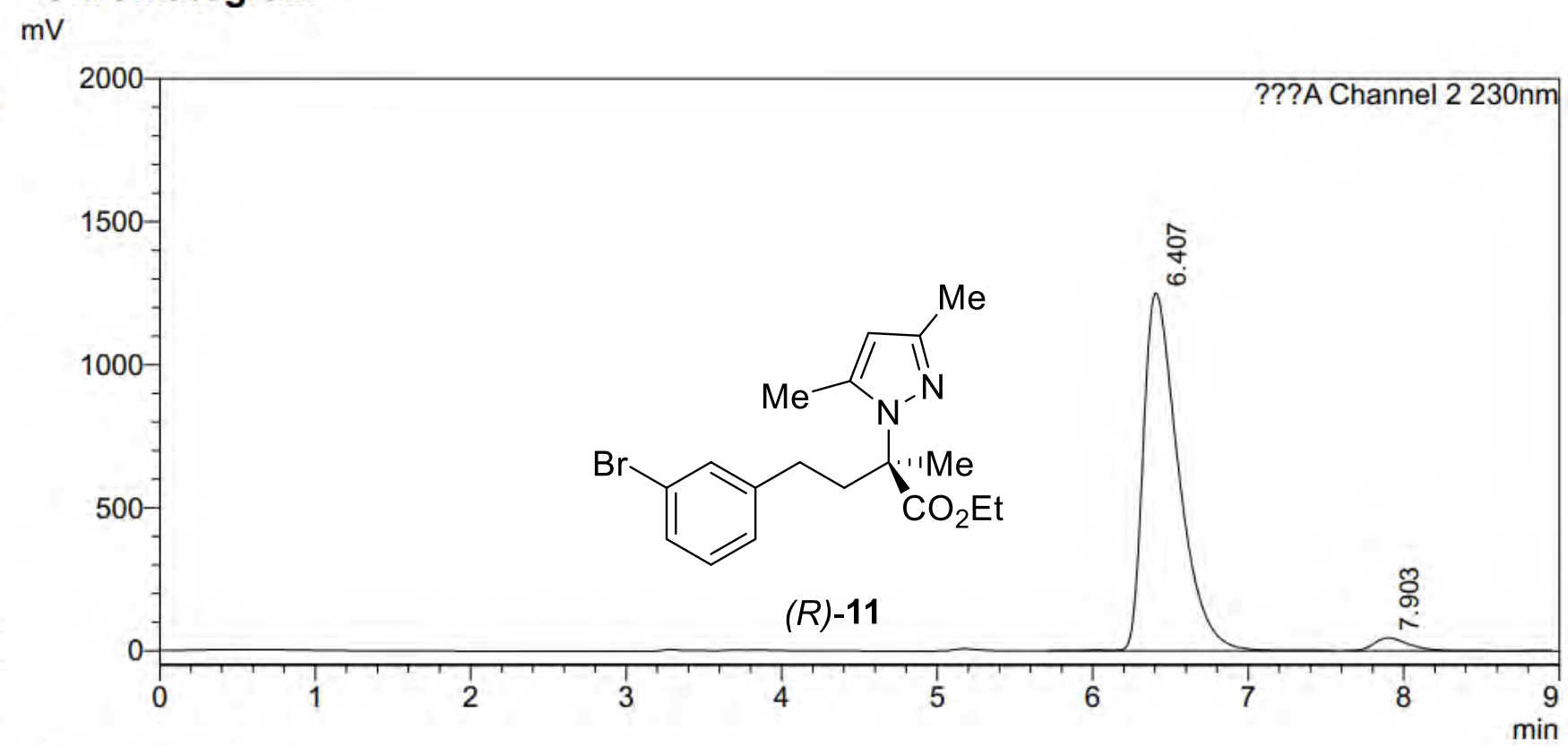

<Peak Table>

???A Channel 2 230nm

Peak#	Ret. Time	Area	Height	Conc.
1	6.407	19663598	1251525	96.557
2	7.903	701208	45391	3.443
Total		20364806	1296916	

<Sample Information>

Sample Name : GOY-GQ-137-asy-ADH-9505-230205-1.0
Sample ID :
Data Filename : GOY-GQ-137-rac-ADH-9505-230205-1.0-DATA.lcd
Method Filename : GY-1.0-205.lcm
Batch Filename :
Vial # : 1-1 Sample Type : Unknown
Injection Volume : 10 uL
Date Acquired : 10/12/2022 9:48:58 AM Acquired by : System Administrator
Date Processed : 10/13/2022 9:34:17 PM Processed by : System Administrator

<Chromatogram>

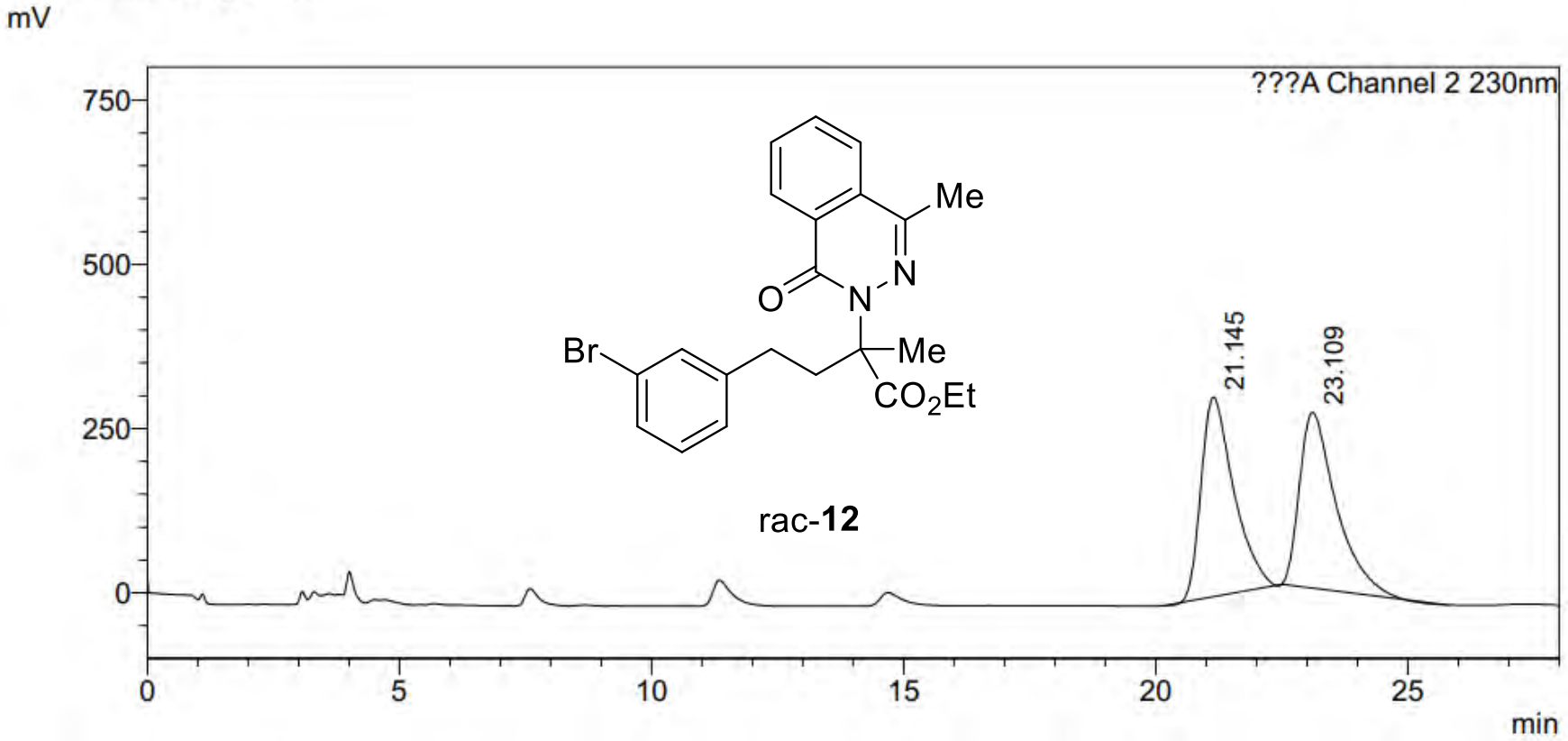

<Peak Table>

???A Channel 2 230nm

Peak#	Ret. Time	Area	Height	Conc.
1	21.145	13699498	303502	50.357
2	23.109	13505428	267225	49.643
Total		27204926	570727	

<Sample Information>

Sample Name : GOY-GQ-137-asy-ADH-9505-230205-1.0
Sample ID :
Data Filename : GOY-GQ-137-asy-ADH-9505-230205-1.0-DATA.lcd
Method Filename : GY-1.0-205.lcm
Batch Filename :
Vial # : 1-1 Sample Type : Unknown
Injection Volume : 10 uL
Date Acquired : 10/12/2022 10:36:00 AM Acquired by : System Administrator
Date Processed : 10/12/2022 11:11:27 AM Processed by : System Administrator

<Chromatogram>

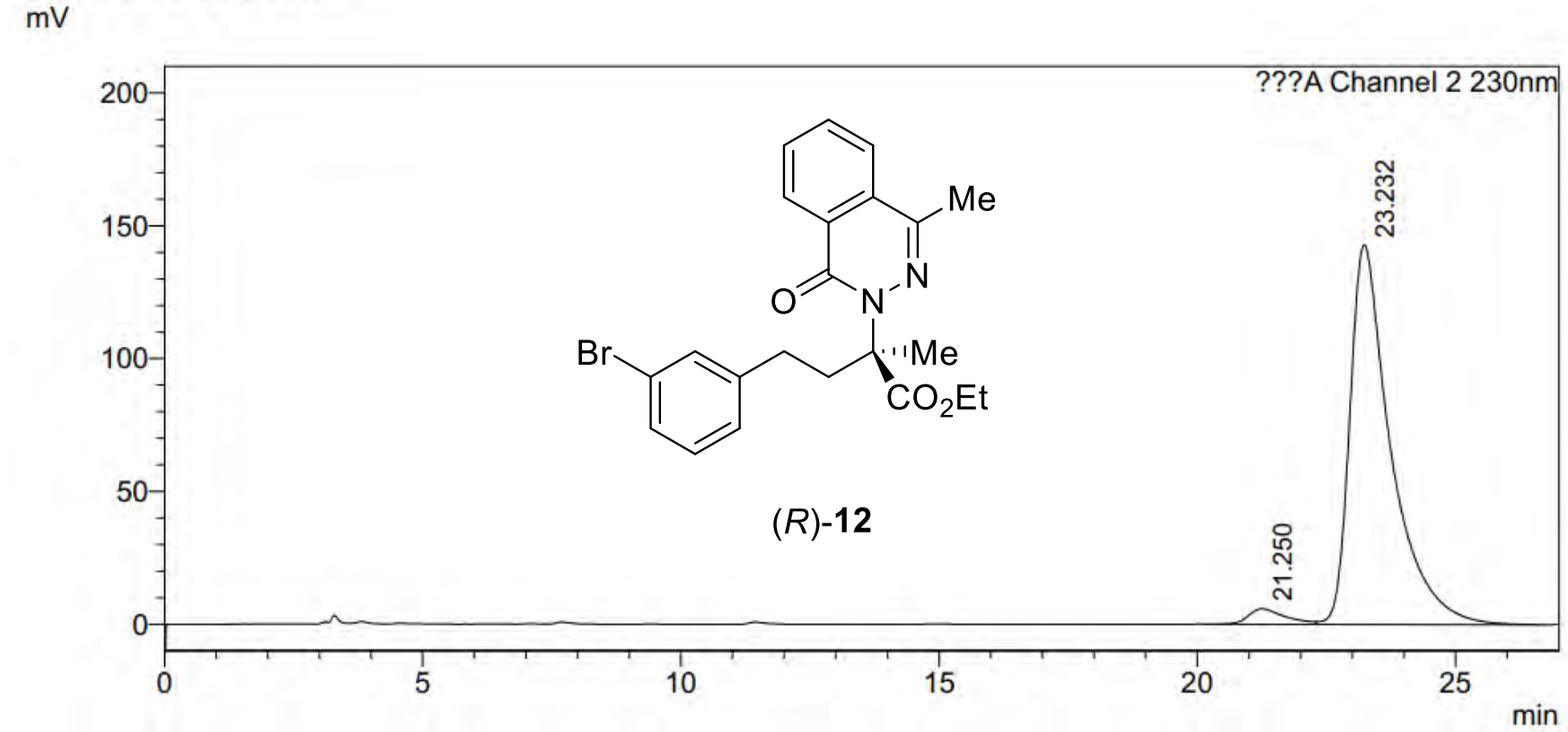

<Peak Table>

???A Channel 2 230nm

Peak#	Ret. Time	Area	Height	Conc.
1	21.250	301977	5951	3.689
2	23.232	7884924	142924	96.311
Total		8186901	148875	

14. References

[1] a) X.-P. Yin, P.-W. Xu, K. Dong, K. Liao, F. Zhou, J. Zhou, *Acta Chim. Sin.* **2015**, *73*, 685; b) R. R. Paccani, D. Donati, S. Fusi, L. Latterini, G. Farina, V. Zanirato, M. Olivucci, *J. Org. Chem.* **2012**, *77*, 1738; c) J. Tummatorn, C. Thongsornkleeb, Ruchirawat, S. Ruchirawat, Thongaram, P. Thongaram, B. Kaewmee, *Synthesis* **2015**, *47*, 323; d) Y.-N. Duan, Z. Zhang, C. Zhang, *Org. Lett.* **2016**, *18*, 6176.

[2] Y. Hu, Z. Zhang, J. Zhang, Y. Liu,I. D. Gridnev,W. Zhang, *Angew. Chem. Int. Ed.* **2019**, *58*, 15767.

[3] Y. Gong, C. Wang, F. Zhou, K. Liao, X.-Y. Wang, Y. Sun, Y.-X. Zhang, Z. Tu, X. Wang, J. Zhou, Sulfonyl-PYBOX Ligands Enable Kinetic Resolution of α-Tertiary Azides by CuAAC. *Angew. Chem. Int. Ed.* **2023**, *62*, e202301470.

[4]Y.-H. Hu, Z.-F. Zhang, J. Zhang, Y.-G. Liu, I. D. Gridnev, W.-B. Zhang, *Angew. Chem. Int. Ed.* **2019**, *58*, 15767; *Angew. Chem.* **2019**, *131*, 15914.

[5] L. Zhang, X.-F. Zheng, G. Linn, K. Zhao, *Synlett* **2007**, 3, 374.

[6] J. M. Keith, E. N. Jacobsen, *Org. Lett.* **2004**, *6*, 153.

[7] H.-Y. Yang, P. Xu, P. Chuanprasit, H. Hirao, J.-R. Zhou, *Angew. Chem. Int. Ed.* **2015**, *54*, 5112; *Angew. Chem.* **2015**, *127*, 52.

[8] C. Ogawa, M. Sugiura, S. Kobayashi, *J. Org. Chem.* **2002**, *67*, 5359.

[9] H. M. Mack, E. A. Davis, B. Kadkhodayan, R. A. Taylor, D. C. Duncan, C. F. Beam, *J. Heterocycl. Chem.* **1987**, *24*, 1733.

[10] Y. Upegui, K. Rios, W. Quiñones, F. Echeverri, R. Archbold, J. D. Murillo, F. Torres, G. Escobar, I. D. Vélez, S. M. Robledo, *Med. Chem. Res.* **2019**, *28*, 2184.

[11] A.K. Yadav, L. D. S. Yadav, *Tetrahedron Lett.* **2014**, *55*, 2065.

[12] Keith, J. M.; Jacobsen, E. N. Asymmetric Hydrocyanation of Hydrazones Catalyzed by Lanthanide-PYBOX Complexes. *Org. Lett.* **2004**, *6*, 153.

[13] À. Pericas, A.Shafir, A. Vallribera, *Org. Lett.* **2011**, *15*, 1448.

[14] D. H. Ryu, E. J. Corey. Highly Enantioselective Cyanosilylation of Aldehydes Catalyzed by a Chiral Oxazaborolidinium Ion. *J. Am. Chem. Soc.* **2004**, *126*, 8106.

[15] X. Chen, Q. Li, L. Andrews, Y. Gong. Infrared Spectroscopic and Theoretical Studies of Group 3 Metal Isocyanide Molecules. *J. Phys. Chem. A* **2018**, *122*, 7099.

[16] Supplementary crystallographic data have been deposited at the Cambridge Crystallographic Data Center (CCDC number: 2221754).

[17] Supplementary crystallographic data have been deposited at the Cambridge Crystallographic Data Center (CCDC number: 2422479).

[18] Supplementary crystallographic data have been deposited at the Cambridge Crystallographic Data Center (CCDC number: 2044037).

[19] Supplementary crystallographic data have been deposited at the Cambridge Crystallographic Data Center (CCDC number: 2390295).

[20] Supplementary crystallographic data have been deposited at the Cambridge Crystallographic Data Center (CCDC number: 2390296).
