# Supplementary material for: Sulfonyl-PYBOX/ErCl3 complex enable highly enantioselective synthesis of α,α-dialkyl and α-alkyl-α-aryl hydrazinonitriles
Source: Nat Commun. 2026 Jun 18;17:7688. doi: 10.1038/s41467-026-74600-0 (PMC13434049; doi:10.1038/s41467-026-74600-0)
Supplement: Supplementary file 2 [file 41467_2026_74600_MOESM2_ESM.pdf]

nature portfolio

Peer Review File

Sulfonyl-PYBOX/$ErCl_3$ Complex Enable Highly Enantioselective Synthesis of α,α-Dialkyl and α-Alkyl-α-aryl Hydrazinonitriles

Corresponding Author: Professor Jian Zhou

This file contains all reviewer reports in order by version, followed by all author rebuttals in order by version.

Version 1:

Reviewer comments:

Reviewer #1

(Remarks to the Author)
The authors introduce an asymmetric cyanation of both aryl and aliphatic ketone hydrazones. ErCl3 in combination with a sterically confined PYBOX, was identified as an efficient catalyst for this process. Furthermore, author demonstrated that introducing a bulky shielding group at the C4 position of pyridine may improve the chiral pocket of PYBOX derived monomeric metal complexes. Which is conducive to attaining a better enantioselective control in some challenging asymmetric reactions that involve monomeric metal center. Notably, the subsequent applications and transformations have significant potential for use in drug synthesis. The reaction exhibits a broad substrate scope and excellent functional group compatibility. Furthermore, this work presents a novel strategic approach to access tetrasubstituted α-hydrazino nitriles derivatives. Overall, I find the chemistry compelling and support its publication. after the authors address the following points:
1. In my opinion, author should further expand the substrate scope such as diaryl hydrazones. Furthermore, what about the heteroaryl (thienyl, furyl etc.) hydrazones?
2. The synthesis method of the substrate or the references need to be provided by the authors. Moreover, new substrates need to provide NMR, HRMs and other characterization data.
3. Rf values should be provided for all compounds.

Reviewer #2

(Remarks to the Author)
Zhou, et al described a highly enantioselective Strecker reaction of ketone hydrazones providing the functionally and optically enriched Ca-tetrasubstituted nitriles in high yield and with up to 96% ee. The methodology is very general, both aryl alkyl ketone and dialkyl ketone hydrazones are applicable. The scale-up experiment and transformation of the newly obtained adducts are fully exhibited. Noteworthy is the brief preparation L-Carbidopa in 82% overall yield by only 4 steps. The mechanism was also well investigated. Biological analysis discovered that chiral alpha-hydrazino nitriles shows good insecticidal activity.
Enantioselective nucleophilic addition to ketoimine is very challenging, and up to now, no highly enantioselective nucleophilic addition has been addressed for ketone hydrazones. This work presents the first successful example of ketone hydrazones. To solve the problem, the author employed a new sterically confined pyridinebisoxazoline ligand. In my own view, the present work seems to be an excellent extension of E. N. Jacobsen in 2004. Same metal (ErCl3) and same kind of pyridine PYBOX are combined to catalyze the reaction but solve the real problem! I prefer to recommend the work to be published in Nature Commun. after minor revisions.
Additional concern:
1. Although the authors emphasized that a suitable C4 relay group can help the formation of a sterically constrained chiral pocket for nucleophilic addition of ketone hydrazones, I do not see how 4- phenylsulfonyl effect the reaction. By steric hindrance or electronic effect? Is DFT investigation helpful to elucidate the stereochemistry outcome? How about the reaction if switch phenylsulfonyl group to trifluoromethane sulfonyl (CF3SO2-) group?

Reviewer #3

(Remarks to the Author)
Asymmetric cyanation to prochiral C=N double bonds is a useful transformation affording enantiomerically enriched α-aminonitrile derivatives. The reaction using ketone derived imine derivatives forming the chiral tetrasubstituted carbon centers is challenging from both fundamental and practical viewpoints. The submitted manuscript reports the reaction of *N*-benzoyl ketone hydrazones using the sulfonyl-PYBOX/$ErCl^3$ catalyst. The PYBOX/$ErCl^3$ catalyst for *N*-benzoyl hydrazones was reported by Jacobsen (*Org. Lett.* **2004**, *6*, 153), therefore the basic principle of catalytic function is known. The original PYBOX/$ErCl^3$ catalyst showed medium enantioselectivity in the reaction of the acetophenone derived hydrazone (44% ee), but the novel sulfonyl-PYBOX/$ErCl^3$ catalyst achieves much higher ee of 90% using the same substrate. A variety of hydrazones derived from alkyl aryl ketones is applicable to this reaction. Notably, this catalyst enables high enantioselectivity in the reaction of hydrazones derived from alkyl methyl ketones. Mechanistic studies and synthetic and biological applications are successfully performed. Originality of basic catalytic design is not excellent, but improvement of enantioselectivity and expansion of substrate scope by using the sulfonyl-PYBOX ligand are significant. Such ligand-modification concept seems to affect the catalyst-design of asymmetric reaction. I recommend this manuscript to be published in this journal.
Other comments:
1. Page 9, first paragraph: Effect of C4 sulfonyl group on the pyridine of PYBOX is a key issue of this manuscript. It is described as “adjust the steric effect” and “form a more contact chiral pocket” in the text. These are vague expressions. The more detailed explanations are required.
2. Title, Fig. 1, and Table 1: “α,α-Dialkyl” is shown in the manuscript title. However, compounds shown in the first part of Table 1 are all α-alkyl-α-methyl hydrazinonitriles. I recommend showing the results of formation of α-alkyl-α-ethyl hydrazinonitrile, α-alkyl-α-isopropyl hydrazinonitrile, etc., otherwise the indication of title and molecular structure of Fig. 1d should be changed as “α-alkyl-α-methyl hydrazinonitriles”.
3. Fig 1: α-*tert*-Butyl-α-methyl hydrazinonitrile, α-*tert*-butyl-α-aryl hydrazinonitrile, and α-*iso*-propyl-α-aryl hydrazinonitrile are required to clarify the scope of reaction.
4. Supporting Information: Enantiomer peak separation of HPLC analysis for **3p** and **4a** is not appropriate. Reanalysis is required.

Reviewer #4

(Remarks to the Author)
The authors discovered a highly enantioselective nucleophilic addition reaction of simple ketone-derived hydrazones, and investigated the reaction mechanism and insecticidal activity. Here are some suggestions:
1. In the preliminary screening of insecticidal activity, only some of the target compounds were screened instead of all. What is the rationale for selecting these compounds?
2. The significant difference analysis should be supplemented in Figure 5 (b)
3. The authors found that compound 3u exhibited superior insecticidal activity against Aphid gossypii to Deltamethrin. Why did the authors only choose Deltamethrin as the positive control? Including other highly effective insecticides as positive controls would provide a better comparative assessment of the insecticidal potential of compound 3u.
4. Compound 3u showed promising insecticidal activity. The authors could further investigate its insecticidal mechanism to provide theoretical guidance for the development of novel insecticides. Of course, if this falls outside the scope of the authors' research, collaborative efforts with other researchers could be considered, and further investigation into the insecticidal mechanism could be conducted in subsequent studies.

Version 2:

Reviewer comments:

Reviewer #1

(Remarks to the Author)
In the revised manuscript, because the comments raised by the reviewer have been properly answered, the current manuscript can be accepted.

Reviewer #2

(Remarks to the Author)
My concerns have been fully addressed, anf I have no other questions. I recommend the work to be published as it is!

Reviewer #3

(Remarks to the Author)
The revised manuscript reporting the sulfonyl-PYBOX/$ErCl_3$-catalyzed asymmetric cyanation of N-benzoyl hydrazones was added with several useful data to prove generality of this catalytic transformation. Importance of the sulfonyl group at the pyridine C4 position of PYBOX ligand was also improved, although the detailed role of this functionality still includes ambiguity. I recommend this manuscript to be published as it stands.

Reviewer #4

(Remarks to the Author)
The authors have carefully revised the manuscript in accordance with the reviewers' comments. Acceptance is recommended.

Open Access This Peer Review File is licensed under a Creative Commons Attribution 4.0 International License, which permits use, sharing, adaptation, distribution and reproduction in any medium or format, as long as you give appropriate credit to the original author(s) and the source, provide a link to the Creative Commons license, and indicate if changes were made.
In cases where reviewers are anonymous, credit should be given to 'Anonymous Referee' and the source.
The images or other third party material in this Peer Review File are included in the article's Creative Commons license, unless indicated otherwise in a credit line to the material. If material is not included in the article's Creative Commons license and your intended use is not permitted by statutory regulation or exceeds the permitted use, you will need to obtain permission directly from the copyright holder.
To view a copy of this license, visit https://creativecommons.org/licenses/by/4.0/

Jian Zhou
Shanghai Key Laboratory of Green Chemistry and Chemical Processes,
School of Chemistry and Molecular Engineering, East China Normal University
3663 N. Zhongshan Road, Shanghai 200062, China
Email: jzhou@chem.ecnu.edu.cn

Reviewer comments:

Reviewer #1 (Remarks to the Author)

The authors introduce an asymmetric cyanation of both aryl and aliphatic ketone hydrazones. $ErCl_3$ in combination with a sterically confined PYBOX, was identified as an efficient catalyst for this process. Furthermore, author demonstrated that introducing a bulky shielding group at the C4 position of pyridine may improve the chiral pocket of PYBOX derived monomeric metal complexes. Which is conducive to attaining a better enantioselective control in some challenging asymmetric reactions that involve monomeric metal center. Notably, the subsequent applications and transformations have significant potential for use in drug synthesis. The reaction exhibits a broad substrate scope and excellent functional group compatibility. Furthermore, this work presents a novel strategic approach to access tetrasubstituted α-hydrazino nitriles derivatives. Overall, I find the chemistry compelling and support its publication. after the authors address the following points:

We thank reviewer 1 for her/his kind recommendation.

1. In my opinion, author should further expand the substrate scope such as diaryl hydrazones. Furthermore, what about the heteroaryl (thienyl, furyl etc.) hydrazones?

As requested by reviewers, we have further expanded the substrate scope by developing new sterically confined PYBOX ligands. Totally twelve examples have been added, including some challenging substrates. Specifically, the cyanation of 2-methylfuryl, 2-methylthiophenyl, or 3-methylthiophenyl ketone derived hydrazones were realized in high enantioselectivity. After screening PYBOX bearing different bulky shielding groups at the pyridine C4 position, the phosphate-PYBOX **L_4** proved to be better than the sulfonyl-PYBOX **L_6**, delivering the corresponding chiral hydrazines **3ao–aq** in 81–83% ee. These results have been added to Table 1 of the revised manuscript.

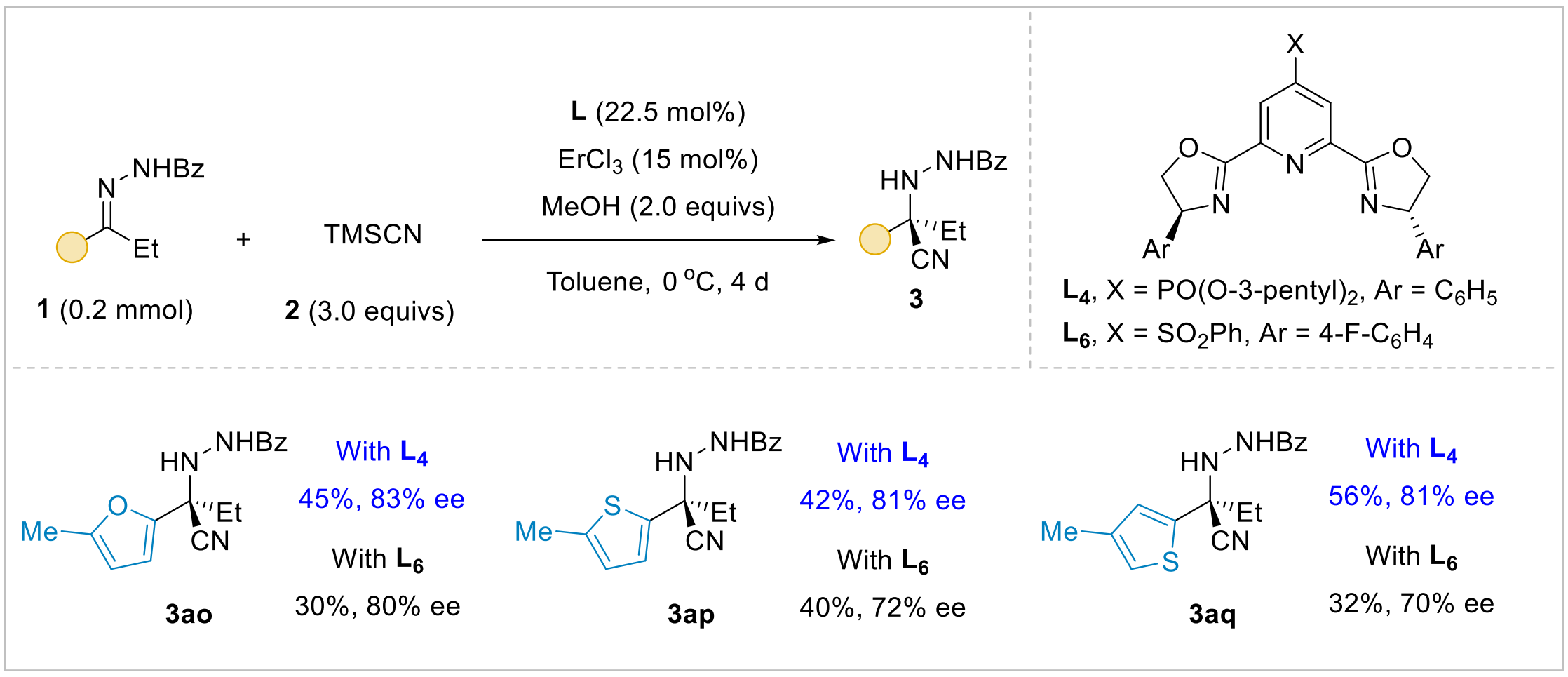

We also tried the asymmetric cyanation of diarylketone derived hydrazones. Unfortunately, despite the screening of a series of reaction parameters such as PYBOX ligands, additives, and solvents, no reaction took place. It should be noted that the enantioselective nucleophilic addition to diaryl ketimines is considerably more challenging than that

to aryl-alkyl ketimines, likely due to their decreased electrophilicity and increased steric bulk (Feng X. M. et al. *Chem. Rev.* **2011**, *111*, 6947). To our knowledge, despite intensive studies into ketimine cyanation, only one protocol enabled diaryl ketimines to work successfully (Feng X. M. et al. *Angew. Chem. Int. Ed.* **2007**, *46*, 8468).

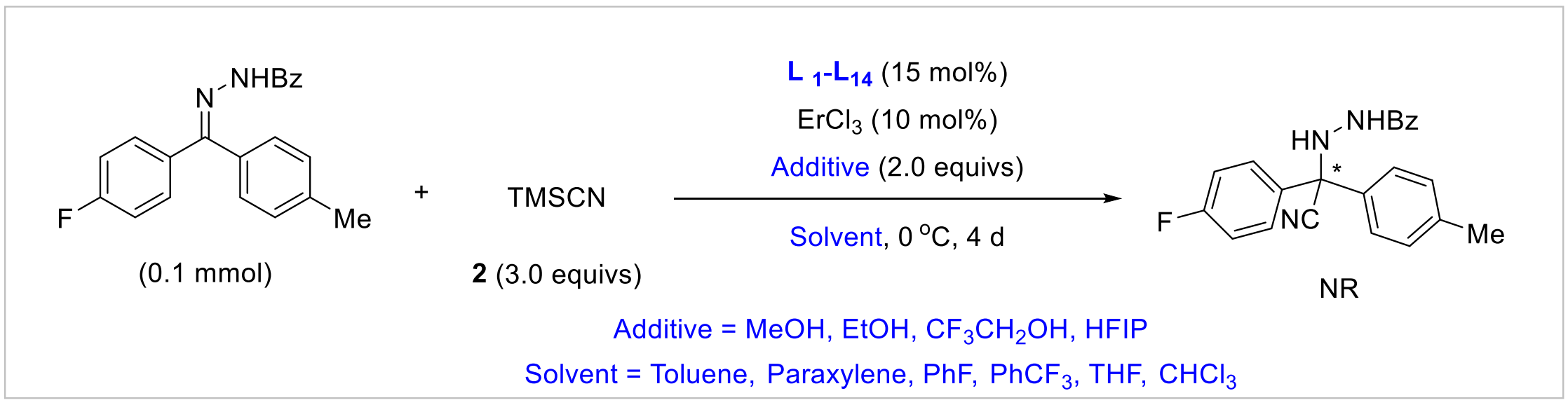

2. The synthesis method of the substrate or the references need to be provided by the authors. Moreover, new substrates need to provide NMR, HRMs and other characterization data.

3. Rf values should be provided for all compounds.

As suggested, the synthesis method, related characterization data and Rf values of the hydrazone substrates have been added to Part 3 of the Supporting Information (SI). The hydrazones **1** were prepared from the corresponding ketones or aldehydes according to the following general procedure: To a solution of ketone (5.0 mmol) and benzoyl hydrazine (1.1 equiv) in ethanol (5.0 mL) was slowly added AcOH (10 mol%) at room temperature. The resulting mixture was stirred for 2–8 h. The precipitate was filtered and recrystallized from ethanol to afford the desired products. The representative characterization data of hydrazone **1a** are shown as follows:

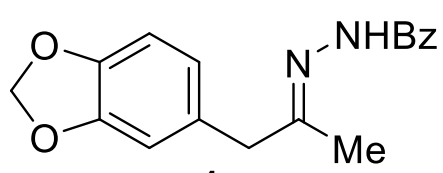

1a

1a was obtained in 72% yield as white solid (Rf value: 0.2, PE/EtOAc = 2:1). m.p. 197-199 °C; ^{1}H NMR (400 MHz, DMSO-*d*$_6$): δ 10.48 (s, 1H), 7.83 (d, *J* = 7.5 Hz, 2H), 7.55-7.46 (m, 3H), 6.88-6.73 (m, 3H), 5.99 (s, 2H), 3.53 (s, 2H), 1.86 (s, 3H); ^{13}C NMR (100 MHz, DMSO-*d*$_6$): δ 163.4, 161.8, 147.4, 146.0, 134.1, 131.4, 130.9, 130.0, 128.3, 127.7, 122.0, 109.2, 108.3, 100.9, 44.2, 16.3. IR (neat): 3005, 2360, 1717, 1440, 1364, 1225 cm^{-1}; HRMS (ESI): Exact mass calcd for $C_{17}H_{16}N_2NaO_3$ [M+Na]$^+$: 319.1053, Found: 319.1052.

Reviewer #2 (Remarks to the Author)

Zhou, et al described a highly enantioselective Strecker reaction of ketone hydrazones providing the functionally and optically enriched Ca-tetrasubstituted nitriles in high yield and with up to 96% ee. The methodology is very general, both aryl alkyl ketone and dialkyl ketone hydrazones are applicable. The scale-up experiment and transformation of the newly obtained adducts are fully exhibited. Noteworthy is the brief preparation L-Carbidopa in 82% overall yield by only 4 steps. The mechanism was also well investigated. Biological analysis discovered that chiral alpha-hydrazino nitriles shows good insecticidal activity.

Enantioselective nucleophilic addition to ketoimine is very challenging, and up to now, no highly enantioselective nucleophilic addition has been addressed for ketone hydrazones. This work presents the first successful example of ketone hydrazones. To solve the problem, the author employed a new sterically confined pyridinebisoxazoline ligand. In my own view, the present work seems to be an excellent extension of E. N. Jacobsen in 2004. Same metal (ErCl3) and same kind of pyridine PYBOX are combined to catalyze the reaction but solve the real problem! I prefer to recommend the work to be published in Nature Commun. after minor revisions. Additional concern:

1. Although the authors emphasized that a suitable C4 relay group can help the formation of a sterically

constrained chiral pocket for nucleophilic addition of ketone hydrazones, I do not see how 4-phenylsulfonyl effect the reaction. By steric hindrance or electronic effect? Is DFT investigation helpful to elucidate the stereochemistry outcome? How about the reaction if switch phenylsulfonyl group to trifluoromethane sulfonyl ($CF_3SO_2^-$) group?

We thank Reviewer 2 for the kind recommendation and valuable advice to improve the quality of this work. Generally, chiral catalysts primarily leverage steric interactions between the catalyst and the substrate to influence stereoselectivity, although studies highlight the critical role of non-covalent interactions in shaping this selectivity. In our previous work on sterically confined PYBOX-enabled Cu-catalyzed asymmetric propargylic substitution (*Nat. Chem.* **2024**, *16*, 521; *Nat. Commun.* **2025**, *16*, 4571) and CuAAC reactions (*Angew. Chem. Int. Ed.* **2023**, *62*, e202301470), we studied the role of the pyridine C4 shielding group by control experiments, X-ray analysis and DFT calculations. These studies suggest that the pyridine C4 shielding group may facilitate the formation of a sterically confined microenvironment via the cooperation with the substituents on the chiral center of oxazoline, and modulate the Lewis acidity of the metal catalysts via electronic effects. Based on these studies, we believe increasing the steric hindrance of the C4 sulfonyl group is helpful to make a restricted chiral cavity to differentiate the sterically similar substituents on the prochiral carbon. In addition, tuning the bulk of this shielding group can finely tunes the chiral cavity for different substrates. For example, in the cyanation of challenging substrate isopropyl phenyl ketone derived hydrazone, increasing the steric hindrance of the C4 sulfonyl group, the enantioselectivity of the product **3z** gradually improved, showing the importance of the steric effect in creating a sterically restricted chiral pocket.

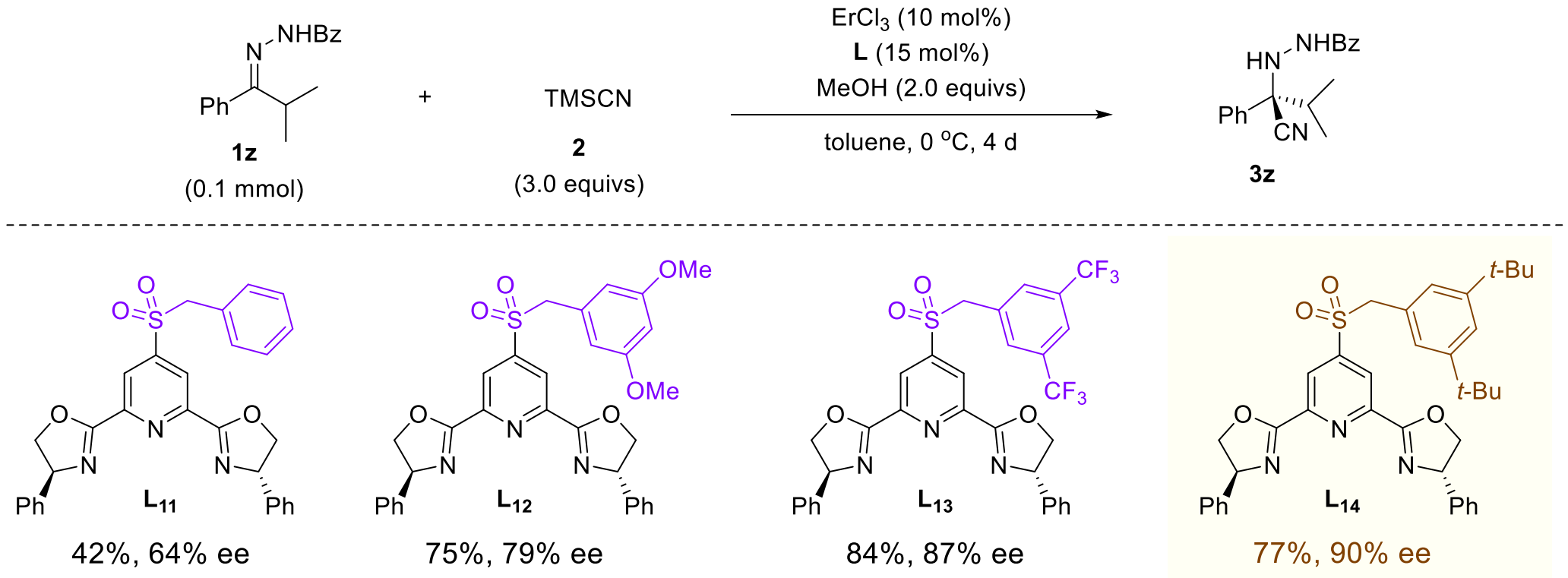

To shed light on the role of the C4 sulfonyl group of PYBOX in reinforcing enantiofacial discrimination. We also obtained the single crystals derived from $Er(OTf)_3$ with the unmodified PYBOX **L₁** or sulfonyl-PYBOX **L₁**, as shown below. The X-ray analysis of the two complexes revealed that upon introduction of the C4 sulfonyl group, the coordination number of Er(III) decreased from 9 to 8. Although these two complexes are in a 2:1 ligand:metal fashion, different from the substrate-catalyst complex, it supported our hypothesis that the presence of a bulky sulfonyl make the chiral pocket sterically confined. Additionally, the linear relationship indicated the involvement of a monomeric Er-complex in the enantiofacial discrimination. Moreover, the NMR and HRMS analyses of the combination of the $Er(OTf)_3$/PYBOX complex with hydrazone **1a** indicated the coordination of **1a** to the monomeric Er-complex.

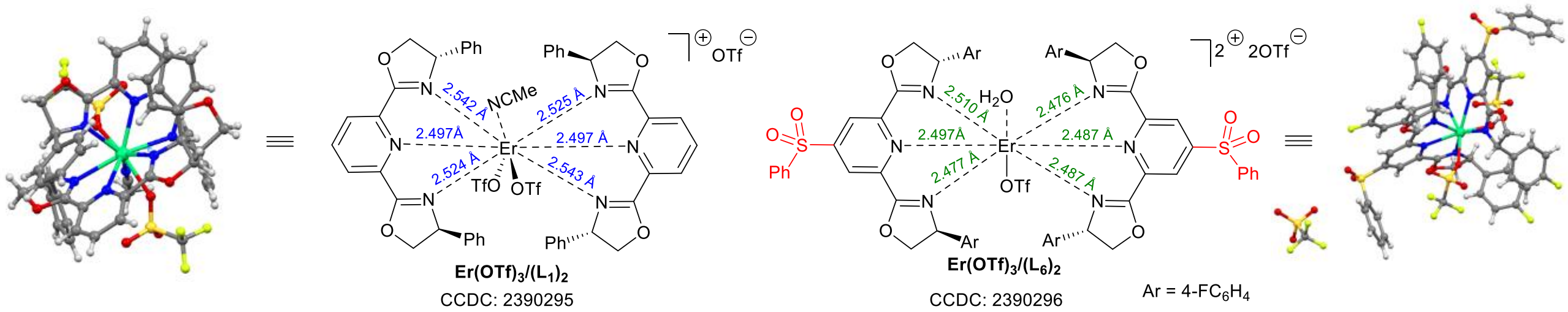

With the above information, we collaborated with Prof. Xin Wang at Sichuan University and researchers at the Beijing Computing Center to perform DFT calculations. However, despite attempts, we failed to obtain properly converged transition-state (**TS**) models. As shown below, even the TS structures corresponding to the *R* and *S* configurations were difficult to converge to models with the same coordination number (**TS-L1-*R***: eight-coordinate complex; **TS-L1-*S***: seven-coordinate complex). In addition, the free energy of **TS-L1-*R*** is calculated to be higher than that of **TS-L1-*S*** by 20.7kcal/mol. This is opposite to the experiment result that product (*R*)-**3a** was obtained as the major enantiomer. This is also possibly due to the failure to get accurate structure of the catalyst-substrate complex. X-ray analysis suggested a coordination number of 8 of Er(III) in the sulfonyl-PYBOX $\mathbf{L_6}$/Er(III) complex, but the ligand and the substrate account for only 5 coordination sites. It is unclear whether chloride, cyanide, or MeOH occupies the remaining sites, although ESI analysis of the reaction mixture was tried under a variety of different conditions. This made the DFT calculation very difficult.

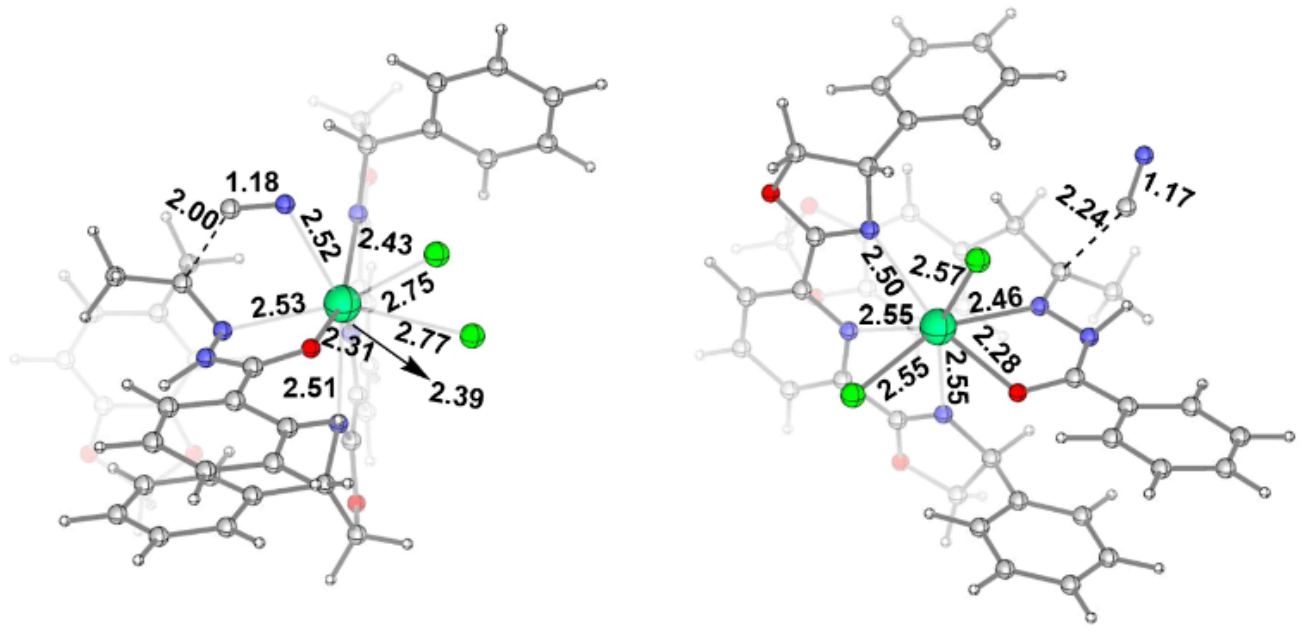

TS-L1-R TS-L1-S

This challenge is well recognized in the theoretical studies of lanthanide compounds, mainly arising from the highly localized and strongly correlated nature of the 4f electrons, which are often not adequately described by conventional DFT methods. In addition, the electronic structures of lanthanide centers frequently involve multiple near-degenerate states, complex spin configurations, and significant spin-orbit coupling effects, all of which can cause severe self-consistent field (SCF) convergence problems and strong sensitivity to the initial guess, the choice of functional, and the treatment of the f electrons (*Phys. Rev. B* **2007**, *75*, 045121). These suggest that enantioselective control in this system is influenced not only by steric effects, but also by the complex electronic structure and coordination behavior of the Er(III) center.

It is worth mentioning that DFT calculations of Er(III)-catalyzed reactions are far from trivial. As pointed out by Wilson: "*Lanthanides often require a treatment of static correlation effects via multireference methods* (*J. Chem. Theory Comput.* **2016**, *12*, 1259)." Furthermore, Furche noted that "*The KS-DFT calculations of lanthanide complexes, especially those with unconventional configurations, often suffer from slow convergence and metastable solutions* (*Dalton Trans.* **2024**, *53*, 410)." To our knowledge, no DFT calculation on chiral Er complexes catalyzed enantioselective reactions has been reported to date. A comprehensive computational investigation of the various possible coordination models would require substantial computational resources and time. We therefore believe that such studies are more appropriate for future in-depth investigation rather than for inclusion in the present work.

As for varying the pyridine C4-SO_2Ph group to a SO_2CF_3 group, we intensively tried but failed. Since neither this ligand nor its precursors have been reported, we tried two synthetic routes shown below, starting from 2,6-dicyano (or diester) pyridines with a C4-halogen or OTf group at the C4 position. Unfortunately, we tried in vain to obtain the precursor 2,6-dicyano (or diester) pyridines featuring a SO_2CF_3 group, no matter direct introduction of the SO_2CF_3 group or the installation of a SCF_3 group followed by oxidation to SO_2CF_3. Typical attempts are shown as follows.

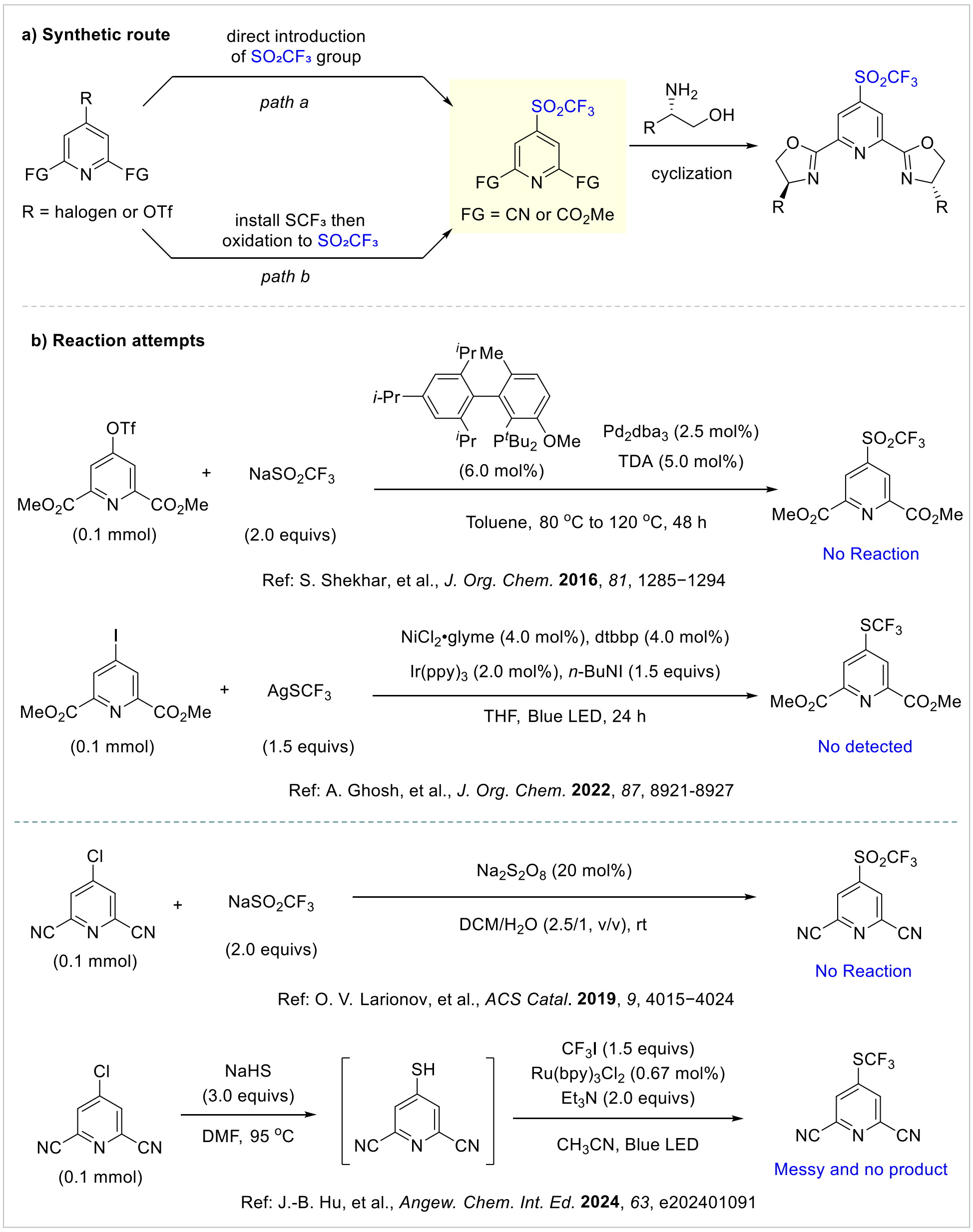

Reviewer #3 (Remarks to the Author)

Asymmetric cyanation to prochiral C=N double bonds is a useful transformation affording enantiomerically enriched α-aminonitrile derivatives. The reaction using ketone derived imine derivatives forming the chiral tetrasubstituted carbon centers is challenging from both fundamental and practical viewpoints. The submitted manuscript reports the reaction of N-benzoyl ketone hydrazones using the sulfonyl-PYBOX/ErCl3 catalyst. The PYBOX/ErCl3 catalyst for N-benzoyl hydrazones was reported by Jacobsen (Org. Lett. 2004, 6, 153), therefore the basic principle of catalytic function is known. The original PYBOX/ErCl3 catalyst showed medium enantioselectivity in the reaction of the acetophenone derived hydrazone (44% ee), but the novel sulfonyl-PYBOX/ErCl3 catalyst achieves much higher ee of 90% using the same substrate. A variety of hydrazones derived from alkyl aryl ketones is applicable to this reaction. Notably, this catalyst enables high enantioselectivity in the reaction of hydrazones derived from alkyl methyl ketones. Mechanistic studies and synthetic and biological applications are successfully performed. Originality of basic catalytic design is not excellent, but improvement of enantioselectivity and expansion of substrate scope by using the sulfonyl-PYBOX ligand are significant. Such ligand-modification concept seems to affect the catalyst-design of asymmetric reaction. I recommend this manuscript to be published in this journal.

The kind recommendation of Reviewer 3 is highly appreciated.

Other comments:

1. Page 9, first paragraph: Effect of C4 sulfonyl group on the pyridine of PYBOX is a key issue of this manuscript. It is described as "adjust the steric effect" and "form a more contact chiral pocket" in the text. These are vague expressions. The more detailed explanations are required.

We sincerely thank Reviewer 3 for this insightful comment. We acknowledge that the original descriptions were somewhat vague, and changed the description "form a more contact chiral pocket" to "form a more sterically confined chiral pocket". Notably, Reviewer 2 also raised a related question regarding the effect of C4 sulfonyl group of PYBOX, and we have provided a detailed response including experimental evidence and DFT computational attempts (please refer to the above response to Comment 1 from Reviewer 2). Accordingly, these detailed explanations have also been added in Part 8 of the SI.

2. Title, Fig. 1, and Table 1: "α,α-Dialkyl" is shown in the manuscript title. However, compounds shown in the first part of Table 1 are all α-alkyl-α-methyl hydrazinonitriles. I recommend showing the results of formation of α-alkyl-α-ethyl hydrazinonitrile, α-alkyl-α-isopropyl hydrazinonitrile, etc., otherwise the indication of title and molecular structure of Fig. 1d should be changed as "α-alkyl-α-methyl hydrazinonitriles".

As requested, we investigated the reactions of hydrazones bearing α-substituents other than the α-methyl group. After evaluation of the ligand and other reaction conditions, we found that when PYBOX $\mathbf{L_8}$ was employed as the ligand, the Strecker reaction of the α-*tert*-butyl-α-ethyl hydrazone proceeded with 80% ee, and cyclic ketone hydrazones gave the corresponding chiral product **3s** in 76% ee. These results have been incorporated into Table 1 of the revised manuscript, demonstrating that our protocol is not limited to α-alkyl-α-methyl ketone hydrazones, and α,α-dialkyl substrates are also workable substrates. Therefore, we kept the use of "α,α-dialkyl".

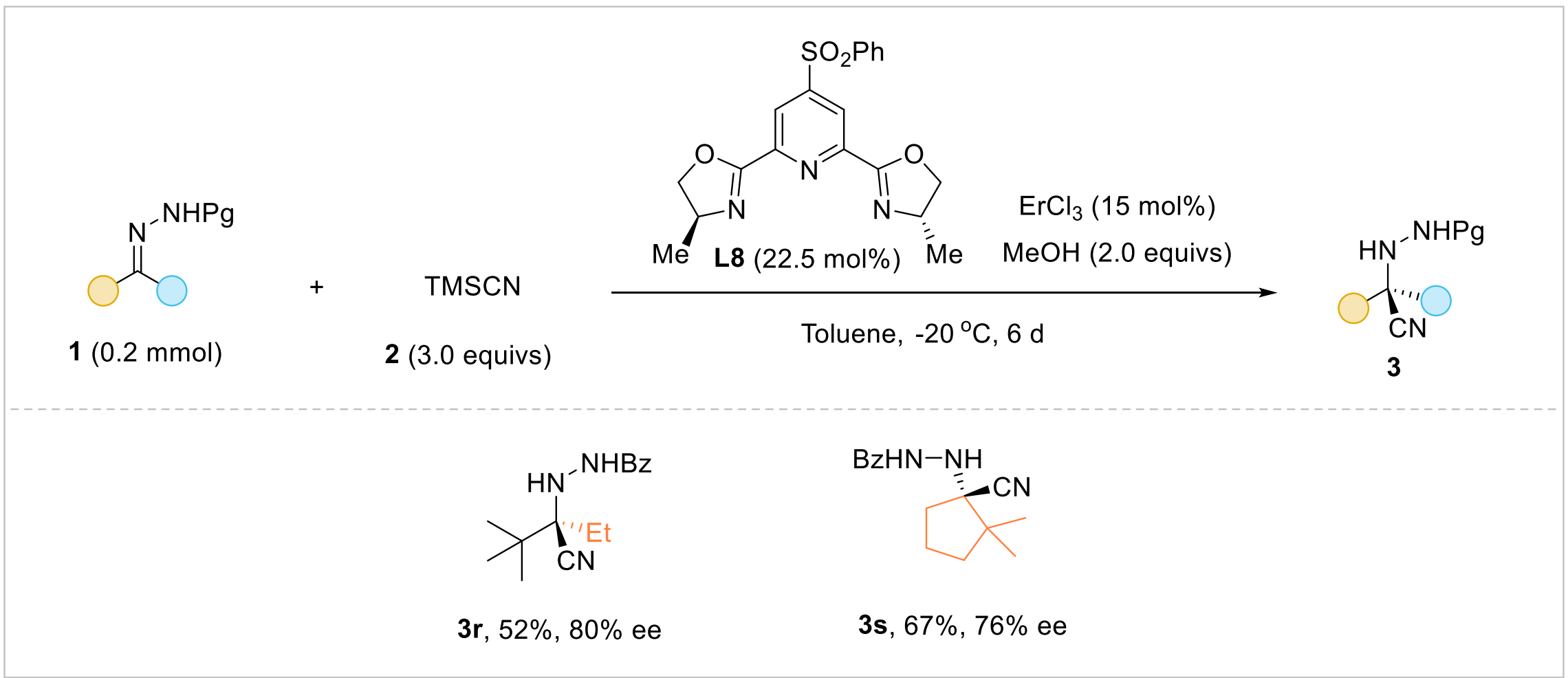

3. Fig 1: α-tert-Butyl-α-methyl hydrazinonitrile, α-tert-butyl-α-aryl hydrazinonitrile, and α-iso-propyl-α-aryl hydrazinonitrile are required to clarify the scope of reaction.

We appreciate the expertise of reviewer 3, because these substrates are really challenging substrates for Strecker-type reaction. Jacobsen et al. tried the cyanation of *tert*-butyl aldehyde hydrazone by using unmodified PYBOX $\mathbf{L_1}$ (*Org. Lett.* **6**, 153–155 (2004)), but the product **4d** was in only 66% ee. While the use of C4-sulfonyl-PYBOX $\mathbf{L_6}$ improved the ee of adduct **4d** to 90%, it failed to afford **3o** in satisfactory outcome. Fortunately, newly developed

PYBOX $\mathbf{L_8}$ could afford chiral hydrazine **3o** in 96% yield and 90% ee. Based on this result, we further examined other α-branched alkyl ketone hydrazones, which afforded the corresponding chiral products **3p**–**r** in 80–94% ee. Additionally, cyclic ketone hydrazones were also investigated, and the desired **3s** could be obtained in 76% ee. These results have been added into Table 1 of the revised manuscript.

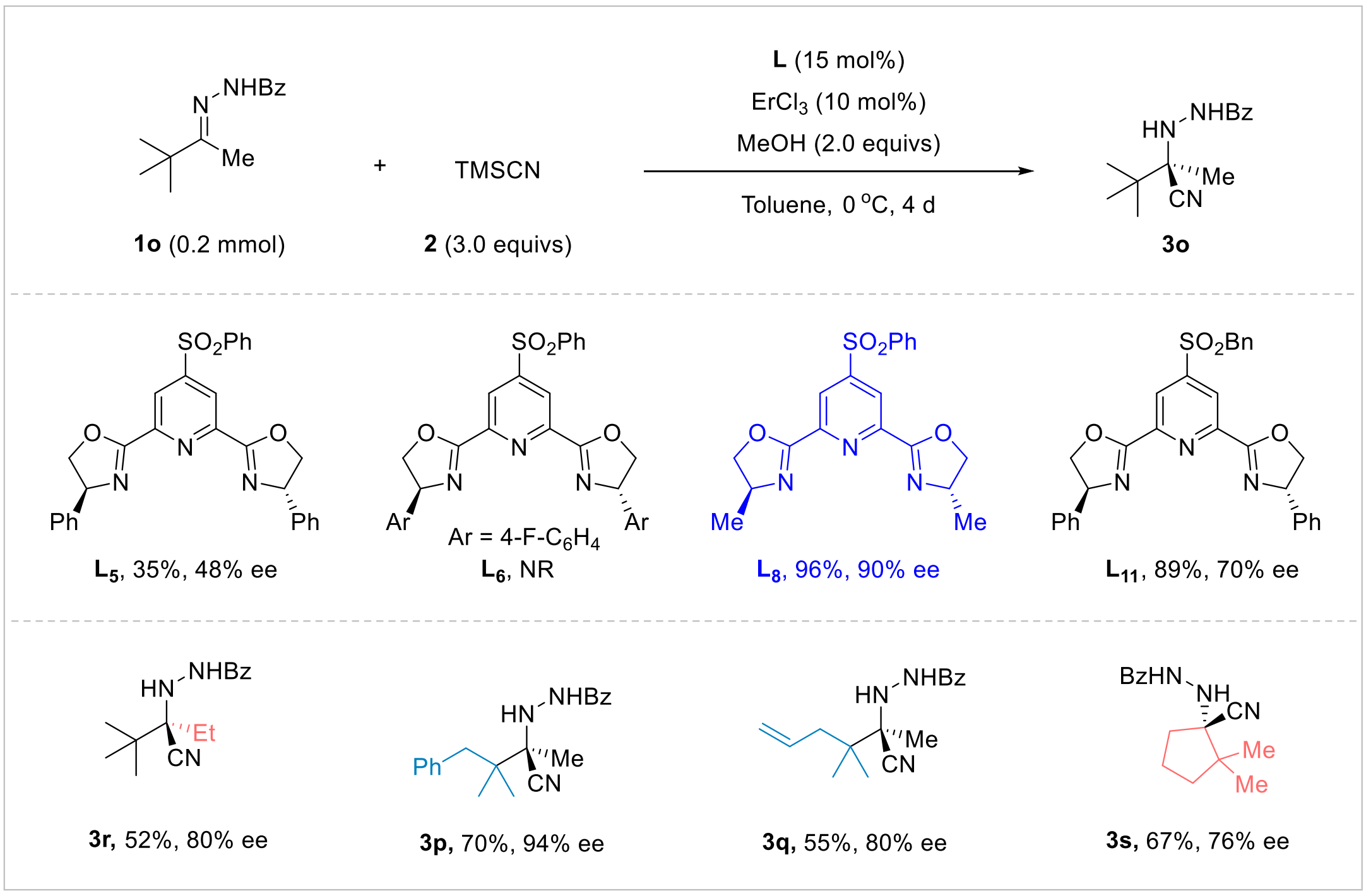

The isopropyl phenyl ketone-derived hydrazone **1z** was then examined. To our knowledge, it is highly challenging to differentiate the prochiral faces of the C=N double bond derived from this type of ketone, and only one successful catalytic enantioselective protocol has been reported to date (Hoveyda et al. *Nat. Chem.* **9**, 1269−1275 (2017)). Not unexpectedly, previous condition using sulfonyl-PYBOX $\mathbf{L_6}$ afforded the product **3z** in only 35% yield and 38% ee. Gratifyingly, newly developed PYBOX ligand $\mathbf{L_{14}}$ could furnish product **3z** in 77% yield and 90% ee. Then aromatic ketone hydrazones with α-*tert*-butyl, α-cyclopropyl, and α-cyclohexyl groups were also tried, and the corresponding products **3aa**–**ac** were obtained in 83–85% ee. These results have been incorporated into Fig. 2 and Table 1 of the revised manuscript. These results further confirm that introducing a bulky C4 shielding group is a flexible strategy to create a sterically confined chiral pocket for achieving challenging asymmetric reactions that are unattainable with conventional PYBOX ligands.

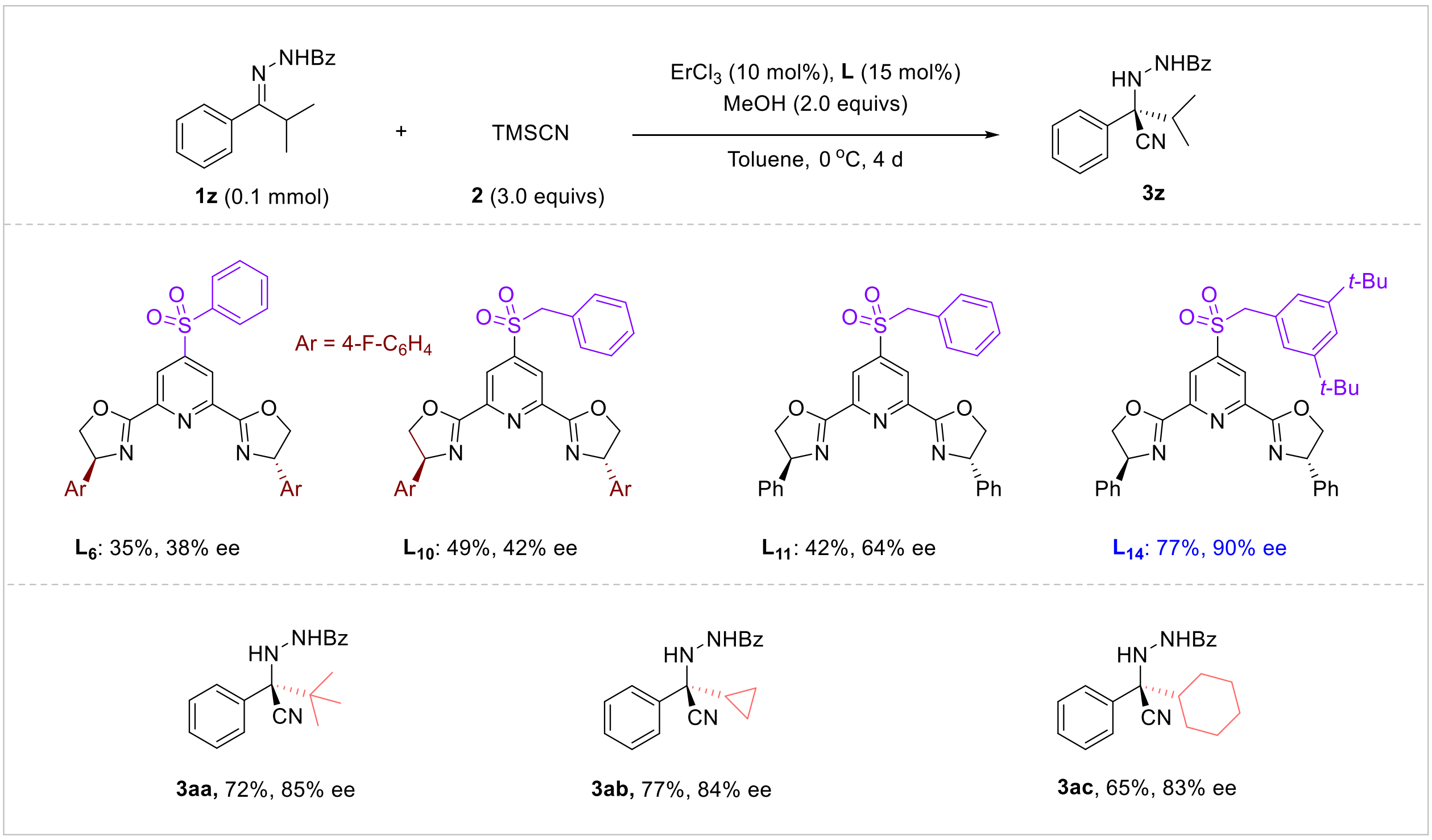

4. Supporting Information: Enantiomer peak separation of HPLC analysis for **3p** and **4a** is not appropriate. Reanalysis is required.

Accordingly, the HPLC analysis of **3p** (**3n** in the revised manuscript) and **4a** has been reanalyzed, and the results are provided in the SI.

Reviewer #4 (Remarks to the Author):

The authors discovered a highly enantioselective nucleophilic addition reaction of simple ketone-derived hydrazones, and investigated the reaction mechanism and insecticidal activity. Here are some suggestions: In the preliminary screening of insecticidal activity, only some of the target compounds were screened instead of all. What is the rationale for selecting these compounds?

We first thank reviewer 4 for her/his time and attention to our manuscript. We selected compounds containing different electronic groups based on the modifiable sites of the core structure of α-hydrazino nitriles. The detailed screening process of insecticidal active molecules has been added in the revised SI, which are copied as below:

"*Based on the different modifiable sites of α-hydrazino nitrile scaffold, we investigate the influence of substituent effects on insecticidal activity (**Figure S11**). The activities of compounds **3f**, **3h**, **3u**, and **3r** were first evaluated. The results showed that the phenyl-substituted **3w** exhibited significantly higher insecticidal activity (61.72 ± 6.46%) than the alkyl-substituted analogs **3f**, **3h**, and **3u** (**Figure S11A**). Next, the phenyl group was retained, and a series alkyl substituents was investigated. As shown in **Figure S11B**, the methyl-substituted derivative **3w** and the ethyl-substituted derivative **3x** displayed comparable moderate insecticidal activities, whereas the hydrogen-substituted analog **4a** showed a marked reduction in activity. These findings suggest that the tetra-substituted carbon center contributes positively to enhancing insecticidal activity. The insecticidal activities of various aryl-substituted derivatives (**3au**, **3aw**, **3ax**, **3af**, **3ai**, **3ae**, **3ak**, and **3aj**) were further evaluated. In most cases, the introduction of electron-withdrawing groups (F, Cl, or CF_3) increased the activity to >80% (**Figure S11C**). Finally, insecticidal activity was assessed for the hydrazino nitrile scaffold bearing different protecting groups and its derivatives, and*

*compound **12** exhibited activity >80% (**Figure S11D**).”*

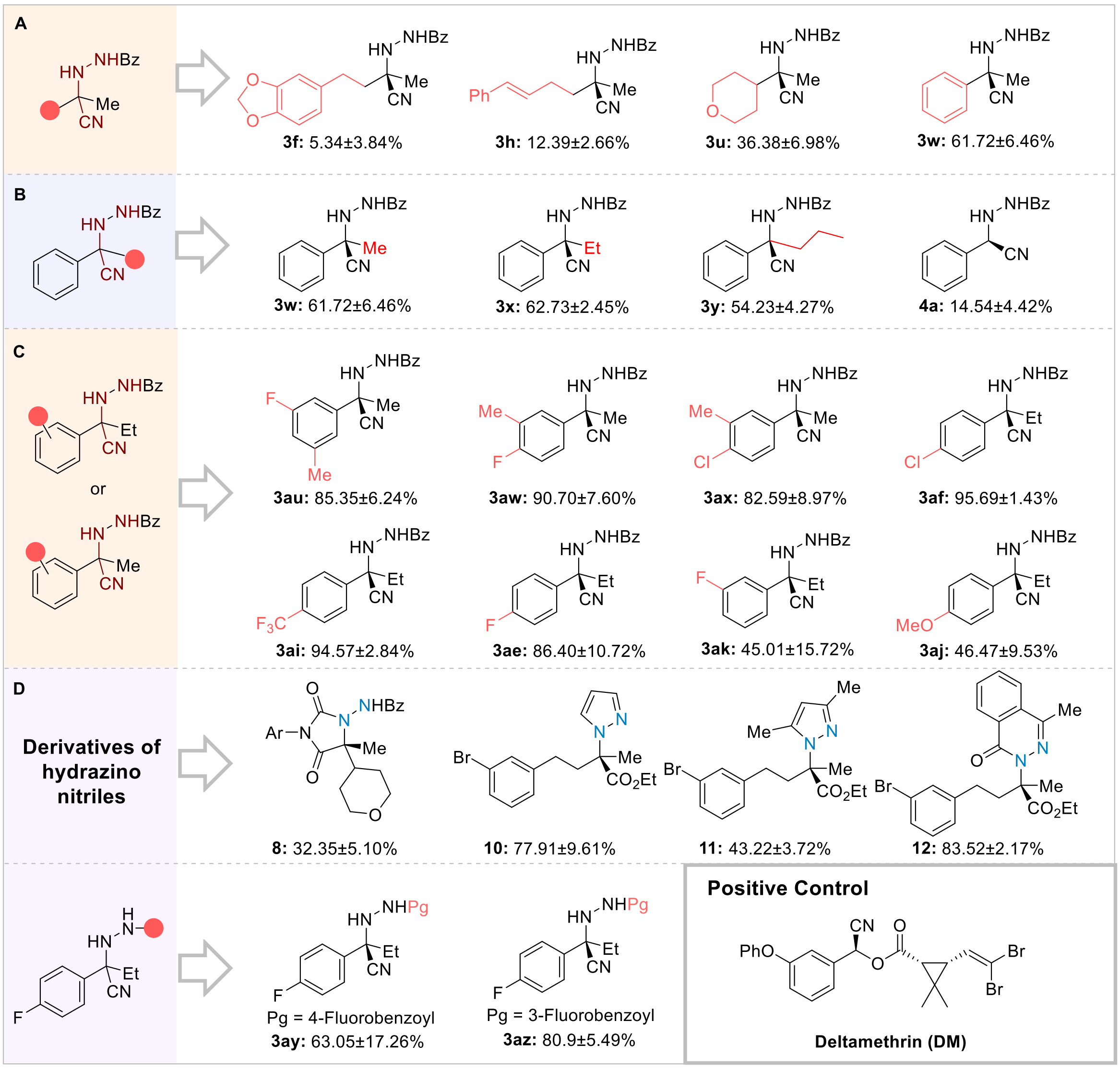

Insecticidal activity against *A. gossypii* of selected α-hydrazino nitrile compounds

2. The significant difference analysis should be supplemented in Figure 5 (b)

-We thank the reviewer for this suggestion. The significant difference analysis has been presented in figure 5b. The compounds **3au, 3aw, 3ax, 3af, 3ai and 3ae** have excellent activities at 100 mg·L^{-1} concentration and show no significant difference (n.s.) compared to Deltamethrin. All the significant difference analyses have been added to the supporting information, as shown below.

Comparisons	Summary	P Value	Comparisons	Summary	P Value
DM vs. 3f	****	<0.0001	DM vs. 3ai	ns	0.5802
DM vs. 3h	****	<0.0001	DM vs. 3ae	ns	0.3189
DM vs. 3u	****	<0.0001	DM vs. 3ak	**	0.0060
DM vs. 3w	**	0.0011	DM vs. 3aj	**	0.0011
DM vs. 3x	****	<0.0001	DM vs. 3ay	*	0.0381
DM vs. 3y	***	0.0001	DM vs. 3az	*	0.0182
DM vs. 4a	****	<0.0001	DM vs. 8	****	<0.0001
DM vs. 3au	ns	0.0914	DM vs. 10	*	0.0497
DM vs. 3aw	ns	0.5648	DM vs. 11	****	<0.0001
DM vs. 3ax	ns	0.1056	DM vs. 12	**	0.0024
DM vs. 3af	ns	0.1168			

Significant difference analysis of insecticidal activity

3. The authors found that compound **3u** exhibited superior insecticidal activity against Aphid gossypii to Deltamethrin. Why did the authors only choose Deltamethrin as the positive control? Including other highly effective insecticides as positive controls would provide a better comparative assessment of the insecticidal potential of compound **3u**.

We thank the reviewer and fully agree with the comment. Deltamethrin (DM) was selected as the positive control in this study, as it is one of the most widely used commercial chiral cyano-substituted insecticide. To provide a more comprehensive assessment of the insecticidal potential of the target compound, Esfenvalerate (ES) and Imidacloprid (IMI) were additionally included as positive controls in the LC_{50} assay. Although both computational simulation results and poisoning observations indicate different insecticidal mechanism between IMI and α-tertiary hydrazine compounds, IMI was still incorporated to achieve a more systematic activity evaluation. The relevant data have been updated in the revised Figures 5d and 5e, with detailed statistical analysis (including 95% confidence intervals) added to the figure legends. Concurrently, the discussion section of the LC_{50} study has been revised, and the updated content is provided below (Compound **3u** has been renumbered as **3af** in the revised manuscript):

*"The LC_{50} values of candidate compounds **3au, 3aw, 3ax, 3af, 3ai** and **3ae** were determined, with Deltamethrin (DM), Esfenvalerate (ES), and Imidacloprid (IMI) serving as positive controls. The dose-response relationship between the logarithm of compound concentration and insect mortality is shown in Fig. 5d. Hydrazino nitrile **3af** exhibited the lowest LC_{50} value (15.11 mg L^{-1}) against A. gossypii, outperforming **3au** (38.35 mg L^{-1}), **3aw** (24.80 mg L^{-1}), **3ax** (50.48 mg L^{-1}), **3ai** (43.95 mg L^{-1}) and **3ae** (37.92 mg L^{-1}). The LC_{50} value of **3af** was lower than those of the positive controls DM (23.99 mg L^{-1}) and ES (20.72 mg L^{-1}), but higher than that of IMI (3.07 mg L^{-1}). Furthermore, the LC_{50} value of the enantiopure form of **3af** was significantly lower than that of its racemic counterpart, highlighting the critical role of chirality in insecticide design (Fig. 5e)."*

4. Compound **3u** showed promising insecticidal activity. The authors could further investigate its insecticidal mechanism to provide theoretical guidance for the development of novel insecticides. Of course, if this falls outside the scope of the authors' research, collaborative efforts with other researchers could be considered, and further

investigation into the insecticidal mechanism could be conducted in subsequent studies.

Thanks a lot for this suggestion. A highly enantioselective synthesis of α-hydrazino nitriles was developed as core content in this study. Given the structural similarity between chiral α-hydrazino nitriles and pesticide molecules, we conducted a preliminary investigation into the insecticidal activity. The promising insecticidal activity of chiral α-hydrazino nitriles shows the nice application potential of our chemistry in agrochemical research, which requires further studies that is not the focus of this manuscript. Nevertheless, we conducted additional computational analysis to further elucidate the insecticidal mechanism of compound **3u** (renumbered as **3af** in the revised manuscript). The corresponding results have been incorporated into the revised manuscript. The relevant content is copied as below:

*"To further elucidate the insecticidal mechanism of α-hydrazino nitrile **3af**, we conducted computational analysis. Given that compound **3af** shares a similar scaffold with pyrethroid insecticides, and that ES shows the highest structural similarity to **3af** according to Morgan fingerprint analysis, we hypothesized that compound **3af** targets the same voltage-gated sodium channel (VGSC) as ES. Based on this hypothesis, molecular docking and molecular dynamics simulations were performed focusing on VGSC. The results indicate that compound **3af** can form a stable binding mode with VGSC, preliminarily supporting VGSC as the target for this class of compounds (Fig. 5f and Fig. S12 in SI). Further investigation of the insecticidal mechanism will be carried out in subsequent studies."*

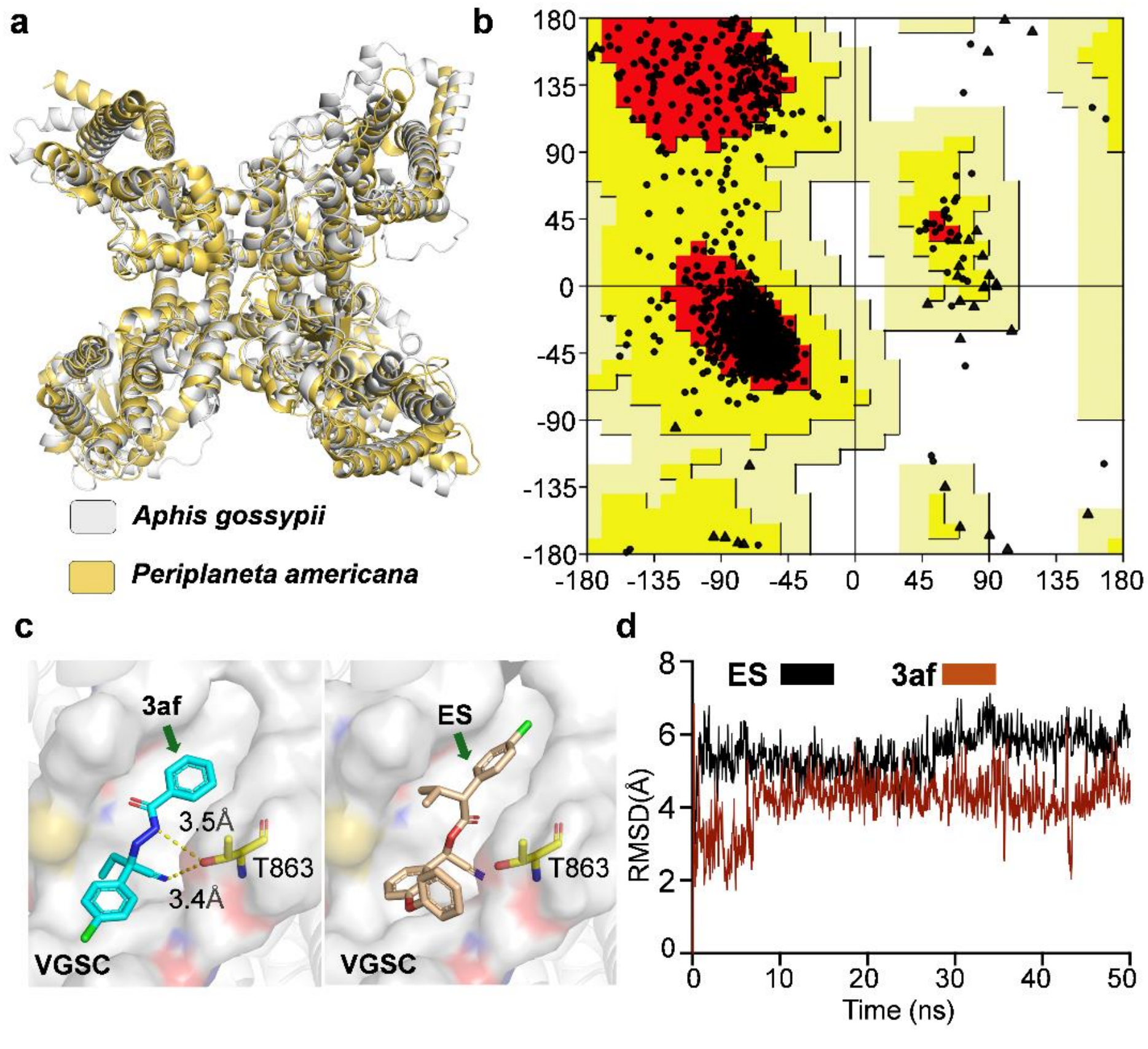

Structural modelling, validation and molecular dynamics analysis of the 3af-VGSC complex. a, The AlphaFold3-predicted Aphis gossypii VGSC model was superposed with the Periplaneta americana VGSC crystal structure (PDB: 5X0M), with an RMSD of 2.03 Å. **b**, Ramachandran plot of the Aphis gossypii VGSC model, showing 90.6% of residues in the most favoured regions, 8.4% in additional allowed regions, 0.5% in generously allowed regions and 0.5% in disallowed regions. **c**, Binding modes of **3af** and ES in the VGSC binding pocket. In the **3af**-bound model, the cyano group of **3af** engages in hydrogen-bonding interactions with T863, indicated by yellow dashed lines. **d**, RMSD trajectories of **3af** and ES during 50 ns molecular dynamics simulations.

The detailed computational analysis results have been added in part 10 of the revised SI, which is copied as below:

*"Molecular docking of **3af** and ES into the Aphis gossypii VGSC model was performed using the Glide module in Schrödinger in Standard Precision (SP) mode. The Aphis gossypii VGSC structure was predicted by AlphaFold3. The membrane-embedded orientation of the channel was assigned with reference to the OPM entry for the Periplaneta americana VGSC crystal structure (PDB: 5X0M). Prior to docking, the protein structure was prepared using the Protein Preparation Wizard, in which hydrogen atoms were added, the hydrogen-bonding network was optimized, and restrained energy minimization was performed. The ligands **3af** and ES were prepared using LigPrep. Representative docking poses were selected for subsequent MD simulations.*

MD simulations were carried out using the Desmond module in Schrödinger with the OPLS4 force field. The protein-ligand complexes were embedded in a membrane environment, solvated with the SPC water model, and neutralized with appropriate counterions. NaCl was added to a final concentration of 0.15 M. Following energy minimization, equilibration was performed under NVT and NPT ensembles. Production runs were then conducted for 50 ns at 300 K and 1 atm. The stability of the complexes and the key ligand-protein interactions throughout the simulations were evaluated using the Simulation Interaction Diagram module."

Thank you very much.

With best regards.

Sincerely yours,

Jian Zhou, Prof. Dr.
